# VESPUCCI: Exploring Patterns of Gene Expression in Grapevine

**DOI:** 10.3389/fpls.2016.00633

**Published:** 2016-05-10

**Authors:** Marco Moretto, Paolo Sonego, Stefania Pilati, Giulia Malacarne, Laura Costantini, Lukasz Grzeskowiak, Giorgia Bagagli, Maria Stella Grando, Claudio Moser, Kristof Engelen

**Affiliations:** ^1^Department of Computational Biology, Research and Innovation Center, Fondazione Edmund MachTrento, Italy; ^2^Department of Biology, University of PadovaPadova, Italy; ^3^Department of Genomics and Biology of Fruit Crop, Research and Innovation Center, Fondazione Edmund MachTrento, Italy

**Keywords:** gene expression, grapevine, transcriptomics, compendium, microarray, RNA-Seq

## Abstract

Large-scale transcriptional studies aim to decipher the dynamic cellular responses to a stimulus, like different environmental conditions. In the era of high-throughput omics biology, the most used technologies for these purposes are microarray and RNA-Seq, whose data are usually required to be deposited in public repositories upon publication. Such repositories have the enormous potential to provide a comprehensive view of how different experimental conditions lead to expression changes, by comparing gene expression across all possible measured conditions. Unfortunately, this task is greatly impaired by differences among experimental platforms that make direct comparisons difficult. In this paper, we present the *Vitis Expression Studies Platform Using COLOMBOS Compendia Instances* (VESPUCCI), a gene expression compendium for grapevine which was built by adapting an approach originally developed for bacteria, and show how it can be used to investigate complex gene expression patterns. We integrated nearly all publicly available microarray and RNA-Seq expression data: 1608 gene expression samples from 10 different technological platforms. Each sample has been manually annotated using a controlled vocabulary developed *ad hoc* to ensure both human readability and computational tractability. Expression data in the compendium can be visually explored using several tools provided by the web interface or can be programmatically accessed using the REST interface. VESPUCCI is freely accessible at http://vespucci.colombos.fmach.it.

## Introduction

Grapevine (*Vitis* spp.) is an economically important fruit crop and one of the most cultivated crops worldwide (Vivier and Pretorius, 2002). Grape berries are consumed as fresh fruit or used for high-valued commodities as wine or spirits. Grapevine transcriptomics studies started over a decade ago, initially using microarrays but later, exploiting the sequenced genomes (Jaillon et al., 2007; Velasco et al., 2007) and the availability of high-throughput sequencing, also using RNA-Seq approaches. As system biology becomes more prevailing in everyday analysis, one of the pressing aspect of analysis is how to integrate different sources of information into one coherent framework that can be interrogated in order to gain knowledge about the system as a whole (Rhee et al., 2006). Prior to biological information integration across several levels (such as proteomics, transcriptomics, and metabolomics), it is important to acquire and combine all the possible available information within each specific field. Together with the methodological problem of combining different sources of information, there’s the more practical issue of having sufficient data to justify data integration in the first place, because in order to draw general and valid conclusions a large amount of data is a desirable feature. While for model species this is hardly an issue, for non-model crop species the number of performed experiments might be limited, the technological platforms less established, and heterogeneous data a further complicating factor. Nevertheless, as biology is turning into a data-driven science the prospect of large dataset availability becomes more and more feasible even for non-model species, and in terms of gene expression and functional analysis there have been several efforts to fulfill data integration in different organisms including grapevine (Wong et al., 2013; Pulvirenti et al., 2015), strawberry (Yue et al., 2015), and citrus (Wong et al., 2014).

In this paper, we present an expansive grapevine gene expression compendium that can be used to analyze grapevine gene expression at a broad level. It was created based on an approach for dealing with the large heterogeneity of data formats present in public databases, and to integrate cross-platform gene expression experiments in one dedicated, coherent database. The proof-of-concept of this approach was presented in Engelen et al. (2011) as a web-application for exploring and analyzing specific expression data of several bacterial species. This original technology platform has already been used as a basic framework for creating a gene expression compendium for a more complex case as the multicellular, higher eukaryote *Zea mays* (Fu et al., 2014). Here, we used the most updated version of the COLOMBOS technology (Moretto et al., 2016) to show how this approach can be further extended for the creation of gene expression compendia on other important crop species, focusing our attention on grapevine gene expression studies. Regardless of the available tools, most of the steps toward the creation of such a compendium, require a massive amount of manual curation, from defining a controlled vocabulary for description of experimental conditions to the interpretation of experiment designs and annotation of the included samples. The benefits of *Vitis* expression studies platform using COLOMBOS compendia instances (VESPUCCI) lie in the availability of the whole known measured transcriptome activity of grapevine in a single programmatically accessible repository and the possibility to extensively explore gene expression patterns through the visual tools made available by the web interface.

## Materials and Methods

### Data Sources

The experiments included in VESPUCCI have been collected from the Gene Expression Omnibus (GEO; Barrett et al., 2013), ArrayExpress (Kolesnikov et al., 2015), and the Sequence Read Archive (SRA)^1^. The majority is made up of microarray experiments (91% of samples), with the ‘NimbleGen 090918 Vitus HX12 array’ and ‘Illumina HiSeq 1000’ being the most used platforms among microarray and RNA-Seq experiments, respectively. **Table 1** shows the summary of samples imported per platform. The complete overview of imported experiments and platforms is available in **Supplementary Table S1**.

**Table 1:** Number of samples per technology platform.

Platform name	Platform type	Number of samples
NimbleGen 090918 vitus vinifera exp HX12	Microarray	583
Affymetrix *V. vinifera* (grape) genome array	Microarray	502
Affymetrix GrapeGen *V. vinifera* GrapeGena520510F	Microarray	219
INRA *V. vinifera* oligo array 15K v3	Microarray	100


Combimatrix GrapeArray 1.2	Microarray	69


Illumina HiSeq 1000	RNA-Seq	60


Illumina HiSeq 2500	RNA-Seq	36


AB 5500 xl genetic analyzer	RNA-Seq	20


Illumina HiSeq 2000	RNA-Seq	12


Illumina genome analyzer IIx	RNA-Seq	7

*Overview of all samples imported in VESPUCCI ordered by number of samples. The first column contains the name of the transcriptomics platform, the second column is the type of platform either microarray or RNA-Seq. The third column contains the number of samples measured with the respective platform imported in VESPUCCI*.

### Gene Annotation

The CRIBI V1 gene prediction^2^ and associated sequences for *Vitis vinifera* PN40024 (cv. Pinot Noir) have been used as the base gene transcript list. Corresponding gene functional annotations have also been added. Together with the original CRIBI annotation, which comprises GO (Blake et al., 2015), KEGG (Kanehisa et al., 2016), Pfam (Finn et al., 2015), ProSite (Sigrist et al., 2013), and Smart (Letunic et al., 2015), the VitisNet (Grimplet et al., 2009) molecular network was also included.

### Sample Annotation

Samples in VESPUCCI have been manually curated using a controlled vocabulary to precisely describe which parameters have changed across different experimental conditions. The creation of the controlled vocabulary is an ongoing adaptive manual process, in which curators add or modify new terms as needed during the acquisition of new experiment samples, keeping the vocabulary as concise and organized as possible. Terms in the vocabulary have largely been introduced *ex novo* following the original experimental designs, but on occasion have also been borrowed from other annotation systems like the Plant Ontology^3^ (Cooper et al., 2013) for describing the plant anatomical structures or the modified Eichhorn–Lorenz scale (Dry and Coombe, 2004) for describing grapevine-specific developmental stages. The complete vocabulary, along with its hierarchical structure, is available in the **Supplementary Table S2**.

### Compendium Creation

The compendium creation process can be divided in three major steps: data collection and parsing, sample annotation, and data homogenization. To facilitate these three steps and to deal with the complexity of maintaining big amounts of data and meta-data, we have relied mostly on the COLOMBOS v2.0 (Meysman et al., 2014) and v3.0 (Moretto et al., 2016) backend managing applications.

For this *V. vinifera* expression compendium, new tools were added to the COLOMBOS backend software, mainly related to the probe-to-gene (re)mapping. Specifically, microarray probes are now aligned by a two-step filtering procedure using the BLAST+ program (Camacho et al., 2009). The two filtering steps are done to ensure that probes not only map to genes with high similarity (restrictive alignment threshold), but also that they map uniquely (unambiguously) to a single location and be less prone to cross-hybridization (less restrictive alignment threshold). Probes of different microarray platforms generally vary in terms of length, species/cultivar of origin, and sequence quality. To always obtain the reasonably best possible alignment according to each platform’s specific characteristics, parameters, and cutoff thresholds were employed on a platform-specific basis.

## Results

### *Vitis vinifera* Gene Expression Compendium

At the core of the VESPUCCI *V. vinifera* compendium is a gene expression matrix that combines publicly available transcriptome experiments from the most common microarray and RNA-Seq platforms (an overview is given in **Table 1** and **Supplementary Table S1**). VESPUCCI’s distinctive characteristics are its data integration strategy and the way in which it handles information coming from different platforms and technologies, which is based on COLOMBOS technology. Data and meta-data are gathered and curated starting from raw intensities or sequence reads for microarrays and RNA-Seq, respectively. A robust normalization and quality control procedure is performed to permit direct comparison of gene expression values across different experiments and platforms. This results in a single expression matrix in which each row represents a gene and each column represents a ‘sample contrast.’ Sample contrasts measure the difference between a ‘test’ and a ‘reference’ sample from the same experiment. The decision as to which samples are paired to form contrasts, is made in part based on technical considerations as explained in Engelen et al. (2011), and in part on the desire to deviate as little as possible from the original intent of the experiment. Both samples, and the differences between them, are then extensively annotated with various sorts of meta-data. The expression data itself are log-ratios (in base 2), so that positive values represent up-regulation, and negative values represent down-regulation of a gene in the test sample compared to the reference sample. VESPUCCI’s compendium was built with specific modifications and additions for *V. vinifera* to the COLOMBOS technology, and these are described in the following sections.

### Defining Measurable Gene Transcripts

The list of measurable gene transcripts, representing the rows of the expression matrix, is based on the CRIBI V1 gene annotation, with some modifications to optimize probe-to-gene remapping (see next section), and read alignment. An important consideration for this remapping is that the CRIBI V1 gene predictions can show (regions of) high similarity, which is not uncommon for plant crop species. As a result, probes can end up matching perfectly, or near perfectly, to more than one gene. According to the way in which, we built the compendium, such shared, ambiguous probes would usually be discarded because of their inability to reliably measure one single gene. Instead of removing these probes, with consequent loss of information, we decided to keep them as a measurement of a whole cluster of genes, implying those genes expression changes can only be assessed as a whole but not individually. The decision is a trade-off between losing probes (measurements) and losing the possibility to distinctively measure each gene as a single entity. We used the Nimblegen platform to investigate both ambiguous probes behavior and gene prediction structure, and decided on 466 cases in which genes can be “clustered” together according to their sequence similarity and the probes they share.

**FIGURE 1:**
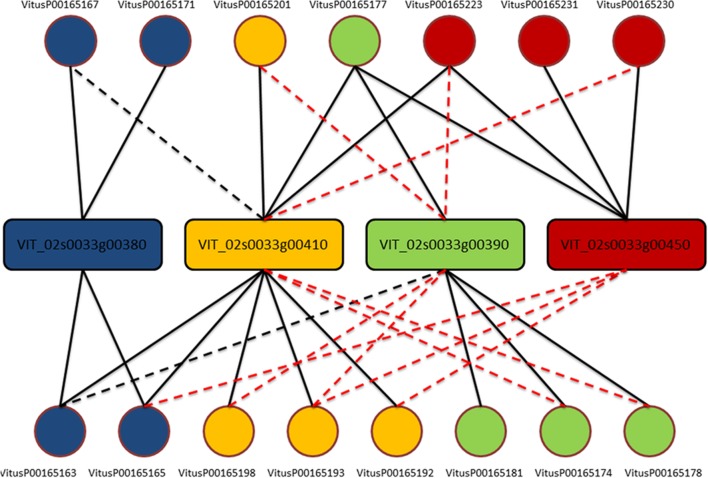
**Probe-to-gene mapping for cluster 170**. Genes (in rectangles) are colored accordingly to probes (circles) based on the original platform mapping. Each line corresponds to an alignment of the whole probe against one gene. A solid line means no mismatches, a black dashed line means one mismatch while a red dashed line means two or three mismatches.

**FIGURE 2:**
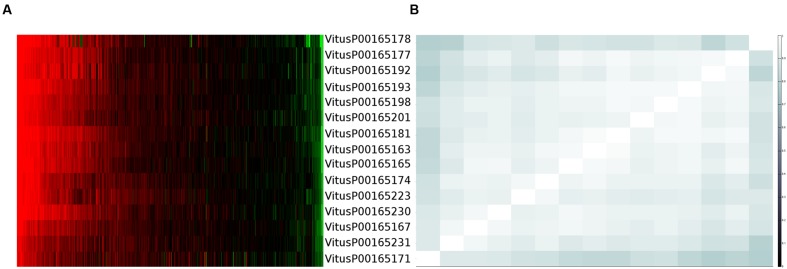
**Probe expression values and correlation for cluster 170. (A)** Probes expression values measured across more than 500 Nimblegen sample contrasts sorted by values. **(B)** Probes correlation matrix using uncentered Pearson correlation.

One clear–cut case to present the complexity of the issue is depicted in **Figure 1**. From this example is clear that each gene is actually measured on average by four probes (as expected) but, except for three probes (VitusP00165181, VitusP00165231, and VitusP00165171) all the other probes align perfectly (or near perfectly) to other genes, making impossible to distinguish one gene from another. In particular these four genes, beside being different among each other, are all annotated as Myb-related, a well-known transcription factor gene family composed by 100s of genes (Matus et al., 2008) and are positioned one after the other across chromosome 2 in a region of approximately 130 kb. This target cross-talk is corroborated by the actual probe-level intensities, which are highly correlated across all sample contrasts included in the compendium (**Figure 2**).

**FIGURE 3:**
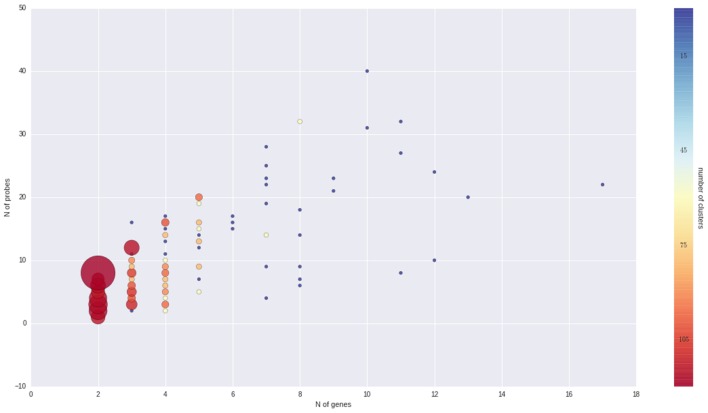
**Overview of gene clusters**. Both the size and color of the spheres are proportional to the number of clusters that is made up of a given number of genes and probes. It is clear that the great majority of clusters are composed by just few genes and probes.

To better understand the nature of gene-probe clusters, we carried out a survey of each of the 466 clusters. They consist in total of 1366 genes and 3472 probes, distributed across clusters as depicted in **Figure 3**. We inspected the clusters based on the probe-to-gene alignment quality and probe-level expression values across all Nimblegen experiments imported in VESPUCCI (38% of sample contrasts). The great majority of clusters consist of only a few genes with consistent behavior (according to probe expression patterns) and that are part of gene families and positioned one after the other along the same chromosome (or predicted on un-anchored loci). Other clusters are extremely dense and highly connected (e.g., clusters 1, 15, 176, and 177). Another set of clusters is composed by weakly connected genes (few probes) positioned on different chromosomes. For example cluster 283 is composed by five putative kinase proteins that span four chromosomes, and for which probes might be designed on a conserved catalytic domain. Some clusters present a ‘perfect ambiguity’ structure (e.g., clusters 47, 65) for which each probe aligns perfectly to each gene, making impossible to distinguish across measured genes. Interestingly, clusters with a non-perfect alignment (e.g., clusters 134, 220) instead show how probe level expression values reflect alignment mismatches, exposing the issue of measuring genomic variability instead of expression changes. Cases such clusters 185, 213, and others suggest that the measured genes could be allelic variants of the same gene as they are 99% similar with similar structure and predicted on contiguous or un-anchored loci. Finally, few other clusters appear to be problematic due to bad expression data and ambiguous probe-to-gene alignment (e.g., clusters 20, 21, and 42). All of the gene cluster related information (probe-to-gene alignment graphs, probe-level expression, and correlation heatmaps) is available as Supplementary Materials.

### Probe-to-Gene Remapping

To take full advantage of an updated gene annotation and for a more coherent integration of different platforms, we remapped probes for each microarray platform to the CRIBI V1 gene prediction. Such remapping of probes to transcripts has advantages over original annotations (Yin et al., 2010).

Different microarray platforms have different probe-to-gene alignment qualities. Given the disparateness in terms of number of samples, number of measured transcripts, and probe-to-gene mapping quality not all the available microarray platforms have been imported. The top performing platform is the Nimblegen microarray that shows a nearly perfect correspondence to the one in VESPUCCI. This is easily explained by the fact that it contains 118015 probes of 60 nucleotides with an average of four probes per gene and was specifically designed to match the CRIBI V1 gene prediction. It measures the expression of 29549 (out of 29971) gene predictions representing ∼98.6% of the genes of the CRIBI V1 gene prediction and 19091 random probes as negative controls (Fasoli et al., 2012; Cookson and Ollat, 2013). On the other hand, platforms like the ‘University of Arizona *Vitis* buds spotted DNA/cDNA array’ exhibit quite poor performance in terms of number of measured transcripts, probe-to-gene mapping, and probe signal (data not shown), which made us decide to exclude it from the compendium. The low quality can be ascribed to the fact that its 10369 probes have been designed from ESTs of two *V. vinifera* cultivars (Perlette and Superior) as well as the *V. riparia* species, and have an average length of nearly 1 kb.

We compared our probe-to-gene mapping results to the original mappings for the microarray platforms using the complete gene annotation^4^ (Grimplet et al., 2012). The results are reported in **Table 2**. The mapping is quite consistent to the original mappings, with the notable exception being the ‘Combimatrix GrapeArray 1.2’ platform, which lacks nearly 40% of correspondence between the mapped genes. The higher numbers for our mapping can be attributed to a different mapping program and strategy used, while the differences in overlapping gene mappings in the INRA and Combimatrix arrays could be due to the need of double mapping the probeset to the corresponding tentative consensus (TC) and then to the CRIBI V1 gene prediction in the gene annotation file. This could lead to two different gene ids if the genes are similar to each other or if the TC has been wrongly annotated.

**Table 2:** Probe-to-gene mapping comparison.

Platform name	Original mapping	VESPUCCI mapping	Overlap	Missing values
NimbleGen 090918 vitus vinifera exp HX12	28811	29061	28069	3.7%
Affymetrix *V. vinifera* (grape) genome array	8581	9873	7954	66%
Affymetrix GrapeGen *V. vinifera* GrapeGena520510F	12593	13385	12200	53.9%
INRA *V. vinifera* oligo array 15 K v3	6153	6582	4795	77.3%
Combimatrix GrapeArray 1.2	8956	9193	5448	69.5%

*Total number of genes measured per platform. First column contains the microarray platform name. The second column holds the number of measured genes according to the platform original probe-to-gene mapping. The third column contains the number of measured genes according to VESPUCCI probe-to-gene mapping. The fourth column contains the number of overlapping genes between the two mappings. The last column contains the percentage of genes for which there is no measurement*.

### Sample Annotation

The *V. vinifera* gene expression compendium in VESPUCCI comes with an expansive and curated annotation of the biological conditions for all the included samples. Each sample in the compendium has been manually annotated using qualitative and quantitative terms from a controlled vocabulary specifically created for *V. Vinifera* (more information can be found in the Section “Materials and Methods” and **Supplementary Table S2**). Annotating test and reference samples to conveniently show the differences and similarities between these samples provides a useful way to assess which are the potential driving properties responsible for the observed changes in expression.

**FIGURE 4:**
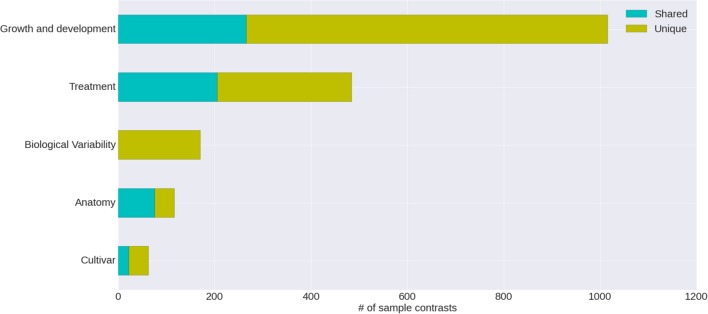
**Categories of annotated sample contrasts**. Number of sample contrasts annotated as measuring a change in one of five major categories. The differences between test and reference sample for some contrasts are related to more than one category; the proportion of these is indicated as ‘shared’ versus ‘unique.’

The condition annotation system, with its hierarchical vocabulary, provides a broad view of publicly available grapevine gene expression studies and the nature of the experiments that have been carried out (**Figure 4**). Nearly half of the VESPUCCI compendium is composed of sample contrasts measuring changes in developmental stages, particularly in the berry around véraison (Eichhorn–Lorenz stage 33–38), which is by far the most investigated topic. Together with development-related traits, biotic, and abiotic treatments also comprise a big chunk of available experiments. They include a variety of infections with several grapevine pathogens, together with temperature, water, and salinity stresses among others, while the preferred sampled tissue is fruit, as a whole or as separated parts, e.g., skin and flesh.

### *Vitis* Expression Studies Platform Using COLOMBOS Compendia Instances (VESPUCCI)

The VESPUCCI web application is a specifically designed interface to interact with the expression data, without the need for external tools or programmatic skills. It is built around the idea of expression modules. A module is a subset of the whole gene expression matrix composed by rows and columns that represent genes and sample contrasts, respectively. A set of built-in tools serves for creation and modification of modules by querying the database for genes and sample contrasts in several ways. Users can look for expression patterns starting from specific genes, conditions or whole experiments they are most interested in and extend or reduce expression modules with more genes or sample contrasts either manually or automatically relying on VESPUCCI’s clustering algorithm. Similar to a BLAST search, VESPUCCI tries to retrieve expression values for a given set of conditions, but using expression correlation instead of sequence similarity to score the best matches. Alongside tools for building and modifying modules, the web interface comprises several tools to convey information, like annotation term enrichment, the correlation network and the complete contrast annotation that display the link between changes in biological condition and gene expression. The VESPUCCI compendium is also accessible through a set of REST API calls, or from within the statistical software environment R (R Development Core Team, 2013) via the R package Rcolombos.

**FIGURE 5:**
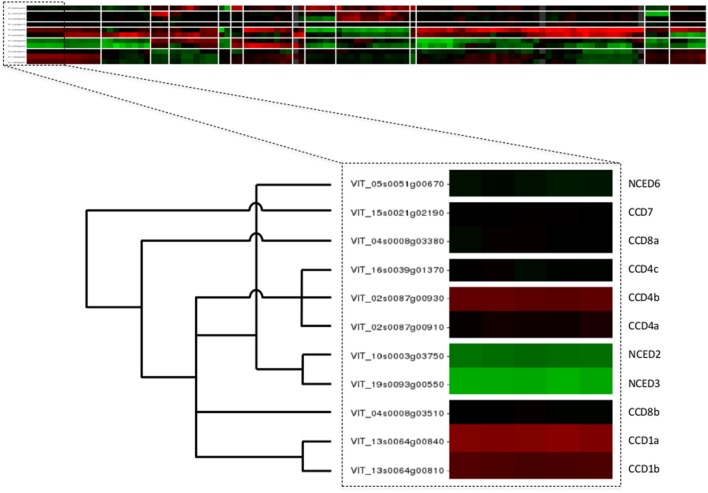
**Case study of carotenoid cleavage dioxygenases gene family**. The top part of the figure shows the VESPUCCI Quicksearch result for the 11 genes of the carotenoid cleavage dioxygenases (CCD/NCED), while the bottom depicts the superimposed phylogeny adapted from (Grimplet et al., 2014).

The web application of VESPUCCI is very much an exploratory tool to help researchers explore patterns of gene expression behavior for genes of interest. A prototype of VESPUCCI (dubbed MARCOPAOLO) has already been used to identify candidate genes involved in the fine regulation of anthocyanin and flavonol biosynthesis. In particular, co-expression with genes involved in the regulation of flavonoid biosynthesis was one of the criteria adopted to refine the list of genes identified in the genomic regions deduced by a QTL analysis for anthocyanin and flavonol content in ripe berries (Costantini et al., 2015; Malacarne et al., 2015). A co-expression analysis against VESPUCCI was also carried out to find putative interacting partners and target genes of *VvibZIPC22*, one of the candidate genes specifically associated to flavonol biosynthesis, which is being proposed as a new regulator of flavonoid biosynthesis in grapevine (Malacarne et al., 2016). While both these cases represent a ‘*guilt-by-association*’ co-expression analysis, VESPUCCI’s tools are not limited to that and are designed to encourage users to play around with data in the compendium given the biological process they are interested in. One could also query for experiments of interest instead of genes, or simply study the behavior of (a set of) genes of interest across the different biological conditions without necessarily looking for other co-expressed genes. For instance, the top part of **Figure 5** shows the results of a default Quicksearch for the 11 genes of the carotenoid cleavage dioxygenases (CCD/NCED) gene family, part of the grape carotenoid pathway (Young et al., 2012). The results of such a default search do not show all condition contrasts in the compendium, but only the top most relevant for the query genes, and can already provide insights into their behavior. First and foremost, it appears that the genes of this small gene family are not at all expressed in the same manner, and that for this particular family similarities in expression profiles are correlated up to a certain extent with the phylogenetic relationships between its genes [the superimposed tree in the bottom part of the figure is adapted from the phylogeny presented in Figure 6 of Grimplet et al. (2014)]. A deeper inspection of that behavior not only confirms previously reported results, such as up-regulation at berry ripening of CCD4a and CCD4b, but not CCD4c (Lashbrooke et al., 2013), but it also provides some novel, potentially interesting leads for further exploration. For instance, there is a prominent -but not consistent- anti-correlation of NCED2 and NCED3 with CCD4a and CCD4b. There are also strong changes in expression of some gene family members in response to *Eutypa lata* infection. These sort of observations generally only represent the initial starting point of further VESPUCCI analyses, such as investigating these genes’ behavior in other infection processes contained in the compendium, or maybe looking for co-expressed genes with NCED2/NCED3 or CCD4a/CCD4b.

**FIGURE 6:**
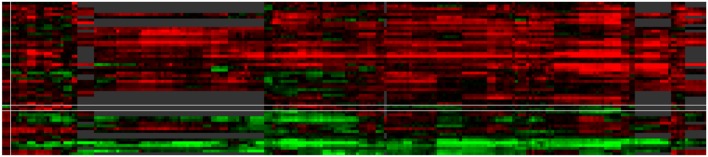
**Case study of ABA modulated genes**. The default ‘by expression’ visualization of VESPUCCI orders both genes and contrasts in this heatmap (resp. rows and columns) in such a way as to highlight the different patterns of condition-dependent gene expression behavior.

For an in-depth illustration of these concepts, we have included another case study in the website as well, which is presented there as a detailed step-by-step tutorial with the ability to load associated data directly in the interface. This particular case study is meant to show off VESPUCCI’s most common features and capabilities in a hands-on manner. It focuses on a set of genes found to be modulated by the phytohormone abscisic acid (ABA) in pre-véraison berries (Stefania Pilati, personal communication); this list of genes was used as input to query the database. After performing any database query, VESPUCCI creates an expression ‘module,’ a subset of the whole expression compendium determined by a set of genes and a set of sample contrasts and the corresponding expression values. The returned gene expression module indicated that the 55 ABA genes appear highly modulated in 353 experimental conditions in the VESPUCCI compendium. The default visualization of this module (‘by expression’; **Figure 6**) emphasizes the interesting patterns of condition-dependent (anti-)co-expression behavior among this set of ABA genes. The gene annotation enrichment in turn reports their involvement in the response to stress and ABA, as well as in galactose metabolism. The main biological processes represented in our module, correspond to different biological contexts in which ABA affects gene expression: fruit and berry development, bud development, and water and salinity stress. The explorative purpose of the web-interface is strengthened by tools used to modify the module by extending (or shrinking) it with new genes and/or contrasts. Continuing the analysis, the module was split according to these three biological processes, and these sub-modules then formed the basis for new queries to include additional genes with highly similar (or opposite) expression profiles in these three specific biological contexts. The final lists of (anti)-co-expressed genes are candidates for being involved in the pathways regulated by ABA, and/or for sharing similar, but currently unannotated mechanisms of regulation with the genes in the module.

## Discussion

In this paper, we present VESPUCCI, a gene expression compendium for grapevine that integrates publicly available transcriptomics data from several microarray and RNA-Seq platforms into one coherent database, queryable via a web or REST interface. The web interface is meant to be intuitive and flexible for non-expert users, and is designed to encourage them to ‘play around’ with the data in the compendium, centering on the biological processes and/or genes they are interested in. In that sense, it is very much an exploratory tool, meant to assist more dedicated research in grapevine genomics, biology, and physiology, even if the integration of over 1500 transcriptomics samples into a single data set can be quite powerful in and of itself. The case studies presented in the results are examples of the type of analyses that can be done with VESPUCCI, and the sort of insights that can be gained from the combined data in the compendium. They all represent cases where VESPUCCI shows interesting modular gene expression responses that were not known previously, whether from the individual experiments included in the compendium, from published papers, or from other, independent (even non-transcriptomics) experiments or sources of information.

In contrast to model organisms for which available -omics experiments are considerable, crop species usually lack of a substantial amount of data. Nevertheless, there is an increasing interest for a more systemic view of crop species (Yin and Struik, 2010; Sheth and Thaker, 2014), driven by the ever-decreasing cost of high-throughput technologies and the development of new analysis tools. The availability of transcriptomics technology has increased substantially during recent years. Nowadays, RNA-Seq experiments enable scientists to reliably measure the majority of expressed genes. However, during the early days of transcriptomics, microarray measurements often comprised only a part of the complete transcriptome. The end result is that across the entire VESPUCCI gene expression compendium, the proportion of missing values is substantial (36%). Even though the great majority of samples have been measured using the Nimblegen or RNA-Seq technologies which can both cover the near complete transcriptome, the probes of the other microarray platforms are not able to provide measurements for as many genes. This is irremediable and intrinsic to the source data. We dealt with it by attempting to provide optimal, as reliable as possible expression measurements across the compendium, both at the level of the actual probe-to-gene mapping, as well as at the level of defining of the list of measurable gene transcripts.

These measurable transcripts (representing the rows of the gene expression matrix) incur some limitations in and of themselves as they are entirely based on the gene predictions for *V. vinifera* cv. Pinot Noir, with implications for experiments done on other cultivars. When microarray experiments are performed to measure expression for a specific cultivar with platforms containing probes designed from different cultivars, this generally leads to poorer signals, given the impossibility to distinguish expression variability from genomic differences among those cultivars. The reason is the lack of available high-quality gene predictions for each cultivar. While RNA-Seq has the advantage of enhancing its value over time with better genomes and gene annotations by re-doing the transcriptome mapping on the appropriate cultivar, the situation is more complicated for microarray data. The solution is never ideal as nothing can be done to increase the quality of intensity signals if there is a mismatch between the cultivar used to design the probes, and the one used to do the experiment. Nevertheless, remapping the microarray probes on the cultivar-specific genes of the experiment would improve the gene annotation of the array platform and ensure only the reliable probes are considered to generate the final expression values. A novelty in the latest release of COLOMBOS is the option to explicitly recognize genomic differences between strains or cultivars instead of using a single reference genome to represent a species. This improves read alignment (RNA-Seq) or probe-to-gene mapping (microarrays) and generates higher quality expression data. In the long term, as more grapevine cultivar genomes become available, we can rely on these COLOMBOS innovations to build compendia for different cultivars and integrate them at the species level using homolog mappings, creating a proper ‘meta-compendium’ for grapevine varieties.

Currently VESPUCCI is limited to our knowledge of the *V. vinifera* cv. Pinot Noir genome, and despite the existence of a more recent version of the CRIBI gene prediction (Vitulo et al., 2014), we decided to keep V1 as the basis for this first release. From a practical perspective, by the time V2 was made publicly available, most of the compendium was already built and the switch to the newer version didn’t show a significant increase quality-wise. The great majority of genes does not change in terms of gene structure, and as such for our purposes the end result was largely unaffected by the enhancements of the newer version over V1. Nevertheless, as the number of experiments (especially RNA-Seq) increases, the benefits of relying on V2 will become more prominent; for future VESPUCCI releases, we will most likely shift toward V2 (or more recent versions) to take advantage of the extended UTR regions for which NGS technologies provide better measurements.

The measurable gene transcripts that, we defined do not correspond one-to-one to the CRIBI gene predictions, but instead contain some ‘gene clusters.’ Expression data for these gene clusters are a compromise between our ability to measure each and every single gene individually, and how many genes can be reliably measured in total. While not absolute proof that these probes are unable to adequately distinguish the intended target genes, our results (**Figures 1** and **2**, and Supplementary Materials) showed that it is almost impossible to measure differences between each single gene in the clusters. This supports our decision to throw them together: even if these probes were capable of capturing different transcripts, the results do not indicate that this was the case for the more than 500 Nimblegen sample contrasts in the compendium. Therefore, instead of discarding the shared probes and lose potentially valuable information, we accepted the impossibility to unambiguously discriminate each and every single gene, gaining the opportunity to have a single measurement for those gene transcripts as a whole. Note that while the issue itself is (microarray) platform specific, the proposed ‘gene clusters’ are not. We chose to define them based on the platform with the highest data representation: the Nimblegen platform holds the largest number of samples as well as the highest quality of probe-to-gene mapping. This has no detrimental effect on data from the other microarray platforms, but RNA-Seq technology can provide individual gene measurements for at least some of our defined clusters (given that the corresponding gene sequences show enough dissimilarity). Due to the current low number of RNA-Seq experiments compared to the Nimblegen ones, we decided on clustering genes together in measurable sets to get the best out of all the data as a whole. As soon as RNA-Seq experiments will be more prevalent, we will revise the gene clusters to gain the ability to measure more genes separately for RNA-Seq, at the expense of losing the corresponding probes on the Nimblegen platform.

VESPUCCI includes nearly all of the gene expression data that is publicly available for grapevine at the moment; it provides a snapshot of the current situation of transcriptomics experiments performed. We’re planning to keep it up to date by releasing yearly content updates. In the current release, berry development studies are the most represented experiments (especially during véraison) and this comes with no surprise given the importance of fruit quality in wine and spirits’ production. This will be all the more obvious when mining for genes related to fruit ripening. Given the complexity of this developmental process, in which the fruit undergoes radical phenotypic and biochemical modifications (related to shape, size, color, sugar and aroma content, etc.), the number of modulated genes is quite big. VESPUCCI is meant as an exploratory tool to help researcher not only in finding patterns of gene expression for genes of interest, but also to aid the design of new experiments providing the most complete transcriptomics information currently available.

## Author Contributions

MM and KE conceived the work, implemented the procedures, analyzed the data, and wrote the manuscript; KE supervised the work. MM, PS, and KE collected and processed the data. MM, PS, SP, GM, LC, LG, GB, SG, CM, and KE built the controlled vocabulary, did the meta-data annotation, beta-tested the application, provided the case studies, and revised and edited the manuscript.

## Conflict of Interest Statement

The authors declare that the research was conducted in the absence of any commercial or financial relationships that could be construed as a potential conflict of interest.

## Footnotes

1 http://www.ncbi.nlm.nih.gov/sra

2 http://genomes.cribi.unipd.it/DATA/V1/

3 http://www.plantontology.org/

4 http://www.sdstate.edu/ps/research/vitis/pathways.cfm

## Supplementary material

FIGURE S1 **Probe correlation heatmaps using Pearson uncentered correlation of all 466 gene clusters**.

FIGURE S2 **Probe-to-gene alignment graphs of all 466 gene clusters**.

FIGURE S3 **Probe-level expression measured across more than 500 Nimblegen sample contrasts sorted by values of all 466 gene clusters**.

Table S1 **Complete list of imported experiments and platform in VESPUCCI**.

Table S2 **Complete controlled vocabulary terms and structure used for the annotation of sample contrasts**.
