# Supplementary material for: VESPUCCI: Exploring Patterns of Gene Expression in Grapevine
Source: Front Plant Sci. 2016 May 10;7:633. doi: 10.3389/fpls.2016.00633 (PMC4862315; doi:10.3389/fpls.2016.00633)
Supplement: FIGURE S1 — Probe correlation heatmaps using Pearson uncentered correlation of all 466 gene clusters. [file Image_1.PDF]

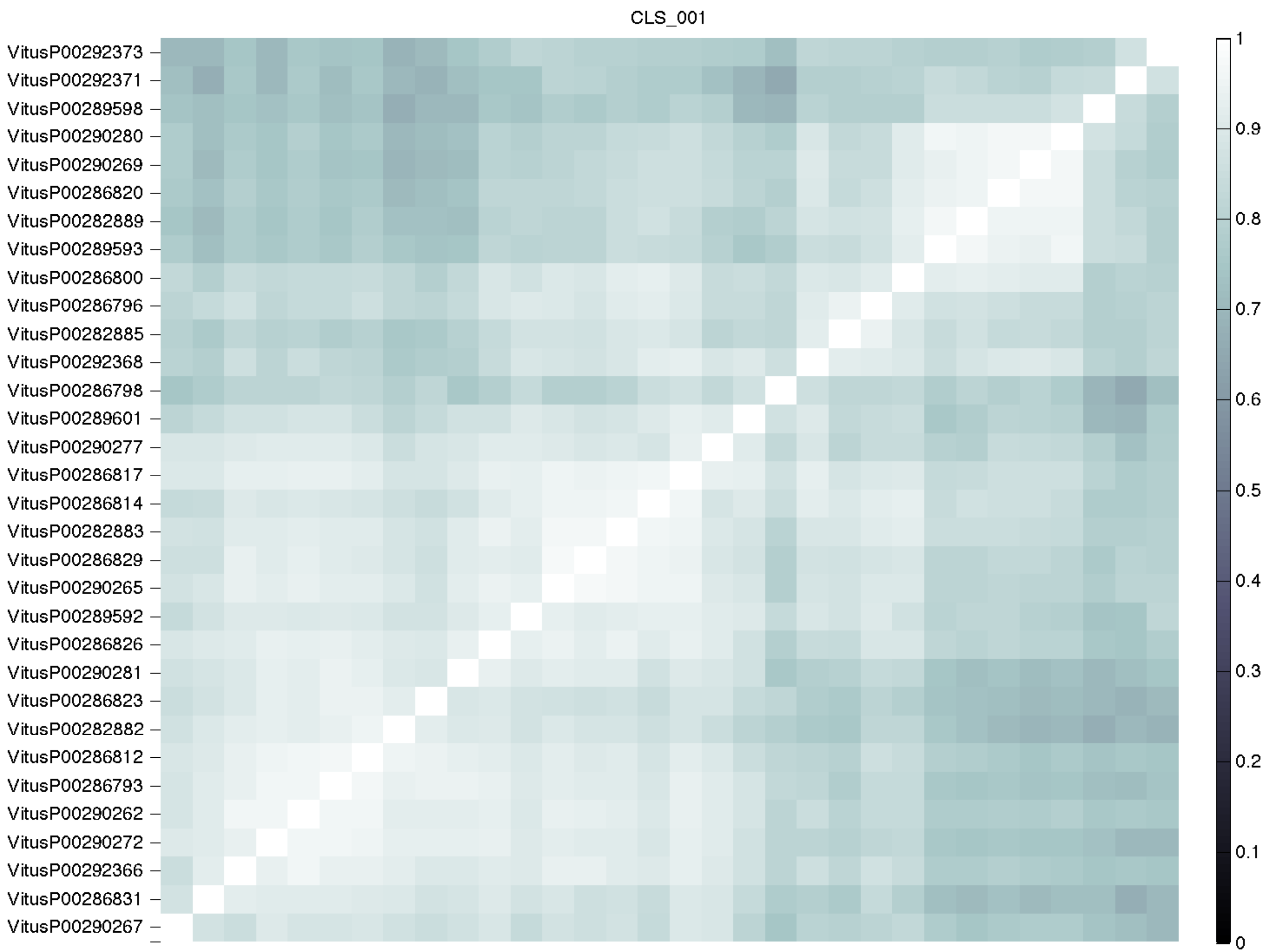
CLS_001
VitusP00292373
VitusP00292371
VitusP00289598
VitusP00290280
VitusP00290269
VitusP00286820
VitusP00282889
VitusP00289593
VitusP00286800
VitusP00286796
VitusP00282885
VitusP00292368
VitusP00286798
VitusP00289601
VitusP00290277
VitusP00286817
VitusP00286814
VitusP00282883
VitusP00286829
VitusP00290265
VitusP00289592
VitusP00286826
VitusP00290281
VitusP00286823
VitusP00282882
VitusP00286812
VitusP00286793
VitusP00290262
VitusP00290272
VitusP00292366
VitusP00286831
VitusP00290267
1
0.9
0.8
0.7
0.6
0.5
0.4
0.3
0.2
0.1
0

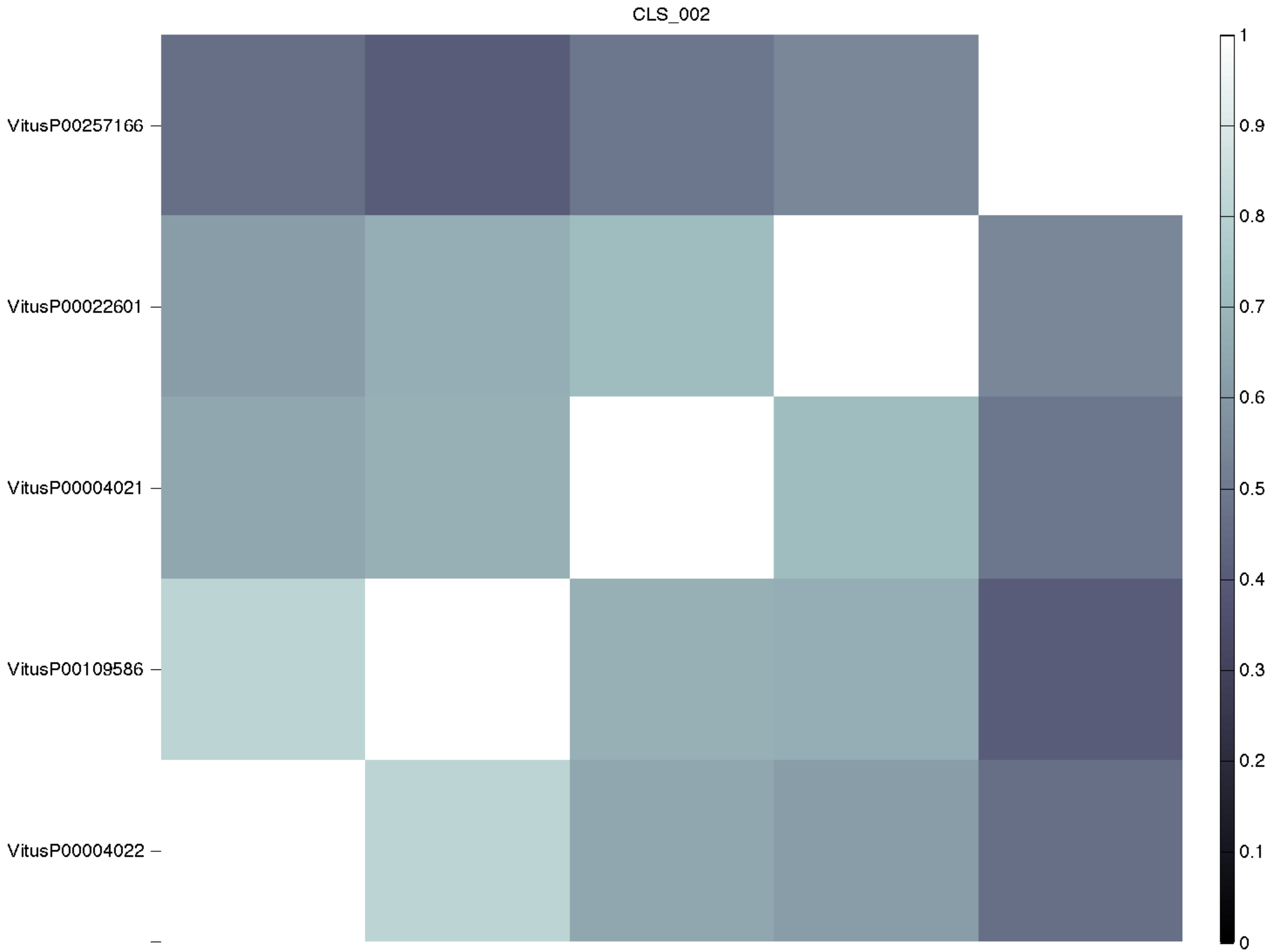
CLS_002
VitusP00257166
VitusP00022601
VitusP00004021
VitusP00109586
VitusP00004022
1
0.9
0.8
0.7
0.6
0.5
0.4
0.3
0.2
0.1
0

CLS_003

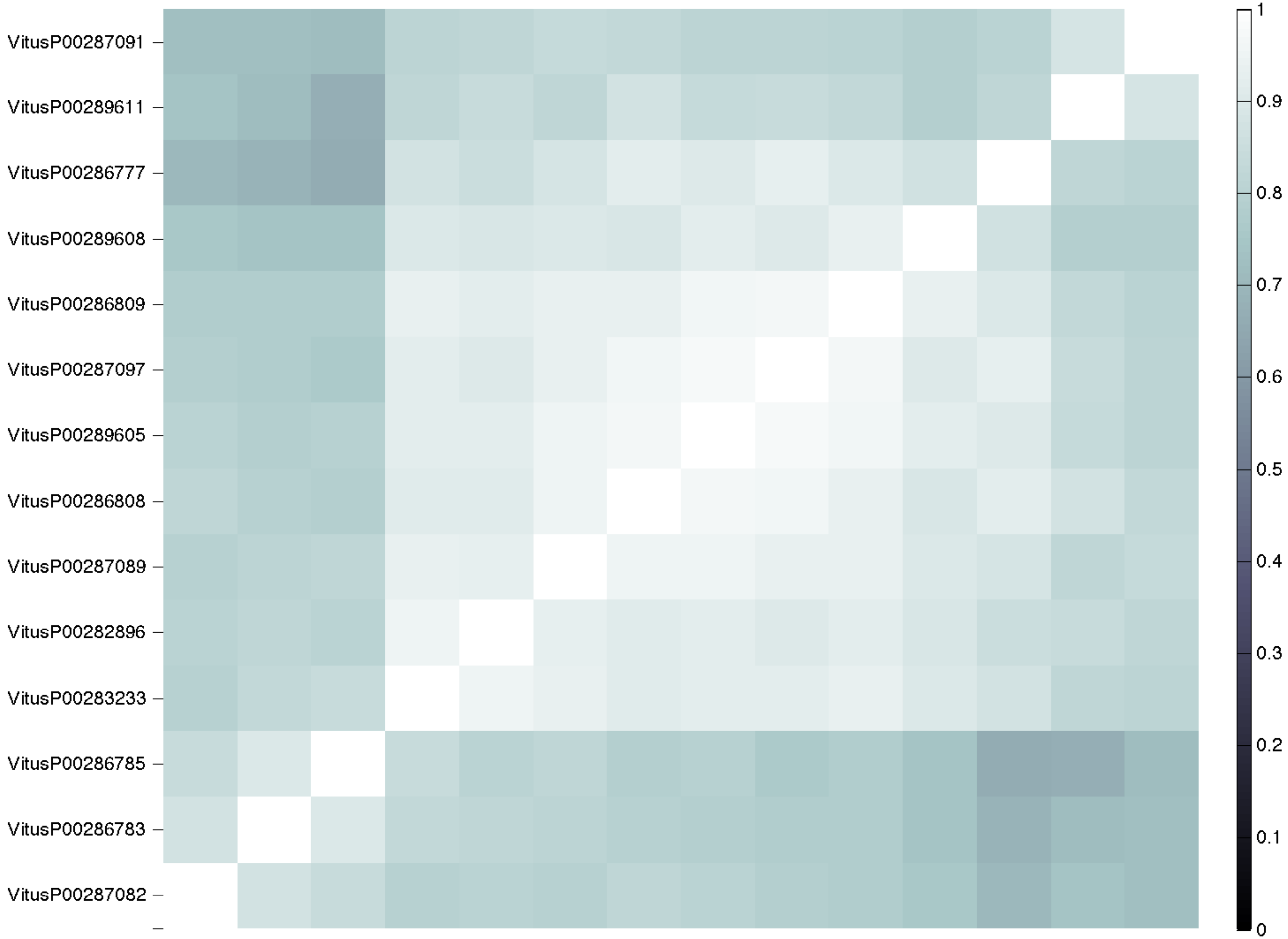

VitusP00287091
VitusP00289611
VitusP00286777
VitusP00289608
VitusP00286809
VitusP00287097
VitusP00289605
VitusP00286808
VitusP00287089
VitusP00282896
VitusP00283233
VitusP00286785
VitusP00286783
VitusP00287082
1
0.9
0.8
0.7
0.6
0.5
0.4
0.3
0.2
0.1
0

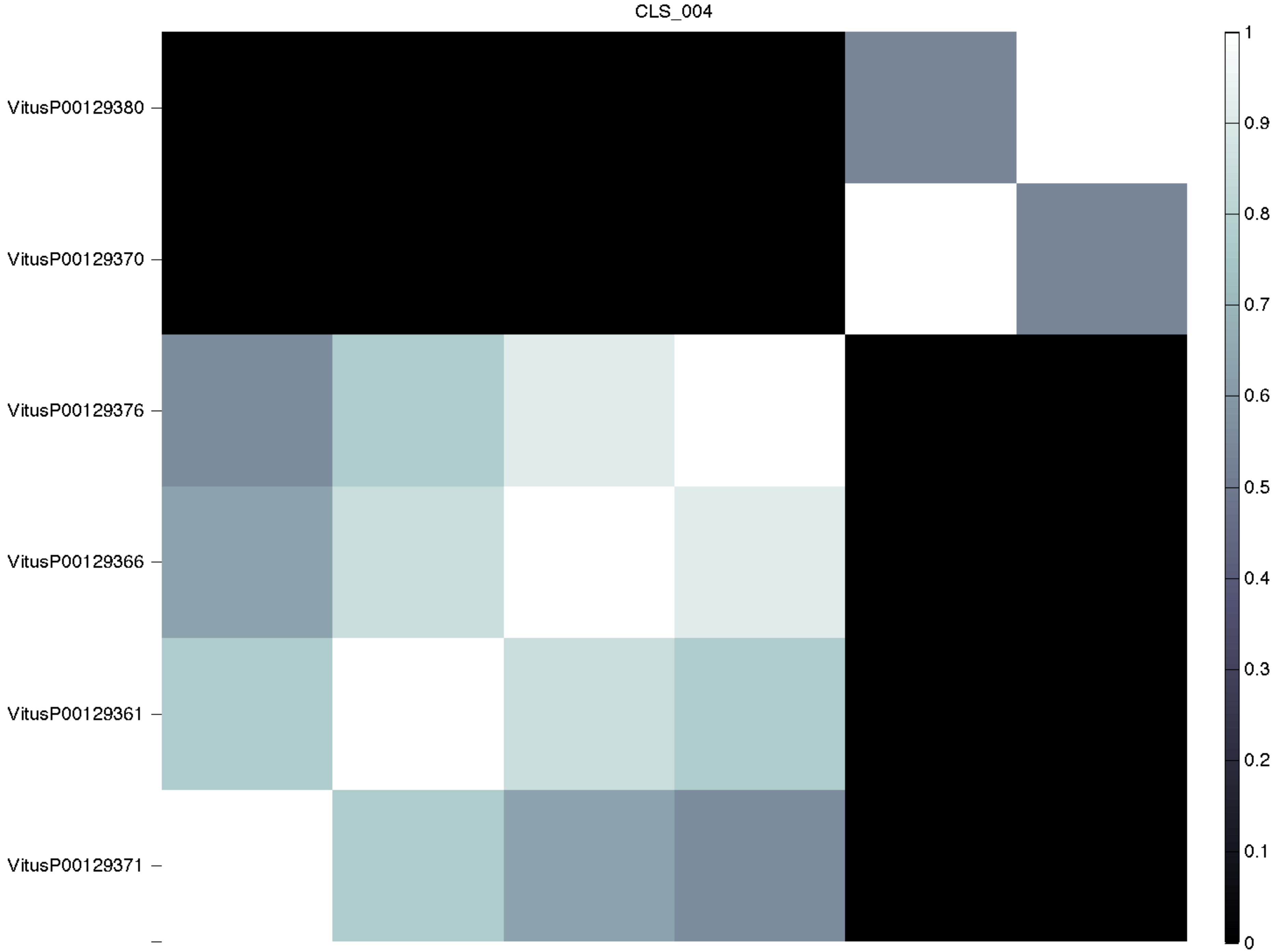
CLS_004
VitusP00129380
VitusP00129370
VitusP00129376
VitusP00129366
VitusP00129361
VitusP00129371
1
0.9
0.8
0.7
0.6
0.5
0.4
0.3
0.2
0.1
0

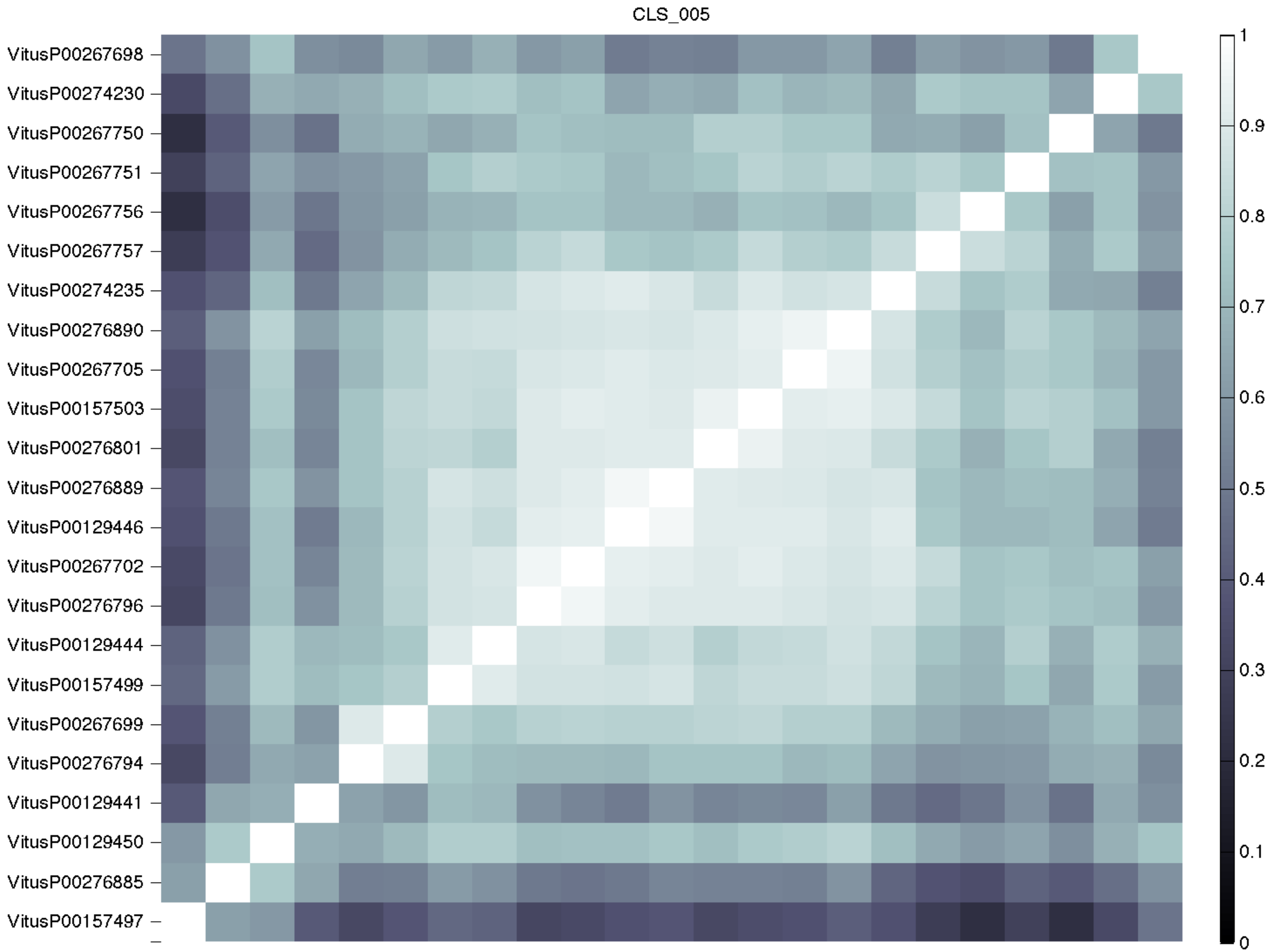
CLS_005
VitusP00267698
VitusP00274230
VitusP00267750
VitusP00267751
VitusP00267756
VitusP00267757
VitusP00274235
VitusP00276890
VitusP00267705
VitusP00157503
VitusP00276801
VitusP00276889
VitusP00129446
VitusP00267702
VitusP00276796
VitusP00129444
VitusP00157499
VitusP00267699
VitusP00276794
VitusP00129441
VitusP00129450
VitusP00276885
VitusP00157497
1
0.9
0.8
0.7
0.6
0.5
0.4
0.3
0.2
0.1
0

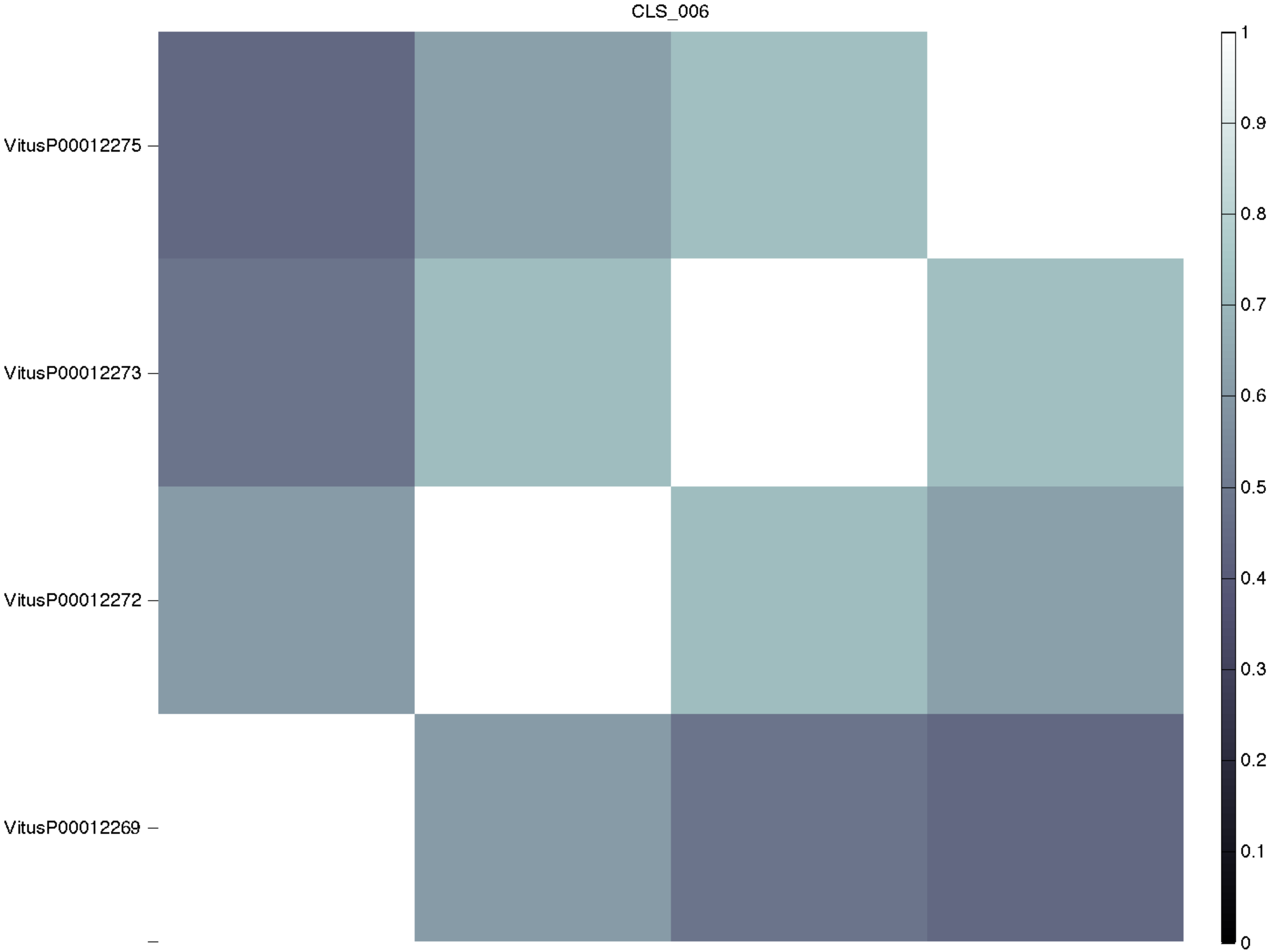

CLS_006
VitusP00012275
VitusP00012273
VitusP00012272
VitusP00012269
1
0.9
0.8
0.7
0.6
0.5
0.4
0.3
0.2
0.1
0

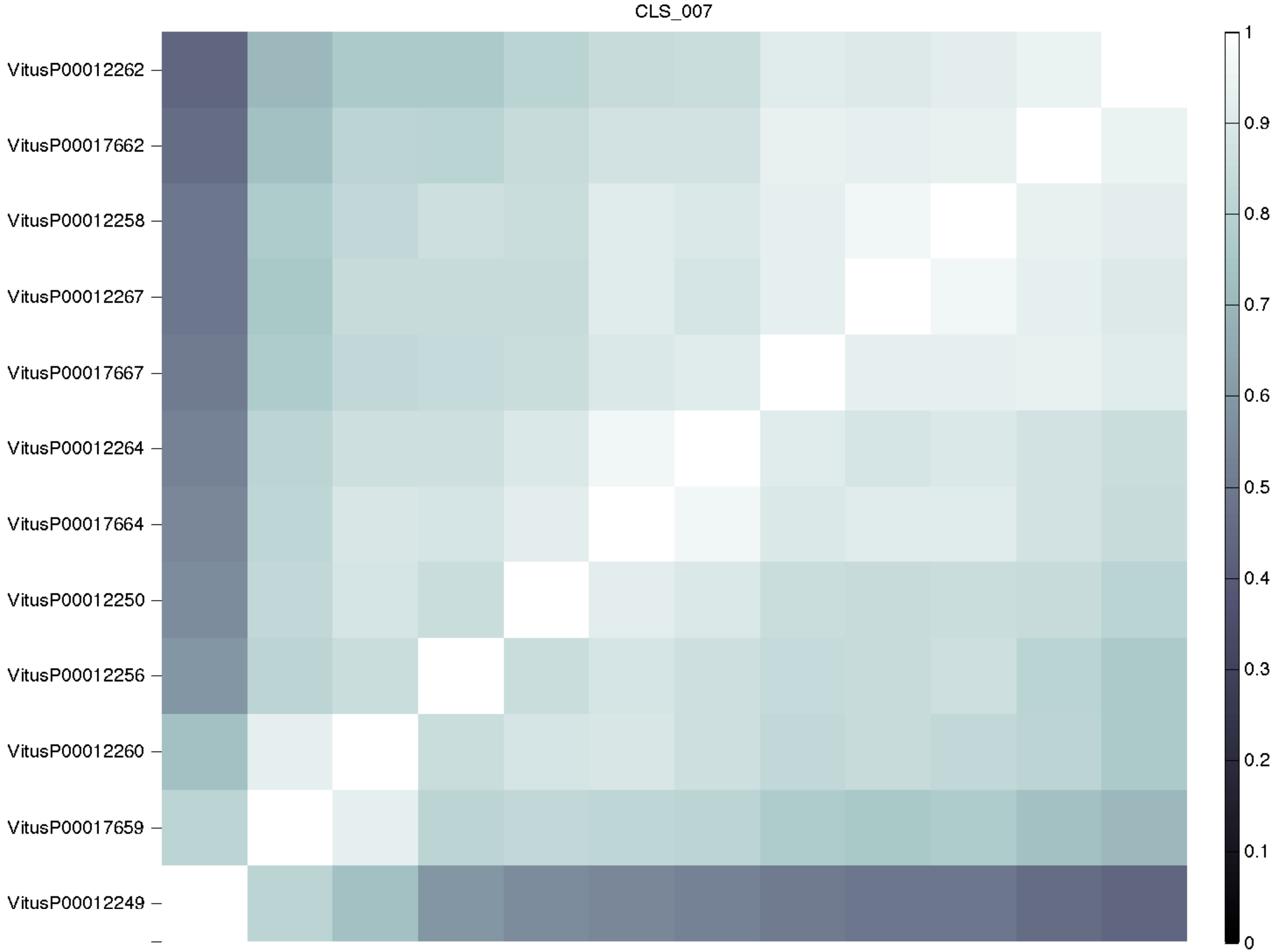
CLS_007
VitusP00012262
VitusP00017662
VitusP00012258
VitusP00012267
VitusP00017667
VitusP00012264
VitusP00017664
VitusP00012250
VitusP00012256
VitusP00012260
VitusP00017659
VitusP00012249
1
0.9
0.8
0.7
0.6
0.5
0.4
0.3
0.2
0.1
0

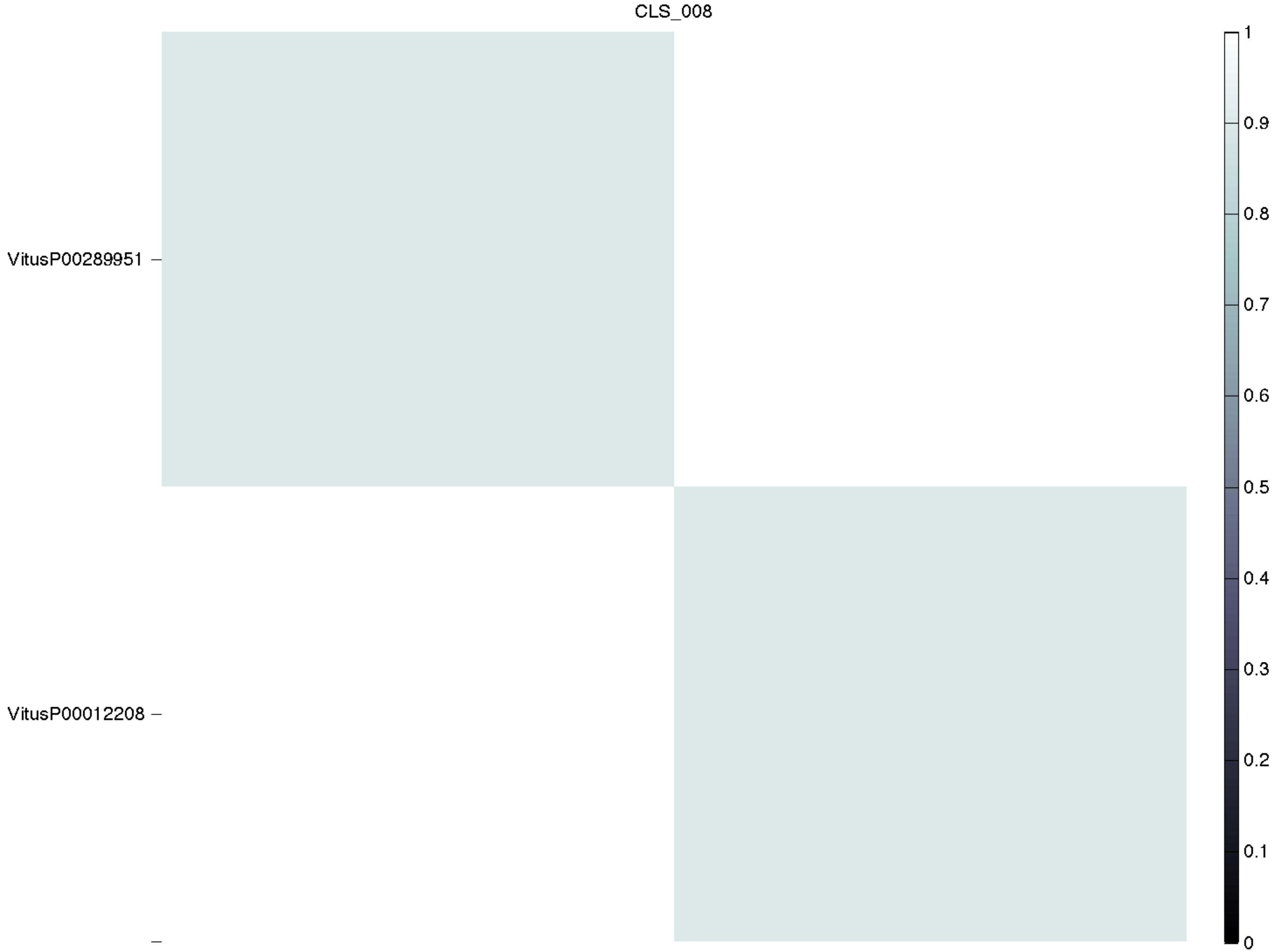

CLS_008
VitusP00289951
VitusP00012208
1
0.9
0.8
0.7
0.6
0.5
0.4
0.3
0.2
0.1
0

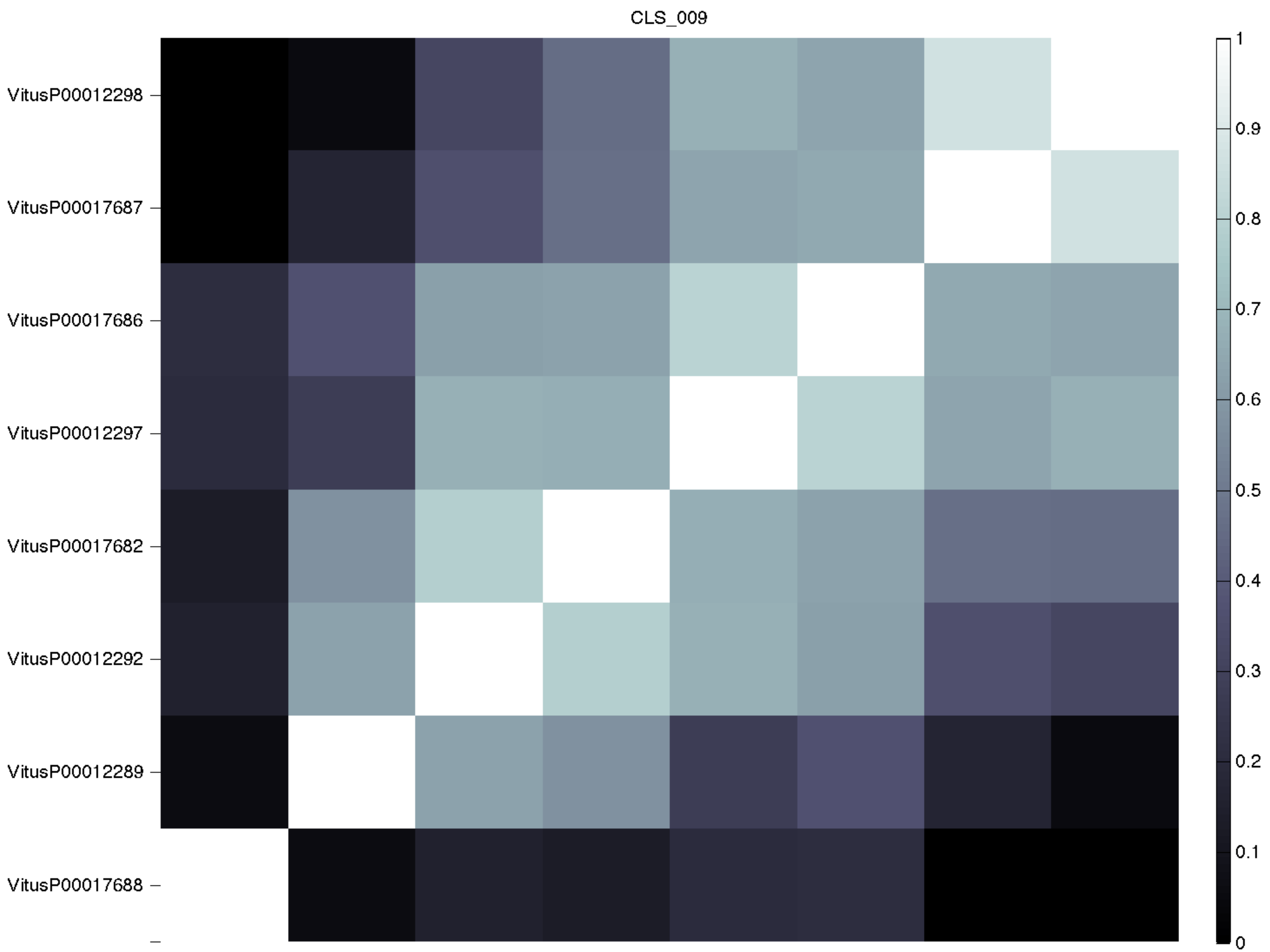

CLS_009
VitusP00012298
VitusP00017687
VitusP00017686
VitusP00012297
VitusP00017682
VitusP00012292
VitusP00012289
VitusP00017688
1
0.9
0.8
0.7
0.6
0.5
0.4
0.3
0.2
0.1
0

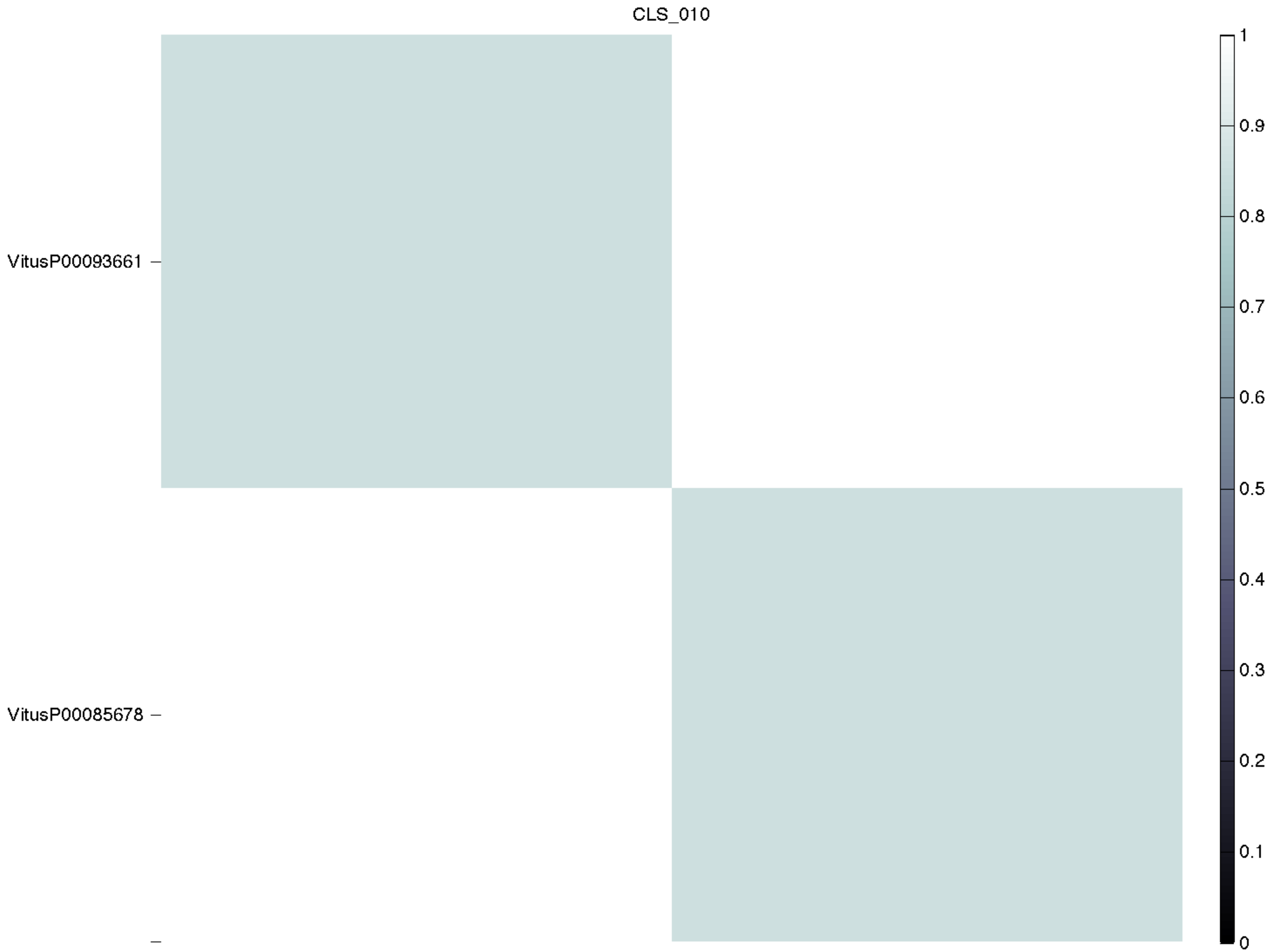

CLS_010
VitusP00093661
VitusP00085678
1
0.9
0.8
0.7
0.6
0.5
0.4
0.3
0.2
0.1
0

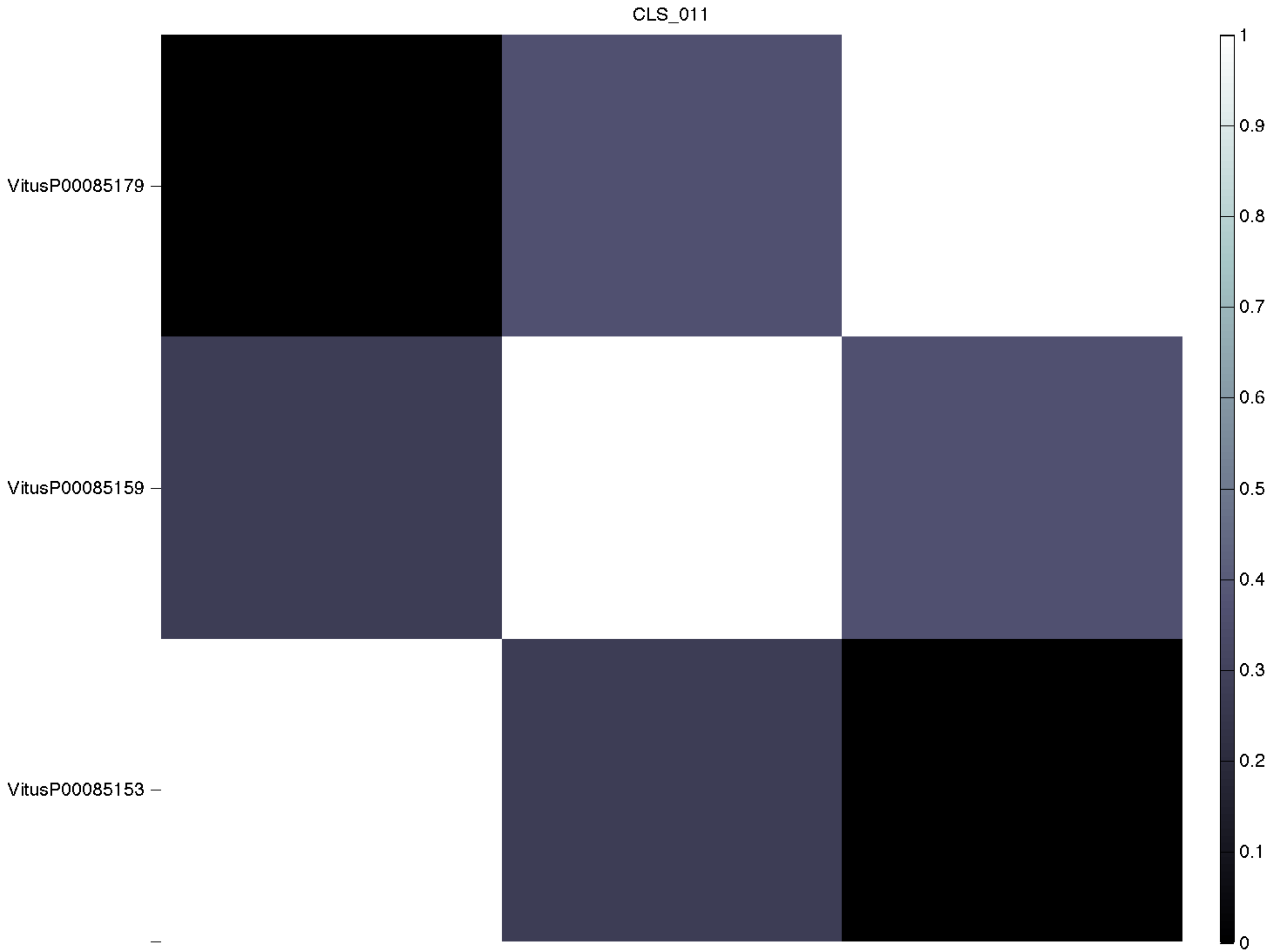
CLS_011
VitusP00085179
VitusP00085159
VitusP00085153
1
0.9
0.8
0.7
0.6
0.5
0.4
0.3
0.2
0.1
0

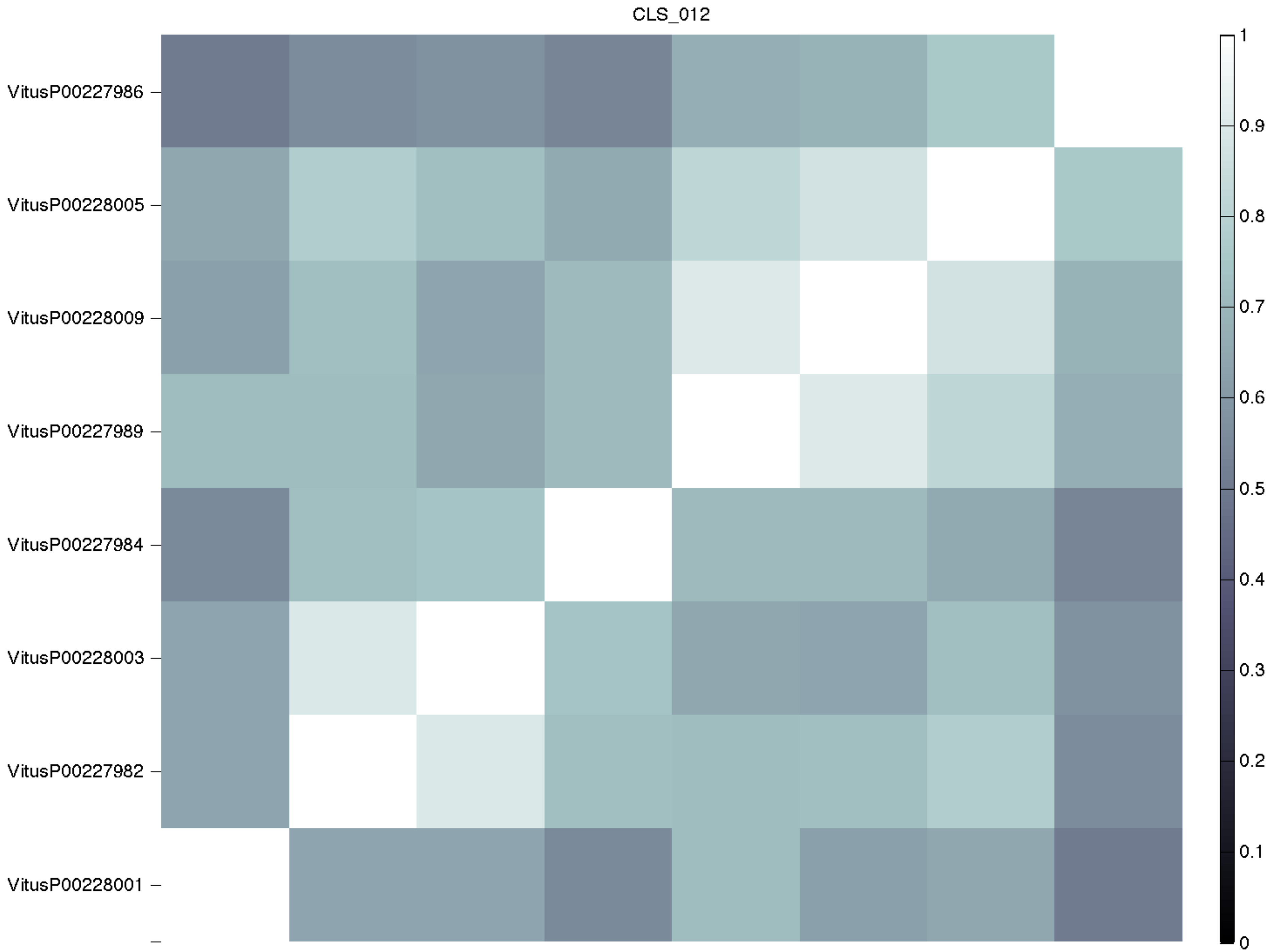

CLS_012
VitusP00227986
VitusP00228005
VitusP00228009
VitusP00227989
VitusP00227984
VitusP00228003
VitusP00227982
VitusP00228001
1
0.9
0.8
0.7
0.6
0.5
0.4
0.3
0.2
0.1
0

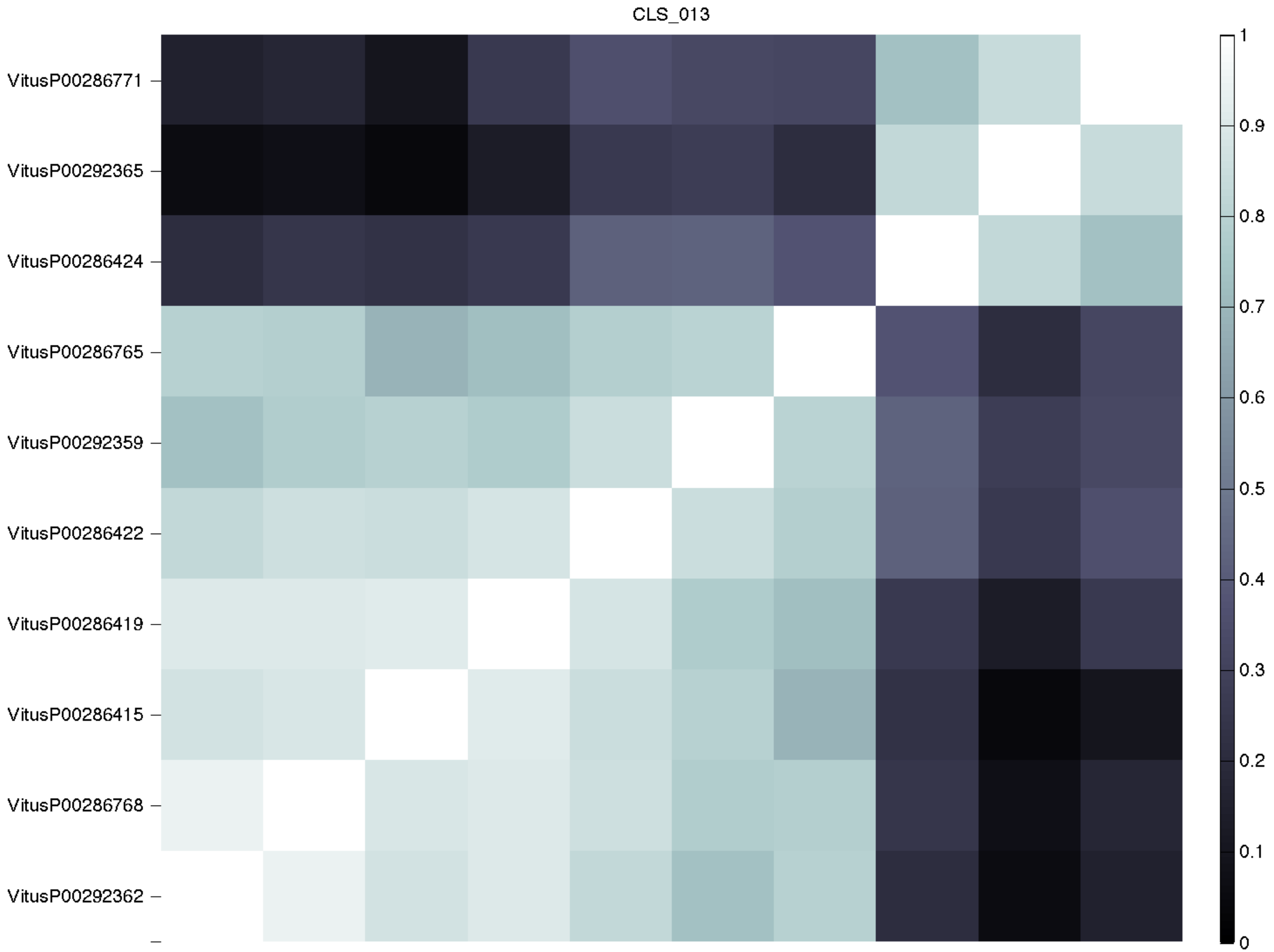
CLS_013
VitusP00286771
VitusP00292365
VitusP00286424
VitusP00286765
VitusP00292359
VitusP00286422
VitusP00286419
VitusP00286415
VitusP00286768
VitusP00292362
1
0.9
0.8
0.7
0.6
0.5
0.4
0.3
0.2
0.1
0

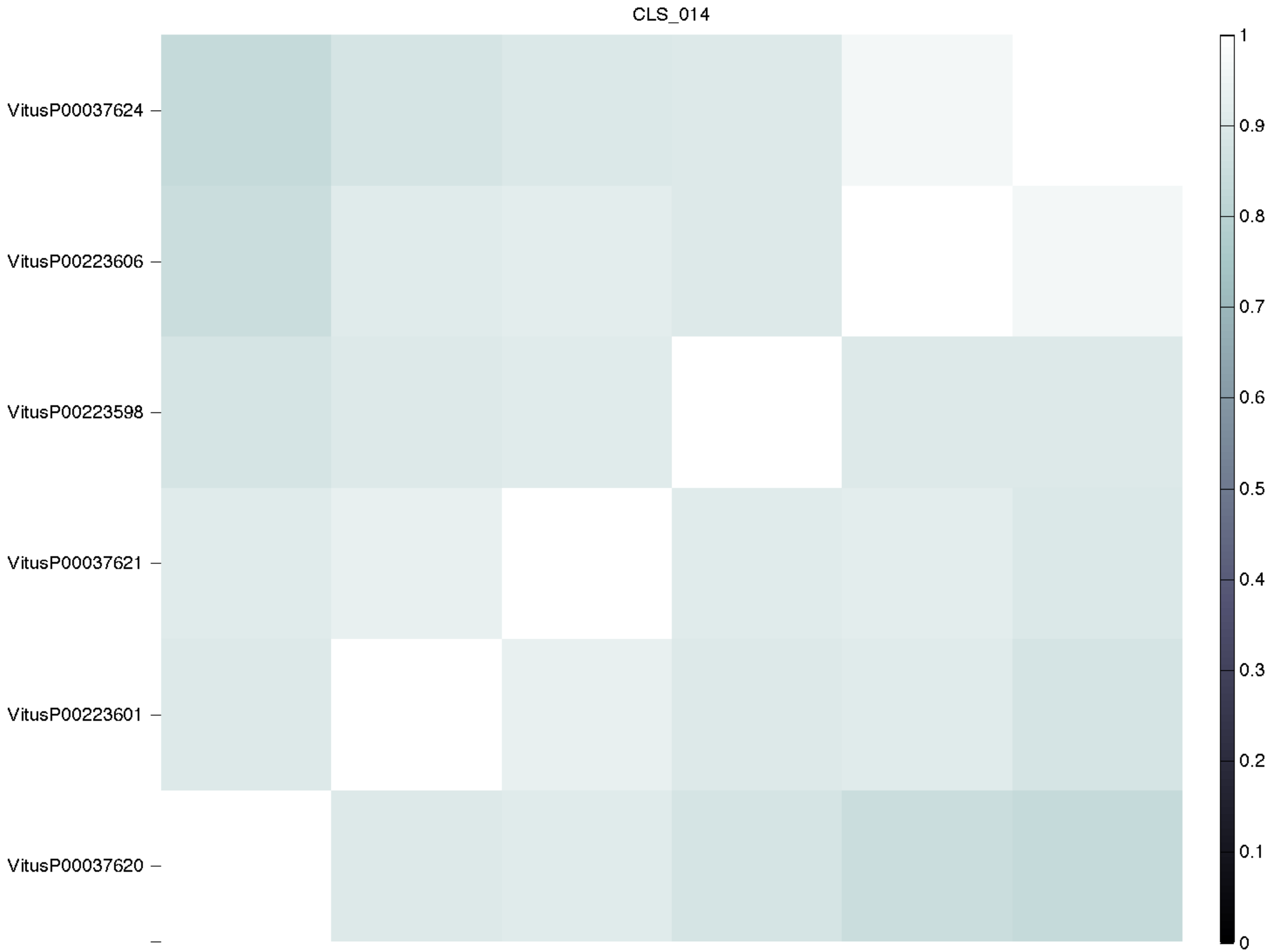
CLS_014
VitusP00037624
VitusP00223606
VitusP00223598
VitusP00037621
VitusP00223601
VitusP00037620
1
0.9
0.8
0.7
0.6
0.5
0.4
0.3
0.2
0.1
0

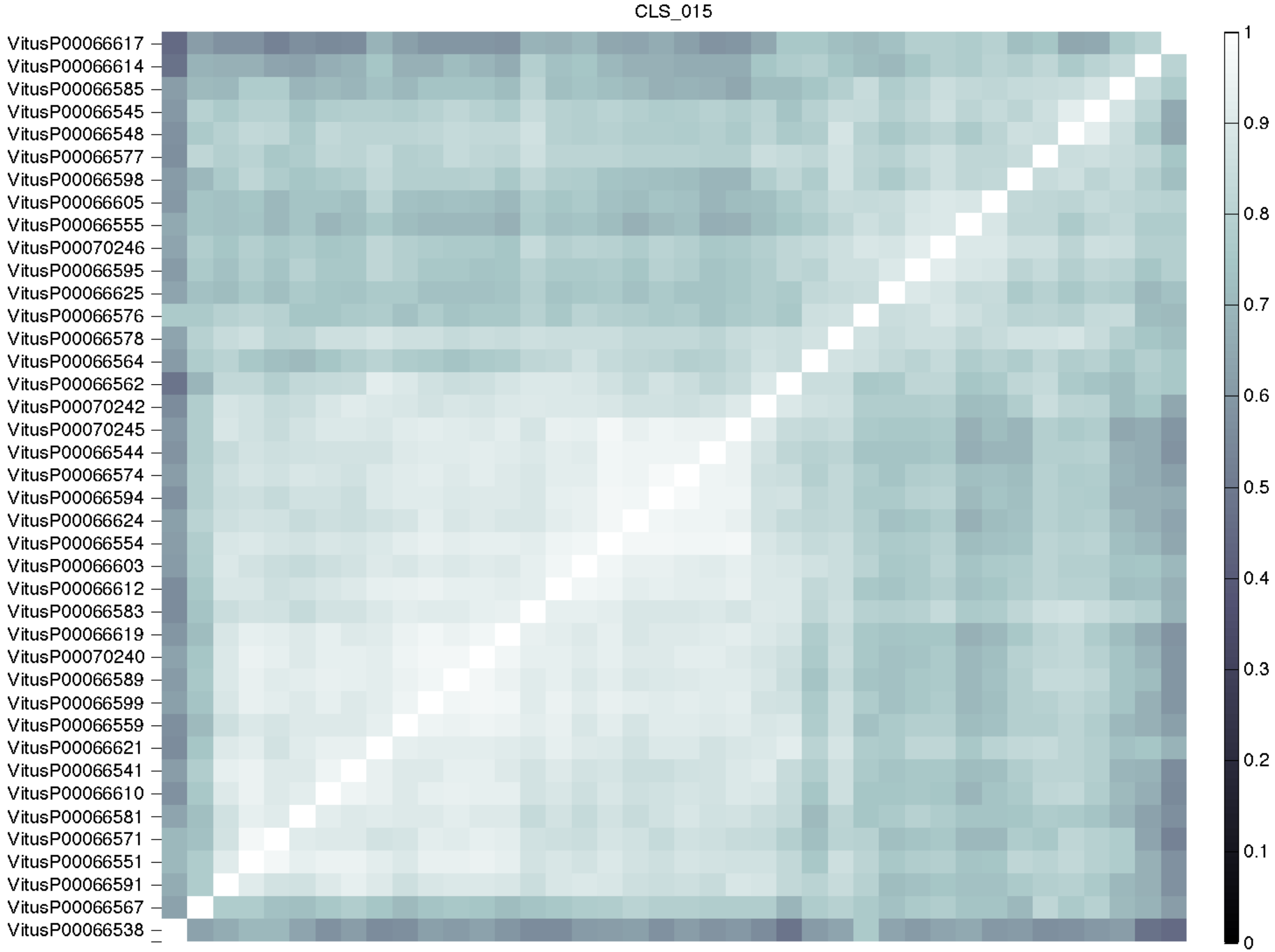
CLS_015
VitusP00066617
VitusP00066614
VitusP00066585
VitusP00066545
VitusP00066548
VitusP00066577
VitusP00066598
VitusP00066605
VitusP00066555
VitusP00070246
VitusP00066595
VitusP00066625
VitusP00066576
VitusP00066578
VitusP00066564
VitusP00066562
VitusP00070242
VitusP00070245
VitusP00066544
VitusP00066574
VitusP00066594
VitusP00066624
VitusP00066554
VitusP00066603
VitusP00066612
VitusP00066583
VitusP00066619
VitusP00070240
VitusP00066589
VitusP00066599
VitusP00066559
VitusP00066621
VitusP00066541
VitusP00066610
VitusP00066581
VitusP00066571
VitusP00066551
VitusP00066591
VitusP00066567
VitusP00066538
1
0.9
0.8
0.7
0.6
0.5
0.4
0.3
0.2
0.1
0

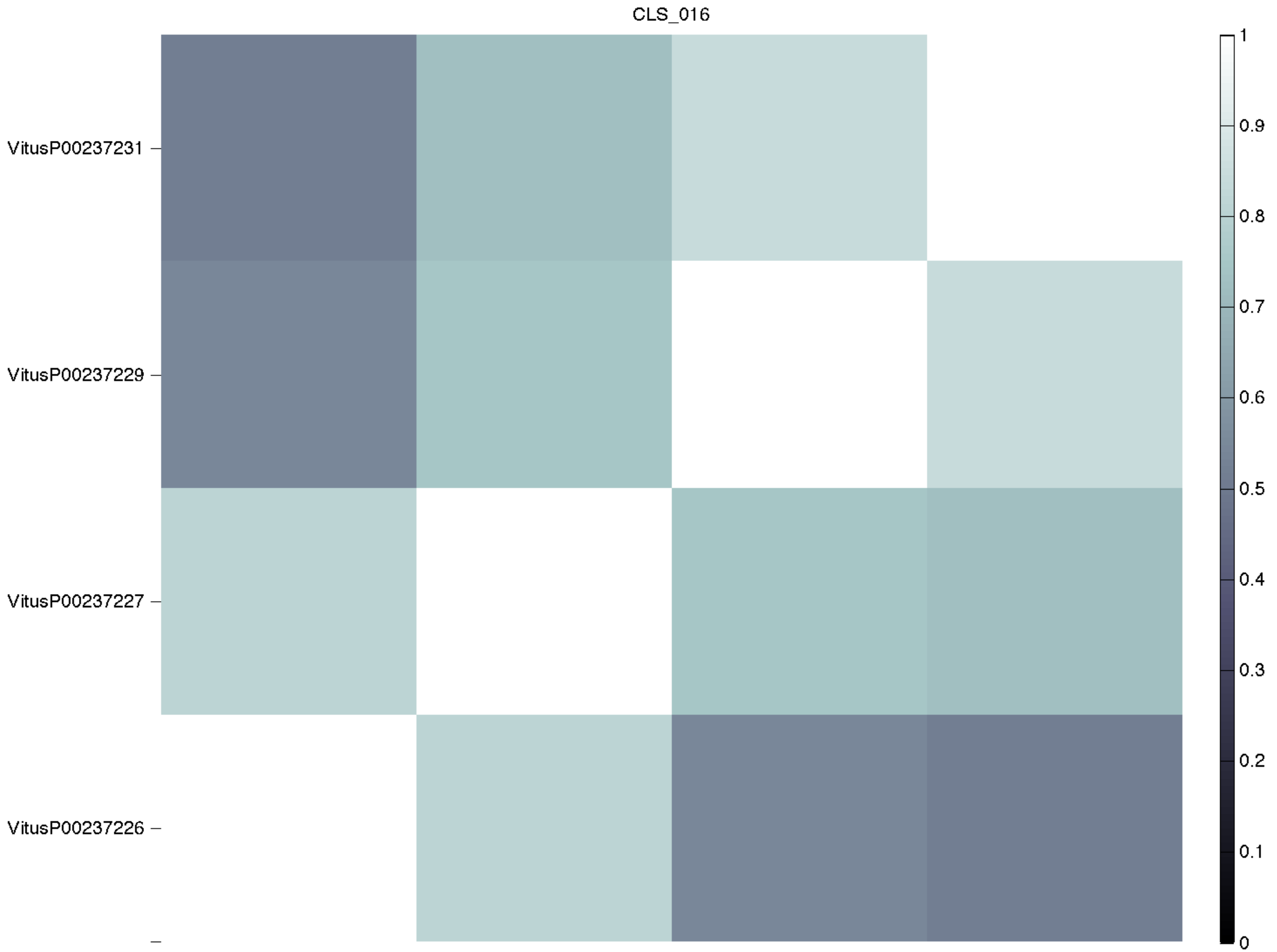
CLS_016
VitusP00237231
VitusP00237229
VitusP00237227
VitusP00237226
1
0.9
0.8
0.7
0.6
0.5
0.4
0.3
0.2
0.1
0

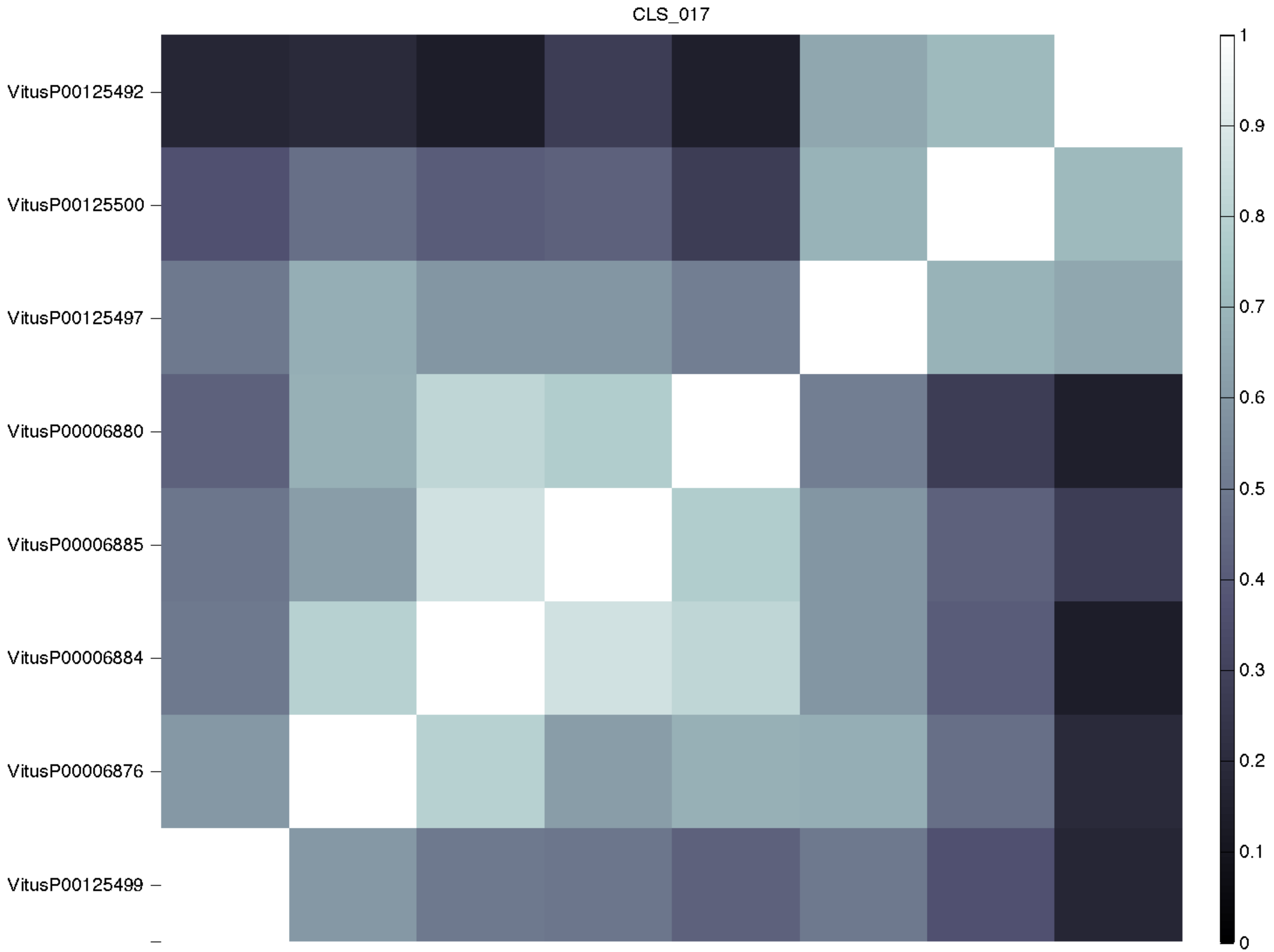

CLS_017
VitusP00125492
VitusP00125500
VitusP00125497
VitusP00006880
VitusP00006885
VitusP00006884
VitusP00006876
VitusP00125499
1
0.9
0.8
0.7
0.6
0.5
0.4
0.3
0.2
0.1
0

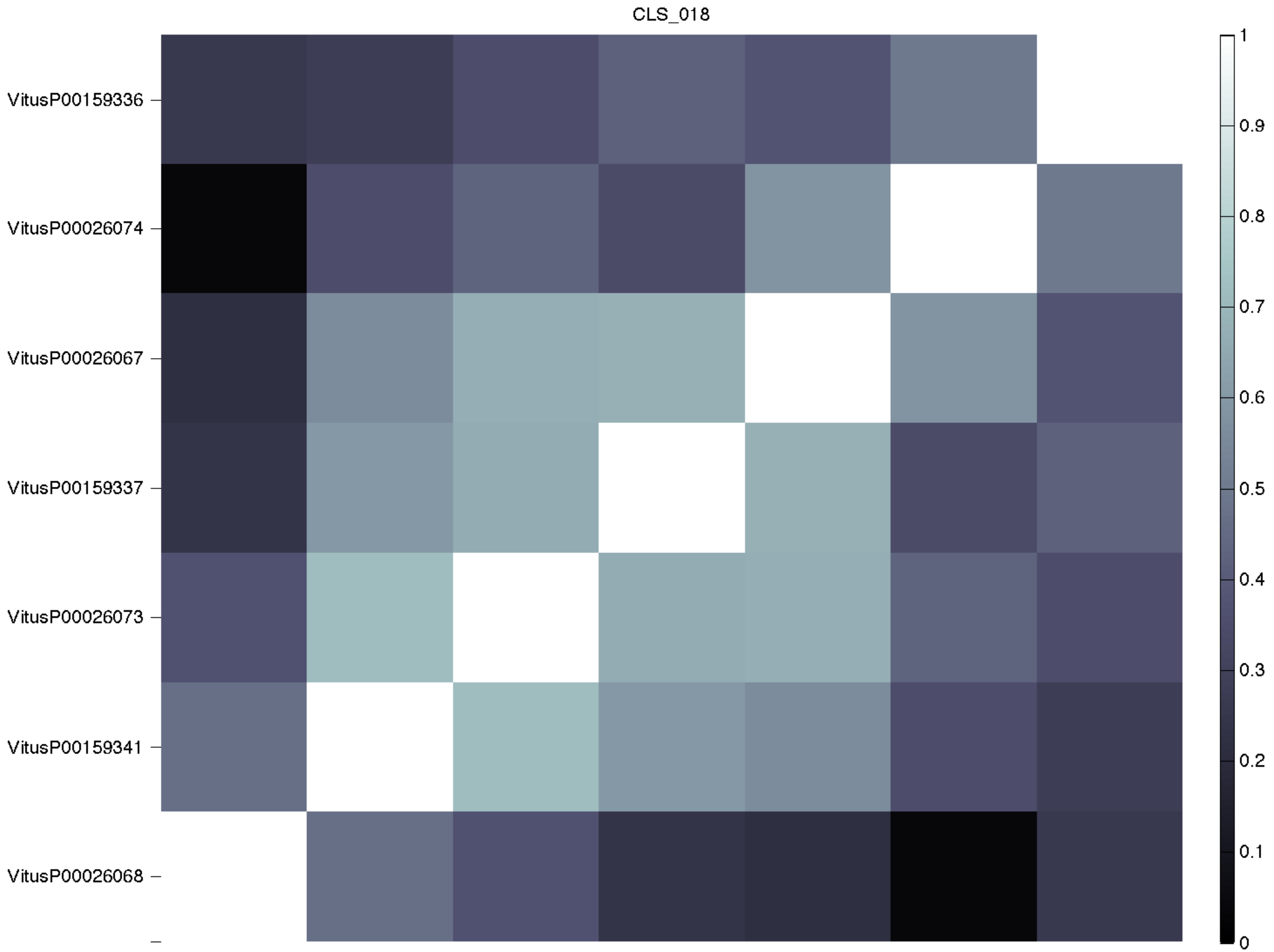

CLS_018
VitusP00159336
VitusP00026074
VitusP00026067
VitusP00159337
VitusP00026073
VitusP00159341
VitusP00026068
1
0.9
0.8
0.7
0.6
0.5
0.4
0.3
0.2
0.1
0

CLS_019

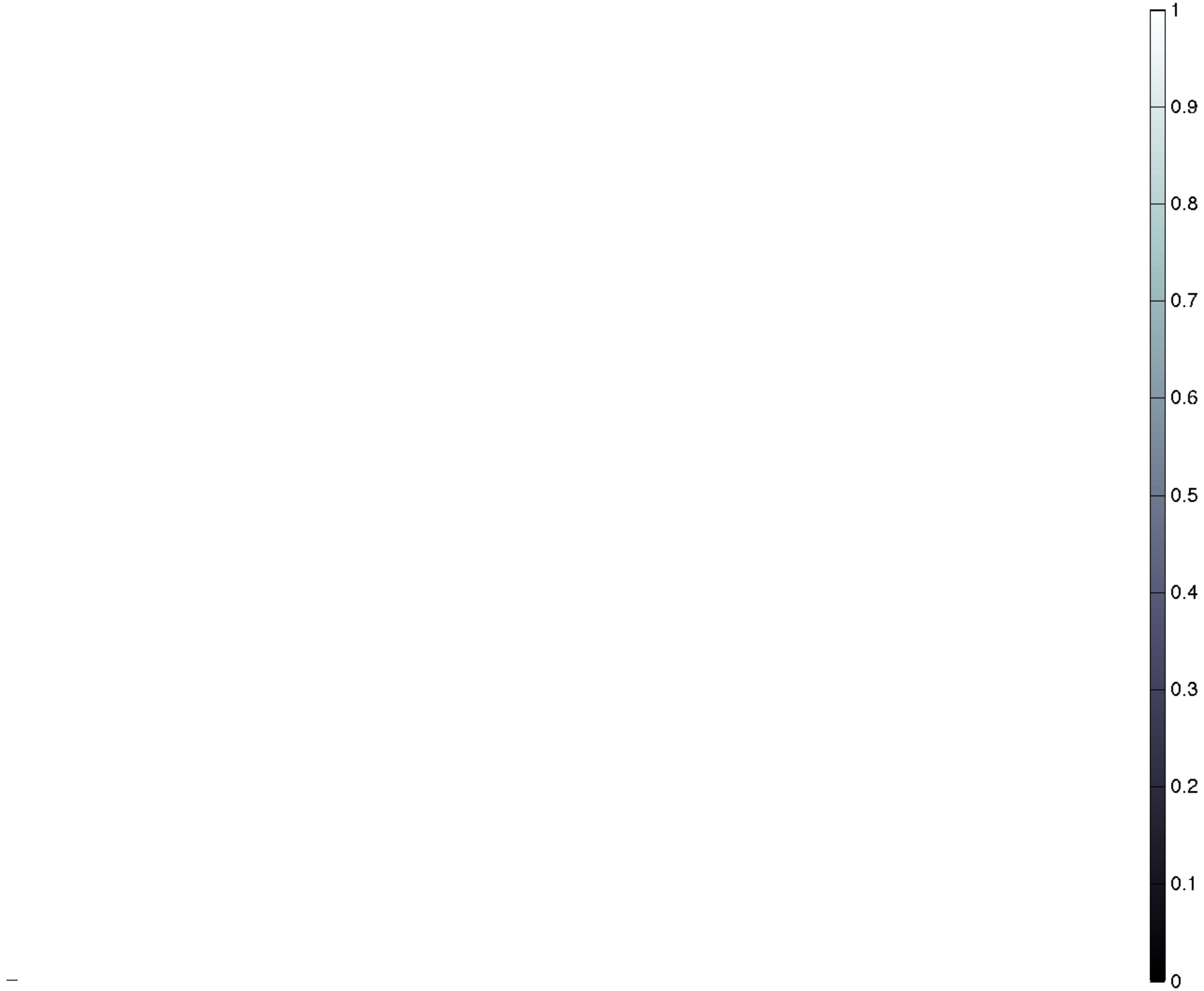

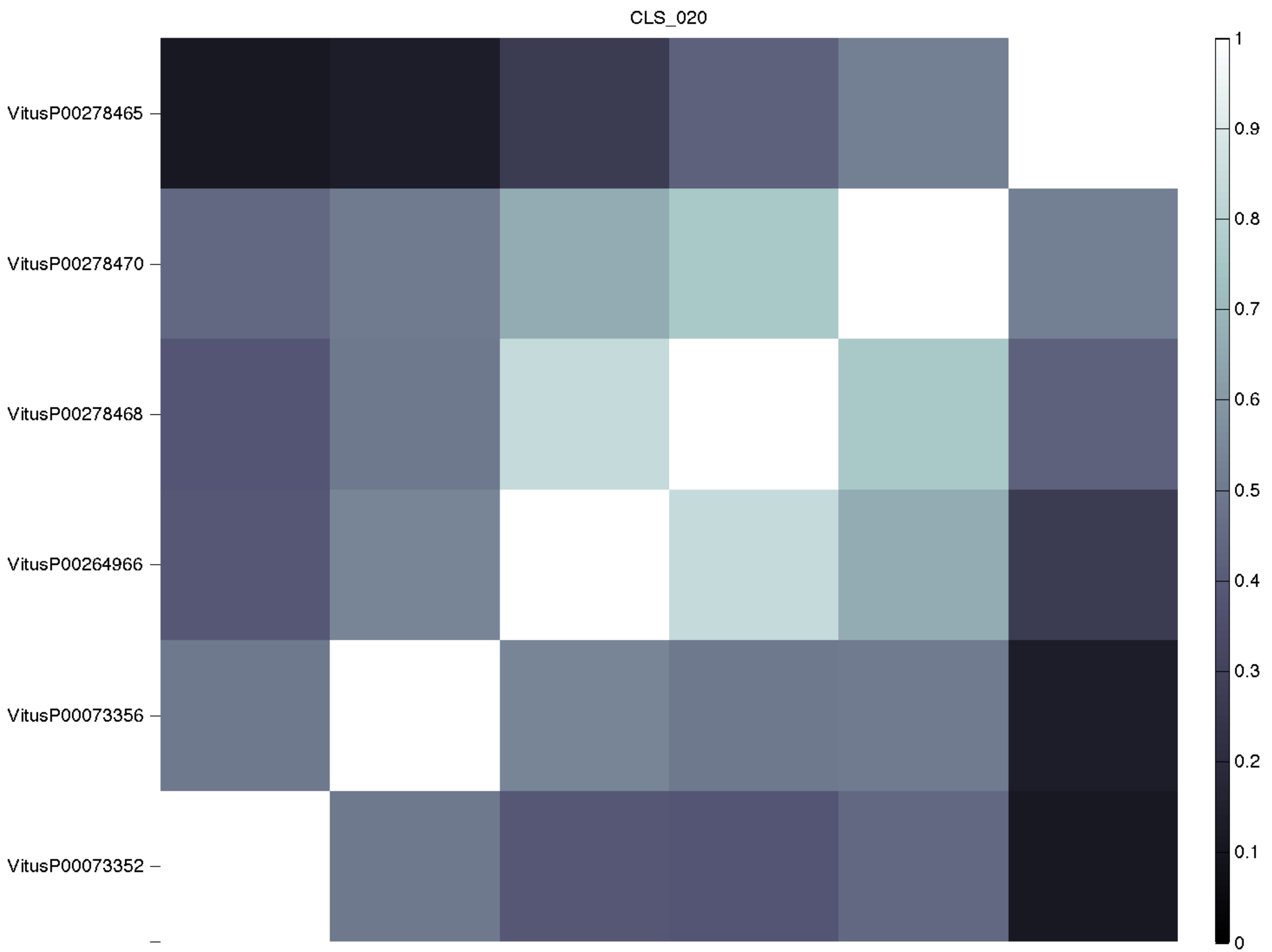
CLS_020
VitusP00278465
VitusP00278470
VitusP00278468
VitusP00264966
VitusP00073356
VitusP00073352
1
0.9
0.8
0.7
0.6
0.5
0.4
0.3
0.2
0.1
0

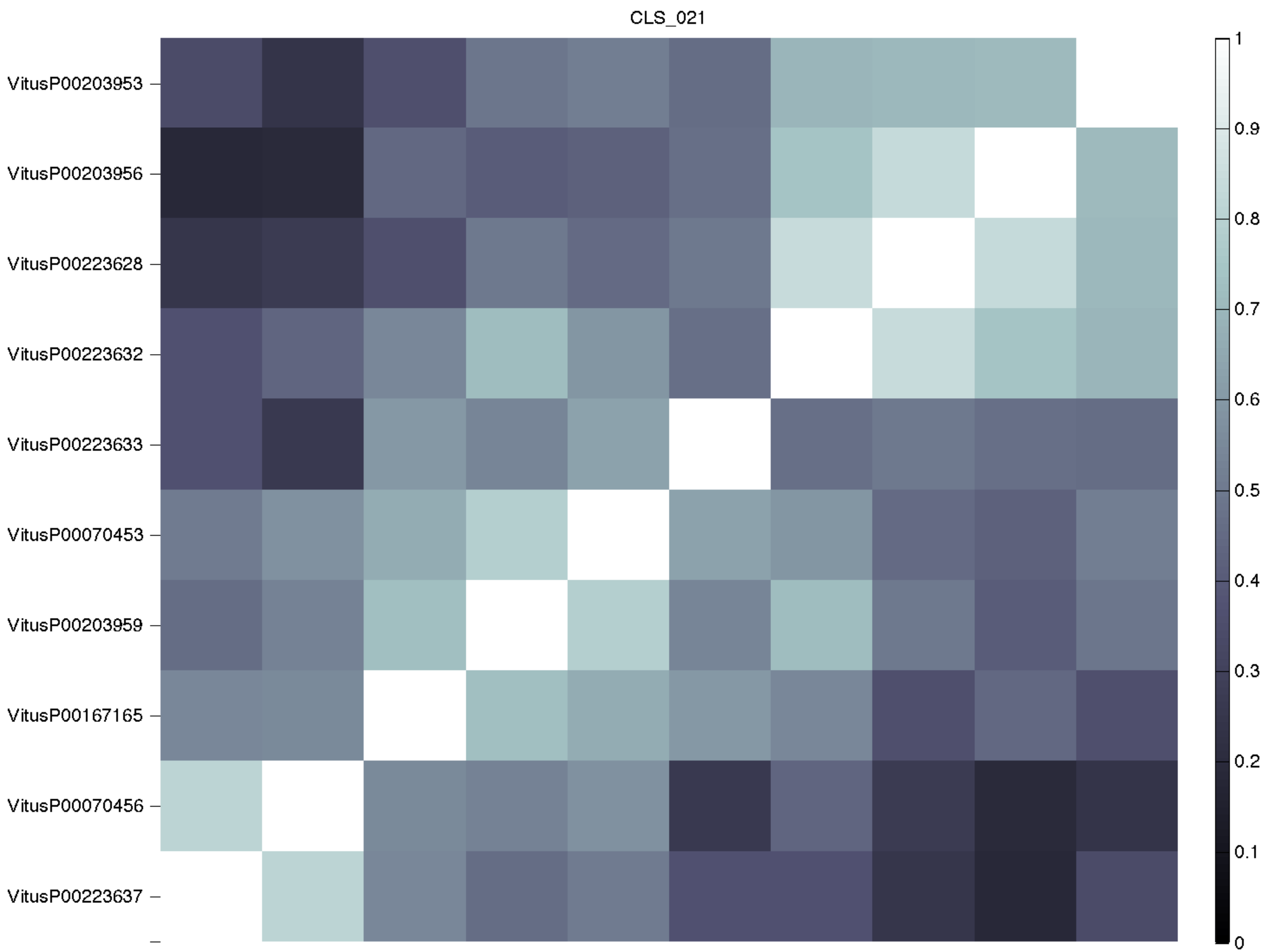
CLS_021
VitusP00203953
VitusP00203956
VitusP00223628
VitusP00223632
VitusP00223633
VitusP00070453
VitusP00203959
VitusP00167165
VitusP00070456
VitusP00223637
1
0.9
0.8
0.7
0.6
0.5
0.4
0.3
0.2
0.1
0

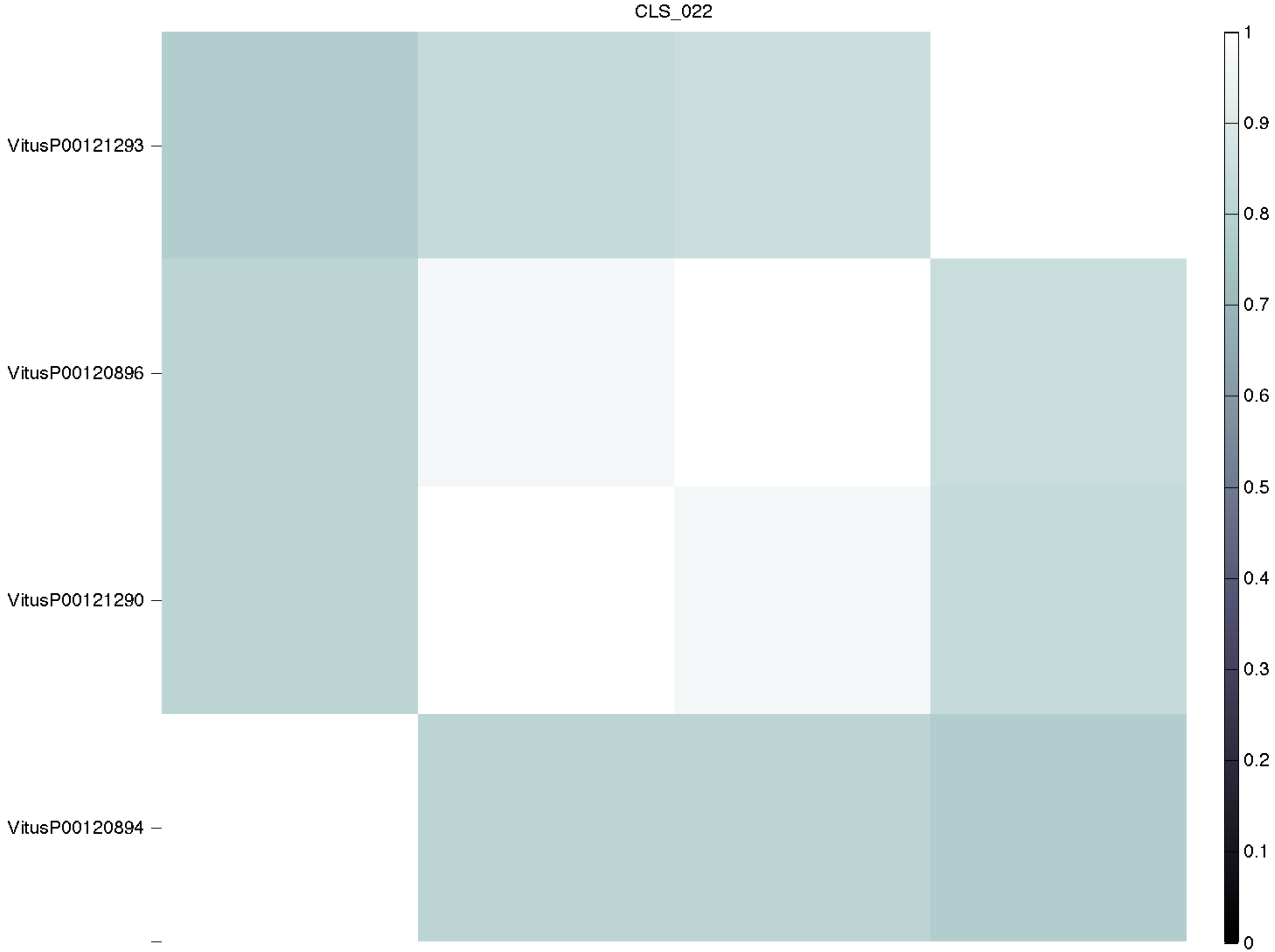

CLS_022
VitusP00121293
VitusP00120896
VitusP00121290
VitusP00120894
1
0.9
0.8
0.7
0.6
0.5
0.4
0.3
0.2
0.1
0

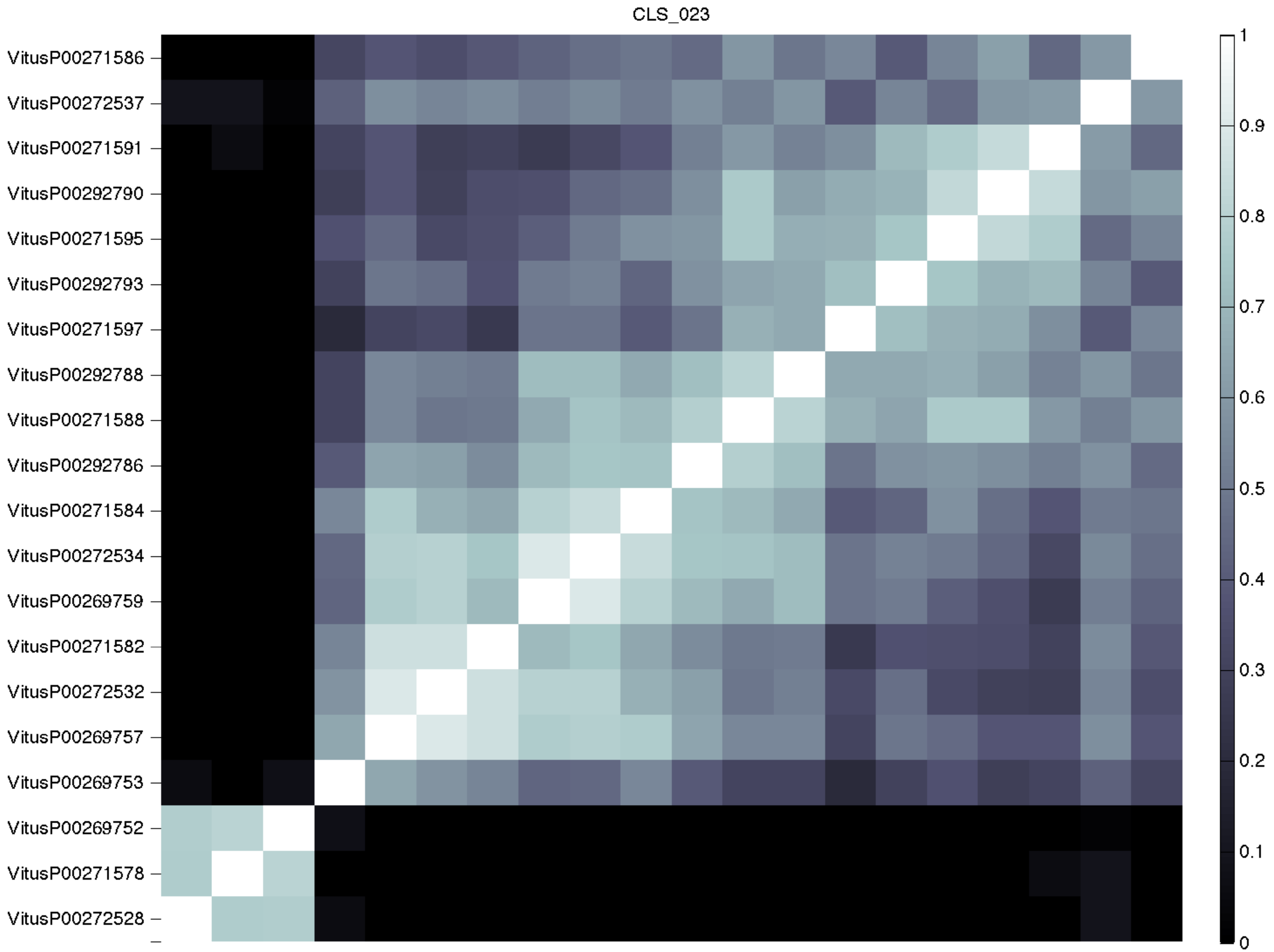
CLS_023
VitusP00271586
VitusP00272537
VitusP00271591
VitusP00292790
VitusP00271595
VitusP00292793
VitusP00271597
VitusP00292788
VitusP00271588
VitusP00292786
VitusP00271584
VitusP00272534
VitusP00269759
VitusP00271582
VitusP00272532
VitusP00269757
VitusP00269753
VitusP00269752
VitusP00271578
VitusP00272528
1
0.9
0.8
0.7
0.6
0.5
0.4
0.3
0.2
0.1
0

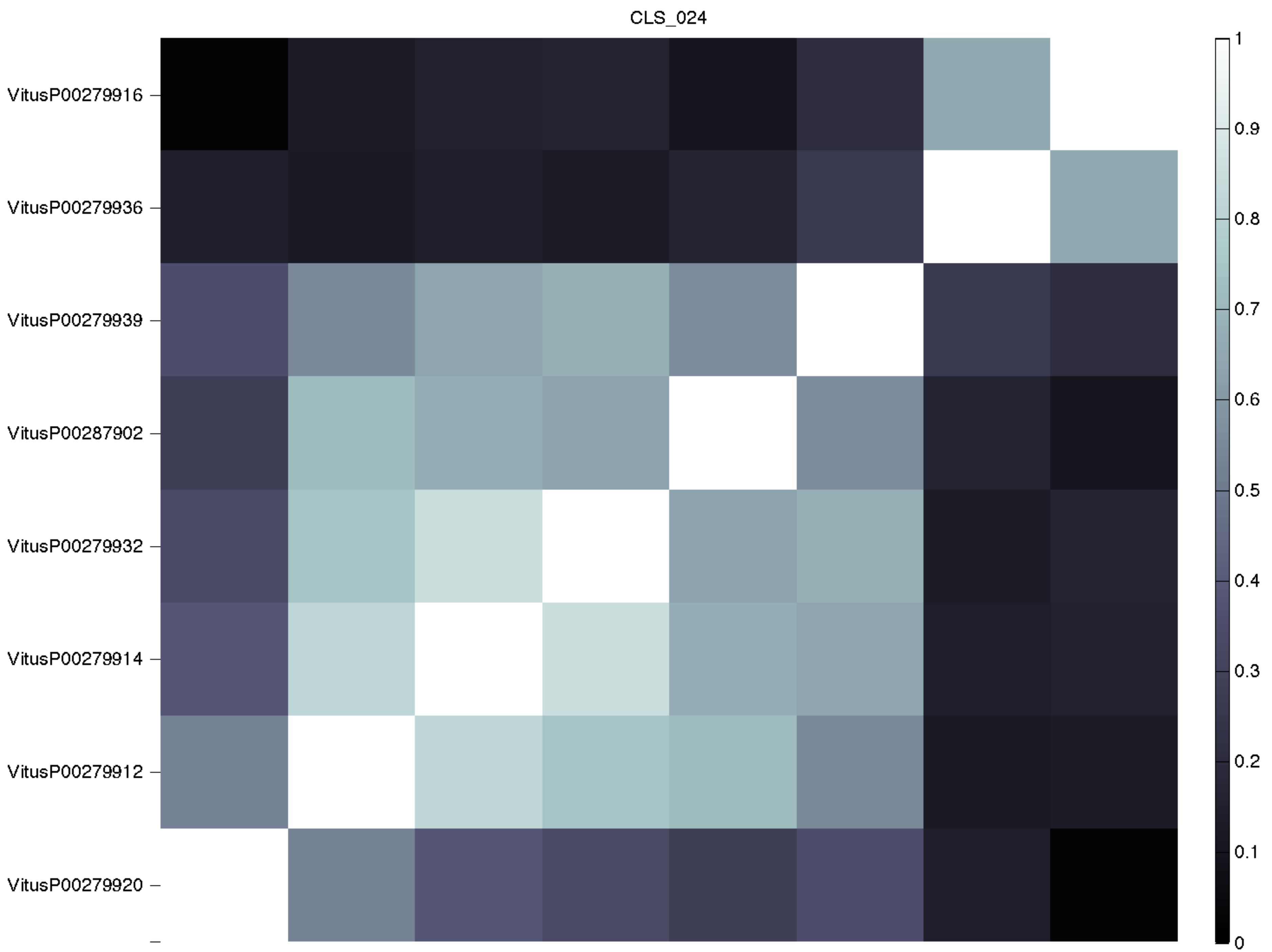
CLS_024
VitusP00279916
VitusP00279936
VitusP00279939
VitusP00287902
VitusP00279932
VitusP00279914
VitusP00279912
VitusP00279920
1
0.9
0.8
0.7
0.6
0.5
0.4
0.3
0.2
0.1
0

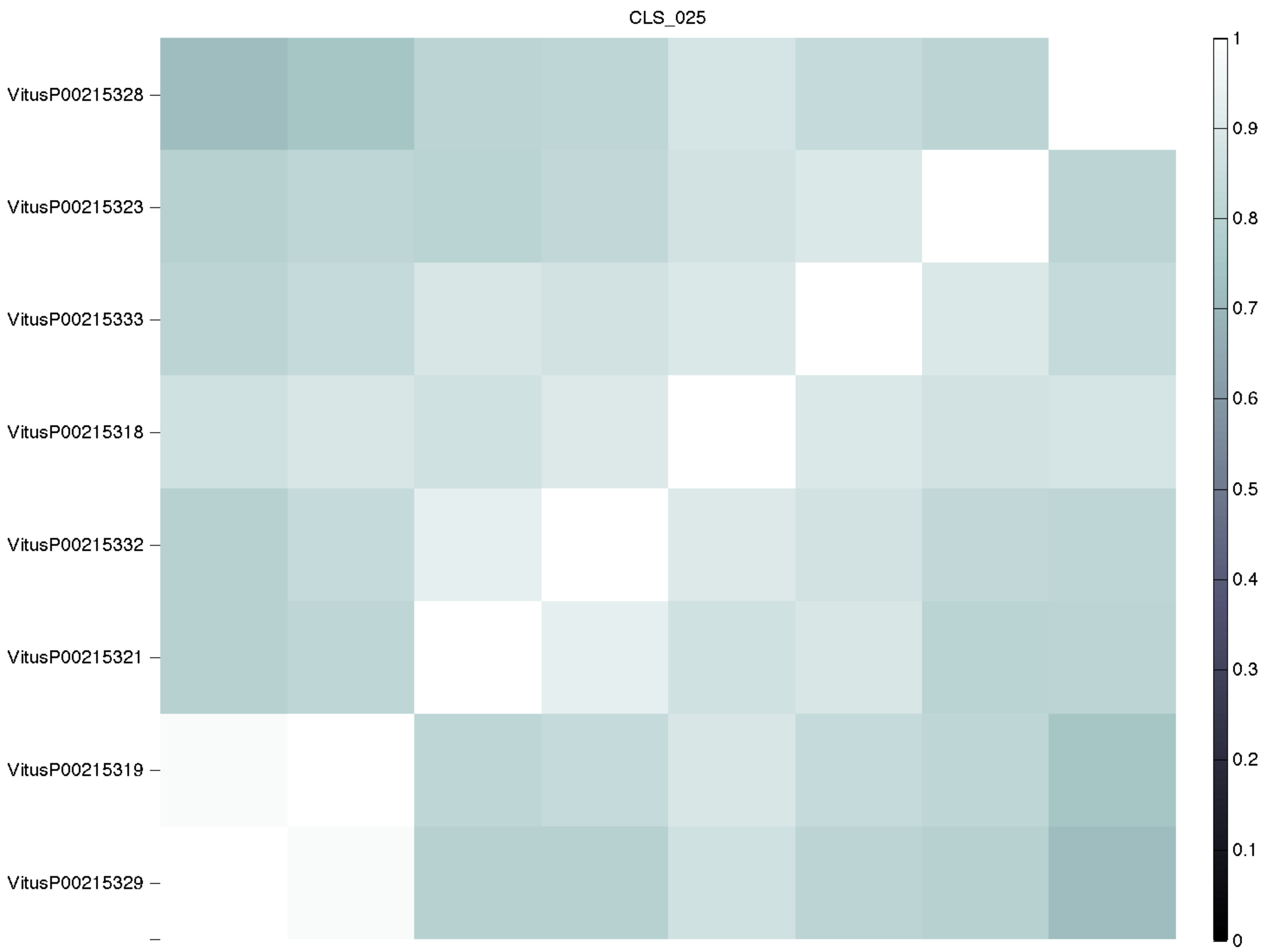

CLS_025
VitusP00215328
VitusP00215323
VitusP00215333
VitusP00215318
VitusP00215332
VitusP00215321
VitusP00215319
VitusP00215329
1
0.9
0.8
0.7
0.6
0.5
0.4
0.3
0.2
0.1
0

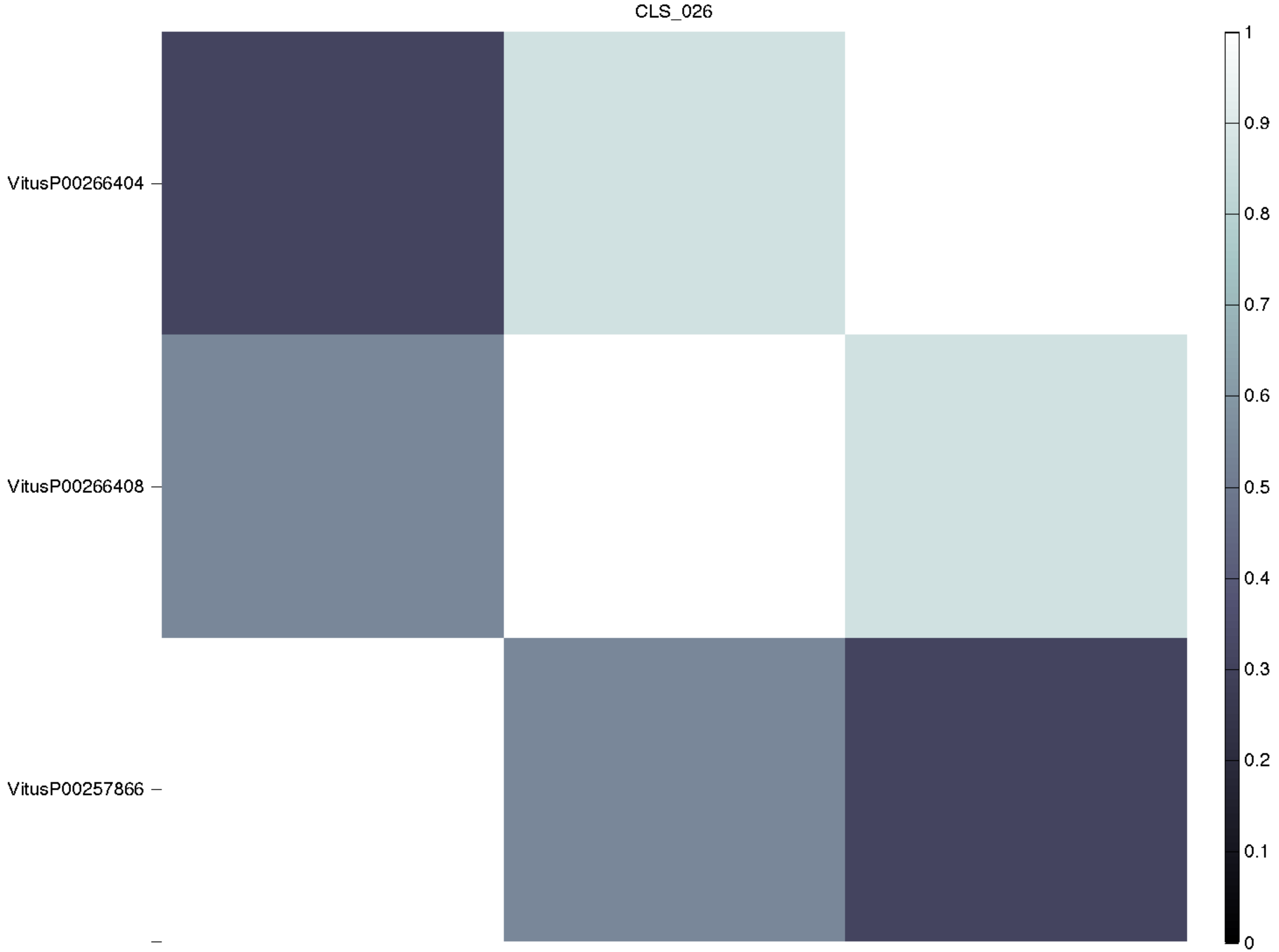
CLS_026
VitusP00266404
VitusP00266408
VitusP00257866
1
0.9
0.8
0.7
0.6
0.5
0.4
0.3
0.2
0.1
0

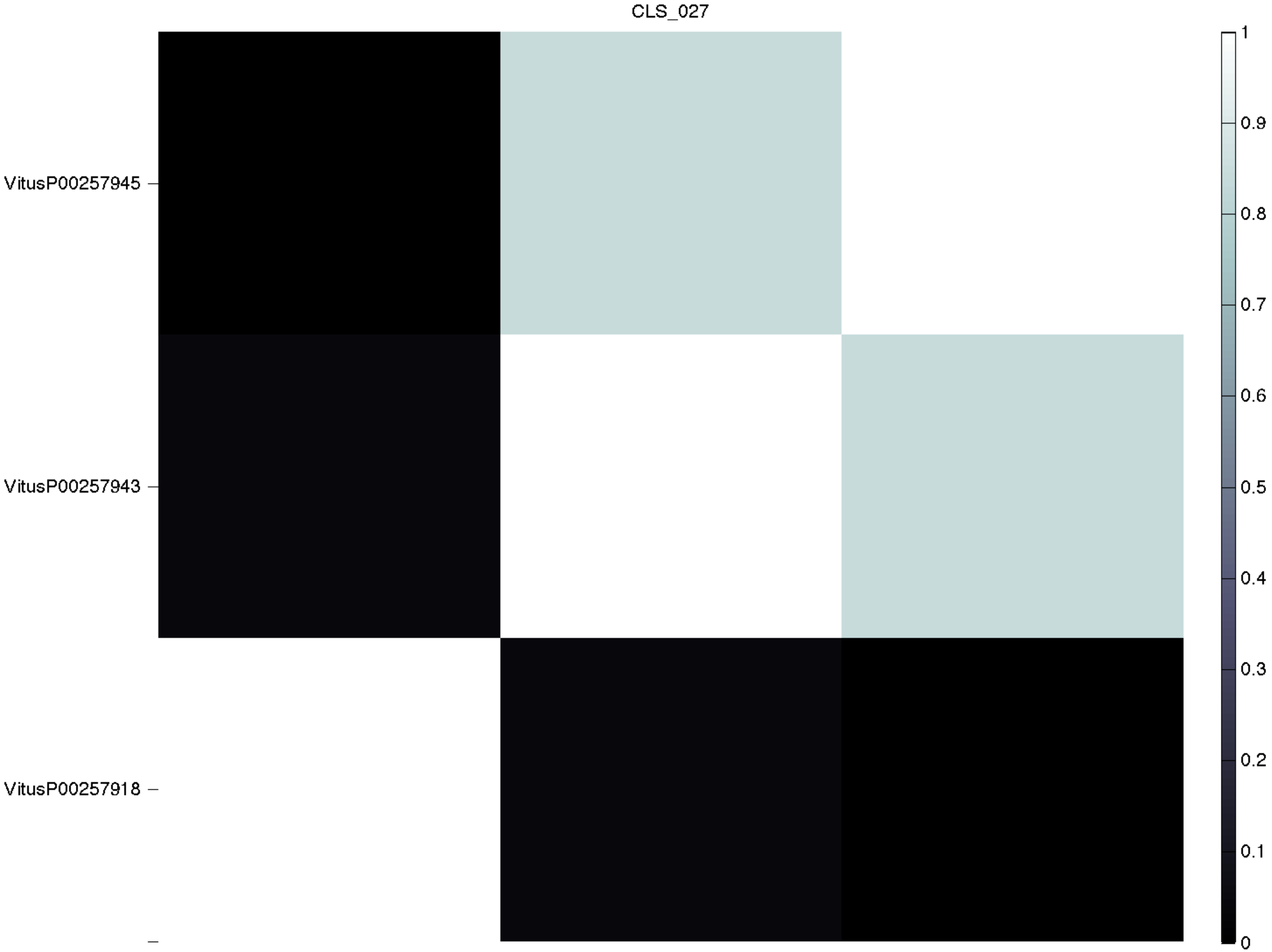

CLS_027
VitusP00257945
VitusP00257943
VitusP00257918
1
0.9
0.8
0.7
0.6
0.5
0.4
0.3
0.2
0.1
0

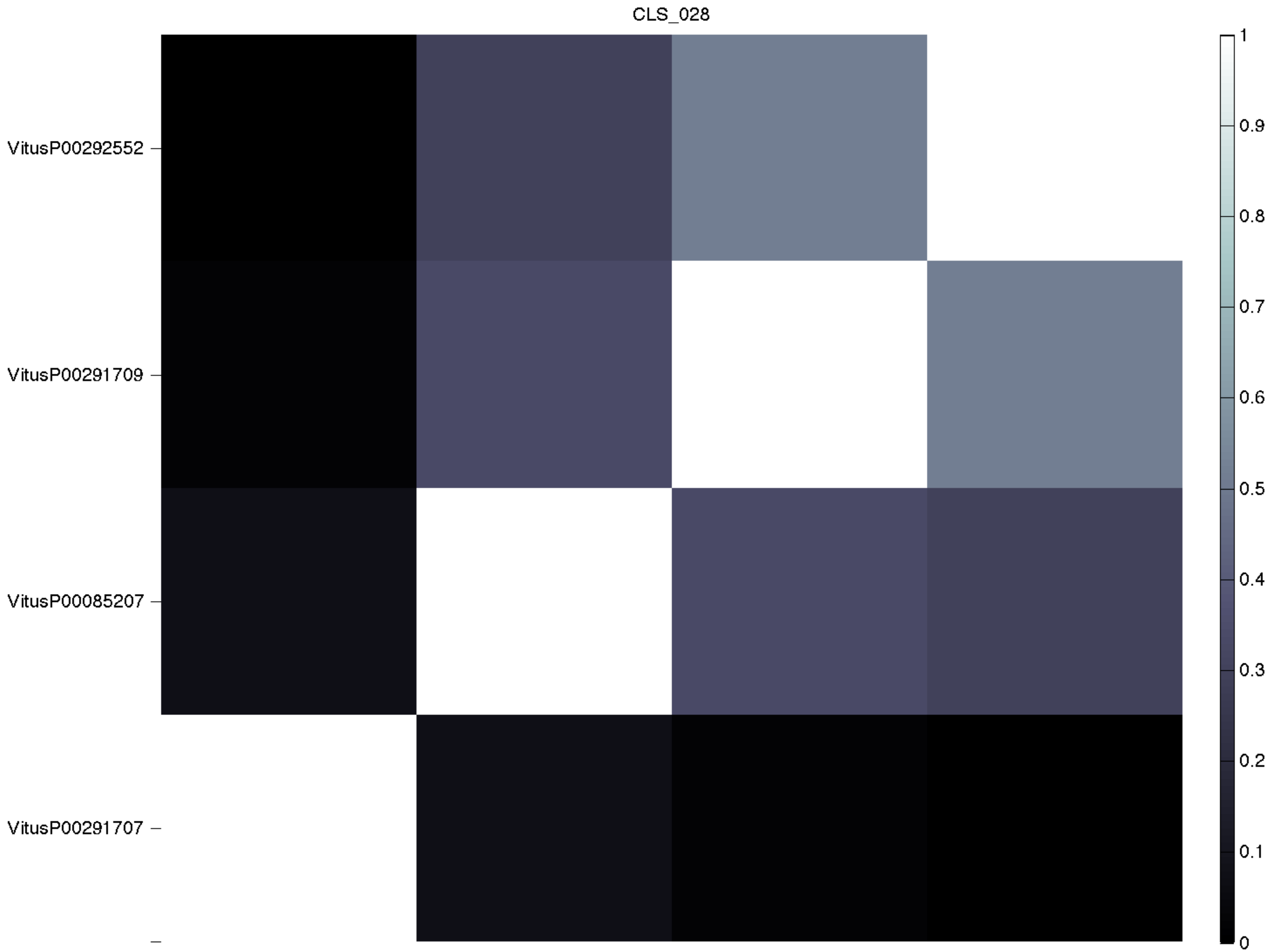
CLS_028
VitusP00292552
VitusP00291709
VitusP00085207
VitusP00291707
1
0.9
0.8
0.7
0.6
0.5
0.4
0.3
0.2
0.1
0

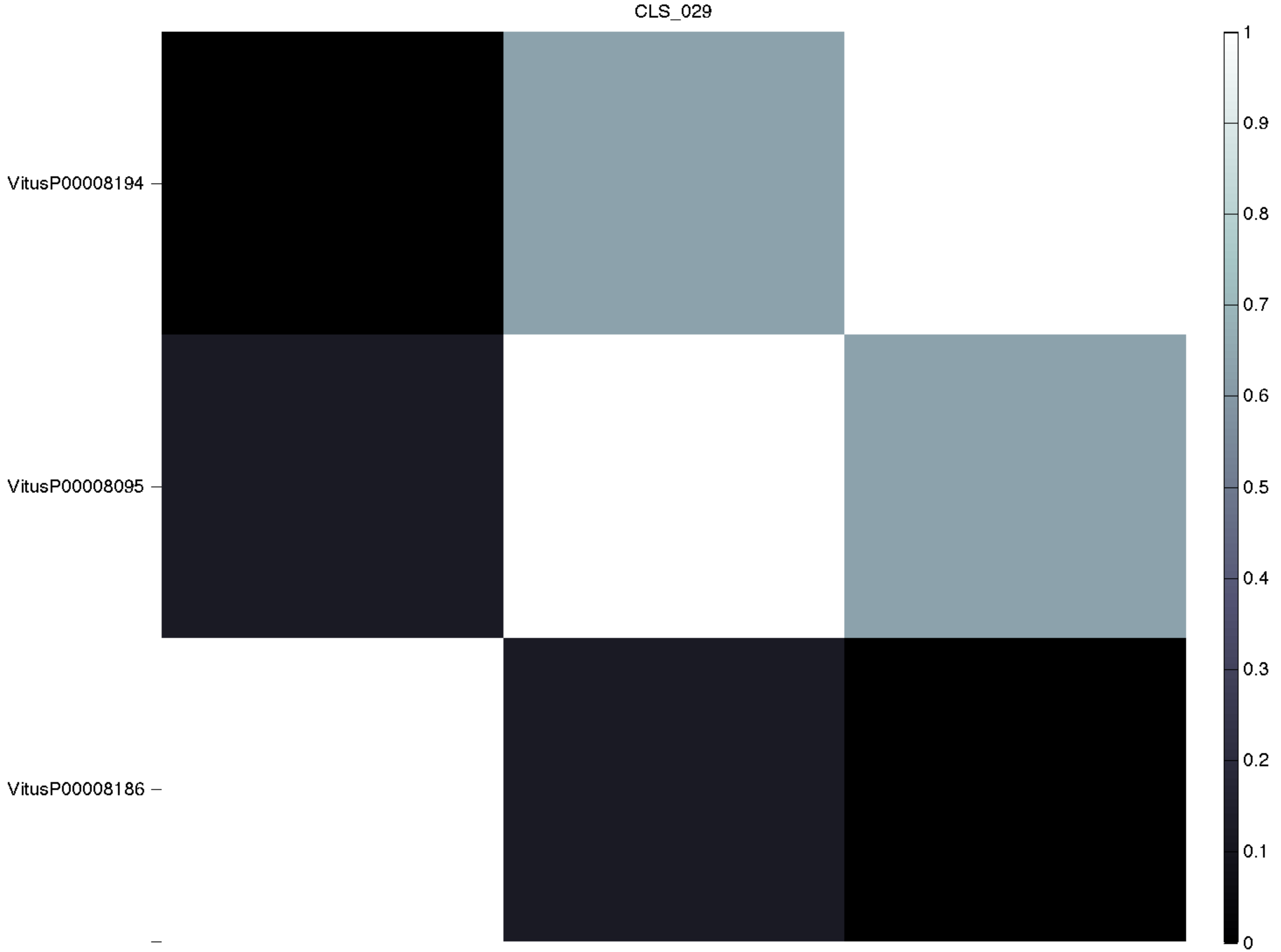
CLS_029
VitusP00008194
VitusP00008095
VitusP00008186
1
0.9
0.8
0.7
0.6
0.5
0.4
0.3
0.2
0.1
0

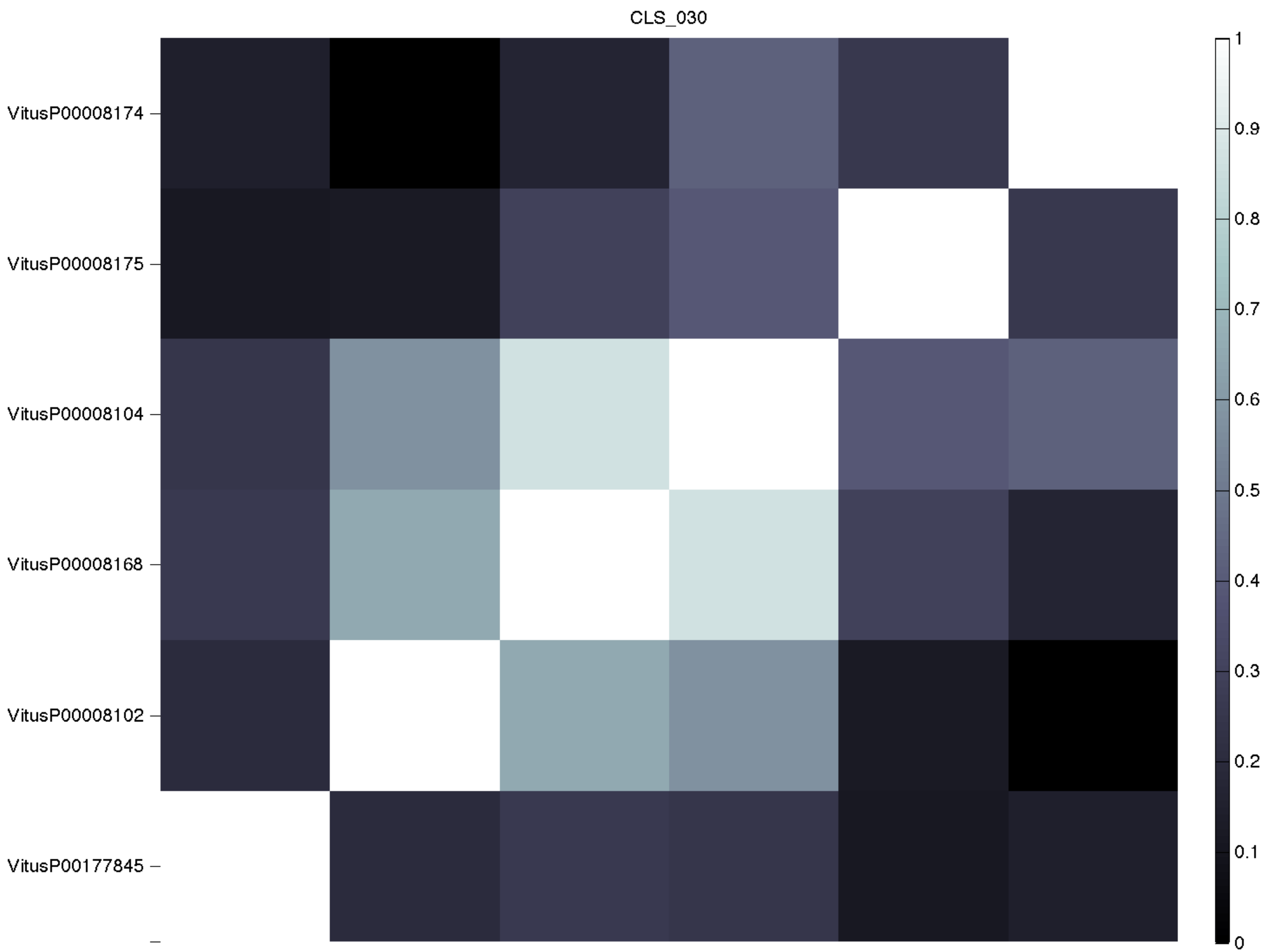
CLS_030
VitusP00008174
VitusP00008175
VitusP00008104
VitusP00008168
VitusP00008102
VitusP00177845
1
0.9
0.8
0.7
0.6
0.5
0.4
0.3
0.2
0.1
0

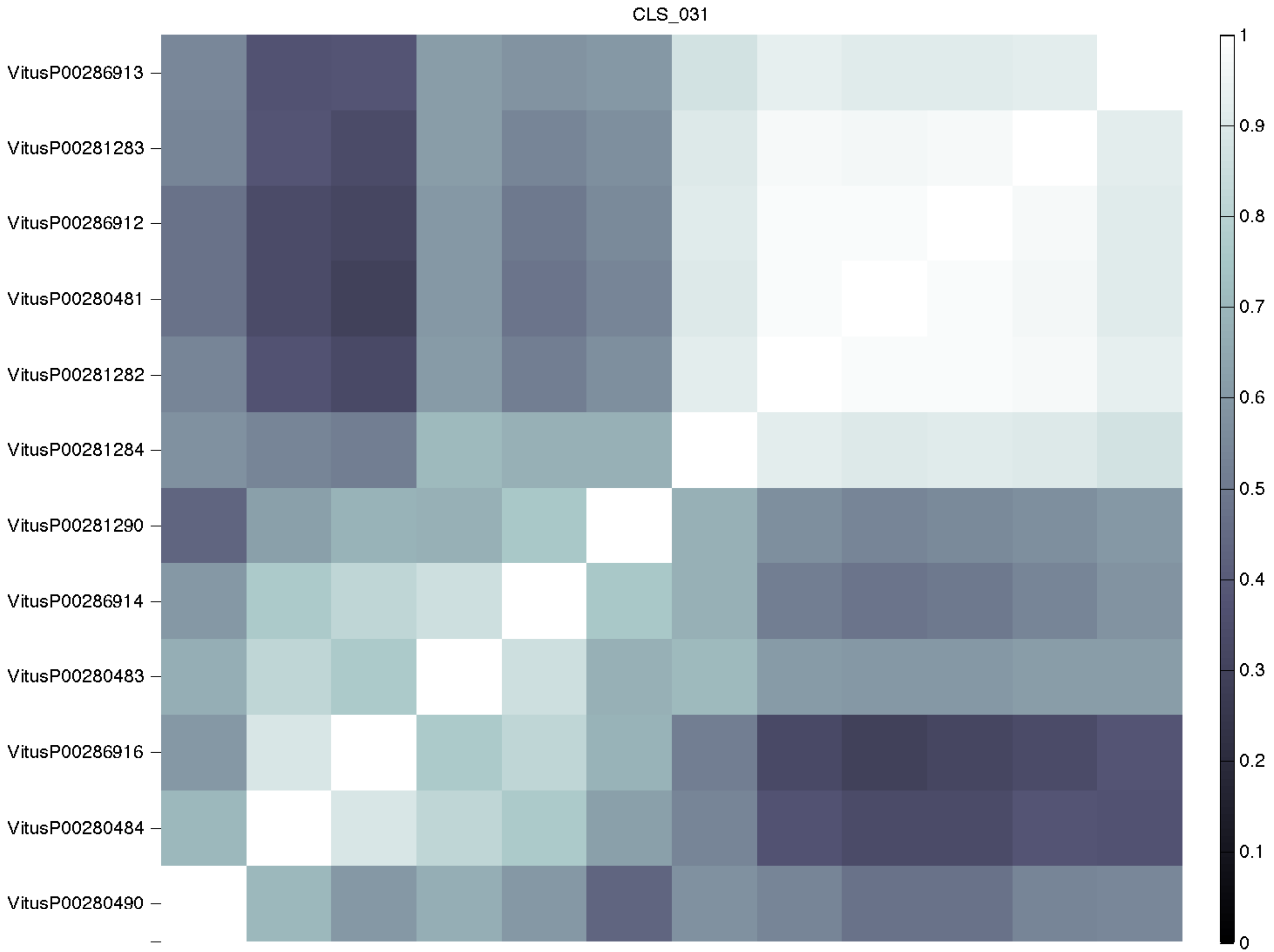
CLS_031
VitusP00286913
VitusP00281283
VitusP00286912
VitusP00280481
VitusP00281282
VitusP00281284
VitusP00281290
VitusP00286914
VitusP00280483
VitusP00286916
VitusP00280484
VitusP00280490
1
0.9
0.8
0.7
0.6
0.5
0.4
0.3
0.2
0.1
0

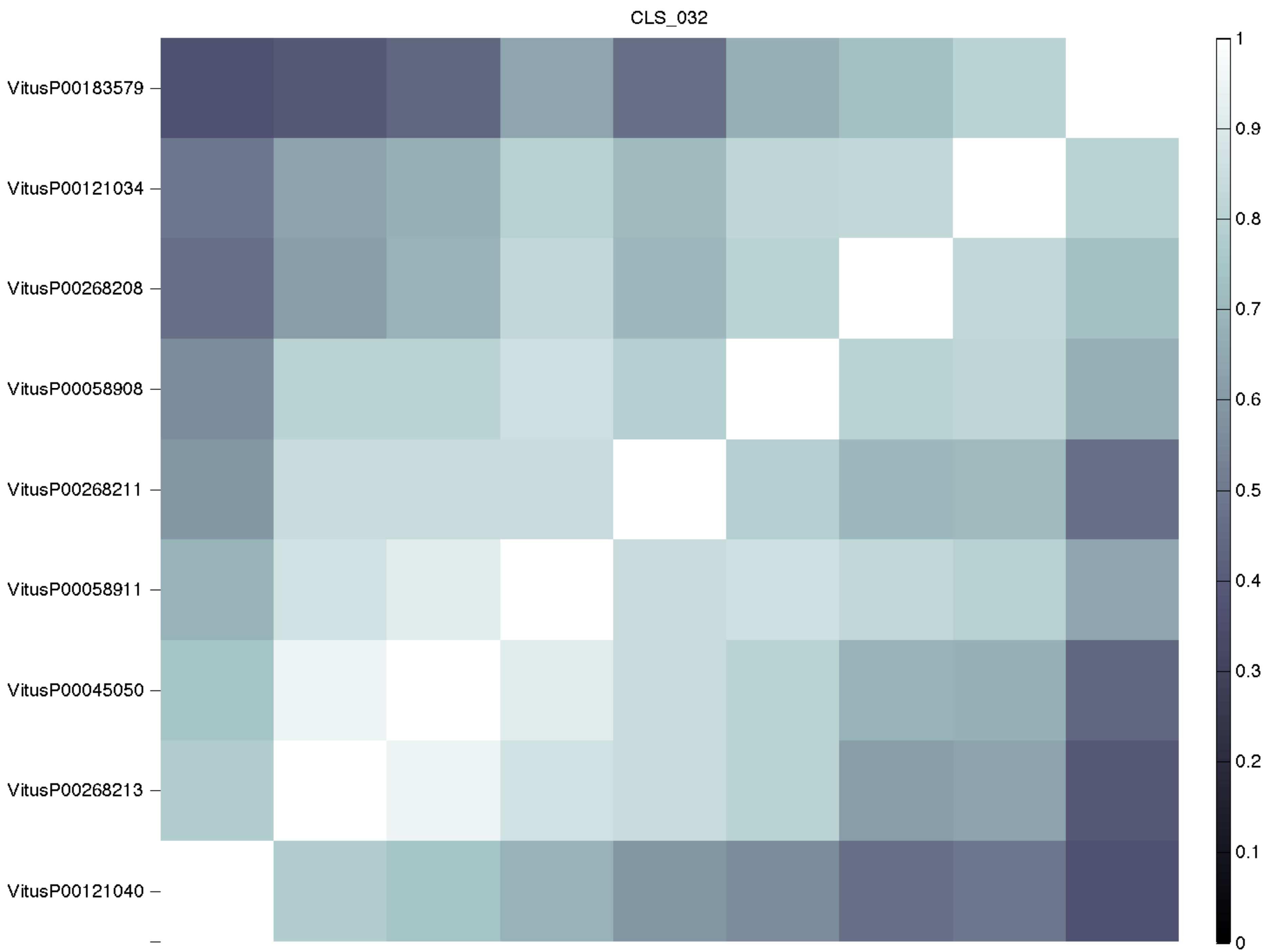
CLS_032
VitusP00183579
VitusP00121034
VitusP00268208
VitusP00058908
VitusP00268211
VitusP00058911
VitusP00045050
VitusP00268213
VitusP00121040
1
0.9
0.8
0.7
0.6
0.5
0.4
0.3
0.2
0.1
0

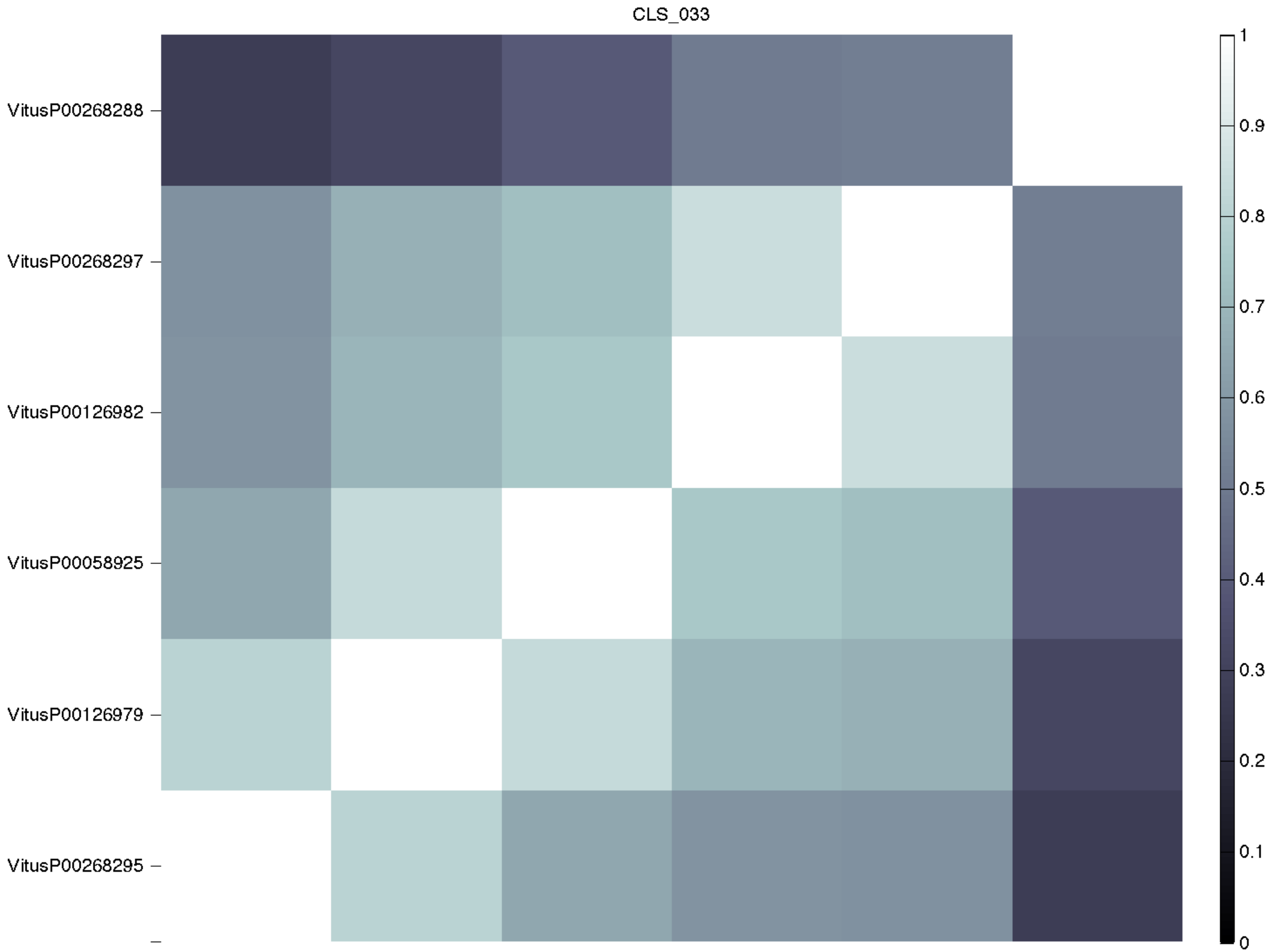
CLS_033
VitusP00268288
VitusP00268297
VitusP00126982
VitusP00058925
VitusP00126979
VitusP00268295
1
0.9
0.8
0.7
0.6
0.5
0.4
0.3
0.2
0.1
0

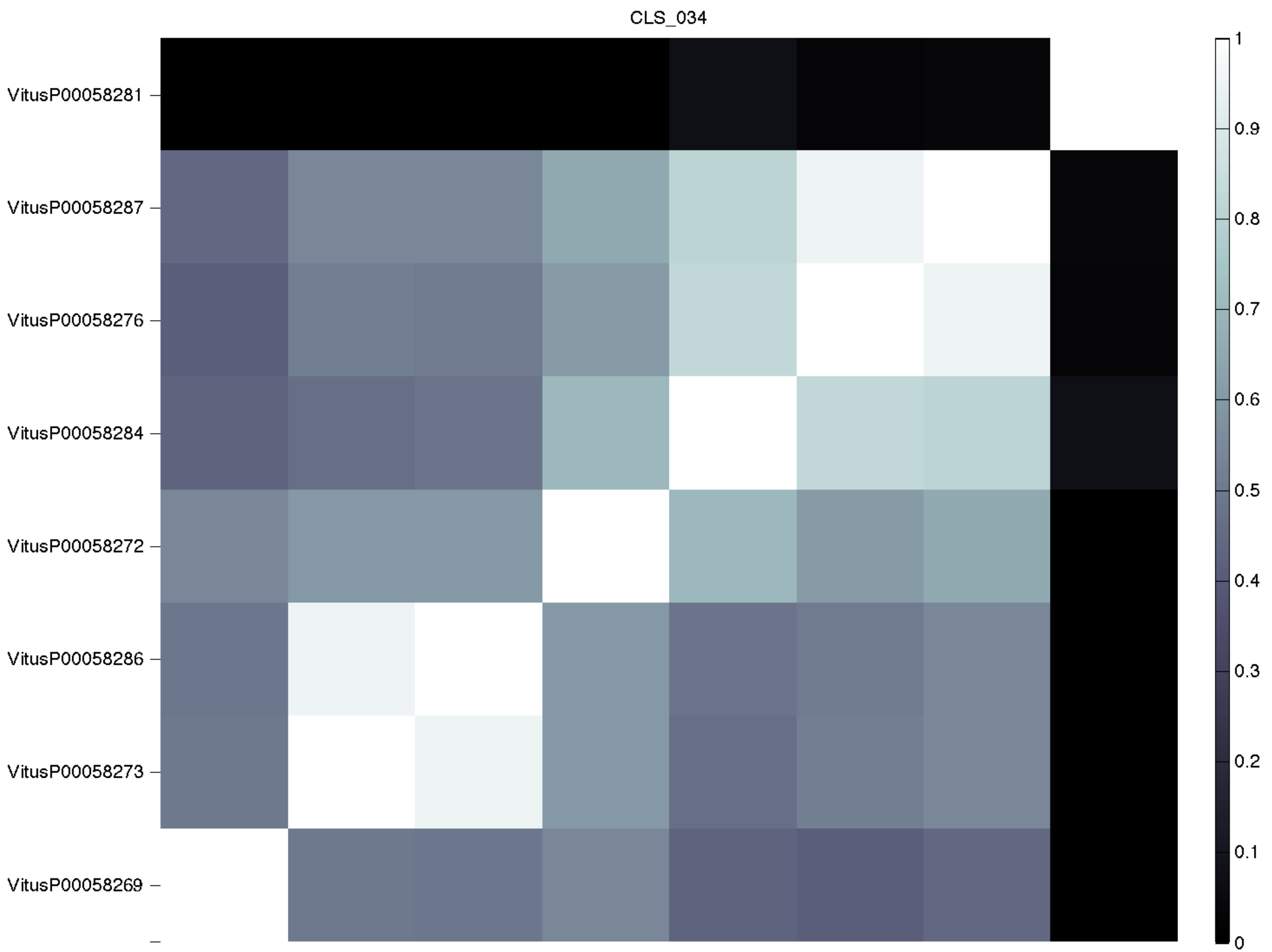
CLS_034
VitusP00058281
VitusP00058287
VitusP00058276
VitusP00058284
VitusP00058272
VitusP00058286
VitusP00058273
VitusP00058269
1
0.9
0.8
0.7
0.6
0.5
0.4
0.3
0.2
0.1
0

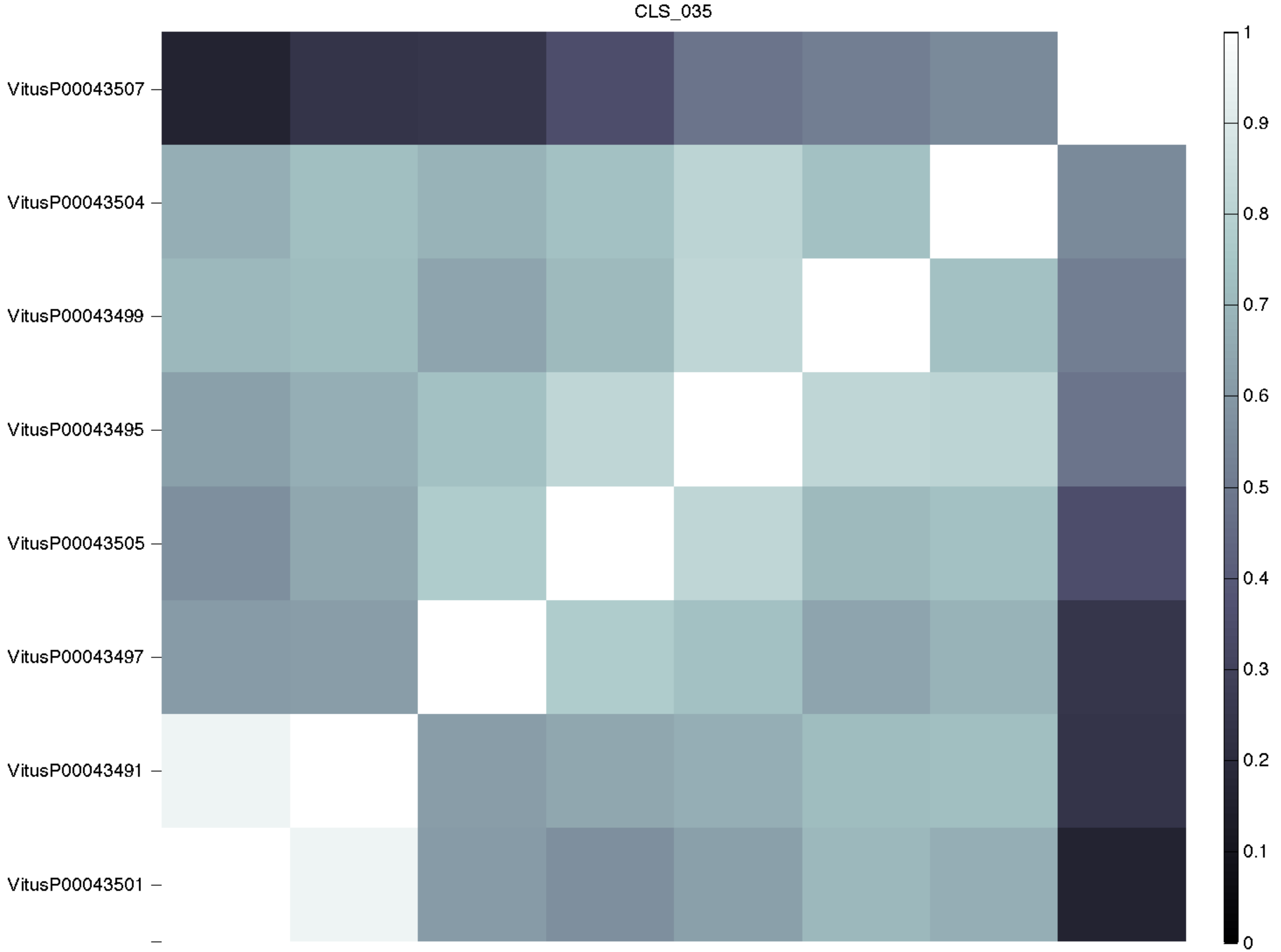
CLS_035
VitusP00043507
VitusP00043504
VitusP00043499
VitusP00043495
VitusP00043505
VitusP00043497
VitusP00043491
VitusP00043501
1
0.9
0.8
0.7
0.6
0.5
0.4
0.3
0.2
0.1
0

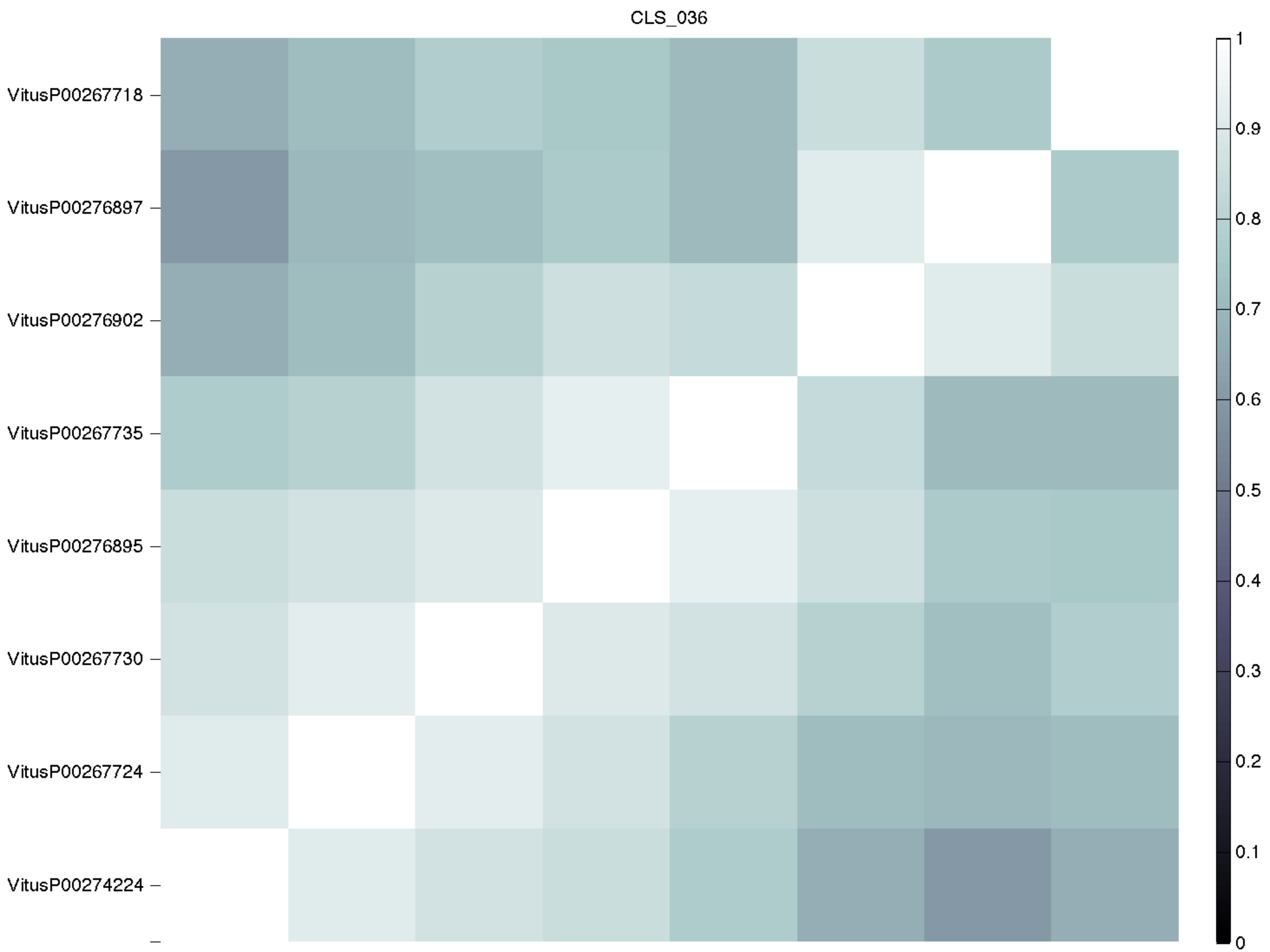
CLS_036
VitusP00267718
VitusP00276897
VitusP00276902
VitusP00267735
VitusP00276895
VitusP00267730
VitusP00267724
VitusP00274224
1
0.9
0.8
0.7
0.6
0.5
0.4
0.3
0.2
0.1
0

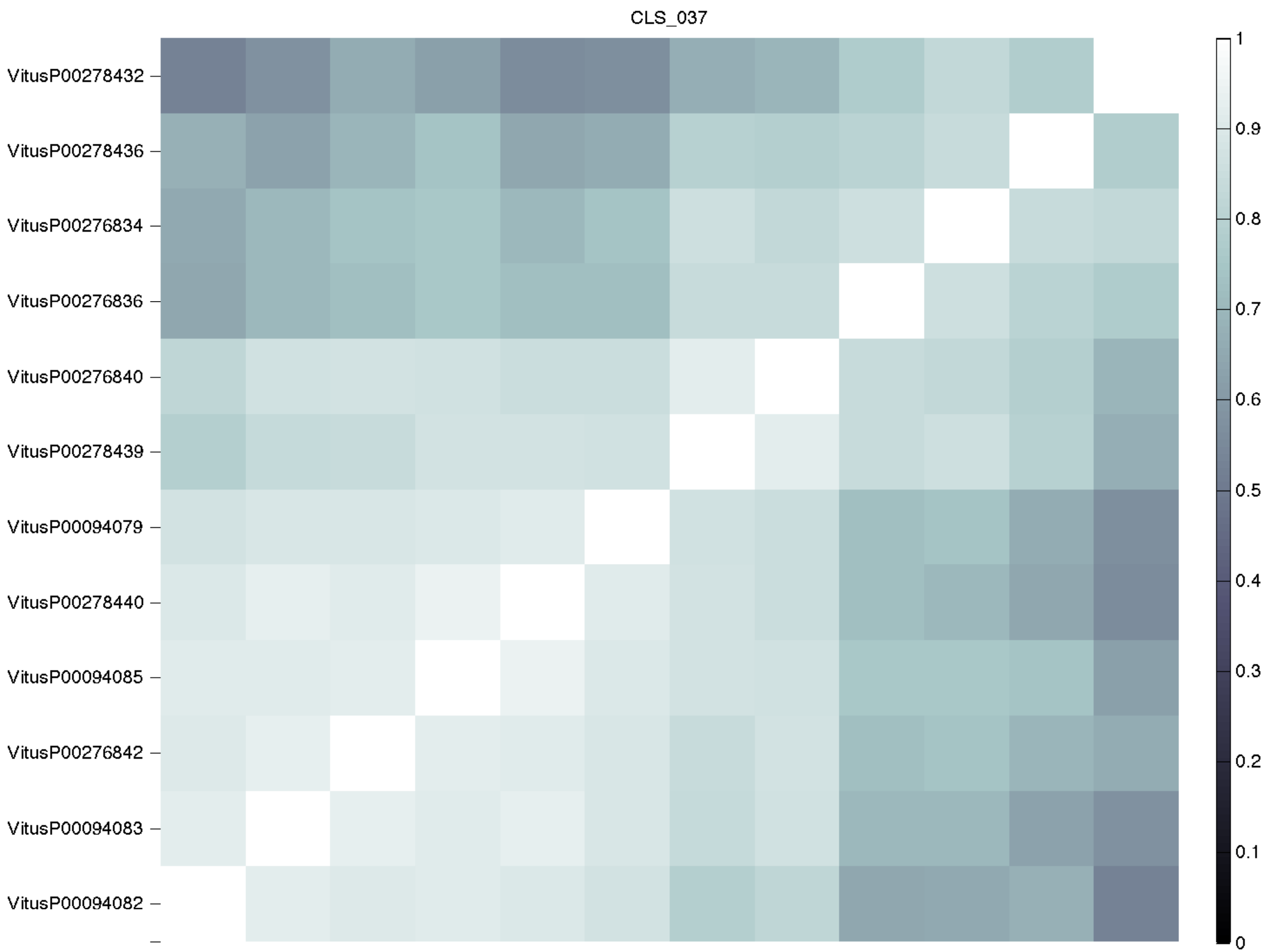

CLS_037
VitusP00278432
VitusP00278436
VitusP00276834
VitusP00276836
VitusP00276840
VitusP00278439
VitusP00094079
VitusP00278440
VitusP00094085
VitusP00276842
VitusP00094083
VitusP00094082
1
0.9
0.8
0.7
0.6
0.5
0.4
0.3
0.2
0.1
0

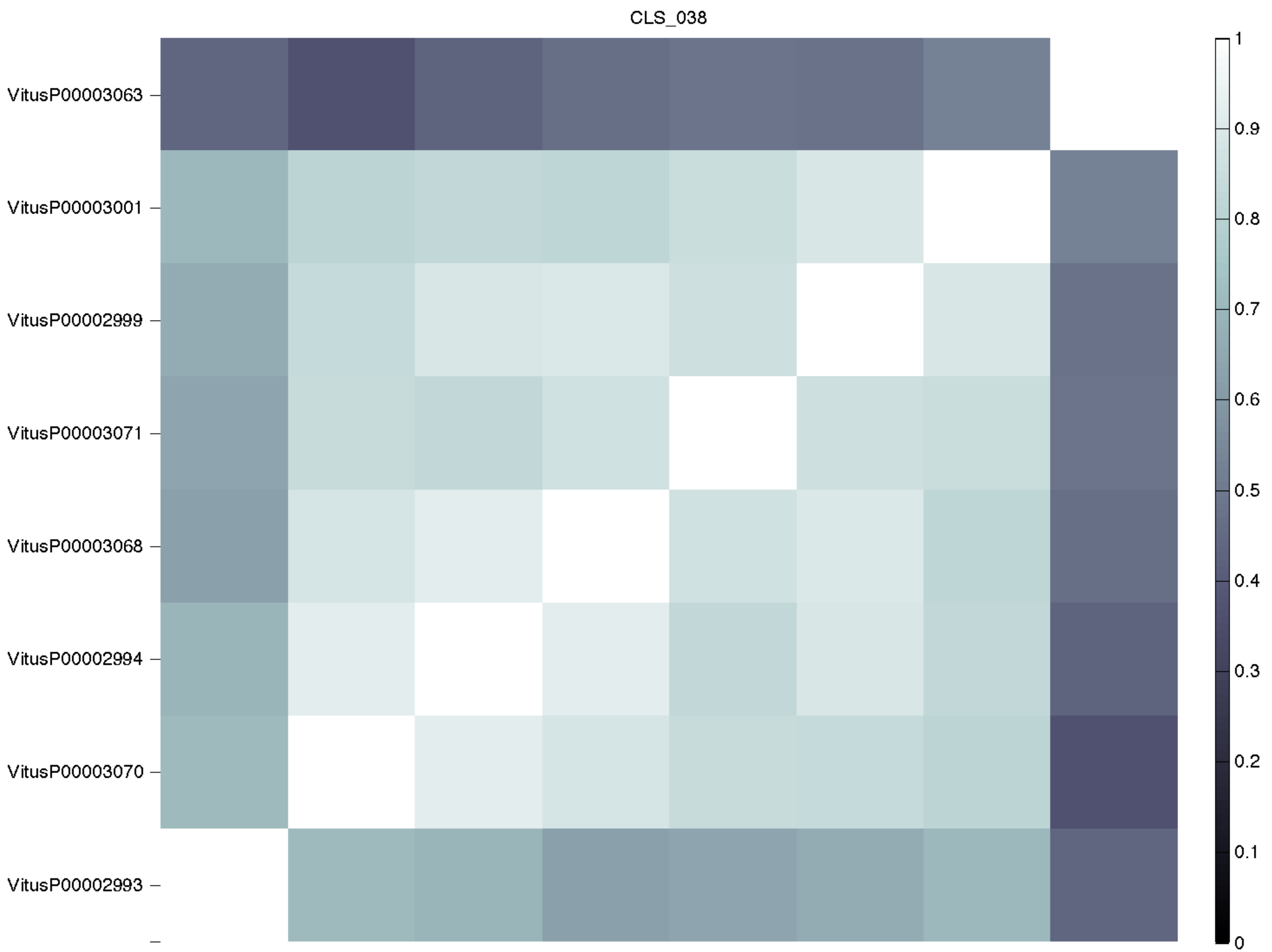
CLS_038
VitusP00003063
VitusP00003001
VitusP00002999
VitusP00003071
VitusP00003068
VitusP00002994
VitusP00003070
VitusP00002993
1
0.9
0.8
0.7
0.6
0.5
0.4
0.3
0.2
0.1
0

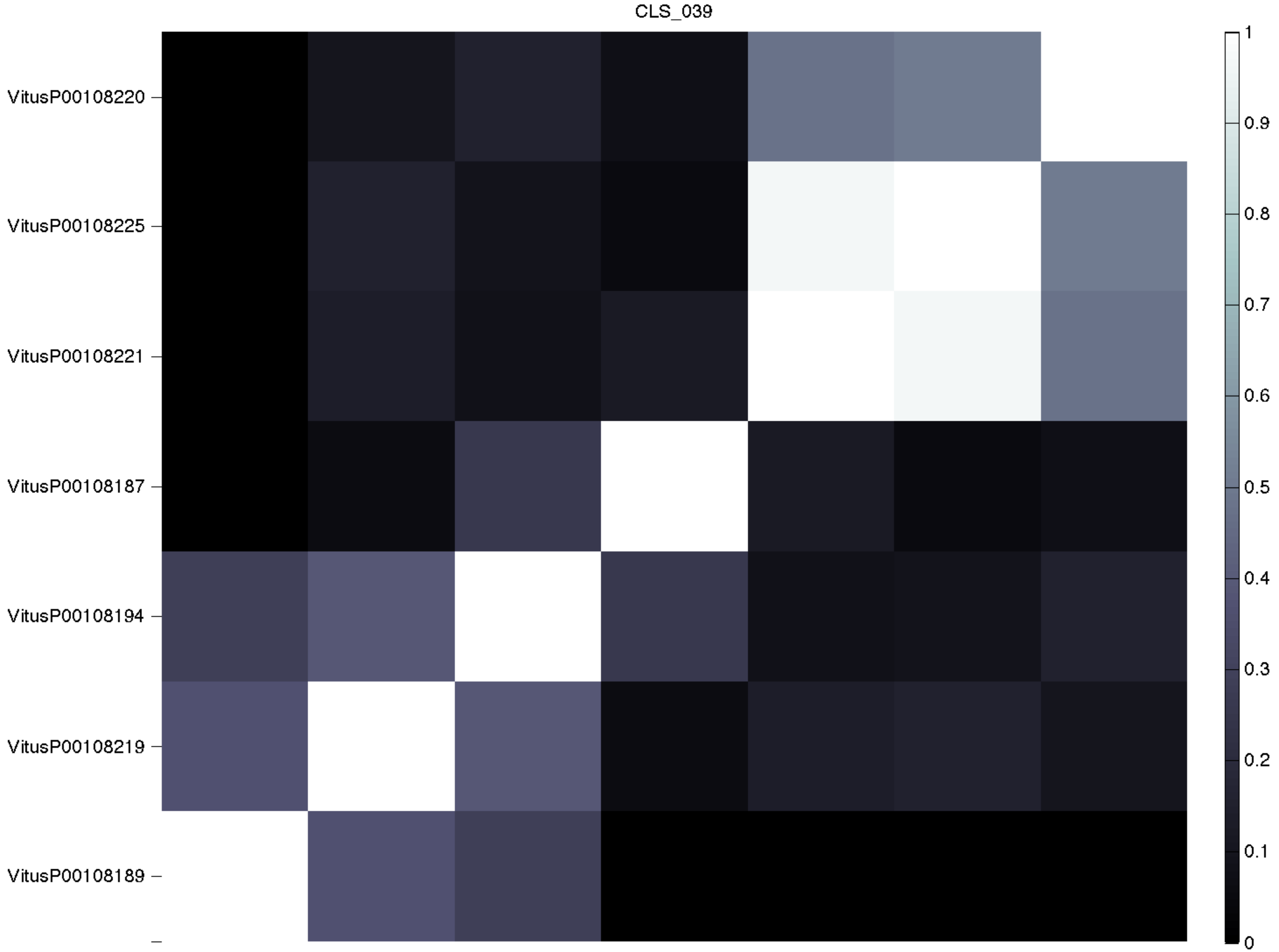
CLS_039
VitusP00108220
VitusP00108225
VitusP00108221
VitusP00108187
VitusP00108194
VitusP00108219
VitusP00108189
1
0.9
0.8
0.7
0.6
0.5
0.4
0.3
0.2
0.1
0

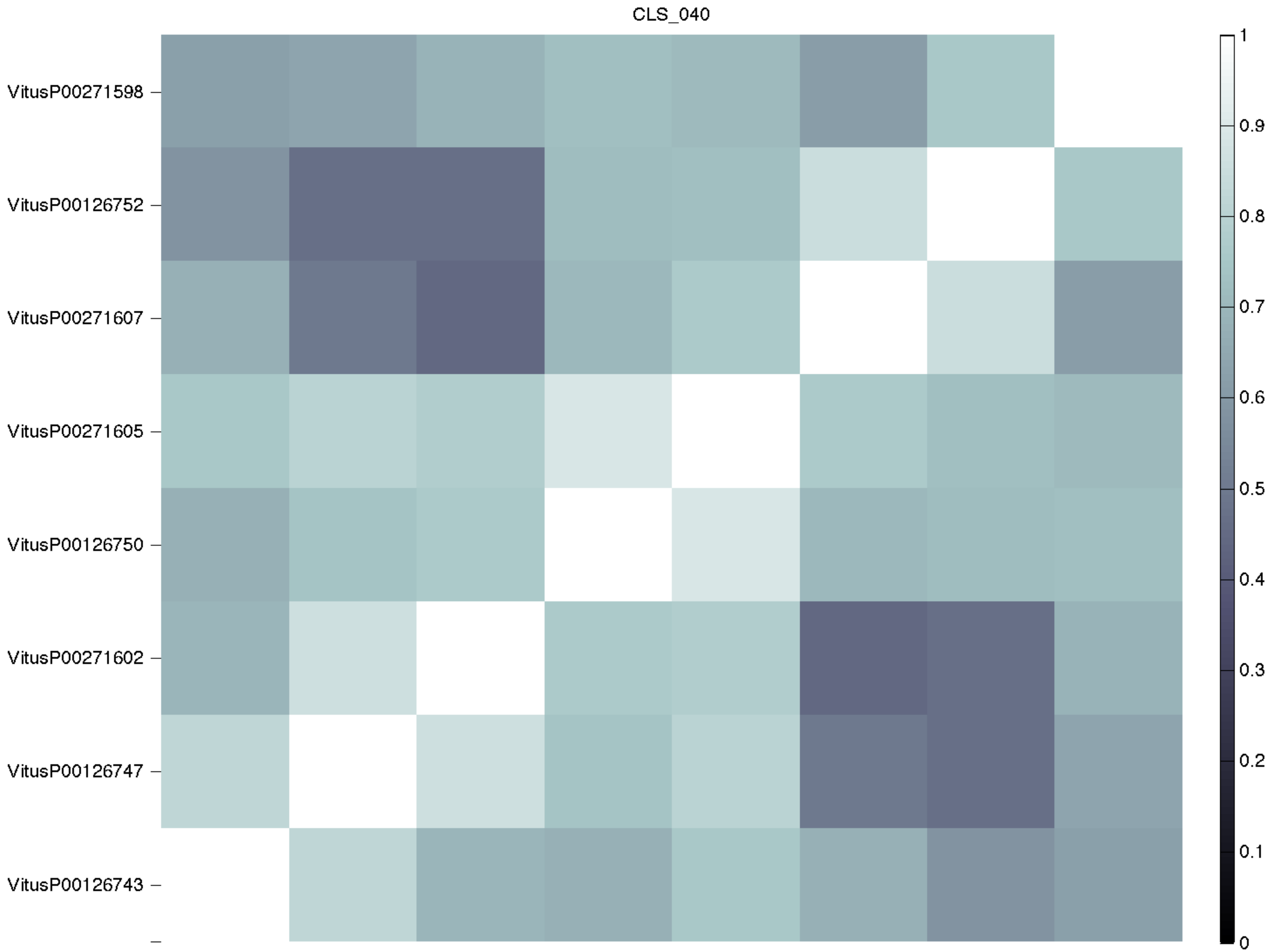

CLS_040
VitusP00271598
VitusP00126752
VitusP00271607
VitusP00271605
VitusP00126750
VitusP00271602
VitusP00126747
VitusP00126743
1
0.9
0.8
0.7
0.6
0.5
0.4
0.3
0.2
0.1
0

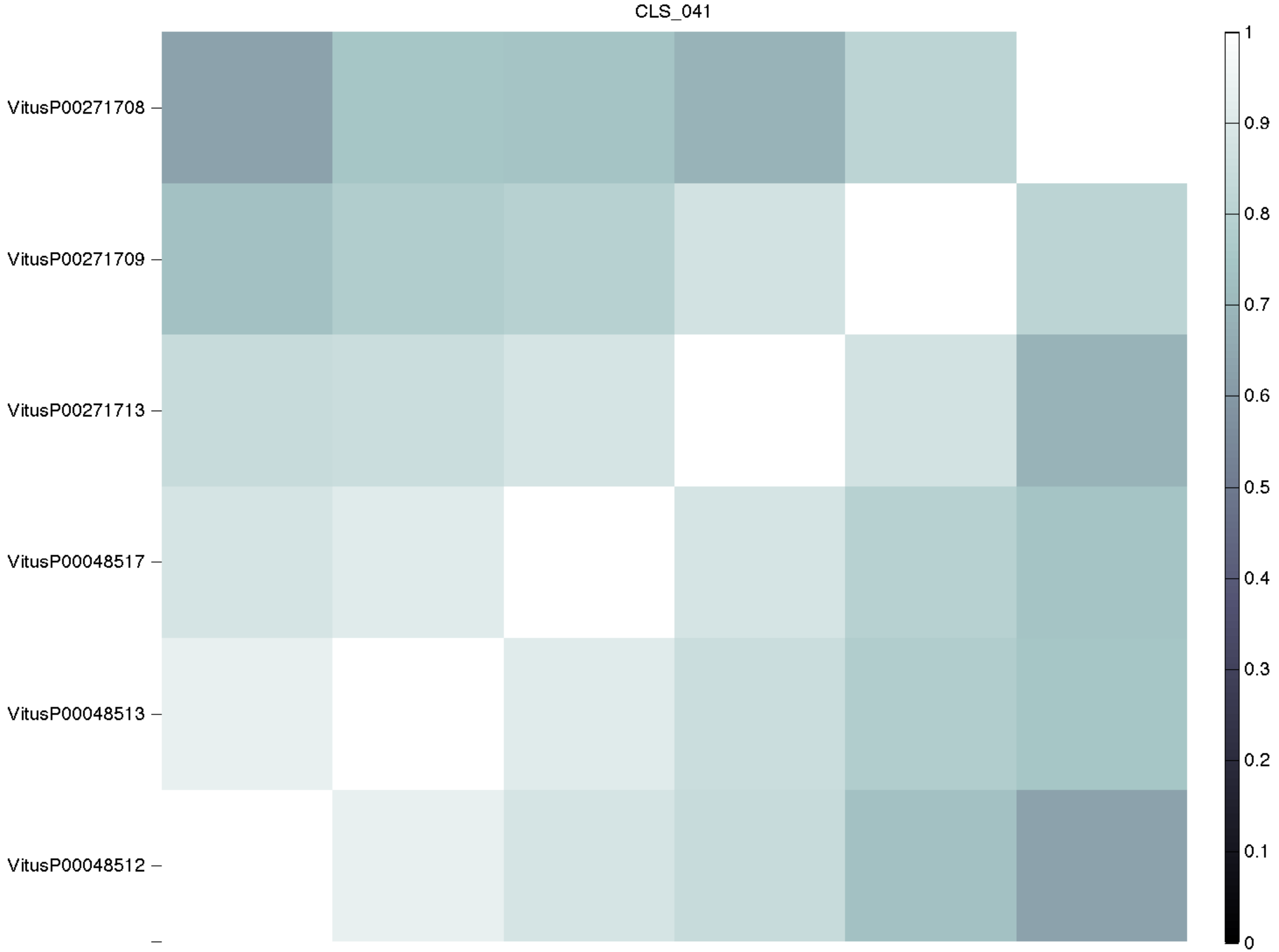
CLS_041
VitusP00271708
VitusP00271709
VitusP00271713
VitusP00048517
VitusP00048513
VitusP00048512
1
0.9
0.8
0.7
0.6
0.5
0.4
0.3
0.2
0.1
0

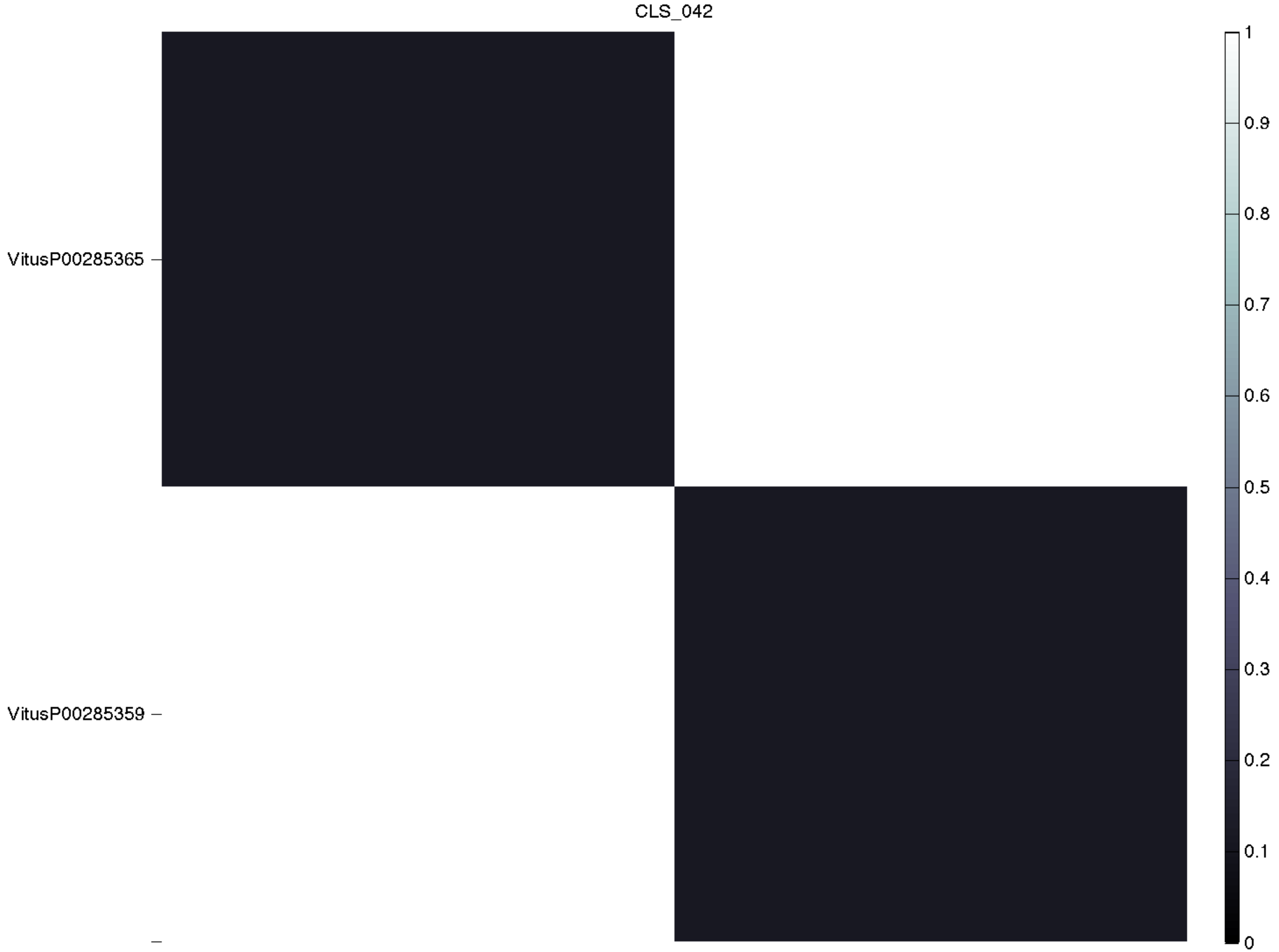
CLS_042
VitusP00285365
VitusP00285359
1
0.9
0.8
0.7
0.6
0.5
0.4
0.3
0.2
0.1
0

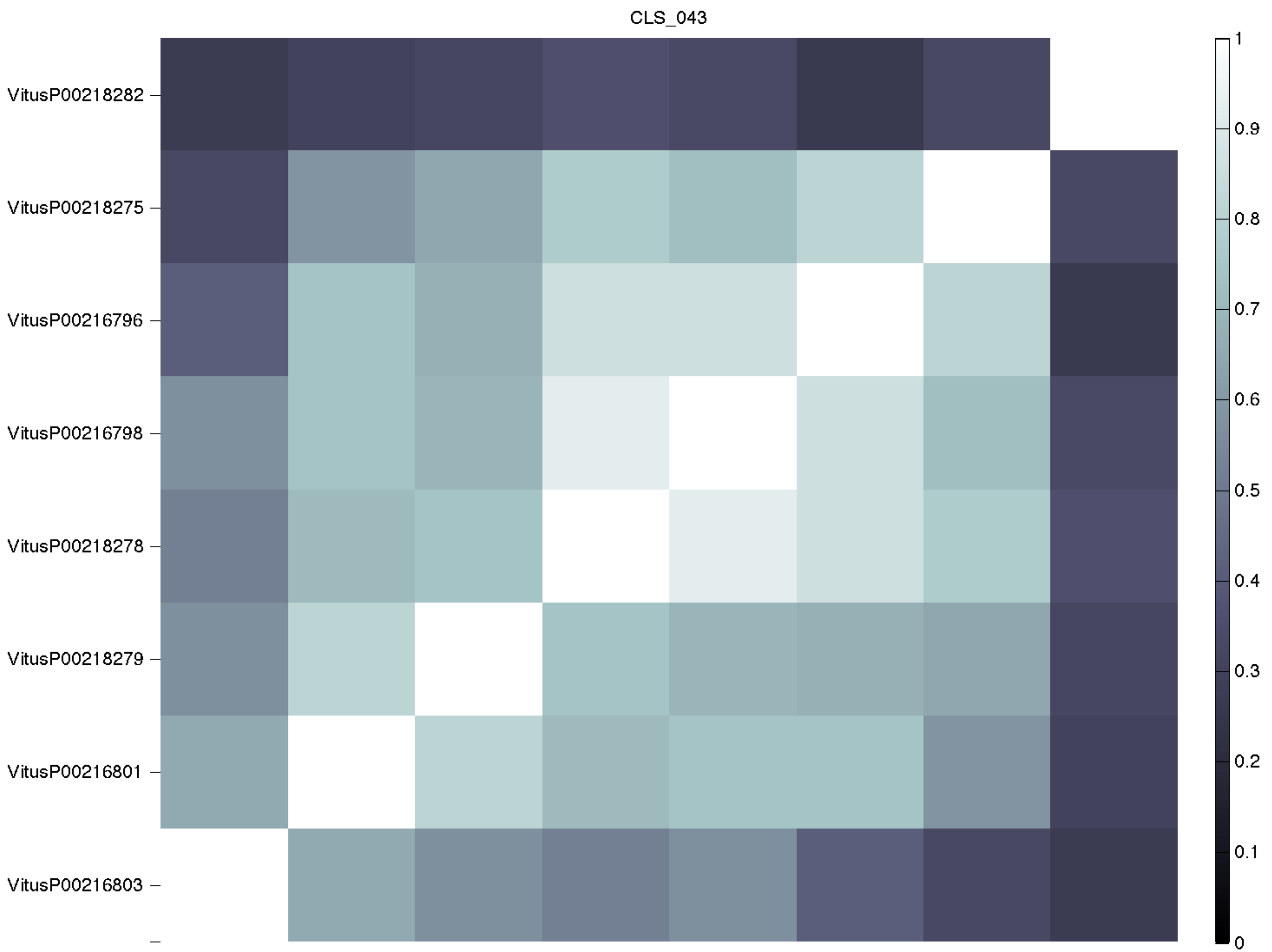
CLS_043
VitusP00218282
VitusP00218275
VitusP00216796
VitusP00216798
VitusP00218278
VitusP00218279
VitusP00216801
VitusP00216803
1
0.9
0.8
0.7
0.6
0.5
0.4
0.3
0.2
0.1
0

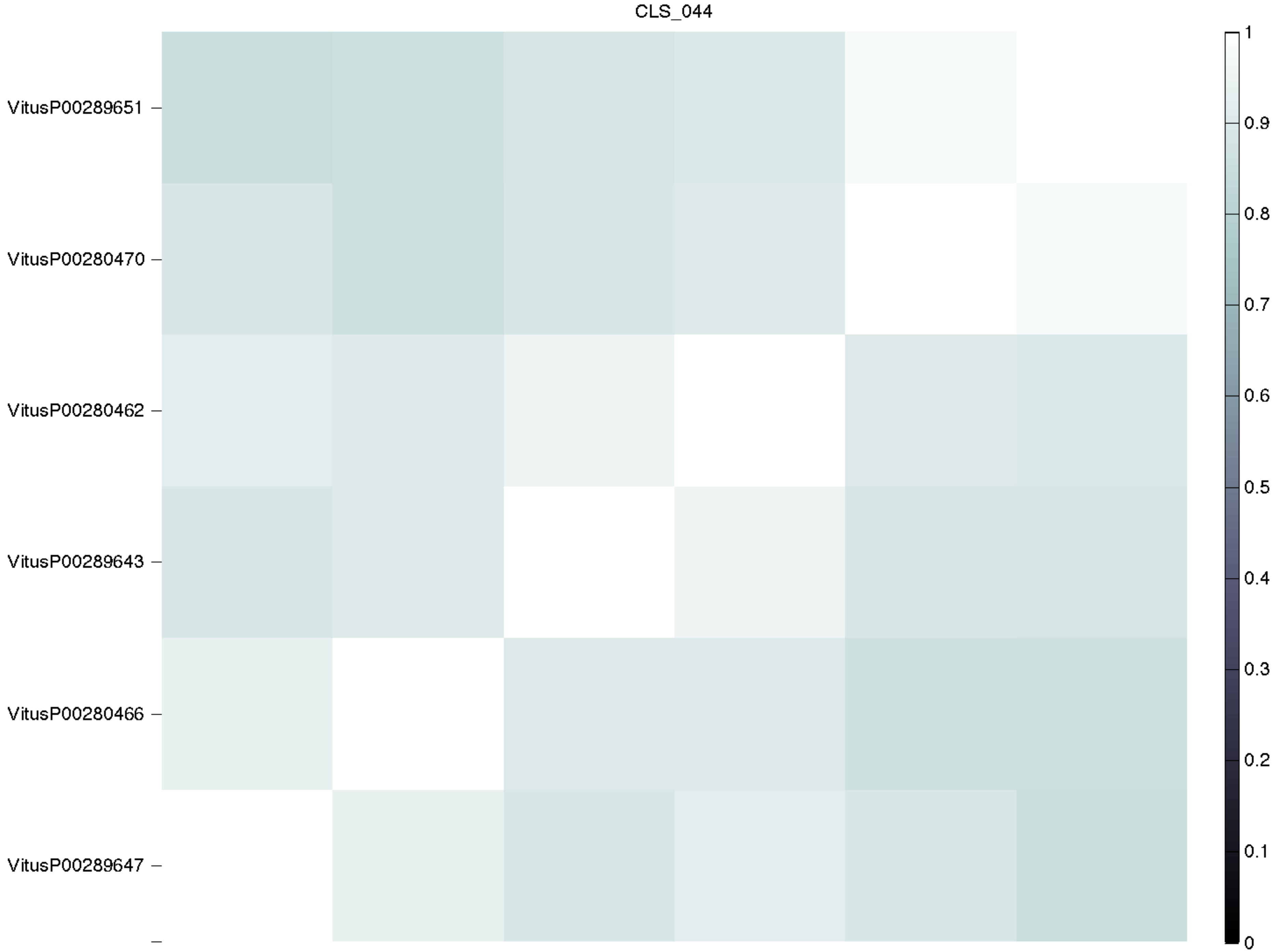
CLS_044
VitusP00289651
VitusP00280470
VitusP00280462
VitusP00289643
VitusP00280466
VitusP00289647
1
0.9
0.8
0.7
0.6
0.5
0.4
0.3
0.2
0.1
0

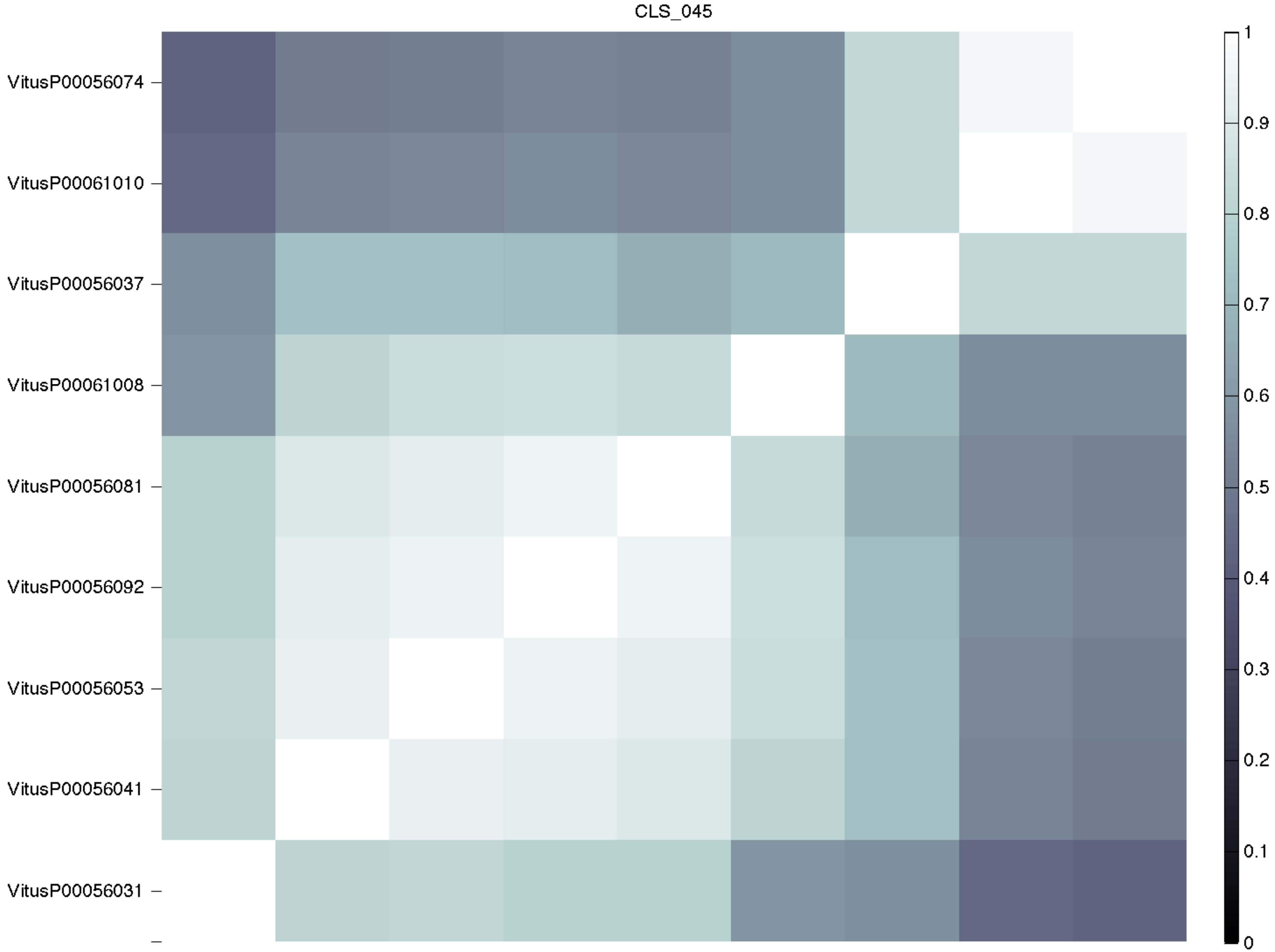
CLS_045
VitusP00056074
VitusP00061010
VitusP00056037
VitusP00061008
VitusP00056081
VitusP00056092
VitusP00056053
VitusP00056041
VitusP00056031
1
0.9
0.8
0.7
0.6
0.5
0.4
0.3
0.2
0.1
0

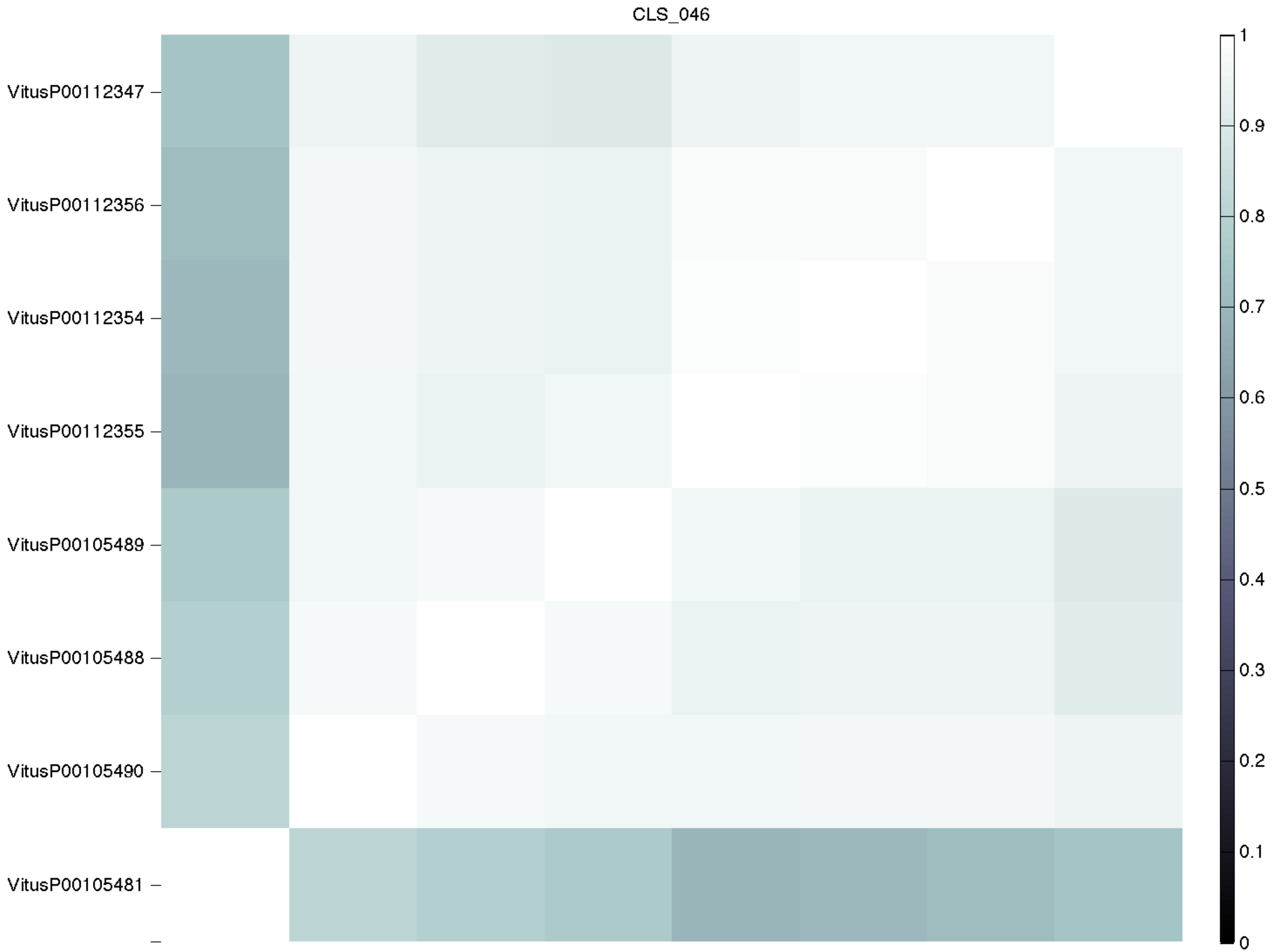

CLS_046
VitusP00112347
VitusP00112356
VitusP00112354
VitusP00112355
VitusP00105489
VitusP00105488
VitusP00105490
VitusP00105481
1
0.9
0.8
0.7
0.6
0.5
0.4
0.3
0.2
0.1
0

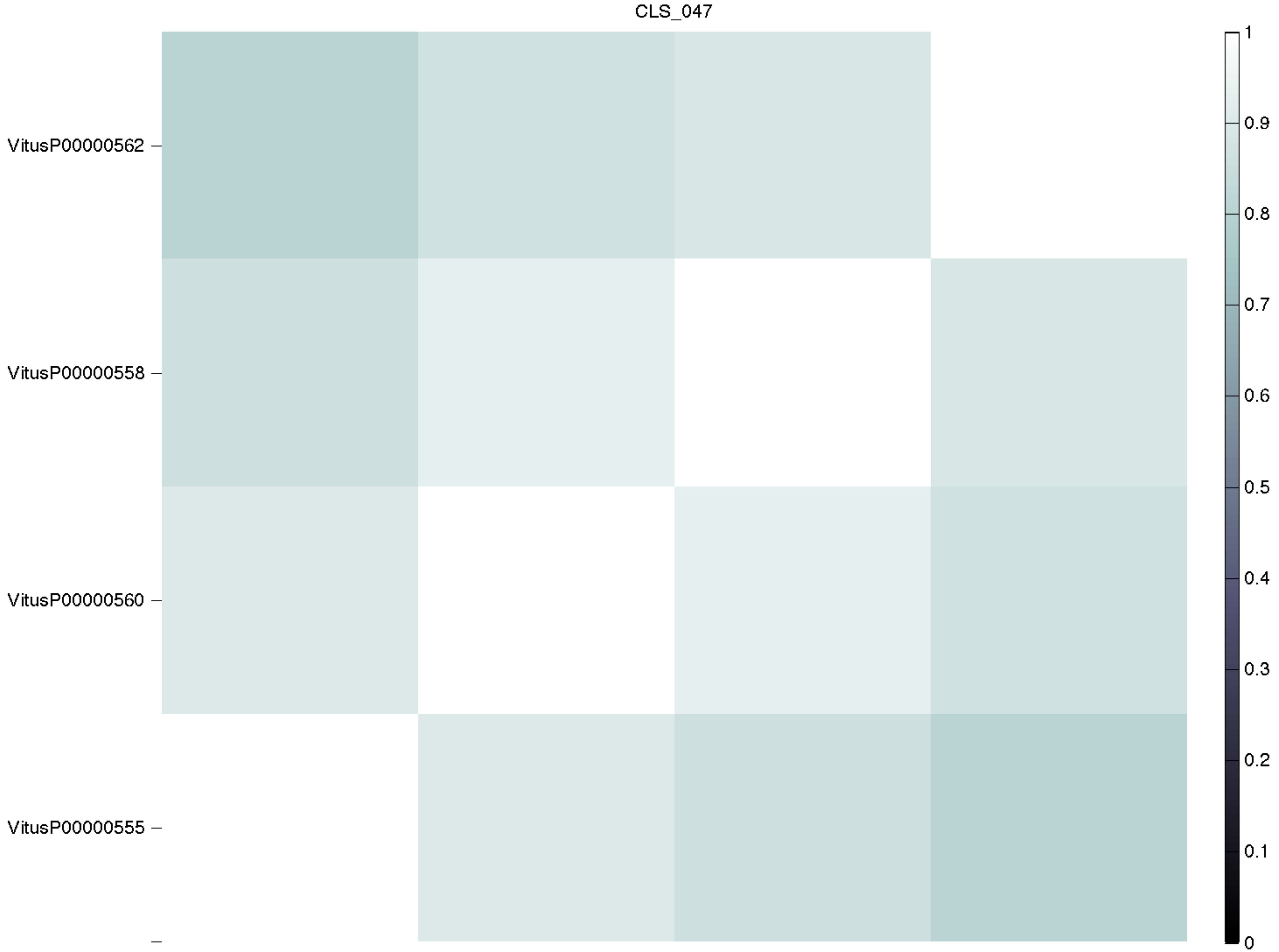
CLS_047
VitusP00000562
VitusP00000558
VitusP00000560
VitusP00000555
1
0.9
0.8
0.7
0.6
0.5
0.4
0.3
0.2
0.1
0

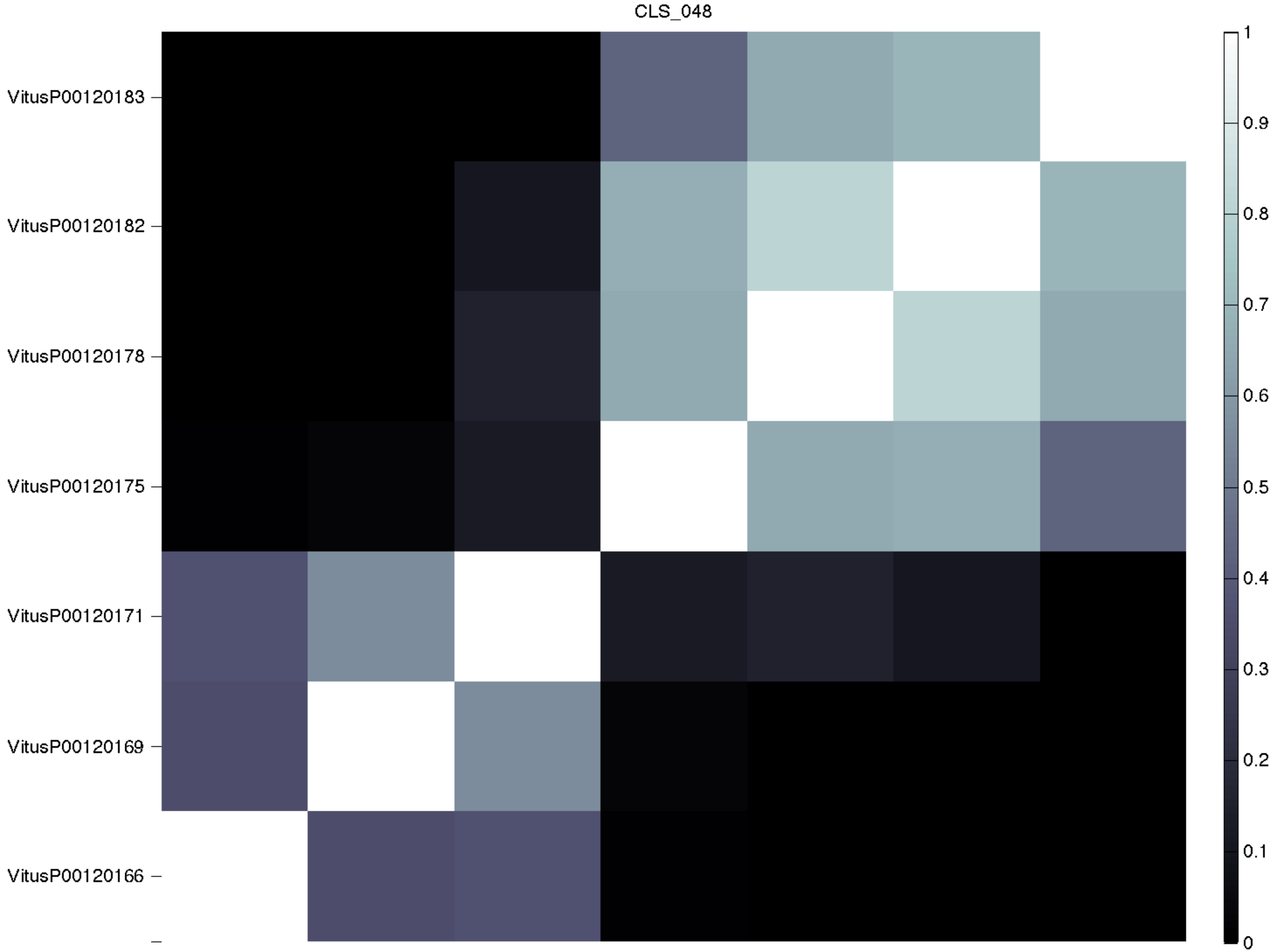
CLS_048
VitusP00120183
VitusP00120182
VitusP00120178
VitusP00120175
VitusP00120171
VitusP00120169
VitusP00120166
1
0.9
0.8
0.7
0.6
0.5
0.4
0.3
0.2
0.1
0

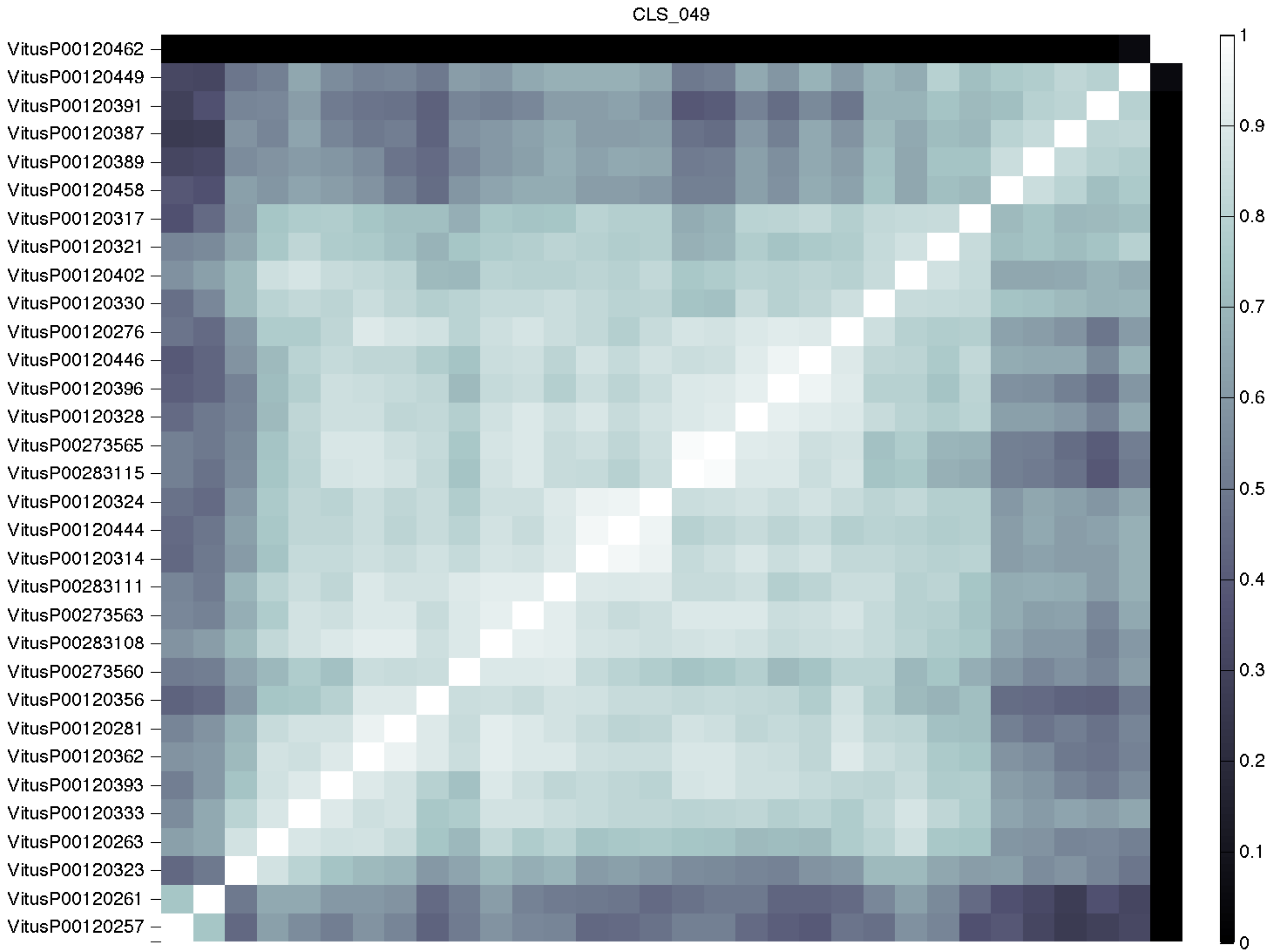
CLS_049
VitusP00120462
VitusP00120449
VitusP00120391
VitusP00120387
VitusP00120389
VitusP00120458
VitusP00120317
VitusP00120321
VitusP00120402
VitusP00120330
VitusP00120276
VitusP00120446
VitusP00120396
VitusP00120328
VitusP00273565
VitusP00283115
VitusP00120324
VitusP00120444
VitusP00120314
VitusP00283111
VitusP00273563
VitusP00283108
VitusP00273560
VitusP00120356
VitusP00120281
VitusP00120362
VitusP00120393
VitusP00120333
VitusP00120263
VitusP00120323
VitusP00120261
VitusP00120257
1
0.9
0.8
0.7
0.6
0.5
0.4
0.3
0.2
0.1
0

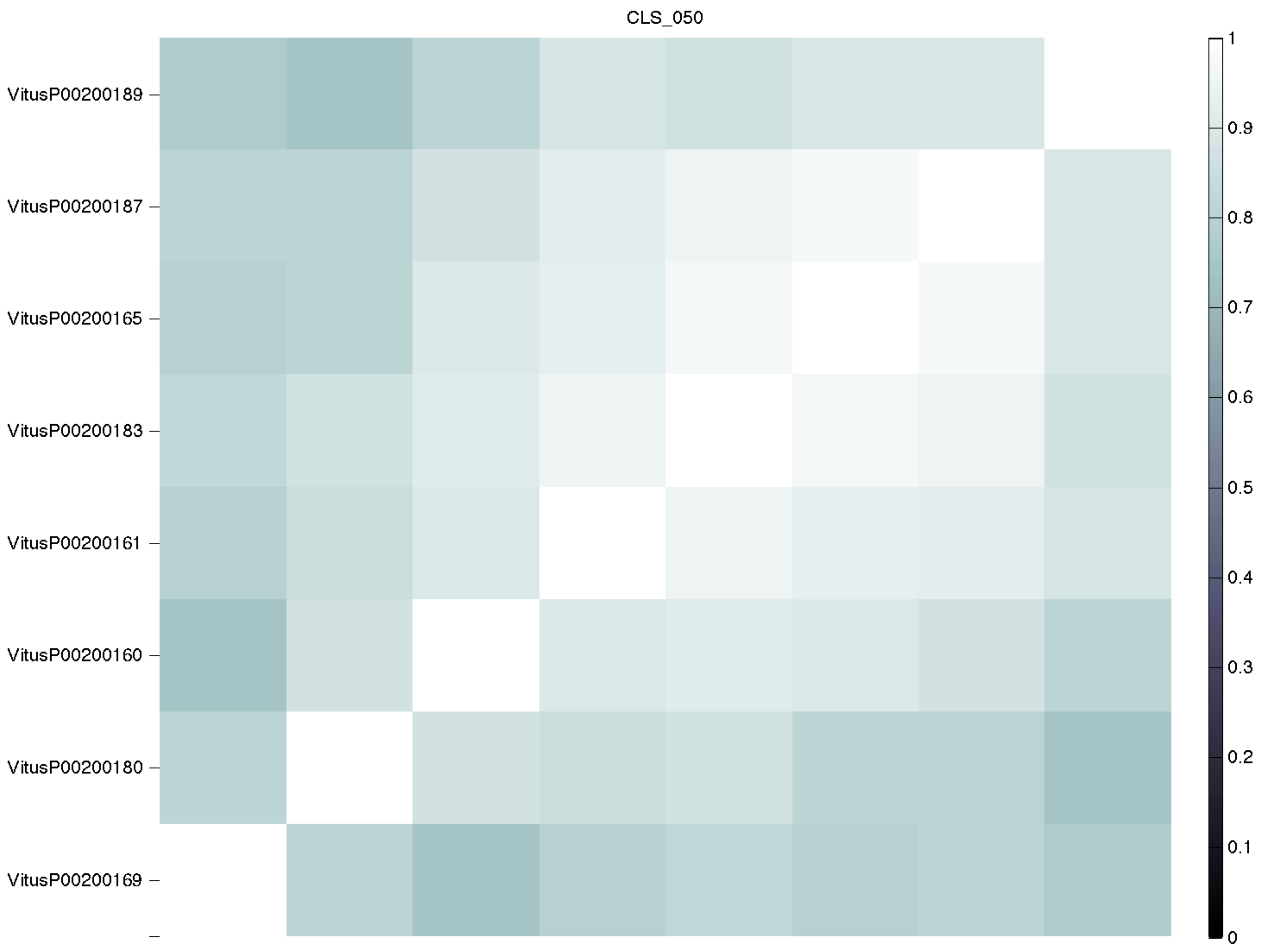
CLS_050
VitusP00200189
VitusP00200187
VitusP00200165
VitusP00200183
VitusP00200161
VitusP00200160
VitusP00200180
VitusP00200169
1
0.9
0.8
0.7
0.6
0.5
0.4
0.3
0.2
0.1
0

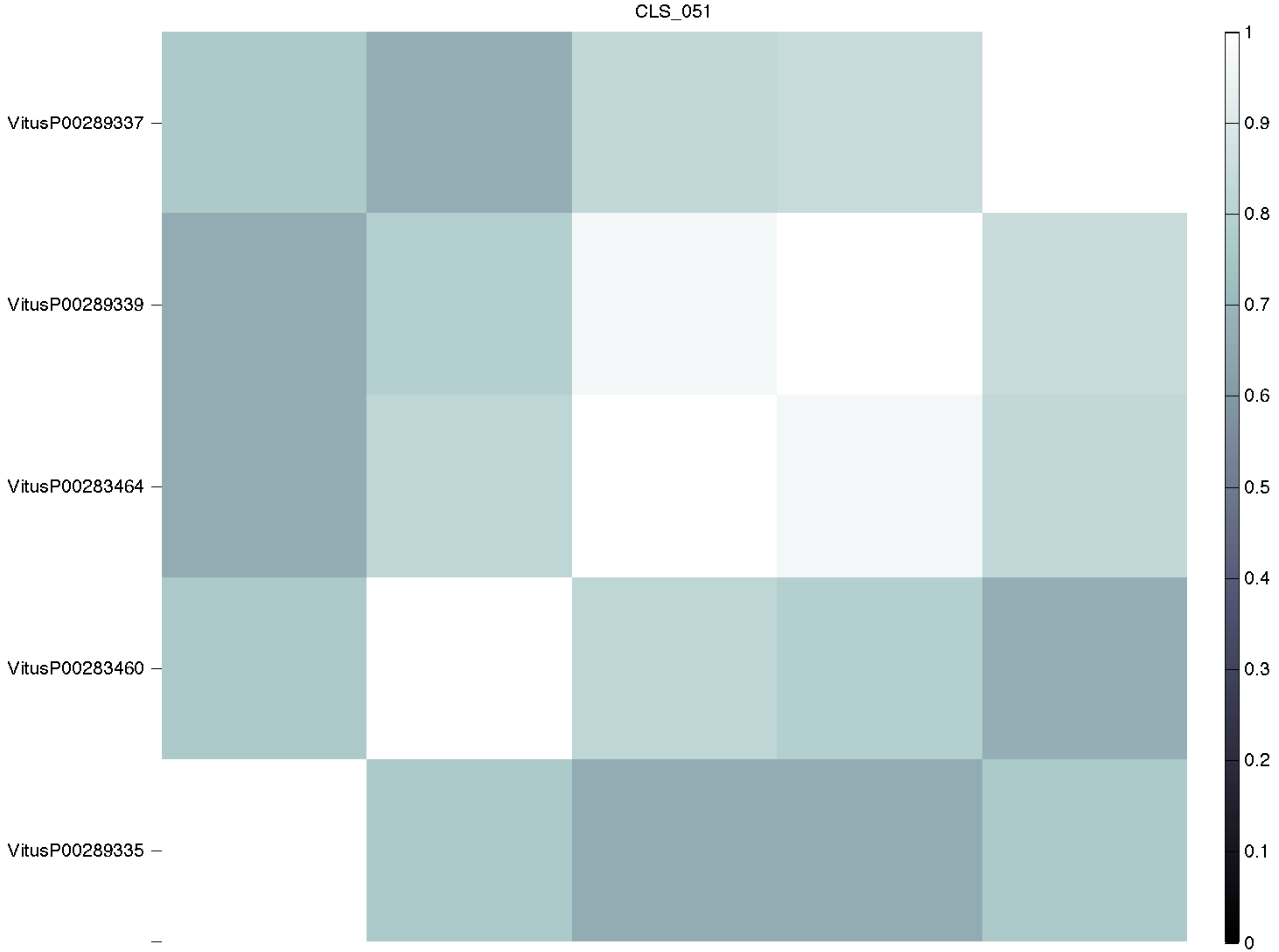

CLS_051
VitusP00289337
VitusP00289339
VitusP00283464
VitusP00283460
VitusP00289335
1
0.9
0.8
0.7
0.6
0.5
0.4
0.3
0.2
0.1
0

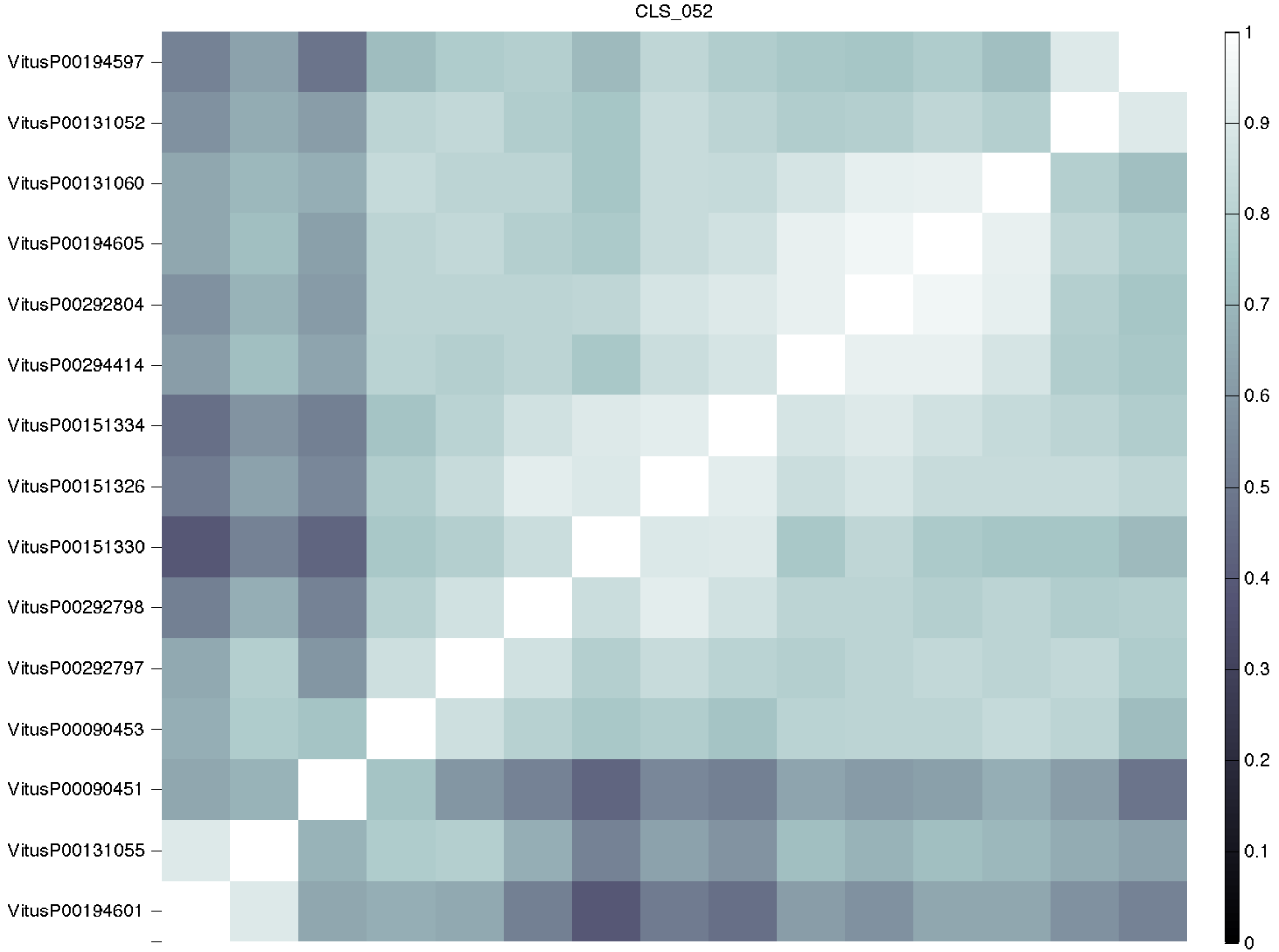

CLS_052
VitusP00194597
VitusP00131052
VitusP00131060
VitusP00194605
VitusP00292804
VitusP00294414
VitusP00151334
VitusP00151326
VitusP00151330
VitusP00292798
VitusP00292797
VitusP00090453
VitusP00090451
VitusP00131055
VitusP00194601
1
0.9
0.8
0.7
0.6
0.5
0.4
0.3
0.2
0.1
0

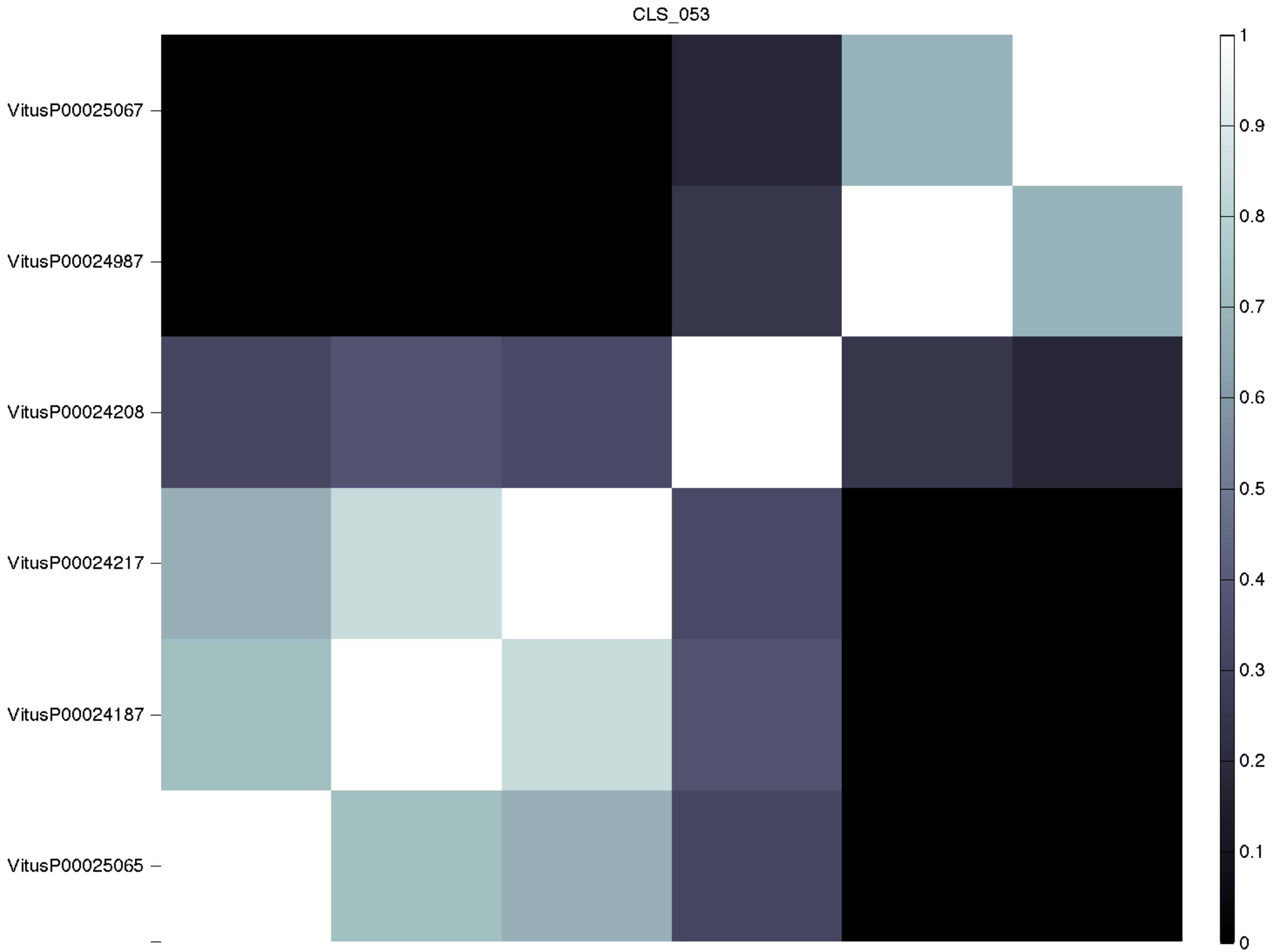
CLS_053
VitusP00025067
VitusP00024987
VitusP00024208
VitusP00024217
VitusP00024187
VitusP00025065
1
0.9
0.8
0.7
0.6
0.5
0.4
0.3
0.2
0.1
0

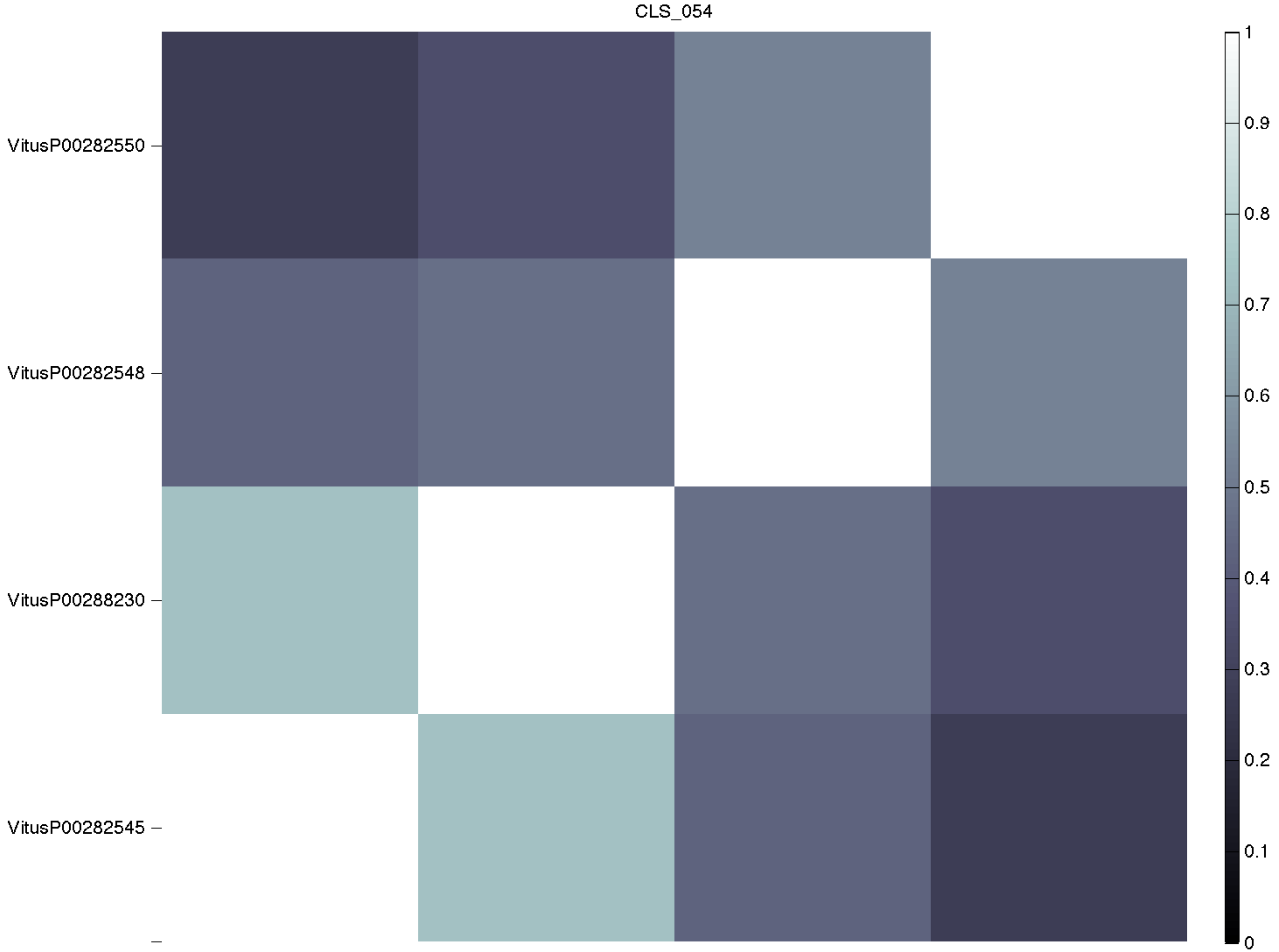

CLS_054
VitusP00282550
VitusP00282548
VitusP00288230
VitusP00282545
1
0.9
0.8
0.7
0.6
0.5
0.4
0.3
0.2
0.1
0

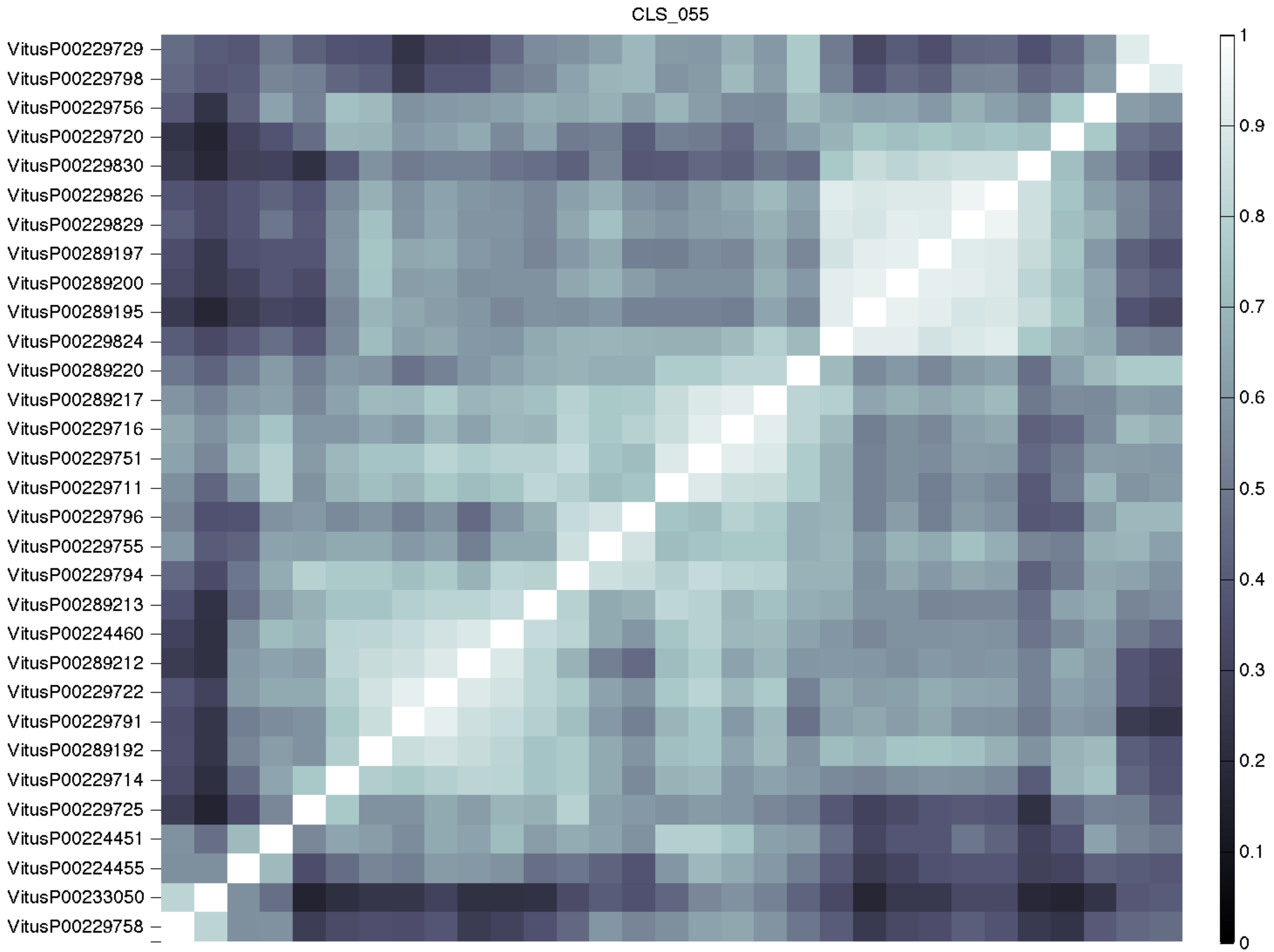

CLS_055
VitusP00229729
VitusP00229798
VitusP00229756
VitusP00229720
VitusP00229830
VitusP00229826
VitusP00229829
VitusP00289197
VitusP00289200
VitusP00289195
VitusP00229824
VitusP00289220
VitusP00289217
VitusP00229716
VitusP00229751
VitusP00229711
VitusP00229796
VitusP00229755
VitusP00229794
VitusP00289213
VitusP00224460
VitusP00289212
VitusP00229722
VitusP00229791
VitusP00289192
VitusP00229714
VitusP00229725
VitusP00224451
VitusP00224455
VitusP00233050
VitusP00229758
1
0.9
0.8
0.7
0.6
0.5
0.4
0.3
0.2
0.1
0

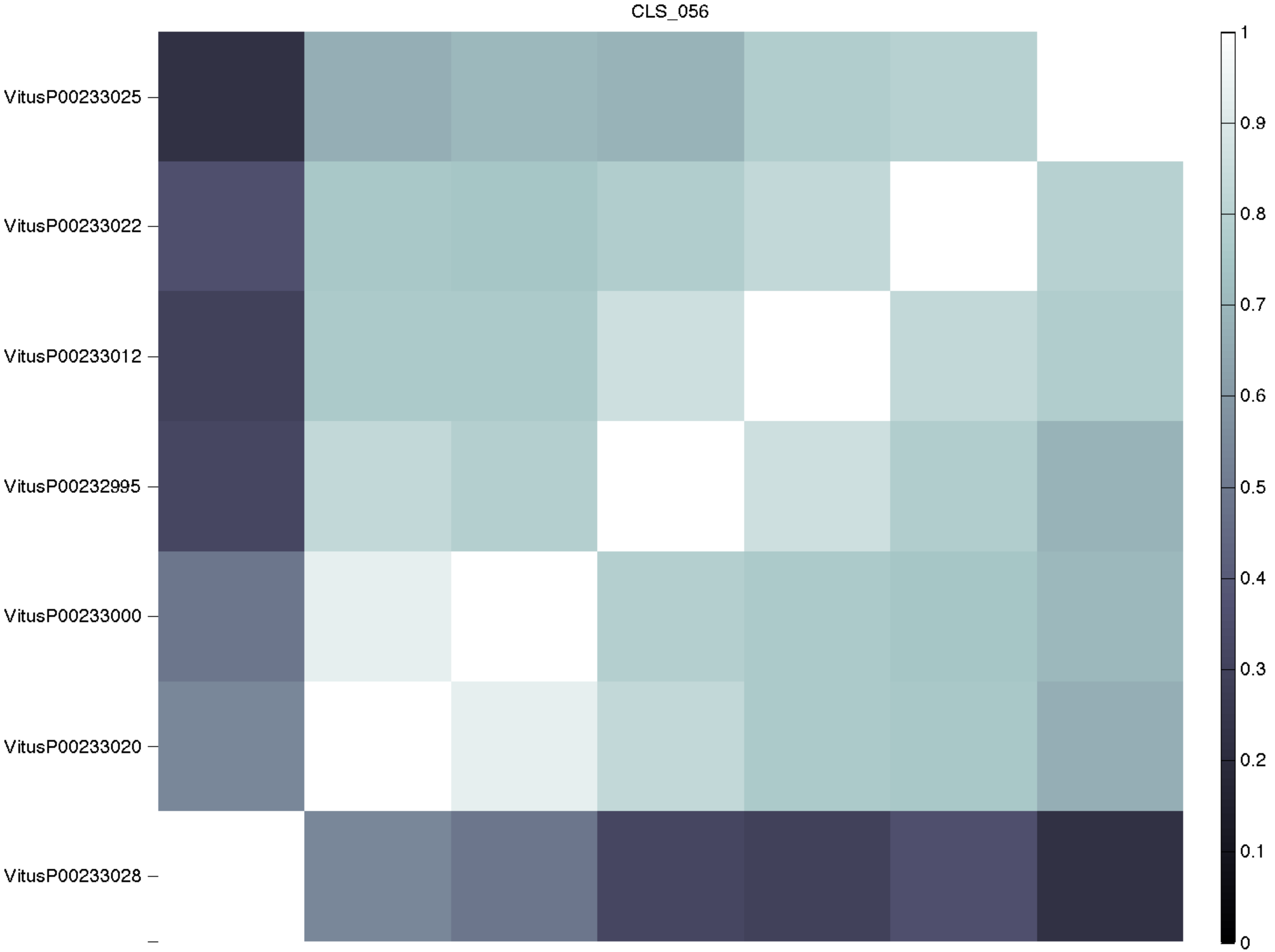
CLS_056
VitusP00233025
VitusP00233022
VitusP00233012
VitusP00232995
VitusP00233000
VitusP00233020
VitusP00233028
1
0.9
0.8
0.7
0.6
0.5
0.4
0.3
0.2
0.1
0

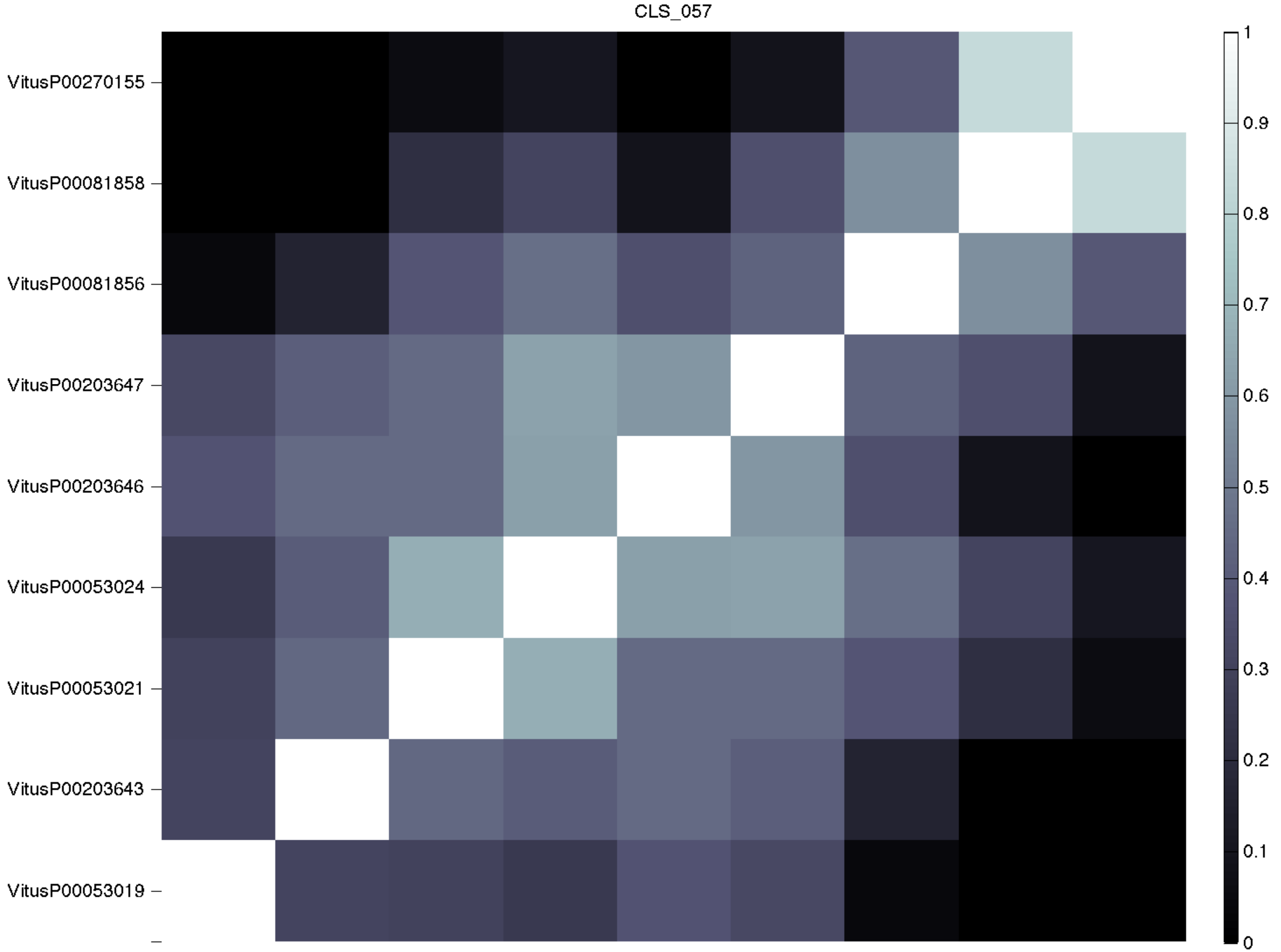
CLS_057
VitusP00270155
VitusP00081858
VitusP00081856
VitusP00203647
VitusP00203646
VitusP00053024
VitusP00053021
VitusP00203643
VitusP00053019
1
0.9
0.8
0.7
0.6
0.5
0.4
0.3
0.2
0.1
0

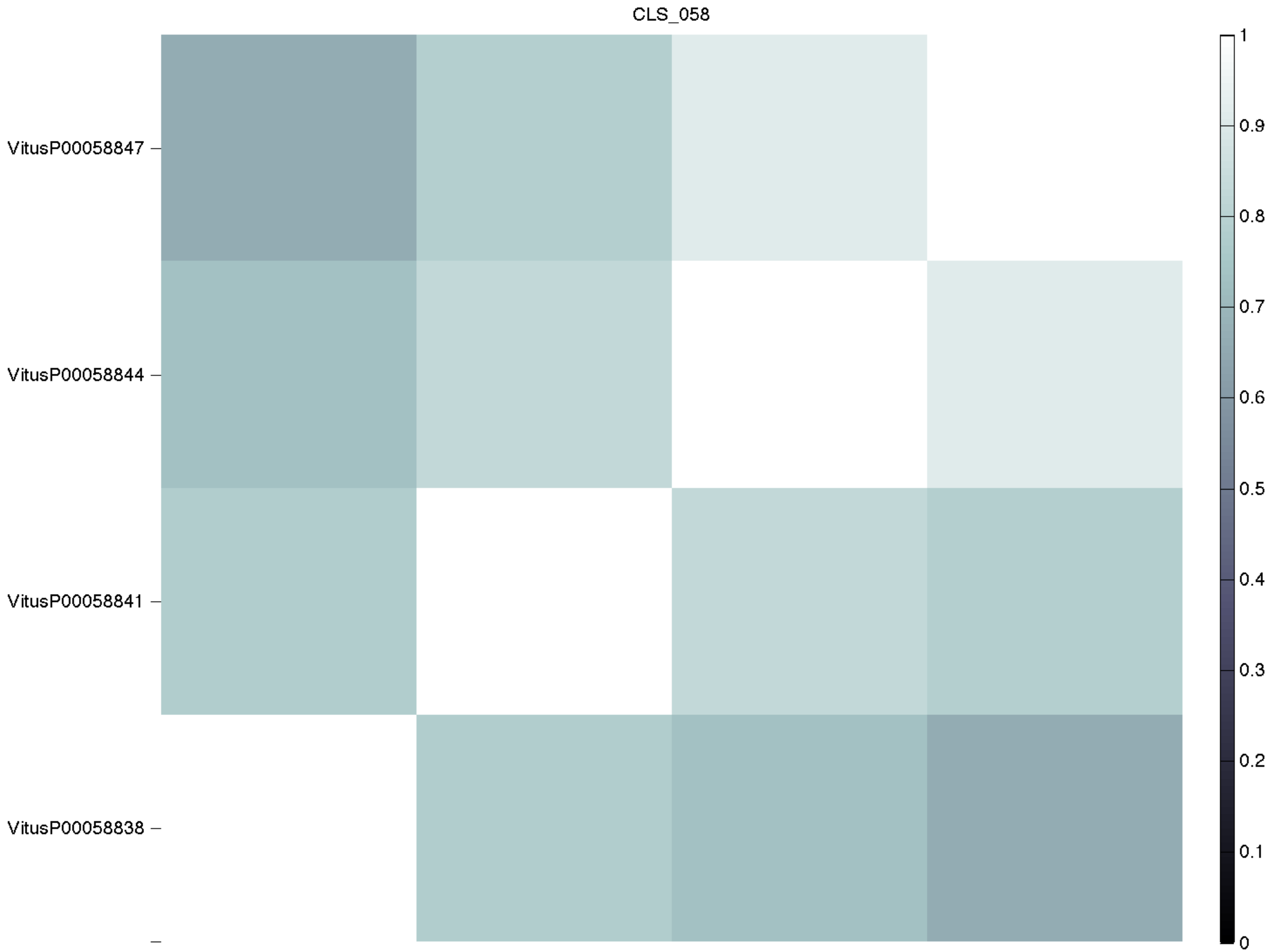
CLS_058
VitusP00058847
VitusP00058844
VitusP00058841
VitusP00058838
1
0.9
0.8
0.7
0.6
0.5
0.4
0.3
0.2
0.1
0

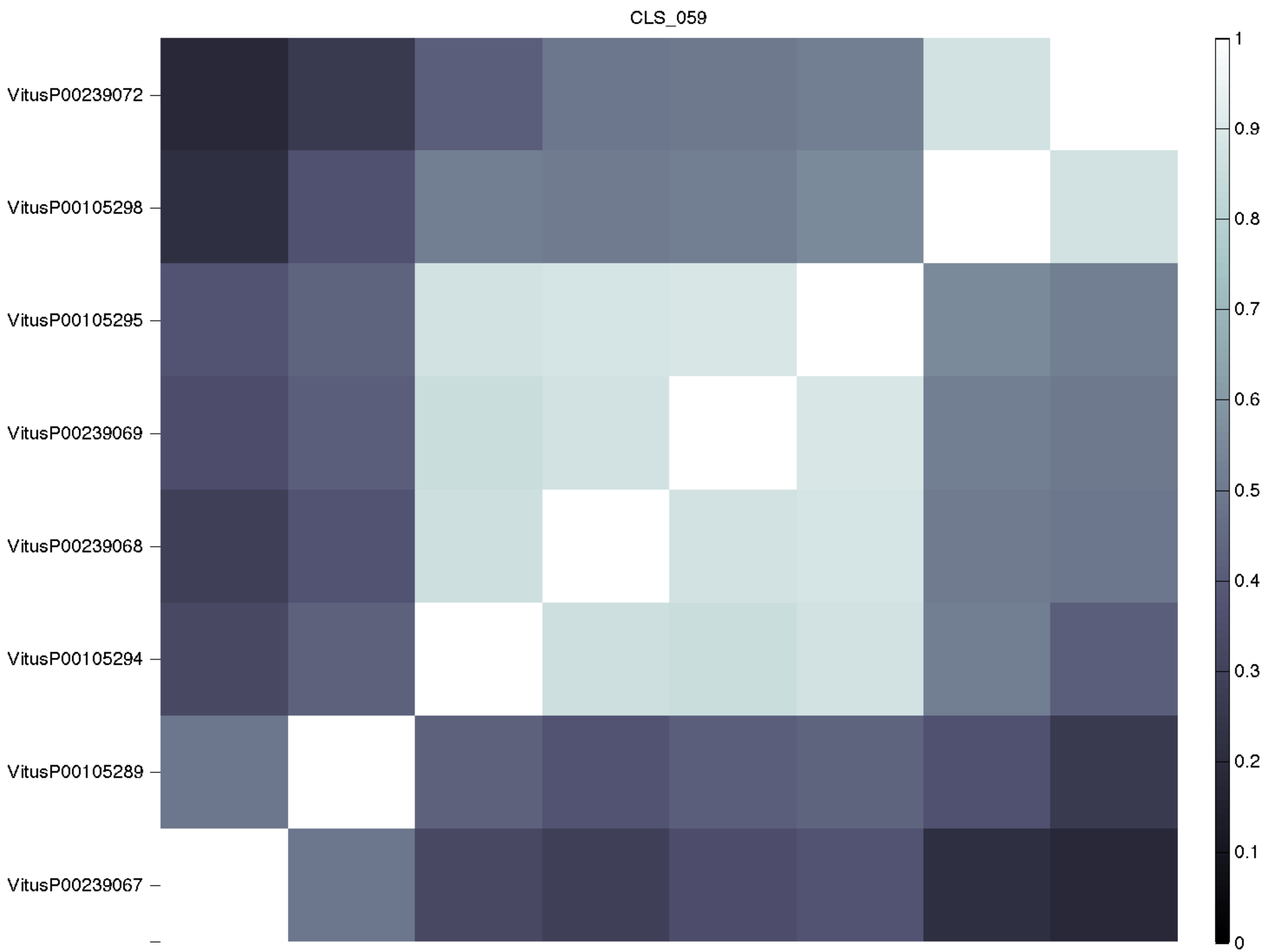
CLS_059
VitusP00239072
VitusP00105298
VitusP00105295
VitusP00239069
VitusP00239068
VitusP00105294
VitusP00105289
VitusP00239067
1
0.9
0.8
0.7
0.6
0.5
0.4
0.3
0.2
0.1
0

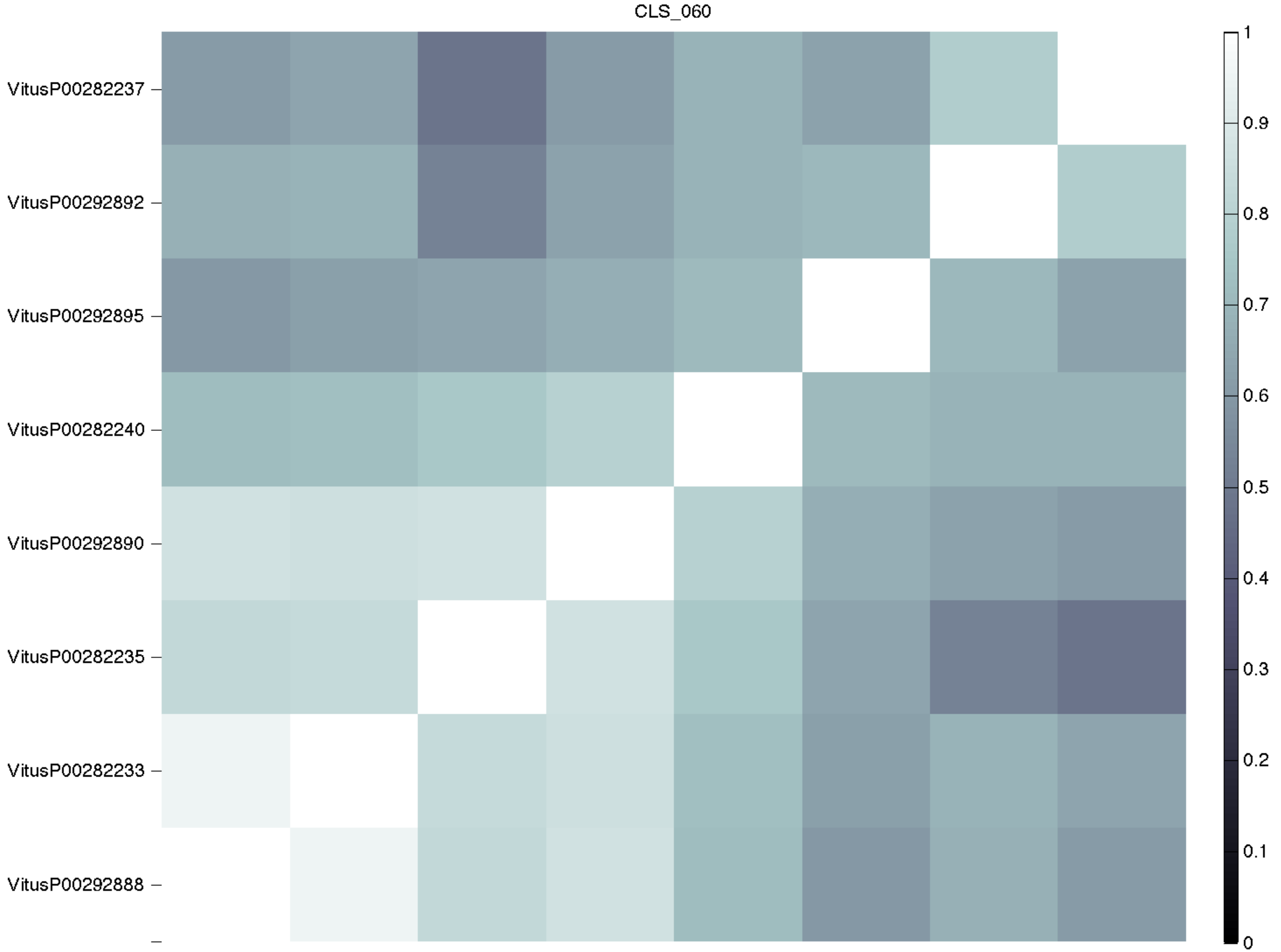
CLS_060
VitusP00282237
VitusP00292892
VitusP00292895
VitusP00282240
VitusP00292890
VitusP00282235
VitusP00282233
VitusP00292888
1
0.9
0.8
0.7
0.6
0.5
0.4
0.3
0.2
0.1
0

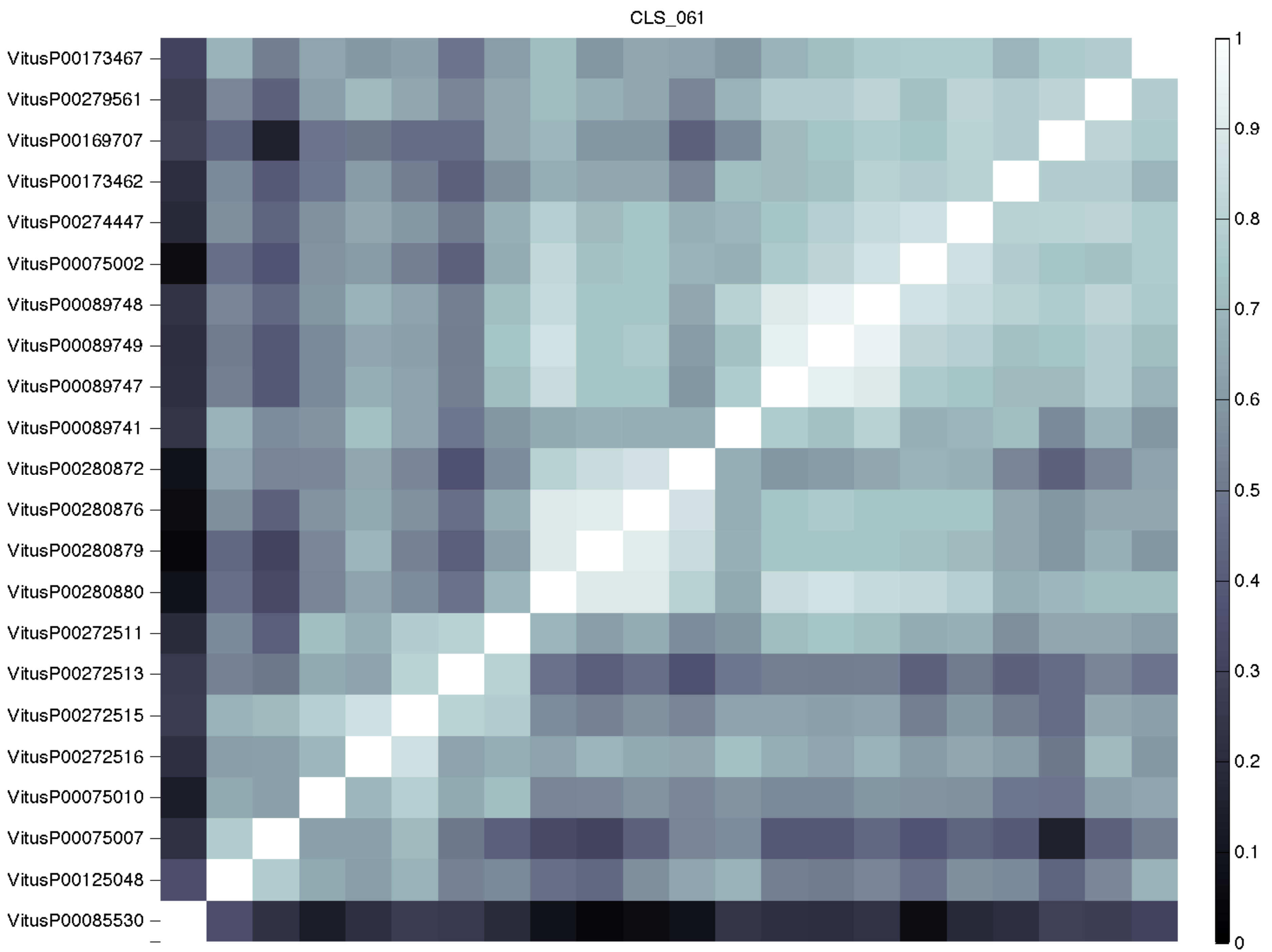
CLS_061
VitusP00173467
VitusP00279561
VitusP00169707
VitusP00173462
VitusP00274447
VitusP00075002
VitusP00089748
VitusP00089749
VitusP00089747
VitusP00089741
VitusP00280872
VitusP00280876
VitusP00280879
VitusP00280880
VitusP00272511
VitusP00272513
VitusP00272515
VitusP00272516
VitusP00075010
VitusP00075007
VitusP00125048
VitusP00085530
1
0.9
0.8
0.7
0.6
0.5
0.4
0.3
0.2
0.1
0

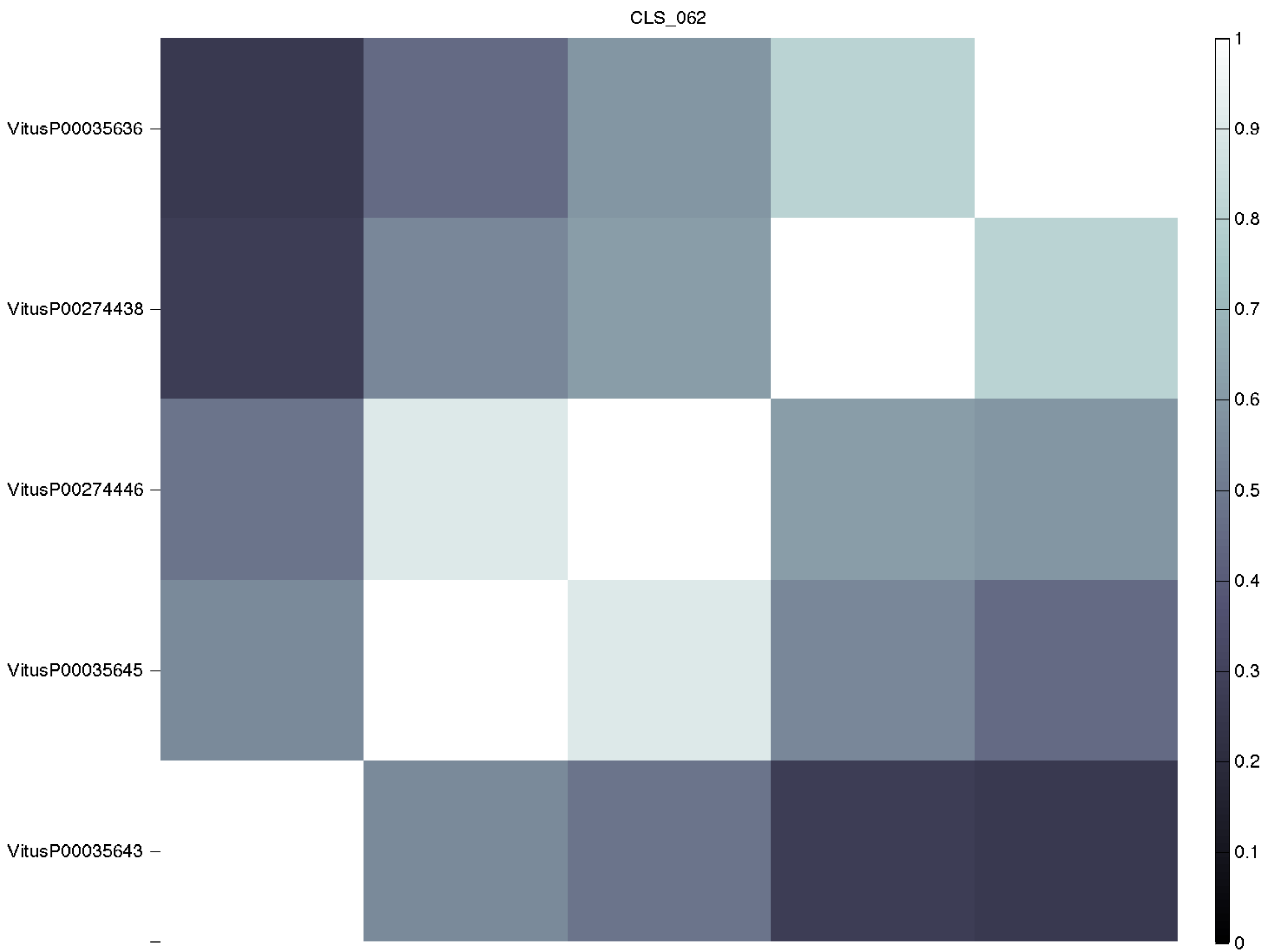
CLS_062
VitusP00035636
VitusP00274438
VitusP00274446
VitusP00035645
VitusP00035643
1
0.9
0.8
0.7
0.6
0.5
0.4
0.3
0.2
0.1
0

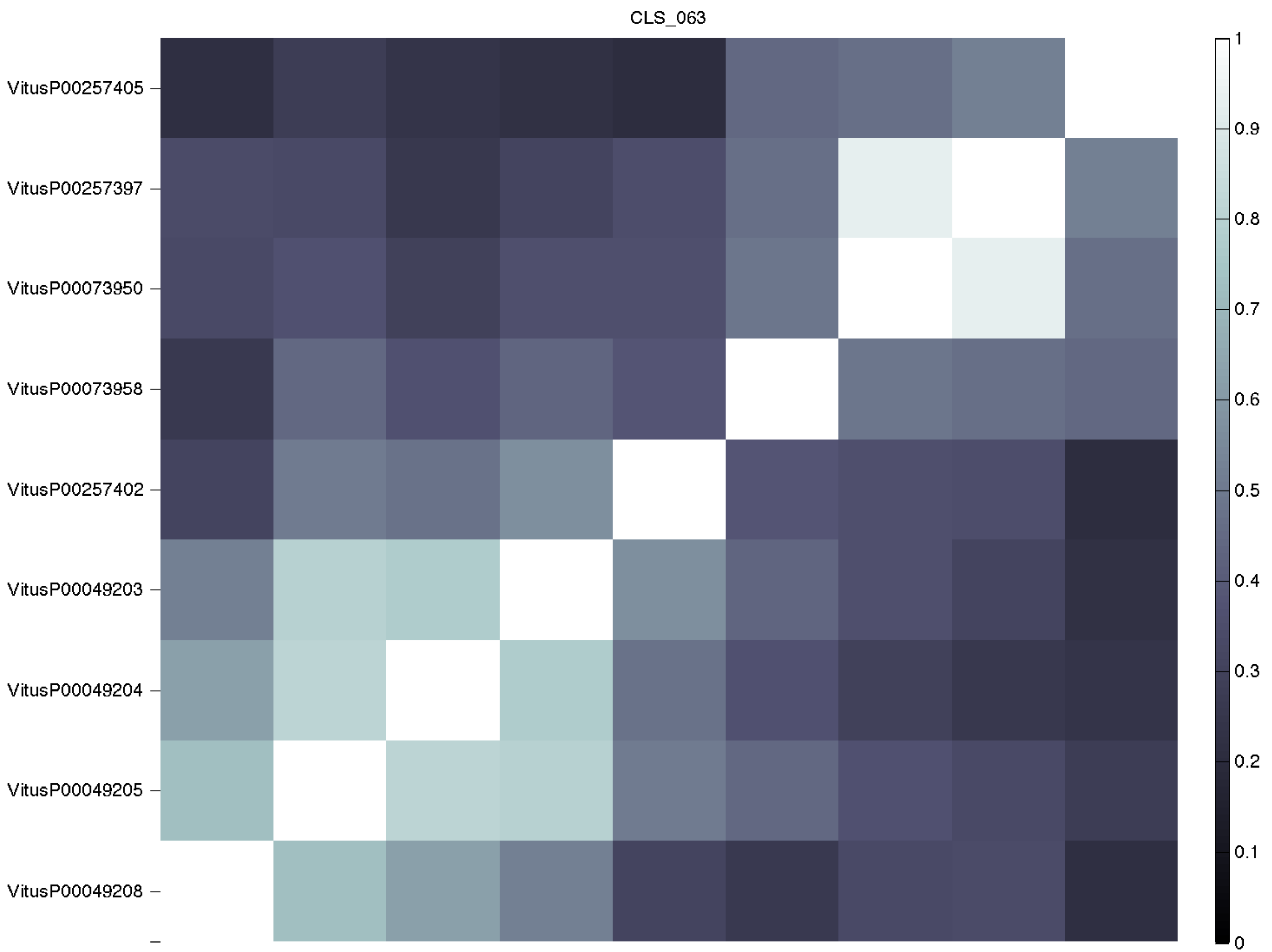
CLS_063
VitusP00257405
VitusP00257397
VitusP00073950
VitusP00073958
VitusP00257402
VitusP00049203
VitusP00049204
VitusP00049205
VitusP00049208
1
0.9
0.8
0.7
0.6
0.5
0.4
0.3
0.2
0.1
0

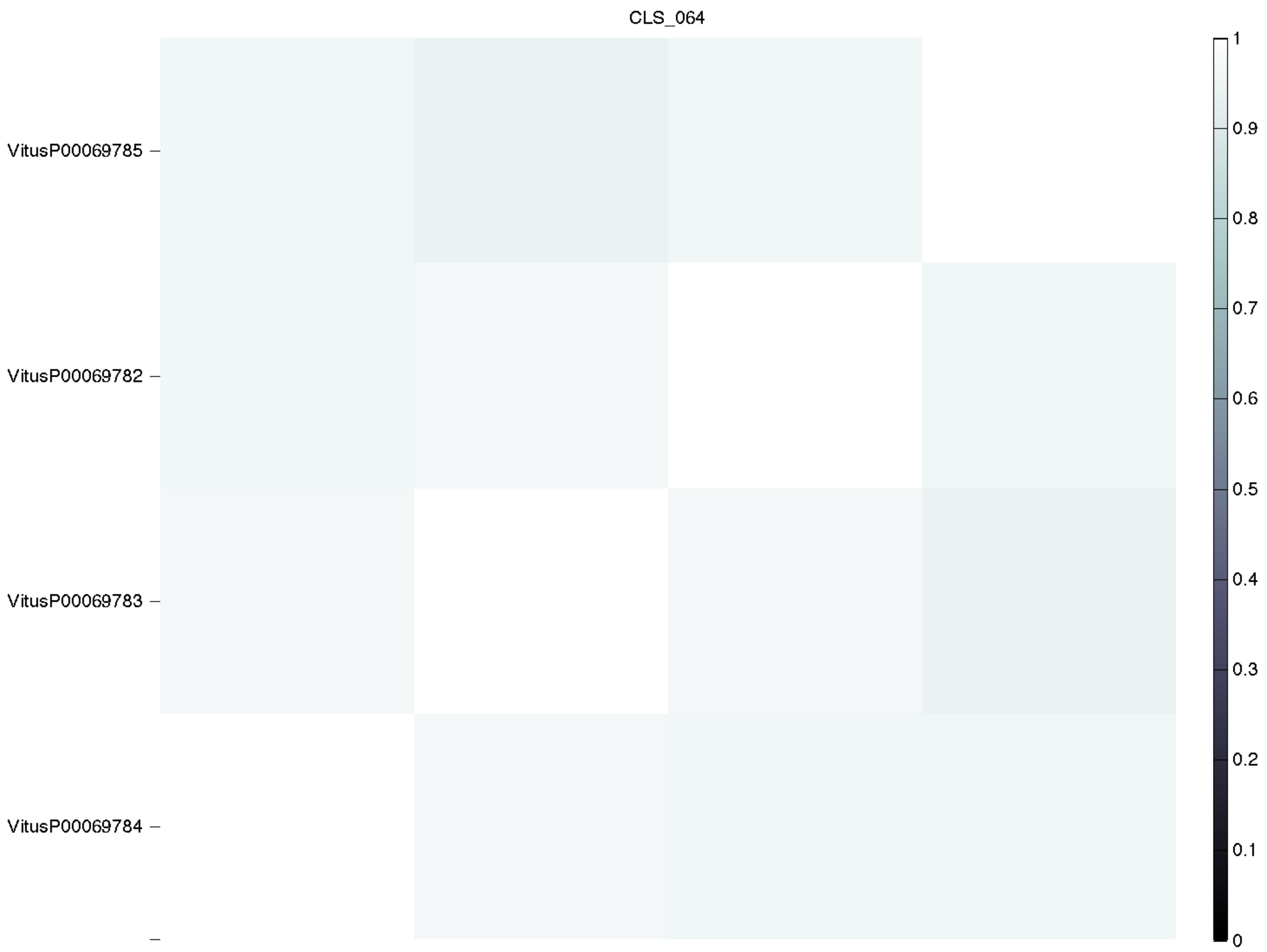
CLS_064
VitusP00069785
VitusP00069782
VitusP00069783
VitusP00069784
1
0.9
0.8
0.7
0.6
0.5
0.4
0.3
0.2
0.1
0

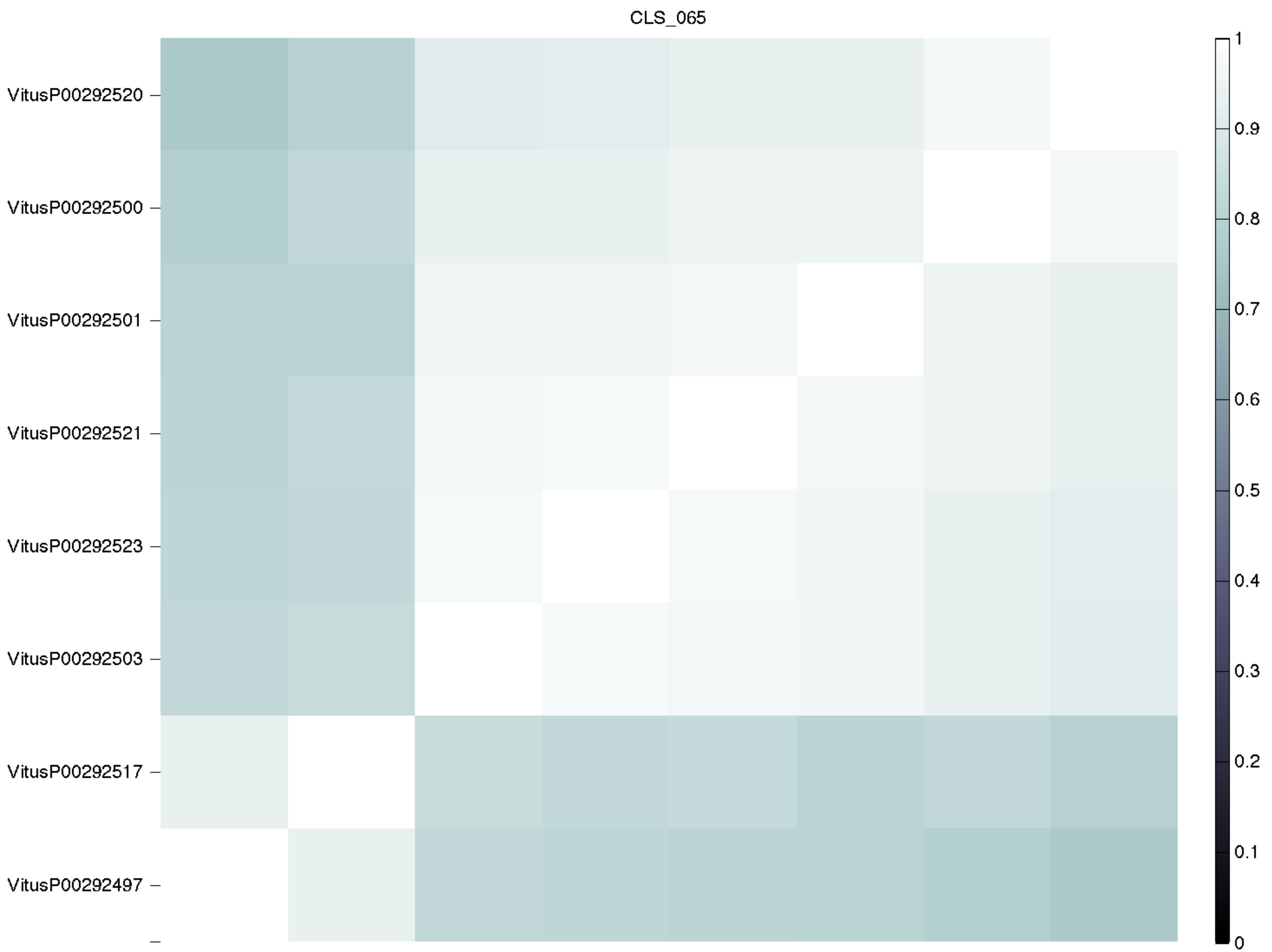
CLS_065
VitusP00292520
VitusP00292500
VitusP00292501
VitusP00292521
VitusP00292523
VitusP00292503
VitusP00292517
VitusP00292497
1
0.9
0.8
0.7
0.6
0.5
0.4
0.3
0.2
0.1
0

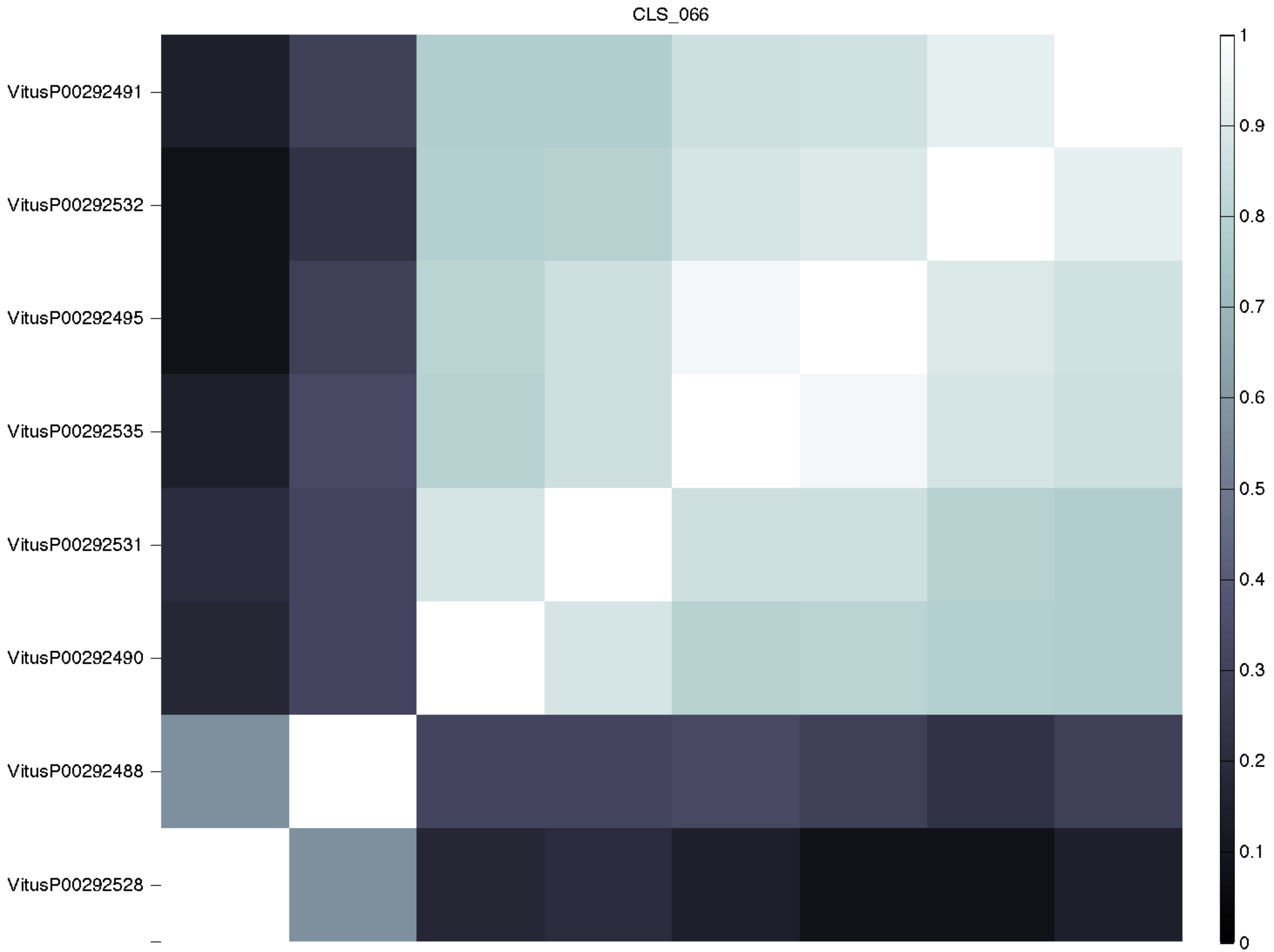CLS_066
VitusP00292491
VitusP00292532
VitusP00292495
VitusP00292535
VitusP00292531
VitusP00292490
VitusP00292488
VitusP00292528
1
0.9
0.8
0.7
0.6
0.5
0.4
0.3
0.2
0.1
0

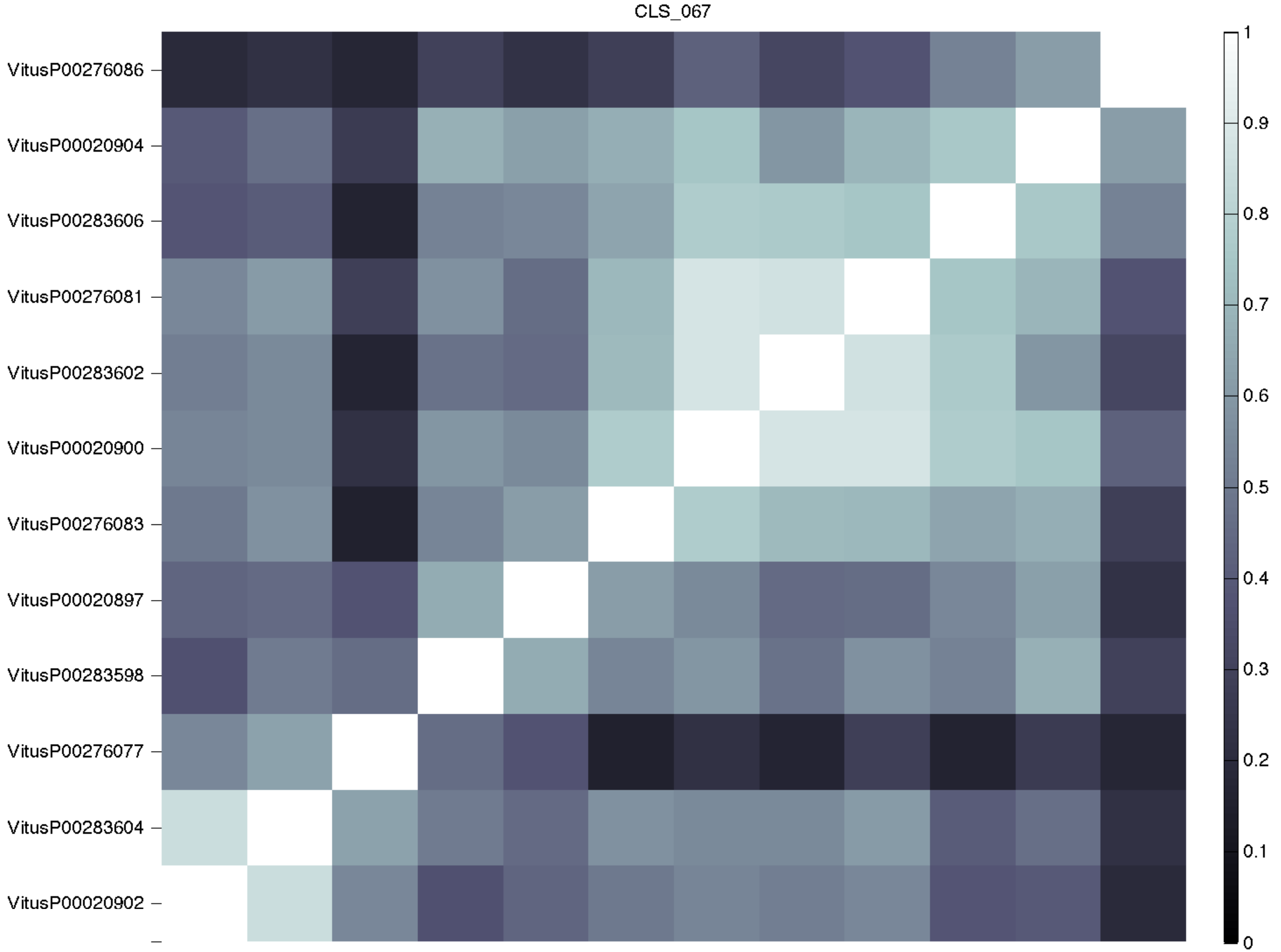
CLS_067
VitusP00276086
VitusP00020904
VitusP00283606
VitusP00276081
VitusP00283602
VitusP00020900
VitusP00276083
VitusP00020897
VitusP00283598
VitusP00276077
VitusP00283604
VitusP00020902
1
0.9
0.8
0.7
0.6
0.5
0.4
0.3
0.2
0.1
0

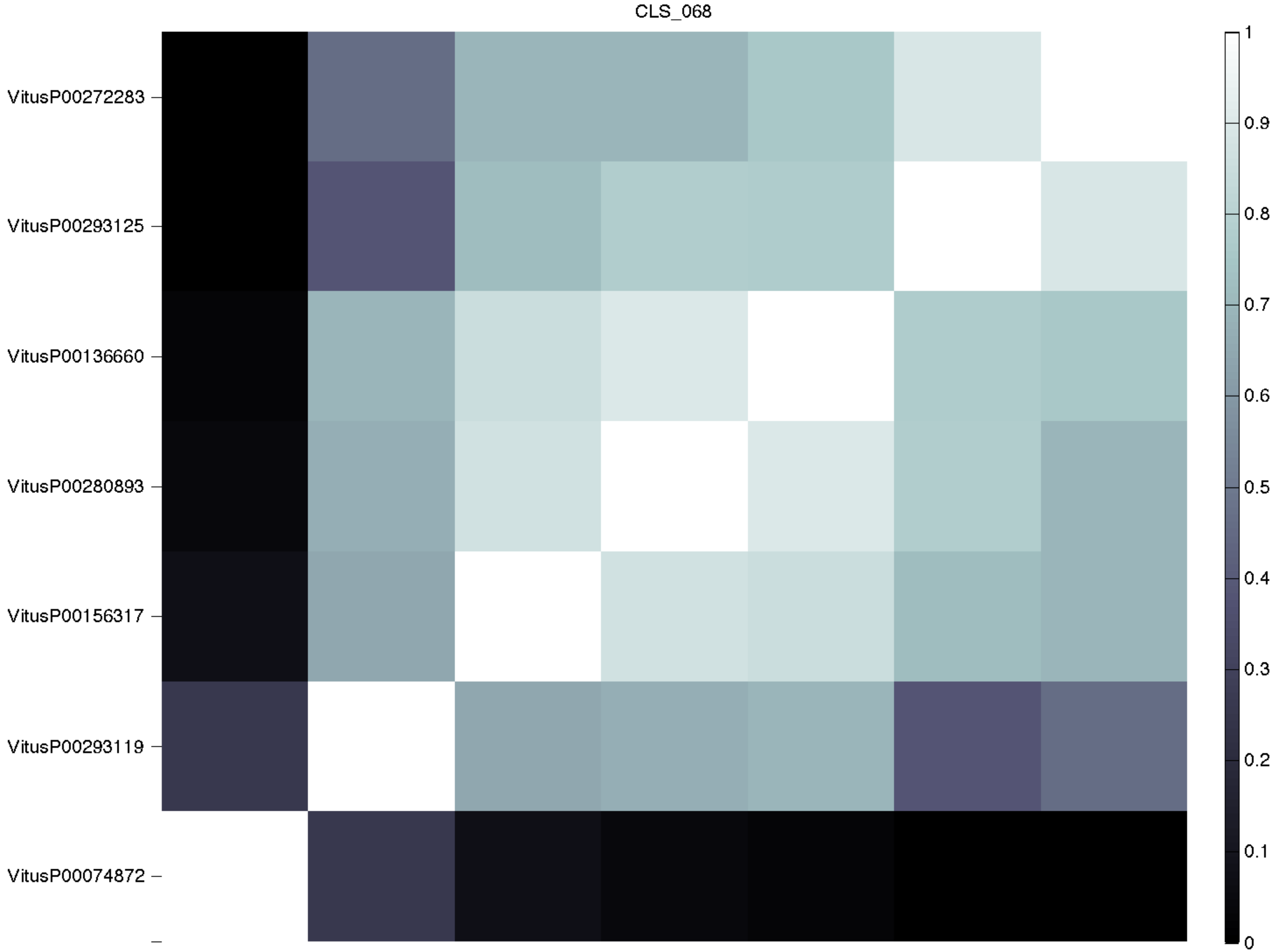
CLS_068
VitusP00272283
VitusP00293125
VitusP00136660
VitusP00280893
VitusP00156317
VitusP00293119
VitusP00074872
1
0.9
0.8
0.7
0.6
0.5
0.4
0.3
0.2
0.1
0

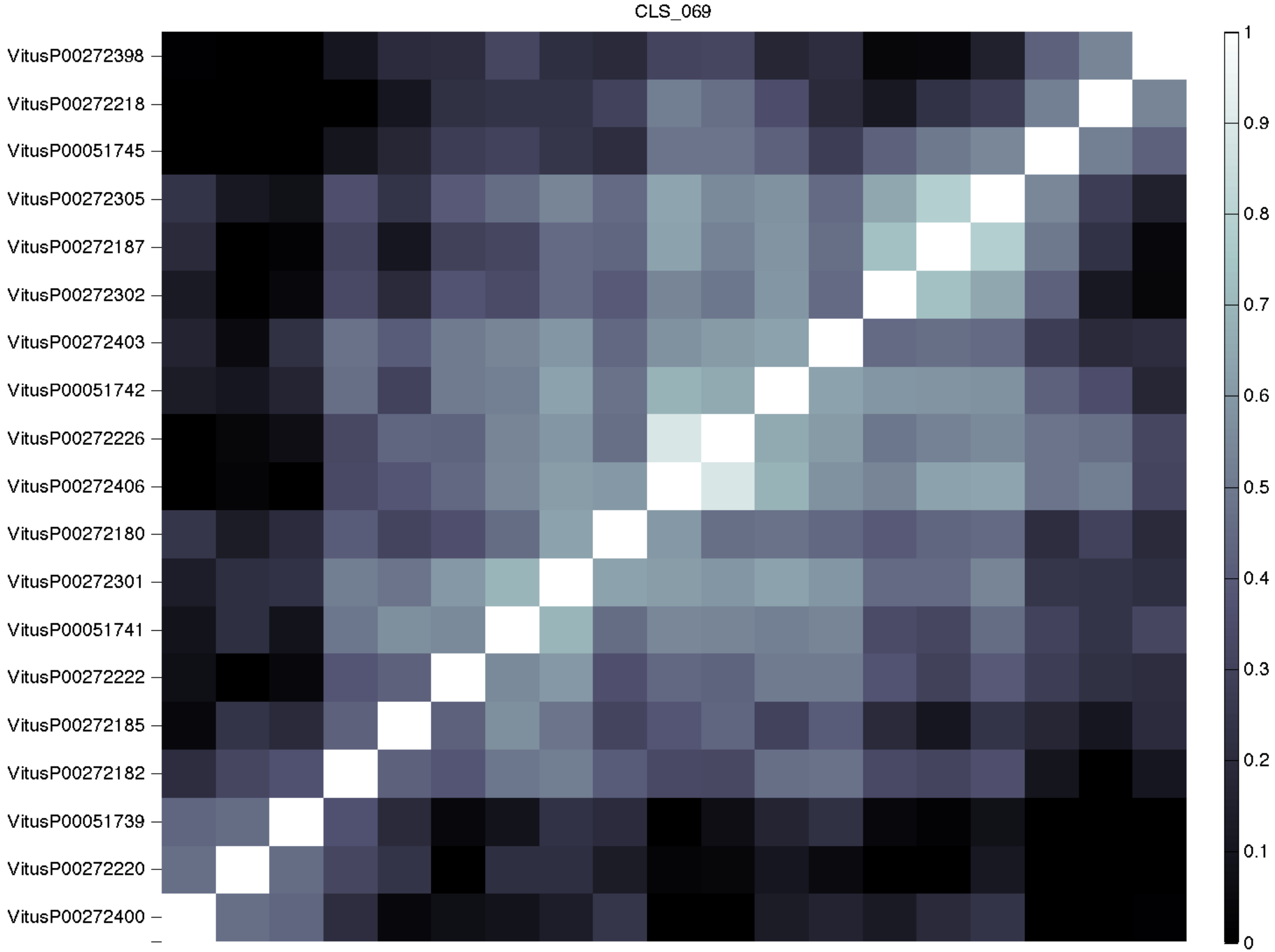
CLS_069
VitusP00272398
VitusP00272218
VitusP00051745
VitusP00272305
VitusP00272187
VitusP00272302
VitusP00272403
VitusP00051742
VitusP00272226
VitusP00272406
VitusP00272180
VitusP00272301
VitusP00051741
VitusP00272222
VitusP00272185
VitusP00272182
VitusP00051739
VitusP00272220
VitusP00272400
1
0.9
0.8
0.7
0.6
0.5
0.4
0.3
0.2
0.1
0

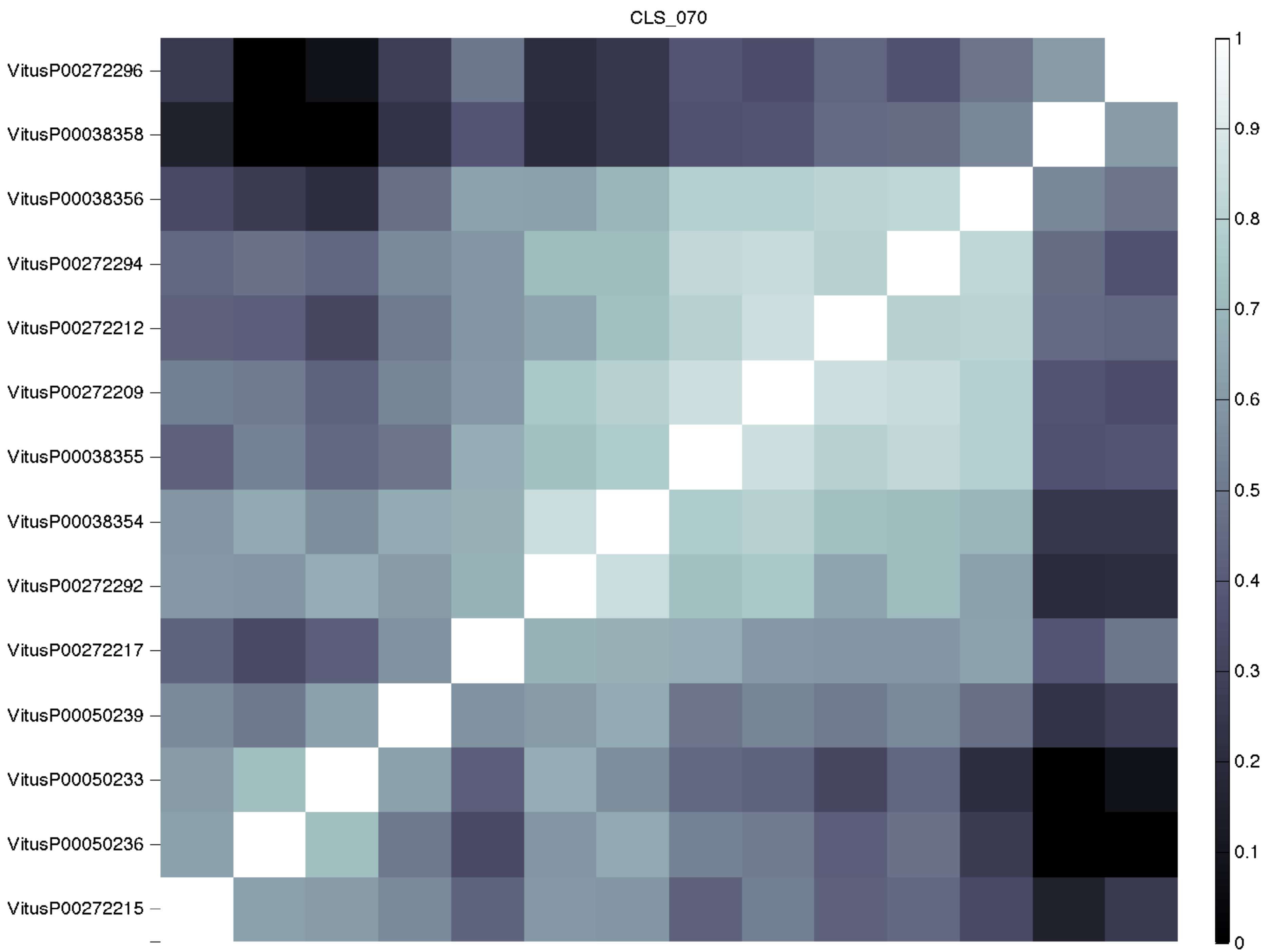

CLS_070
VitusP00272296
VitusP00038358
VitusP00038356
VitusP00272294
VitusP00272212
VitusP00272209
VitusP00038355
VitusP00038354
VitusP00272292
VitusP00272217
VitusP00050239
VitusP00050233
VitusP00050236
VitusP00272215
1
0.9
0.8
0.7
0.6
0.5
0.4
0.3
0.2
0.1
0

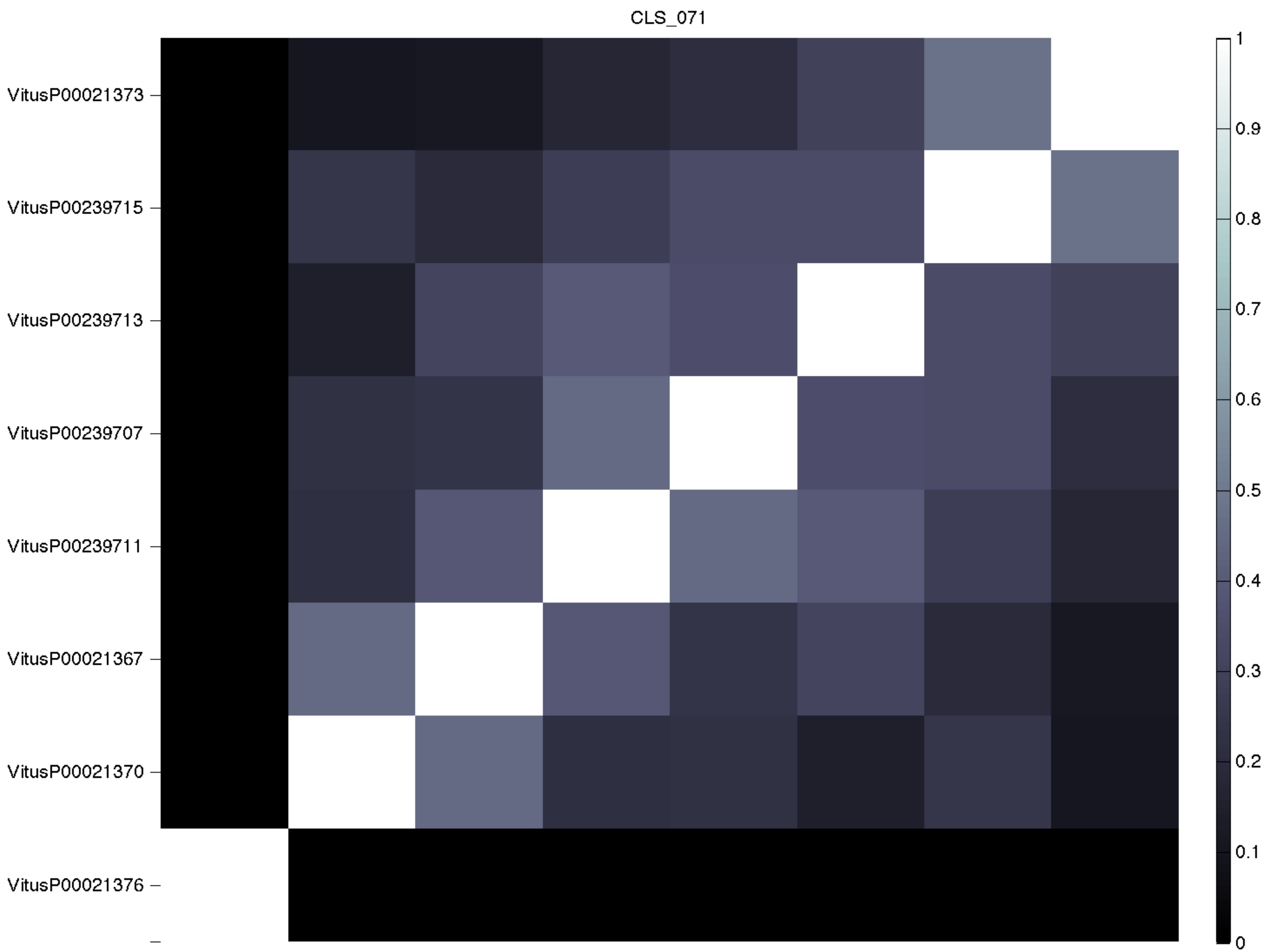

CLS_071
VitusP00021373
VitusP00239715
VitusP00239713
VitusP00239707
VitusP00239711
VitusP00021367
VitusP00021370
VitusP00021376
1
0.9
0.8
0.7
0.6
0.5
0.4
0.3
0.2
0.1
0

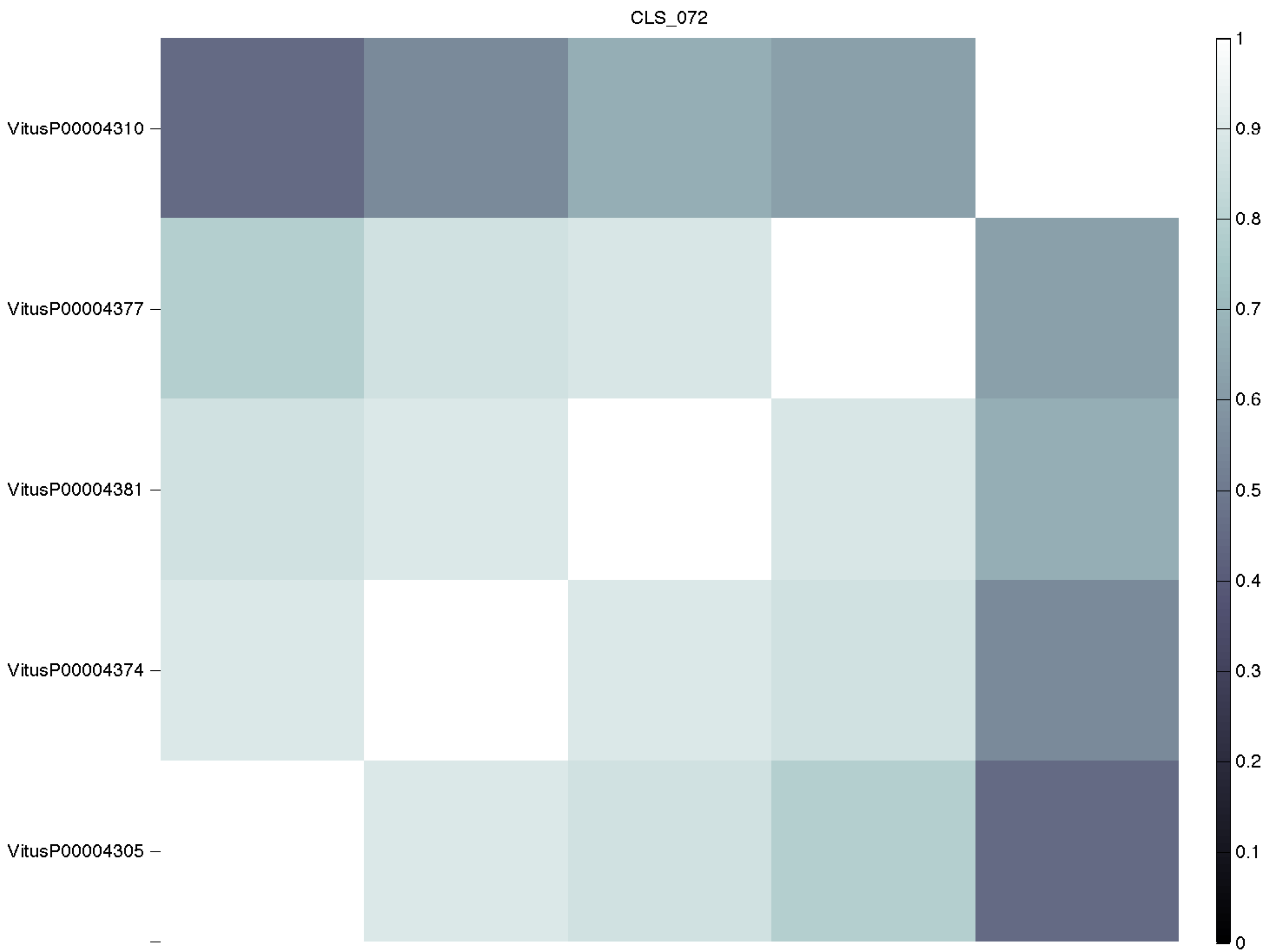
CLS_072
VitusP00004310
VitusP00004377
VitusP00004381
VitusP00004374
VitusP00004305
1
0.9
0.8
0.7
0.6
0.5
0.4
0.3
0.2
0.1
0

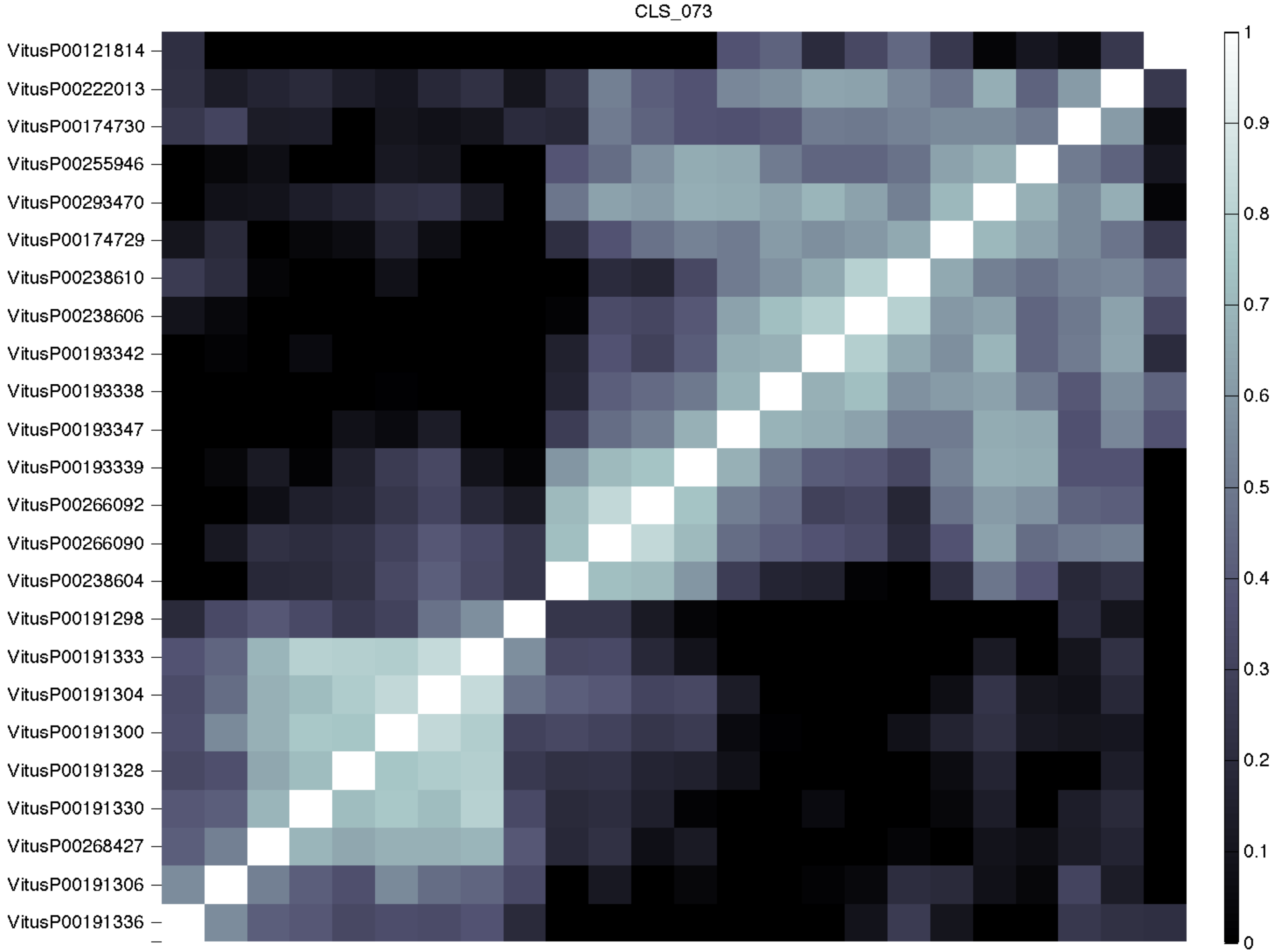
CLS_073
VitusP00121814
VitusP00222013
VitusP00174730
VitusP00255946
VitusP00293470
VitusP00174729
VitusP00238610
VitusP00238606
VitusP00193342
VitusP00193338
VitusP00193347
VitusP00193339
VitusP00266092
VitusP00266090
VitusP00238604
VitusP00191298
VitusP00191333
VitusP00191304
VitusP00191300
VitusP00191328
VitusP00191330
VitusP00268427
VitusP00191306
VitusP00191336
1
0.9
0.8
0.7
0.6
0.5
0.4
0.3
0.2
0.1
0

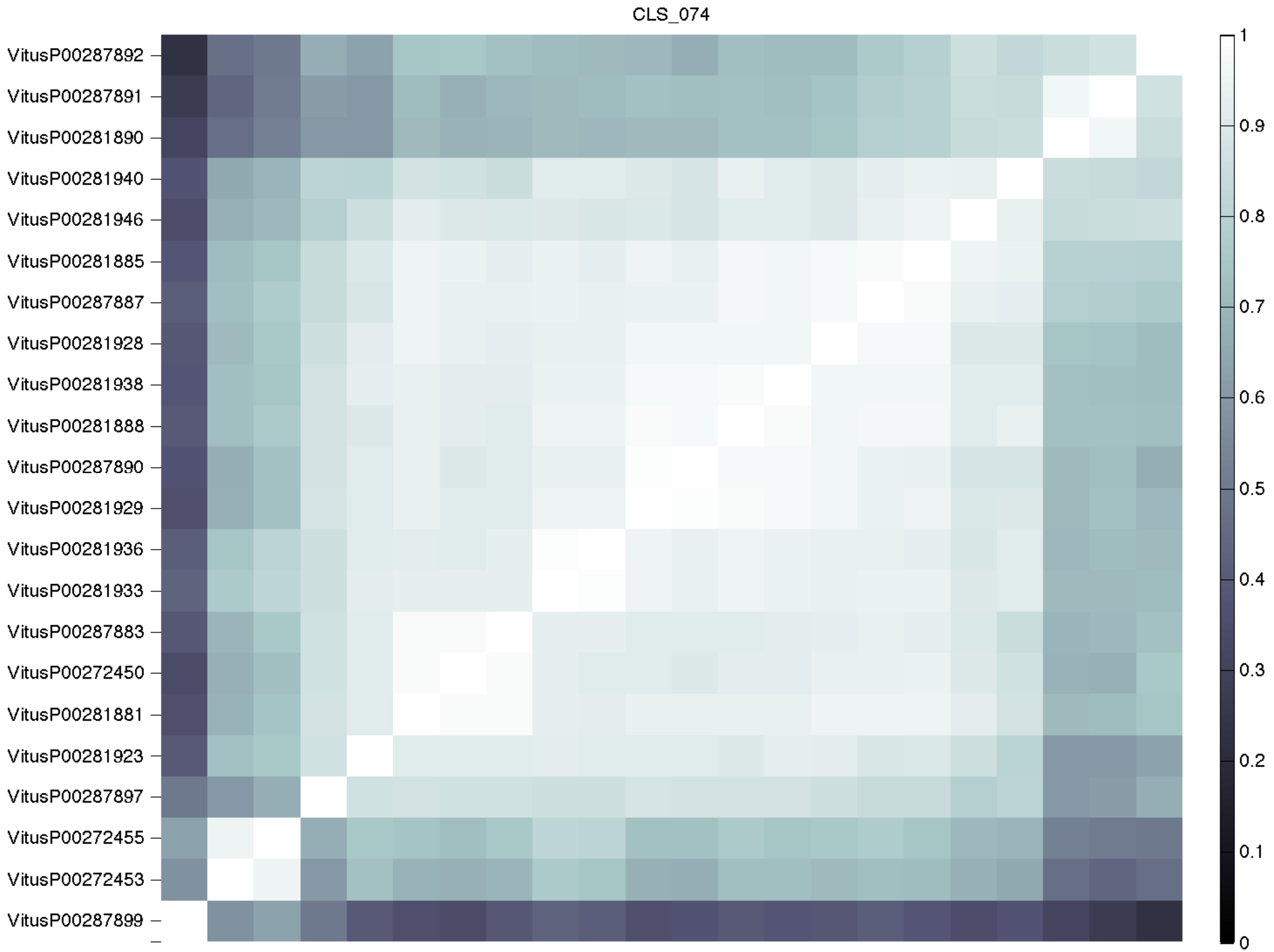
CLS_074
VitusP00287892
VitusP00287891
VitusP00281890
VitusP00281940
VitusP00281946
VitusP00281885
VitusP00287887
VitusP00281928
VitusP00281938
VitusP00281888
VitusP00287890
VitusP00281929
VitusP00281936
VitusP00281933
VitusP00287883
VitusP00272450
VitusP00281881
VitusP00281923
VitusP00287897
VitusP00272455
VitusP00272453
VitusP00287899
1
0.9
0.8
0.7
0.6
0.5
0.4
0.3
0.2
0.1
0

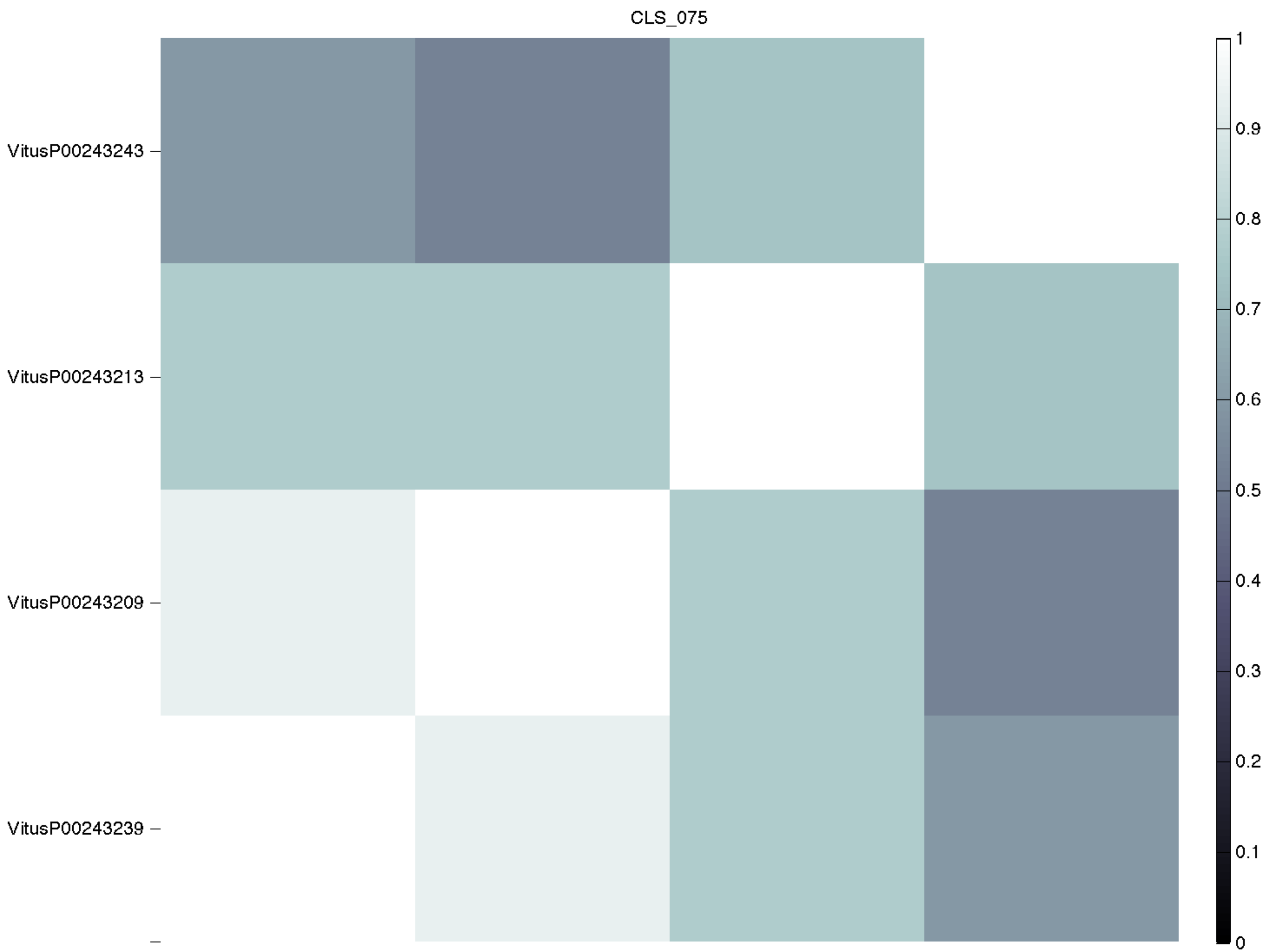
CLS_075
VitusP00243243
VitusP00243213
VitusP00243209
VitusP00243239
1
0.9
0.8
0.7
0.6
0.5
0.4
0.3
0.2
0.1
0

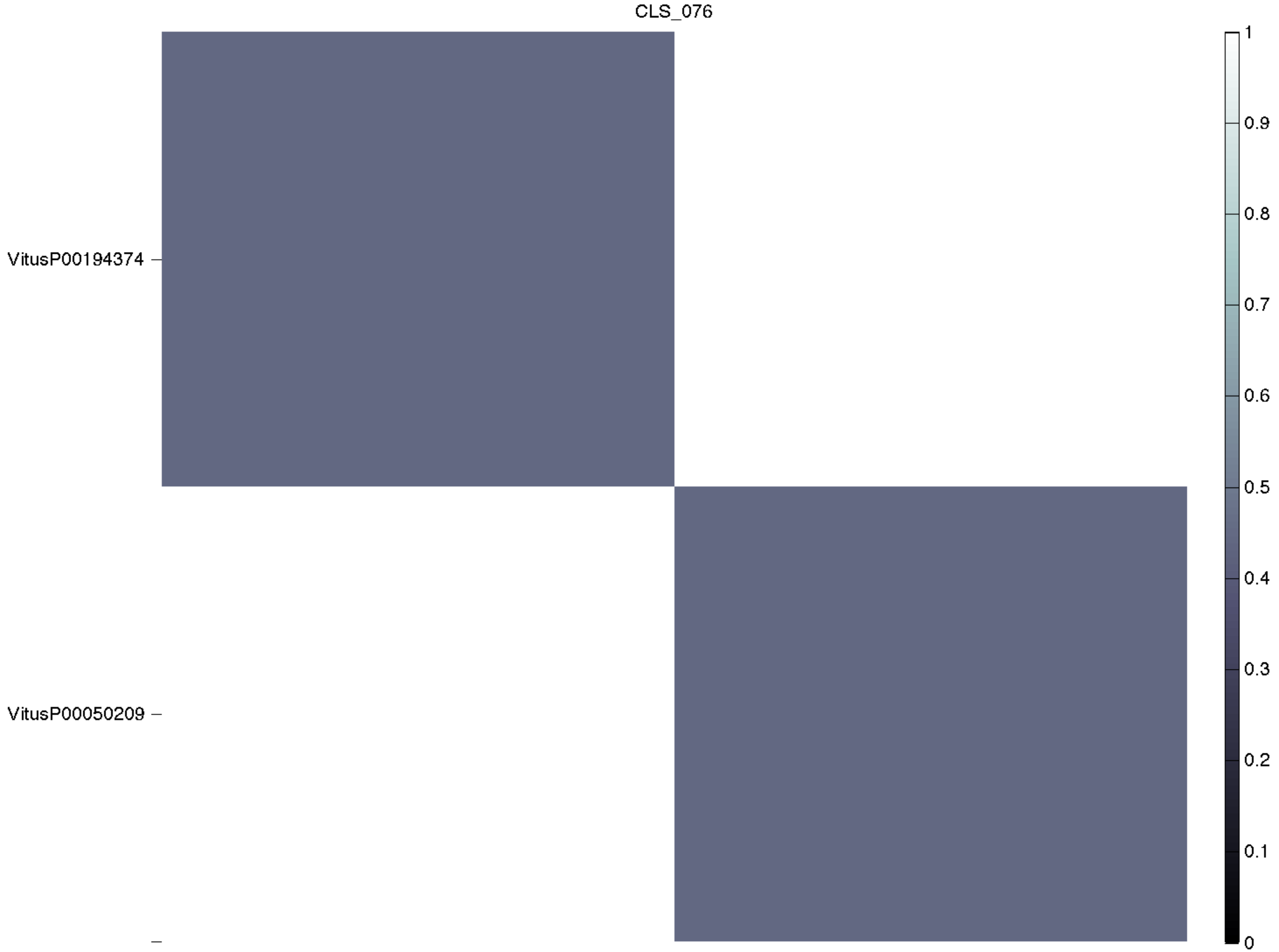
CLS_076
VitusP00194374
VitusP00050209
1
0.9
0.8
0.7
0.6
0.5
0.4
0.3
0.2
0.1
0

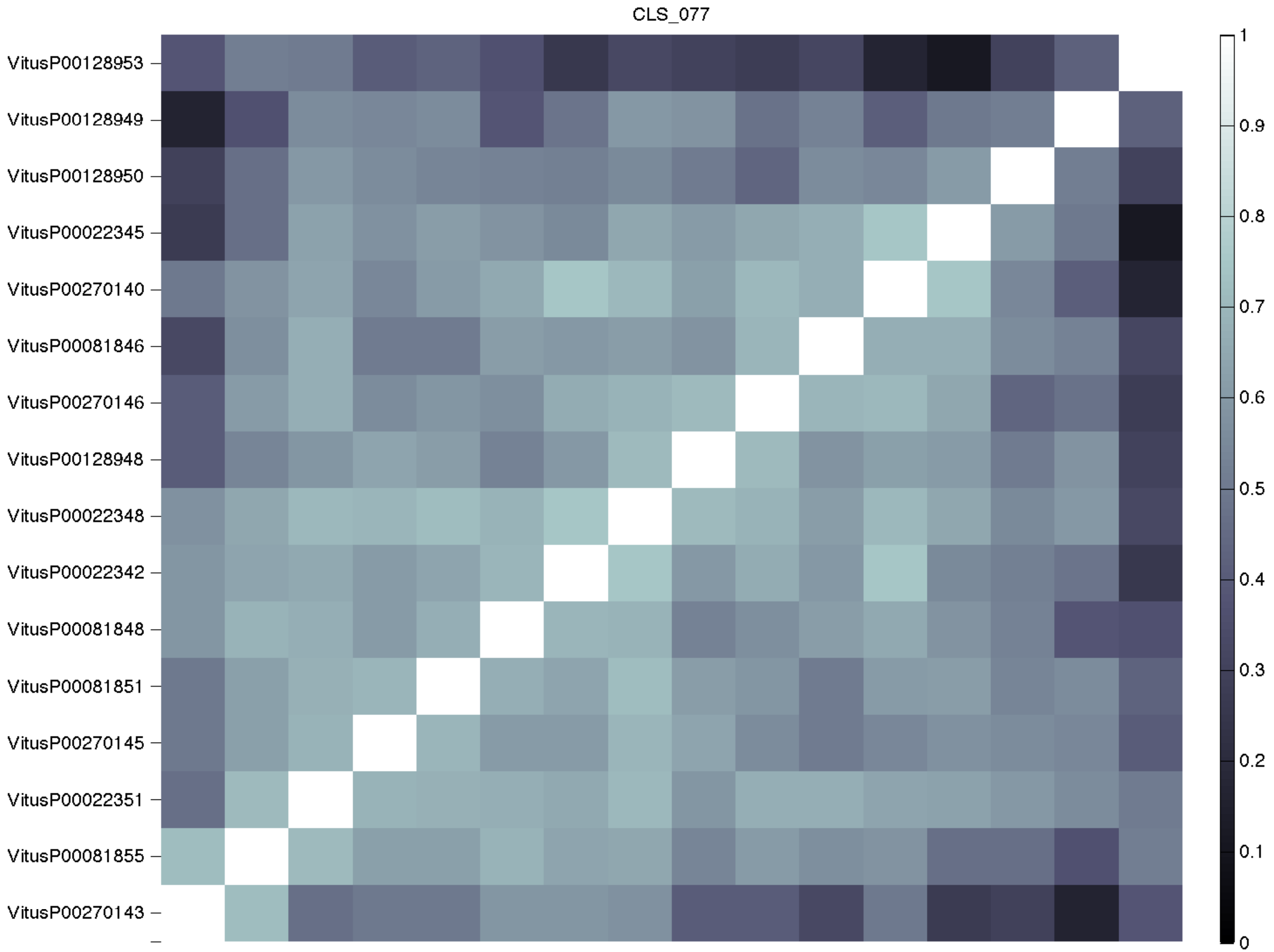
CLS_077
VitusP00128953
VitusP00128949
VitusP00128950
VitusP00022345
VitusP00270140
VitusP00081846
VitusP00270146
VitusP00128948
VitusP00022348
VitusP00022342
VitusP00081848
VitusP00081851
VitusP00270145
VitusP00022351
VitusP00081855
VitusP00270143
1
0.9
0.8
0.7
0.6
0.5
0.4
0.3
0.2
0.1
0

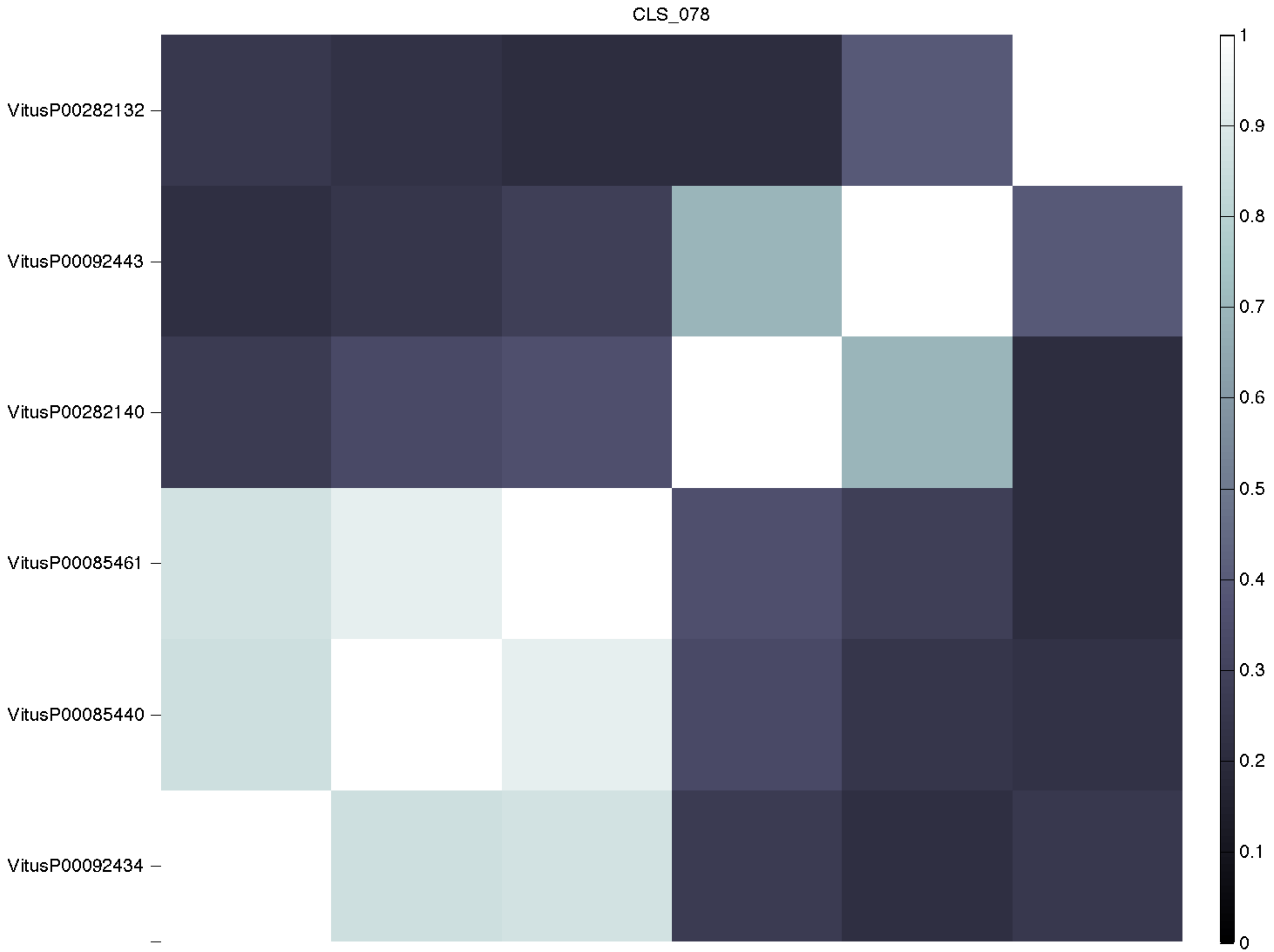
CLS_078
VitusP00282132
VitusP00092443
VitusP00282140
VitusP00085461
VitusP00085440
VitusP00092434
1
0.9
0.8
0.7
0.6
0.5
0.4
0.3
0.2
0.1
0

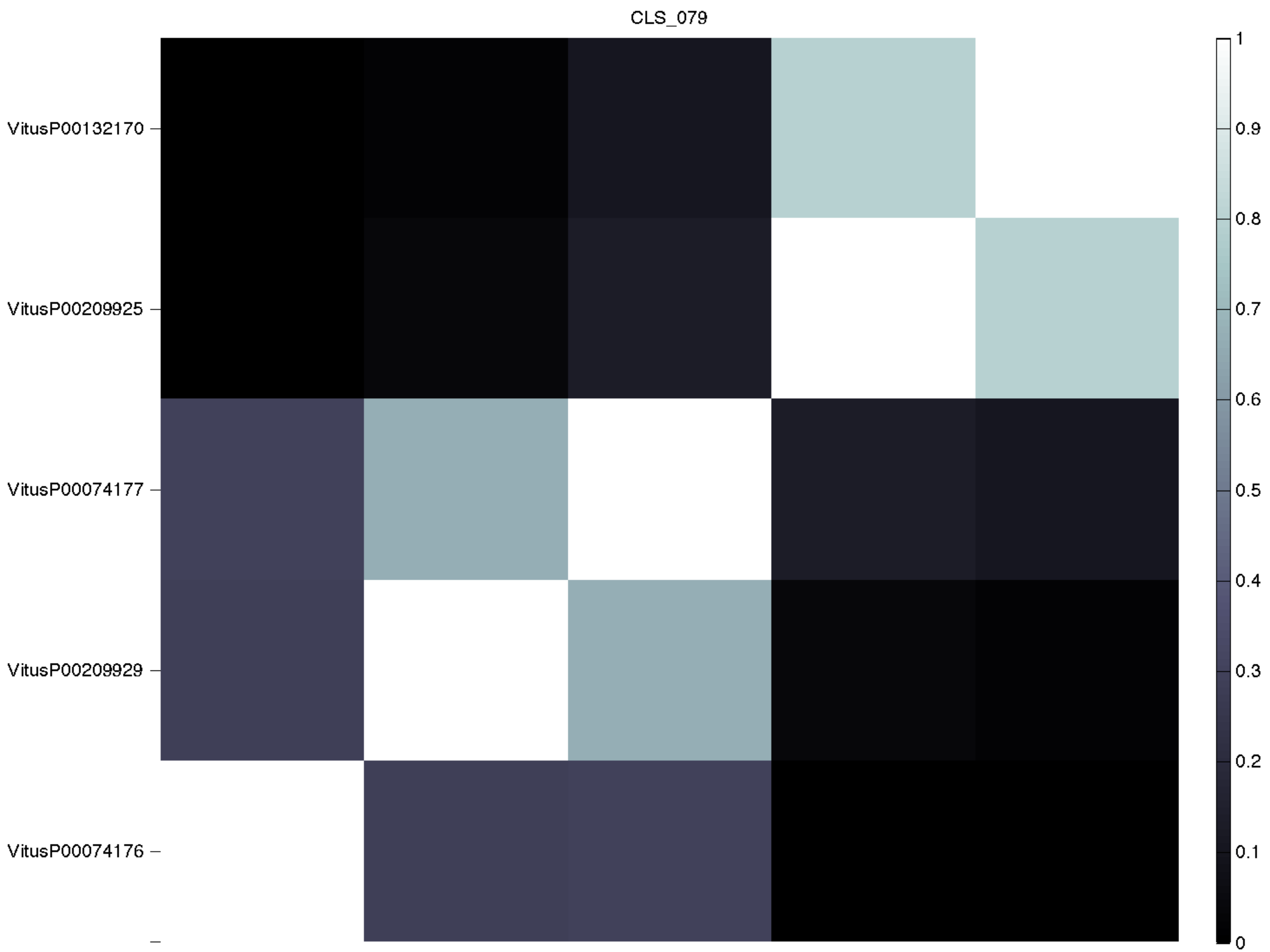
CLS_079
VitusP00132170
VitusP00209925
VitusP00074177
VitusP00209929
VitusP00074176
1
0.9
0.8
0.7
0.6
0.5
0.4
0.3
0.2
0.1
0

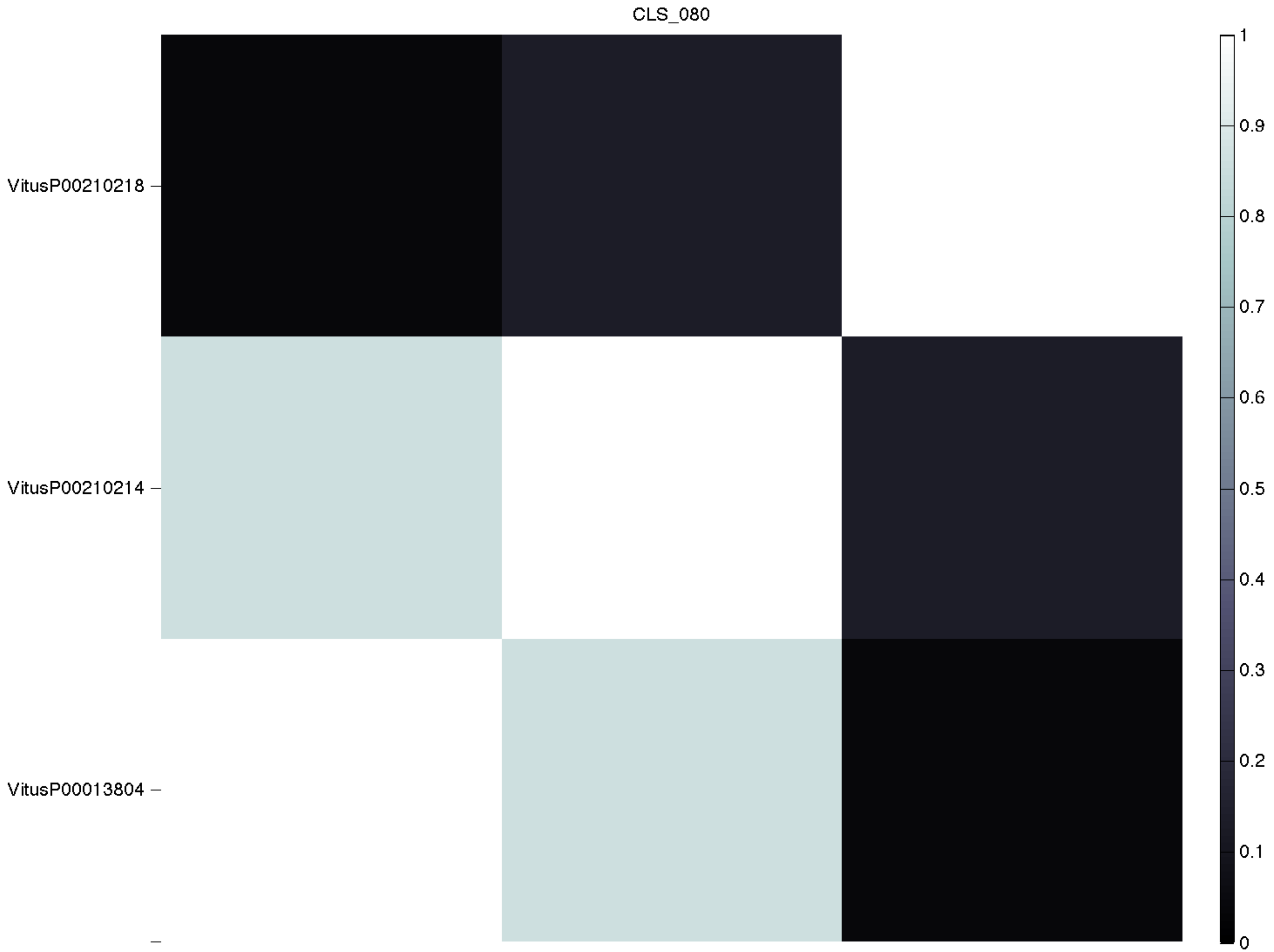

CLS_080
VitusP00210218
VitusP00210214
VitusP00013804
1
0.9
0.8
0.7
0.6
0.5
0.4
0.3
0.2
0.1
0

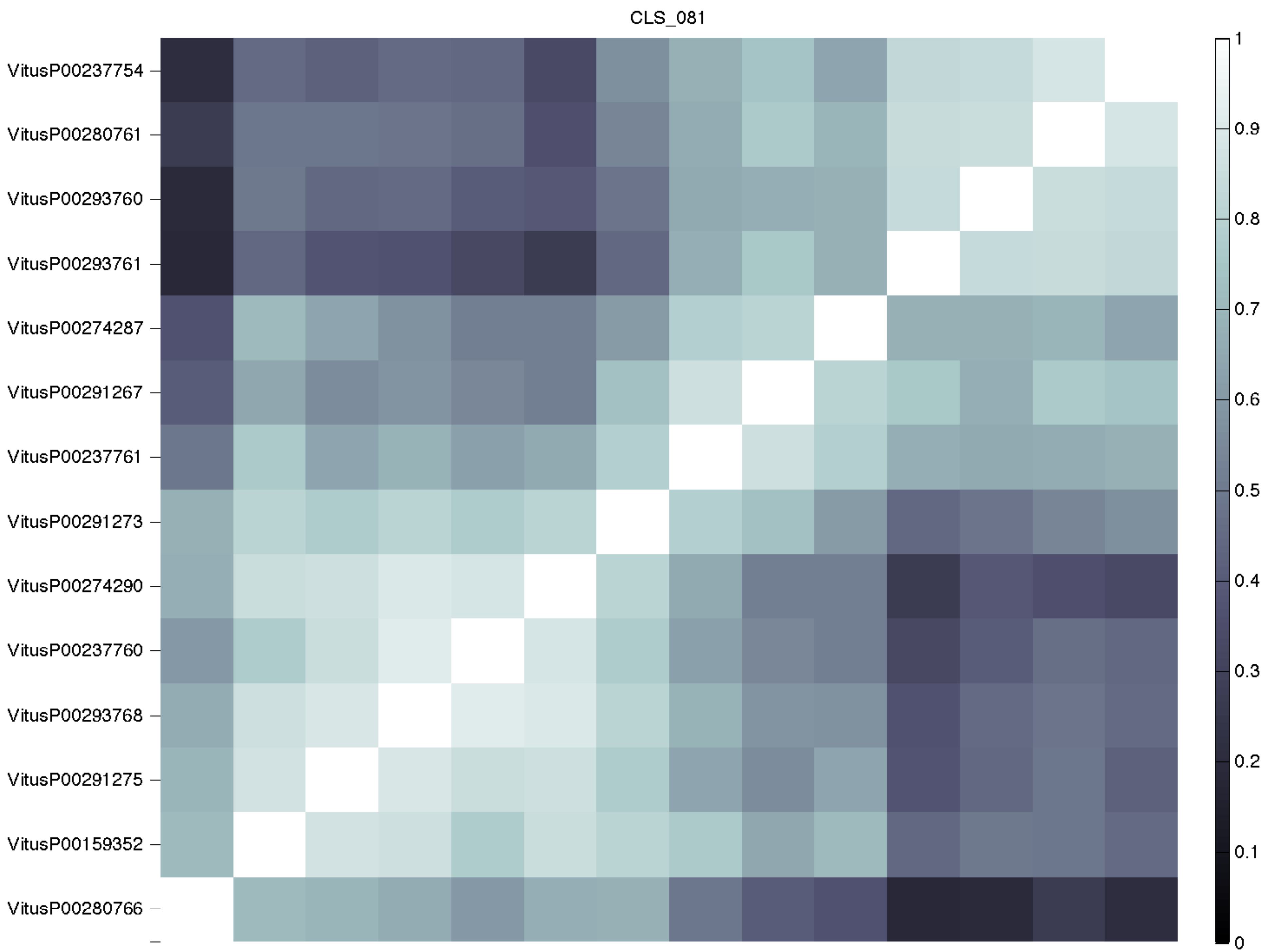
CLS_081
VitusP00237754
VitusP00280761
VitusP00293760
VitusP00293761
VitusP00274287
VitusP00291267
VitusP00237761
VitusP00291273
VitusP00274290
VitusP00237760
VitusP00293768
VitusP00291275
VitusP00159352
VitusP00280766
1
0.9
0.8
0.7
0.6
0.5
0.4
0.3
0.2
0.1
0

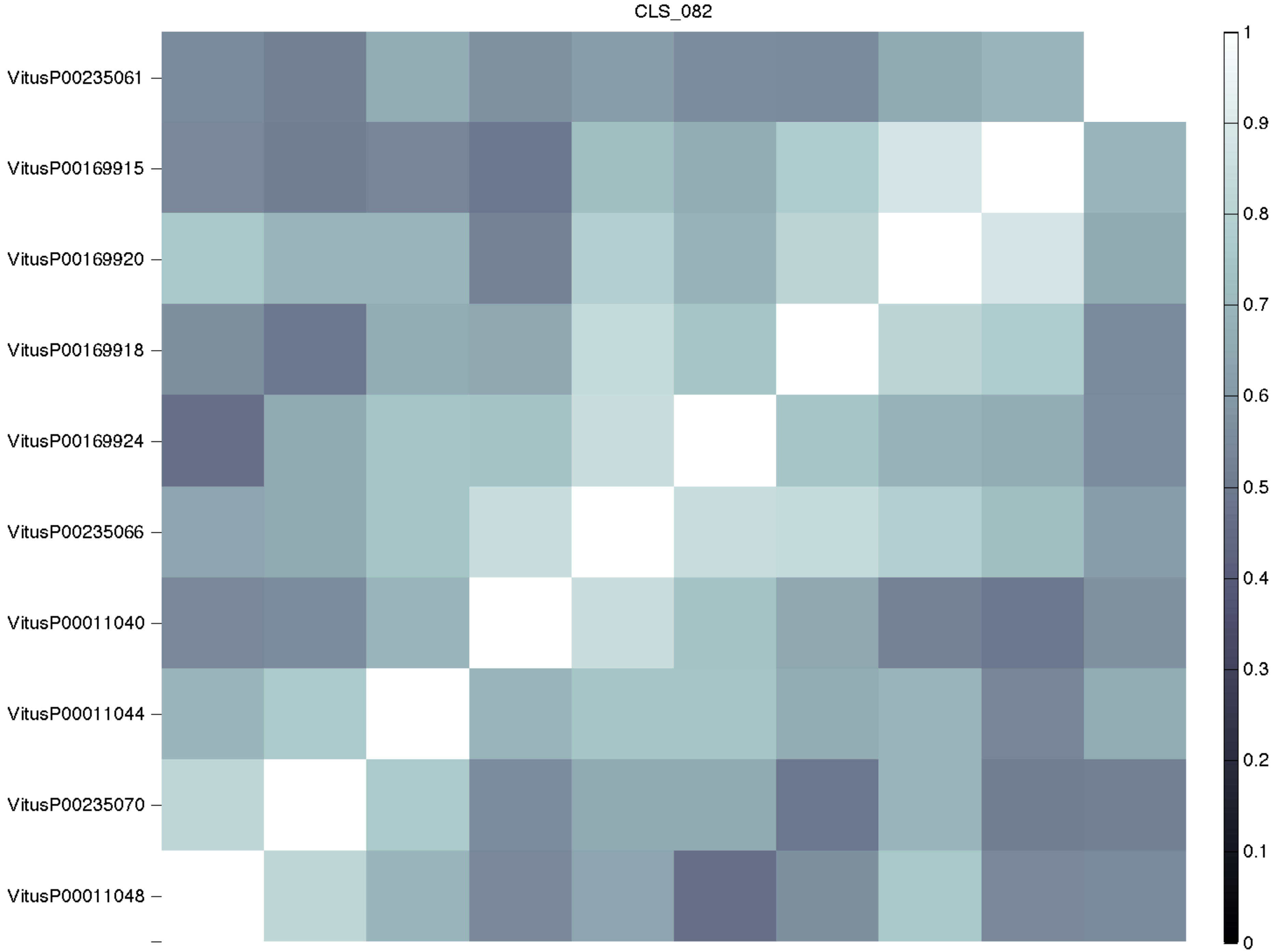
CLS_082
VitusP00235061
VitusP00169915
VitusP00169920
VitusP00169918
VitusP00169924
VitusP00235066
VitusP00011040
VitusP00011044
VitusP00235070
VitusP00011048
1
0.9
0.8
0.7
0.6
0.5
0.4
0.3
0.2
0.1
0

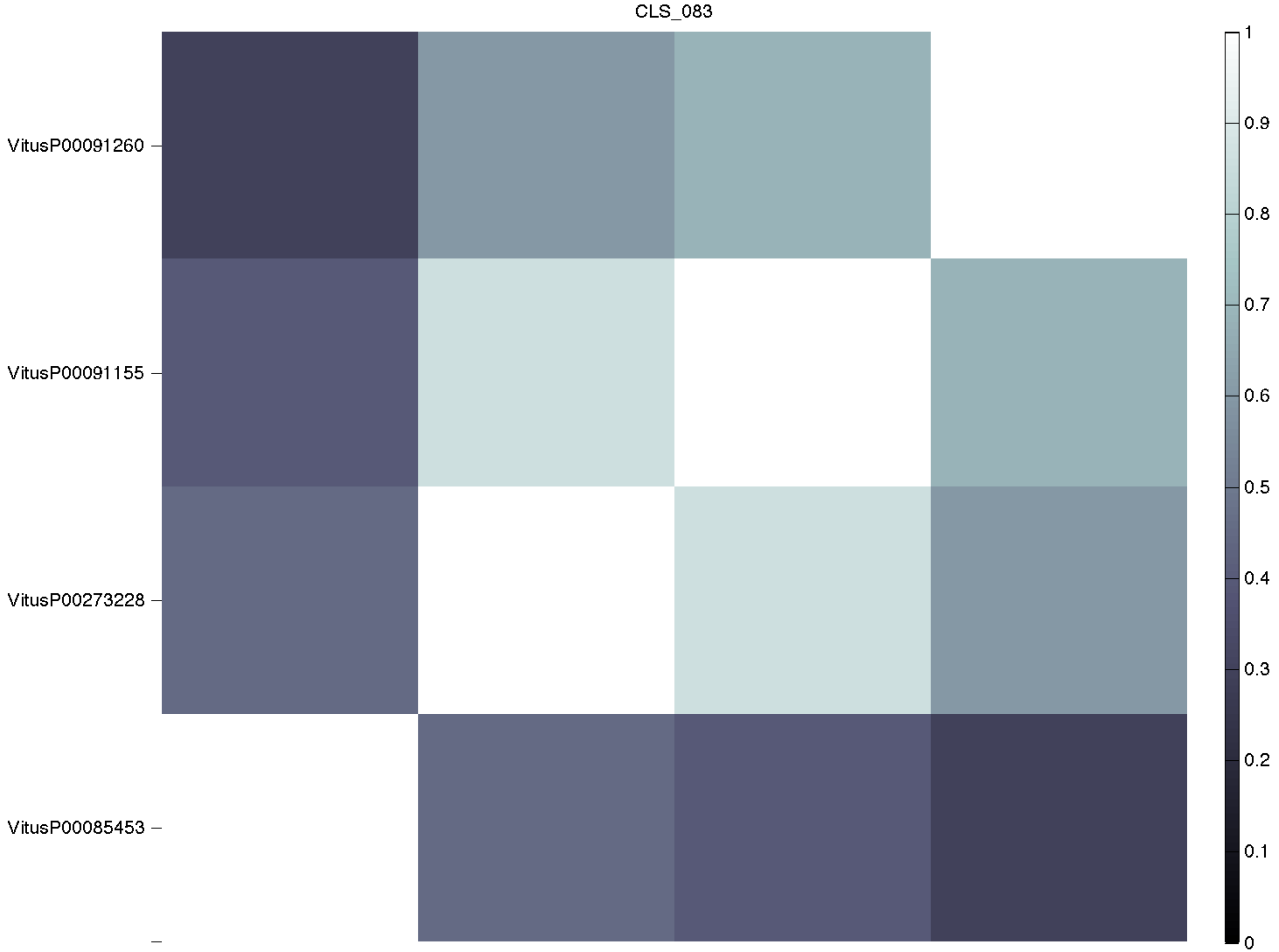
CLS_083
VitusP00091260
VitusP00091155
VitusP00273228
VitusP00085453
1
0.9
0.8
0.7
0.6
0.5
0.4
0.3
0.2
0.1
0

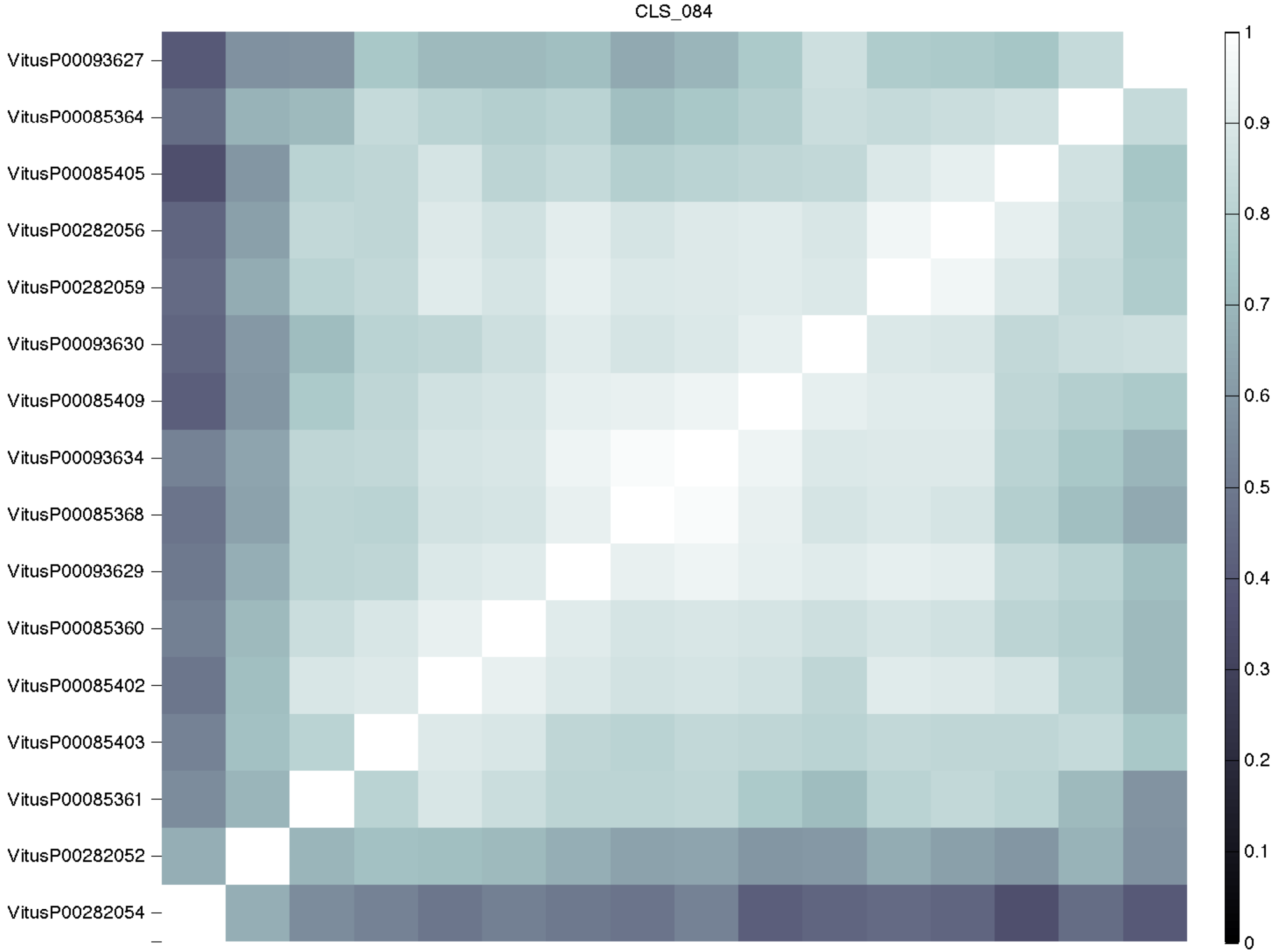

CLS_084
VitusP00093627
VitusP00085364
VitusP00085405
VitusP00282056
VitusP00282059
VitusP00093630
VitusP00085409
VitusP00093634
VitusP00085368
VitusP00093629
VitusP00085360
VitusP00085402
VitusP00085403
VitusP00085361
VitusP00282052
VitusP00282054
1
0.9
0.8
0.7
0.6
0.5
0.4
0.3
0.2
0.1
0

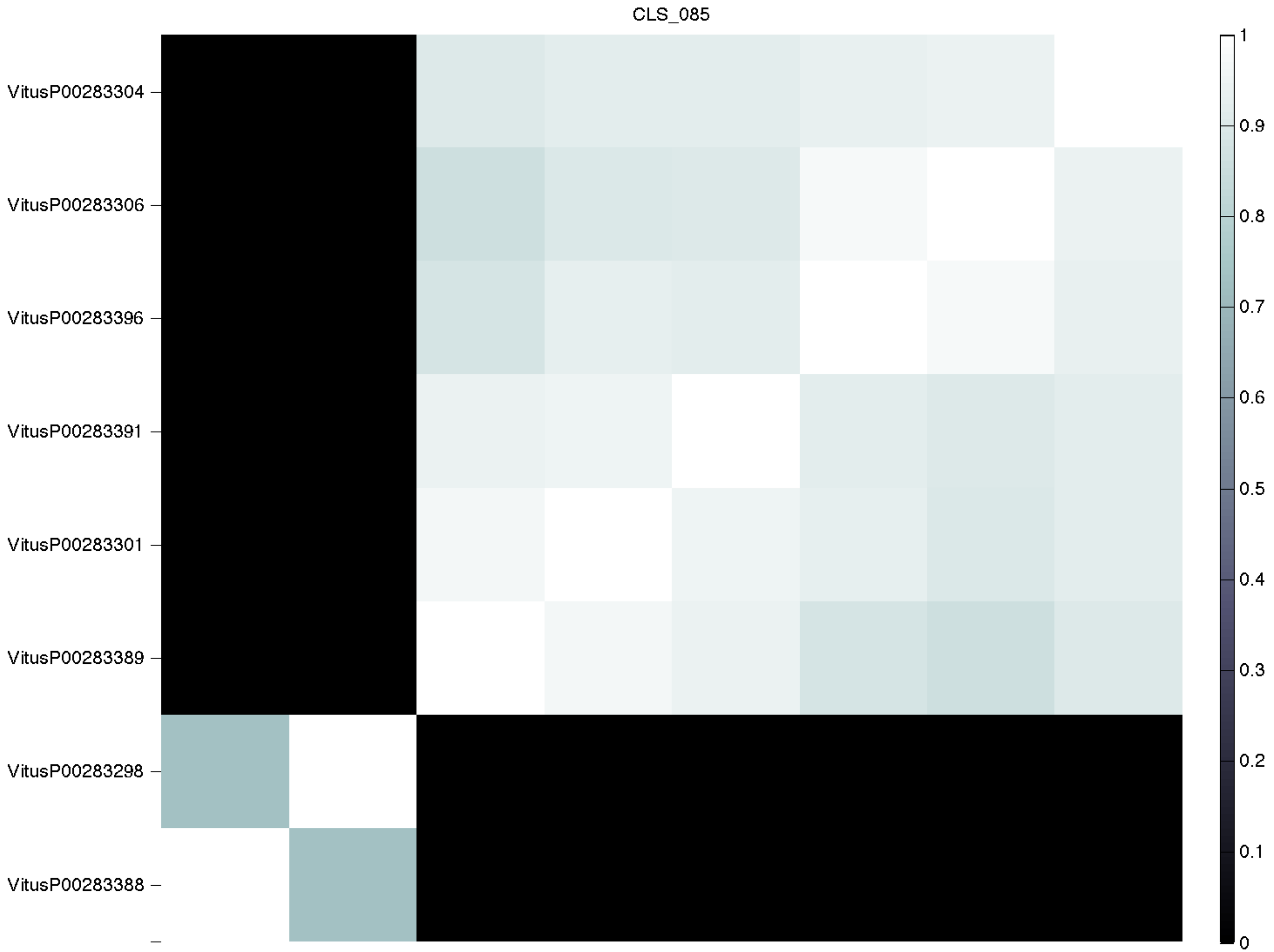

CLS_085
VitusP00283304
VitusP00283306
VitusP00283396
VitusP00283391
VitusP00283301
VitusP00283389
VitusP00283298
VitusP00283388
1
0.9
0.8
0.7
0.6
0.5
0.4
0.3
0.2
0.1
0

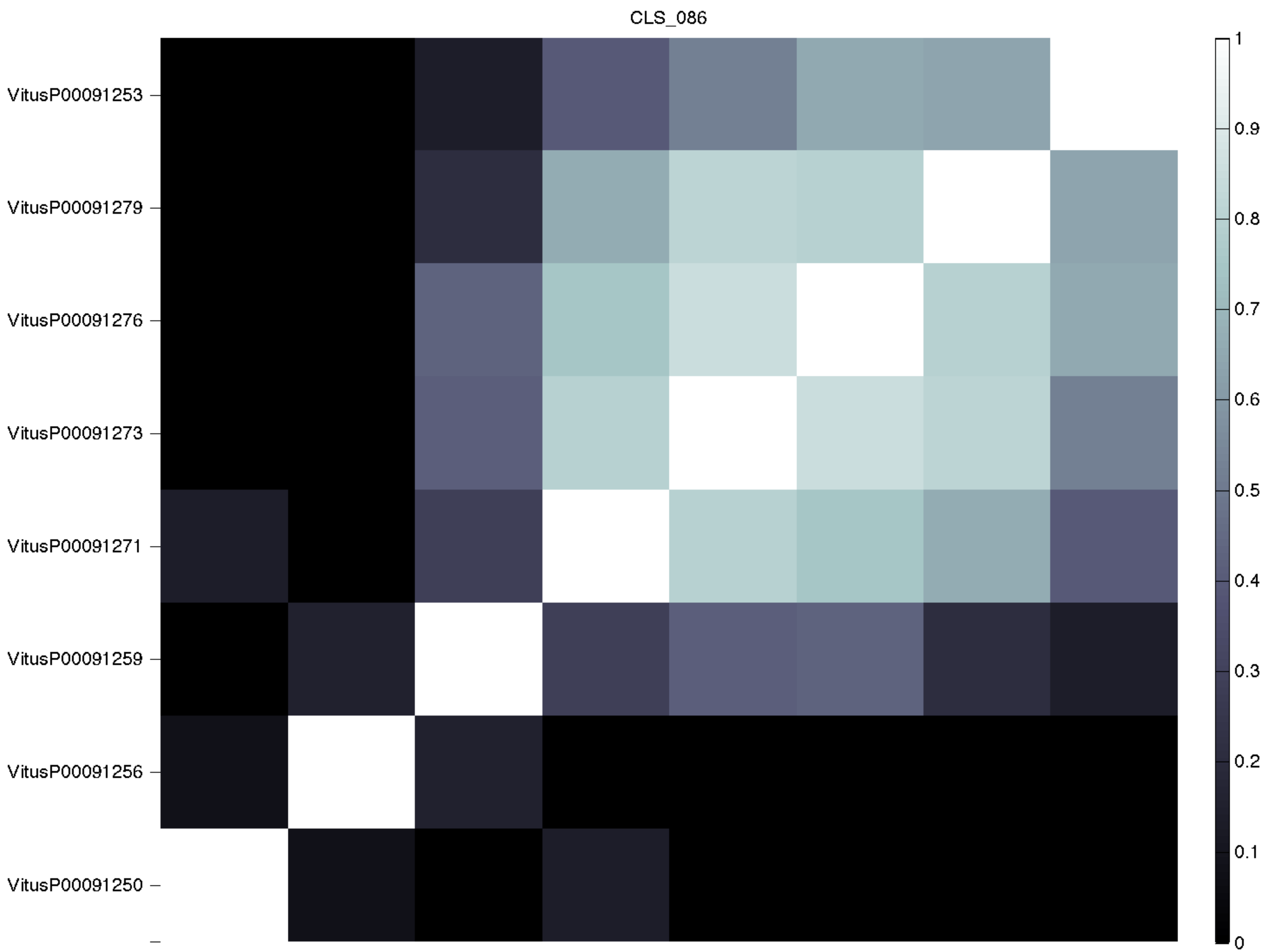
CLS_086
VitusP00091253
VitusP00091279
VitusP00091276
VitusP00091273
VitusP00091271
VitusP00091259
VitusP00091256
VitusP00091250
1
0.9
0.8
0.7
0.6
0.5
0.4
0.3
0.2
0.1
0

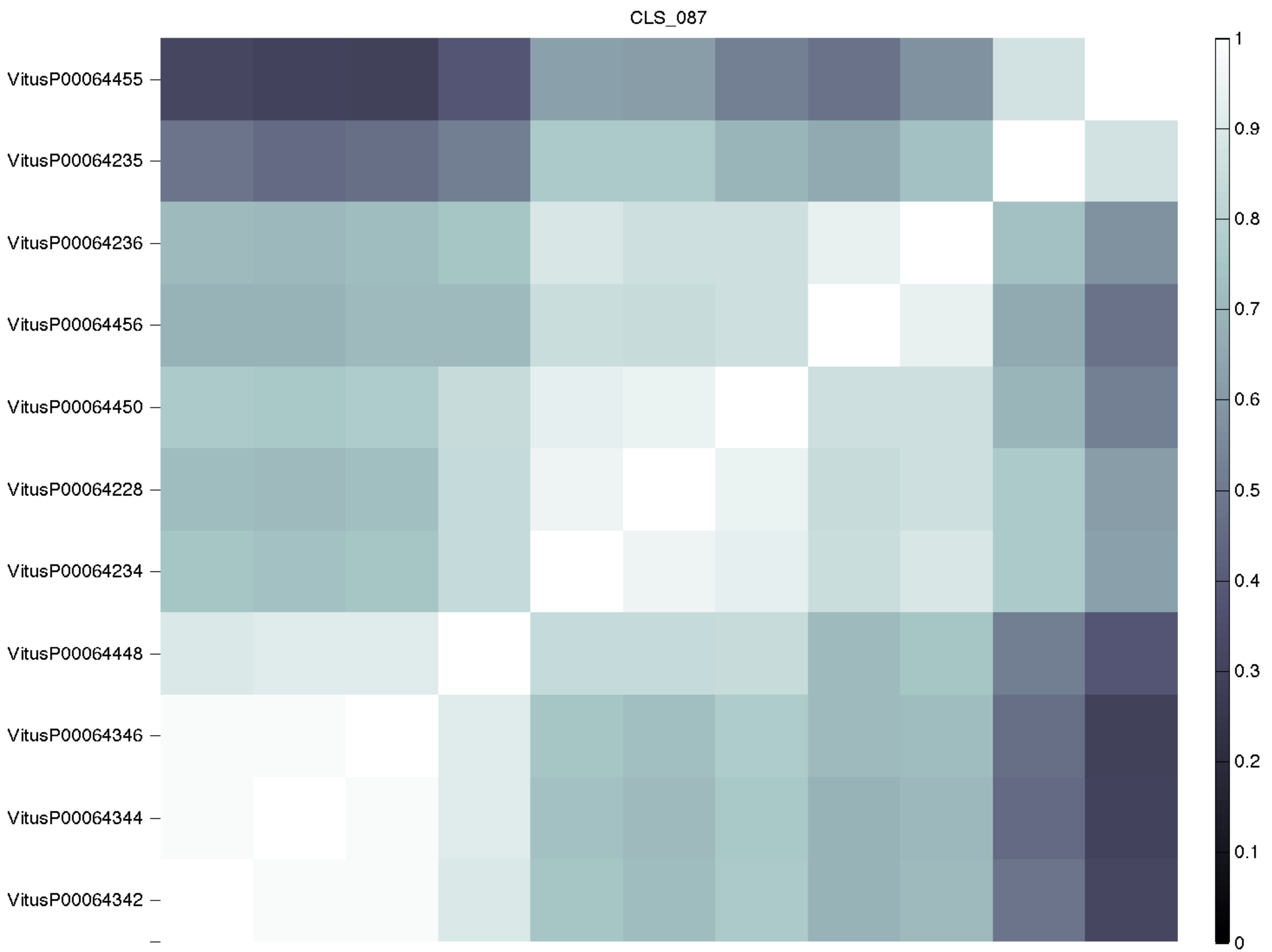
CLS_087
VitusP00064455
VitusP00064235
VitusP00064236
VitusP00064456
VitusP00064450
VitusP00064228
VitusP00064234
VitusP00064448
VitusP00064346
VitusP00064344
VitusP00064342
1
0.9
0.8
0.7
0.6
0.5
0.4
0.3
0.2
0.1
0

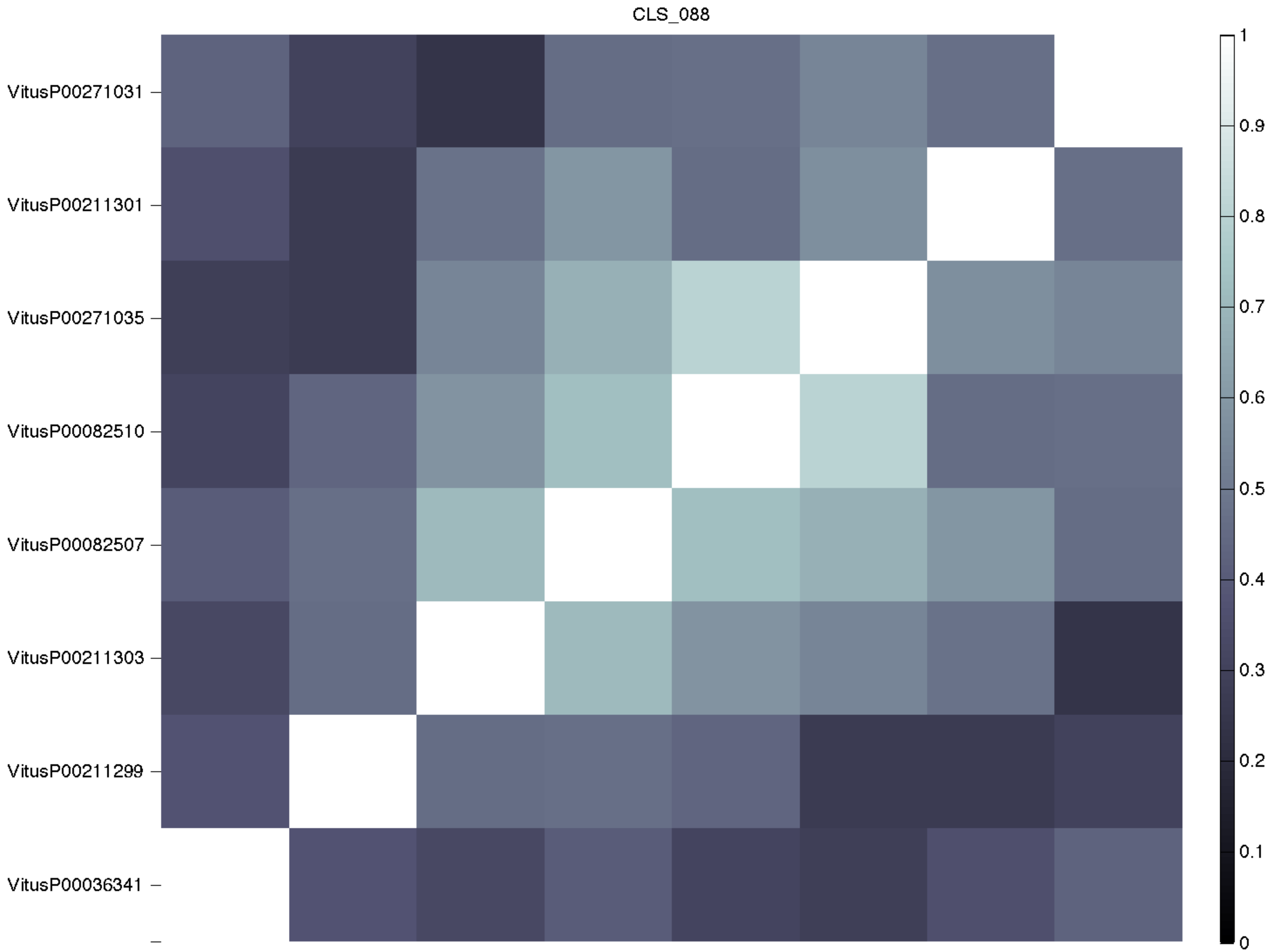
CLS_088
VitusP00271031
VitusP00211301
VitusP00271035
VitusP00082510
VitusP00082507
VitusP00211303
VitusP00211299
VitusP00036341
1
0.9
0.8
0.7
0.6
0.5
0.4
0.3
0.2
0.1
0

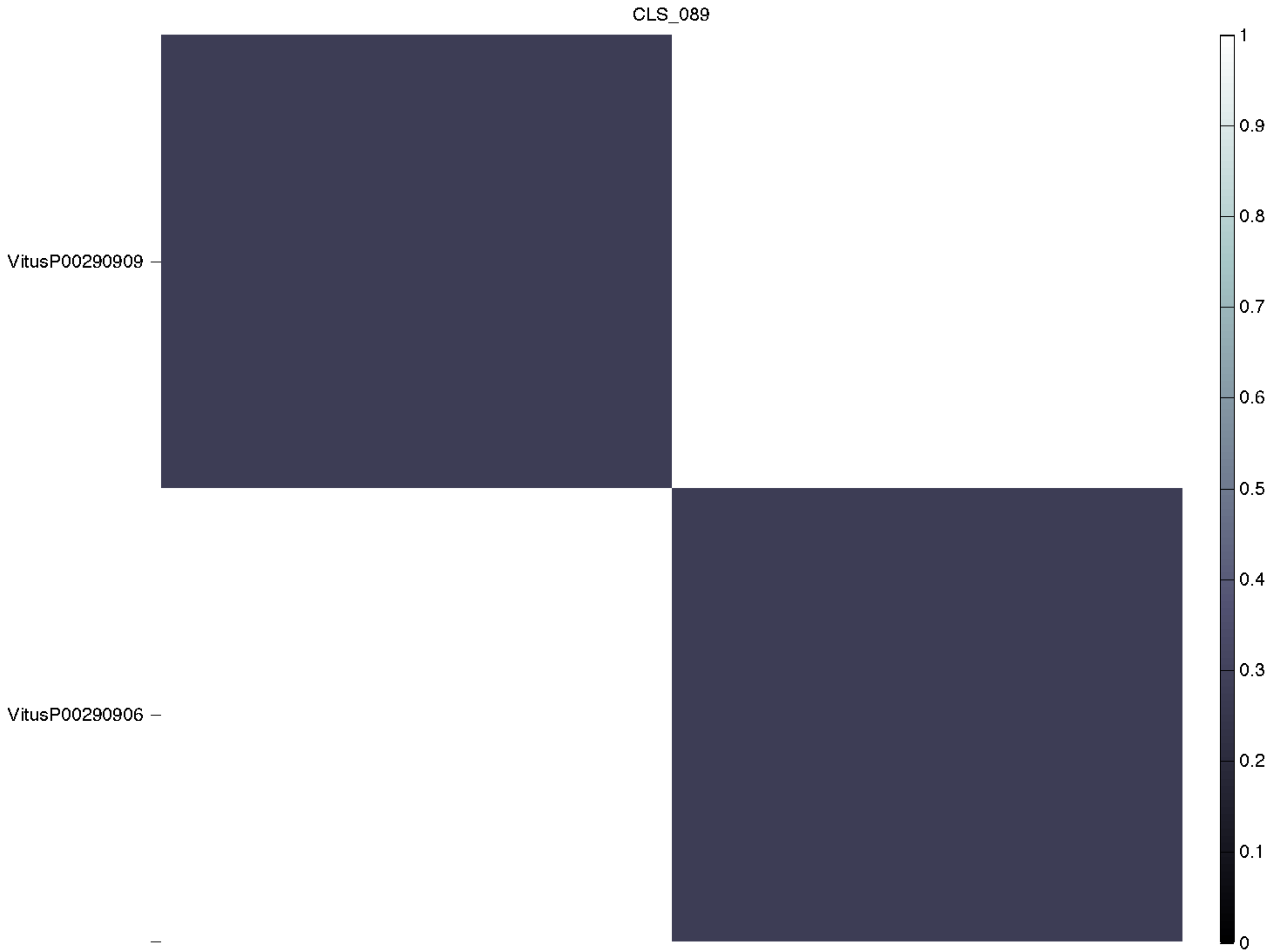
CLS_089
VitusP00290909
VitusP00290906
1
0.9
0.8
0.7
0.6
0.5
0.4
0.3
0.2
0.1
0

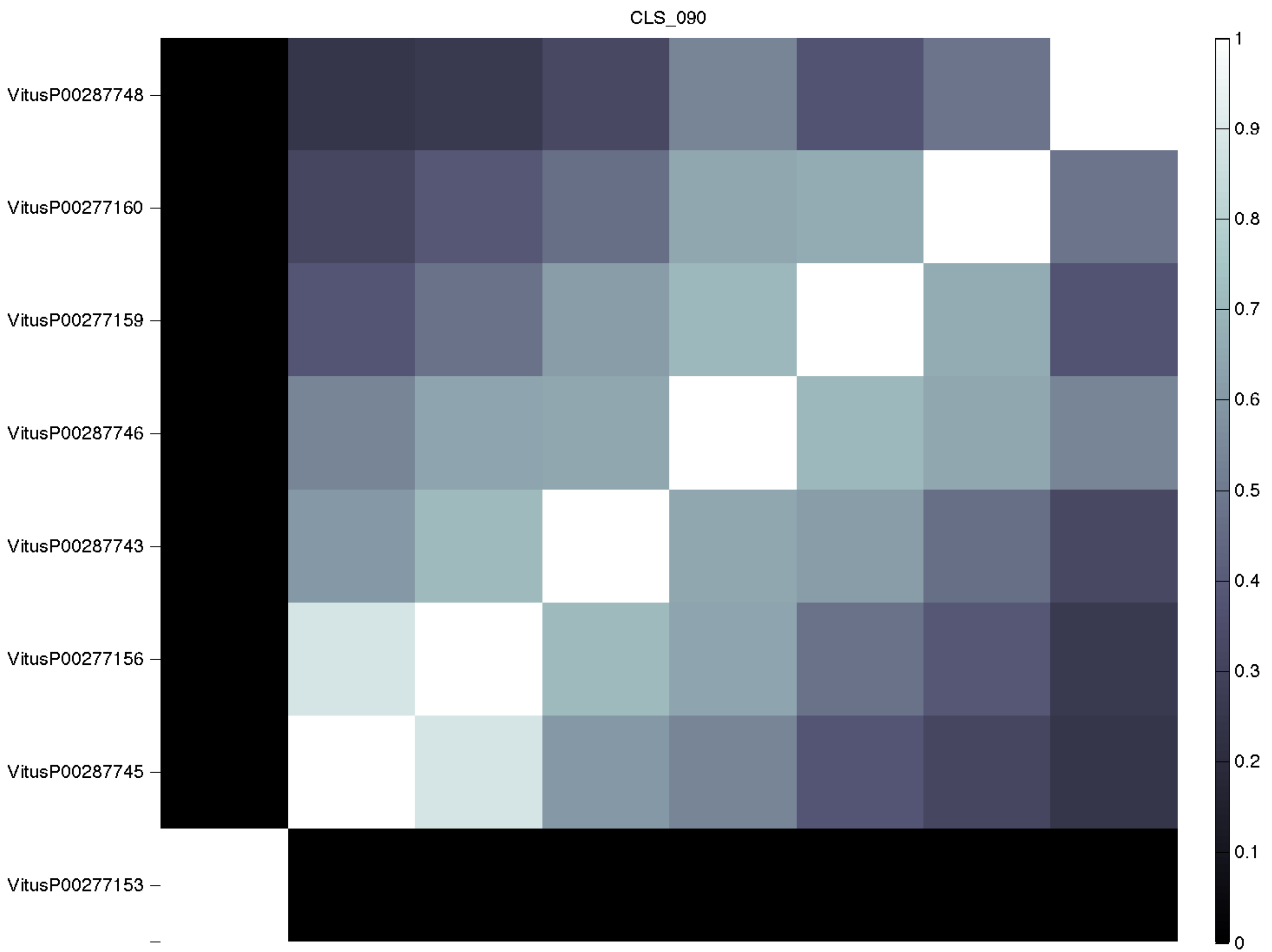

CLS_090
VitusP00287748
VitusP00277160
VitusP00277159
VitusP00287746
VitusP00287743
VitusP00277156
VitusP00287745
VitusP00277153
1
0.9
0.8
0.7
0.6
0.5
0.4
0.3
0.2
0.1
0

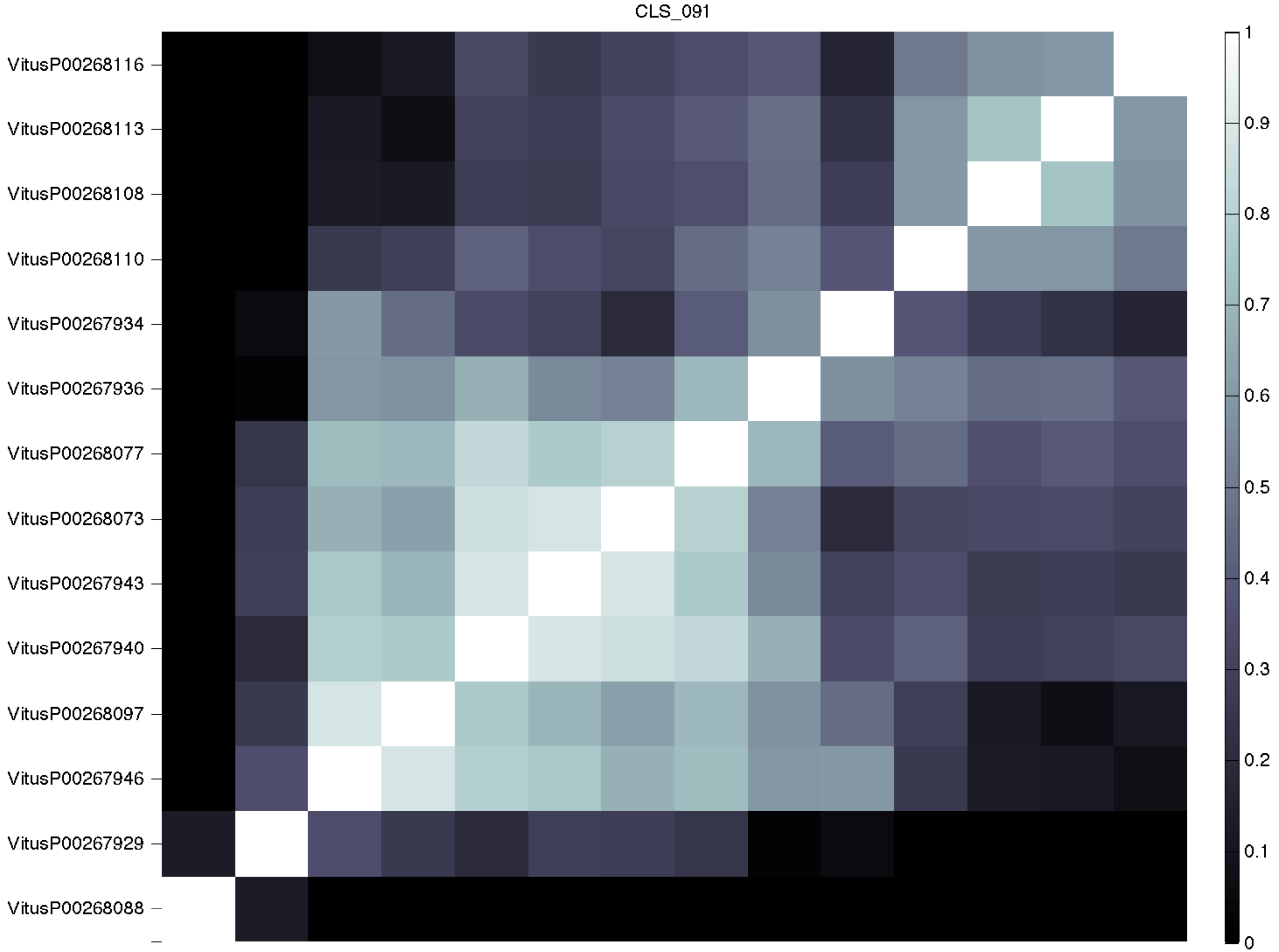

CLS_091
VitusP00268116
VitusP00268113
VitusP00268108
VitusP00268110
VitusP00267934
VitusP00267936
VitusP00268077
VitusP00268073
VitusP00267943
VitusP00267940
VitusP00268097
VitusP00267946
VitusP00267929
VitusP00268088
1
0.9
0.8
0.7
0.6
0.5
0.4
0.3
0.2
0.1
0

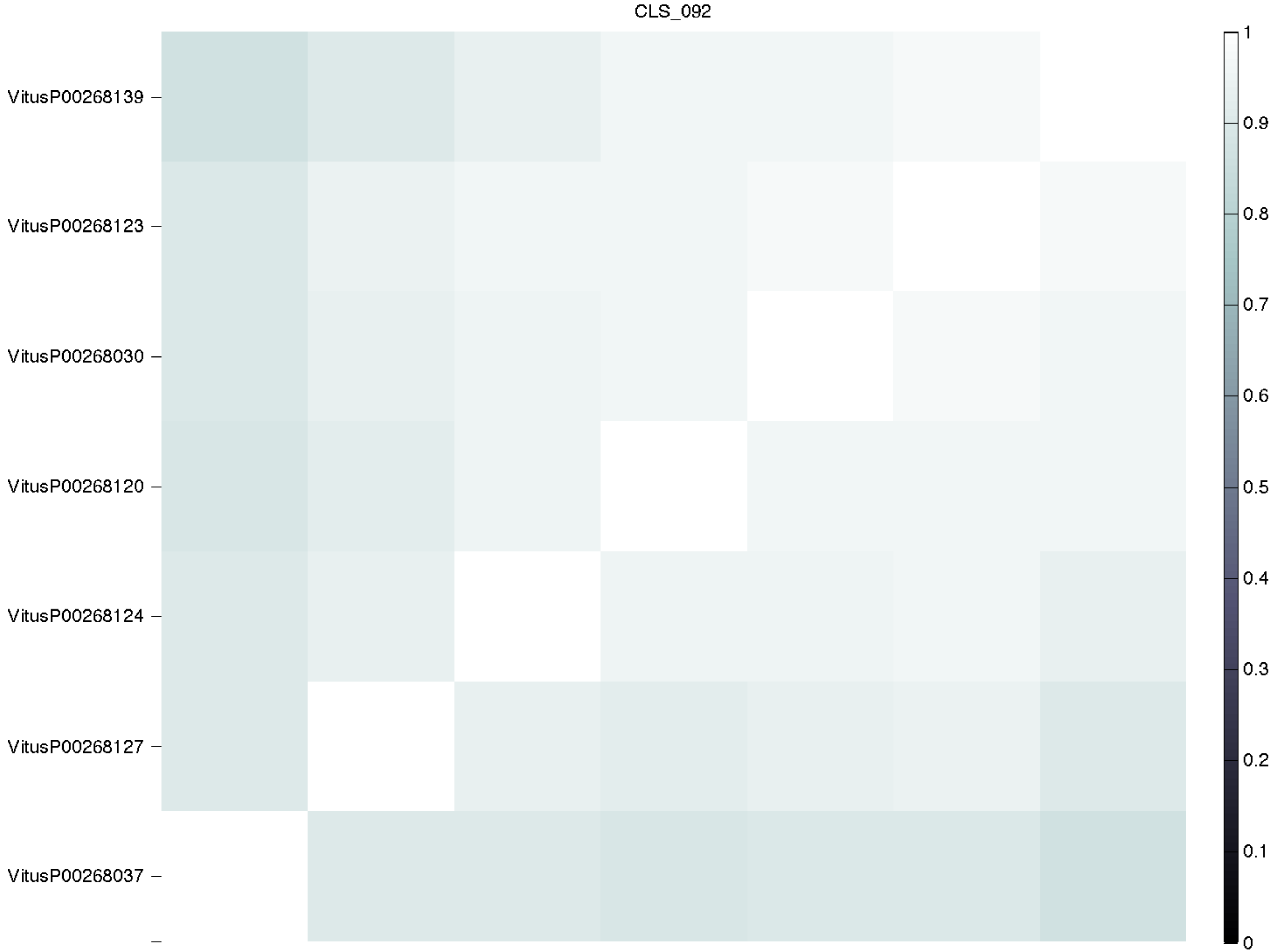
CLS_092
VitusP00268139
VitusP00268123
VitusP00268030
VitusP00268120
VitusP00268124
VitusP00268127
VitusP00268037
1
0.9
0.8
0.7
0.6
0.5
0.4
0.3
0.2
0.1
0

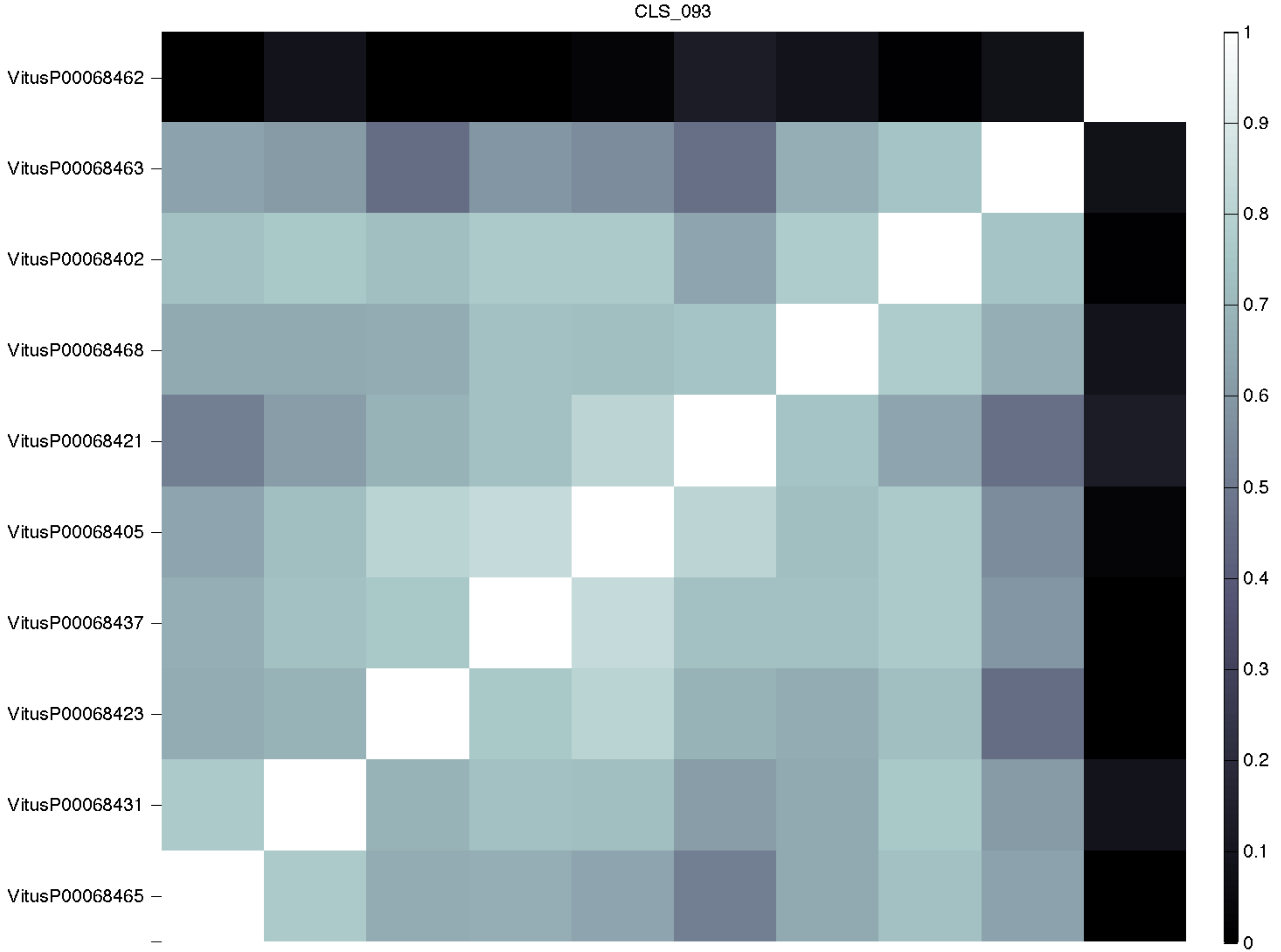
CLS_093
VitusP00068462
VitusP00068463
VitusP00068402
VitusP00068468
VitusP00068421
VitusP00068405
VitusP00068437
VitusP00068423
VitusP00068431
VitusP00068465
1
0.9
0.8
0.7
0.6
0.5
0.4
0.3
0.2
0.1
0

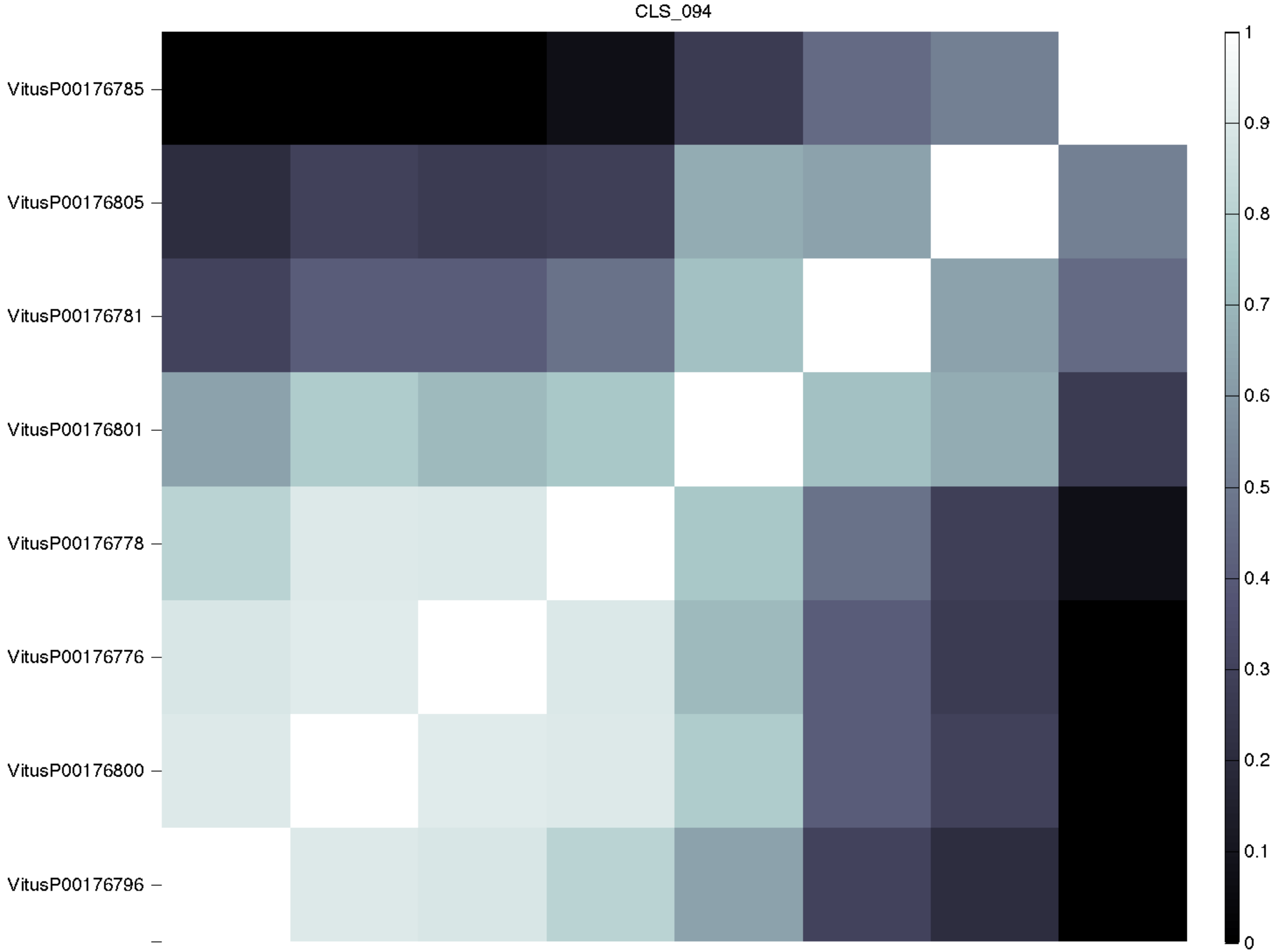
CLS_094
VitusP00176785
VitusP00176805
VitusP00176781
VitusP00176801
VitusP00176778
VitusP00176776
VitusP00176800
VitusP00176796
1
0.9
0.8
0.7
0.6
0.5
0.4
0.3
0.2
0.1
0

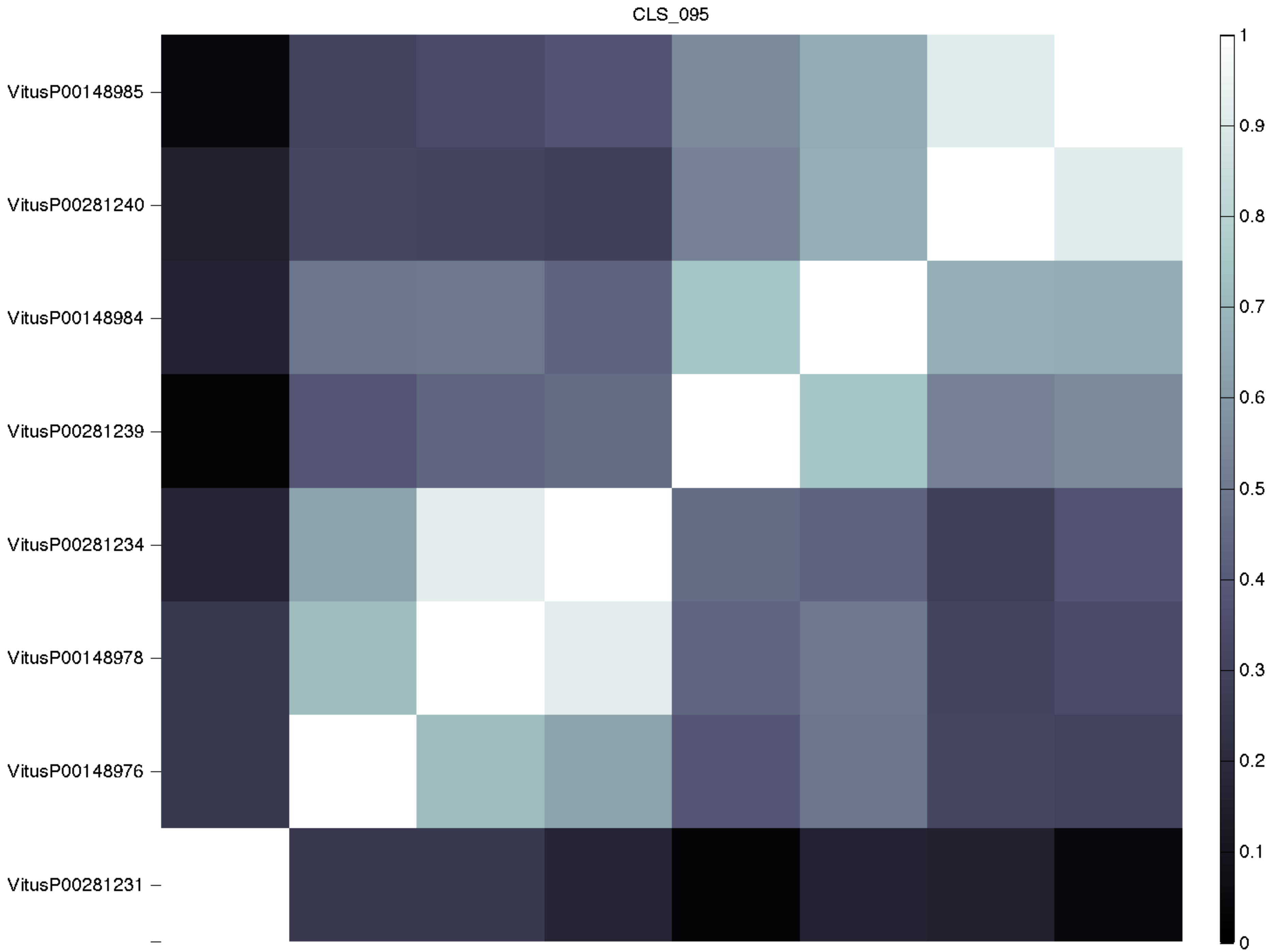
CLS_095
VitusP00148985
VitusP00281240
VitusP00148984
VitusP00281239
VitusP00281234
VitusP00148978
VitusP00148976
VitusP00281231
1
0.9
0.8
0.7
0.6
0.5
0.4
0.3
0.2
0.1
0

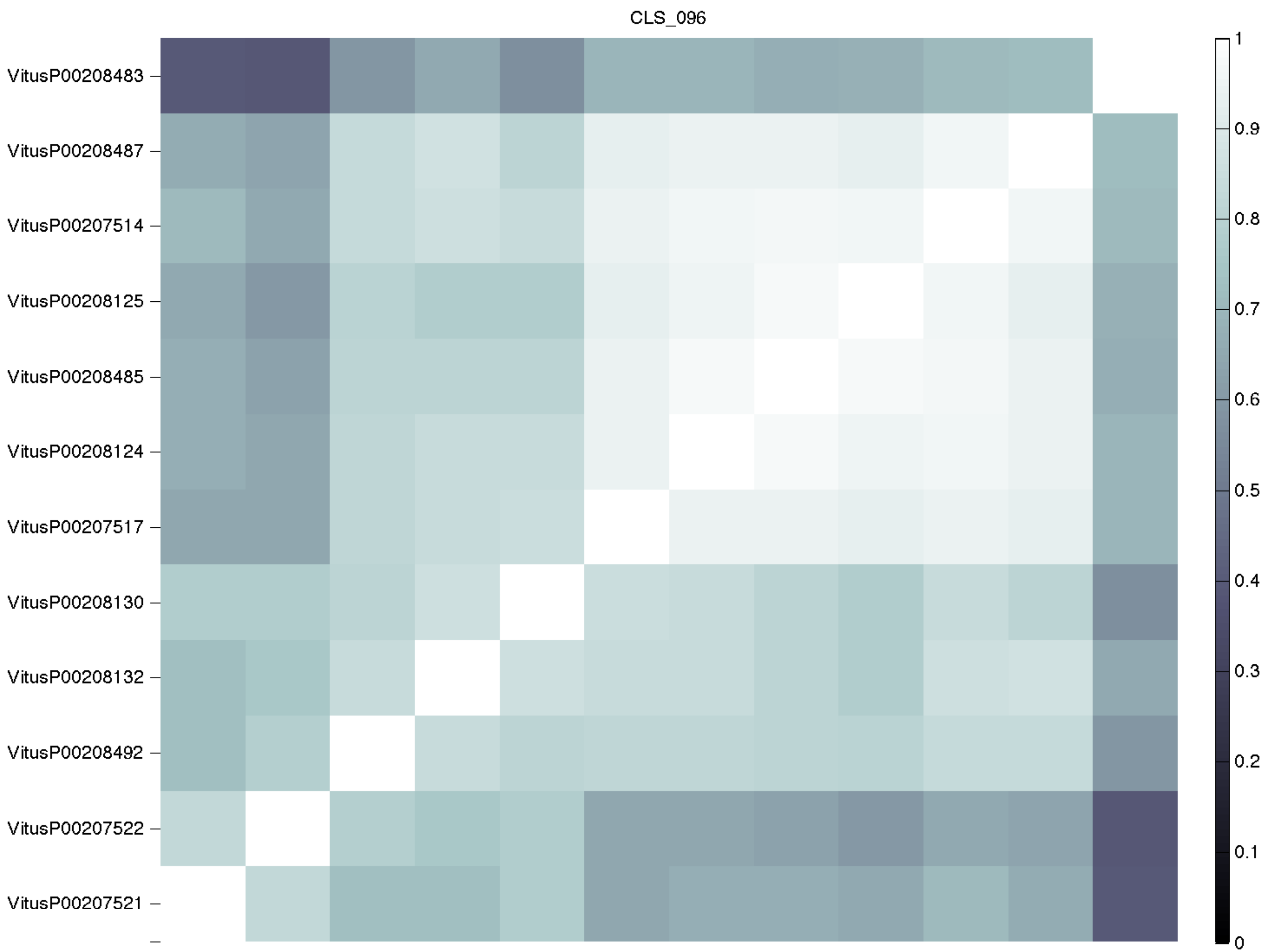
CLS_096
VitusP00208483
VitusP00208487
VitusP00207514
VitusP00208125
VitusP00208485
VitusP00208124
VitusP00207517
VitusP00208130
VitusP00208132
VitusP00208492
VitusP00207522
VitusP00207521
1
0.9
0.8
0.7
0.6
0.5
0.4
0.3
0.2
0.1
0

CLS_097

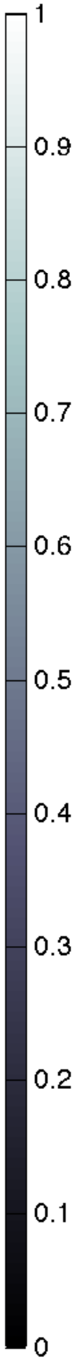

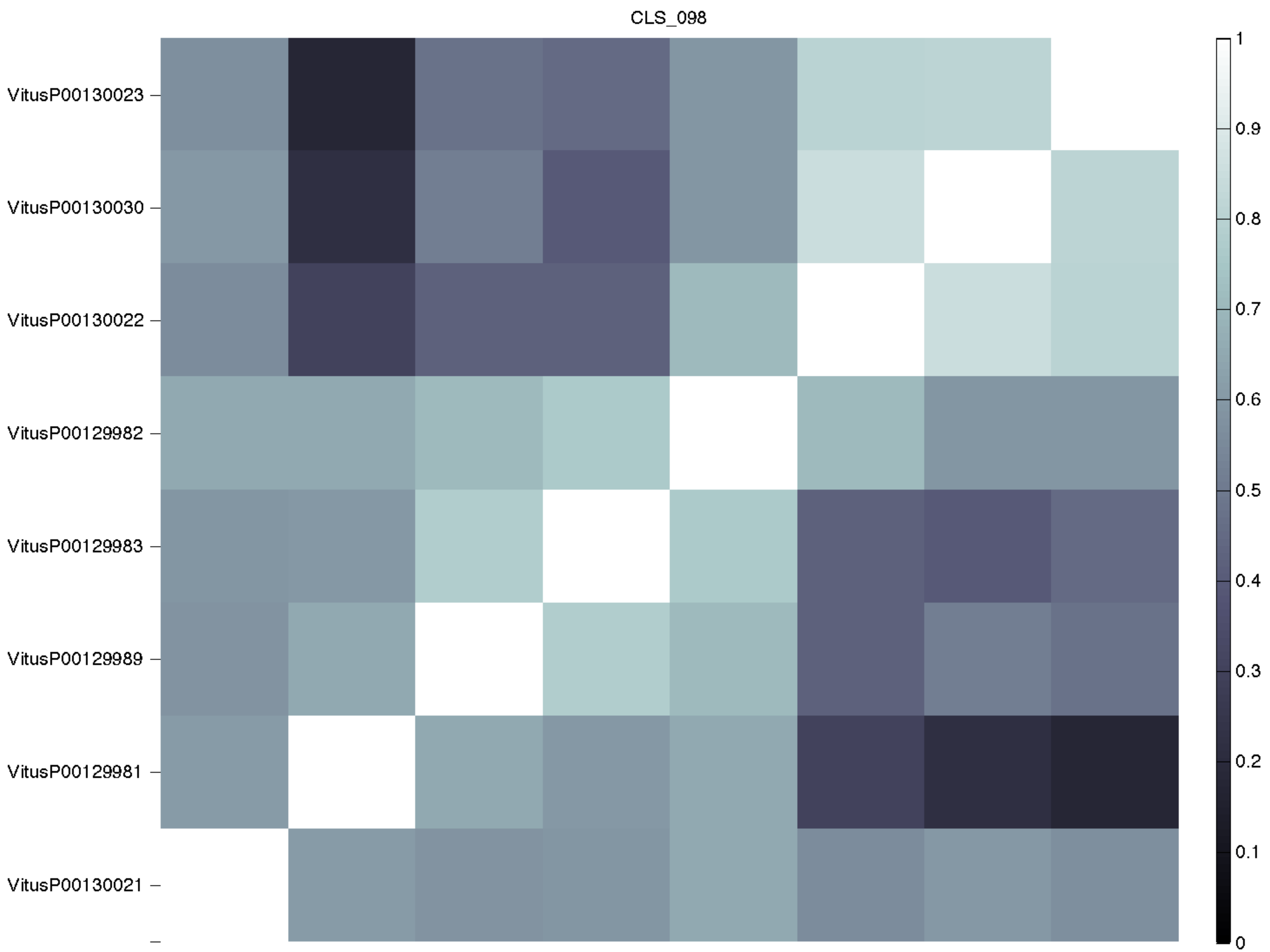
CLS_098
VitusP00130023
VitusP00130030
VitusP00130022
VitusP00129982
VitusP00129983
VitusP00129989
VitusP00129981
VitusP00130021
1
0.9
0.8
0.7
0.6
0.5
0.4
0.3
0.2
0.1
0

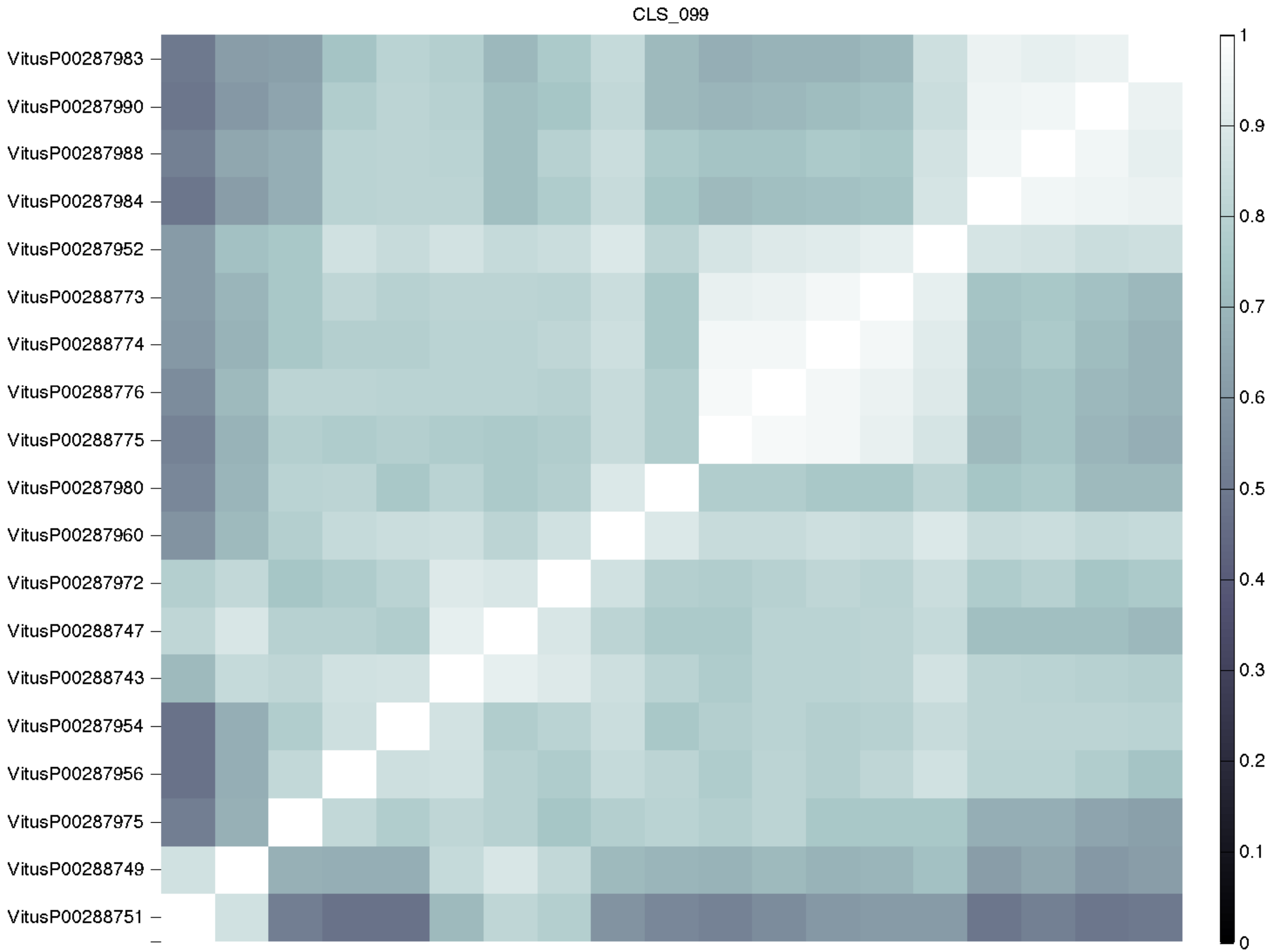
CLS_099
VitusP00287983
VitusP00287990
VitusP00287988
VitusP00287984
VitusP00287952
VitusP00288773
VitusP00288774
VitusP00288776
VitusP00288775
VitusP00287980
VitusP00287960
VitusP00287972
VitusP00288747
VitusP00288743
VitusP00287954
VitusP00287956
VitusP00287975
VitusP00288749
VitusP00288751
1
0.9
0.8
0.7
0.6
0.5
0.4
0.3
0.2
0.1
0

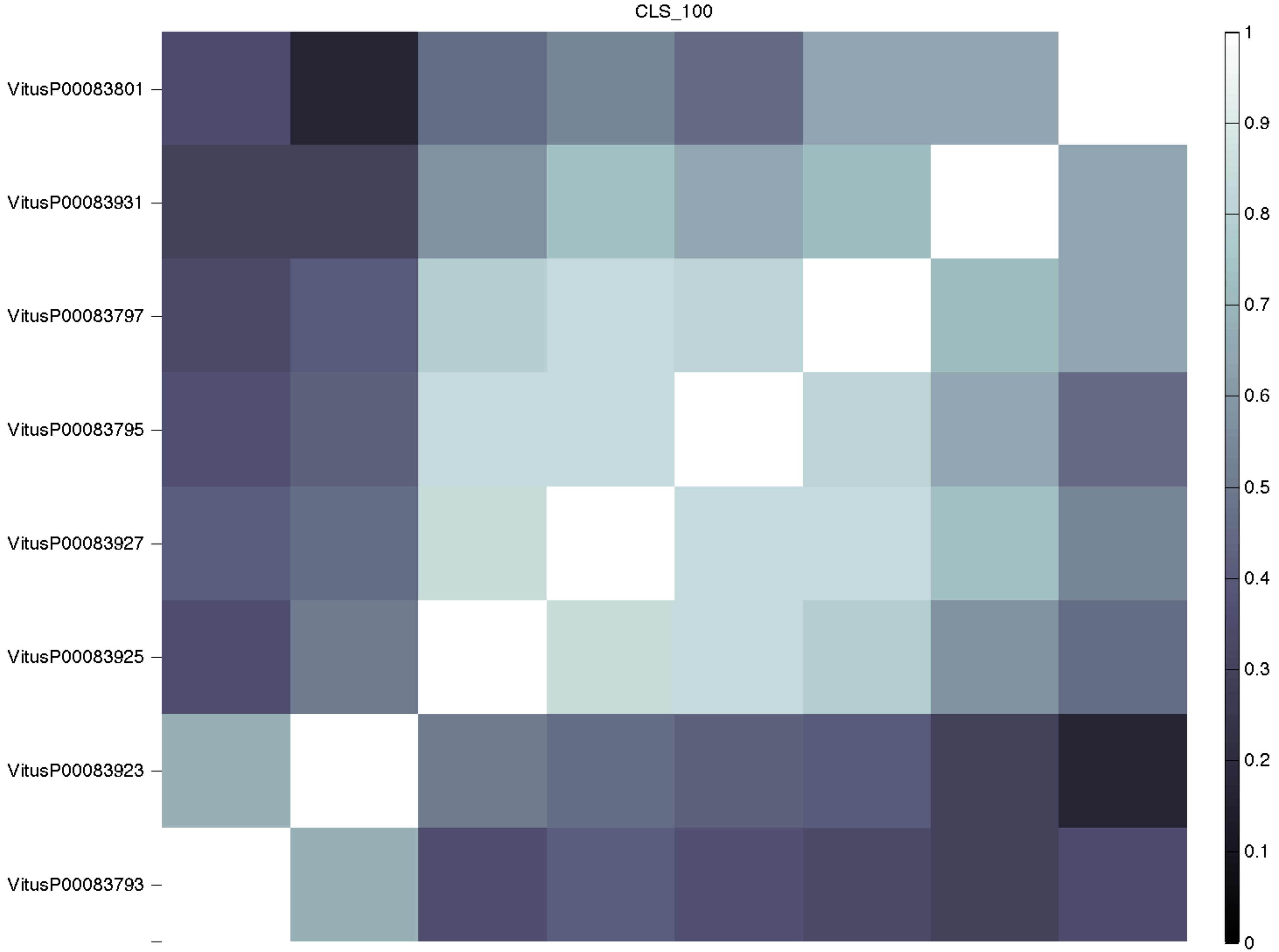

CLS_100
VitusP00083801
VitusP00083931
VitusP00083797
VitusP00083795
VitusP00083927
VitusP00083925
VitusP00083923
VitusP00083793
1
0.9
0.8
0.7
0.6
0.5
0.4
0.3
0.2
0.1
0

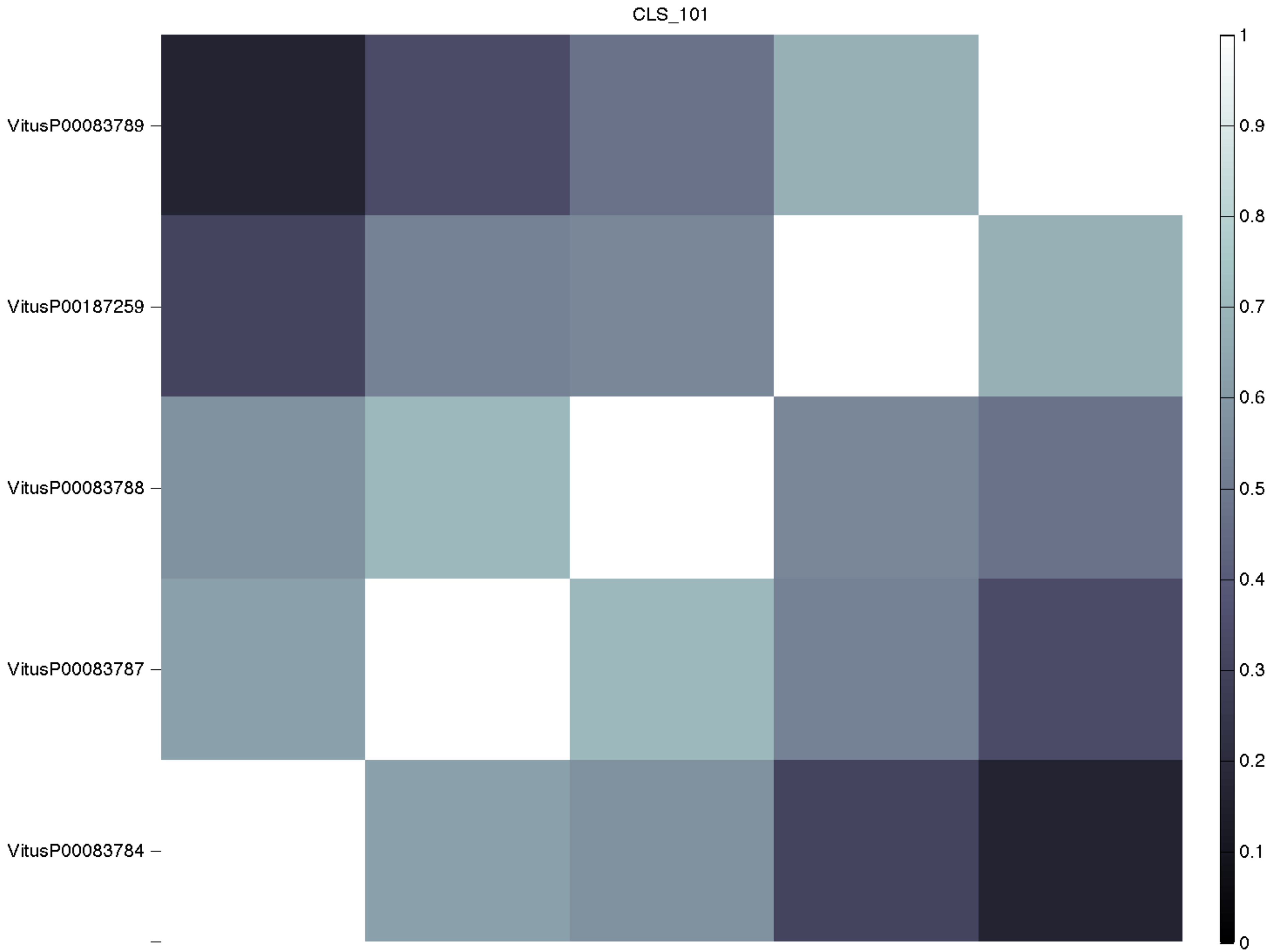
CLS_101
VitusP00083789
VitusP00187259
VitusP00083788
VitusP00083787
VitusP00083784
1
0.9
0.8
0.7
0.6
0.5
0.4
0.3
0.2
0.1
0

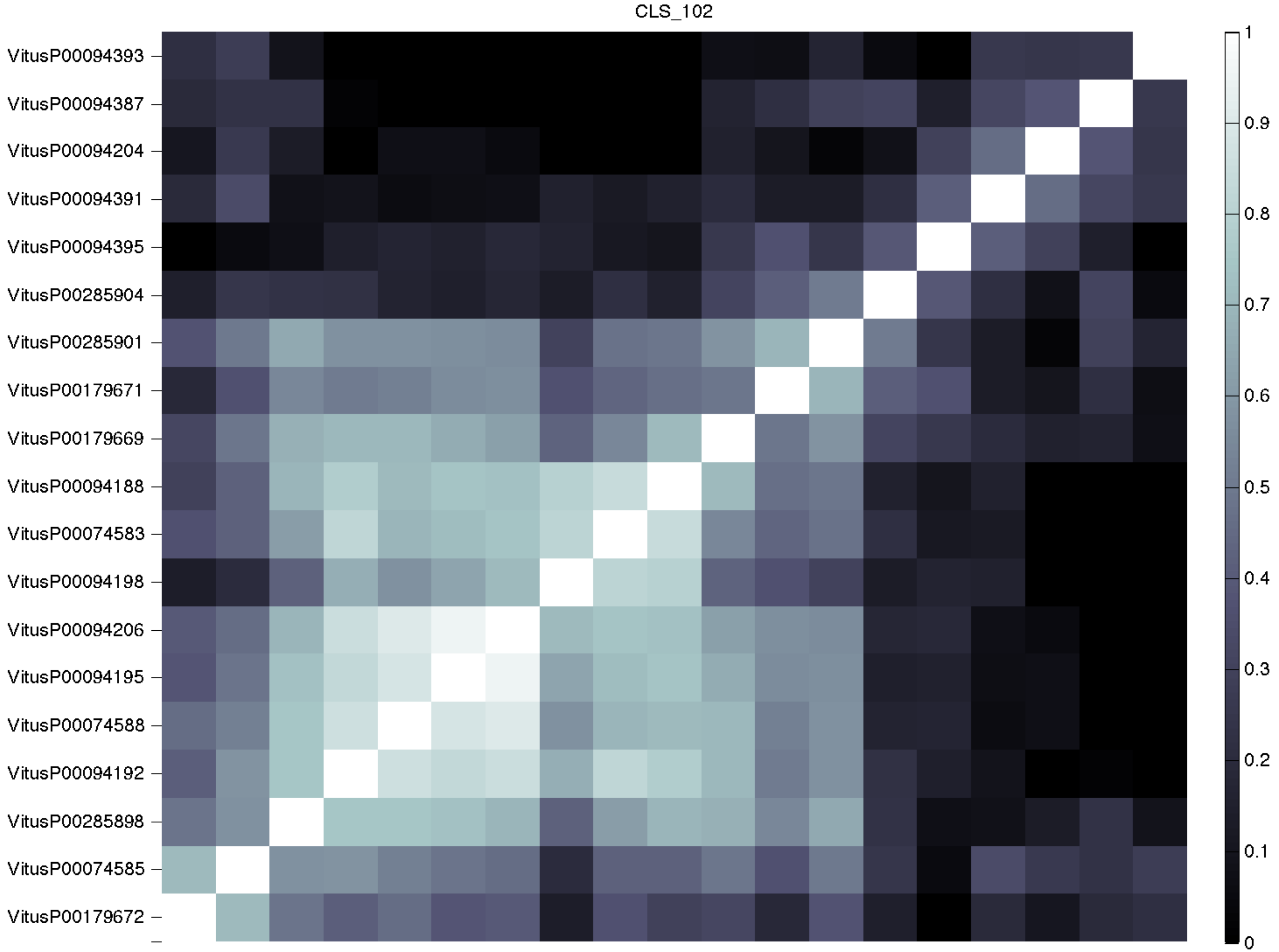

CLS_102
VitusP00094393
VitusP00094387
VitusP00094204
VitusP00094391
VitusP00094395
VitusP00285904
VitusP00285901
VitusP00179671
VitusP00179669
VitusP00094188
VitusP00074583
VitusP00094198
VitusP00094206
VitusP00094195
VitusP00074588
VitusP00094192
VitusP00285898
VitusP00074585
VitusP00179672
1
0.9
0.8
0.7
0.6
0.5
0.4
0.3
0.2
0.1
0

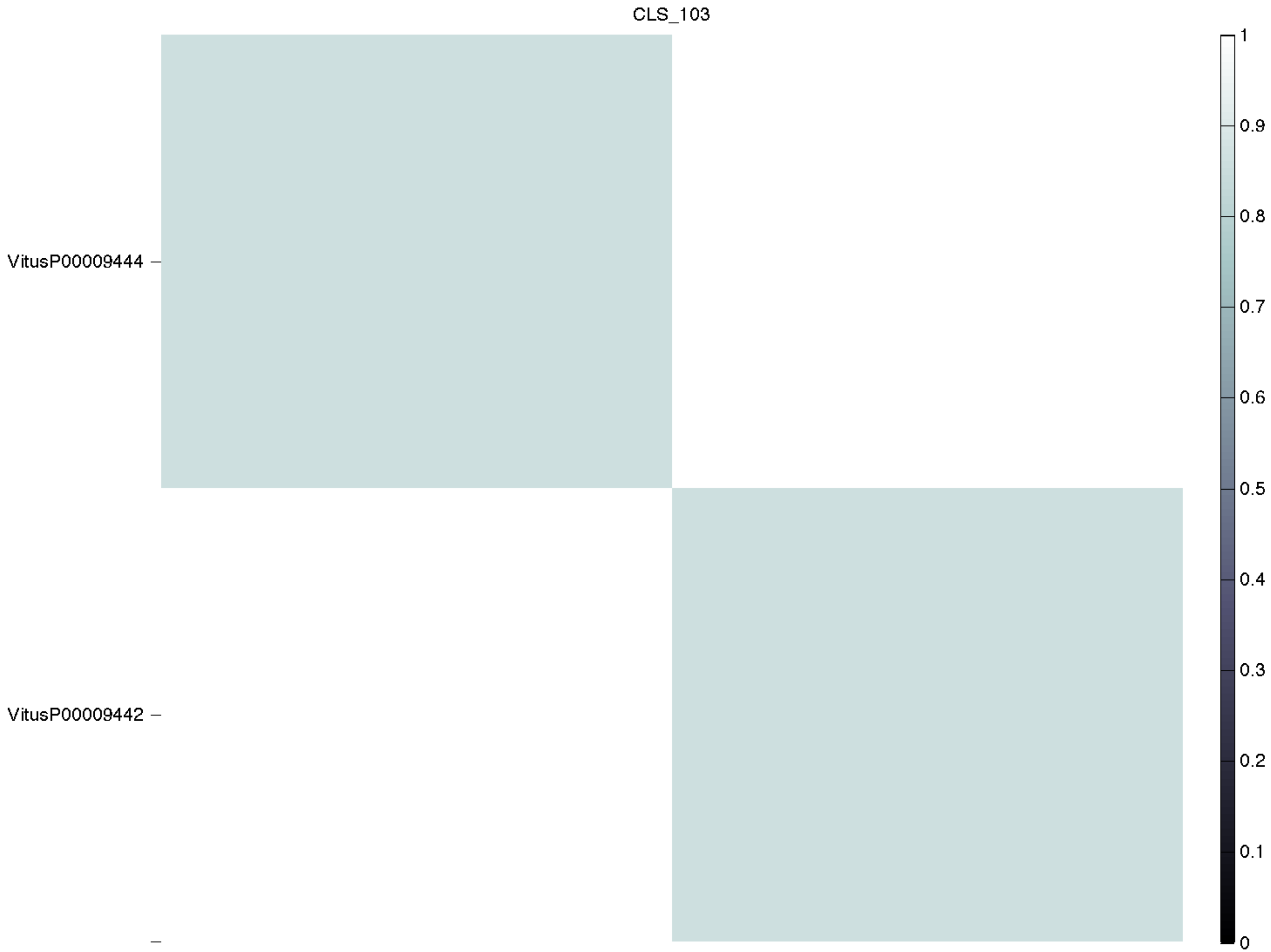
CLS_103
VitusP00009444
VitusP00009442
1
0.9
0.8
0.7
0.6
0.5
0.4
0.3
0.2
0.1
0

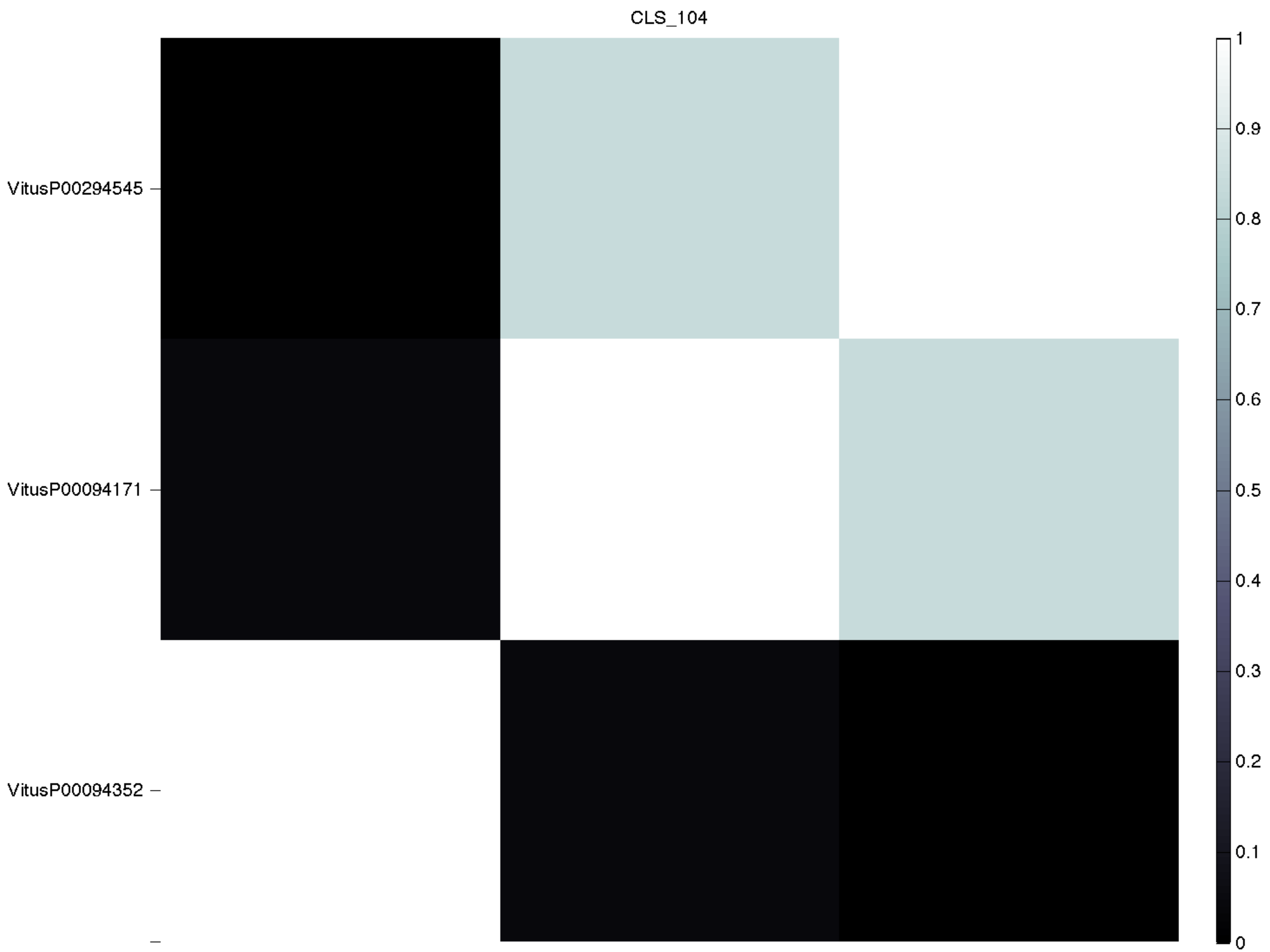

CLS_104
VitusP00294545
VitusP00094171
VitusP00094352
1
0.9
0.8
0.7
0.6
0.5
0.4
0.3
0.2
0.1
0

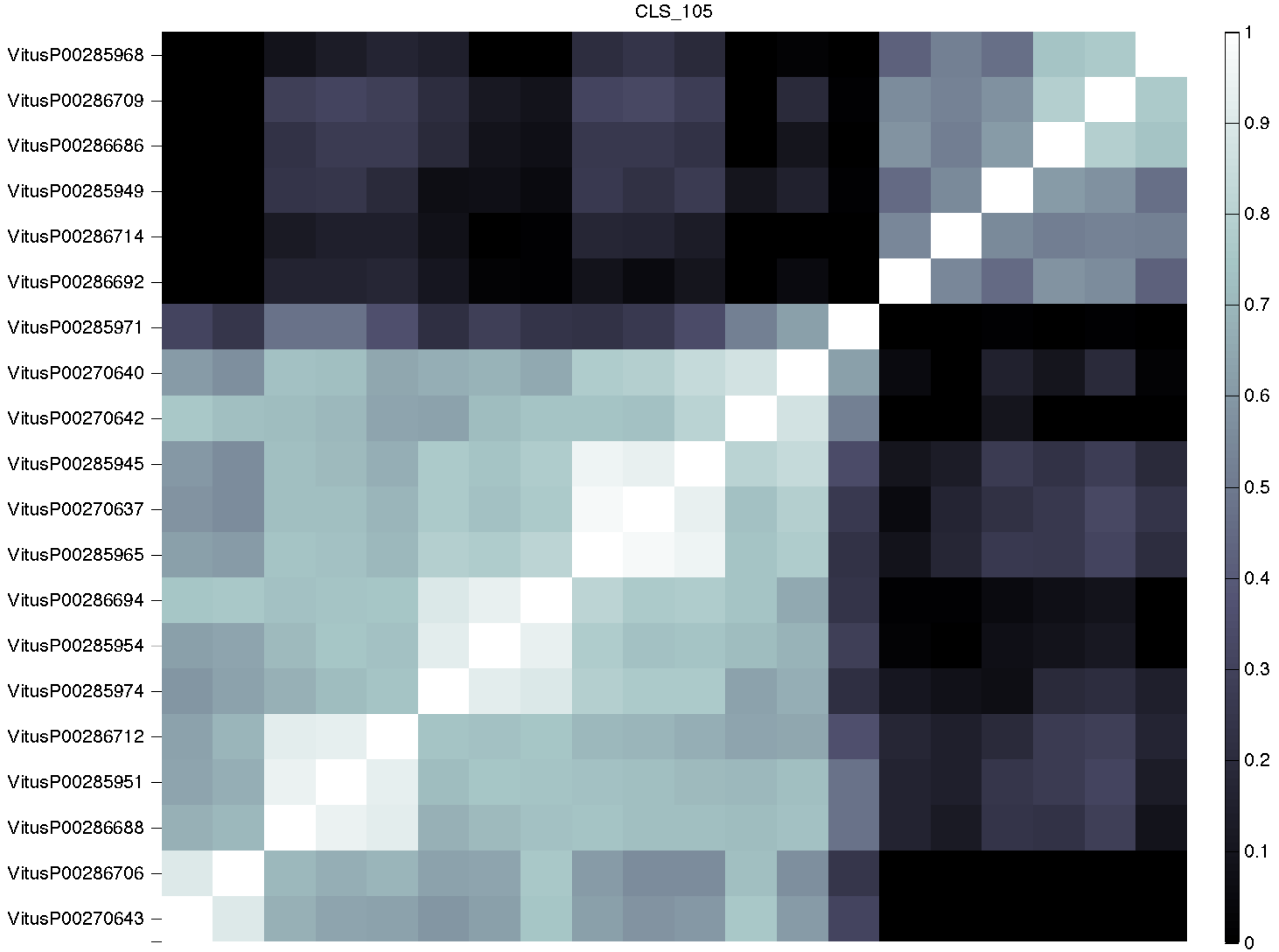

CLS_105
VitusP00285968
VitusP00286709
VitusP00286686
VitusP00285949
VitusP00286714
VitusP00286692
VitusP00285971
VitusP00270640
VitusP00270642
VitusP00285945
VitusP00270637
VitusP00285965
VitusP00286694
VitusP00285954
VitusP00285974
VitusP00286712
VitusP00285951
VitusP00286688
VitusP00286706
VitusP00270643
1
0.9
0.8
0.7
0.6
0.5
0.4
0.3
0.2
0.1
0

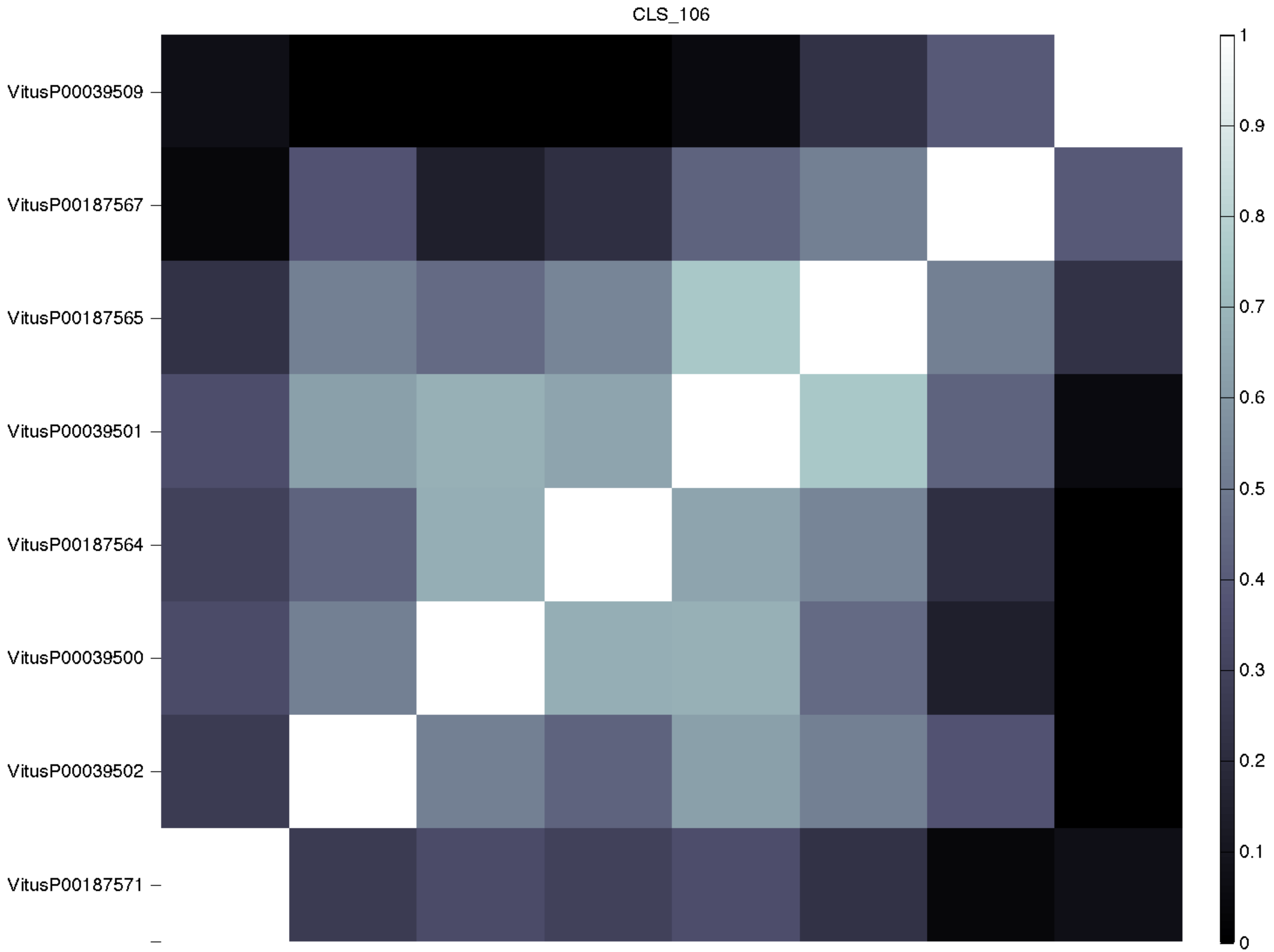
CLS_106
VitusP00039509
VitusP00187567
VitusP00187565
VitusP00039501
VitusP00187564
VitusP00039500
VitusP00039502
VitusP00187571
1
0.9
0.8
0.7
0.6
0.5
0.4
0.3
0.2
0.1
0

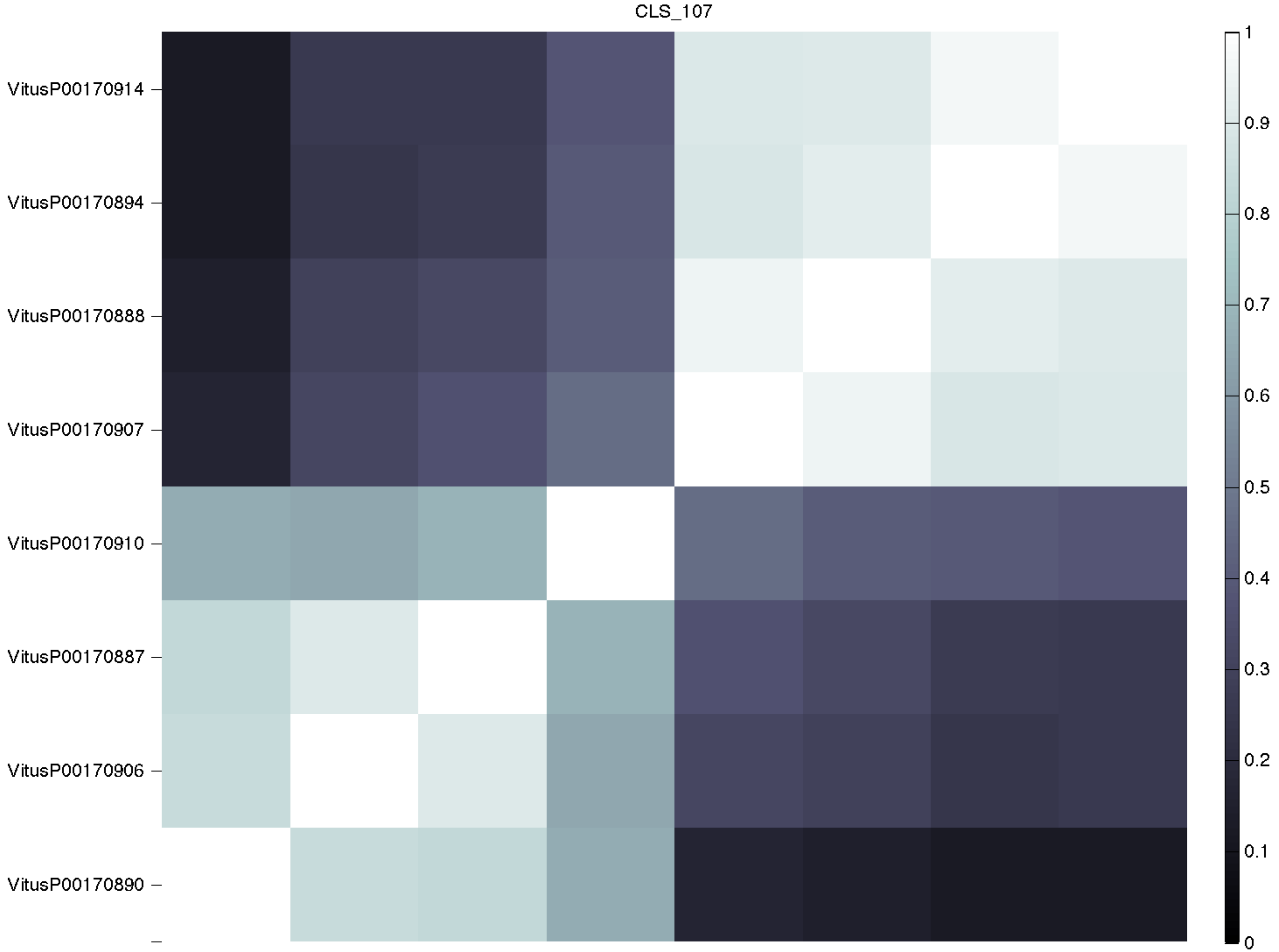
CLS_107
VitusP00170914
VitusP00170894
VitusP00170888
VitusP00170907
VitusP00170910
VitusP00170887
VitusP00170906
VitusP00170890
1
0.9
0.8
0.7
0.6
0.5
0.4
0.3
0.2
0.1
0

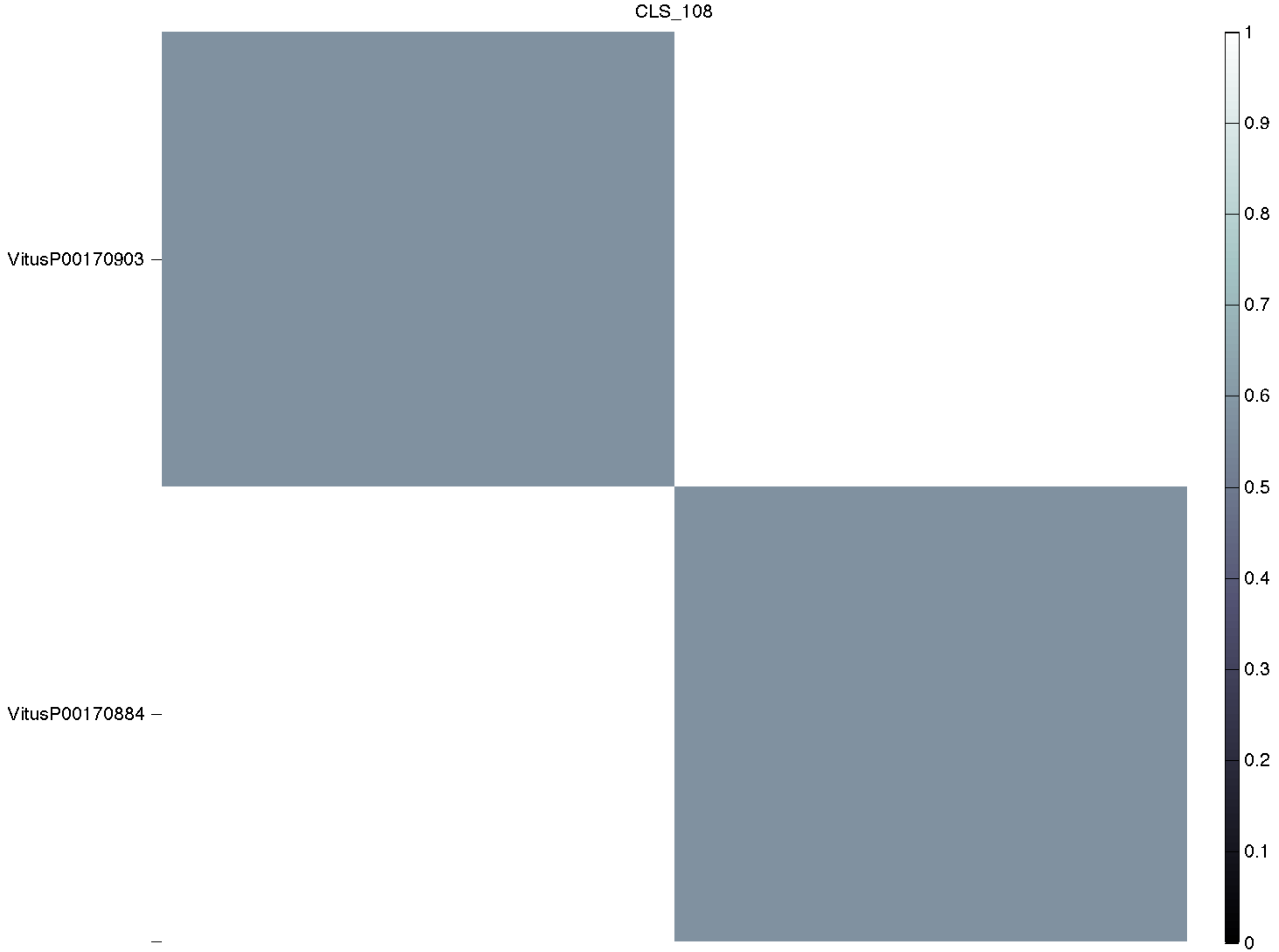
CLS_108
VitusP00170903
VitusP00170884
1
0.9
0.8
0.7
0.6
0.5
0.4
0.3
0.2
0.1
0

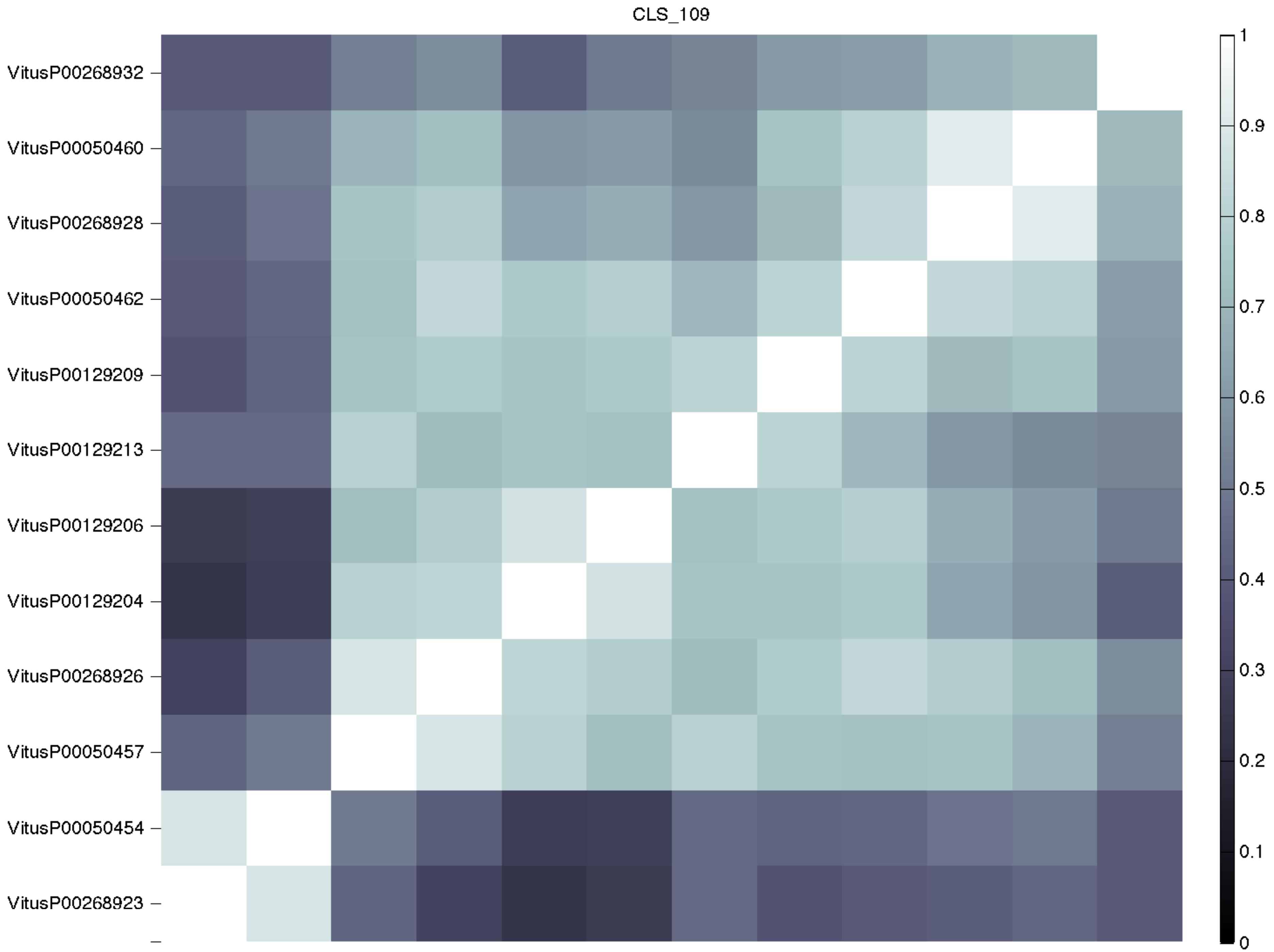

CLS_109
VitusP00268932
VitusP00050460
VitusP00268928
VitusP00050462
VitusP00129209
VitusP00129213
VitusP00129206
VitusP00129204
VitusP00268926
VitusP00050457
VitusP00050454
VitusP00268923
1
0.9
0.8
0.7
0.6
0.5
0.4
0.3
0.2
0.1
0

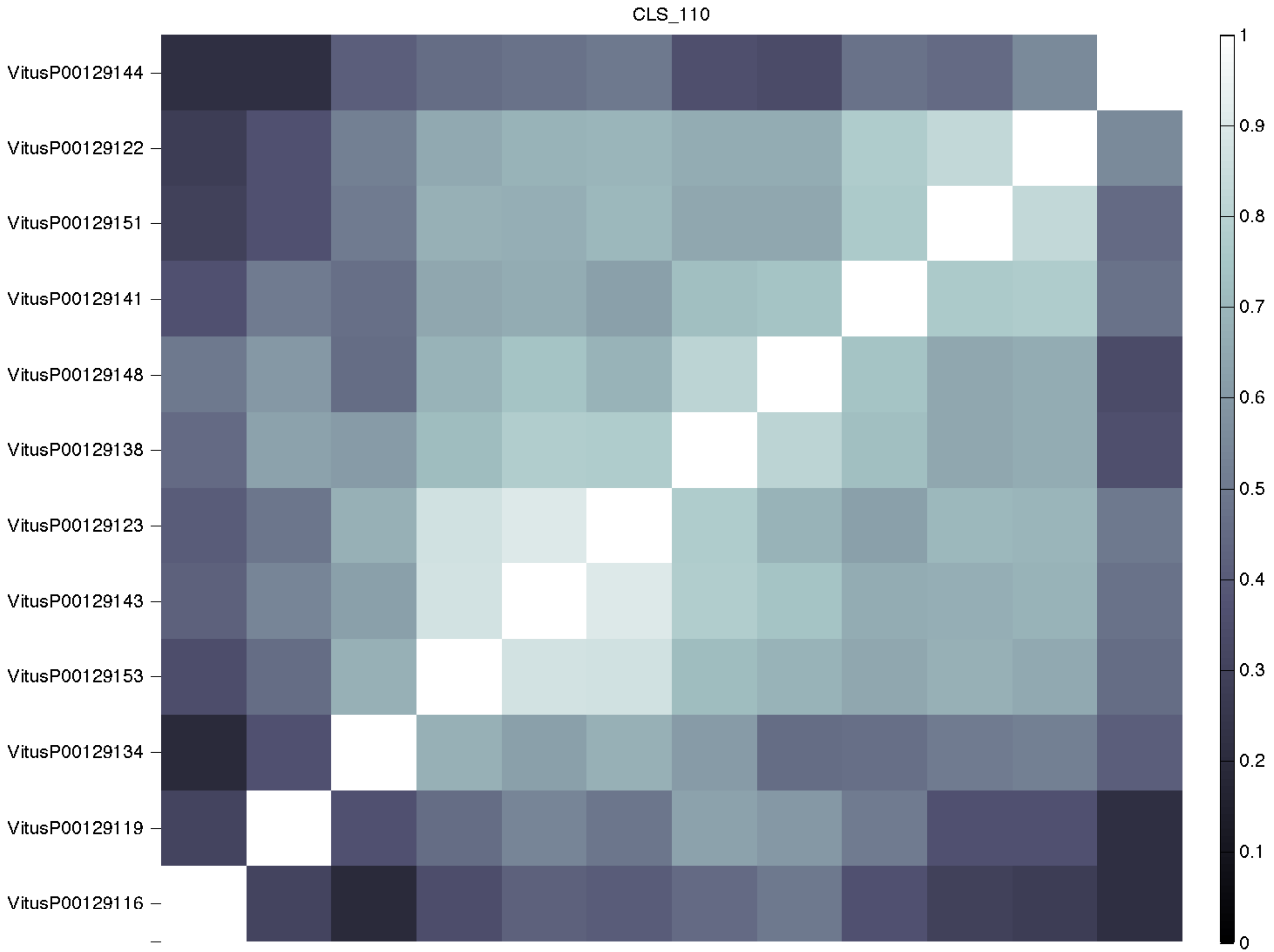
CLS_110
VitusP00129144
VitusP00129122
VitusP00129151
VitusP00129141
VitusP00129148
VitusP00129138
VitusP00129123
VitusP00129143
VitusP00129153
VitusP00129134
VitusP00129119
VitusP00129116
1
0.9
0.8
0.7
0.6
0.5
0.4
0.3
0.2
0.1
0

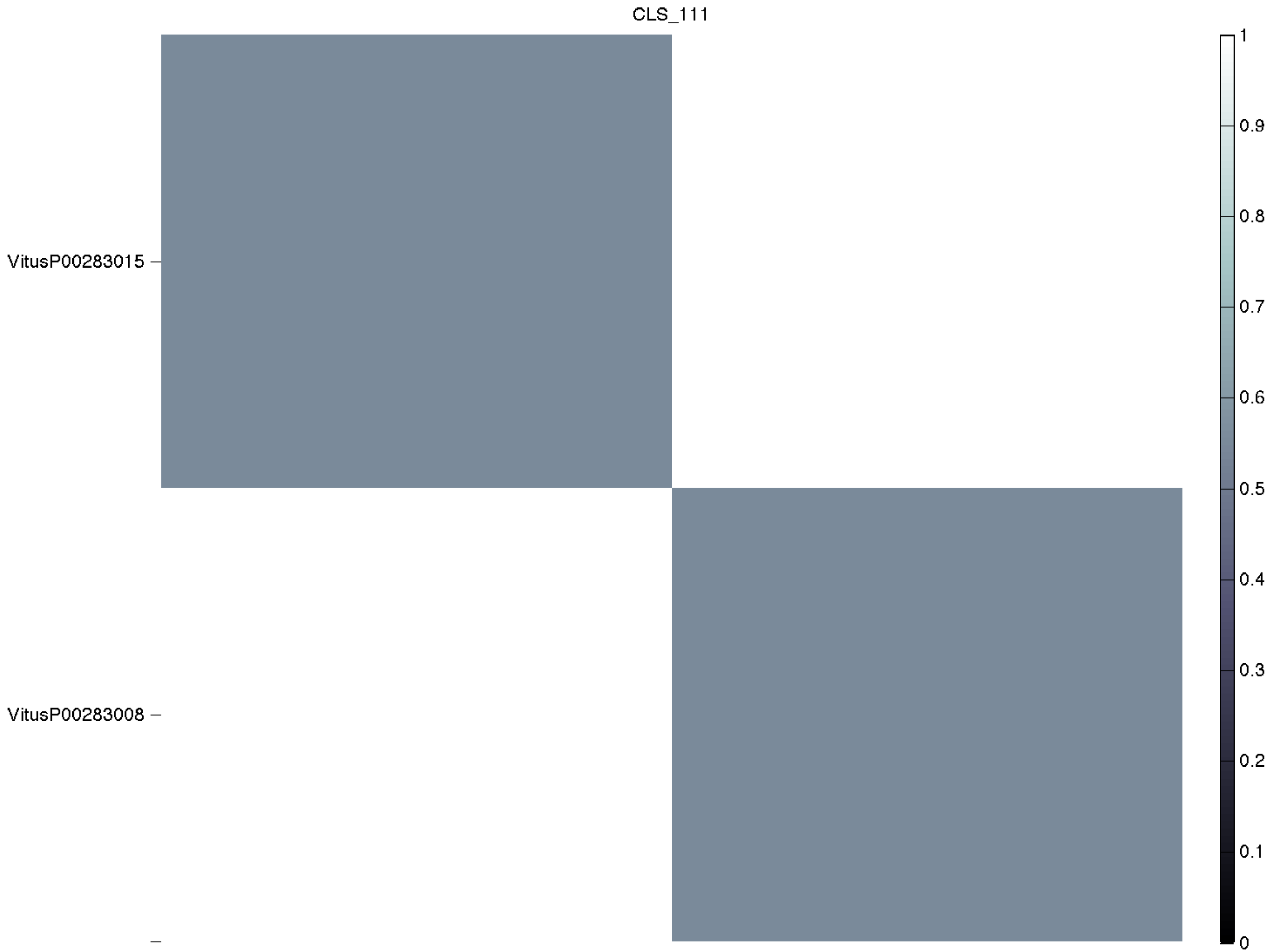
CLS_111
VitusP00283015
VitusP00283008
1
0.9
0.8
0.7
0.6
0.5
0.4
0.3
0.2
0.1
0

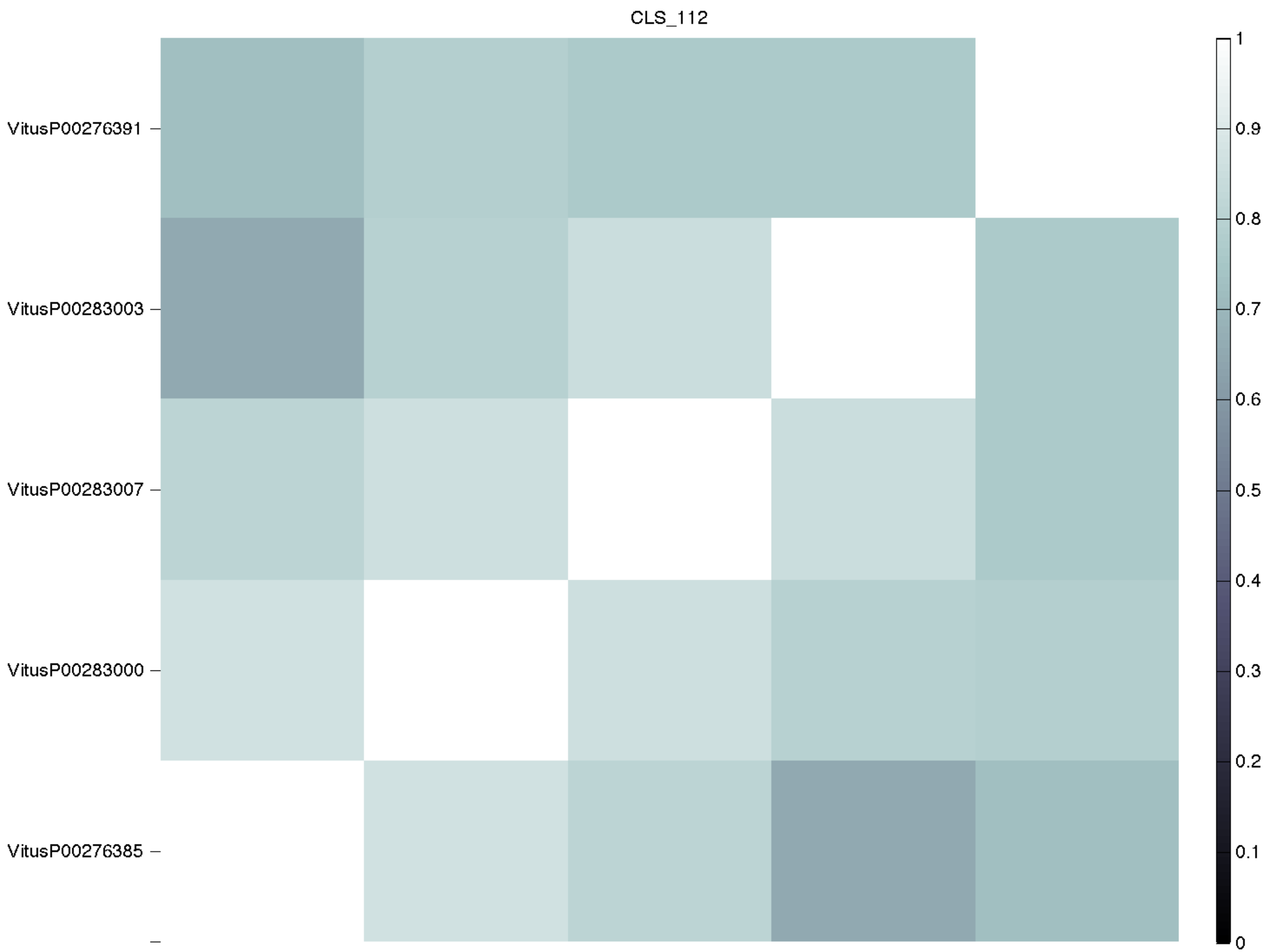
CLS_112
VitusP00276391
VitusP00283003
VitusP00283007
VitusP00283000
VitusP00276385
1
0.9
0.8
0.7
0.6
0.5
0.4
0.3
0.2
0.1
0

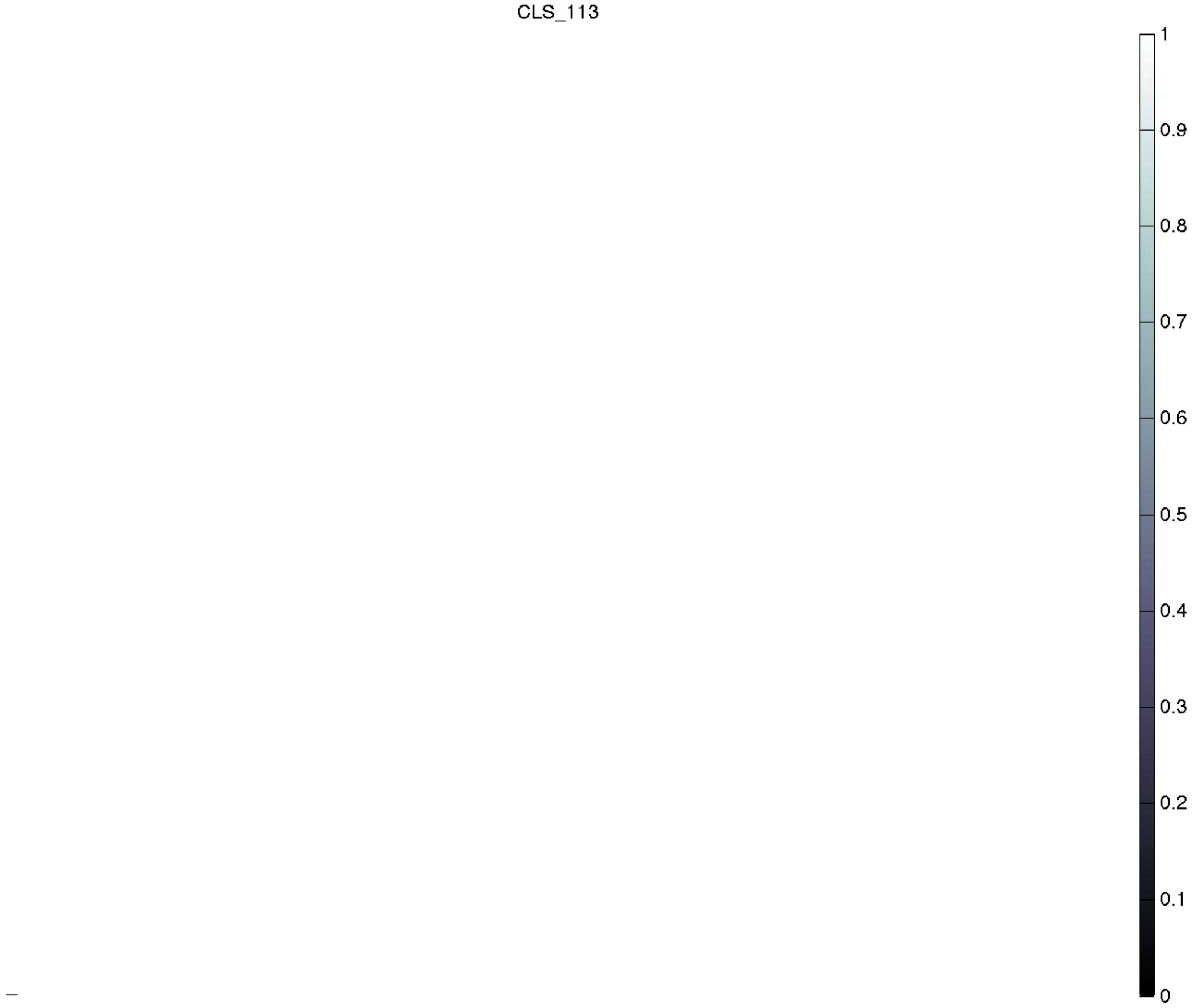
CLS_113
1
0.9
0.8
0.7
0.6
0.5
0.4
0.3
0.2
0.1
0

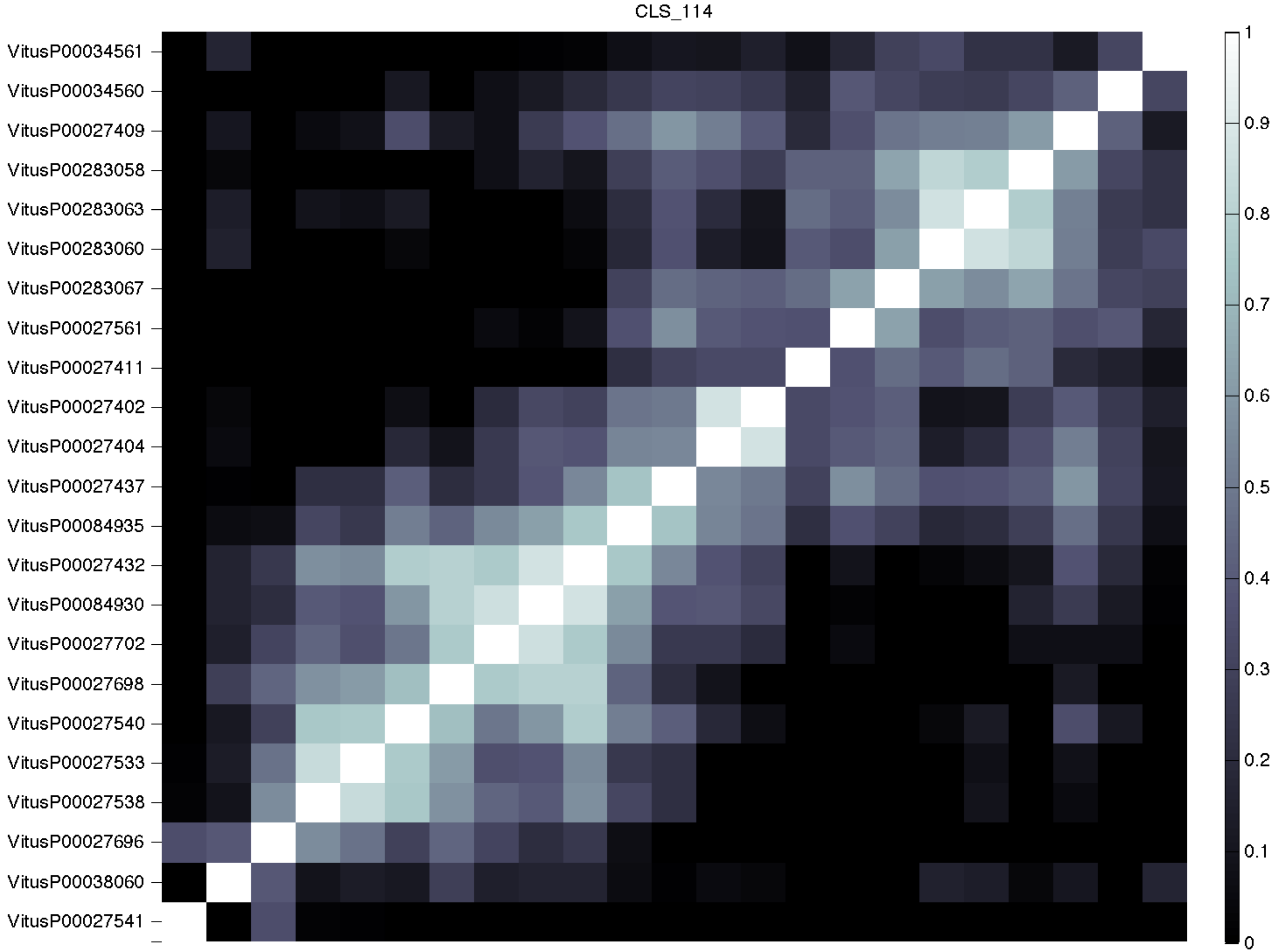
CLS_114
VitusP00034561
VitusP00034560
VitusP00027409
VitusP00283058
VitusP00283063
VitusP00283060
VitusP00283067
VitusP00027561
VitusP00027411
VitusP00027402
VitusP00027404
VitusP00027437
VitusP00084935
VitusP00027432
VitusP00084930
VitusP00027702
VitusP00027698
VitusP00027540
VitusP00027533
VitusP00027538
VitusP00027696
VitusP00038060
VitusP00027541
1
0.9
0.8
0.7
0.6
0.5
0.4
0.3
0.2
0.1
0

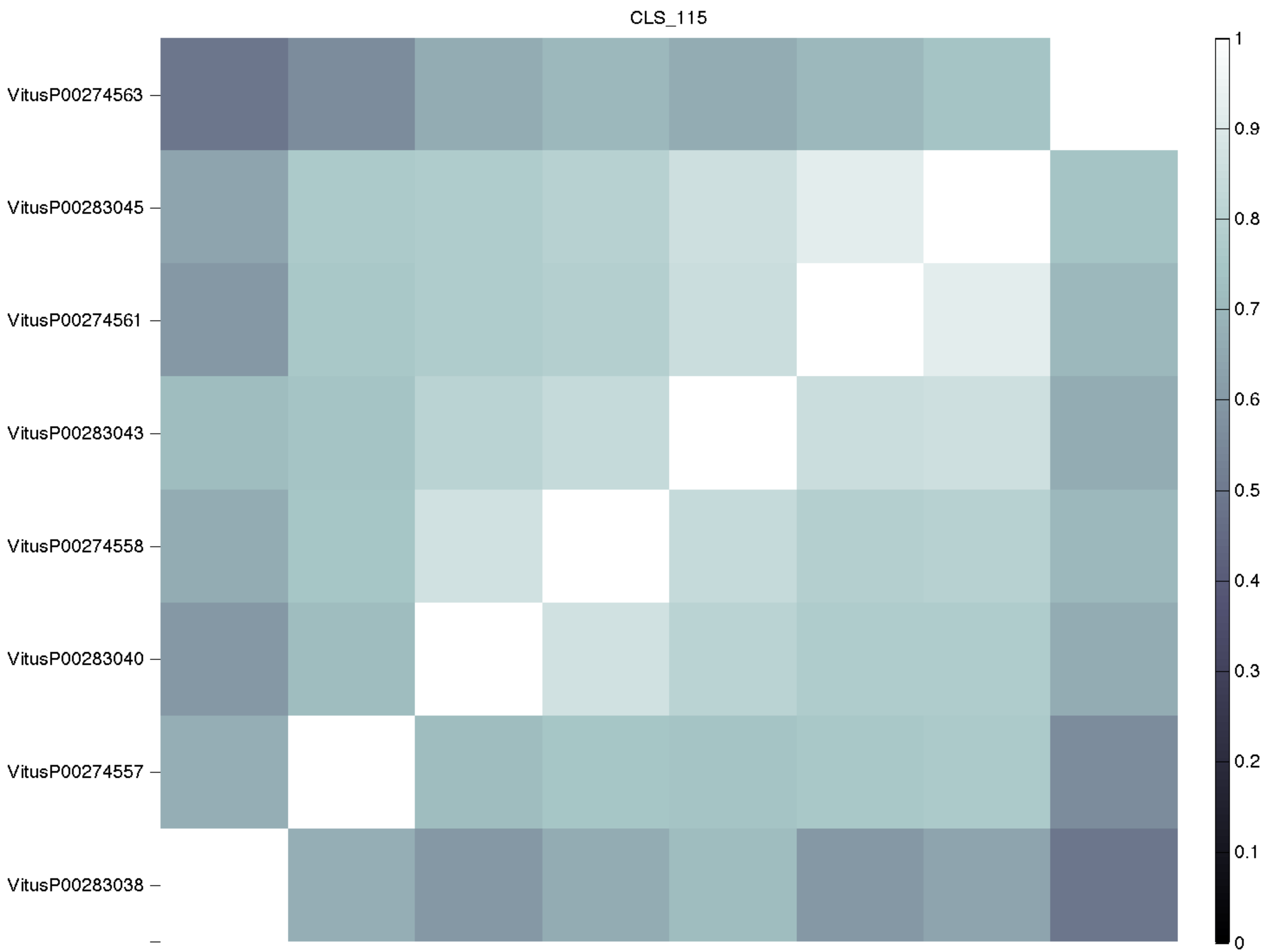

CLS_115
VitusP00274563
VitusP00283045
VitusP00274561
VitusP00283043
VitusP00274558
VitusP00283040
VitusP00274557
VitusP00283038
1
0.9
0.8
0.7
0.6
0.5
0.4
0.3
0.2
0.1
0

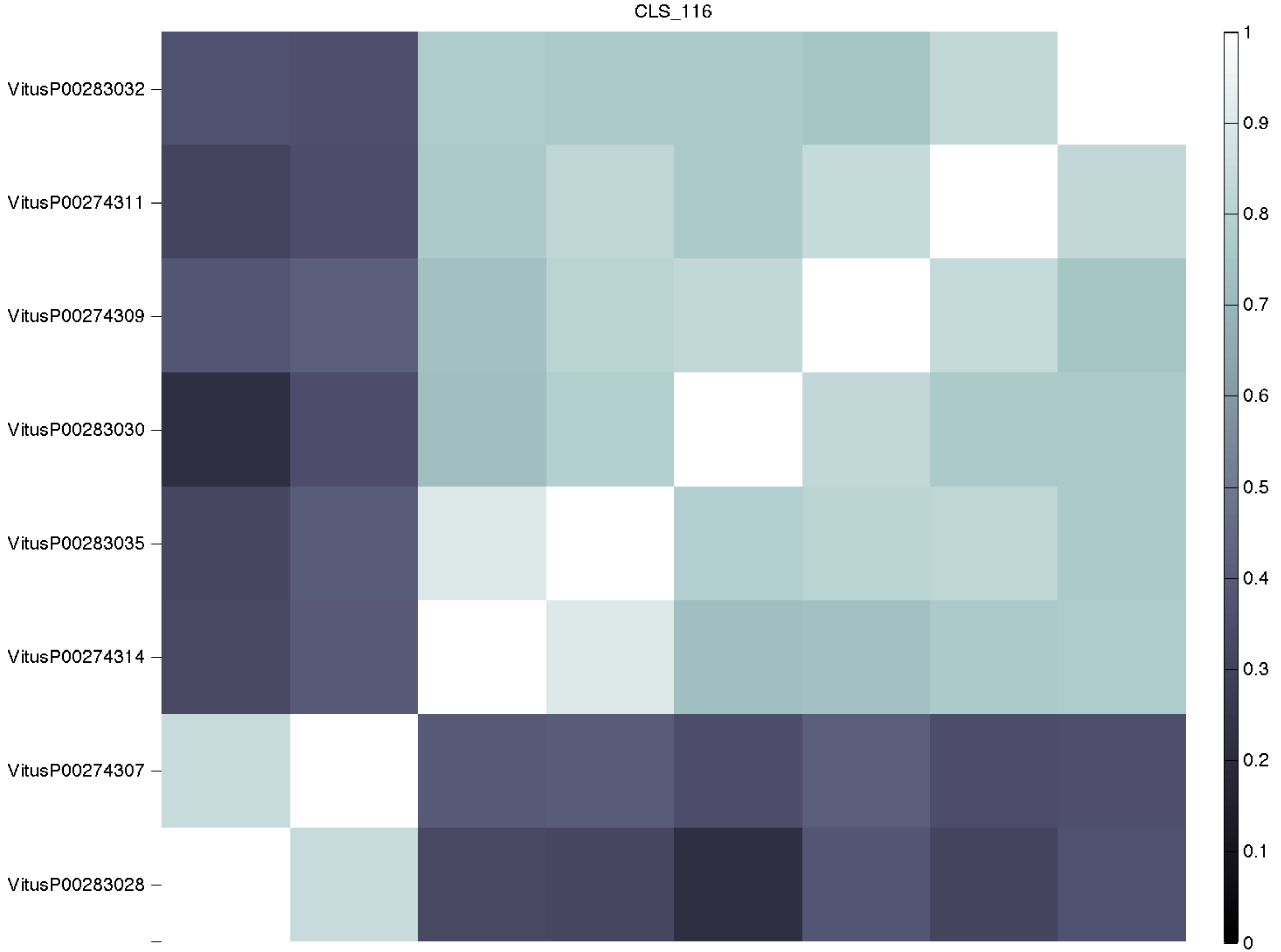

CLS_116
VitusP00283032
VitusP00274311
VitusP00274309
VitusP00283030
VitusP00283035
VitusP00274314
VitusP00274307
VitusP00283028
1
0.9
0.8
0.7
0.6
0.5
0.4
0.3
0.2
0.1
0

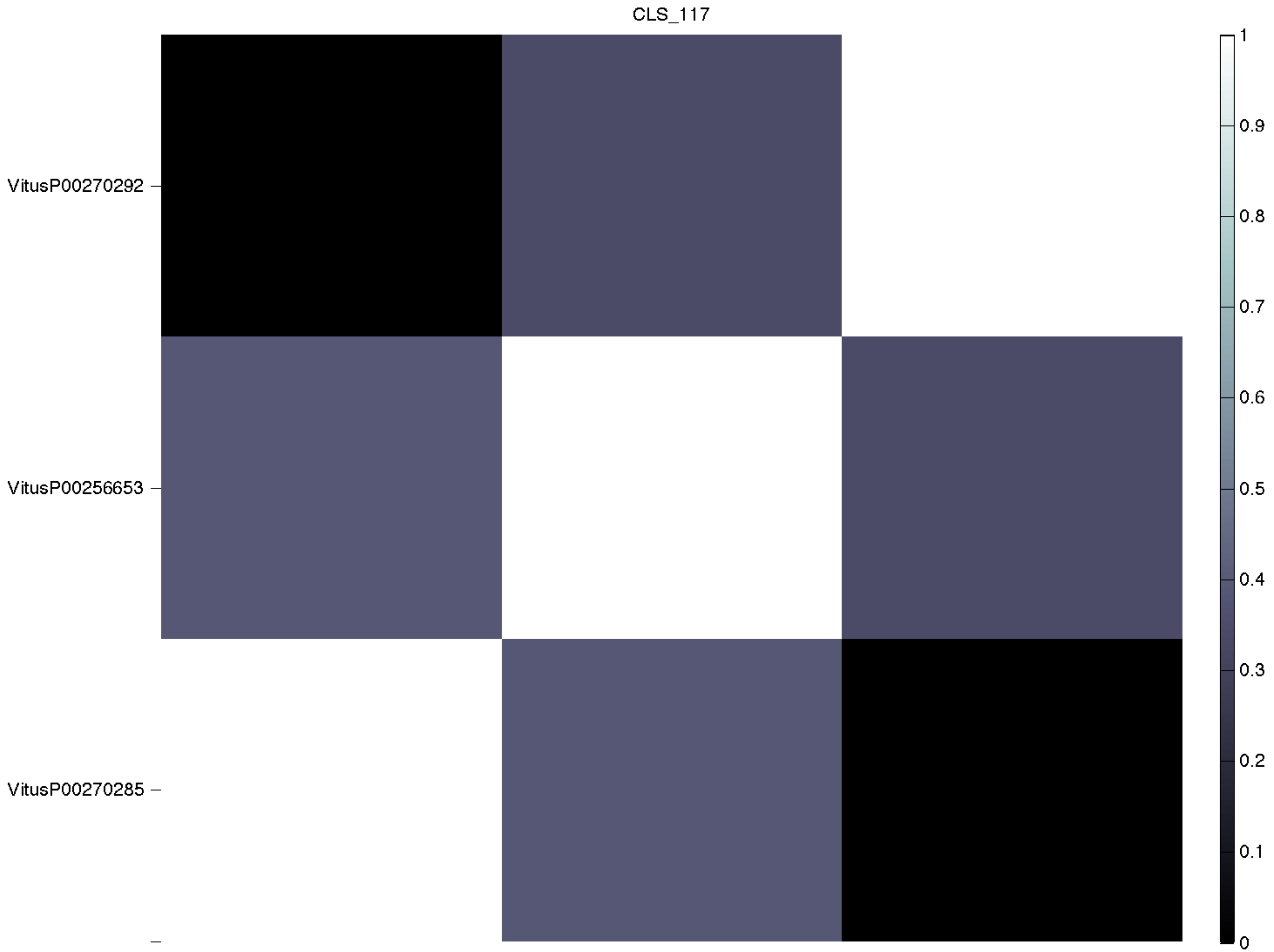

CLS_117
VitusP00270292
VitusP00256653
VitusP00270285
1
0.9
0.8
0.7
0.6
0.5
0.4
0.3
0.2
0.1
0

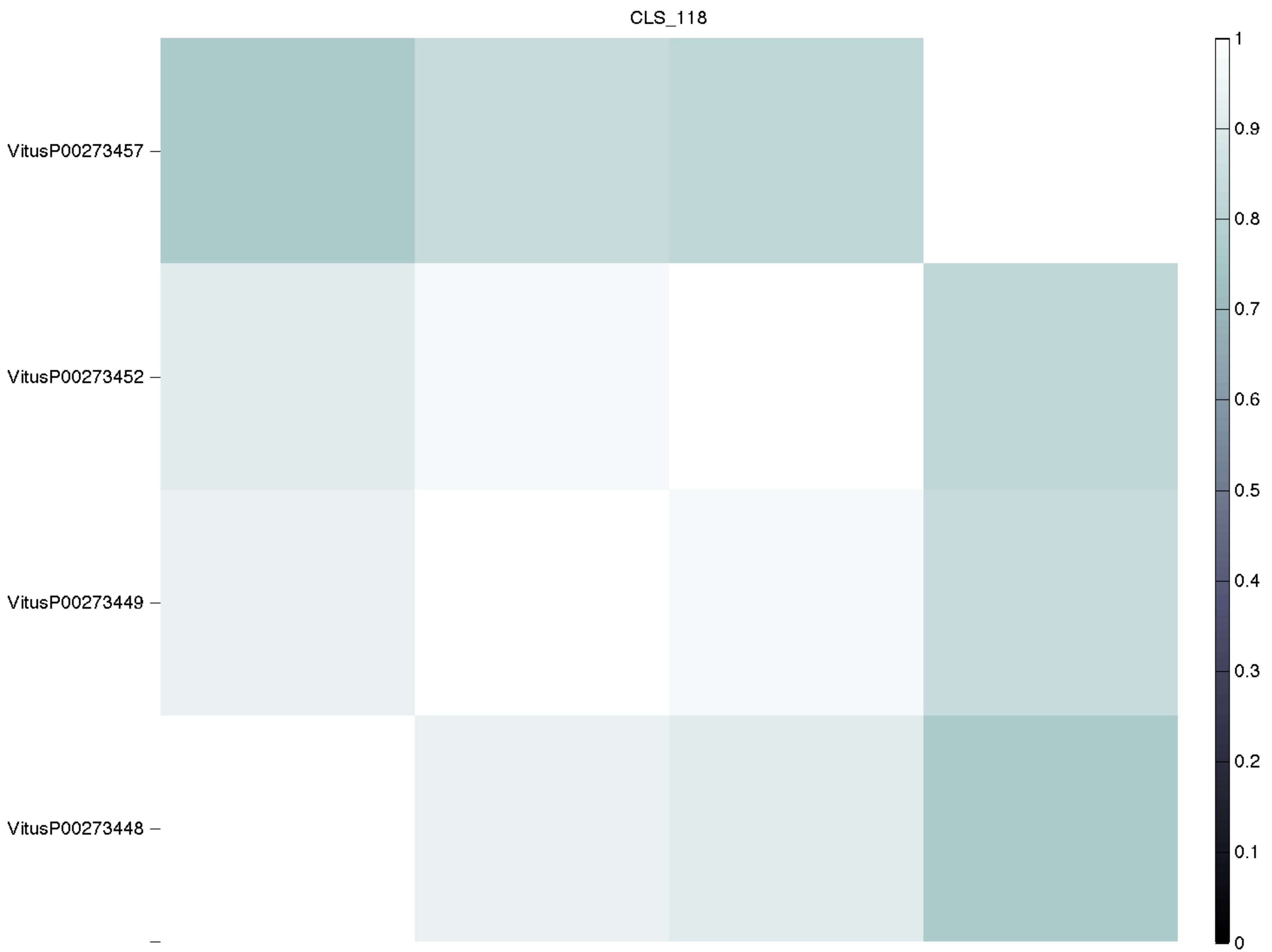
CLS_118
VitusP00273457
VitusP00273452
VitusP00273449
VitusP00273448
1
0.9
0.8
0.7
0.6
0.5
0.4
0.3
0.2
0.1
0

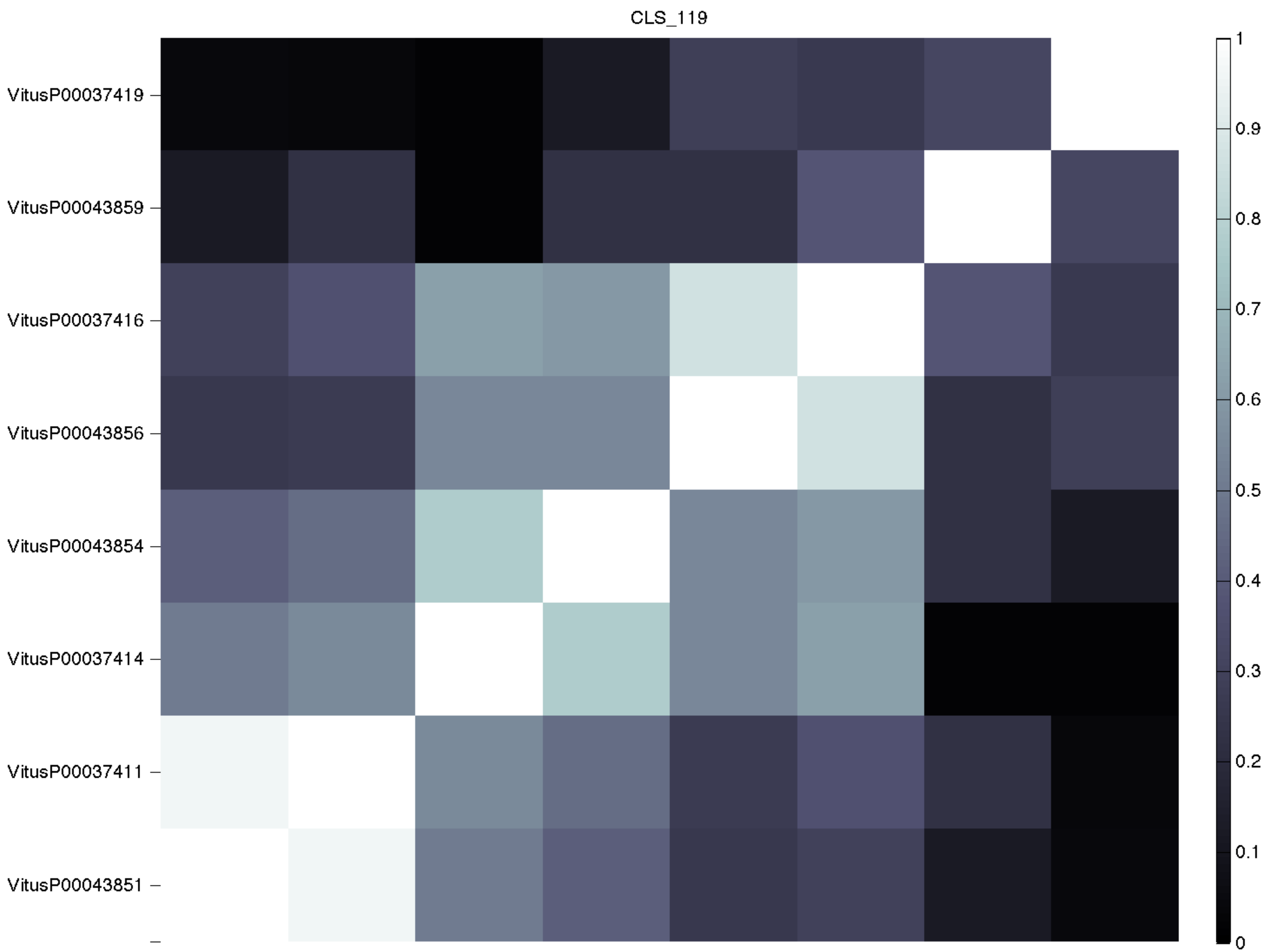

CLS_119
VitusP00037419
VitusP00043859
VitusP00037416
VitusP00043856
VitusP00043854
VitusP00037414
VitusP00037411
VitusP00043851
1
0.9
0.8
0.7
0.6
0.5
0.4
0.3
0.2
0.1
0

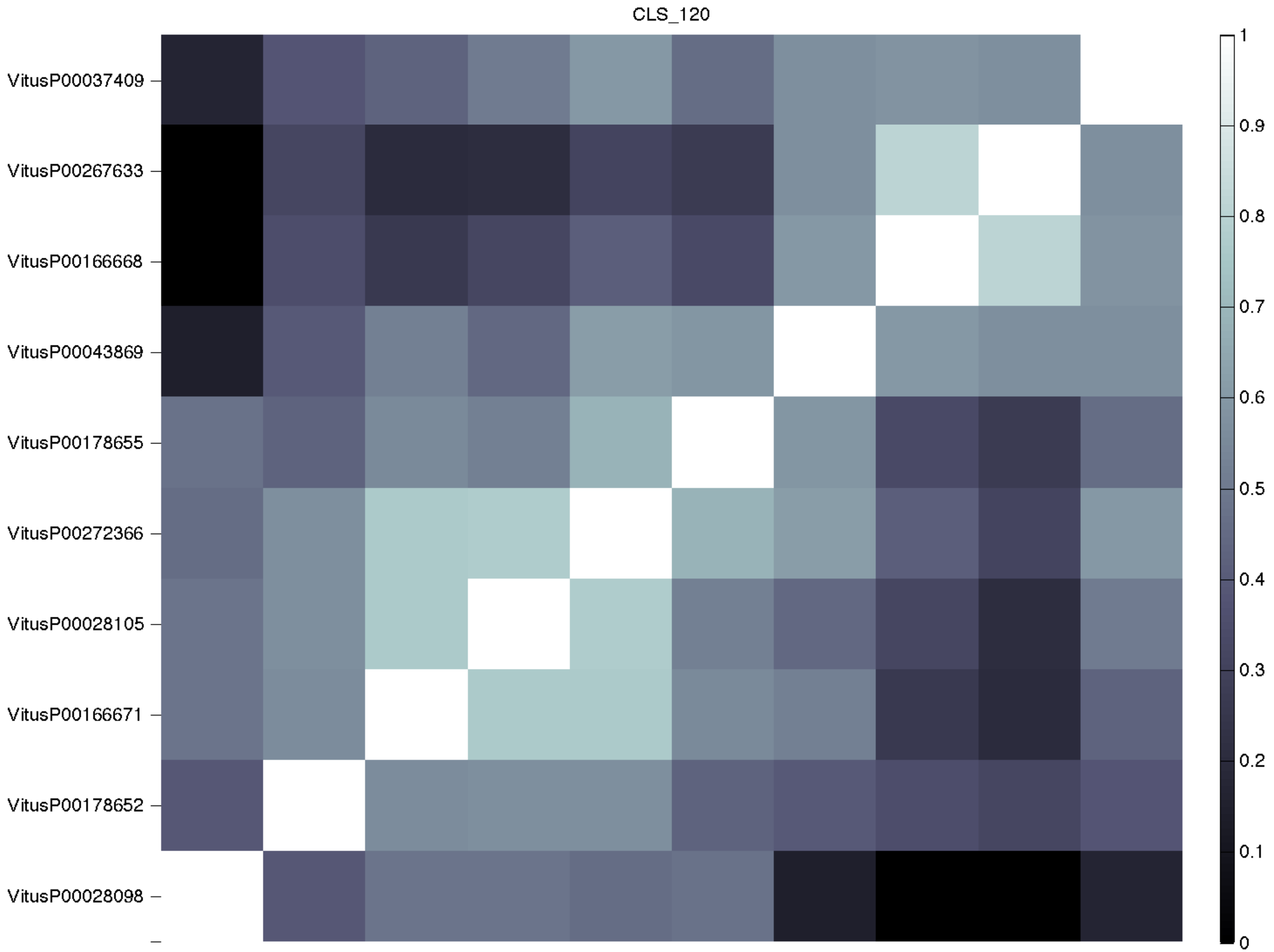
CLS_120
VitusP00037409
VitusP00267633
VitusP00166668
VitusP00043869
VitusP00178655
VitusP00272366
VitusP00028105
VitusP00166671
VitusP00178652
VitusP00028098
1
0.9
0.8
0.7
0.6
0.5
0.4
0.3
0.2
0.1
0

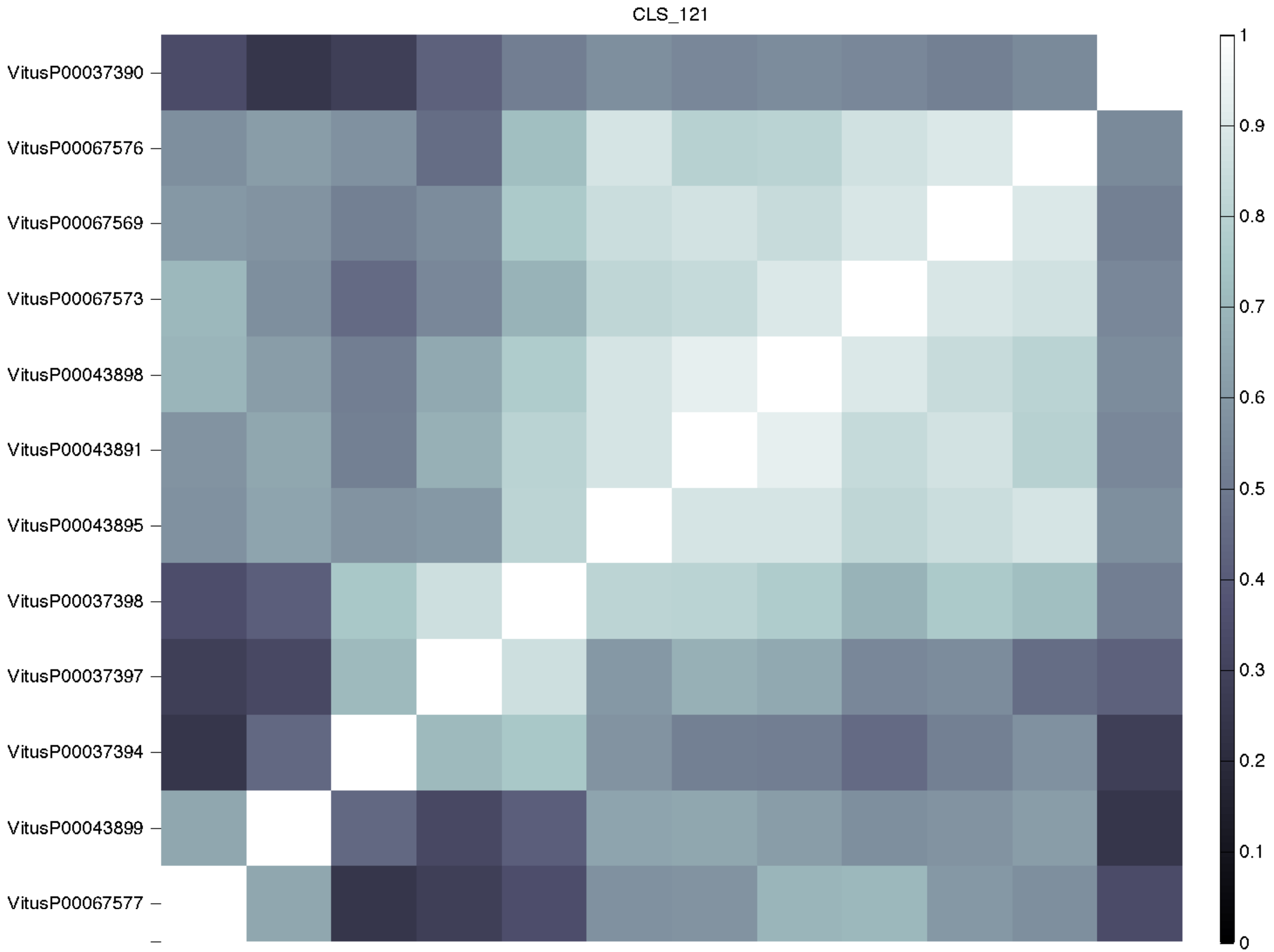

CLS_121
VitusP00037390
VitusP00067576
VitusP00067569
VitusP00067573
VitusP00043898
VitusP00043891
VitusP00043895
VitusP00037398
VitusP00037397
VitusP00037394
VitusP00043899
VitusP00067577
1
0.9
0.8
0.7
0.6
0.5
0.4
0.3
0.2
0.1
0

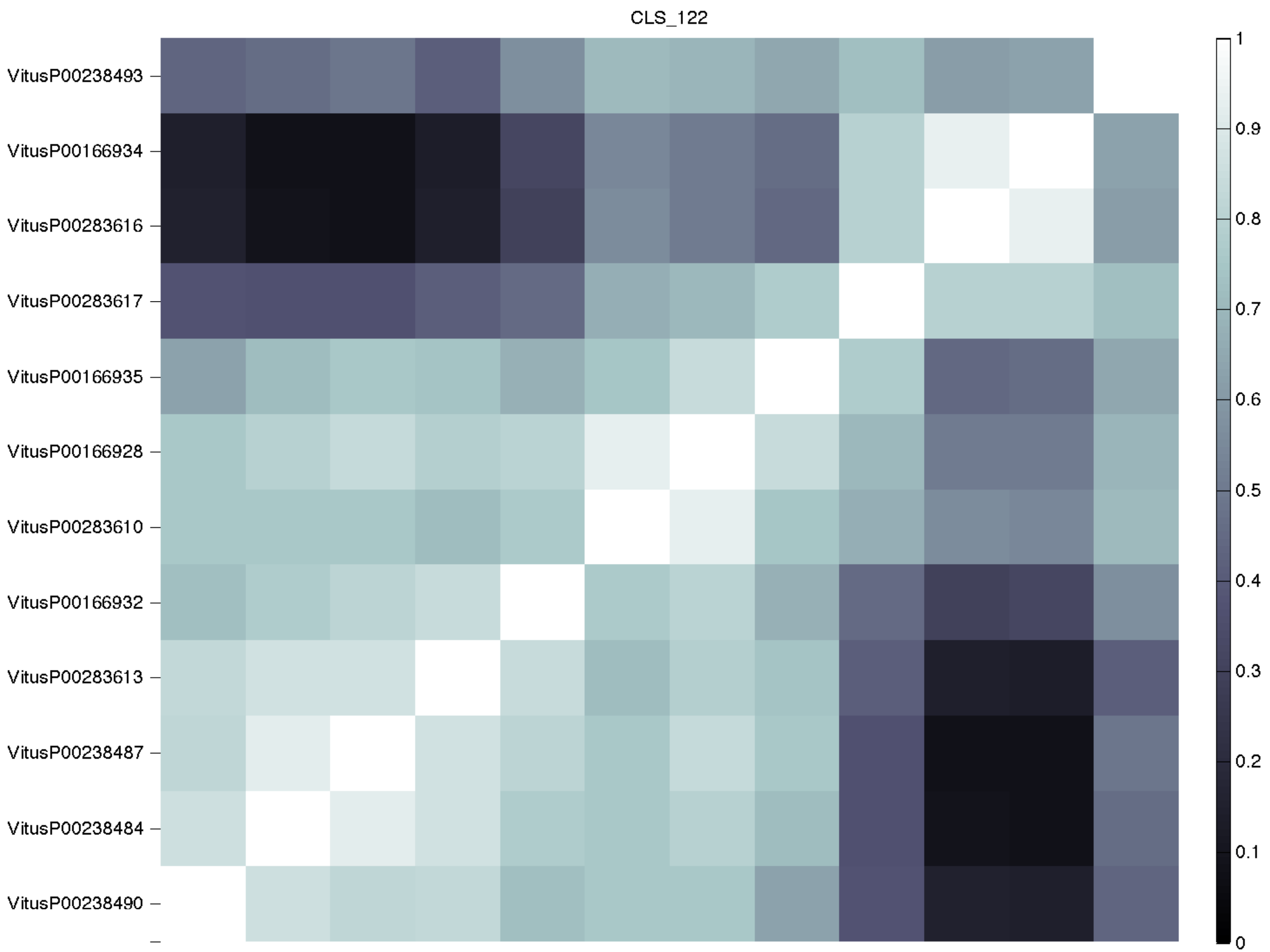
CLS_122
VitusP00238493
VitusP00166934
VitusP00283616
VitusP00283617
VitusP00166935
VitusP00166928
VitusP00283610
VitusP00166932
VitusP00283613
VitusP00238487
VitusP00238484
VitusP00238490
1
0.9
0.8
0.7
0.6
0.5
0.4
0.3
0.2
0.1
0

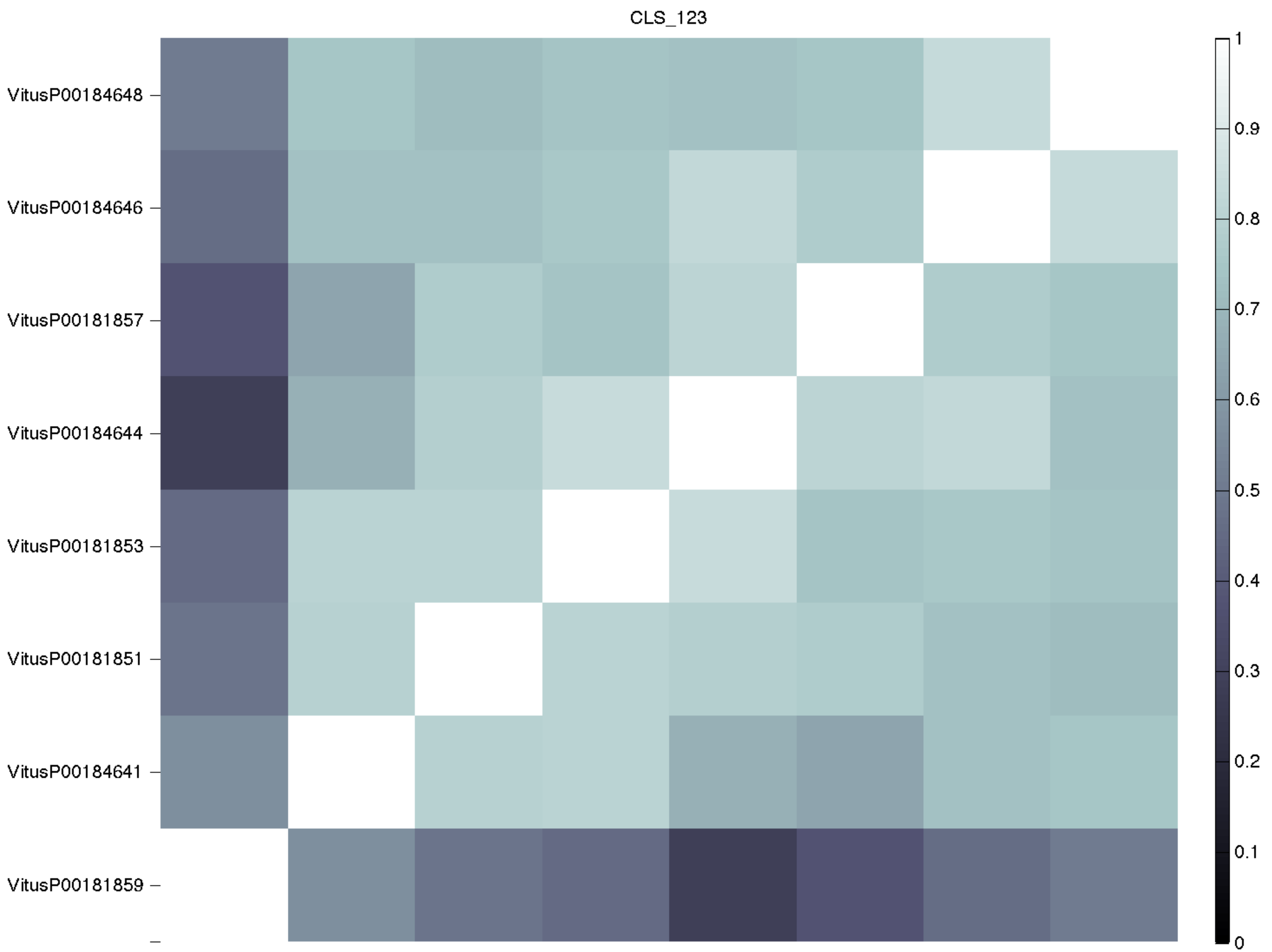

CLS_123
VitusP00184648
VitusP00184646
VitusP00181857
VitusP00184644
VitusP00181853
VitusP00181851
VitusP00184641
VitusP00181859
1
0.9
0.8
0.7
0.6
0.5
0.4
0.3
0.2
0.1
0

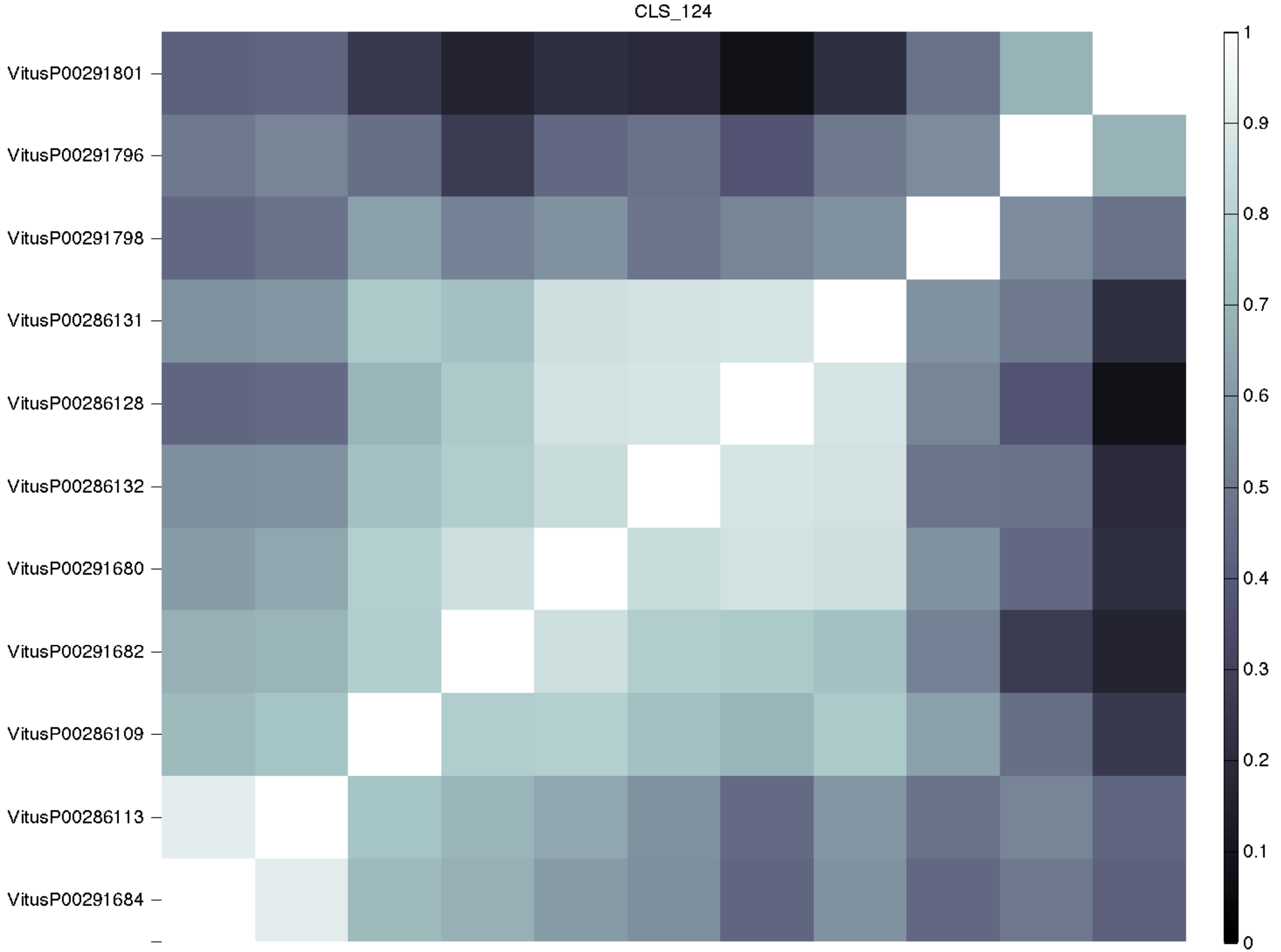

CLS_124
VitusP00291801
VitusP00291796
VitusP00291798
VitusP00286131
VitusP00286128
VitusP00286132
VitusP00291680
VitusP00291682
VitusP00286109
VitusP00286113
VitusP00291684
1
0.9
0.8
0.7
0.6
0.5
0.4
0.3
0.2
0.1
0

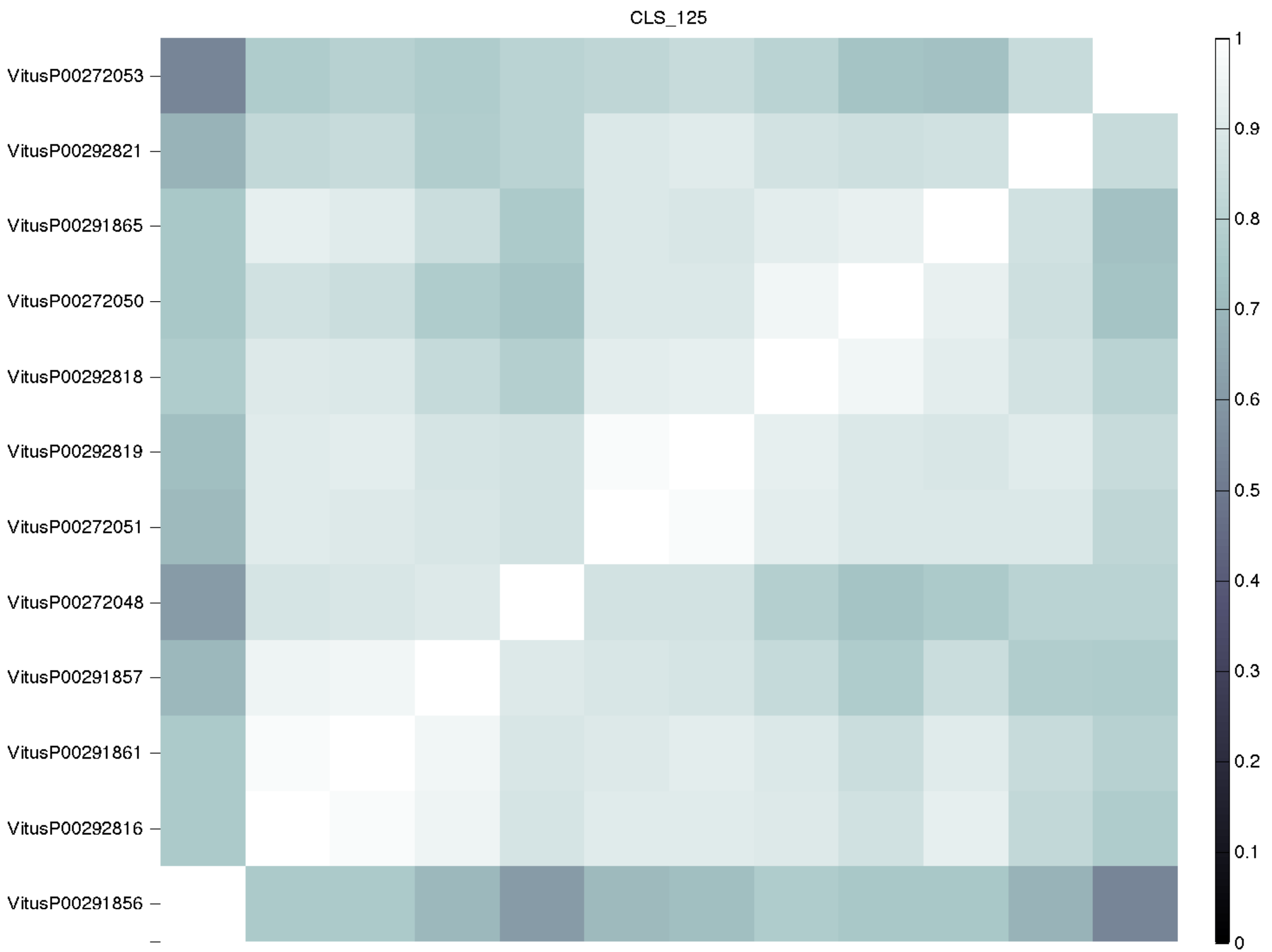
CLS_125
VitusP00272053
VitusP00292821
VitusP00291865
VitusP00272050
VitusP00292818
VitusP00292819
VitusP00272051
VitusP00272048
VitusP00291857
VitusP00291861
VitusP00292816
VitusP00291856
1
0.9
0.8
0.7
0.6
0.5
0.4
0.3
0.2
0.1
0

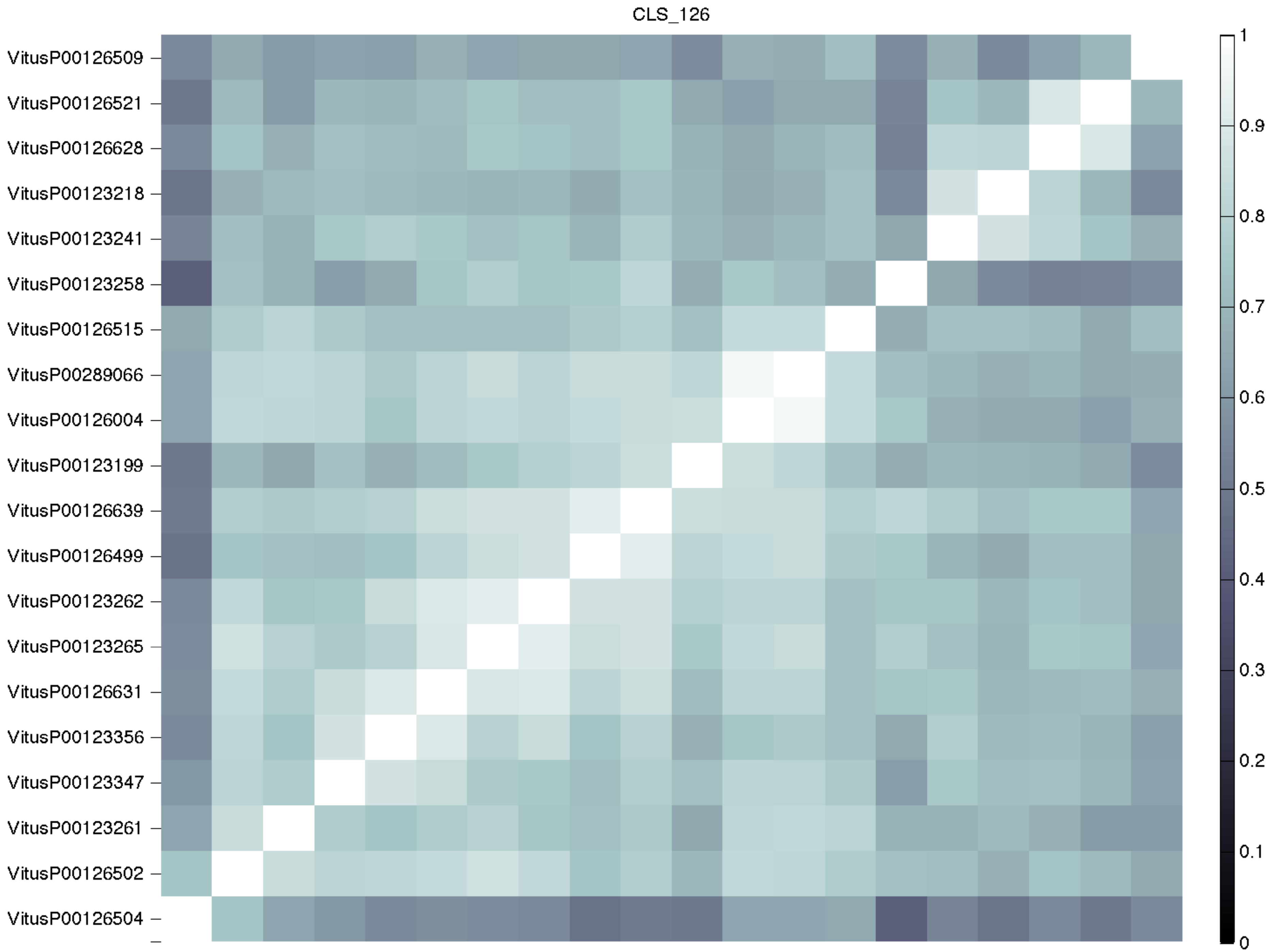
CLS_126
VitusP00126509
VitusP00126521
VitusP00126628
VitusP00123218
VitusP00123241
VitusP00123258
VitusP00126515
VitusP00289066
VitusP00126004
VitusP00123199
VitusP00126639
VitusP00126499
VitusP00123262
VitusP00123265
VitusP00126631
VitusP00123356
VitusP00123347
VitusP00123261
VitusP00126502
VitusP00126504
1
0.9
0.8
0.7
0.6
0.5
0.4
0.3
0.2
0.1
0

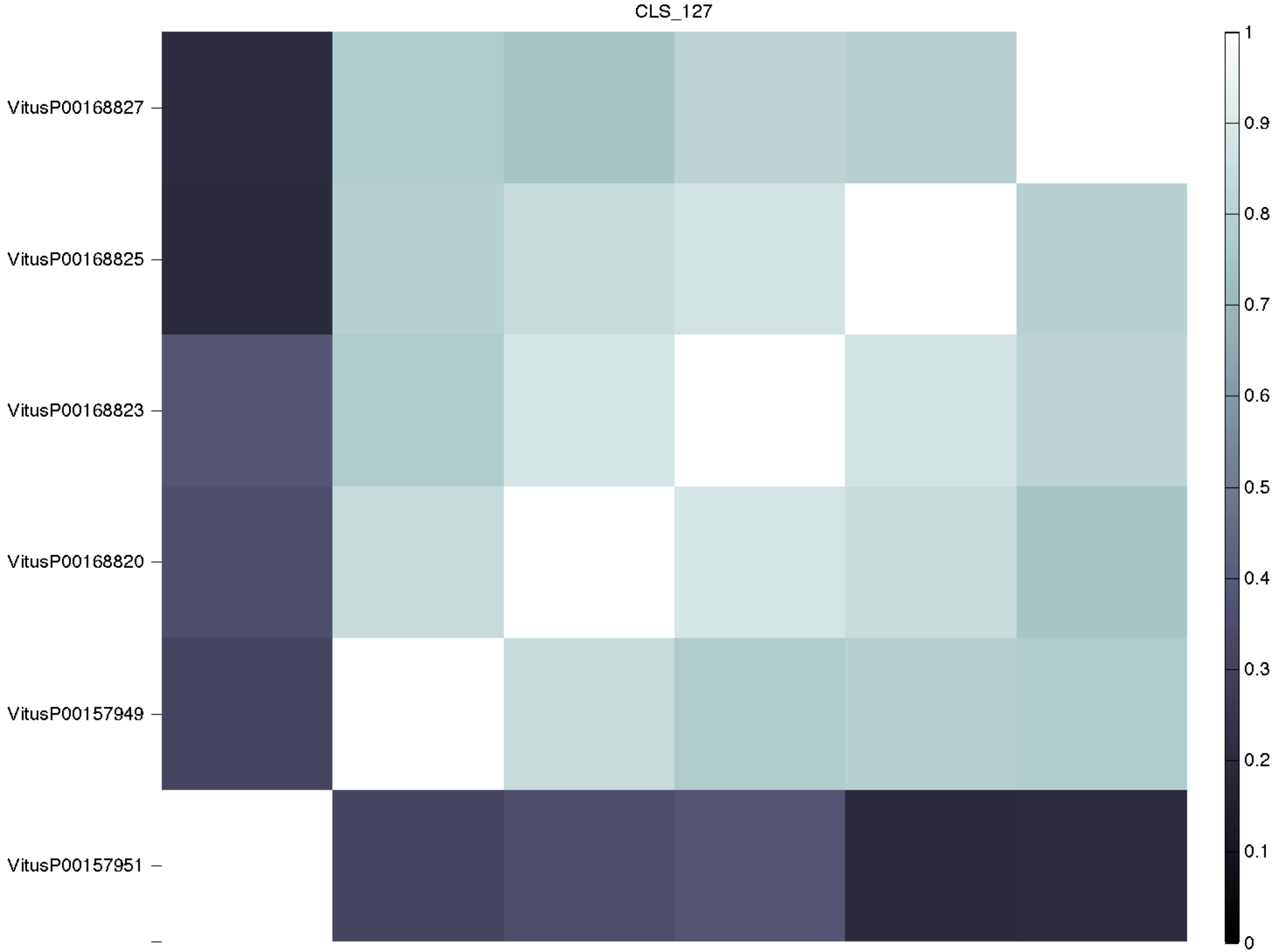

CLS_127
VitusP00168827
VitusP00168825
VitusP00168823
VitusP00168820
VitusP00157949
VitusP00157951
1
0.9
0.8
0.7
0.6
0.5
0.4
0.3
0.2
0.1
0

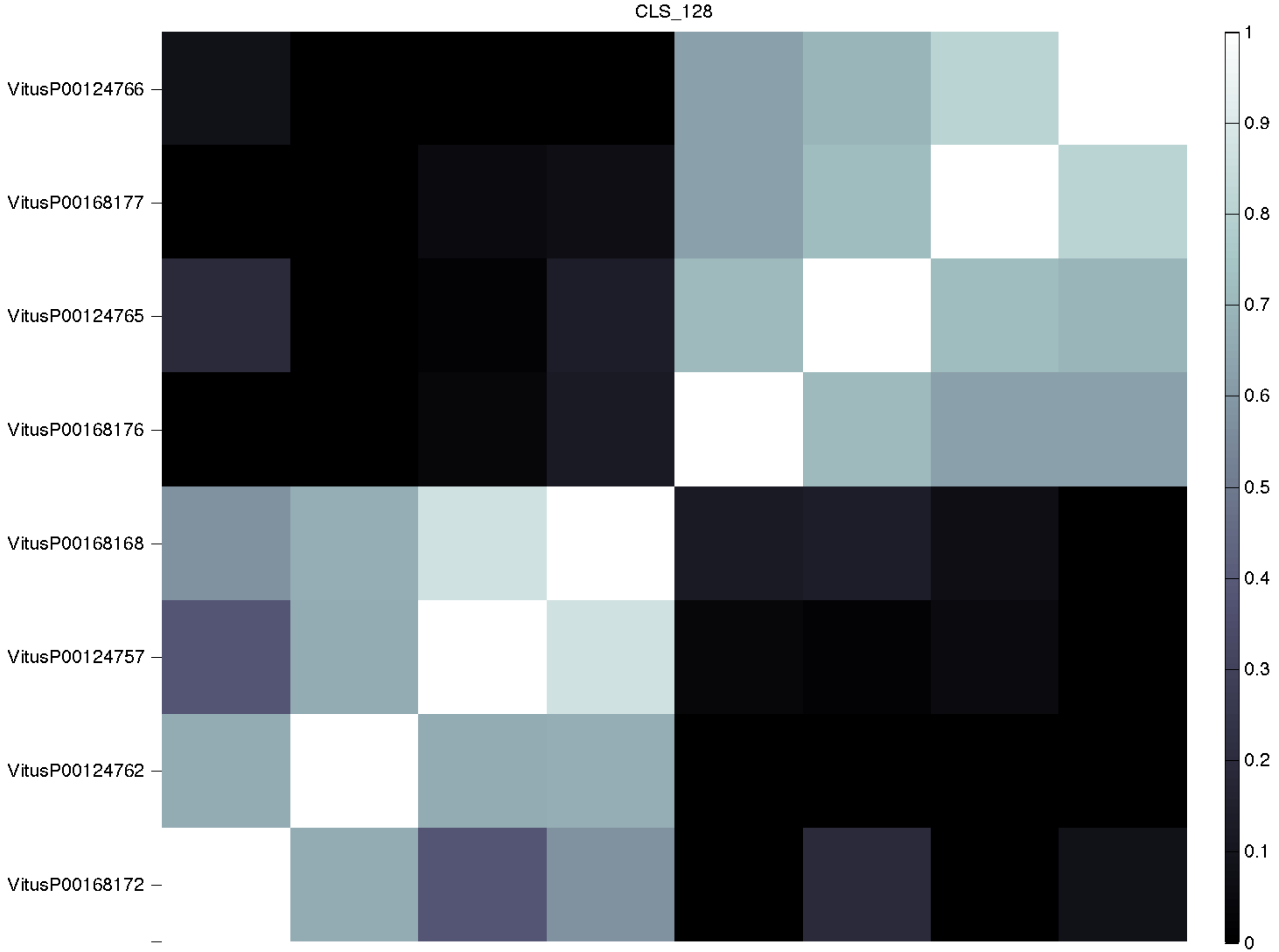
CLS_128
VitusP00124766
VitusP00168177
VitusP00124765
VitusP00168176
VitusP00168168
VitusP00124757
VitusP00124762
VitusP00168172
1
0.9
0.8
0.7
0.6
0.5
0.4
0.3
0.2
0.1
0

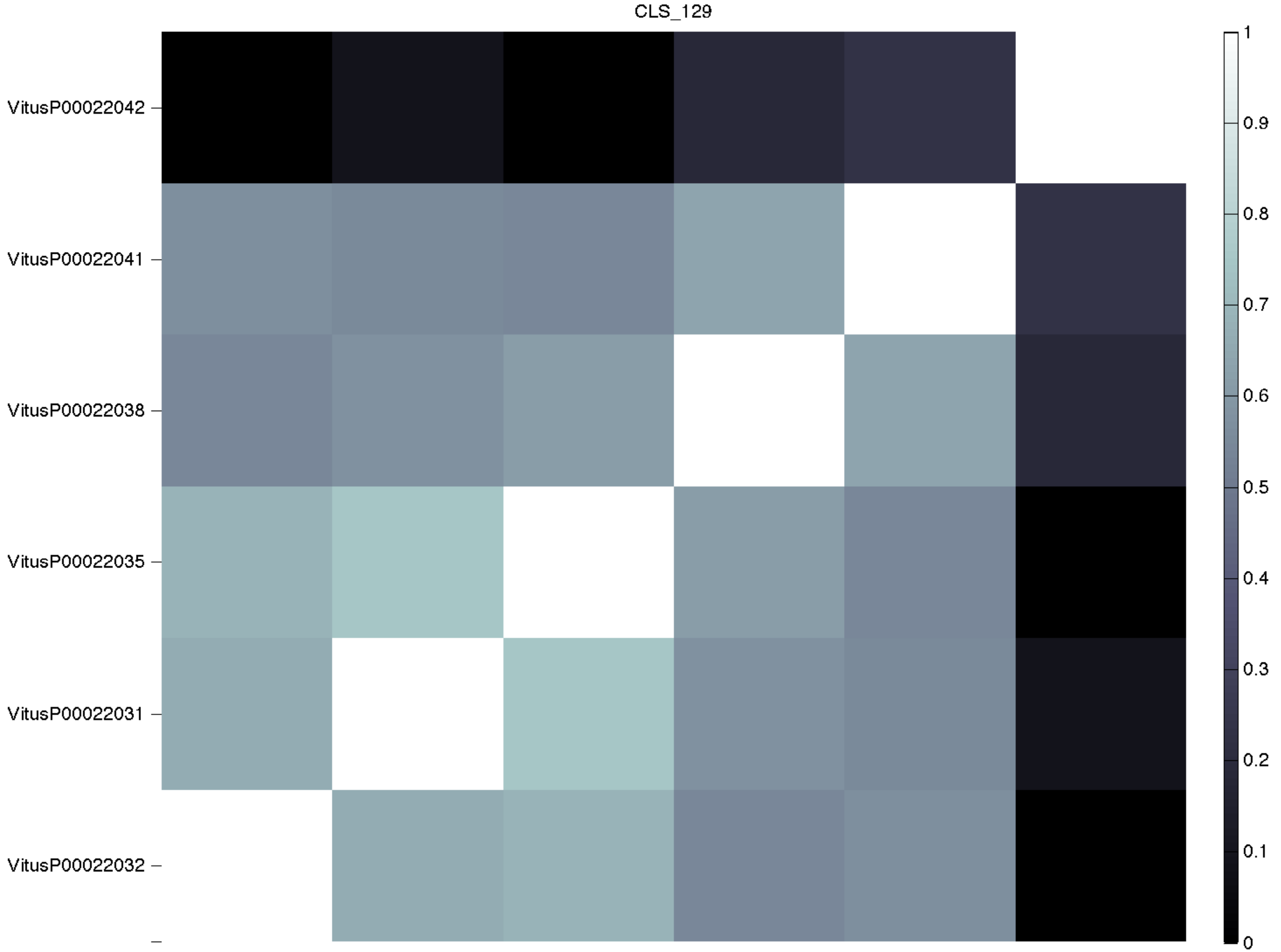
CLS_129
VitusP00022042
VitusP00022041
VitusP00022038
VitusP00022035
VitusP00022031
VitusP00022032
1
0.9
0.8
0.7
0.6
0.5
0.4
0.3
0.2
0.1
0

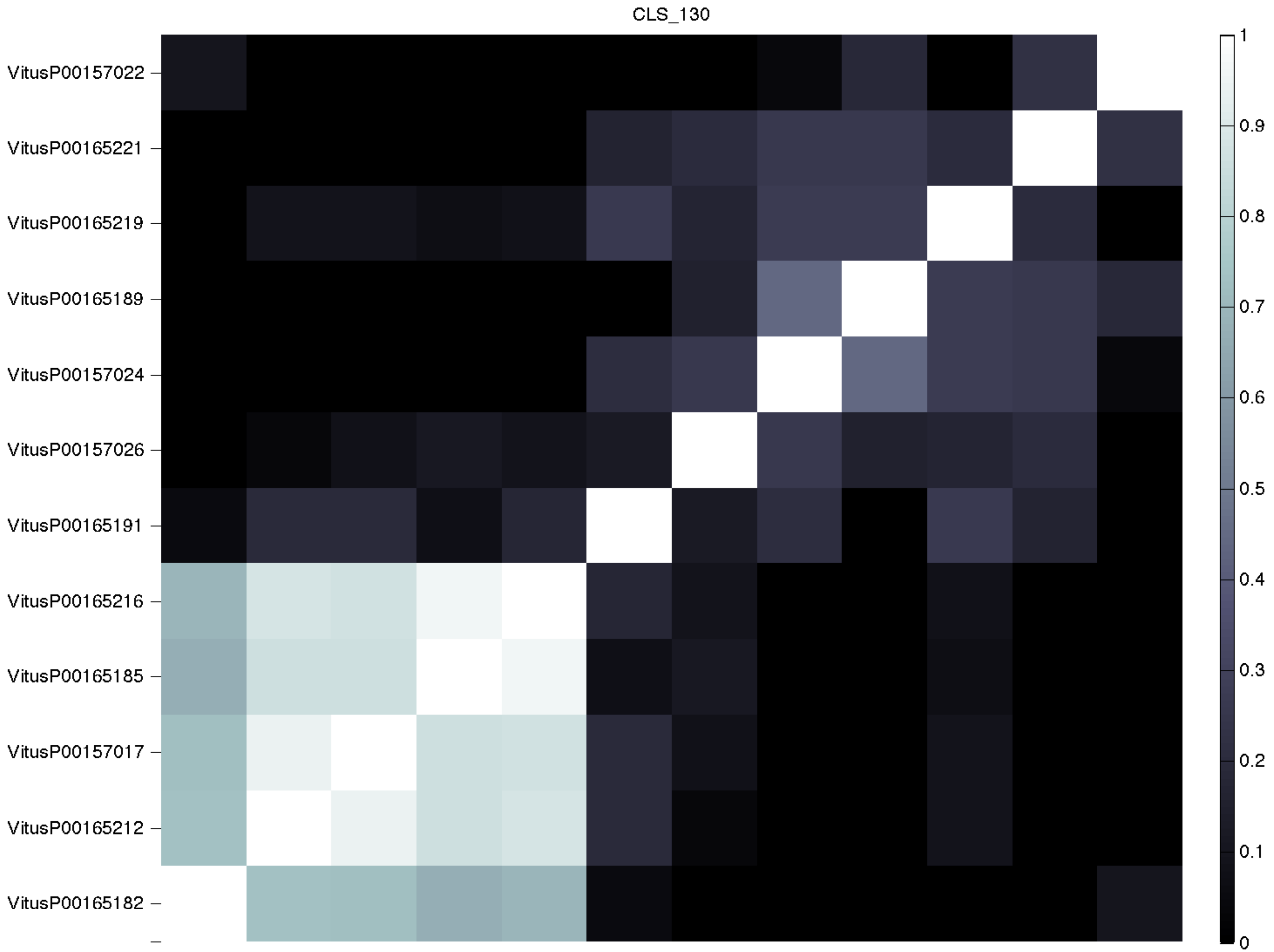
CLS_130
VitusP00157022
VitusP00165221
VitusP00165219
VitusP00165189
VitusP00157024
VitusP00157026
VitusP00165191
VitusP00165216
VitusP00165185
VitusP00157017
VitusP00165212
VitusP00165182
1
0.9
0.8
0.7
0.6
0.5
0.4
0.3
0.2
0.1
0

CLS_131

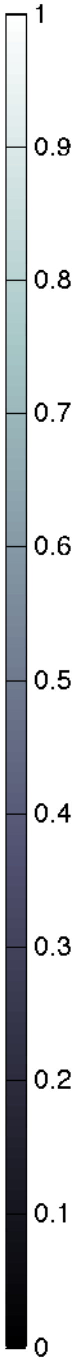

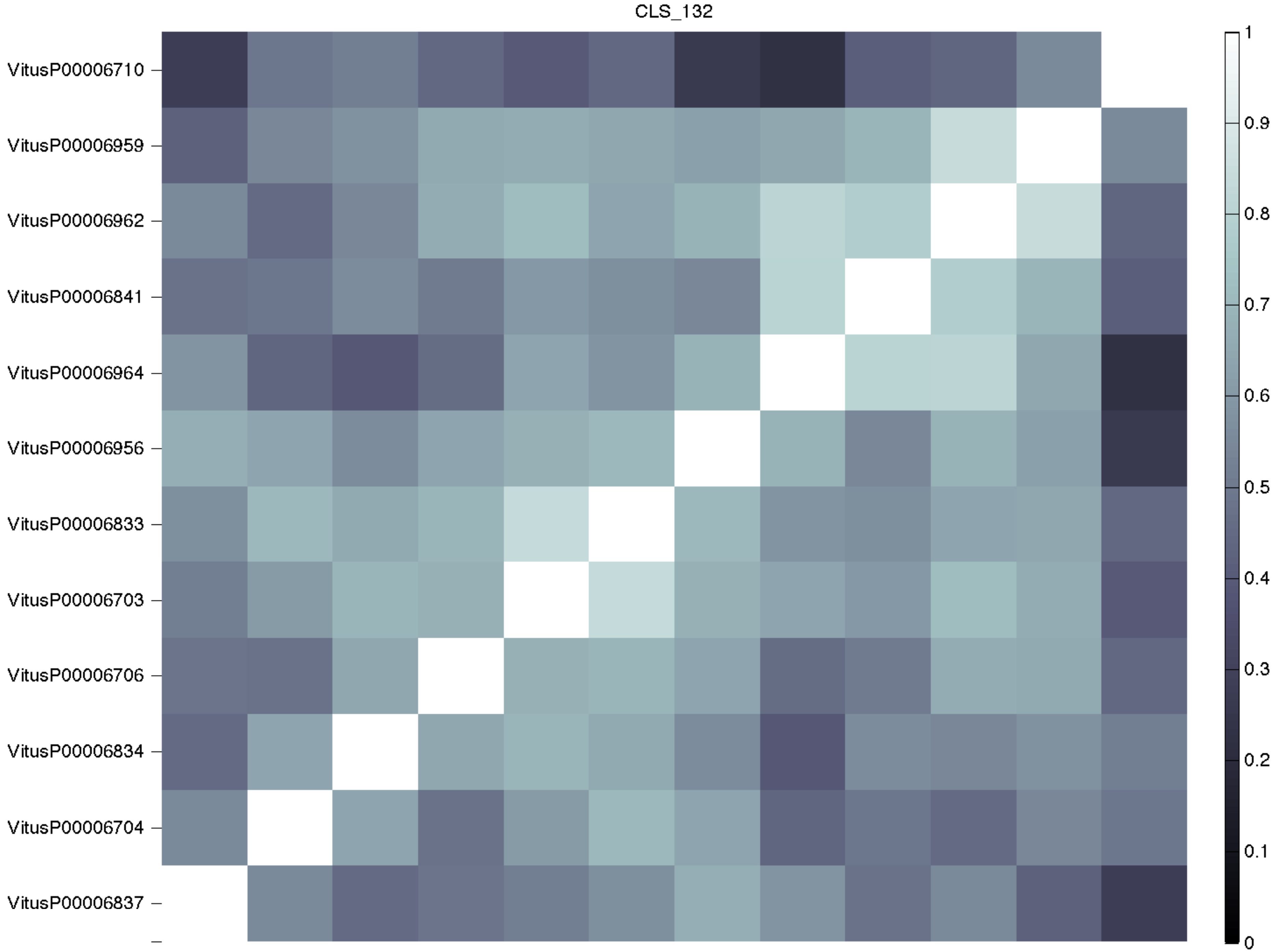
CLS_132
VitusP00006710
VitusP00006959
VitusP00006962
VitusP00006841
VitusP00006964
VitusP00006956
VitusP00006833
VitusP00006703
VitusP00006706
VitusP00006834
VitusP00006704
VitusP00006837
1
0.9
0.8
0.7
0.6
0.5
0.4
0.3
0.2
0.1
0

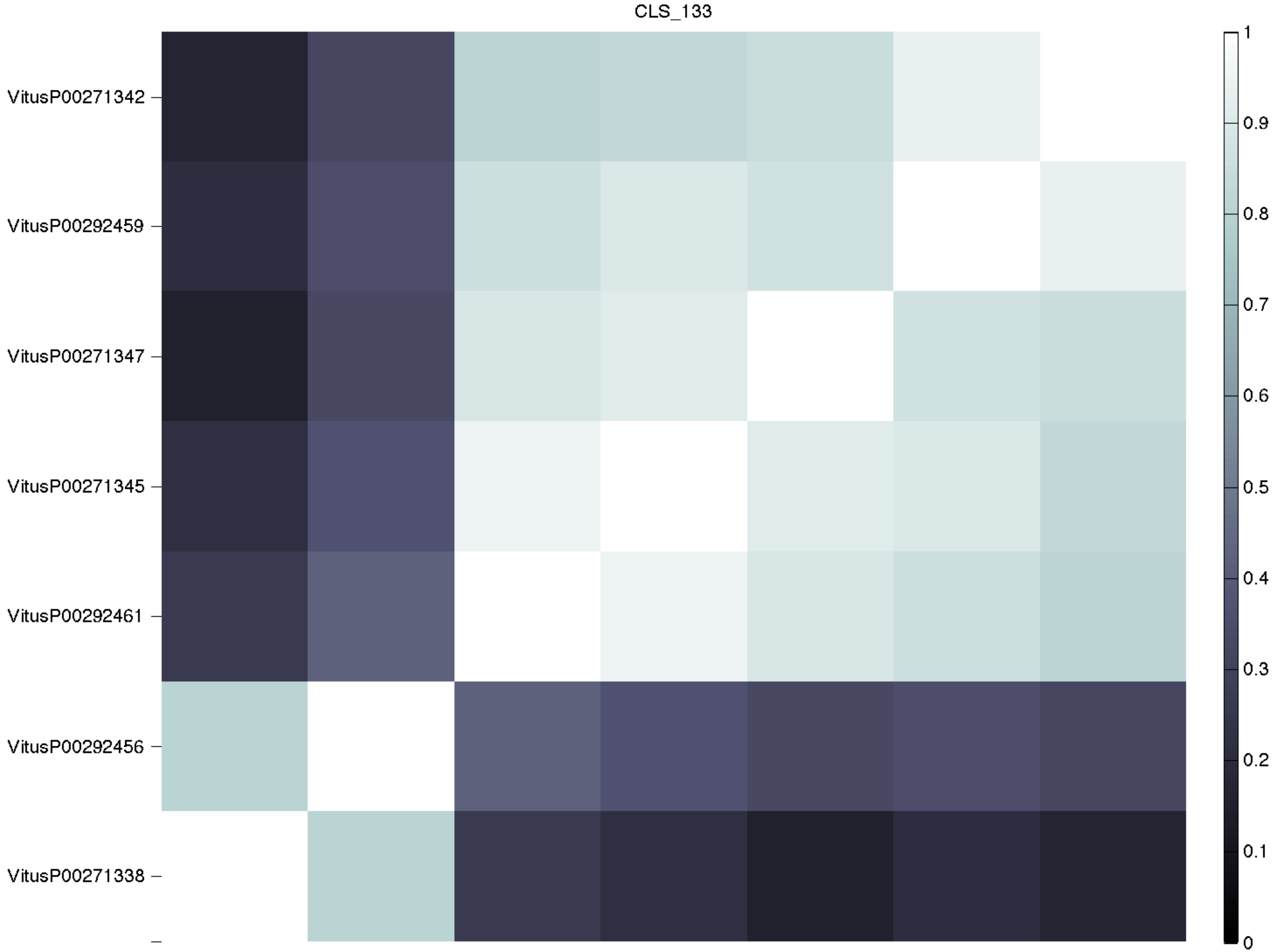
CLS_133
VitusP00271342
VitusP00292459
VitusP00271347
VitusP00271345
VitusP00292461
VitusP00292456
VitusP00271338
1
0.9
0.8
0.7
0.6
0.5
0.4
0.3
0.2
0.1
0

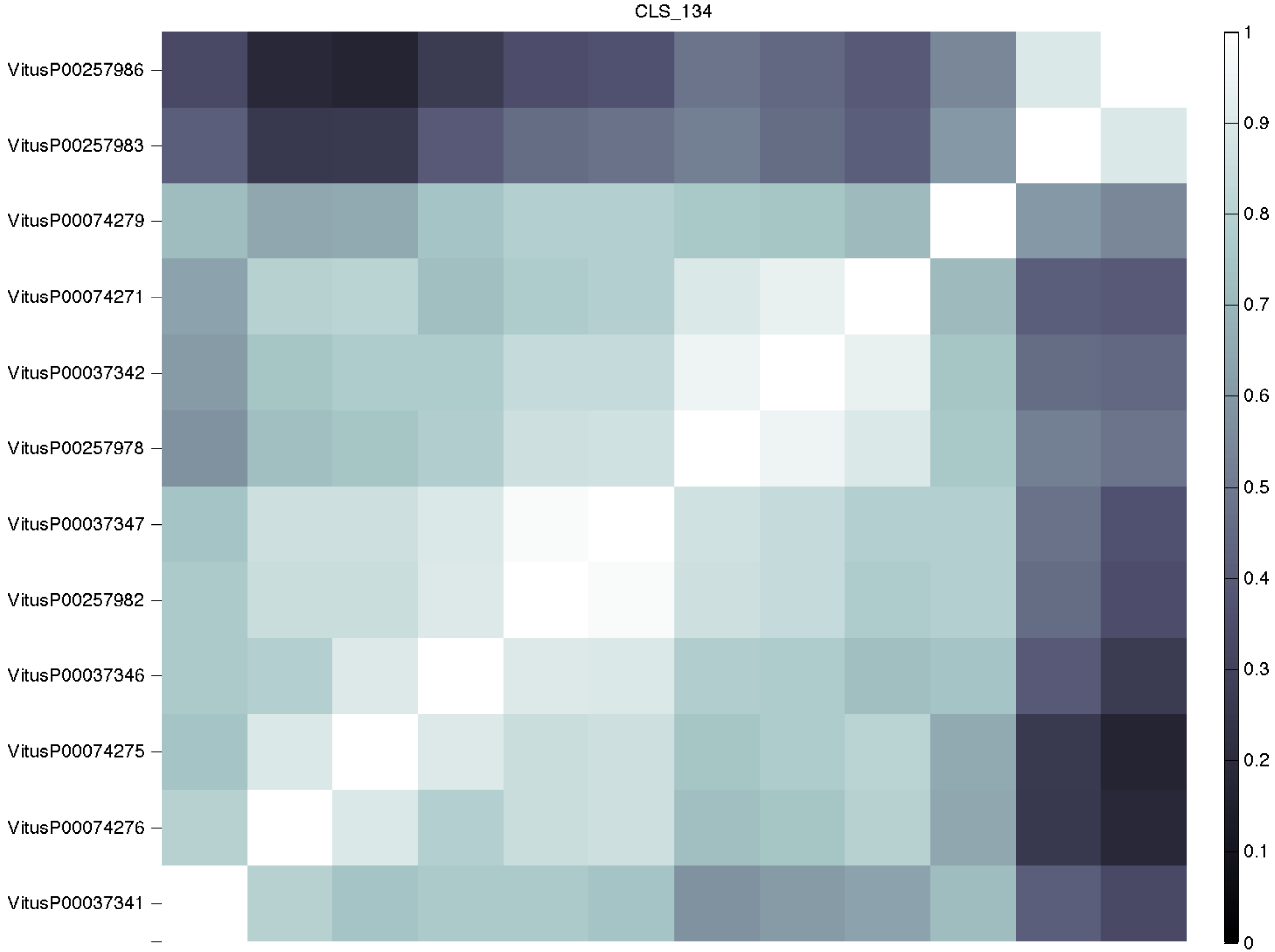

CLS_134
VitusP00257986
VitusP00257983
VitusP00074279
VitusP00074271
VitusP00037342
VitusP00257978
VitusP00037347
VitusP00257982
VitusP00037346
VitusP00074275
VitusP00074276
VitusP00037341
1
0.9
0.8
0.7
0.6
0.5
0.4
0.3
0.2
0.1
0

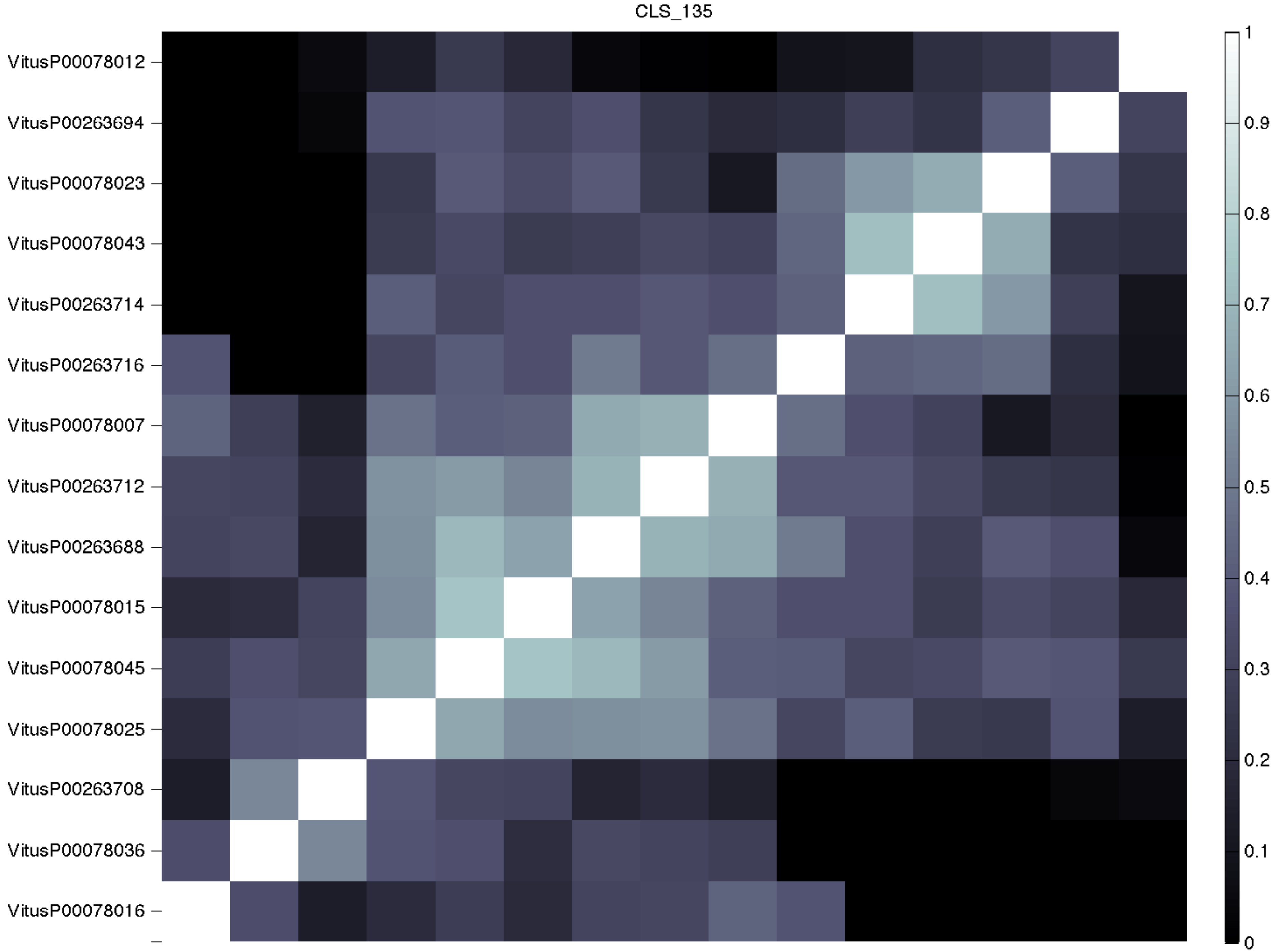

CLS_135
VitusP00078012
VitusP00263694
VitusP00078023
VitusP00078043
VitusP00263714
VitusP00263716
VitusP00078007
VitusP00263712
VitusP00263688
VitusP00078015
VitusP00078045
VitusP00078025
VitusP00263708
VitusP00078036
VitusP00078016
1
0.9
0.8
0.7
0.6
0.5
0.4
0.3
0.2
0.1
0

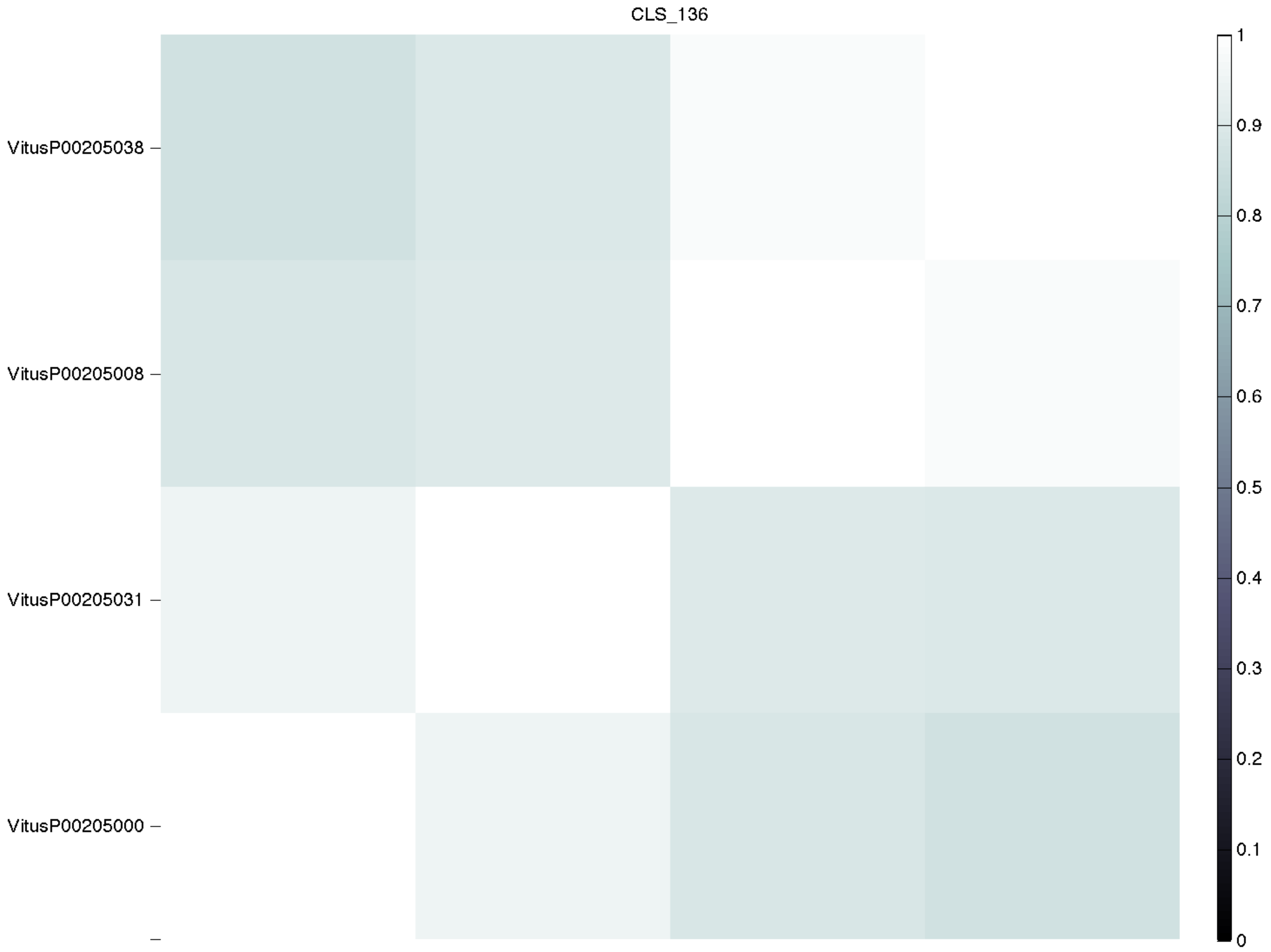
CLS_136
VitusP00205038
VitusP00205008
VitusP00205031
VitusP00205000
1
0.9
0.8
0.7
0.6
0.5
0.4
0.3
0.2
0.1
0

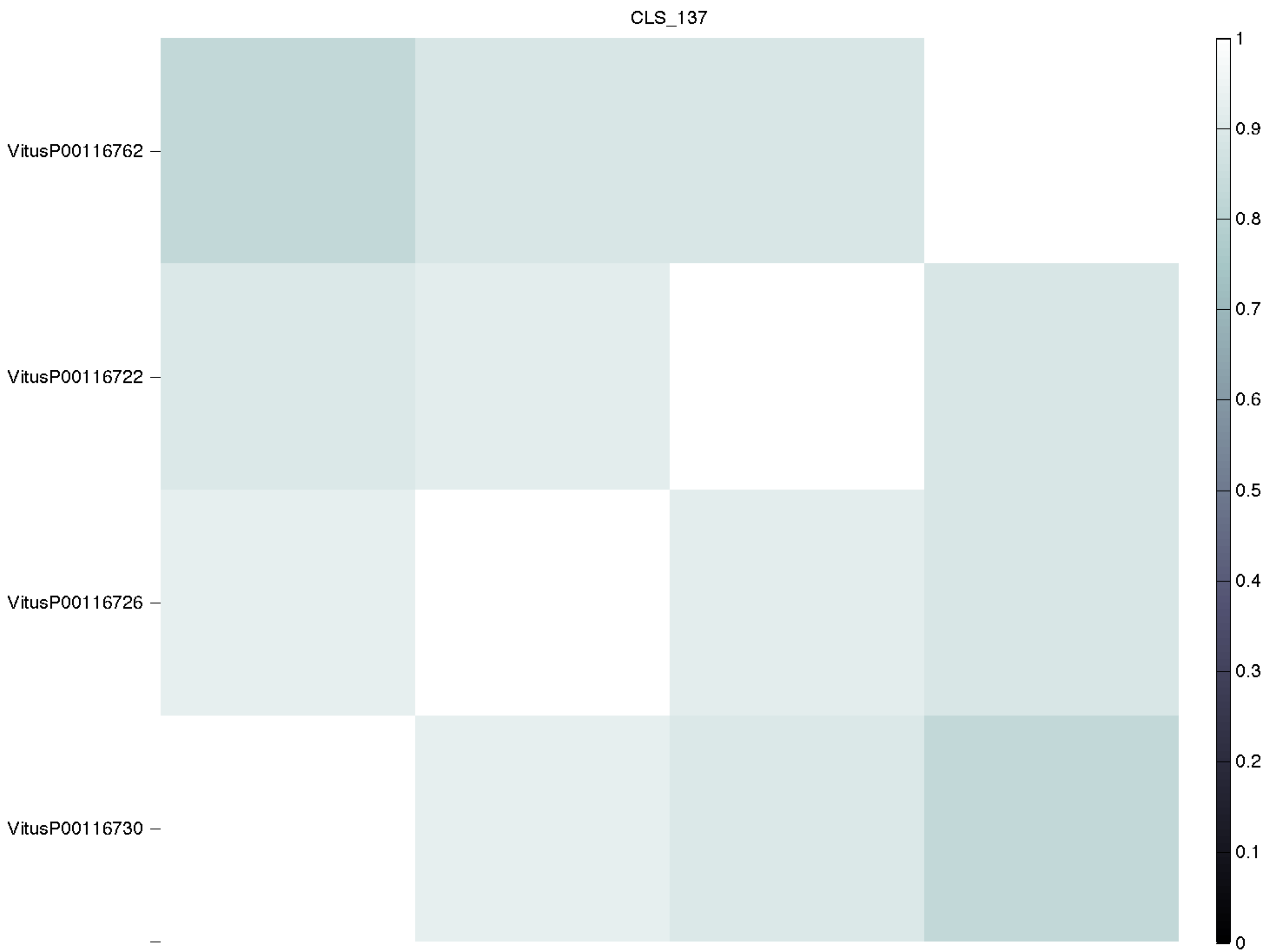
CLS_137
VitusP00116762
VitusP00116722
VitusP00116726
VitusP00116730
1
0.9
0.8
0.7
0.6
0.5
0.4
0.3
0.2
0.1
0

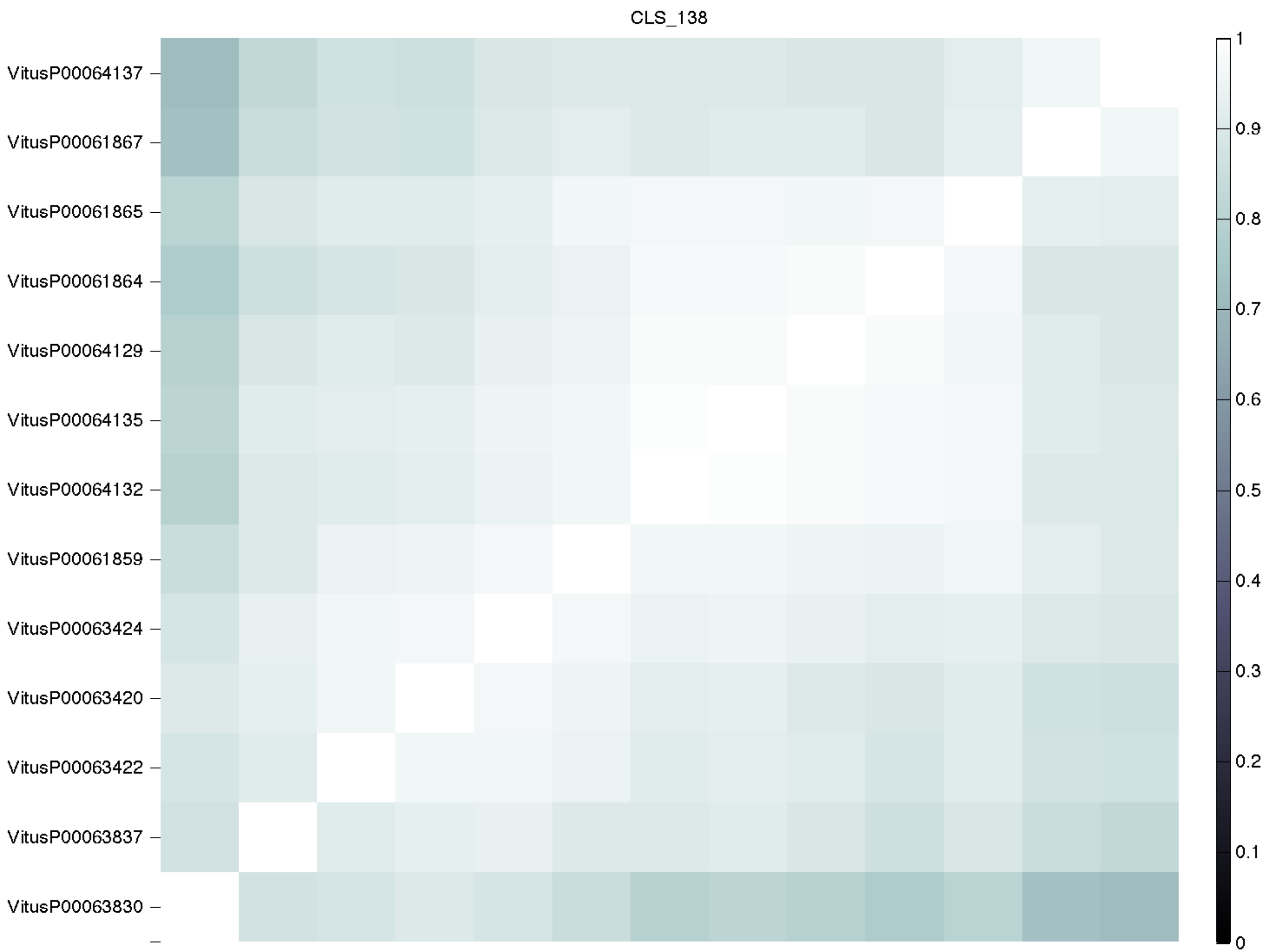
CLS_138
VitusP00064137
VitusP00061867
VitusP00061865
VitusP00061864
VitusP00064129
VitusP00064135
VitusP00064132
VitusP00061859
VitusP00063424
VitusP00063420
VitusP00063422
VitusP00063837
VitusP00063830
1
0.9
0.8
0.7
0.6
0.5
0.4
0.3
0.2
0.1
0

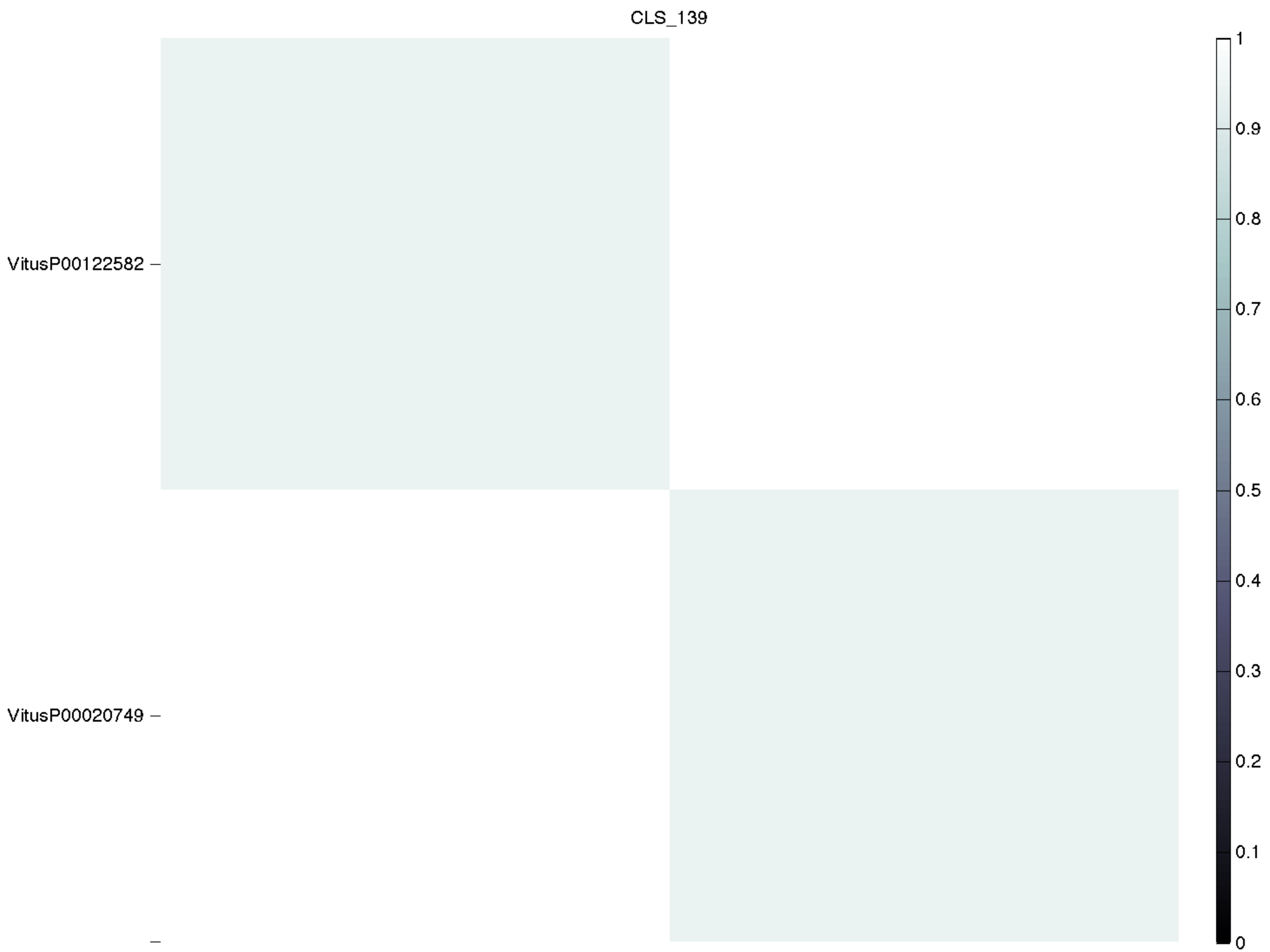
CLS_139
VitusP00122582
VitusP00020749
1
0.9
0.8
0.7
0.6
0.5
0.4
0.3
0.2
0.1
0

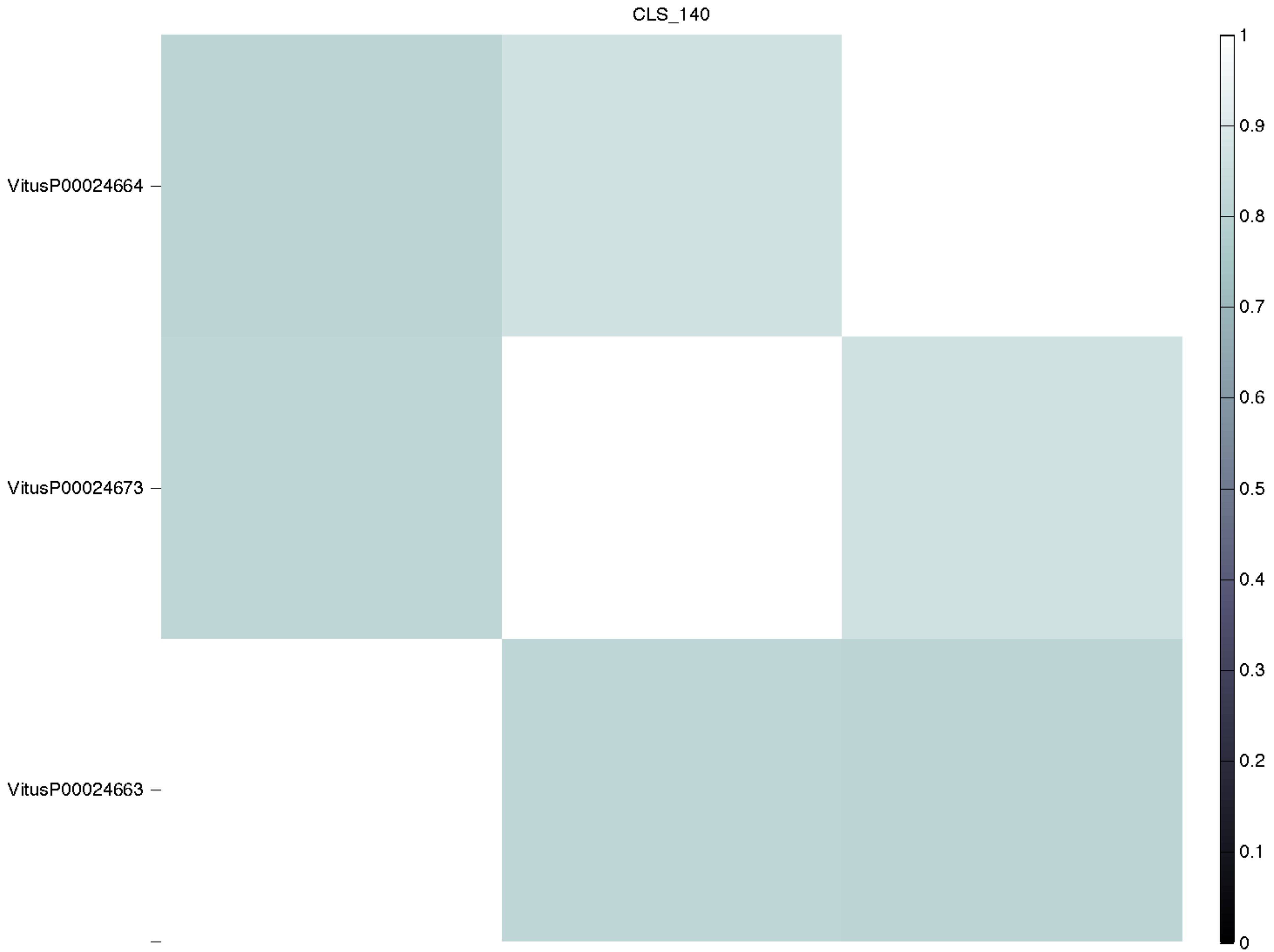
CLS_140
VitusP00024664
VitusP00024673
VitusP00024663
1
0.9
0.8
0.7
0.6
0.5
0.4
0.3
0.2
0.1
0

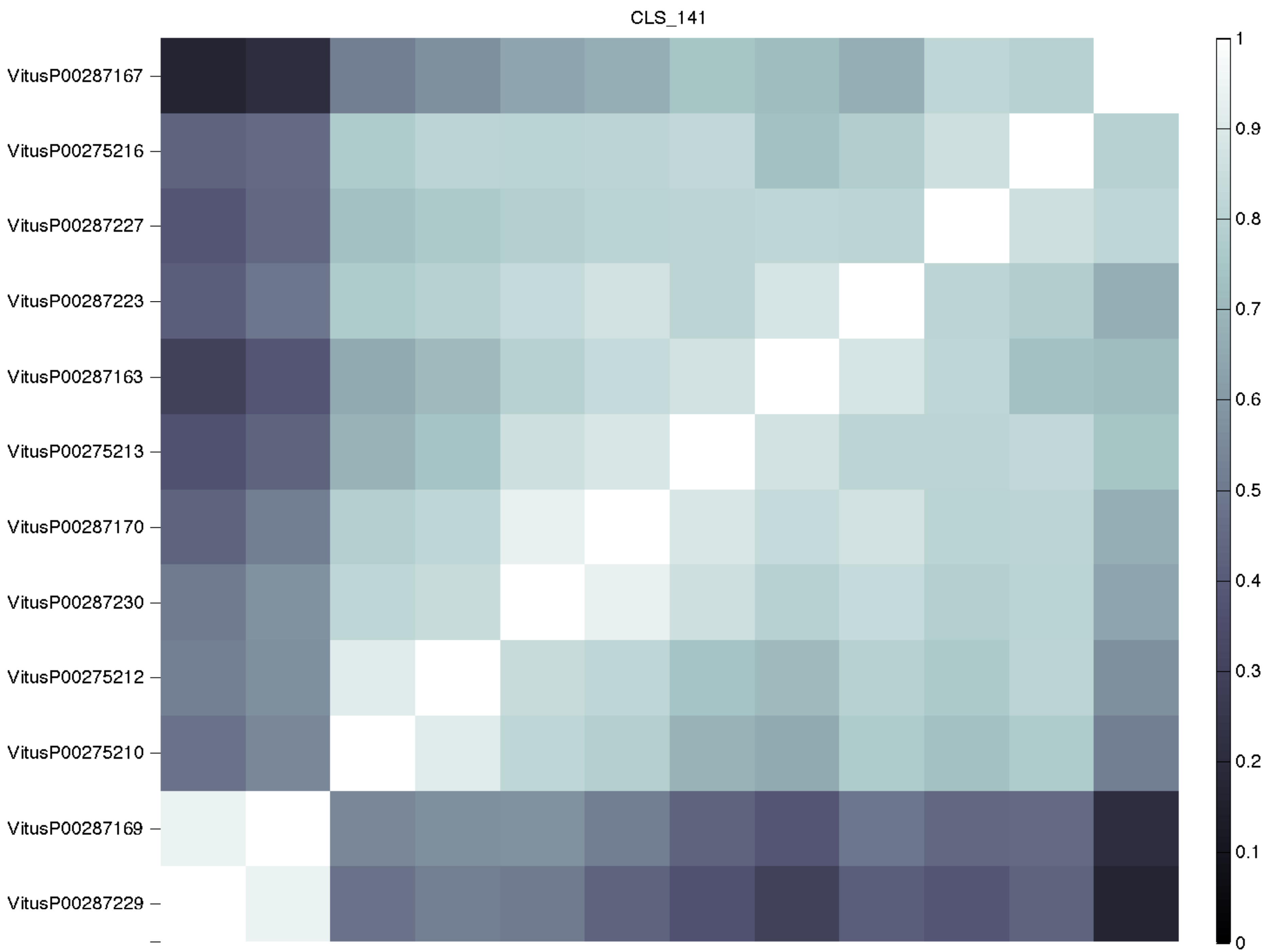

CLS_141
VitusP00287167
VitusP00275216
VitusP00287227
VitusP00287223
VitusP00287163
VitusP00275213
VitusP00287170
VitusP00287230
VitusP00275212
VitusP00275210
VitusP00287169
VitusP00287229
1
0.9
0.8
0.7
0.6
0.5
0.4
0.3
0.2
0.1
0

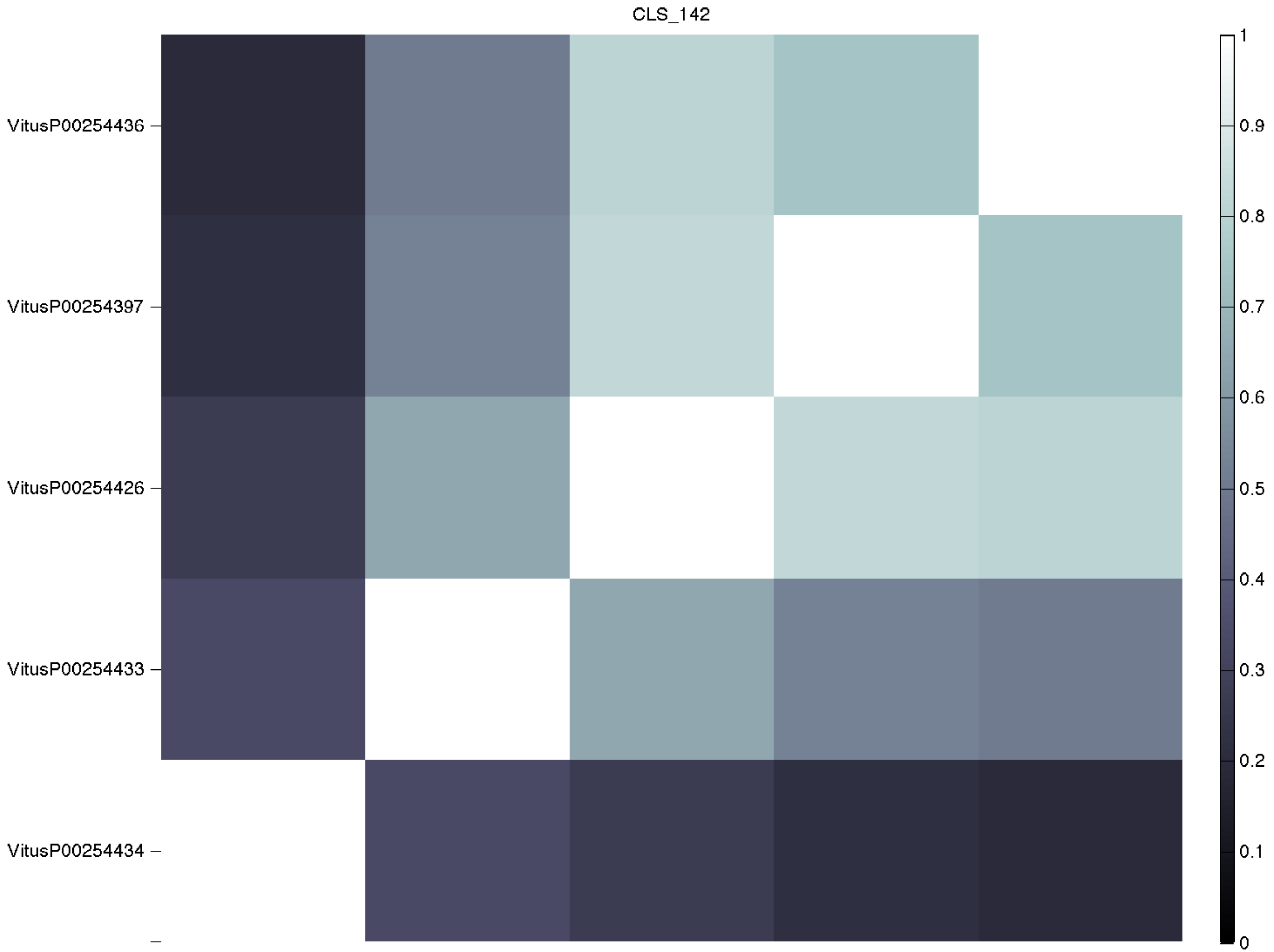

CLS_142
VitusP00254436
VitusP00254397
VitusP00254426
VitusP00254433
VitusP00254434
1
0.9
0.8
0.7
0.6
0.5
0.4
0.3
0.2
0.1
0

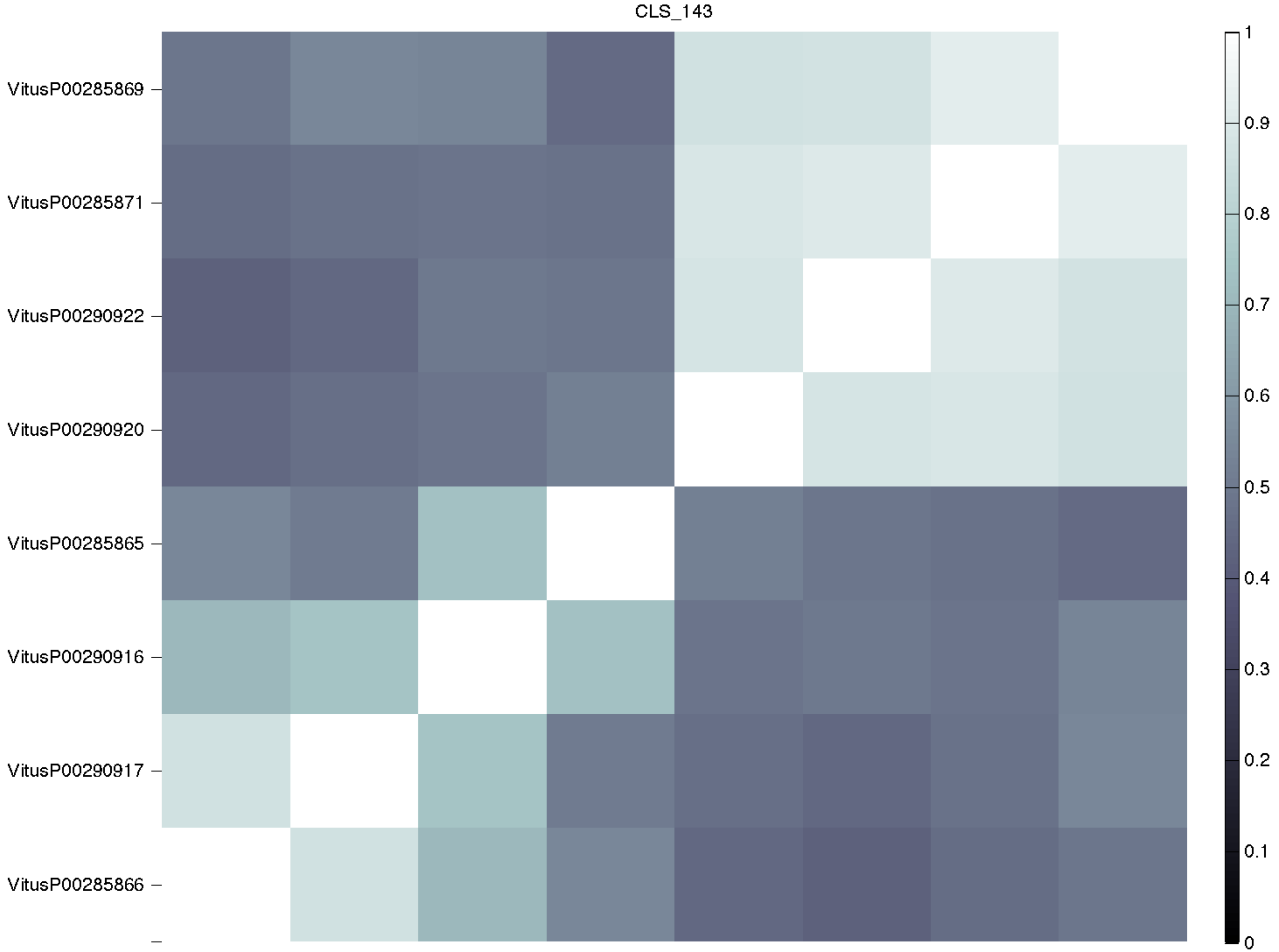

CLS_143
VitusP00285869
VitusP00285871
VitusP00290922
VitusP00290920
VitusP00285865
VitusP00290916
VitusP00290917
VitusP00285866
1
0.9
0.8
0.7
0.6
0.5
0.4
0.3
0.2
0.1
0

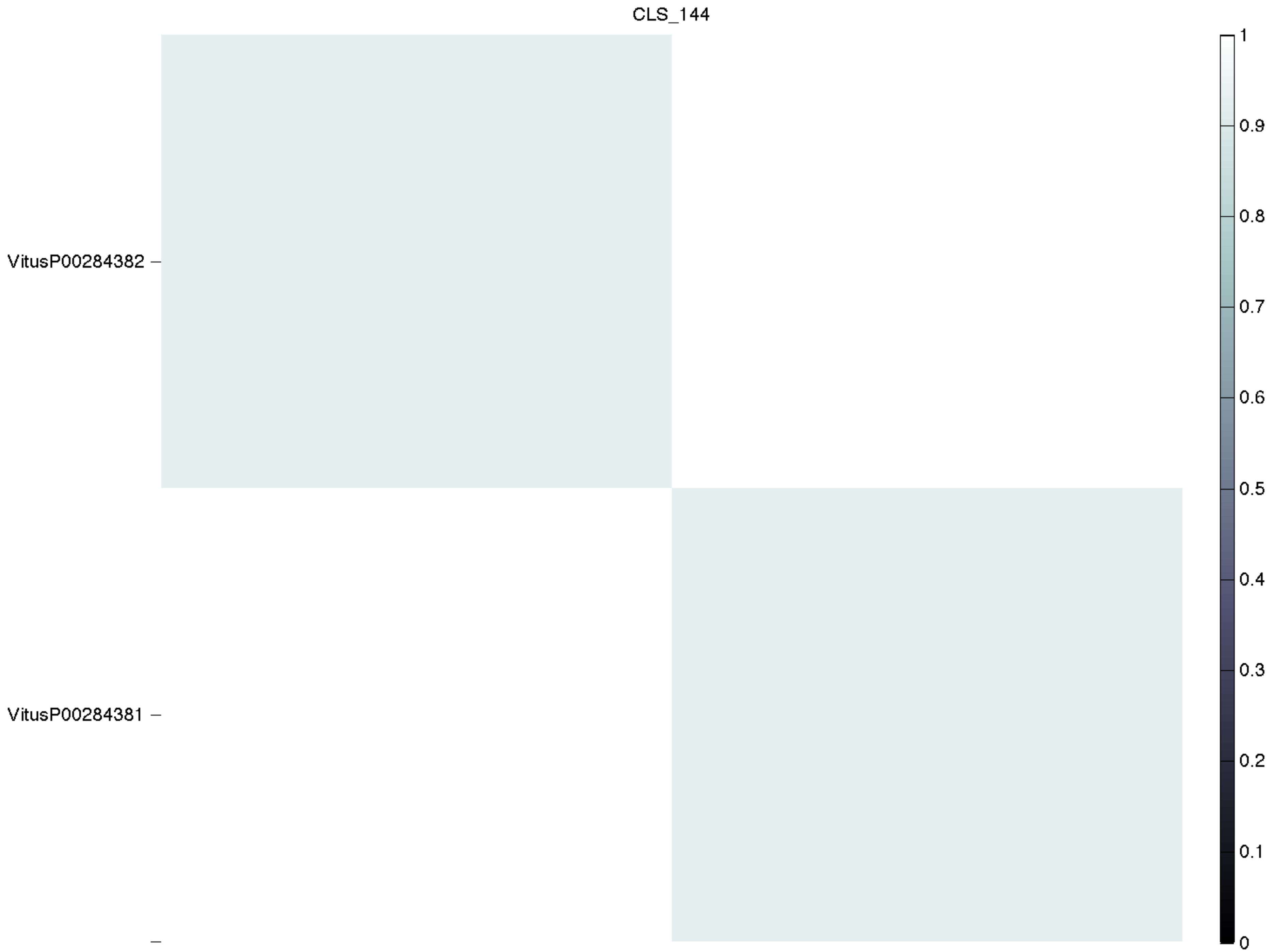
CLS_144
VitusP00284382
VitusP00284381
1
0.9
0.8
0.7
0.6
0.5
0.4
0.3
0.2
0.1
0

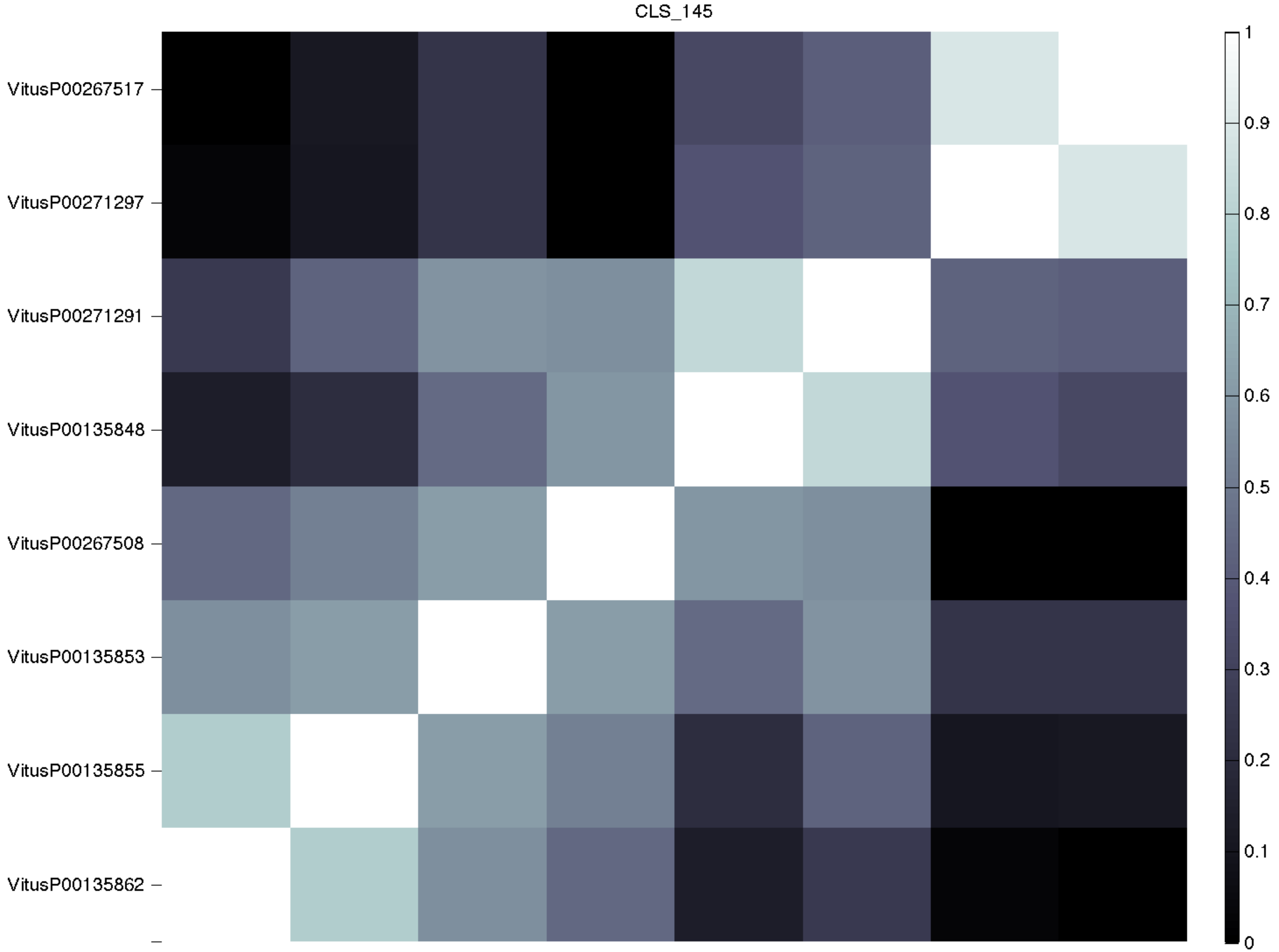
CLS_145
VitusP00267517
VitusP00271297
VitusP00271291
VitusP00135848
VitusP00267508
VitusP00135853
VitusP00135855
VitusP00135862
1
0.9
0.8
0.7
0.6
0.5
0.4
0.3
0.2
0.1
0

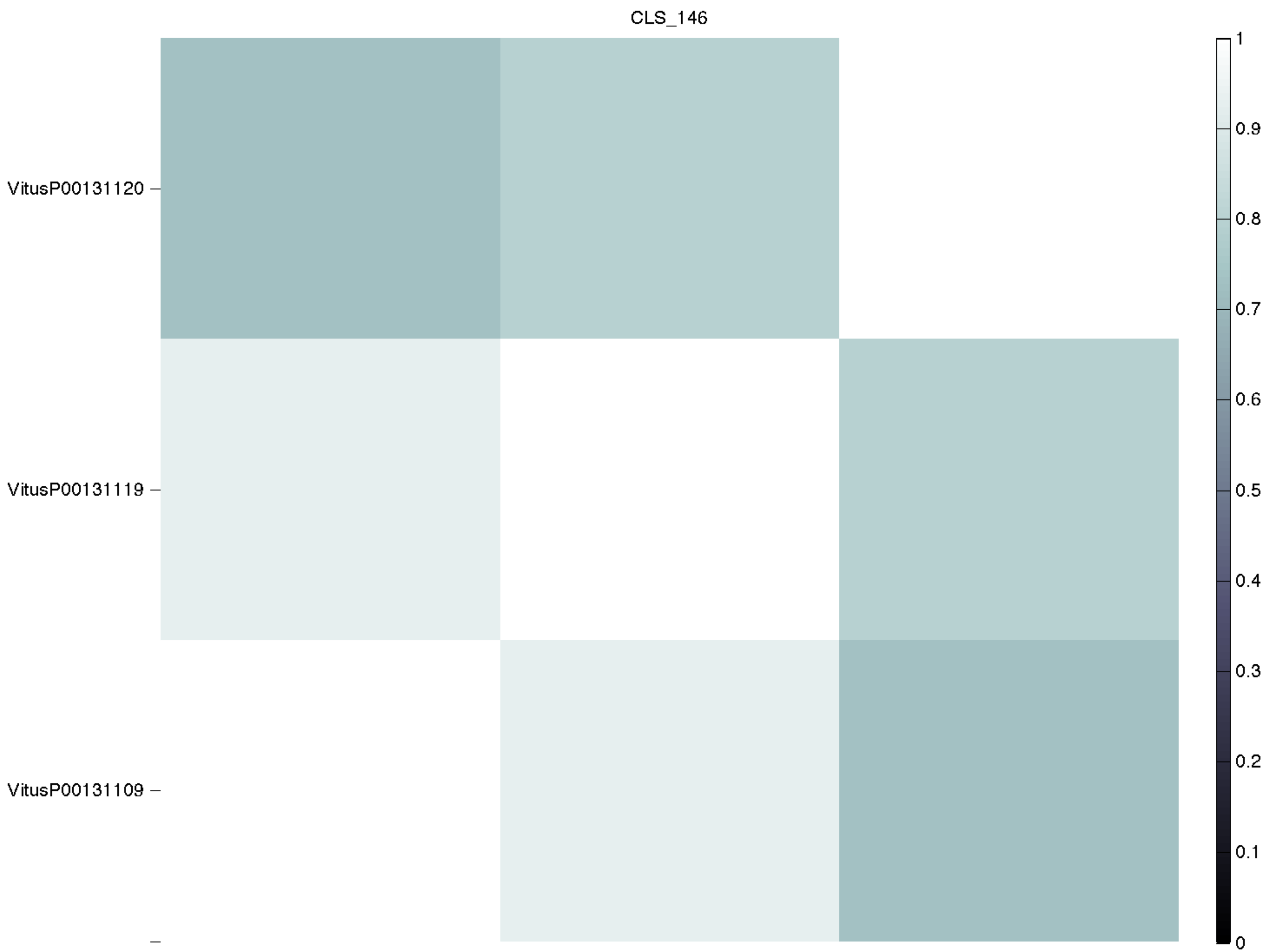

CLS_146
VitusP00131120
VitusP00131119
VitusP00131109
1
0.9
0.8
0.7
0.6
0.5
0.4
0.3
0.2
0.1
0

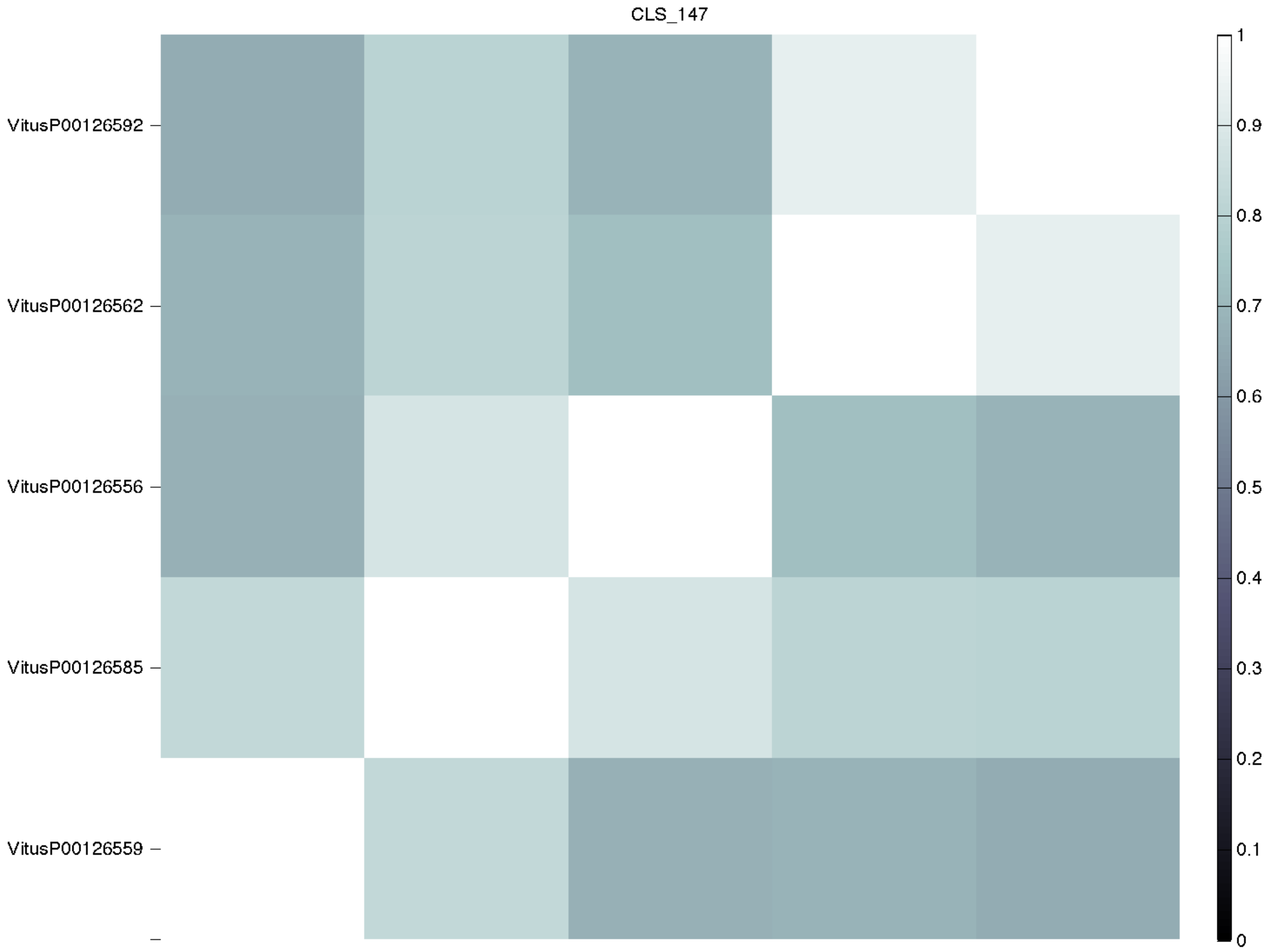
CLS_147
VitusP00126592
VitusP00126562
VitusP00126556
VitusP00126585
VitusP00126559
1
0.9
0.8
0.7
0.6
0.5
0.4
0.3
0.2
0.1
0

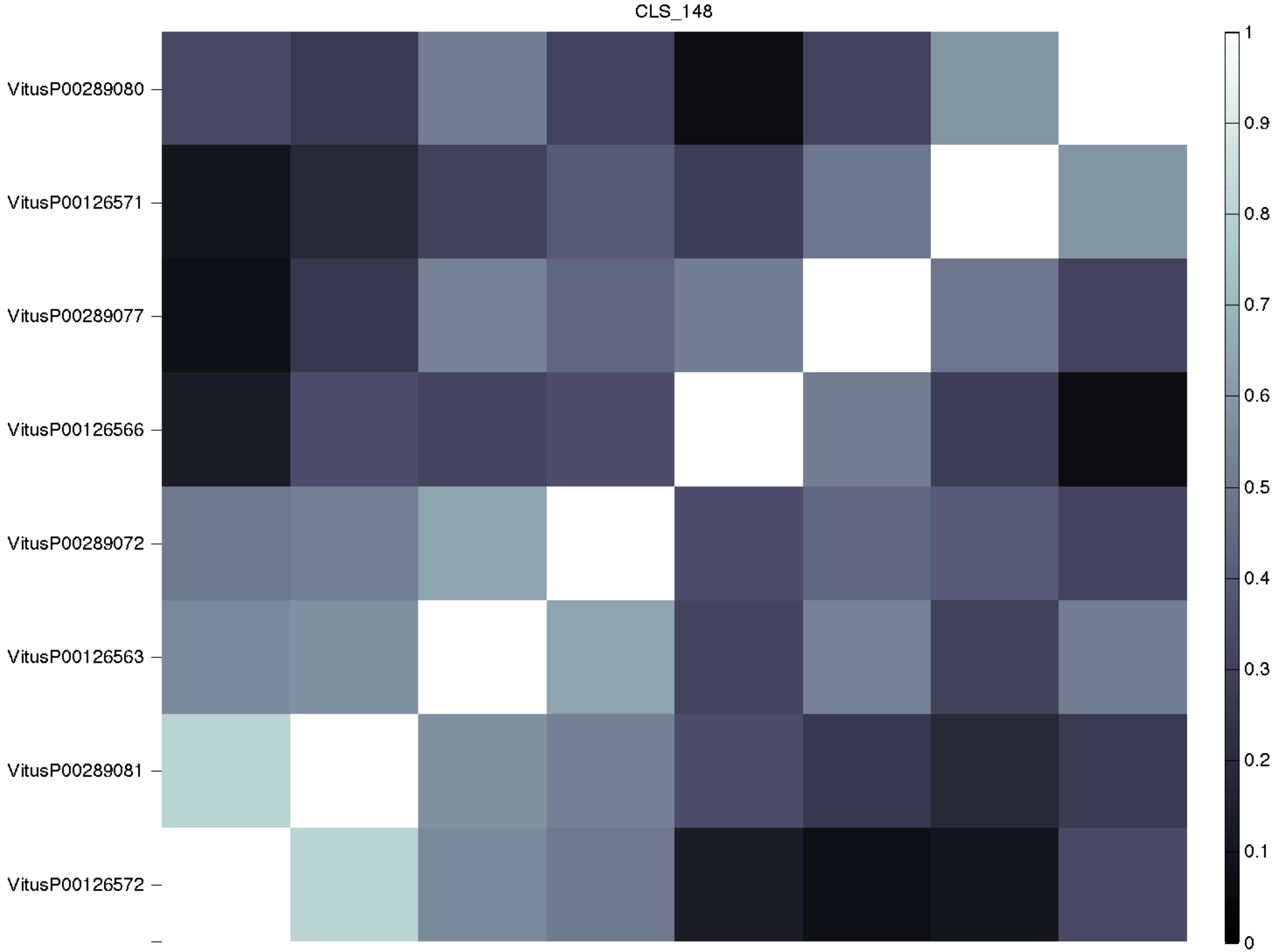

CLS_148
VitusP00289080
VitusP00126571
VitusP00289077
VitusP00126566
VitusP00289072
VitusP00126563
VitusP00289081
VitusP00126572
1
0.9
0.8
0.7
0.6
0.5
0.4
0.3
0.2
0.1
0

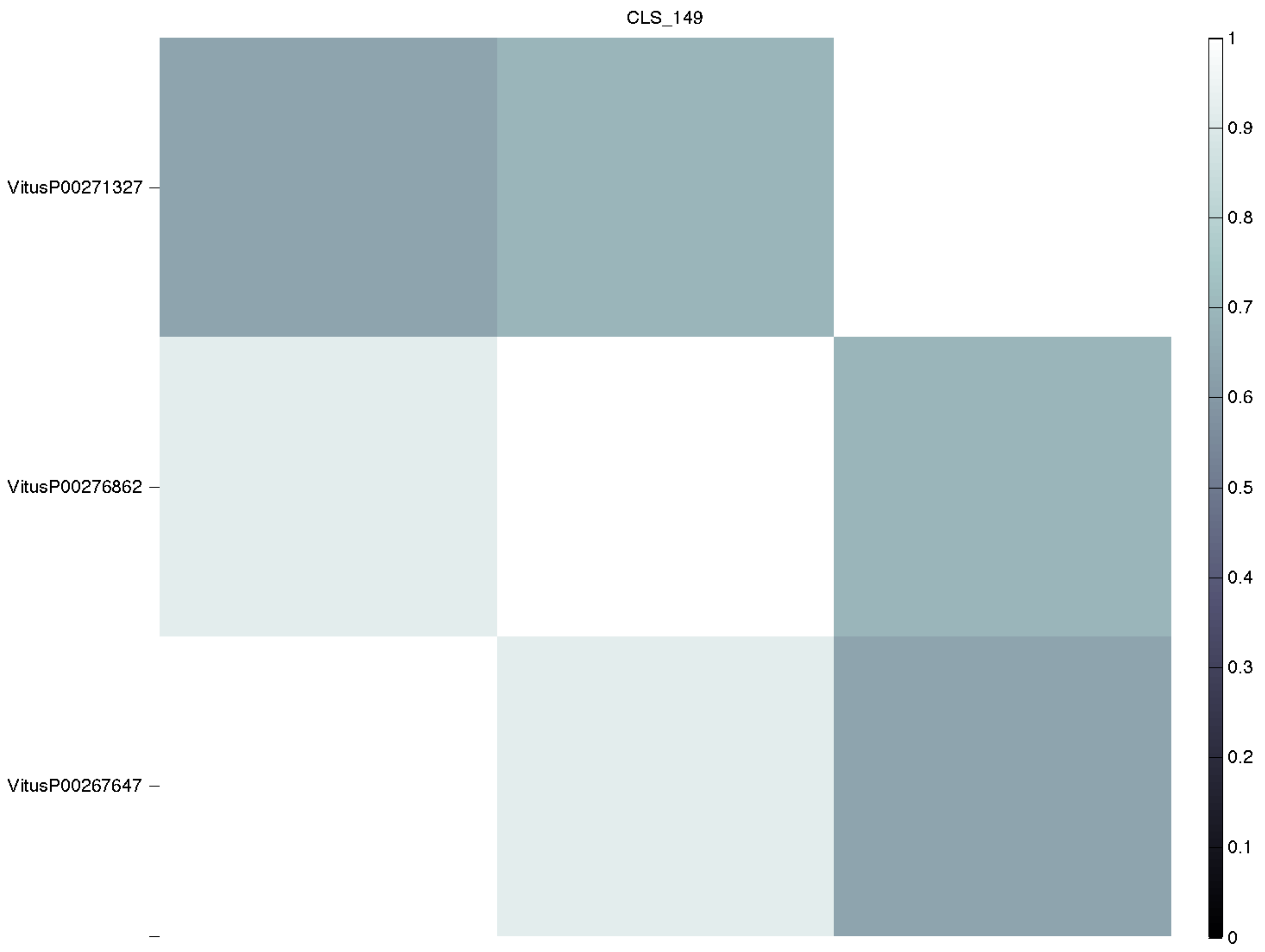
CLS_149
VitusP00271327
VitusP00276862
VitusP00267647
1
0.9
0.8
0.7
0.6
0.5
0.4
0.3
0.2
0.1
0

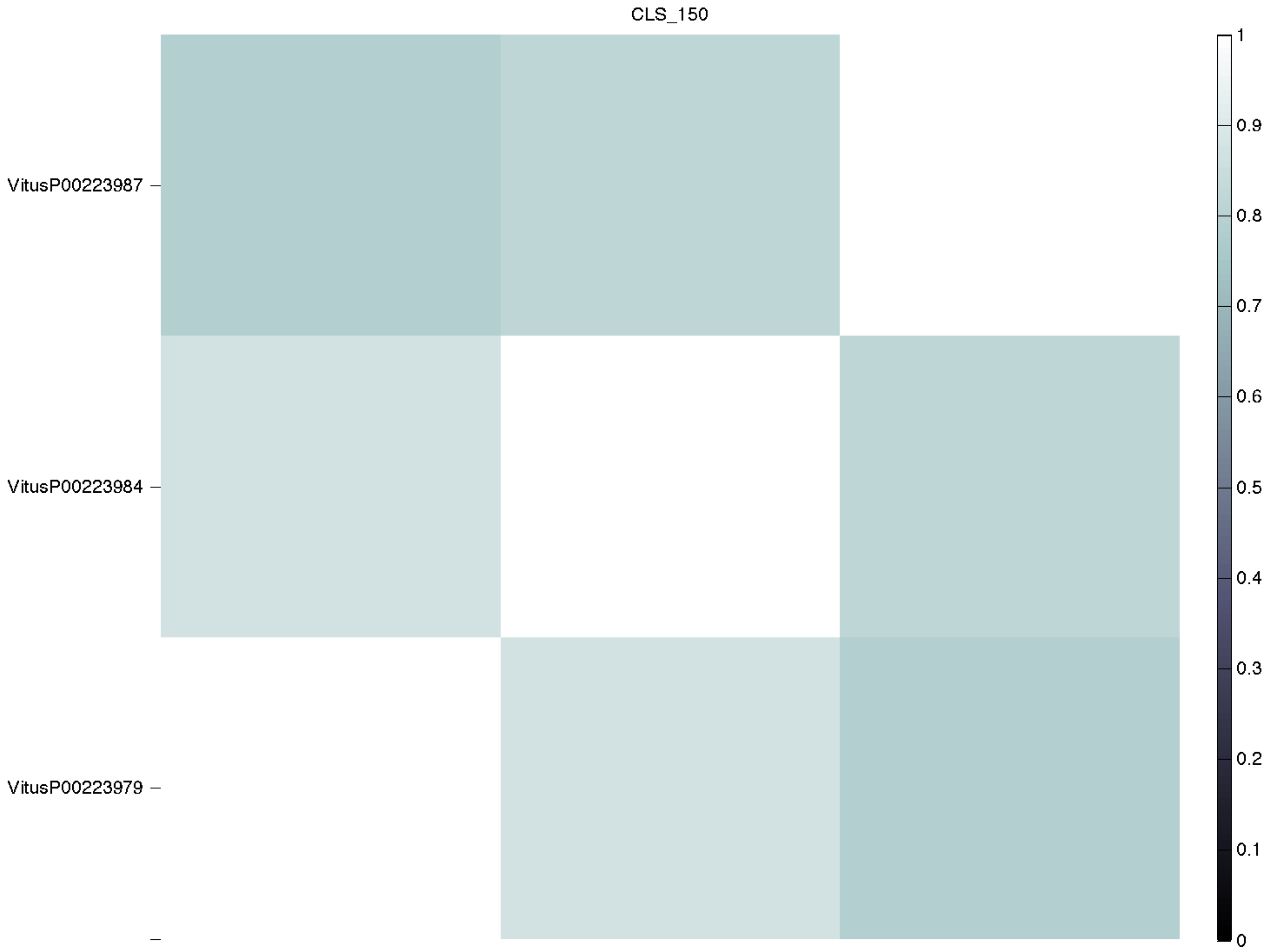

CLS_150
VitusP00223987
VitusP00223984
VitusP00223979
1
0.9
0.8
0.7
0.6
0.5
0.4
0.3
0.2
0.1
0

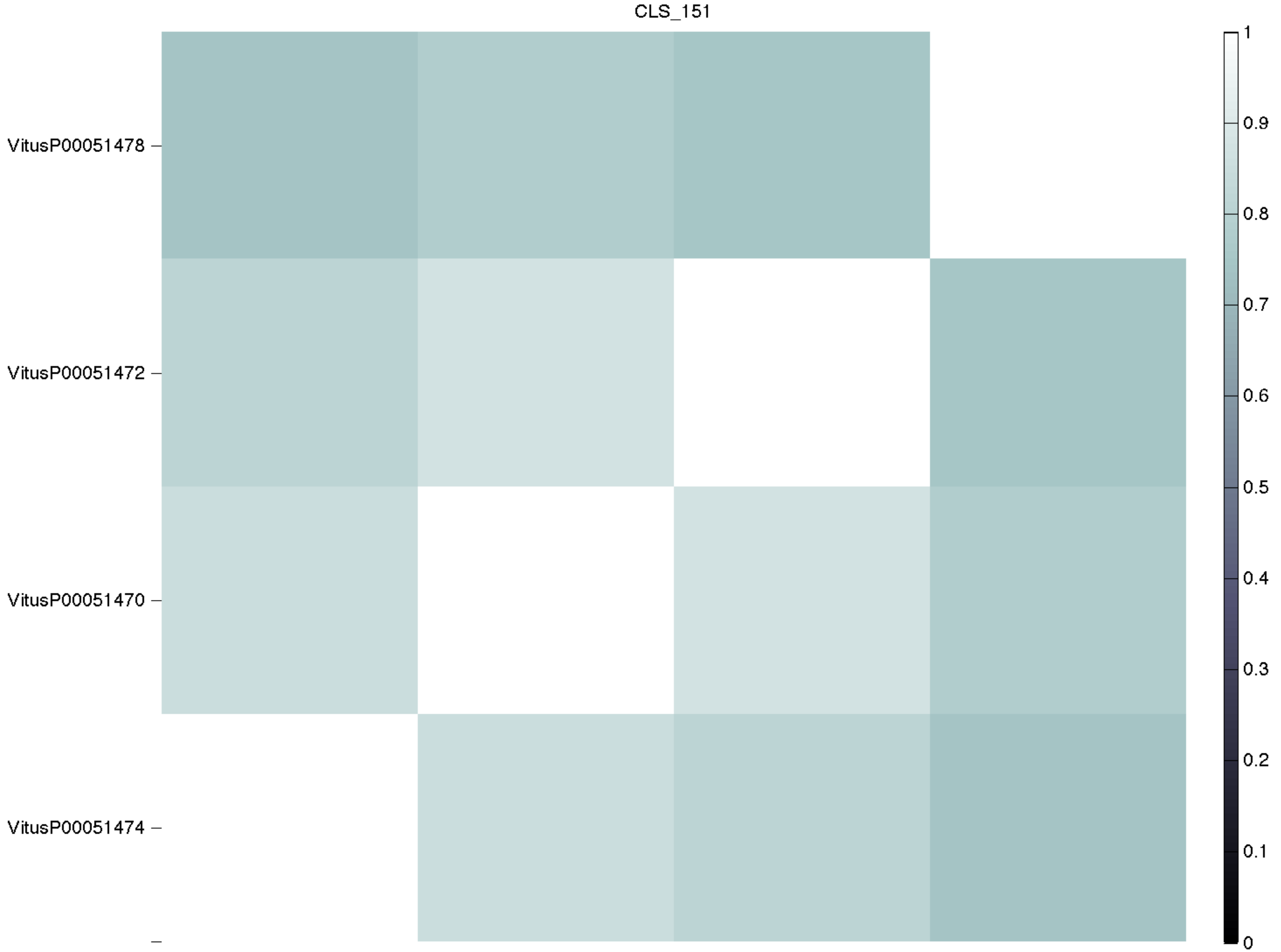
CLS_151
VitusP00051478
VitusP00051472
VitusP00051470
VitusP00051474
1
0.9
0.8
0.7
0.6
0.5
0.4
0.3
0.2
0.1
0

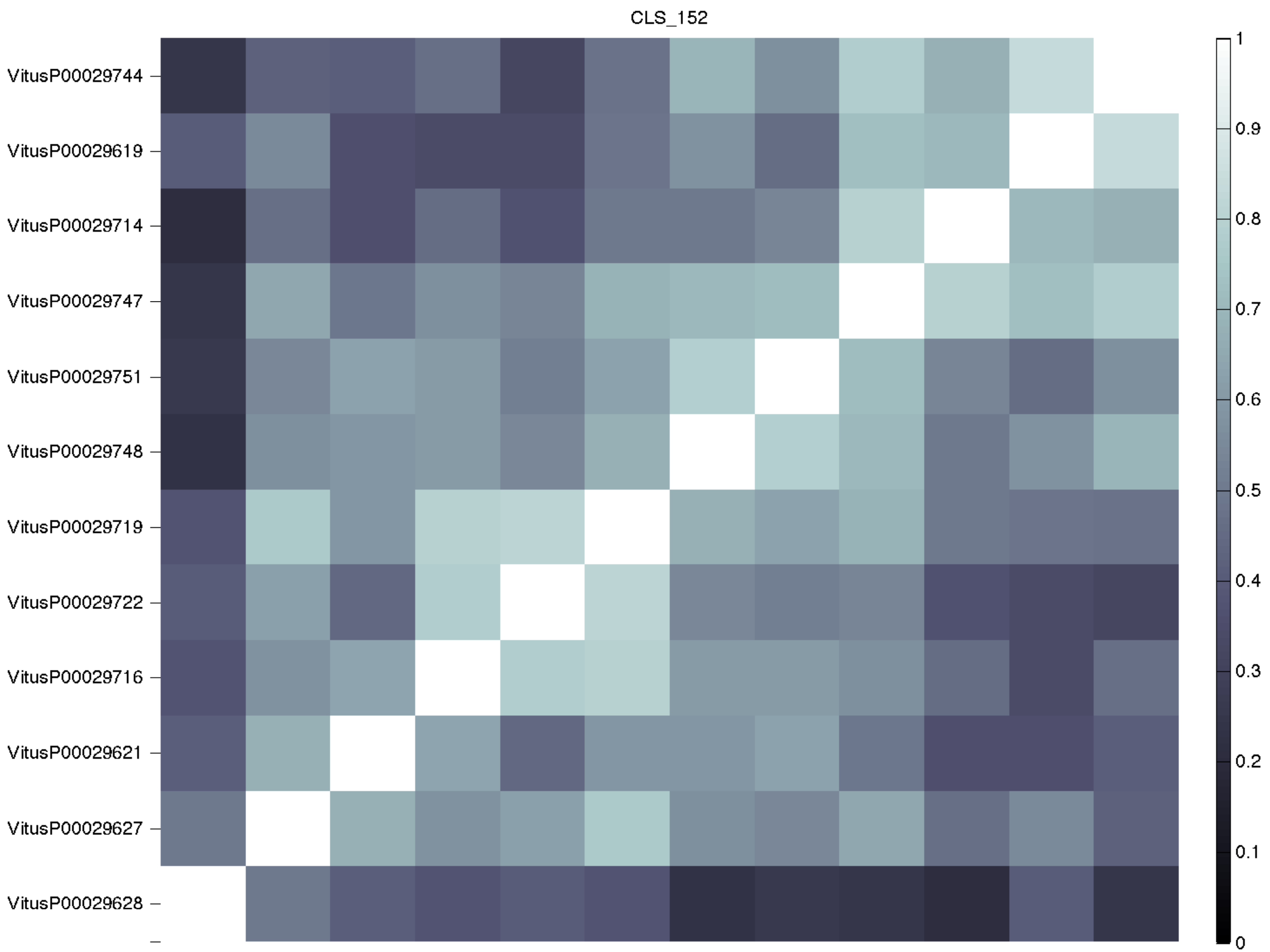

CLS_152
VitusP00029744
VitusP00029619
VitusP00029714
VitusP00029747
VitusP00029751
VitusP00029748
VitusP00029719
VitusP00029722
VitusP00029716
VitusP00029621
VitusP00029627
VitusP00029628
1
0.9
0.8
0.7
0.6
0.5
0.4
0.3
0.2
0.1
0

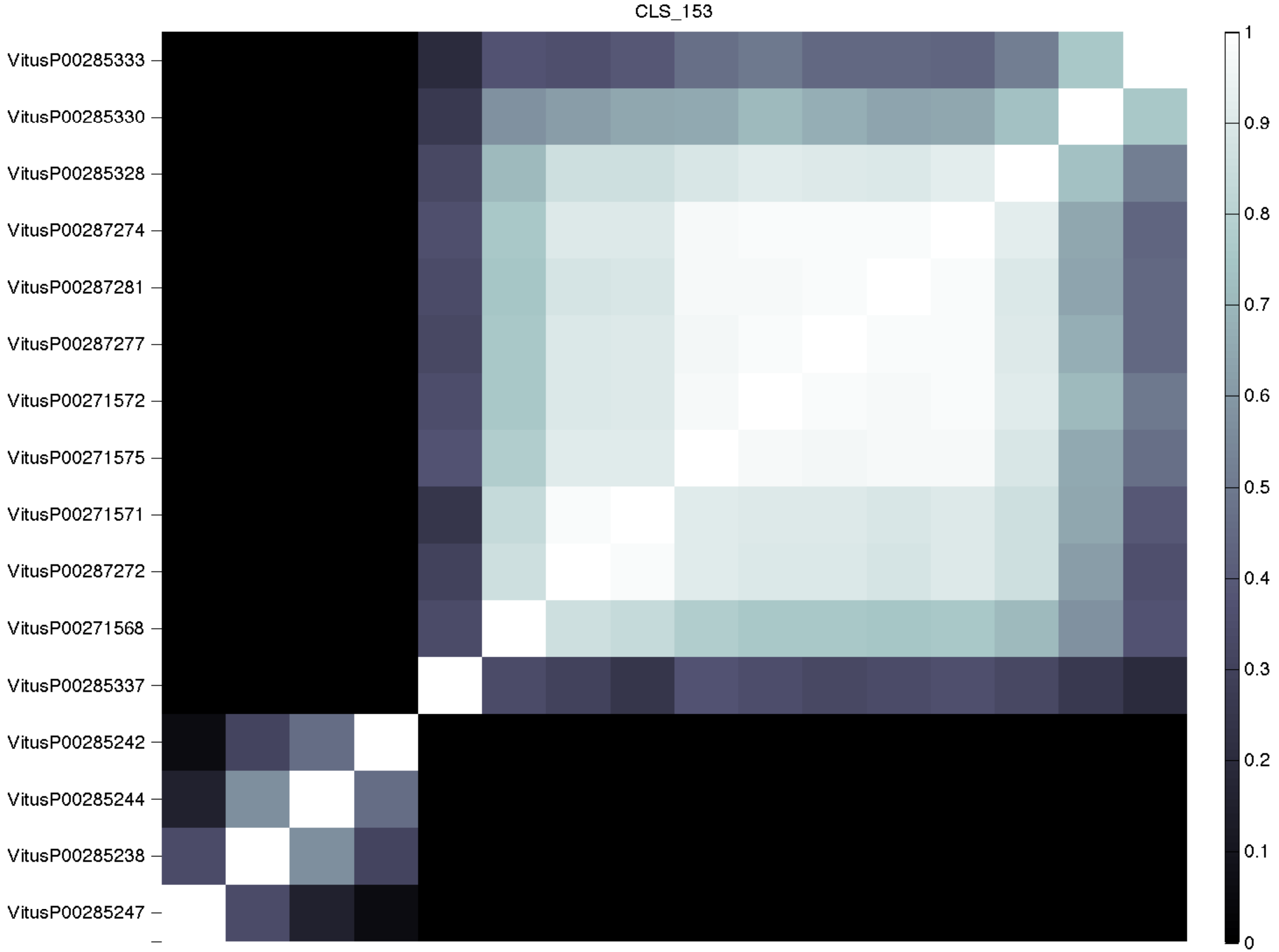
CLS_153
VitusP00285333
VitusP00285330
VitusP00285328
VitusP00287274
VitusP00287281
VitusP00287277
VitusP00271572
VitusP00271575
VitusP00271571
VitusP00287272
VitusP00271568
VitusP00285337
VitusP00285242
VitusP00285244
VitusP00285238
VitusP00285247
1
0.9
0.8
0.7
0.6
0.5
0.4
0.3
0.2
0.1
0

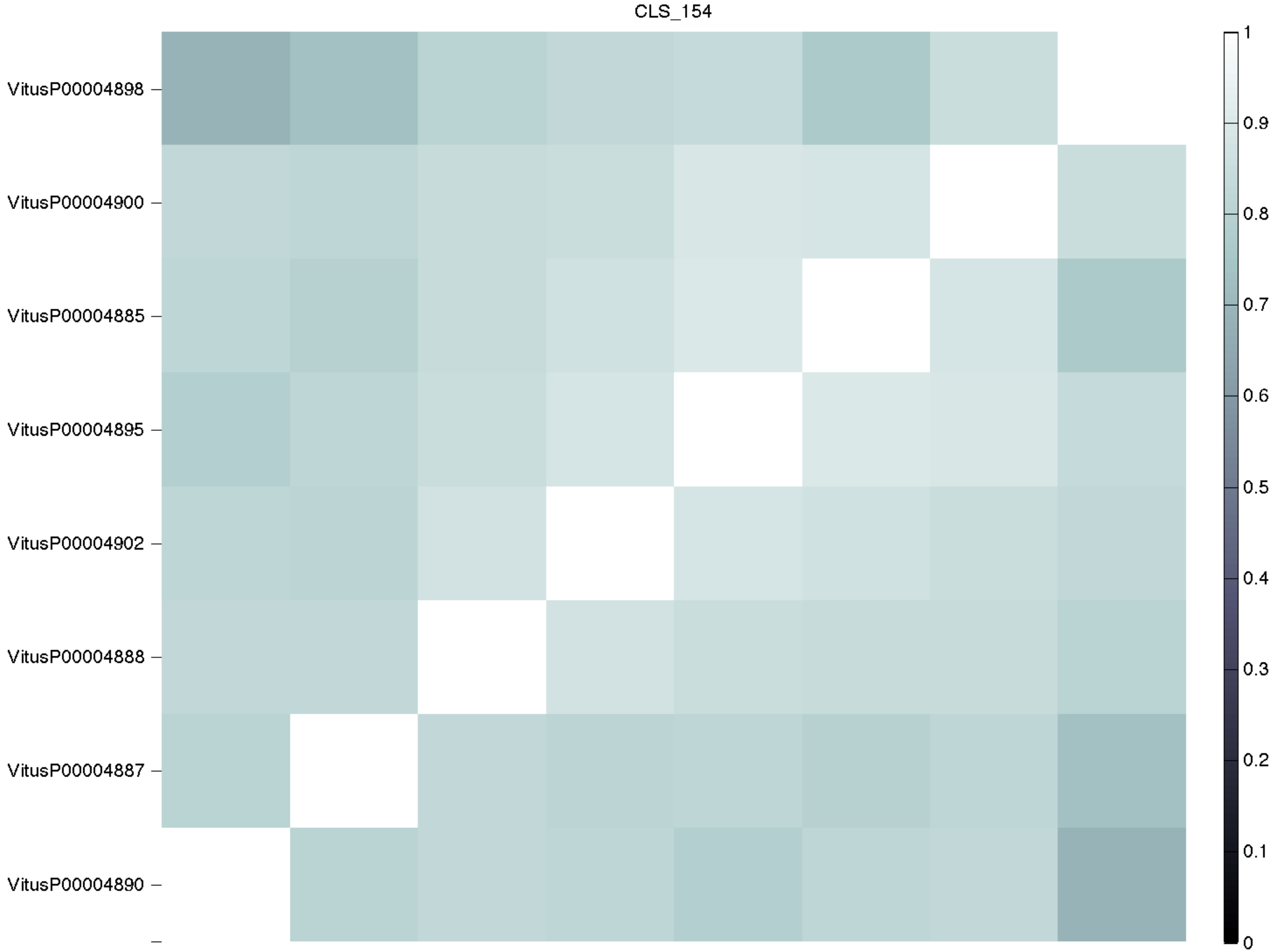

CLS_154
VitusP00004898
VitusP00004900
VitusP00004885
VitusP00004895
VitusP00004902
VitusP00004888
VitusP00004887
VitusP00004890
1
0.9
0.8
0.7
0.6
0.5
0.4
0.3
0.2
0.1
0

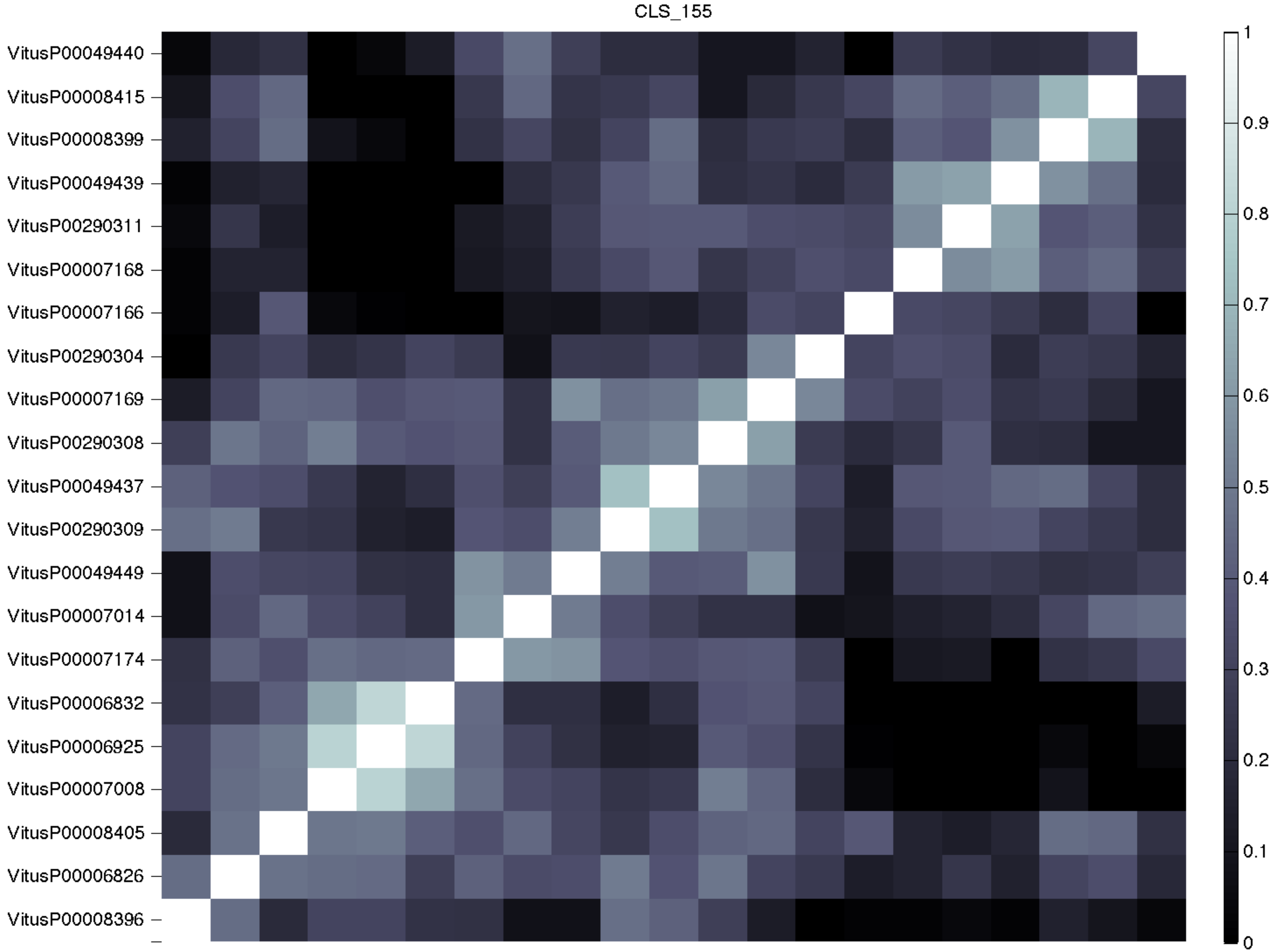
CLS_155
VitusP00049440
VitusP00008415
VitusP00008399
VitusP00049439
VitusP00290311
VitusP00007168
VitusP00007166
VitusP00290304
VitusP00007169
VitusP00290308
VitusP00049437
VitusP00290309
VitusP00049449
VitusP00007014
VitusP00007174
VitusP00006832
VitusP00006925
VitusP00007008
VitusP00008405
VitusP00006826
VitusP00008396
1
0.9
0.8
0.7
0.6
0.5
0.4
0.3
0.2
0.1
0

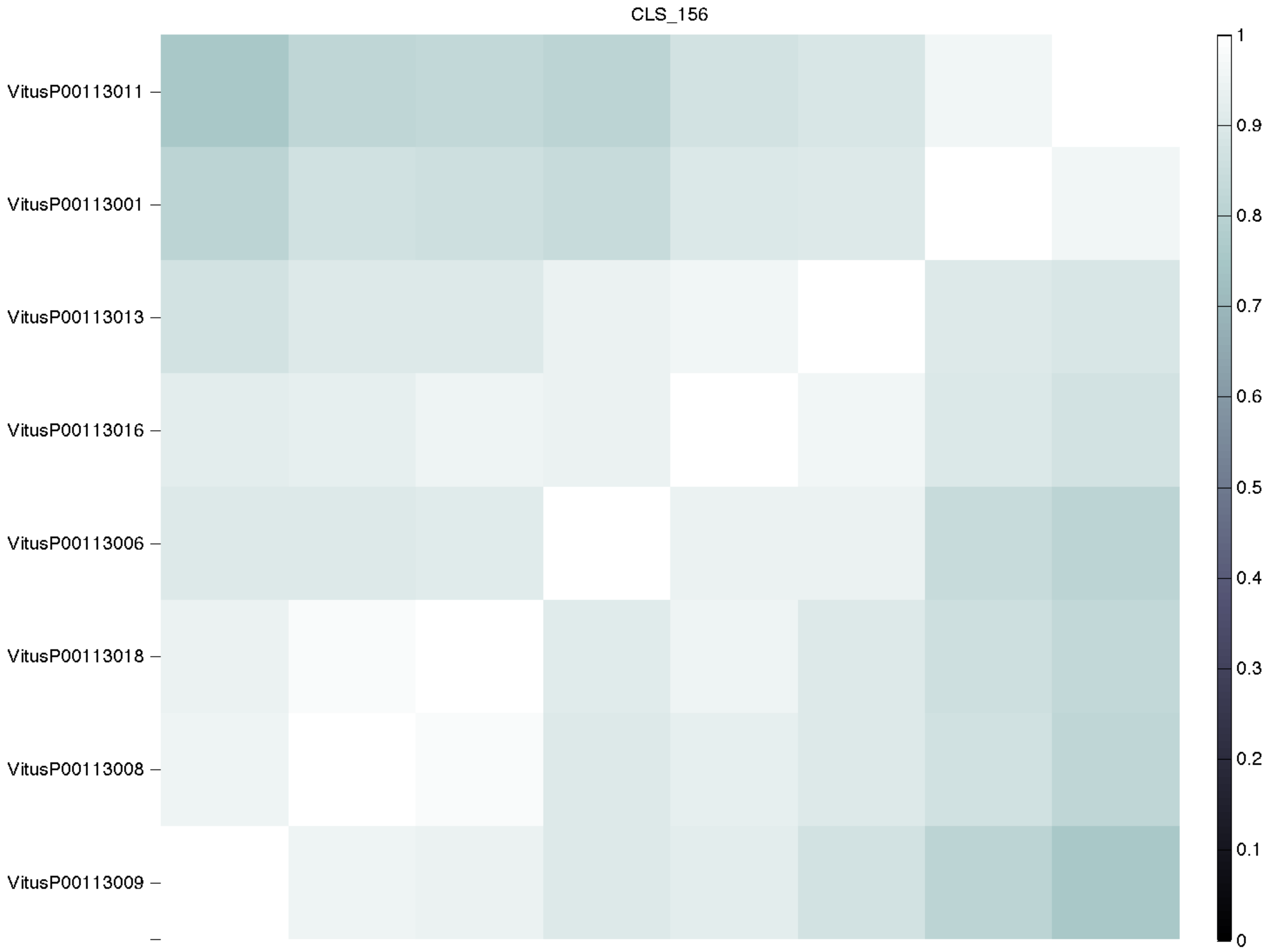
CLS_156
VitusP00113011
VitusP00113001
VitusP00113013
VitusP00113016
VitusP00113006
VitusP00113018
VitusP00113008
VitusP00113009
1
0.9
0.8
0.7
0.6
0.5
0.4
0.3
0.2
0.1
0

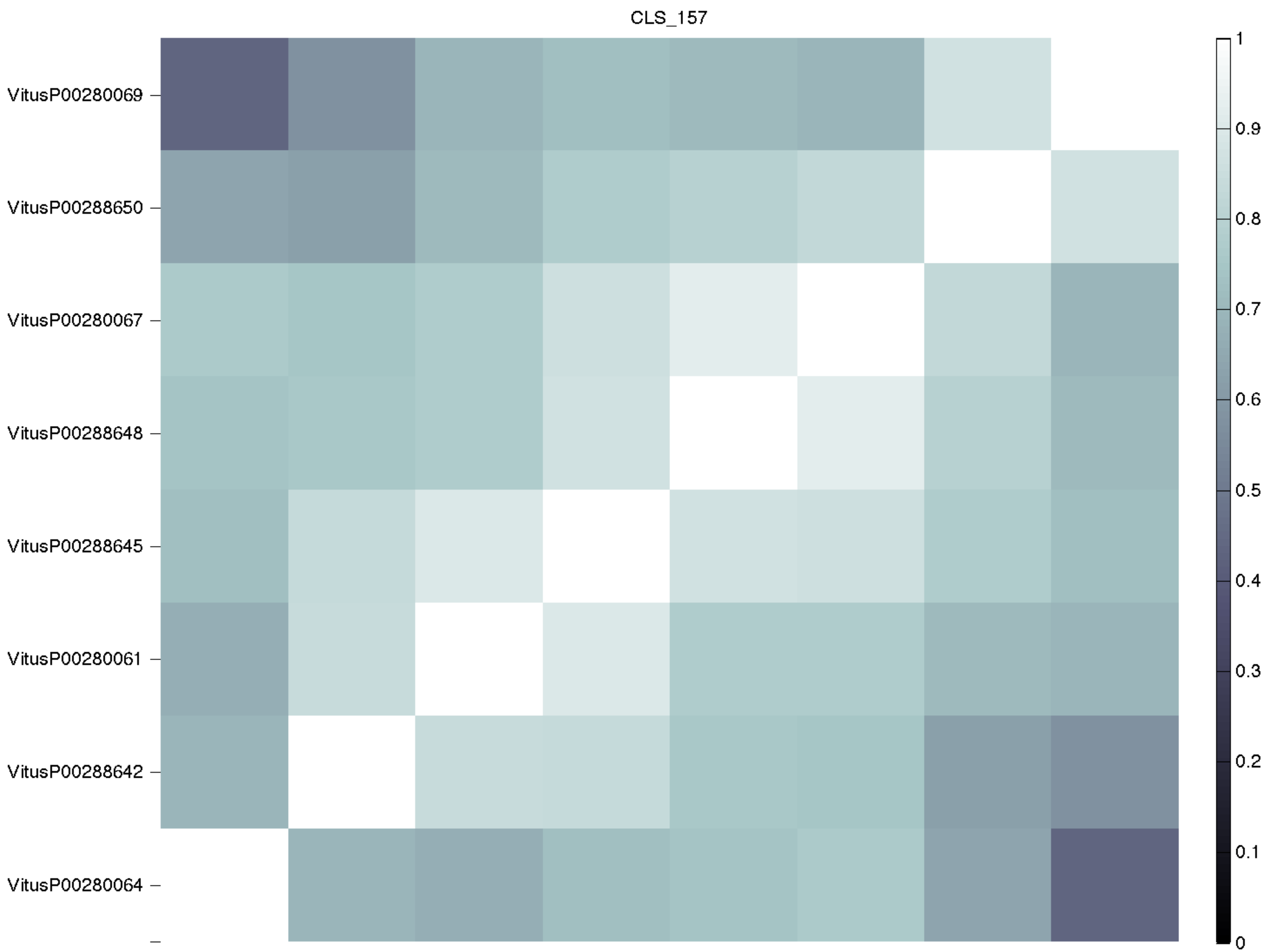
CLS_157
VitusP00280069
VitusP00288650
VitusP00280067
VitusP00288648
VitusP00288645
VitusP00280061
VitusP00288642
VitusP00280064
1
0.9
0.8
0.7
0.6
0.5
0.4
0.3
0.2
0.1
0

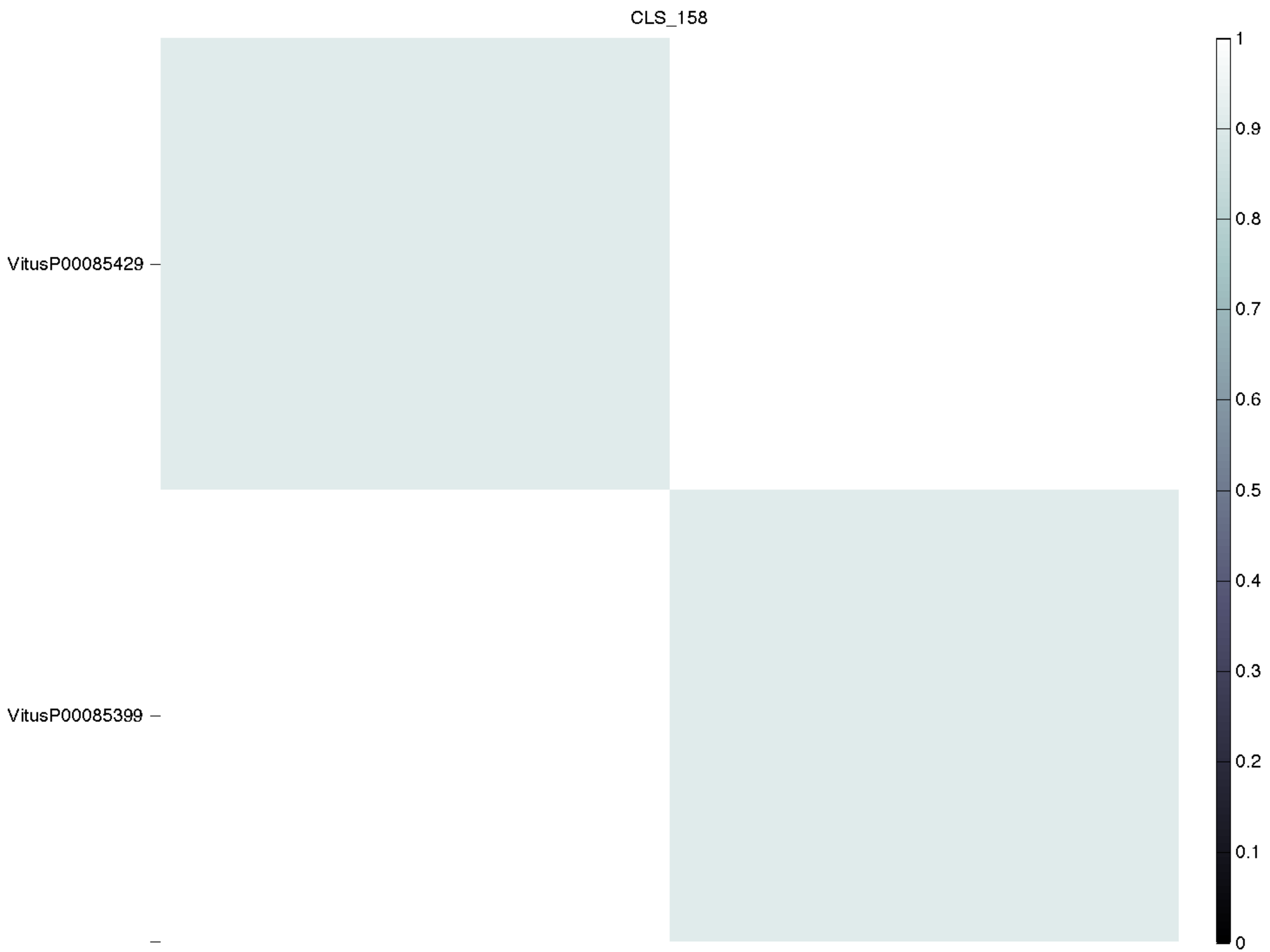
CLS_158
VitusP00085429
VitusP00085399
1
0.9
0.8
0.7
0.6
0.5
0.4
0.3
0.2
0.1
0

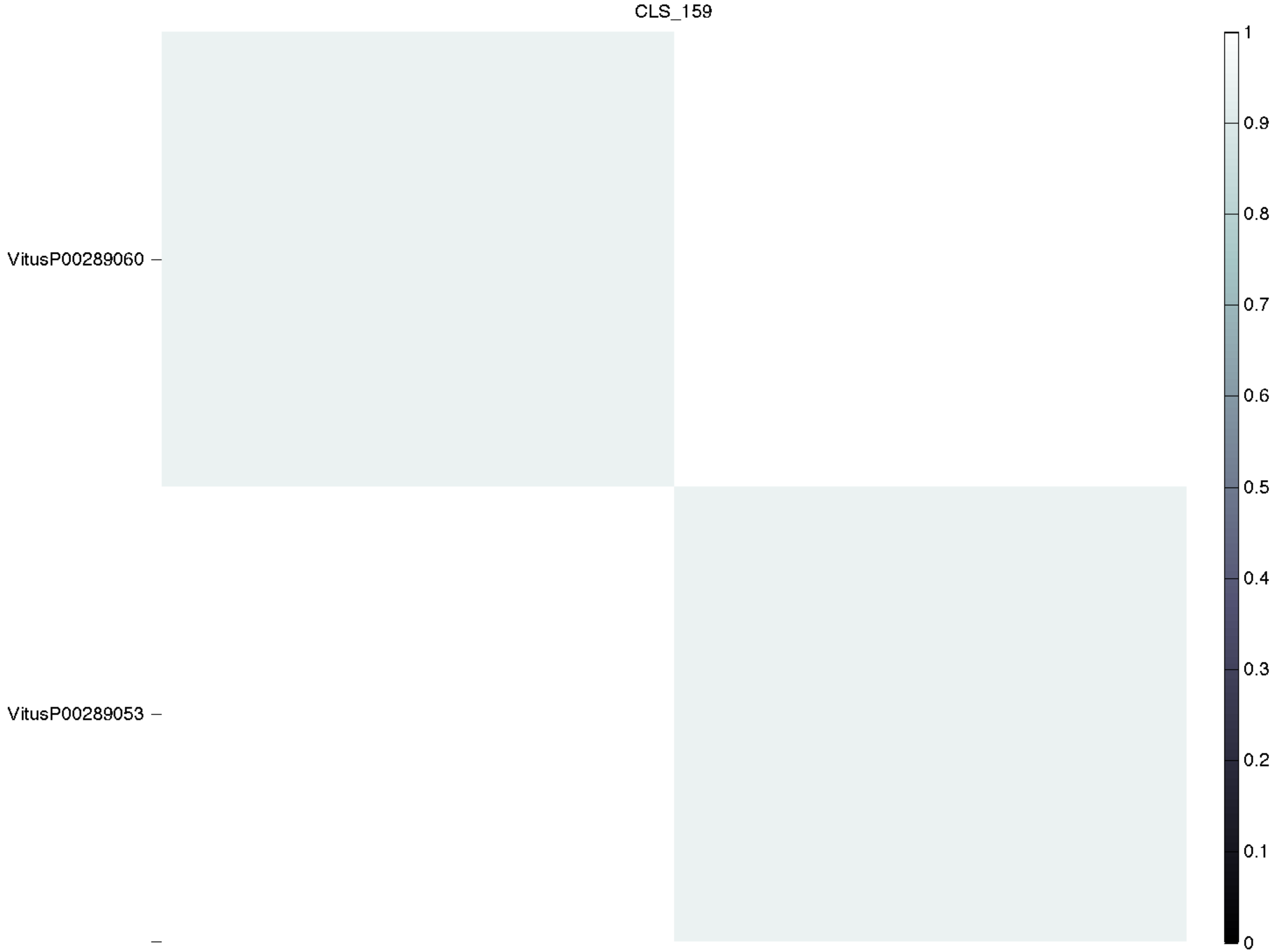
CLS_159
VitusP00289060
VitusP00289053
1
0.9
0.8
0.7
0.6
0.5
0.4
0.3
0.2
0.1
0

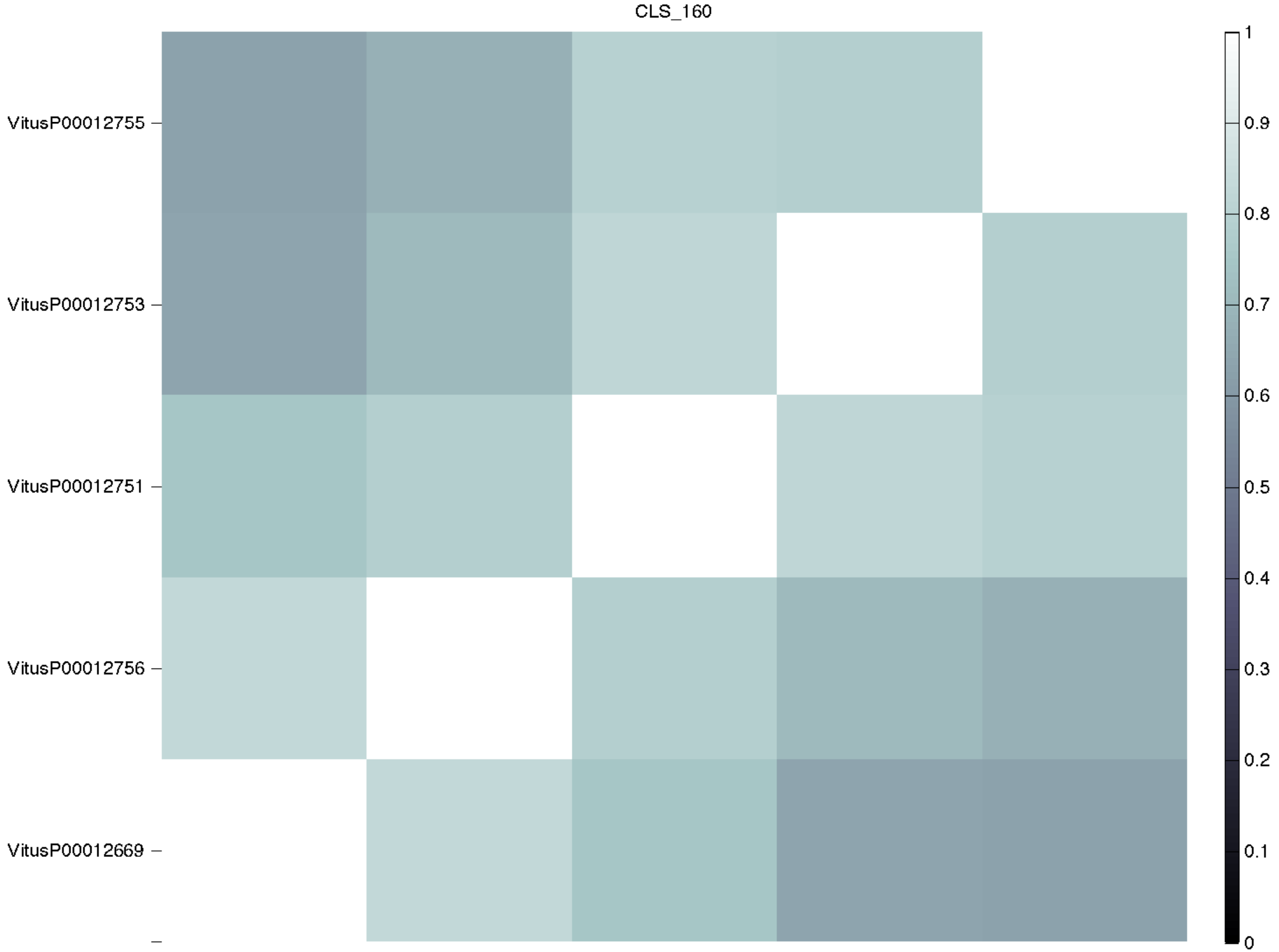
CLS_160
VitusP00012755
VitusP00012753
VitusP00012751
VitusP00012756
VitusP00012669
1
0.9
0.8
0.7
0.6
0.5
0.4
0.3
0.2
0.1
0

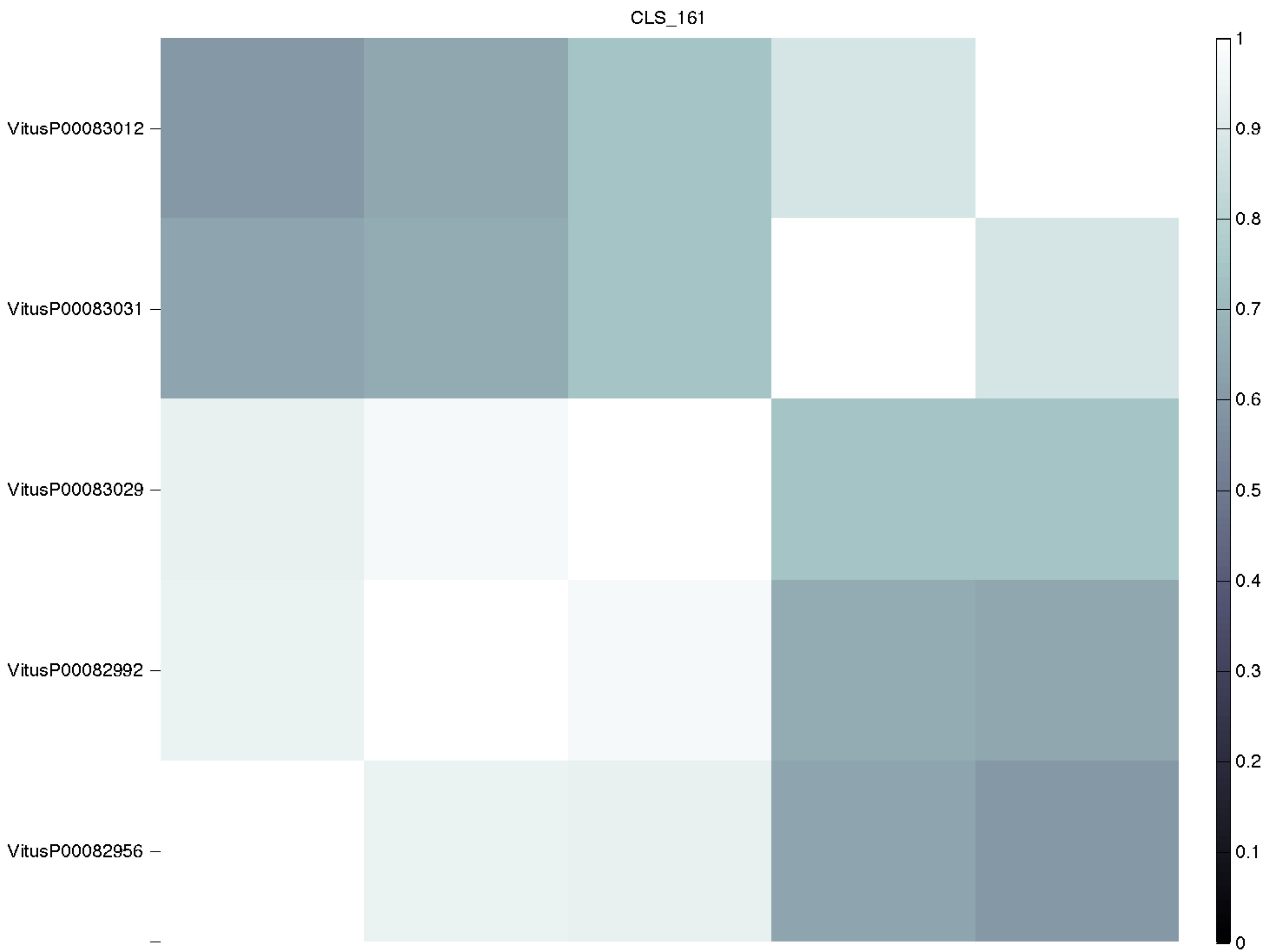

CLS_161
VitusP00083012
VitusP00083031
VitusP00083029
VitusP00082992
VitusP00082956
1
0.9
0.8
0.7
0.6
0.5
0.4
0.3
0.2
0.1
0

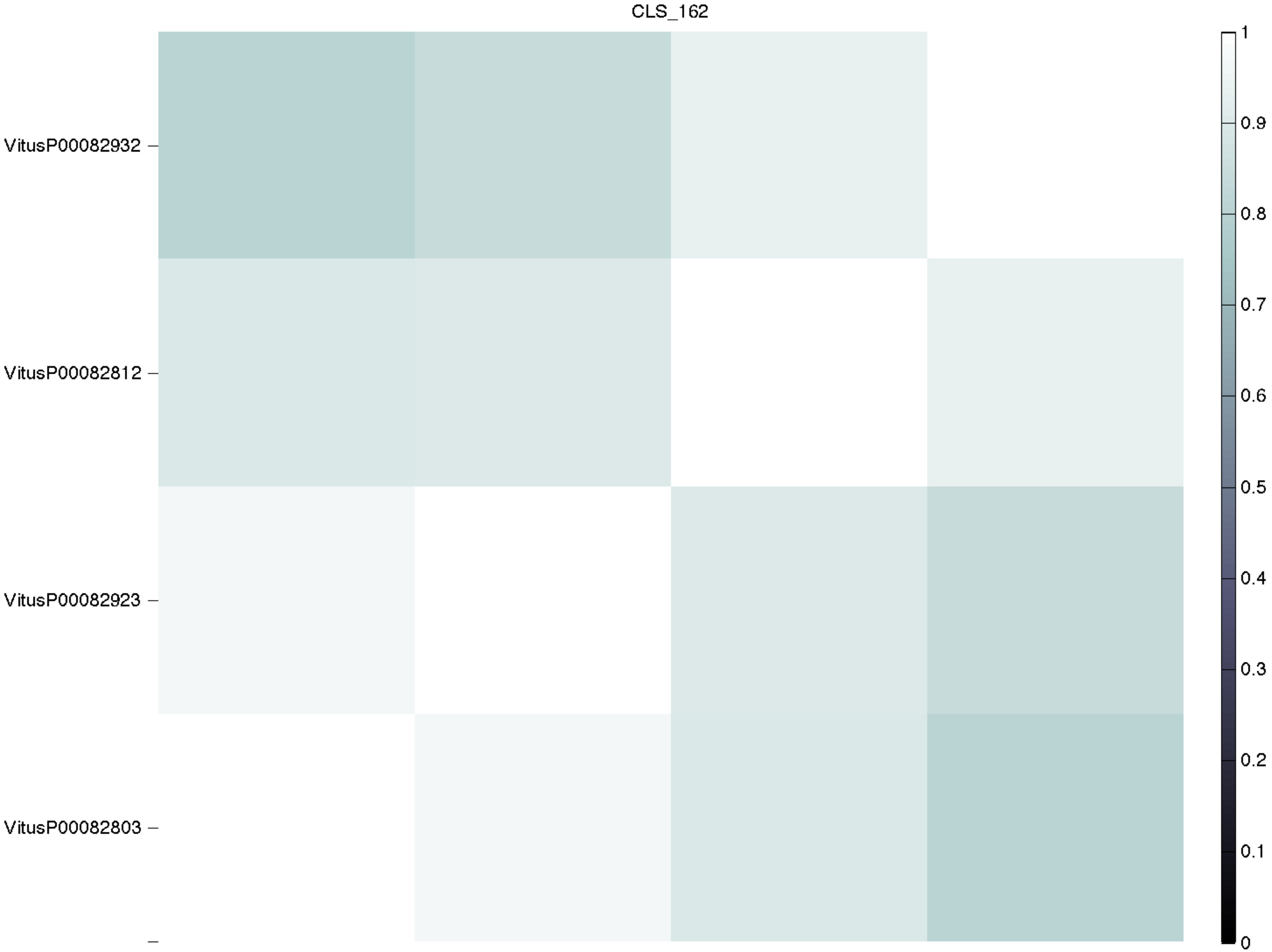
CLS_162
VitusP00082932
VitusP00082812
VitusP00082923
VitusP00082803
1
0.9
0.8
0.7
0.6
0.5
0.4
0.3
0.2
0.1
0

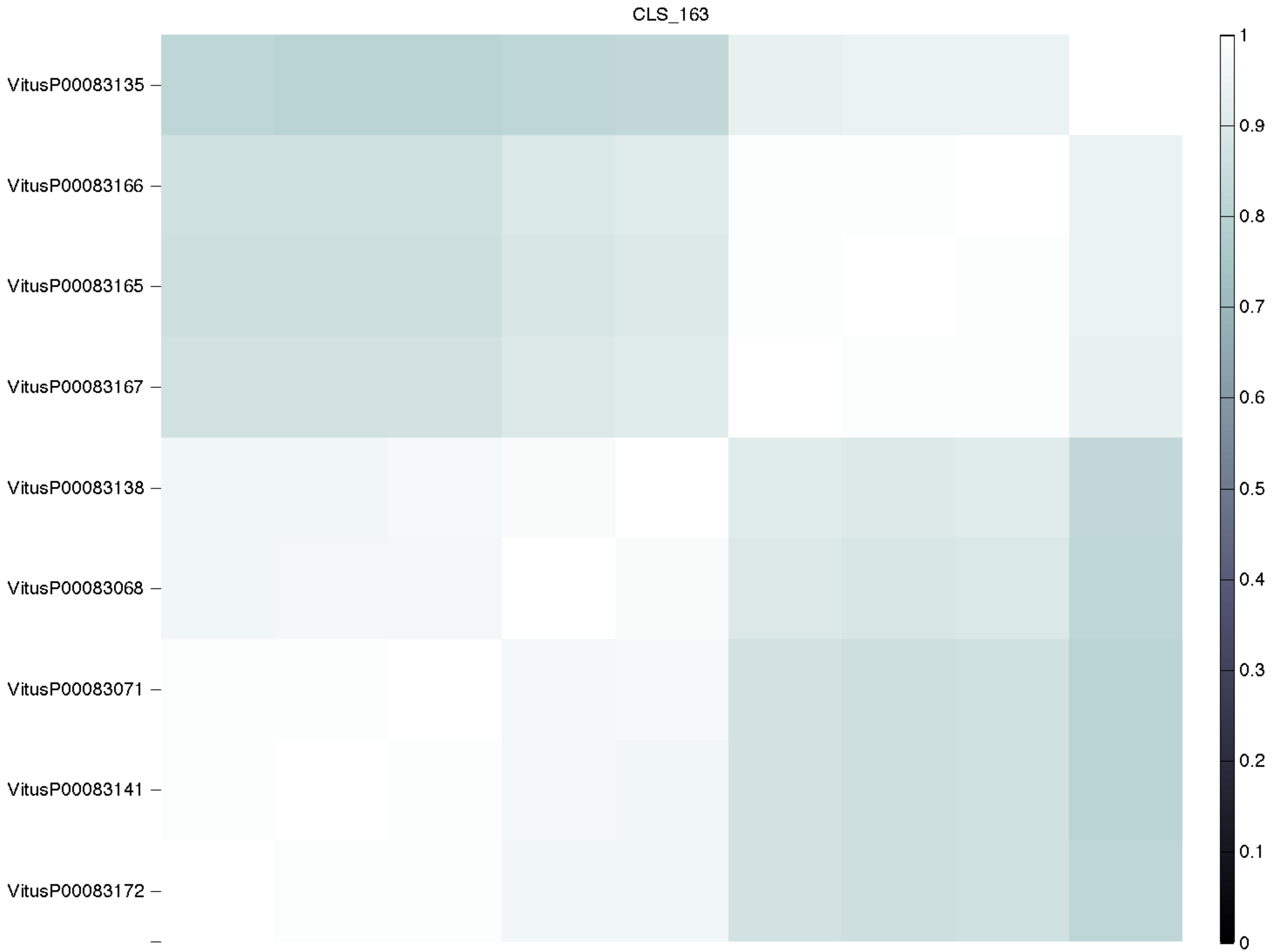

CLS_163
VitusP00083135
VitusP00083166
VitusP00083165
VitusP00083167
VitusP00083138
VitusP00083068
VitusP00083071
VitusP00083141
VitusP00083172
1
0.9
0.8
0.7
0.6
0.5
0.4
0.3
0.2
0.1
0

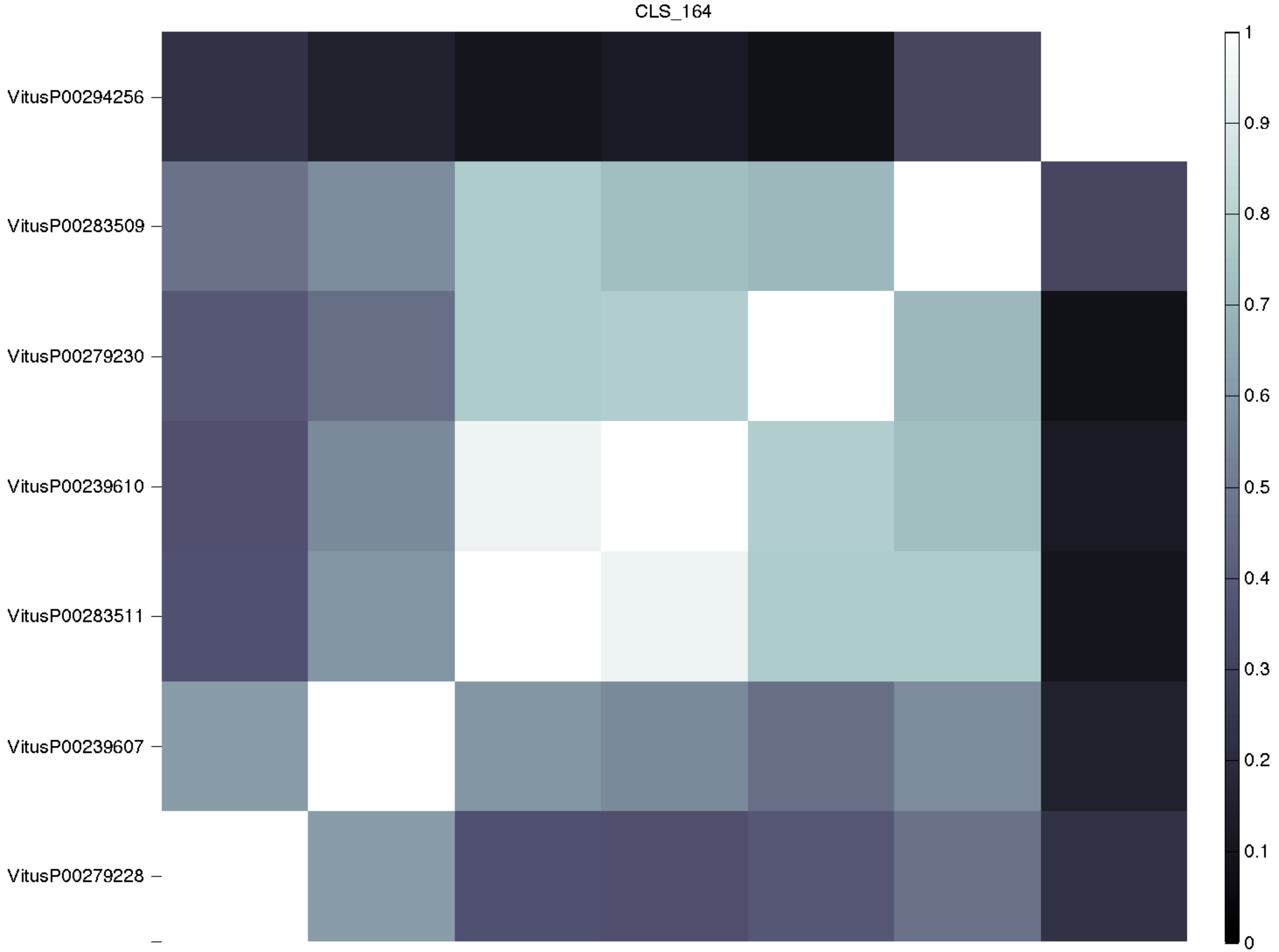

CLS_164
VitusP00294256
VitusP00283509
VitusP00279230
VitusP00239610
VitusP00283511
VitusP00239607
VitusP00279228
1
0.9
0.8
0.7
0.6
0.5
0.4
0.3
0.2
0.1
0

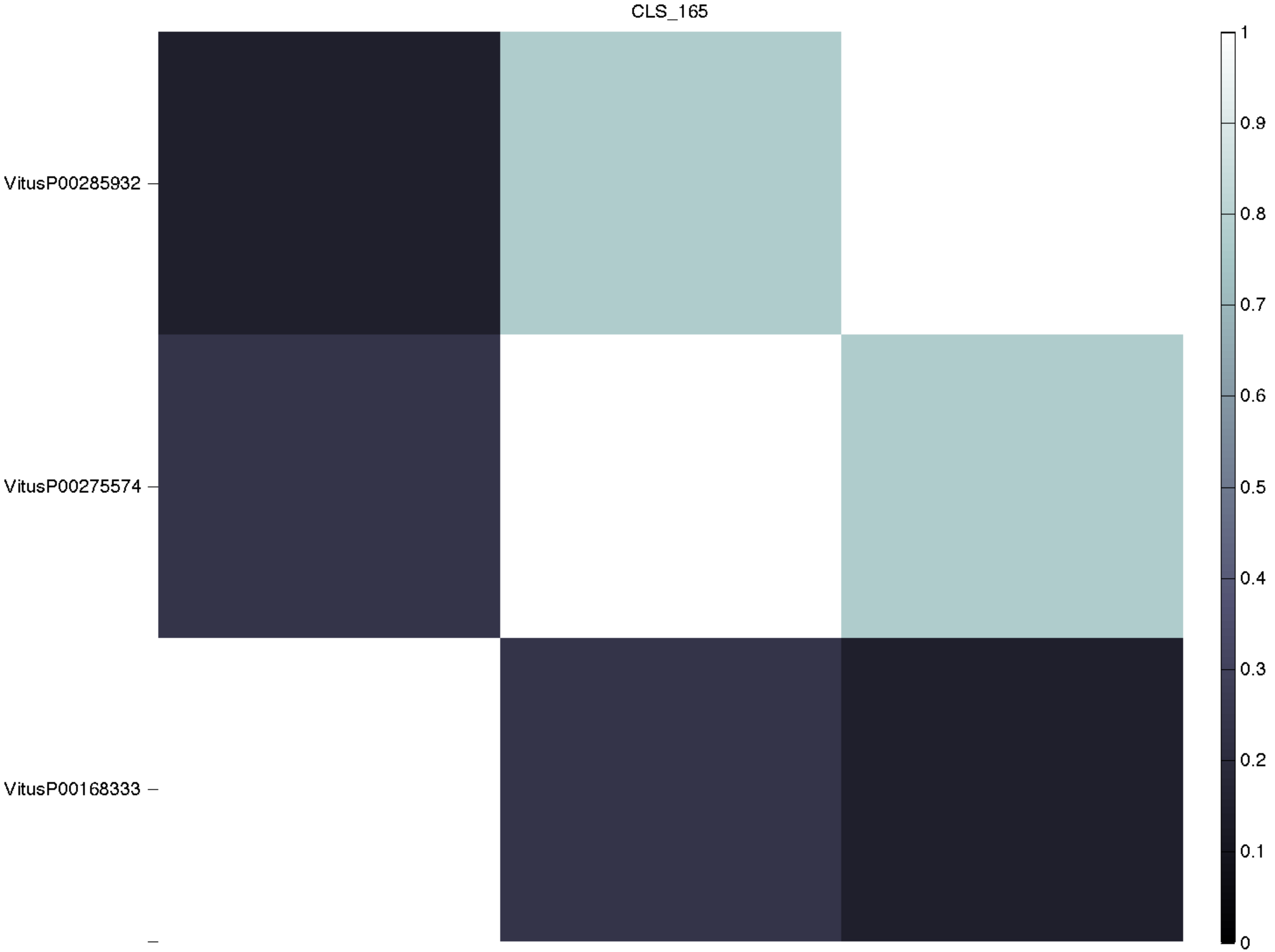

CLS_165
VitusP00285932
VitusP00275574
VitusP00168333
1
0.9
0.8
0.7
0.6
0.5
0.4
0.3
0.2
0.1
0

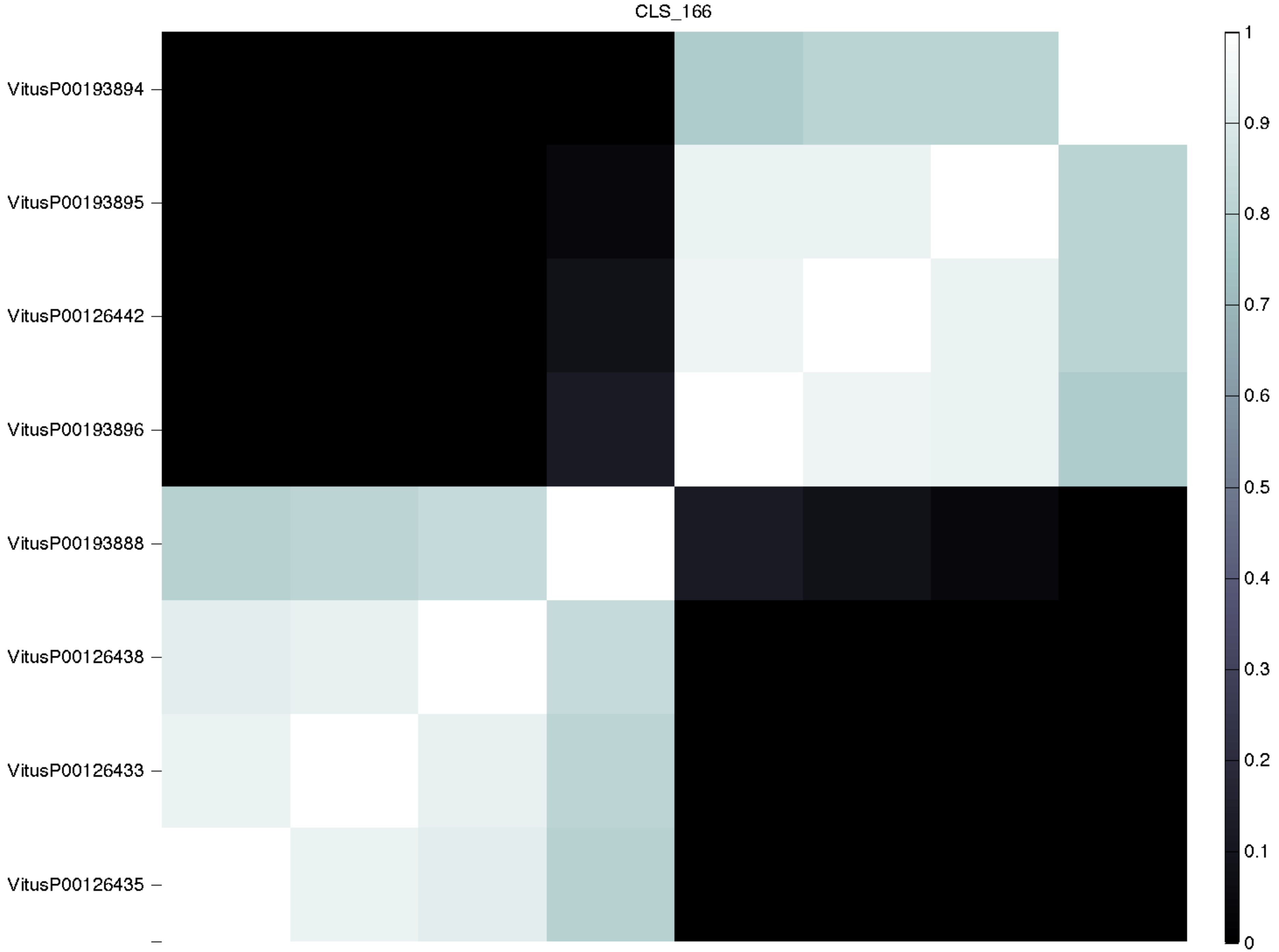

CLS_166
VitusP00193894
VitusP00193895
VitusP00126442
VitusP00193896
VitusP00193888
VitusP00126438
VitusP00126433
VitusP00126435
1
0.9
0.8
0.7
0.6
0.5
0.4
0.3
0.2
0.1
0

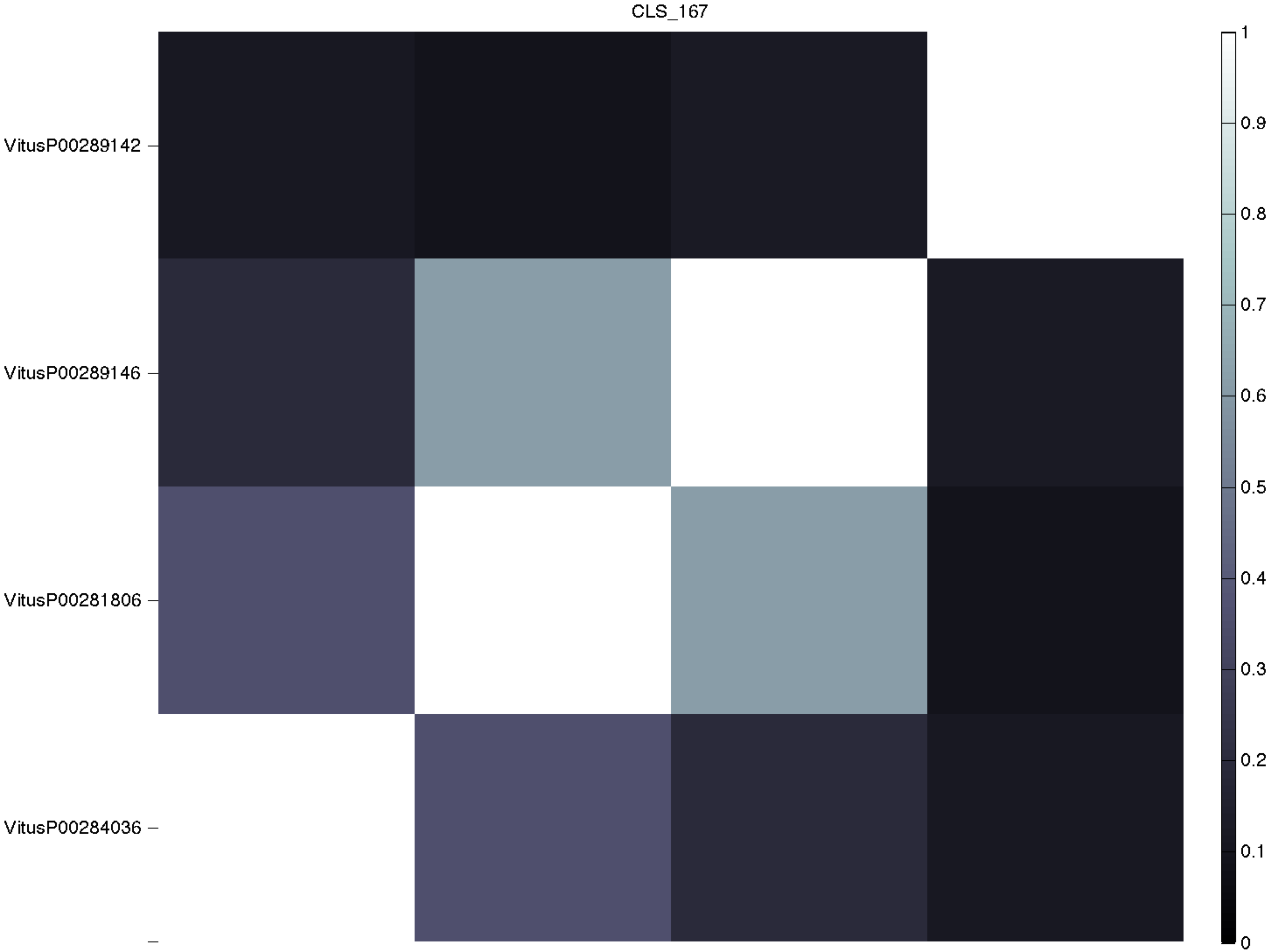
CLS_167
VitusP00289142
VitusP00289146
VitusP00281806
VitusP00284036
1
0.9
0.8
0.7
0.6
0.5
0.4
0.3
0.2
0.1
0

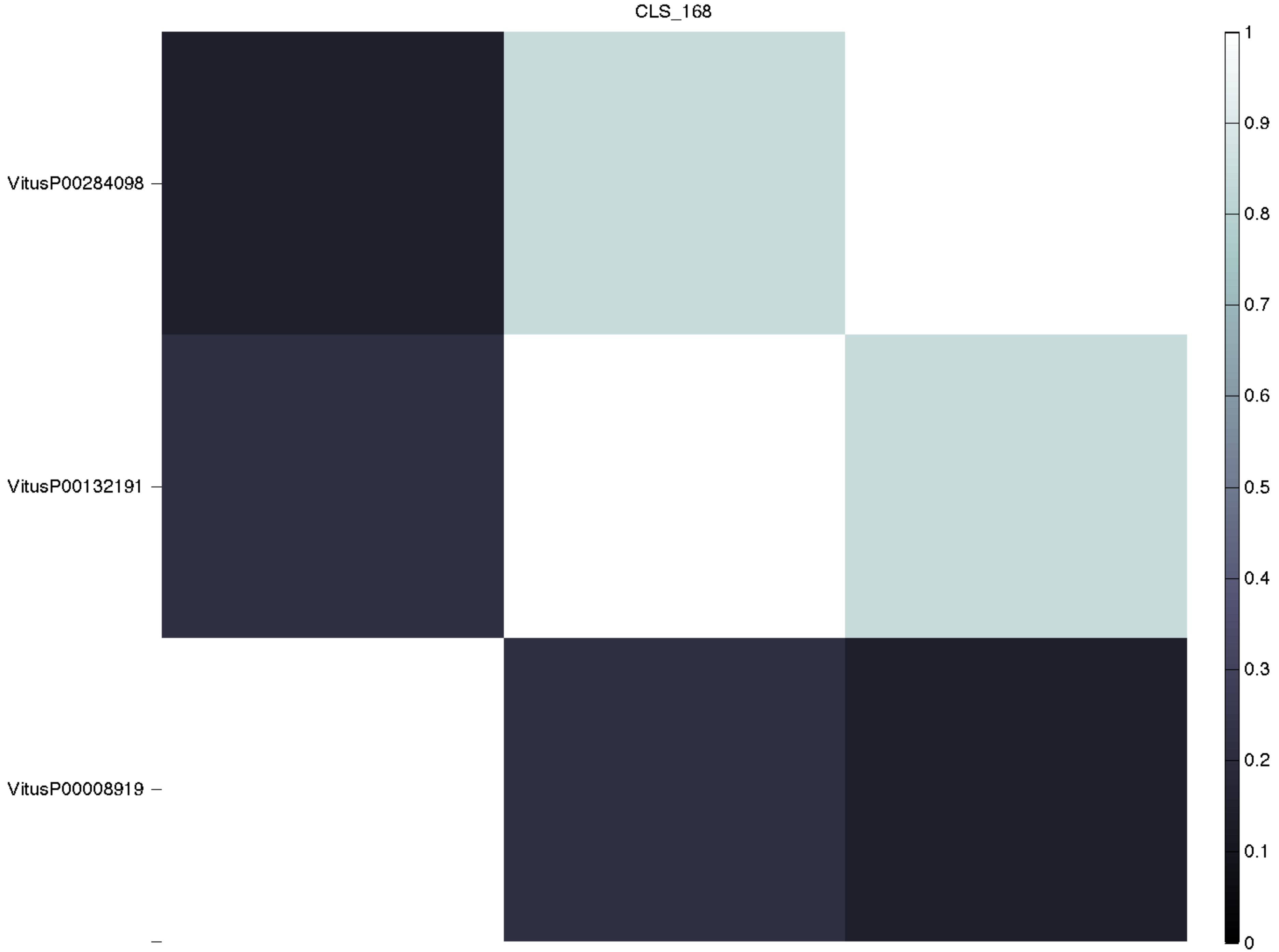

CLS_168
VitusP00284098
VitusP00132191
VitusP00008919
1
0.9
0.8
0.7
0.6
0.5
0.4
0.3
0.2
0.1
0

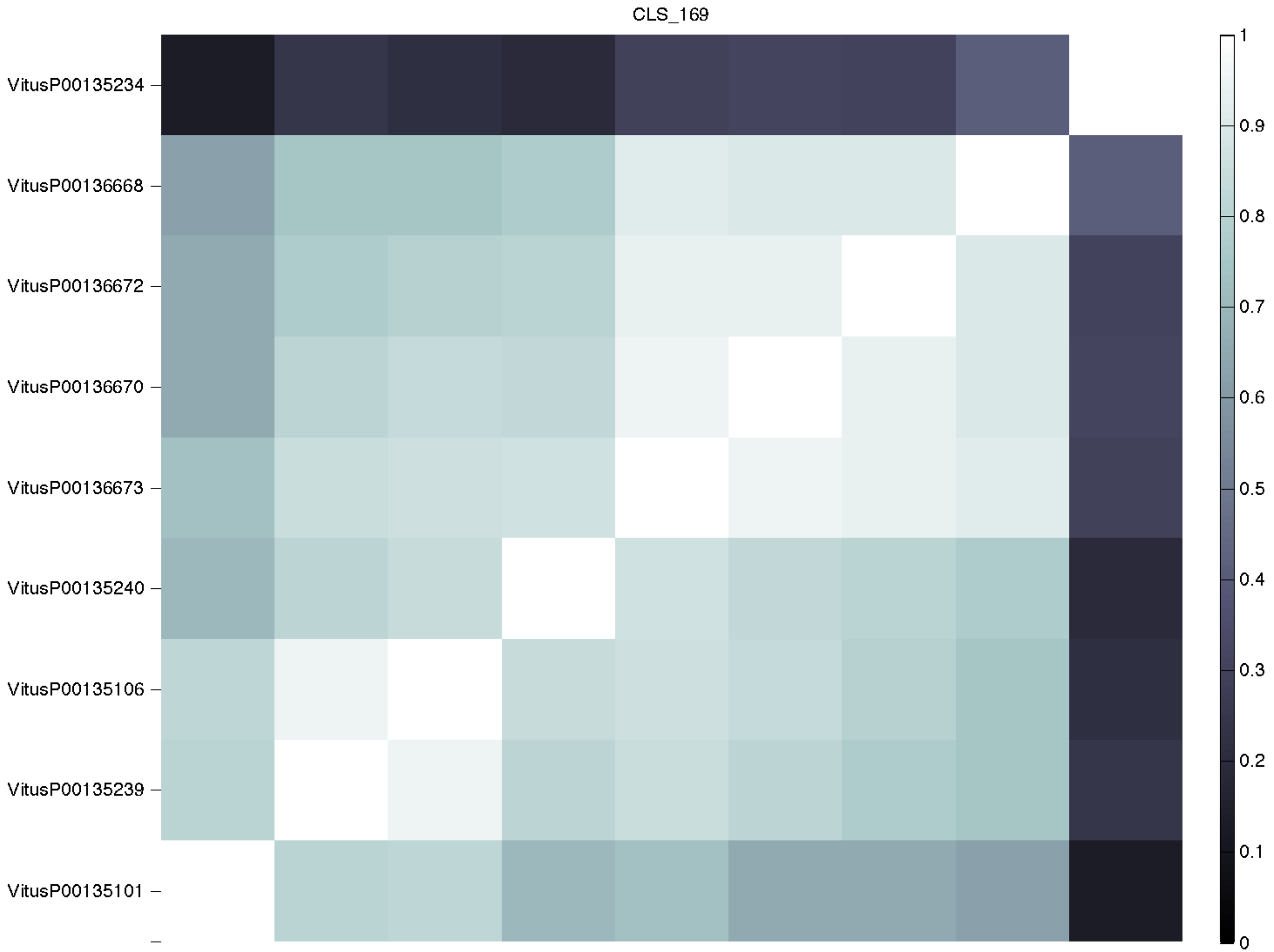
CLS_169
VitusP00135234
VitusP00136668
VitusP00136672
VitusP00136670
VitusP00136673
VitusP00135240
VitusP00135106
VitusP00135239
VitusP00135101
1
0.9
0.8
0.7
0.6
0.5
0.4
0.3
0.2
0.1
0

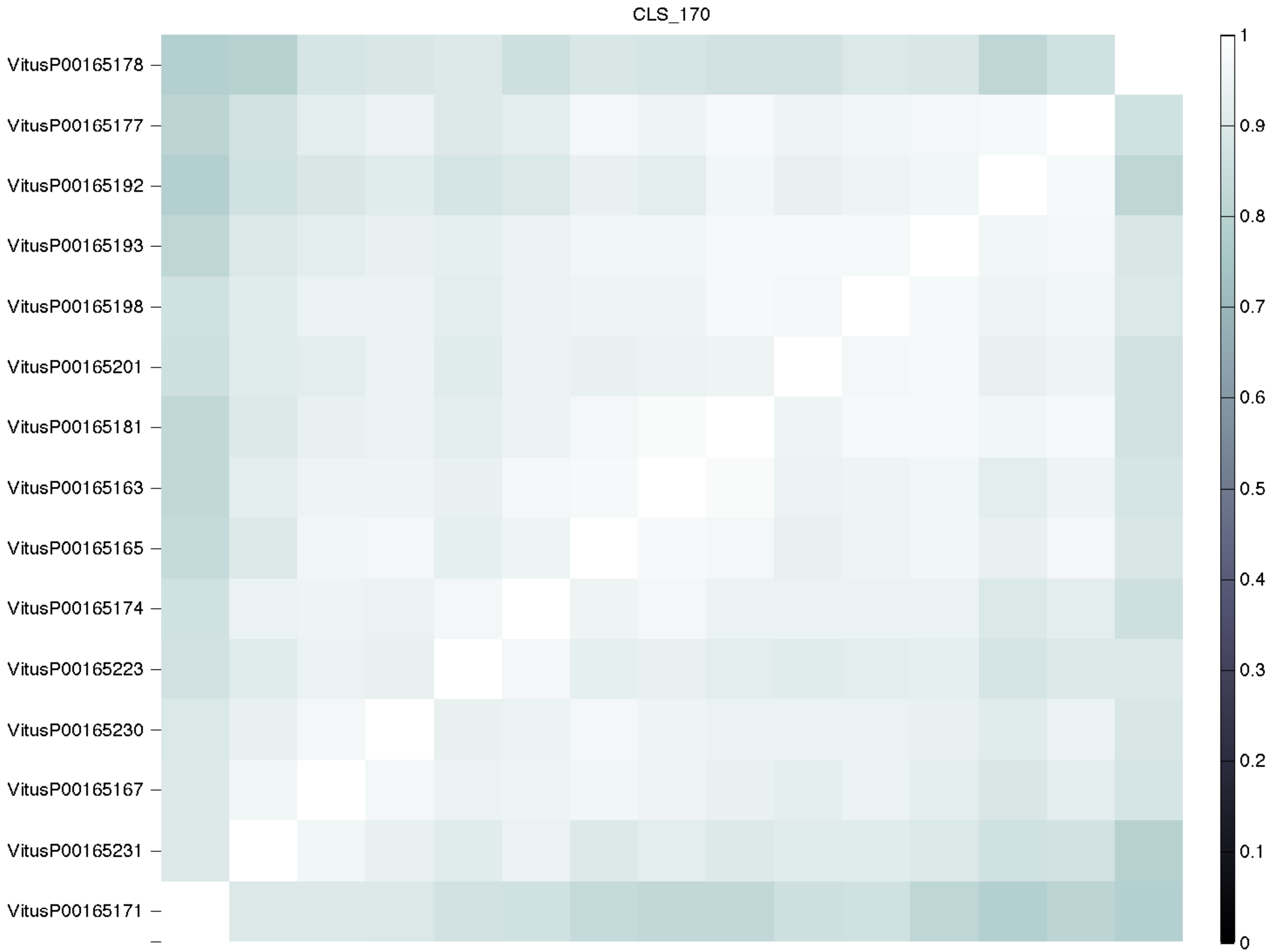
CLS_170
VitusP00165178
VitusP00165177
VitusP00165192
VitusP00165193
VitusP00165198
VitusP00165201
VitusP00165181
VitusP00165163
VitusP00165165
VitusP00165174
VitusP00165223
VitusP00165230
VitusP00165167
VitusP00165231
VitusP00165171
1
0.9
0.8
0.7
0.6
0.5
0.4
0.3
0.2
0.1
0

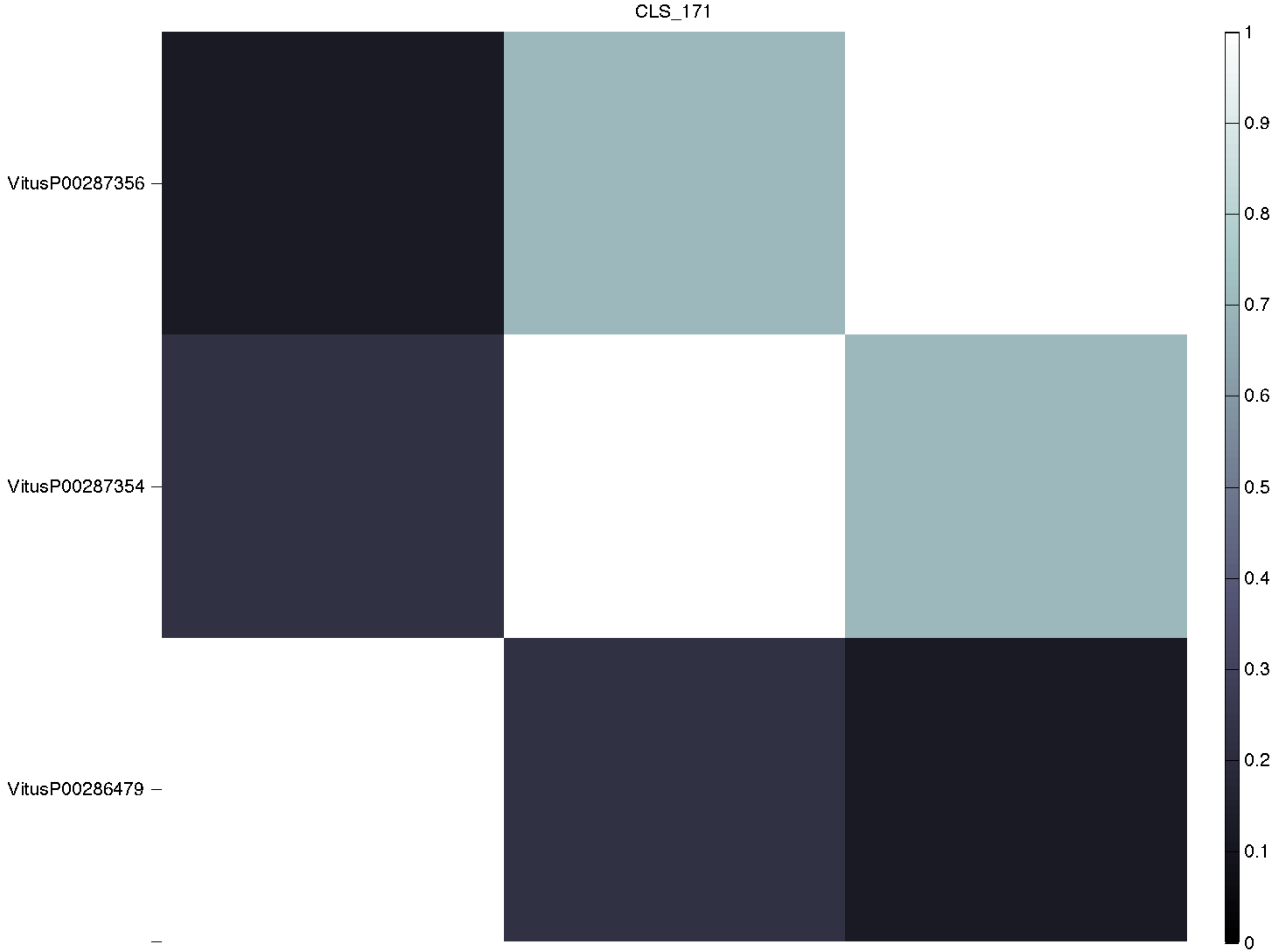
CLS_171
VitusP00287356
VitusP00287354
VitusP00286479
1
0.9
0.8
0.7
0.6
0.5
0.4
0.3
0.2
0.1
0

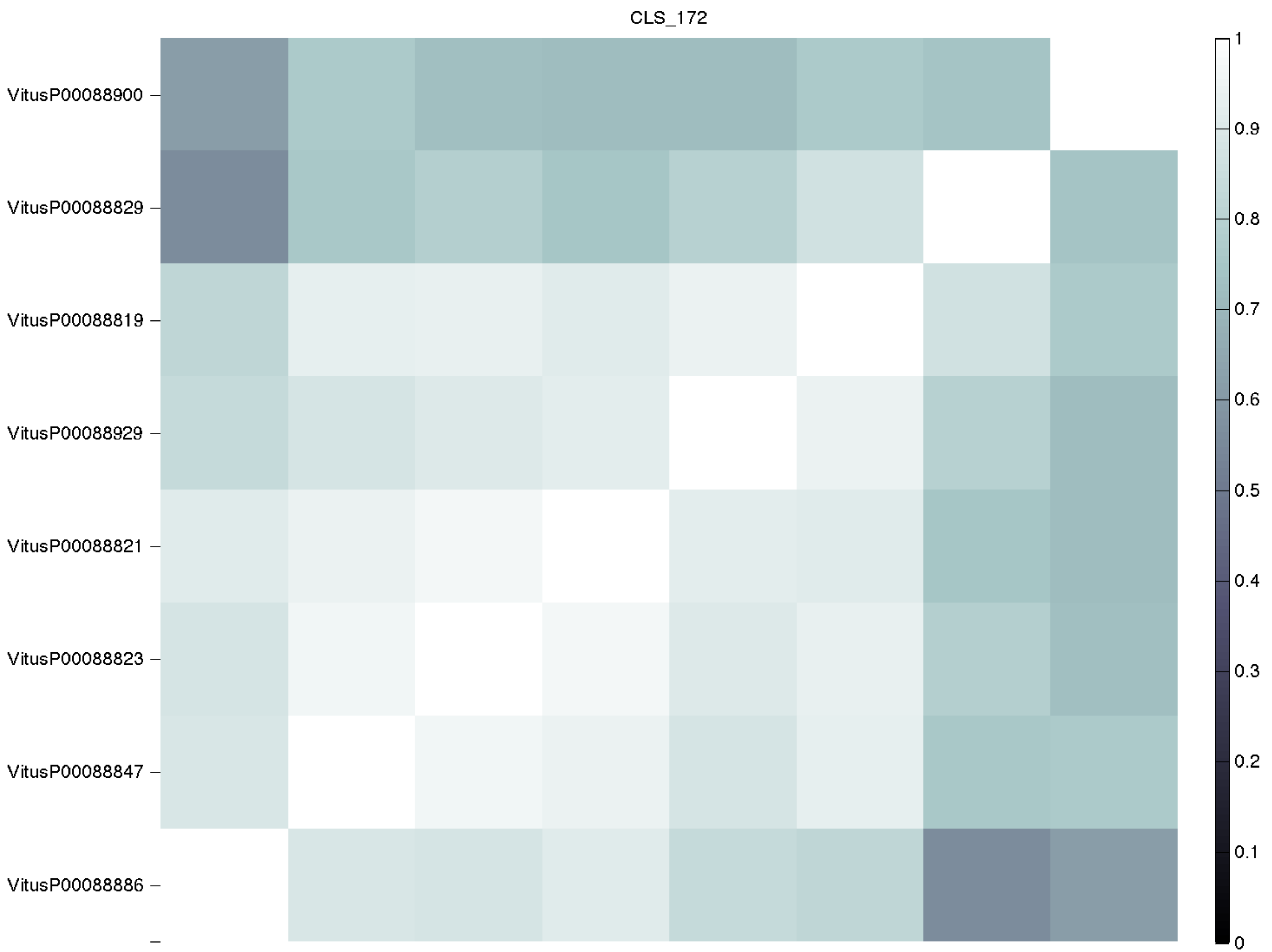
CLS_172
VitusP00088900
VitusP00088829
VitusP00088819
VitusP00088929
VitusP00088821
VitusP00088823
VitusP00088847
VitusP00088886
1
0.9
0.8
0.7
0.6
0.5
0.4
0.3
0.2
0.1
0

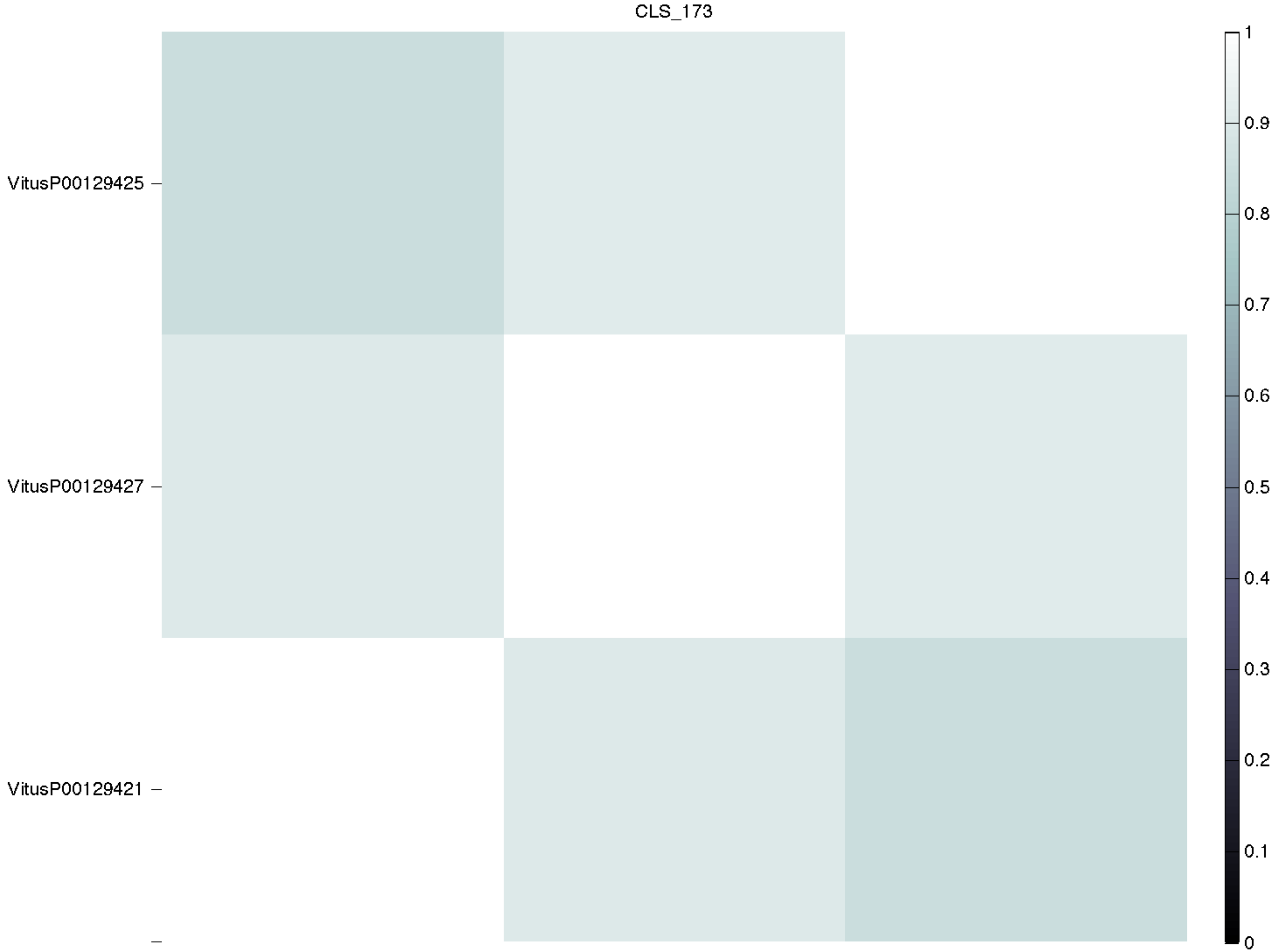

CLS_173
VitusP00129425
VitusP00129427
VitusP00129421
1
0.9
0.8
0.7
0.6
0.5
0.4
0.3
0.2
0.1
0

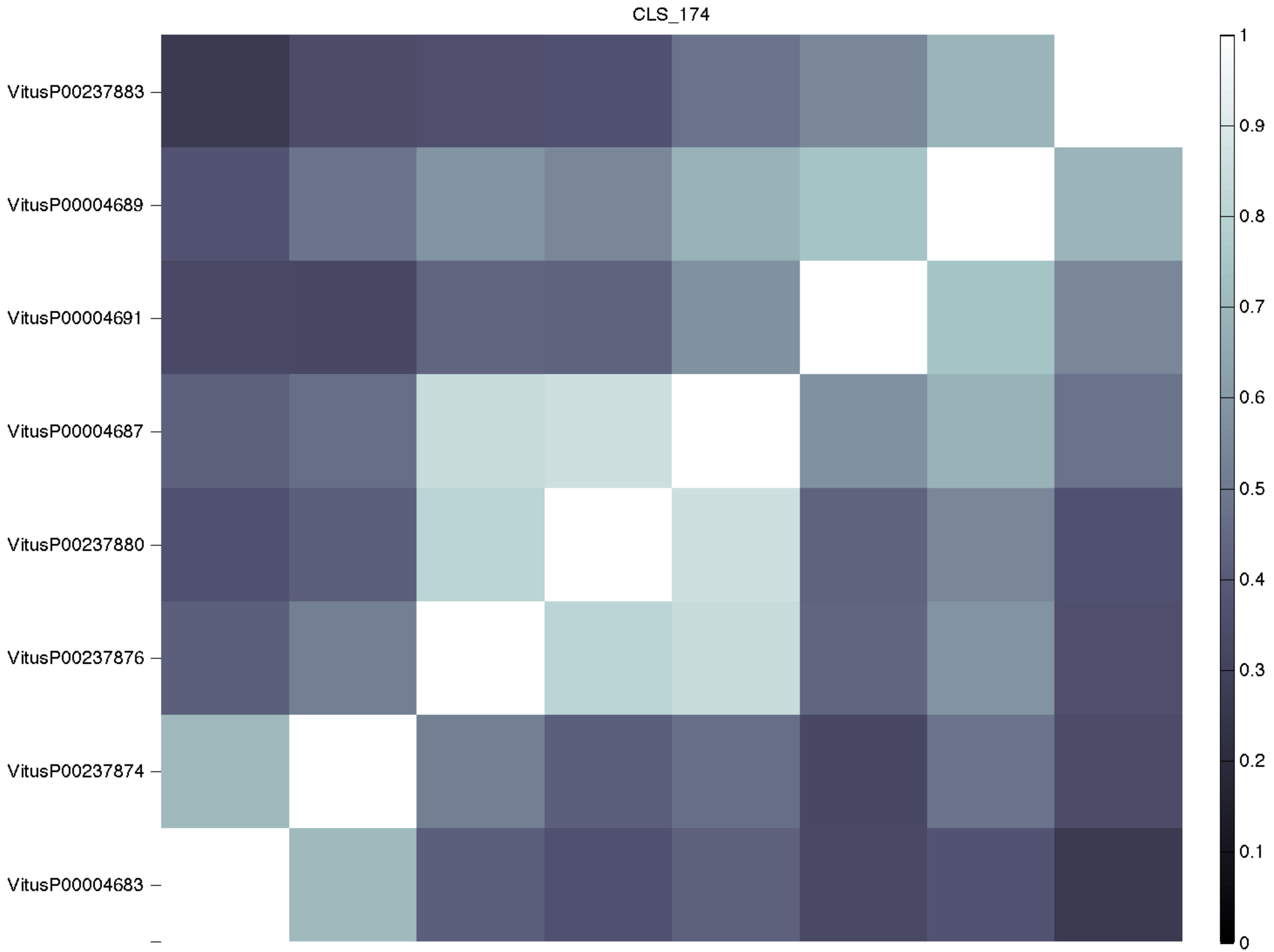

CLS_174
VitusP00237883
VitusP00004689
VitusP00004691
VitusP00004687
VitusP00237880
VitusP00237876
VitusP00237874
VitusP00004683
1
0.9
0.8
0.7
0.6
0.5
0.4
0.3
0.2
0.1
0

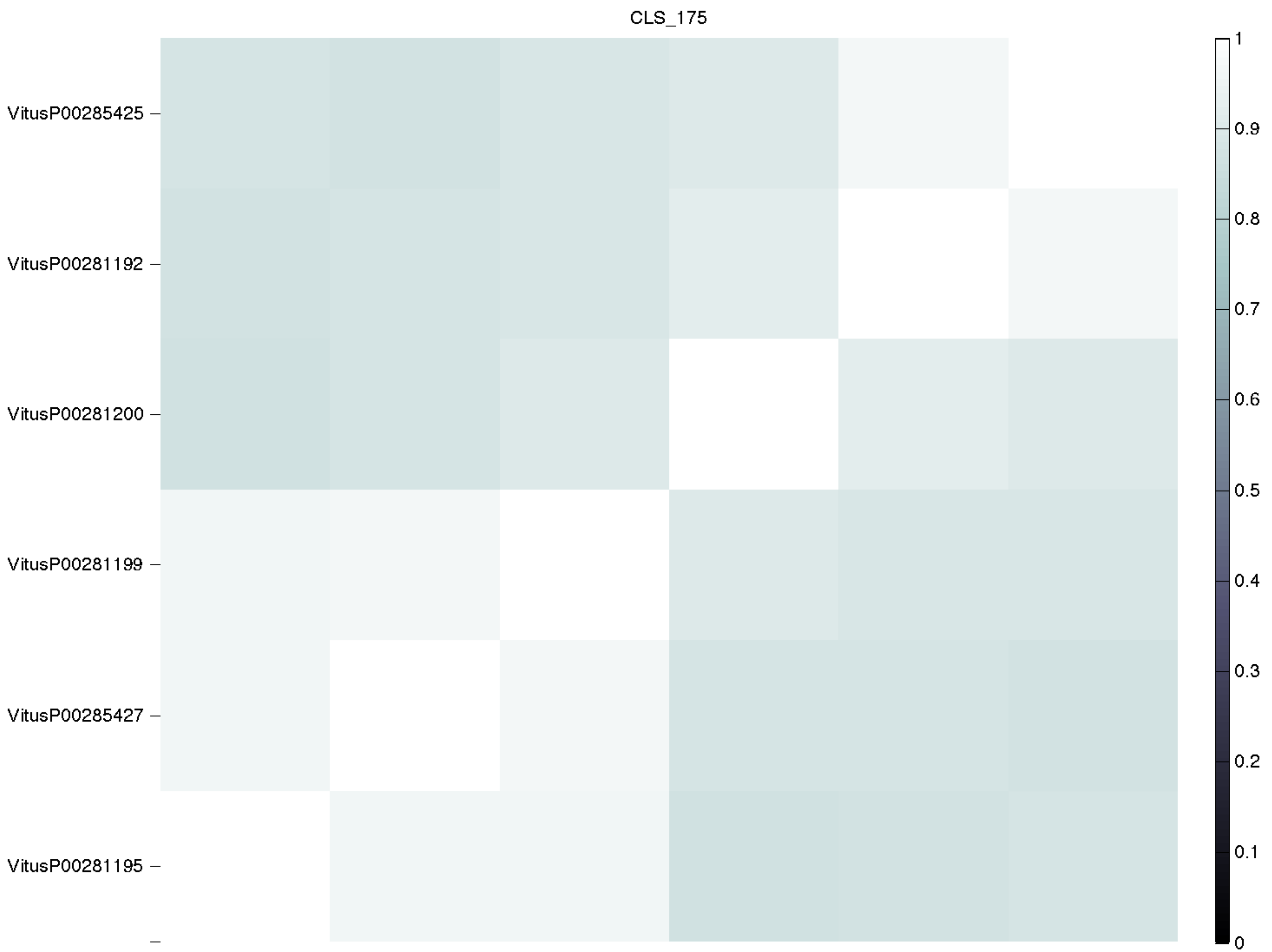
CLS_175
VitusP00285425
VitusP00281192
VitusP00281200
VitusP00281199
VitusP00285427
VitusP00281195
1
0.9
0.8
0.7
0.6
0.5
0.4
0.3
0.2
0.1
0

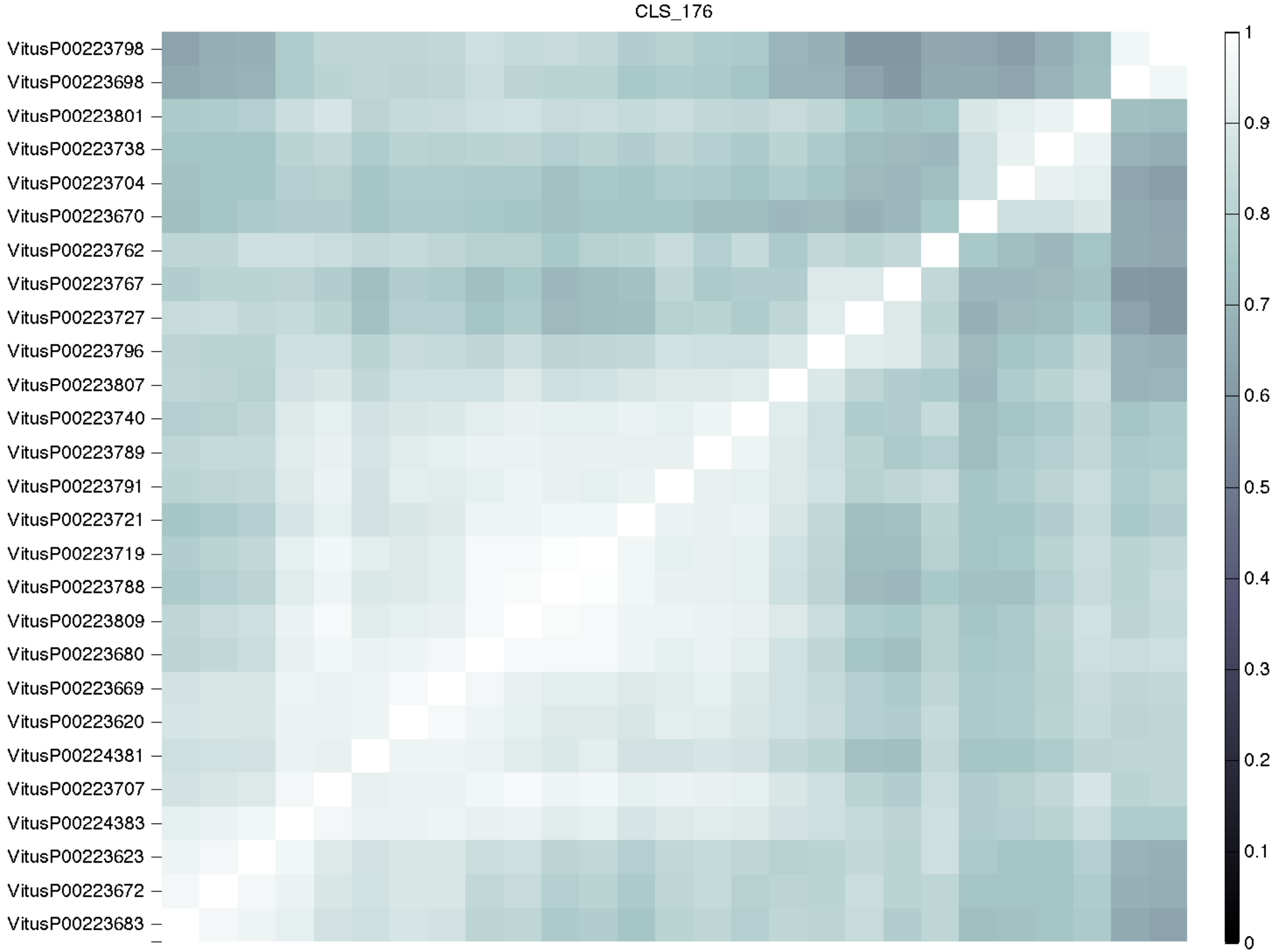

CLS_176
VitusP00223798
VitusP00223698
VitusP00223801
VitusP00223738
VitusP00223704
VitusP00223670
VitusP00223762
VitusP00223767
VitusP00223727
VitusP00223796
VitusP00223807
VitusP00223740
VitusP00223789
VitusP00223791
VitusP00223721
VitusP00223719
VitusP00223788
VitusP00223809
VitusP00223680
VitusP00223669
VitusP00223620
VitusP00224381
VitusP00223707
VitusP00224383
VitusP00223623
VitusP00223672
VitusP00223683
1
0.9
0.8
0.7
0.6
0.5
0.4
0.3
0.2
0.1
0

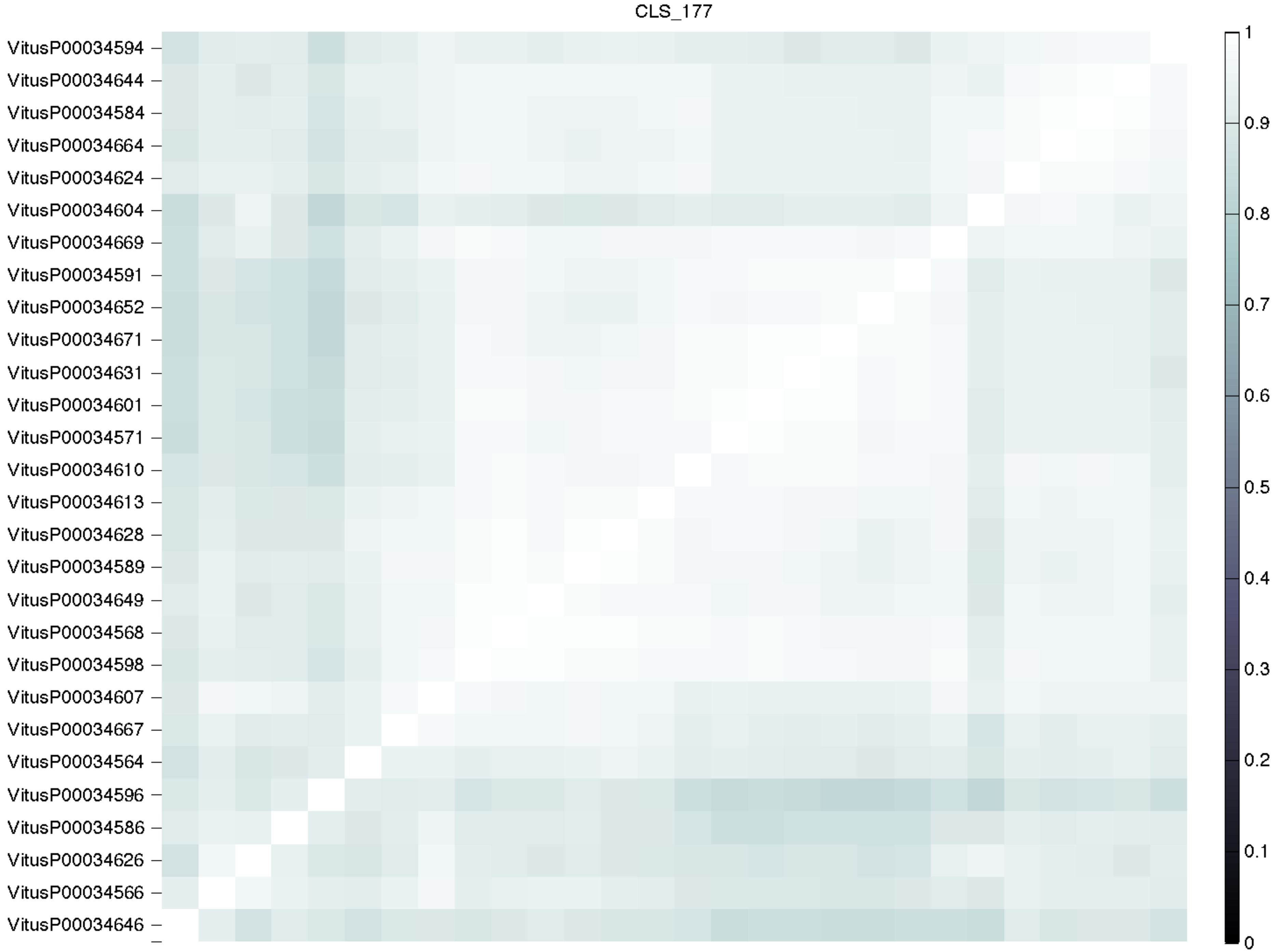

CLS_177
VitusP00034594
VitusP00034644
VitusP00034584
VitusP00034664
VitusP00034624
VitusP00034604
VitusP00034669
VitusP00034591
VitusP00034652
VitusP00034671
VitusP00034631
VitusP00034601
VitusP00034571
VitusP00034610
VitusP00034613
VitusP00034628
VitusP00034589
VitusP00034649
VitusP00034568
VitusP00034598
VitusP00034607
VitusP00034667
VitusP00034564
VitusP00034596
VitusP00034586
VitusP00034626
VitusP00034566
VitusP00034646
1
0.9
0.8
0.7
0.6
0.5
0.4
0.3
0.2
0.1
0

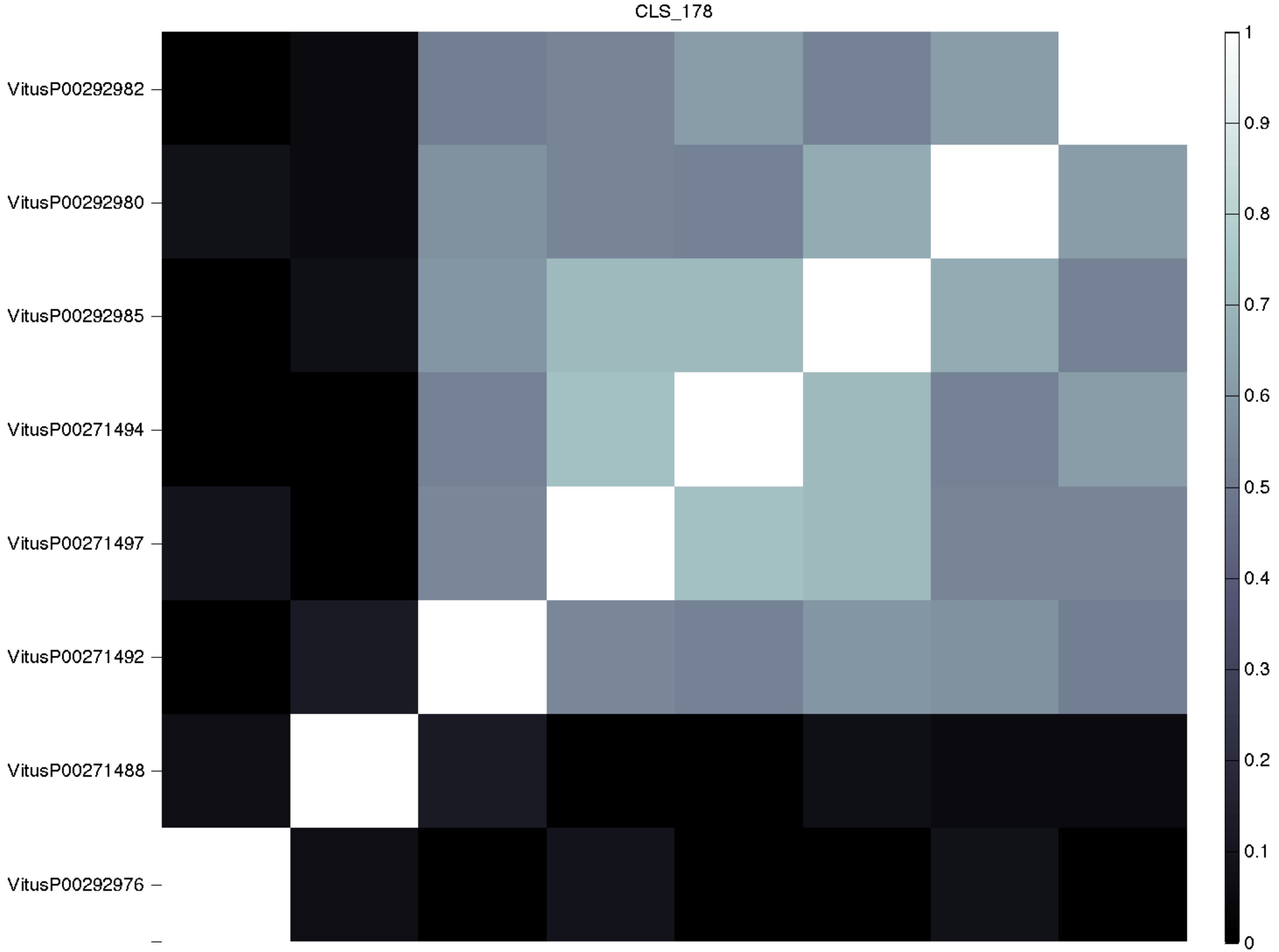
CLS_178
VitusP00292982
VitusP00292980
VitusP00292985
VitusP00271494
VitusP00271497
VitusP00271492
VitusP00271488
VitusP00292976
1
0.9
0.8
0.7
0.6
0.5
0.4
0.3
0.2
0.1
0

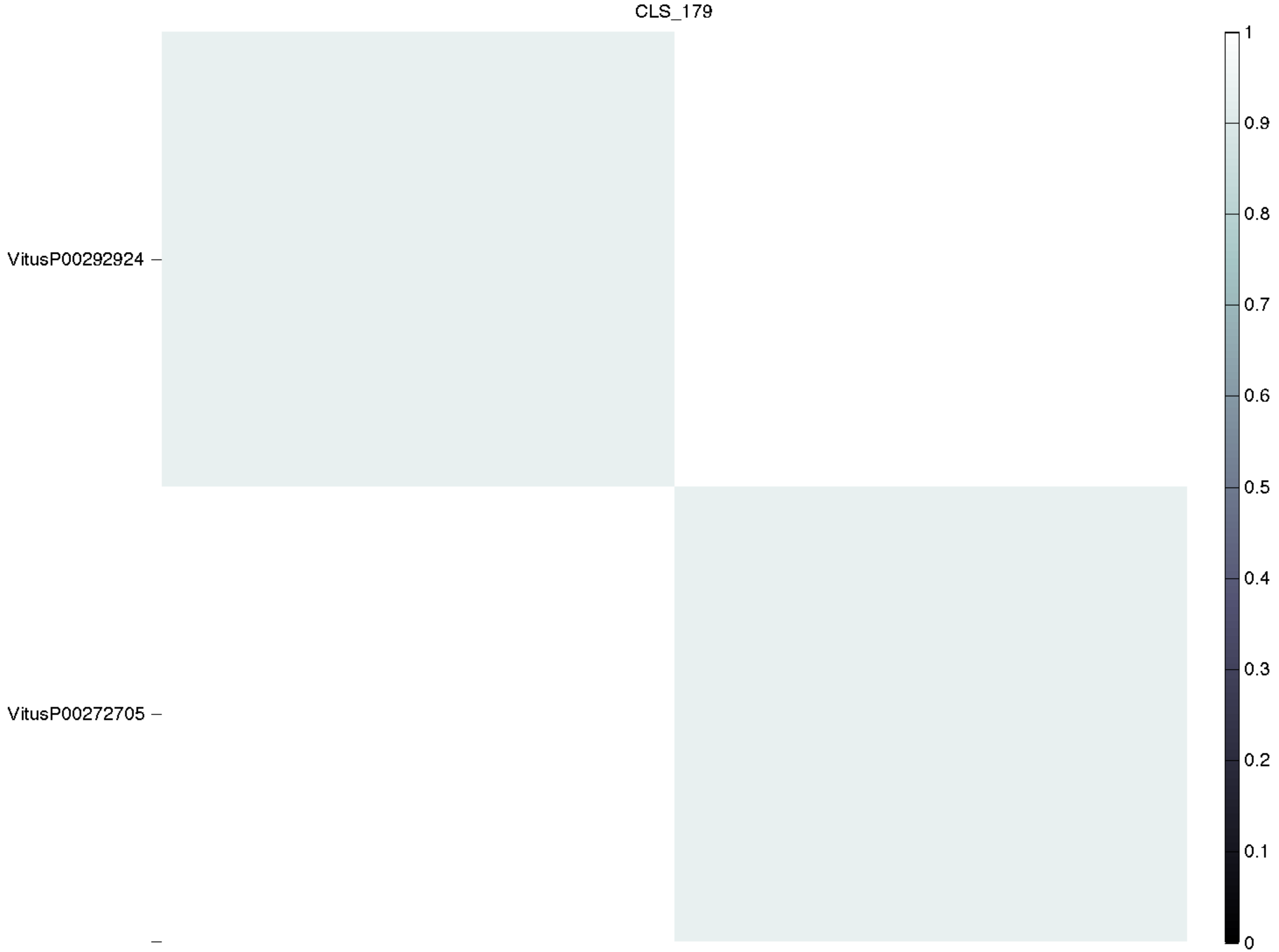
CLS_179
VitusP00292924
VitusP00272705
1
0.9
0.8
0.7
0.6
0.5
0.4
0.3
0.2
0.1
0

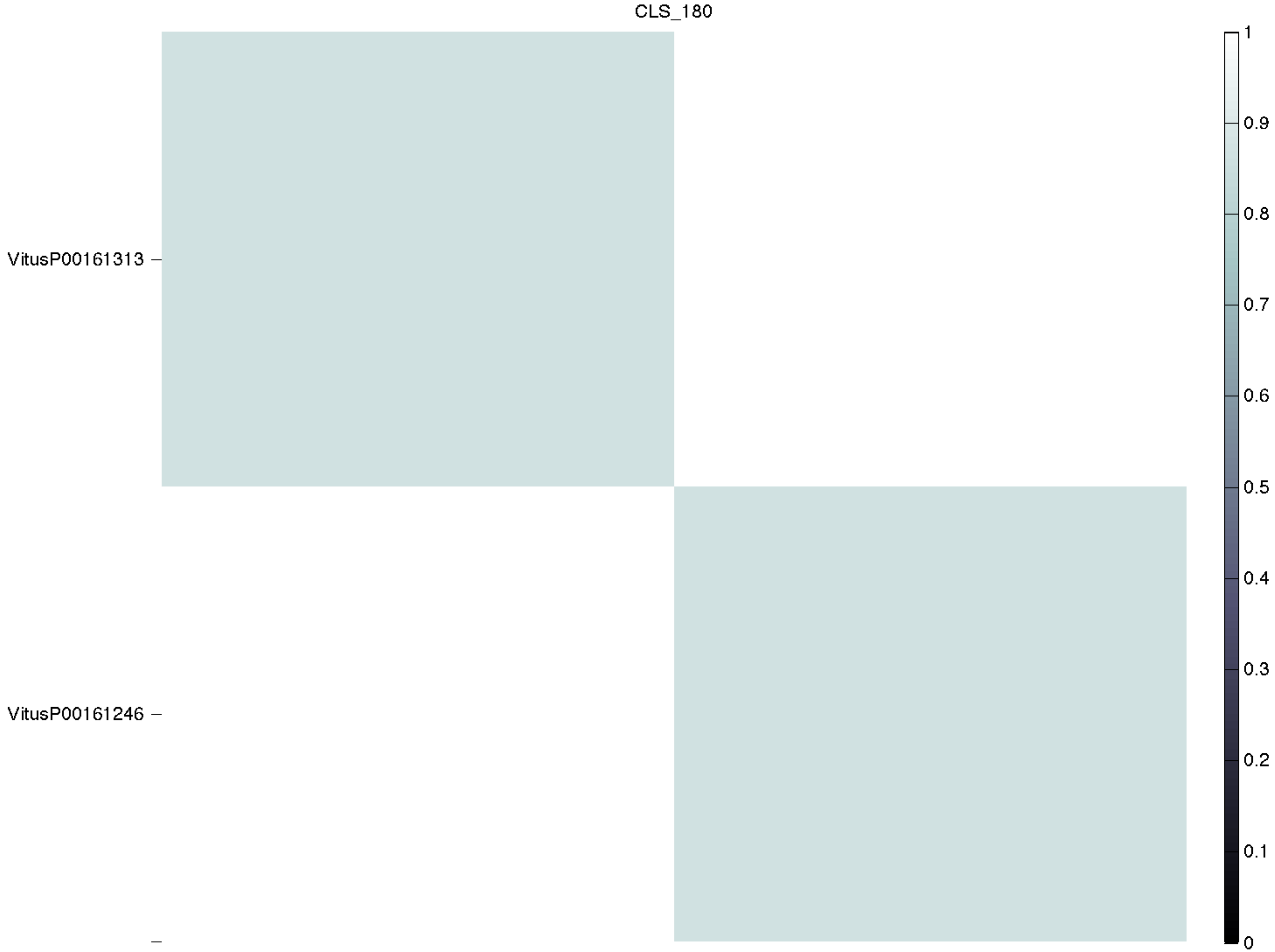
CLS_180
VitusP00161313
VitusP00161246
1
0.9
0.8
0.7
0.6
0.5
0.4
0.3
0.2
0.1
0

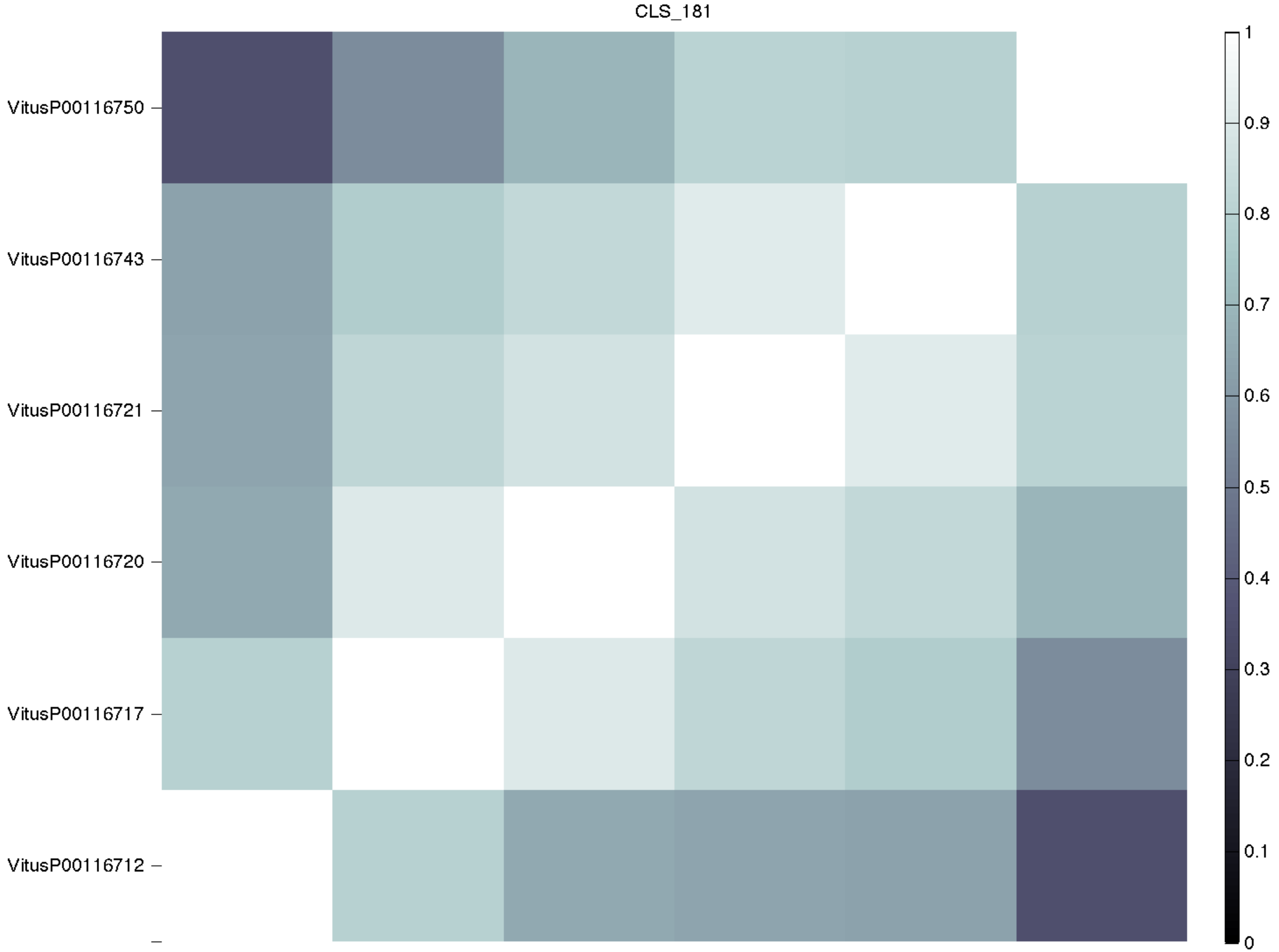

CLS_181
VitusP00116750
VitusP00116743
VitusP00116721
VitusP00116720
VitusP00116717
VitusP00116712
1
0.9
0.8
0.7
0.6
0.5
0.4
0.3
0.2
0.1
0

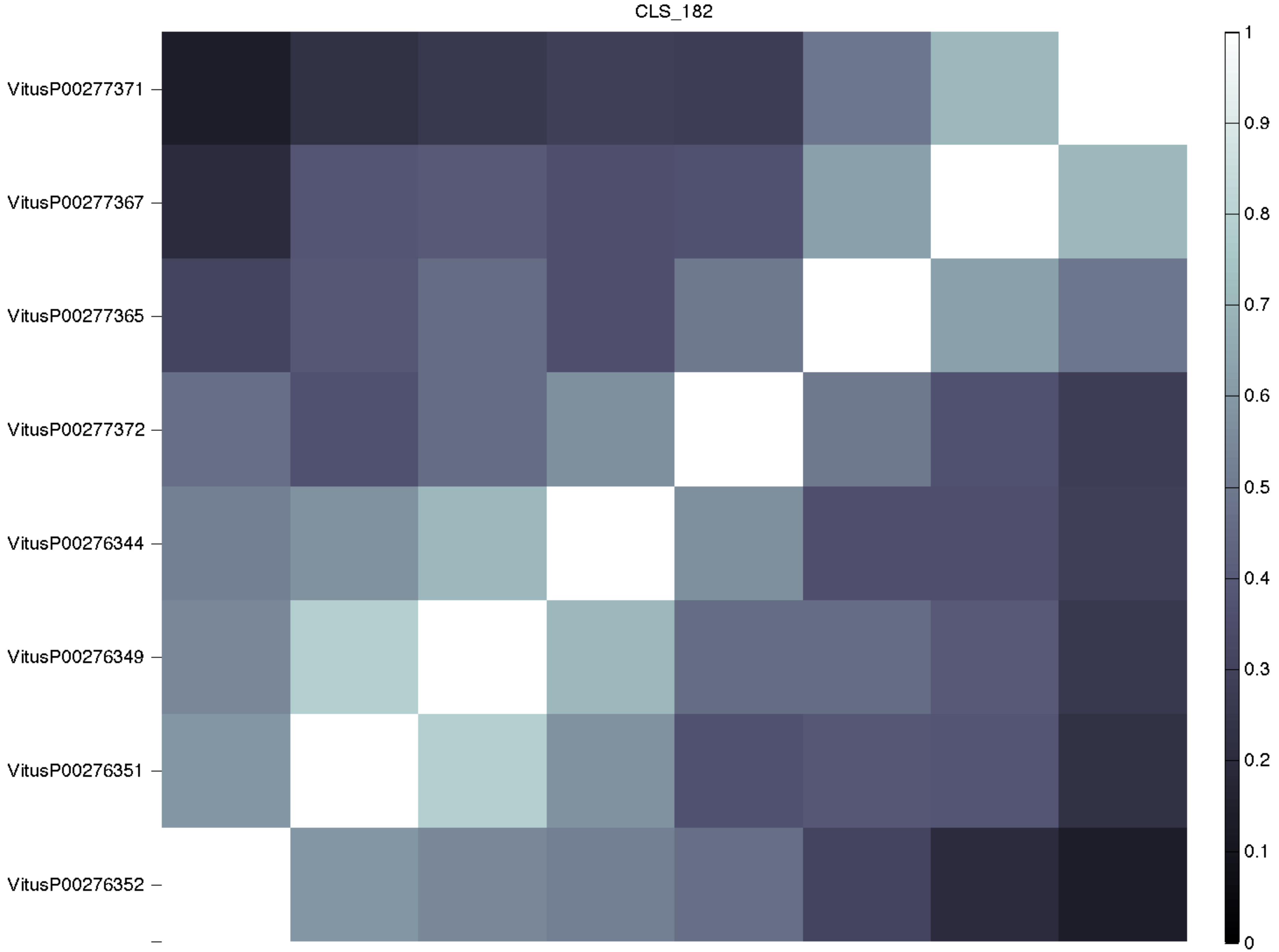
CLS_182
VitusP00277371
VitusP00277367
VitusP00277365
VitusP00277372
VitusP00276344
VitusP00276349
VitusP00276351
VitusP00276352
1
0.9
0.8
0.7
0.6
0.5
0.4
0.3
0.2
0.1
0

CLS_183

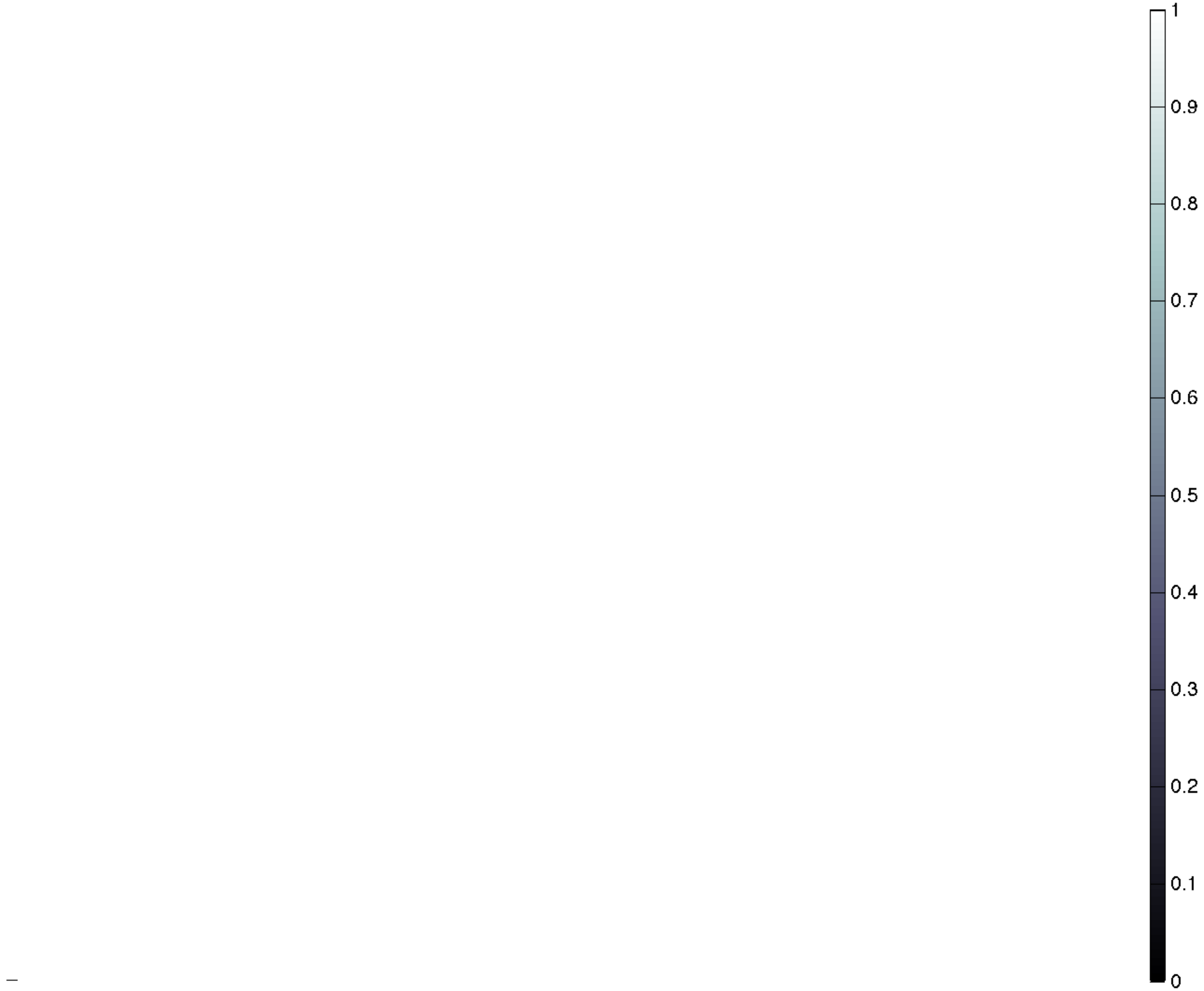

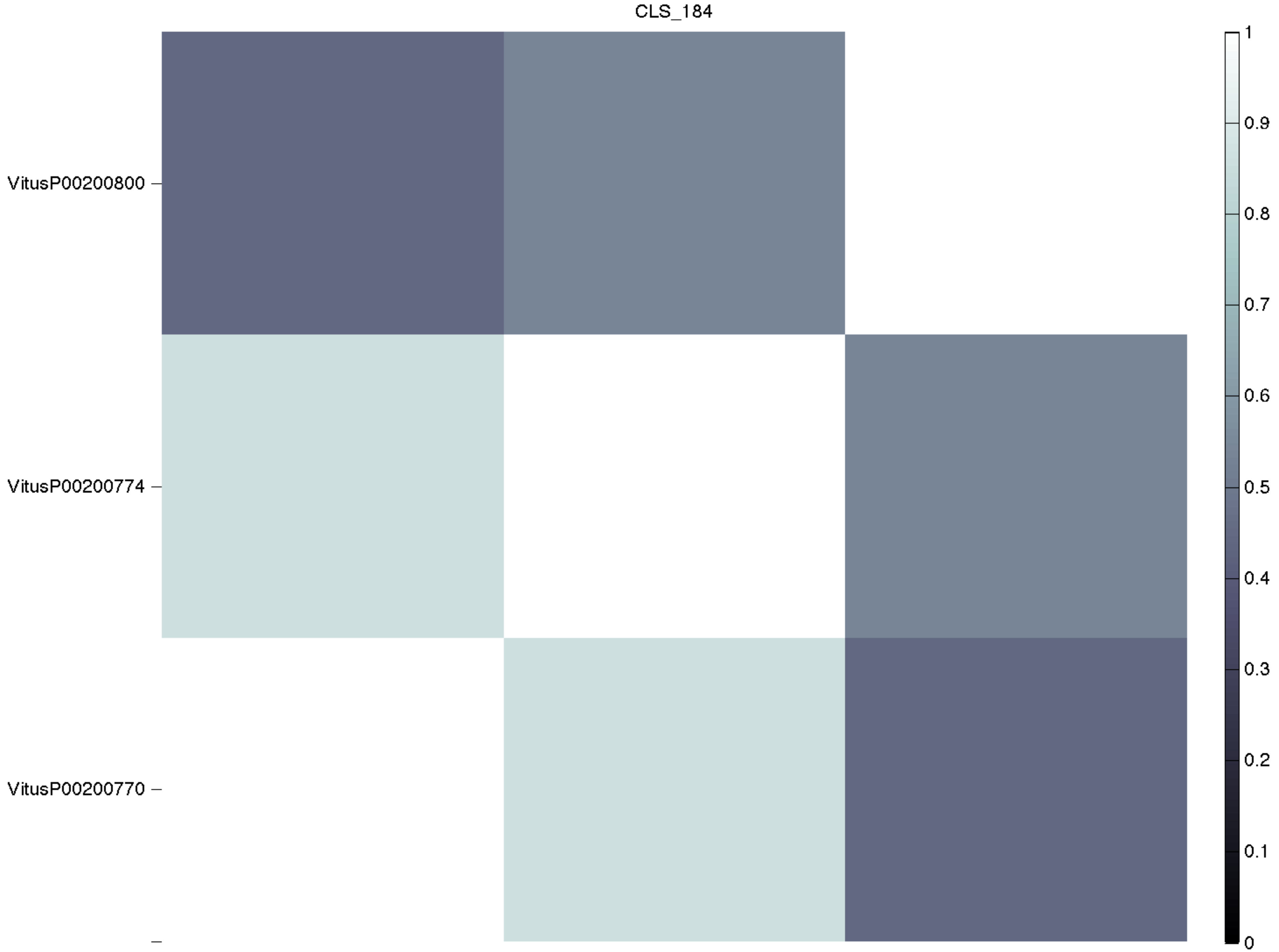
CLS_184
VitusP00200800
VitusP00200774
VitusP00200770
1
0.9
0.8
0.7
0.6
0.5
0.4
0.3
0.2
0.1
0

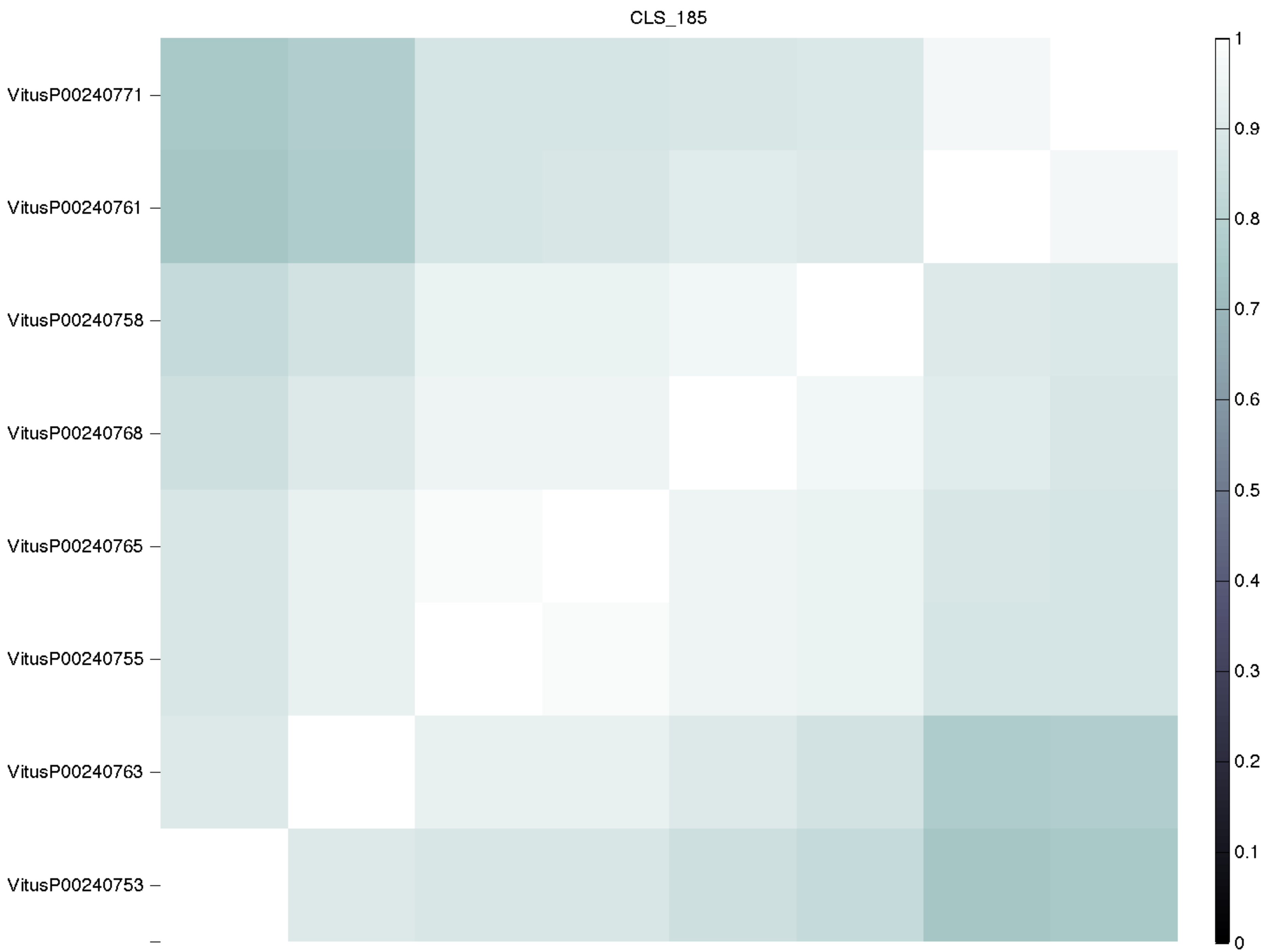
CLS_185
VitusP00240771
VitusP00240761
VitusP00240758
VitusP00240768
VitusP00240765
VitusP00240755
VitusP00240763
VitusP00240753
1
0.9
0.8
0.7
0.6
0.5
0.4
0.3
0.2
0.1
0

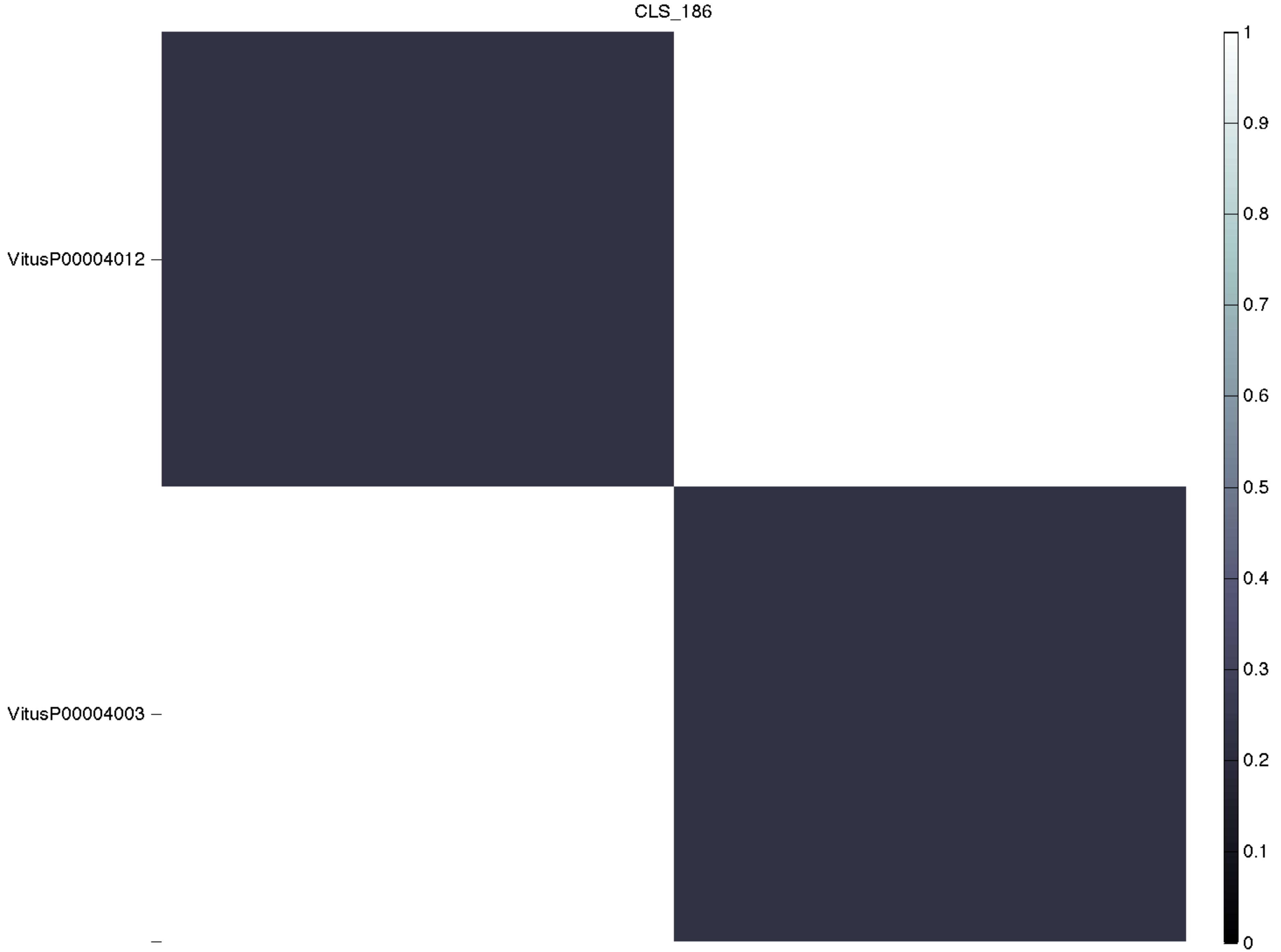
CLS_186
VitusP00004012
VitusP00004003
1
0.9
0.8
0.7
0.6
0.5
0.4
0.3
0.2
0.1
0

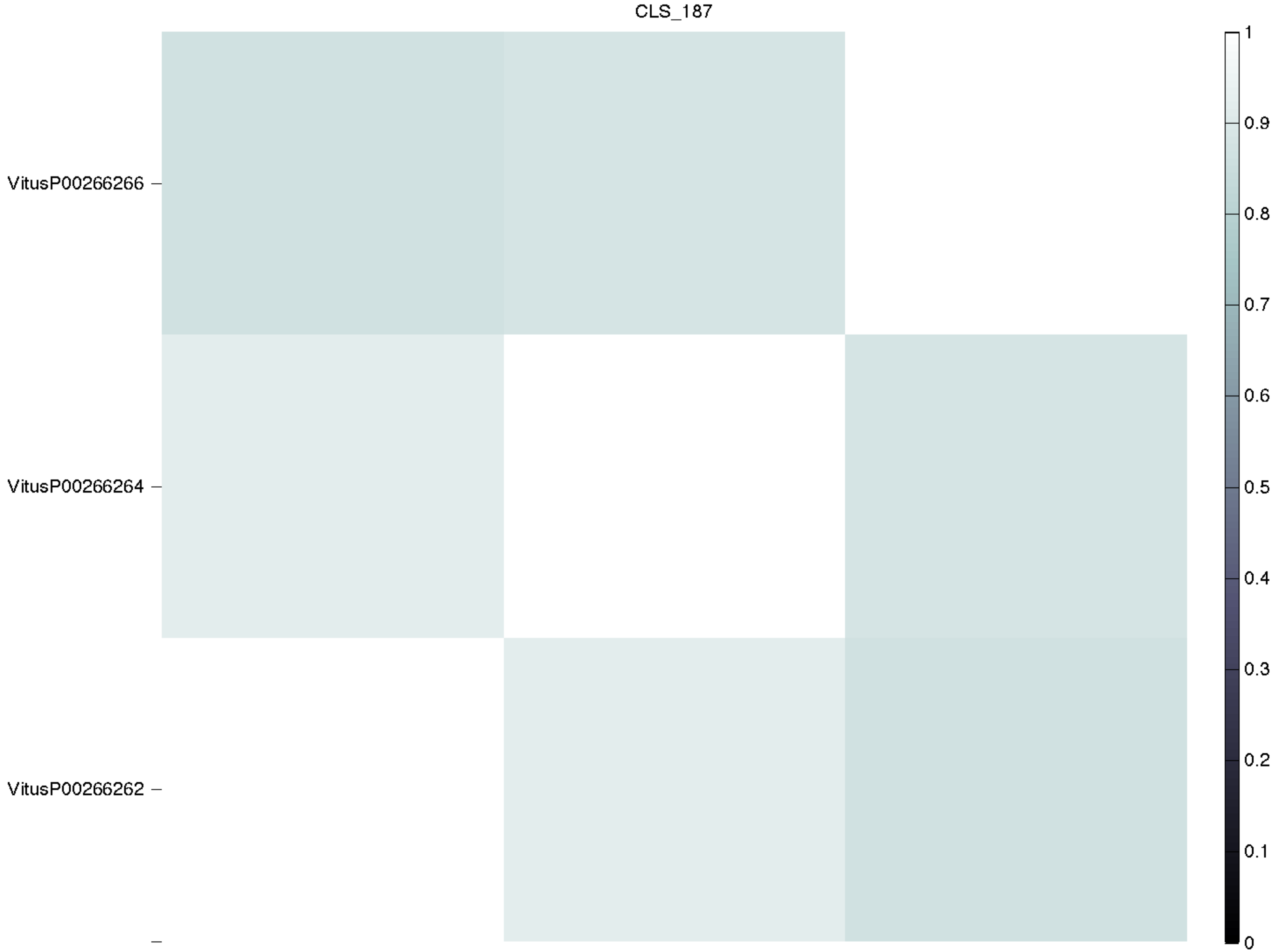
CLS_187
VitusP00266266
VitusP00266264
VitusP00266262
1
0.9
0.8
0.7
0.6
0.5
0.4
0.3
0.2
0.1
0

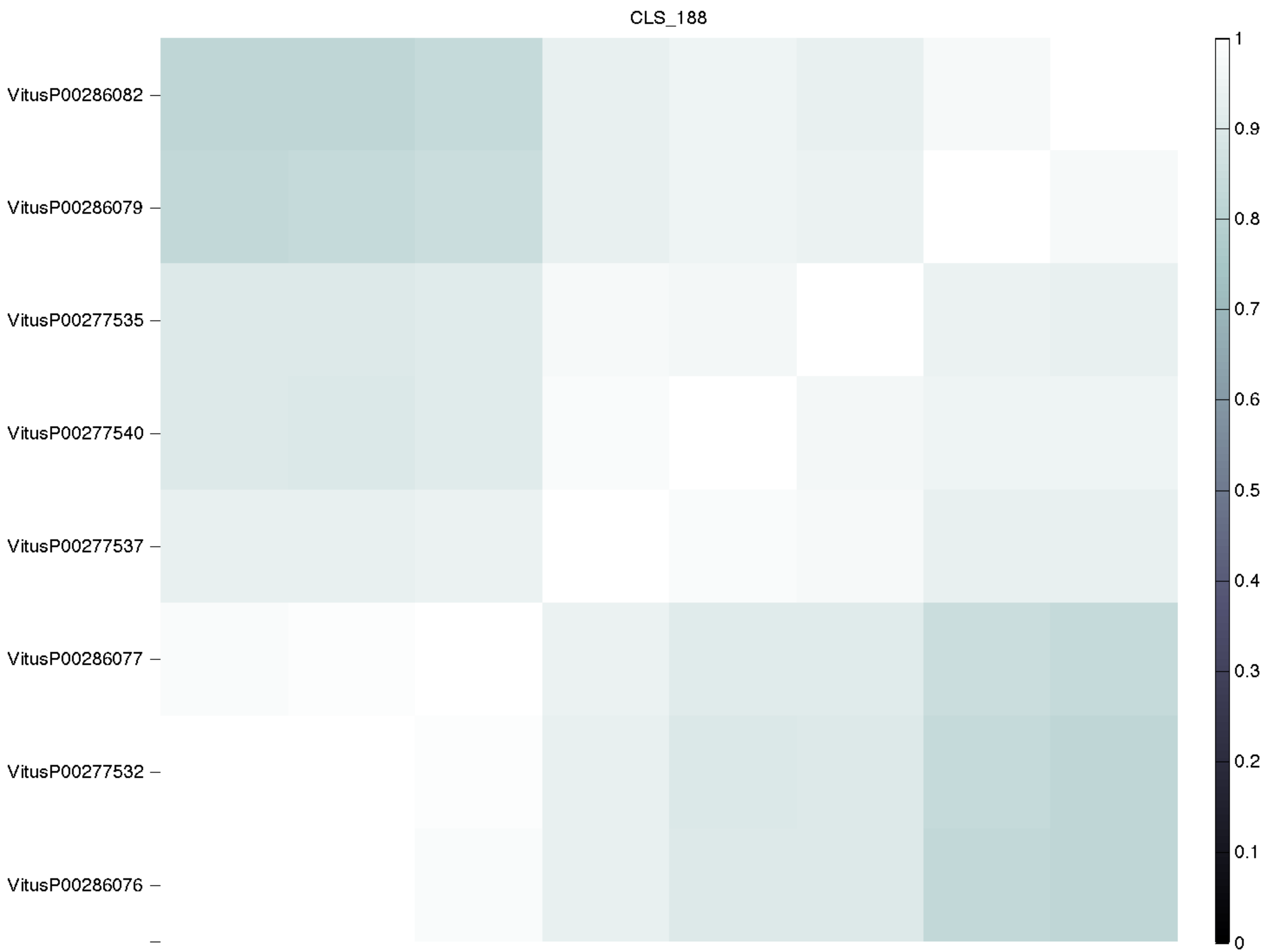
CLS_188
VitusP00286082
VitusP00286079
VitusP00277535
VitusP00277540
VitusP00277537
VitusP00286077
VitusP00277532
VitusP00286076
1
0.9
0.8
0.7
0.6
0.5
0.4
0.3
0.2
0.1
0

CLS_189

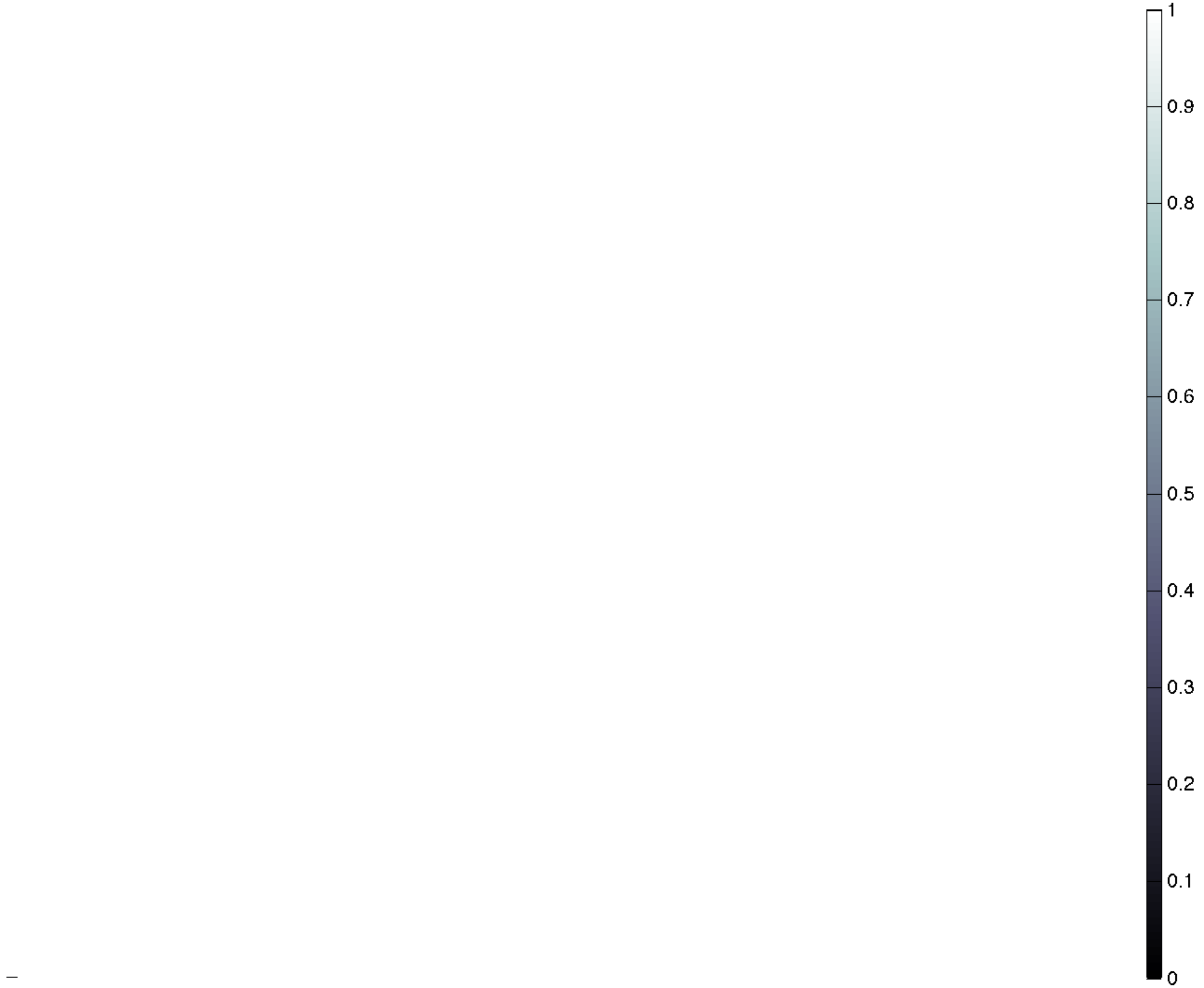

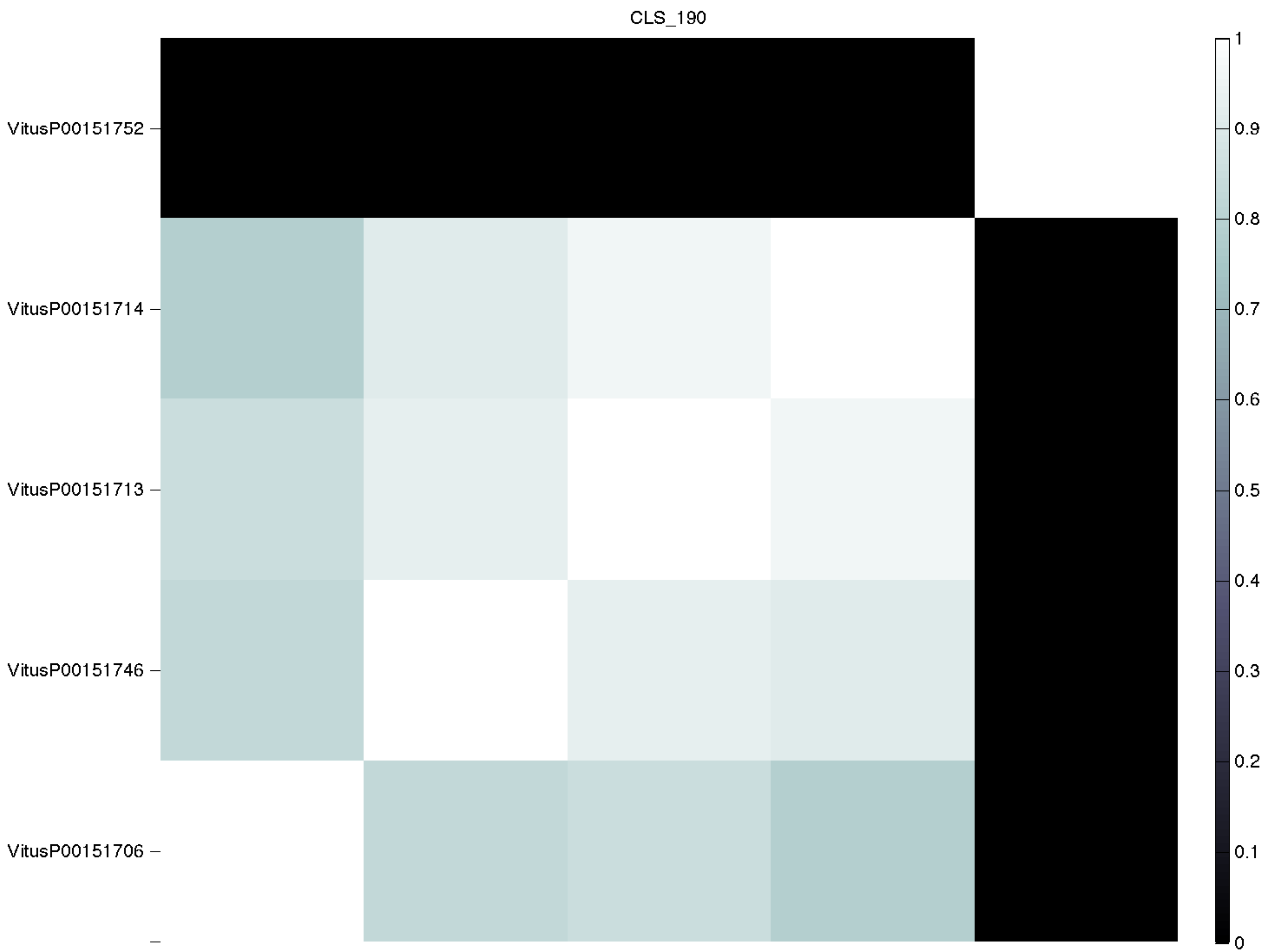

CLS_190
VitusP00151752
VitusP00151714
VitusP00151713
VitusP00151746
VitusP00151706
1
0.9
0.8
0.7
0.6
0.5
0.4
0.3
0.2
0.1
0

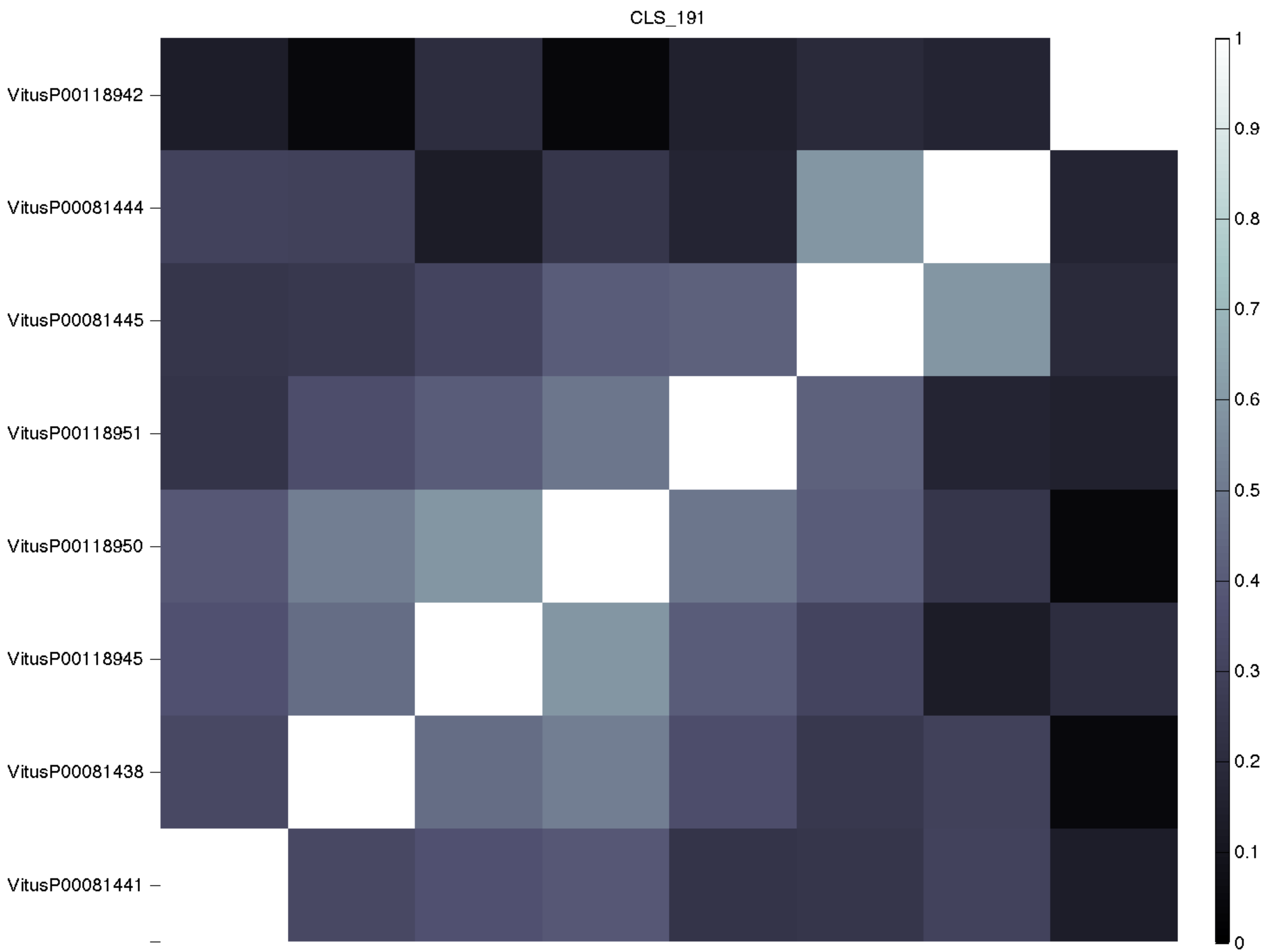

CLS_191
VitusP00118942
VitusP00081444
VitusP00081445
VitusP00118951
VitusP00118950
VitusP00118945
VitusP00081438
VitusP00081441
1
0.9
0.8
0.7
0.6
0.5
0.4
0.3
0.2
0.1
0

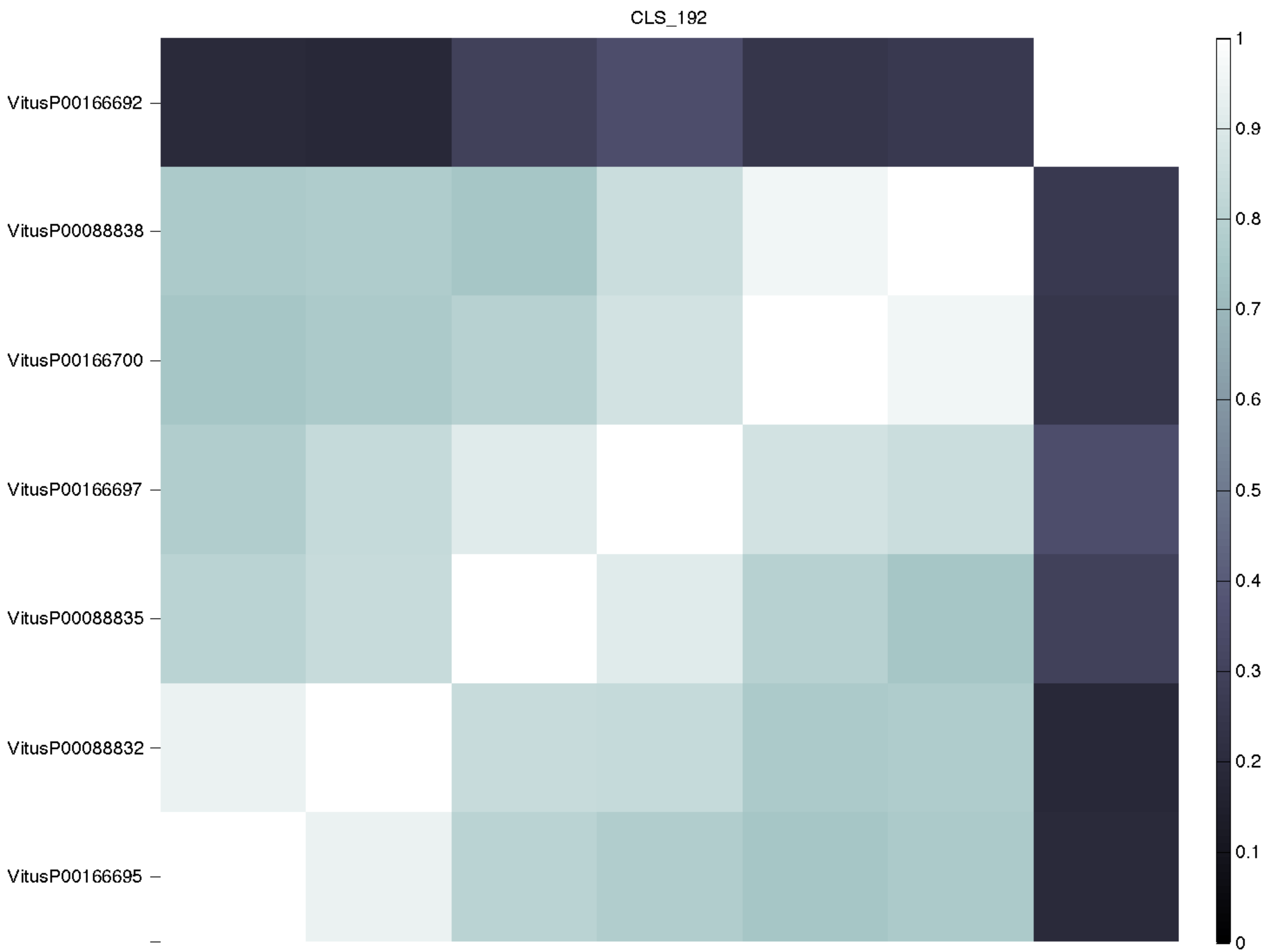

CLS_192
VitusP00166692
VitusP00088838
VitusP00166700
VitusP00166697
VitusP00088835
VitusP00088832
VitusP00166695
1
0.9
0.8
0.7
0.6
0.5
0.4
0.3
0.2
0.1
0

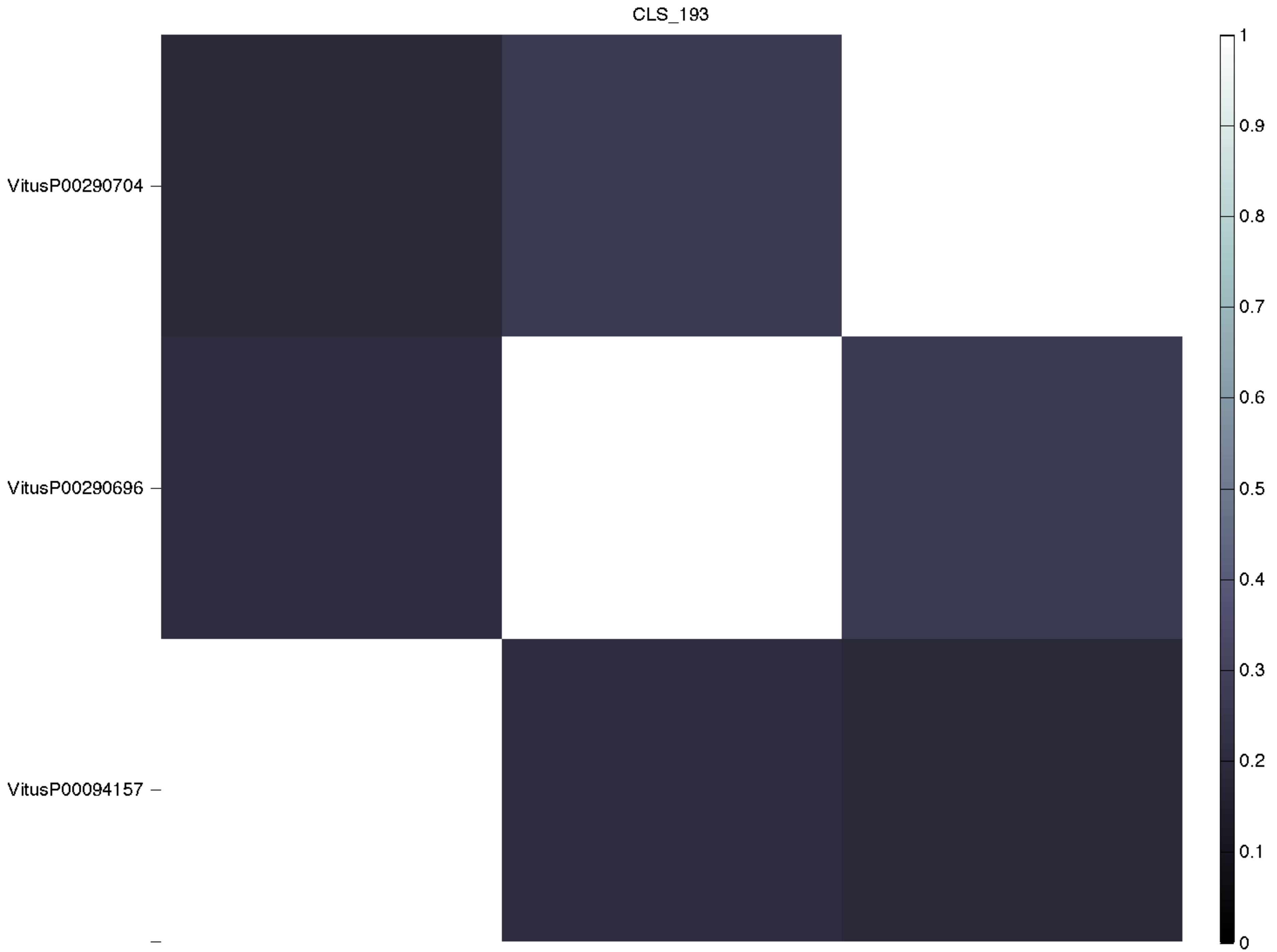
CLS_193
VitusP00290704
VitusP00290696
VitusP00094157
1
0.9
0.8
0.7
0.6
0.5
0.4
0.3
0.2
0.1
0

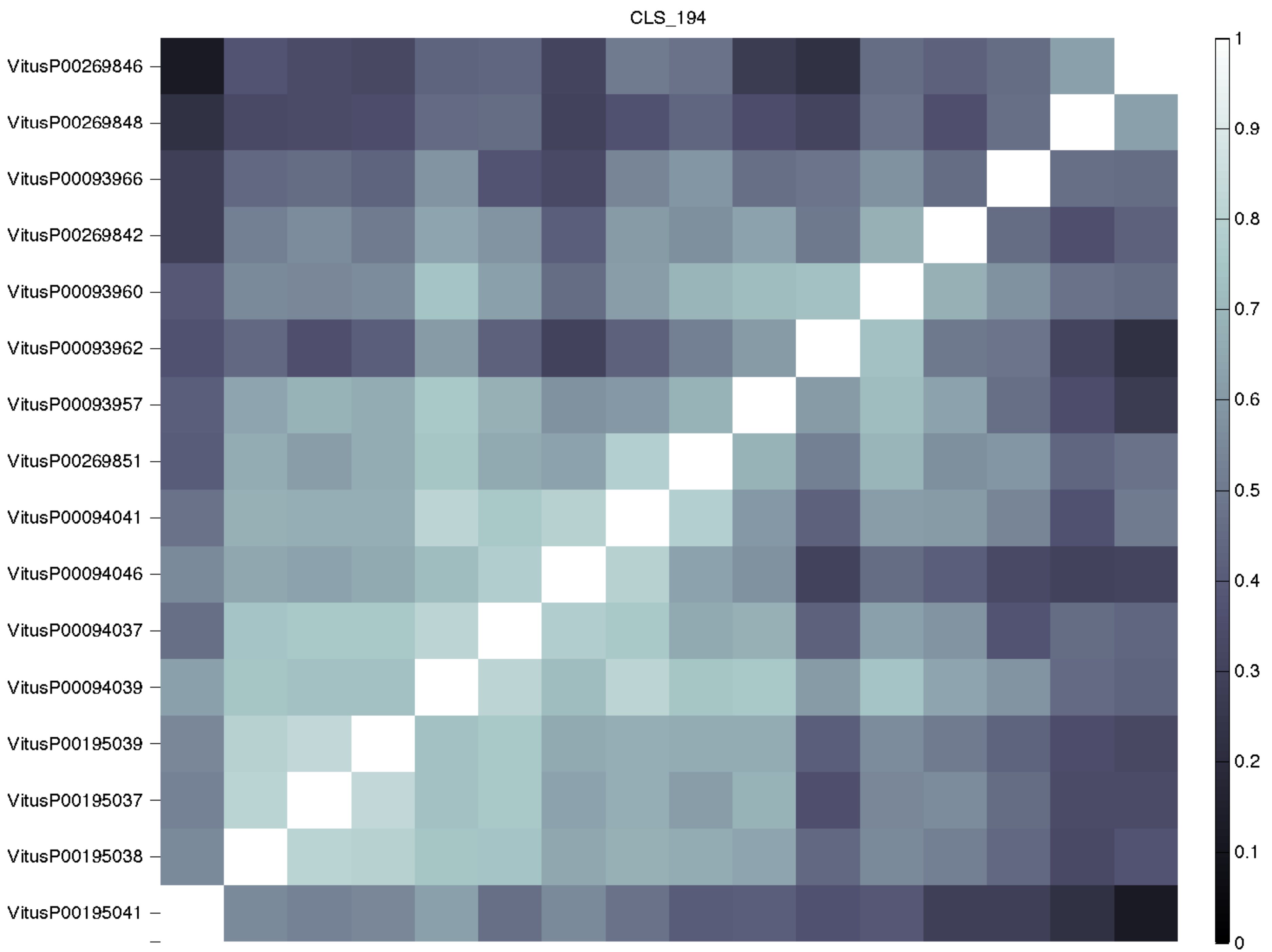
CLS_194
VitusP00269846
VitusP00269848
VitusP00093966
VitusP00269842
VitusP00093960
VitusP00093962
VitusP00093957
VitusP00269851
VitusP00094041
VitusP00094046
VitusP00094037
VitusP00094039
VitusP00195039
VitusP00195037
VitusP00195038
VitusP00195041
1
0.9
0.8
0.7
0.6
0.5
0.4
0.3
0.2
0.1
0

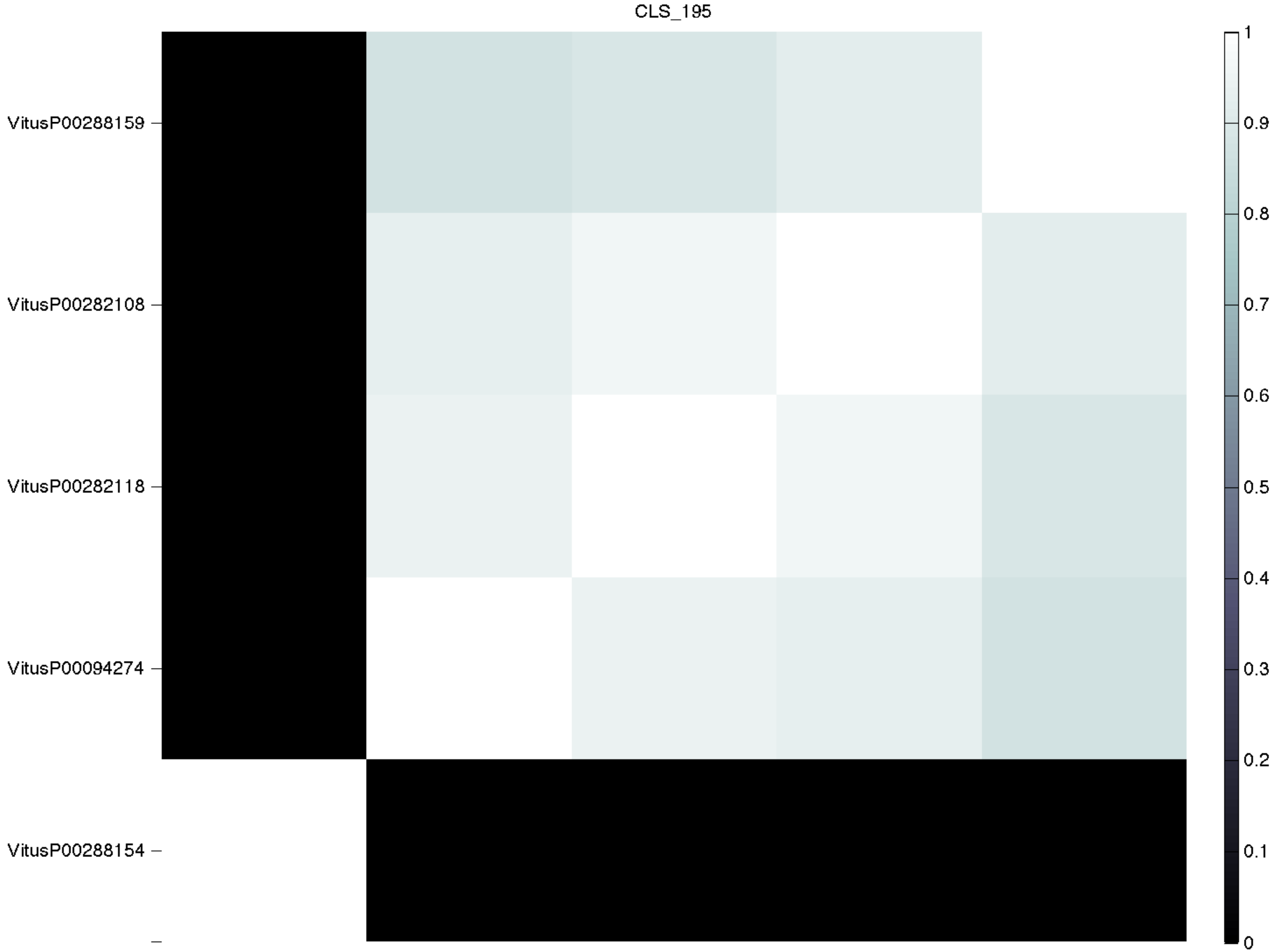

CLS_195
VitusP00288159
VitusP00282108
VitusP00282118
VitusP00094274
VitusP00288154
1
0.9
0.8
0.7
0.6
0.5
0.4
0.3
0.2
0.1
0

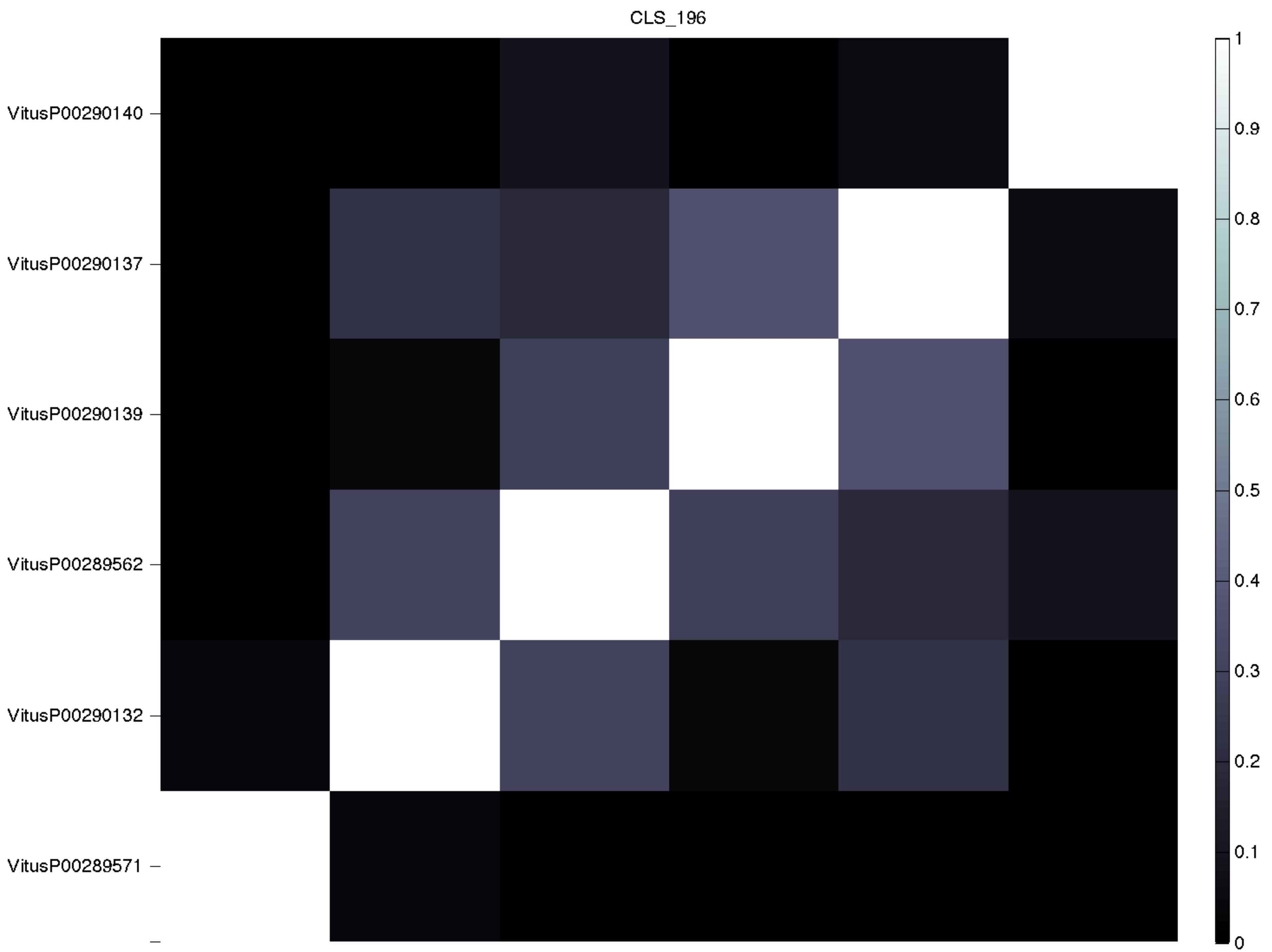
CLS_196
VitusP00290140
VitusP00290137
VitusP00290139
VitusP00289562
VitusP00290132
VitusP00289571
1
0.9
0.8
0.7
0.6
0.5
0.4
0.3
0.2
0.1
0

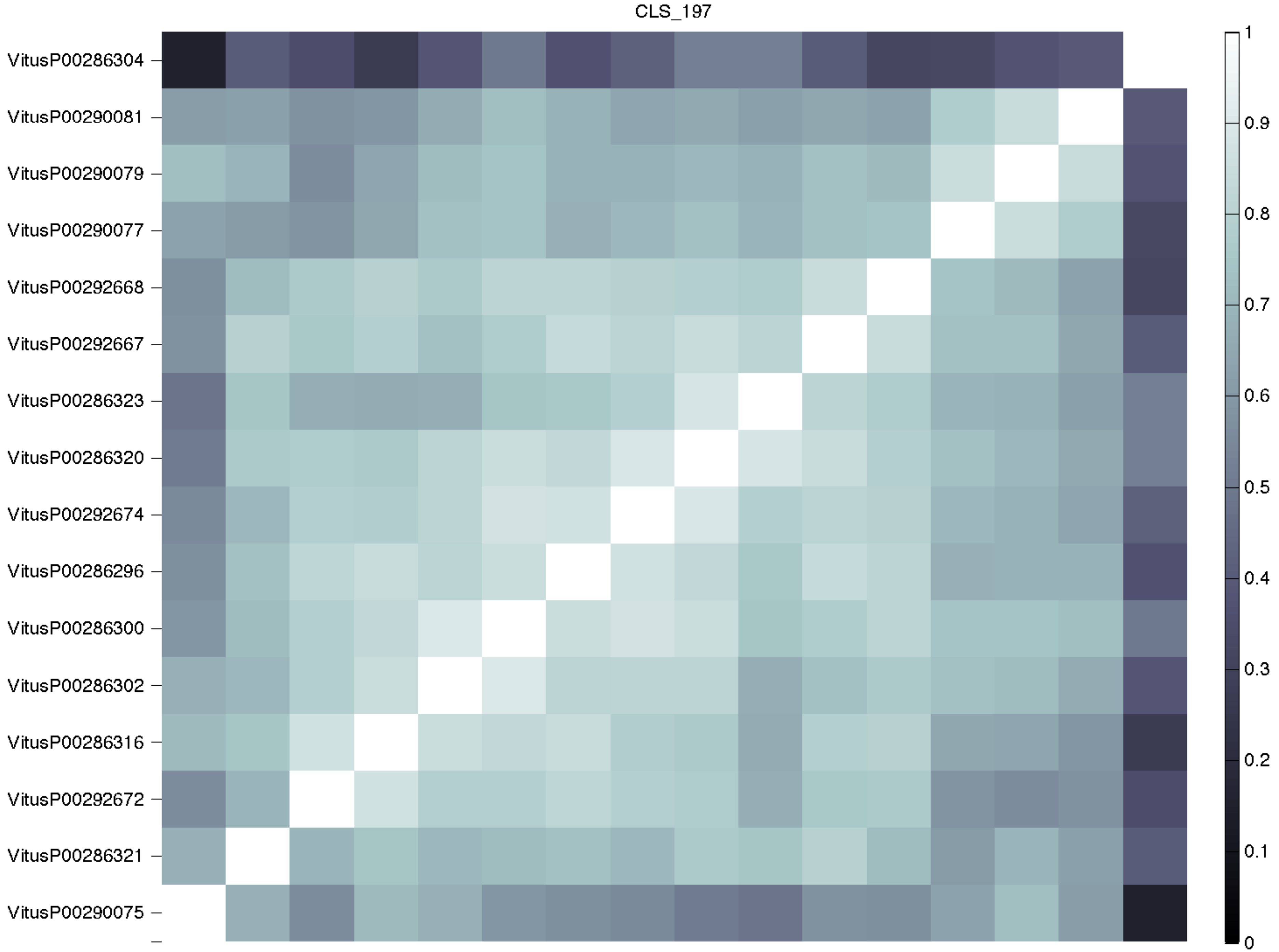
CLS_197
VitusP00286304
VitusP00290081
VitusP00290079
VitusP00290077
VitusP00292668
VitusP00292667
VitusP00286323
VitusP00286320
VitusP00292674
VitusP00286296
VitusP00286300
VitusP00286302
VitusP00286316
VitusP00292672
VitusP00286321
VitusP00290075
1
0.9
0.8
0.7
0.6
0.5
0.4
0.3
0.2
0.1
0

CLS_198

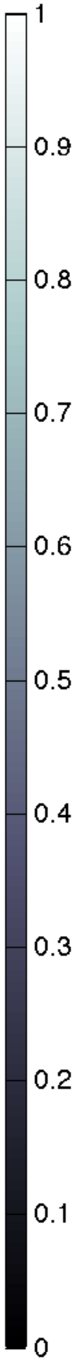

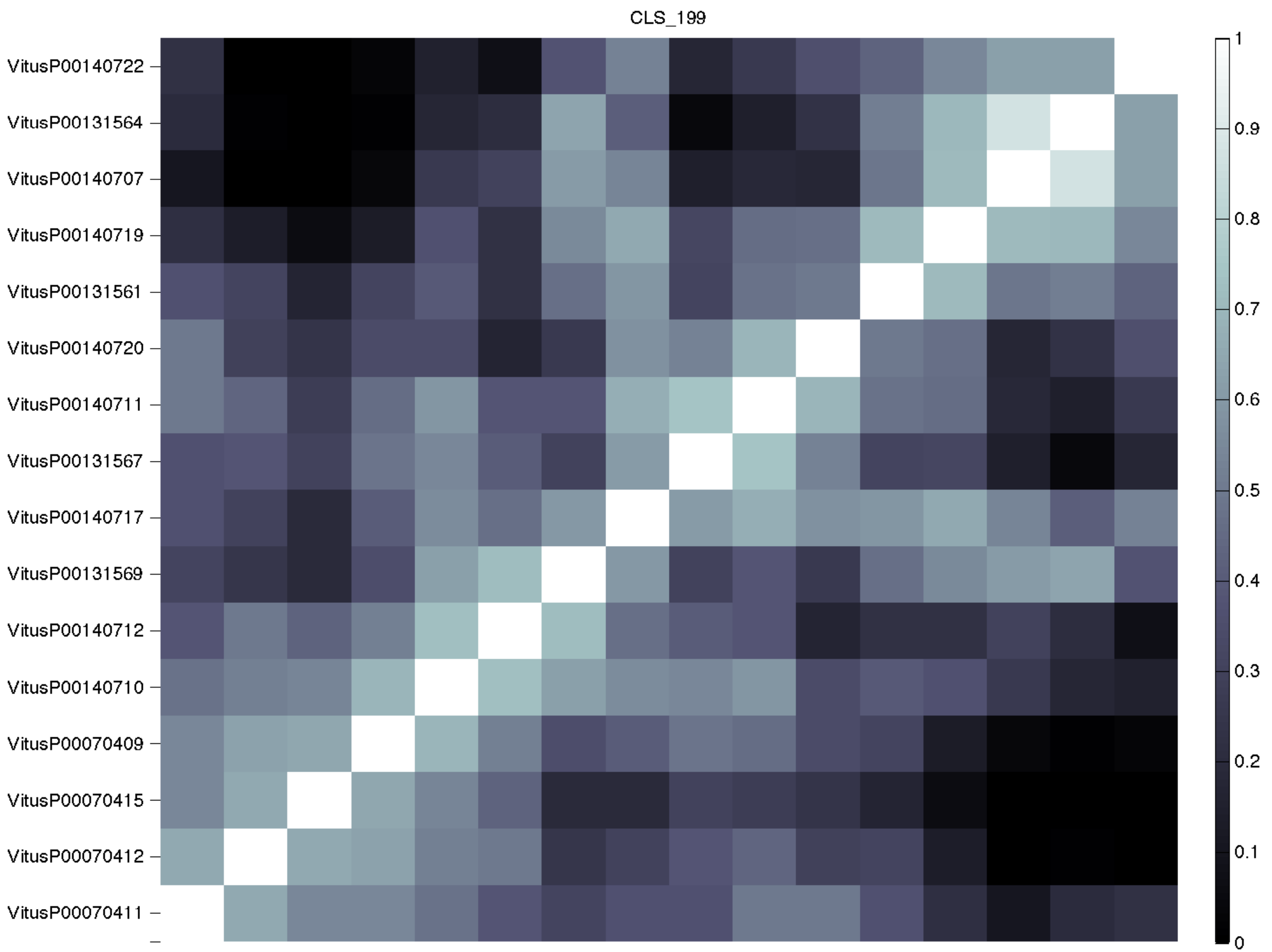
CLS_199
VitusP00140722
VitusP00131564
VitusP00140707
VitusP00140719
VitusP00131561
VitusP00140720
VitusP00140711
VitusP00131567
VitusP00140717
VitusP00131569
VitusP00140712
VitusP00140710
VitusP00070409
VitusP00070415
VitusP00070412
VitusP00070411
1
0.9
0.8
0.7
0.6
0.5
0.4
0.3
0.2
0.1
0

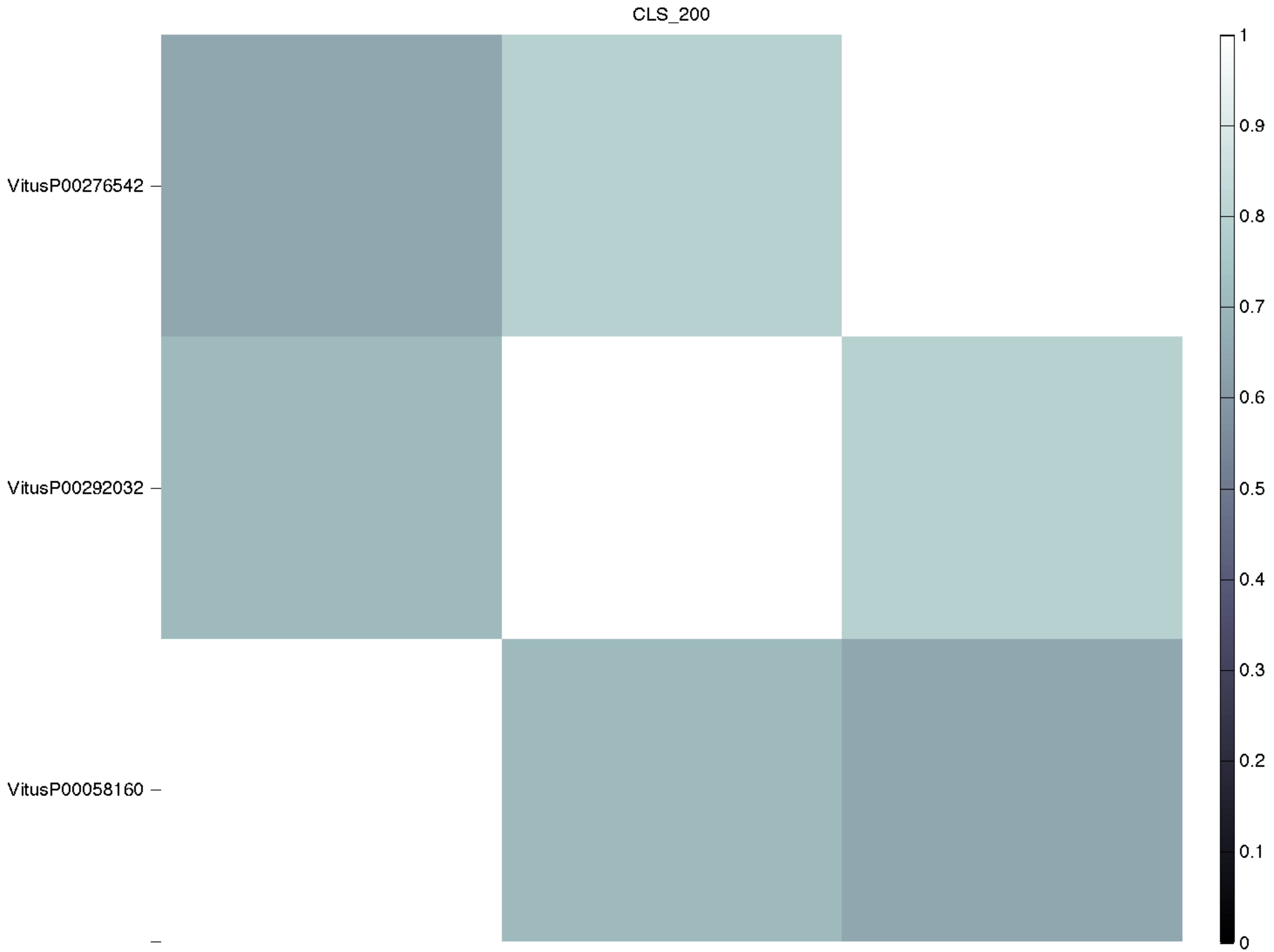
CLS_200
VitusP00276542
VitusP00292032
VitusP00058160
1
0.9
0.8
0.7
0.6
0.5
0.4
0.3
0.2
0.1
0

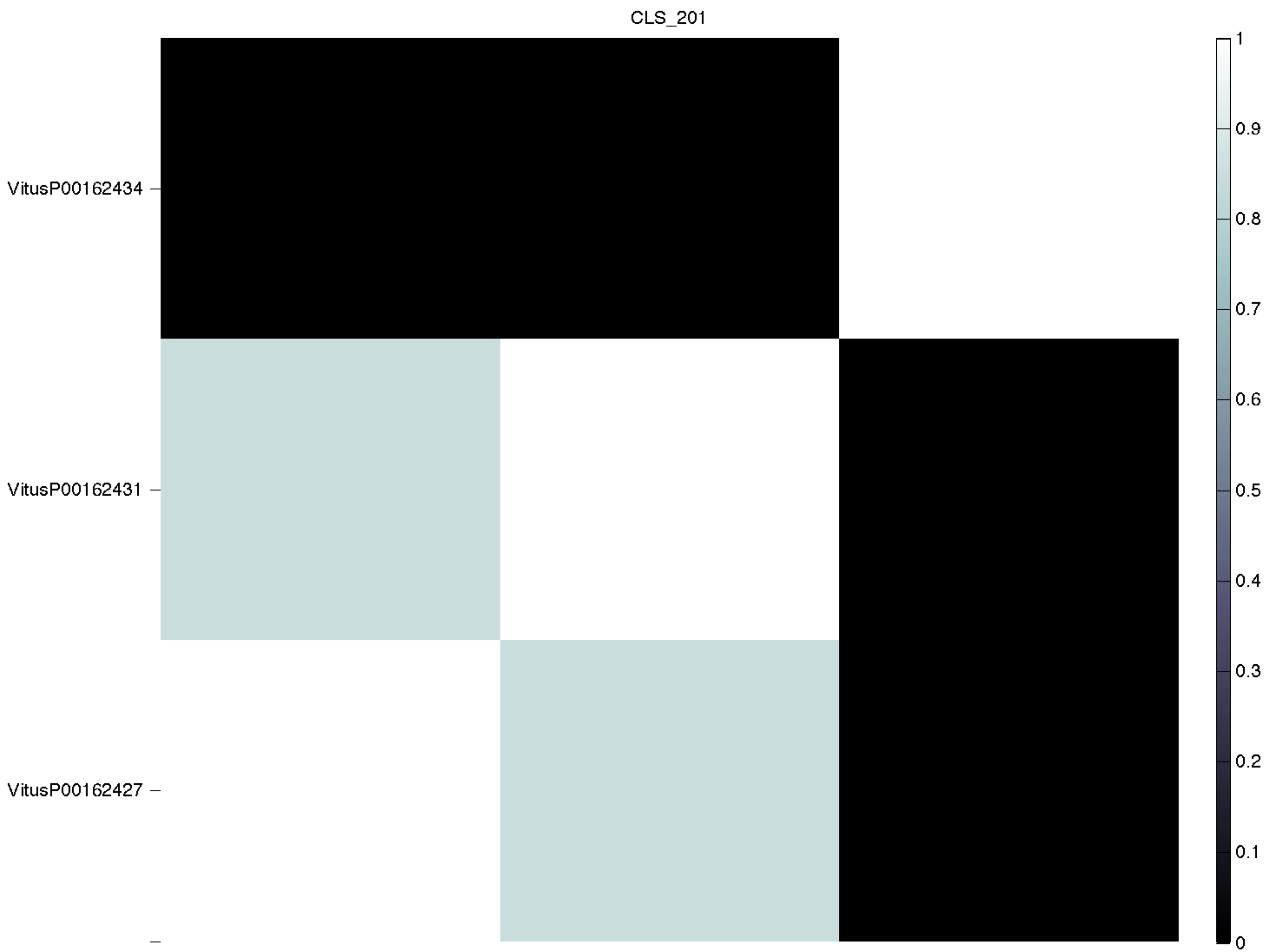
CLS_201
VitusP00162434
VitusP00162431
VitusP00162427
1
0.9
0.8
0.7
0.6
0.5
0.4
0.3
0.2
0.1
0

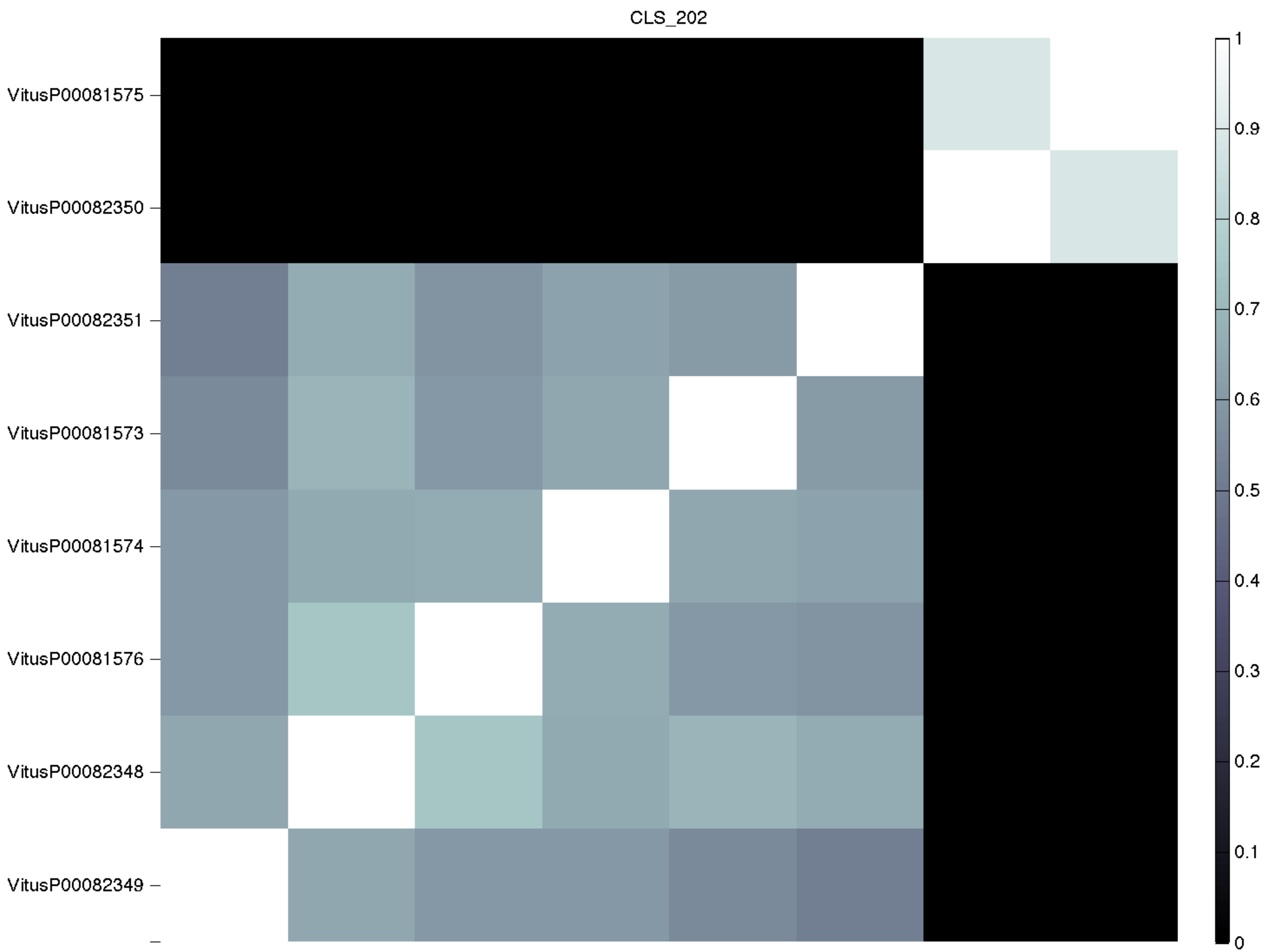

CLS_202
VitusP00081575
VitusP00082350
VitusP00082351
VitusP00081573
VitusP00081574
VitusP00081576
VitusP00082348
VitusP00082349
1
0.9
0.8
0.7
0.6
0.5
0.4
0.3
0.2
0.1
0

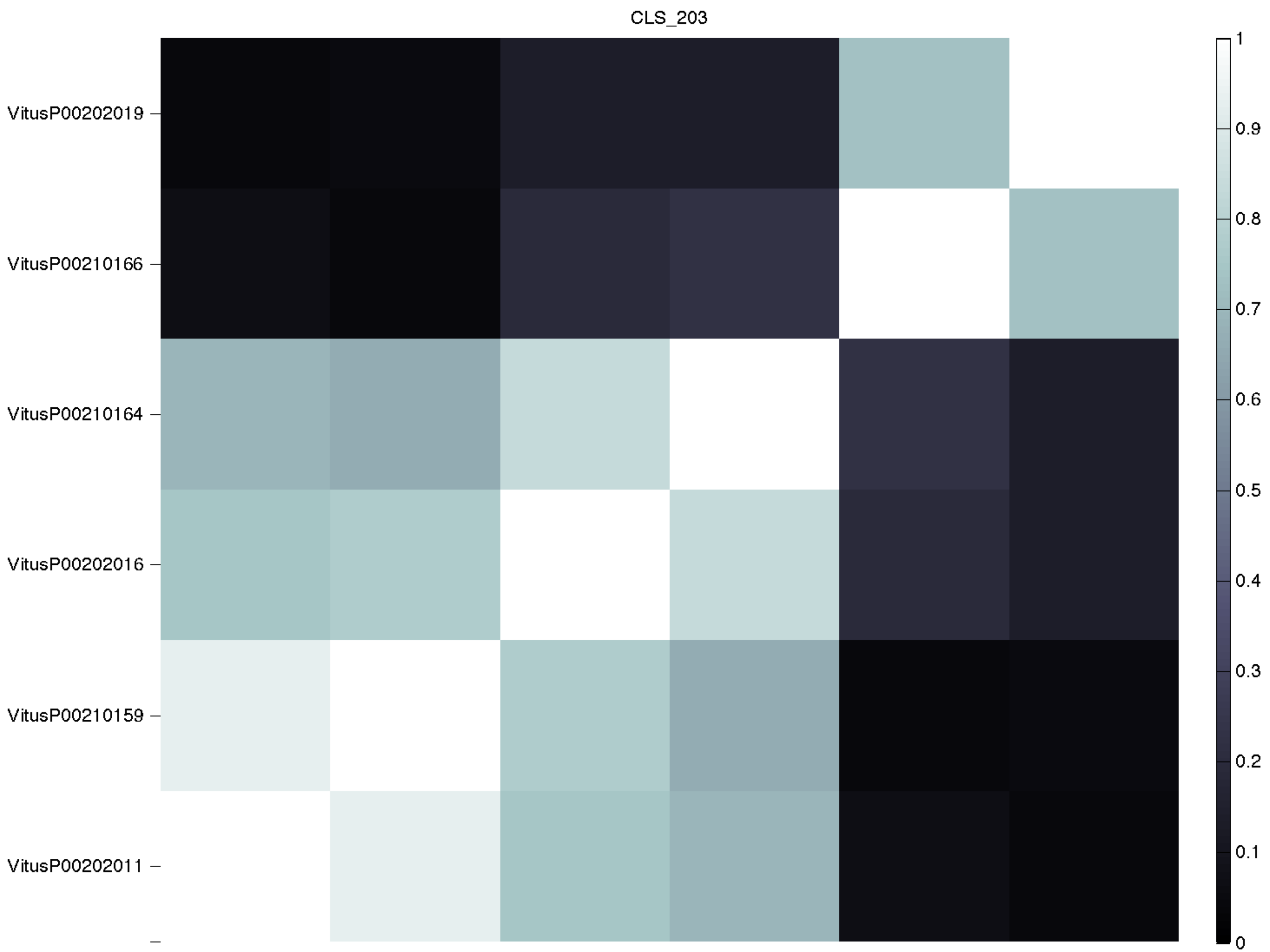
CLS_203
VitusP00202019
VitusP00210166
VitusP00210164
VitusP00202016
VitusP00210159
VitusP00202011
1
0.9
0.8
0.7
0.6
0.5
0.4
0.3
0.2
0.1
0

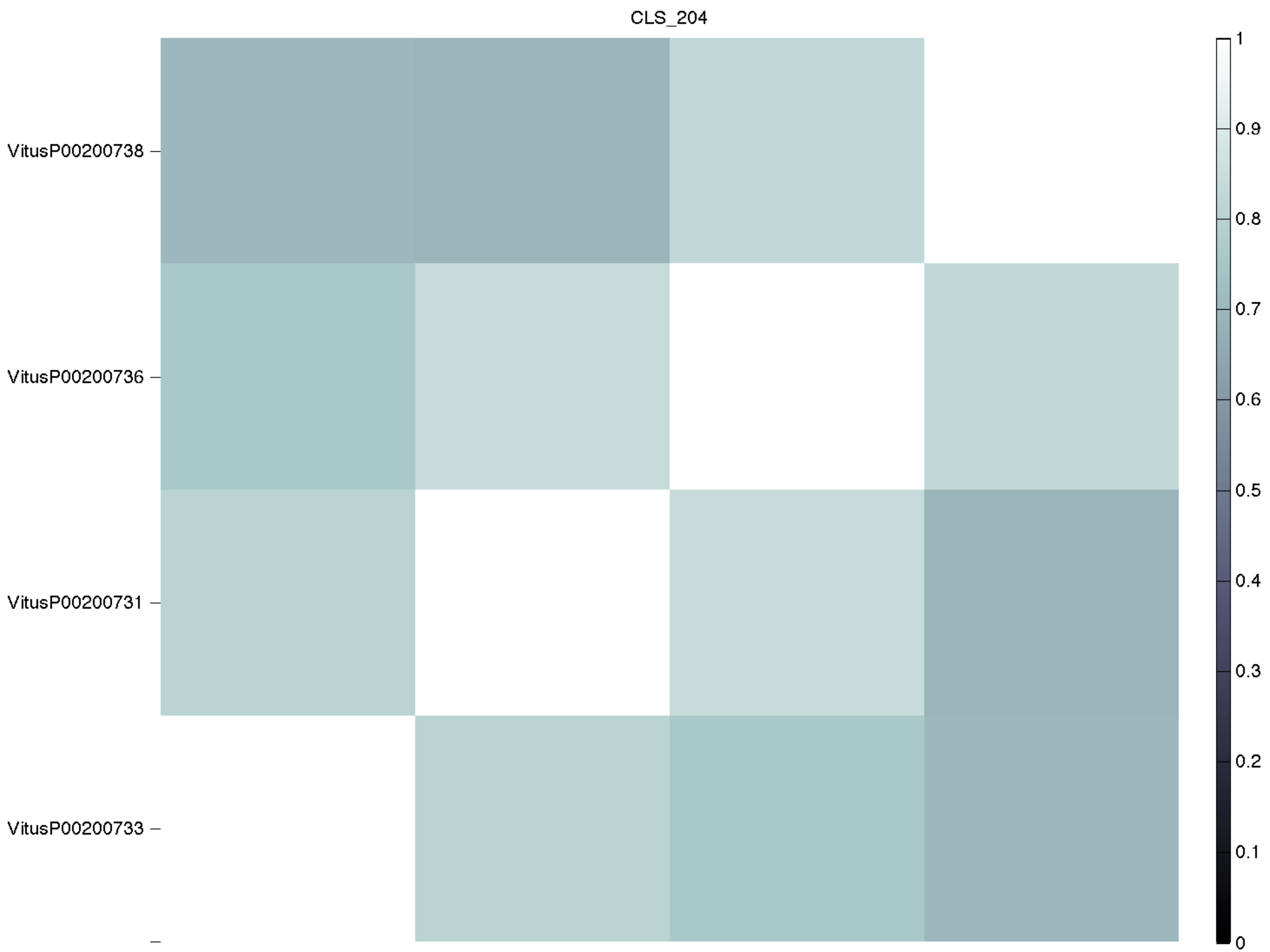

CLS_204
VitusP00200738
VitusP00200736
VitusP00200731
VitusP00200733
1
0.9
0.8
0.7
0.6
0.5
0.4
0.3
0.2
0.1
0

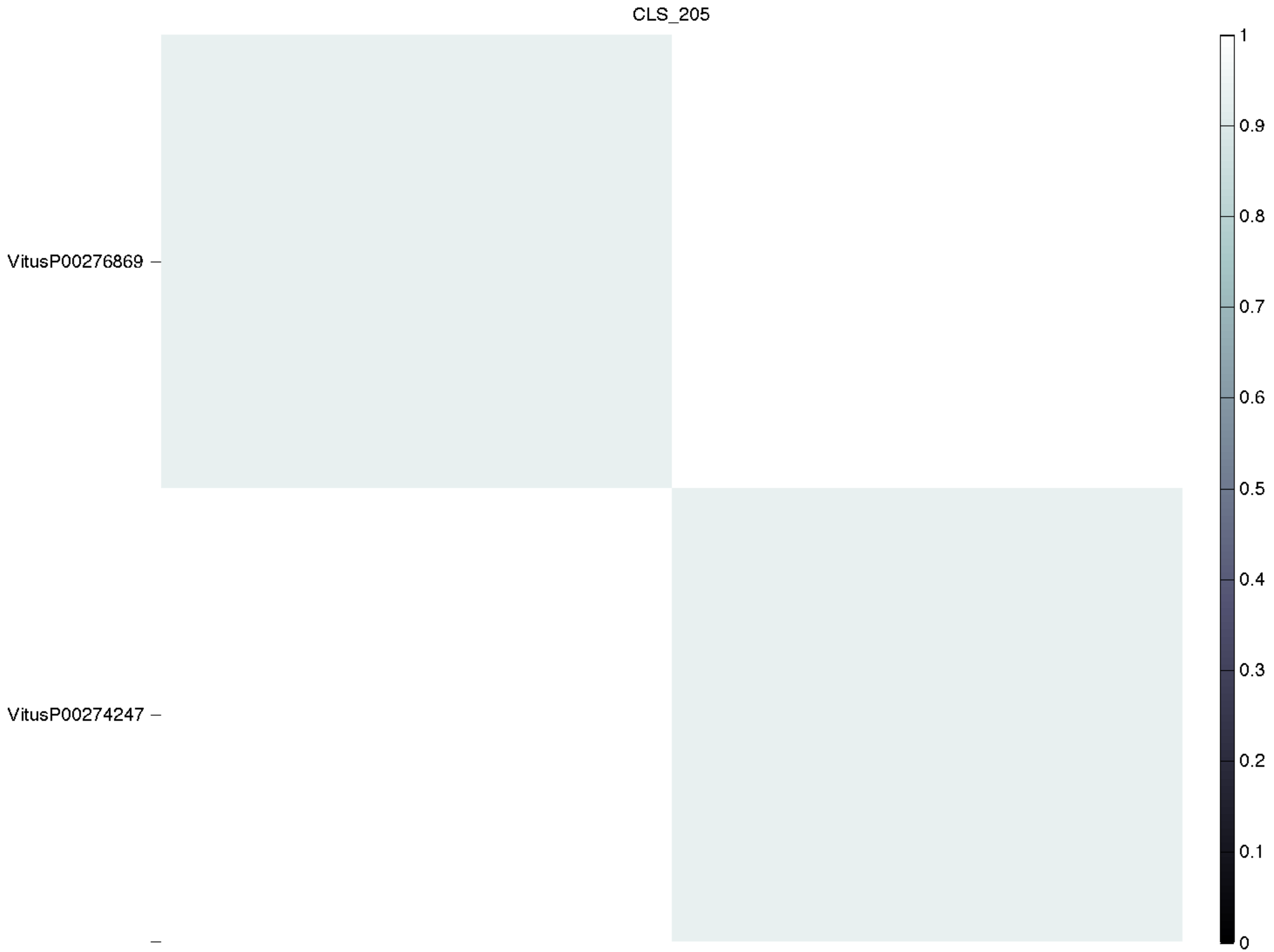

CLS_205
VitusP00276869
VitusP00274247
1
0.9
0.8
0.7
0.6
0.5
0.4
0.3
0.2
0.1
0

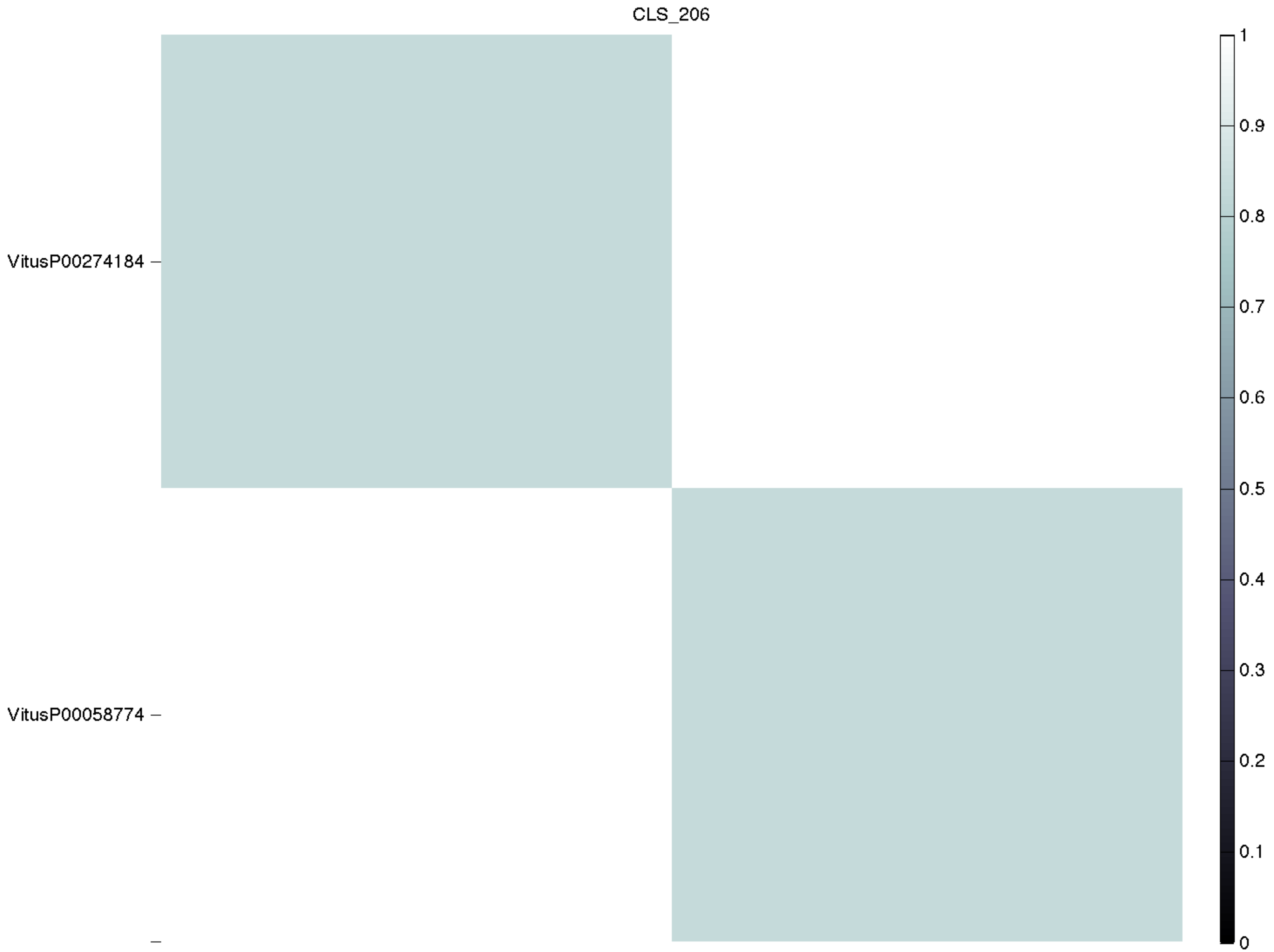
CLS_206
VitusP00274184
VitusP00058774
1
0.9
0.8
0.7
0.6
0.5
0.4
0.3
0.2
0.1
0

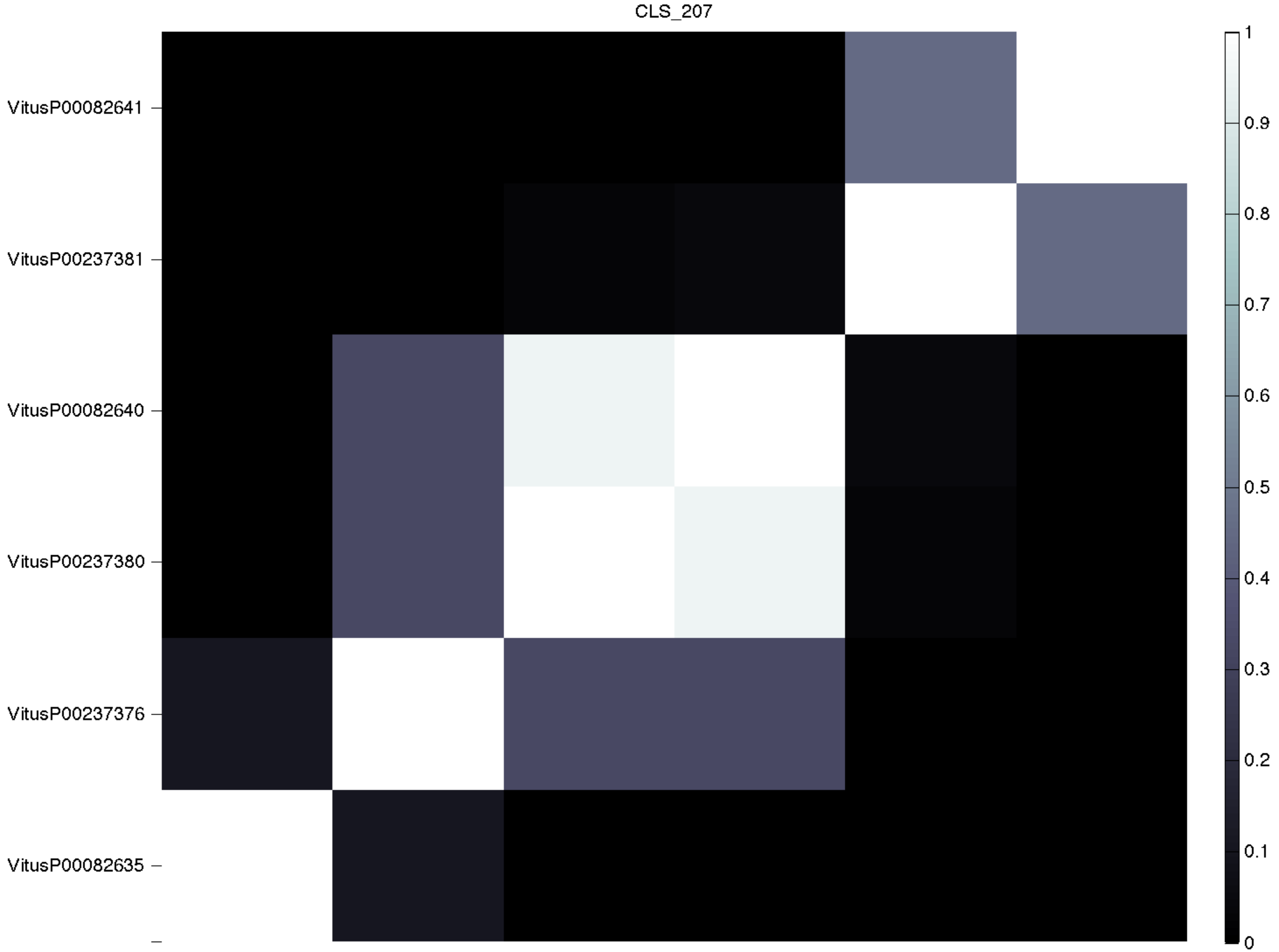

CLS_207
VitusP00082641
VitusP00237381
VitusP00082640
VitusP00237380
VitusP00237376
VitusP00082635
1
0.9
0.8
0.7
0.6
0.5
0.4
0.3
0.2
0.1
0

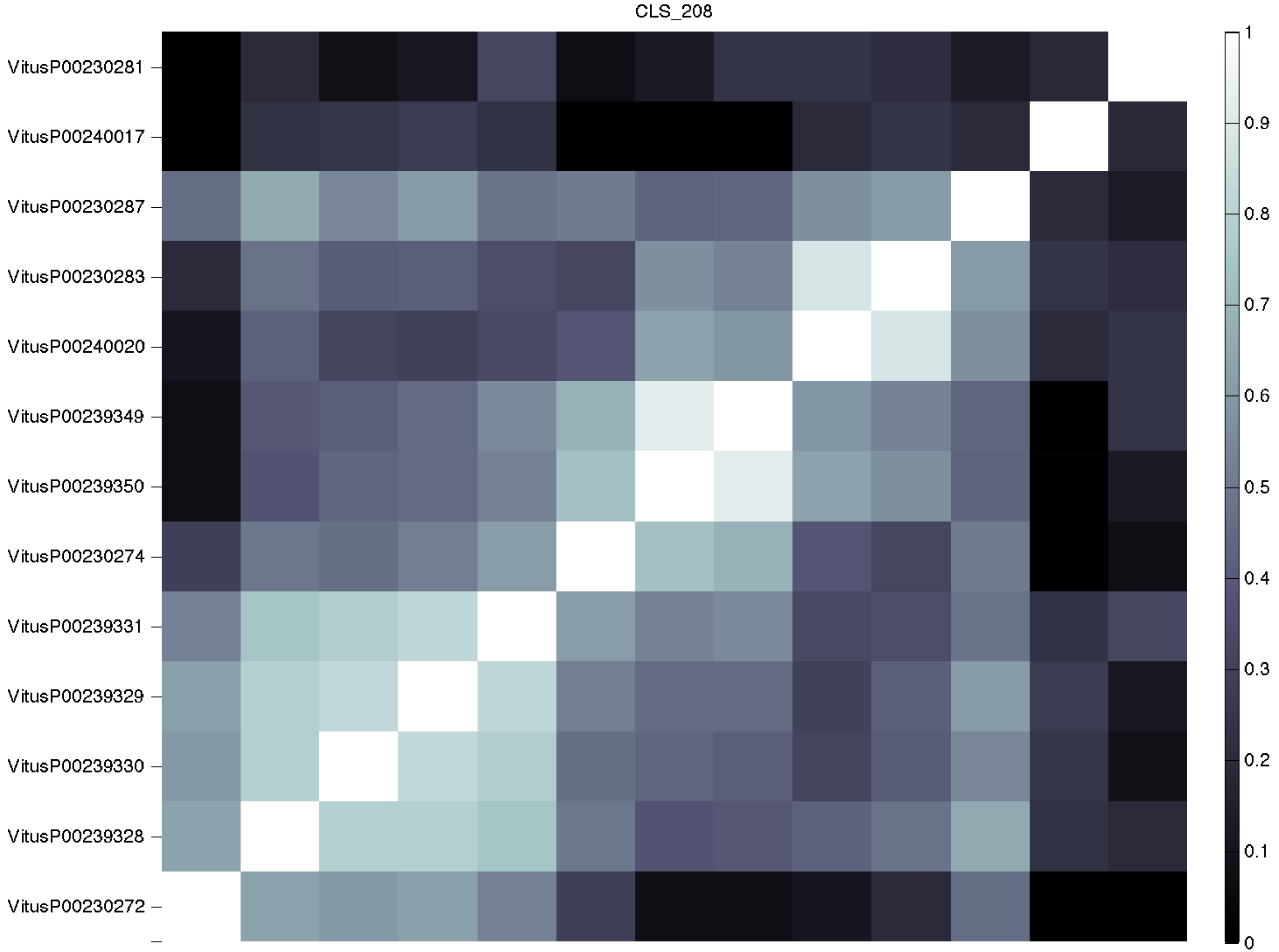

CLS_208
VitusP00230281
VitusP00240017
VitusP00230287
VitusP00230283
VitusP00240020
VitusP00239349
VitusP00239350
VitusP00230274
VitusP00239331
VitusP00239329
VitusP00239330
VitusP00239328
VitusP00230272
1
0.9
0.8
0.7
0.6
0.5
0.4
0.3
0.2
0.1
0

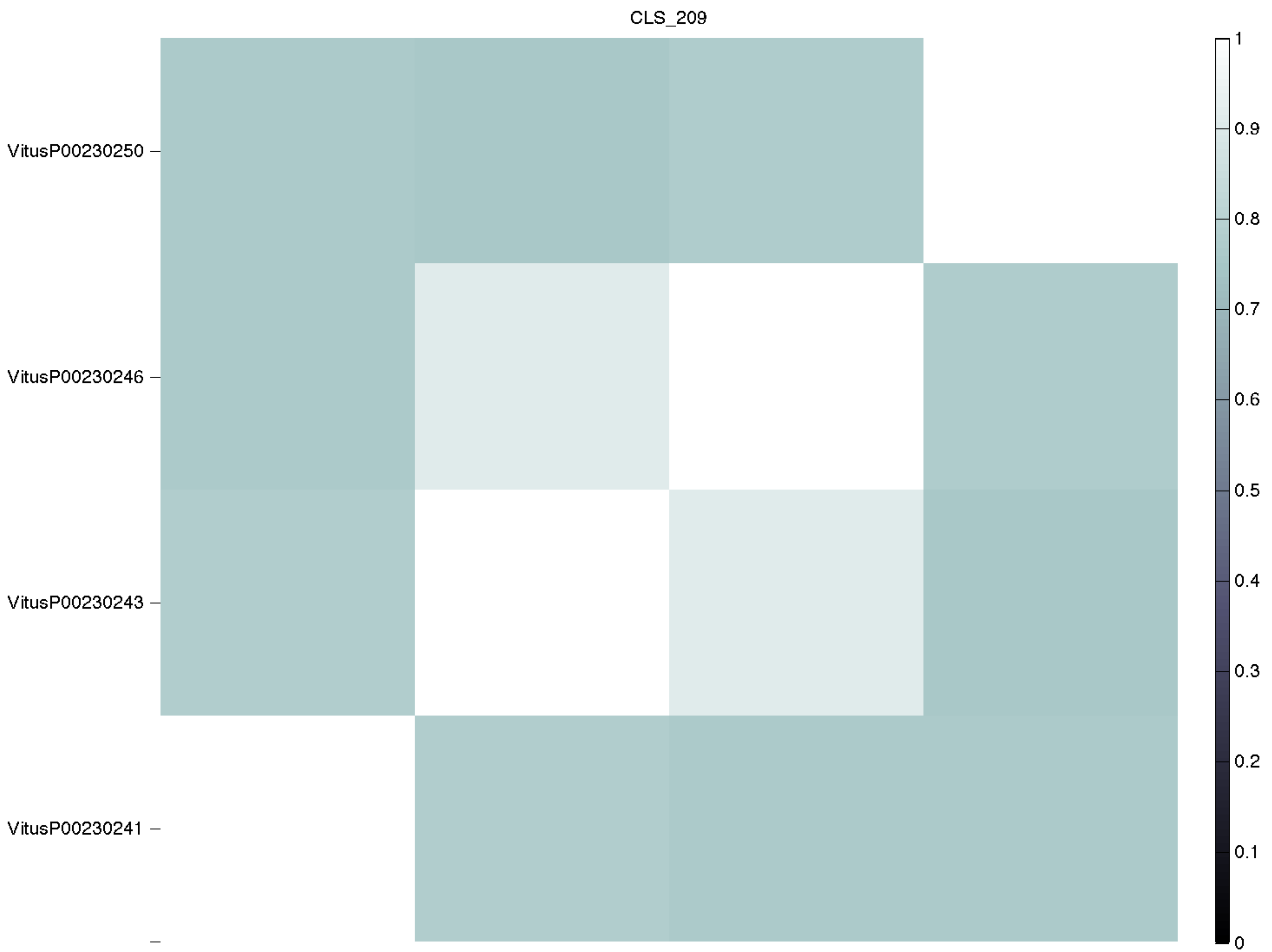
CLS_209
VitusP00230250
VitusP00230246
VitusP00230243
VitusP00230241
1
0.9
0.8
0.7
0.6
0.5
0.4
0.3
0.2
0.1
0

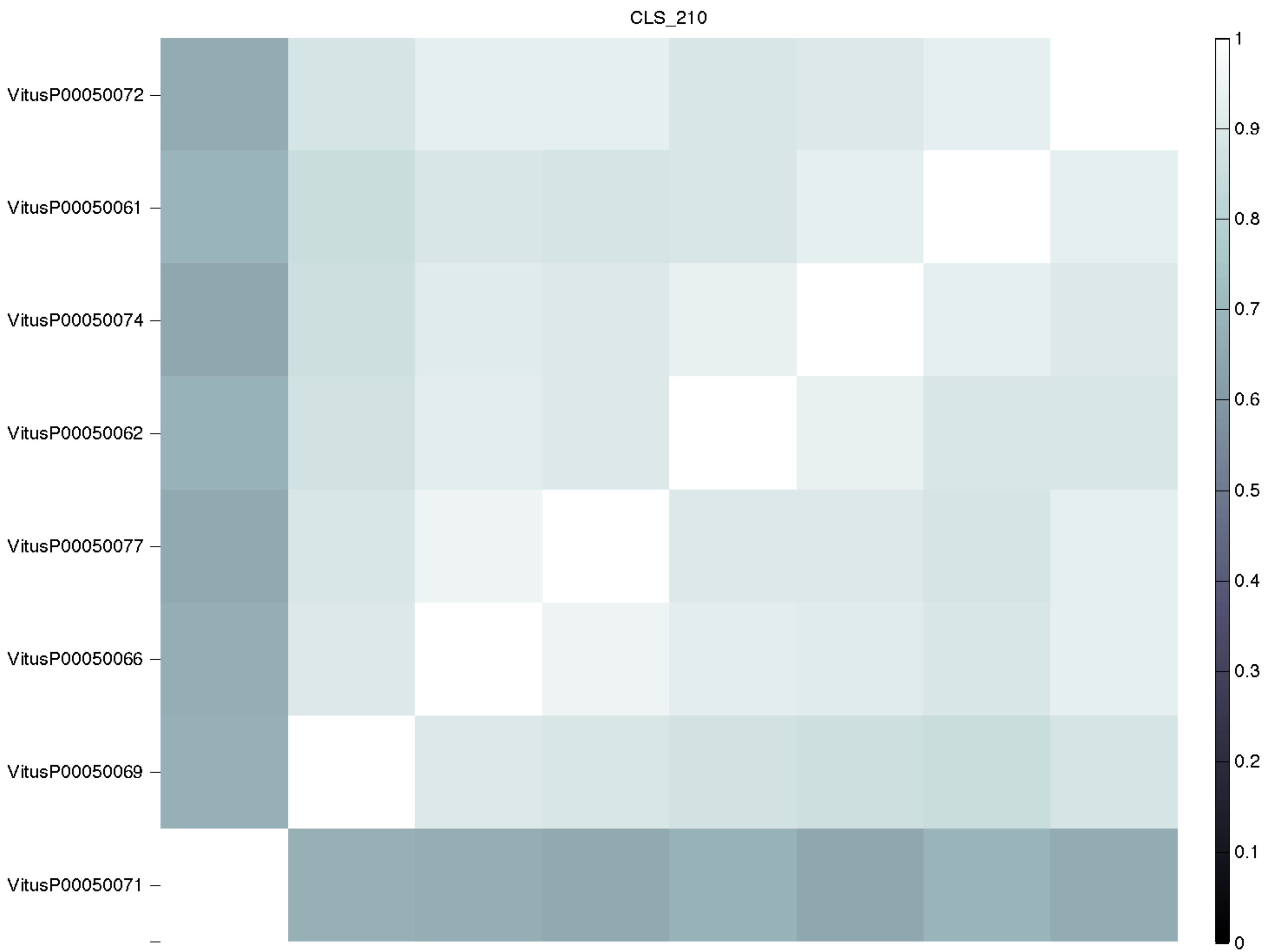
CLS_210
VitusP00050072
VitusP00050061
VitusP00050074
VitusP00050062
VitusP00050077
VitusP00050066
VitusP00050069
VitusP00050071
1
0.9
0.8
0.7
0.6
0.5
0.4
0.3
0.2
0.1
0

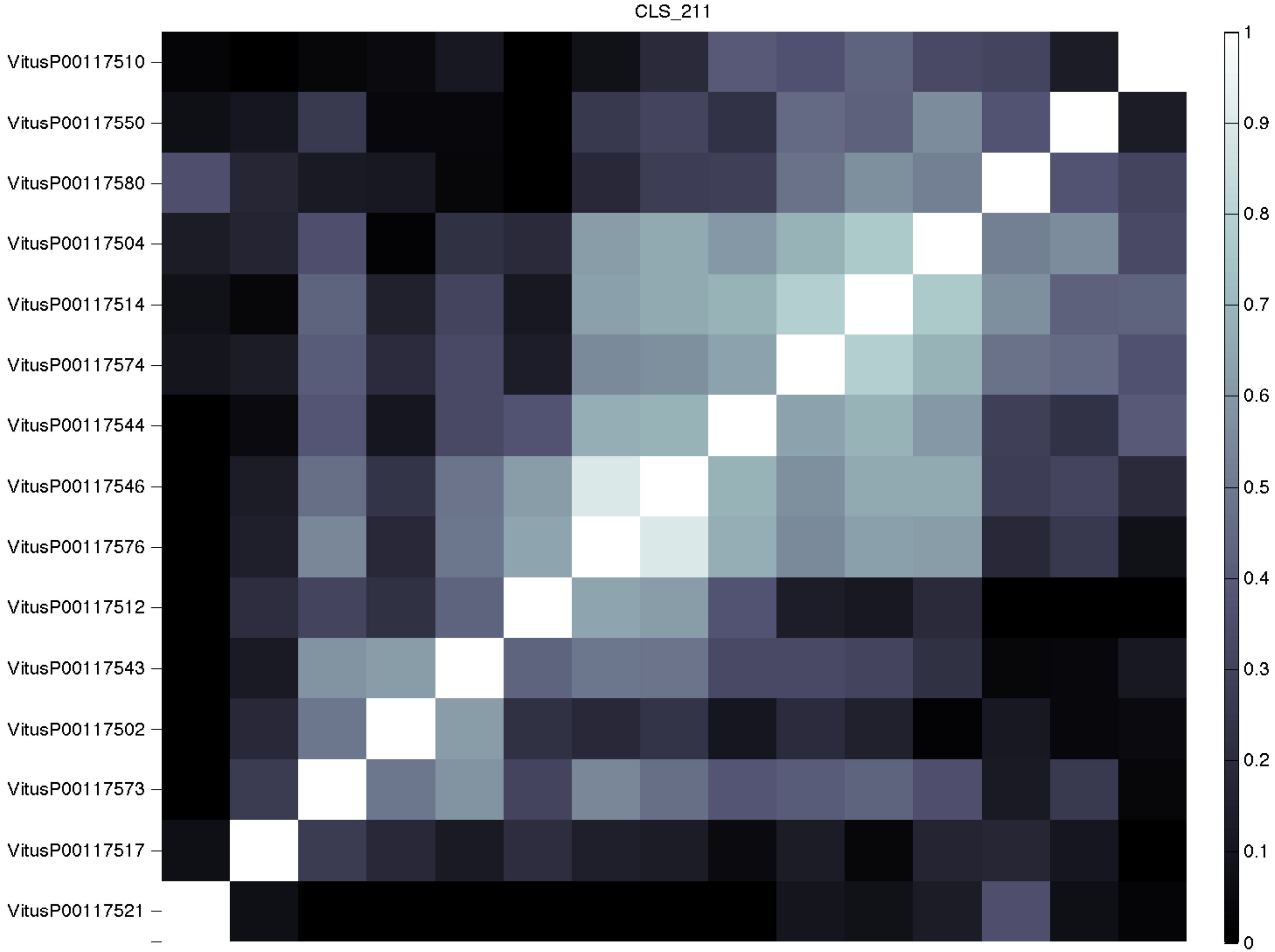
CLS_211
VitusP00117510
VitusP00117550
VitusP00117580
VitusP00117504
VitusP00117514
VitusP00117574
VitusP00117544
VitusP00117546
VitusP00117576
VitusP00117512
VitusP00117543
VitusP00117502
VitusP00117573
VitusP00117517
VitusP00117521
1
0.9
0.8
0.7
0.6
0.5
0.4
0.3
0.2
0.1
0

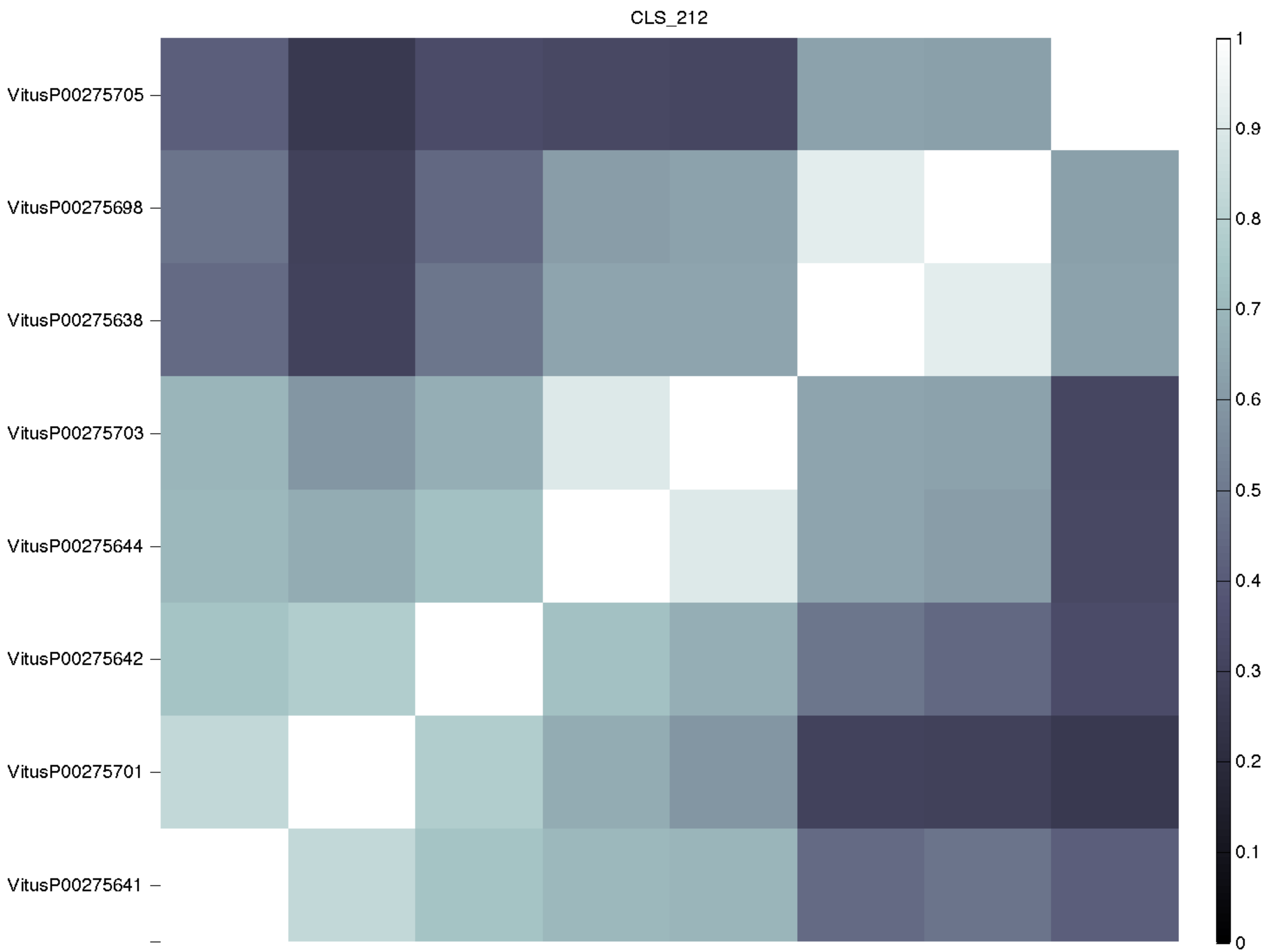
CLS_212
VitusP00275705
VitusP00275698
VitusP00275638
VitusP00275703
VitusP00275644
VitusP00275642
VitusP00275701
VitusP00275641
1
0.9
0.8
0.7
0.6
0.5
0.4
0.3
0.2
0.1
0

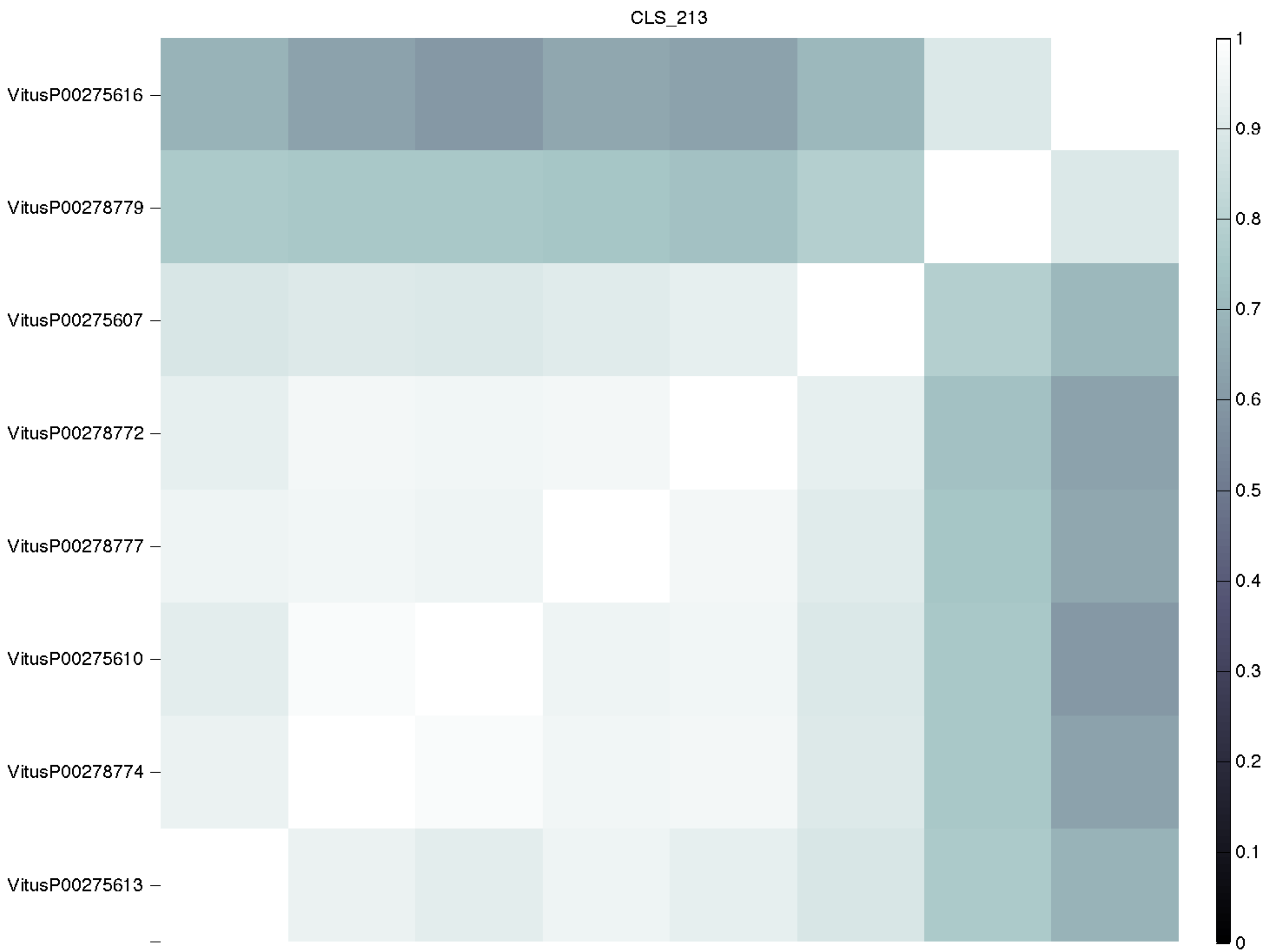
CLS_213
VitusP00275616
VitusP00278779
VitusP00275607
VitusP00278772
VitusP00278777
VitusP00275610
VitusP00278774
VitusP00275613
1
0.9
0.8
0.7
0.6
0.5
0.4
0.3
0.2
0.1
0

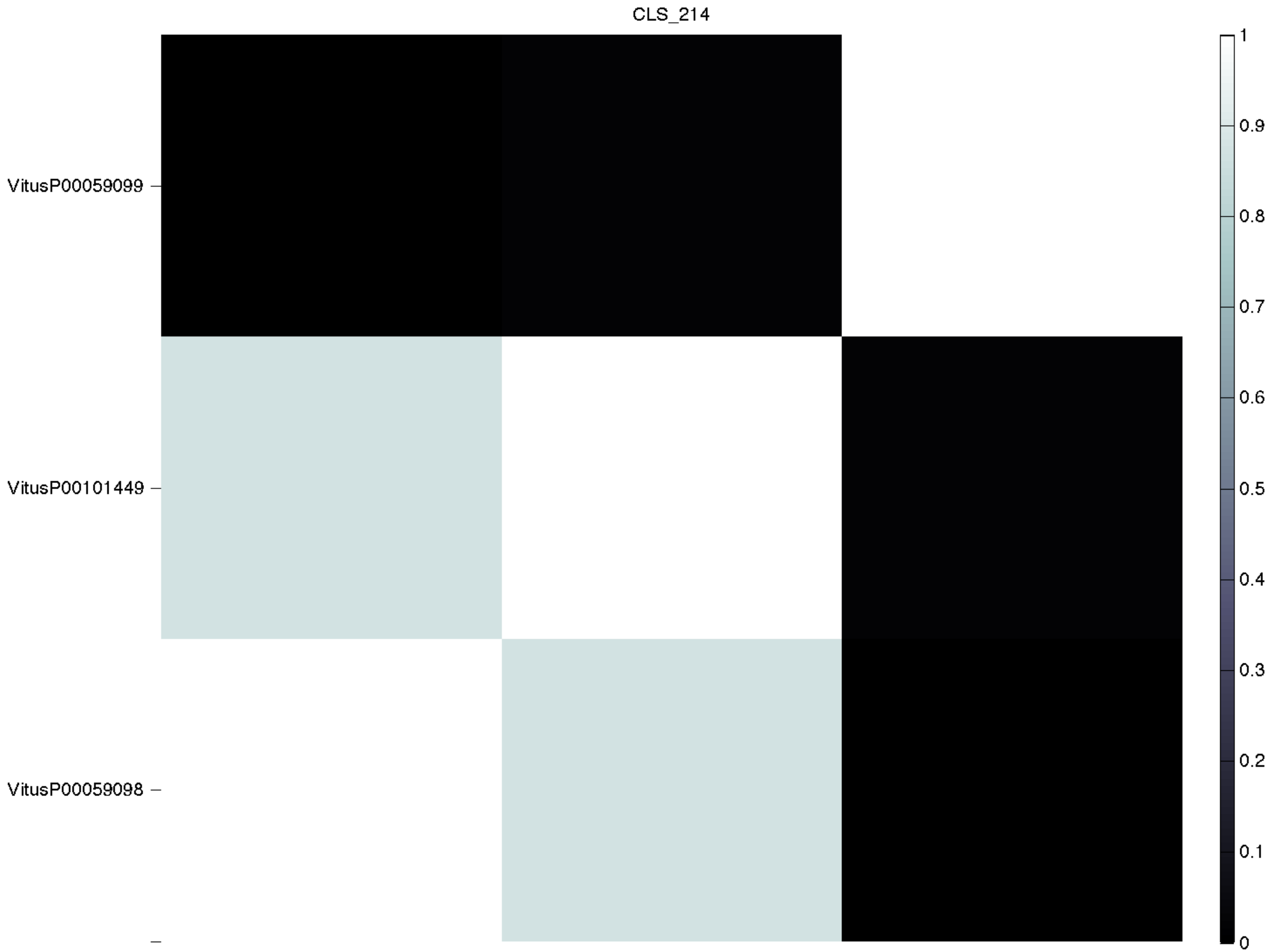

CLS_214
VitusP00059099
VitusP00101449
VitusP00059098
1
0.9
0.8
0.7
0.6
0.5
0.4
0.3
0.2
0.1
0

CLS_215

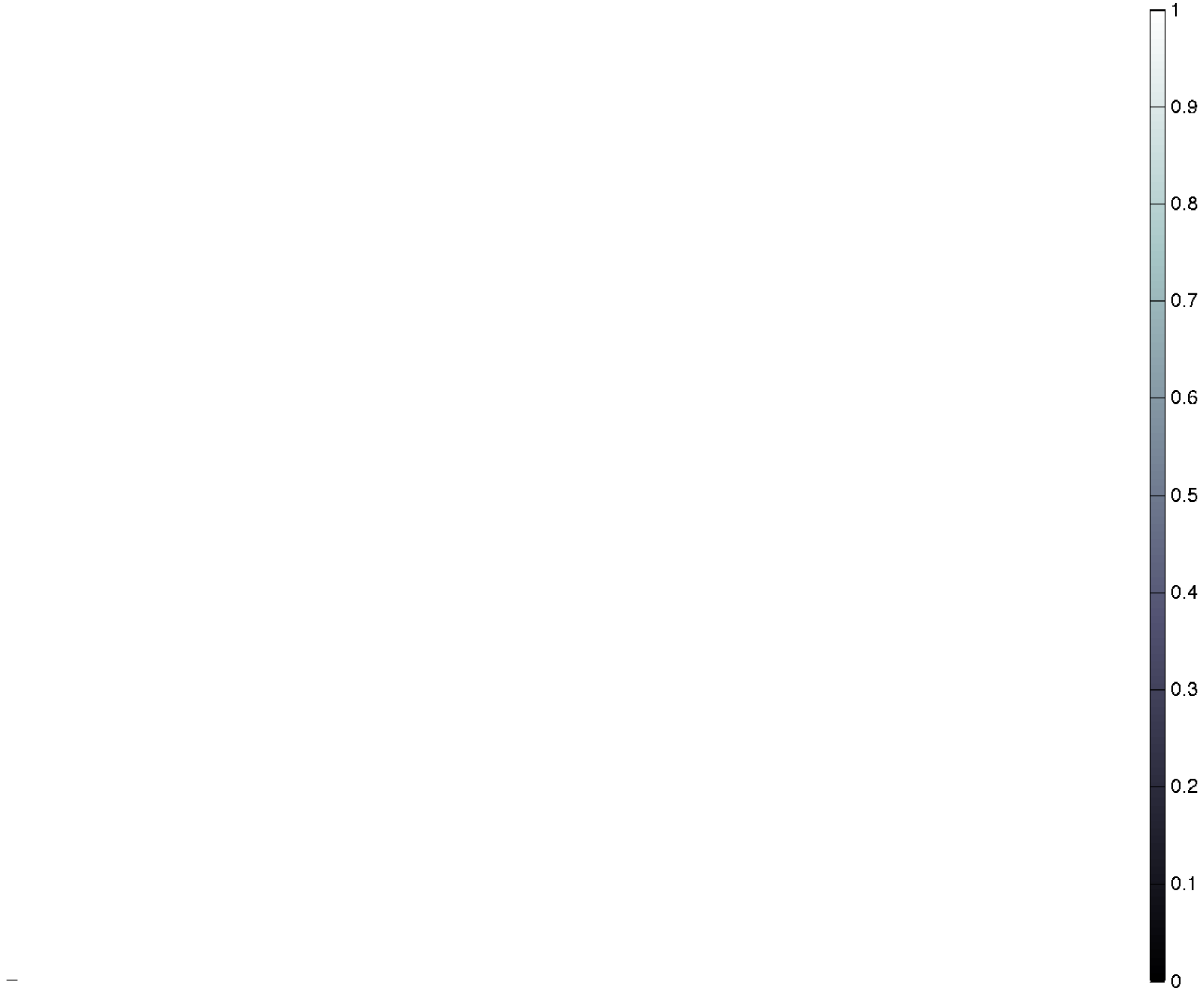

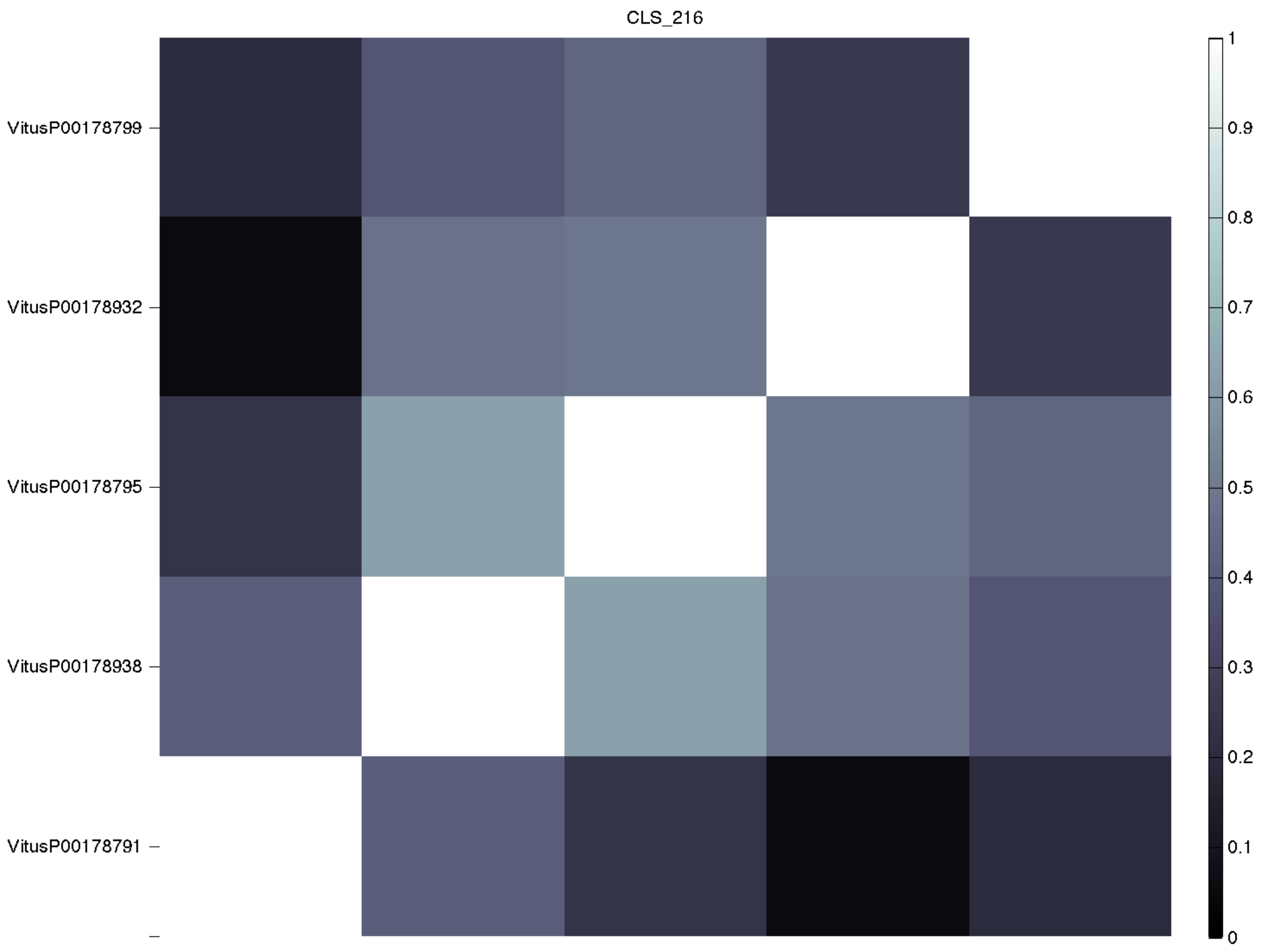
CLS_216
VitusP00178799
VitusP00178932
VitusP00178795
VitusP00178938
VitusP00178791
1
0.9
0.8
0.7
0.6
0.5
0.4
0.3
0.2
0.1
0

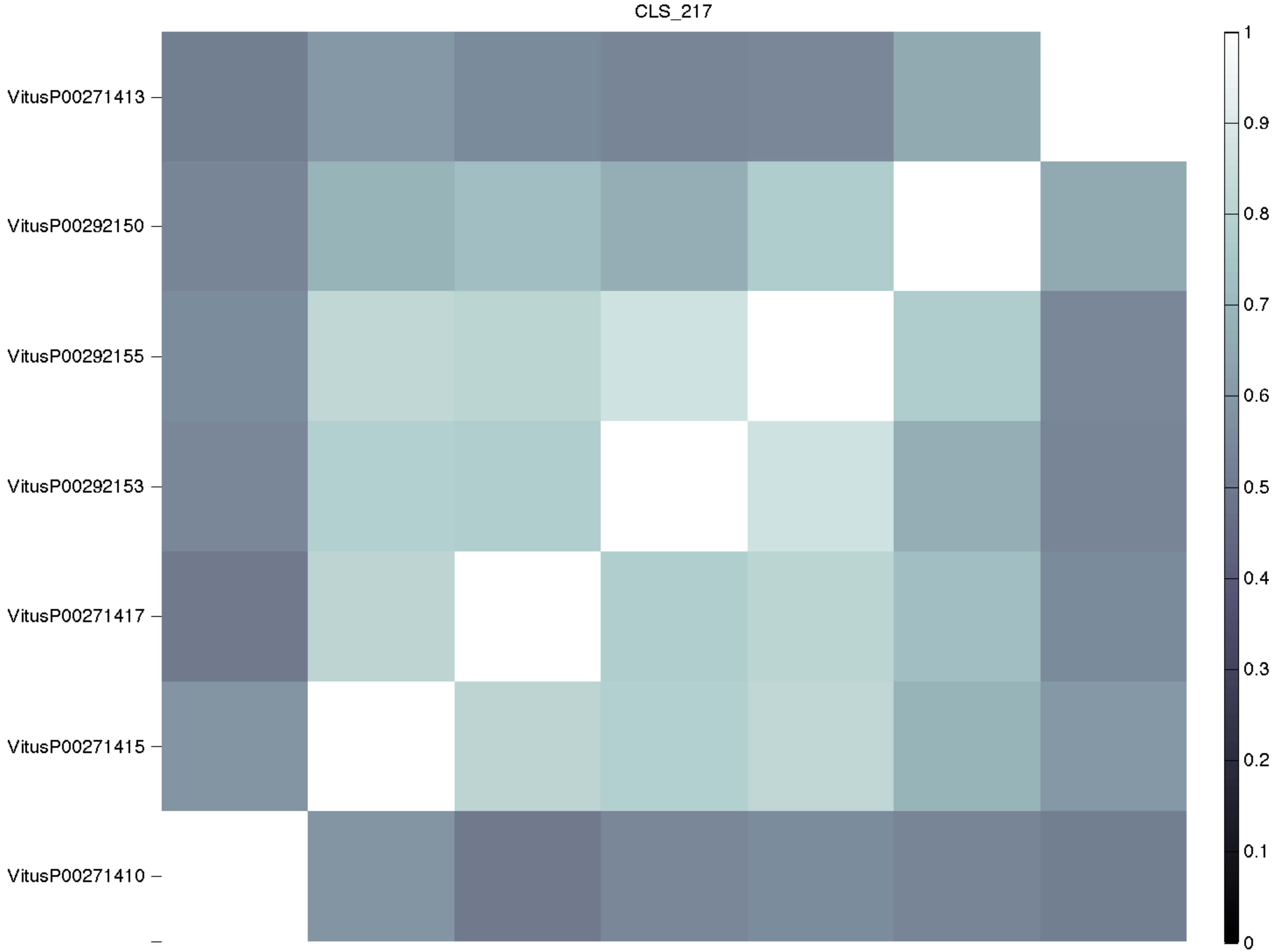
CLS_217
VitusP00271413
VitusP00292150
VitusP00292155
VitusP00292153
VitusP00271417
VitusP00271415
VitusP00271410
1
0.9
0.8
0.7
0.6
0.5
0.4
0.3
0.2
0.1
0

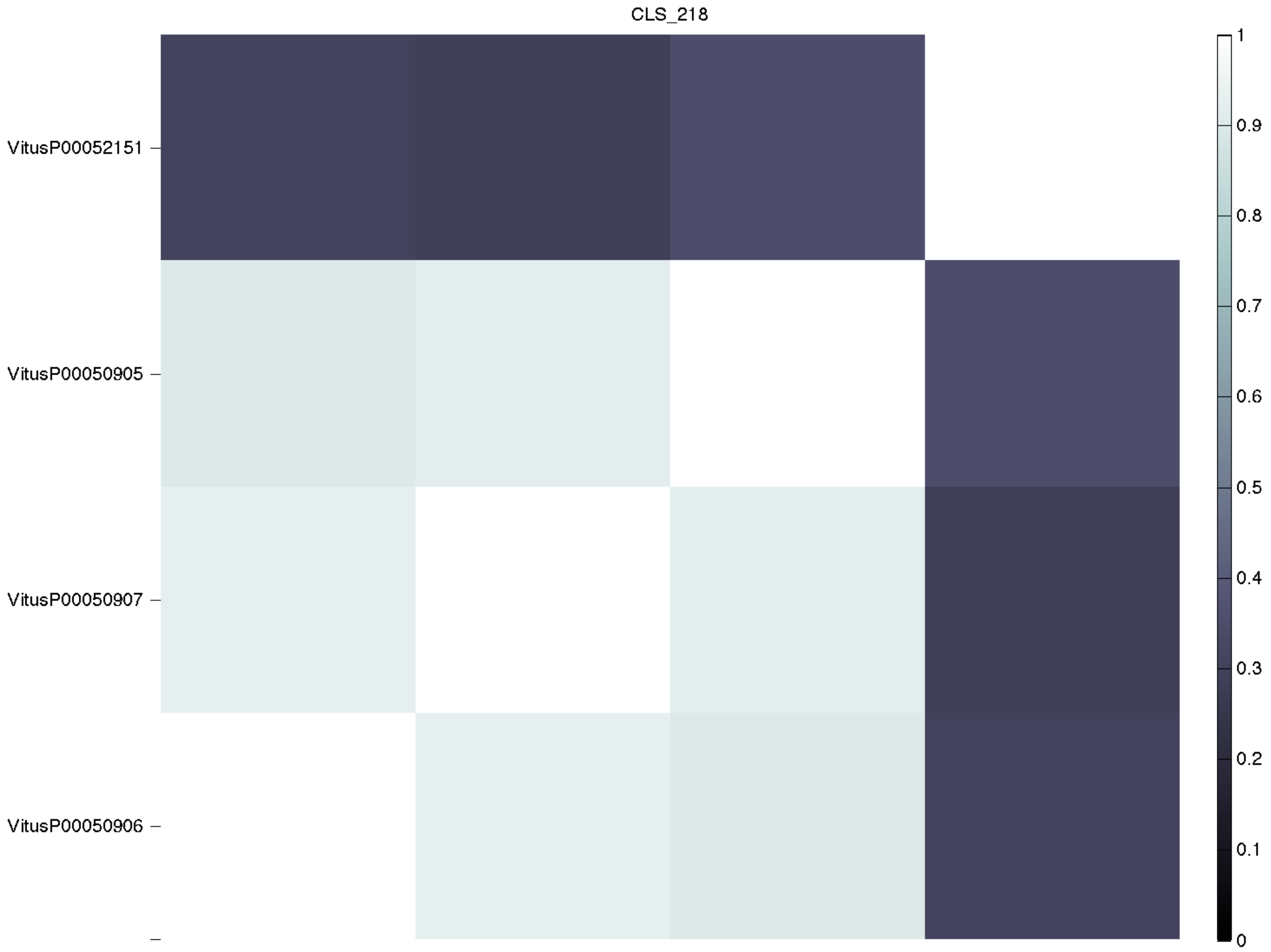

CLS_218
VitusP00052151
VitusP00050905
VitusP00050907
VitusP00050906
1
0.9
0.8
0.7
0.6
0.5
0.4
0.3
0.2
0.1
0

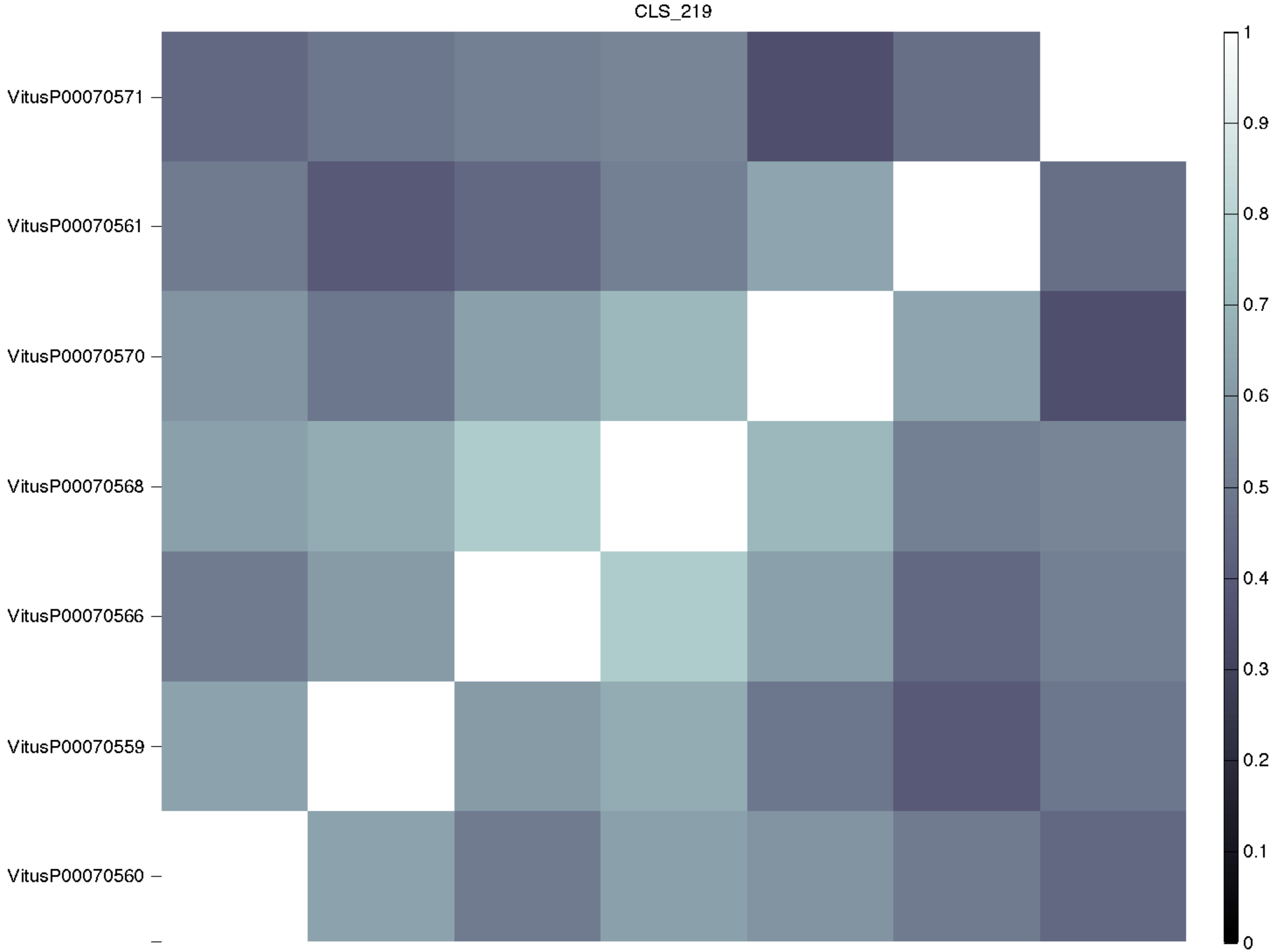
CLS_219
VitusP00070571
VitusP00070561
VitusP00070570
VitusP00070568
VitusP00070566
VitusP00070559
VitusP00070560
1
0.9
0.8
0.7
0.6
0.5
0.4
0.3
0.2
0.1
0

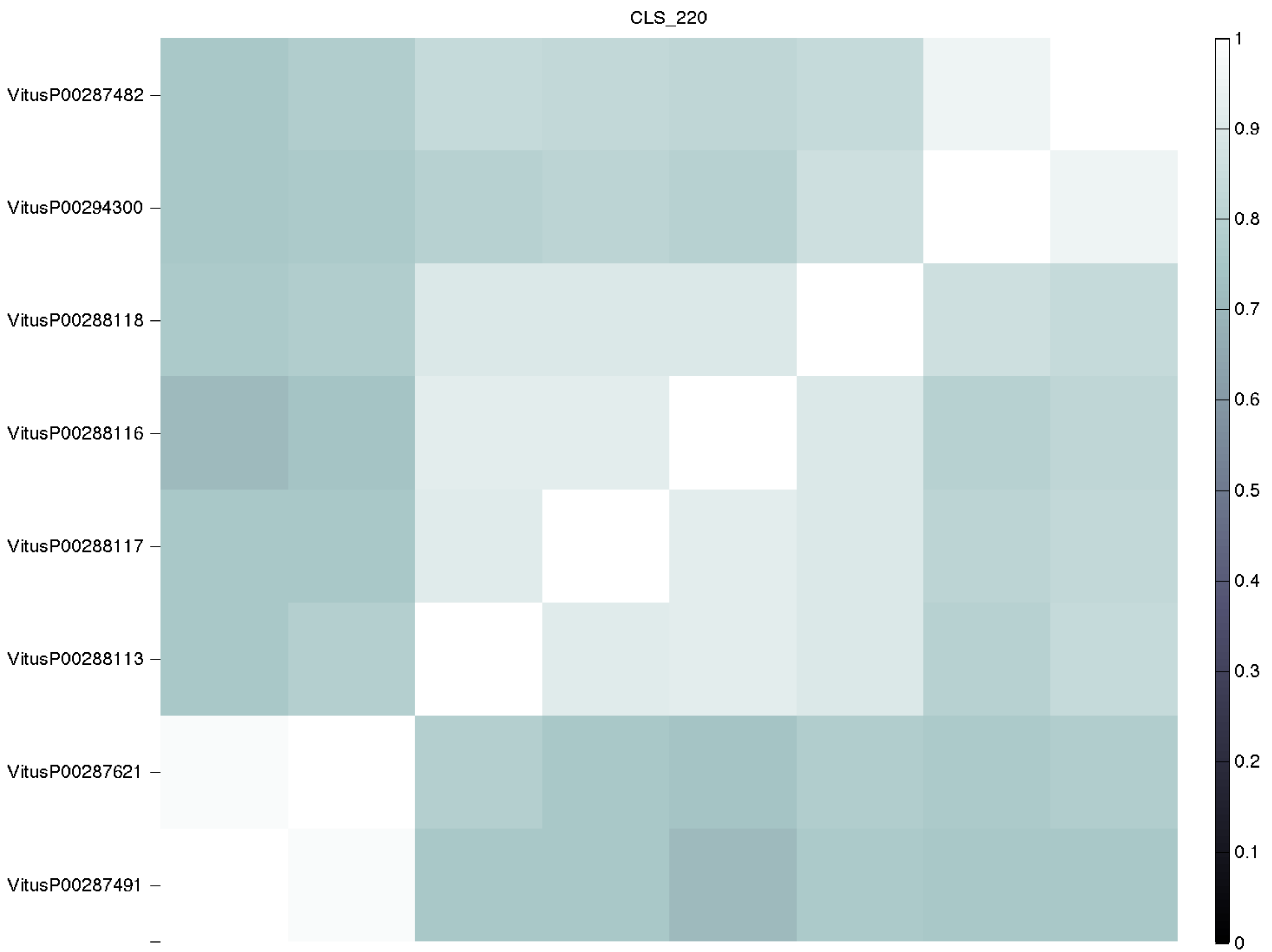

CLS_220
VitusP00287482
VitusP00294300
VitusP00288118
VitusP00288116
VitusP00288117
VitusP00288113
VitusP00287621
VitusP00287491
1
0.9
0.8
0.7
0.6
0.5
0.4
0.3
0.2
0.1
0

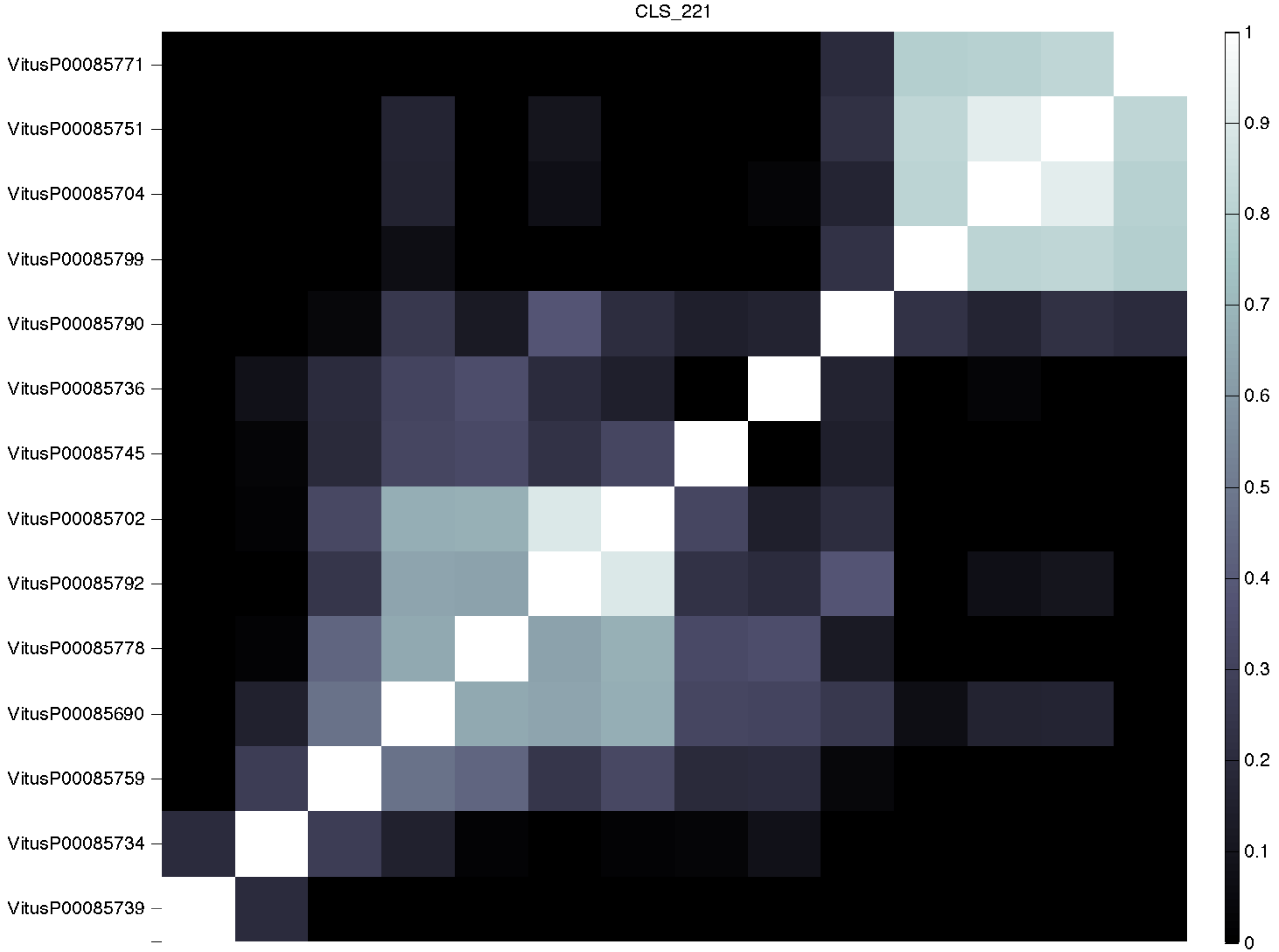

CLS_221
VitusP00085771
VitusP00085751
VitusP00085704
VitusP00085799
VitusP00085790
VitusP00085736
VitusP00085745
VitusP00085702
VitusP00085792
VitusP00085778
VitusP00085690
VitusP00085759
VitusP00085734
VitusP00085739
1
0.9
0.8
0.7
0.6
0.5
0.4
0.3
0.2
0.1
0

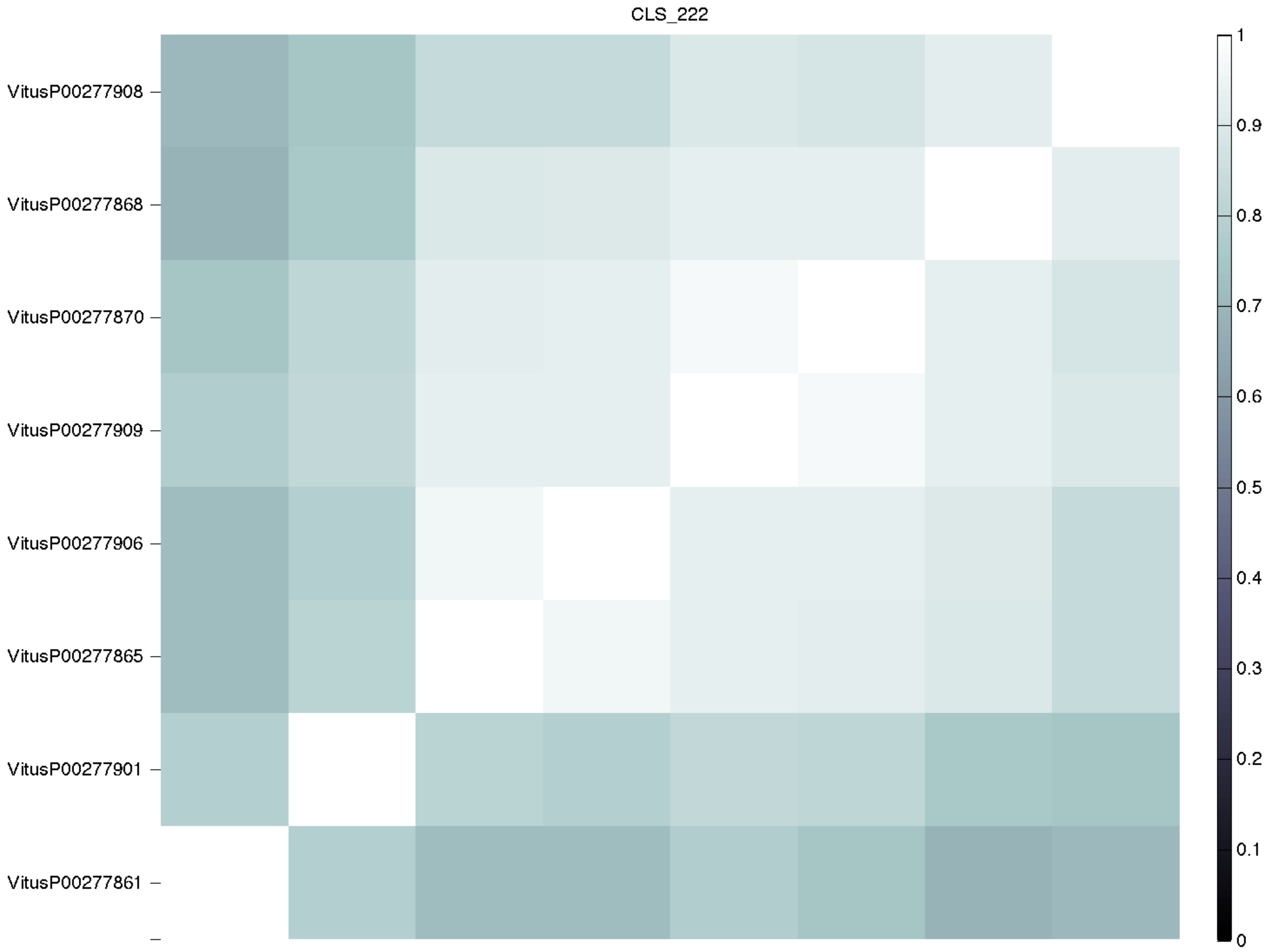

CLS_222
VitusP00277908
VitusP00277868
VitusP00277870
VitusP00277909
VitusP00277906
VitusP00277865
VitusP00277901
VitusP00277861
1
0.9
0.8
0.7
0.6
0.5
0.4
0.3
0.2
0.1
0

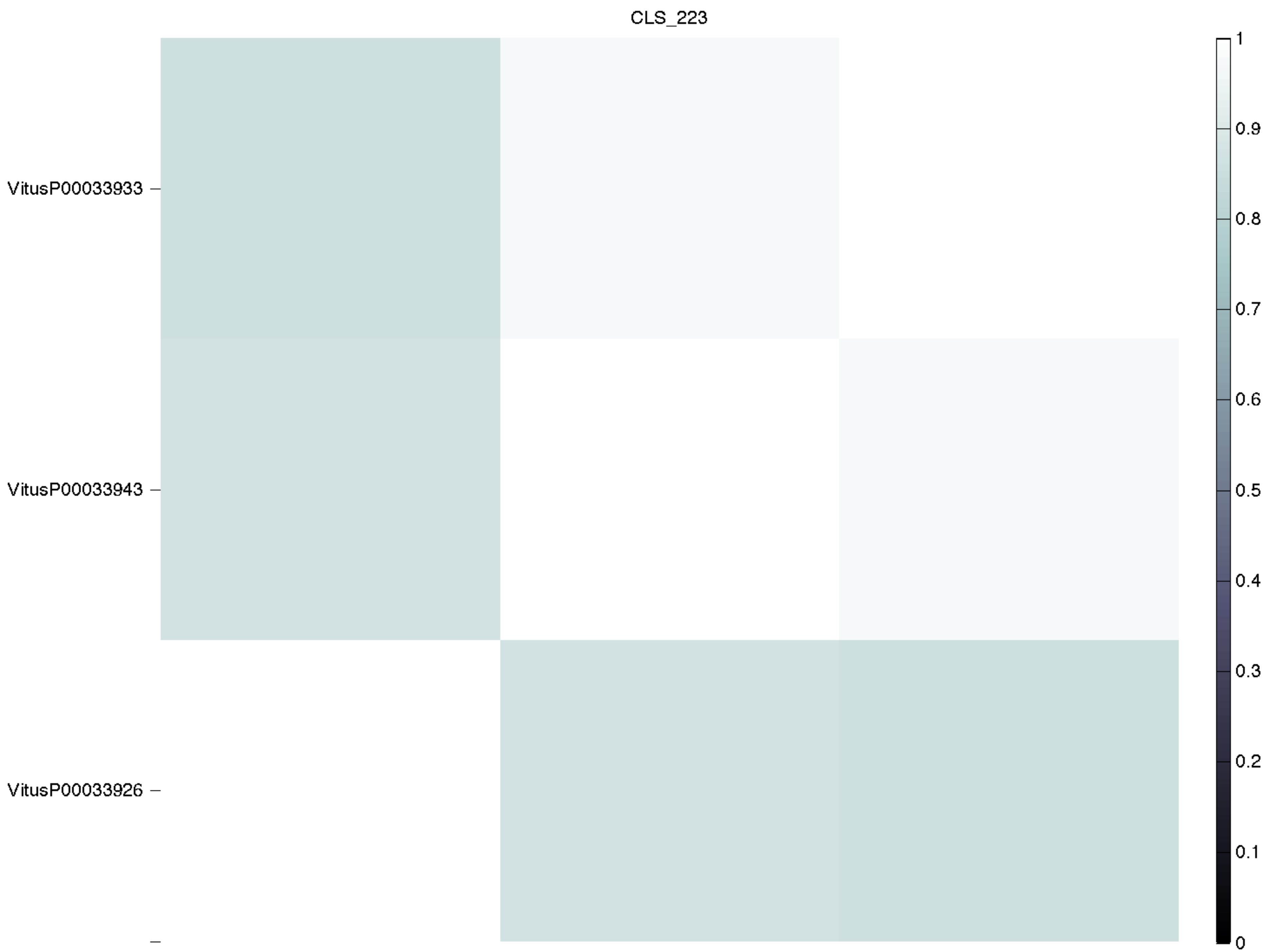

CLS_223
VitusP00033933
VitusP00033943
VitusP00033926
1
0.9
0.8
0.7
0.6
0.5
0.4
0.3
0.2
0.1
0

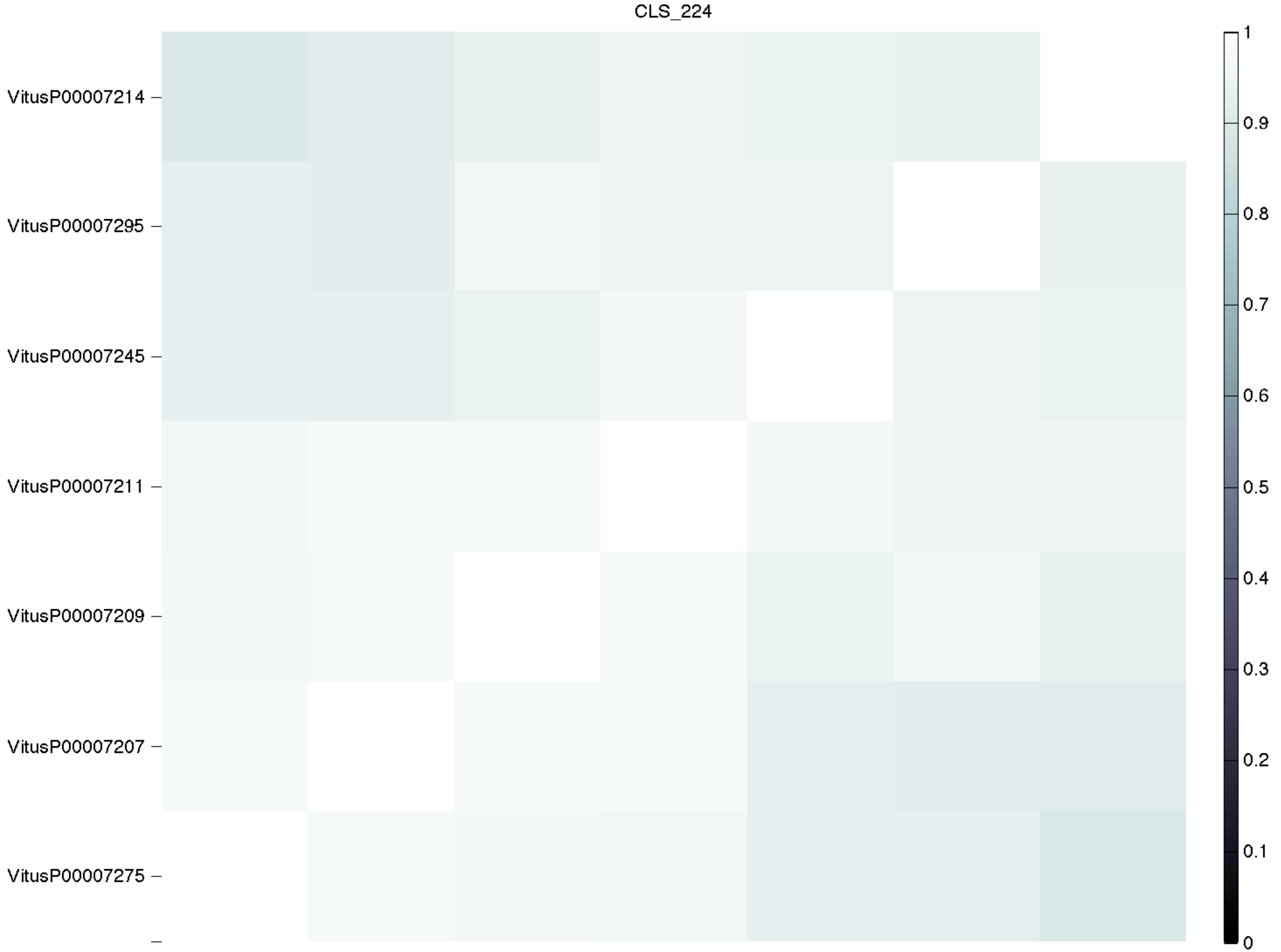
CLS_224
VitusP00007214
VitusP00007295
VitusP00007245
VitusP00007211
VitusP00007209
VitusP00007207
VitusP00007275
1
0.9
0.8
0.7
0.6
0.5
0.4
0.3
0.2
0.1
0

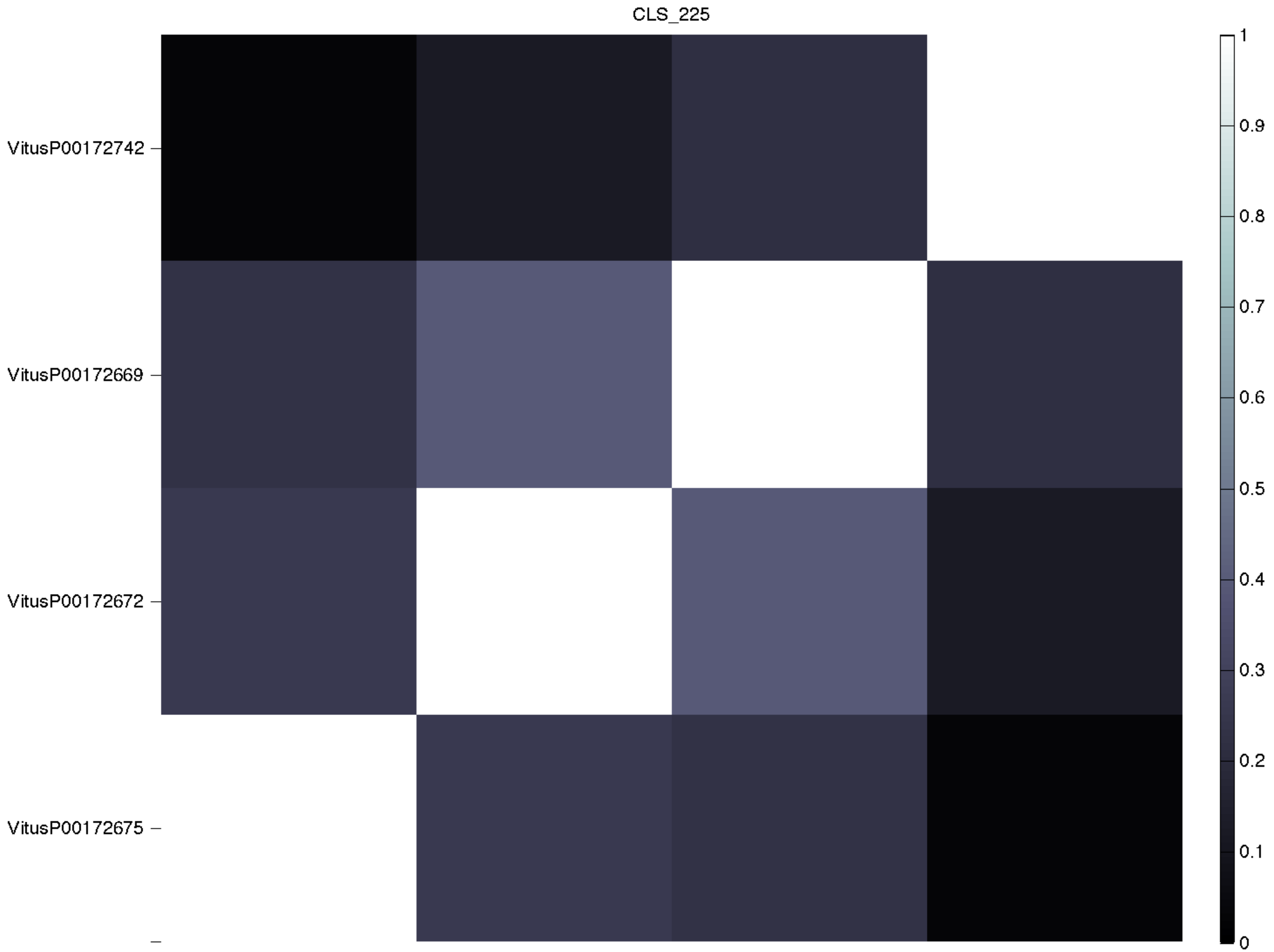
CLS_225
VitusP00172742
VitusP00172669
VitusP00172672
VitusP00172675
1
0.9
0.8
0.7
0.6
0.5
0.4
0.3
0.2
0.1
0

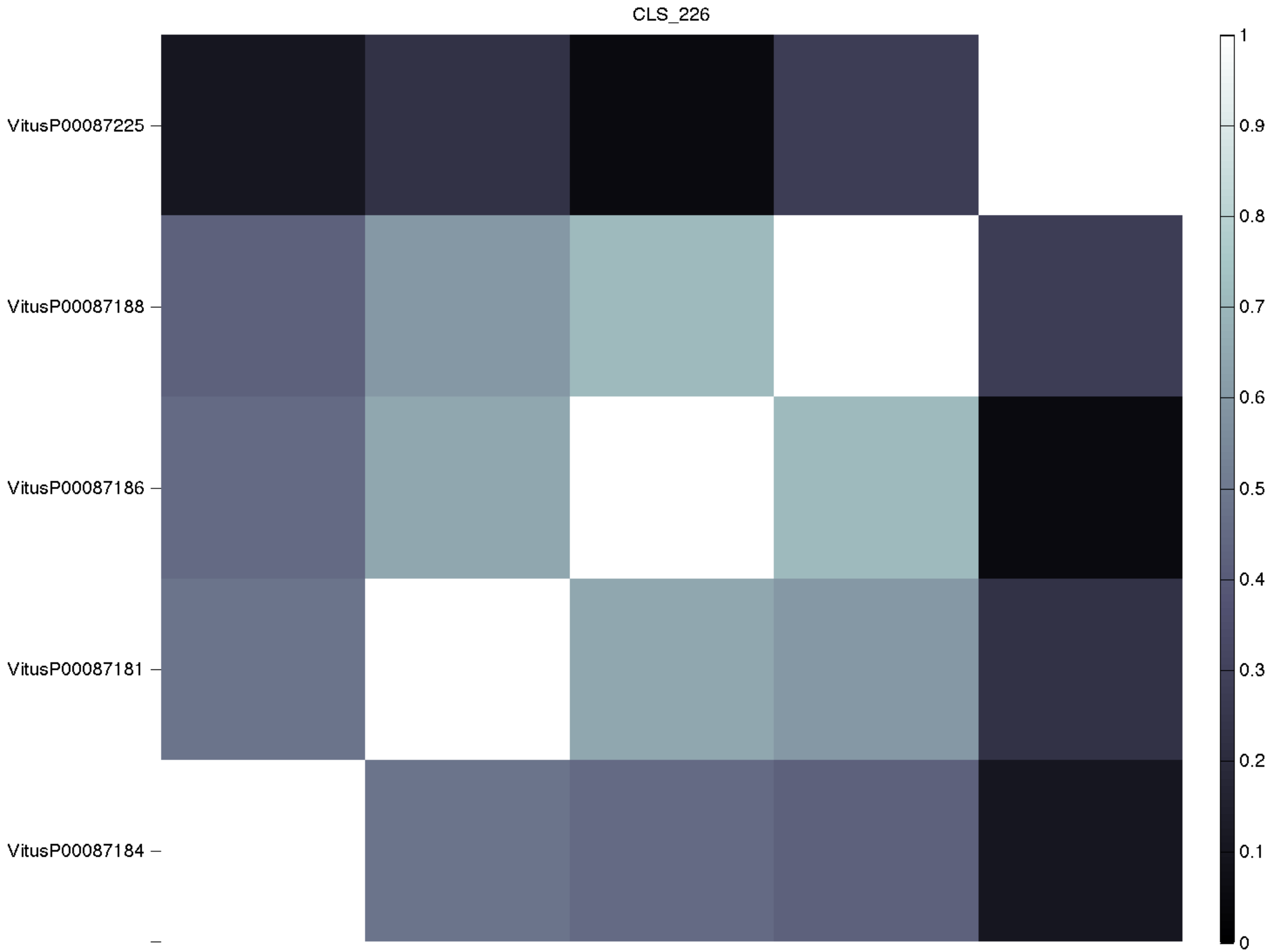
CLS_226
VitusP00087225
VitusP00087188
VitusP00087186
VitusP00087181
VitusP00087184
1
0.9
0.8
0.7
0.6
0.5
0.4
0.3
0.2
0.1
0

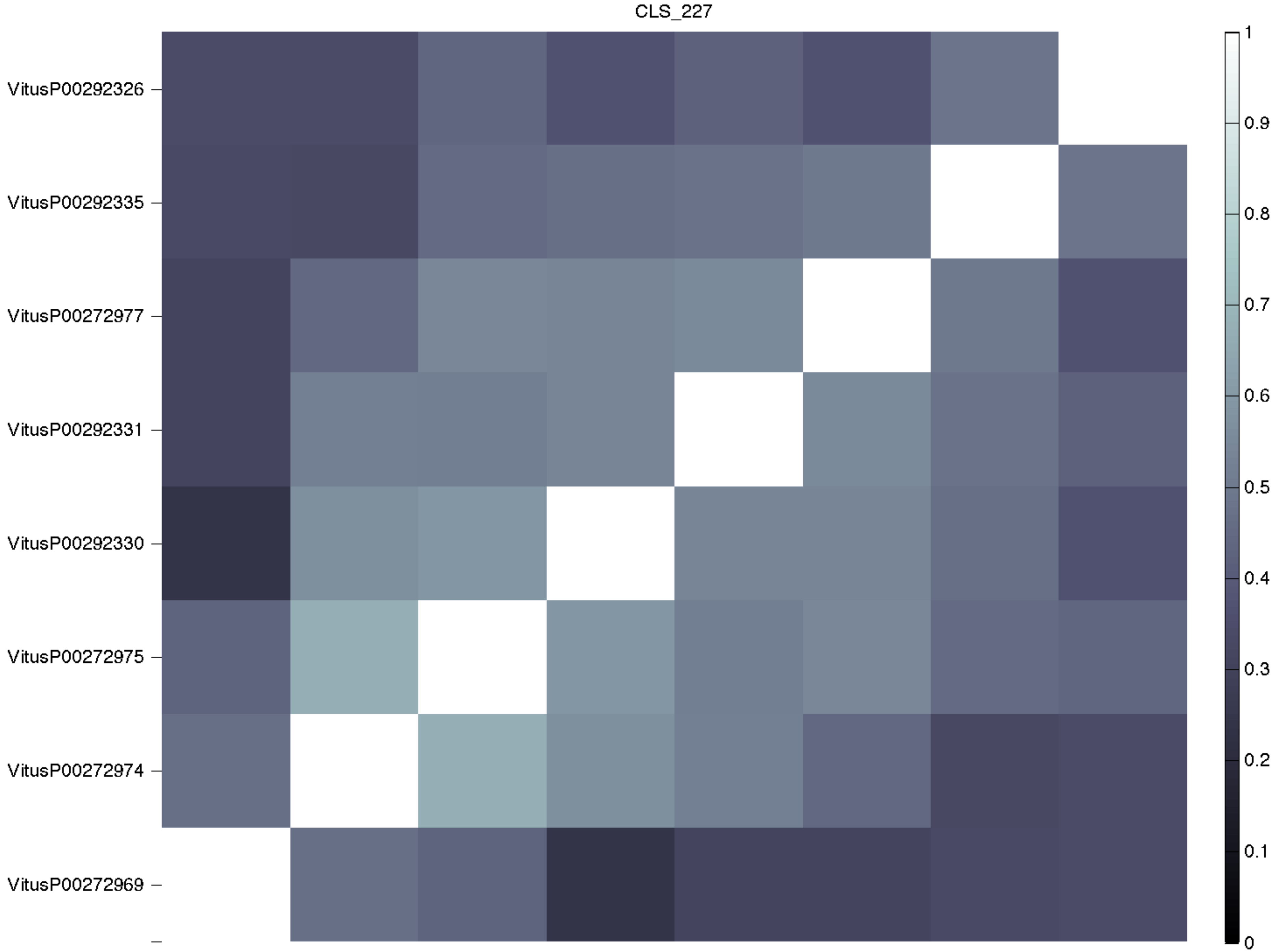

CLS_227
VitusP00292326
VitusP00292335
VitusP00272977
VitusP00292331
VitusP00292330
VitusP00272975
VitusP00272974
VitusP00272969
1
0.9
0.8
0.7
0.6
0.5
0.4
0.3
0.2
0.1
0

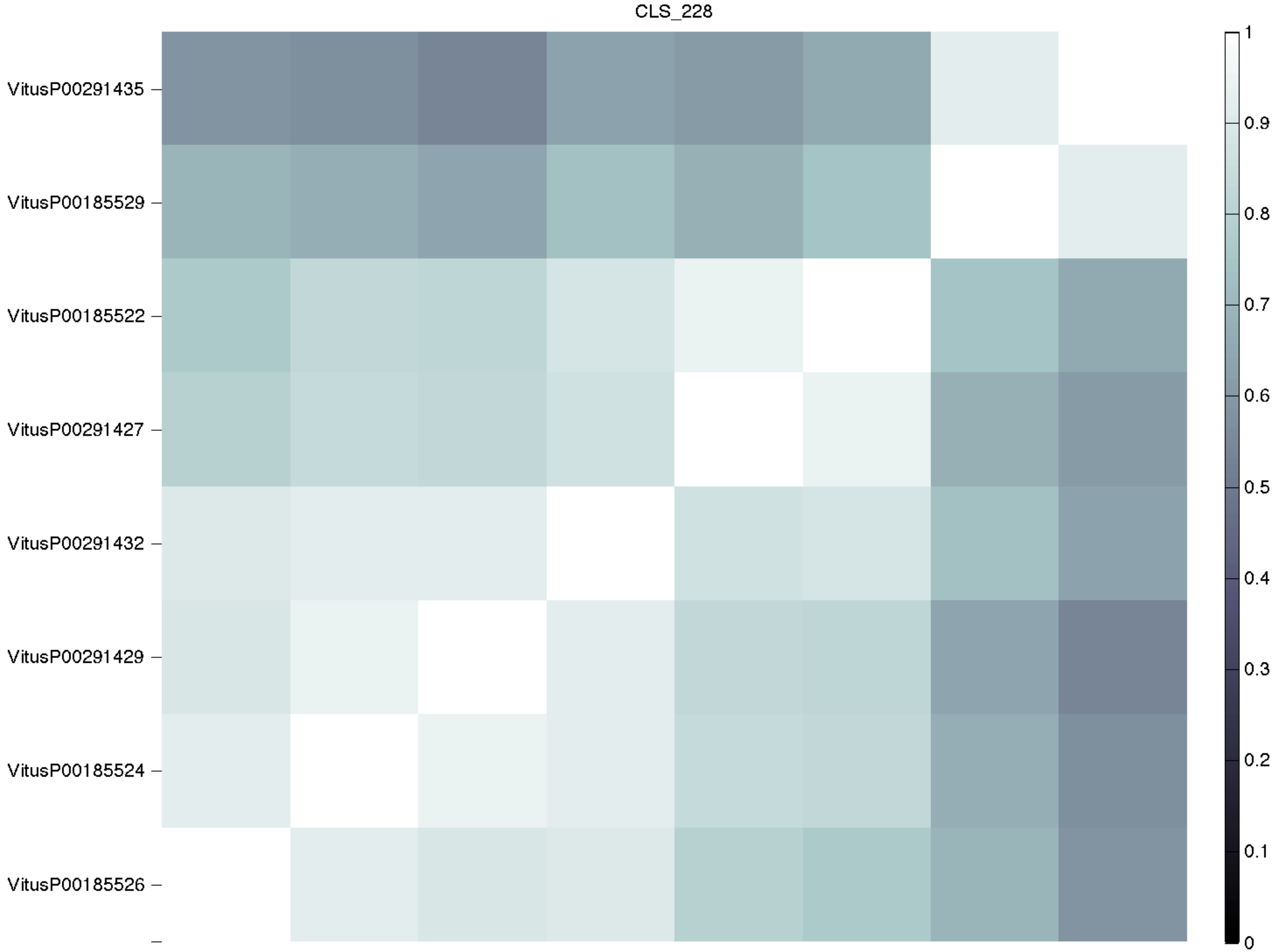
CLS_228
VitusP00291435
VitusP00185529
VitusP00185522
VitusP00291427
VitusP00291432
VitusP00291429
VitusP00185524
VitusP00185526
1
0.9
0.8
0.7
0.6
0.5
0.4
0.3
0.2
0.1
0

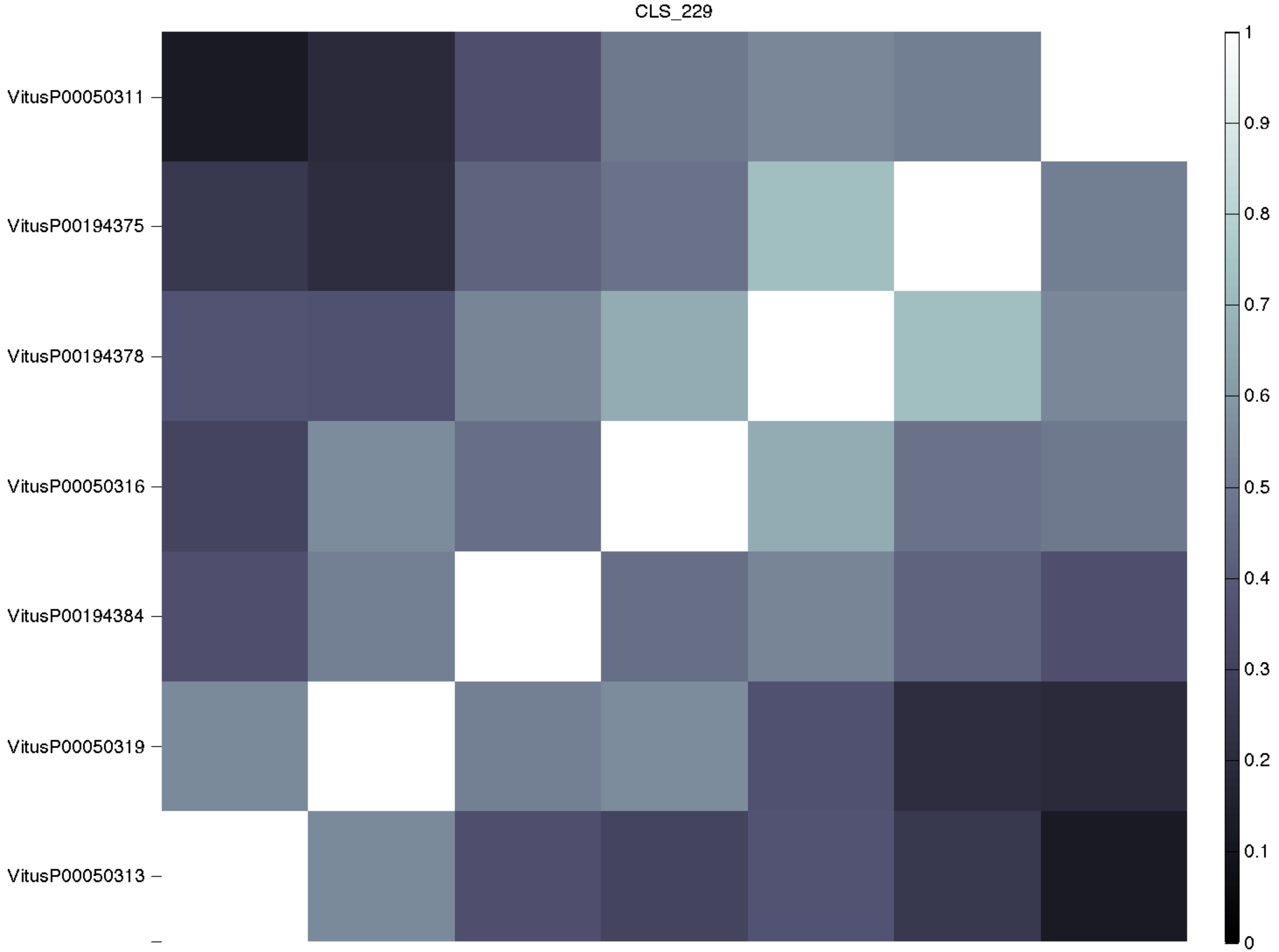

CLS_229
VitusP00050311
VitusP00194375
VitusP00194378
VitusP00050316
VitusP00194384
VitusP00050319
VitusP00050313
1
0.9
0.8
0.7
0.6
0.5
0.4
0.3
0.2
0.1
0

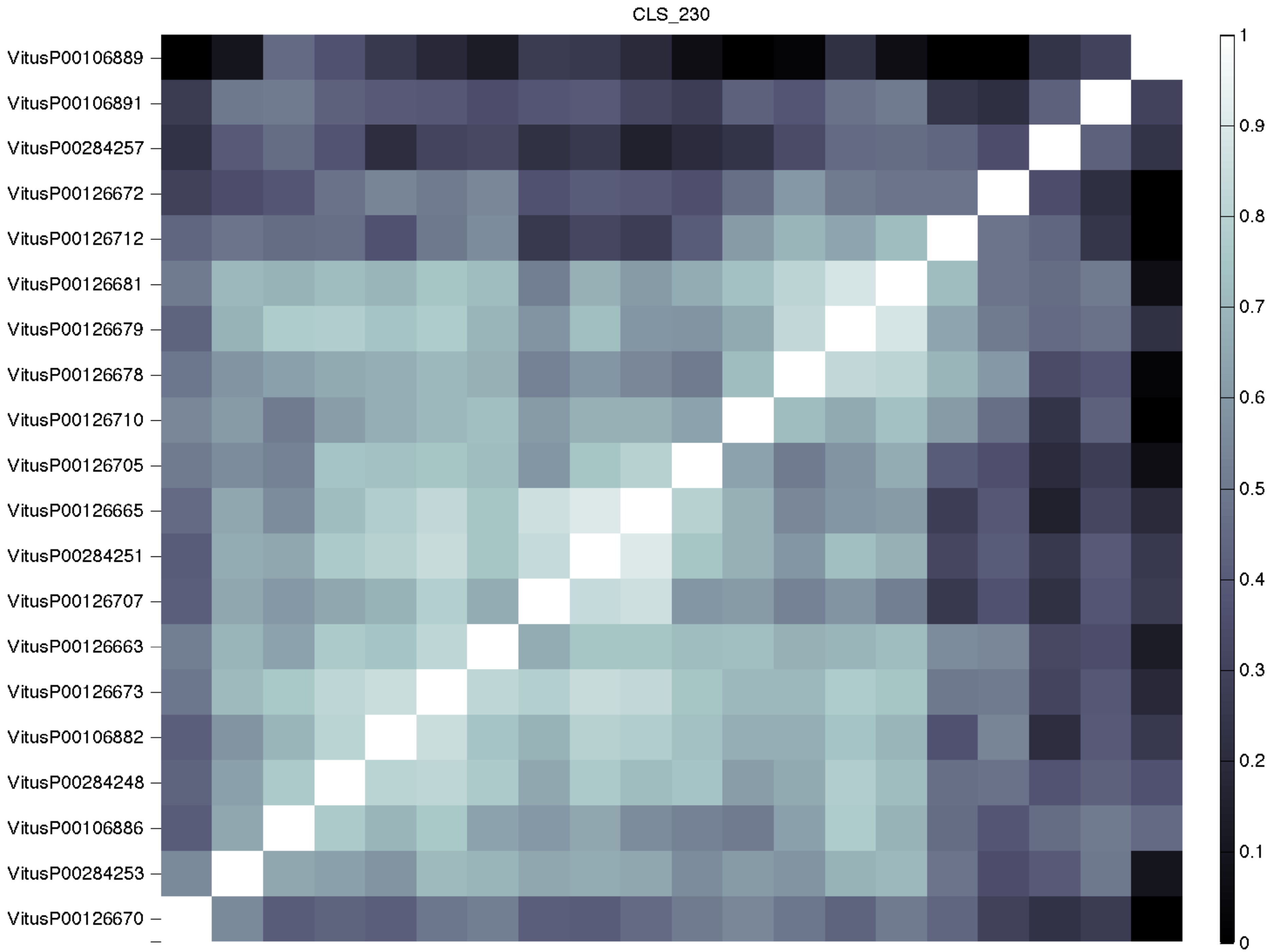
CLS_230
VitusP00106889
VitusP00106891
VitusP00284257
VitusP00126672
VitusP00126712
VitusP00126681
VitusP00126679
VitusP00126678
VitusP00126710
VitusP00126705
VitusP00126665
VitusP00284251
VitusP00126707
VitusP00126663
VitusP00126673
VitusP00106882
VitusP00284248
VitusP00106886
VitusP00284253
VitusP00126670
1
0.9
0.8
0.7
0.6
0.5
0.4
0.3
0.2
0.1
0

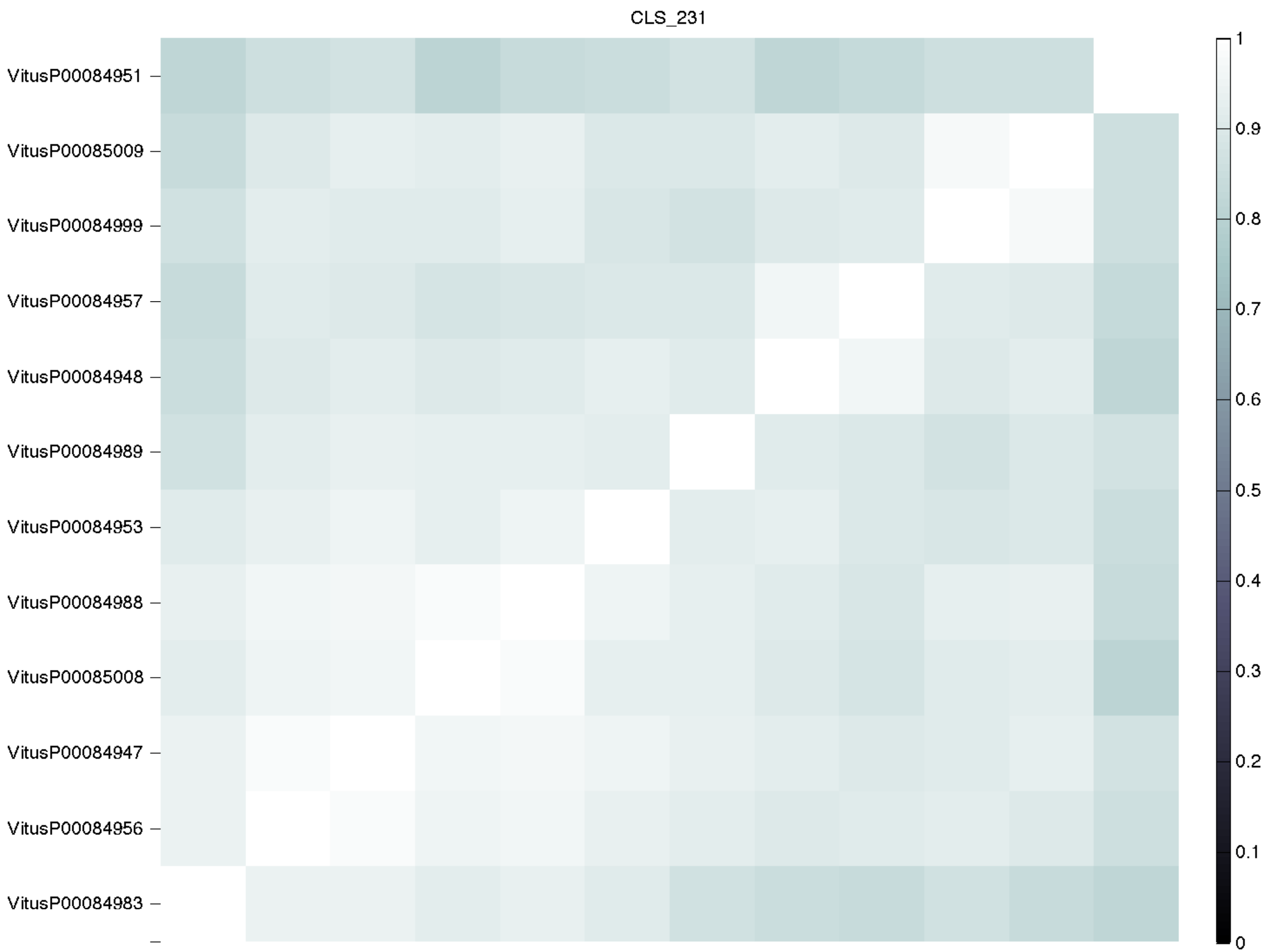
CLS_231
VitusP00084951
VitusP00085009
VitusP00084999
VitusP00084957
VitusP00084948
VitusP00084989
VitusP00084953
VitusP00084988
VitusP00085008
VitusP00084947
VitusP00084956
VitusP00084983
1
0.9
0.8
0.7
0.6
0.5
0.4
0.3
0.2
0.1
0

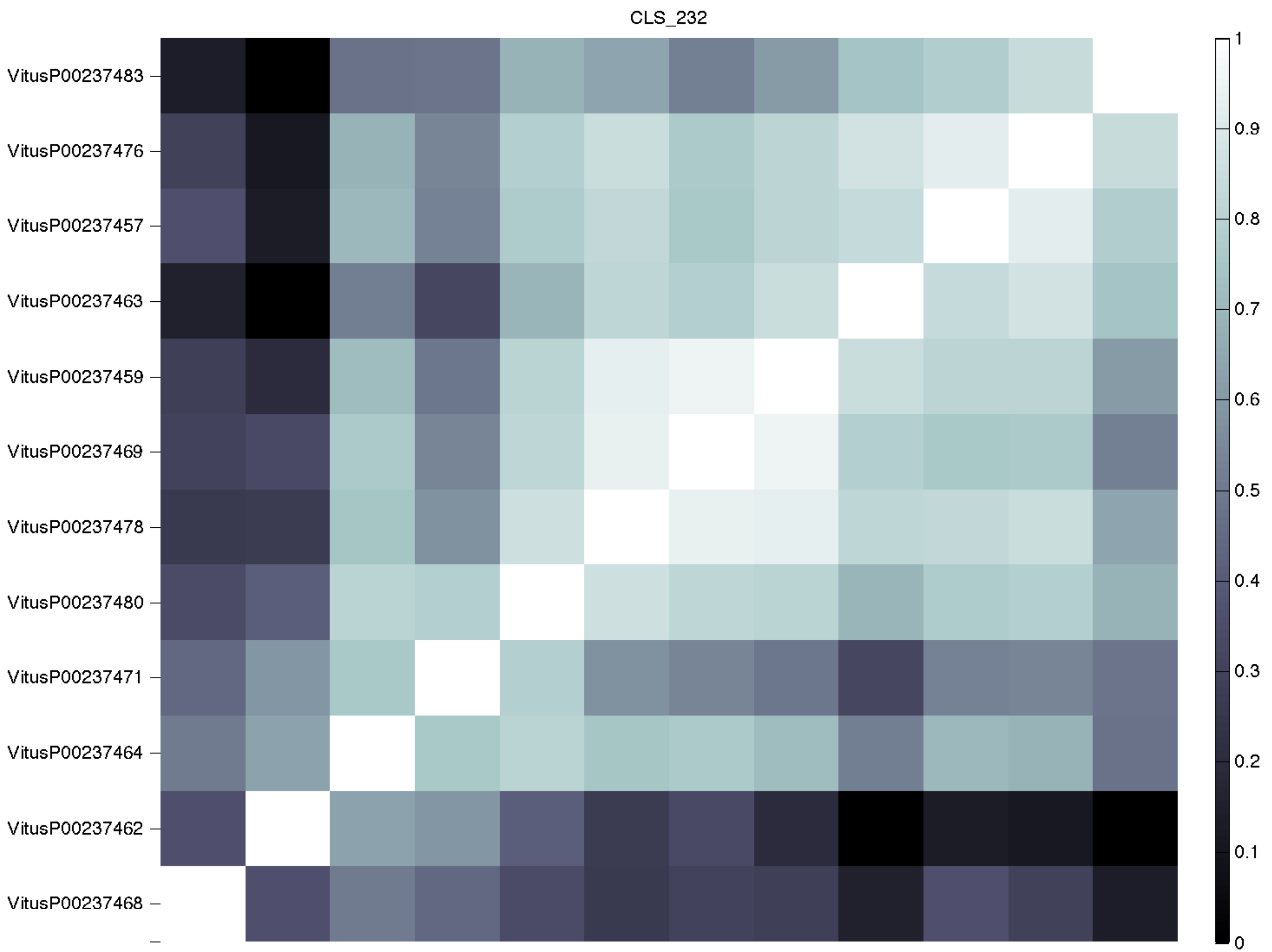
CLS_232
VitusP00237483
VitusP00237476
VitusP00237457
VitusP00237463
VitusP00237459
VitusP00237469
VitusP00237478
VitusP00237480
VitusP00237471
VitusP00237464
VitusP00237462
VitusP00237468
1
0.9
0.8
0.7
0.6
0.5
0.4
0.3
0.2
0.1
0

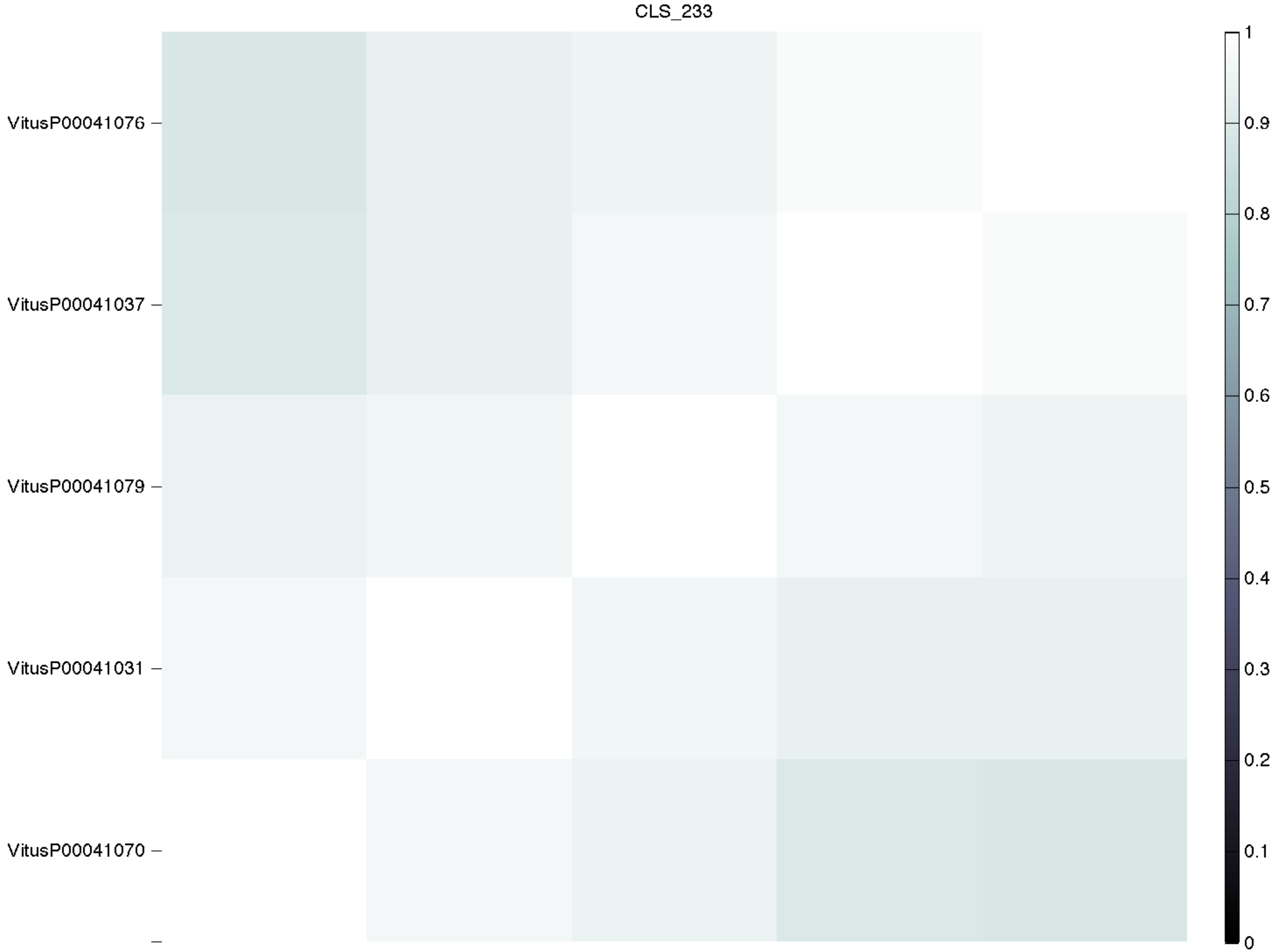

CLS_233
VitusP00041076
VitusP00041037
VitusP00041079
VitusP00041031
VitusP00041070
1
0.9
0.8
0.7
0.6
0.5
0.4
0.3
0.2
0.1
0

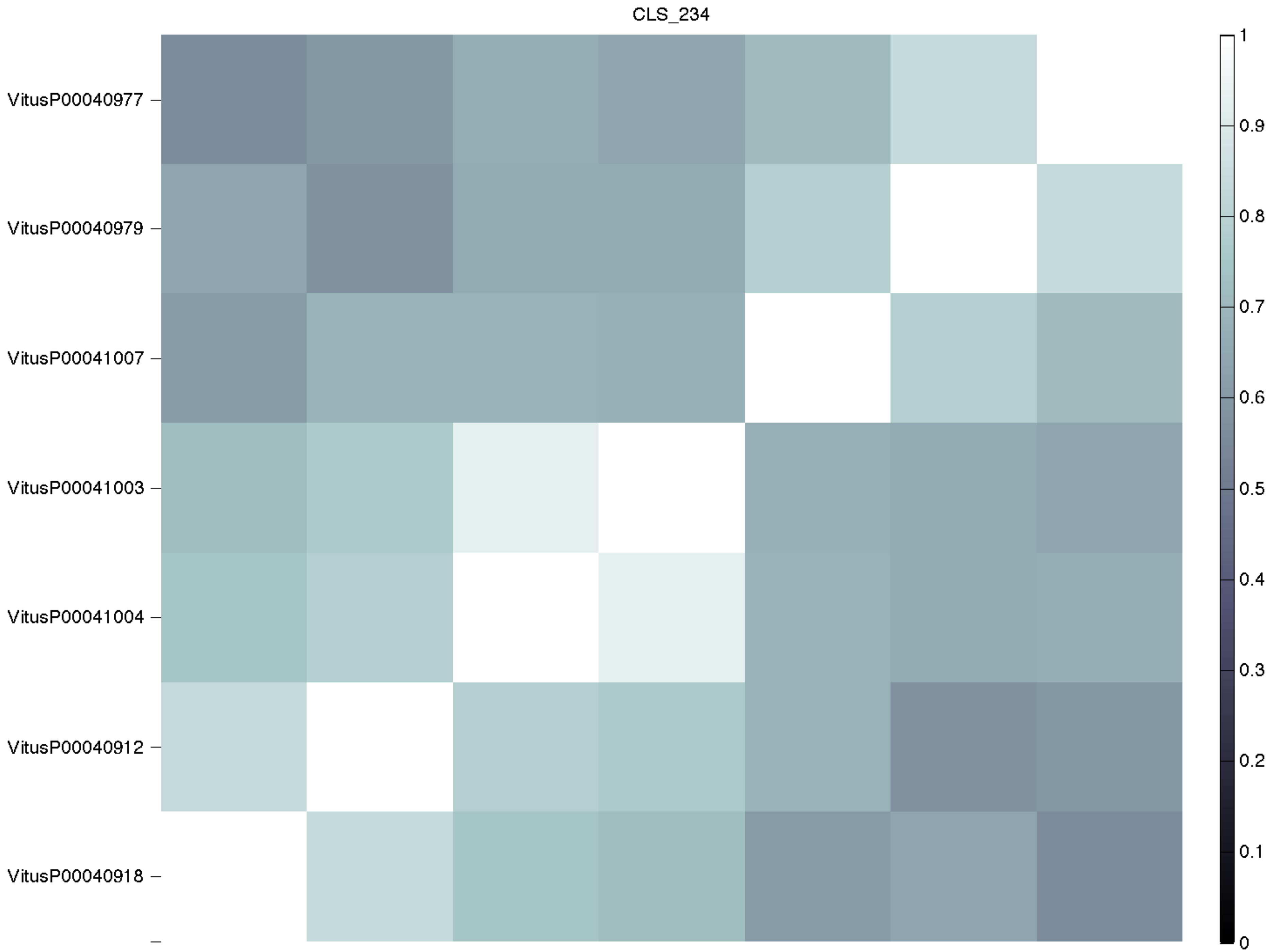
CLS_234
VitusP00040977
VitusP00040979
VitusP00041007
VitusP00041003
VitusP00041004
VitusP00040912
VitusP00040918
1
0.9
0.8
0.7
0.6
0.5
0.4
0.3
0.2
0.1
0

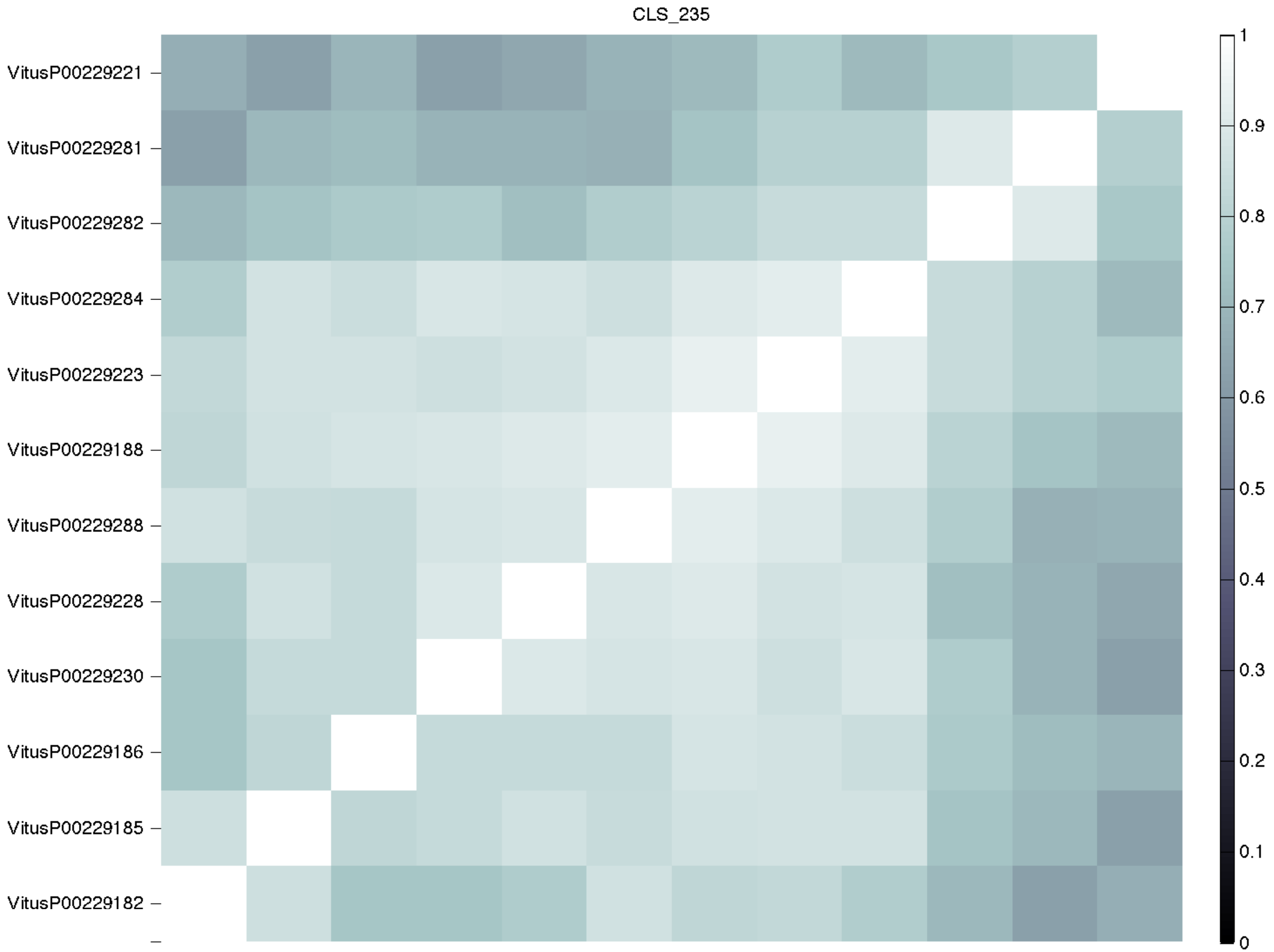

CLS_235
VitusP00229221
VitusP00229281
VitusP00229282
VitusP00229284
VitusP00229223
VitusP00229188
VitusP00229288
VitusP00229228
VitusP00229230
VitusP00229186
VitusP00229185
VitusP00229182
1
0.9
0.8
0.7
0.6
0.5
0.4
0.3
0.2
0.1
0

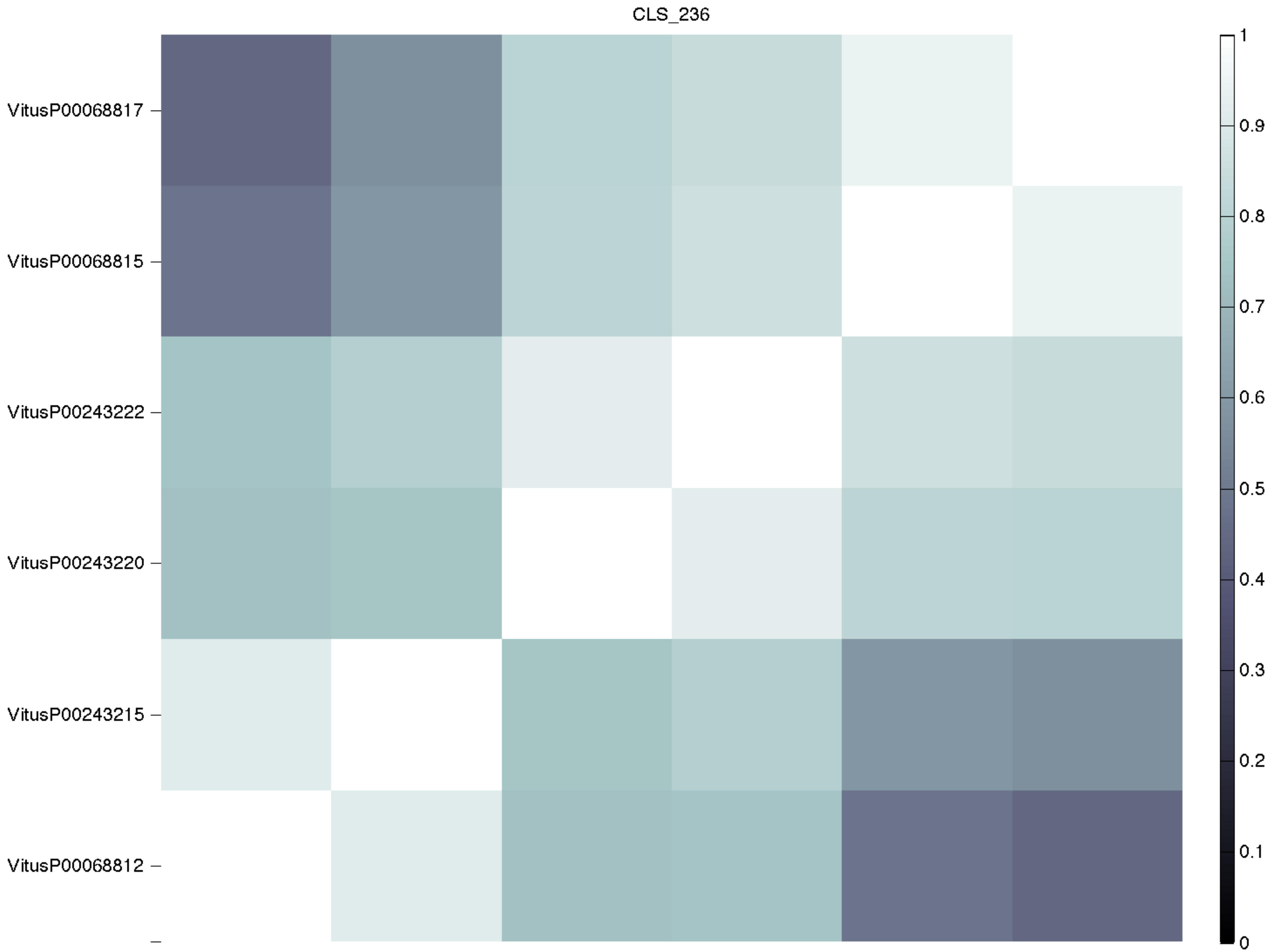
CLS_236
VitusP00068817
VitusP00068815
VitusP00243222
VitusP00243220
VitusP00243215
VitusP00068812
1
0.9
0.8
0.7
0.6
0.5
0.4
0.3
0.2
0.1
0

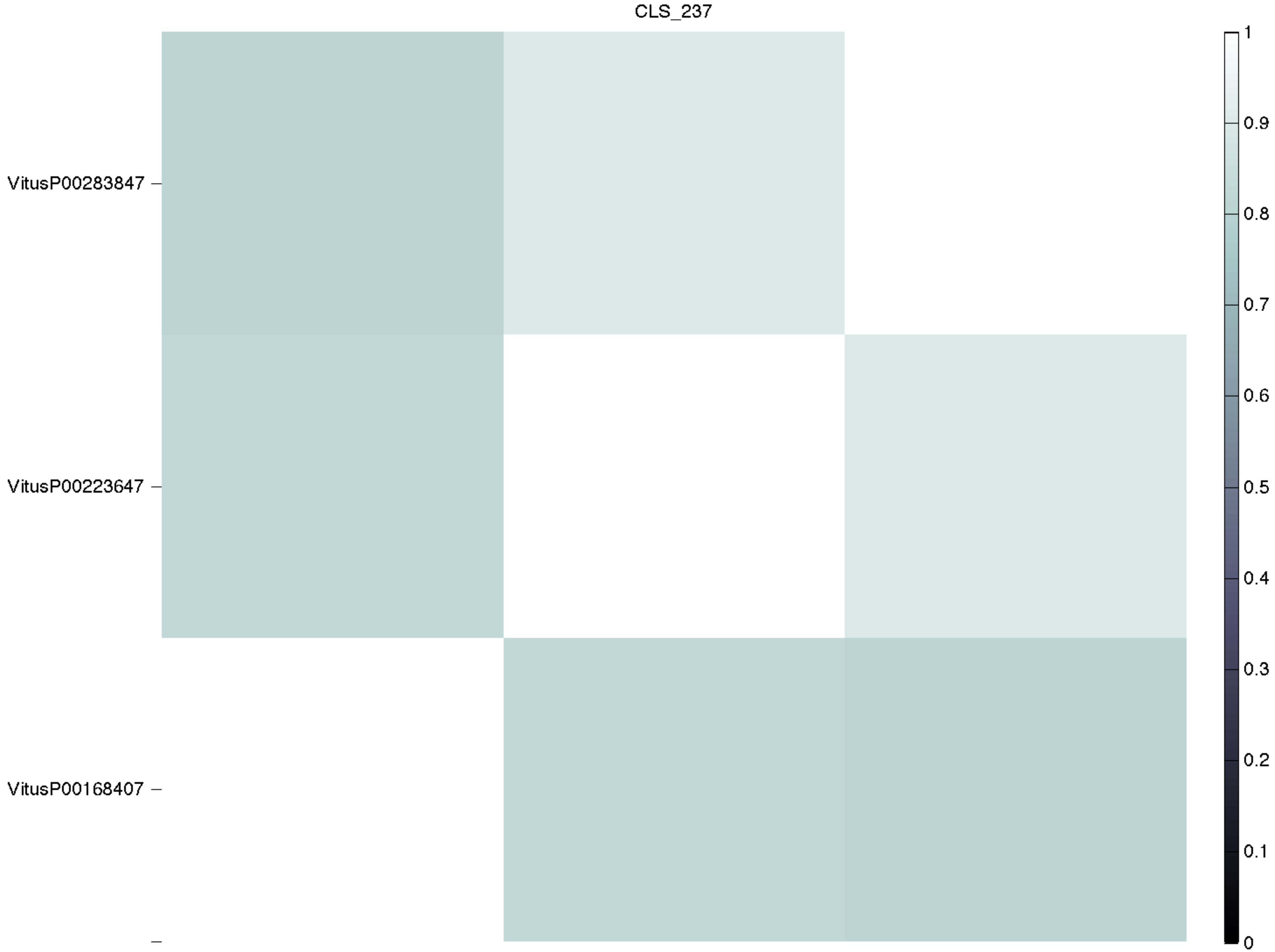

CLS_237
VitusP00283847
VitusP00223647
VitusP00168407
1
0.9
0.8
0.7
0.6
0.5
0.4
0.3
0.2
0.1
0

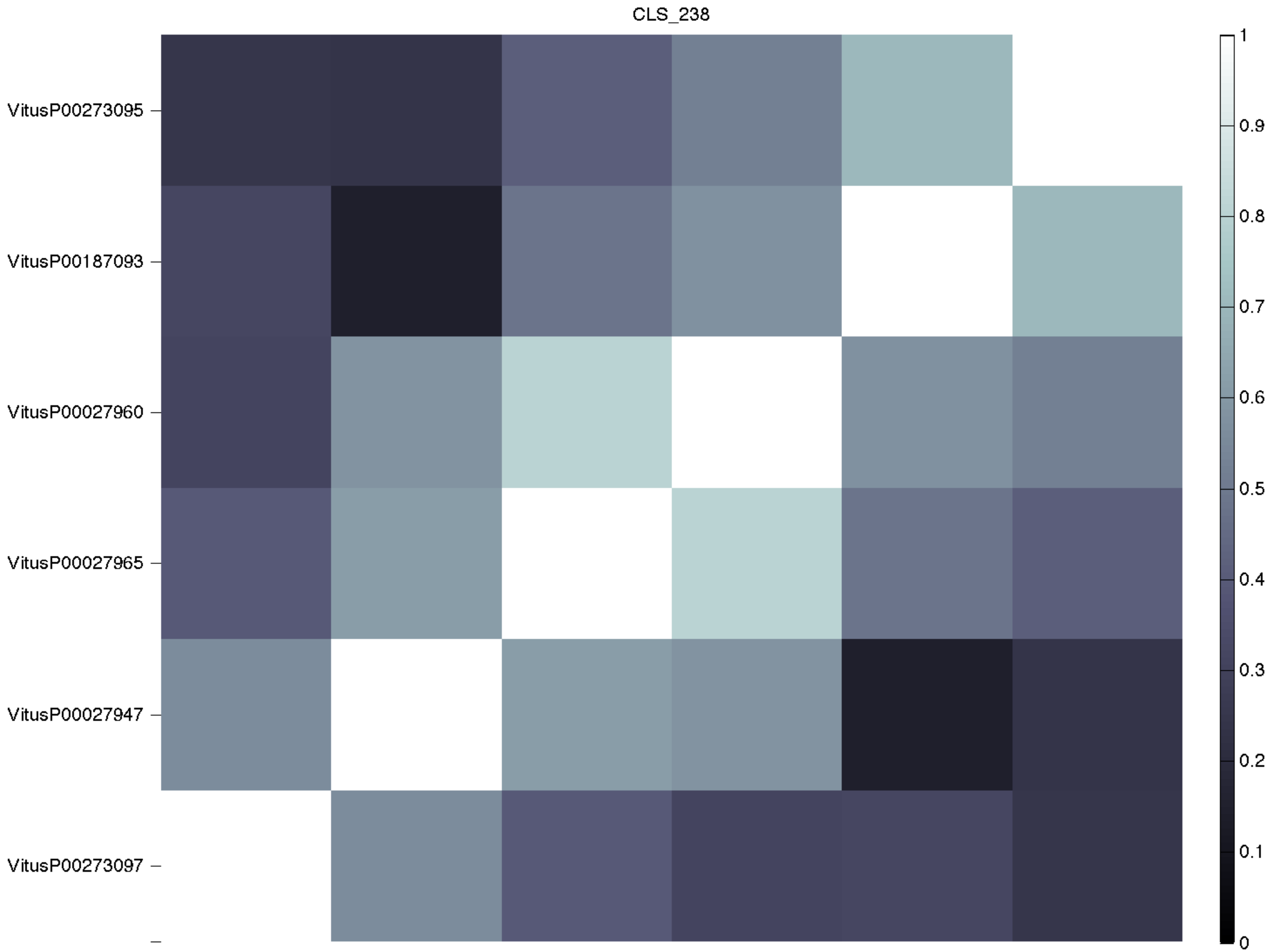
CLS_238
VitusP00273095
VitusP00187093
VitusP00027960
VitusP00027965
VitusP00027947
VitusP00273097
1
0.9
0.8
0.7
0.6
0.5
0.4
0.3
0.2
0.1
0

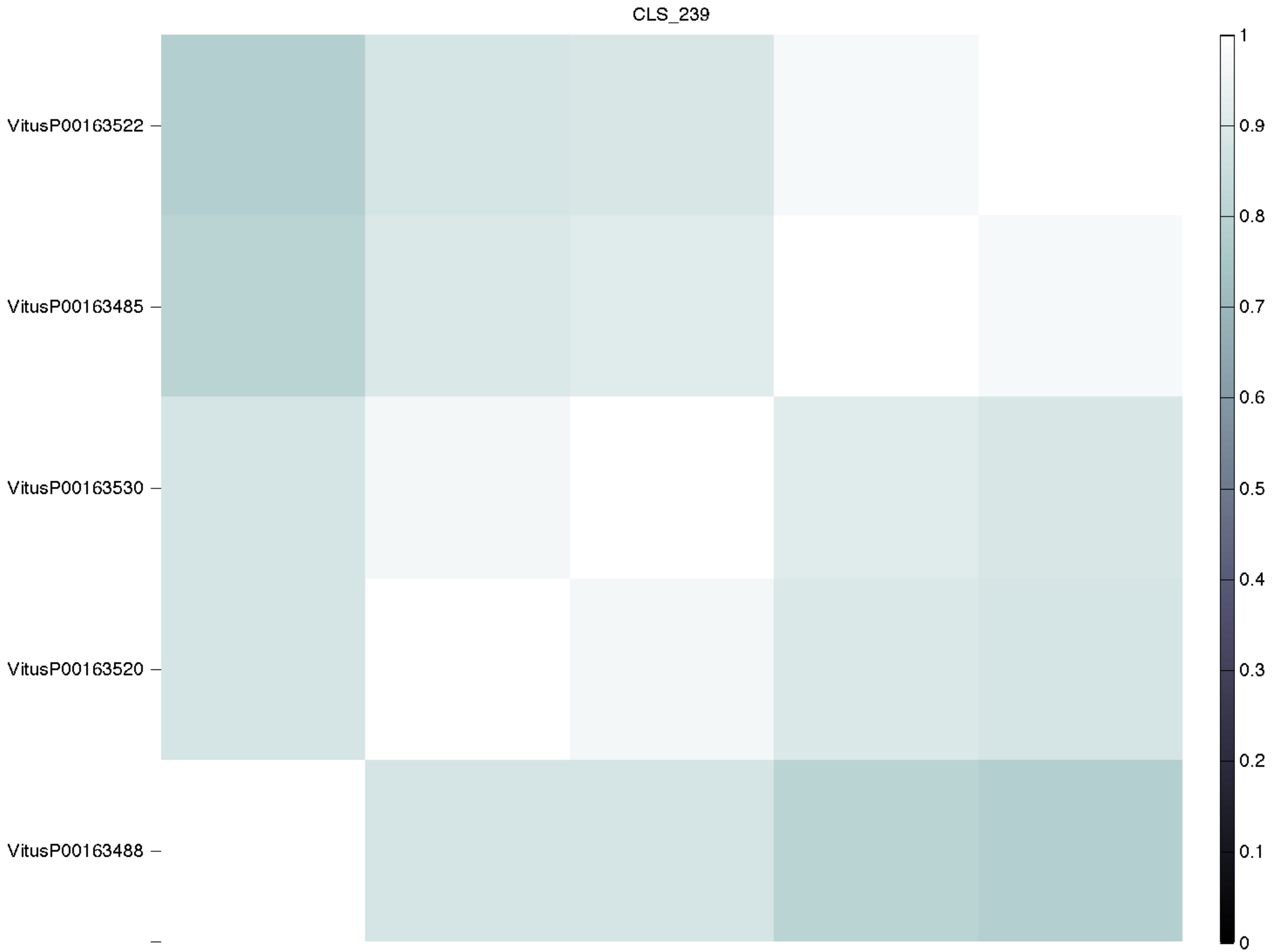
CLS_239
VitusP00163522
VitusP00163485
VitusP00163530
VitusP00163520
VitusP00163488
1
0.9
0.8
0.7
0.6
0.5
0.4
0.3
0.2
0.1
0

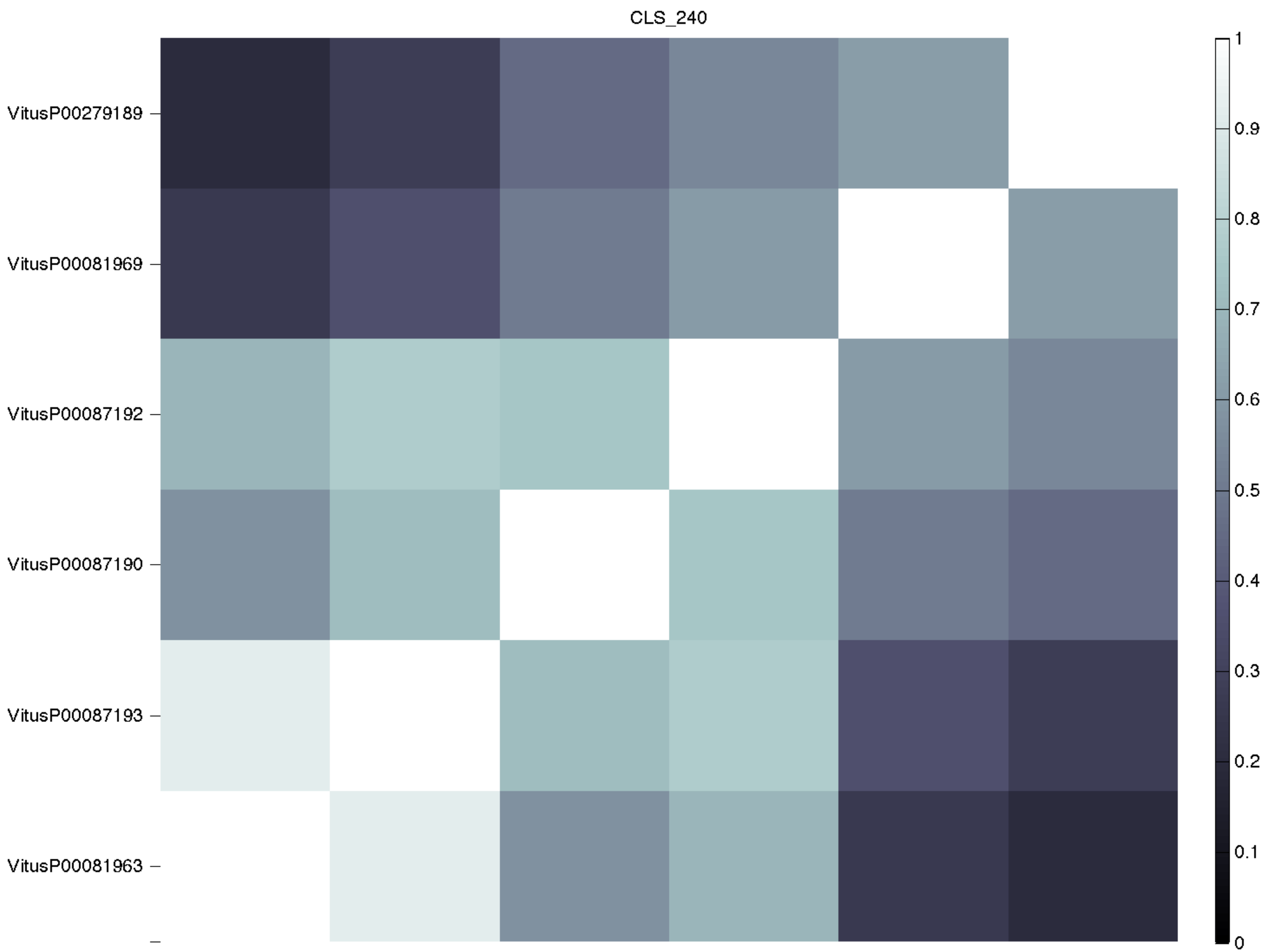
CLS_240
VitusP00279189
VitusP00081969
VitusP00087192
VitusP00087190
VitusP00087193
VitusP00081963
1
0.9
0.8
0.7
0.6
0.5
0.4
0.3
0.2
0.1
0

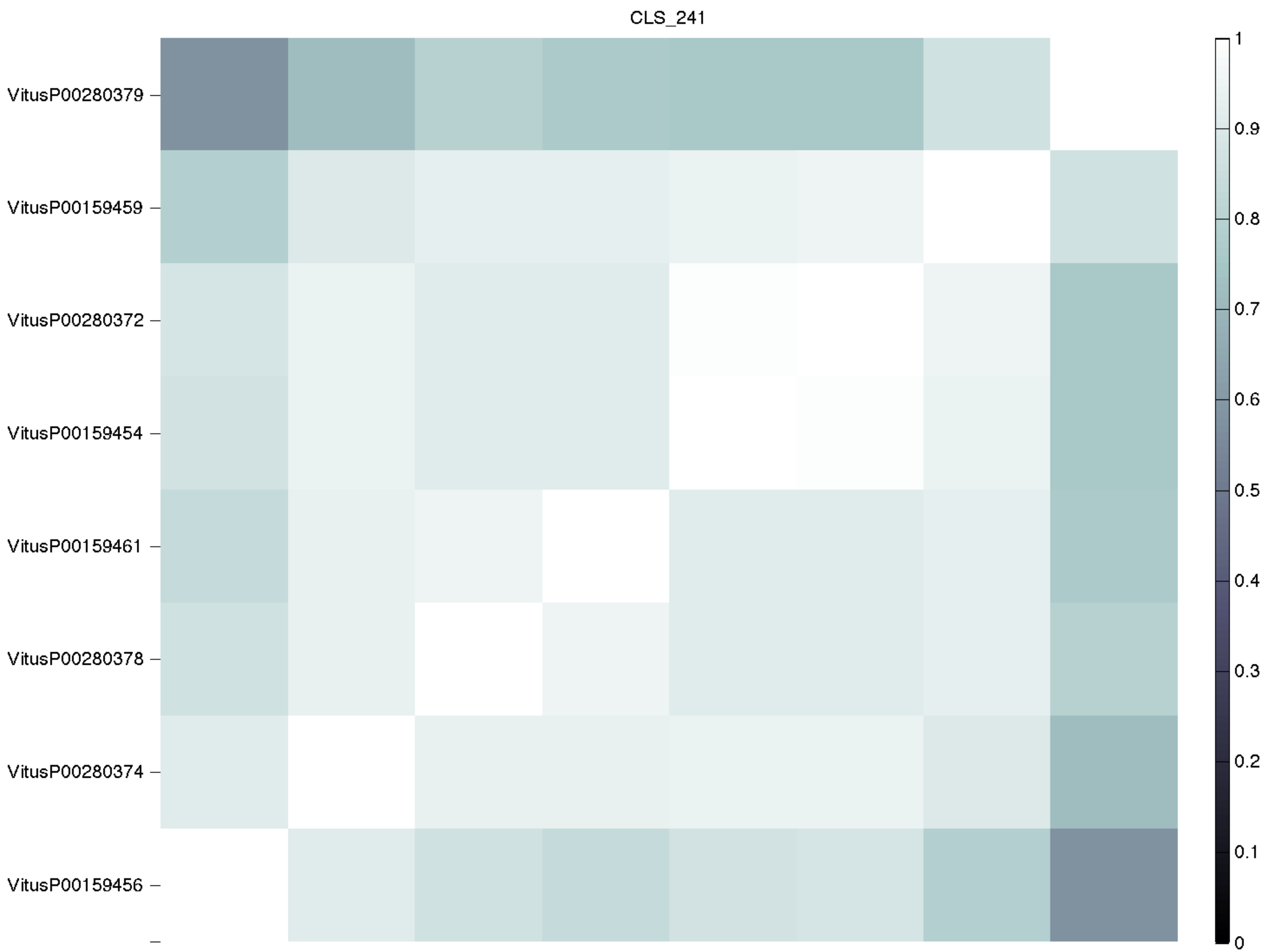
CLS_241
VitusP00280379
VitusP00159459
VitusP00280372
VitusP00159454
VitusP00159461
VitusP00280378
VitusP00280374
VitusP00159456
1
0.9
0.8
0.7
0.6
0.5
0.4
0.3
0.2
0.1
0

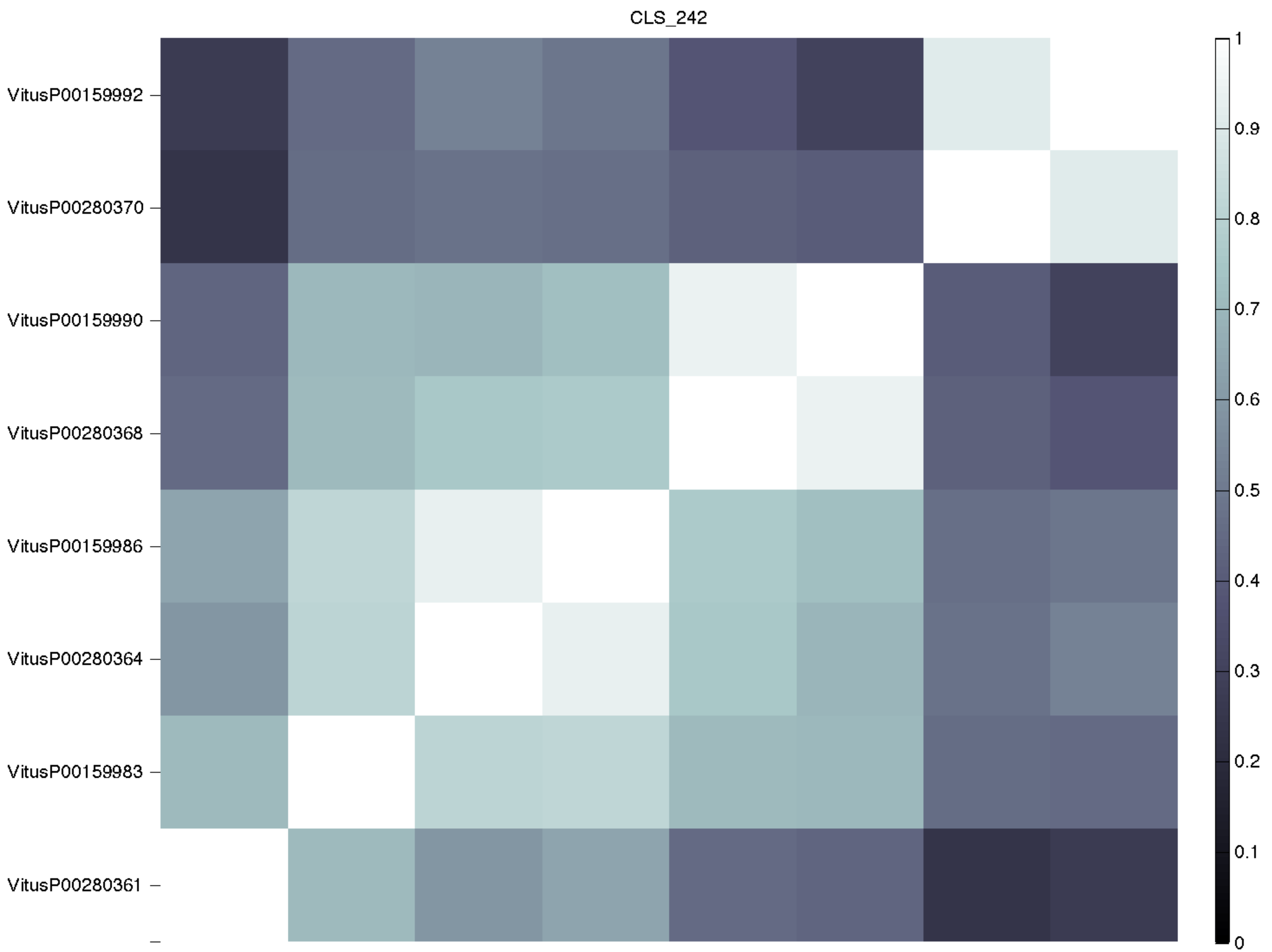

CLS_242
VitusP00159992
VitusP00280370
VitusP00159990
VitusP00280368
VitusP00159986
VitusP00280364
VitusP00159983
VitusP00280361
1
0.9
0.8
0.7
0.6
0.5
0.4
0.3
0.2
0.1
0

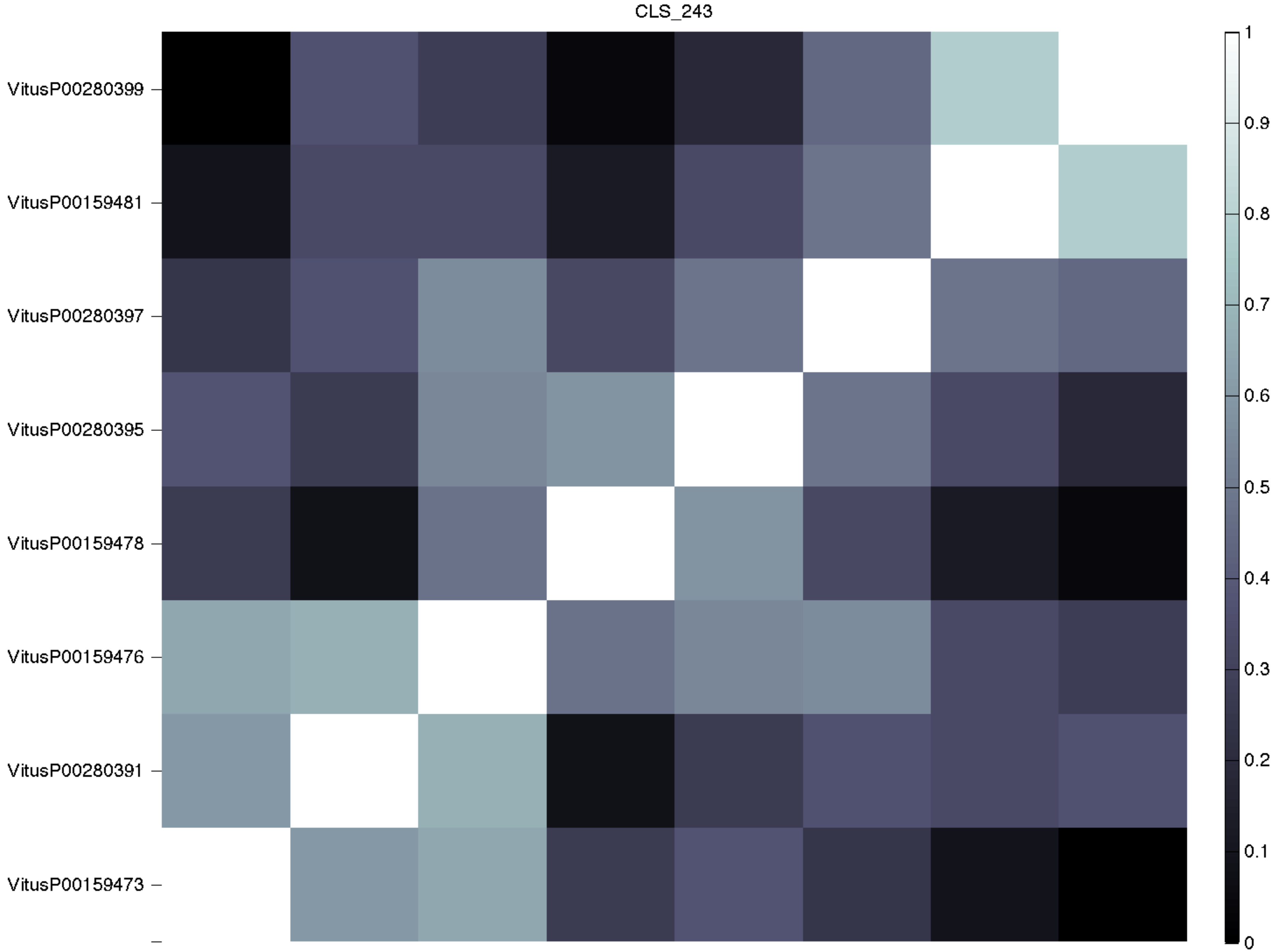
CLS_243
VitusP00280399
VitusP00159481
VitusP00280397
VitusP00280395
VitusP00159478
VitusP00159476
VitusP00280391
VitusP00159473
1
0.9
0.8
0.7
0.6
0.5
0.4
0.3
0.2
0.1
0

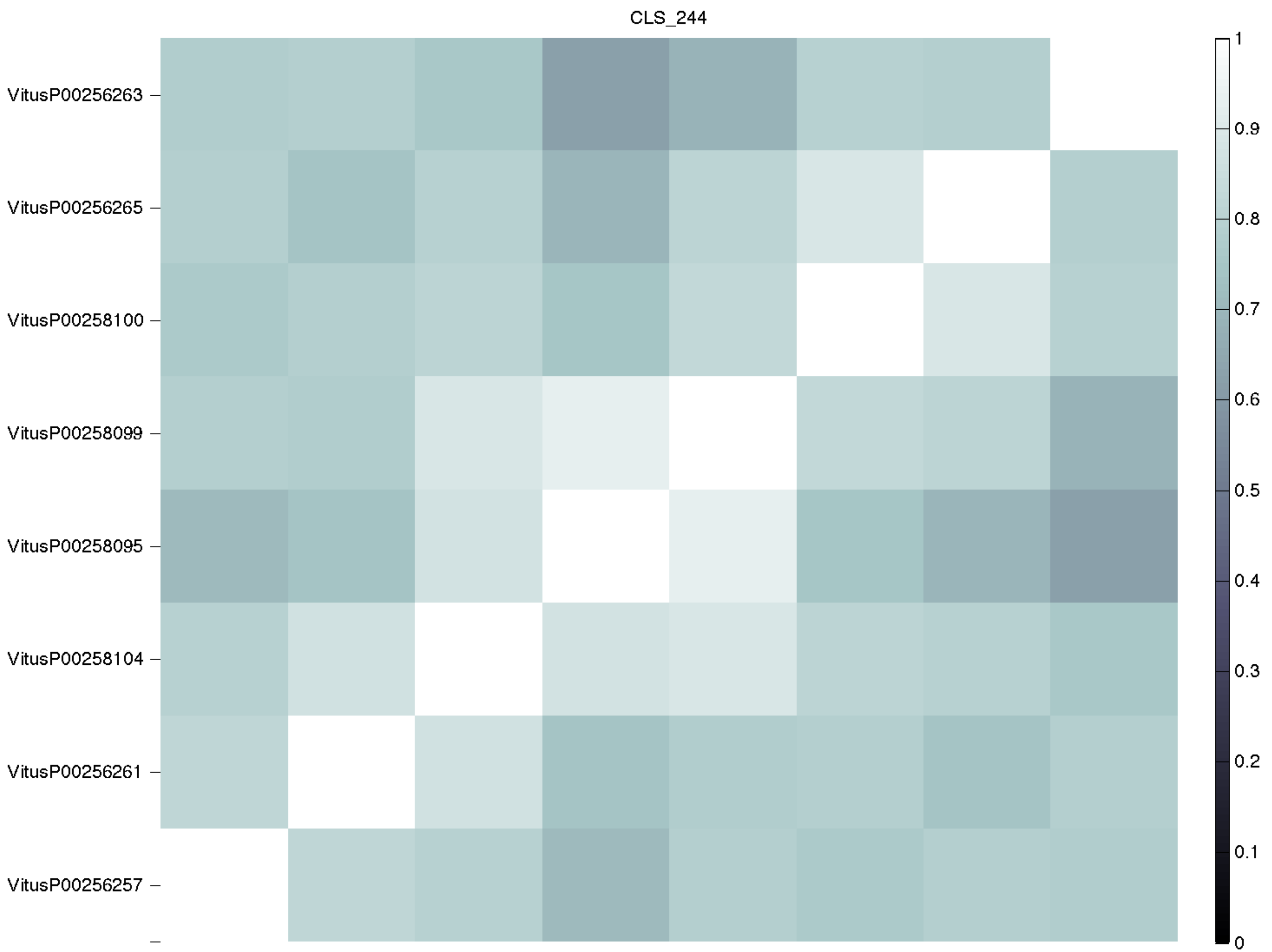

CLS_244
VitusP00256263
VitusP00256265
VitusP00258100
VitusP00258099
VitusP00258095
VitusP00258104
VitusP00256261
VitusP00256257
1
0.9
0.8
0.7
0.6
0.5
0.4
0.3
0.2
0.1
0

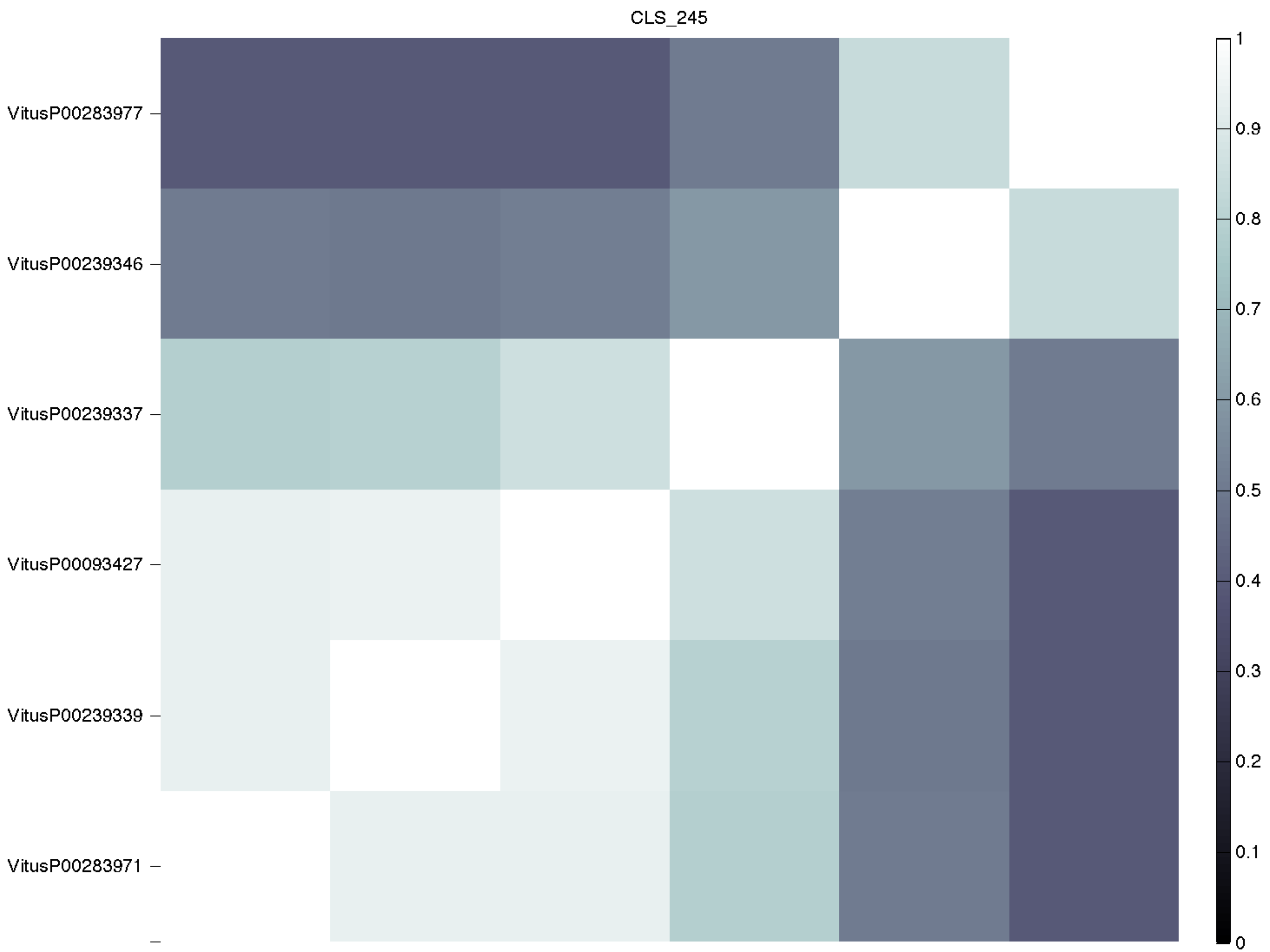
CLS_245
VitusP00283977
VitusP00239346
VitusP00239337
VitusP00093427
VitusP00239339
VitusP00283971
1
0.9
0.8
0.7
0.6
0.5
0.4
0.3
0.2
0.1
0

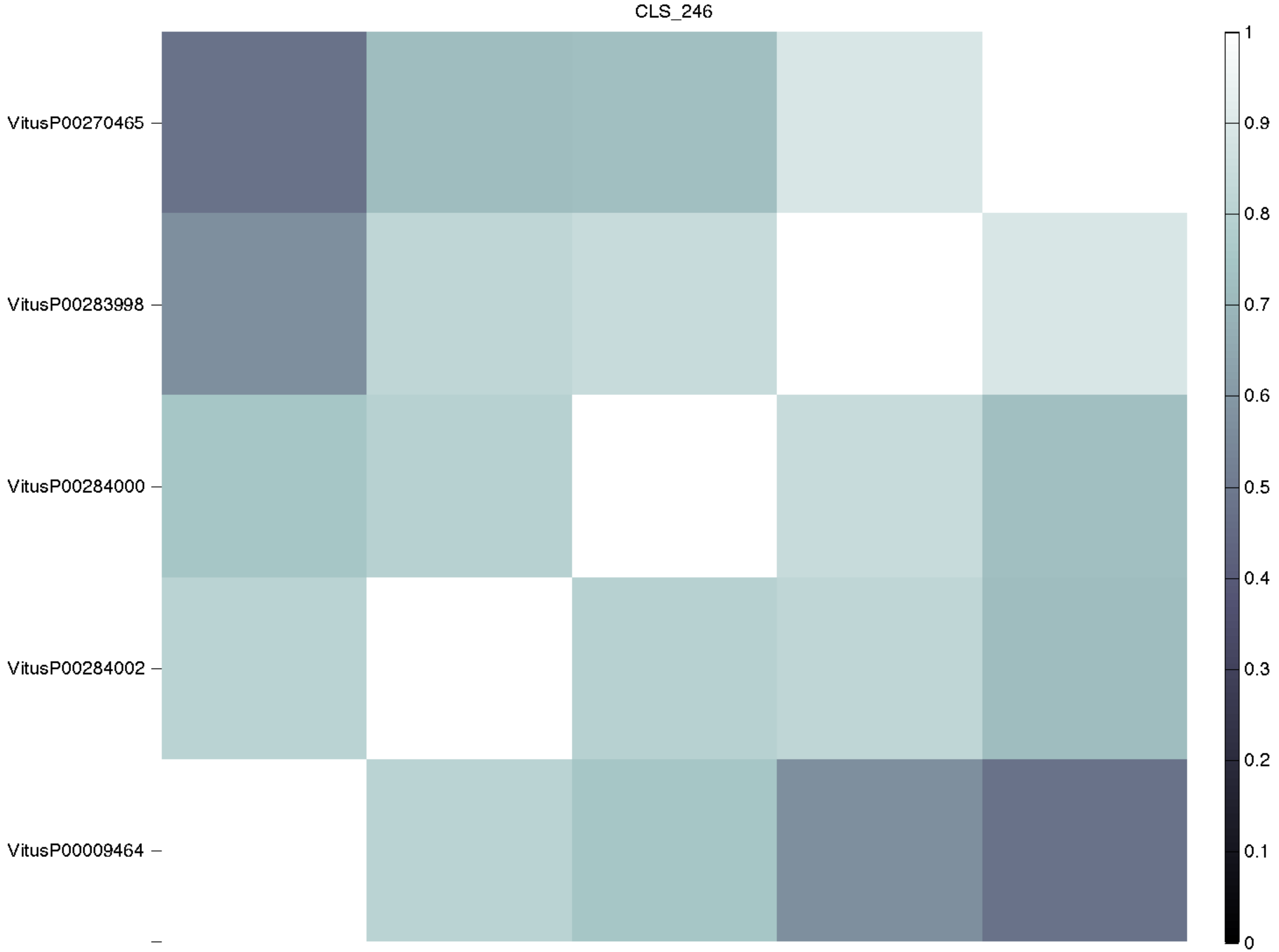

CLS_246
VitusP00270465
VitusP00283998
VitusP00284000
VitusP00284002
VitusP00009464
1
0.9
0.8
0.7
0.6
0.5
0.4
0.3
0.2
0.1
0

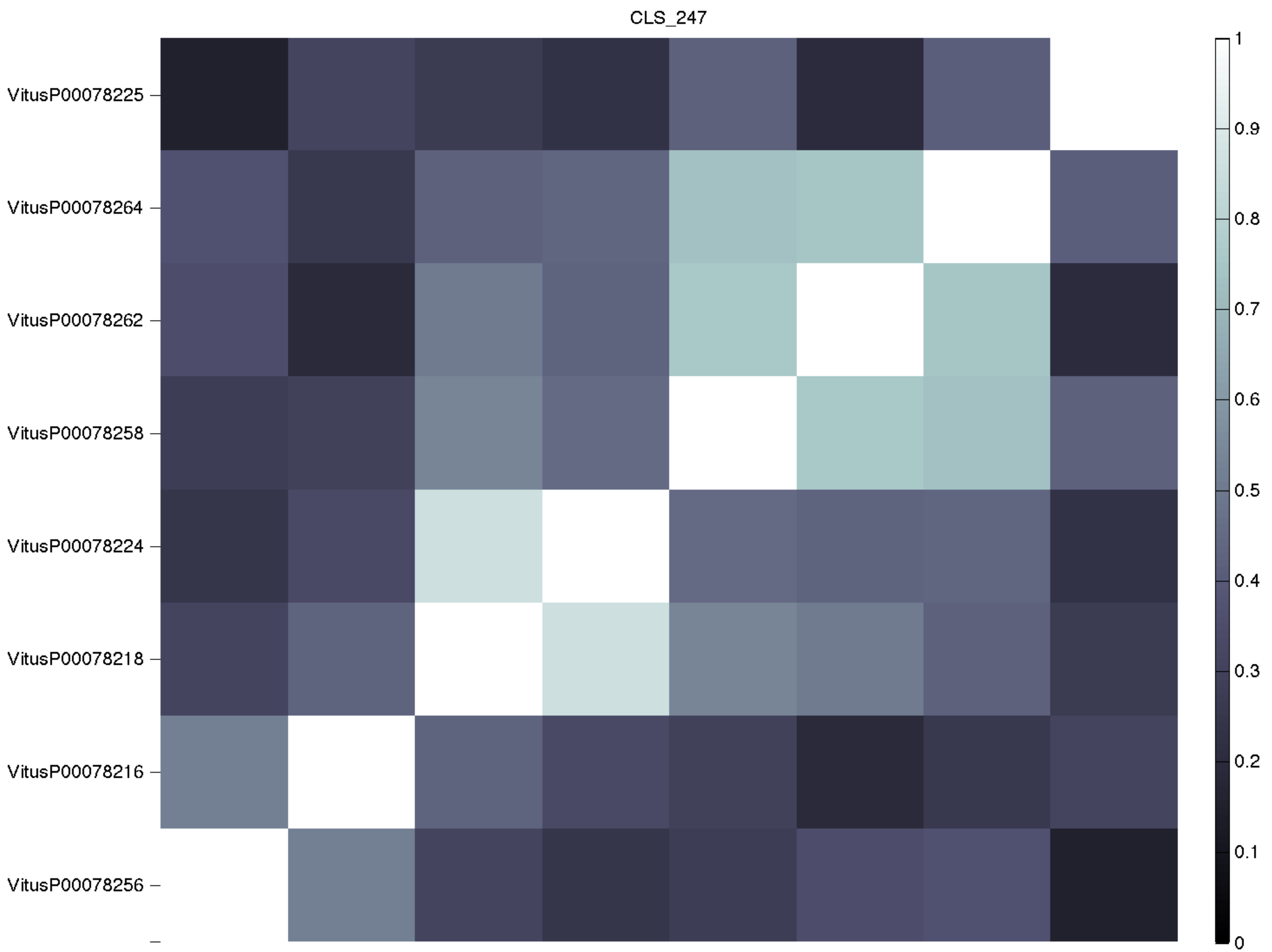

CLS_247
VitusP00078225
VitusP00078264
VitusP00078262
VitusP00078258
VitusP00078224
VitusP00078218
VitusP00078216
VitusP00078256
1
0.9
0.8
0.7
0.6
0.5
0.4
0.3
0.2
0.1
0

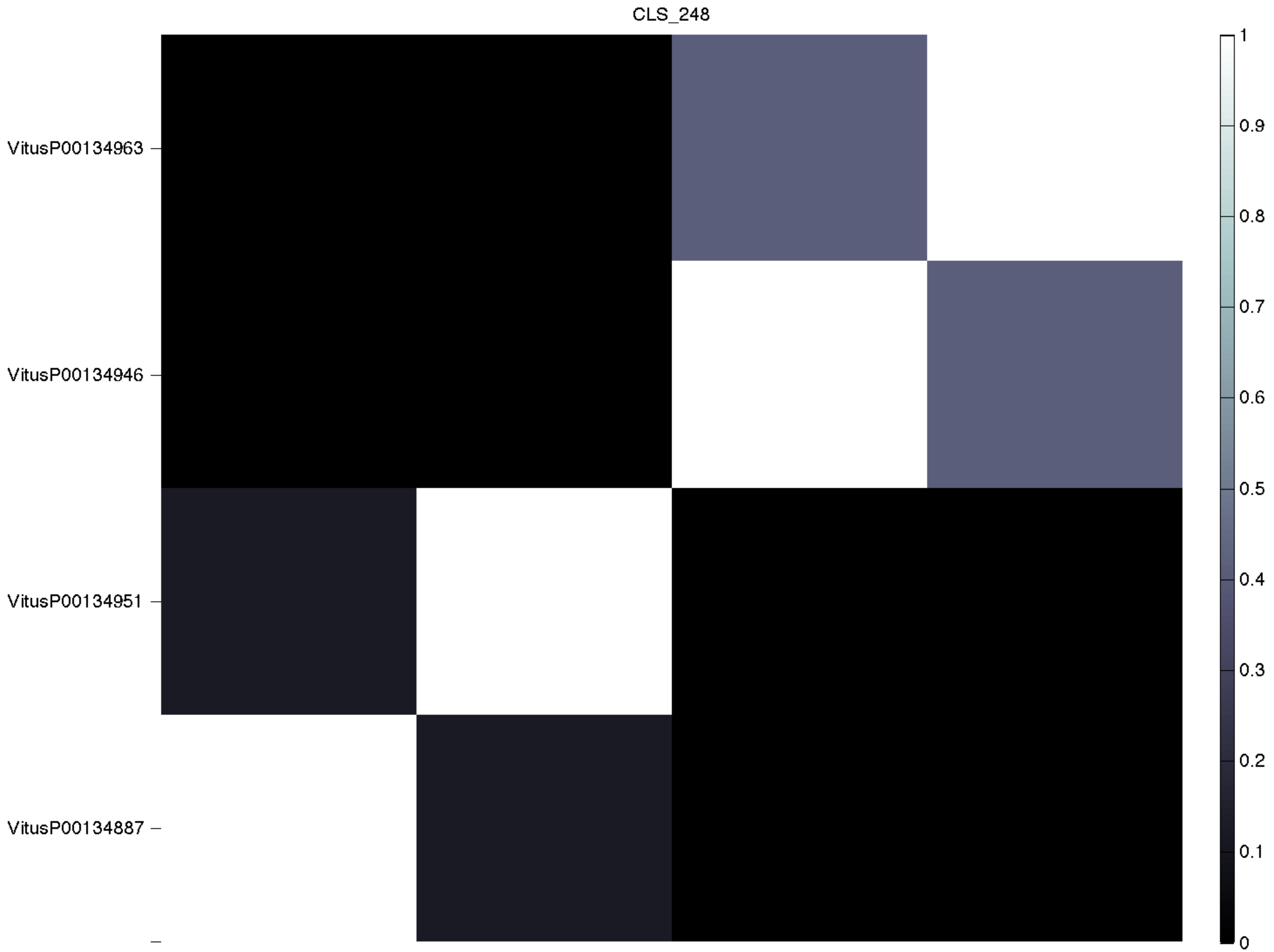
CLS_248
VitusP00134963
VitusP00134946
VitusP00134951
VitusP00134887
1
0.9
0.8
0.7
0.6
0.5
0.4
0.3
0.2
0.1
0

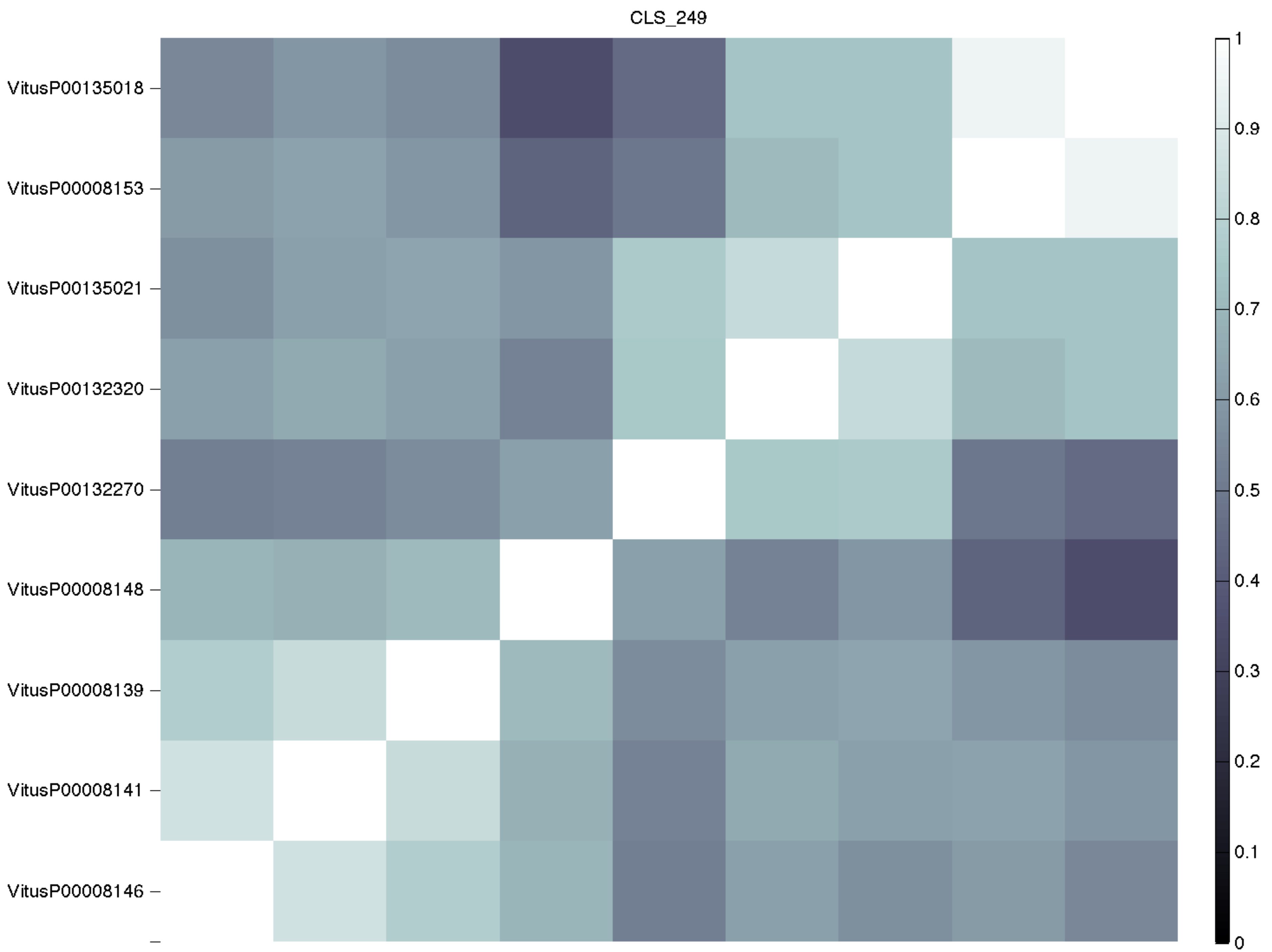
CLS_249
VitusP00135018
VitusP00008153
VitusP00135021
VitusP00132320
VitusP00132270
VitusP00008148
VitusP00008139
VitusP00008141
VitusP00008146
1
0.9
0.8
0.7
0.6
0.5
0.4
0.3
0.2
0.1
0

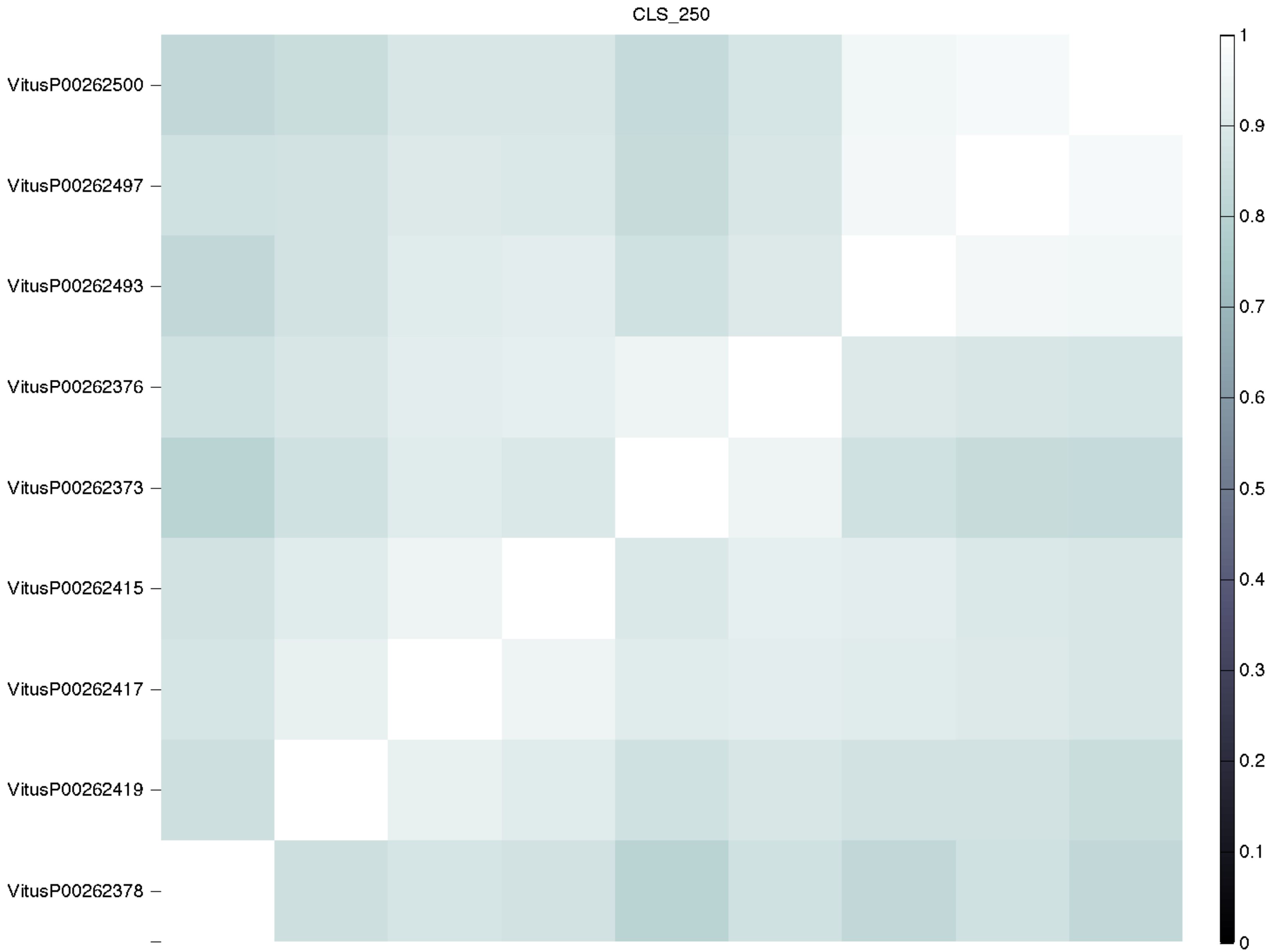
CLS_250
VitusP00262500
VitusP00262497
VitusP00262493
VitusP00262376
VitusP00262373
VitusP00262415
VitusP00262417
VitusP00262419
VitusP00262378
1
0.9
0.8
0.7
0.6
0.5
0.4
0.3
0.2
0.1
0

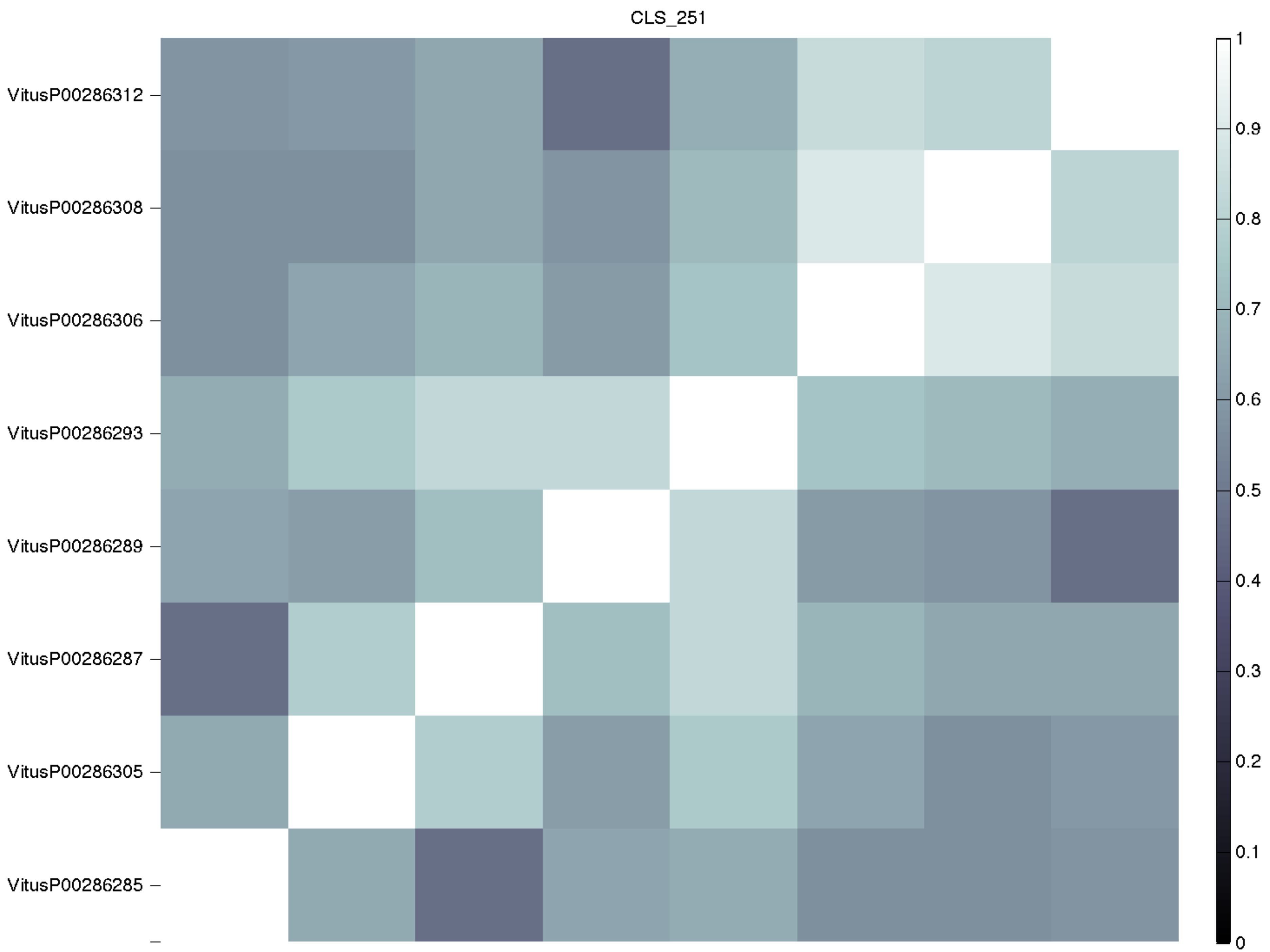
CLS_251
VitusP00286312
VitusP00286308
VitusP00286306
VitusP00286293
VitusP00286289
VitusP00286287
VitusP00286305
VitusP00286285
1
0.9
0.8
0.7
0.6
0.5
0.4
0.3
0.2
0.1
0

CLS_252

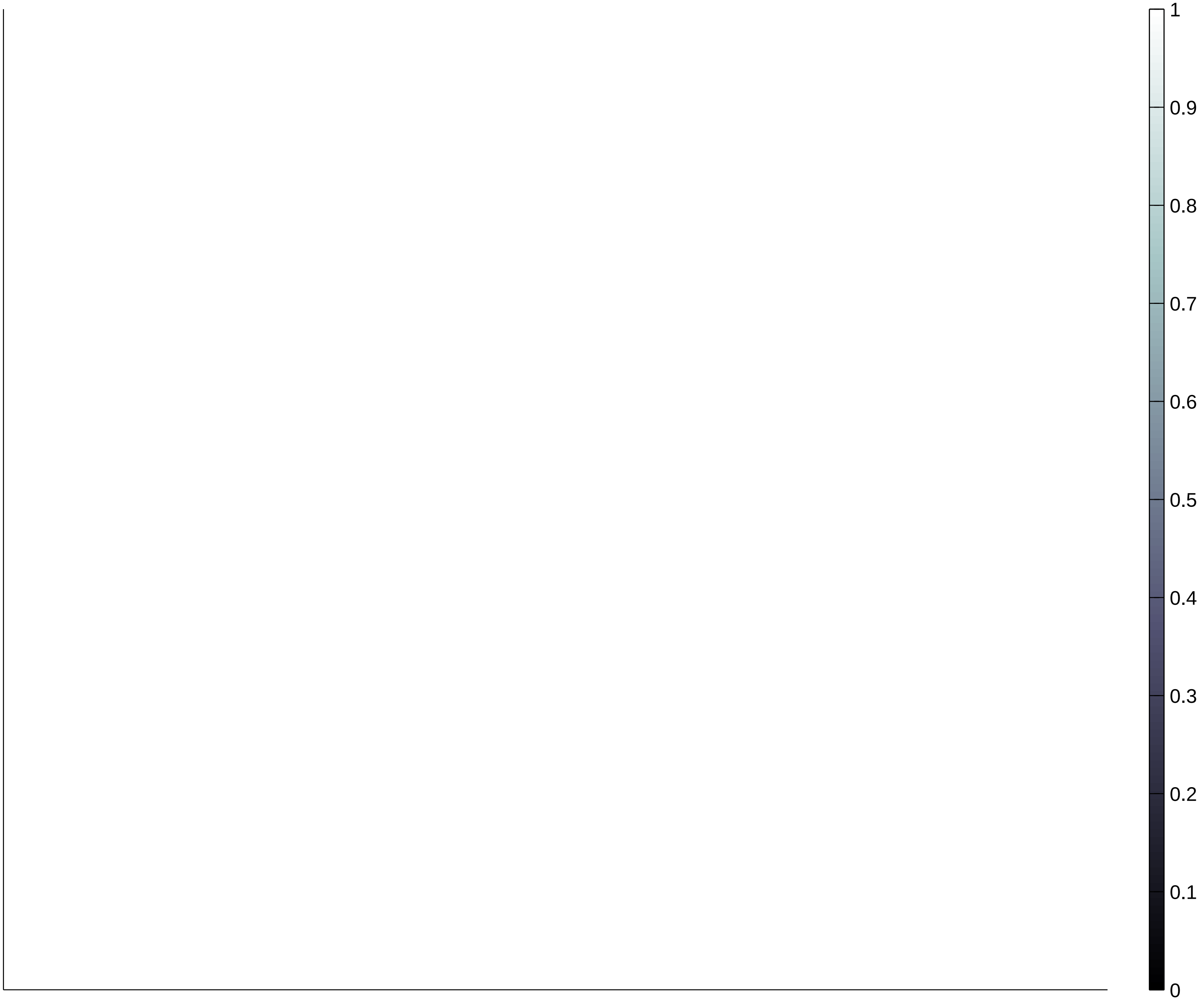

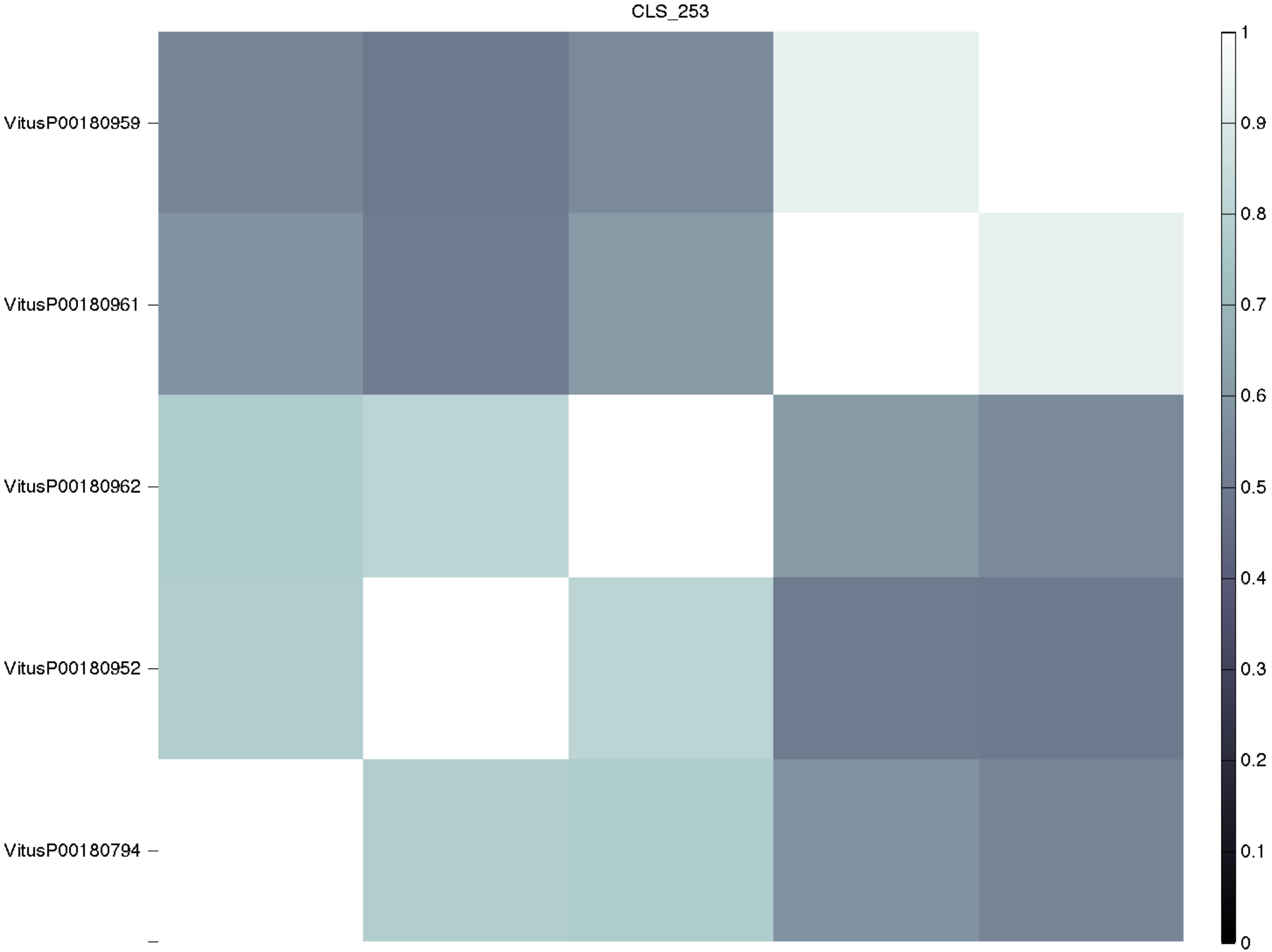
CLS_253
VitusP00180959
VitusP00180961
VitusP00180962
VitusP00180952
VitusP00180794
1
0.9
0.8
0.7
0.6
0.5
0.4
0.3
0.2
0.1
0

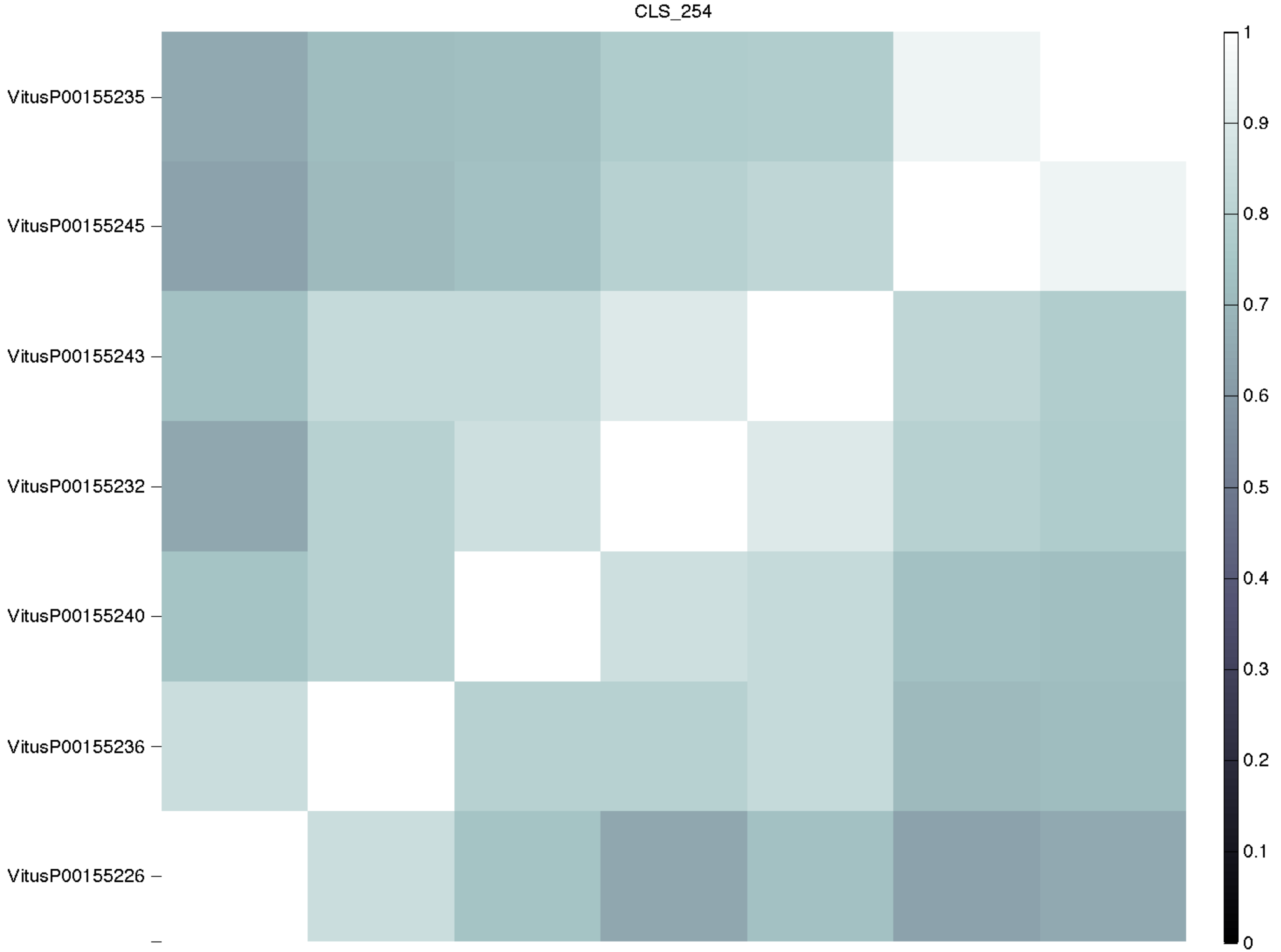

CLS_254
VitusP00155235
VitusP00155245
VitusP00155243
VitusP00155232
VitusP00155240
VitusP00155236
VitusP00155226
1
0.9
0.8
0.7
0.6
0.5
0.4
0.3
0.2
0.1
0

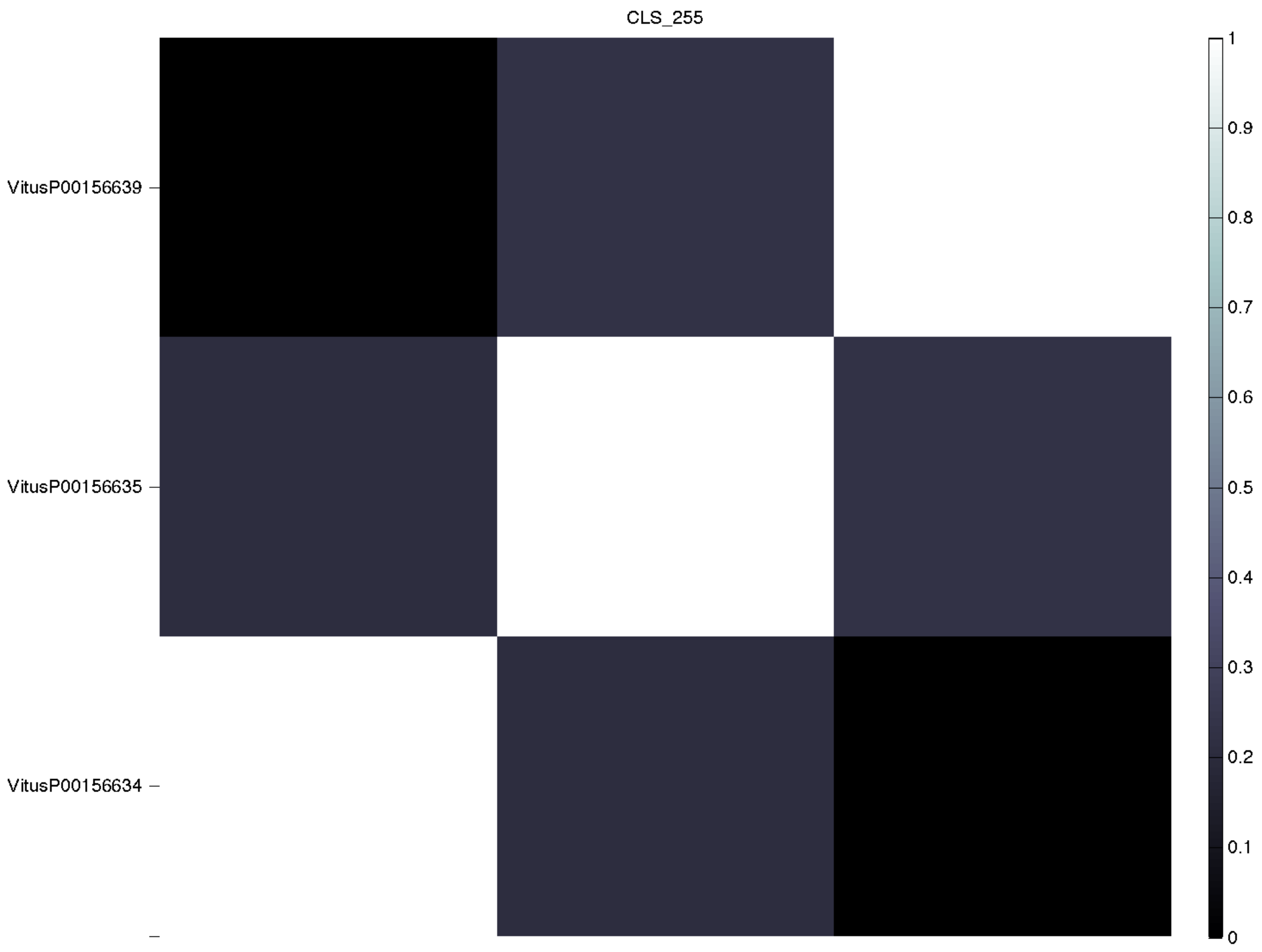

CLS_255
VitusP00156639
VitusP00156635
VitusP00156634
1
0.9
0.8
0.7
0.6
0.5
0.4
0.3
0.2
0.1
0

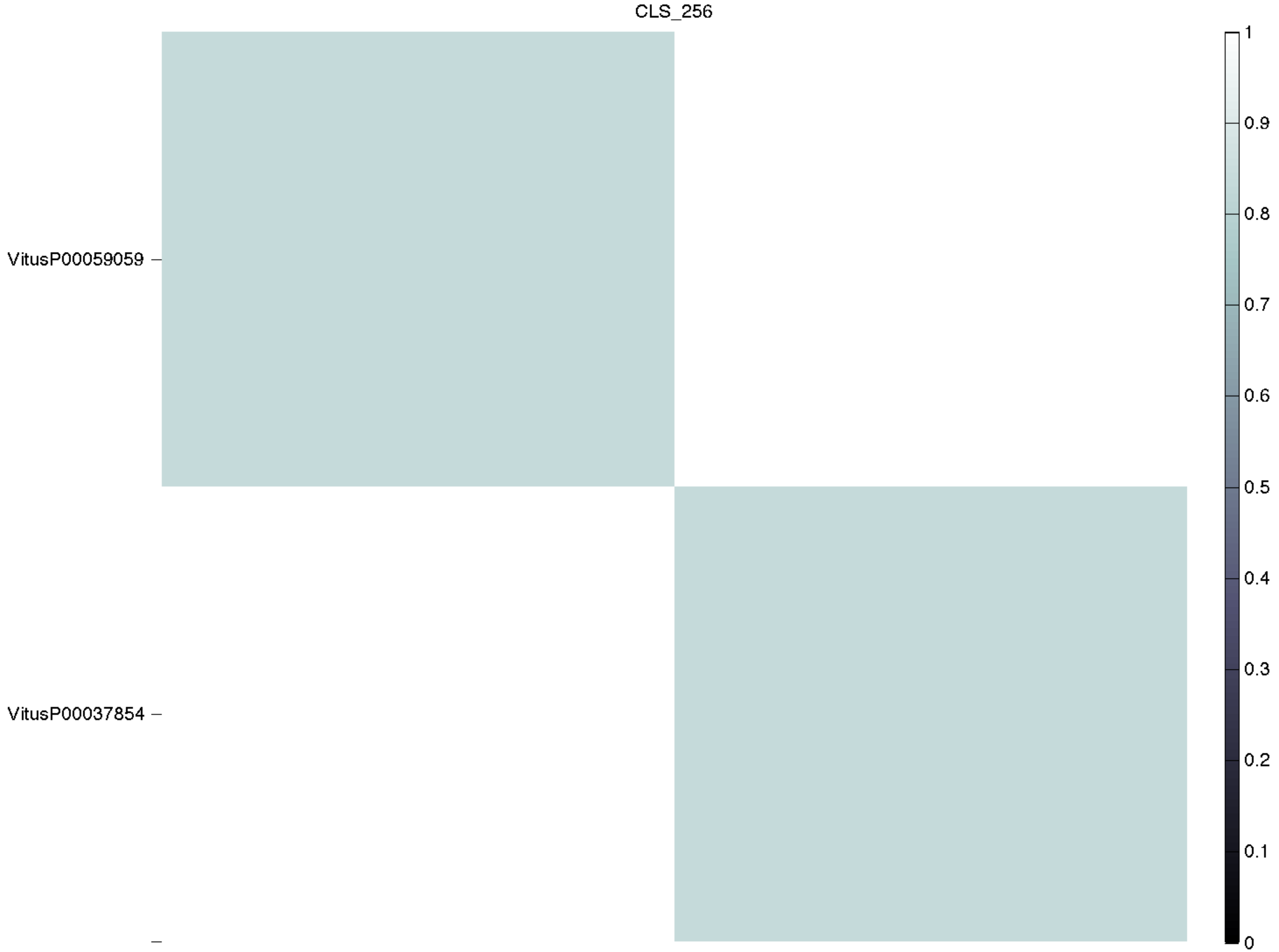
CLS_256
VitusP00059059
VitusP00037854
1
0.9
0.8
0.7
0.6
0.5
0.4
0.3
0.2
0.1
0

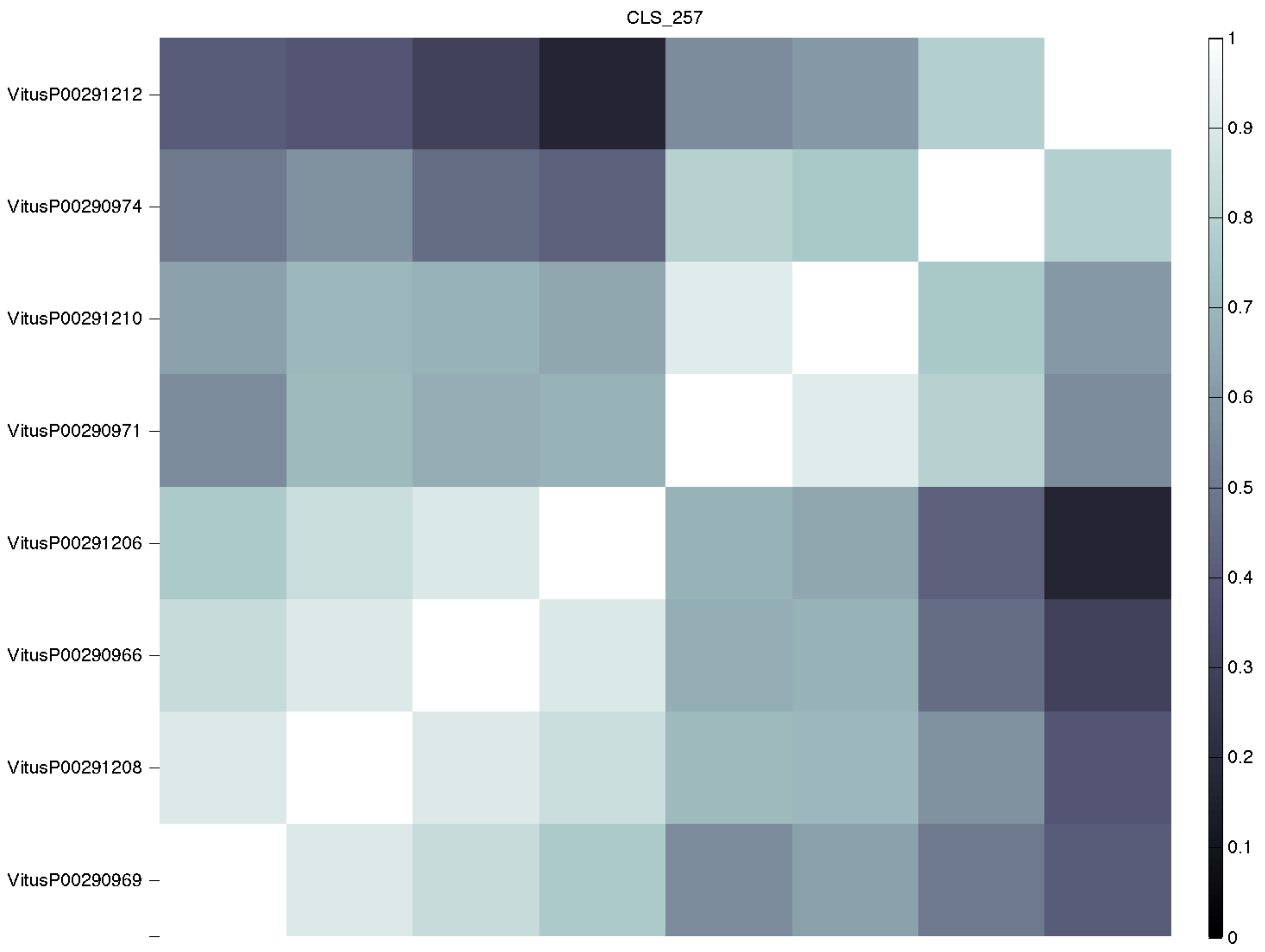
CLS_257
VitusP00291212
VitusP00290974
VitusP00291210
VitusP00290971
VitusP00291206
VitusP00290966
VitusP00291208
VitusP00290969
1
0.9
0.8
0.7
0.6
0.5
0.4
0.3
0.2
0.1
0

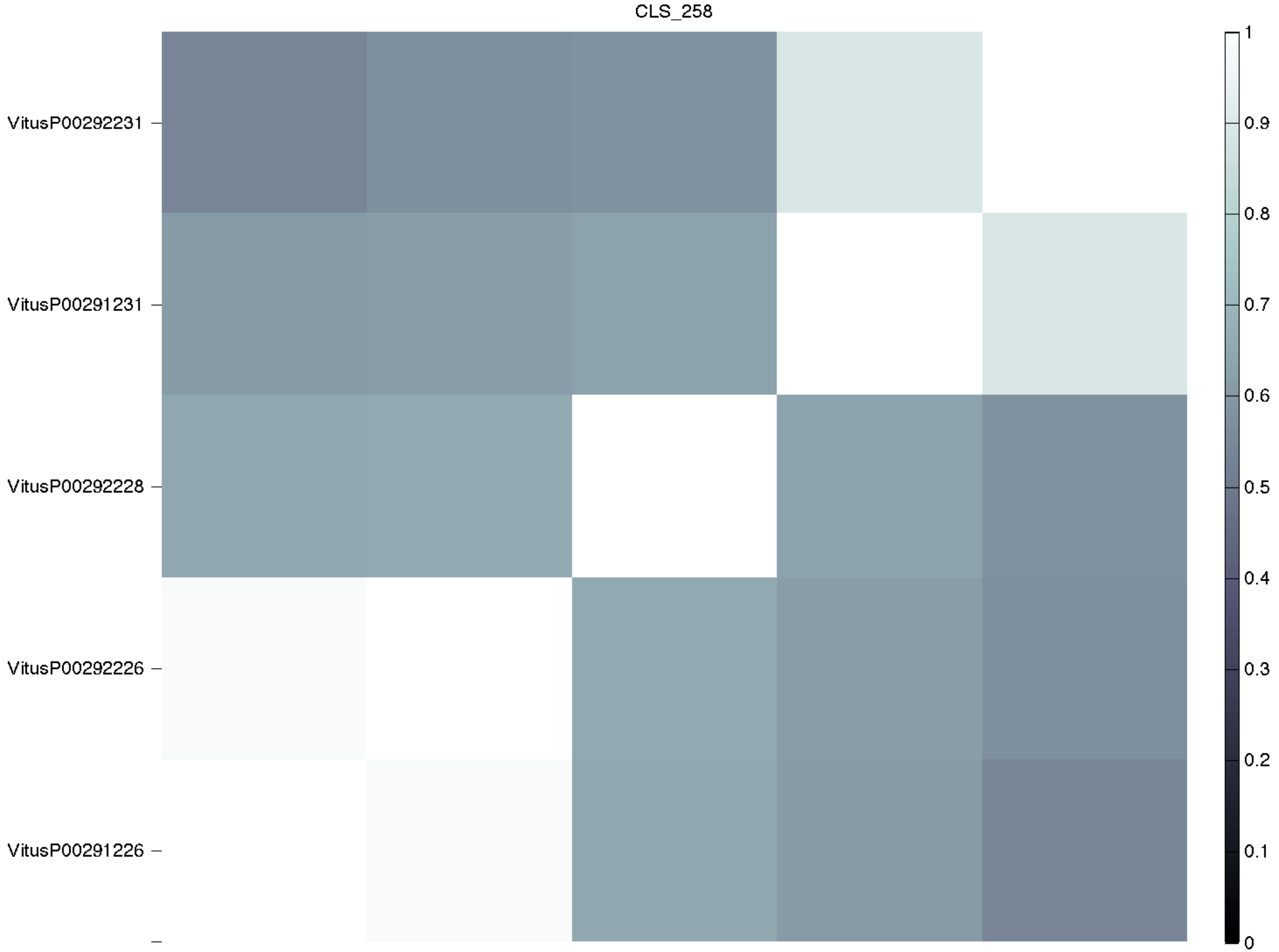
CLS_258
VitusP00292231
VitusP00291231
VitusP00292228
VitusP00292226
VitusP00291226
1
0.9
0.8
0.7
0.6
0.5
0.4
0.3
0.2
0.1
0

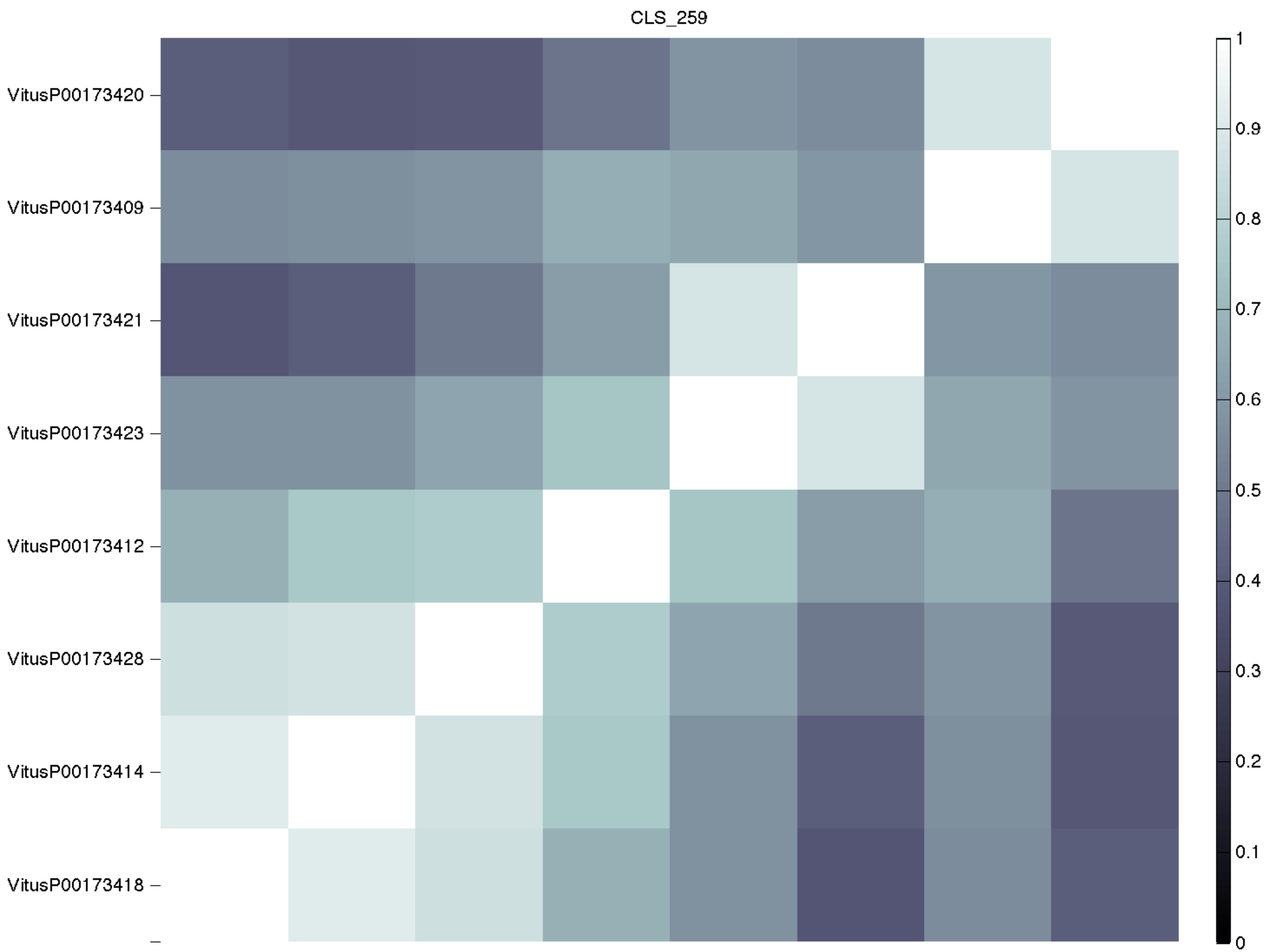
CLS_259
VitusP00173420
VitusP00173409
VitusP00173421
VitusP00173423
VitusP00173412
VitusP00173428
VitusP00173414
VitusP00173418
1
0.9
0.8
0.7
0.6
0.5
0.4
0.3
0.2
0.1
0

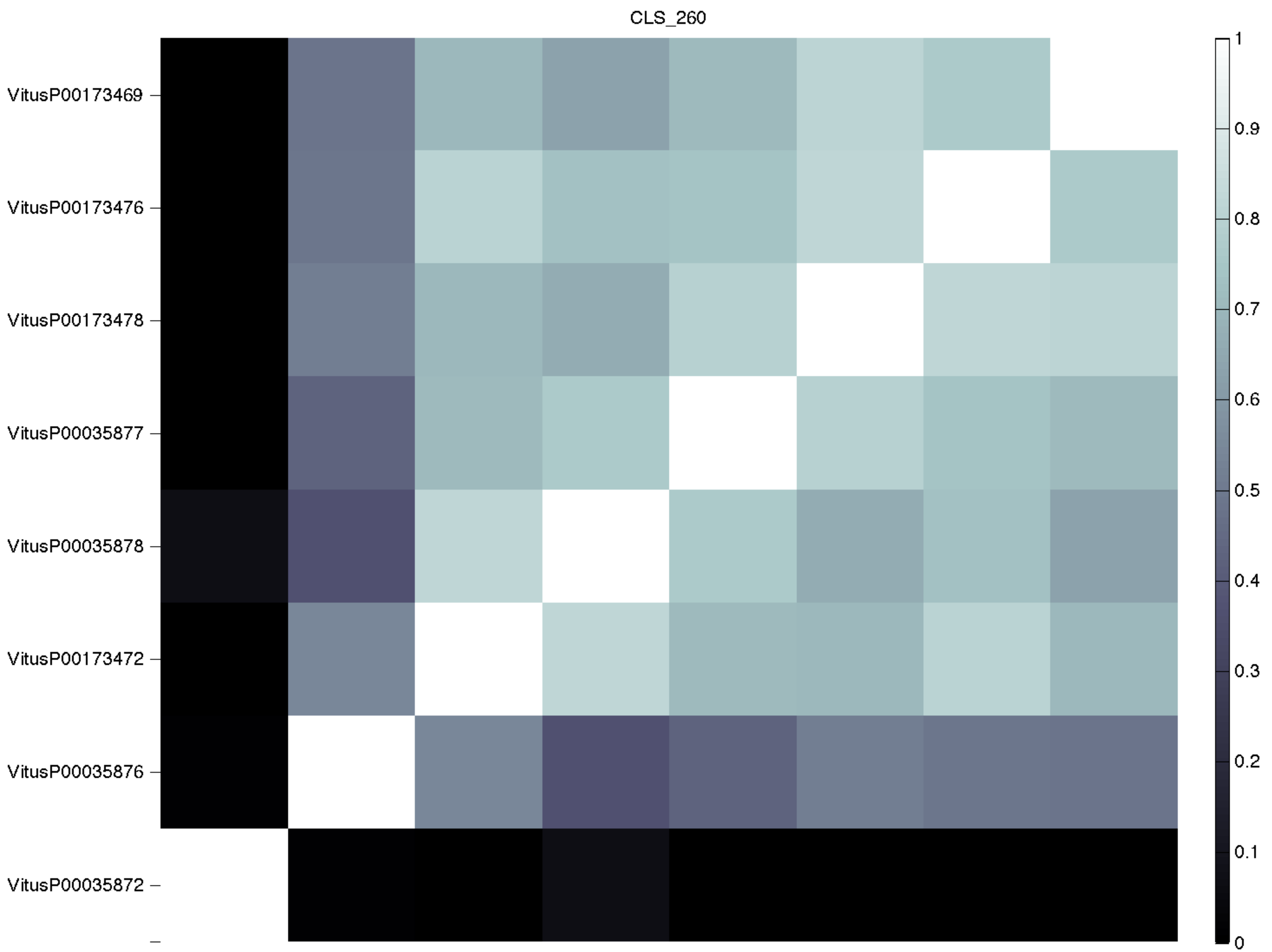
CLS_260
VitusP00173469
VitusP00173476
VitusP00173478
VitusP00035877
VitusP00035878
VitusP00173472
VitusP00035876
VitusP00035872
1
0.9
0.8
0.7
0.6
0.5
0.4
0.3
0.2
0.1
0

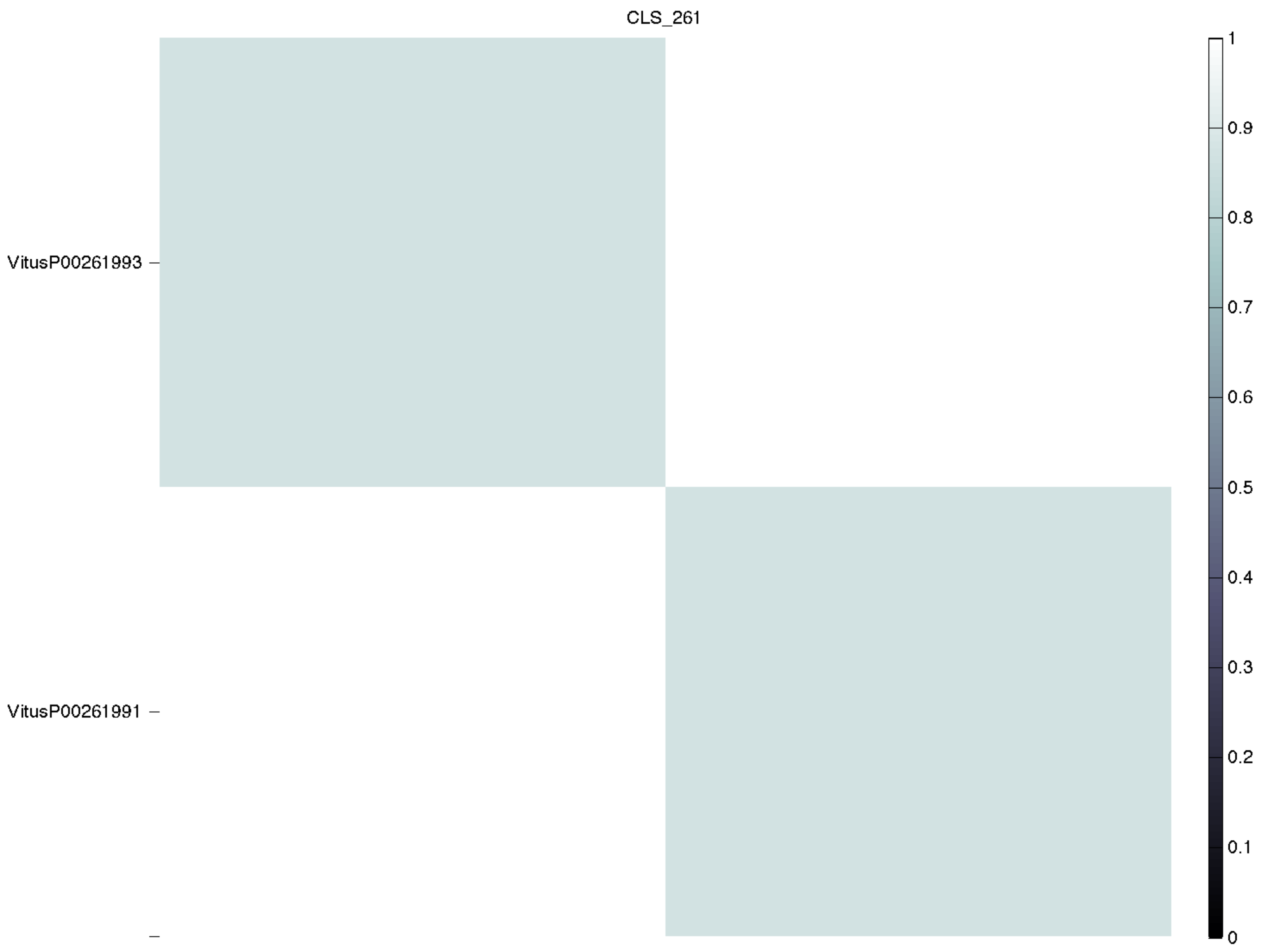
CLS_261
VitusP00261993
VitusP00261991
1
0.9
0.8
0.7
0.6
0.5
0.4
0.3
0.2
0.1
0

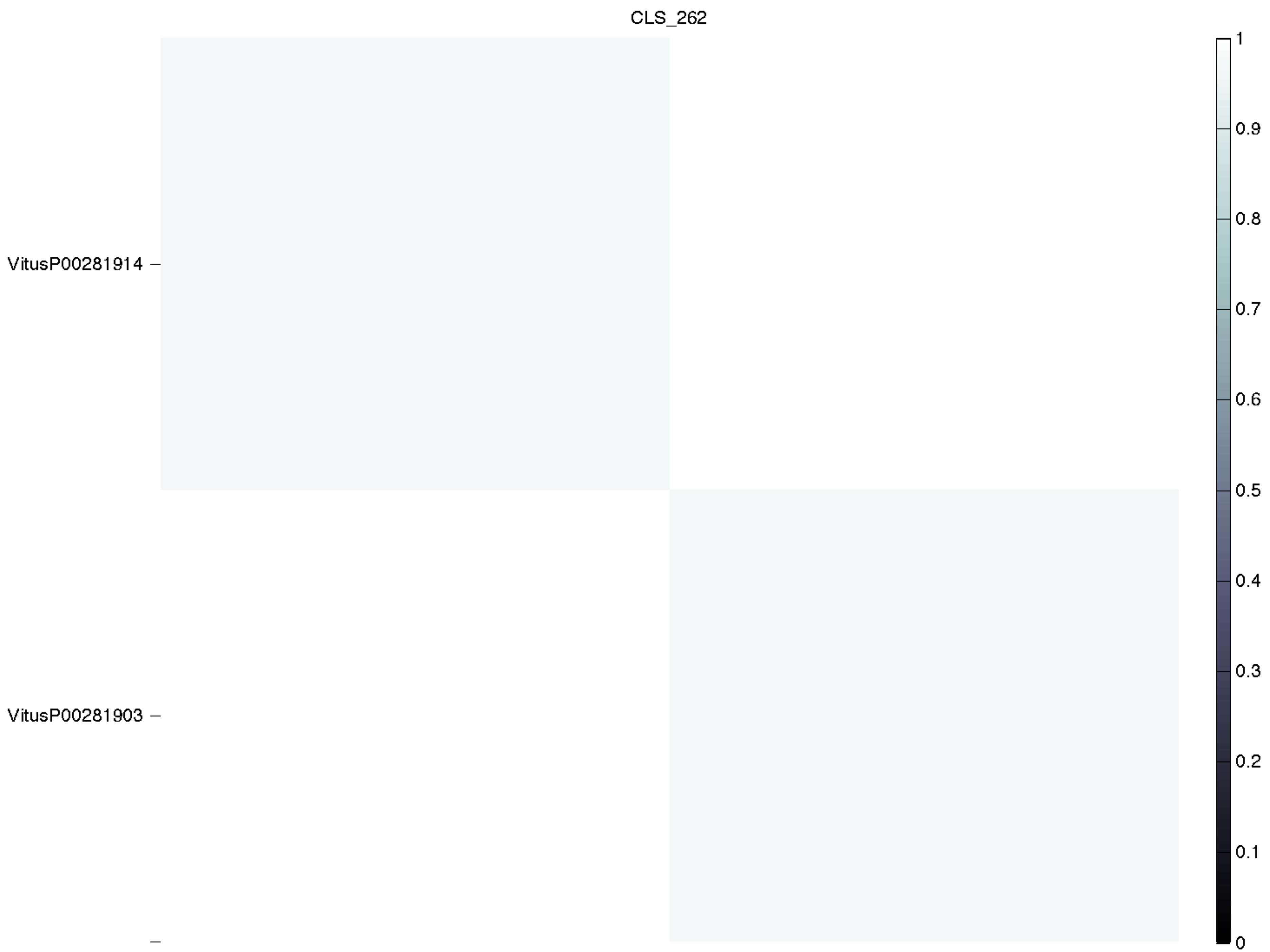
CLS_262
VitusP00281914
VitusP00281903
1
0.9
0.8
0.7
0.6
0.5
0.4
0.3
0.2
0.1
0

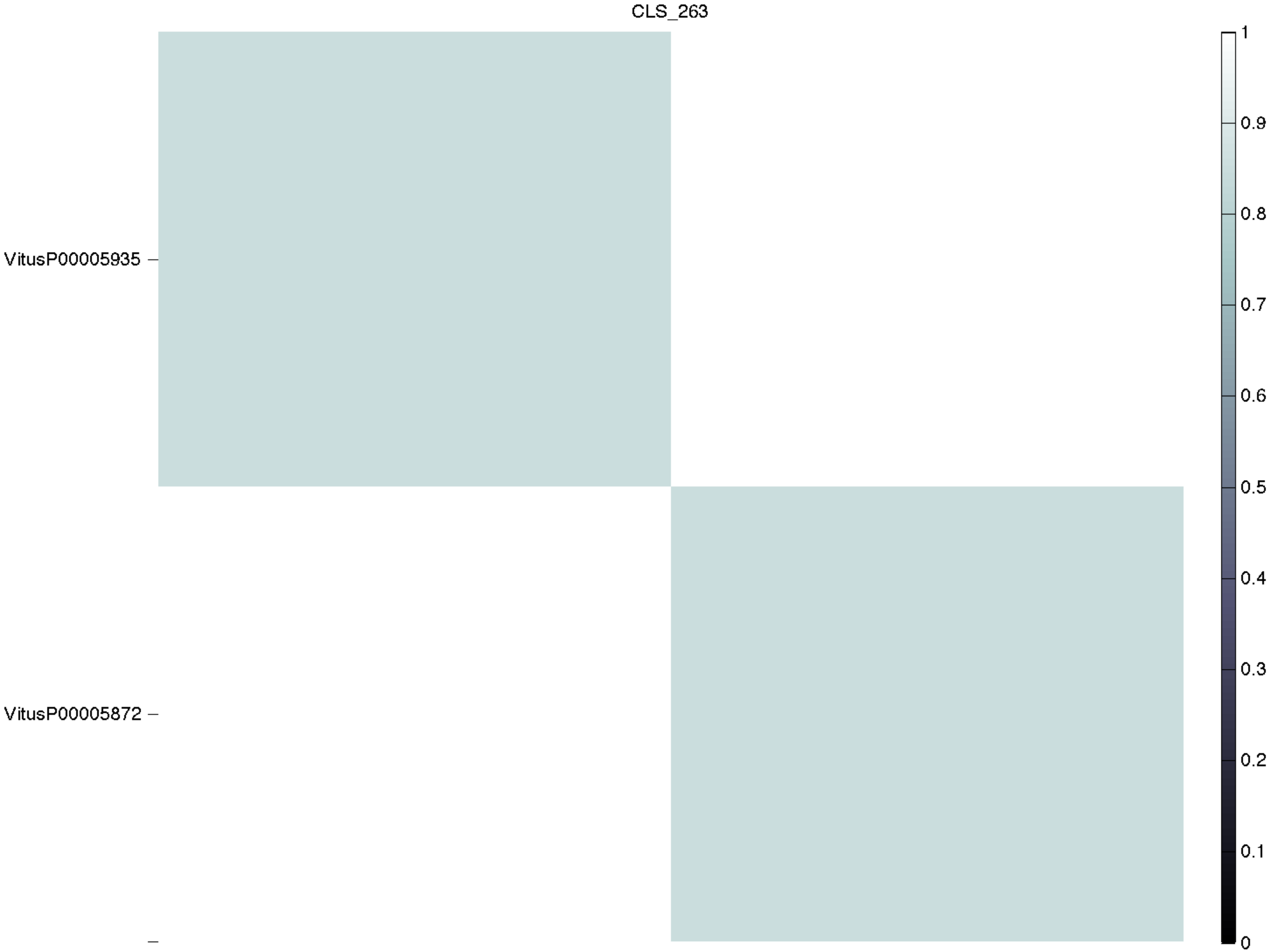
CLS_263
VitusP00005935
VitusP00005872
1
0.9
0.8
0.7
0.6
0.5
0.4
0.3
0.2
0.1
0

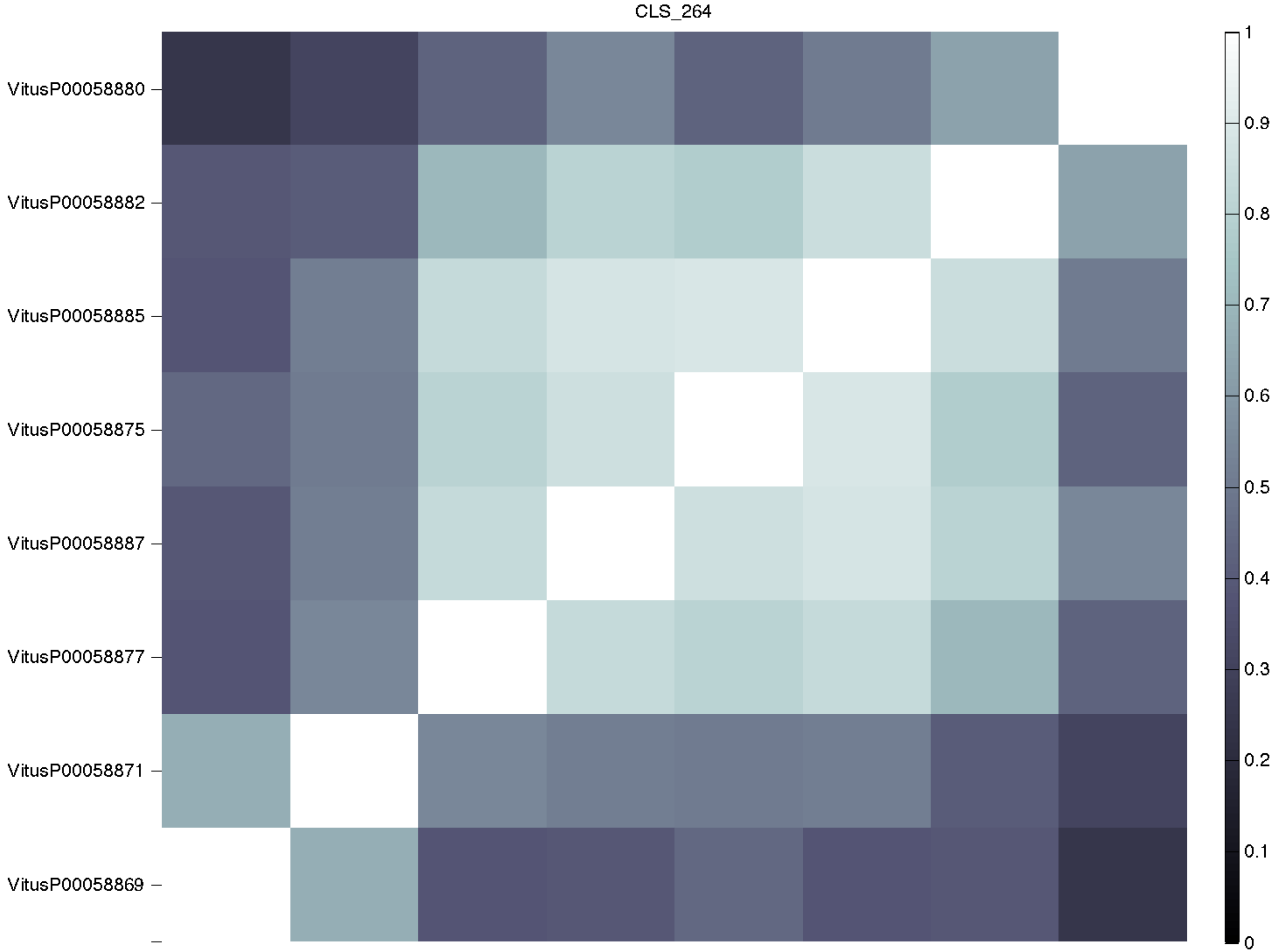
CLS_264
VitusP00058880
VitusP00058882
VitusP00058885
VitusP00058875
VitusP00058887
VitusP00058877
VitusP00058871
VitusP00058869
1
0.9
0.8
0.7
0.6
0.5
0.4
0.3
0.2
0.1
0

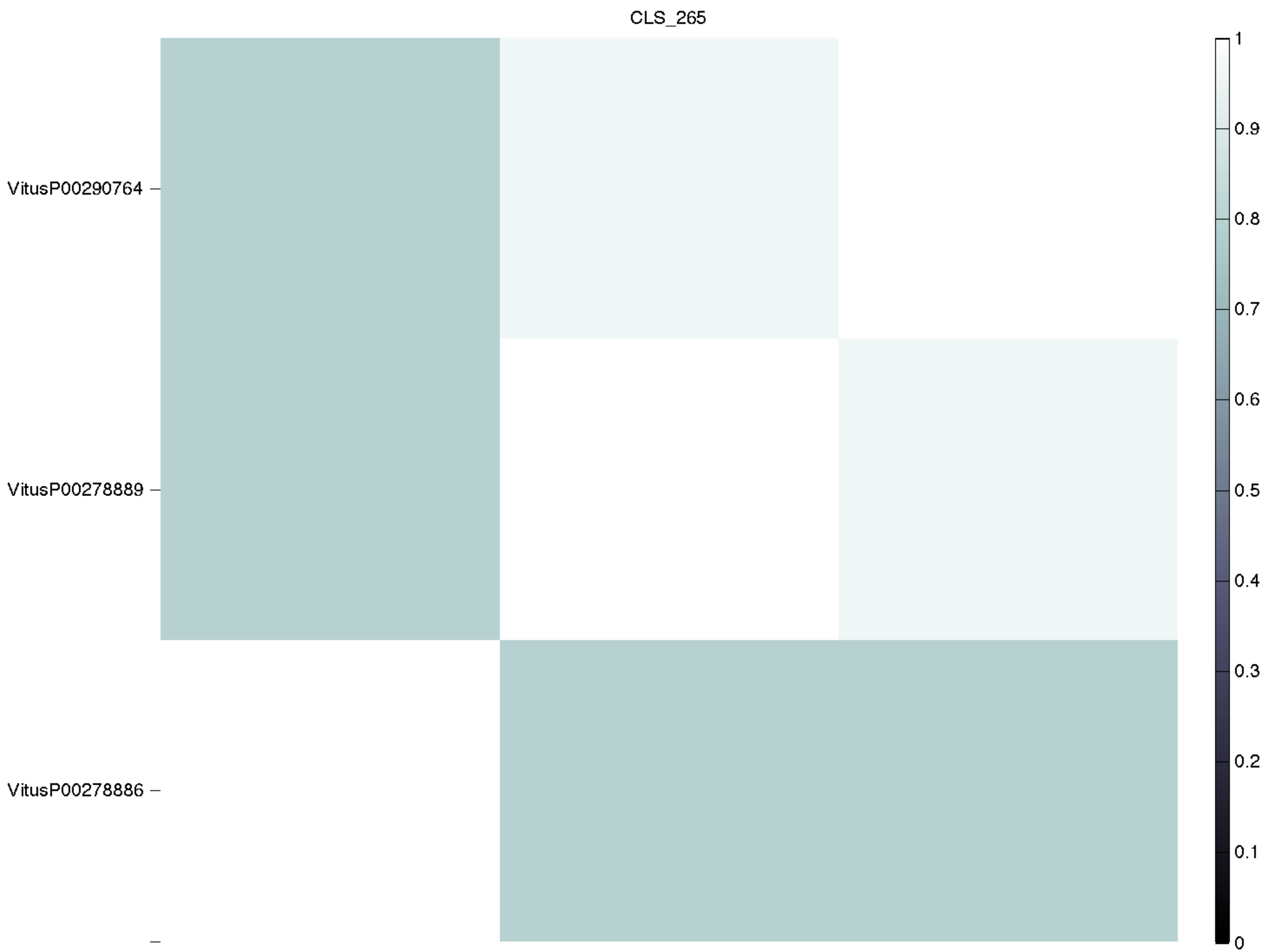

CLS_265
VitusP00290764
VitusP00278889
VitusP00278886
1
0.9
0.8
0.7
0.6
0.5
0.4
0.3
0.2
0.1
0

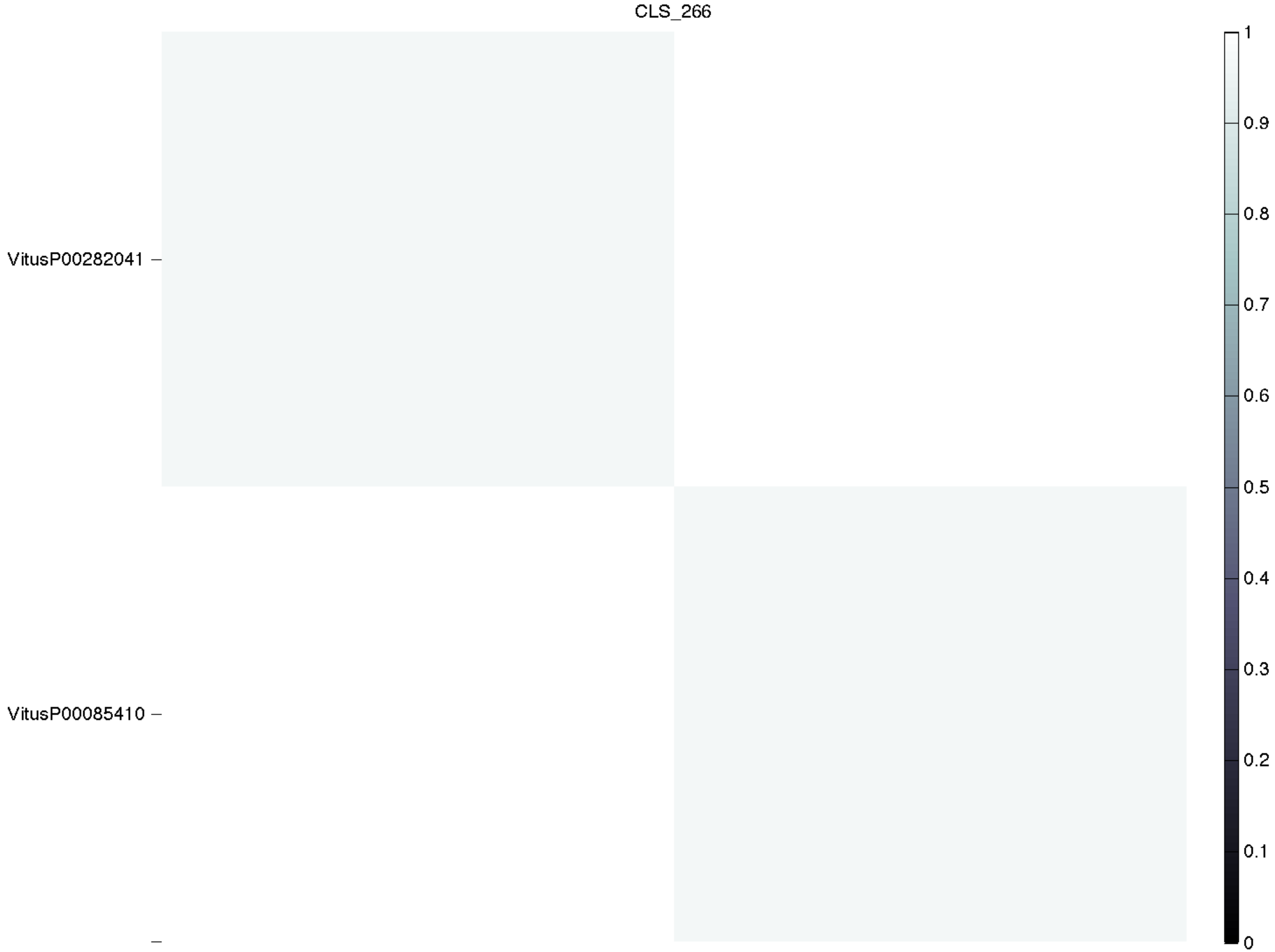
CLS_266
VitusP00282041
VitusP00085410
1
0.9
0.8
0.7
0.6
0.5
0.4
0.3
0.2
0.1
0

CLS_267

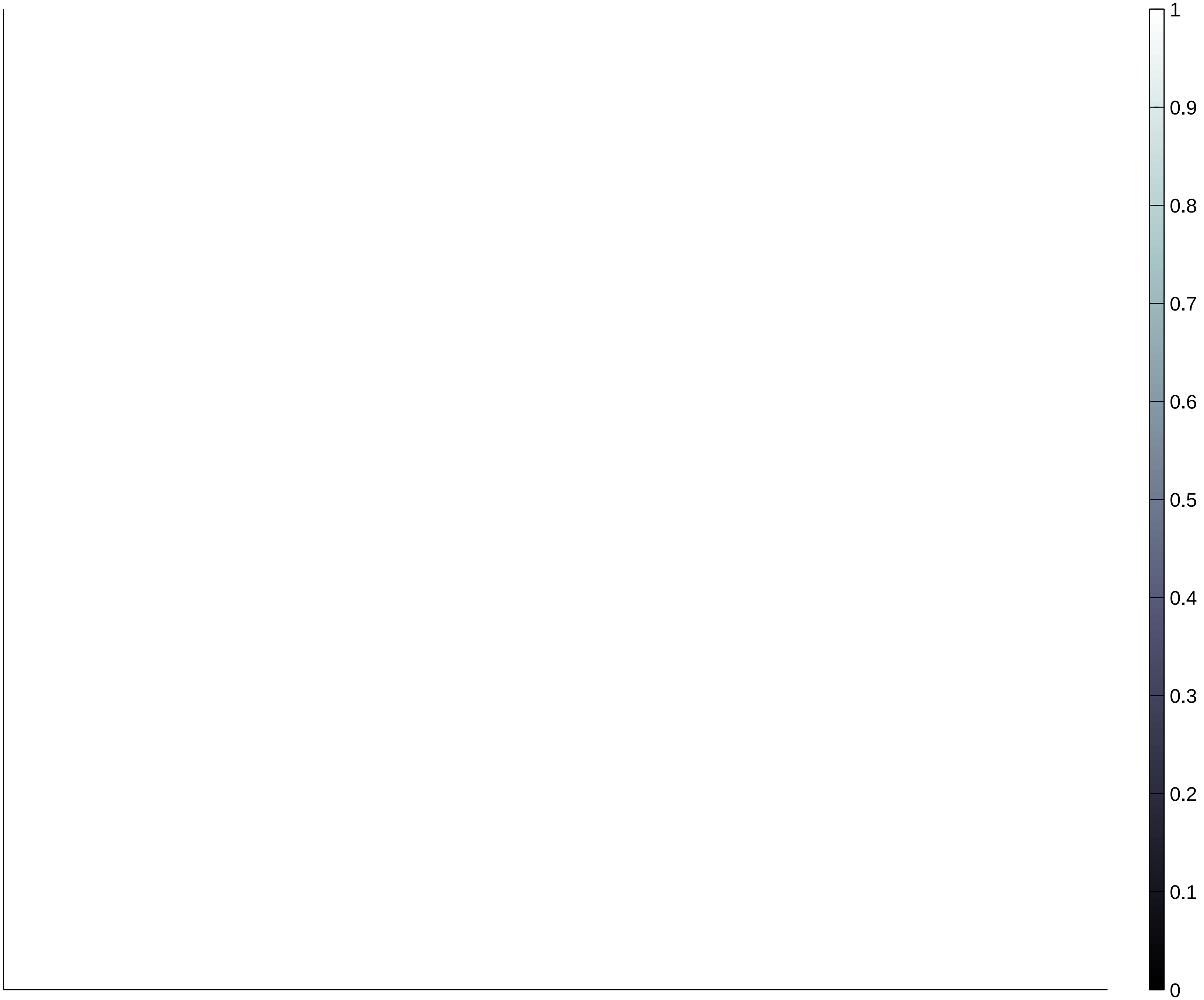

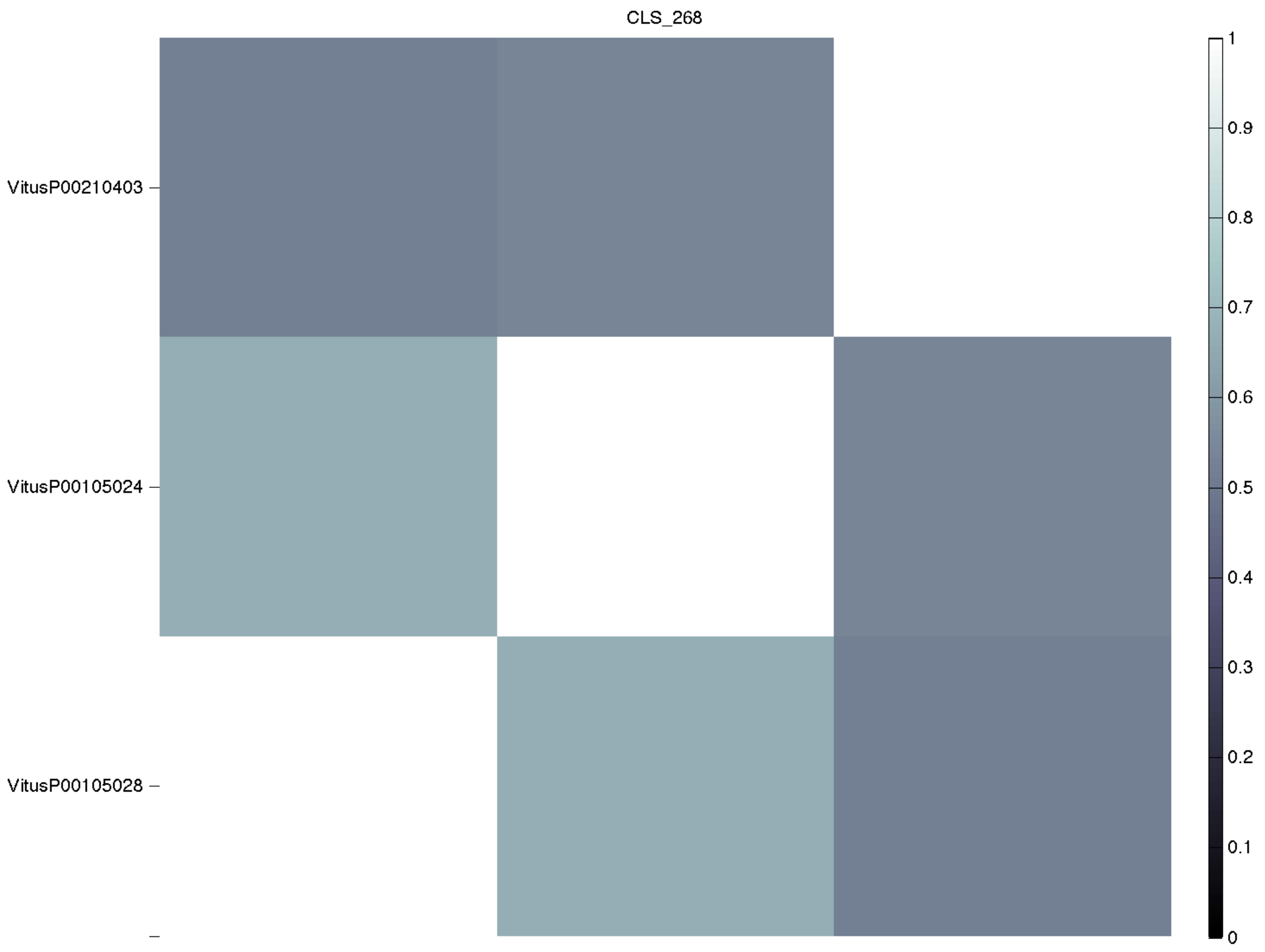
CLS_268
VitusP00210403
VitusP00105024
VitusP00105028
1
0.9
0.8
0.7
0.6
0.5
0.4
0.3
0.2
0.1
0

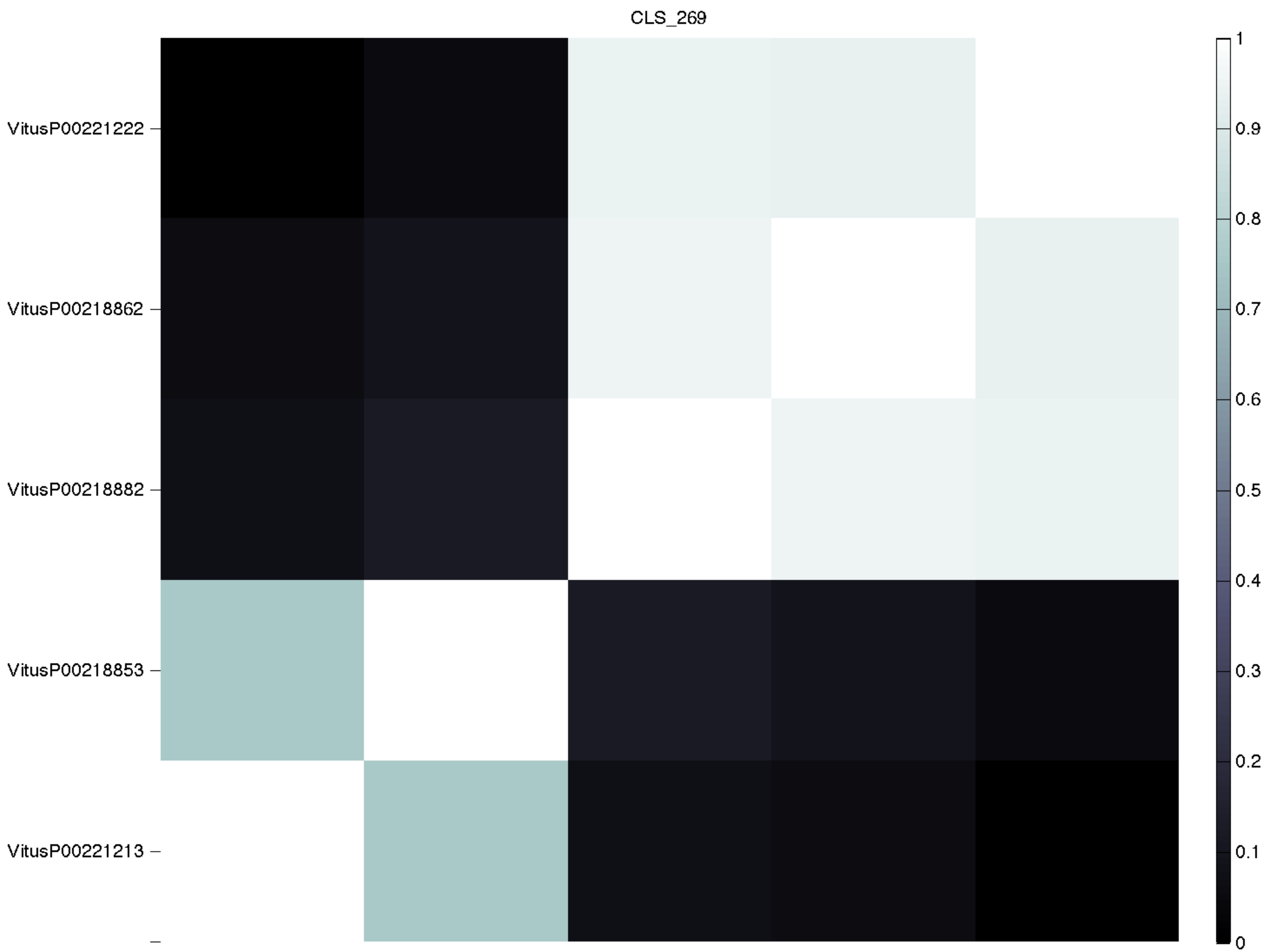
CLS_269
VitusP00221222
VitusP00218862
VitusP00218882
VitusP00218853
VitusP00221213
1
0.9
0.8
0.7
0.6
0.5
0.4
0.3
0.2
0.1
0

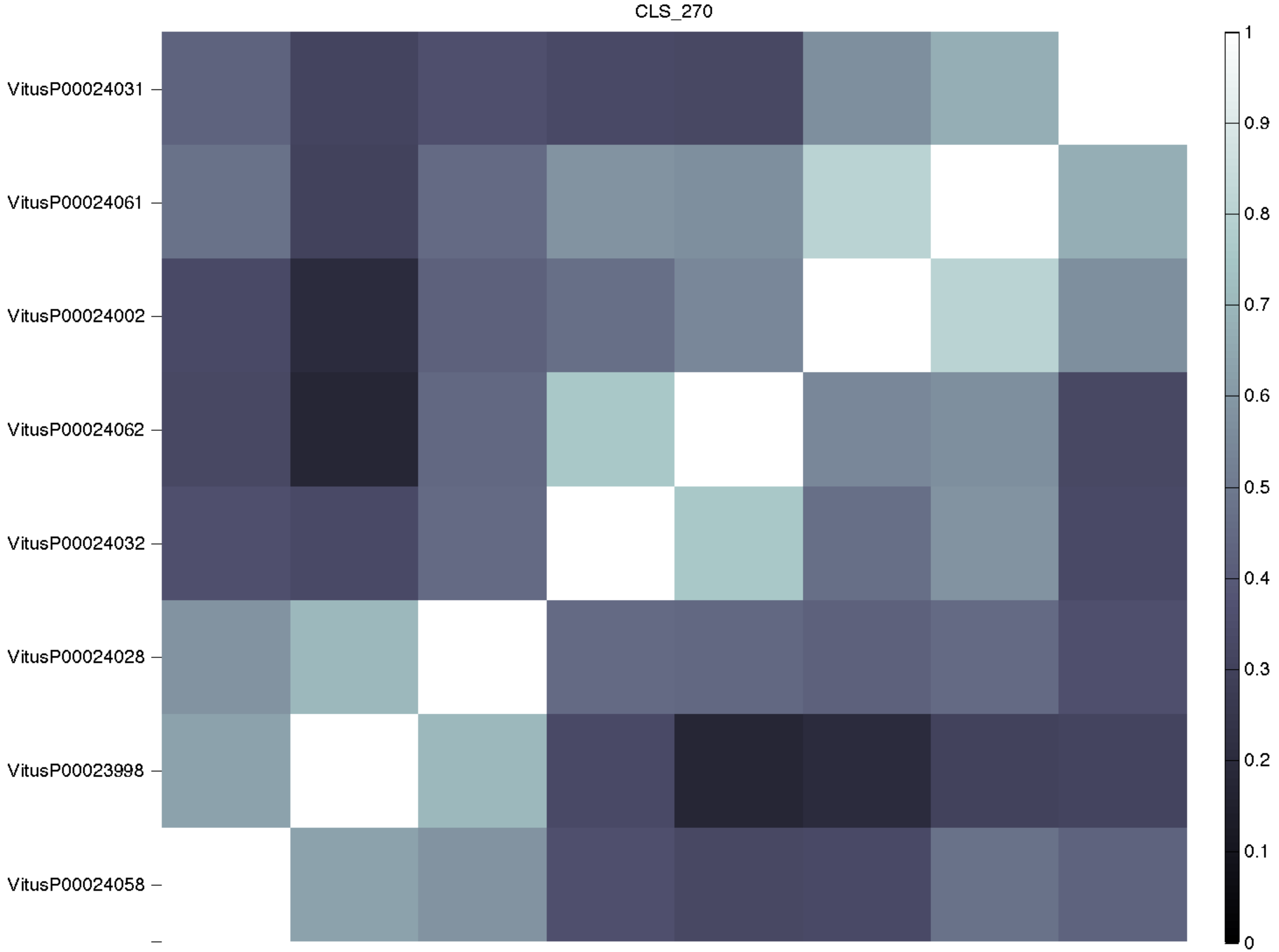
CLS_270
VitusP00024031
VitusP00024061
VitusP00024002
VitusP00024062
VitusP00024032
VitusP00024028
VitusP00023998
VitusP00024058
1
0.9
0.8
0.7
0.6
0.5
0.4
0.3
0.2
0.1
0

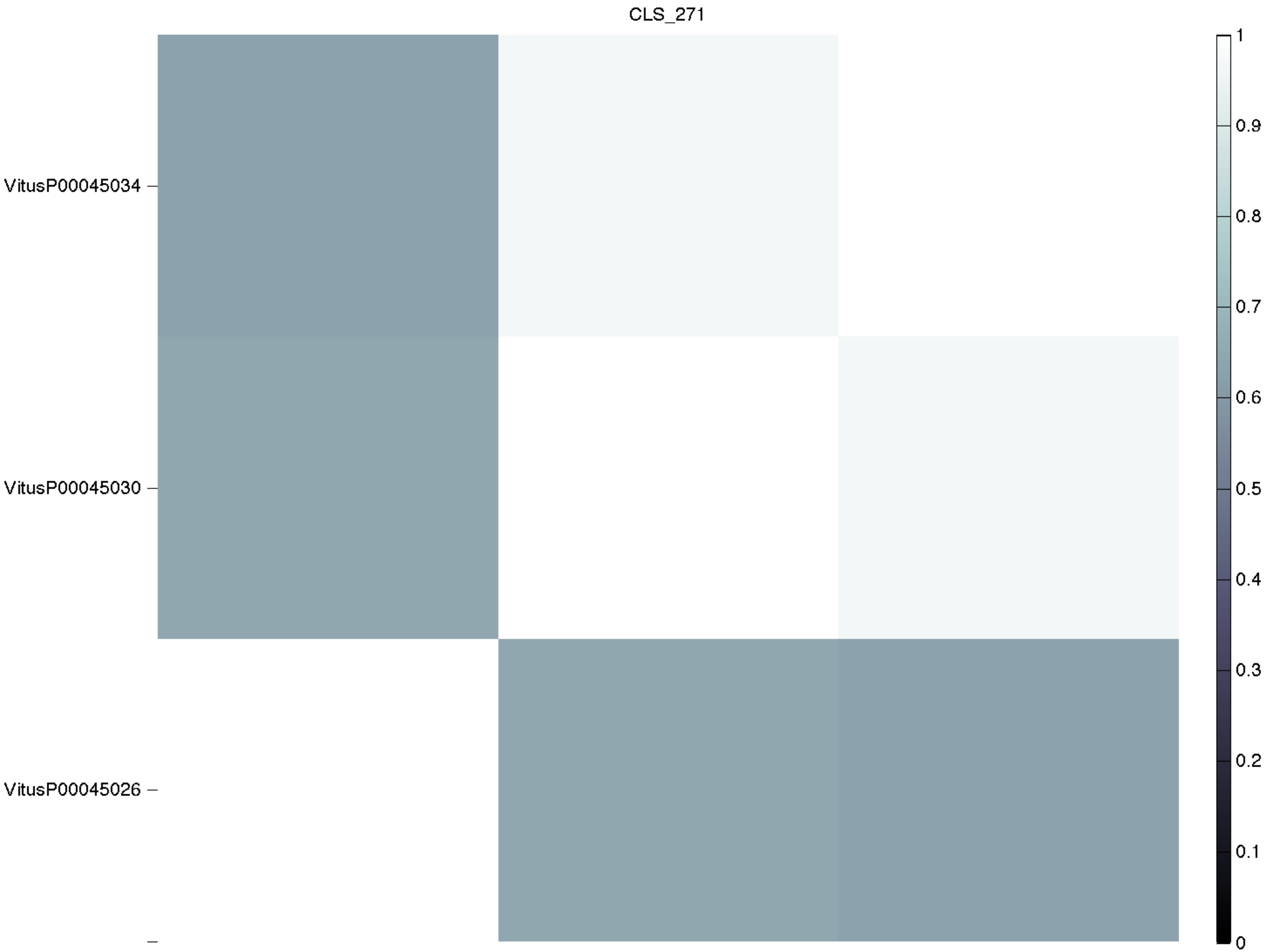

CLS_271
VitusP00045034
VitusP00045030
VitusP00045026
1
0.9
0.8
0.7
0.6
0.5
0.4
0.3
0.2
0.1
0

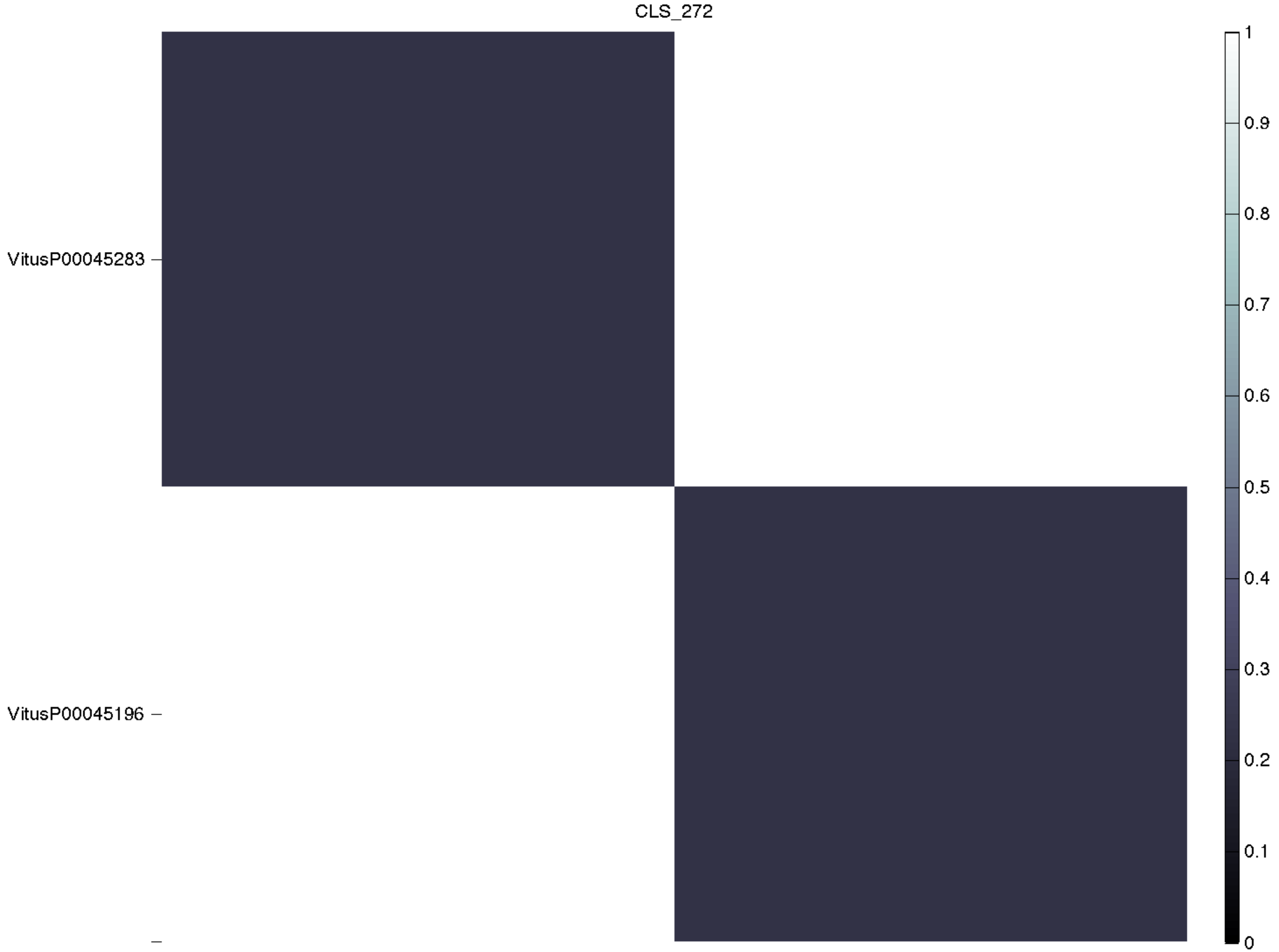
CLS_272
VitusP00045283
VitusP00045196
1
0.9
0.8
0.7
0.6
0.5
0.4
0.3
0.2
0.1
0

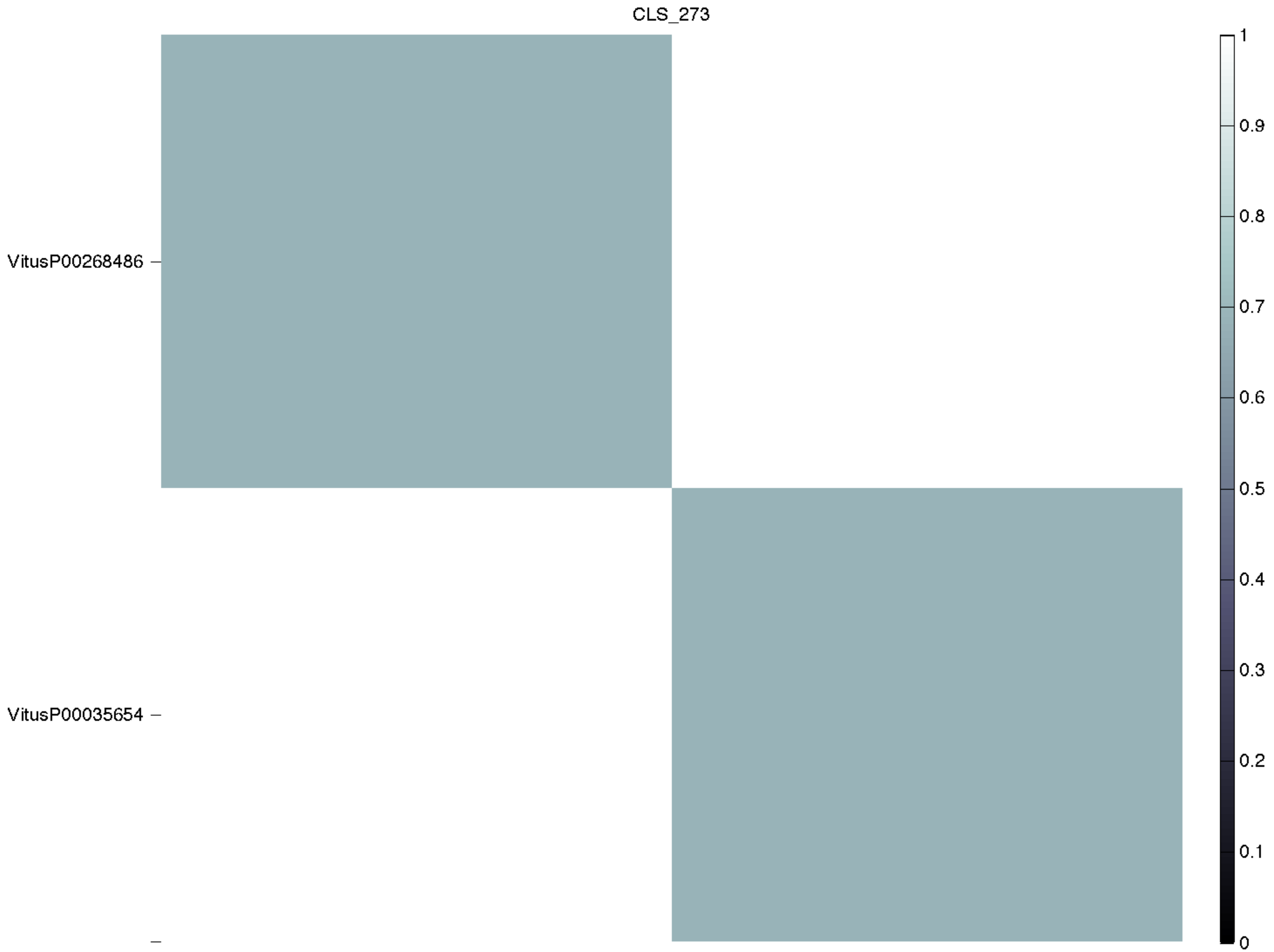

CLS_273
VitusP00268486
VitusP00035654
1
0.9
0.8
0.7
0.6
0.5
0.4
0.3
0.2
0.1
0

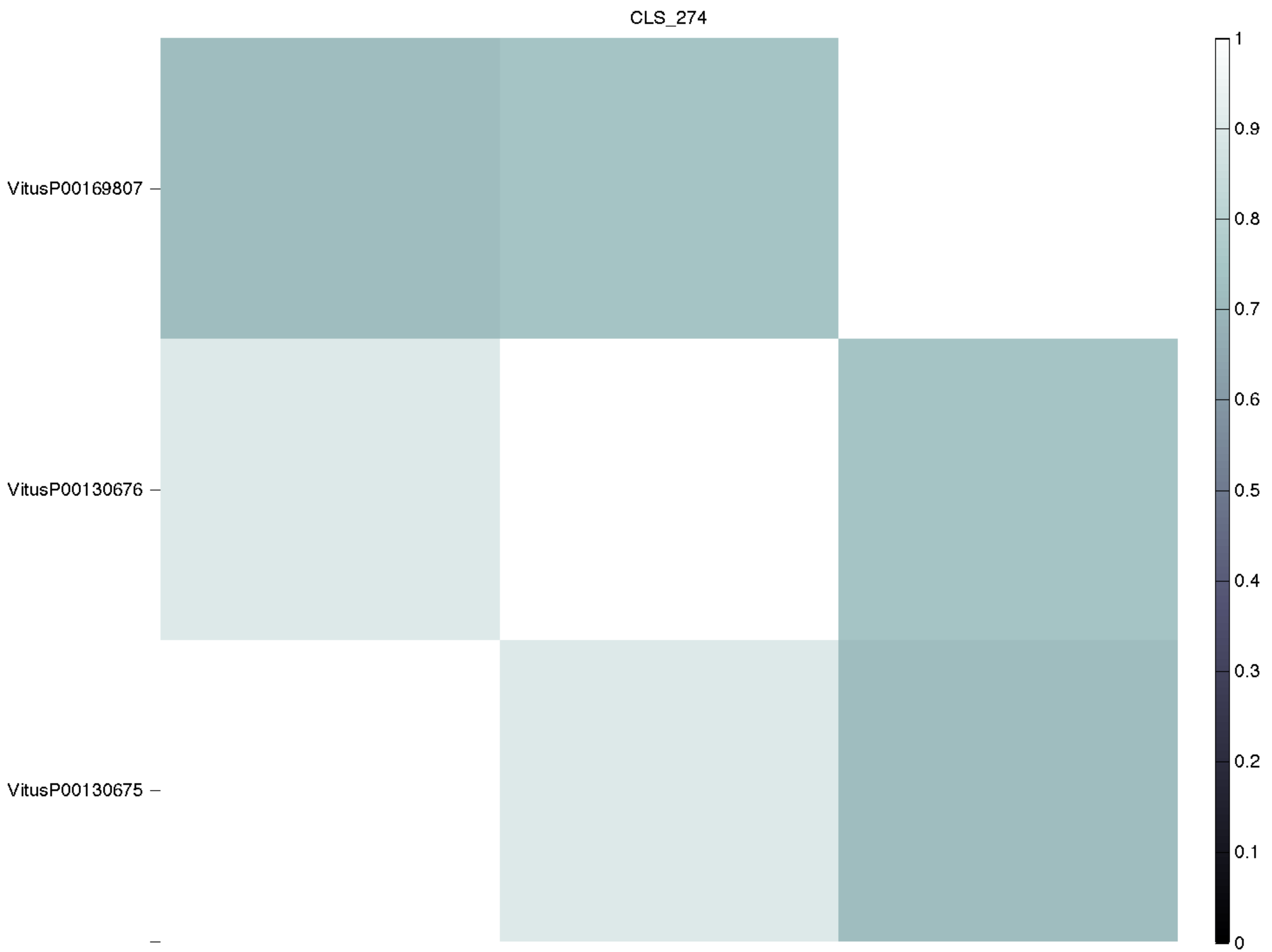

CLS_274
VitusP00169807
VitusP00130676
VitusP00130675
1
0.9
0.8
0.7
0.6
0.5
0.4
0.3
0.2
0.1
0

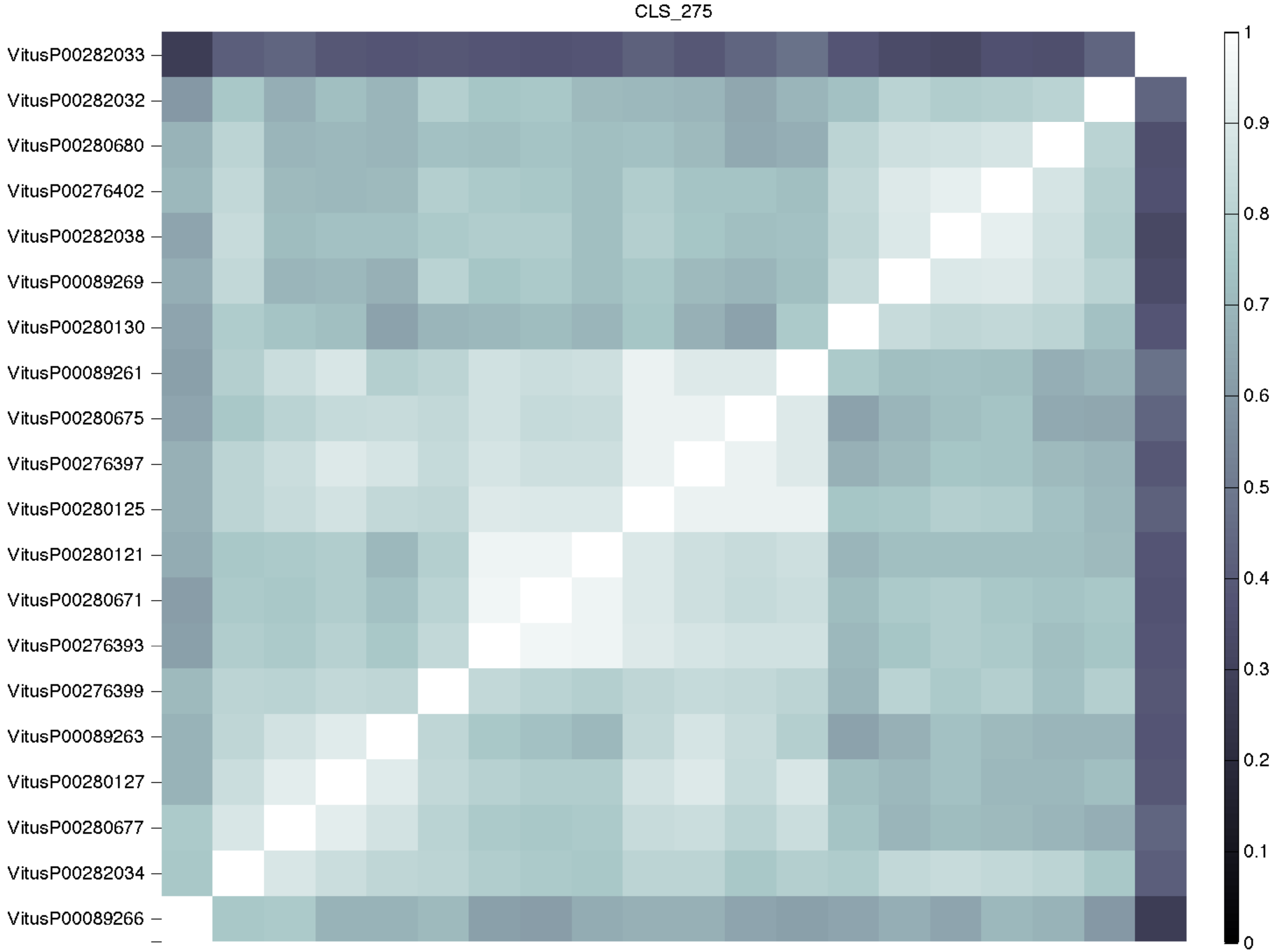

CLS_275
VitusP00282033
VitusP00282032
VitusP00280680
VitusP00276402
VitusP00282038
VitusP00089269
VitusP00280130
VitusP00089261
VitusP00280675
VitusP00276397
VitusP00280125
VitusP00280121
VitusP00280671
VitusP00276393
VitusP00276399
VitusP00089263
VitusP00280127
VitusP00280677
VitusP00282034
VitusP00089266
1
0.9
0.8
0.7
0.6
0.5
0.4
0.3
0.2
0.1
0

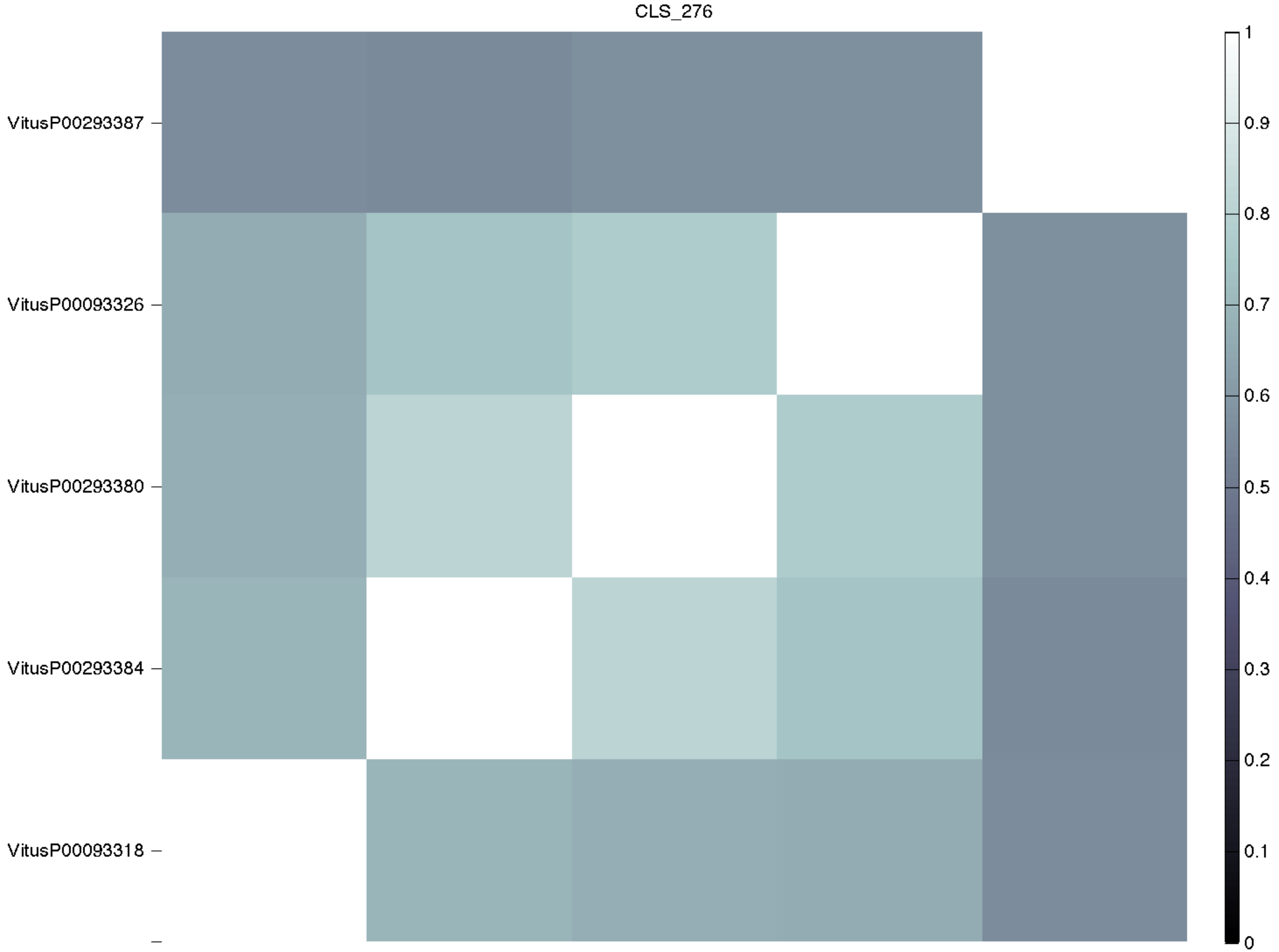
CLS_276
VitusP00293387
VitusP00093326
VitusP00293380
VitusP00293384
VitusP00093318
1
0.9
0.8
0.7
0.6
0.5
0.4
0.3
0.2
0.1
0

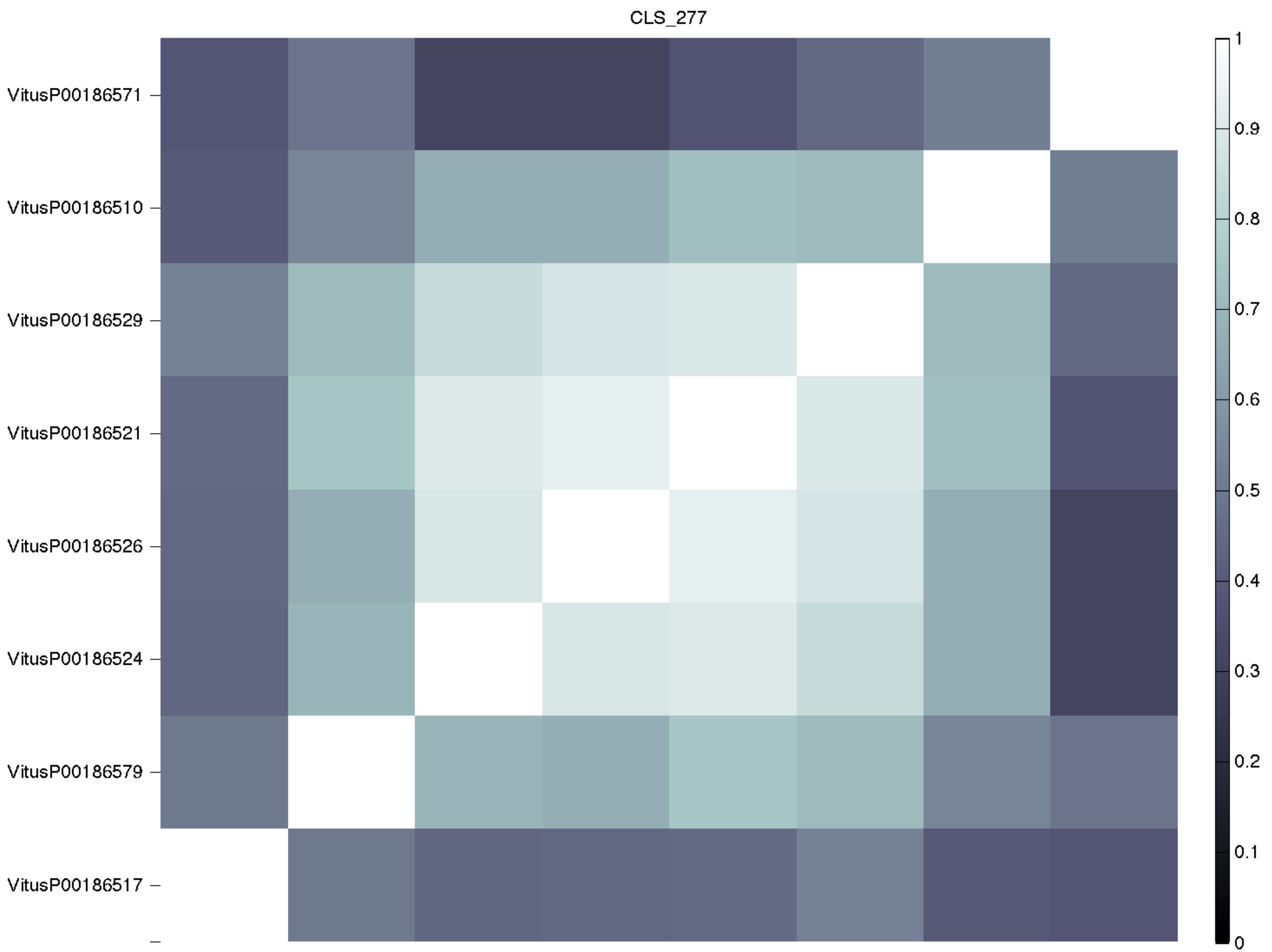
CLS_277
VitusP00186571
VitusP00186510
VitusP00186529
VitusP00186521
VitusP00186526
VitusP00186524
VitusP00186579
VitusP00186517
1
0.9
0.8
0.7
0.6
0.5
0.4
0.3
0.2
0.1
0

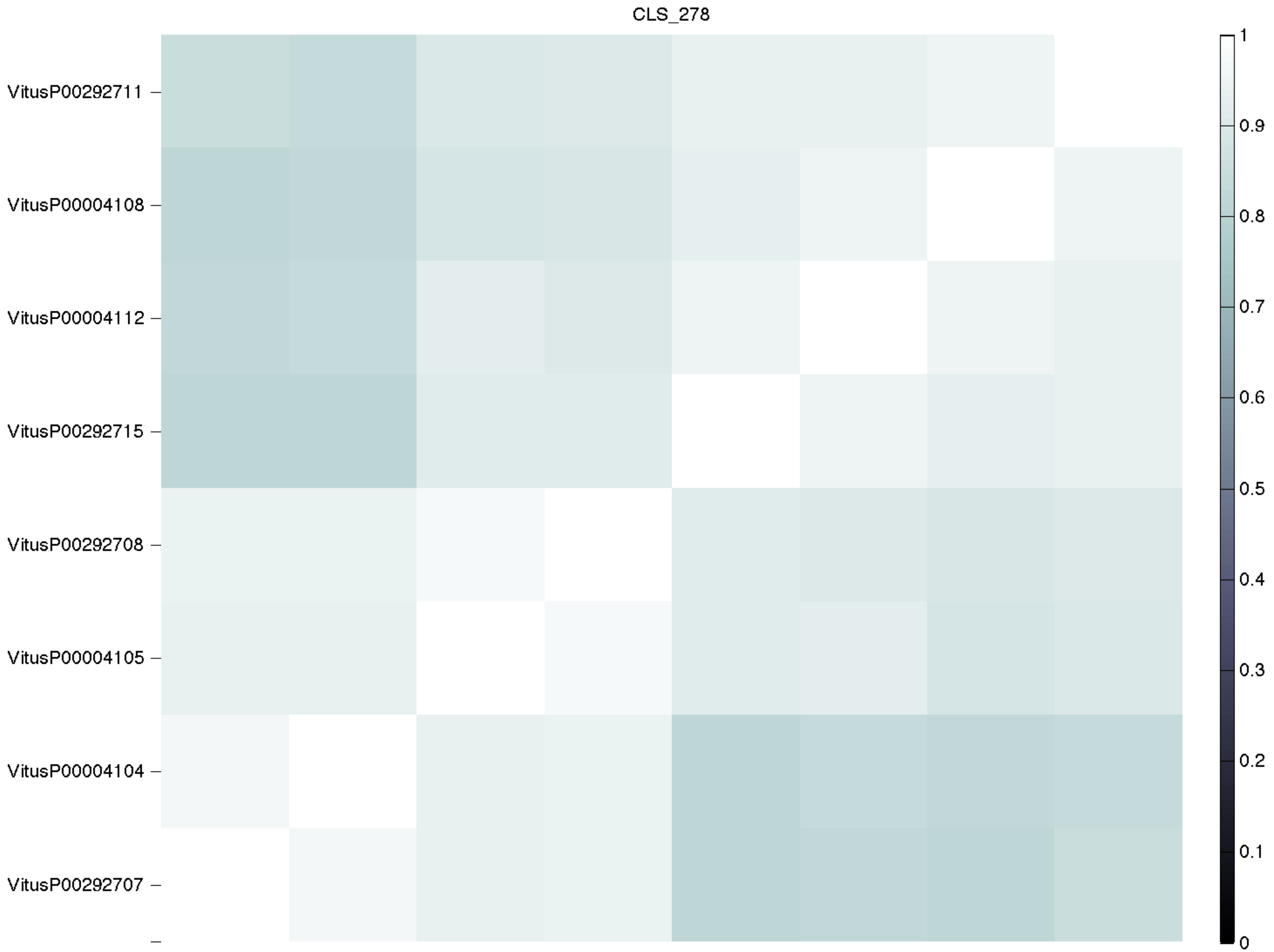
CLS_278
VitusP00292711
VitusP00004108
VitusP00004112
VitusP00292715
VitusP00292708
VitusP00004105
VitusP00004104
VitusP00292707
1
0.9
0.8
0.7
0.6
0.5
0.4
0.3
0.2
0.1
0

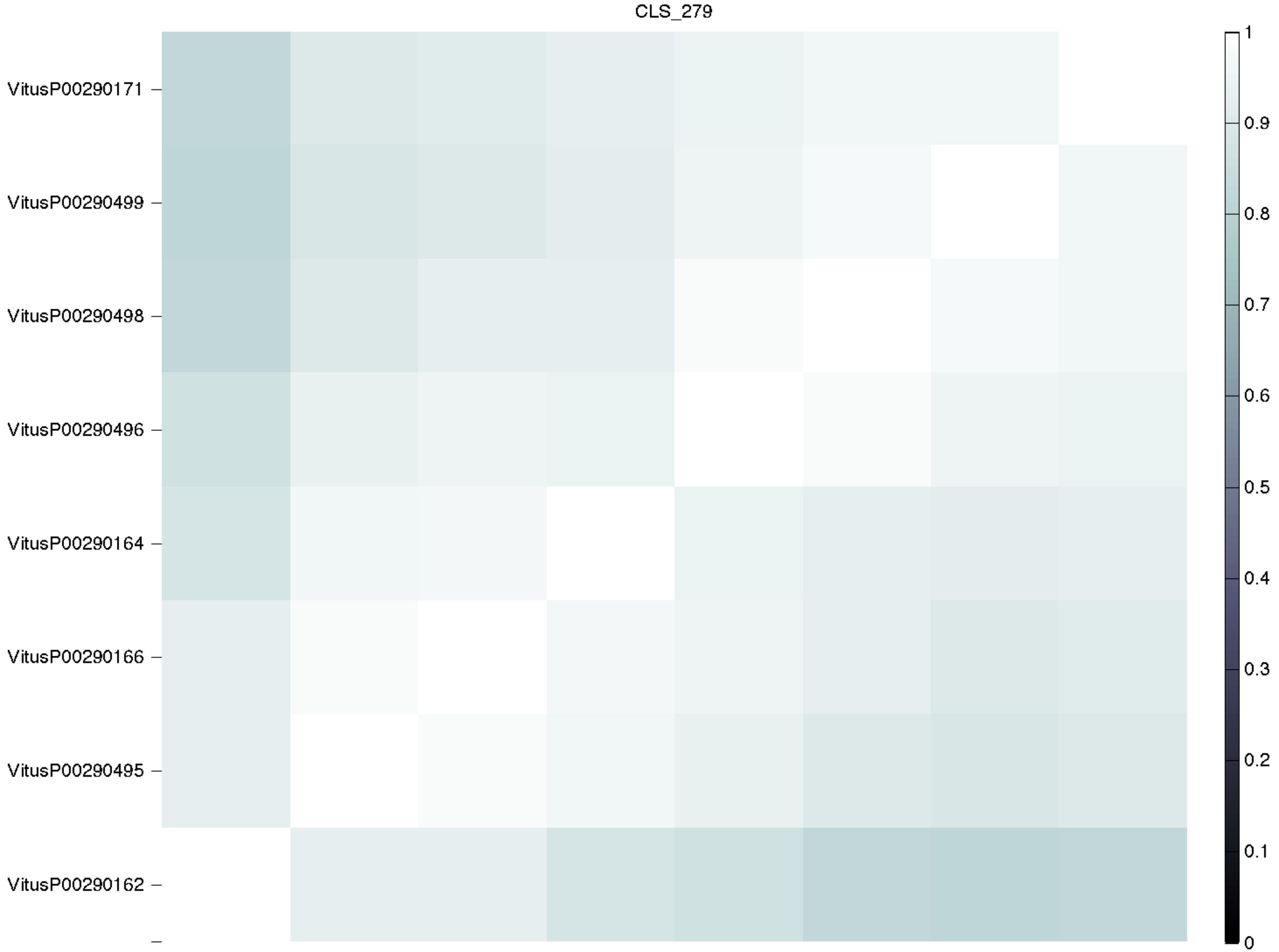

CLS_279
VitusP00290171
VitusP00290499
VitusP00290498
VitusP00290496
VitusP00290164
VitusP00290166
VitusP00290495
VitusP00290162
1
0.9
0.8
0.7
0.6
0.5
0.4
0.3
0.2
0.1
0

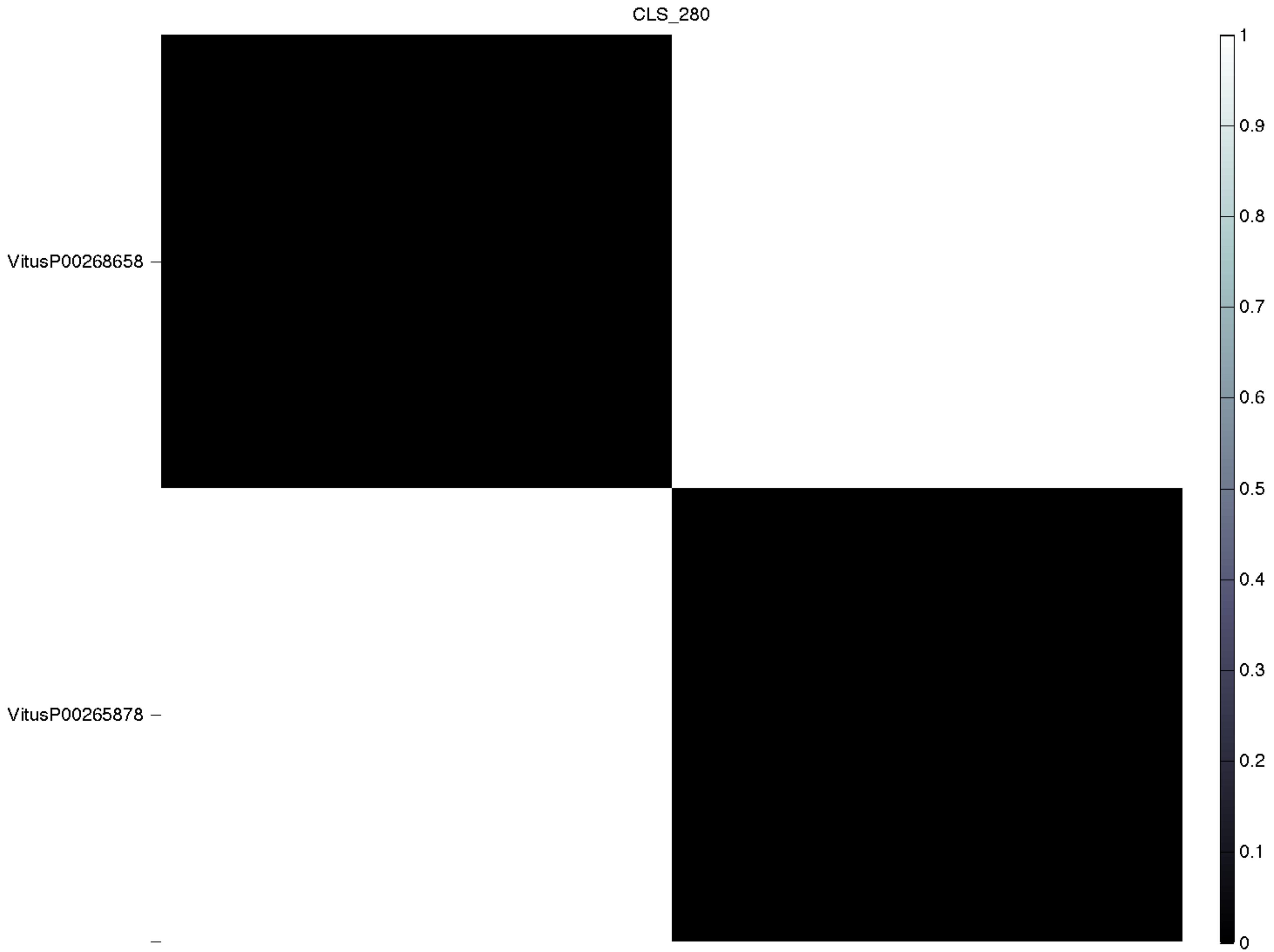
CLS_280
VitusP00268658
VitusP00265878
1
0.9
0.8
0.7
0.6
0.5
0.4
0.3
0.2
0.1
0

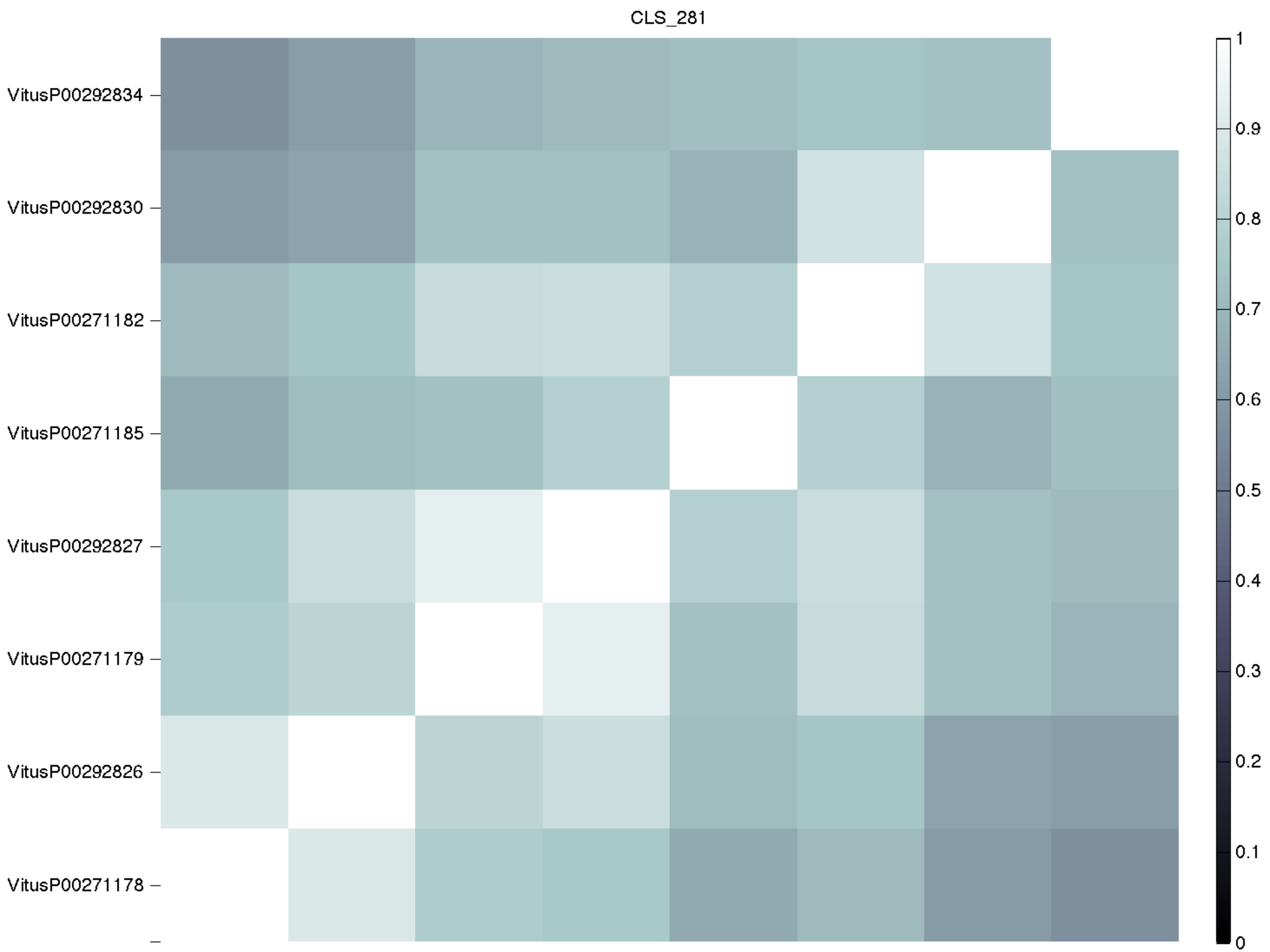
CLS_281
VitusP00292834
VitusP00292830
VitusP00271182
VitusP00271185
VitusP00292827
VitusP00271179
VitusP00292826
VitusP00271178
1
0.9
0.8
0.7
0.6
0.5
0.4
0.3
0.2
0.1
0

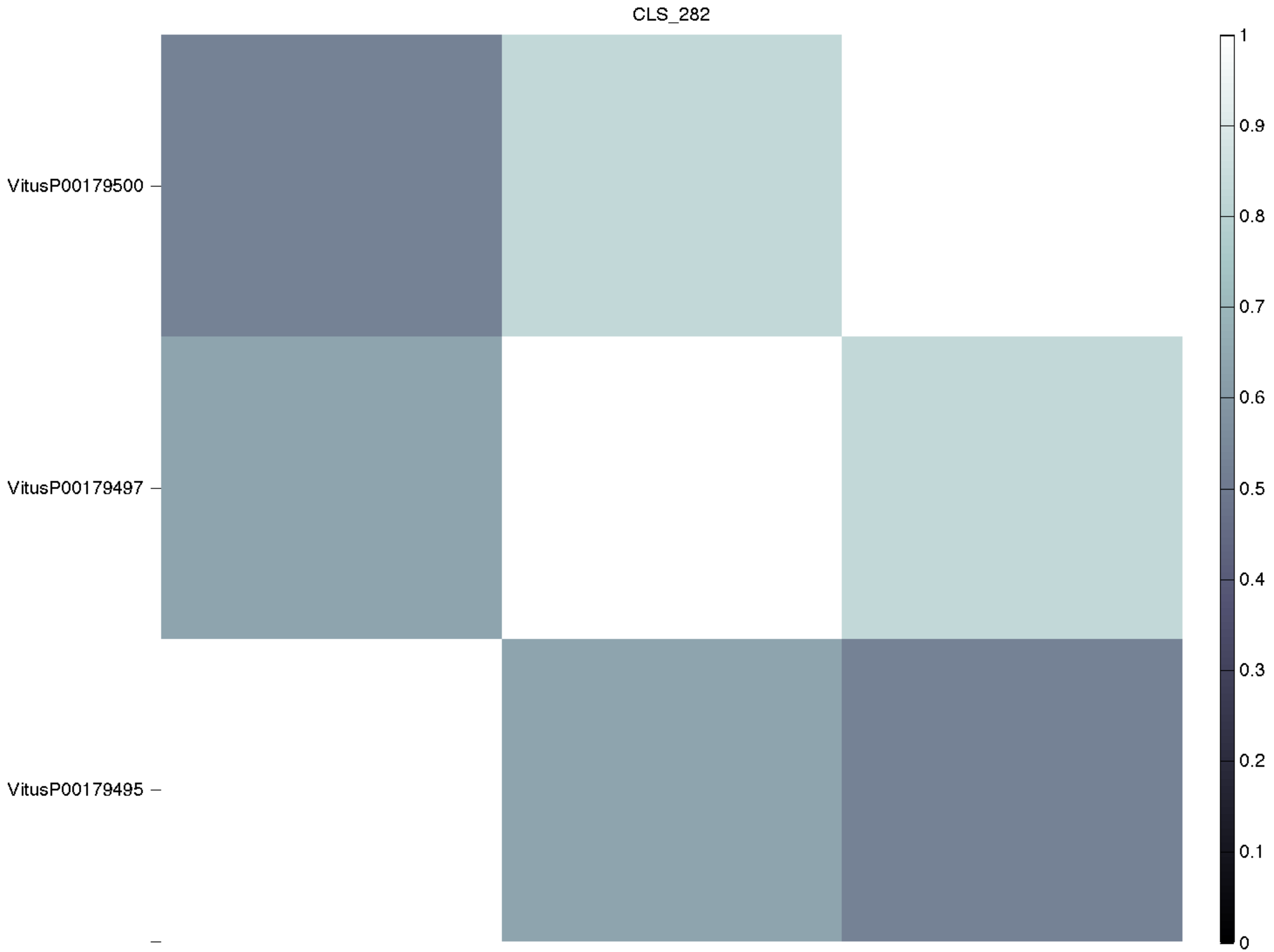

CLS_282
VitusP00179500
VitusP00179497
VitusP00179495
1
0.9
0.8
0.7
0.6
0.5
0.4
0.3
0.2
0.1
0

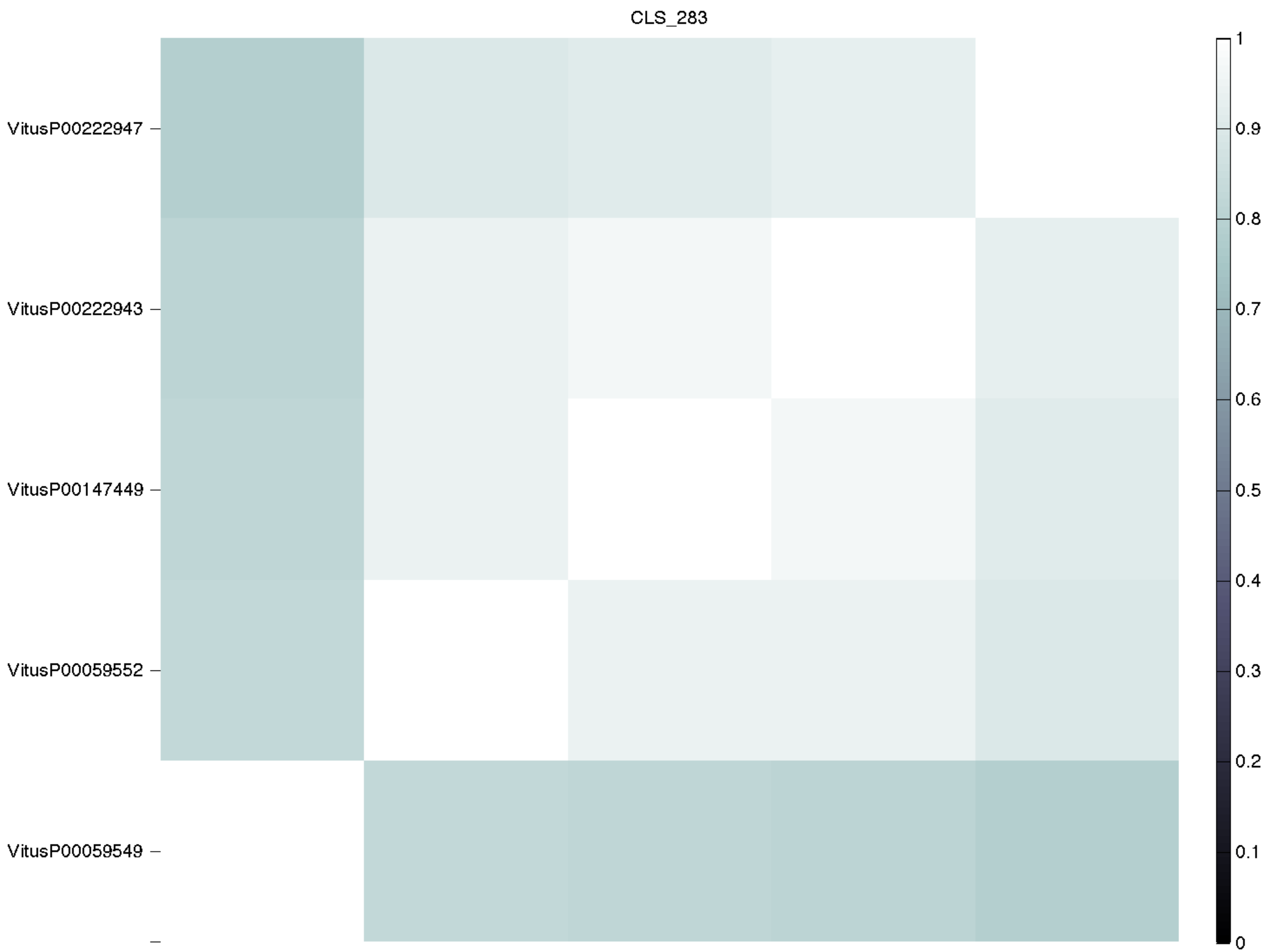
CLS_283
VitusP00222947
VitusP00222943
VitusP00147449
VitusP00059552
VitusP00059549
1
0.9
0.8
0.7
0.6
0.5
0.4
0.3
0.2
0.1
0

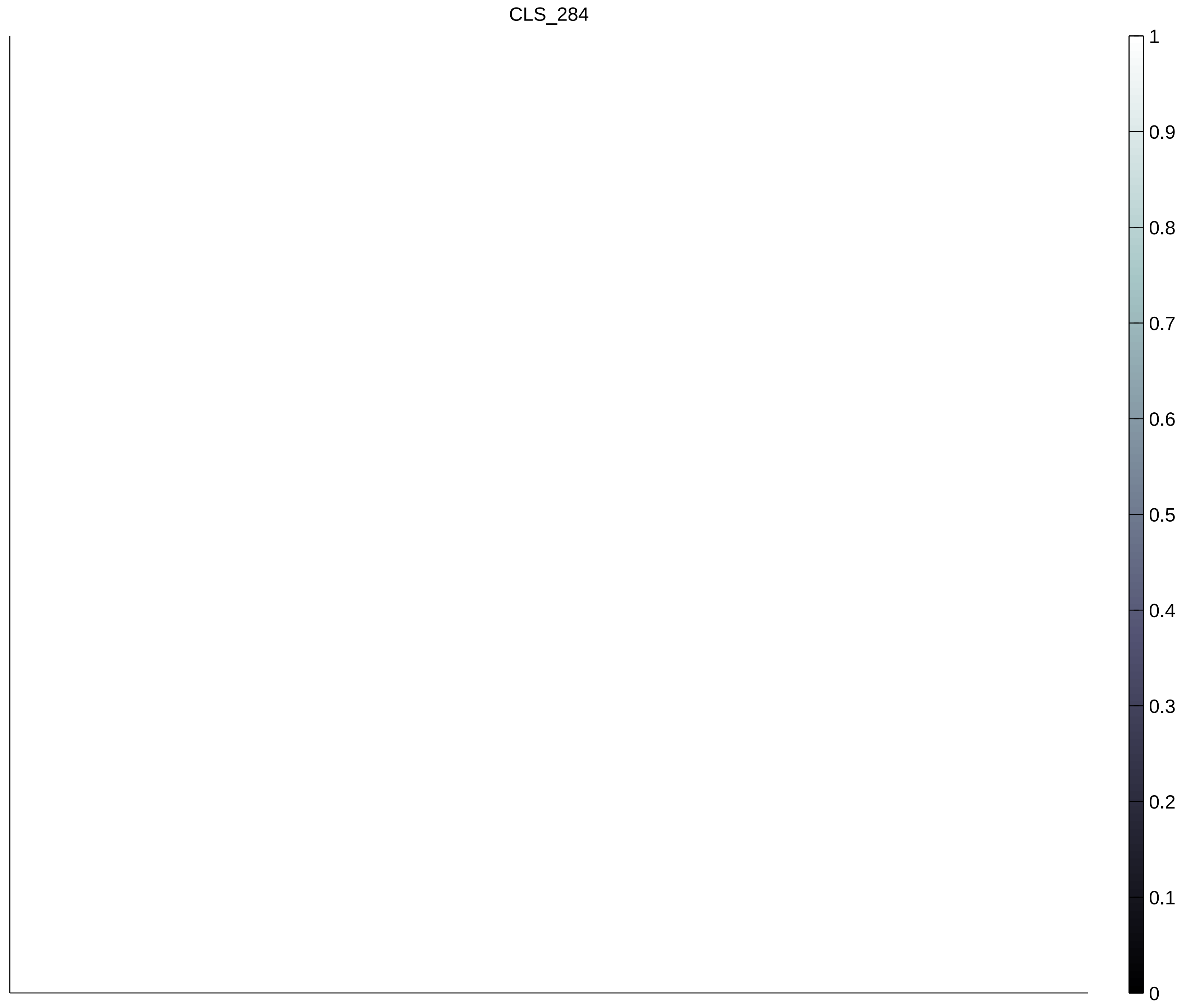
CLS_284
1
0.9
0.8
0.7
0.6
0.5
0.4
0.3
0.2
0.1
0

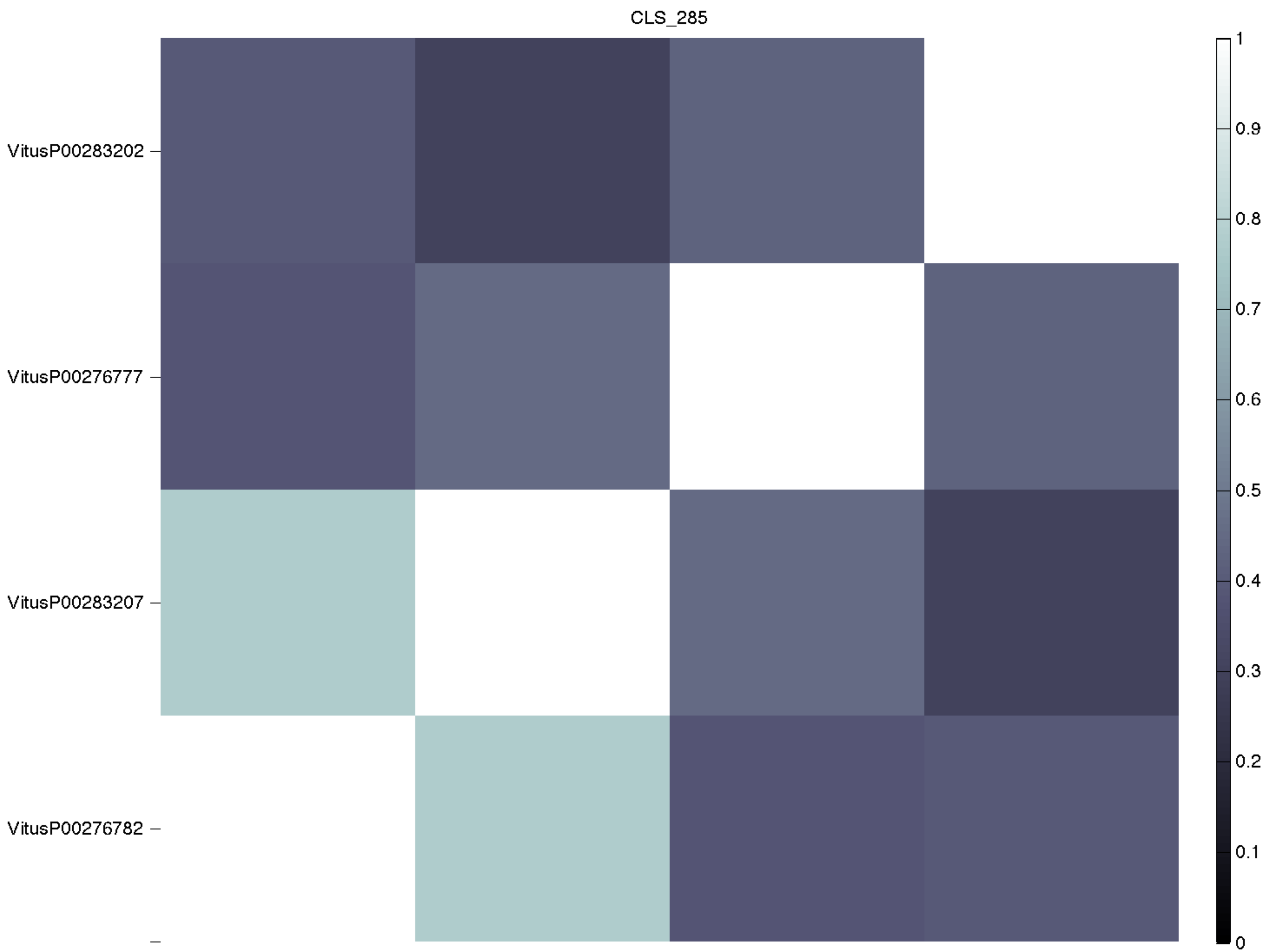

CLS_285
VitusP00283202
VitusP00276777
VitusP00283207
VitusP00276782
1
0.9
0.8
0.7
0.6
0.5
0.4
0.3
0.2
0.1
0

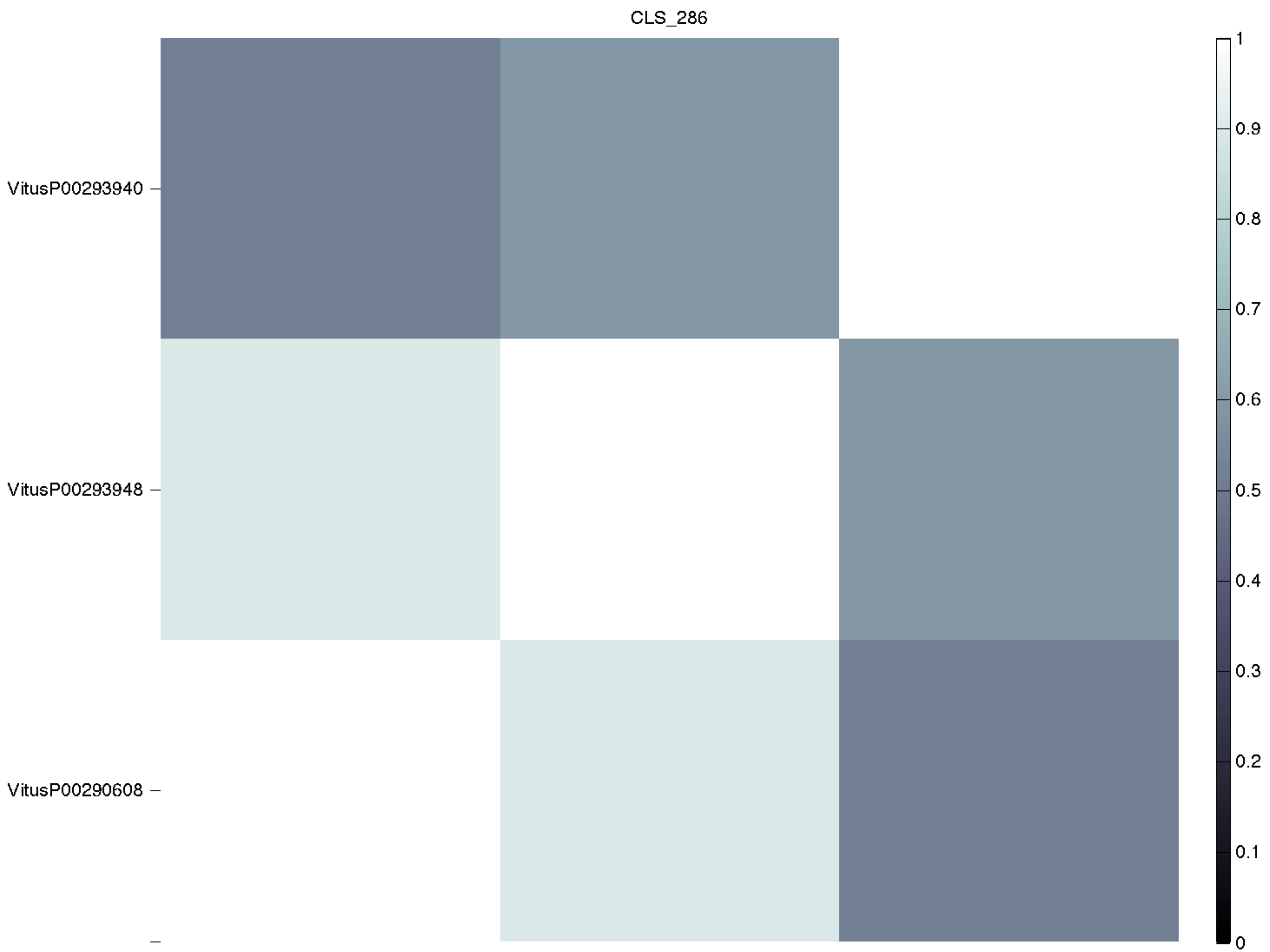

CLS_286
VitusP00293940
VitusP00293948
VitusP00290608
1
0.9
0.8
0.7
0.6
0.5
0.4
0.3
0.2
0.1
0

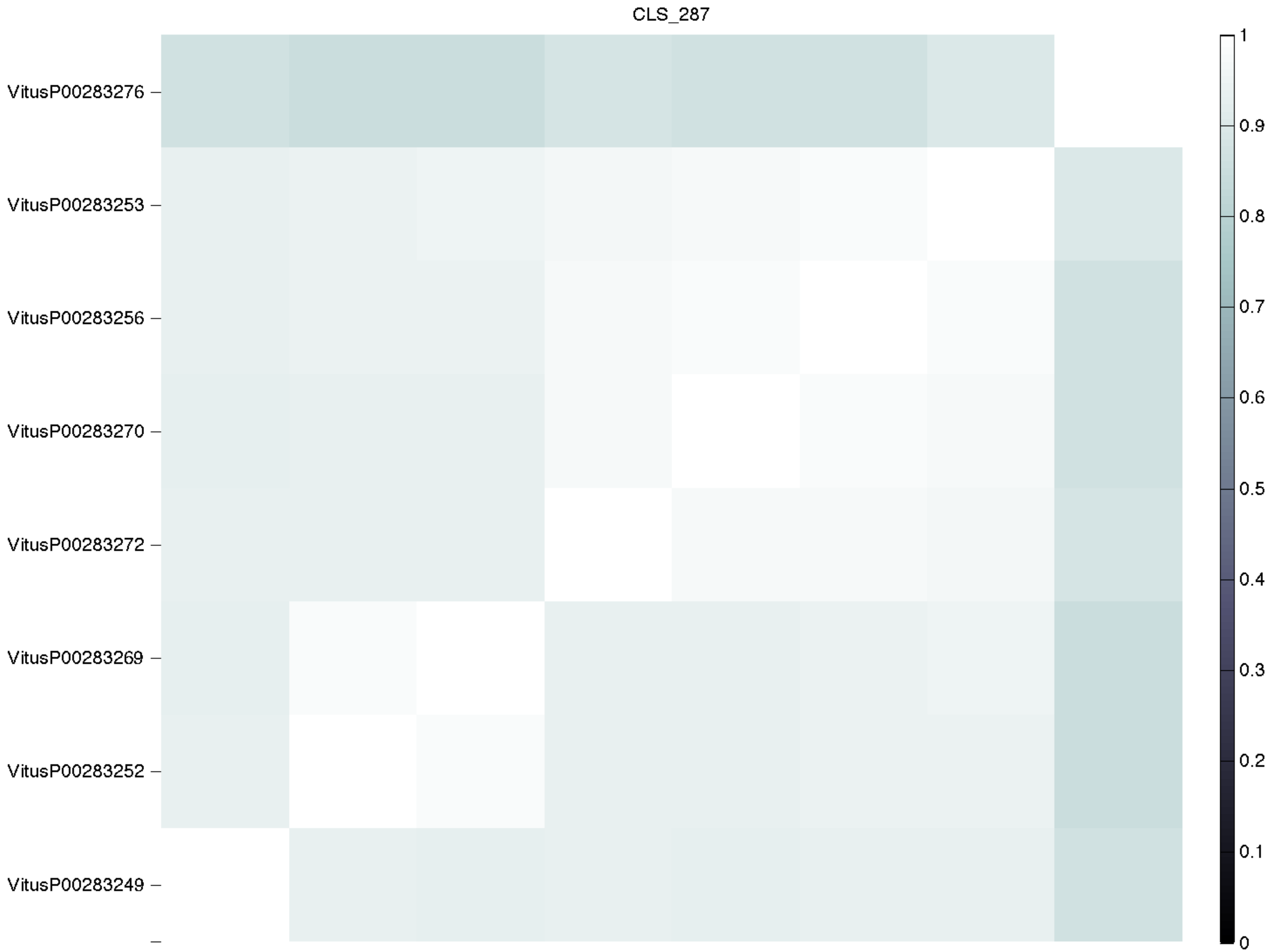
CLS_287
VitusP00283276
VitusP00283253
VitusP00283256
VitusP00283270
VitusP00283272
VitusP00283269
VitusP00283252
VitusP00283249
1
0.9
0.8
0.7
0.6
0.5
0.4
0.3
0.2
0.1
0

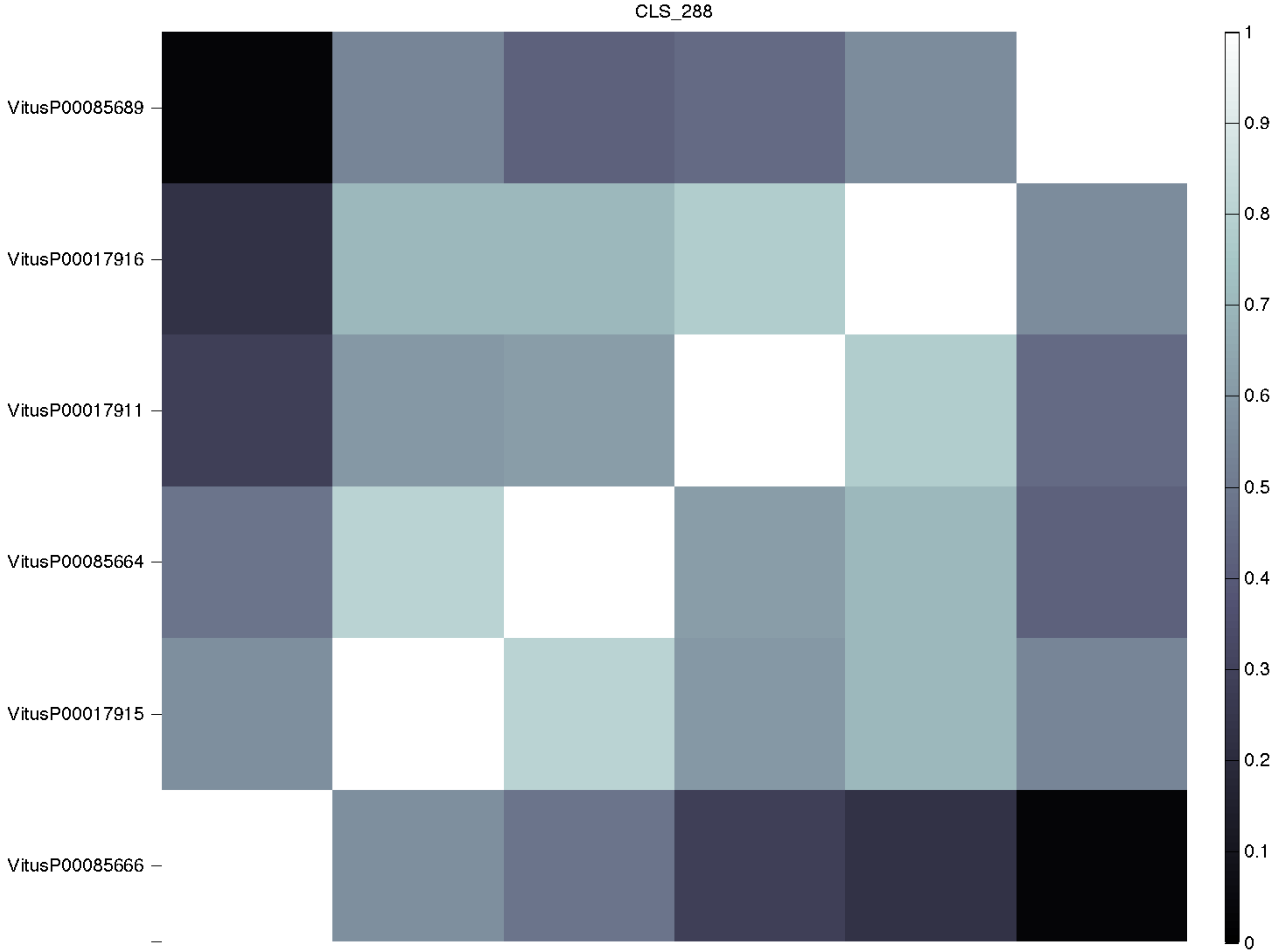
CLS_288
VitusP00085689
VitusP00017916
VitusP00017911
VitusP00085664
VitusP00017915
VitusP00085666
1
0.9
0.8
0.7
0.6
0.5
0.4
0.3
0.2
0.1
0

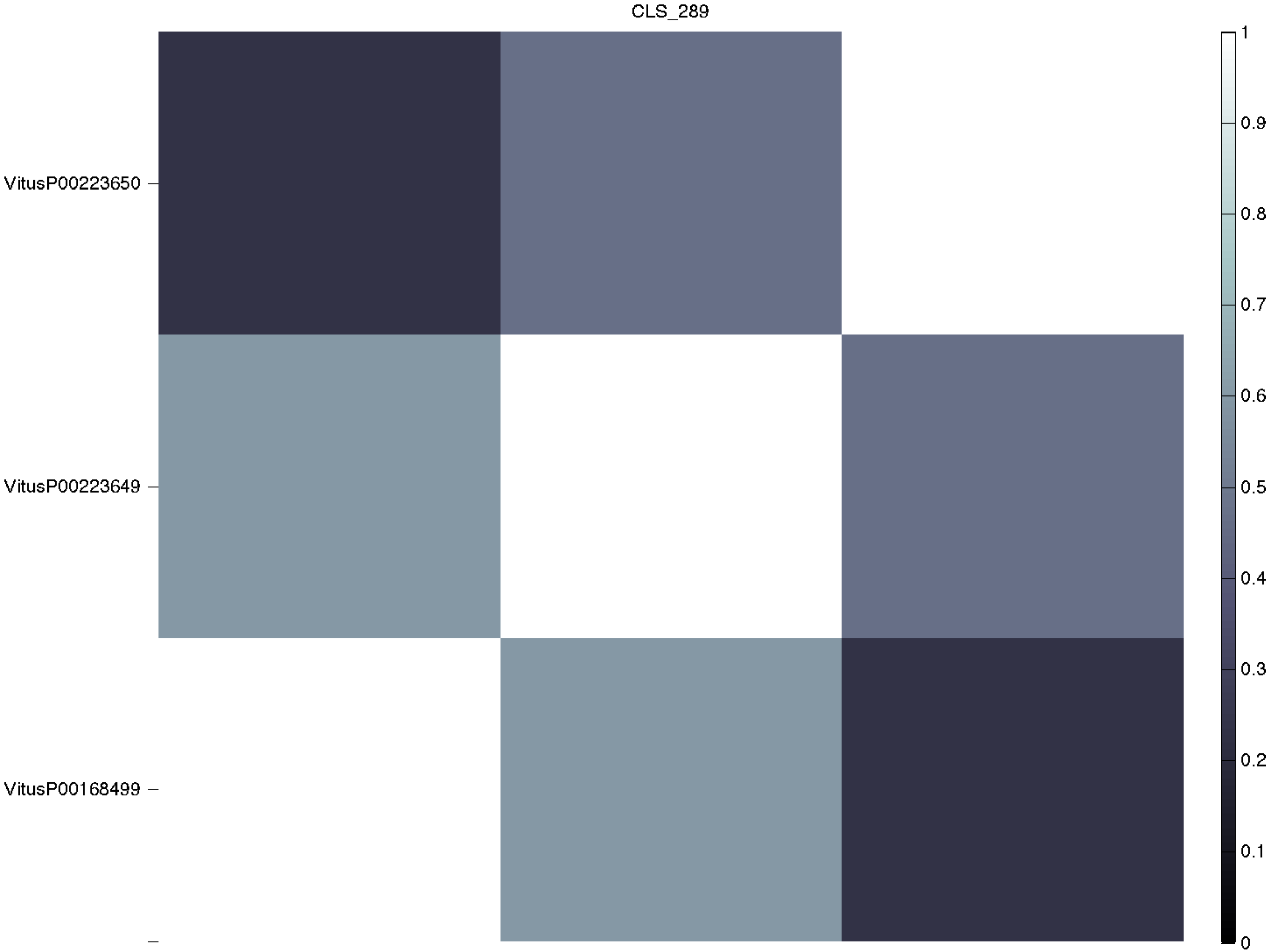
CLS_289
VitusP00223650
VitusP00223649
VitusP00168499
1
0.9
0.8
0.7
0.6
0.5
0.4
0.3
0.2
0.1
0

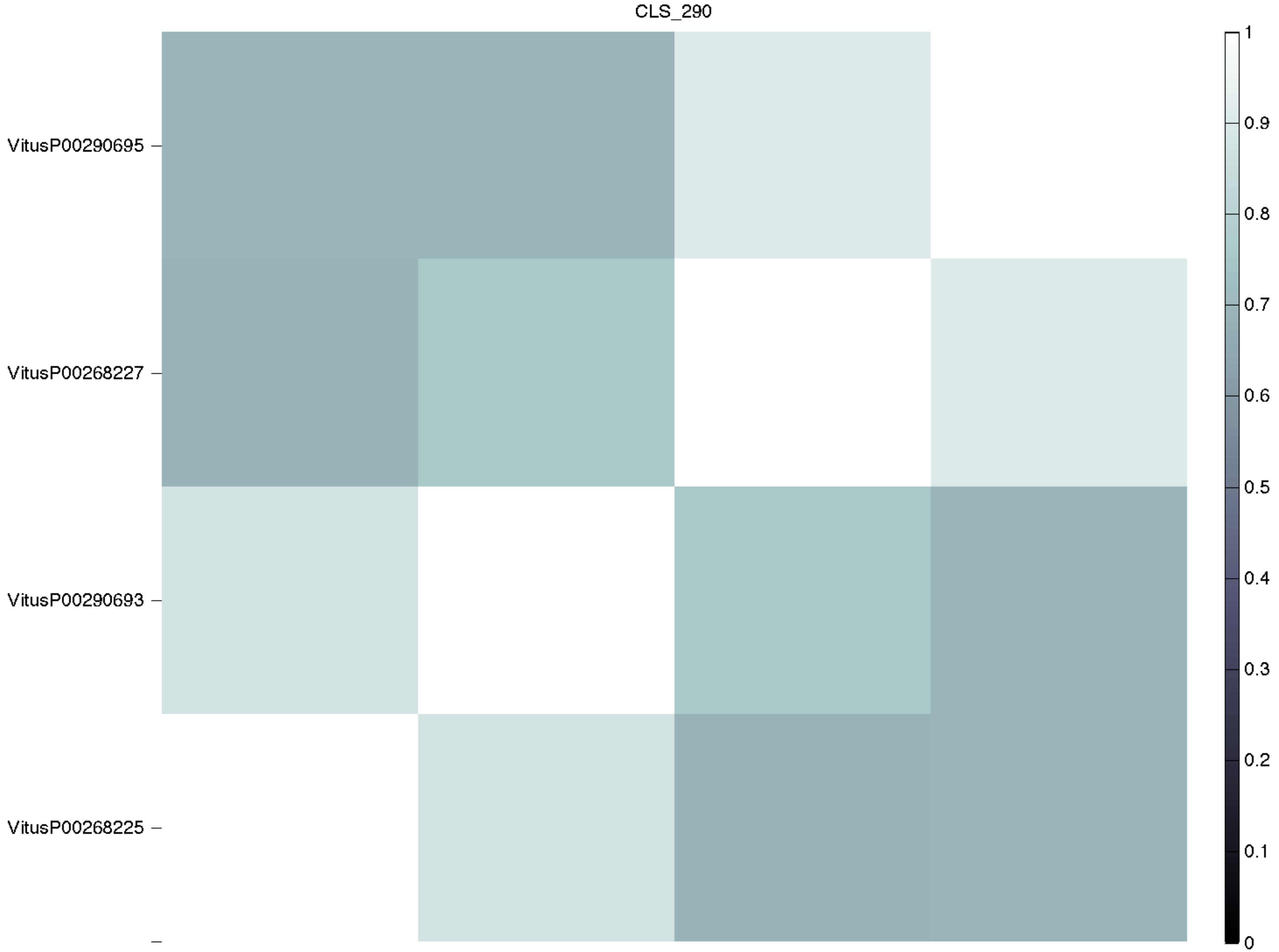
CLS_290
VitusP00290695
VitusP00268227
VitusP00290693
VitusP00268225
1
0.9
0.8
0.7
0.6
0.5
0.4
0.3
0.2
0.1
0

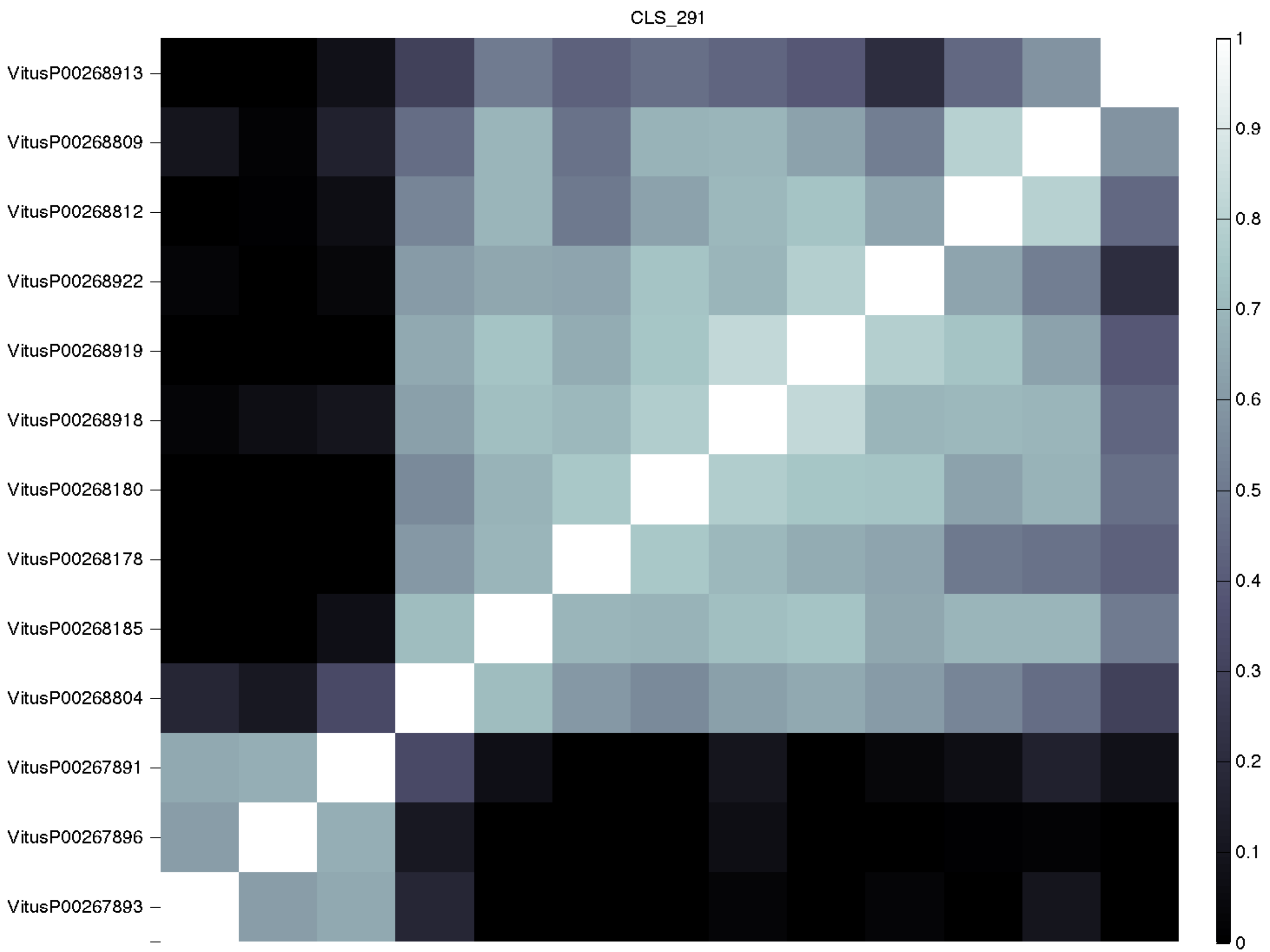

CLS_291
VitusP00268913
VitusP00268809
VitusP00268812
VitusP00268922
VitusP00268919
VitusP00268918
VitusP00268180
VitusP00268178
VitusP00268185
VitusP00268804
VitusP00267891
VitusP00267896
VitusP00267893
1
0.9
0.8
0.7
0.6
0.5
0.4
0.3
0.2
0.1
0

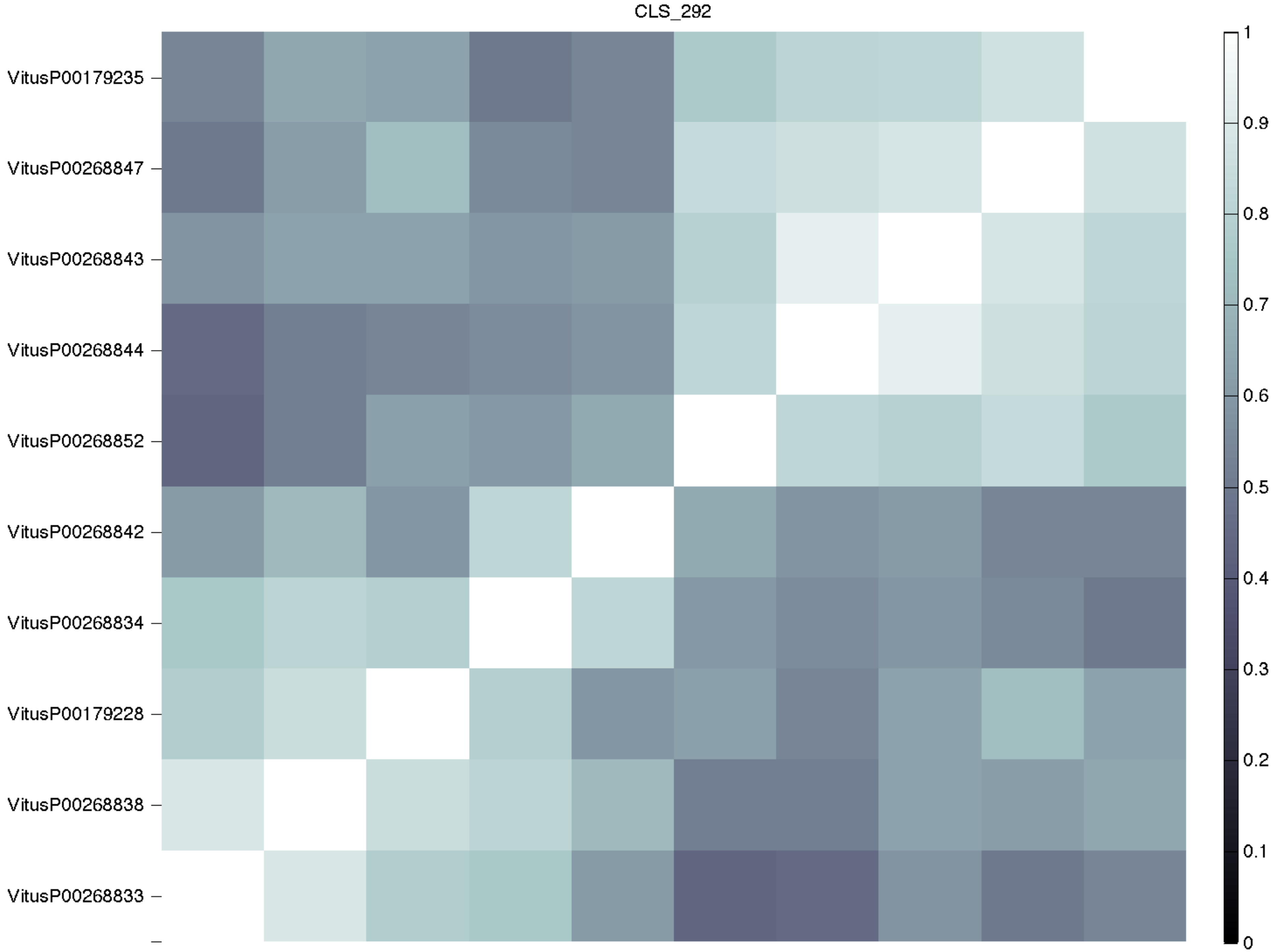
CLS_292
VitusP00179235
VitusP00268847
VitusP00268843
VitusP00268844
VitusP00268852
VitusP00268842
VitusP00268834
VitusP00179228
VitusP00268838
VitusP00268833
1
0.9
0.8
0.7
0.6
0.5
0.4
0.3
0.2
0.1
0

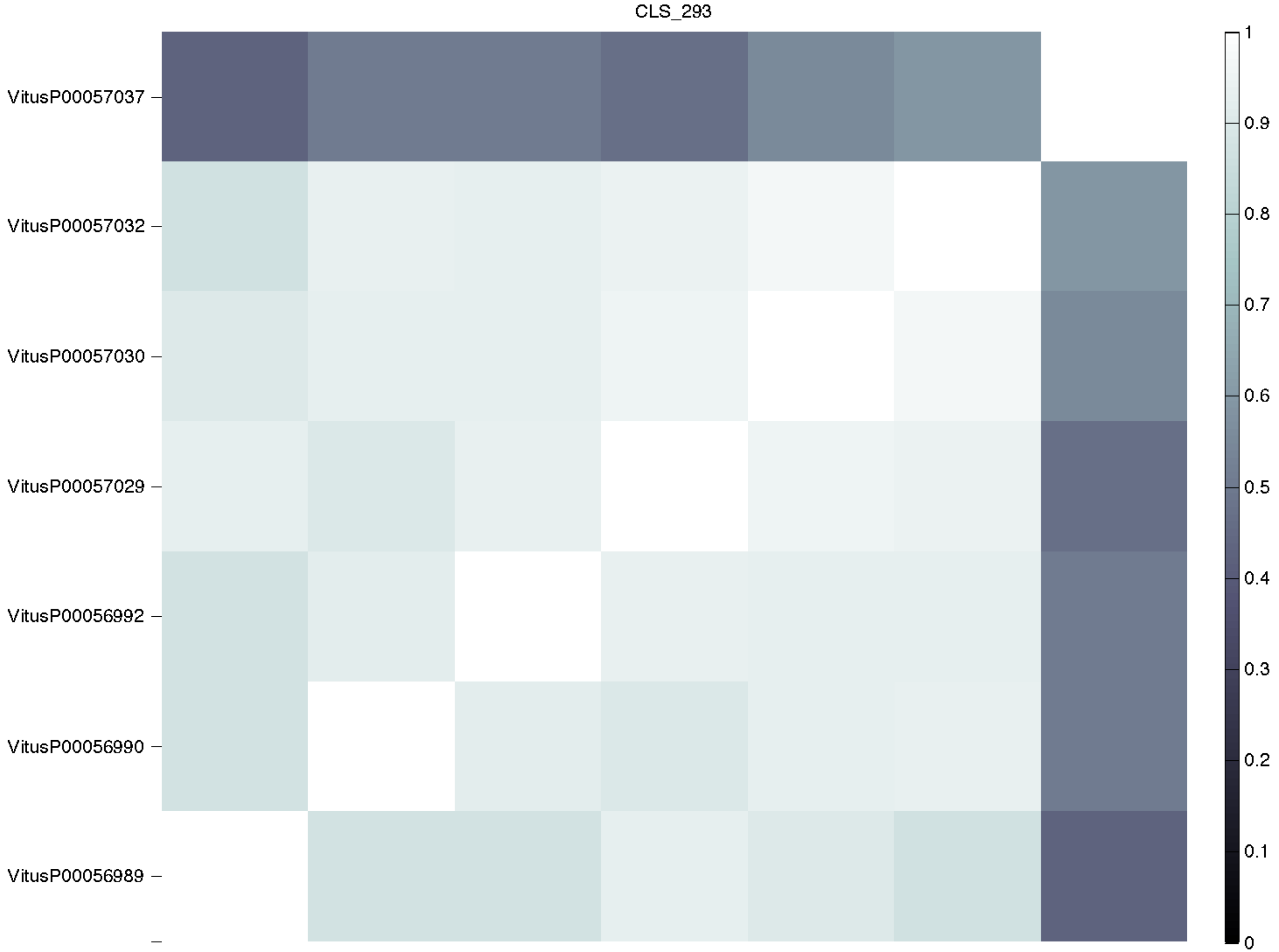

CLS_293
VitusP00057037
VitusP00057032
VitusP00057030
VitusP00057029
VitusP00056992
VitusP00056990
VitusP00056989
1
0.9
0.8
0.7
0.6
0.5
0.4
0.3
0.2
0.1
0

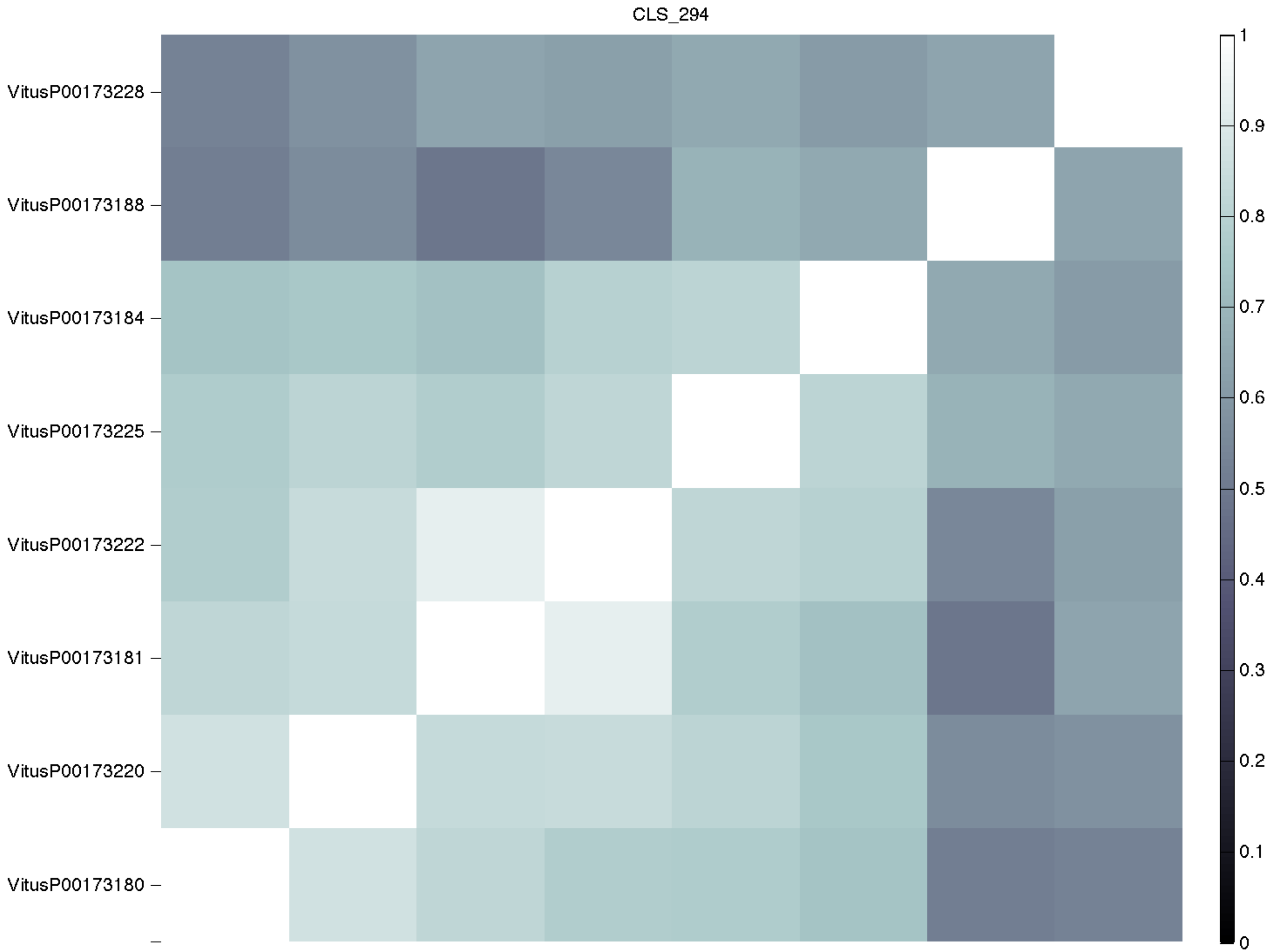
CLS_294
VitusP00173228
VitusP00173188
VitusP00173184
VitusP00173225
VitusP00173222
VitusP00173181
VitusP00173220
VitusP00173180
1
0.9
0.8
0.7
0.6
0.5
0.4
0.3
0.2
0.1
0

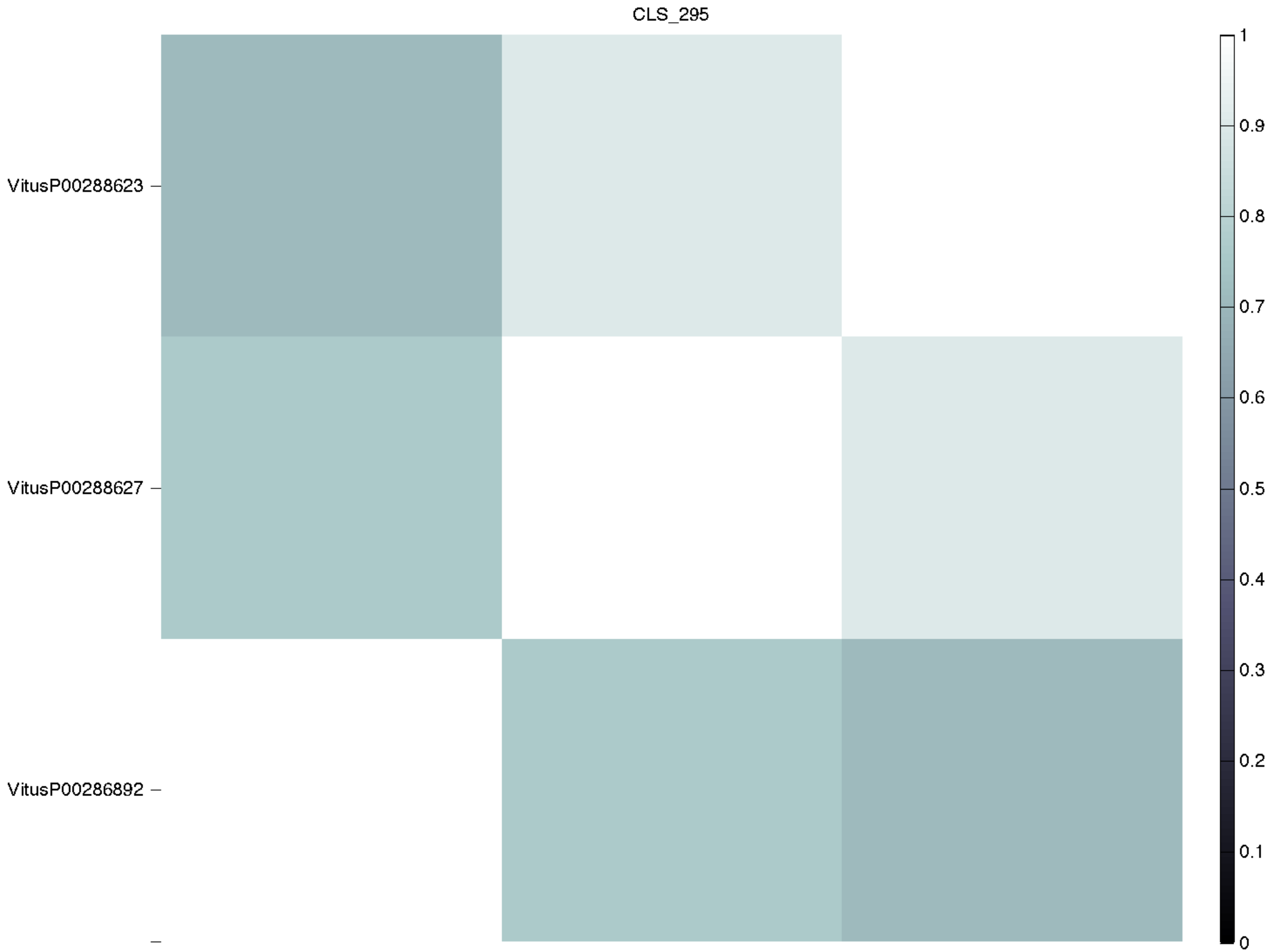

CLS_295
VitusP00288623
VitusP00288627
VitusP00286892
1
0.9
0.8
0.7
0.6
0.5
0.4
0.3
0.2
0.1
0

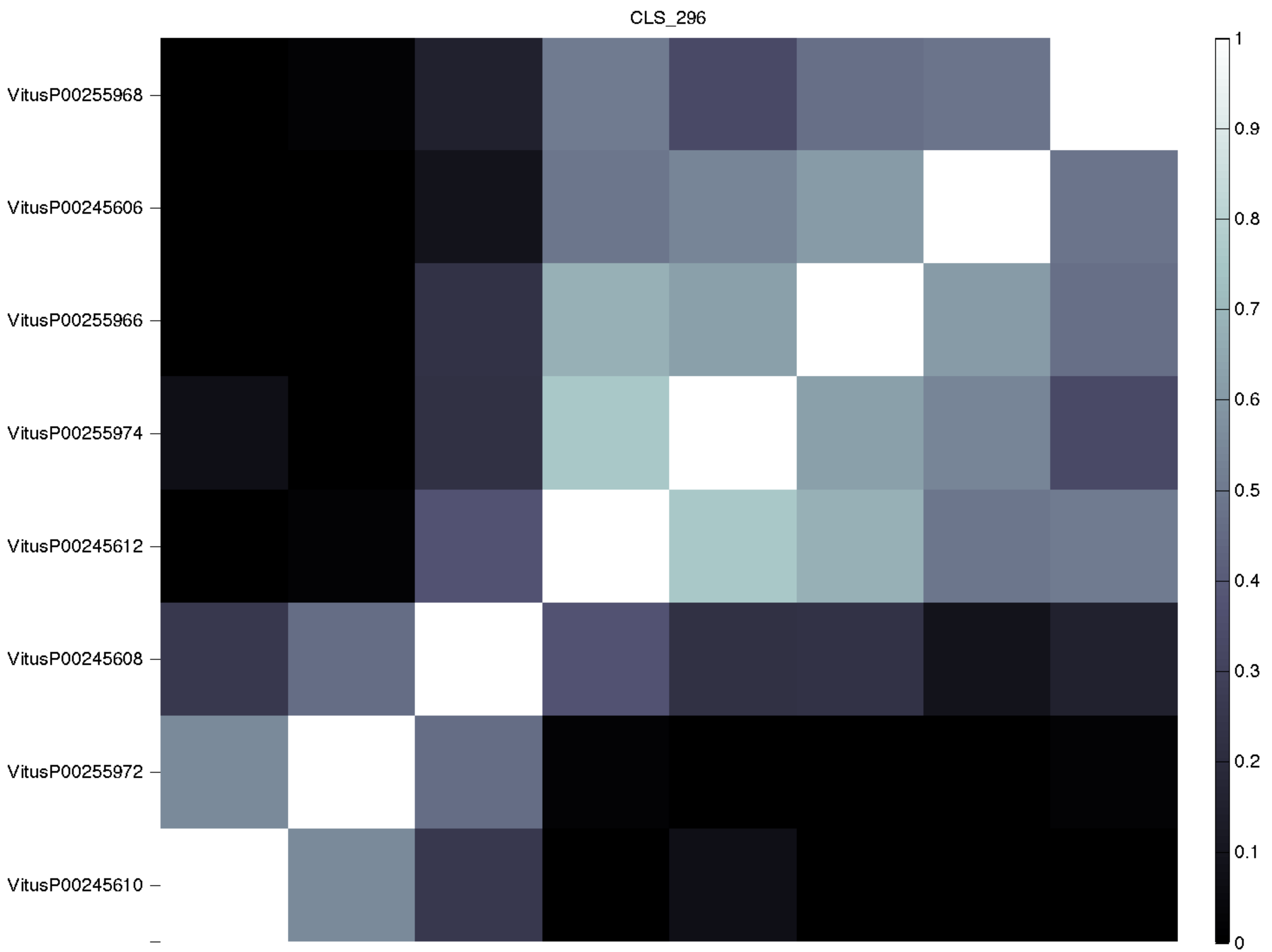

CLS_296
VitusP00255968
VitusP00245606
VitusP00255966
VitusP00255974
VitusP00245612
VitusP00245608
VitusP00255972
VitusP00245610
1
0.9
0.8
0.7
0.6
0.5
0.4
0.3
0.2
0.1
0

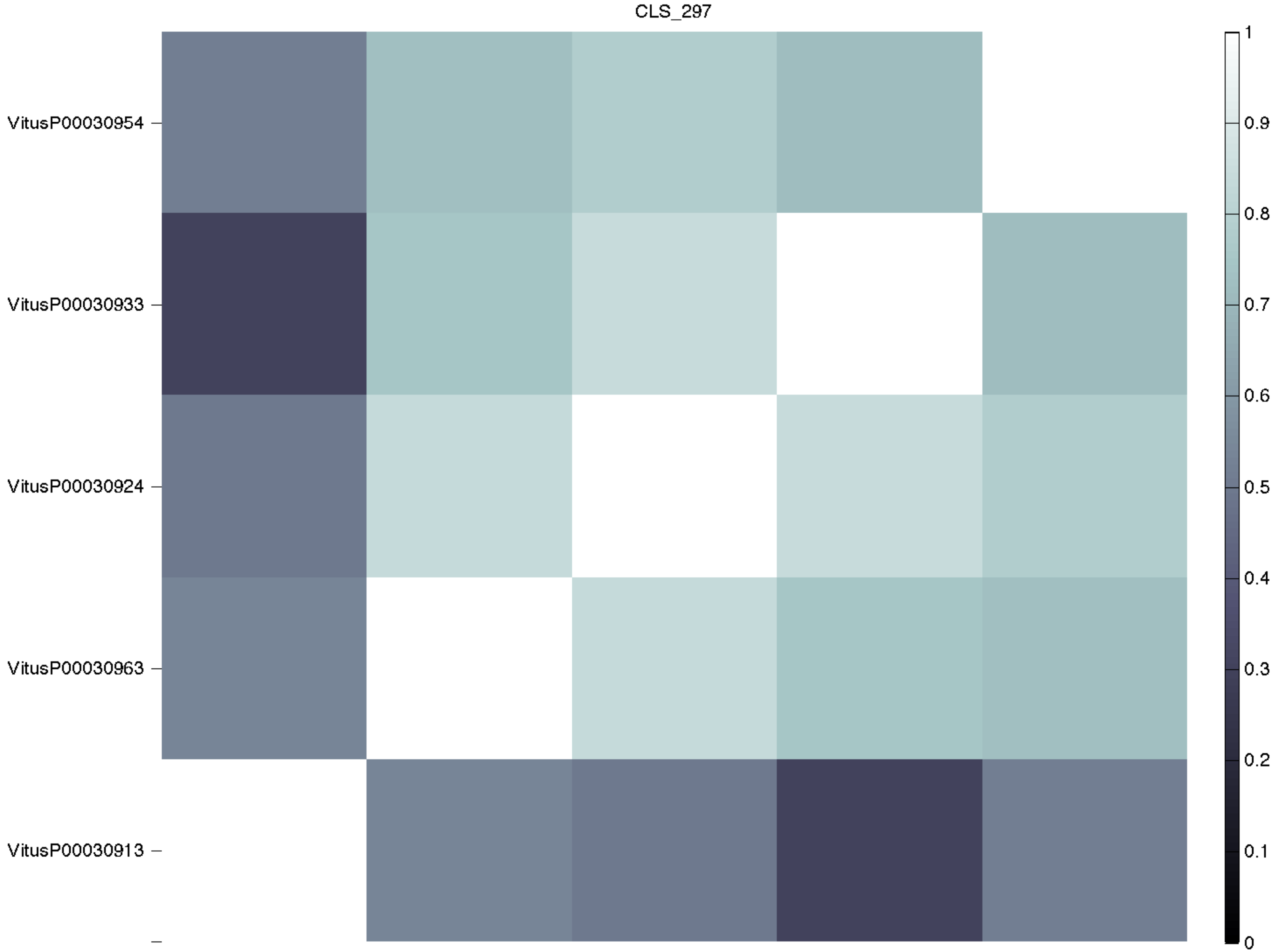

CLS_297
VitusP00030954
VitusP00030933
VitusP00030924
VitusP00030963
VitusP00030913
1
0.9
0.8
0.7
0.6
0.5
0.4
0.3
0.2
0.1
0

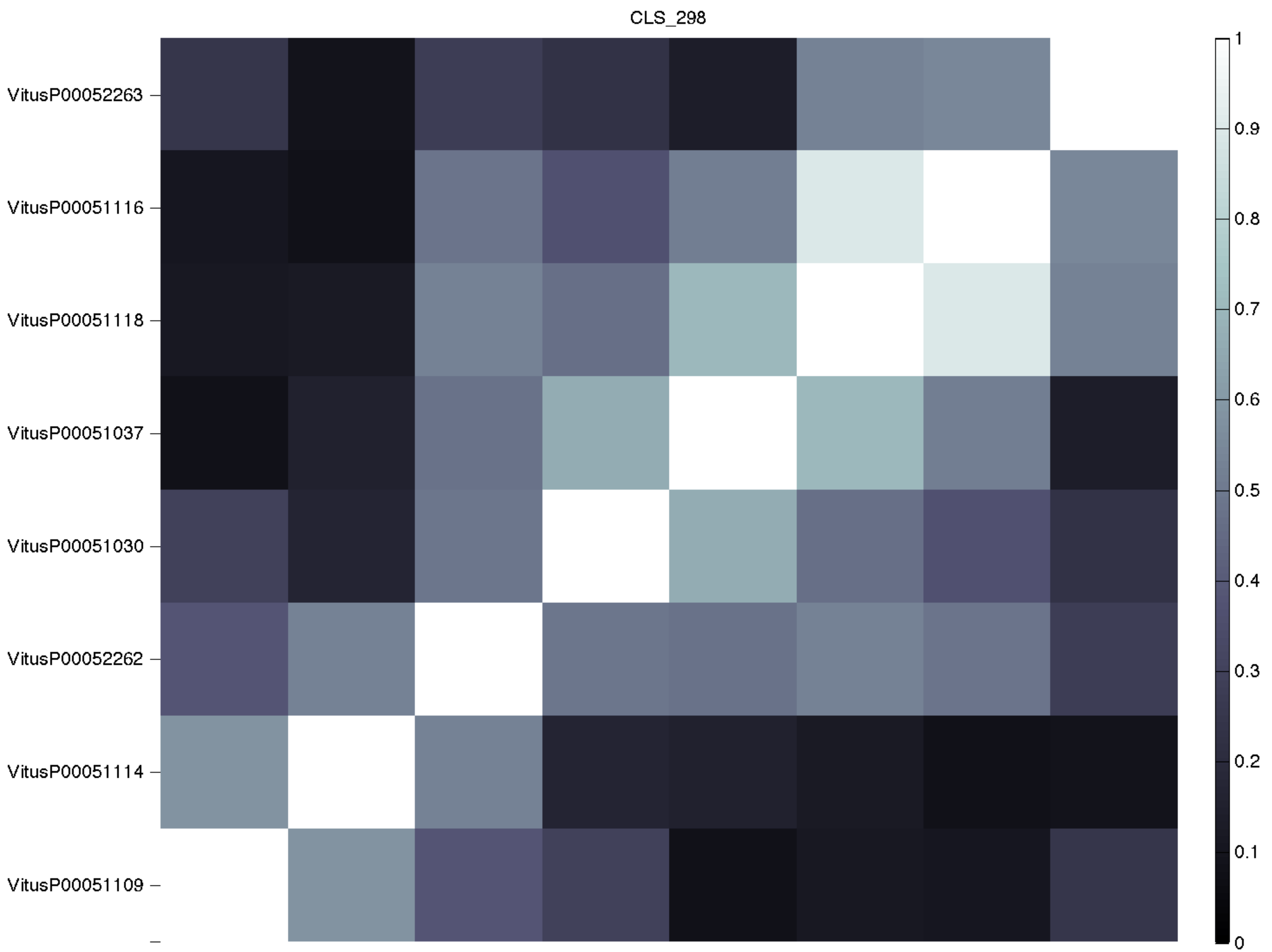
CLS_298
VitusP00052263
VitusP00051116
VitusP00051118
VitusP00051037
VitusP00051030
VitusP00052262
VitusP00051114
VitusP00051109
1
0.9
0.8
0.7
0.6
0.5
0.4
0.3
0.2
0.1
0

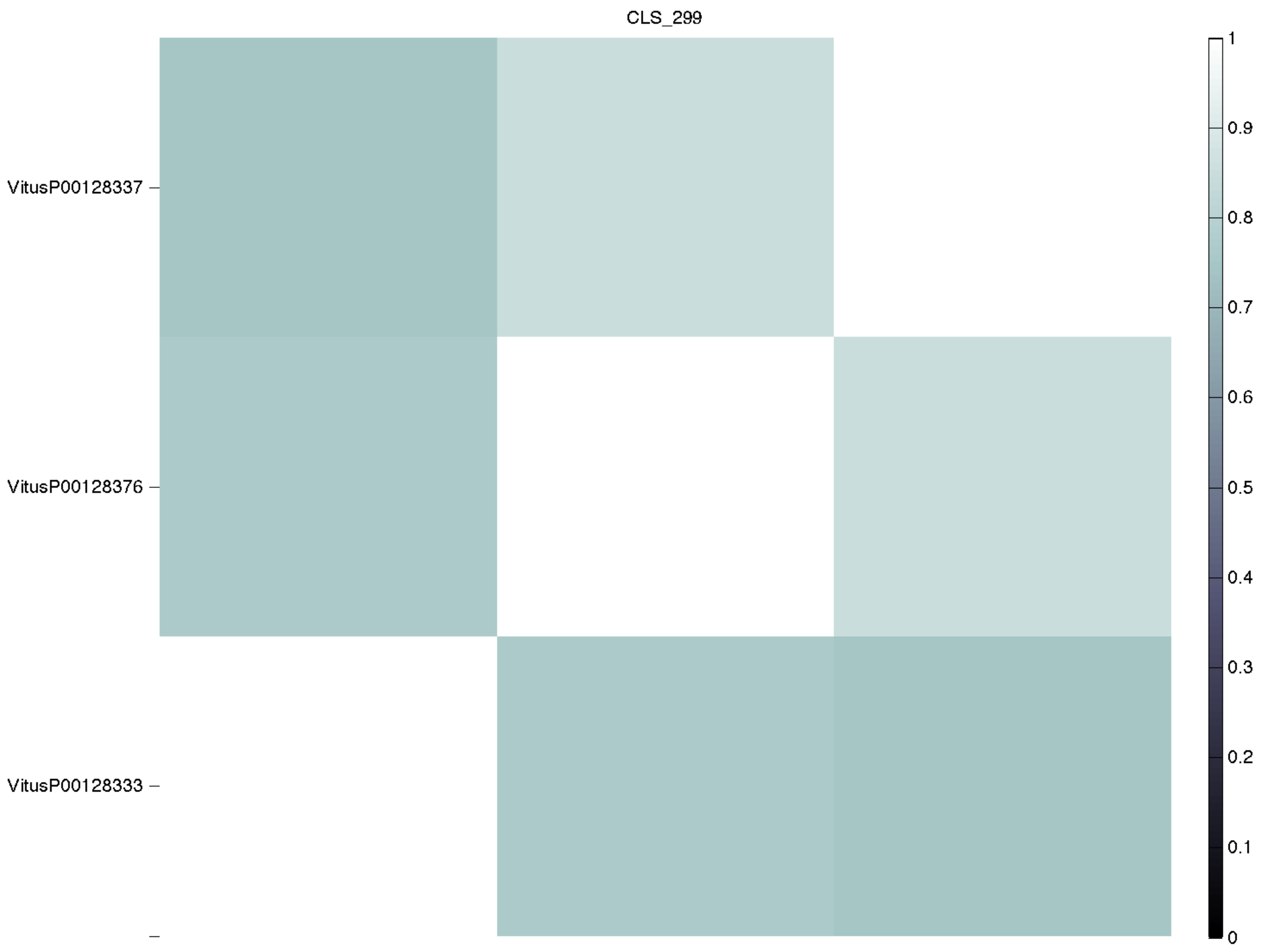
CLS_299
VitusP00128337
VitusP00128376
VitusP00128333
1
0.9
0.8
0.7
0.6
0.5
0.4
0.3
0.2
0.1
0

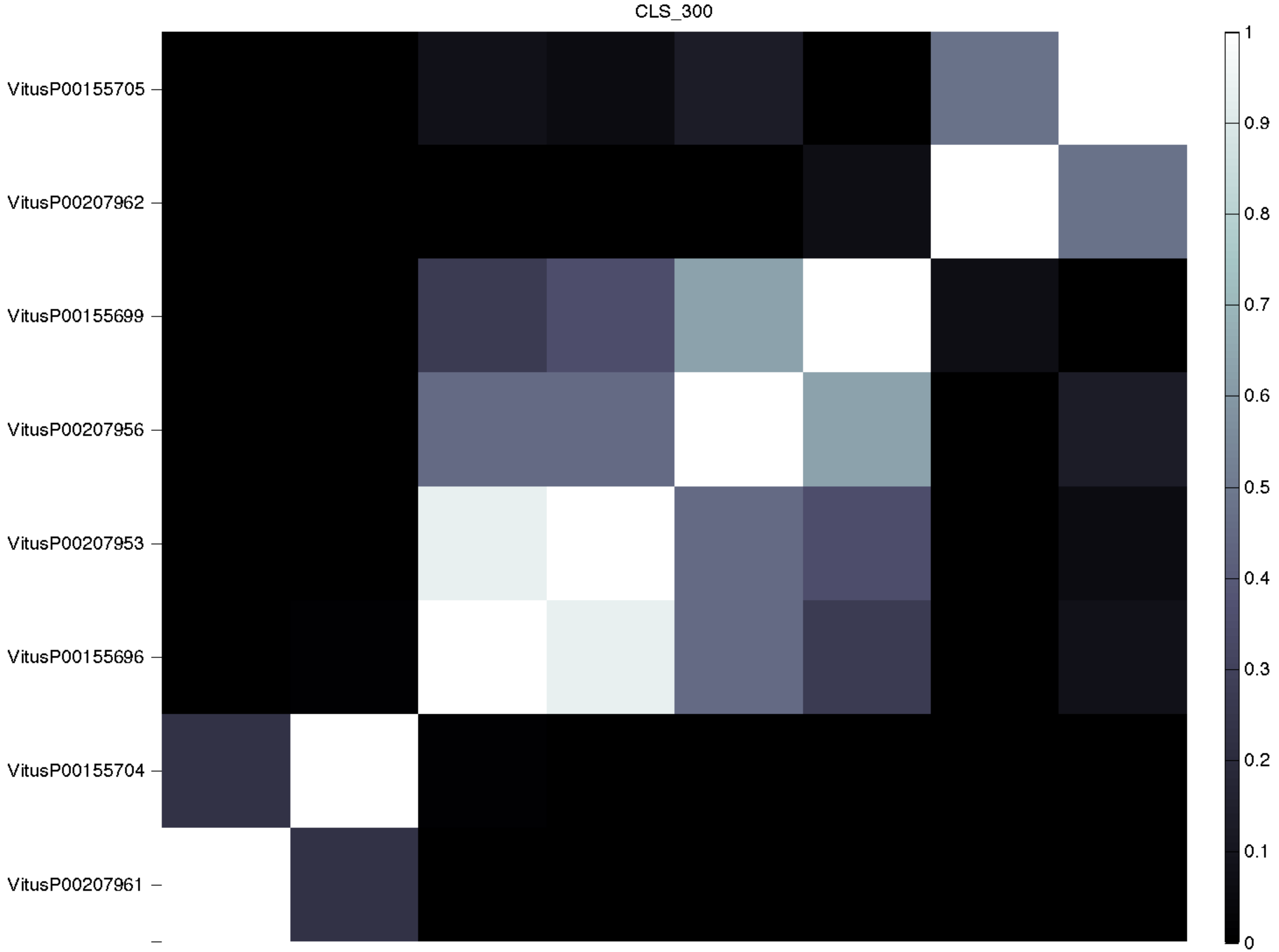
CLS_300
VitusP00155705
VitusP00207962
VitusP00155699
VitusP00207956
VitusP00207953
VitusP00155696
VitusP00155704
VitusP00207961
1
0.9
0.8
0.7
0.6
0.5
0.4
0.3
0.2
0.1
0

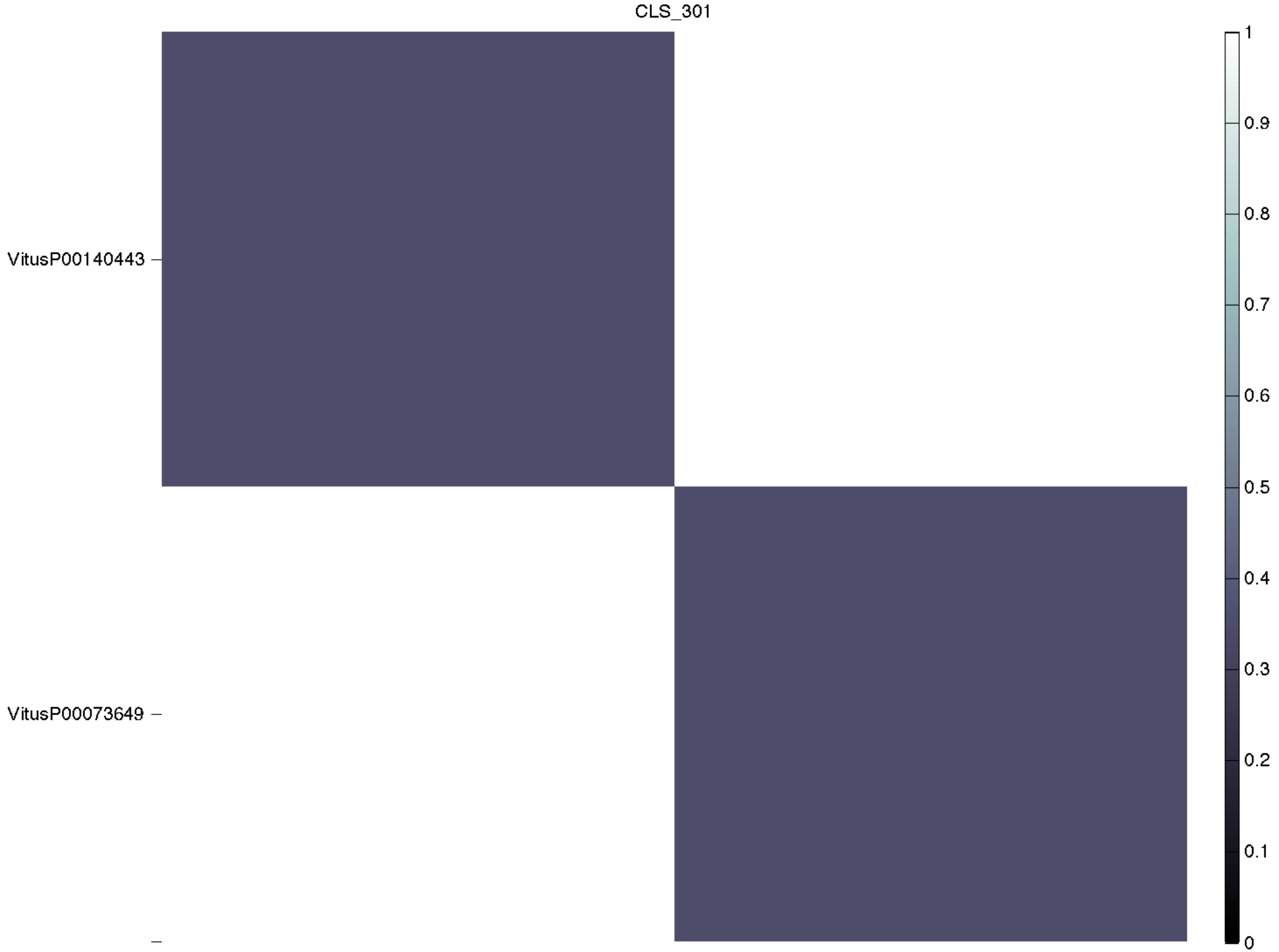

CLS_301
VitusP00140443
VitusP00073649
1
0.9
0.8
0.7
0.6
0.5
0.4
0.3
0.2
0.1
0

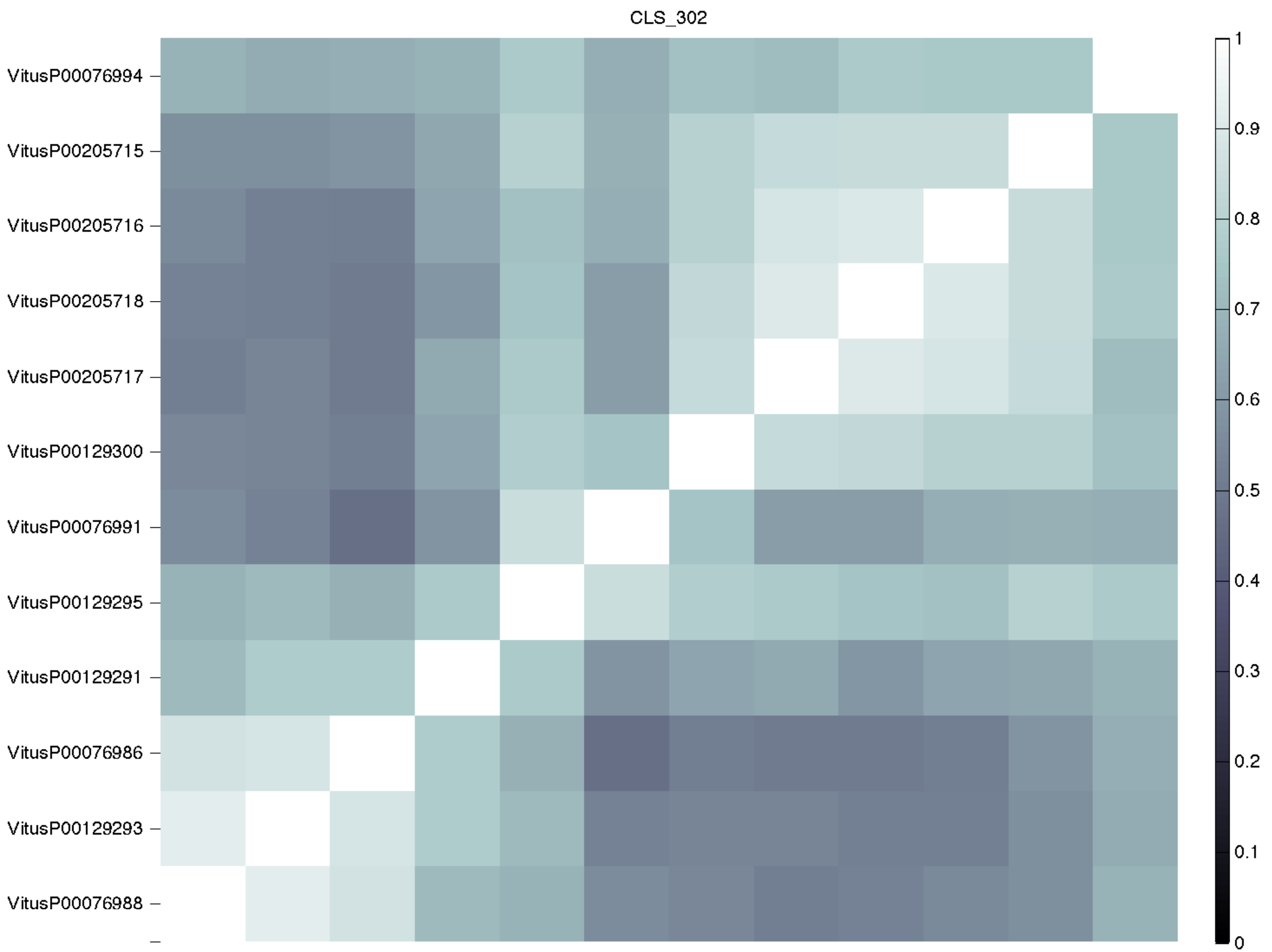
CLS_302
VitusP00076994
VitusP00205715
VitusP00205716
VitusP00205718
VitusP00205717
VitusP00129300
VitusP00076991
VitusP00129295
VitusP00129291
VitusP00076986
VitusP00129293
VitusP00076988
1
0.9
0.8
0.7
0.6
0.5
0.4
0.3
0.2
0.1
0

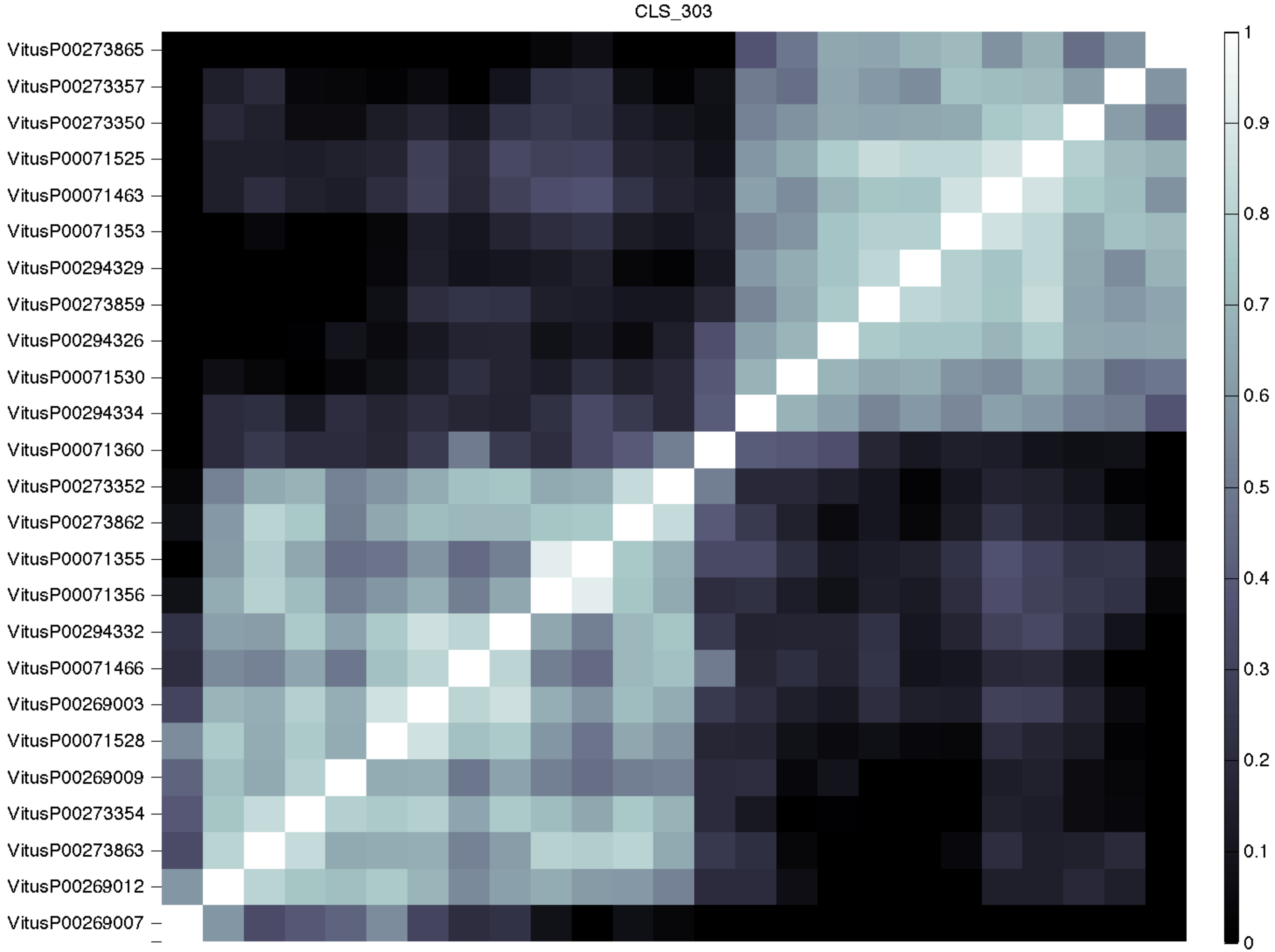

CLS_303
VitusP00273865
VitusP00273357
VitusP00273350
VitusP00071525
VitusP00071463
VitusP00071353
VitusP00294329
VitusP00273859
VitusP00294326
VitusP00071530
VitusP00294334
VitusP00071360
VitusP00273352
VitusP00273862
VitusP00071355
VitusP00071356
VitusP00294332
VitusP00071466
VitusP00269003
VitusP00071528
VitusP00269009
VitusP00273354
VitusP00273863
VitusP00269012
VitusP00269007
1
0.9
0.8
0.7
0.6
0.5
0.4
0.3
0.2
0.1
0

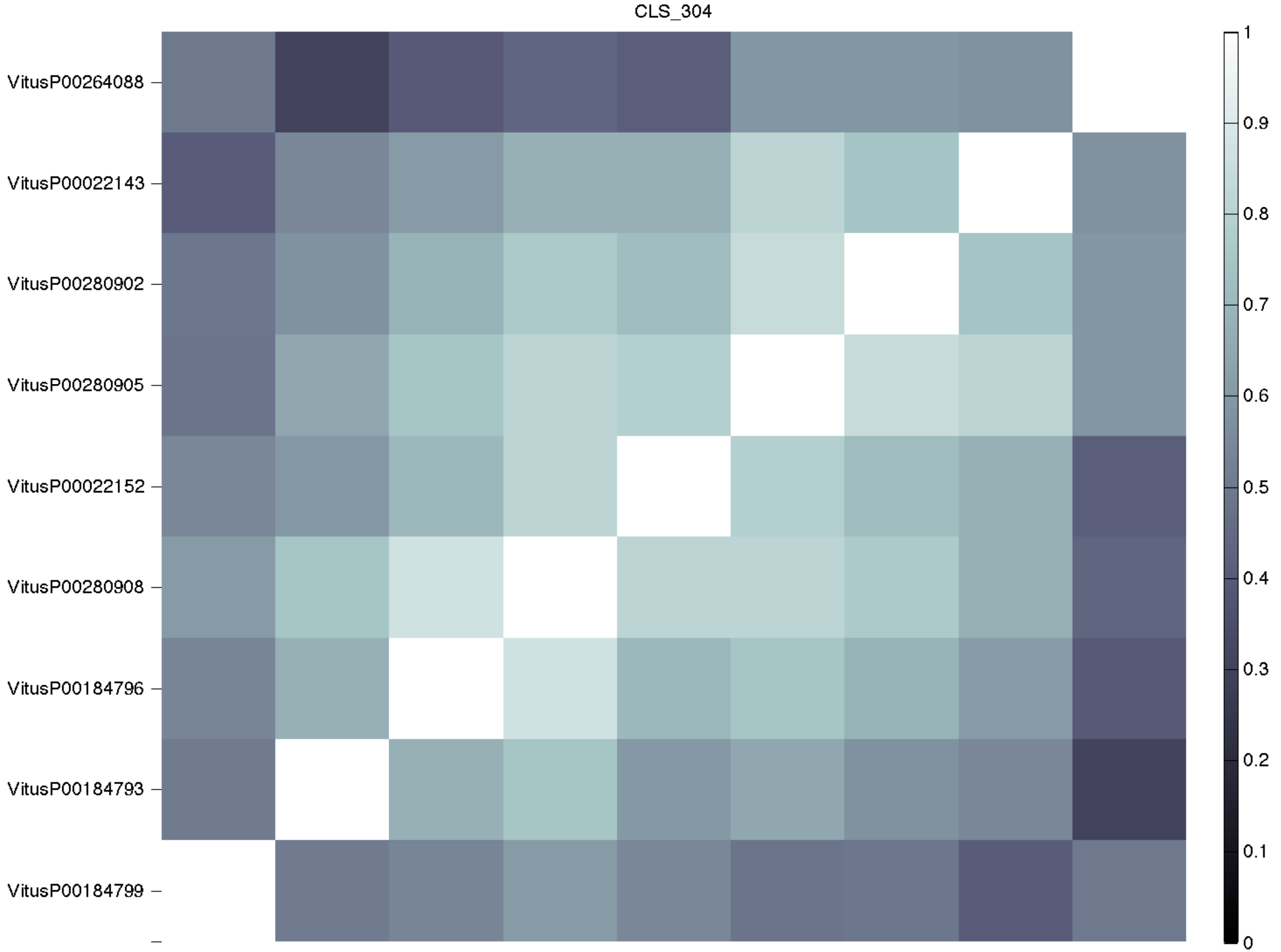
CLS_304
VitusP00264088
VitusP00022143
VitusP00280902
VitusP00280905
VitusP00022152
VitusP00280908
VitusP00184796
VitusP00184793
VitusP00184799
1
0.9
0.8
0.7
0.6
0.5
0.4
0.3
0.2
0.1
0

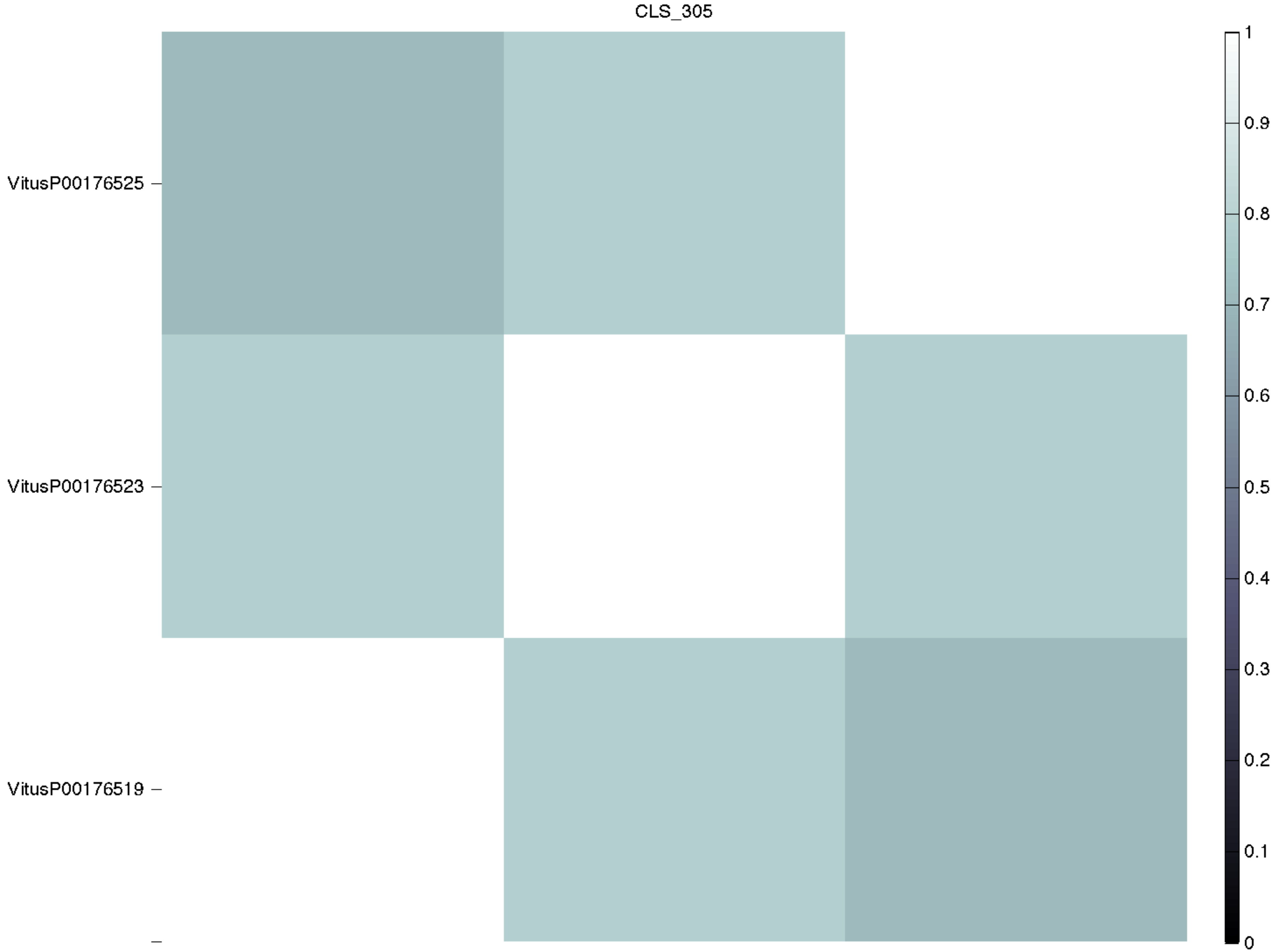
CLS_305
VitusP00176525
VitusP00176523
VitusP00176519
1
0.9
0.8
0.7
0.6
0.5
0.4
0.3
0.2
0.1
0

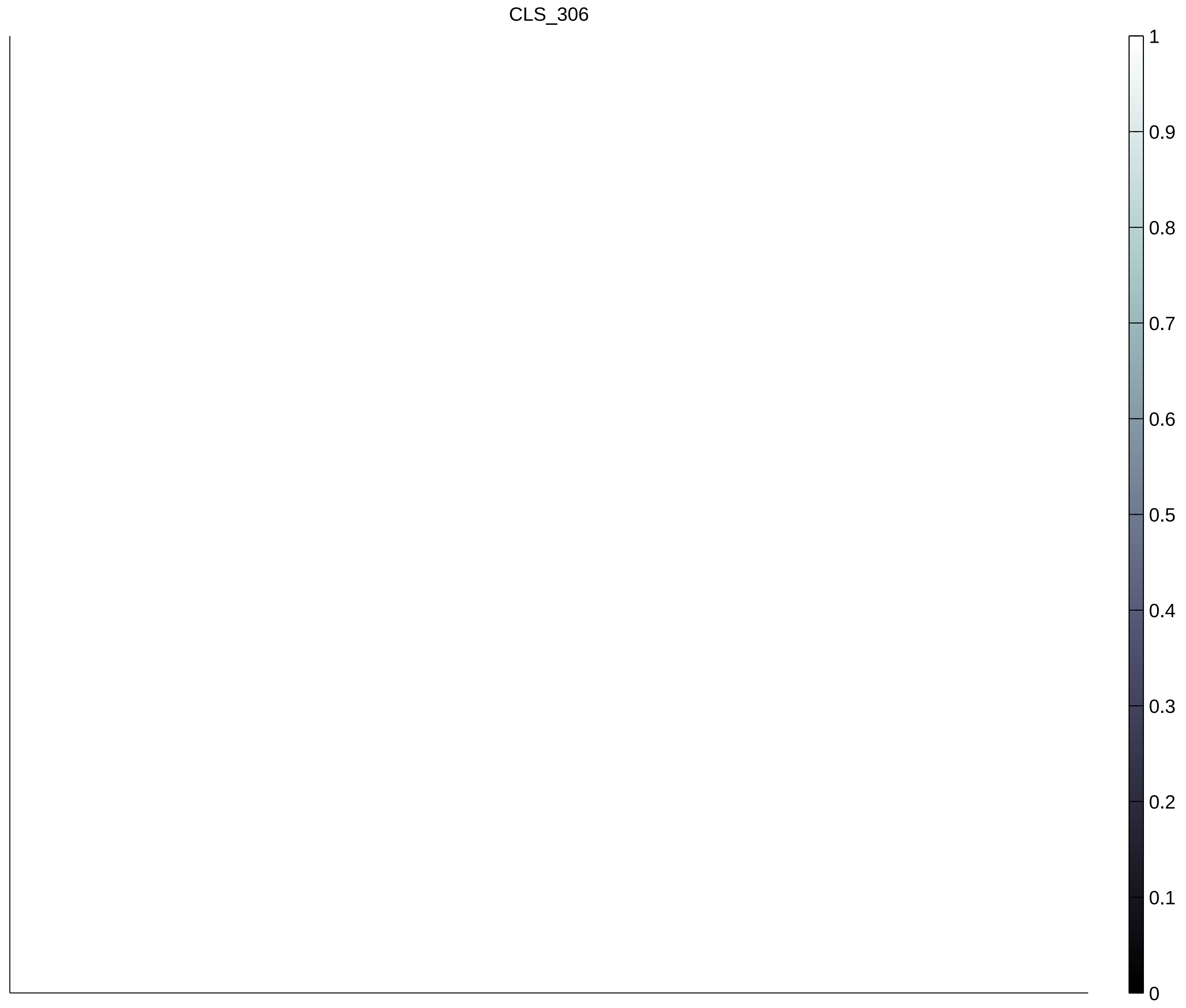
CLS_306
1
0.9
0.8
0.7
0.6
0.5
0.4
0.3
0.2
0.1
0

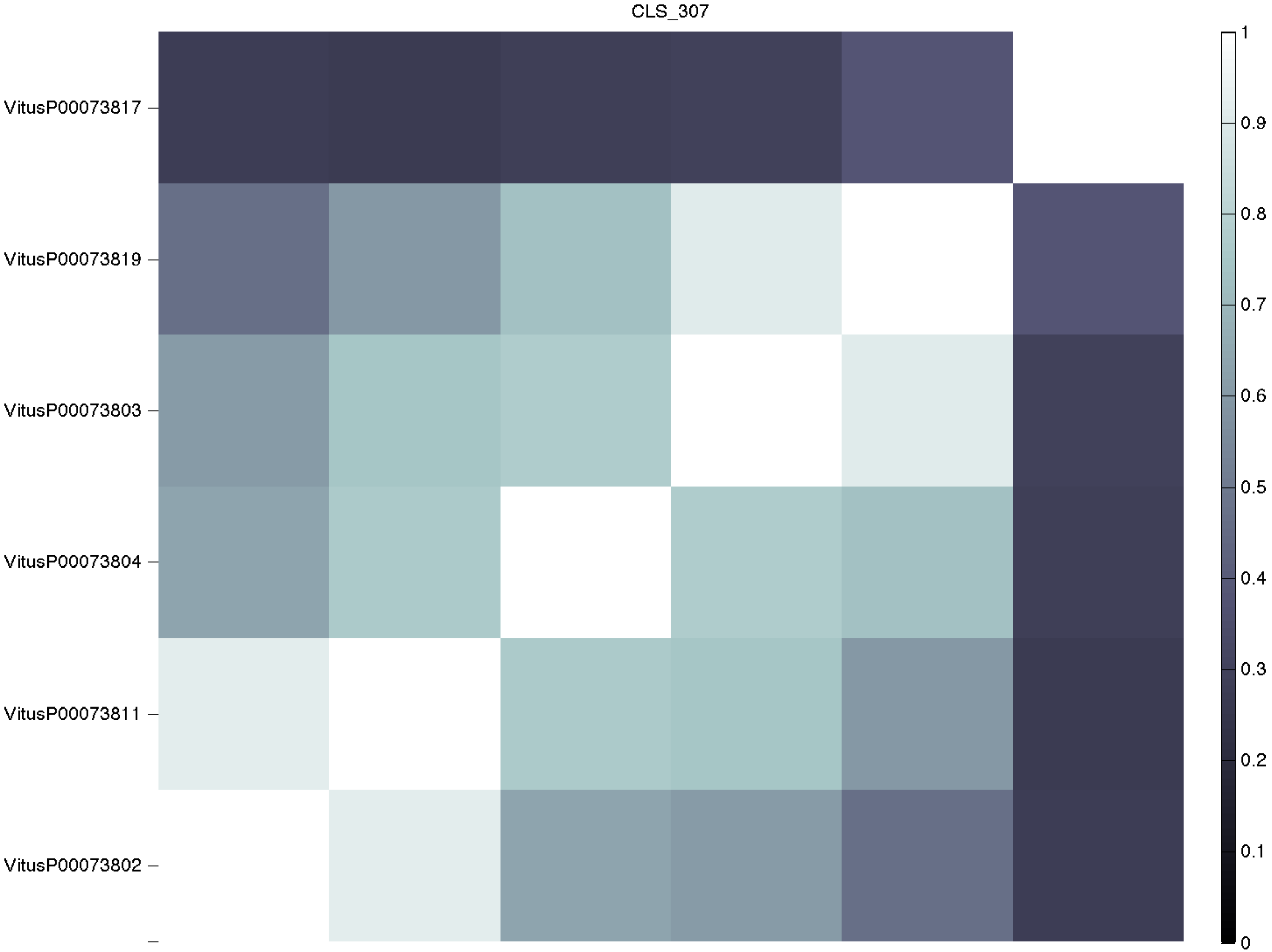

CLS_307
VitusP00073817
VitusP00073819
VitusP00073803
VitusP00073804
VitusP00073811
VitusP00073802
1
0.9
0.8
0.7
0.6
0.5
0.4
0.3
0.2
0.1
0

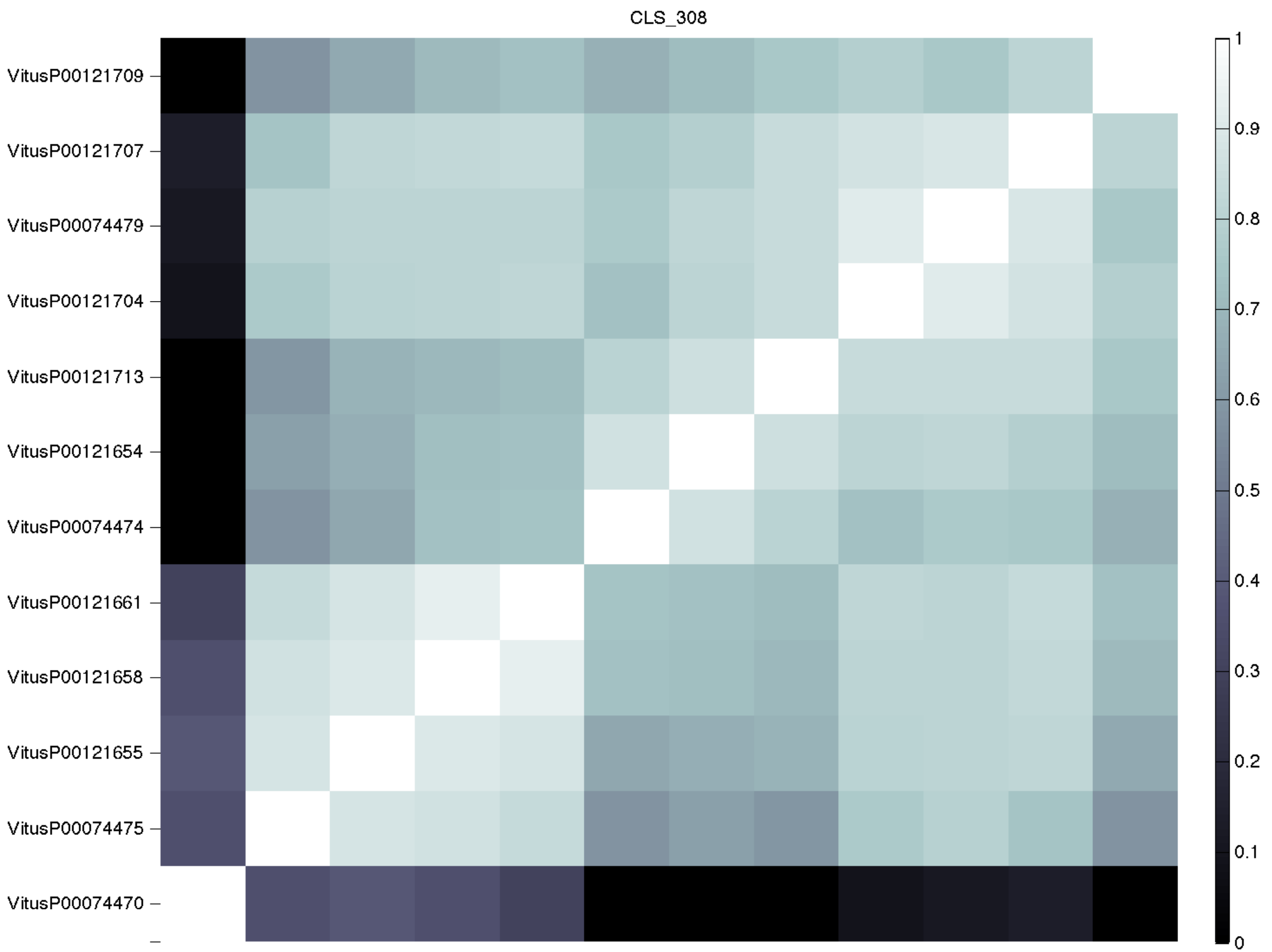

CLS_308
VitusP00121709
VitusP00121707
VitusP00074479
VitusP00121704
VitusP00121713
VitusP00121654
VitusP00074474
VitusP00121661
VitusP00121658
VitusP00121655
VitusP00074475
VitusP00074470
1
0.9
0.8
0.7
0.6
0.5
0.4
0.3
0.2
0.1
0

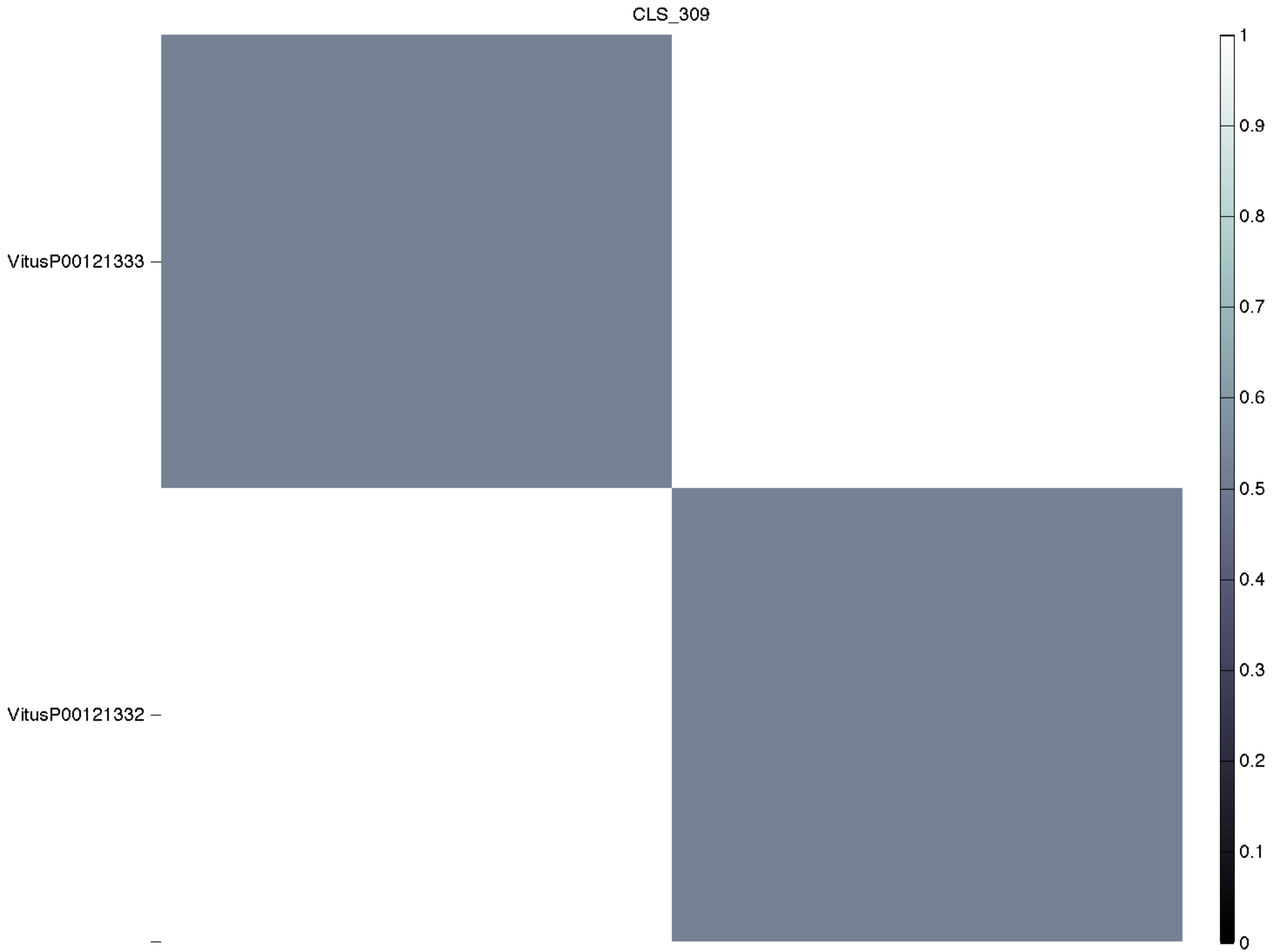

CLS_309
VitusP00121333
VitusP00121332
1
0.9
0.8
0.7
0.6
0.5
0.4
0.3
0.2
0.1
0

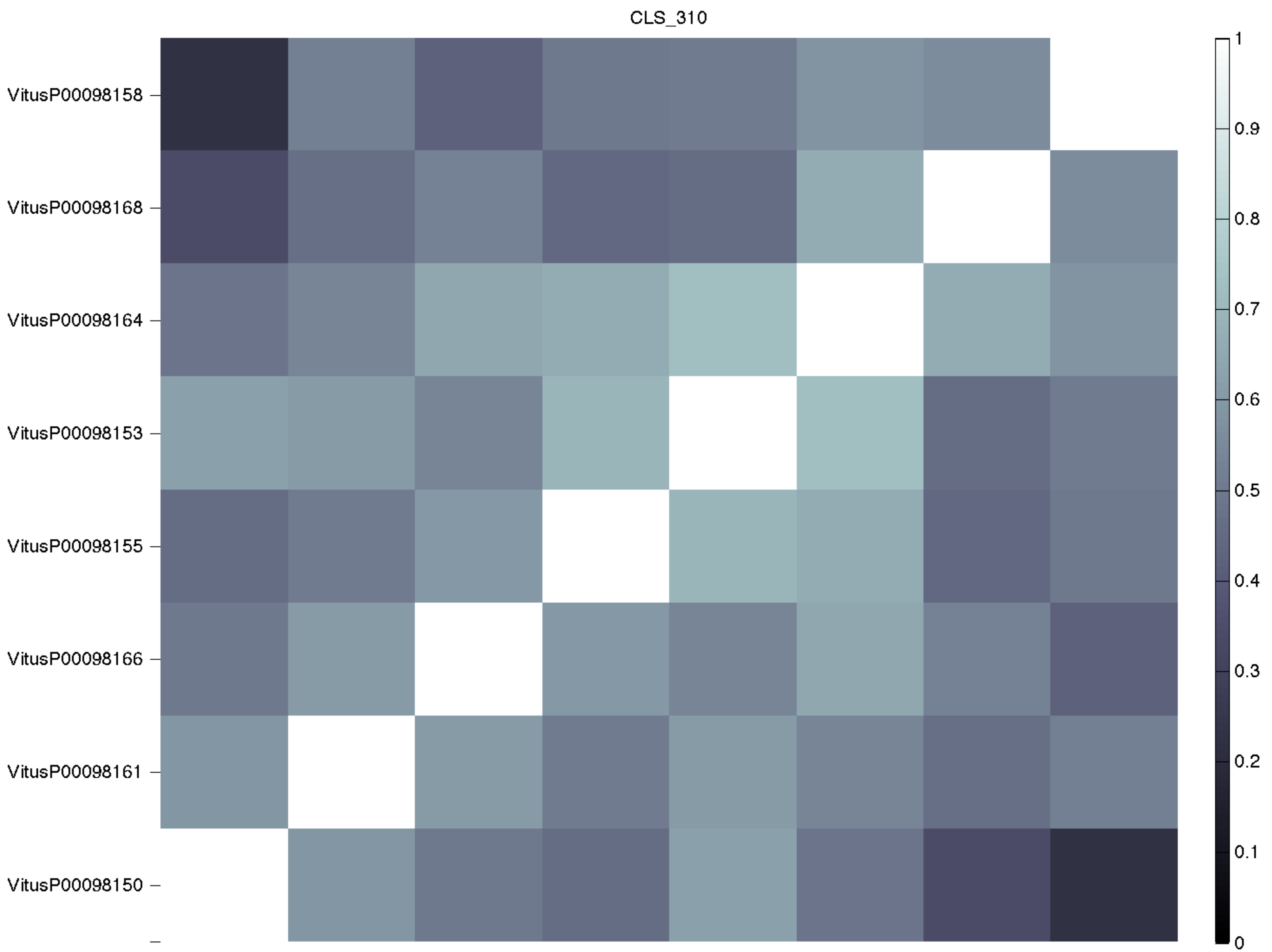
CLS_310
VitusP00098158
VitusP00098168
VitusP00098164
VitusP00098153
VitusP00098155
VitusP00098166
VitusP00098161
VitusP00098150
1
0.9
0.8
0.7
0.6
0.5
0.4
0.3
0.2
0.1
0

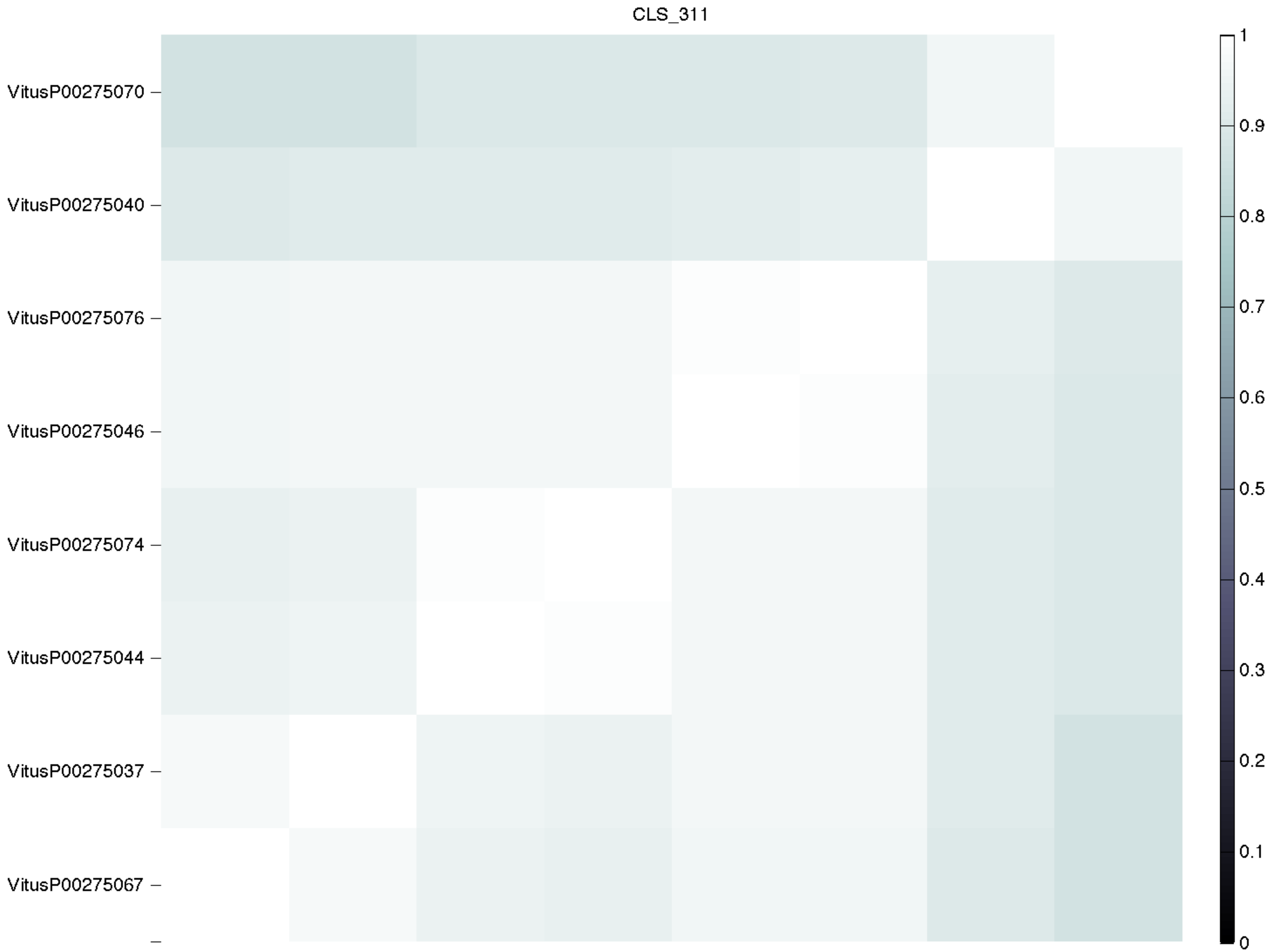

CLS_311
VitusP00275070
VitusP00275040
VitusP00275076
VitusP00275046
VitusP00275074
VitusP00275044
VitusP00275037
VitusP00275067
1
0.9
0.8
0.7
0.6
0.5
0.4
0.3
0.2
0.1
0

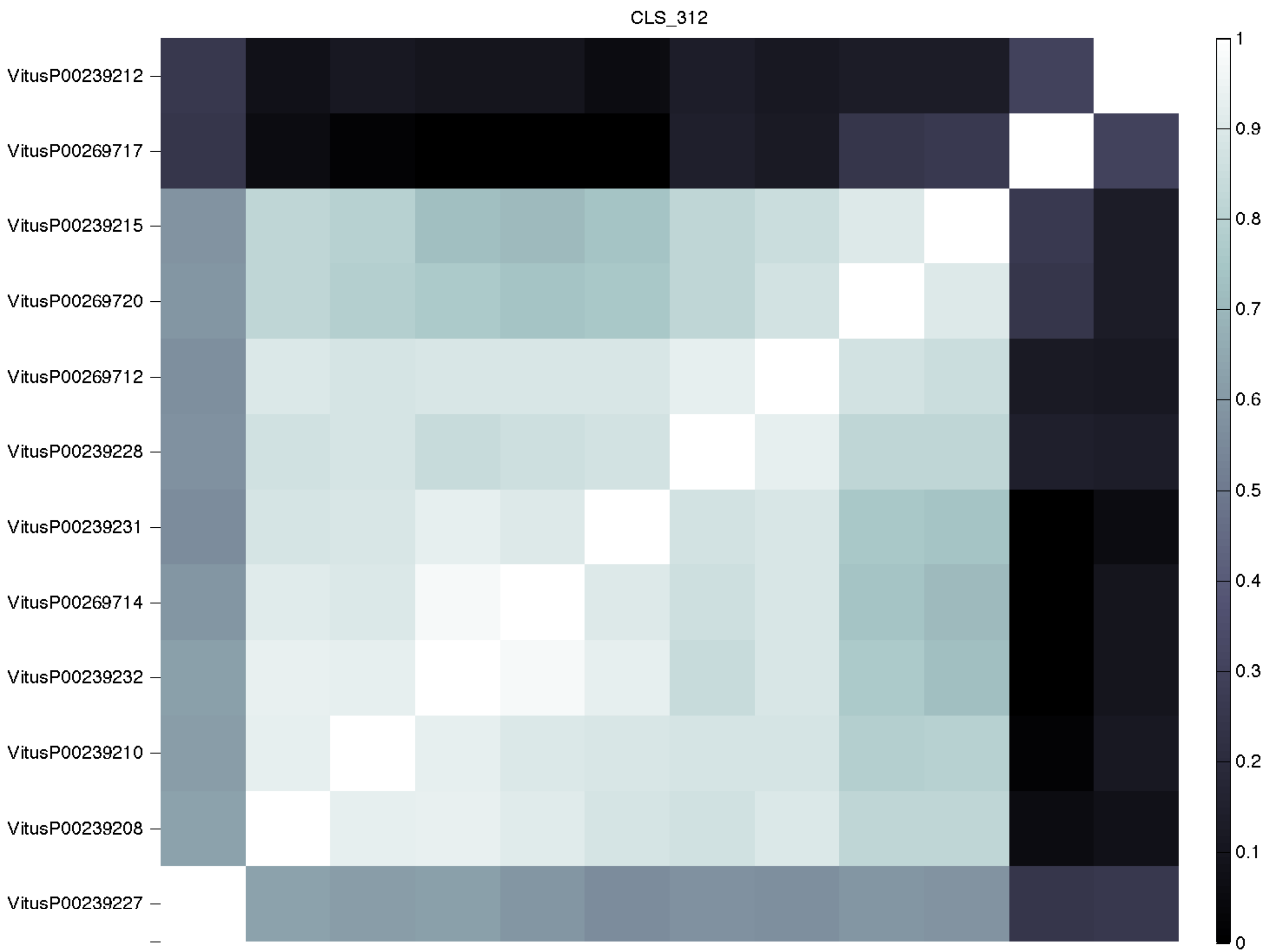
CLS_312
VitusP00239212
VitusP00269717
VitusP00239215
VitusP00269720
VitusP00269712
VitusP00239228
VitusP00239231
VitusP00269714
VitusP00239232
VitusP00239210
VitusP00239208
VitusP00239227
1
0.9
0.8
0.7
0.6
0.5
0.4
0.3
0.2
0.1
0

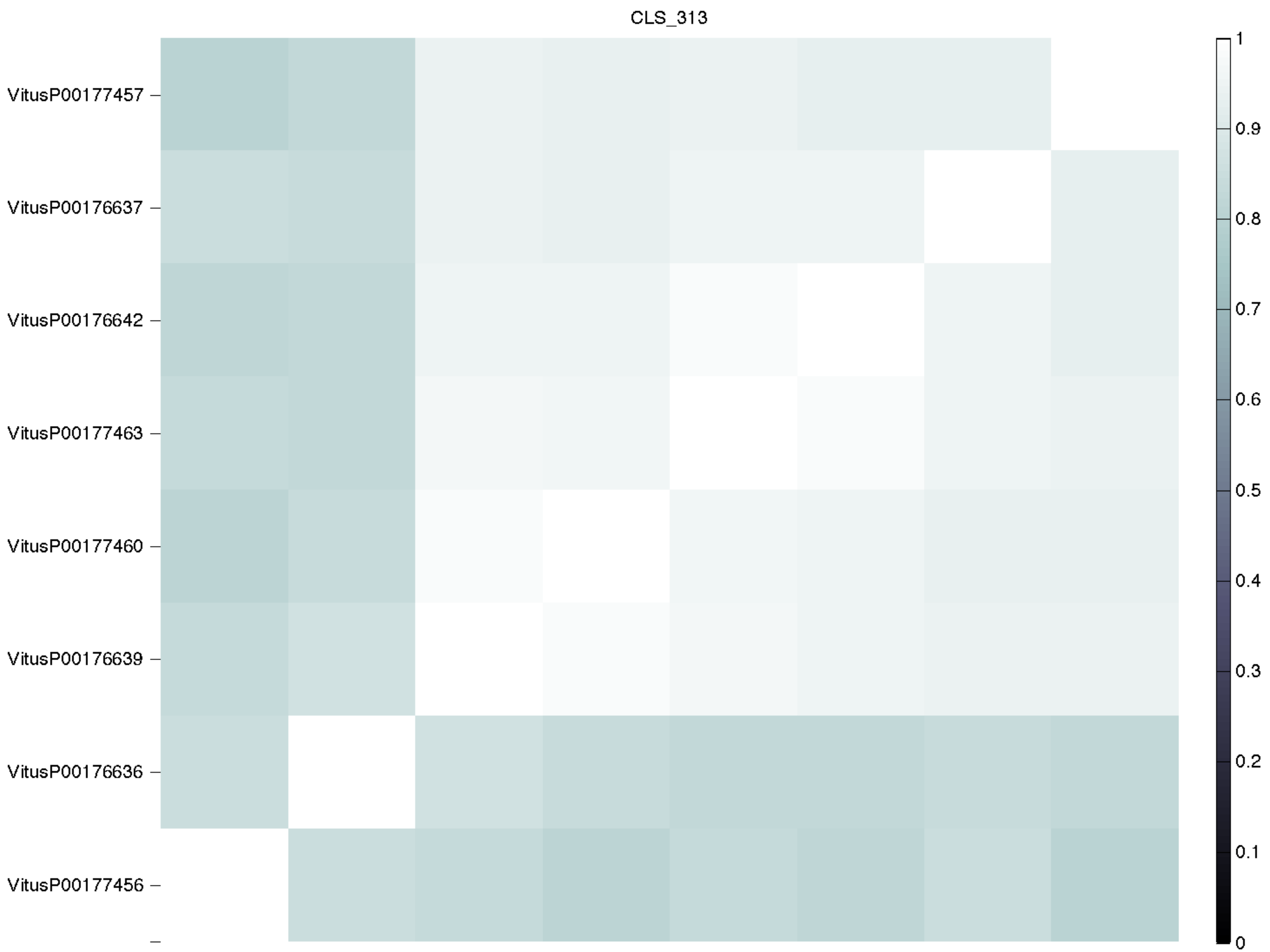
CLS_313
VitusP00177457
VitusP00176637
VitusP00176642
VitusP00177463
VitusP00177460
VitusP00176639
VitusP00176636
VitusP00177456
1
0.9
0.8
0.7
0.6
0.5
0.4
0.3
0.2
0.1
0

CLS_314

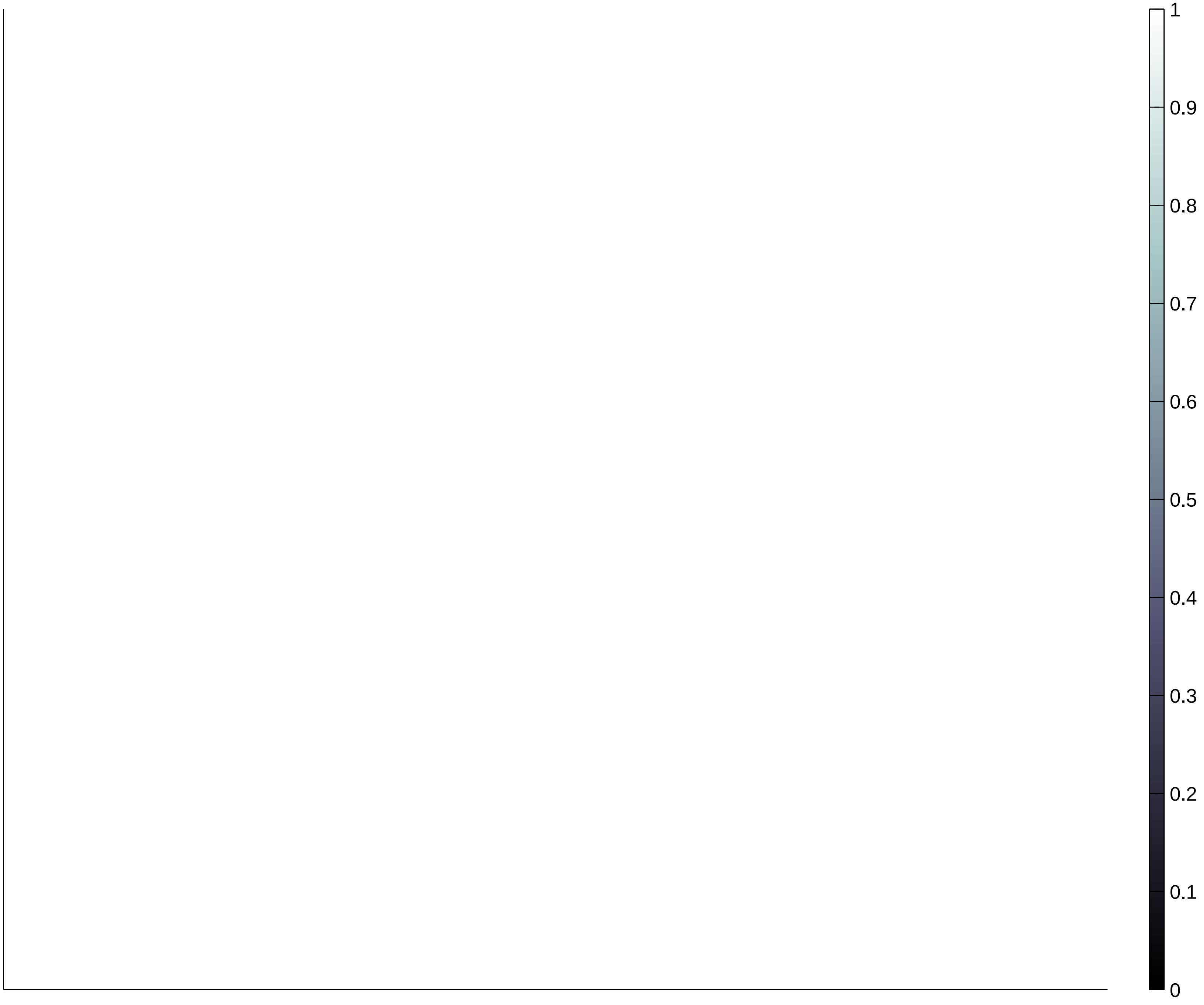

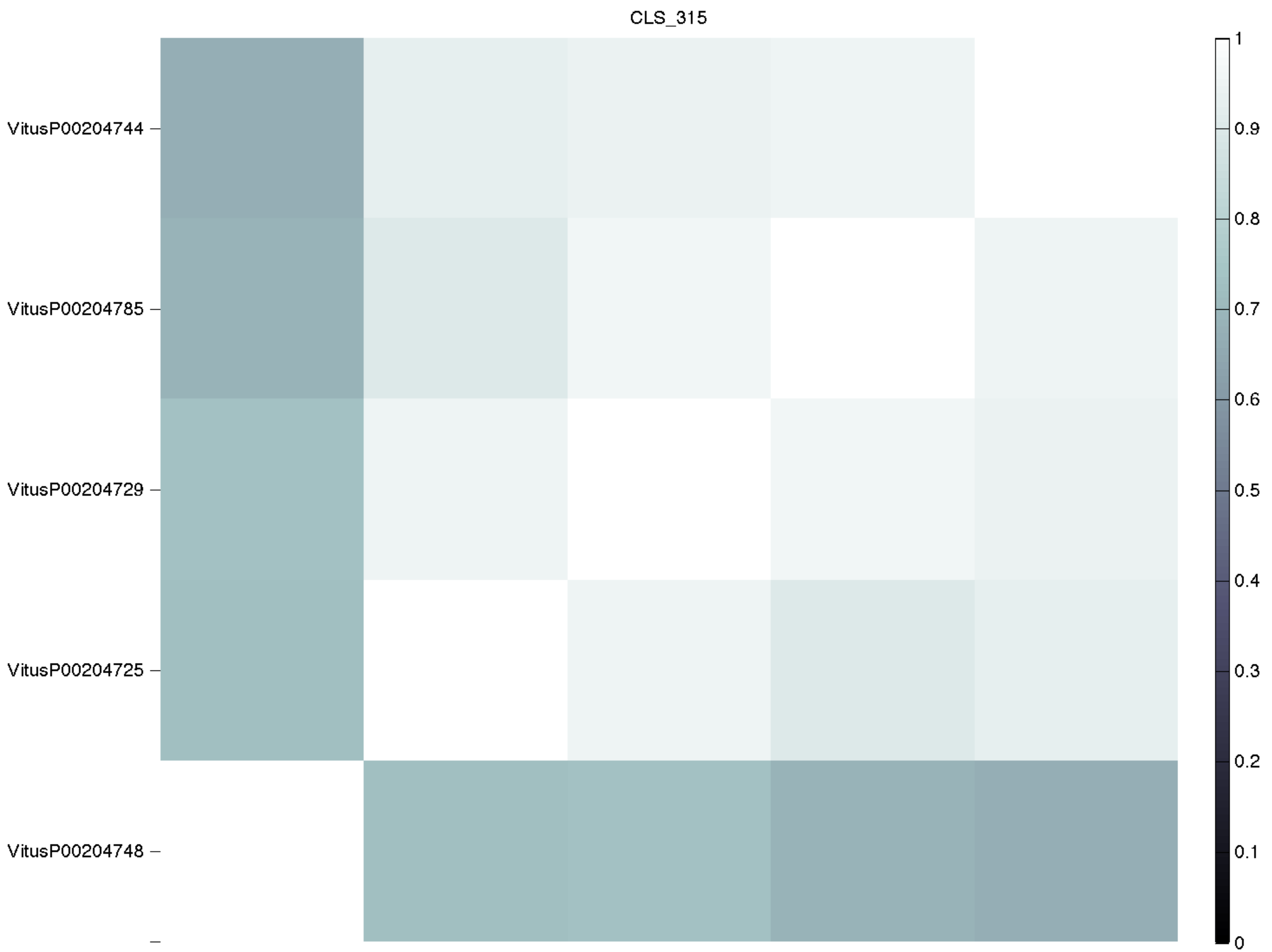
CLS_315
VitusP00204744
VitusP00204785
VitusP00204729
VitusP00204725
VitusP00204748
1
0.9
0.8
0.7
0.6
0.5
0.4
0.3
0.2
0.1
0

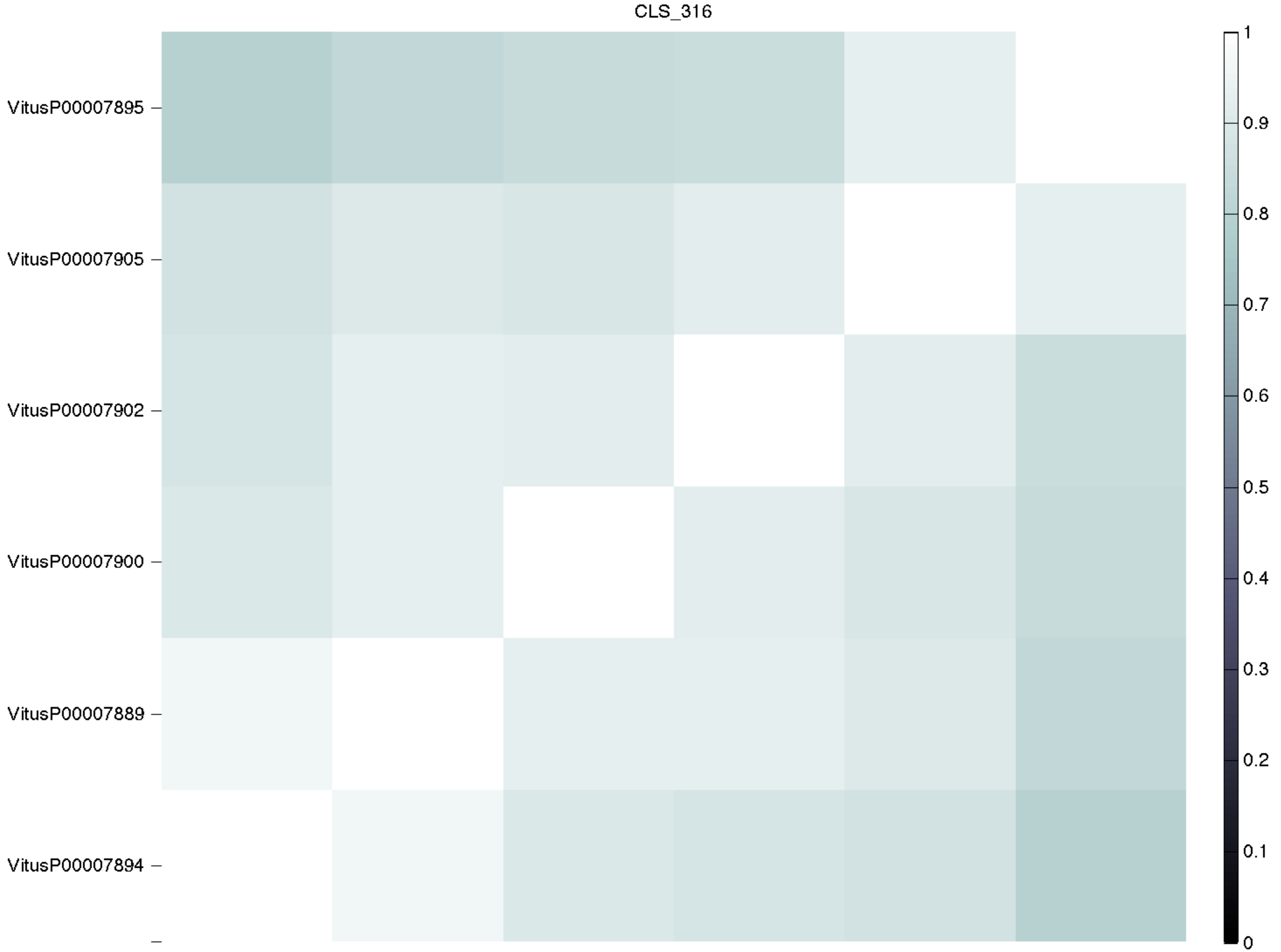
CLS_316
VitusP00007895
VitusP00007905
VitusP00007902
VitusP00007900
VitusP00007889
VitusP00007894
1
0.9
0.8
0.7
0.6
0.5
0.4
0.3
0.2
0.1
0

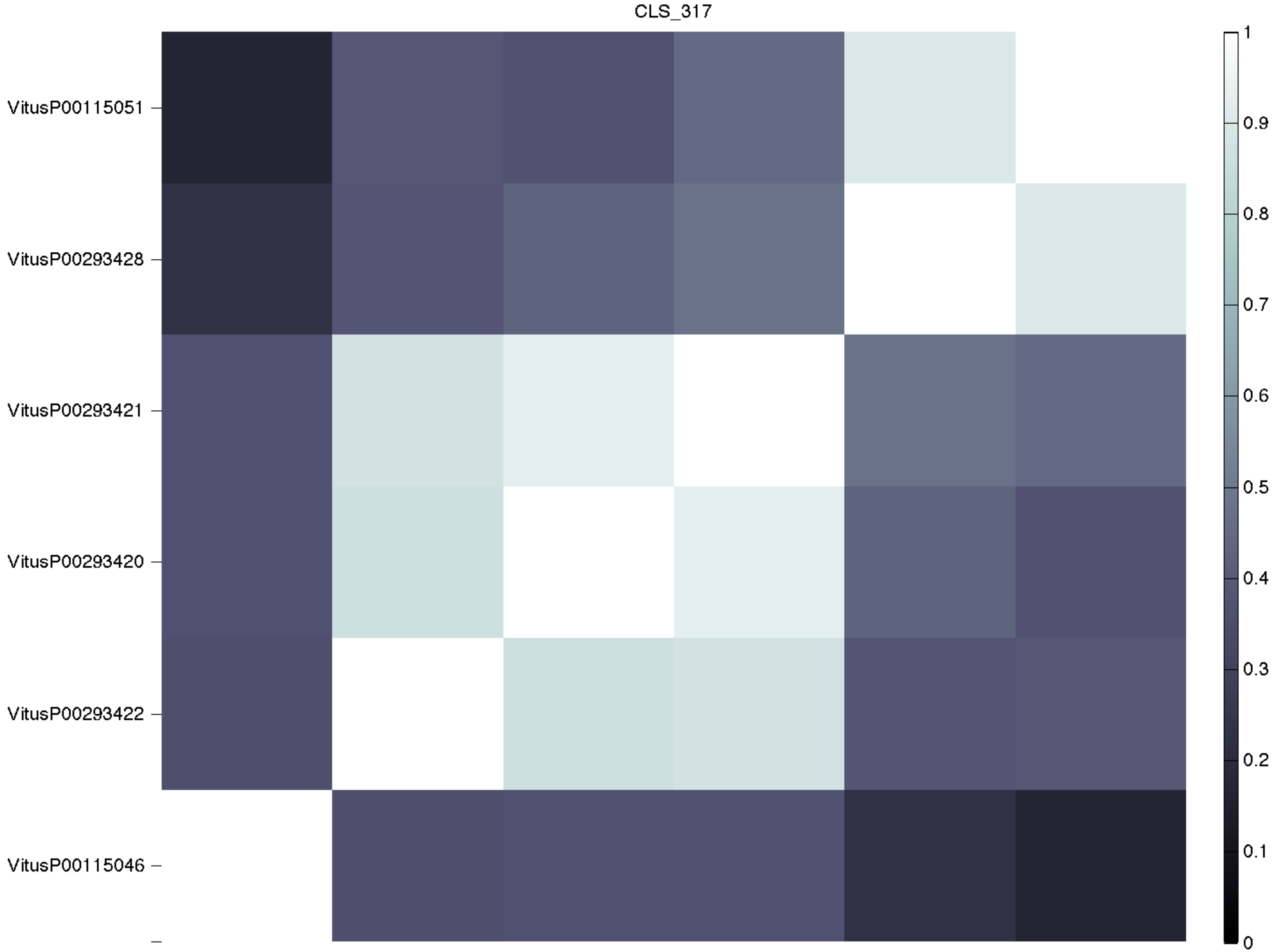

CLS_317
VitusP00115051
VitusP00293428
VitusP00293421
VitusP00293420
VitusP00293422
VitusP00115046
1
0.9
0.8
0.7
0.6
0.5
0.4
0.3
0.2
0.1
0

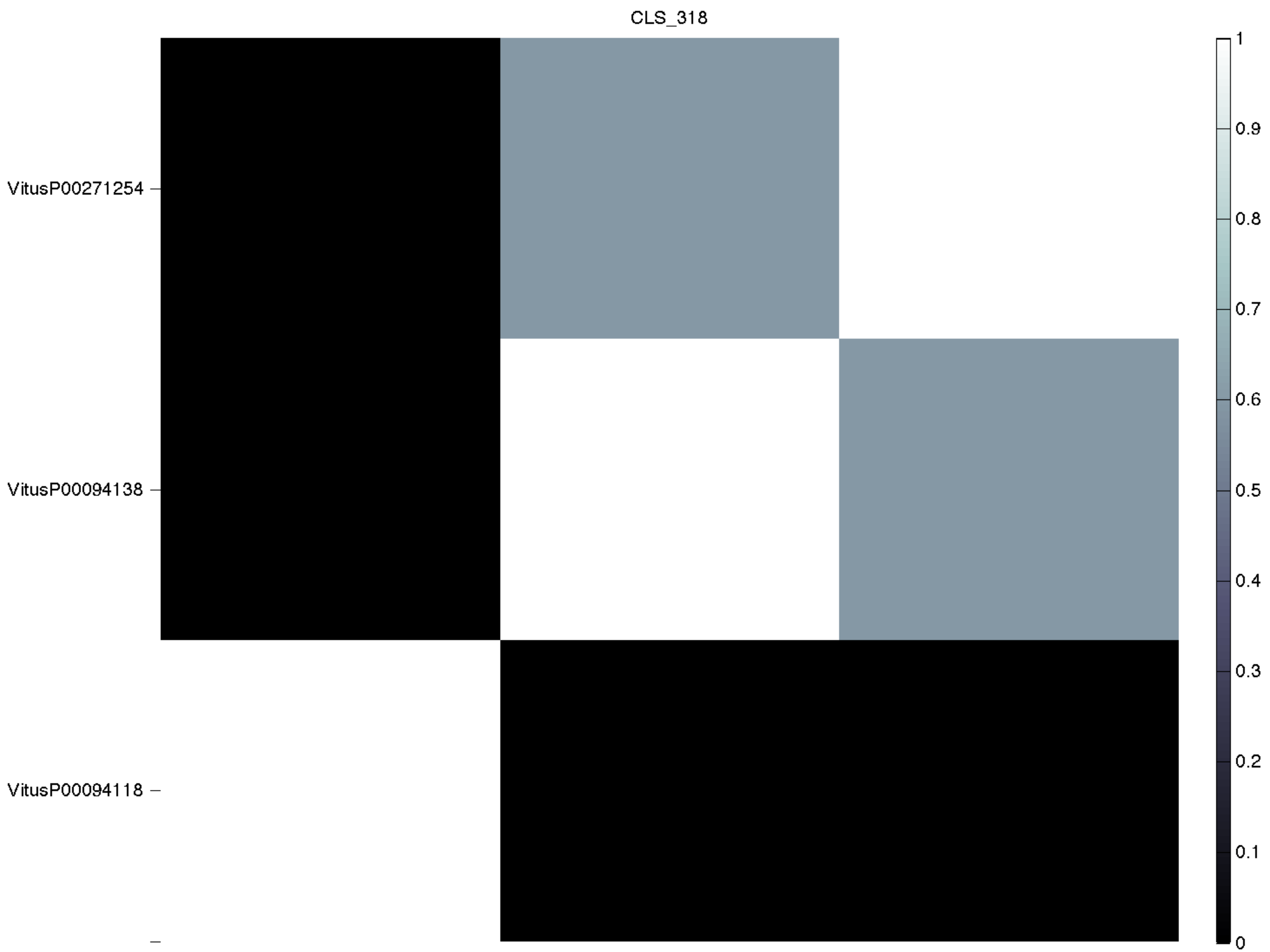
CLS_318
VitusP00271254
VitusP00094138
VitusP00094118
1
0.9
0.8
0.7
0.6
0.5
0.4
0.3
0.2
0.1
0

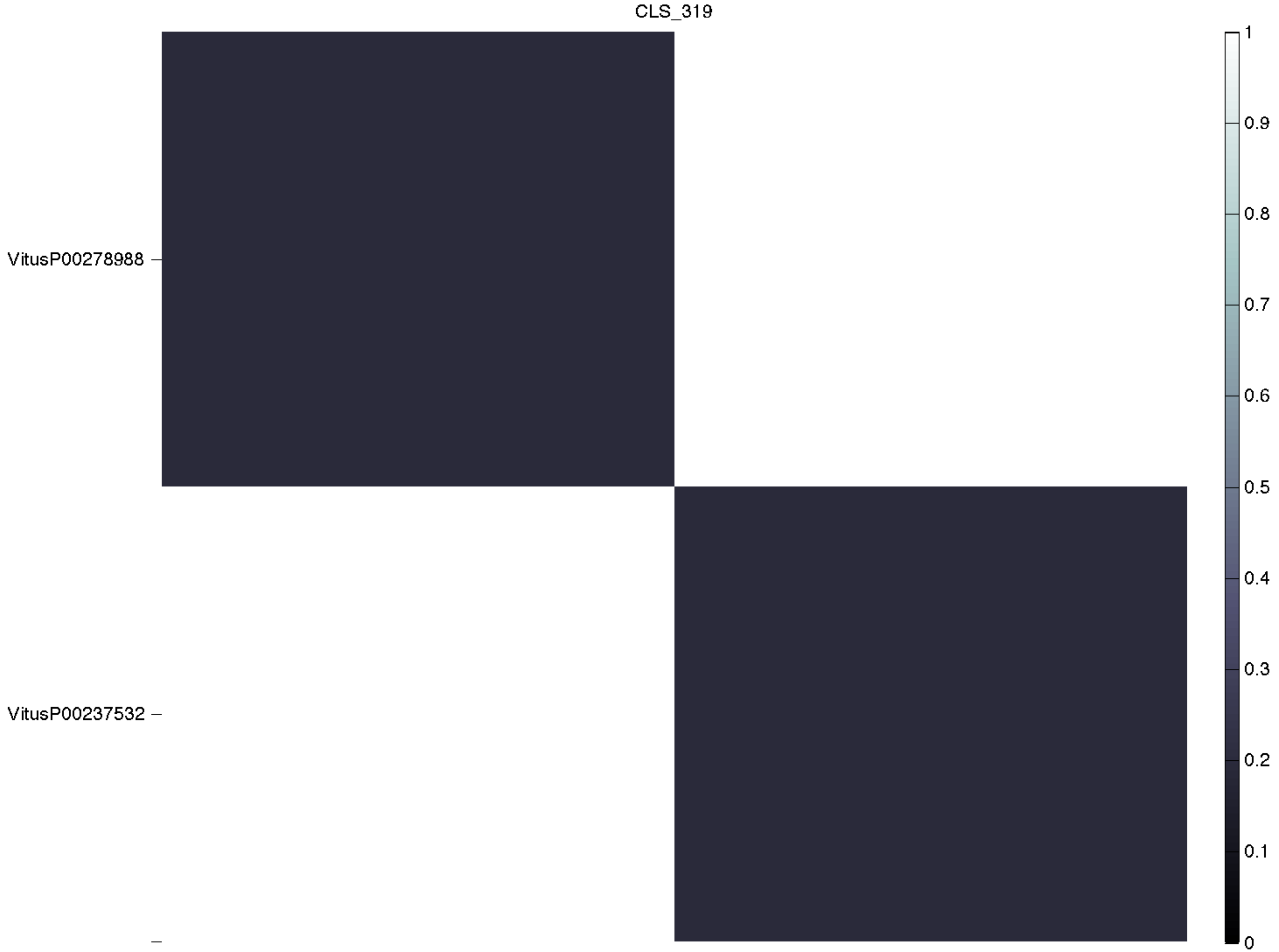
CLS_319
VitusP00278988
VitusP00237532
1
0.9
0.8
0.7
0.6
0.5
0.4
0.3
0.2
0.1
0

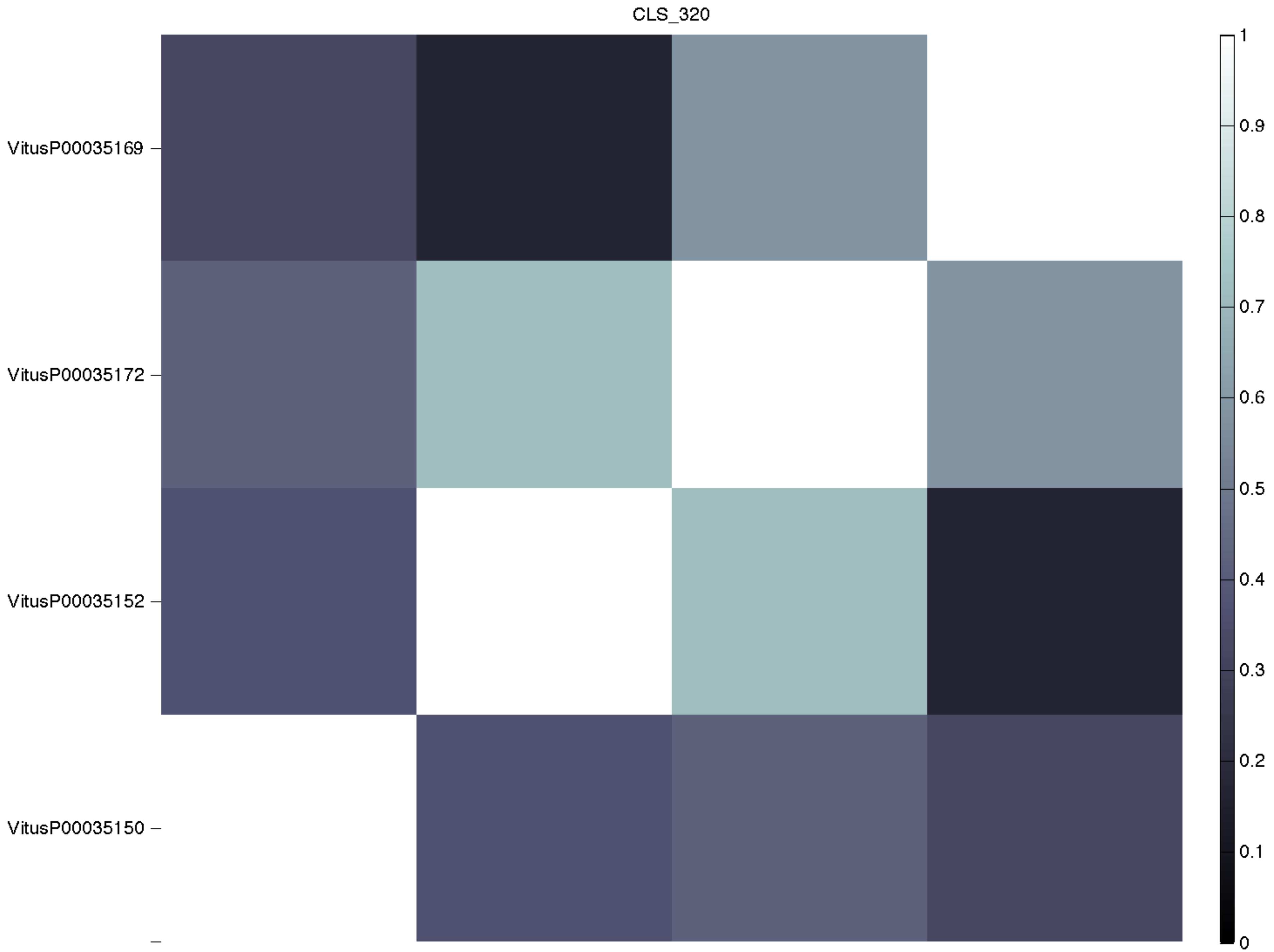

CLS_320
VitusP00035169
VitusP00035172
VitusP00035152
VitusP00035150
1
0.9
0.8
0.7
0.6
0.5
0.4
0.3
0.2
0.1
0

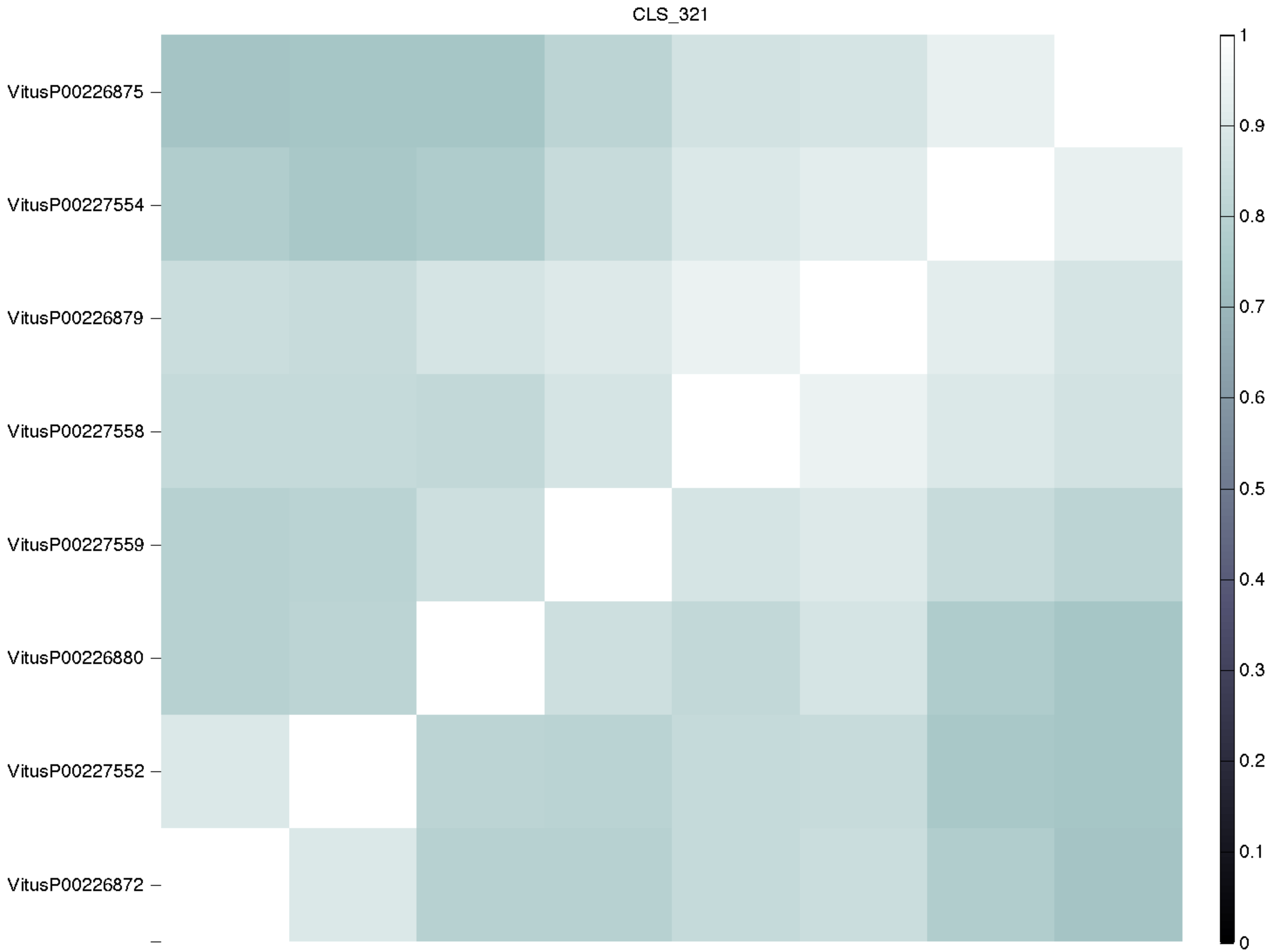
CLS_321
VitusP00226875
VitusP00227554
VitusP00226879
VitusP00227558
VitusP00227559
VitusP00226880
VitusP00227552
VitusP00226872
1
0.9
0.8
0.7
0.6
0.5
0.4
0.3
0.2
0.1
0

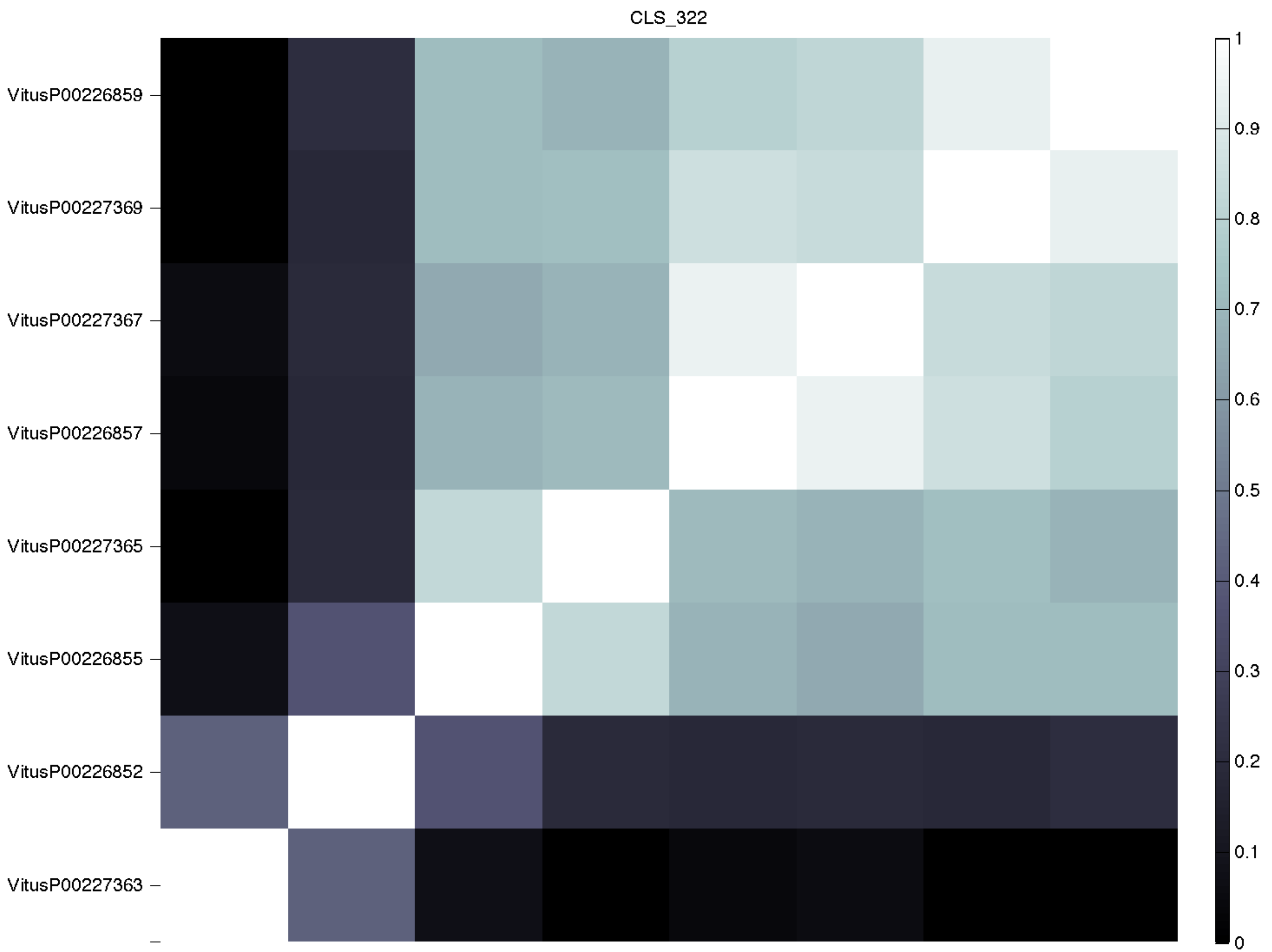
CLS_322
VitusP00226859
VitusP00227369
VitusP00227367
VitusP00226857
VitusP00227365
VitusP00226855
VitusP00226852
VitusP00227363
1
0.9
0.8
0.7
0.6
0.5
0.4
0.3
0.2
0.1
0

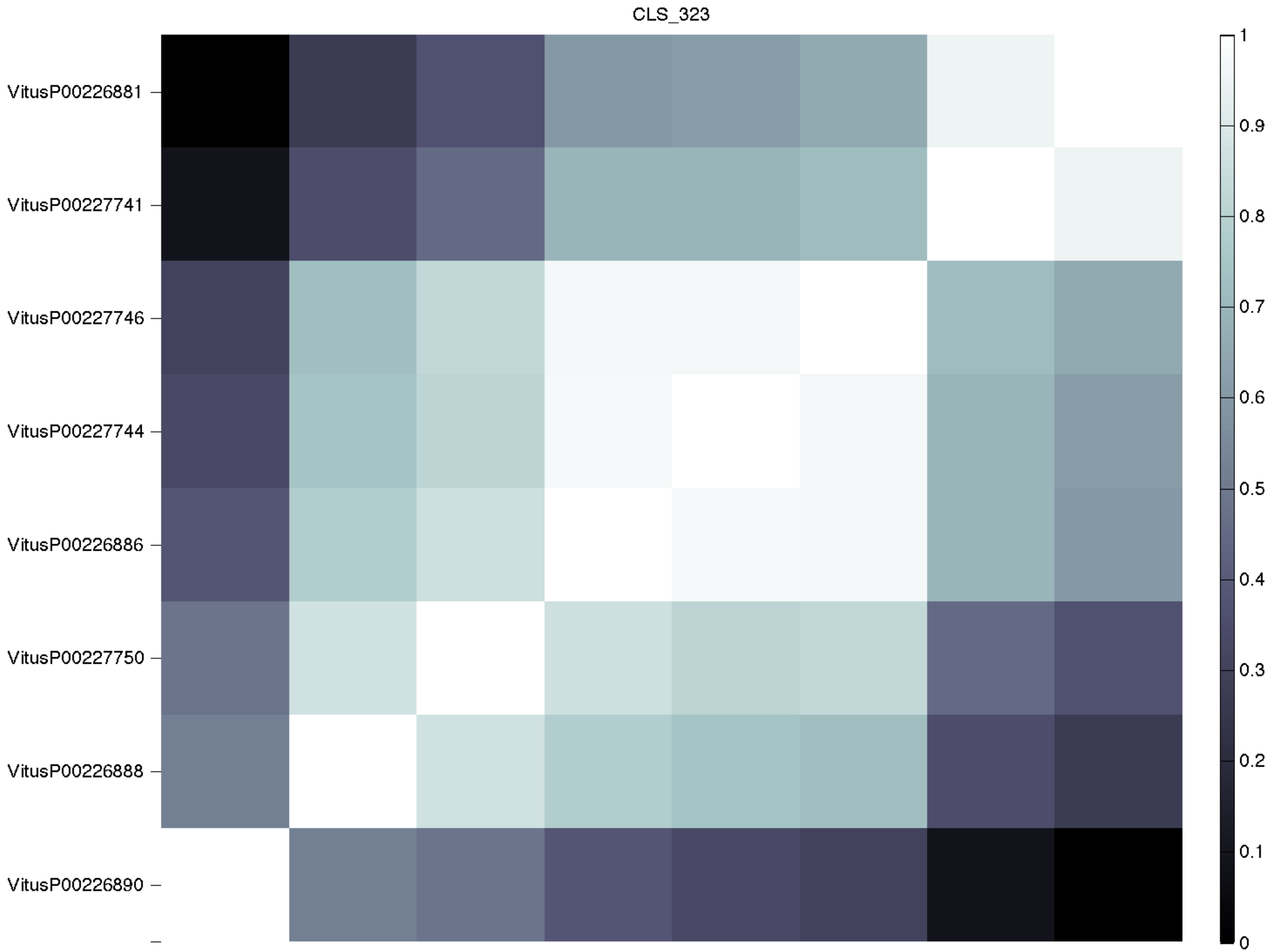
CLS_323
VitusP00226881
VitusP00227741
VitusP00227746
VitusP00227744
VitusP00226886
VitusP00227750
VitusP00226888
VitusP00226890
1
0.9
0.8
0.7
0.6
0.5
0.4
0.3
0.2
0.1
0

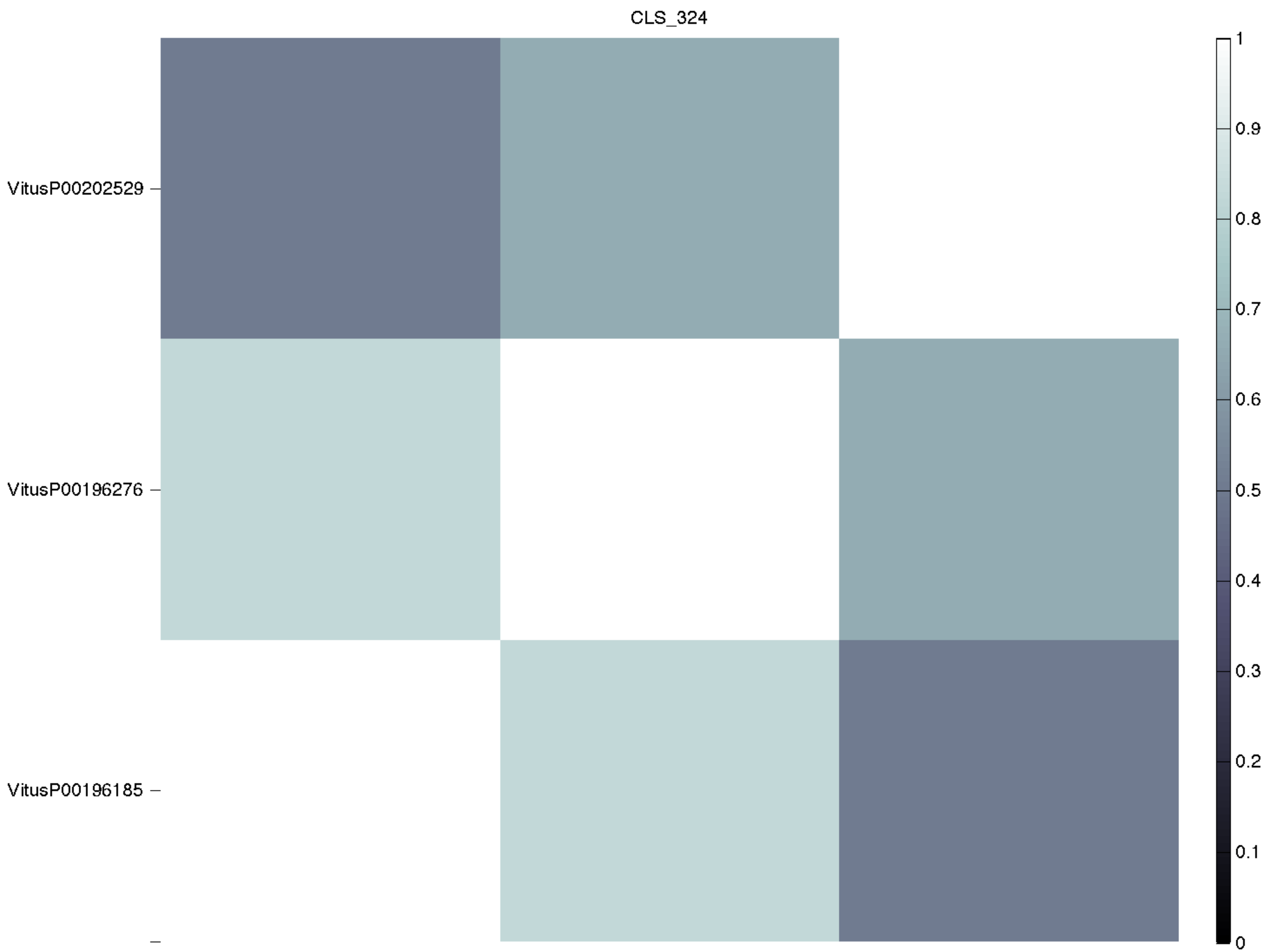

CLS_324
VitusP00202529
VitusP00196276
VitusP00196185
1
0.9
0.8
0.7
0.6
0.5
0.4
0.3
0.2
0.1
0

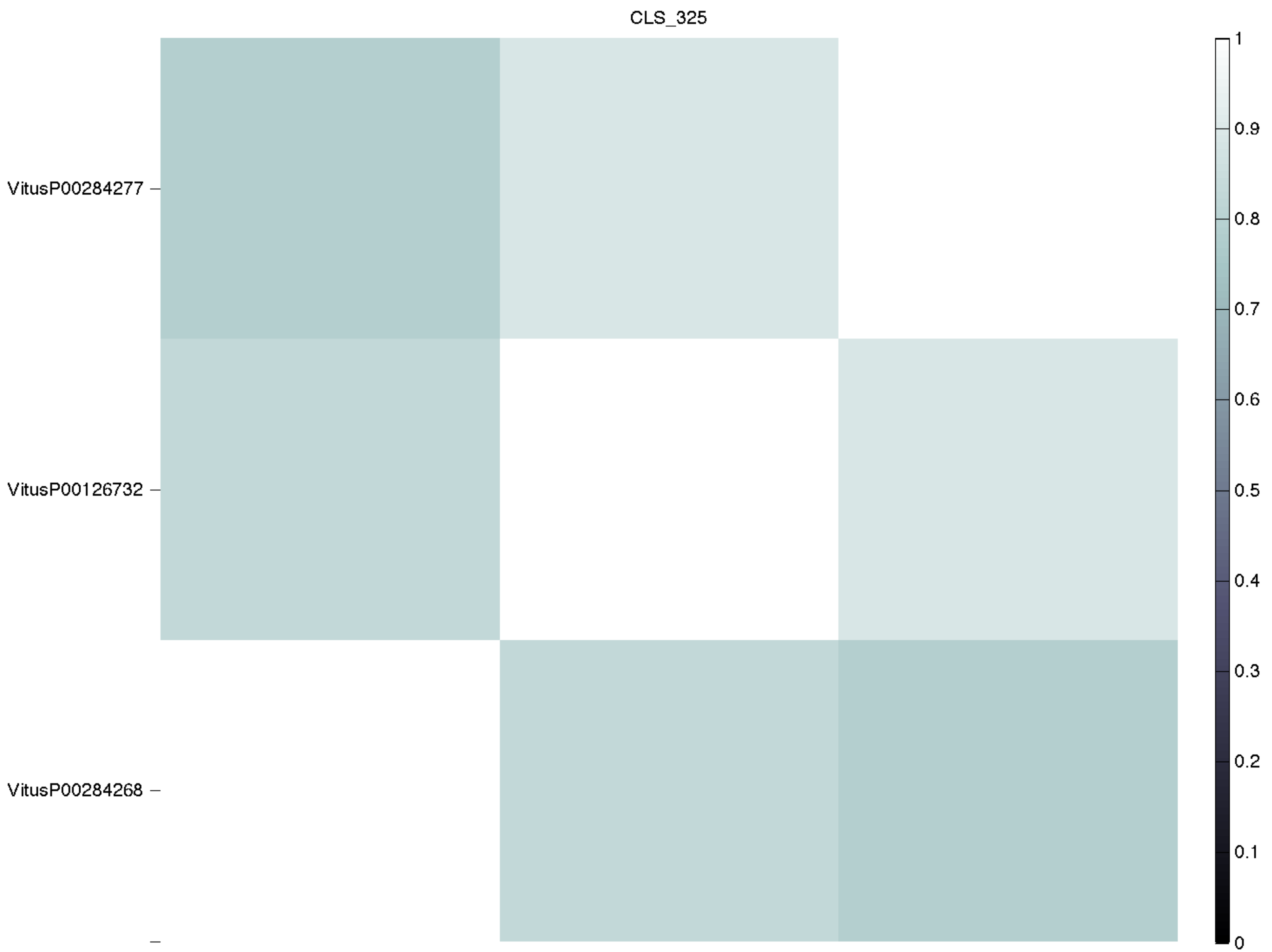
CLS_325
VitusP00284277
VitusP00126732
VitusP00284268
1
0.9
0.8
0.7
0.6
0.5
0.4
0.3
0.2
0.1
0

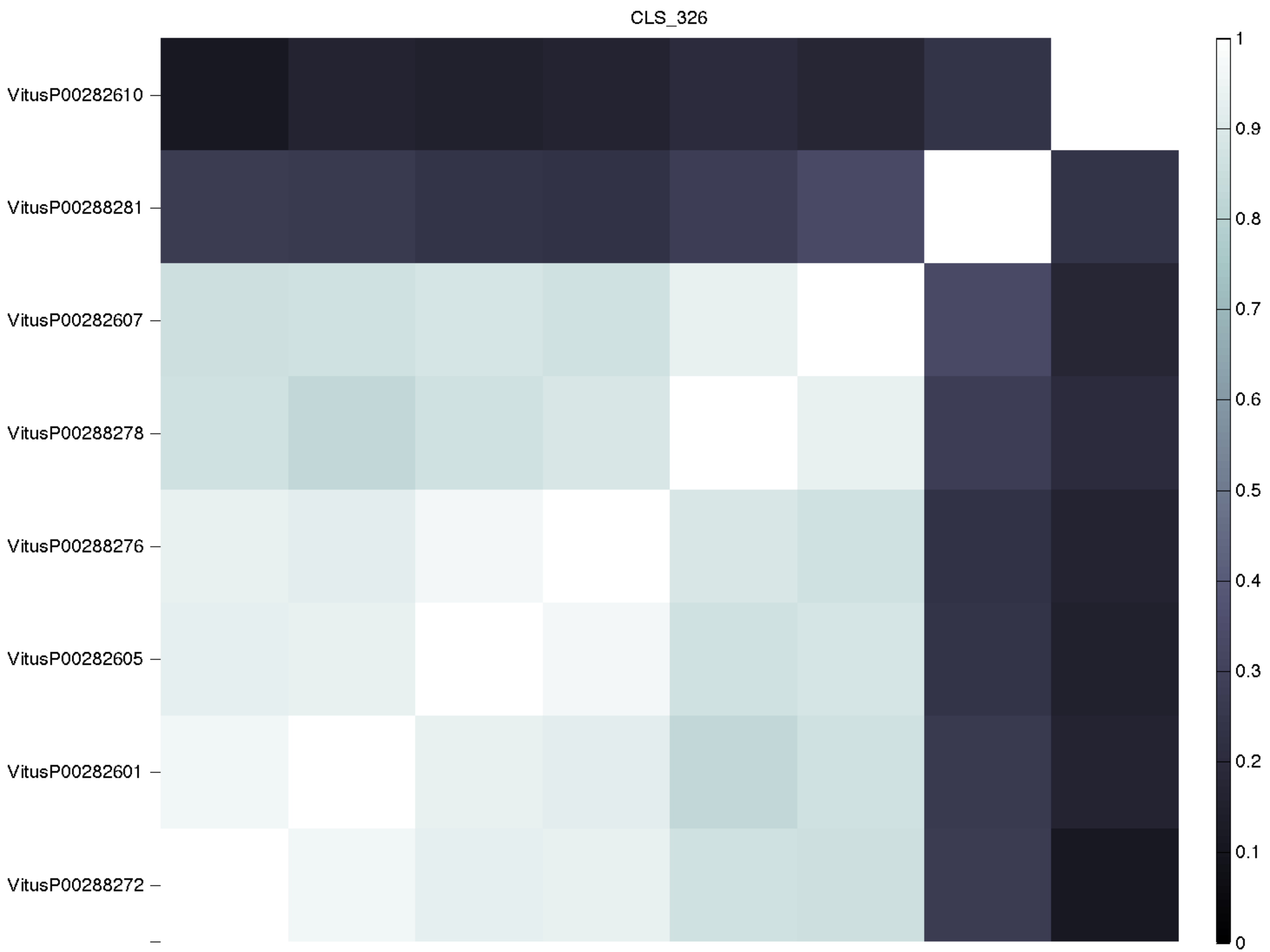
CLS_326
VitusP00282610
VitusP00288281
VitusP00282607
VitusP00288278
VitusP00288276
VitusP00282605
VitusP00282601
VitusP00288272
1
0.9
0.8
0.7
0.6
0.5
0.4
0.3
0.2
0.1
0

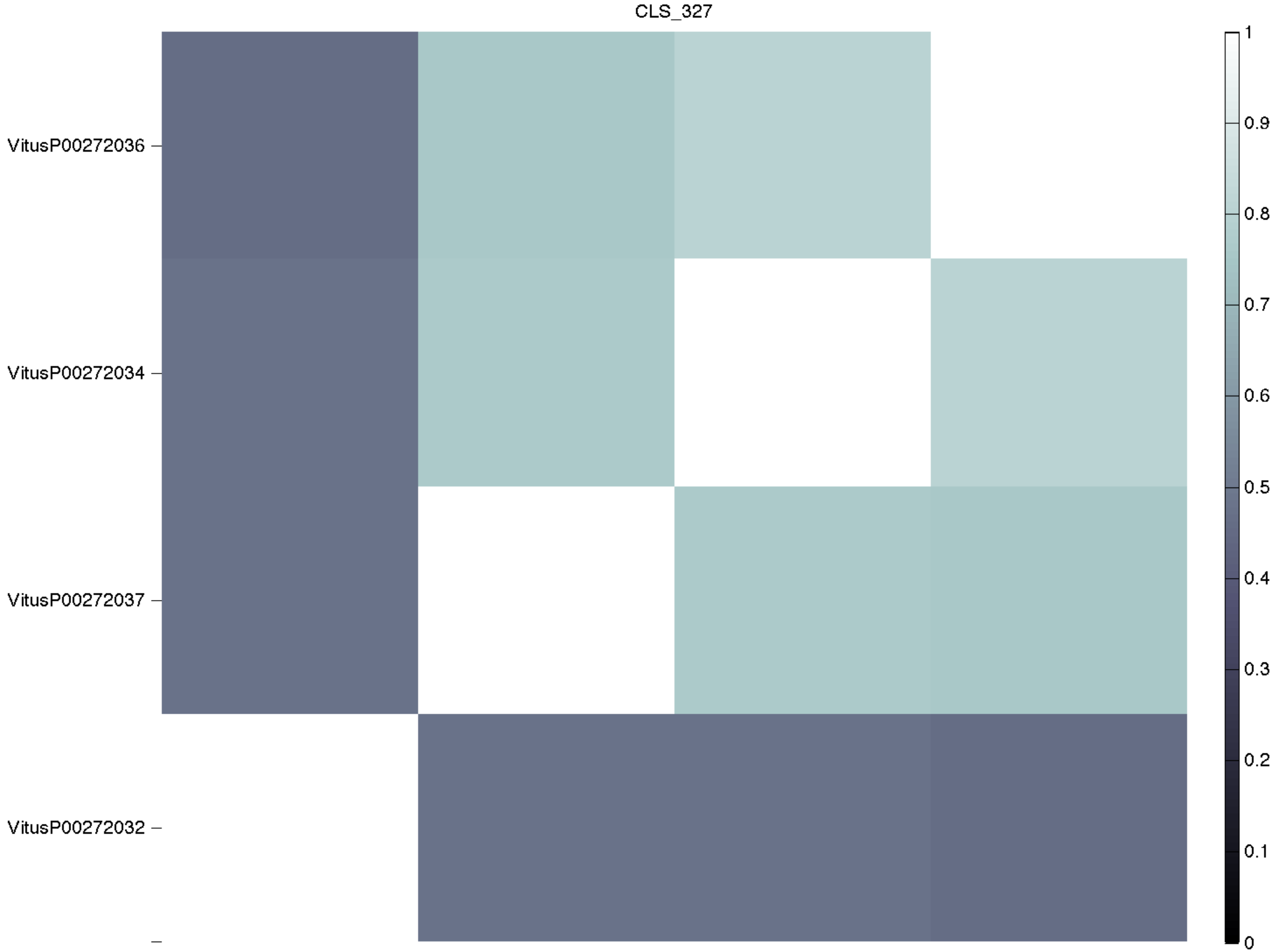
CLS_327
VitusP00272036
VitusP00272034
VitusP00272037
VitusP00272032
1
0.9
0.8
0.7
0.6
0.5
0.4
0.3
0.2
0.1
0

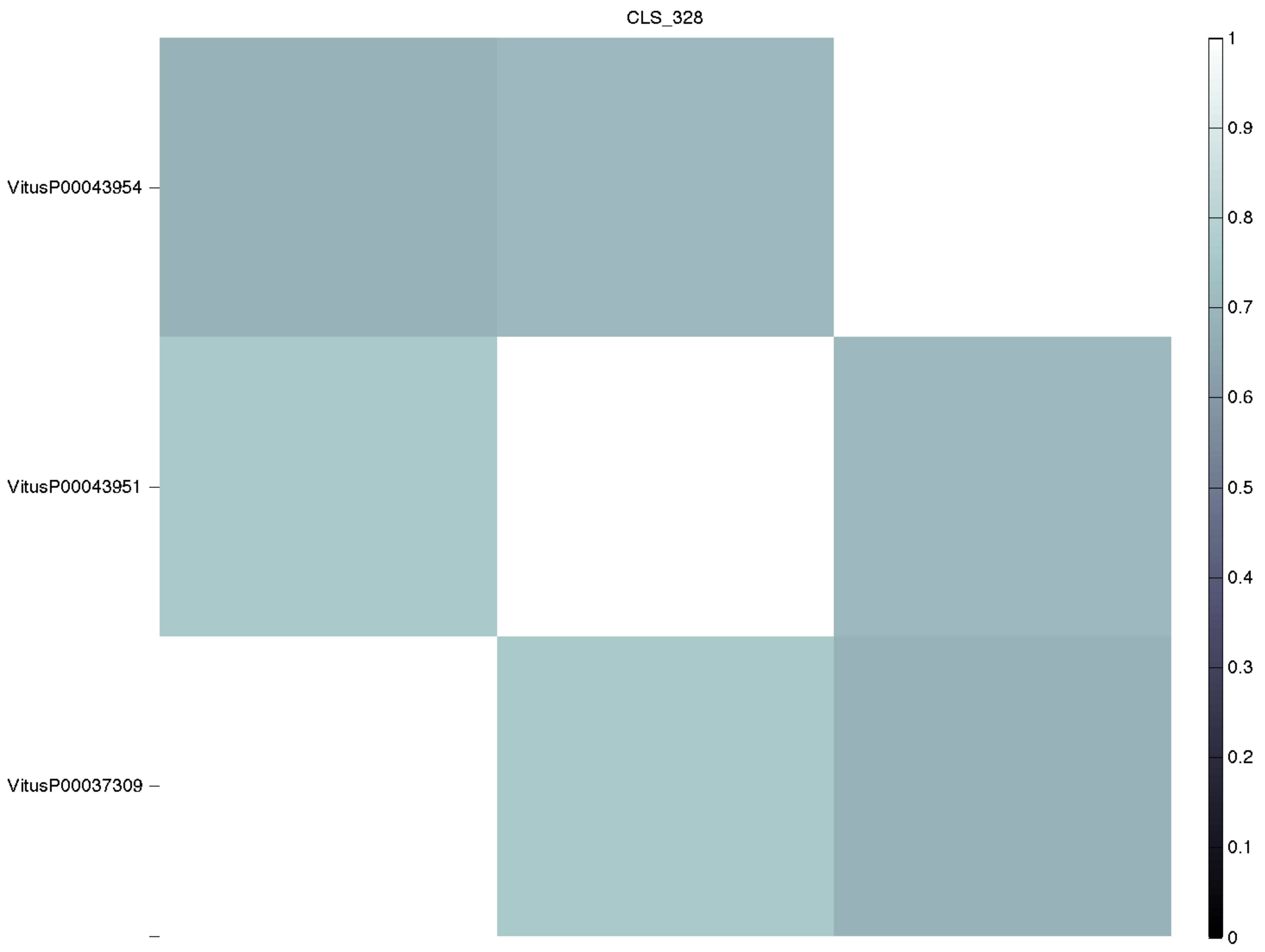
CLS_328
VitusP00043954
VitusP00043951
VitusP00037309
1
0.9
0.8
0.7
0.6
0.5
0.4
0.3
0.2
0.1
0

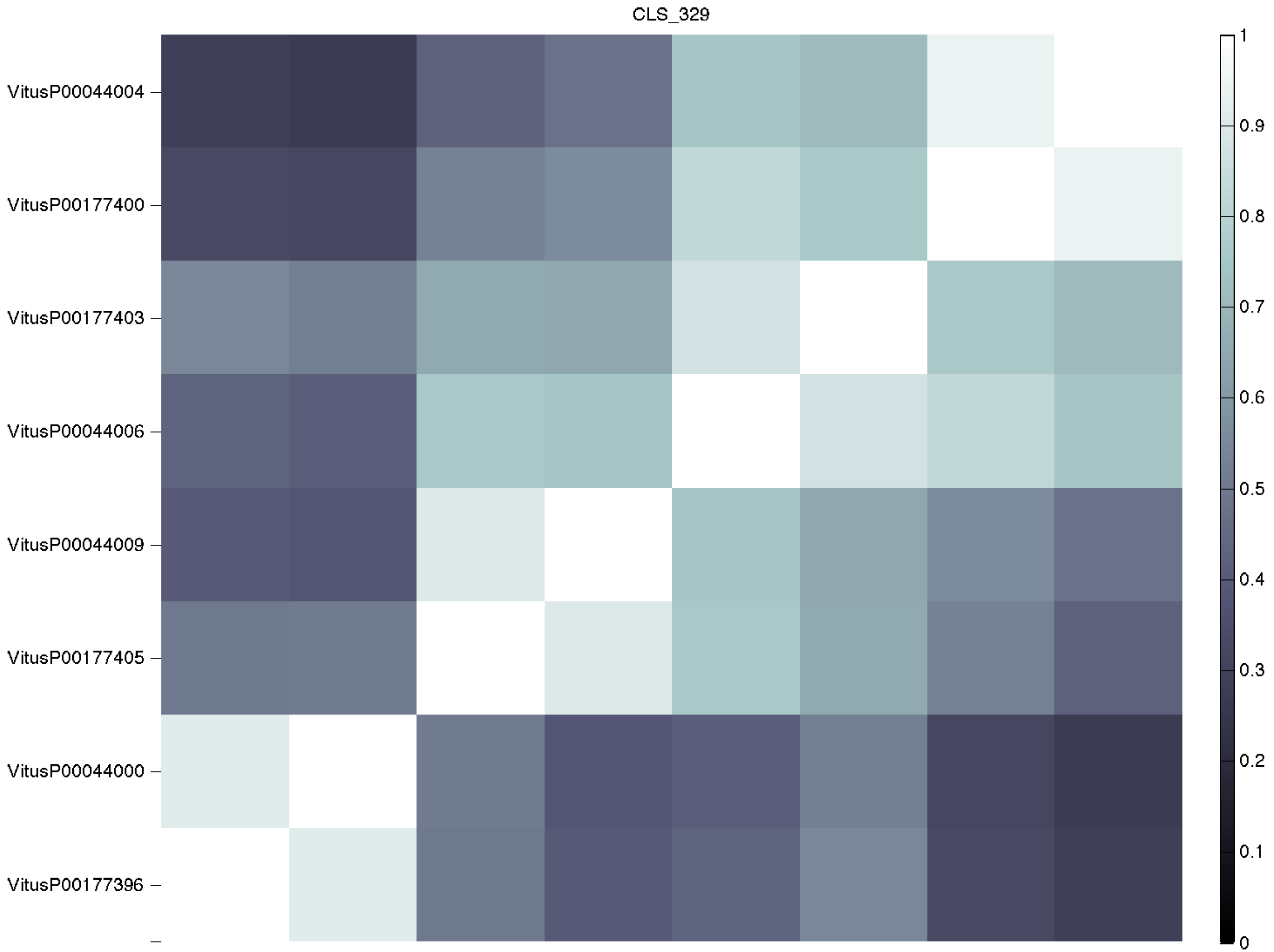
CLS_329
VitusP00044004
VitusP00177400
VitusP00177403
VitusP00044006
VitusP00044009
VitusP00177405
VitusP00044000
VitusP00177396
1
0.9
0.8
0.7
0.6
0.5
0.4
0.3
0.2
0.1
0

CLS_330

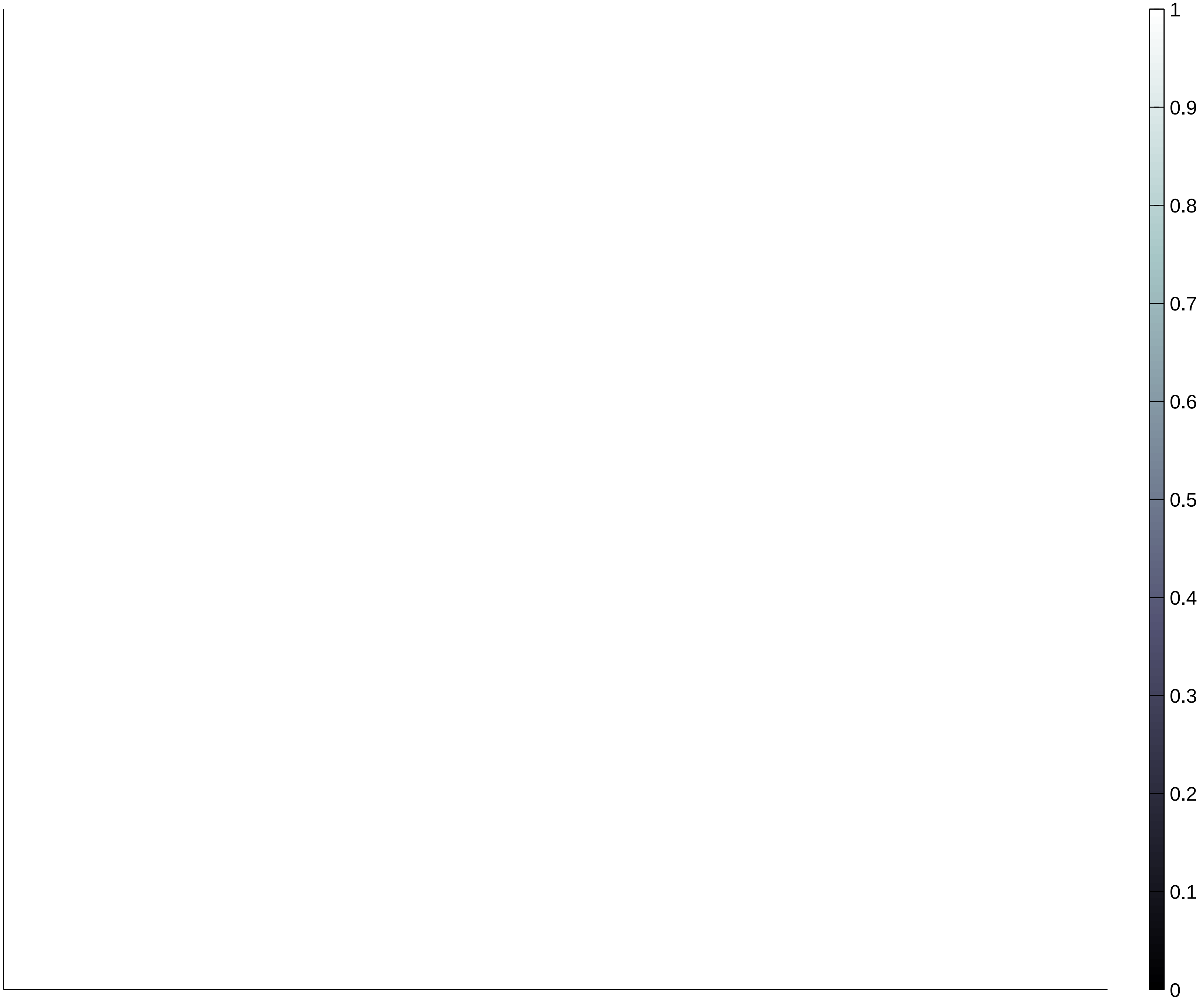

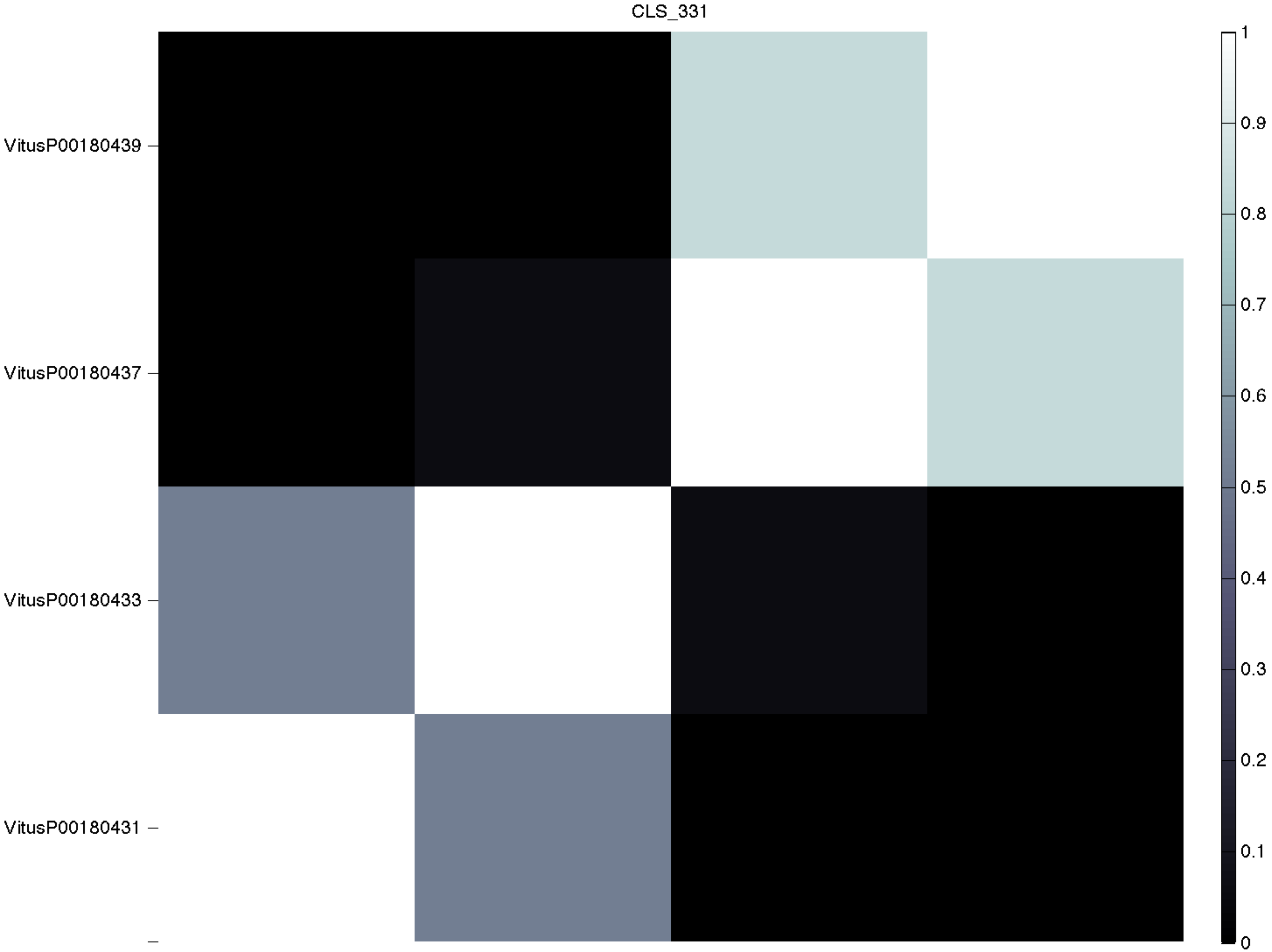
CLS_331
VitusP00180439
VitusP00180437
VitusP00180433
VitusP00180431
1
0.9
0.8
0.7
0.6
0.5
0.4
0.3
0.2
0.1
0

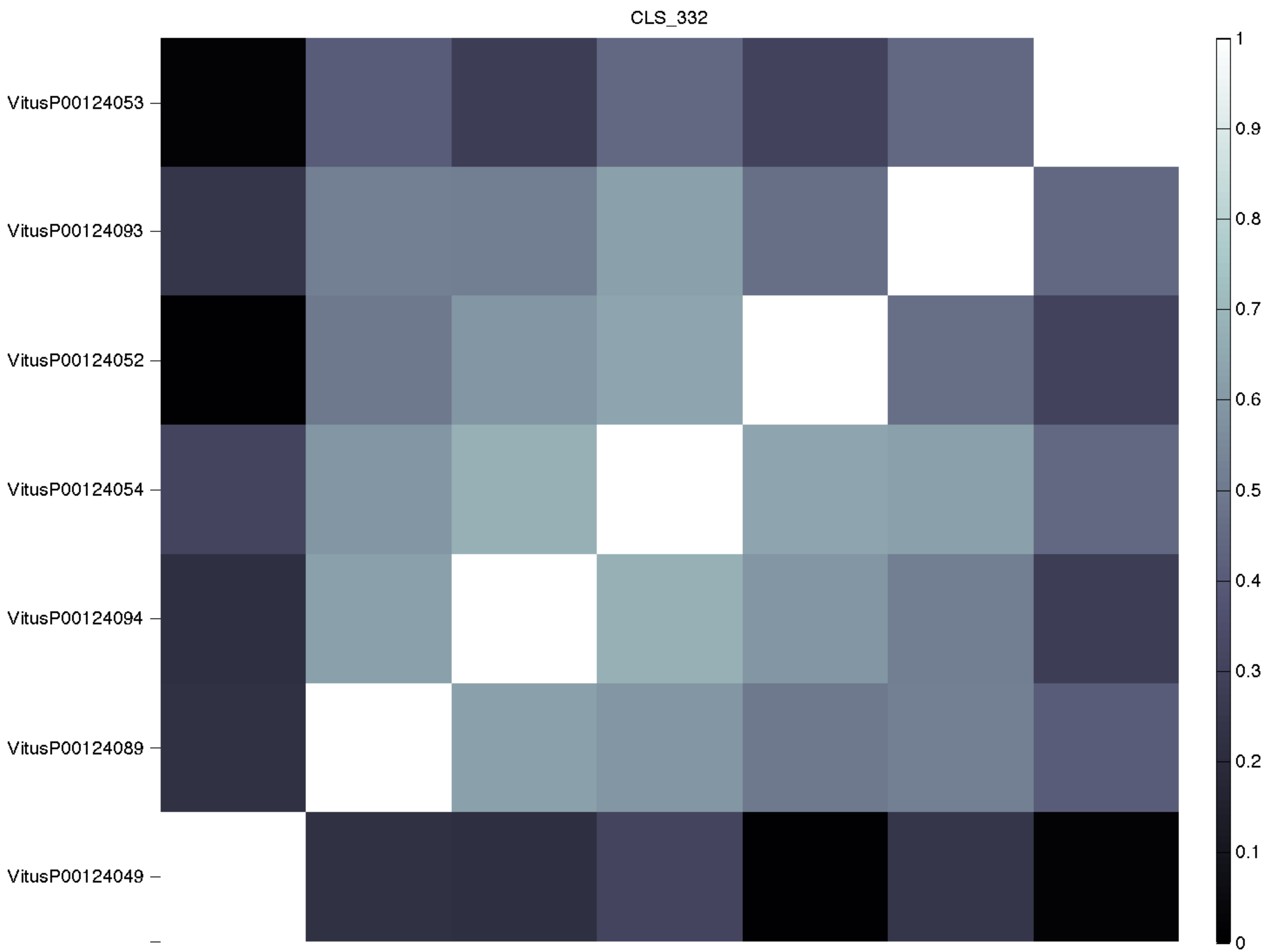

CLS_332
VitusP00124053
VitusP00124093
VitusP00124052
VitusP00124054
VitusP00124094
VitusP00124089
VitusP00124049
1
0.9
0.8
0.7
0.6
0.5
0.4
0.3
0.2
0.1
0

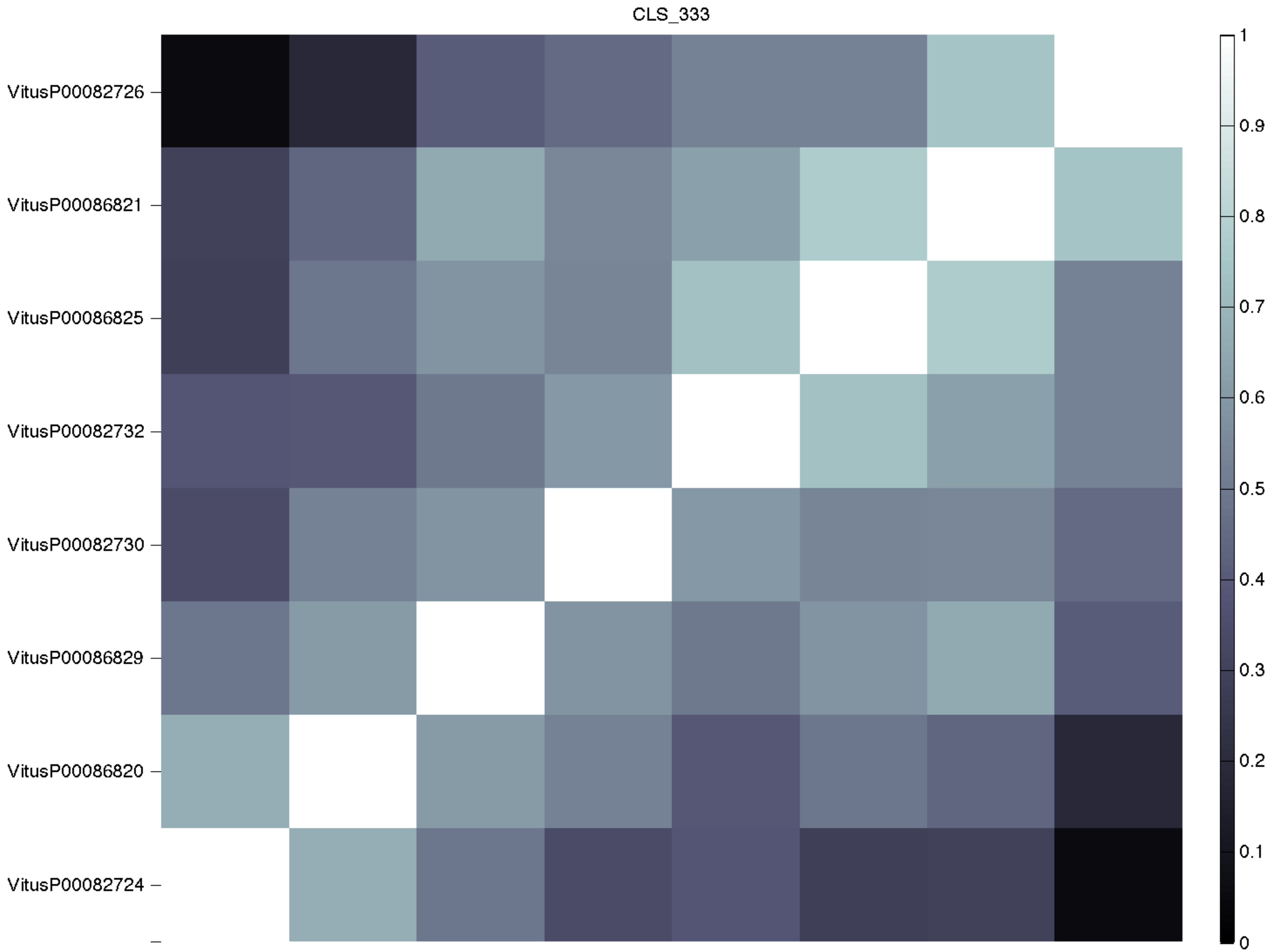

CLS_333
VitusP00082726
VitusP00086821
VitusP00086825
VitusP00082732
VitusP00082730
VitusP00086829
VitusP00086820
VitusP00082724
1
0.9
0.8
0.7
0.6
0.5
0.4
0.3
0.2
0.1
0

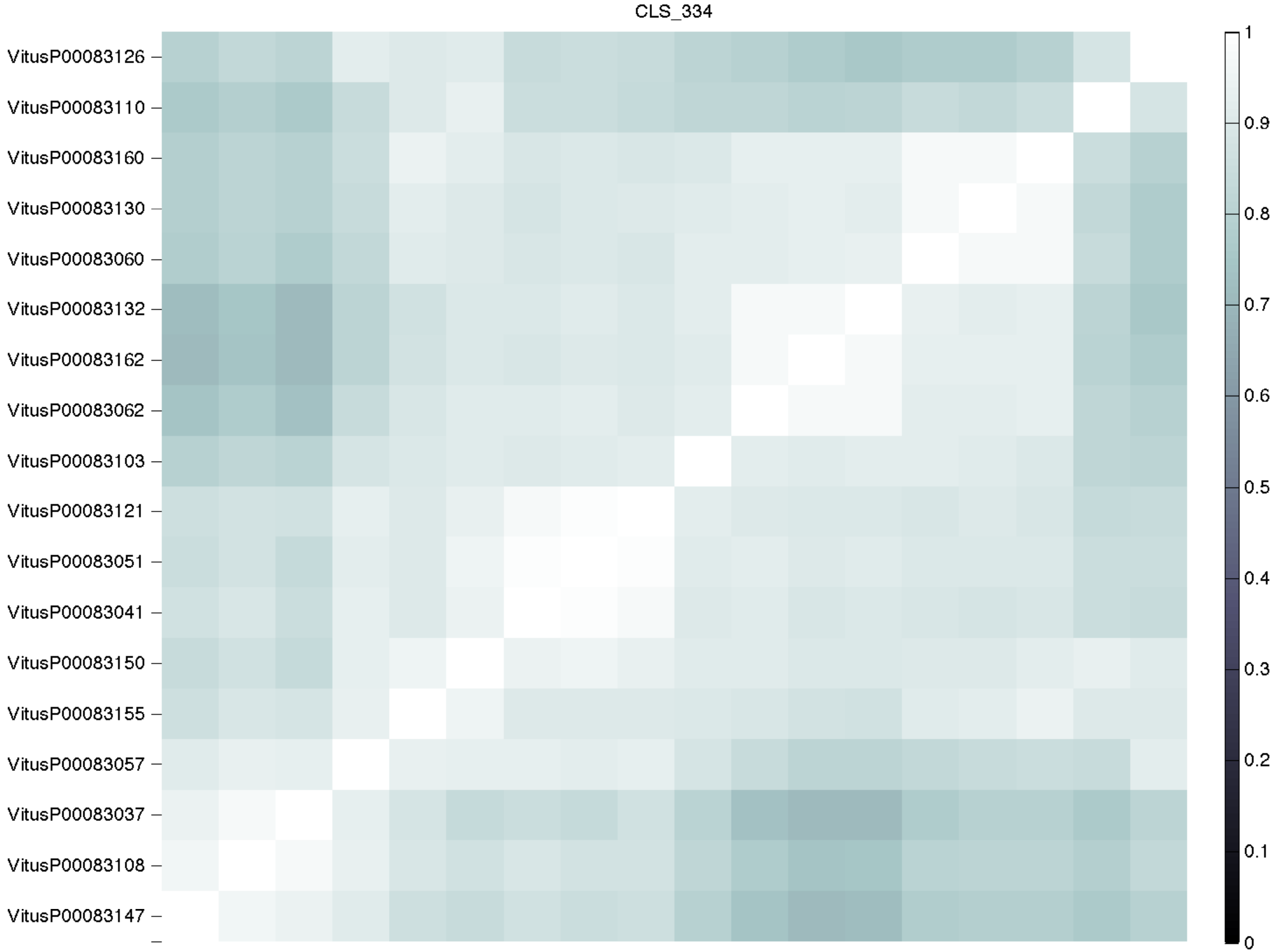
CLS_334
VitusP00083126
VitusP00083110
VitusP00083160
VitusP00083130
VitusP00083060
VitusP00083132
VitusP00083162
VitusP00083062
VitusP00083103
VitusP00083121
VitusP00083051
VitusP00083041
VitusP00083150
VitusP00083155
VitusP00083057
VitusP00083037
VitusP00083108
VitusP00083147
1
0.9
0.8
0.7
0.6
0.5
0.4
0.3
0.2
0.1
0

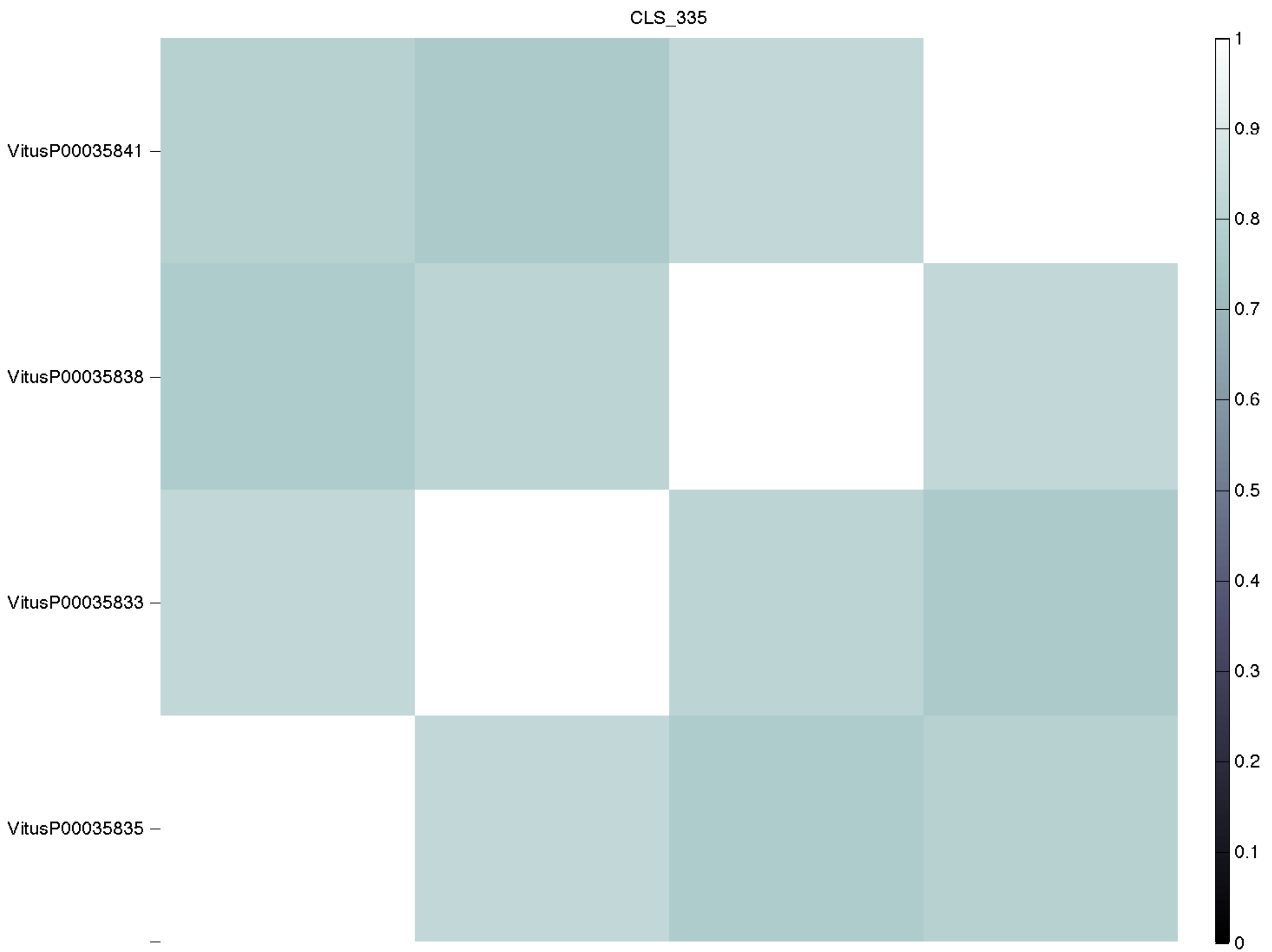
CLS_335
VitusP00035841
VitusP00035838
VitusP00035833
VitusP00035835
1
0.9
0.8
0.7
0.6
0.5
0.4
0.3
0.2
0.1
0

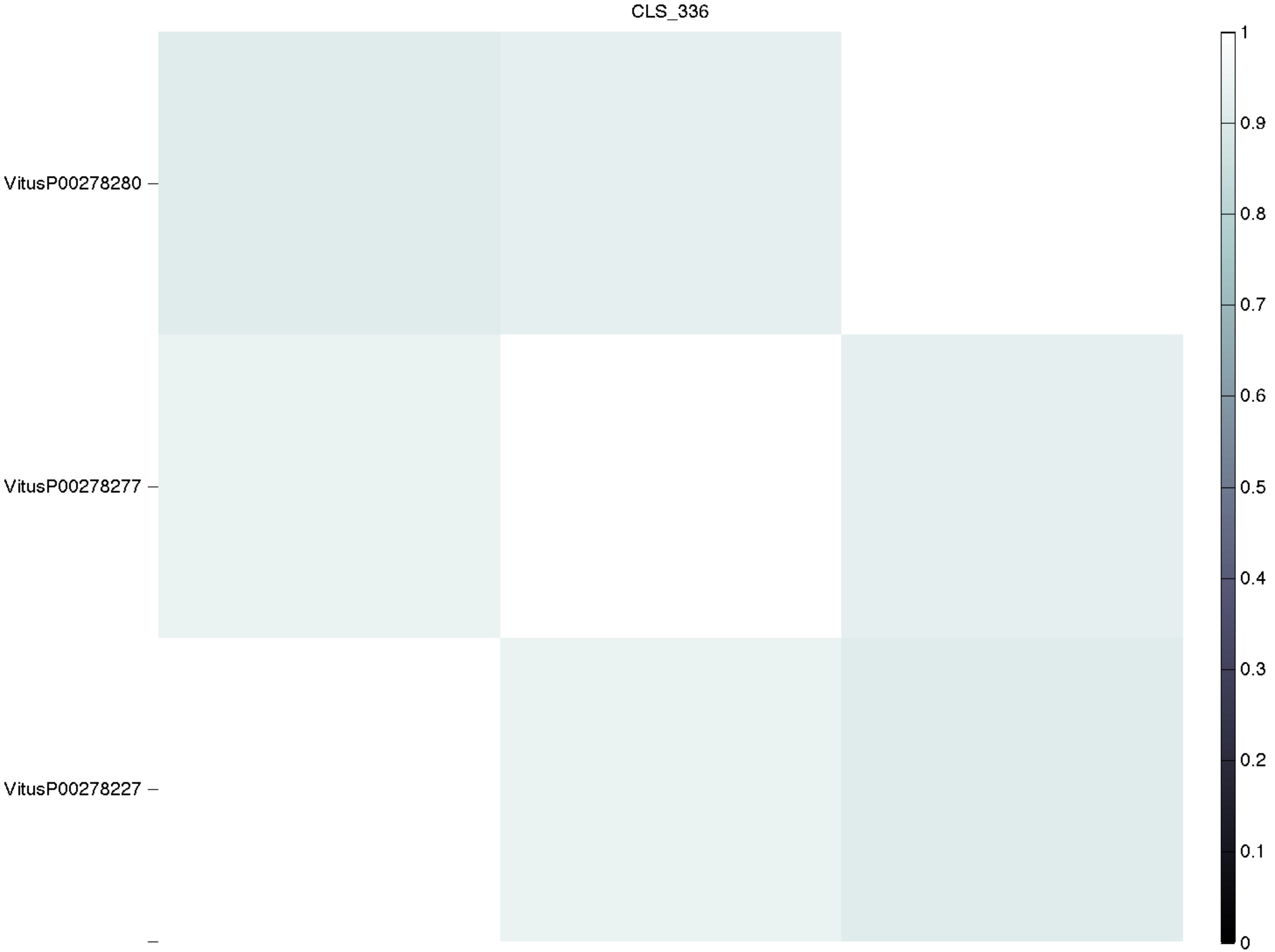

CLS_336
VitusP00278280
VitusP00278277
VitusP00278227
1
0.9
0.8
0.7
0.6
0.5
0.4
0.3
0.2
0.1
0

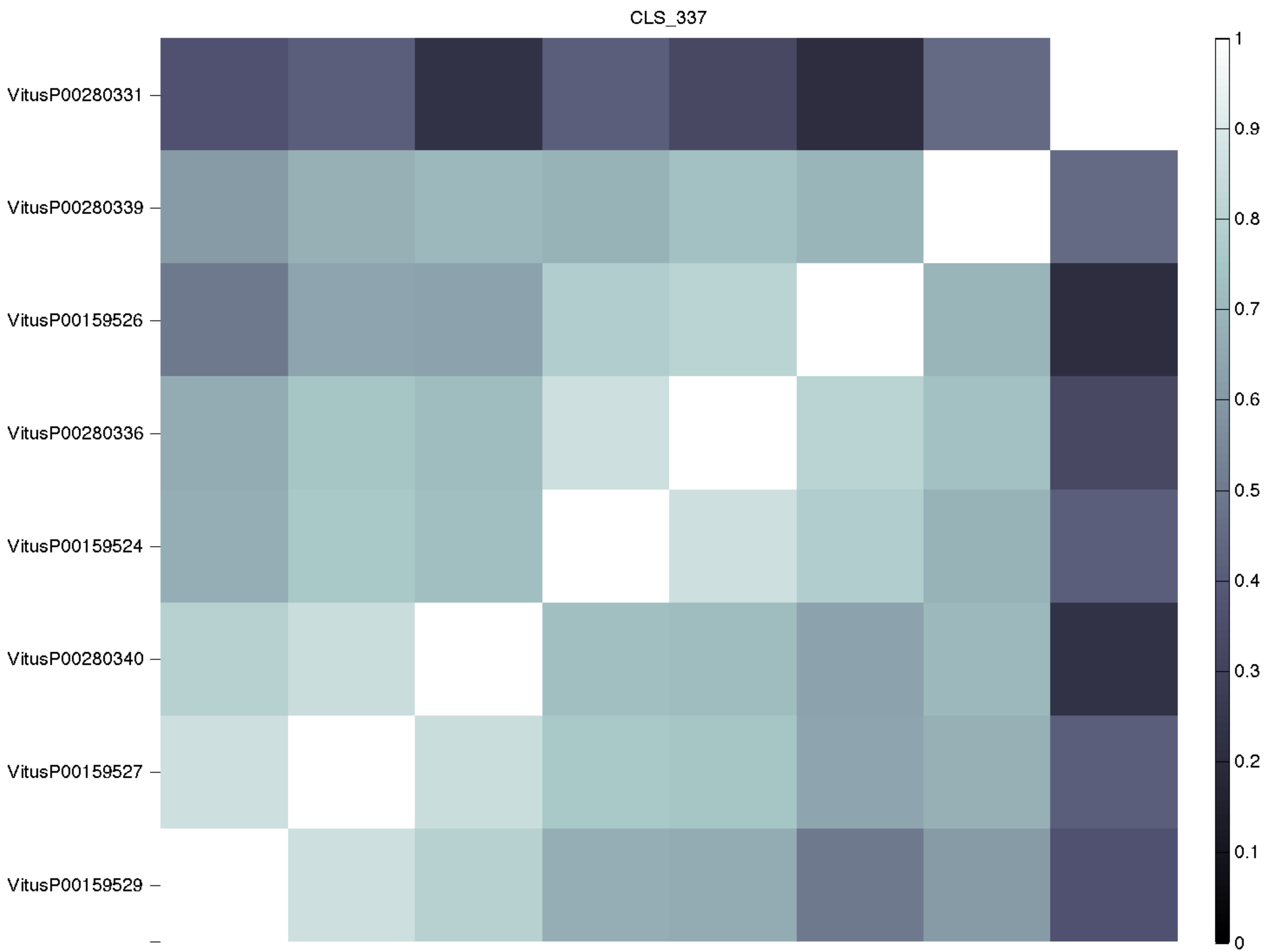
CLS_337
VitusP00280331
VitusP00280339
VitusP00159526
VitusP00280336
VitusP00159524
VitusP00280340
VitusP00159527
VitusP00159529
1
0.9
0.8
0.7
0.6
0.5
0.4
0.3
0.2
0.1
0

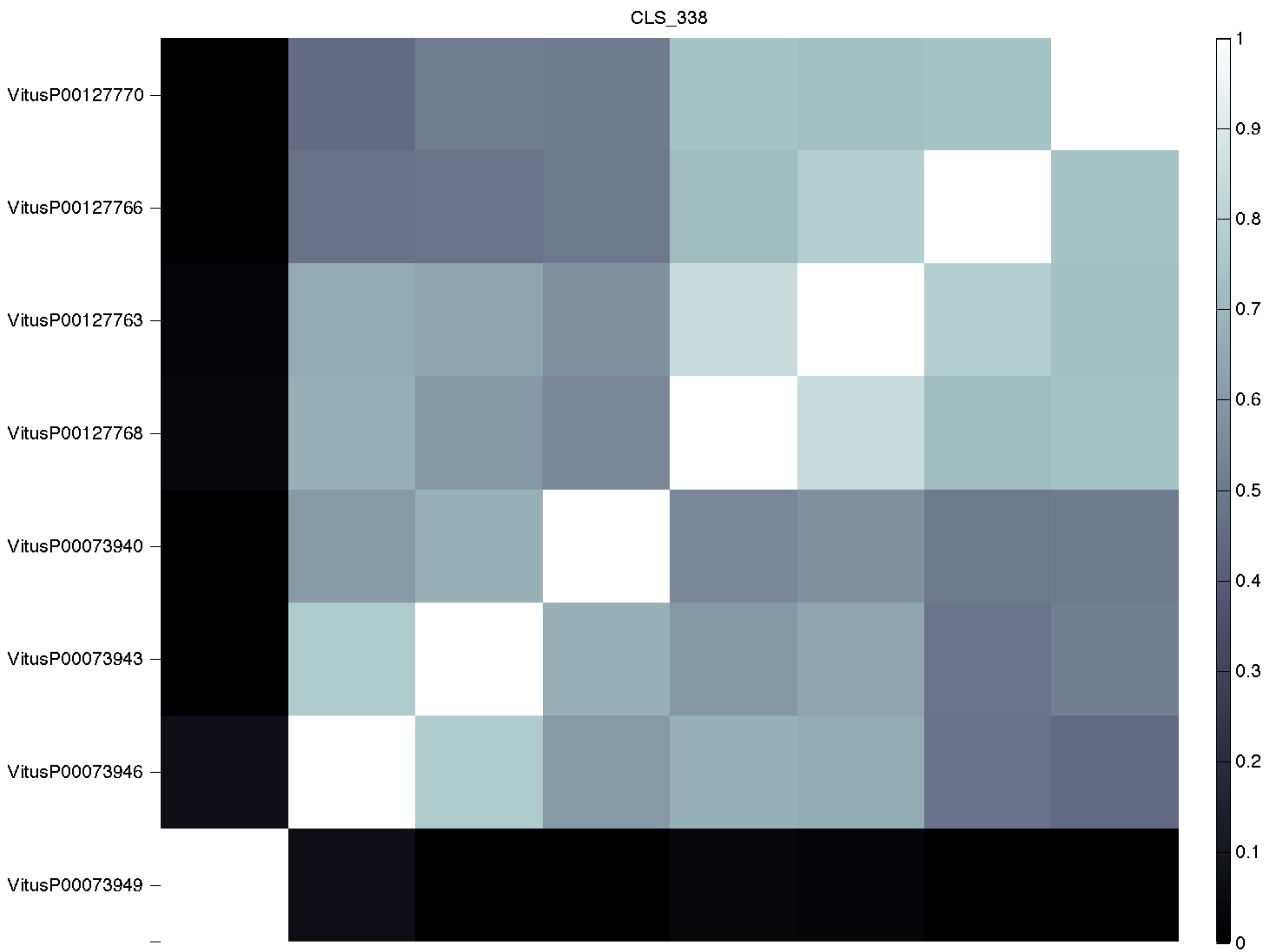

CLS_338
VitusP00127770
VitusP00127766
VitusP00127763
VitusP00127768
VitusP00073940
VitusP00073943
VitusP00073946
VitusP00073949
1
0.9
0.8
0.7
0.6
0.5
0.4
0.3
0.2
0.1
0

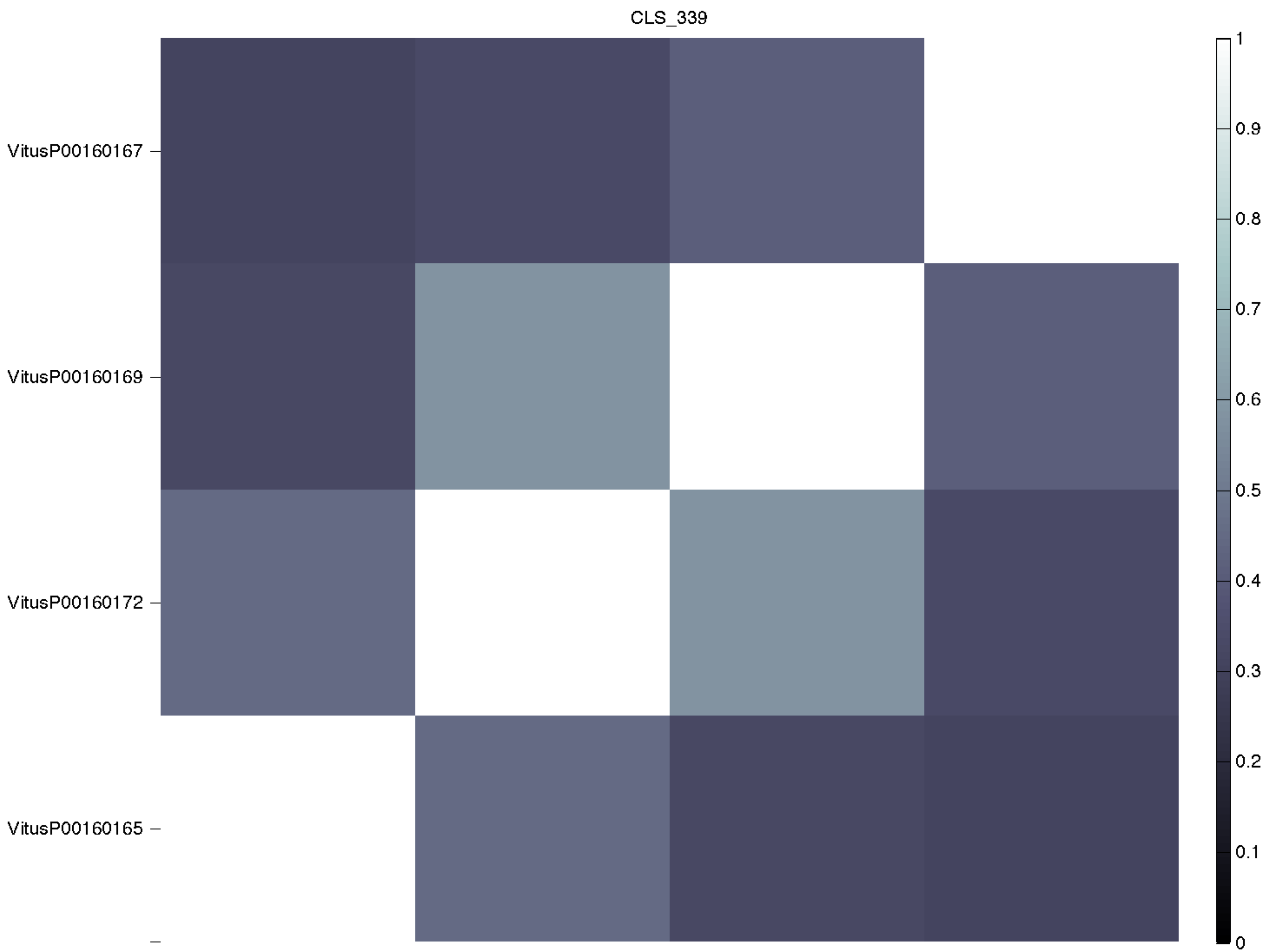
CLS_339
VitusP00160167
VitusP00160169
VitusP00160172
VitusP00160165
1
0.9
0.8
0.7
0.6
0.5
0.4
0.3
0.2
0.1
0

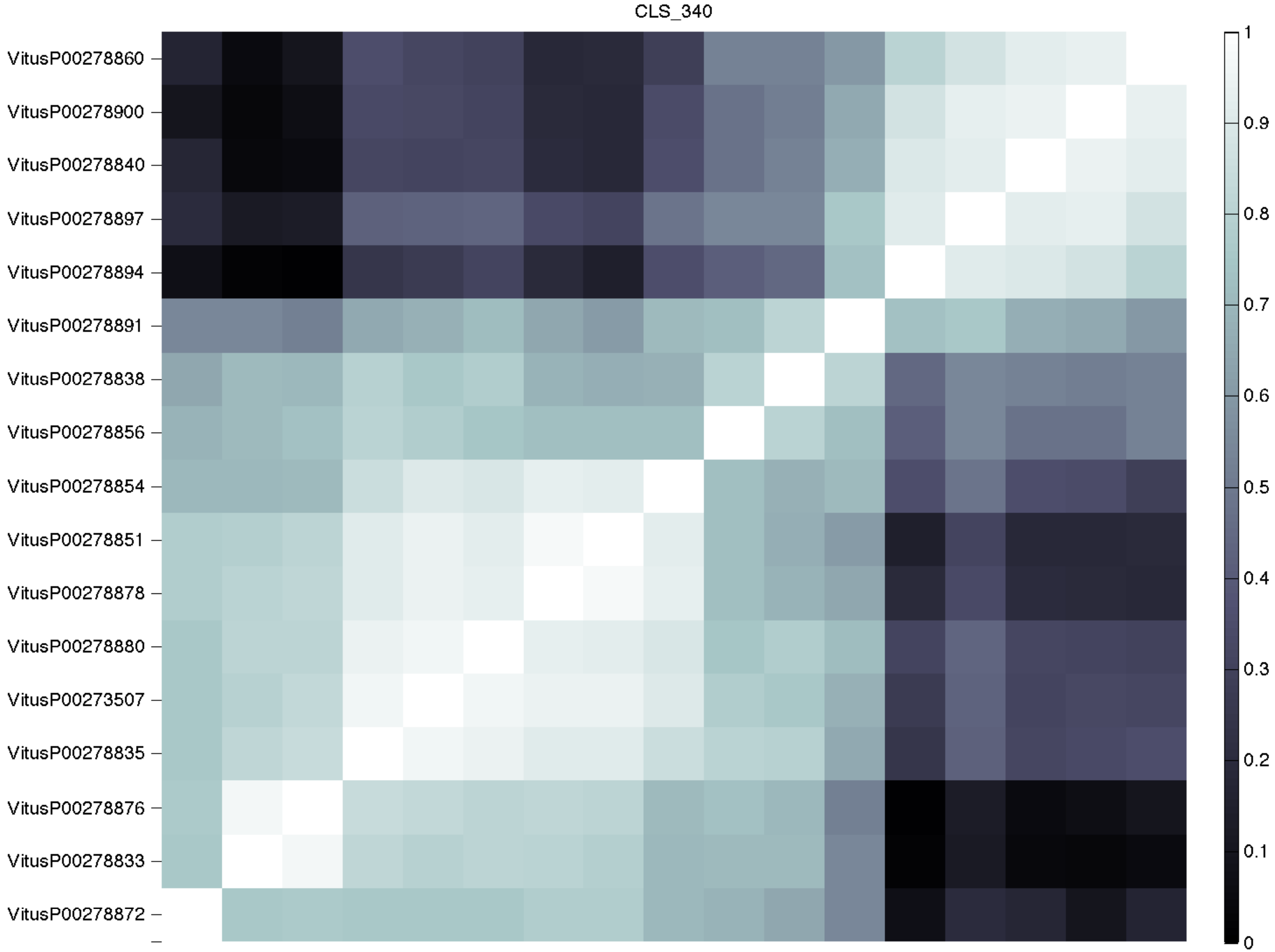
CLS_340
VitusP00278860
VitusP00278900
VitusP00278840
VitusP00278897
VitusP00278894
VitusP00278891
VitusP00278838
VitusP00278856
VitusP00278854
VitusP00278851
VitusP00278878
VitusP00278880
VitusP00273507
VitusP00278835
VitusP00278876
VitusP00278833
VitusP00278872
1
0.9
0.8
0.7
0.6
0.5
0.4
0.3
0.2
0.1
0

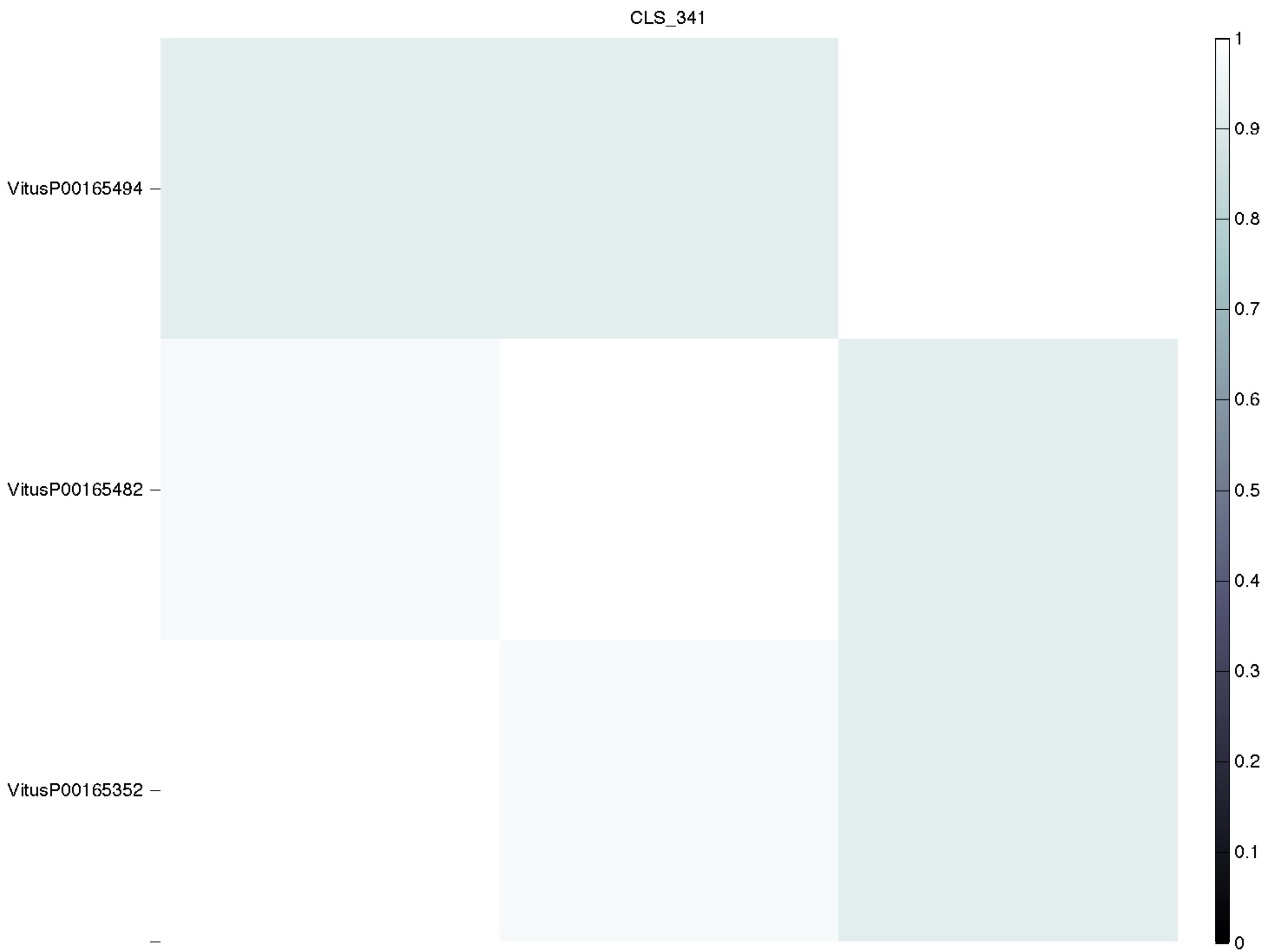
CLS_341
VitusP00165494
VitusP00165482
VitusP00165352
1
0.9
0.8
0.7
0.6
0.5
0.4
0.3
0.2
0.1
0

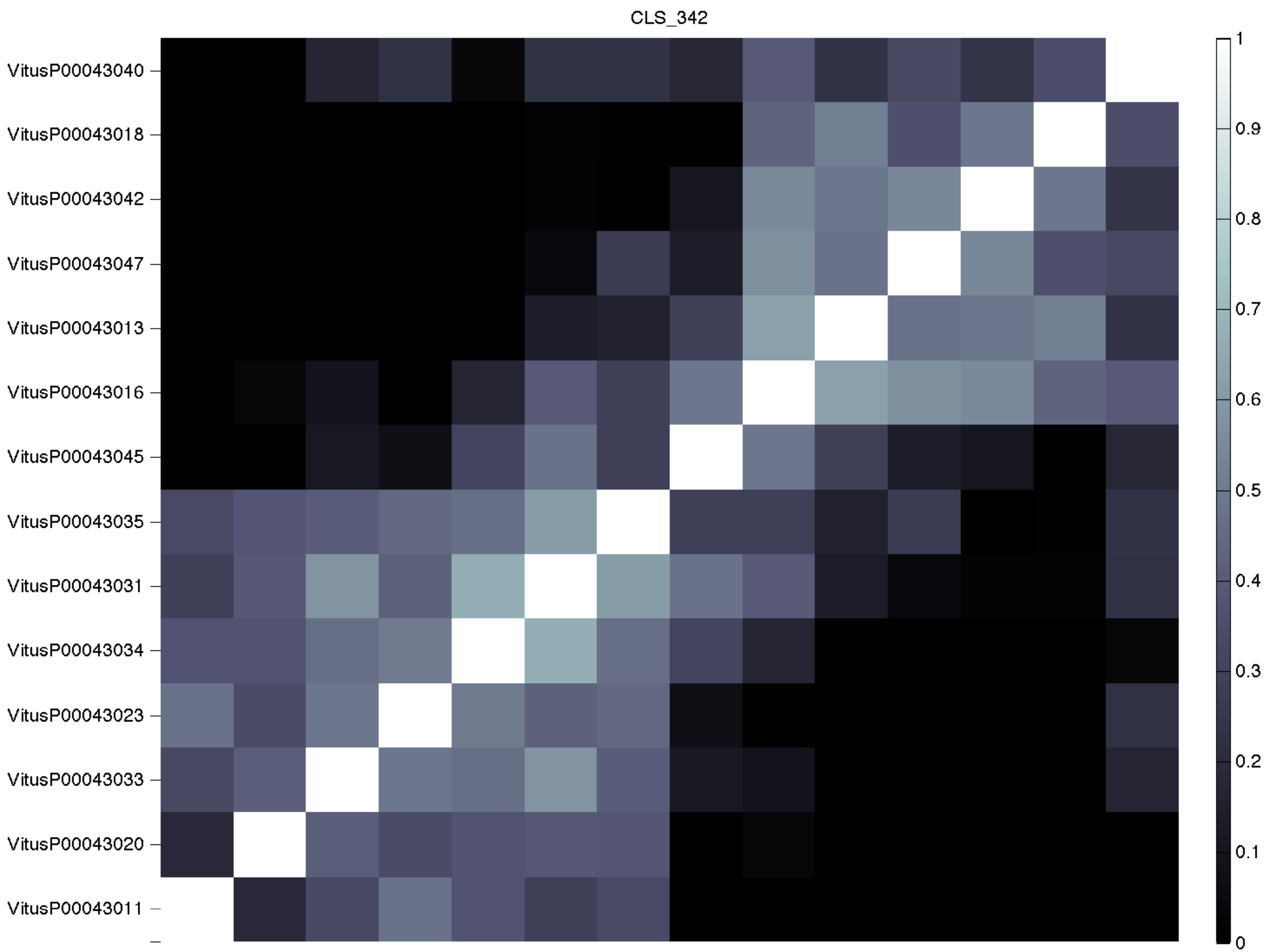
CLS_342
VitusP00043040
VitusP00043018
VitusP00043042
VitusP00043047
VitusP00043013
VitusP00043016
VitusP00043045
VitusP00043035
VitusP00043031
VitusP00043034
VitusP00043023
VitusP00043033
VitusP00043020
VitusP00043011
1
0.9
0.8
0.7
0.6
0.5
0.4
0.3
0.2
0.1
0

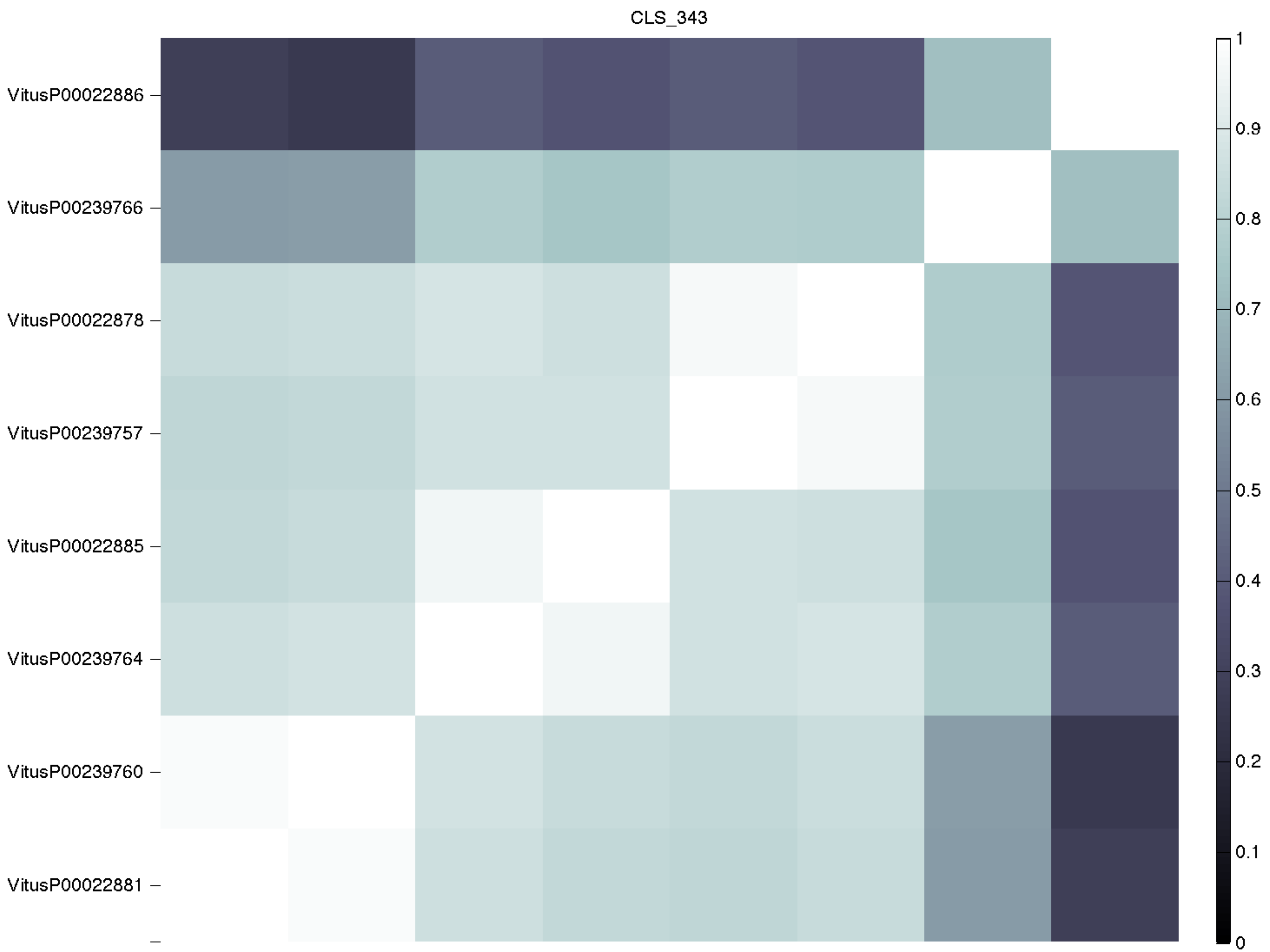
CLS_343
VitusP00022886
VitusP00239766
VitusP00022878
VitusP00239757
VitusP00022885
VitusP00239764
VitusP00239760
VitusP00022881
1
0.9
0.8
0.7
0.6
0.5
0.4
0.3
0.2
0.1
0

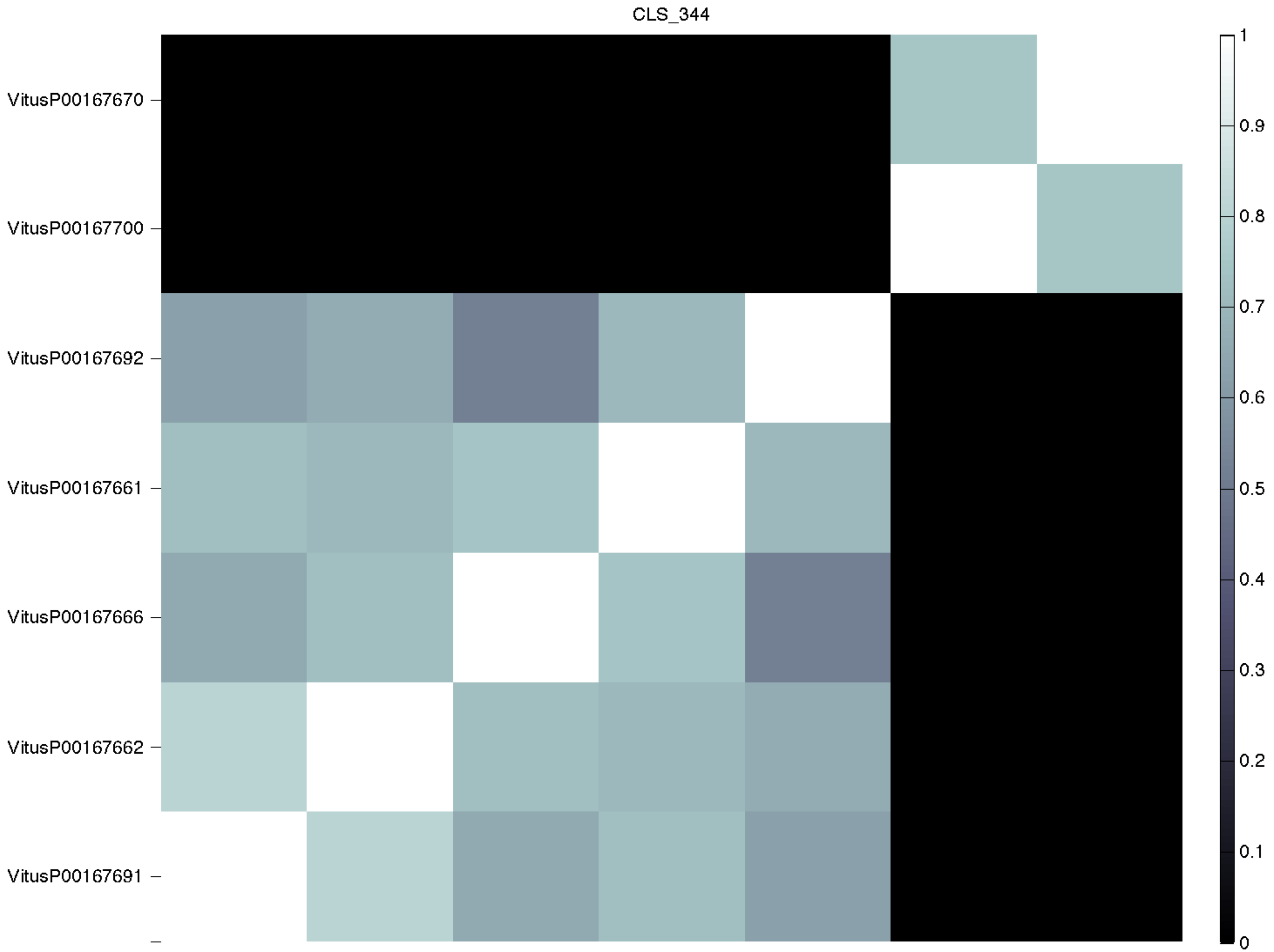
CLS_344
VitusP00167670
VitusP00167700
VitusP00167692
VitusP00167661
VitusP00167666
VitusP00167662
VitusP00167691
1
0.9
0.8
0.7
0.6
0.5
0.4
0.3
0.2
0.1
0

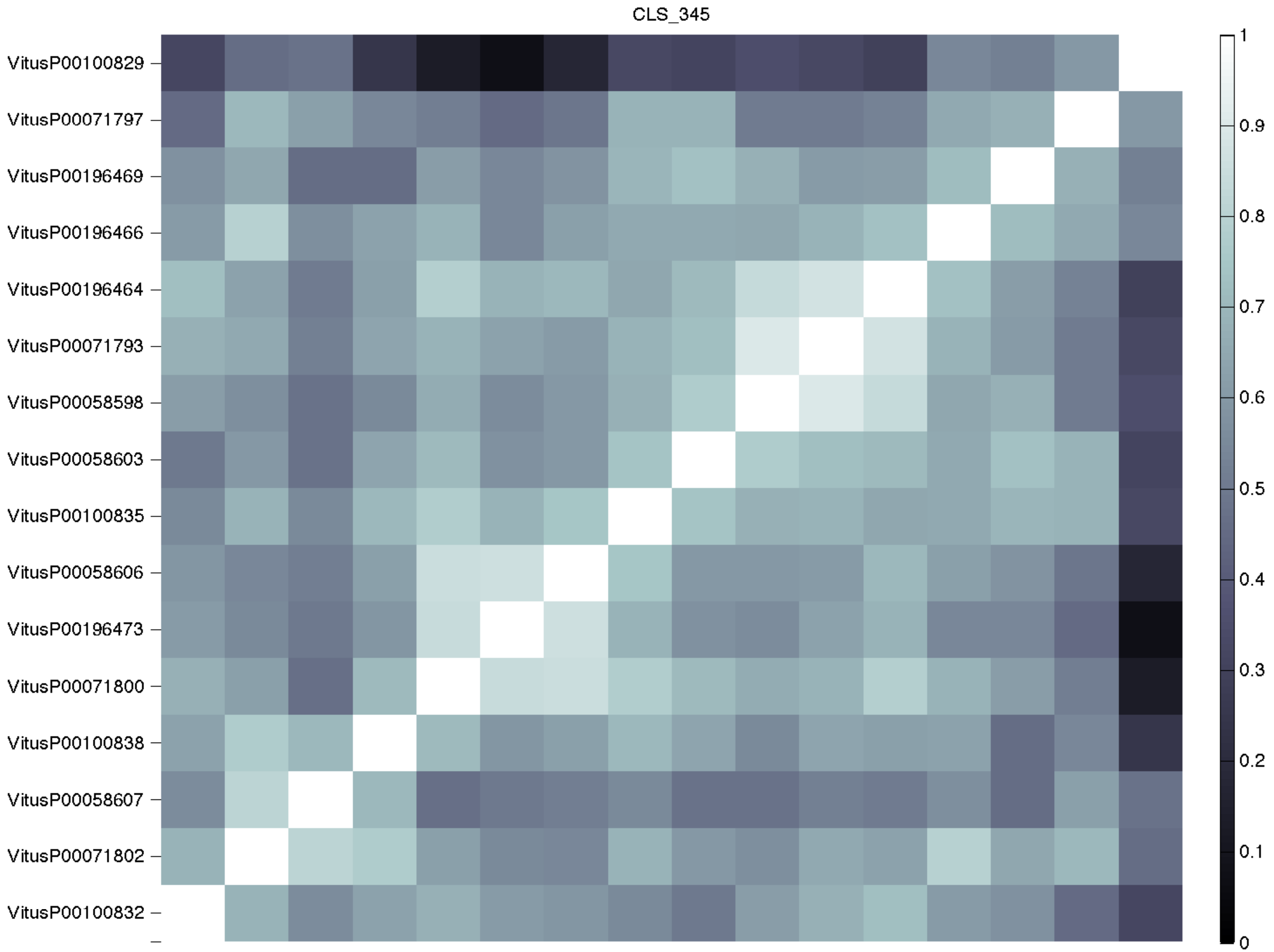
CLS_345
VitusP00100829
VitusP00071797
VitusP00196469
VitusP00196466
VitusP00196464
VitusP00071793
VitusP00058598
VitusP00058603
VitusP00100835
VitusP00058606
VitusP00196473
VitusP00071800
VitusP00100838
VitusP00058607
VitusP00071802
VitusP00100832
1
0.9
0.8
0.7
0.6
0.5
0.4
0.3
0.2
0.1
0

CLS_346

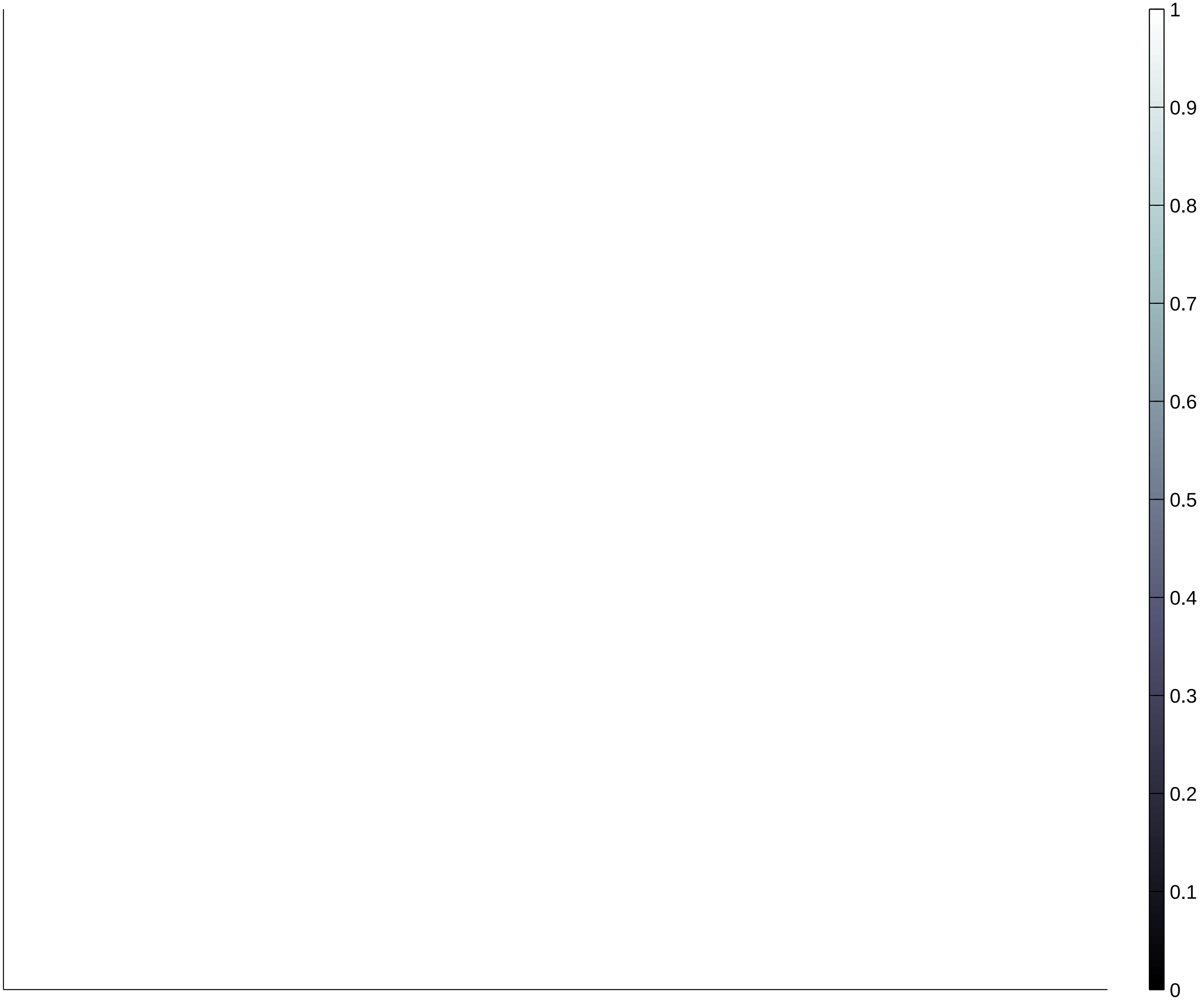

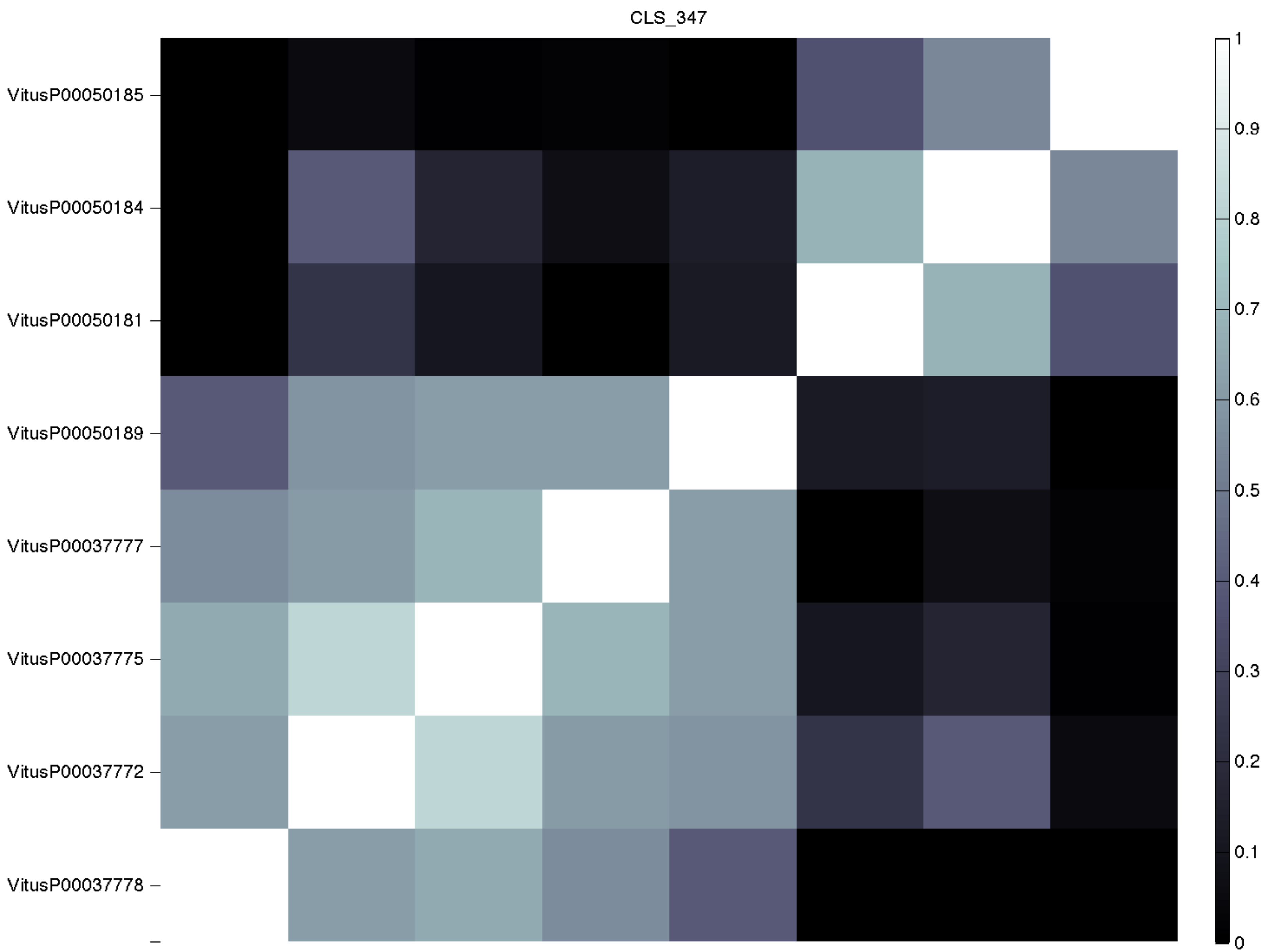
CLS_347
VitusP00050185
VitusP00050184
VitusP00050181
VitusP00050189
VitusP00037777
VitusP00037775
VitusP00037772
VitusP00037778
1
0.9
0.8
0.7
0.6
0.5
0.4
0.3
0.2
0.1
0

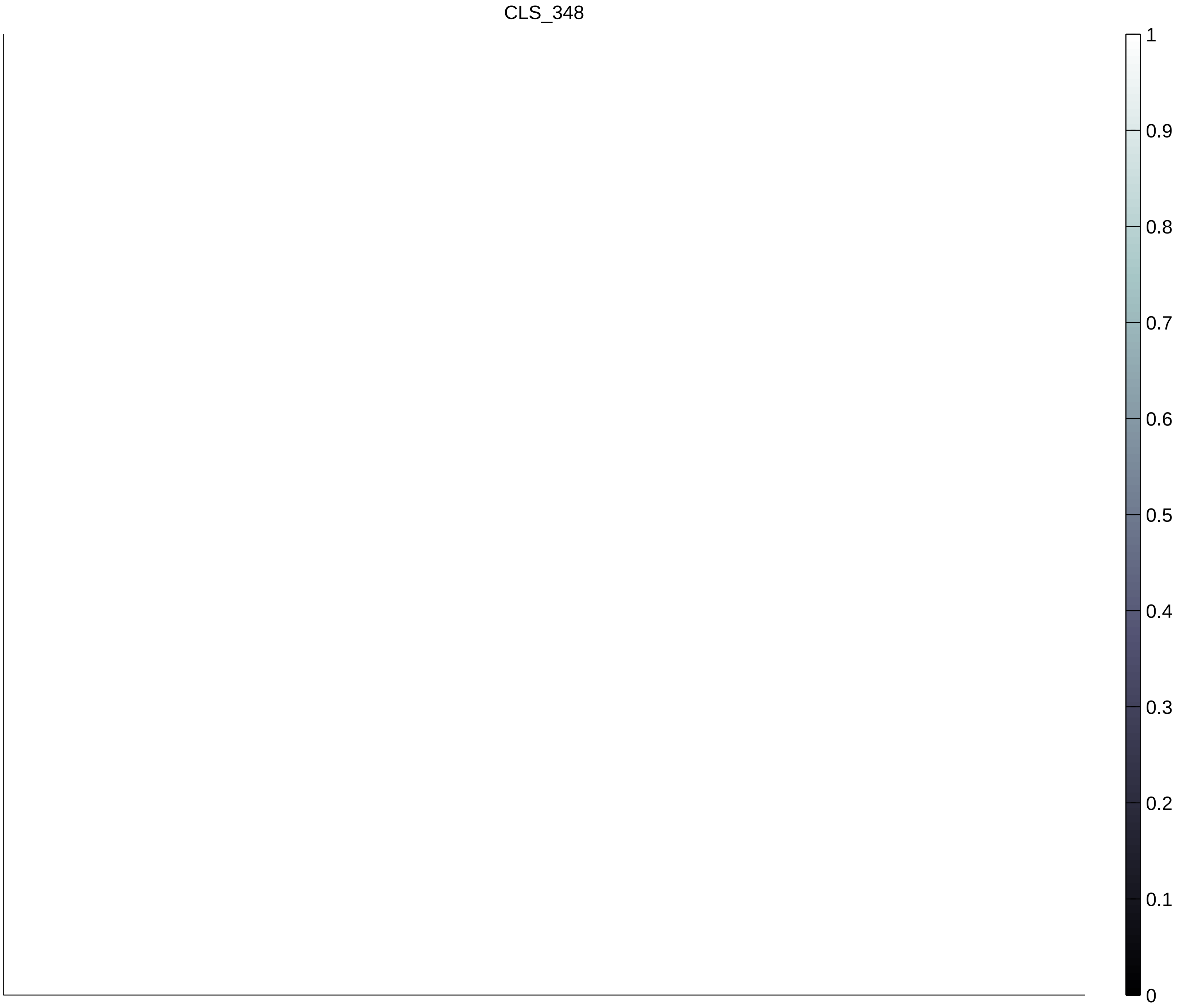
CLS_348
1
0.9
0.8
0.7
0.6
0.5
0.4
0.3
0.2
0.1
0

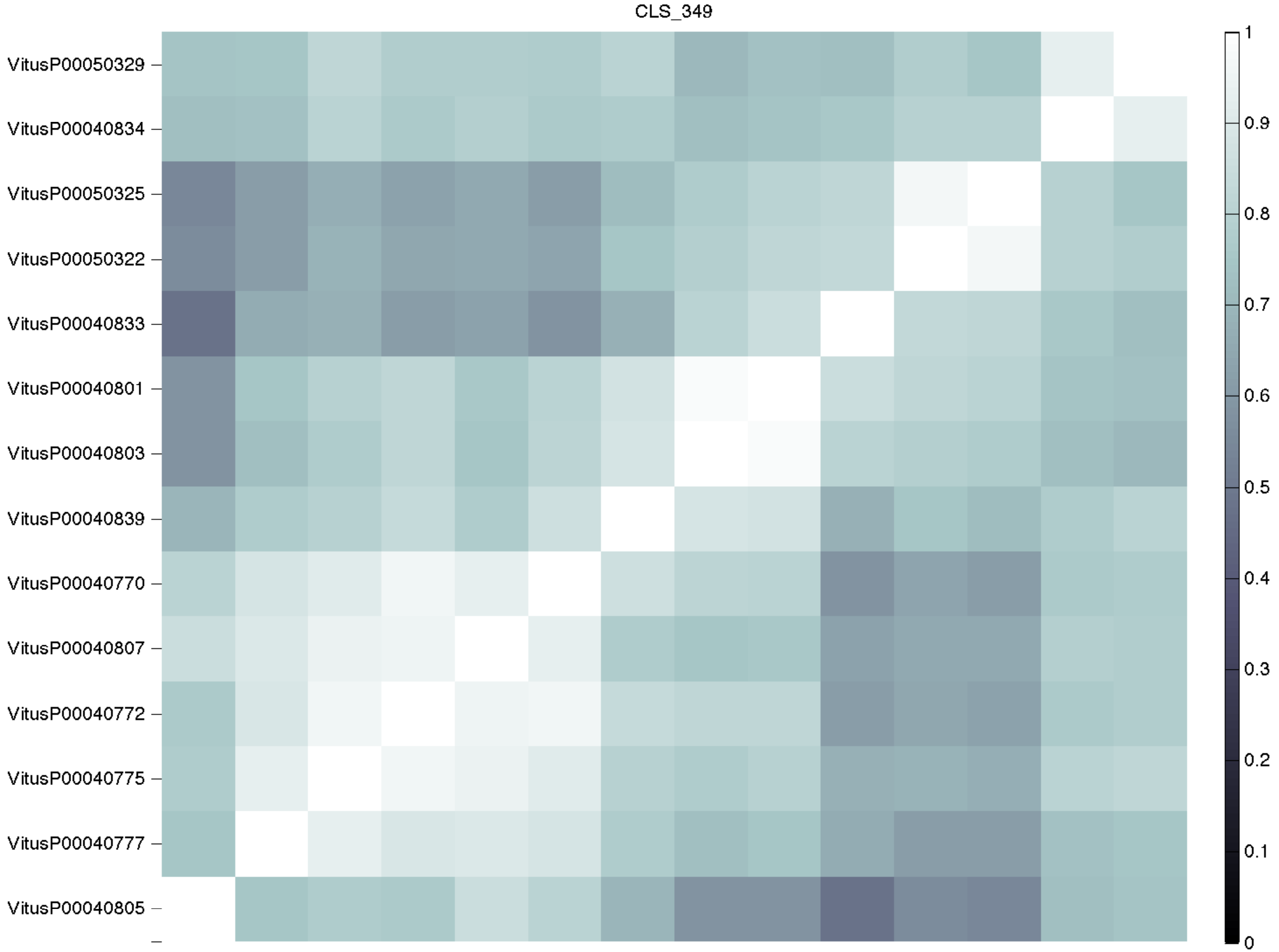

CLS_349
VitusP00050329
VitusP00040834
VitusP00050325
VitusP00050322
VitusP00040833
VitusP00040801
VitusP00040803
VitusP00040839
VitusP00040770
VitusP00040807
VitusP00040772
VitusP00040775
VitusP00040777
VitusP00040805
1
0.9
0.8
0.7
0.6
0.5
0.4
0.3
0.2
0.1
0

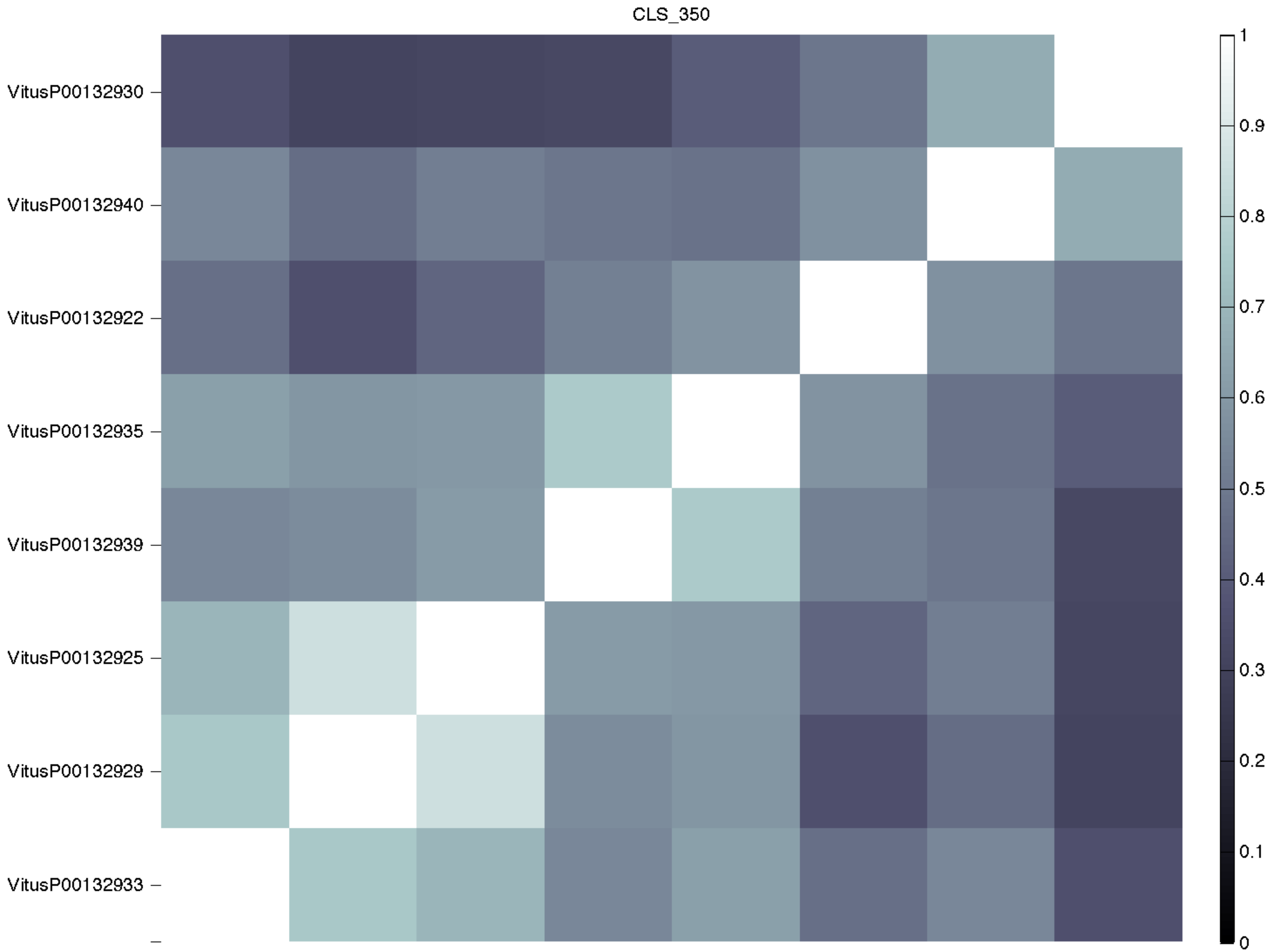

CLS_350
VitusP00132930
VitusP00132940
VitusP00132922
VitusP00132935
VitusP00132939
VitusP00132925
VitusP00132929
VitusP00132933
1
0.9
0.8
0.7
0.6
0.5
0.4
0.3
0.2
0.1
0

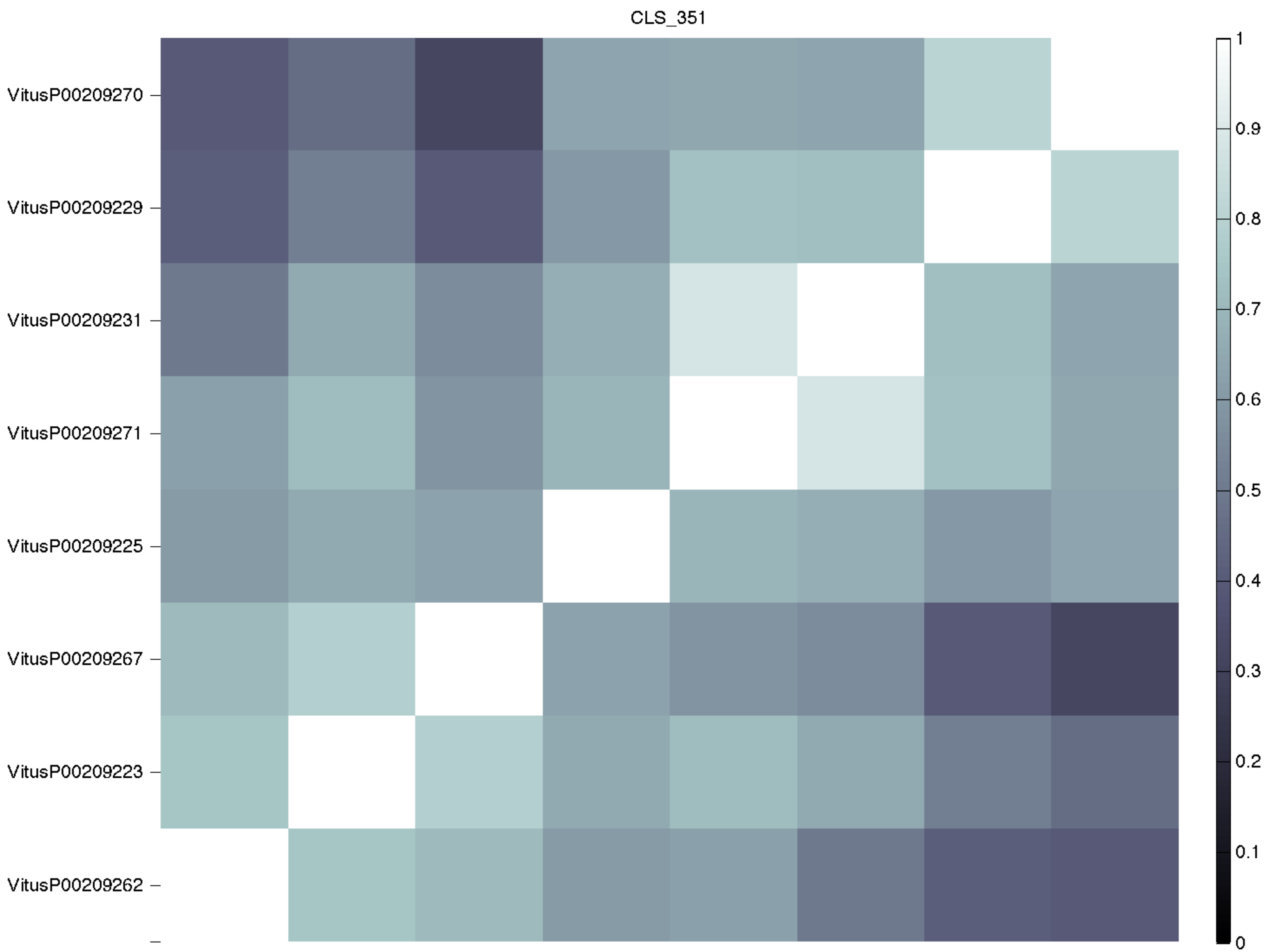

CLS_351
VitusP00209270
VitusP00209229
VitusP00209231
VitusP00209271
VitusP00209225
VitusP00209267
VitusP00209223
VitusP00209262
1
0.9
0.8
0.7
0.6
0.5
0.4
0.3
0.2
0.1
0

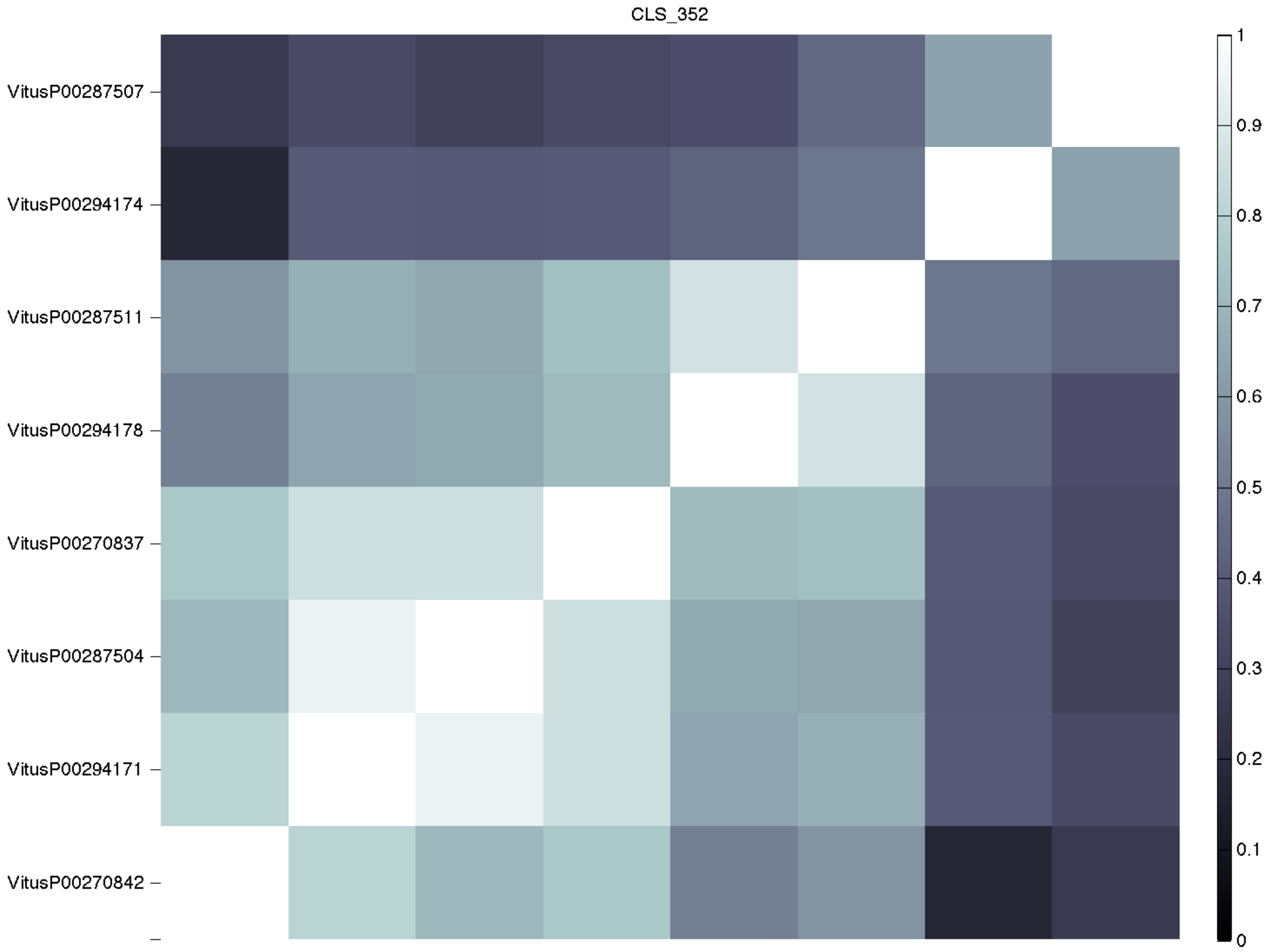

CLS_352
VitusP00287507
VitusP00294174
VitusP00287511
VitusP00294178
VitusP00270837
VitusP00287504
VitusP00294171
VitusP00270842
1
0.9
0.8
0.7
0.6
0.5
0.4
0.3
0.2
0.1
0

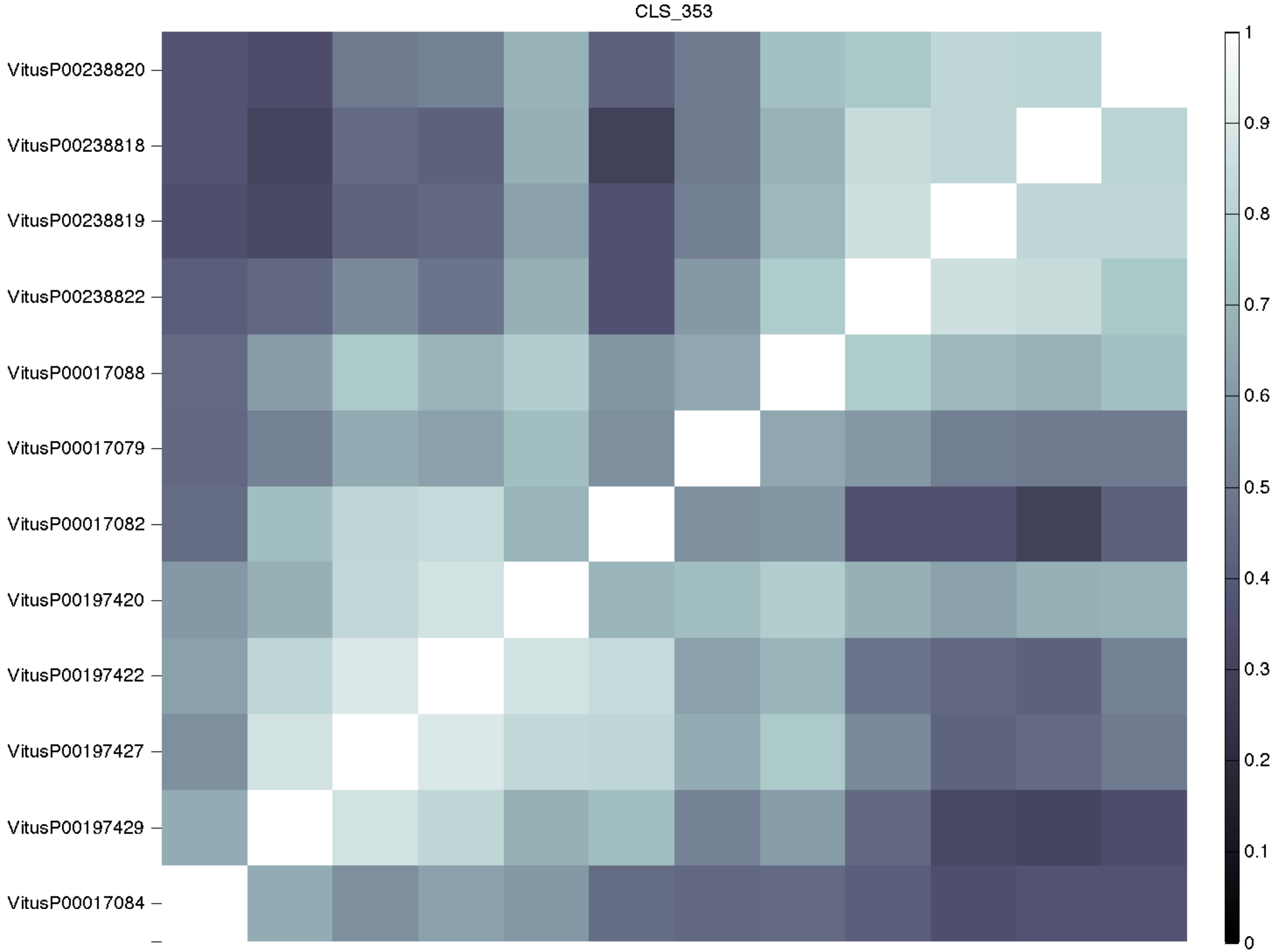
CLS_353
VitusP00238820
VitusP00238818
VitusP00238819
VitusP00238822
VitusP00017088
VitusP00017079
VitusP00017082
VitusP00197420
VitusP00197422
VitusP00197427
VitusP00197429
VitusP00017084
1
0.9
0.8
0.7
0.6
0.5
0.4
0.3
0.2
0.1
0

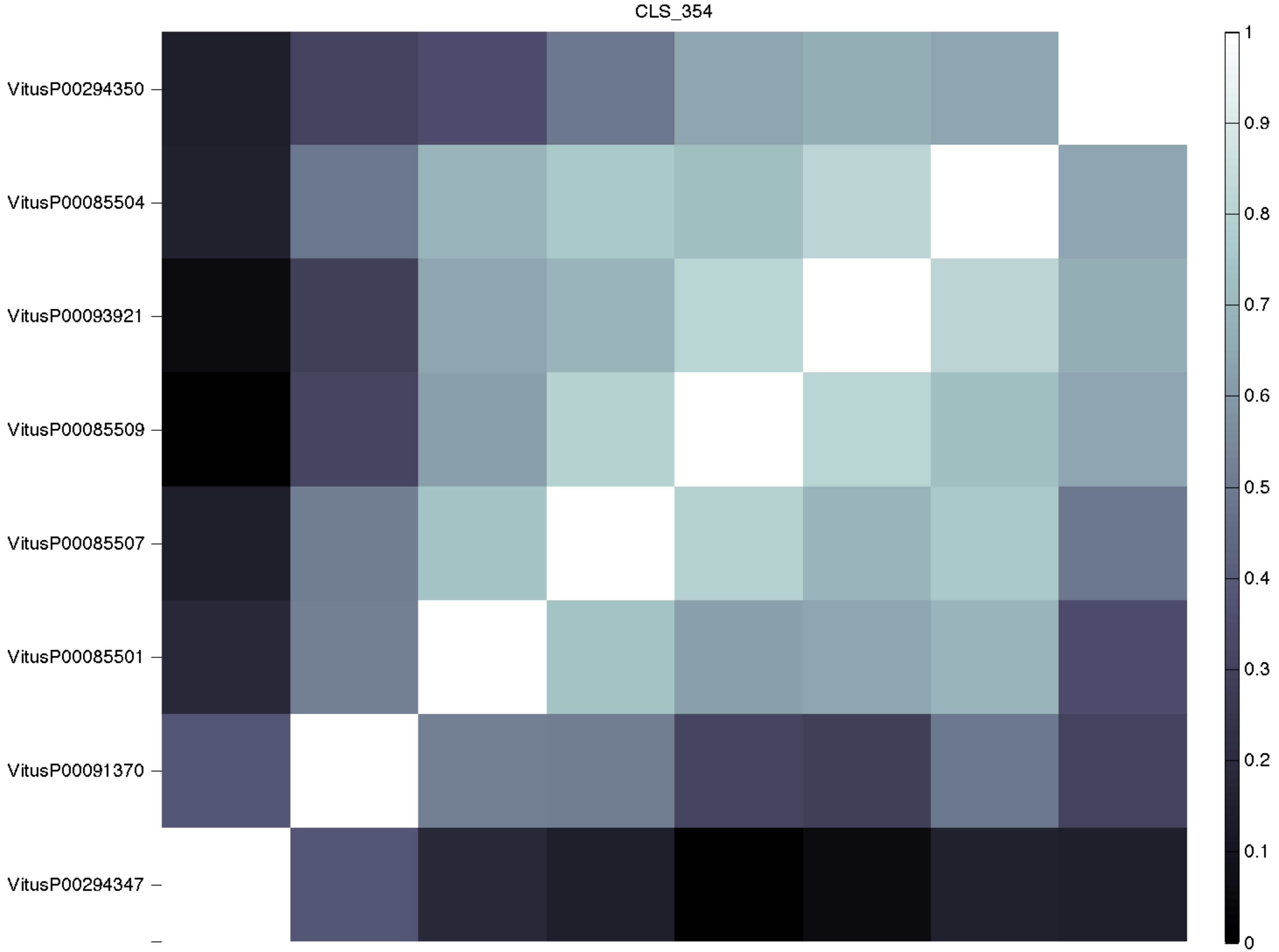
CLS_354
VitusP00294350
VitusP00085504
VitusP00093921
VitusP00085509
VitusP00085507
VitusP00085501
VitusP00091370
VitusP00294347
1
0.9
0.8
0.7
0.6
0.5
0.4
0.3
0.2
0.1
0

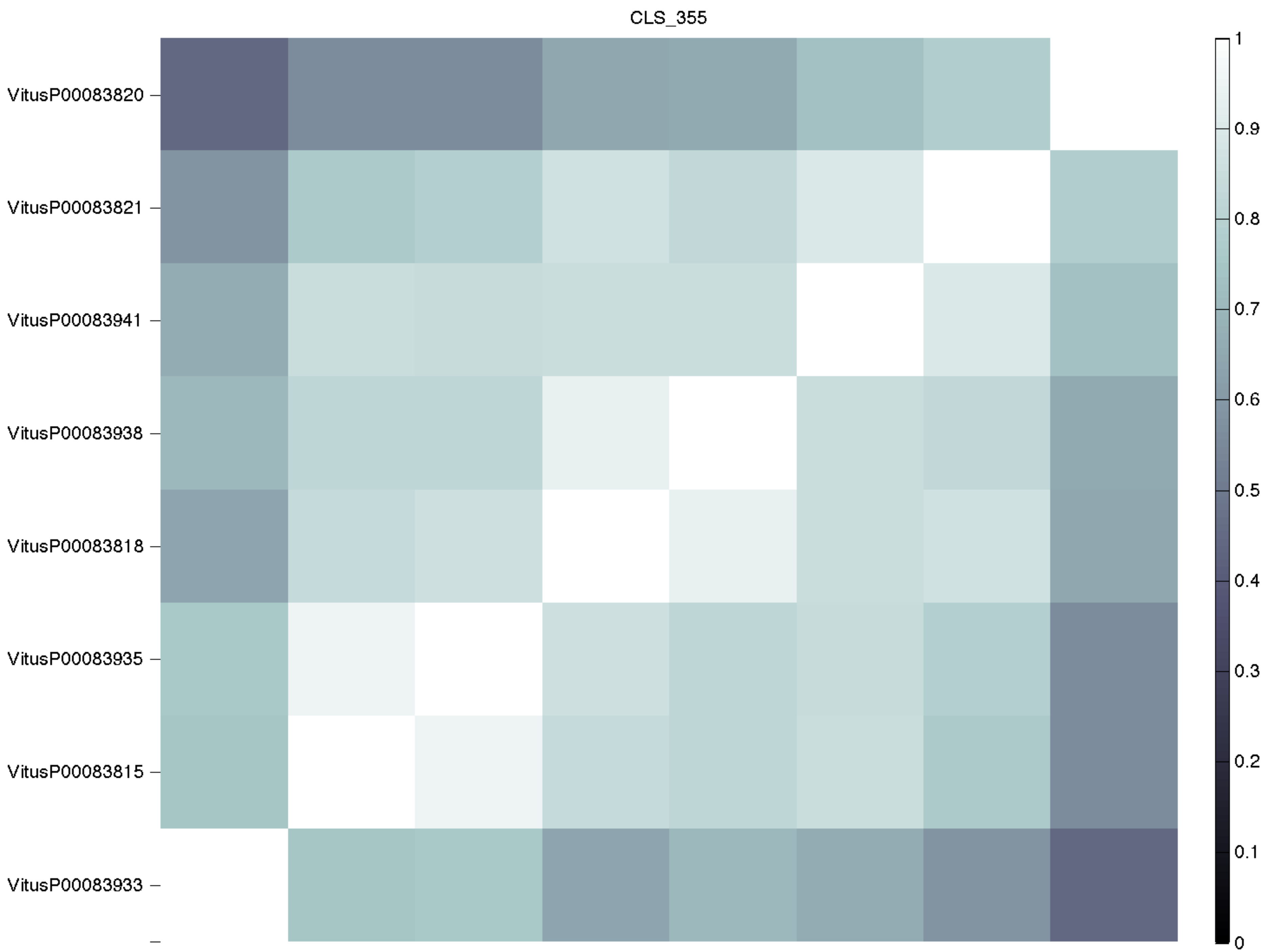

CLS_355
VitusP00083820
VitusP00083821
VitusP00083941
VitusP00083938
VitusP00083818
VitusP00083935
VitusP00083815
VitusP00083933
1
0.9
0.8
0.7
0.6
0.5
0.4
0.3
0.2
0.1
0

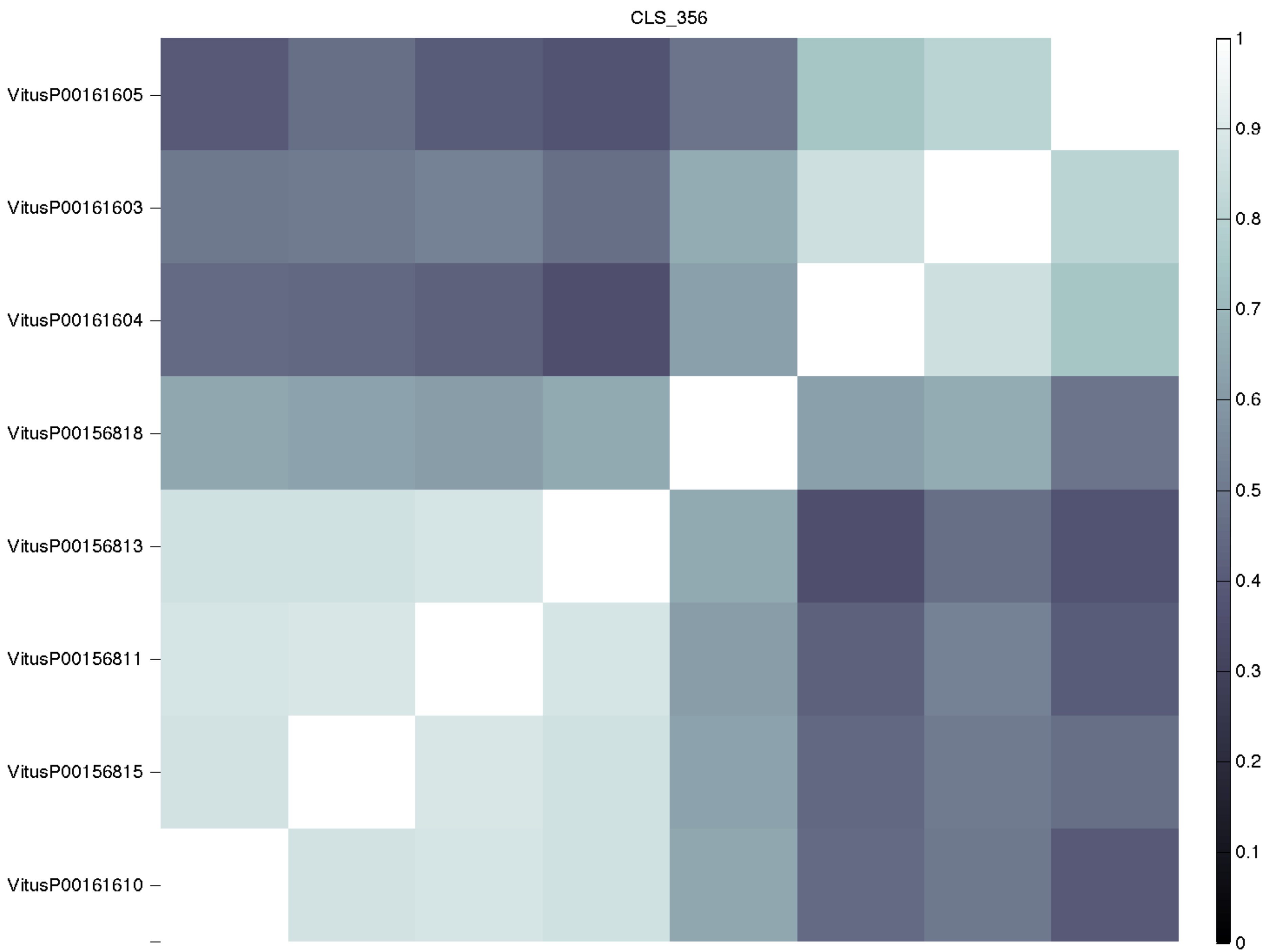

CLS_356
VitusP00161605
VitusP00161603
VitusP00161604
VitusP00156818
VitusP00156813
VitusP00156811
VitusP00156815
VitusP00161610
1
0.9
0.8
0.7
0.6
0.5
0.4
0.3
0.2
0.1
0

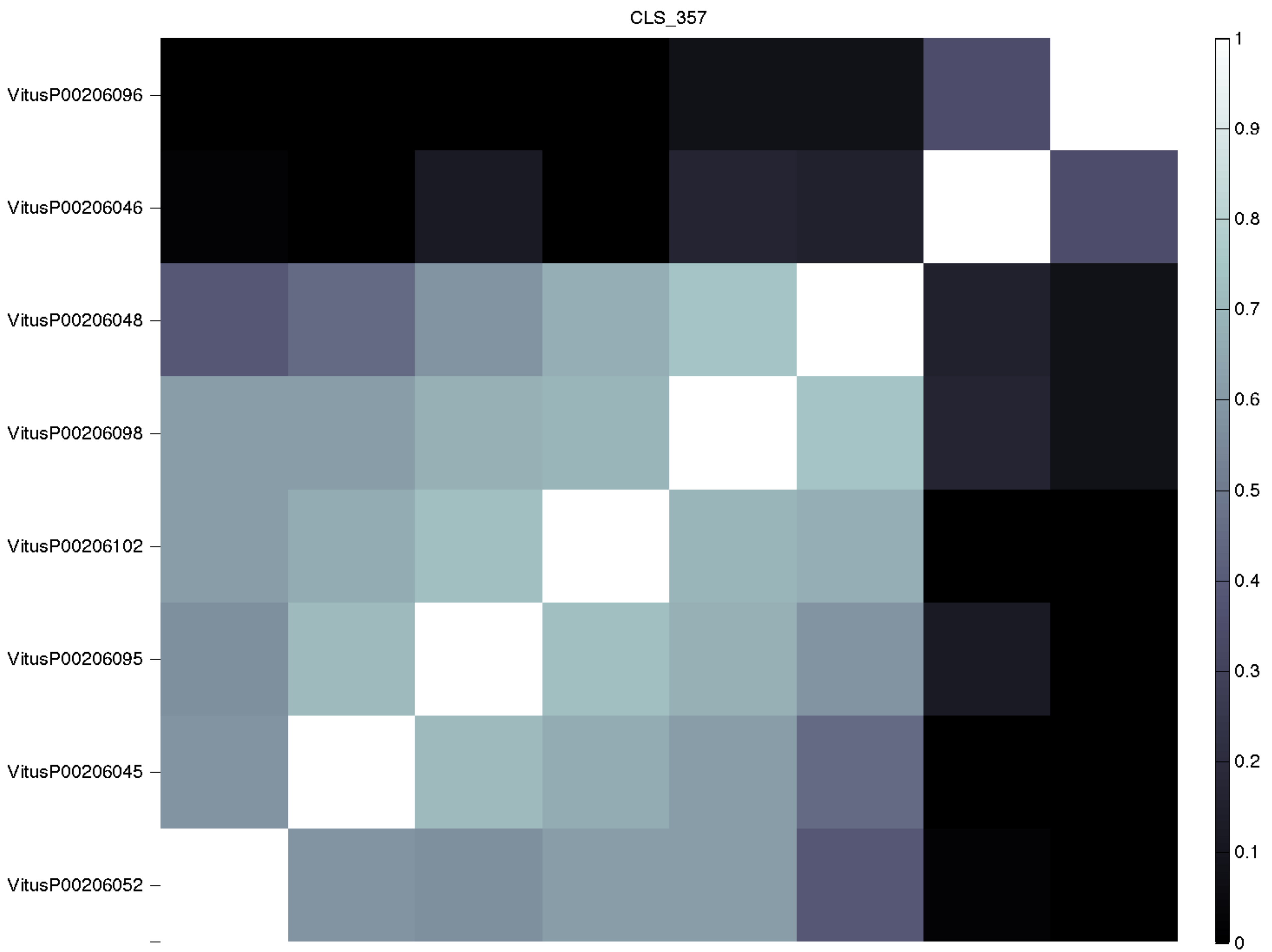

CLS_357
VitusP00206096
VitusP00206046
VitusP00206048
VitusP00206098
VitusP00206102
VitusP00206095
VitusP00206045
VitusP00206052
1
0.9
0.8
0.7
0.6
0.5
0.4
0.3
0.2
0.1
0

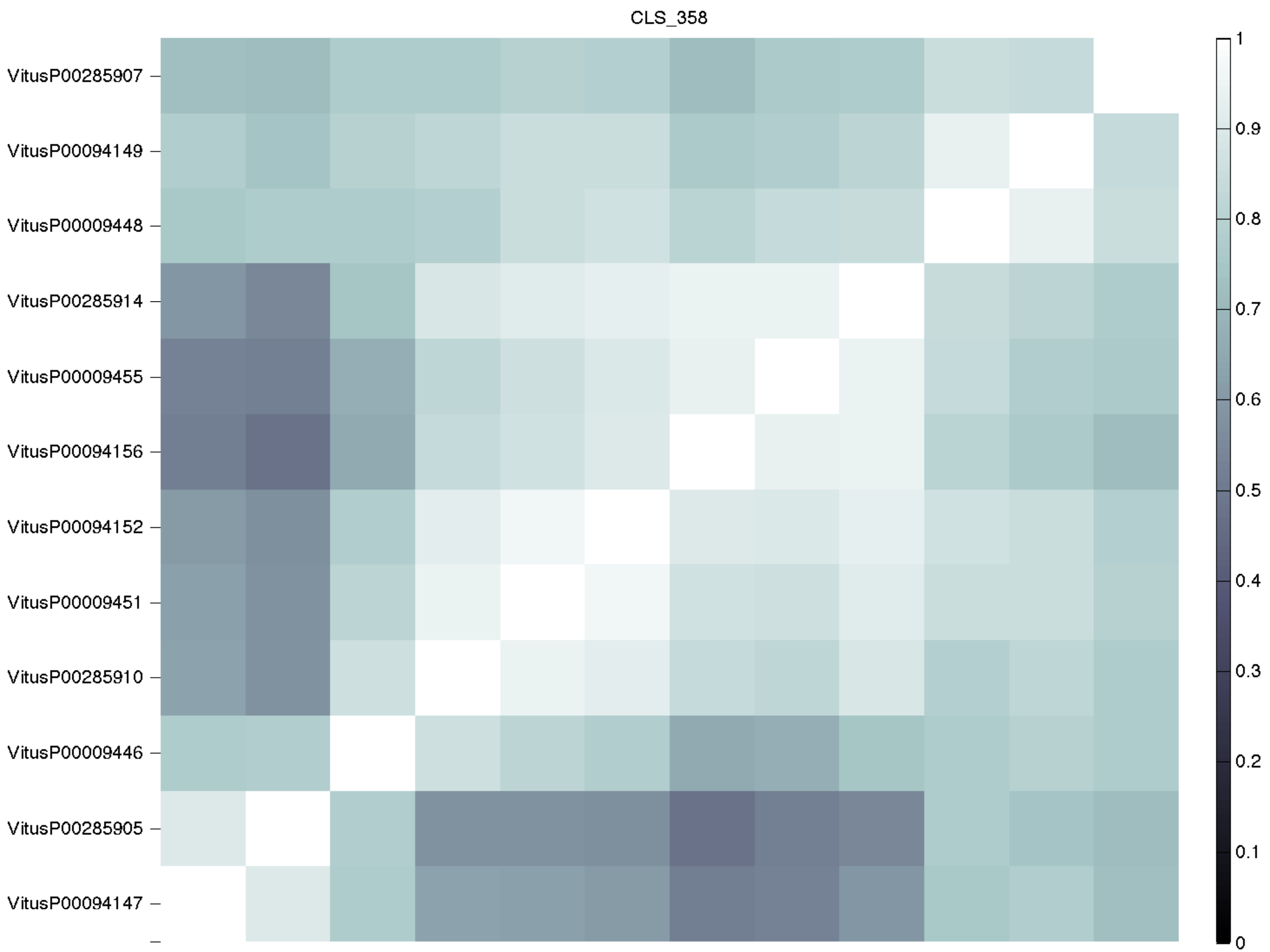
CLS_358
VitusP00285907
VitusP00094149
VitusP00009448
VitusP00285914
VitusP00009455
VitusP00094156
VitusP00094152
VitusP00009451
VitusP00285910
VitusP00009446
VitusP00285905
VitusP00094147
1
0.9
0.8
0.7
0.6
0.5
0.4
0.3
0.2
0.1
0

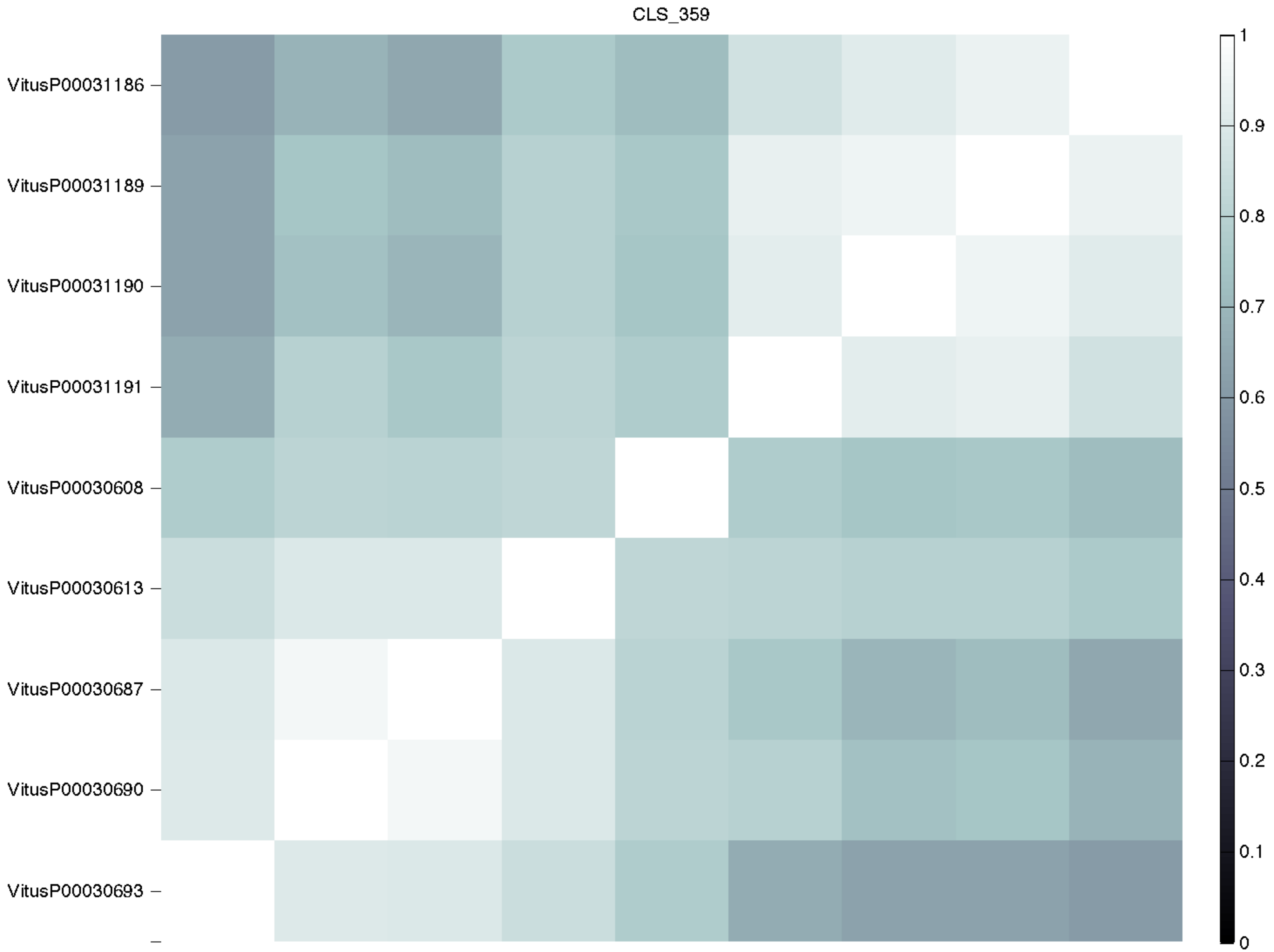
CLS_359
VitusP00031186
VitusP00031189
VitusP00031190
VitusP00031191
VitusP00030608
VitusP00030613
VitusP00030687
VitusP00030690
VitusP00030693
1
0.9
0.8
0.7
0.6
0.5
0.4
0.3
0.2
0.1
0

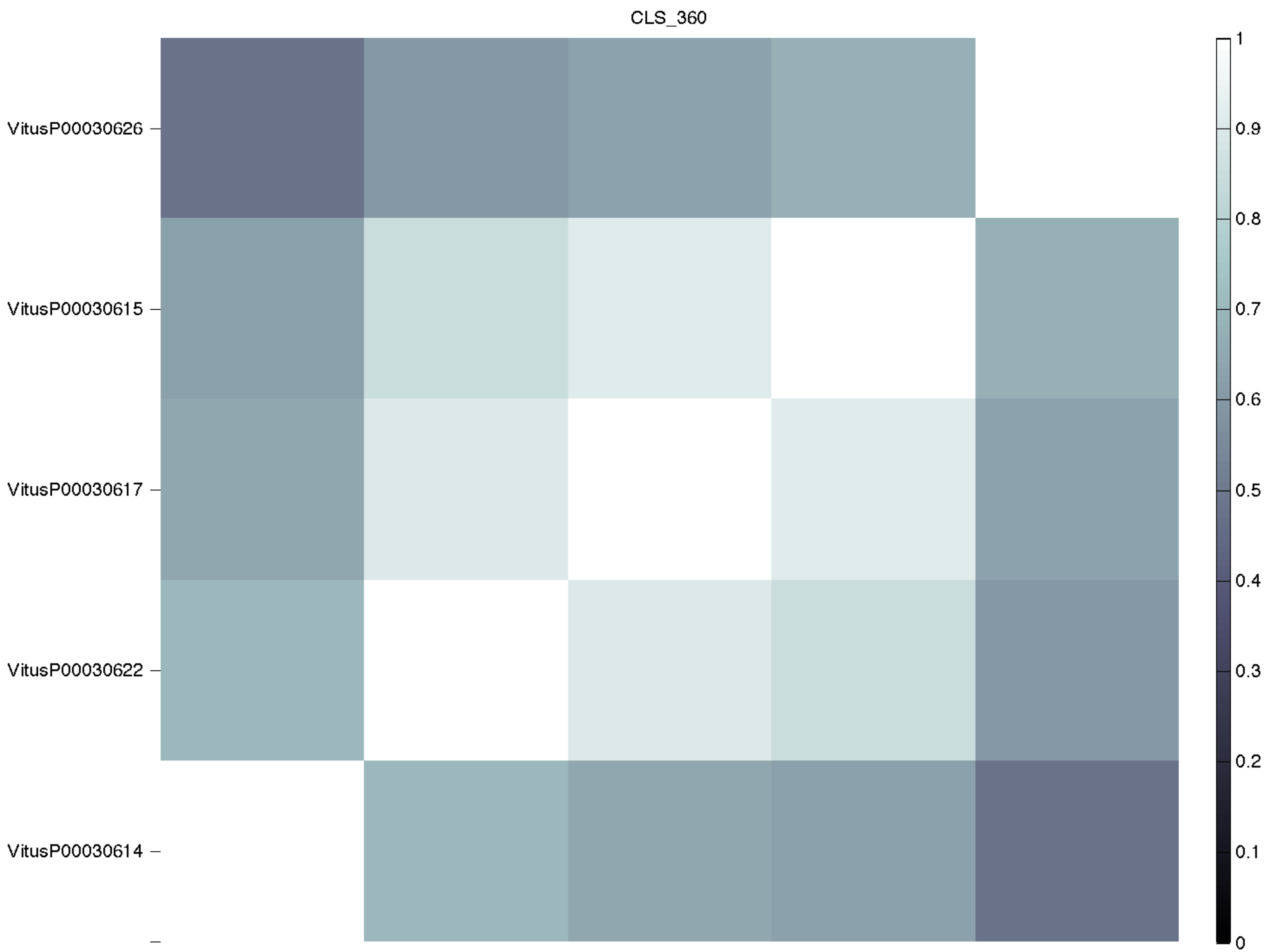
CLS_360
VitusP00030626
VitusP00030615
VitusP00030617
VitusP00030622
VitusP00030614
1
0.9
0.8
0.7
0.6
0.5
0.4
0.3
0.2
0.1
0

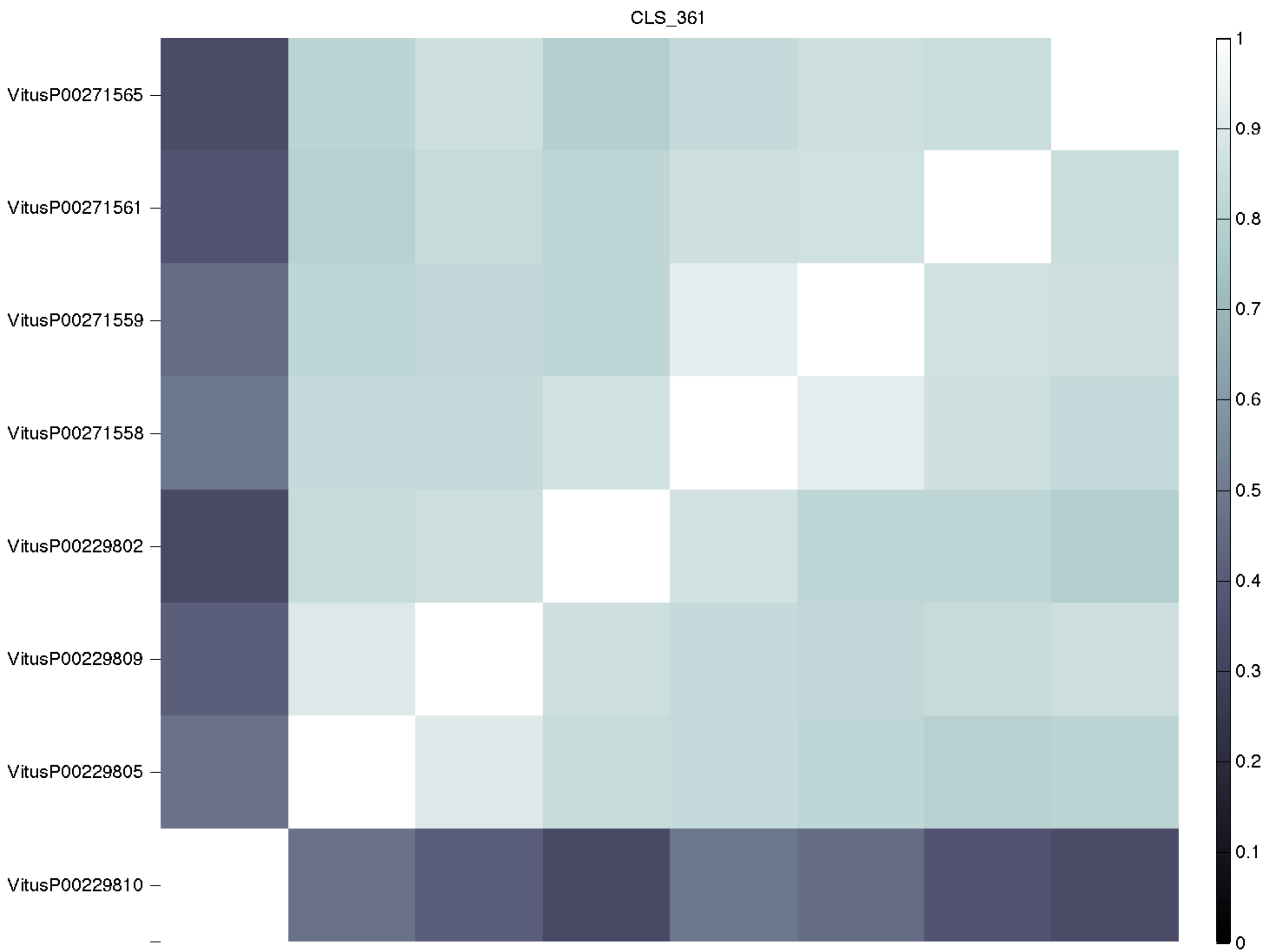
CLS_361
VitusP00271565
VitusP00271561
VitusP00271559
VitusP00271558
VitusP00229802
VitusP00229809
VitusP00229805
VitusP00229810
1
0.9
0.8
0.7
0.6
0.5
0.4
0.3
0.2
0.1
0

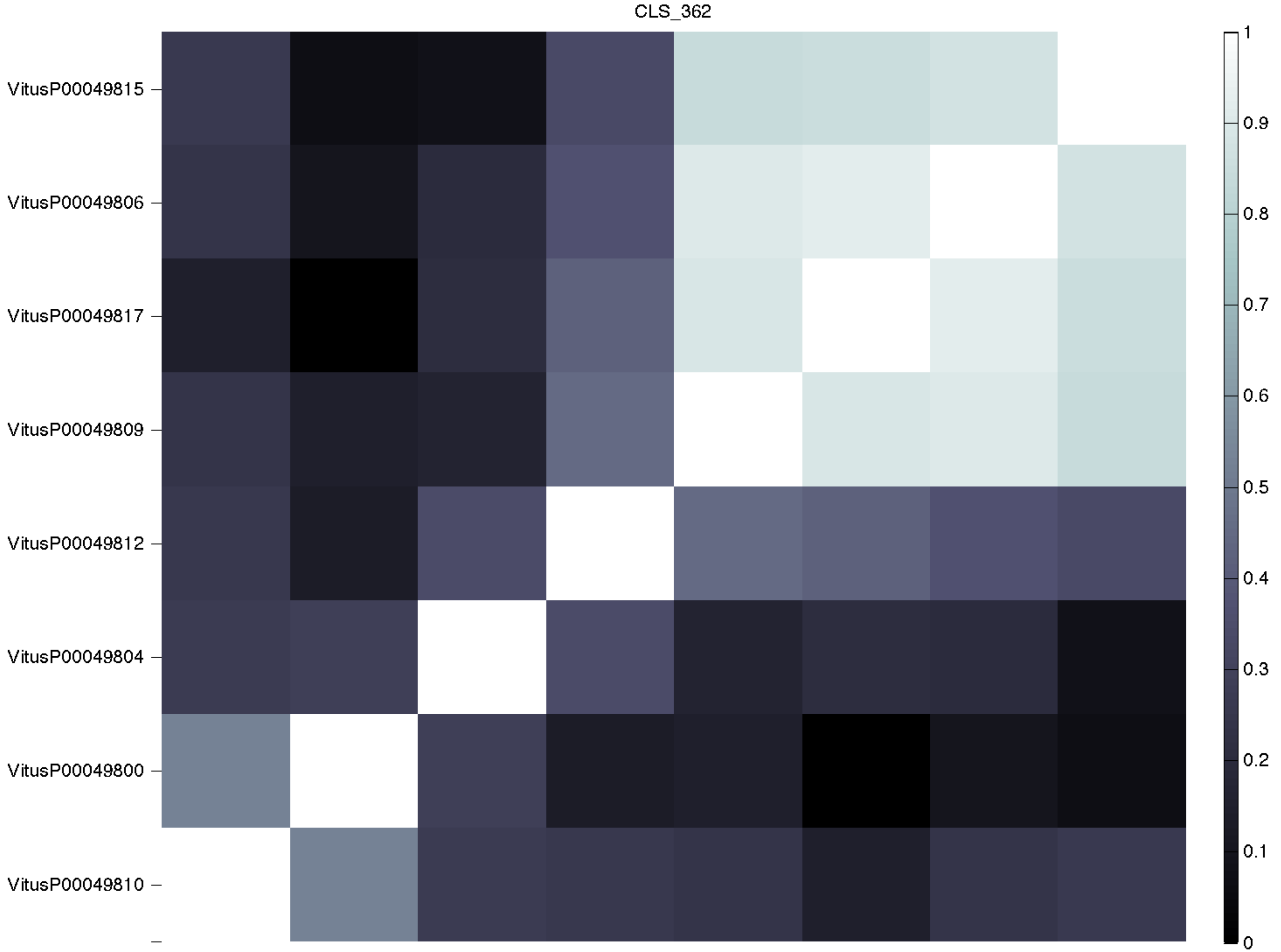
CLS_362
VitusP00049815
VitusP00049806
VitusP00049817
VitusP00049809
VitusP00049812
VitusP00049804
VitusP00049800
VitusP00049810
1
0.9
0.8
0.7
0.6
0.5
0.4
0.3
0.2
0.1
0

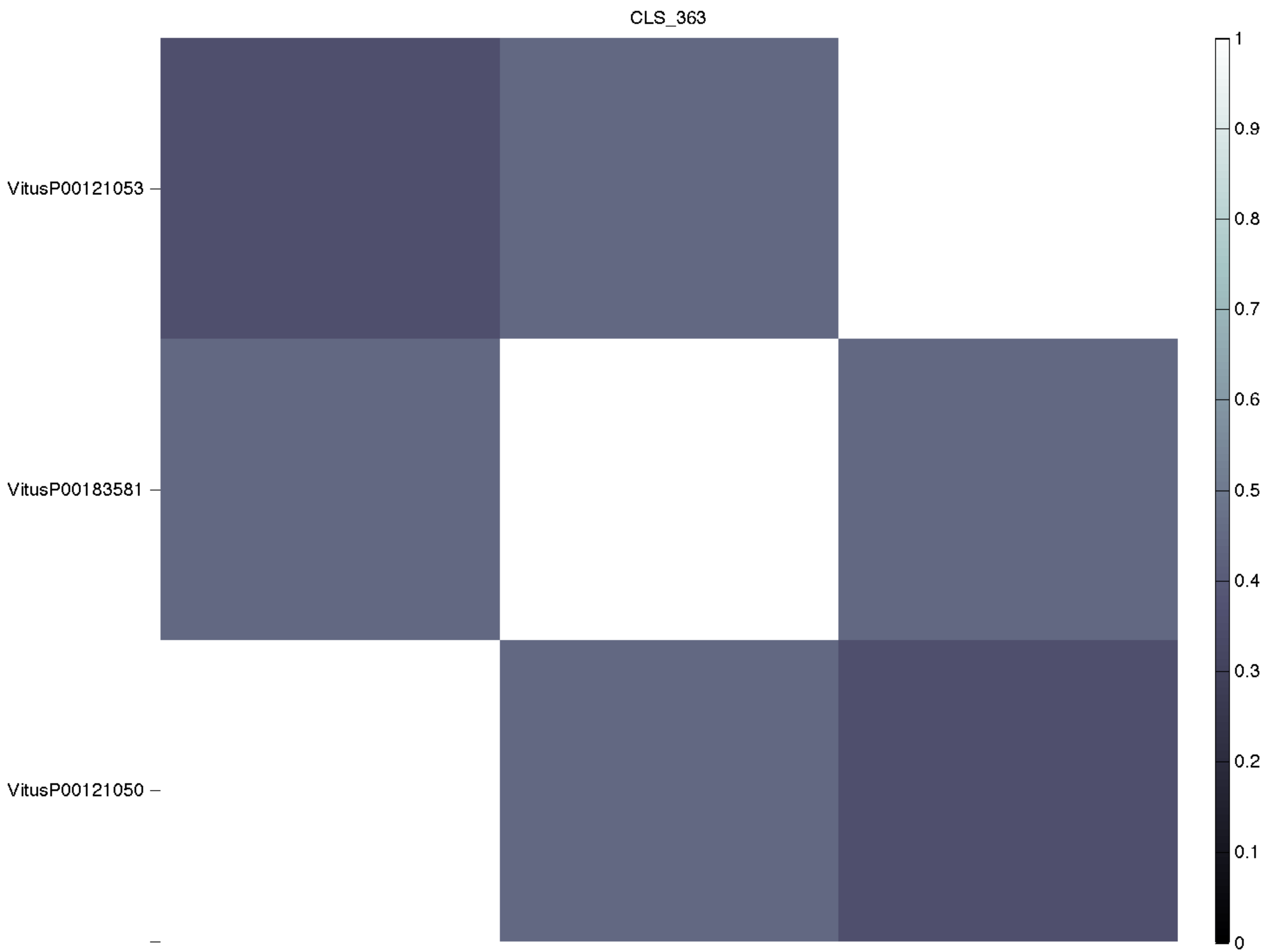
CLS_363
VitusP00121053
VitusP00183581
VitusP00121050
1
0.9
0.8
0.7
0.6
0.5
0.4
0.3
0.2
0.1
0

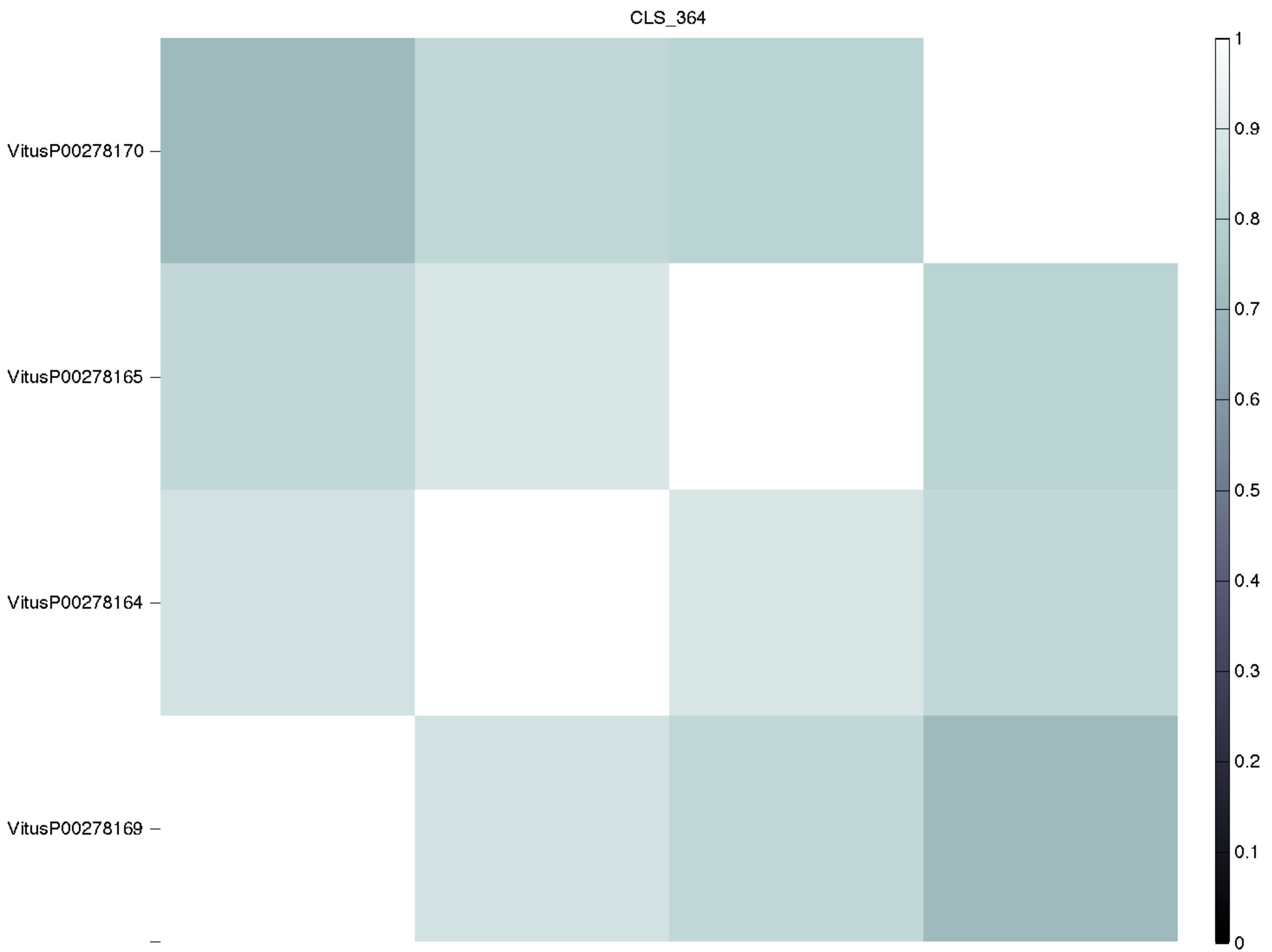
CLS_364
VitusP00278170
VitusP00278165
VitusP00278164
VitusP00278169
1
0.9
0.8
0.7
0.6
0.5
0.4
0.3
0.2
0.1
0

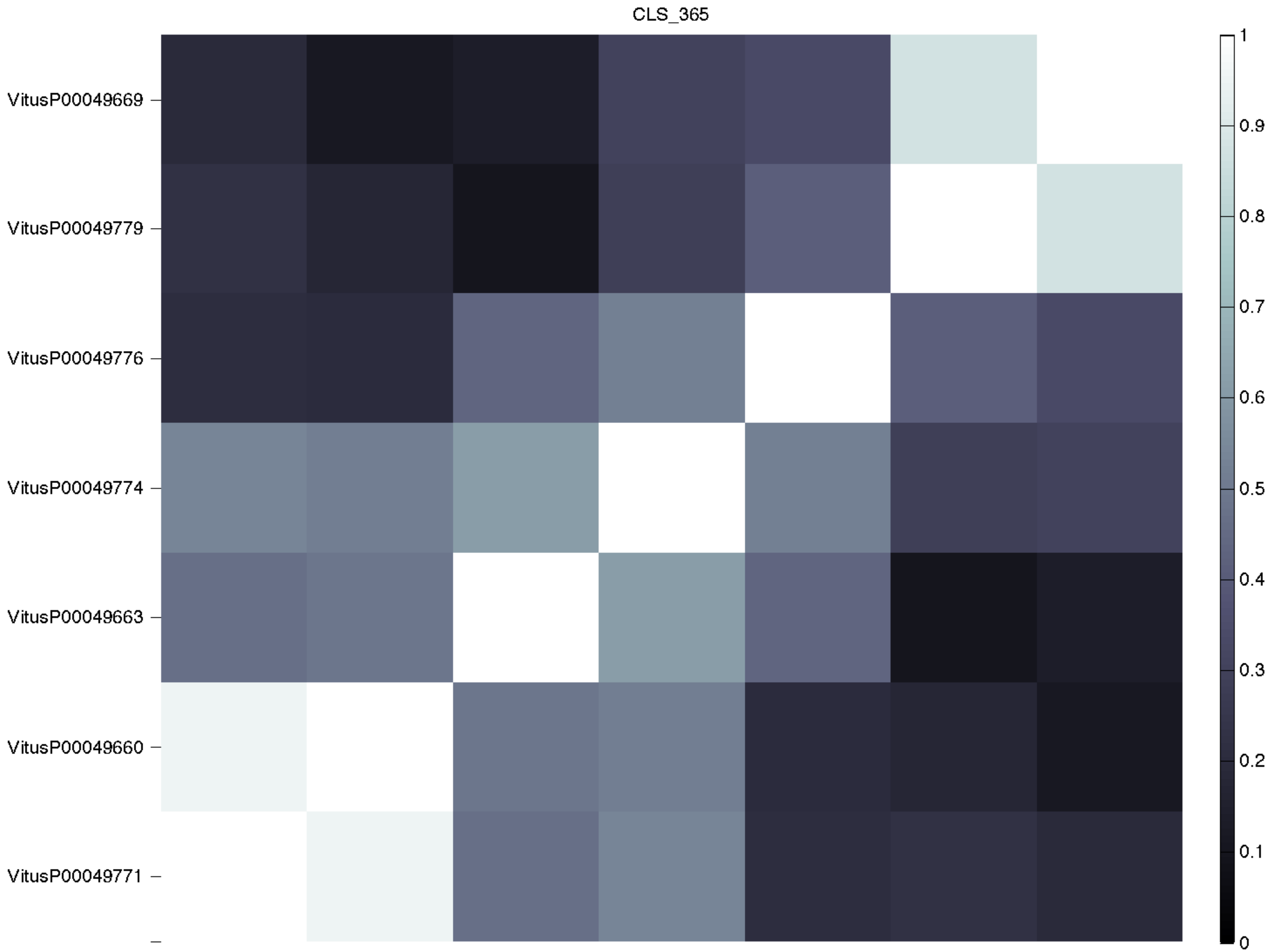
CLS_365
VitusP00049669
VitusP00049779
VitusP00049776
VitusP00049774
VitusP00049663
VitusP00049660
VitusP00049771
1
0.9
0.8
0.7
0.6
0.5
0.4
0.3
0.2
0.1
0

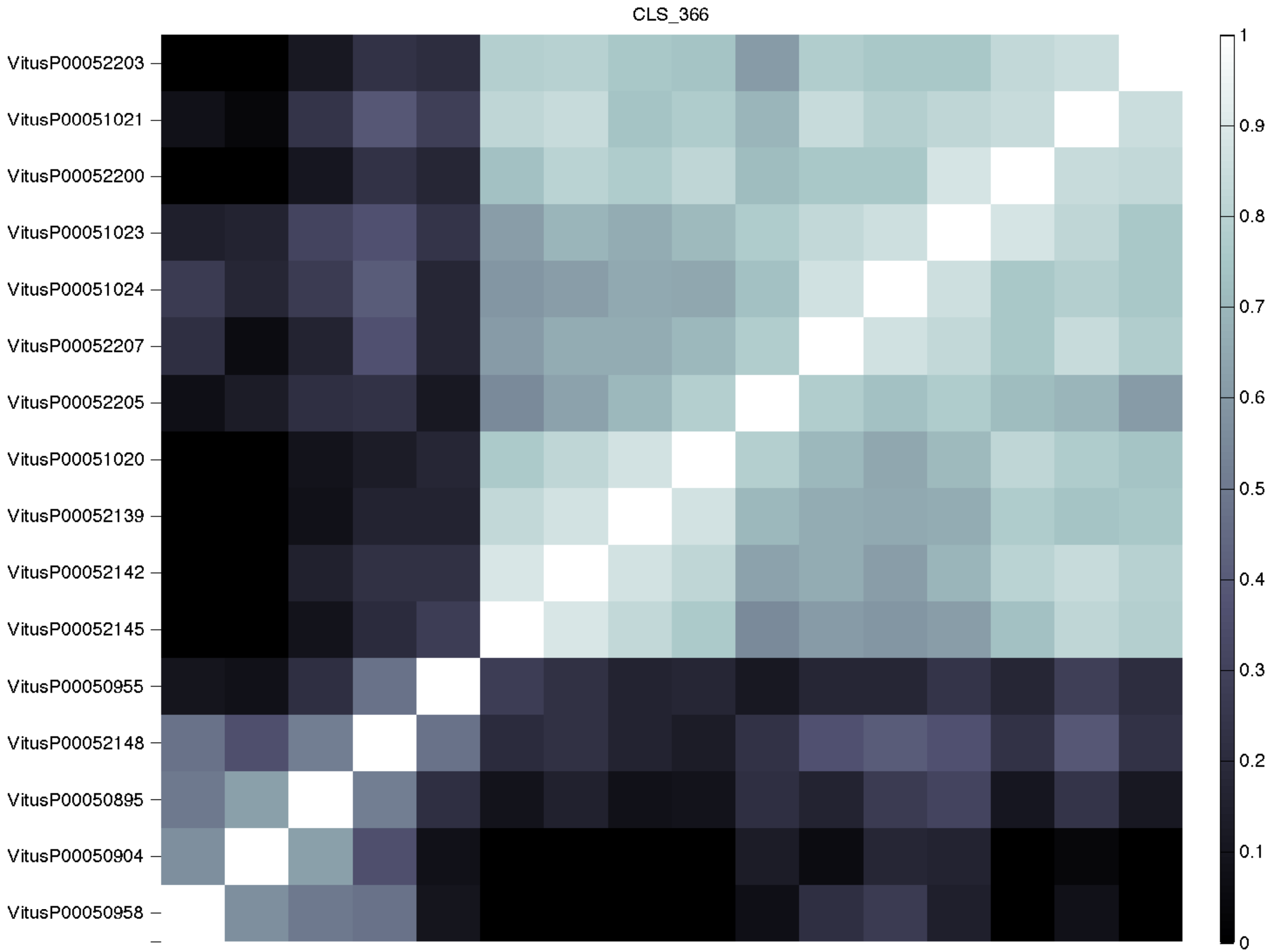
CLS_366
VitusP00052203
VitusP00051021
VitusP00052200
VitusP00051023
VitusP00051024
VitusP00052207
VitusP00052205
VitusP00051020
VitusP00052139
VitusP00052142
VitusP00052145
VitusP00050955
VitusP00052148
VitusP00050895
VitusP00050904
VitusP00050958
1
0.9
0.8
0.7
0.6
0.5
0.4
0.3
0.2
0.1
0

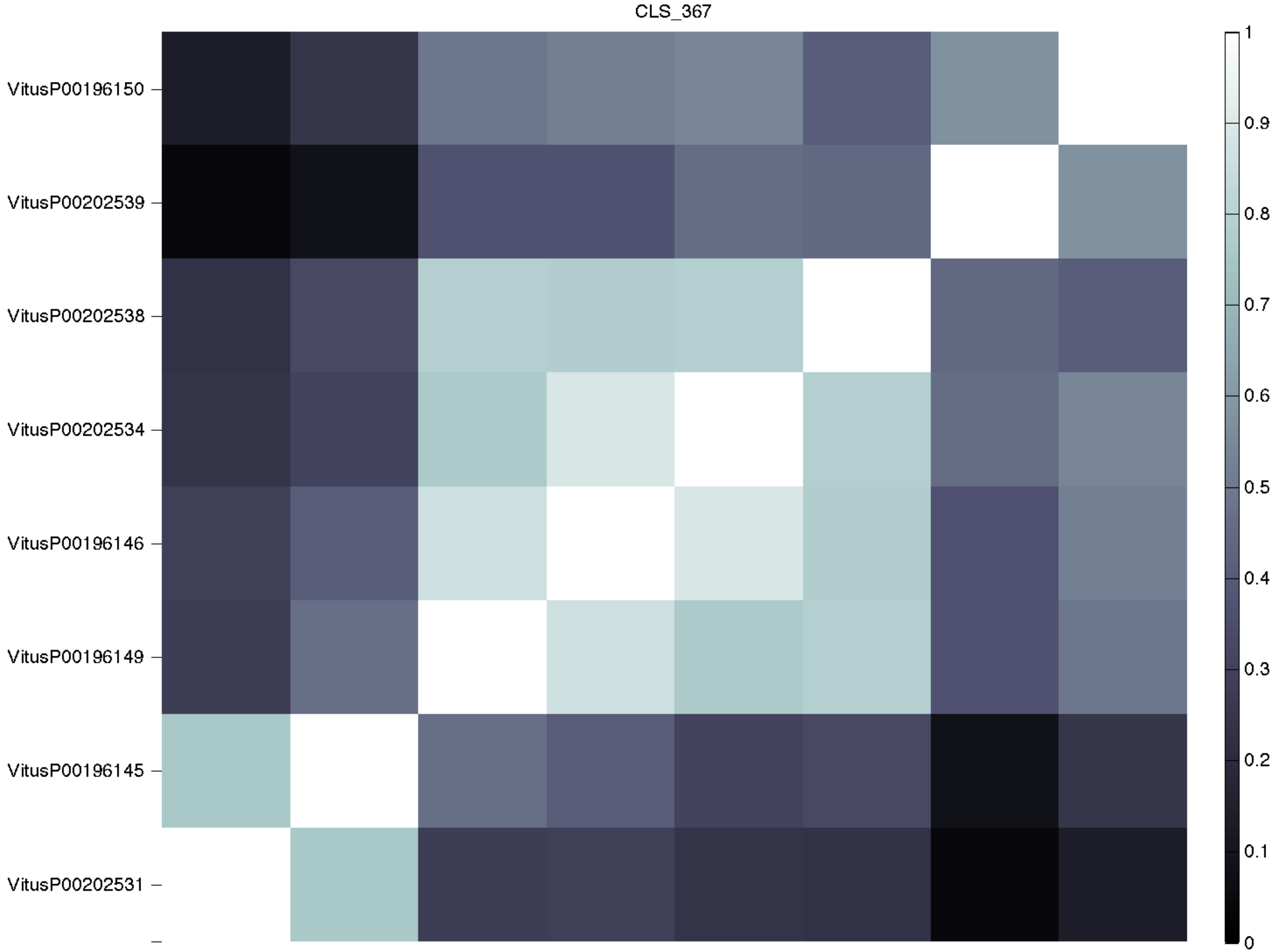
CLS_367
VitusP00196150
VitusP00202539
VitusP00202538
VitusP00202534
VitusP00196146
VitusP00196149
VitusP00196145
VitusP00202531
1
0.9
0.8
0.7
0.6
0.5
0.4
0.3
0.2
0.1
0

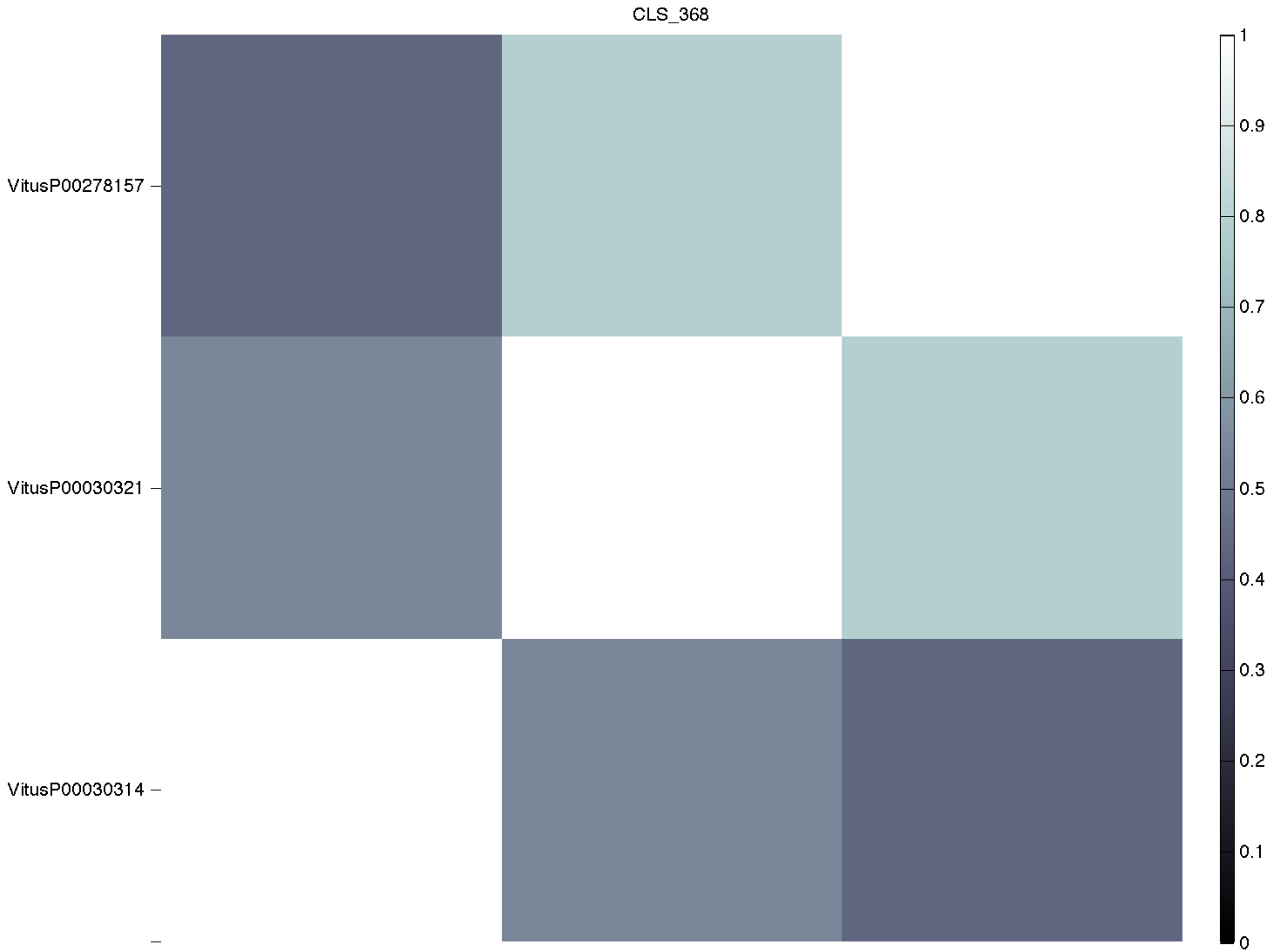
CLS_368
VitusP00278157
VitusP00030321
VitusP00030314
1
0.9
0.8
0.7
0.6
0.5
0.4
0.3
0.2
0.1
0

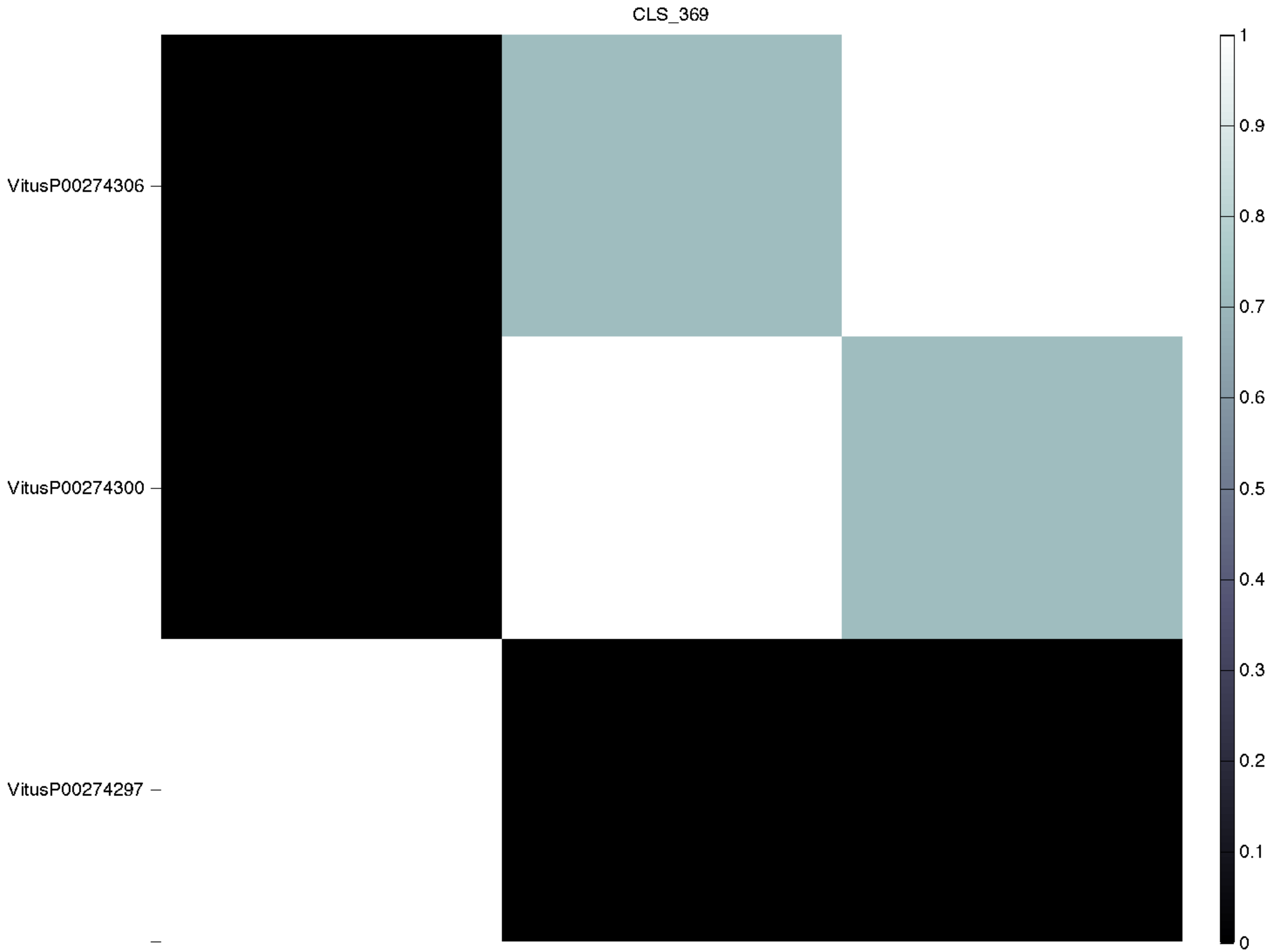
CLS_369
VitusP00274306
VitusP00274300
VitusP00274297
1
0.9
0.8
0.7
0.6
0.5
0.4
0.3
0.2
0.1
0

CLS_370

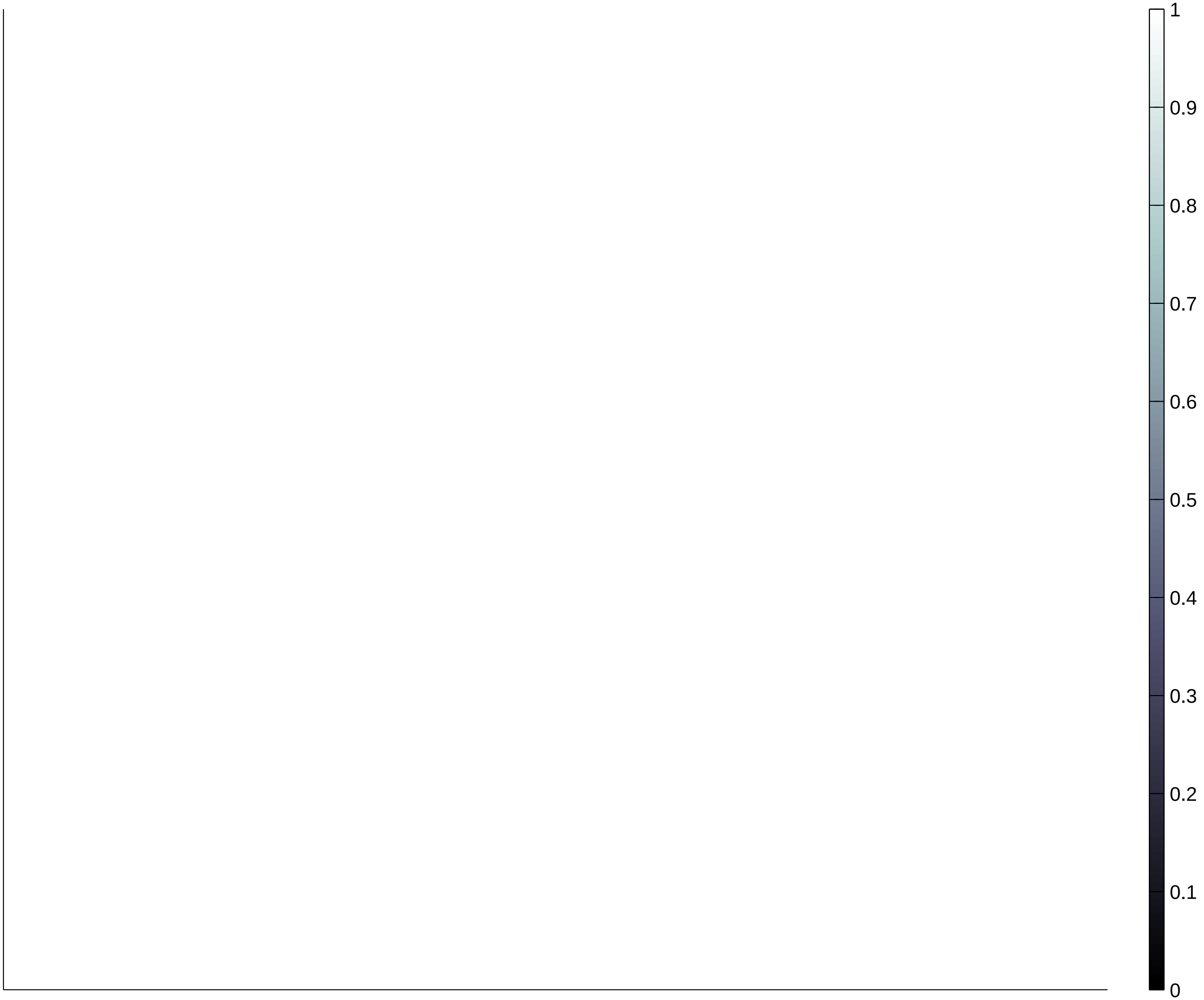

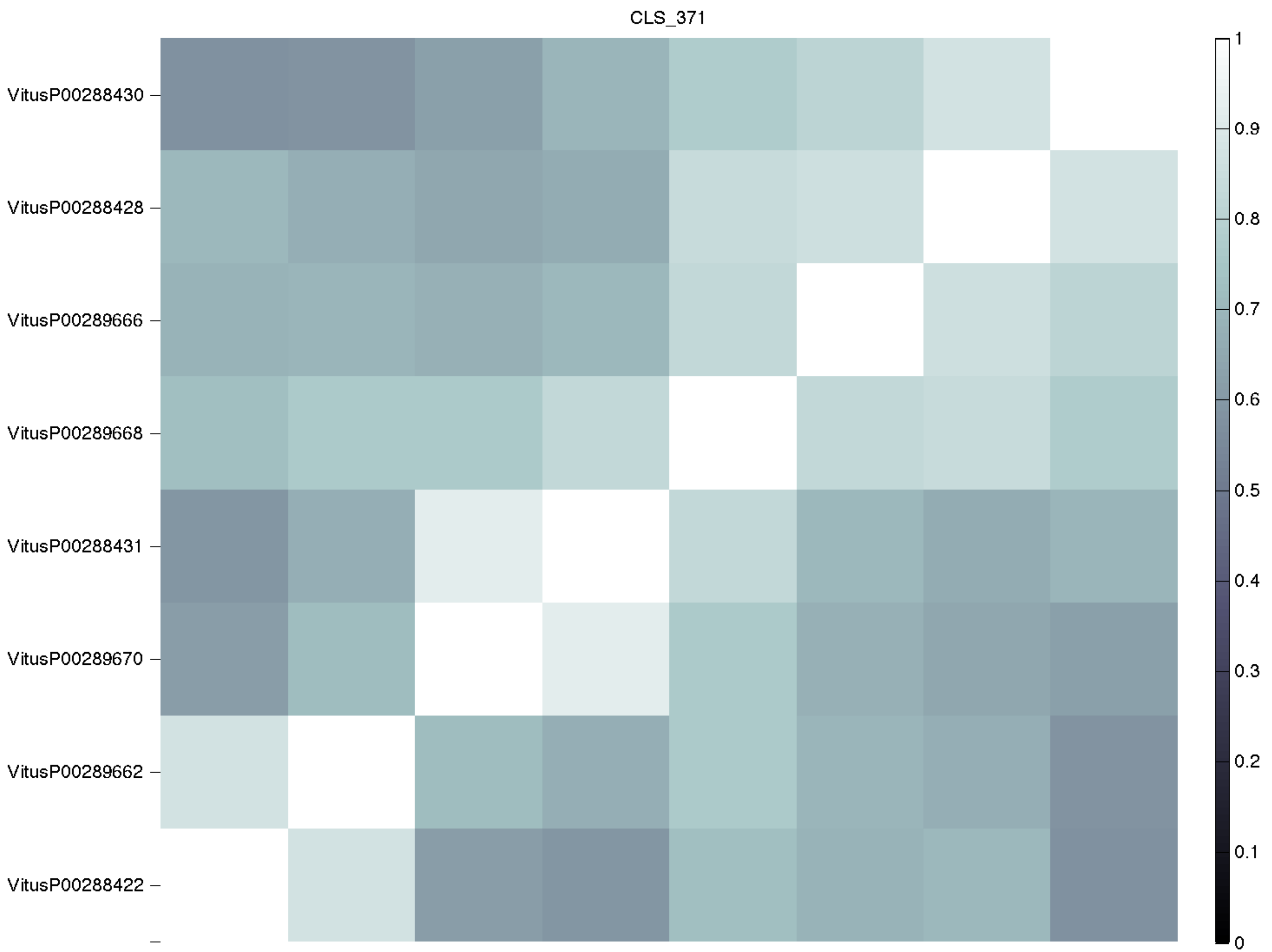

CLS_371
VitusP00288430
VitusP00288428
VitusP00289666
VitusP00289668
VitusP00288431
VitusP00289670
VitusP00289662
VitusP00288422
1
0.9
0.8
0.7
0.6
0.5
0.4
0.3
0.2
0.1
0

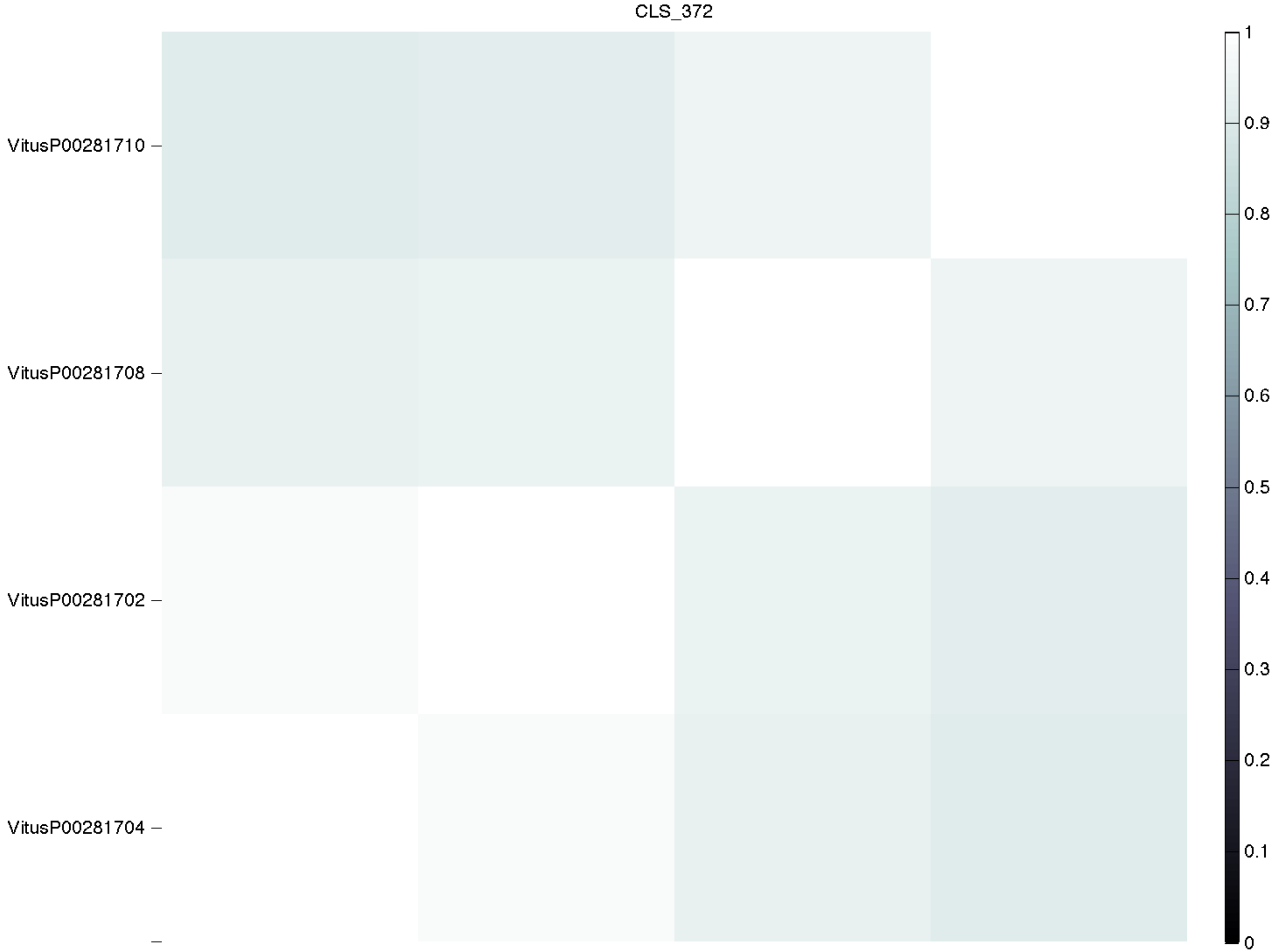

CLS_372
VitusP00281710
VitusP00281708
VitusP00281702
VitusP00281704
1
0.9
0.8
0.7
0.6
0.5
0.4
0.3
0.2
0.1
0

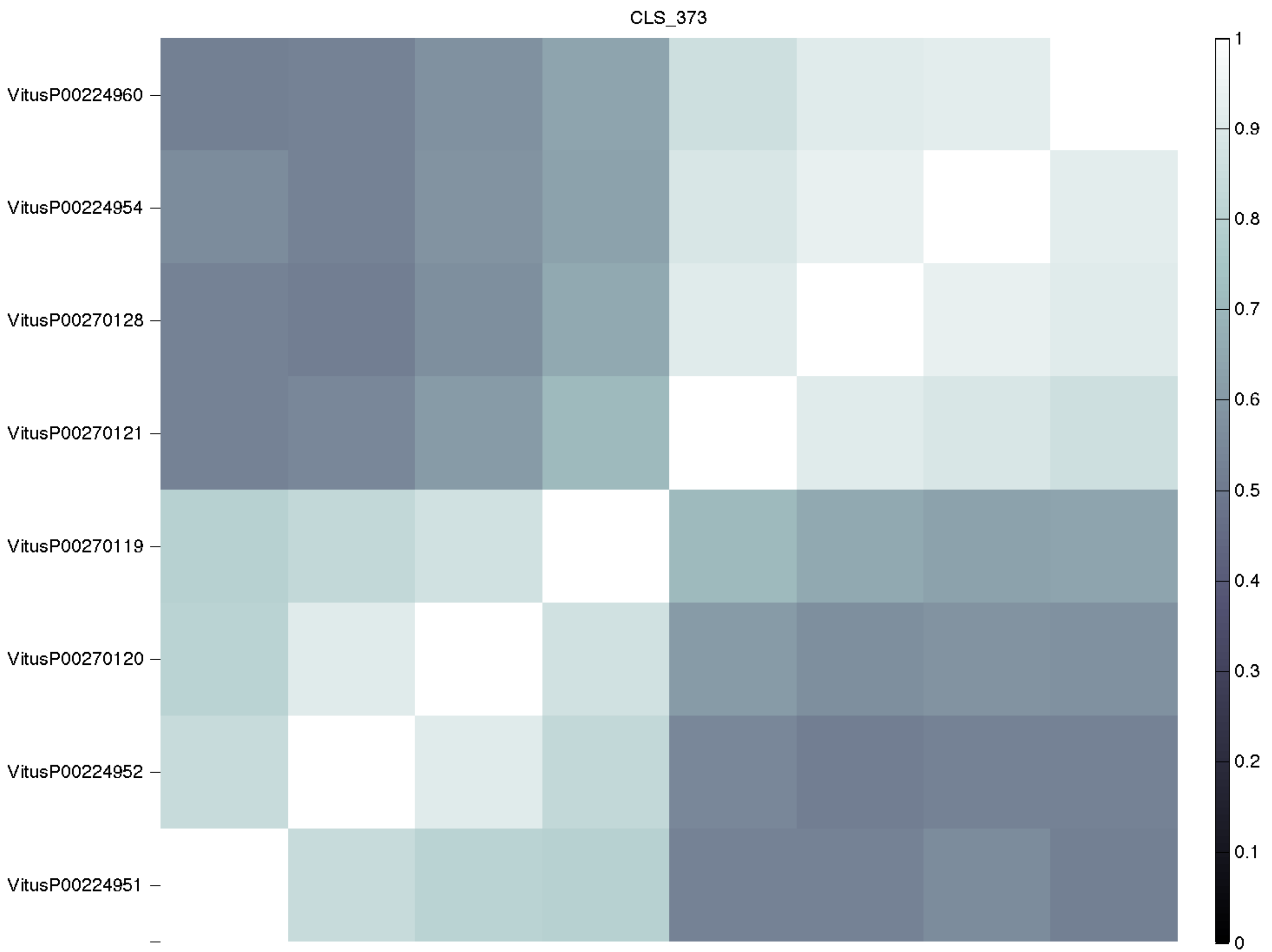
CLS_373
VitusP00224960
VitusP00224954
VitusP00270128
VitusP00270121
VitusP00270119
VitusP00270120
VitusP00224952
VitusP00224951
1
0.9
0.8
0.7
0.6
0.5
0.4
0.3
0.2
0.1
0

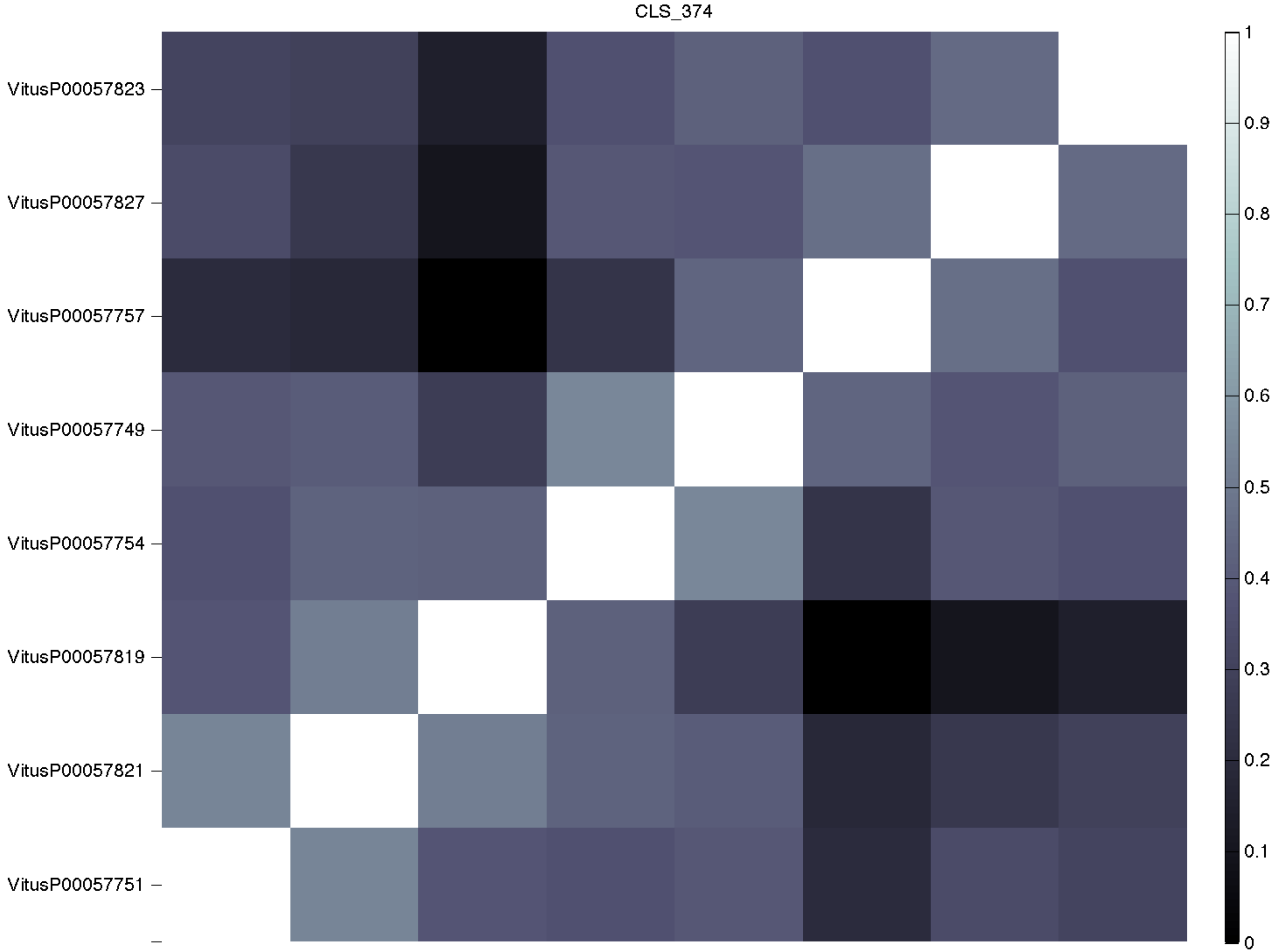
CLS_374
VitusP00057823
VitusP00057827
VitusP00057757
VitusP00057749
VitusP00057754
VitusP00057819
VitusP00057821
VitusP00057751
1
0.9
0.8
0.7
0.6
0.5
0.4
0.3
0.2
0.1
0

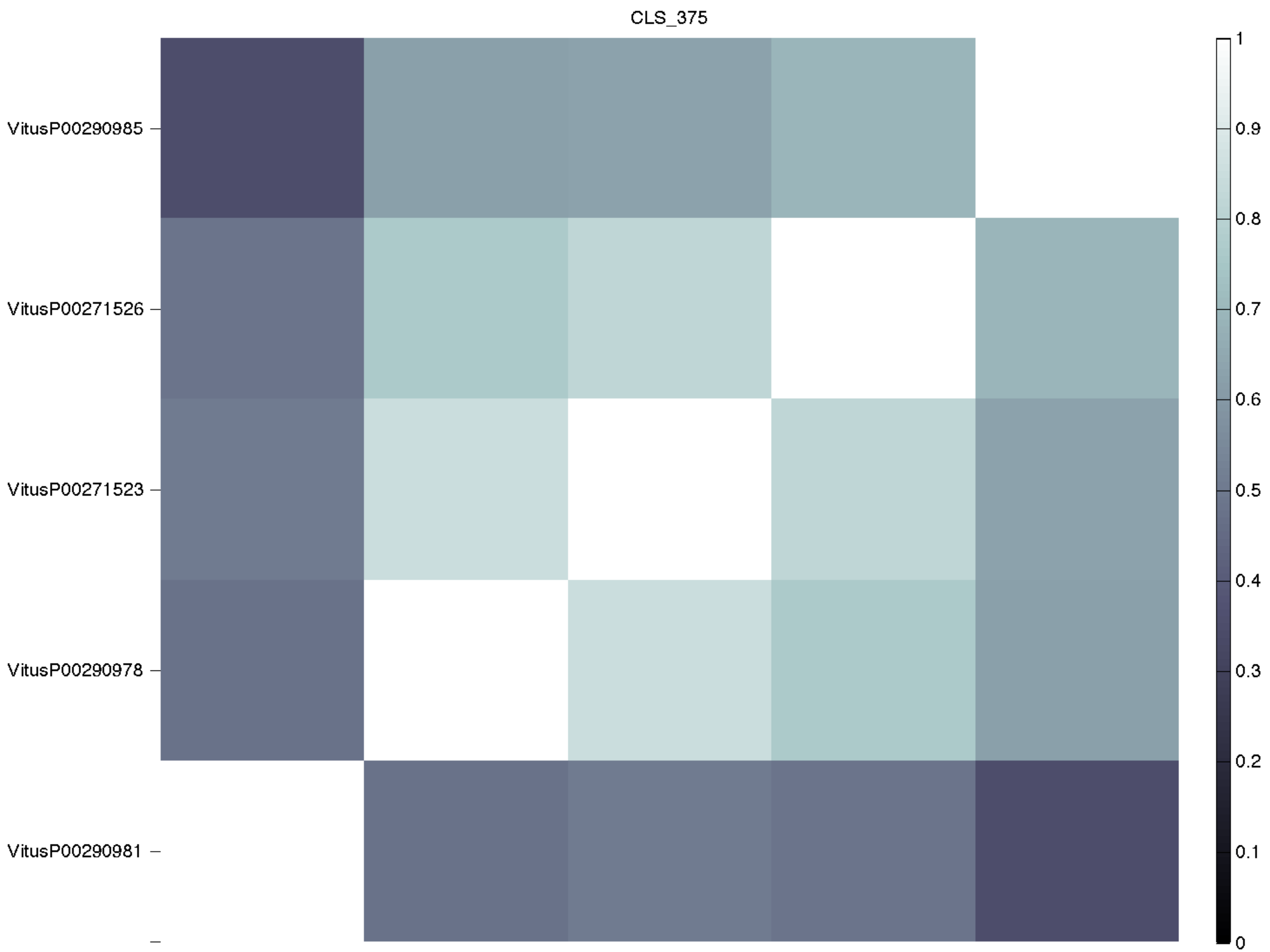
CLS_375
VitusP00290985
VitusP00271526
VitusP00271523
VitusP00290978
VitusP00290981
1
0.9
0.8
0.7
0.6
0.5
0.4
0.3
0.2
0.1
0

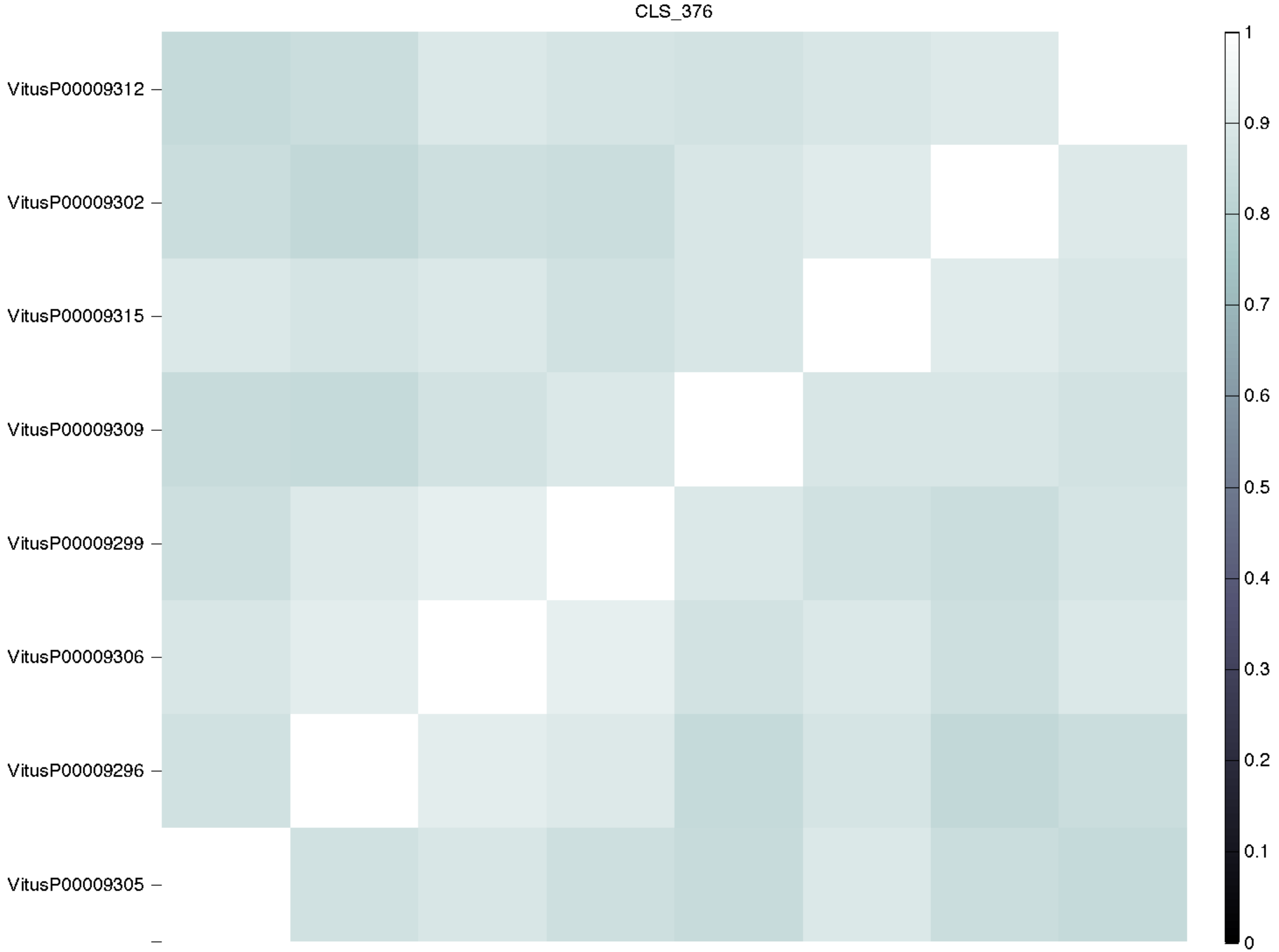
CLS_376
VitusP00009312
VitusP00009302
VitusP00009315
VitusP00009309
VitusP00009299
VitusP00009306
VitusP00009296
VitusP00009305
1
0.9
0.8
0.7
0.6
0.5
0.4
0.3
0.2
0.1
0

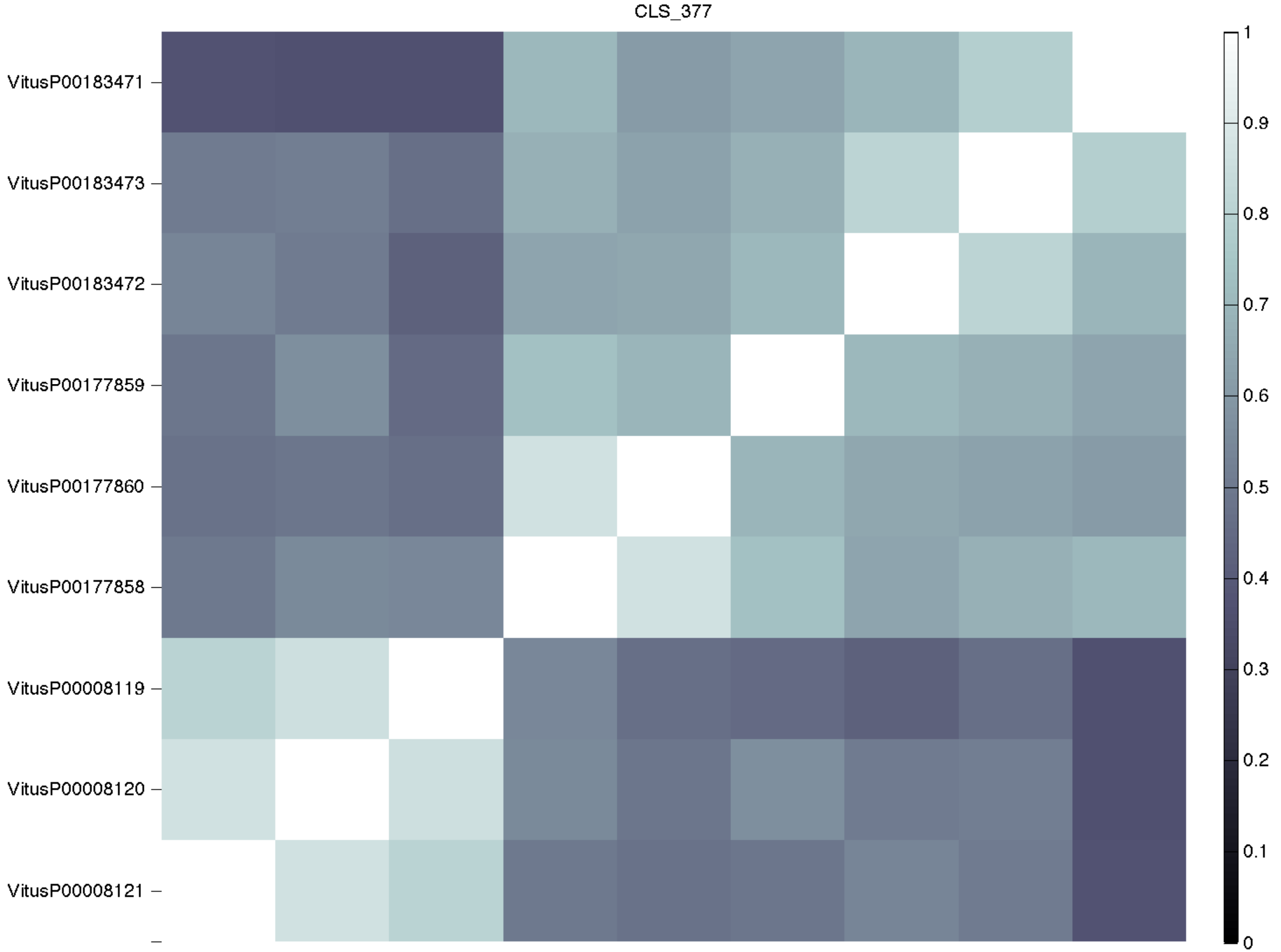
CLS_377
VitusP00183471
VitusP00183473
VitusP00183472
VitusP00177859
VitusP00177860
VitusP00177858
VitusP00008119
VitusP00008120
VitusP00008121
1
0.9
0.8
0.7
0.6
0.5
0.4
0.3
0.2
0.1
0

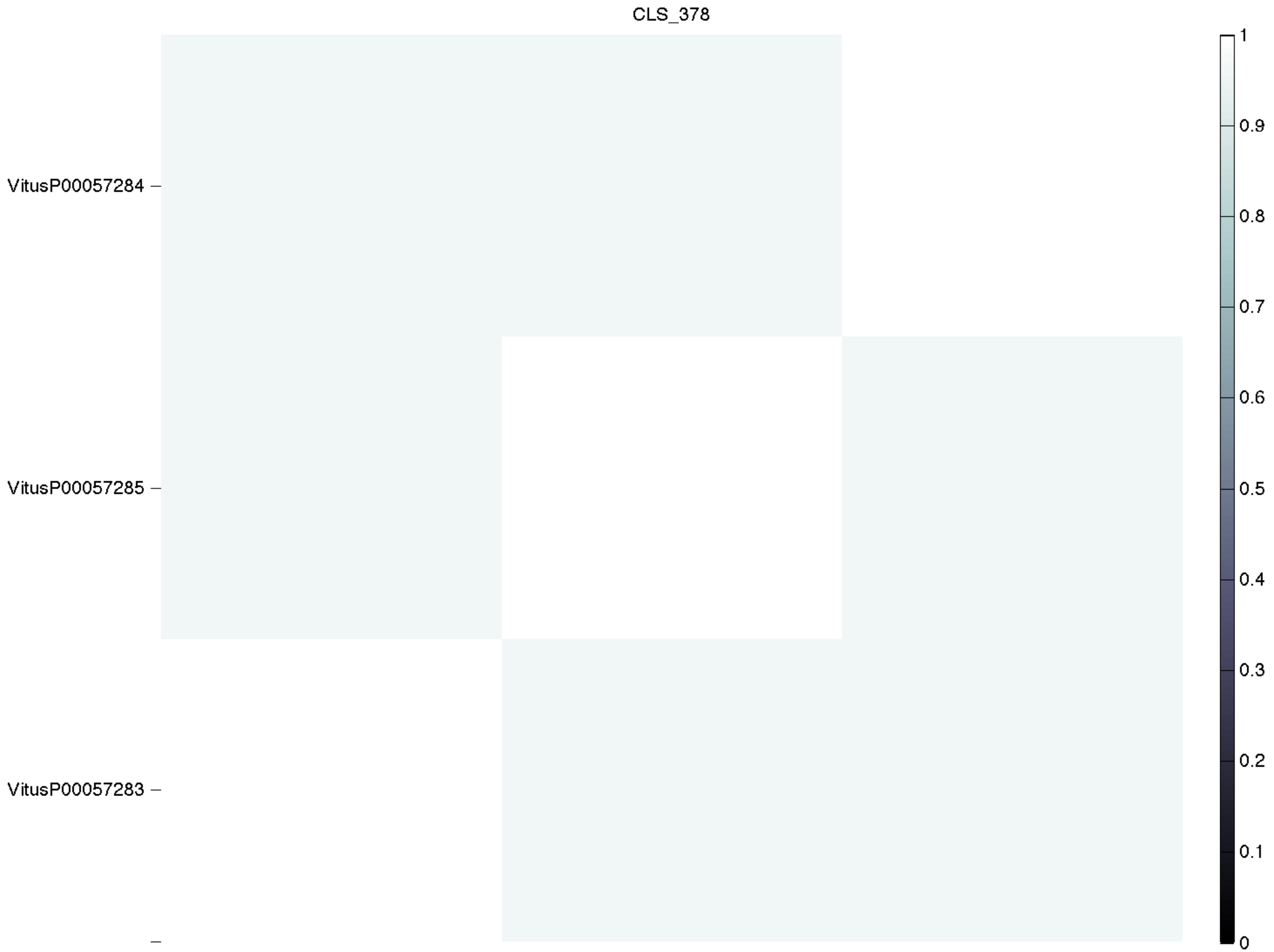
CLS_378
VitusP00057284
VitusP00057285
VitusP00057283
1
0.9
0.8
0.7
0.6
0.5
0.4
0.3
0.2
0.1
0

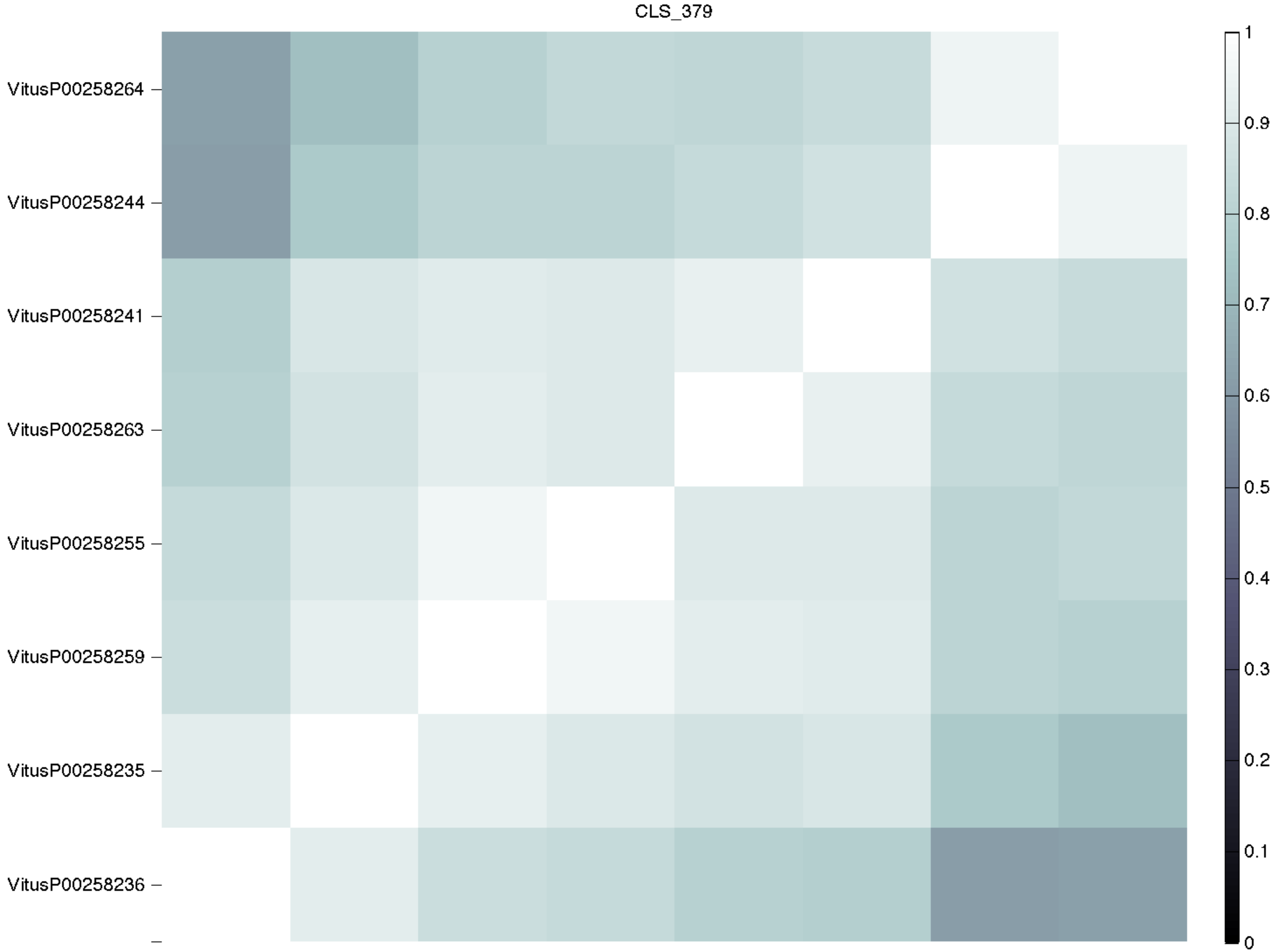
CLS_379
VitusP00258264
VitusP00258244
VitusP00258241
VitusP00258263
VitusP00258255
VitusP00258259
VitusP00258235
VitusP00258236
1
0.9
0.8
0.7
0.6
0.5
0.4
0.3
0.2
0.1
0

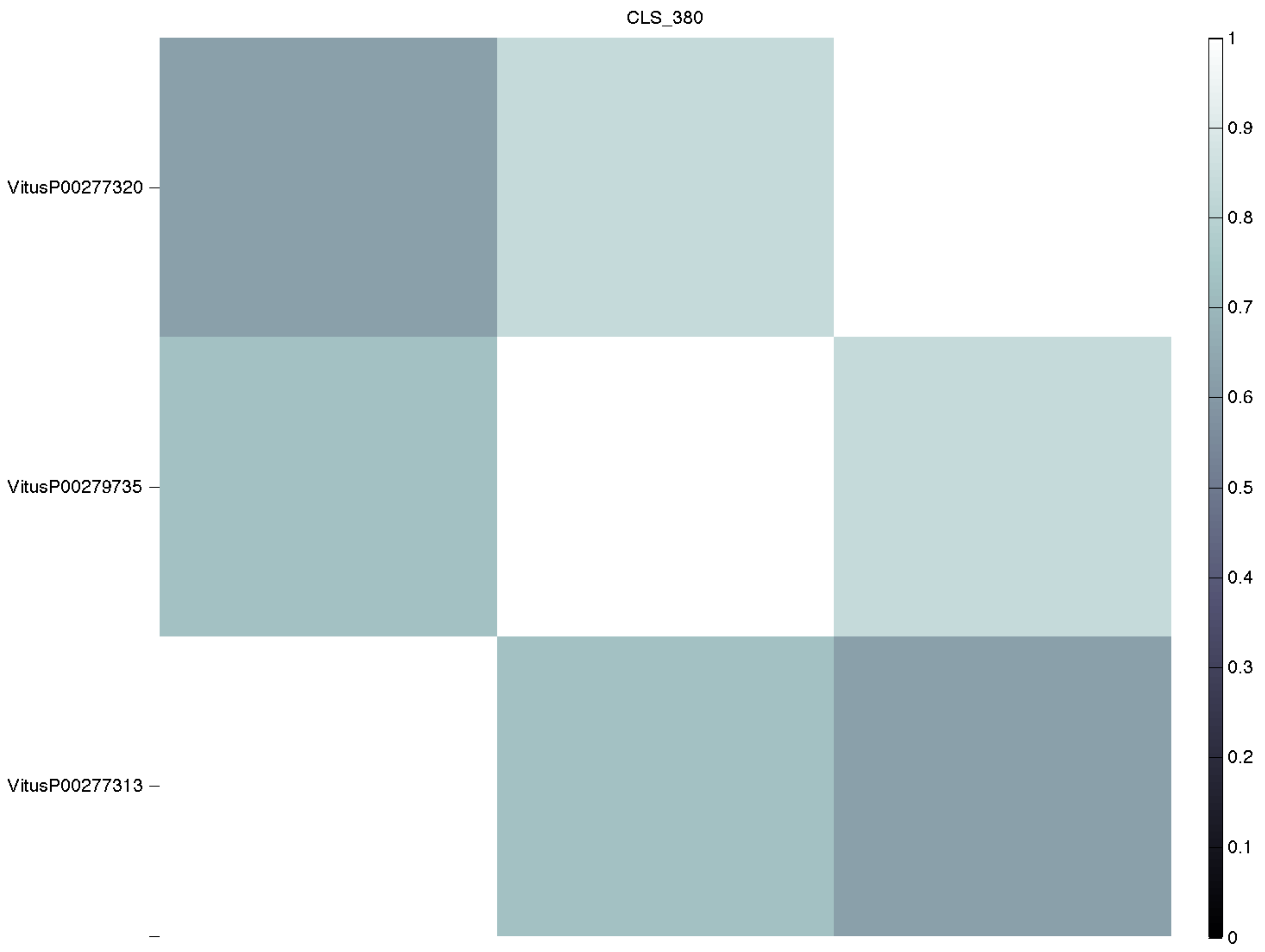
CLS_380
VitusP00277320
VitusP00279735
VitusP00277313
1
0.9
0.8
0.7
0.6
0.5
0.4
0.3
0.2
0.1
0

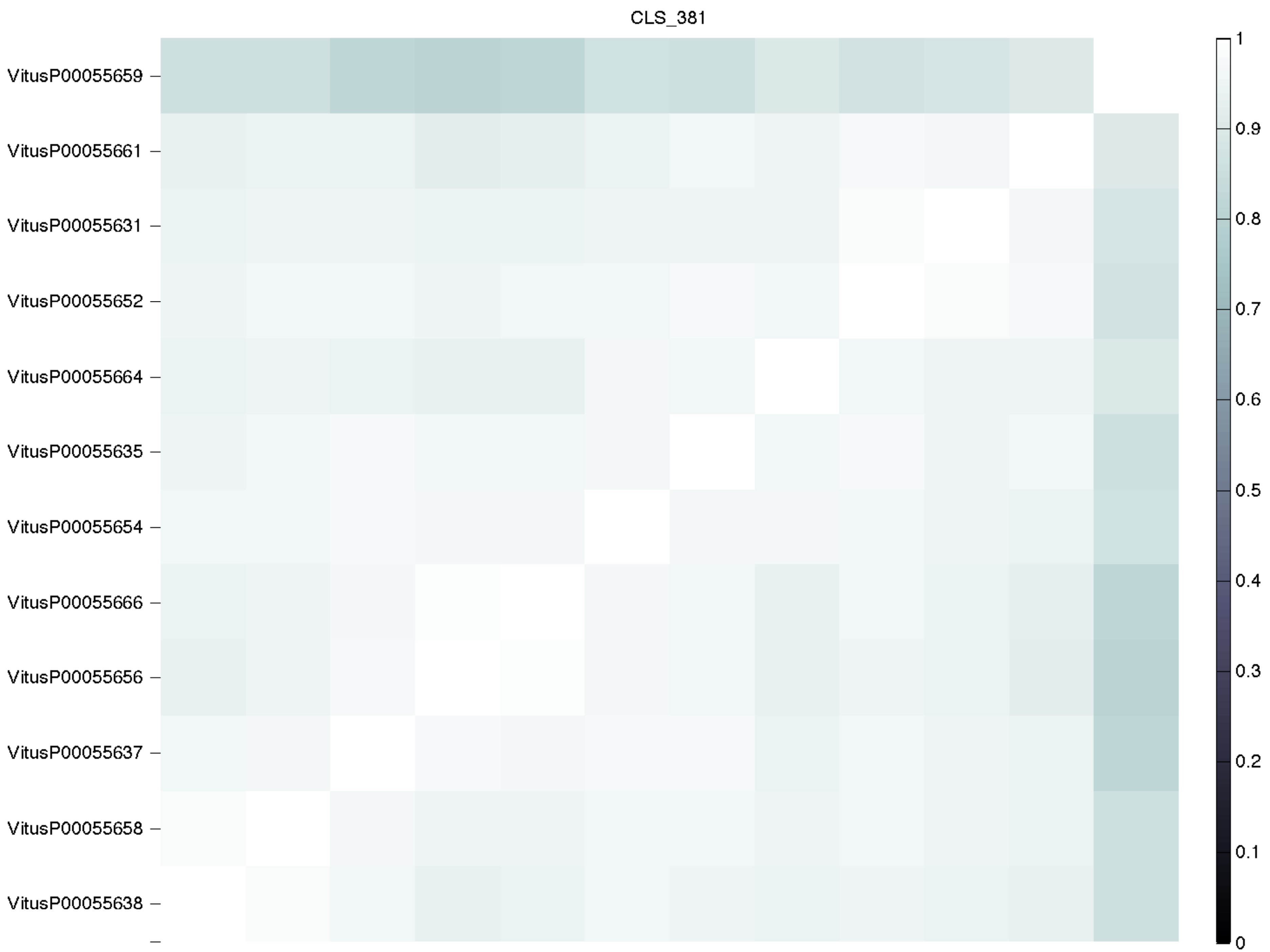
CLS_381
VitusP00055659
VitusP00055661
VitusP00055631
VitusP00055652
VitusP00055664
VitusP00055635
VitusP00055654
VitusP00055666
VitusP00055656
VitusP00055637
VitusP00055658
VitusP00055638
1
0.9
0.8
0.7
0.6
0.5
0.4
0.3
0.2
0.1
0

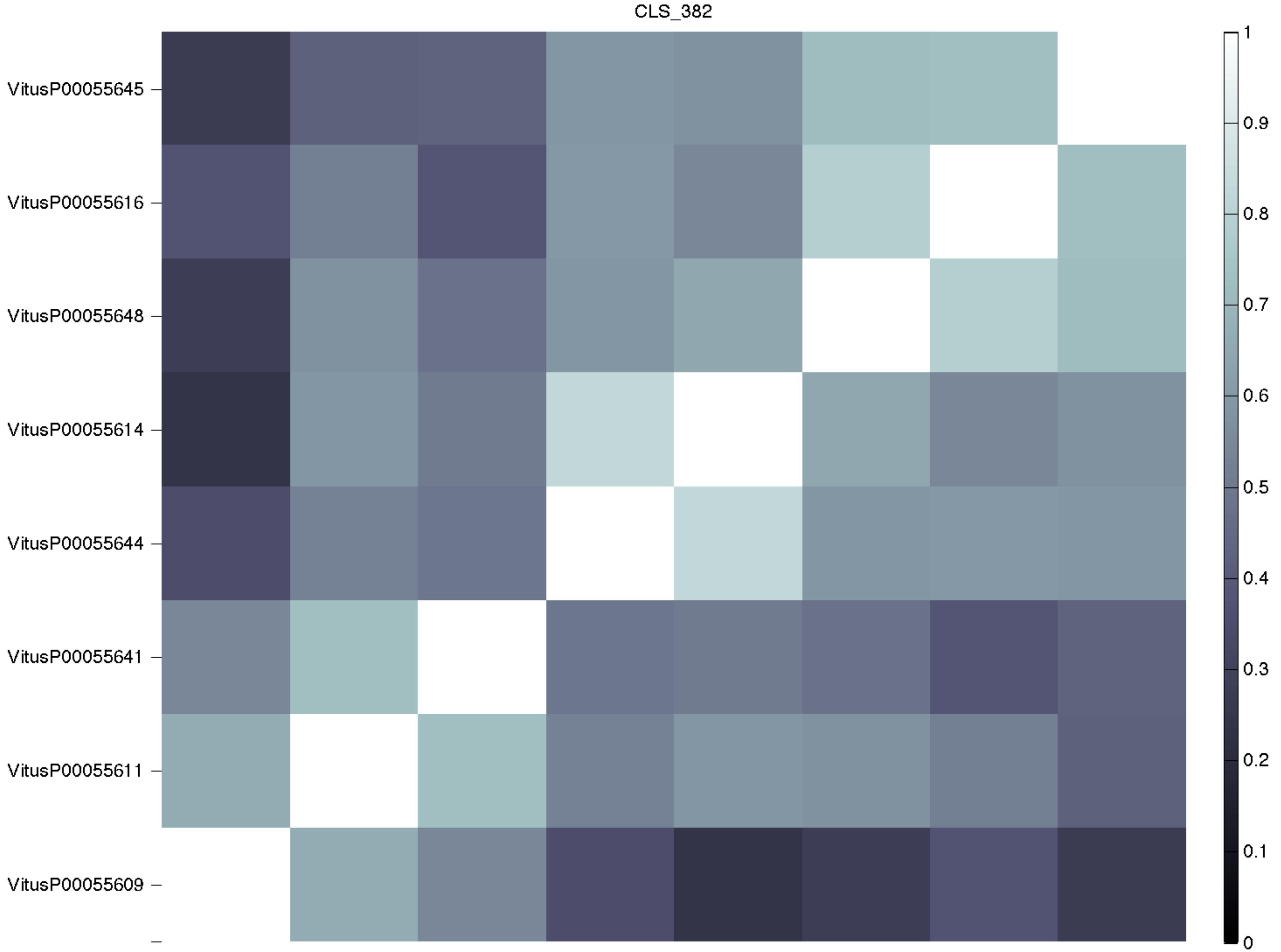
CLS_382
VitusP00055645
VitusP00055616
VitusP00055648
VitusP00055614
VitusP00055644
VitusP00055641
VitusP00055611
VitusP00055609
1
0.9
0.8
0.7
0.6
0.5
0.4
0.3
0.2
0.1
0

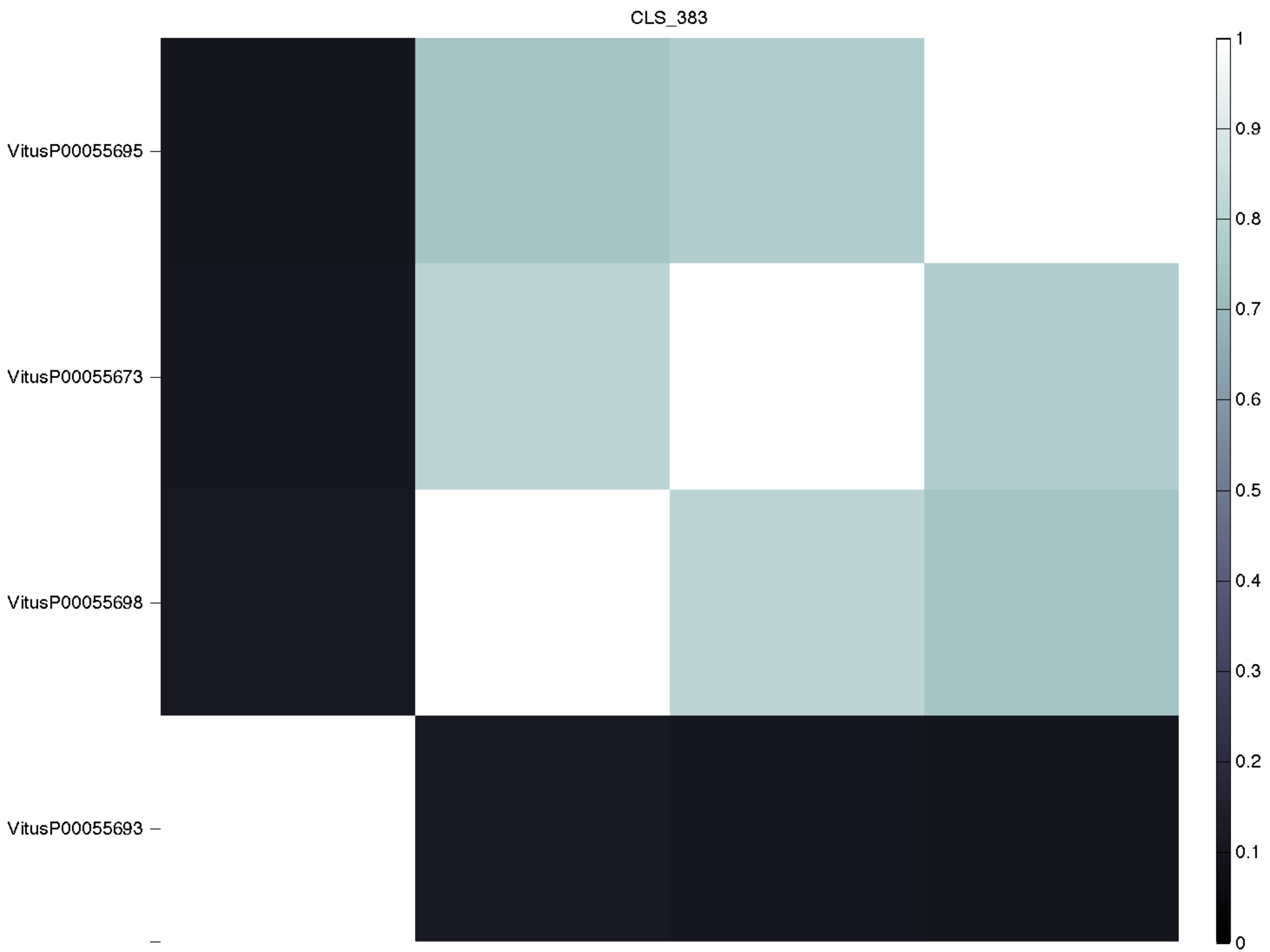
CLS_383
VitusP00055695
VitusP00055673
VitusP00055698
VitusP00055693
1
0.9
0.8
0.7
0.6
0.5
0.4
0.3
0.2
0.1
0

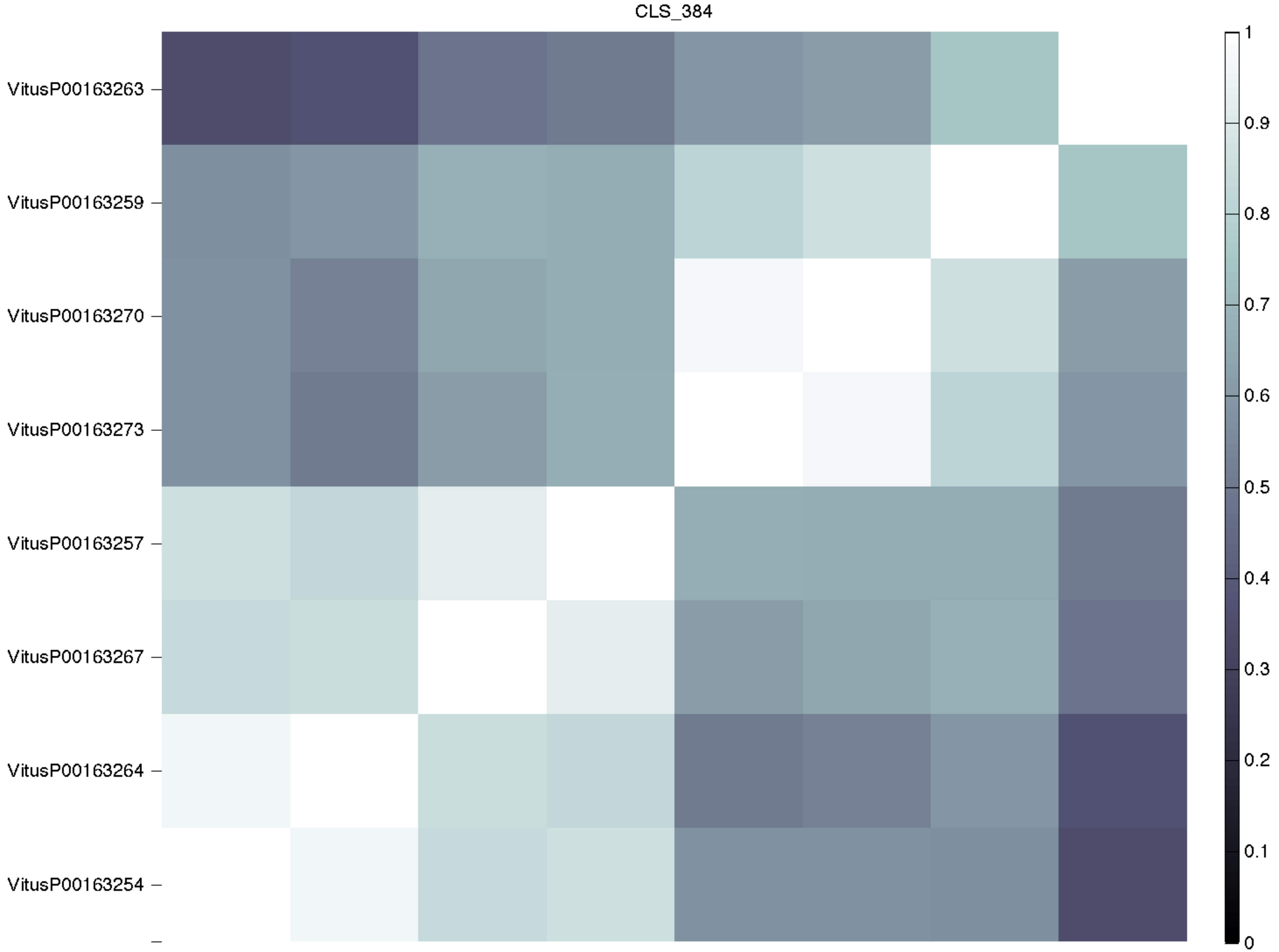
CLS_384
VitusP00163263
VitusP00163259
VitusP00163270
VitusP00163273
VitusP00163257
VitusP00163267
VitusP00163264
VitusP00163254
1
0.9
0.8
0.7
0.6
0.5
0.4
0.3
0.2
0.1
0

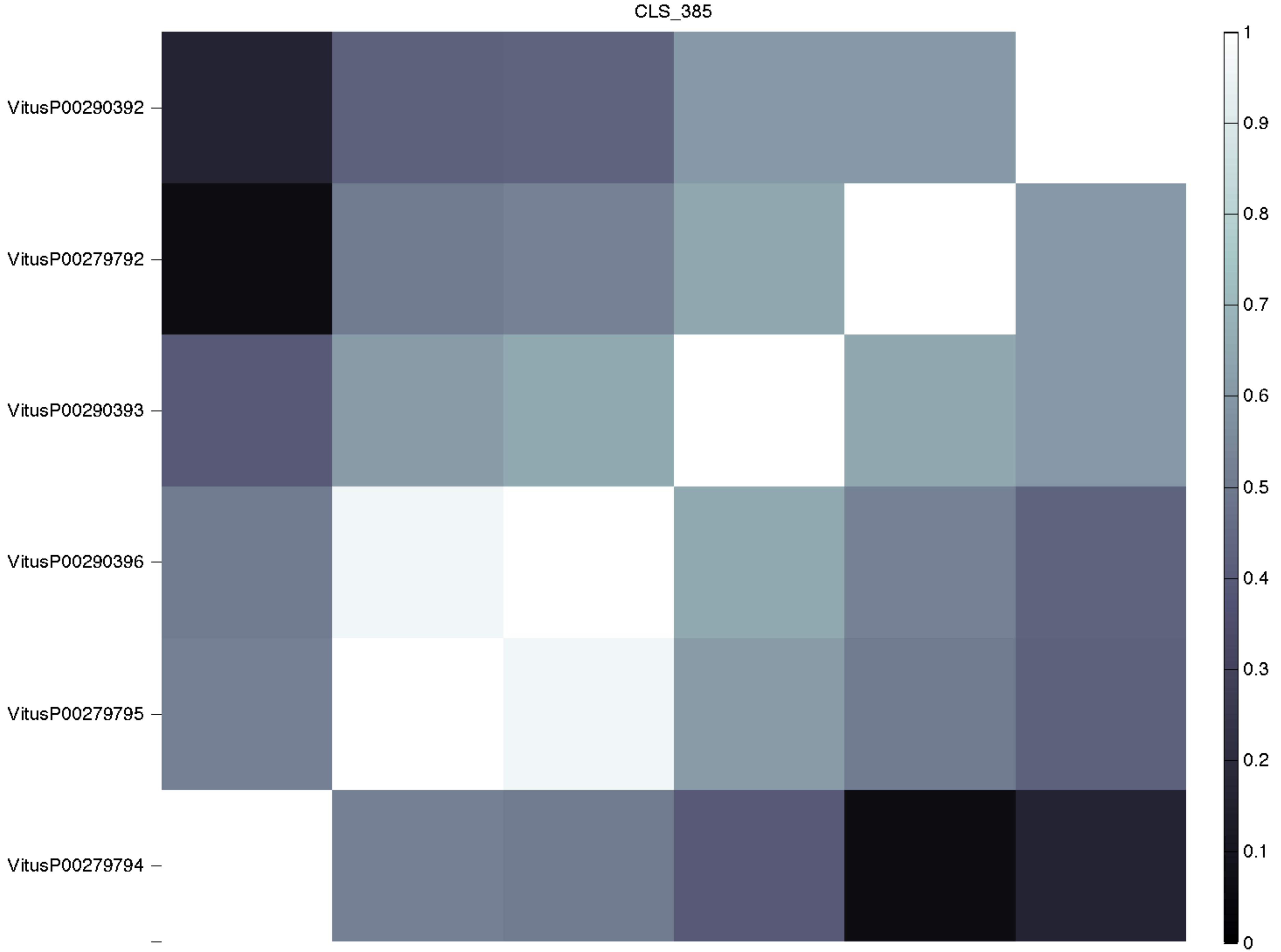
CLS_385
VitusP00290392
VitusP00279792
VitusP00290393
VitusP00290396
VitusP00279795
VitusP00279794
1
0.9
0.8
0.7
0.6
0.5
0.4
0.3
0.2
0.1
0

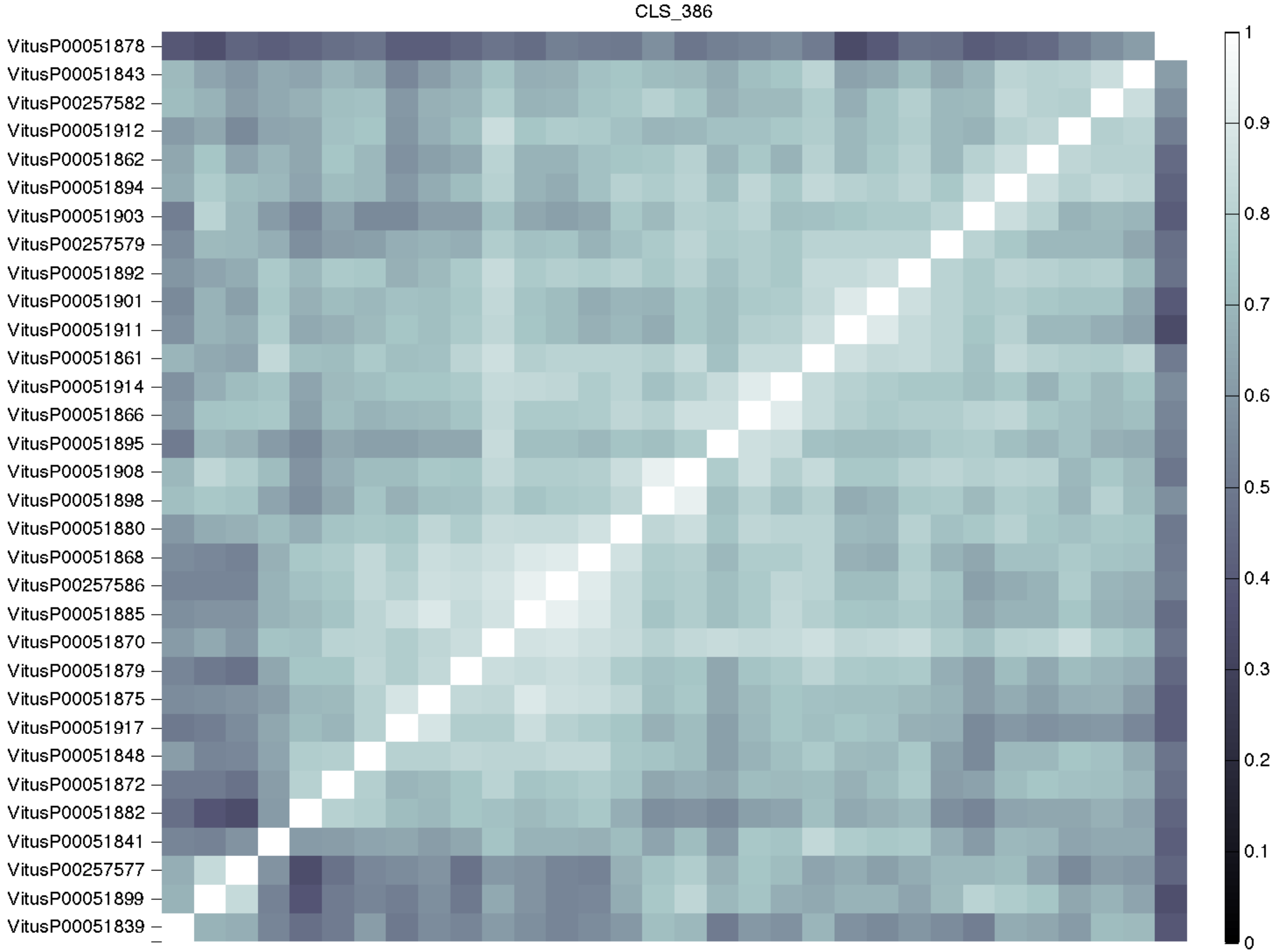
CLS_386
VitusP00051878
VitusP00051843
VitusP00257582
VitusP00051912
VitusP00051862
VitusP00051894
VitusP00051903
VitusP00257579
VitusP00051892
VitusP00051901
VitusP00051911
VitusP00051861
VitusP00051914
VitusP00051866
VitusP00051895
VitusP00051908
VitusP00051898
VitusP00051880
VitusP00051868
VitusP00257586
VitusP00051885
VitusP00051870
VitusP00051879
VitusP00051875
VitusP00051917
VitusP00051848
VitusP00051872
VitusP00051882
VitusP00051841
VitusP00257577
VitusP00051899
VitusP00051839
1
0.9
0.8
0.7
0.6
0.5
0.4
0.3
0.2
0.1
0

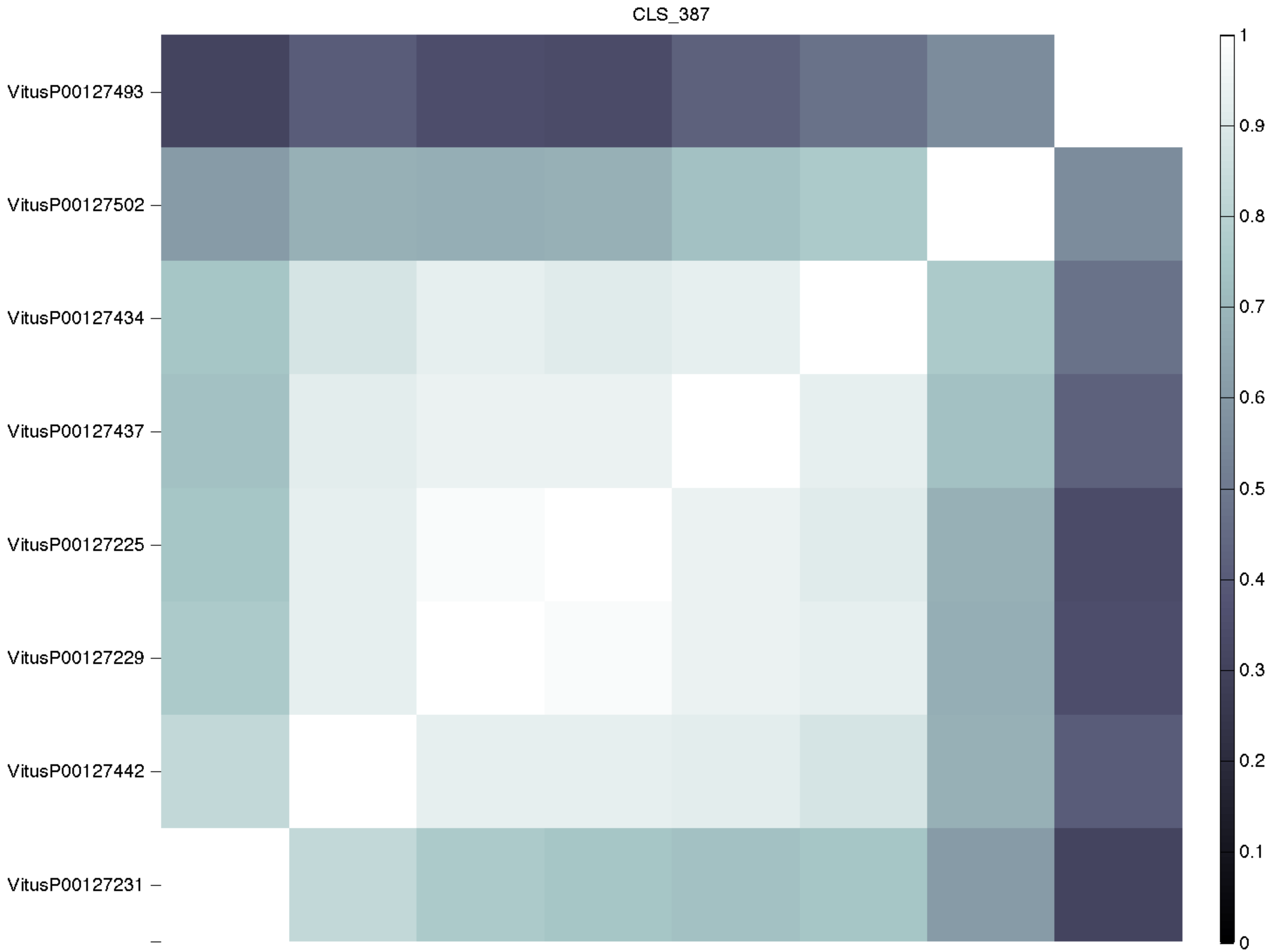

CLS_387
VitusP00127493
VitusP00127502
VitusP00127434
VitusP00127437
VitusP00127225
VitusP00127229
VitusP00127442
VitusP00127231
1
0.9
0.8
0.7
0.6
0.5
0.4
0.3
0.2
0.1
0

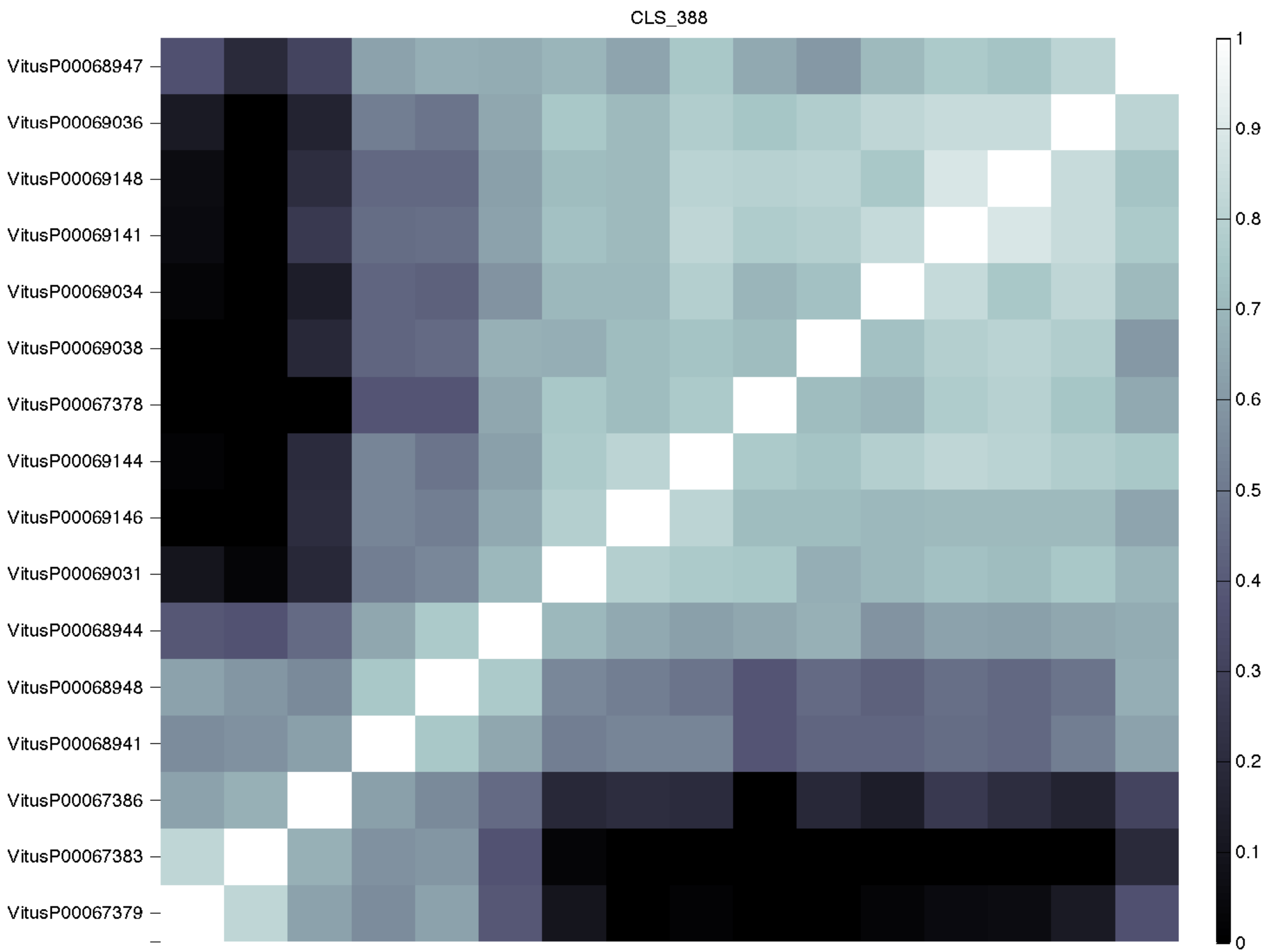
CLS_388
VitusP00068947
VitusP00069036
VitusP00069148
VitusP00069141
VitusP00069034
VitusP00069038
VitusP00067378
VitusP00069144
VitusP00069146
VitusP00069031
VitusP00068944
VitusP00068948
VitusP00068941
VitusP00067386
VitusP00067383
VitusP00067379
1
0.9
0.8
0.7
0.6
0.5
0.4
0.3
0.2
0.1
0

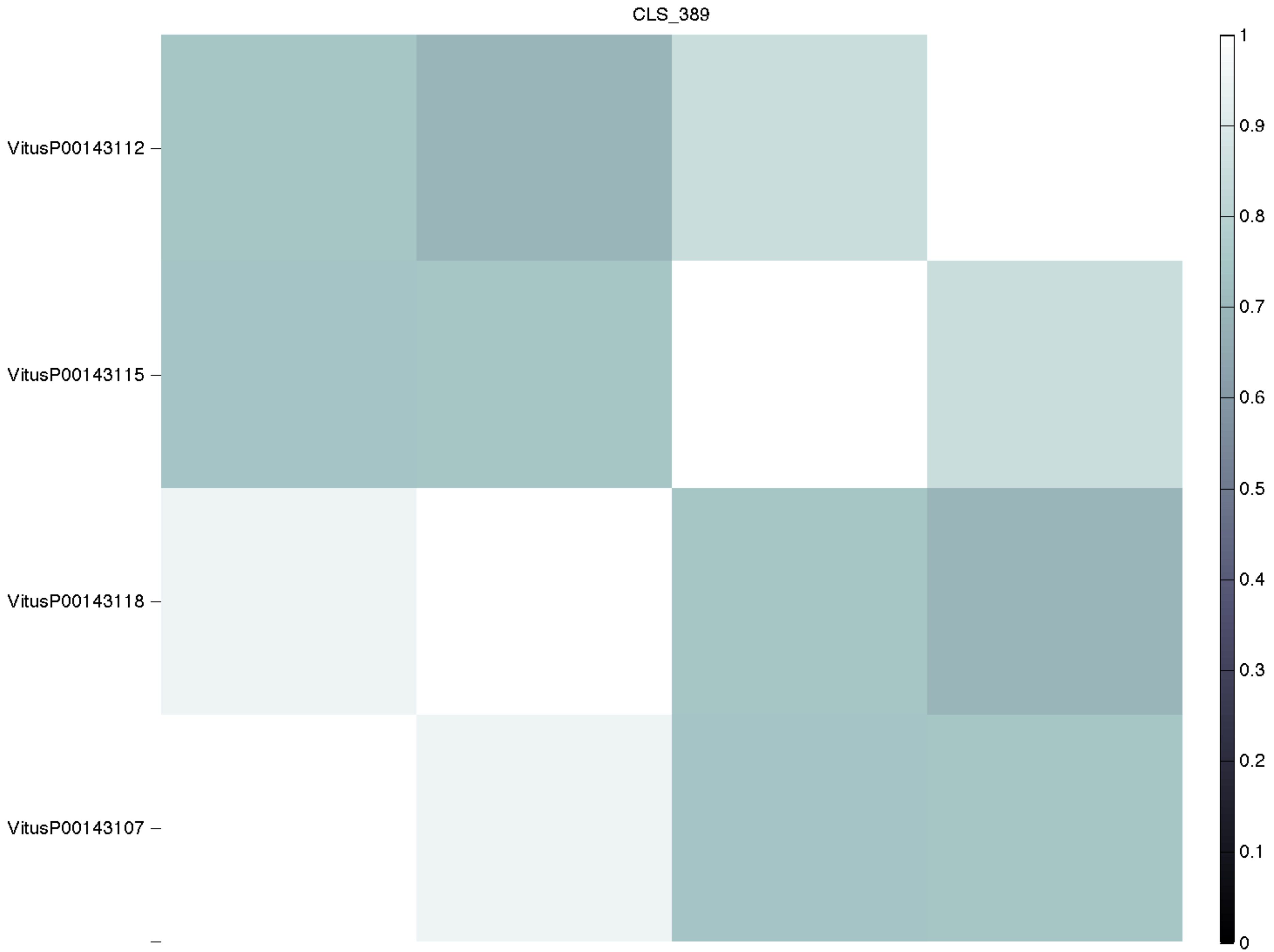
CLS_389
VitusP00143112
VitusP00143115
VitusP00143118
VitusP00143107
1
0.9
0.8
0.7
0.6
0.5
0.4
0.3
0.2
0.1
0

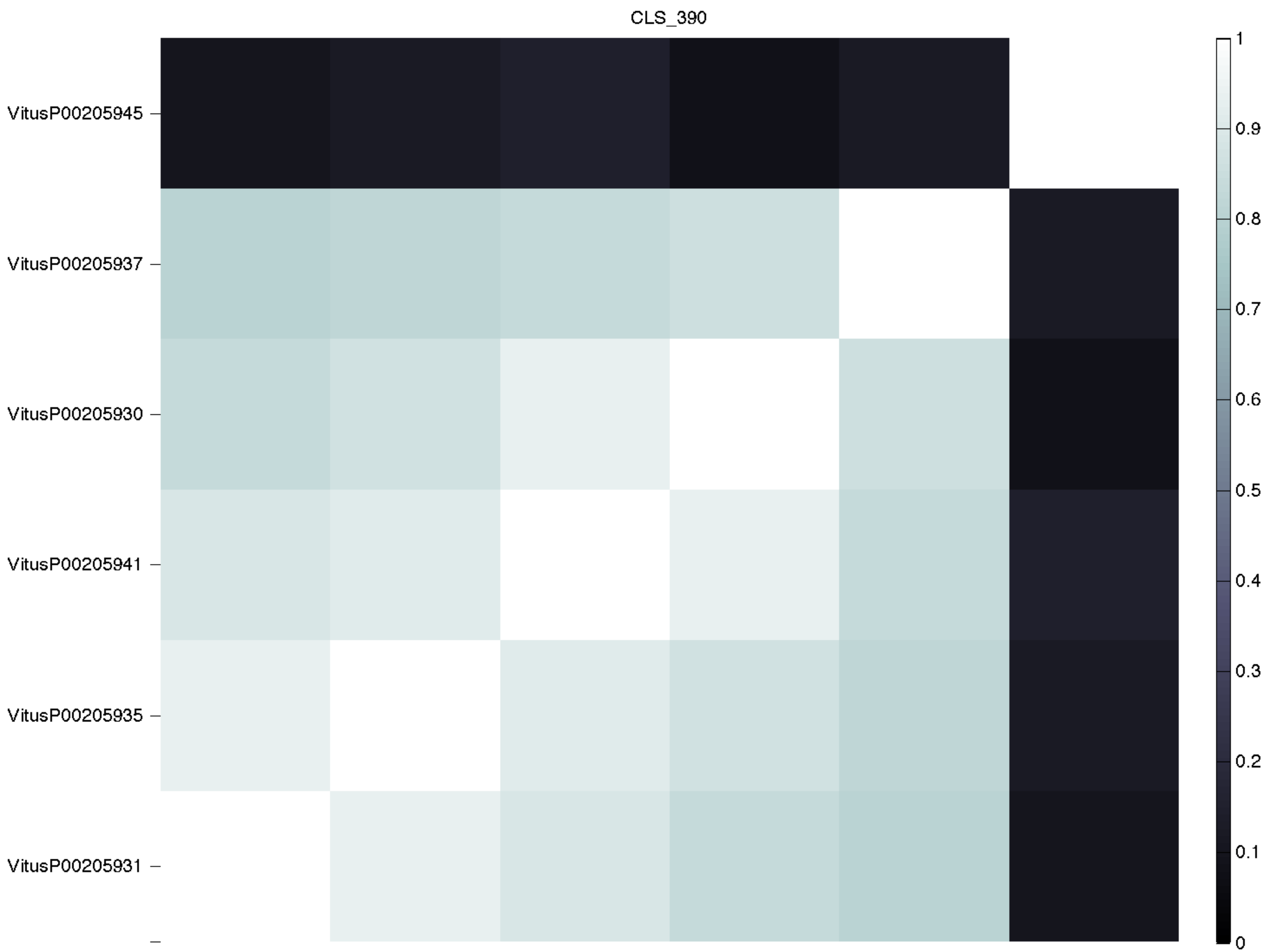
CLS_390
VitusP00205945
VitusP00205937
VitusP00205930
VitusP00205941
VitusP00205935
VitusP00205931
1
0.9
0.8
0.7
0.6
0.5
0.4
0.3
0.2
0.1
0

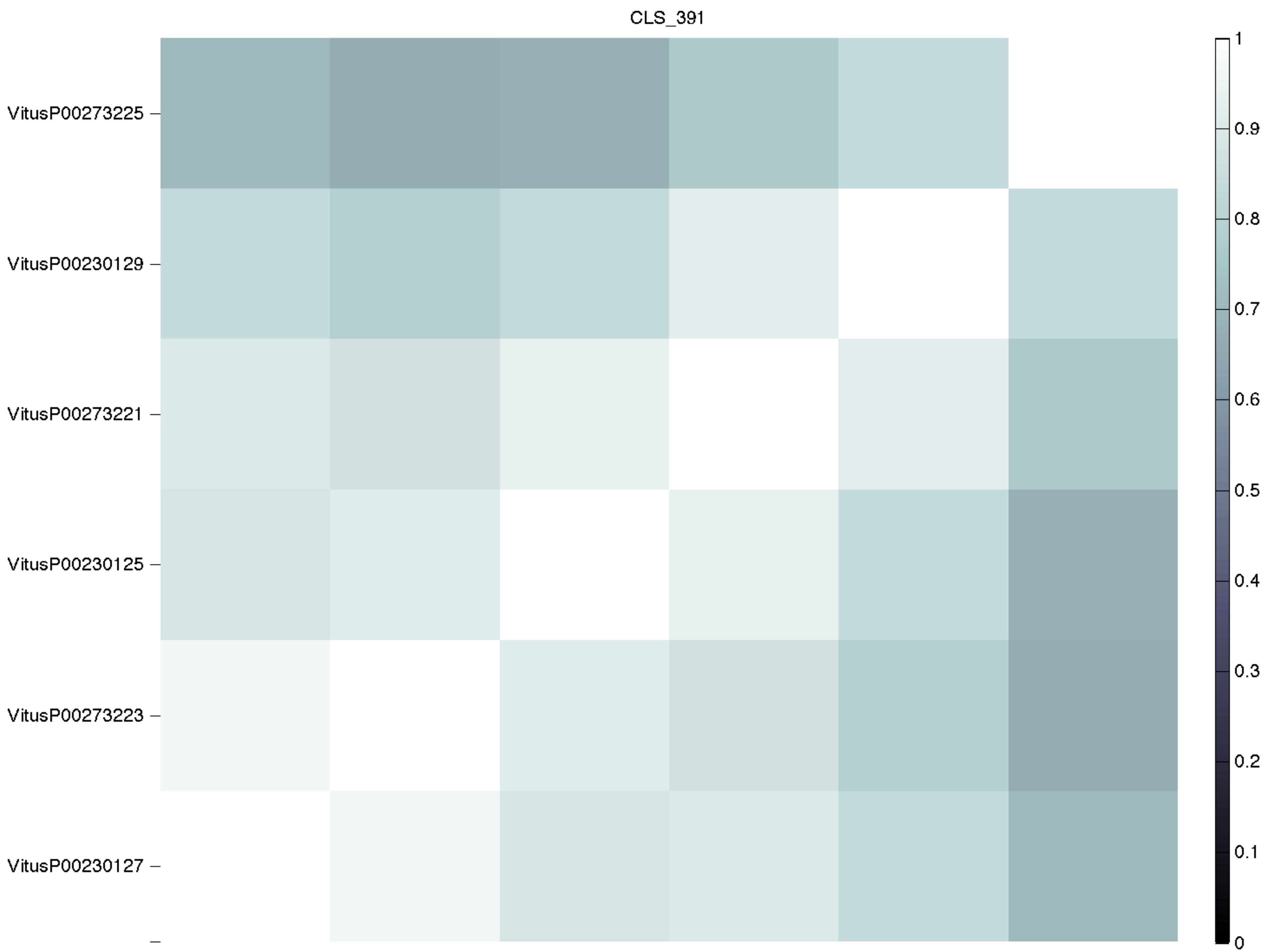

CLS_391
VitusP00273225
VitusP00230129
VitusP00273221
VitusP00230125
VitusP00273223
VitusP00230127
1
0.9
0.8
0.7
0.6
0.5
0.4
0.3
0.2
0.1
0

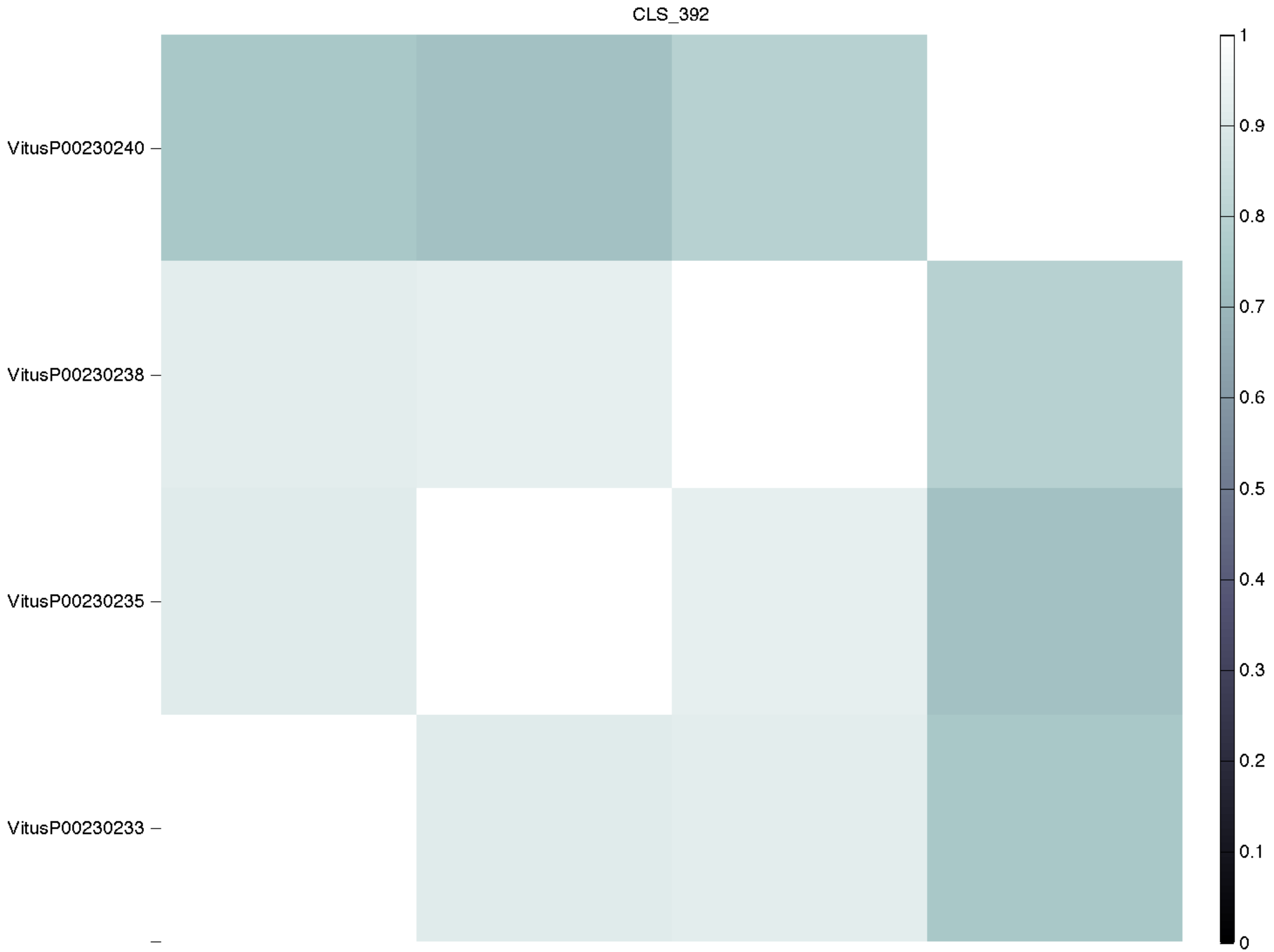
CLS_392
VitusP00230240
VitusP00230238
VitusP00230235
VitusP00230233
1
0.9
0.8
0.7
0.6
0.5
0.4
0.3
0.2
0.1
0

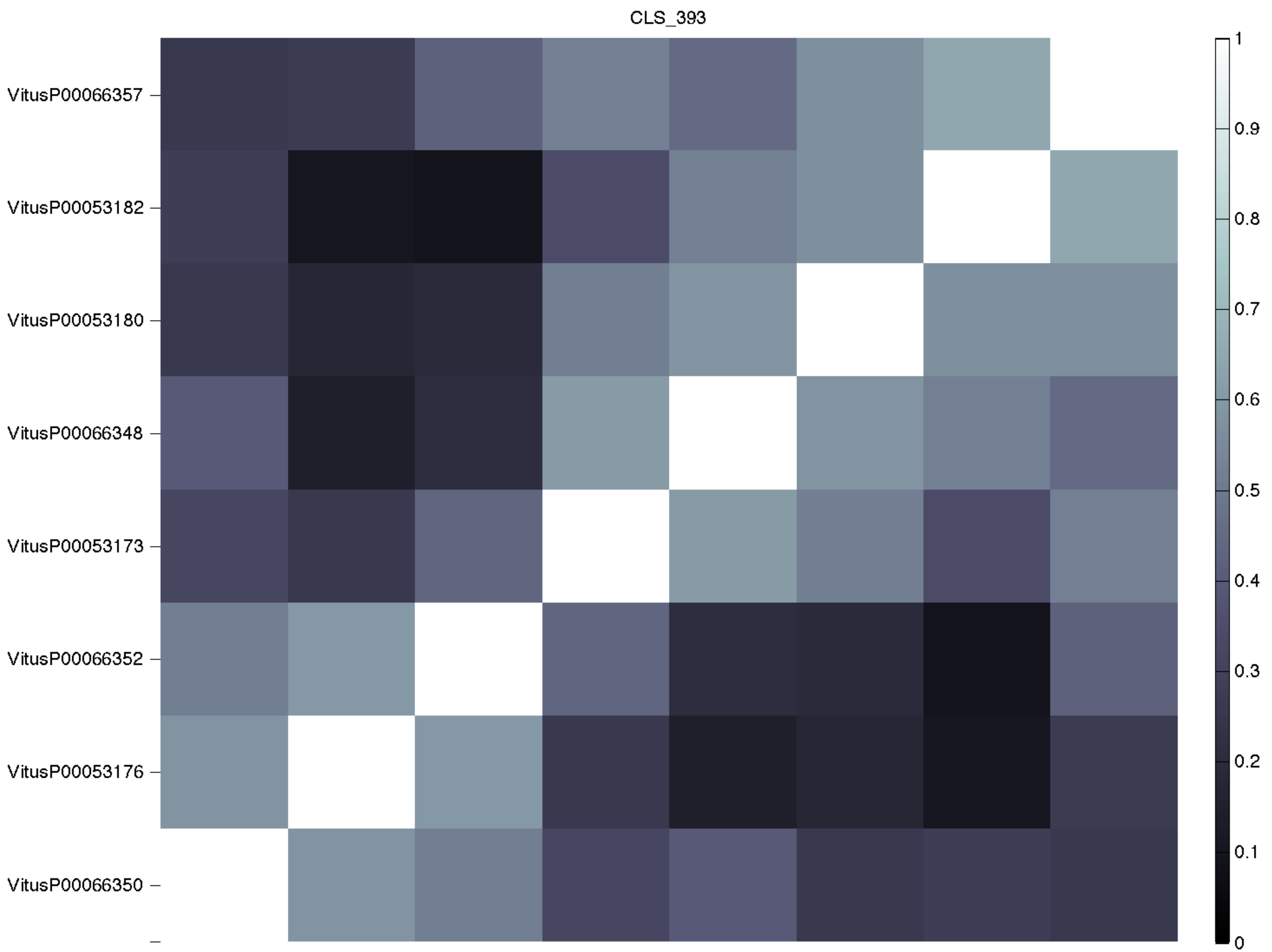
CLS_393
VitusP00066357
VitusP00053182
VitusP00053180
VitusP00066348
VitusP00053173
VitusP00066352
VitusP00053176
VitusP00066350
1
0.9
0.8
0.7
0.6
0.5
0.4
0.3
0.2
0.1
0

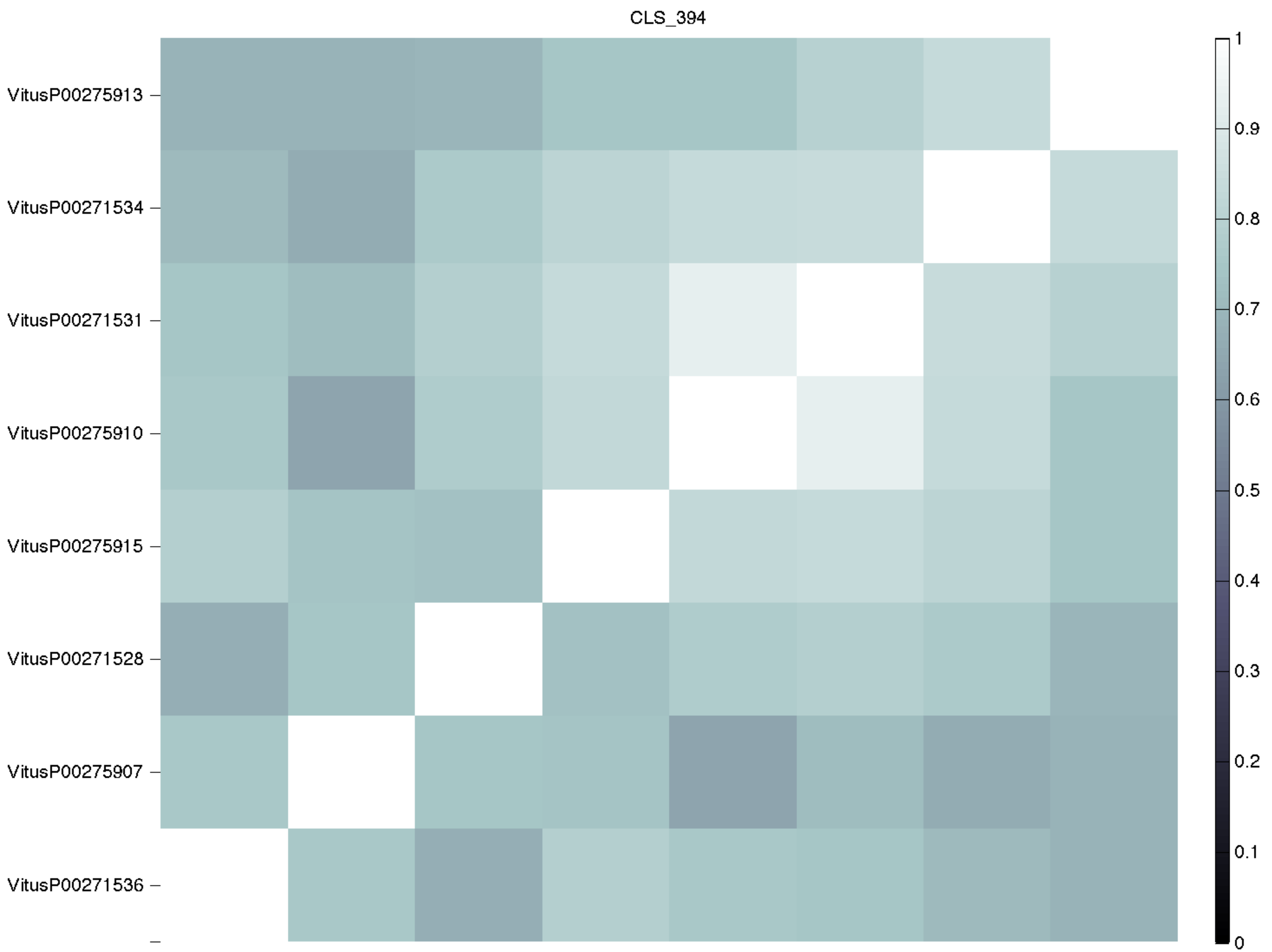
CLS_394
VitusP00275913
VitusP00271534
VitusP00271531
VitusP00275910
VitusP00275915
VitusP00271528
VitusP00275907
VitusP00271536
1
0.9
0.8
0.7
0.6
0.5
0.4
0.3
0.2
0.1
0

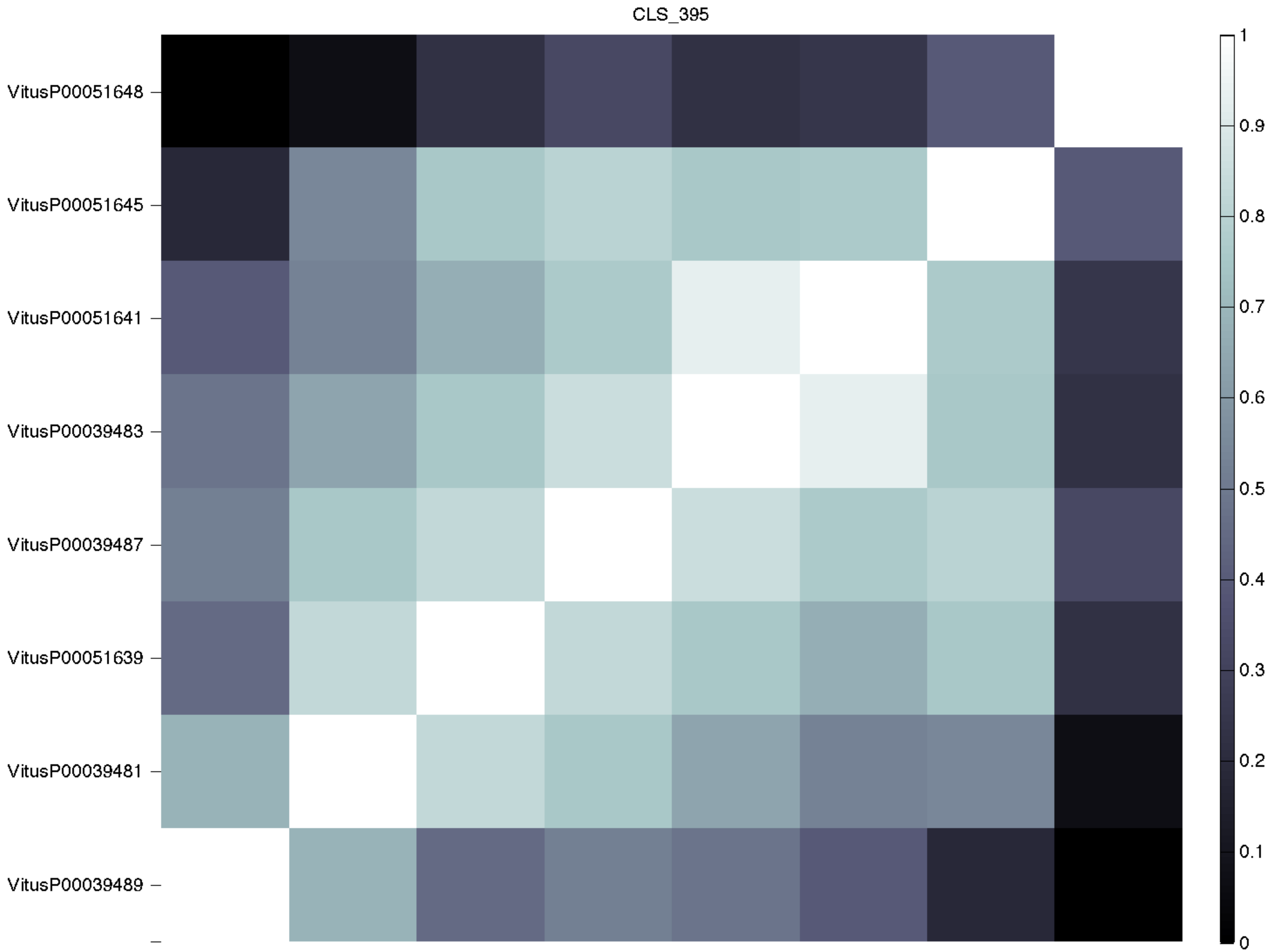

CLS_395
VitusP00051648
VitusP00051645
VitusP00051641
VitusP00039483
VitusP00039487
VitusP00051639
VitusP00039481
VitusP00039489
1
0.9
0.8
0.7
0.6
0.5
0.4
0.3
0.2
0.1
0

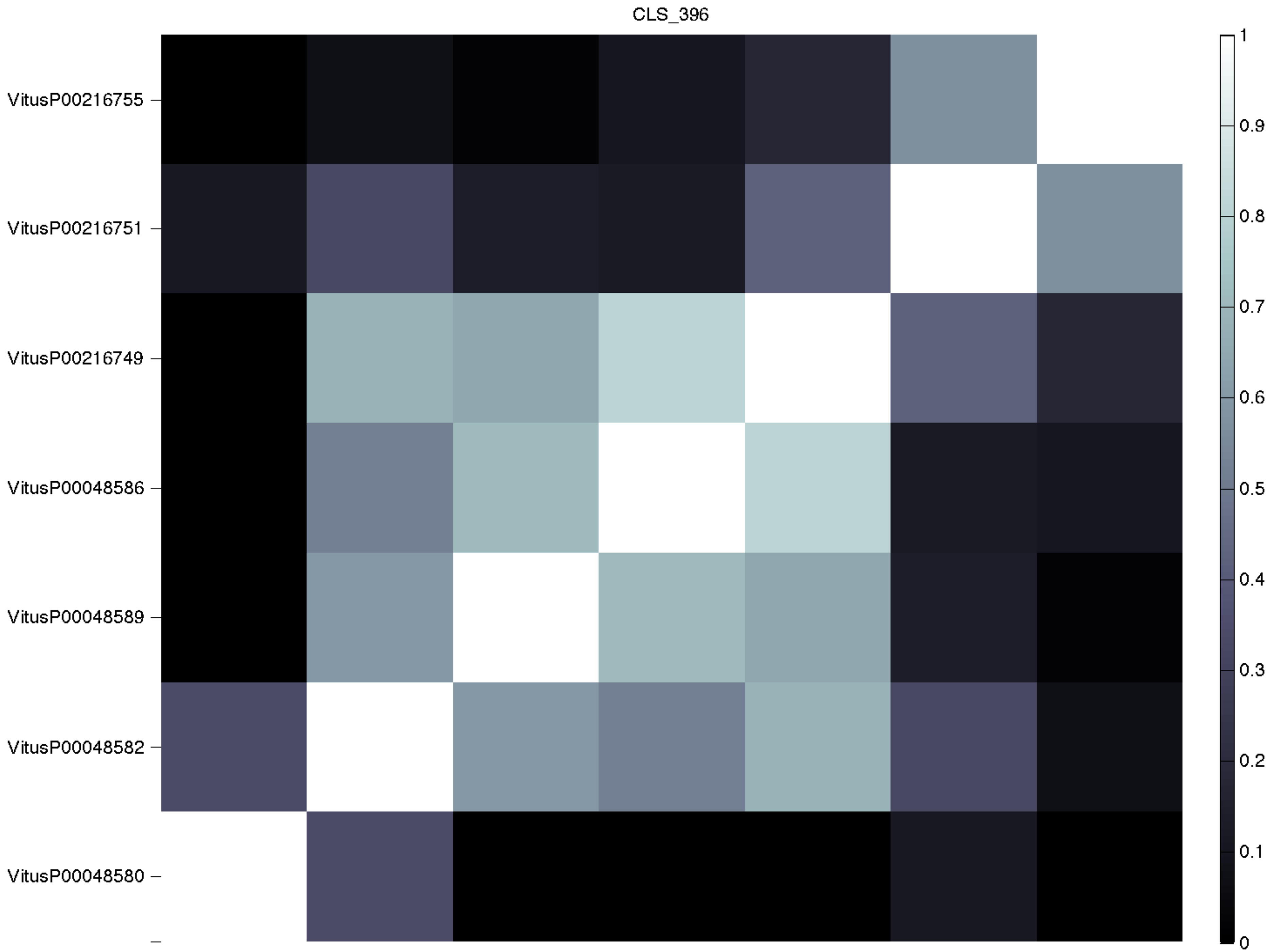
CLS_396
VitusP00216755
VitusP00216751
VitusP00216749
VitusP00048586
VitusP00048589
VitusP00048582
VitusP00048580
1
0.9
0.8
0.7
0.6
0.5
0.4
0.3
0.2
0.1
0

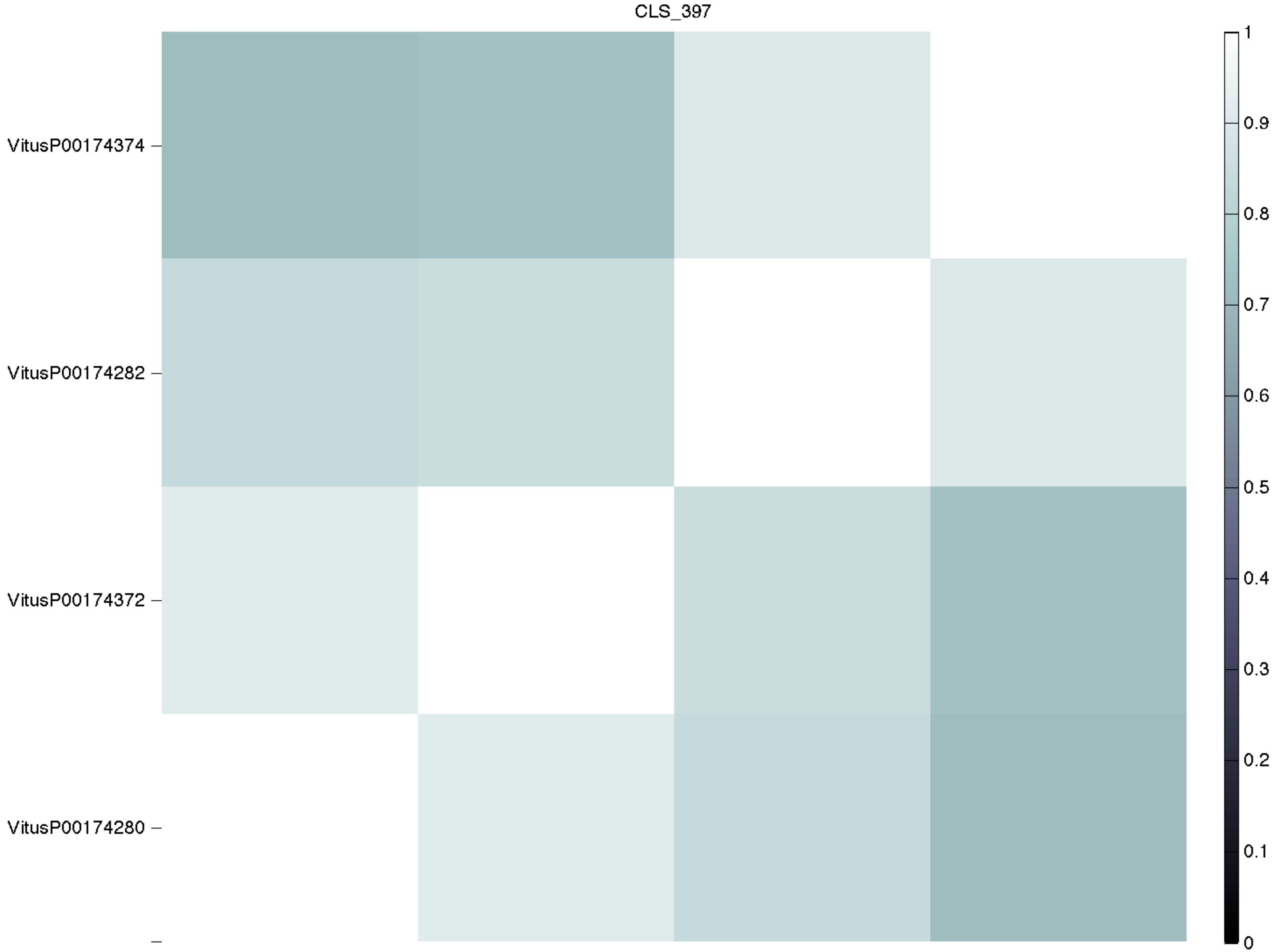
CLS_397
VitusP00174374
VitusP00174282
VitusP00174372
VitusP00174280
1
0.9
0.8
0.7
0.6
0.5
0.4
0.3
0.2
0.1
0

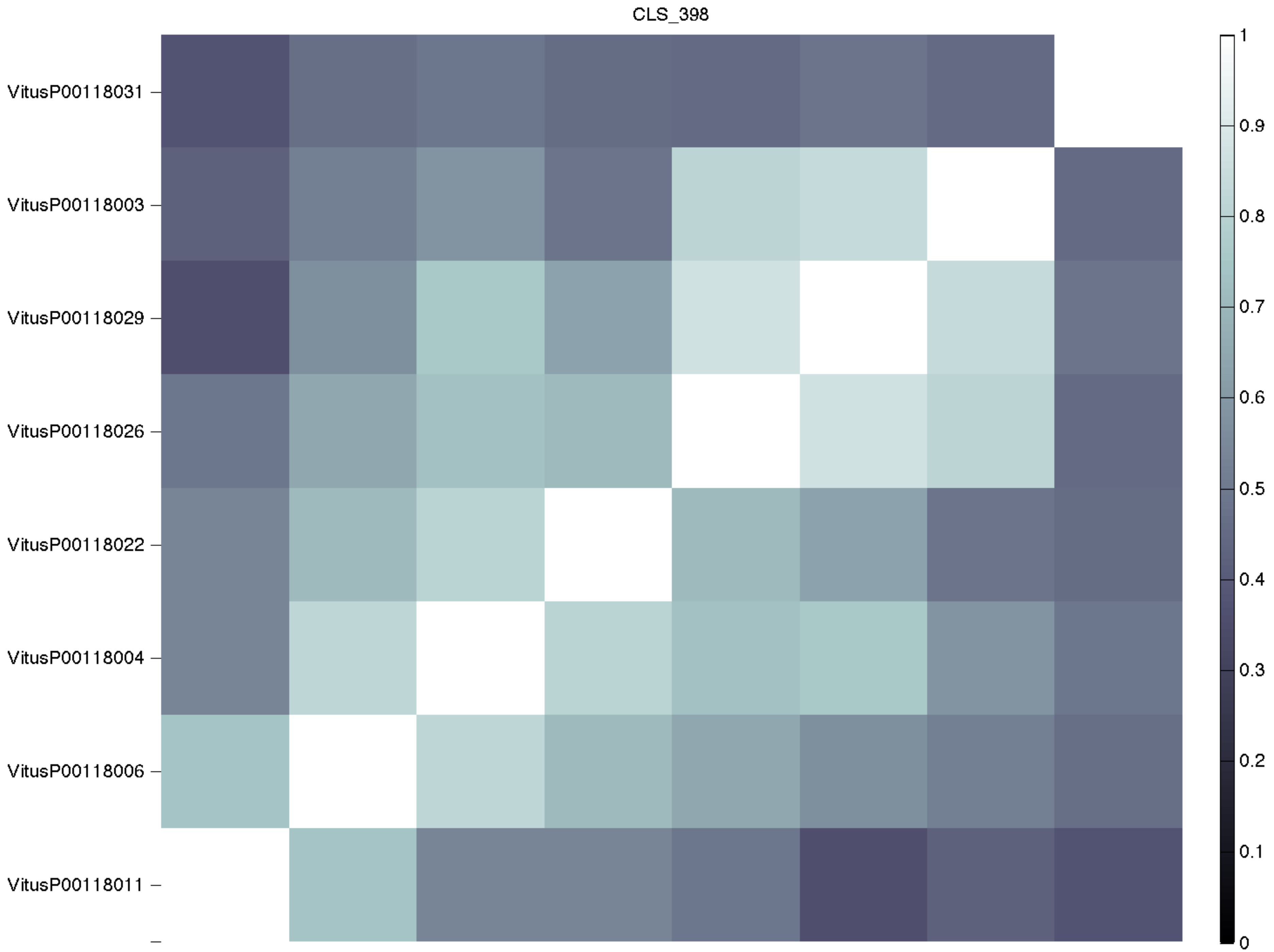
CLS_398
VitusP00118031
VitusP00118003
VitusP00118029
VitusP00118026
VitusP00118022
VitusP00118004
VitusP00118006
VitusP00118011
1
0.9
0.8
0.7
0.6
0.5
0.4
0.3
0.2
0.1
0

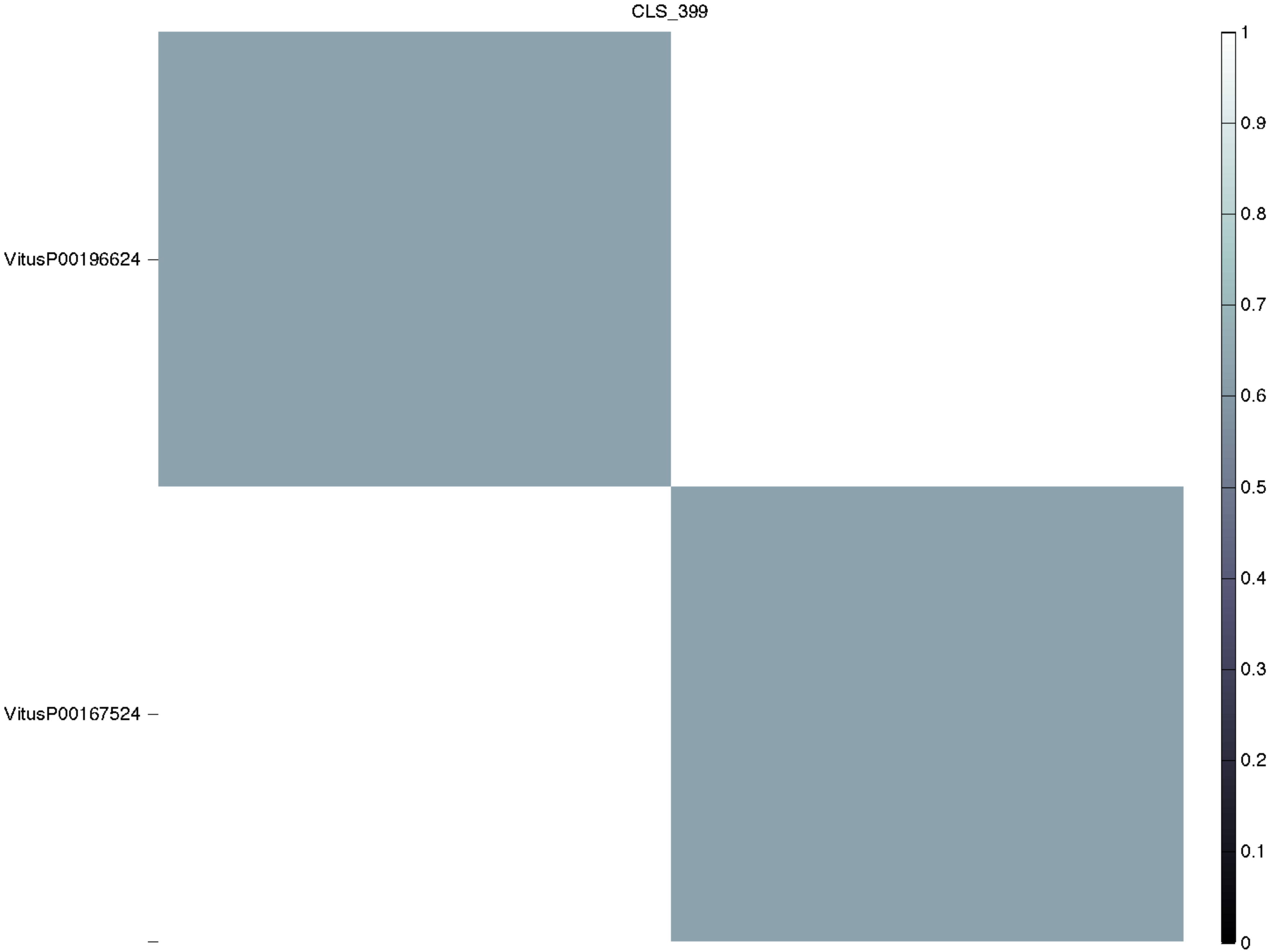
CLS_399
VitusP00196624
VitusP00167524
1
0.9
0.8
0.7
0.6
0.5
0.4
0.3
0.2
0.1
0

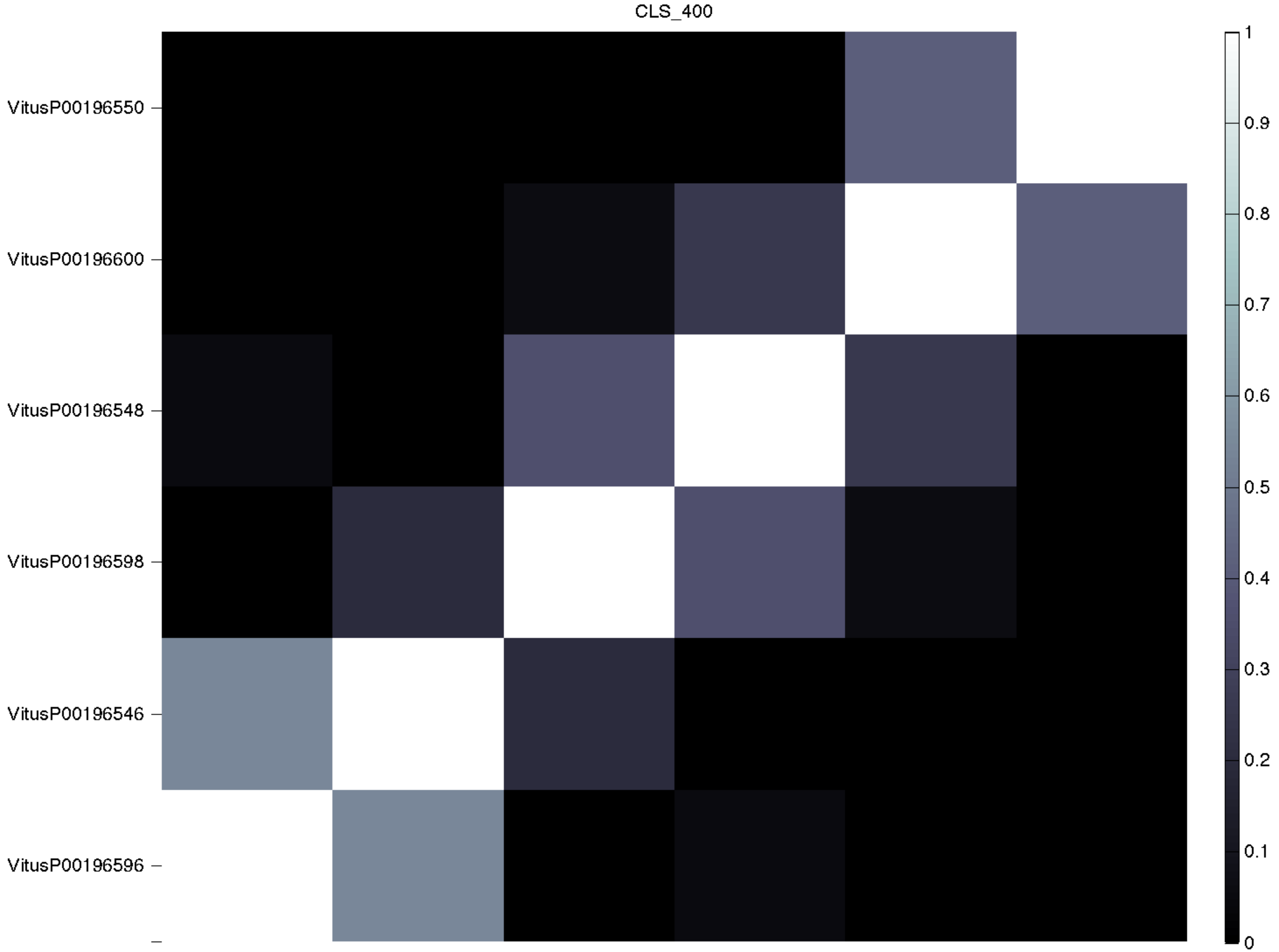

CLS_400
VitusP00196550
VitusP00196600
VitusP00196548
VitusP00196598
VitusP00196546
VitusP00196596
1
0.9
0.8
0.7
0.6
0.5
0.4
0.3
0.2
0.1
0

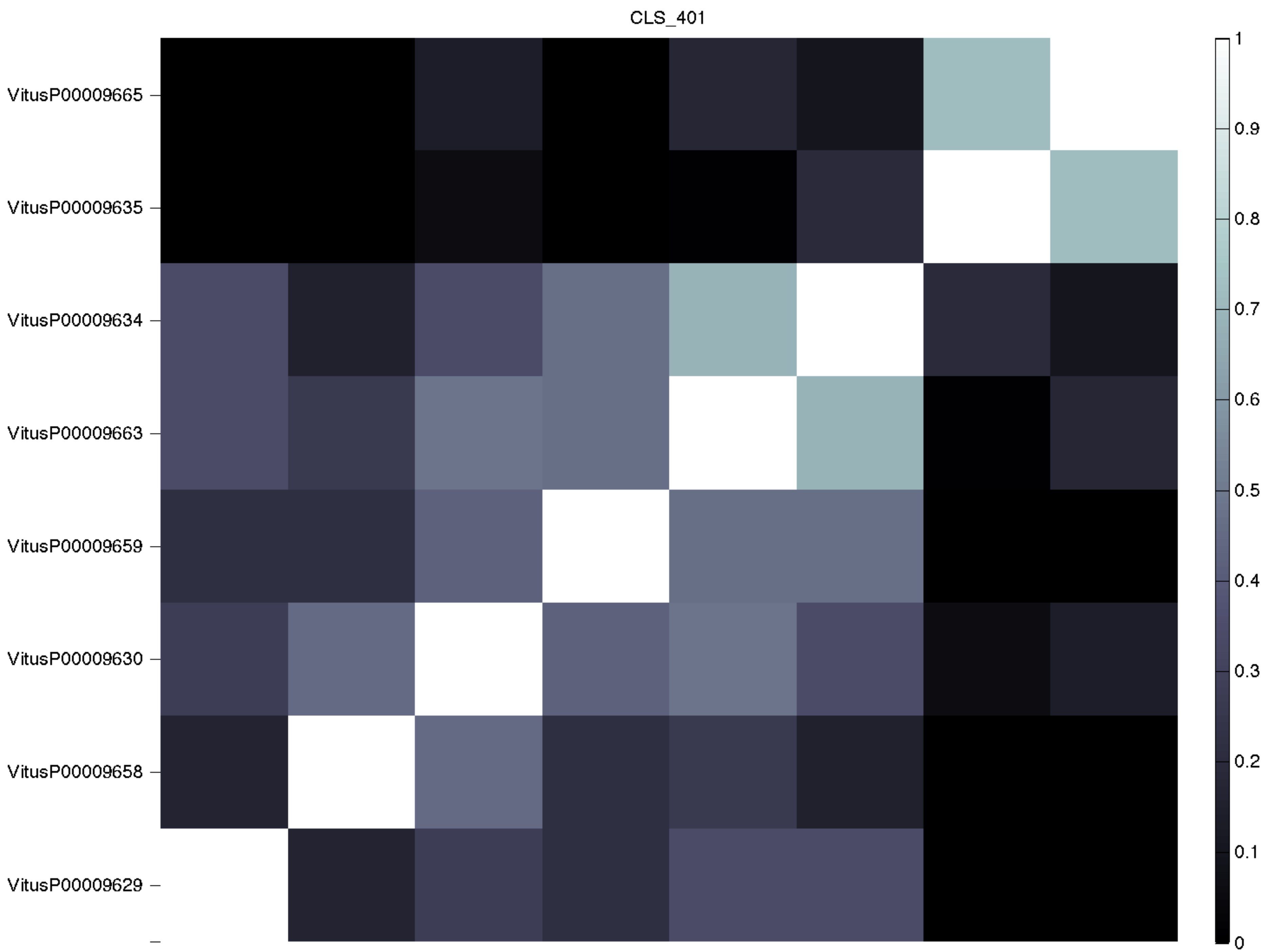
CLS_401
VitusP00009665
VitusP00009635
VitusP00009634
VitusP00009663
VitusP00009659
VitusP00009630
VitusP00009658
VitusP00009629
1
0.9
0.8
0.7
0.6
0.5
0.4
0.3
0.2
0.1
0

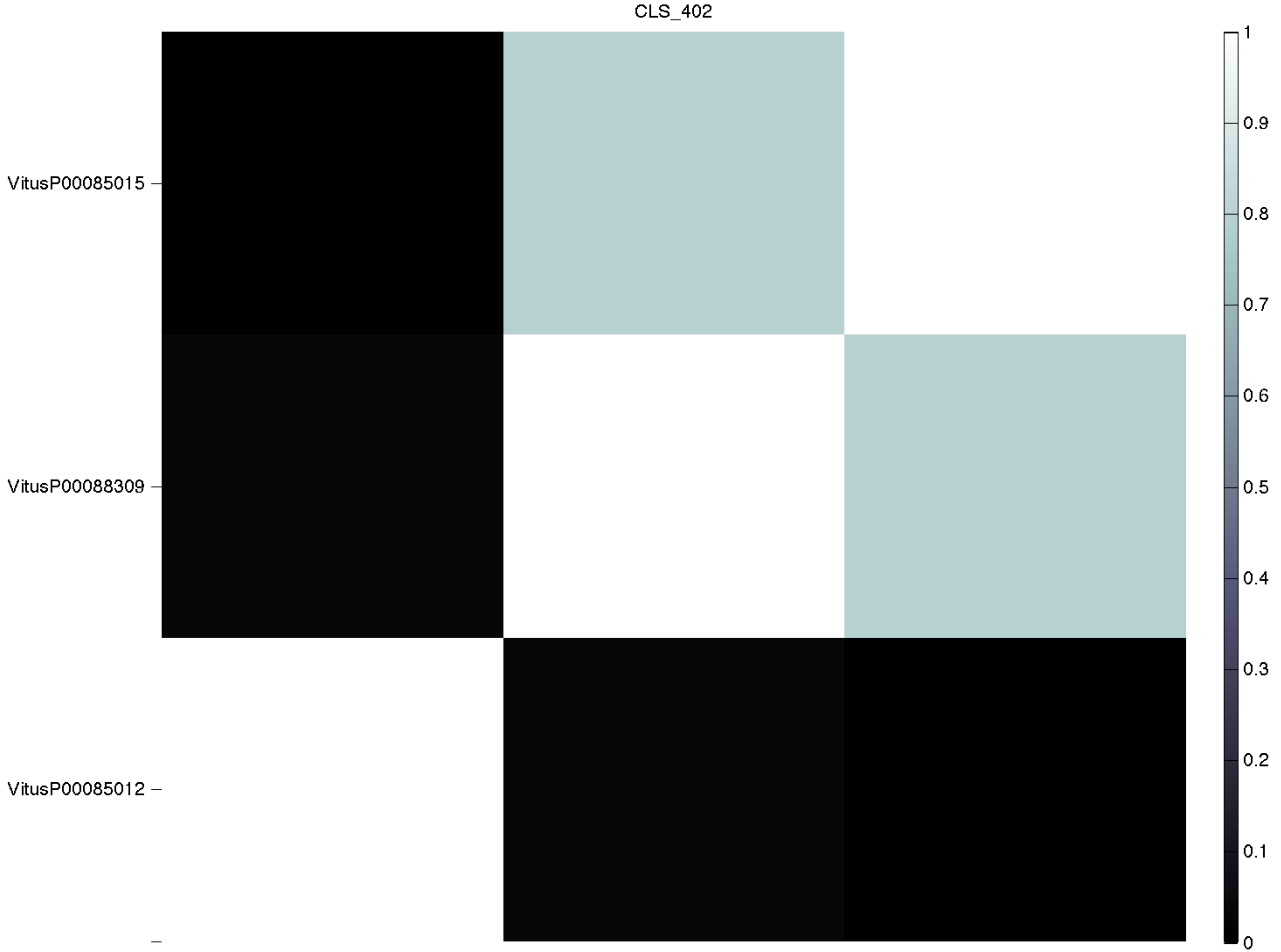
CLS_402
VitusP00085015
VitusP00088309
VitusP00085012
1
0.9
0.8
0.7
0.6
0.5
0.4
0.3
0.2
0.1
0

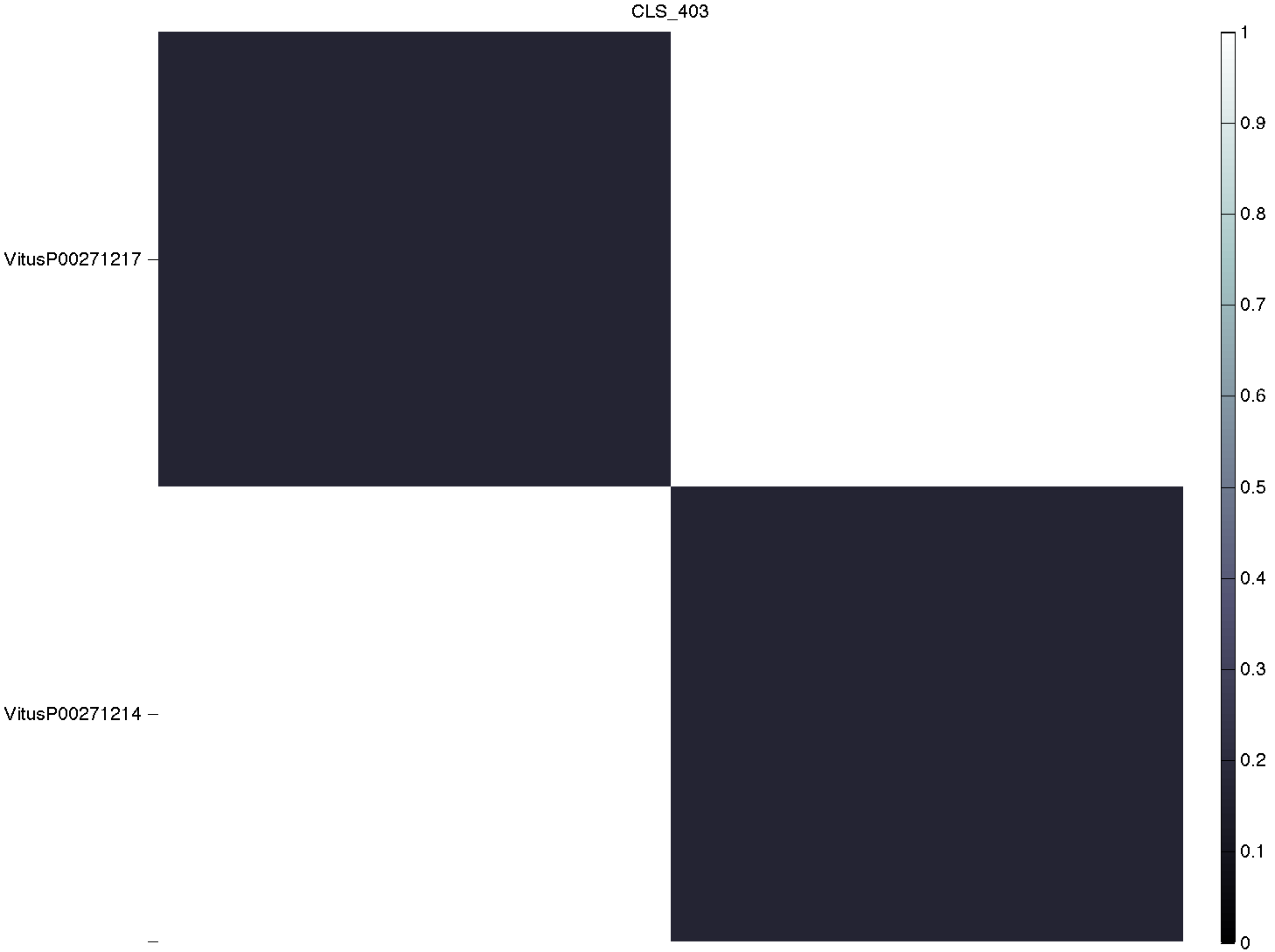
CLS_403
VitusP00271217
VitusP00271214
1
0.9
0.8
0.7
0.6
0.5
0.4
0.3
0.2
0.1
0

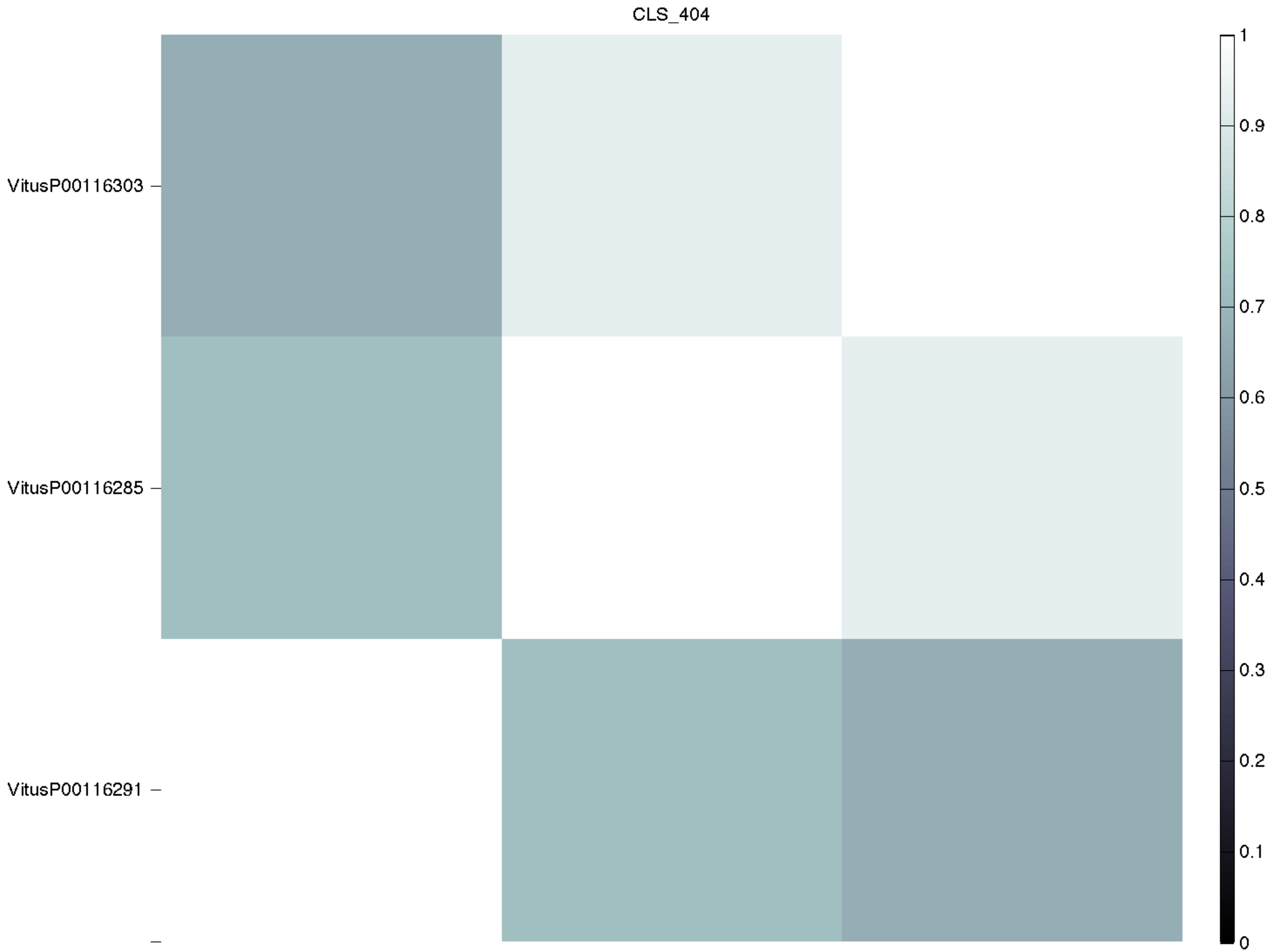
CLS_404
VitusP00116303
VitusP00116285
VitusP00116291
1
0.9
0.8
0.7
0.6
0.5
0.4
0.3
0.2
0.1
0

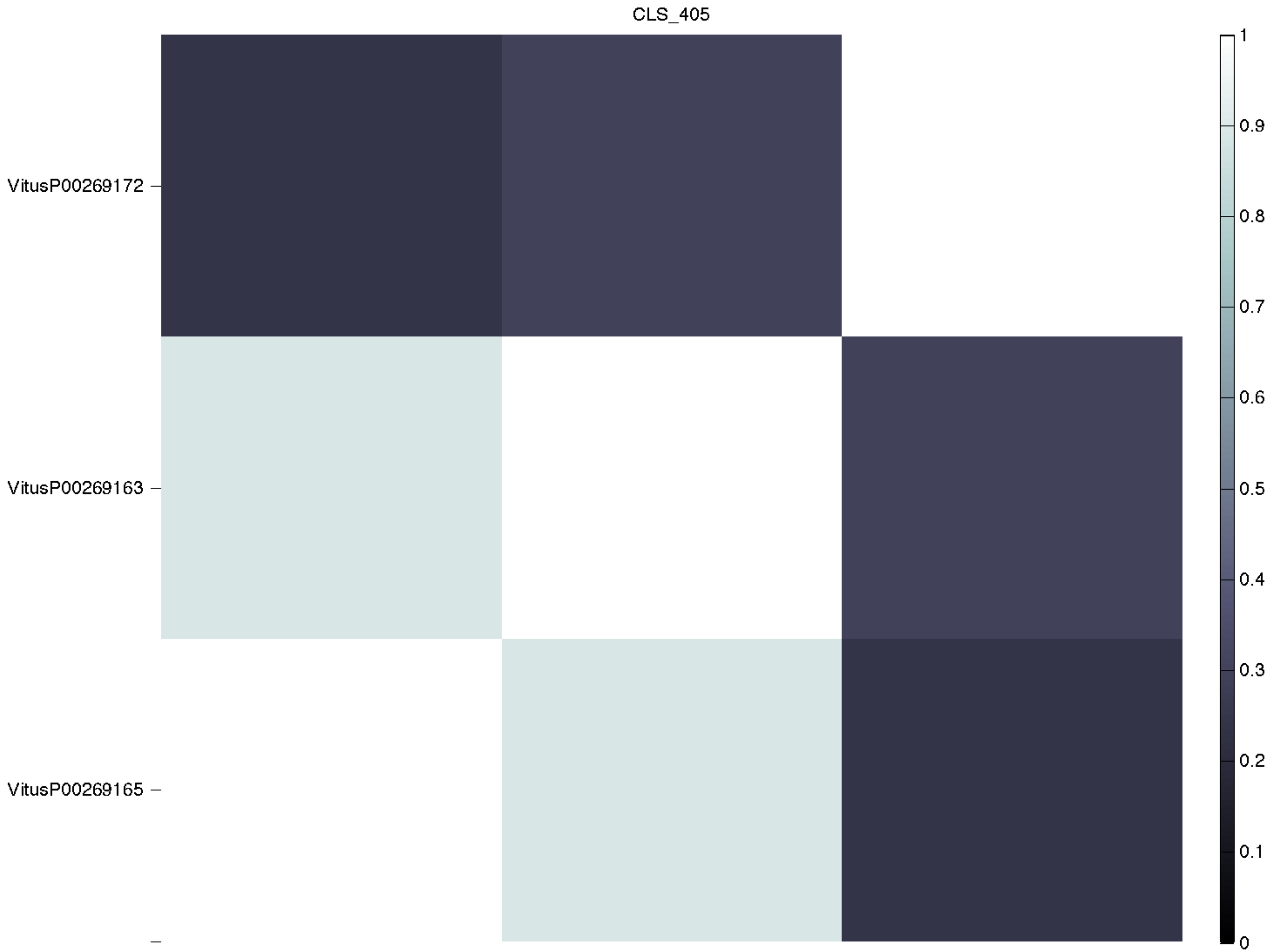
CLS_405
VitusP00269172
VitusP00269163
VitusP00269165
1
0.9
0.8
0.7
0.6
0.5
0.4
0.3
0.2
0.1
0

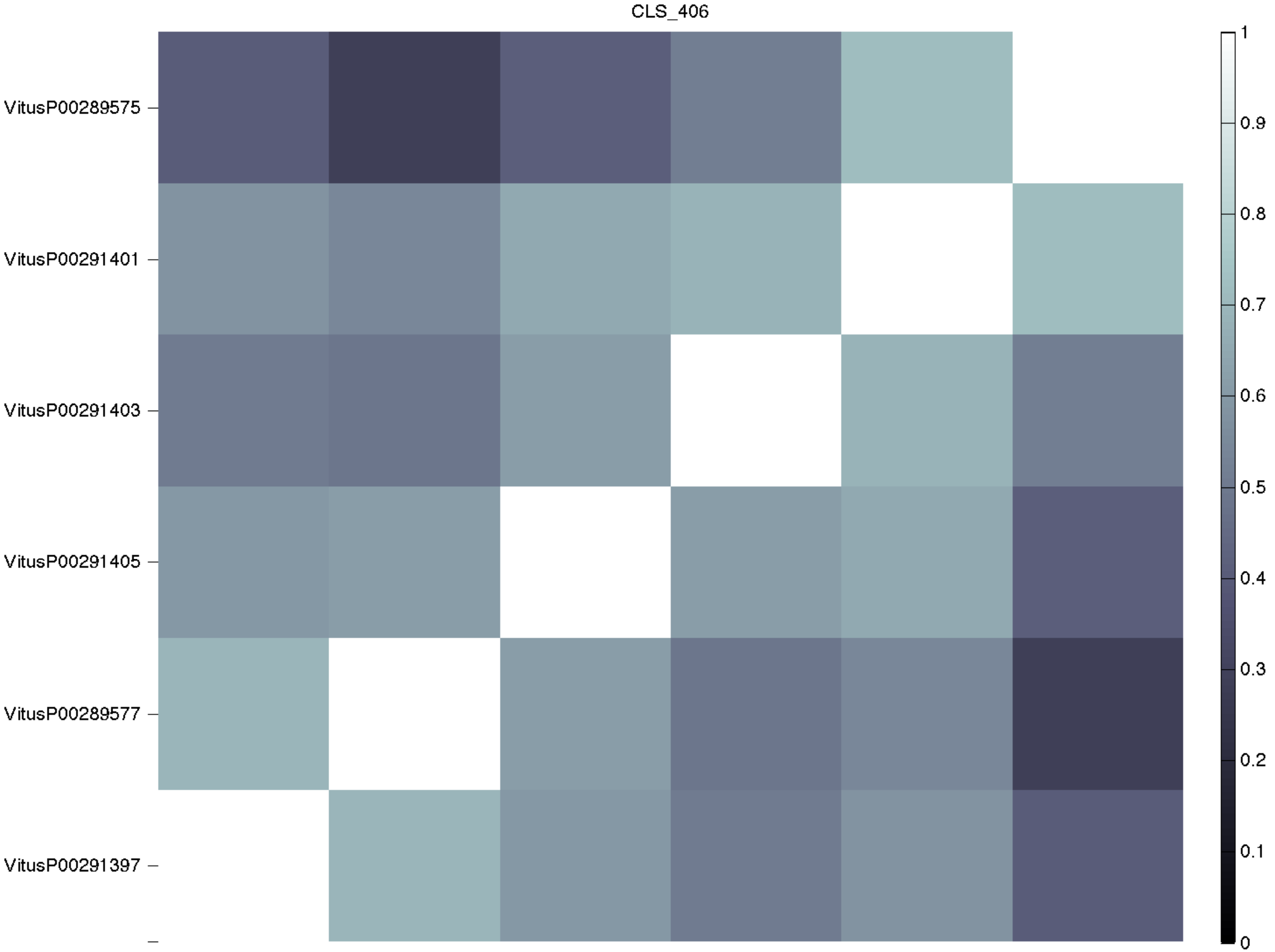

CLS_406
VitusP00289575
VitusP00291401
VitusP00291403
VitusP00291405
VitusP00289577
VitusP00291397
1
0.9
0.8
0.7
0.6
0.5
0.4
0.3
0.2
0.1
0

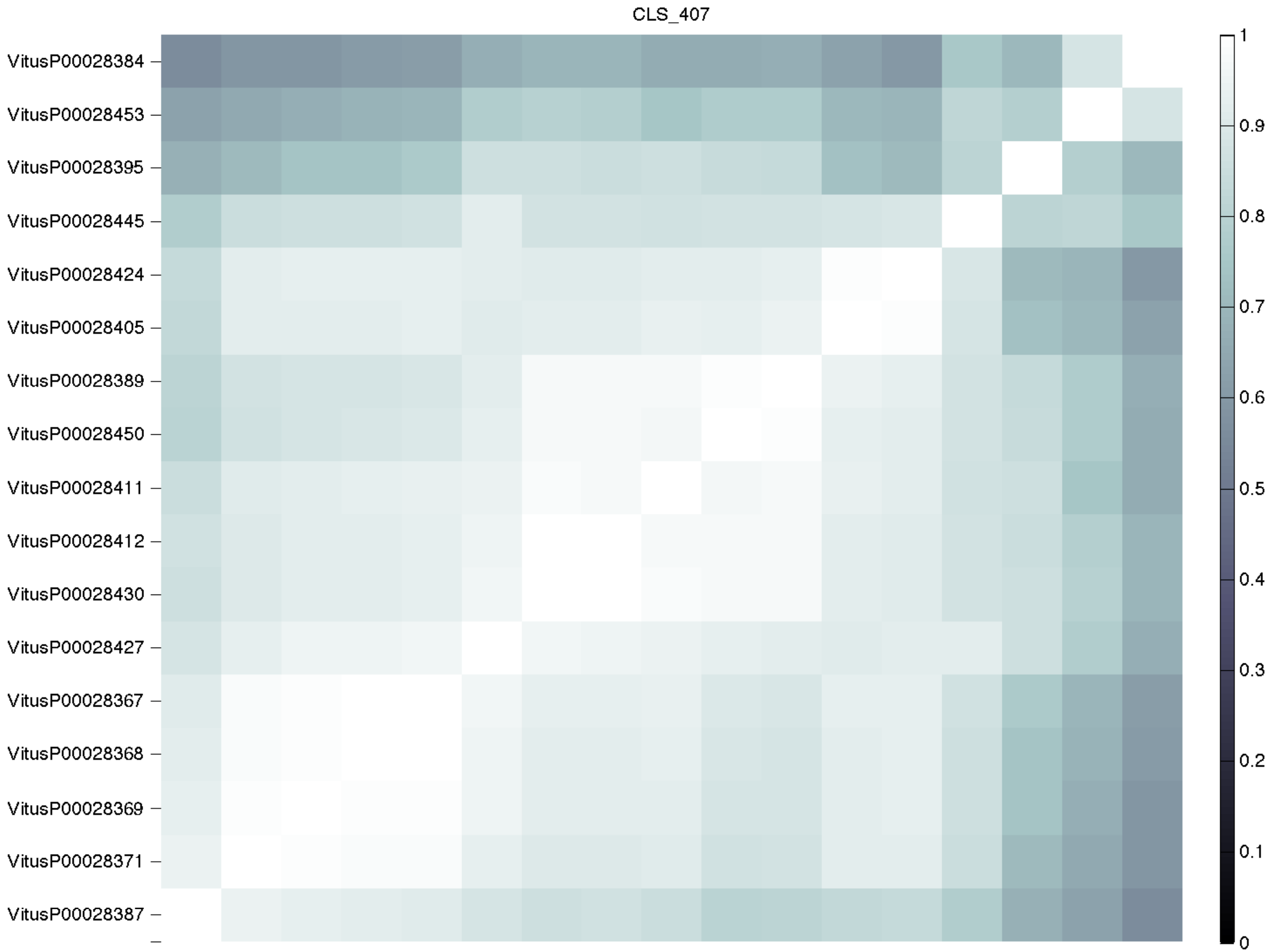
CLS_407
VitusP00028384
VitusP00028453
VitusP00028395
VitusP00028445
VitusP00028424
VitusP00028405
VitusP00028389
VitusP00028450
VitusP00028411
VitusP00028412
VitusP00028430
VitusP00028427
VitusP00028367
VitusP00028368
VitusP00028369
VitusP00028371
VitusP00028387
1
0.9
0.8
0.7
0.6
0.5
0.4
0.3
0.2
0.1
0

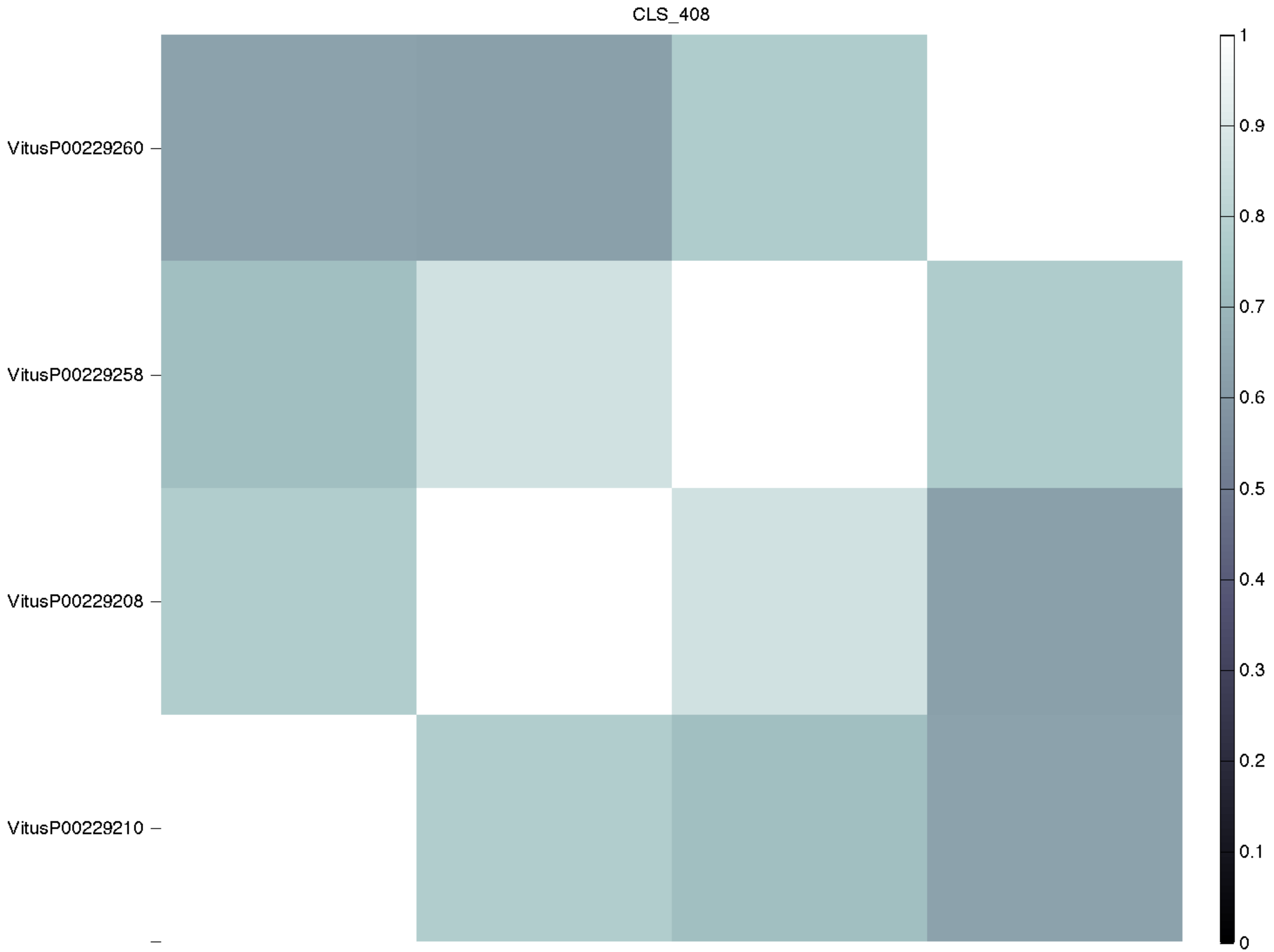

CLS_408
VitusP00229260
VitusP00229258
VitusP00229208
VitusP00229210
1
0.9
0.8
0.7
0.6
0.5
0.4
0.3
0.2
0.1
0

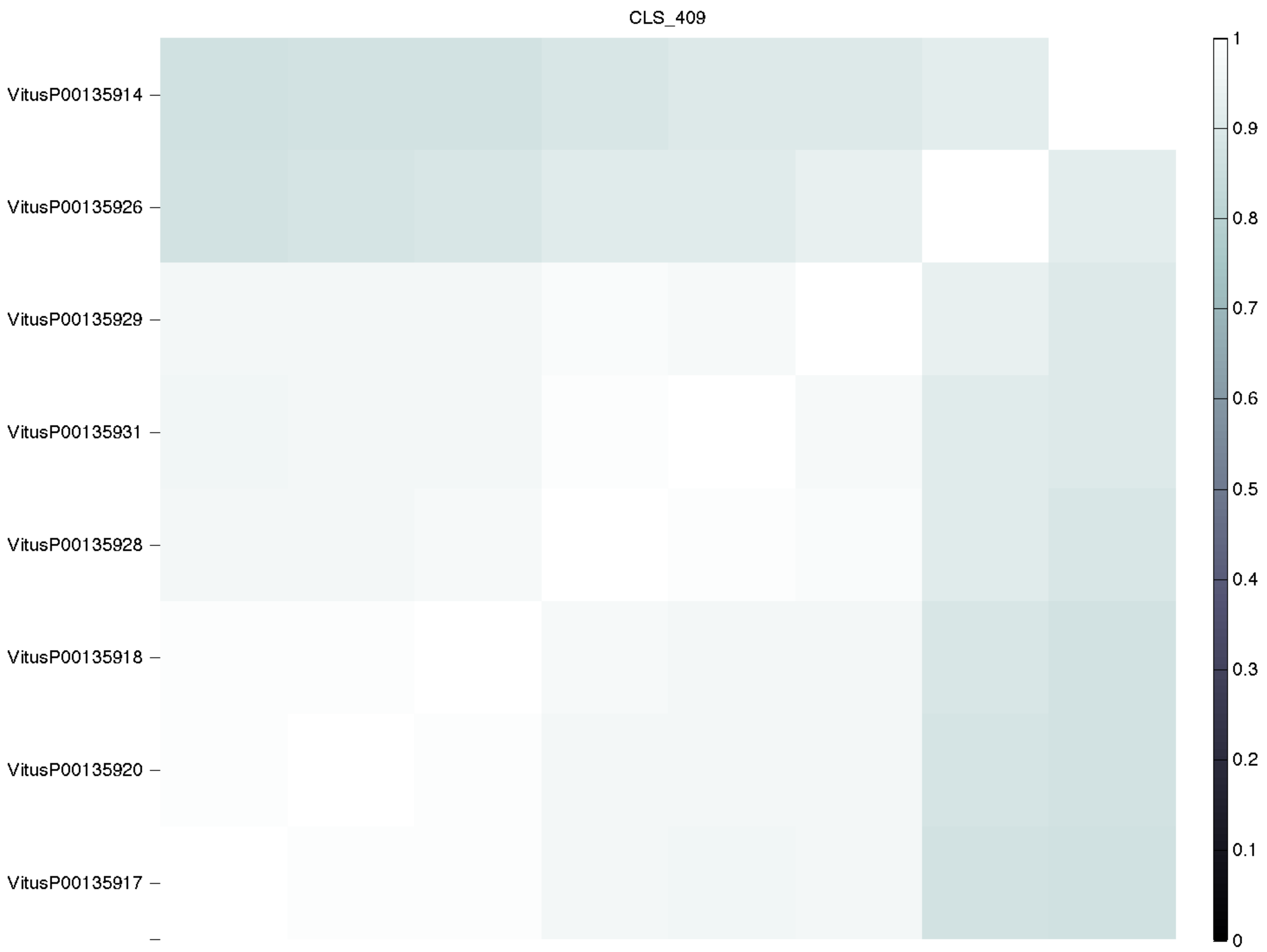
CLS_409
VitusP00135914
VitusP00135926
VitusP00135929
VitusP00135931
VitusP00135928
VitusP00135918
VitusP00135920
VitusP00135917
1
0.9
0.8
0.7
0.6
0.5
0.4
0.3
0.2
0.1
0

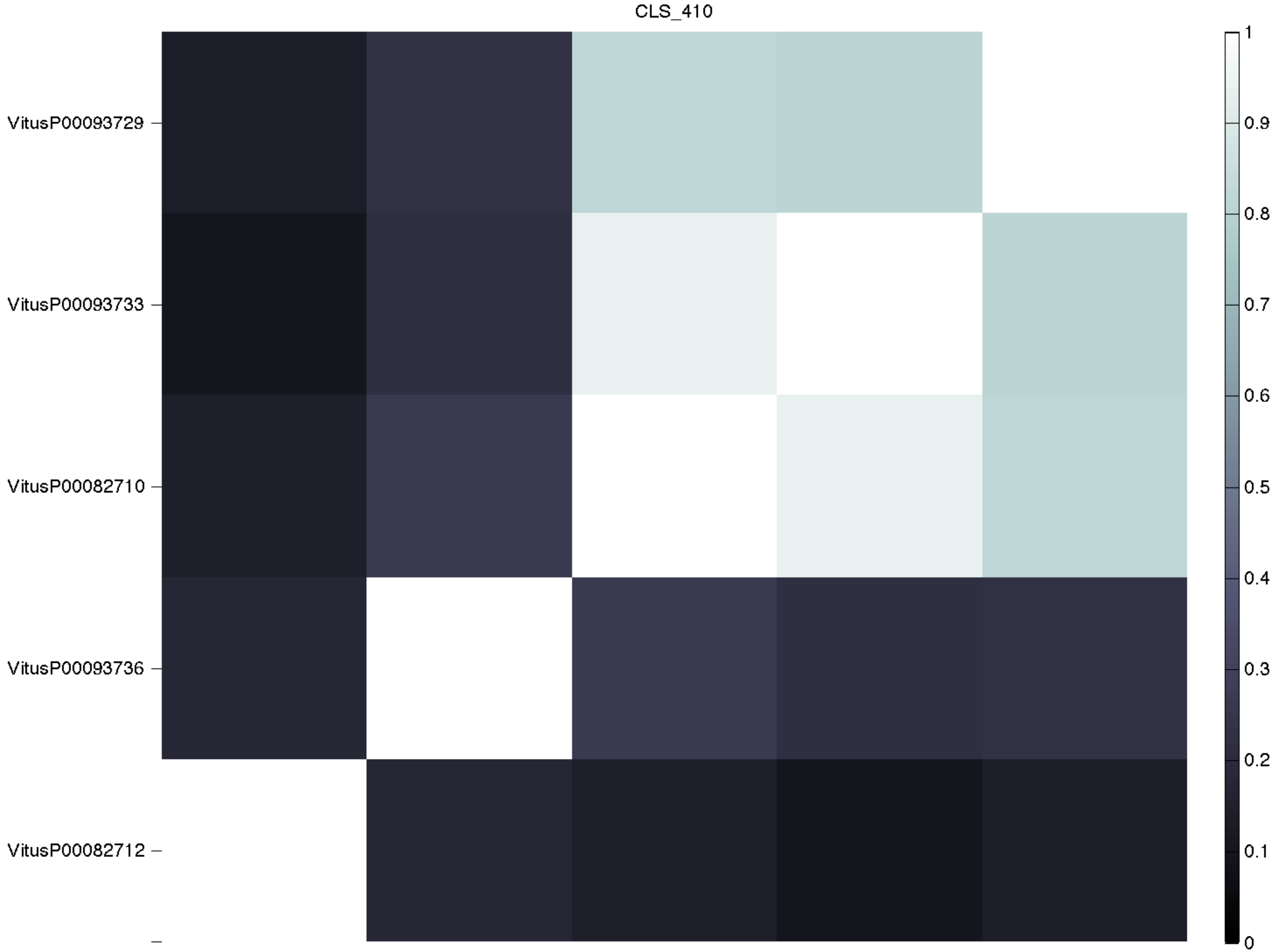
CLS_410
VitusP00093729
VitusP00093733
VitusP00082710
VitusP00093736
VitusP00082712
1
0.9
0.8
0.7
0.6
0.5
0.4
0.3
0.2
0.1
0

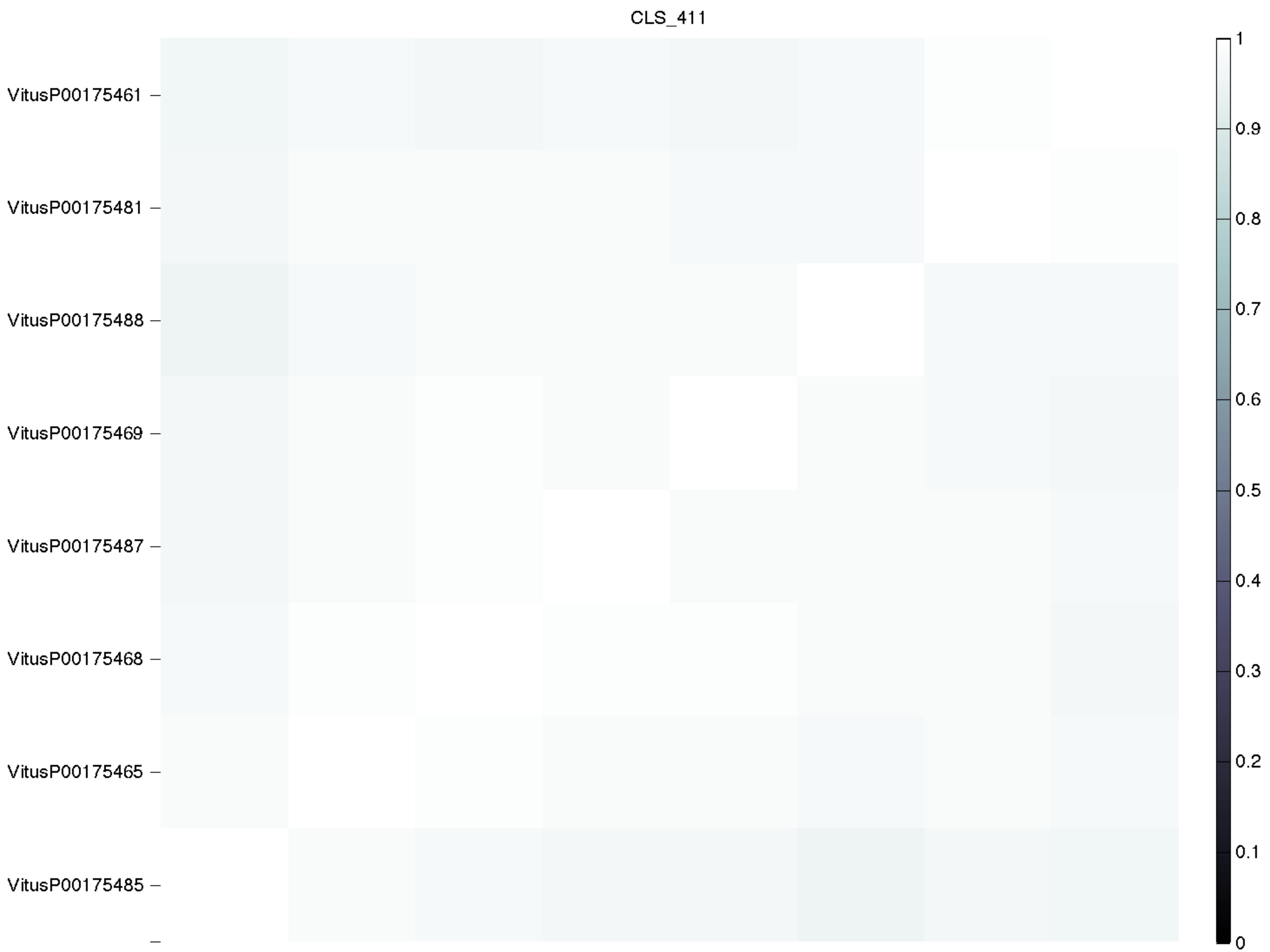
CLS_411
VitusP00175461
VitusP00175481
VitusP00175488
VitusP00175469
VitusP00175487
VitusP00175468
VitusP00175465
VitusP00175485
1
0.9
0.8
0.7
0.6
0.5
0.4
0.3
0.2
0.1
0

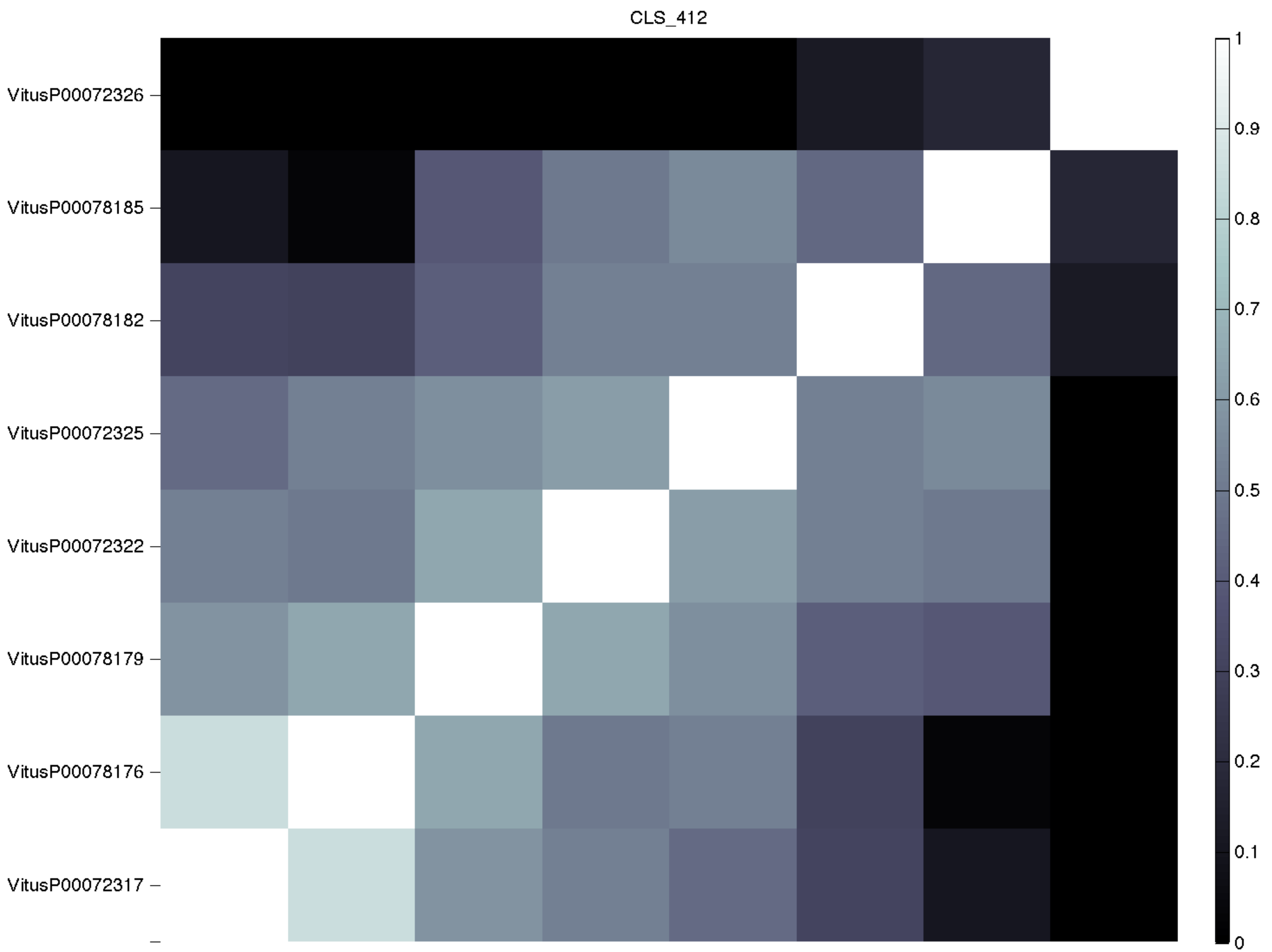
CLS_412
VitusP00072326
VitusP00078185
VitusP00078182
VitusP00072325
VitusP00072322
VitusP00078179
VitusP00078176
VitusP00072317
1
0.9
0.8
0.7
0.6
0.5
0.4
0.3
0.2
0.1
0

CLS_413

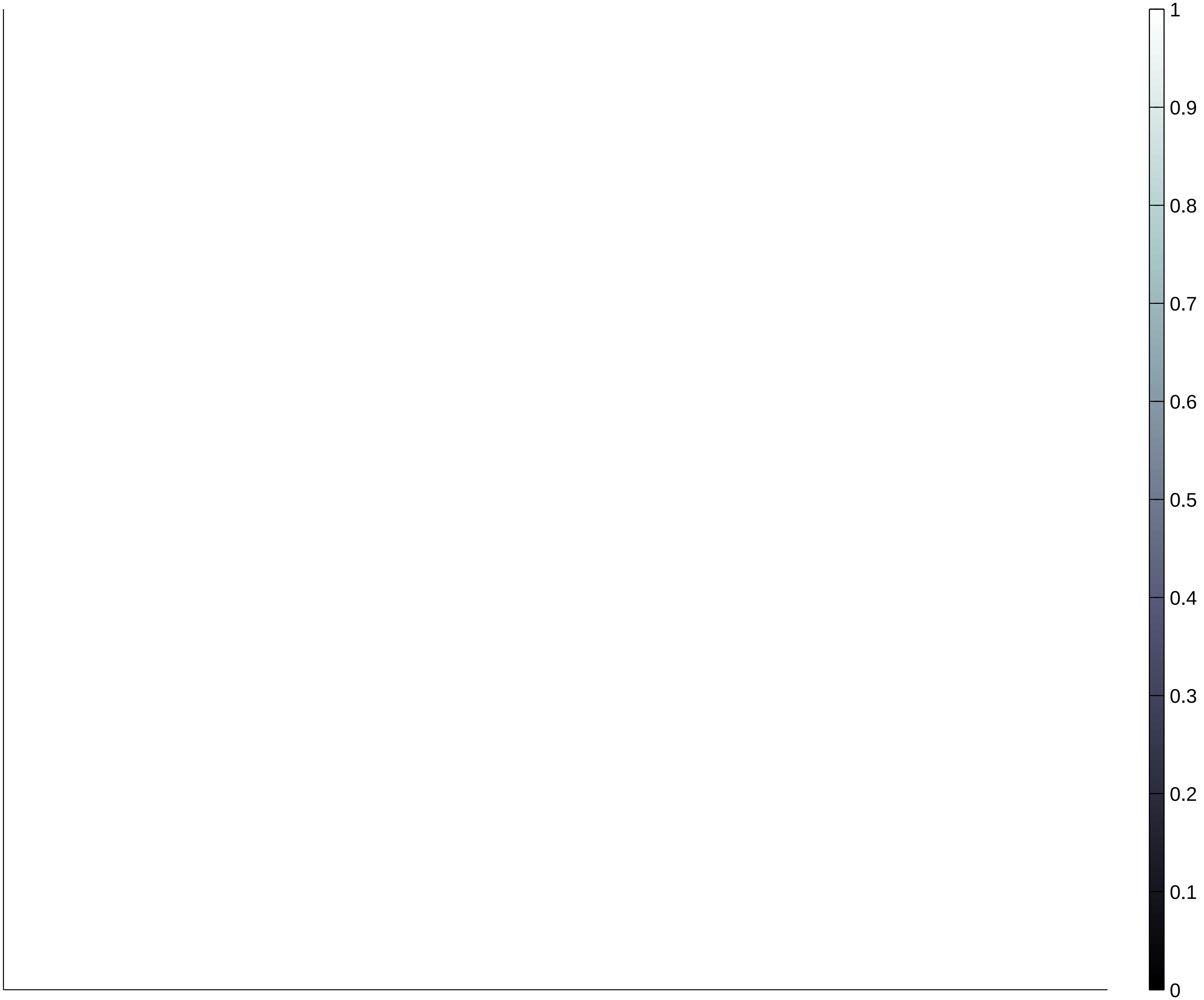

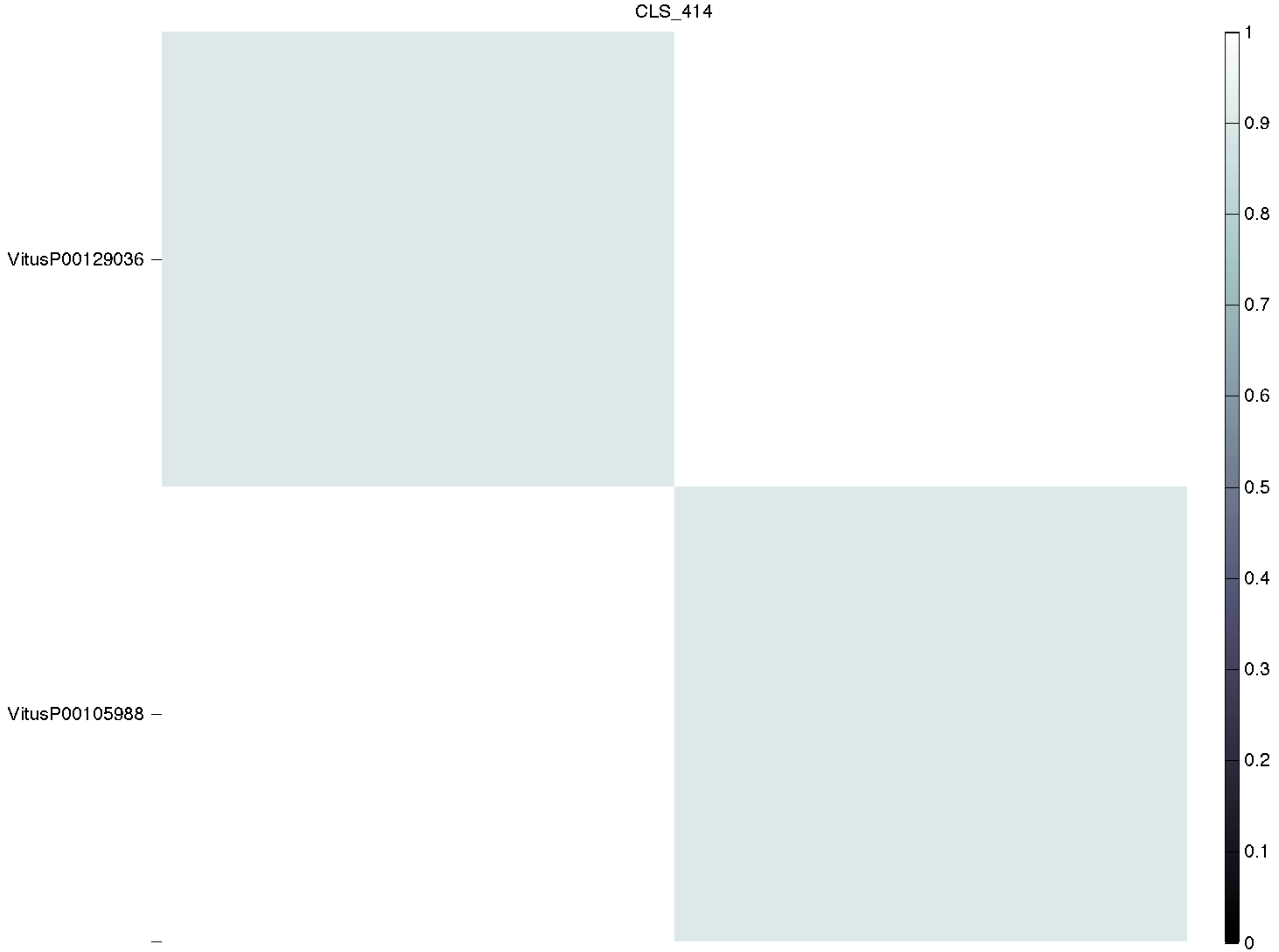
CLS_414
VitusP00129036
VitusP00105988
1
0.9
0.8
0.7
0.6
0.5
0.4
0.3
0.2
0.1
0

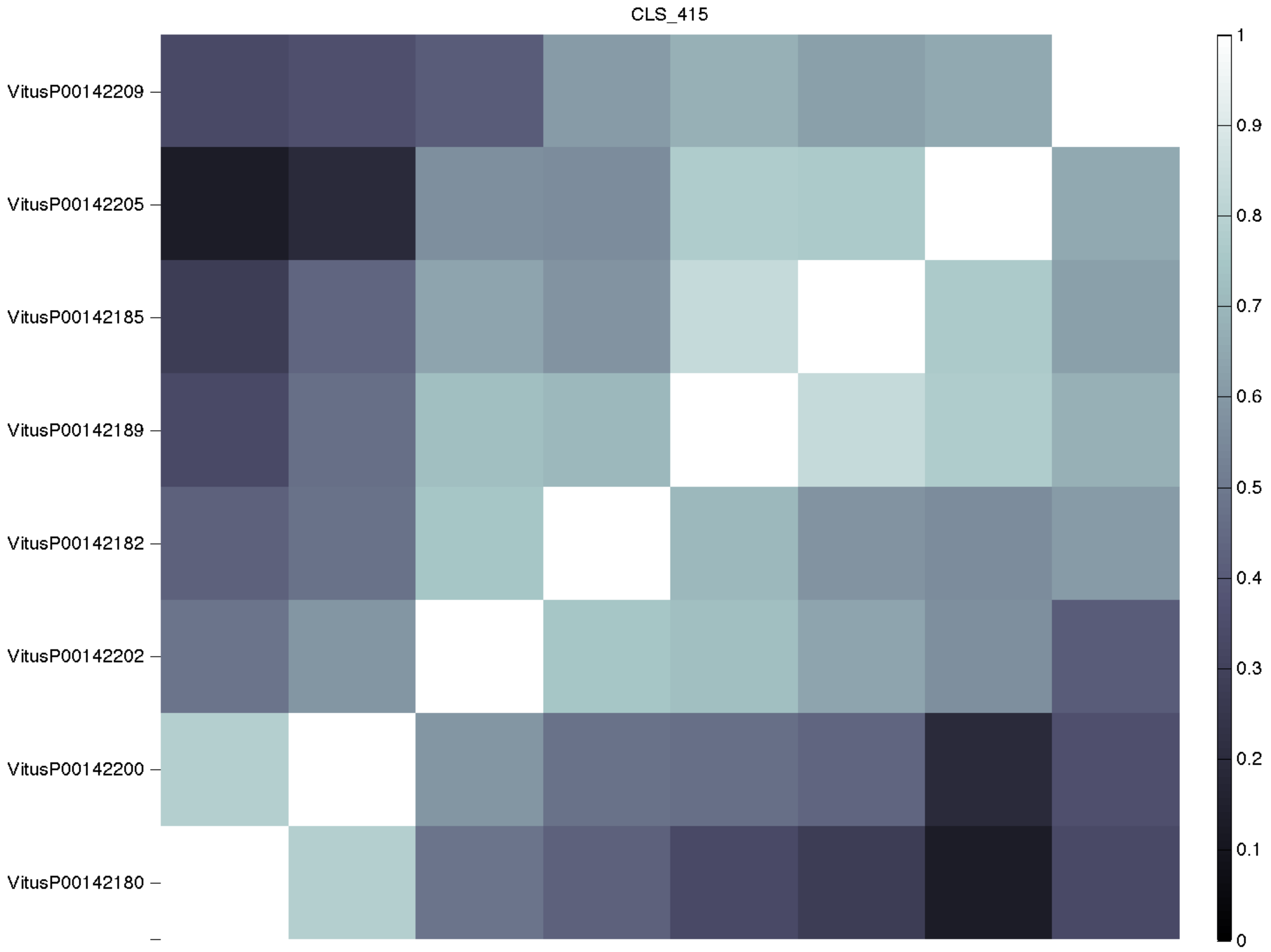

CLS_415
VitusP00142209
VitusP00142205
VitusP00142185
VitusP00142189
VitusP00142182
VitusP00142202
VitusP00142200
VitusP00142180
1
0.9
0.8
0.7
0.6
0.5
0.4
0.3
0.2
0.1
0

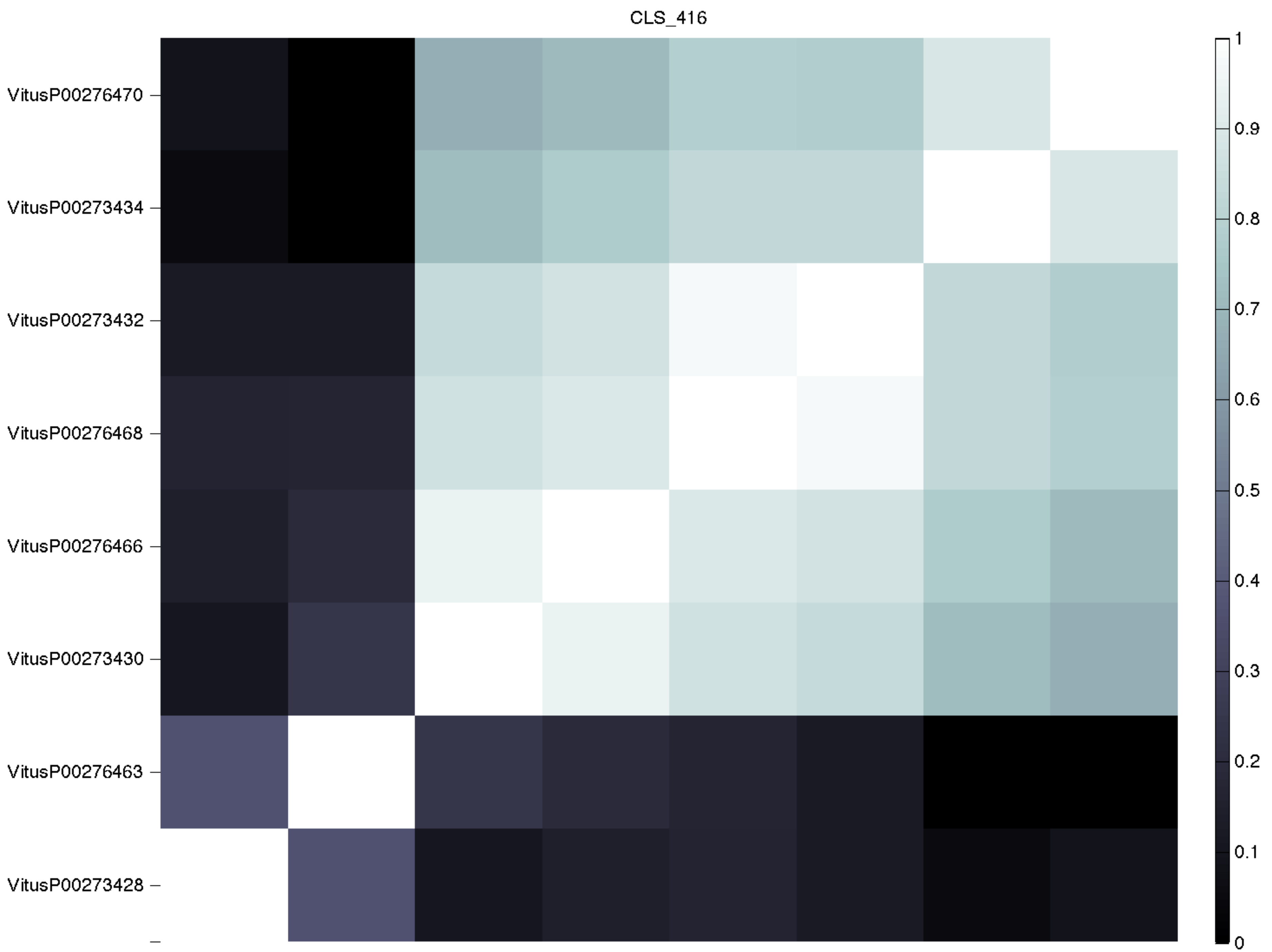
CLS_416
VitusP00276470
VitusP00273434
VitusP00273432
VitusP00276468
VitusP00276466
VitusP00273430
VitusP00276463
VitusP00273428
1
0.9
0.8
0.7
0.6
0.5
0.4
0.3
0.2
0.1
0

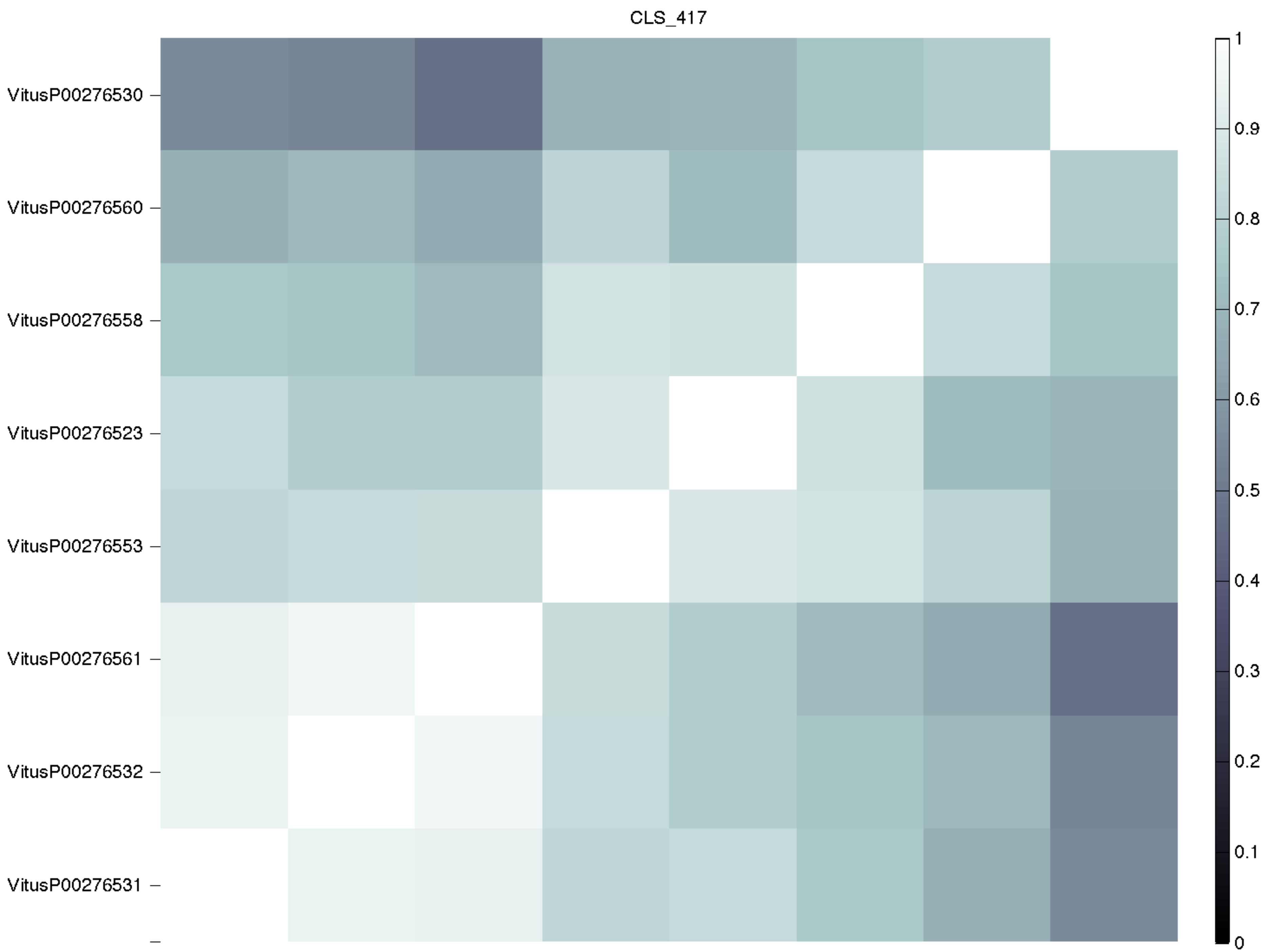
CLS_417
VitusP00276530
VitusP00276560
VitusP00276558
VitusP00276523
VitusP00276553
VitusP00276561
VitusP00276532
VitusP00276531
1
0.9
0.8
0.7
0.6
0.5
0.4
0.3
0.2
0.1
0

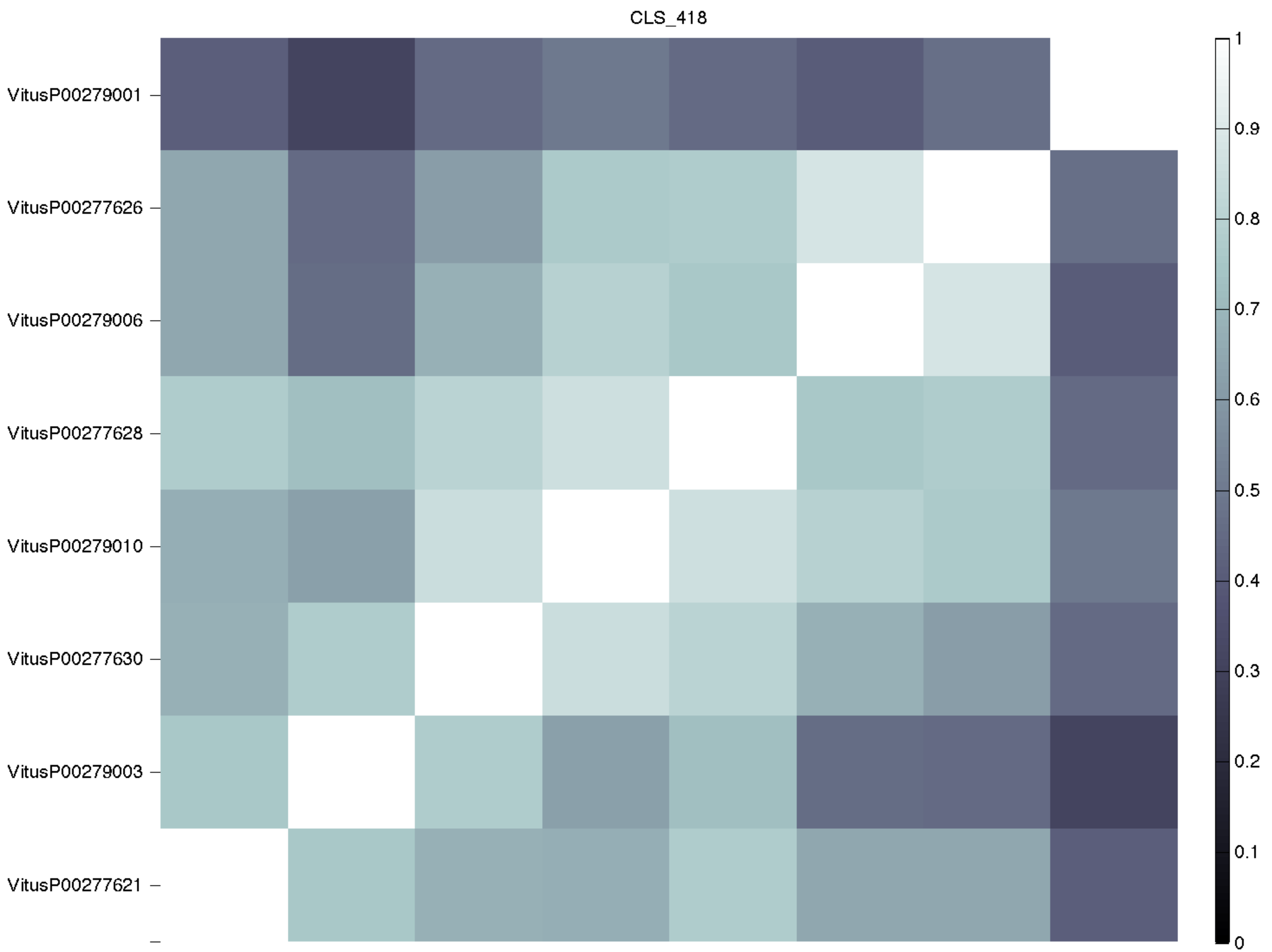
CLS_418
VitusP00279001
VitusP00277626
VitusP00279006
VitusP00277628
VitusP00279010
VitusP00277630
VitusP00279003
VitusP00277621
1
0.9
0.8
0.7
0.6
0.5
0.4
0.3
0.2
0.1
0

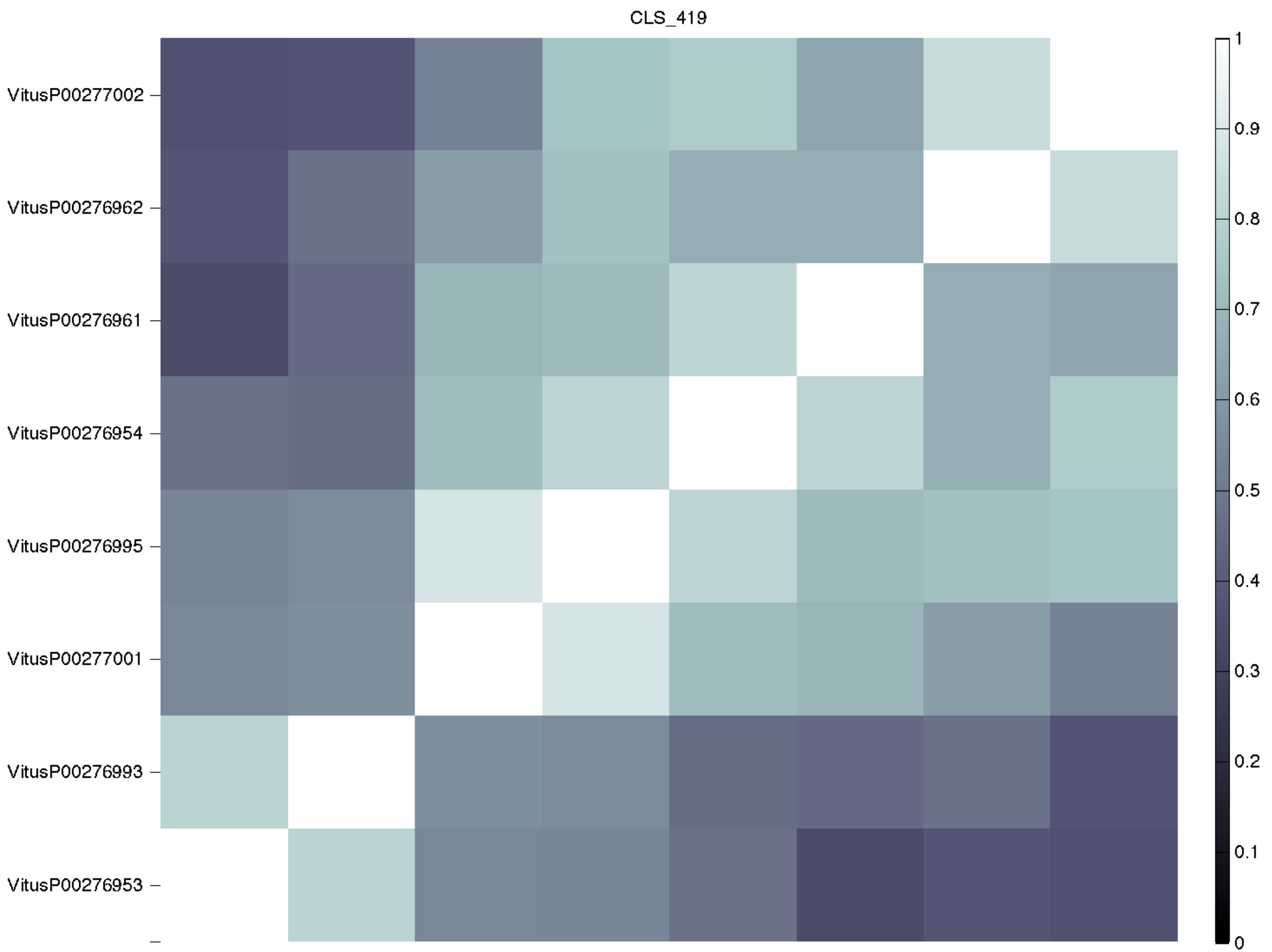
CLS_419
VitusP00277002
VitusP00276962
VitusP00276961
VitusP00276954
VitusP00276995
VitusP00277001
VitusP00276993
VitusP00276953
1
0.9
0.8
0.7
0.6
0.5
0.4
0.3
0.2
0.1
0

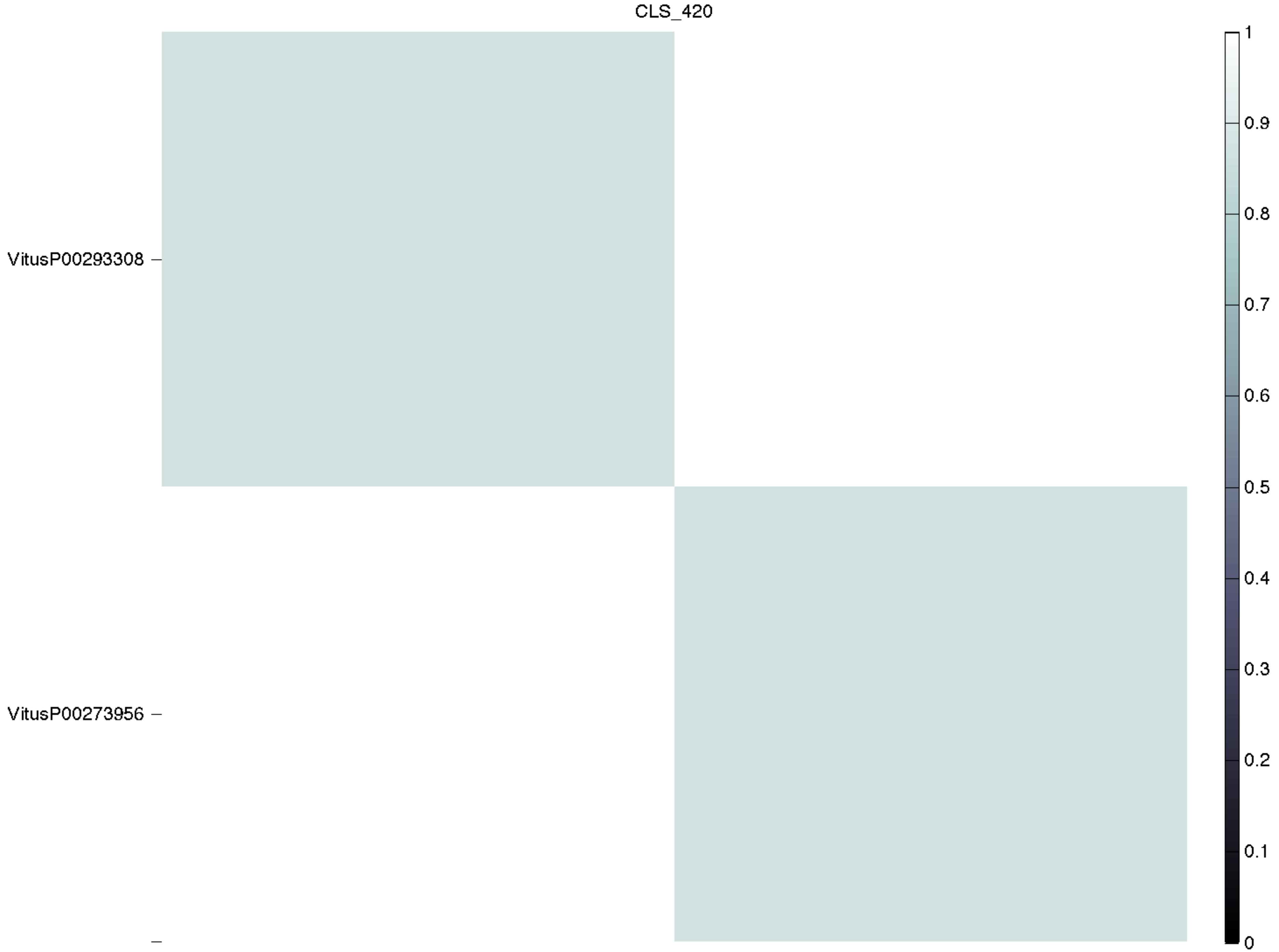
CLS_420
VitusP00293308
VitusP00273956
1
0.9
0.8
0.7
0.6
0.5
0.4
0.3
0.2
0.1
0

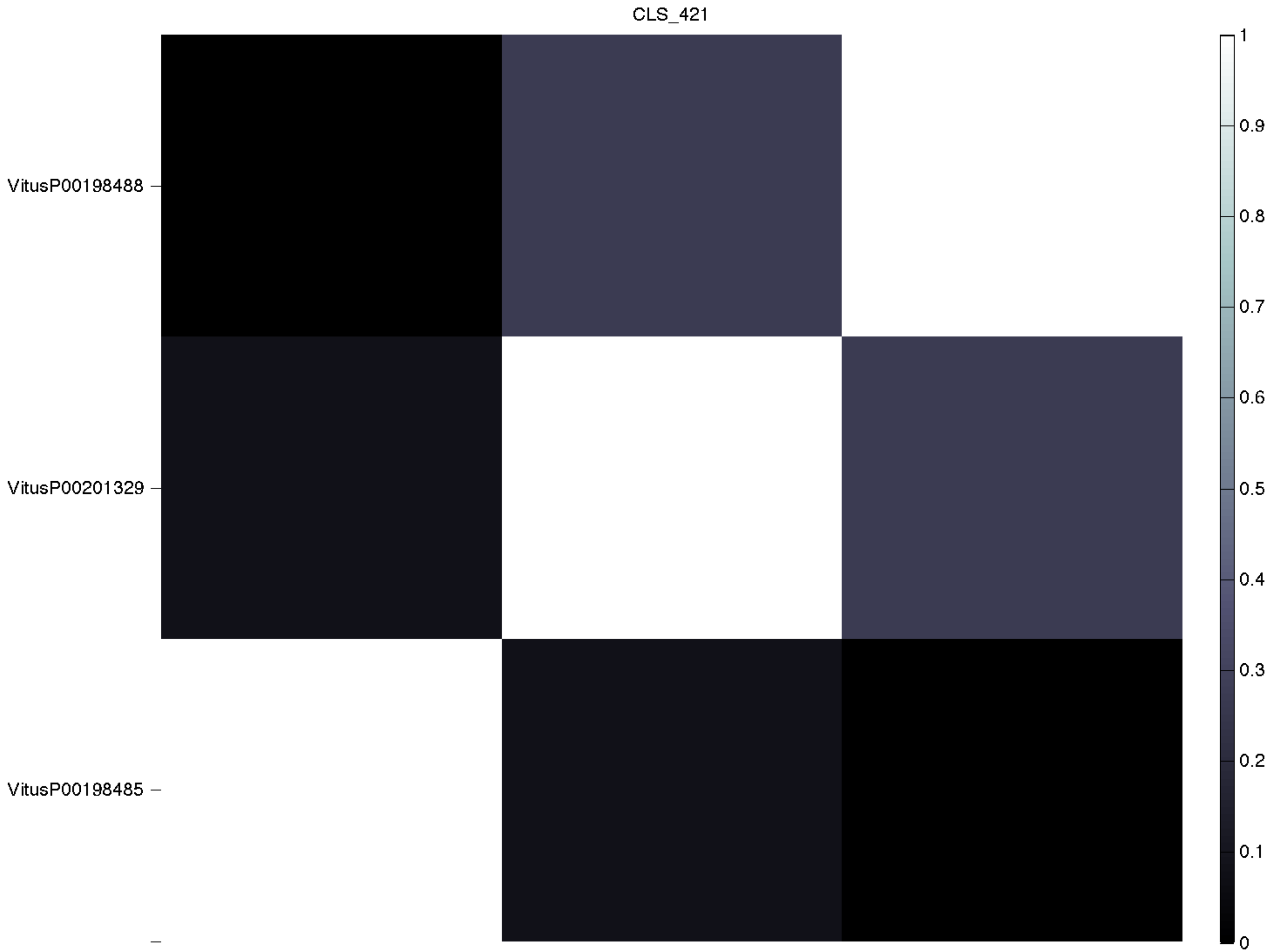
CLS_421
VitusP00198488
VitusP00201329
VitusP00198485
1
0.9
0.8
0.7
0.6
0.5
0.4
0.3
0.2
0.1
0

CLS_422

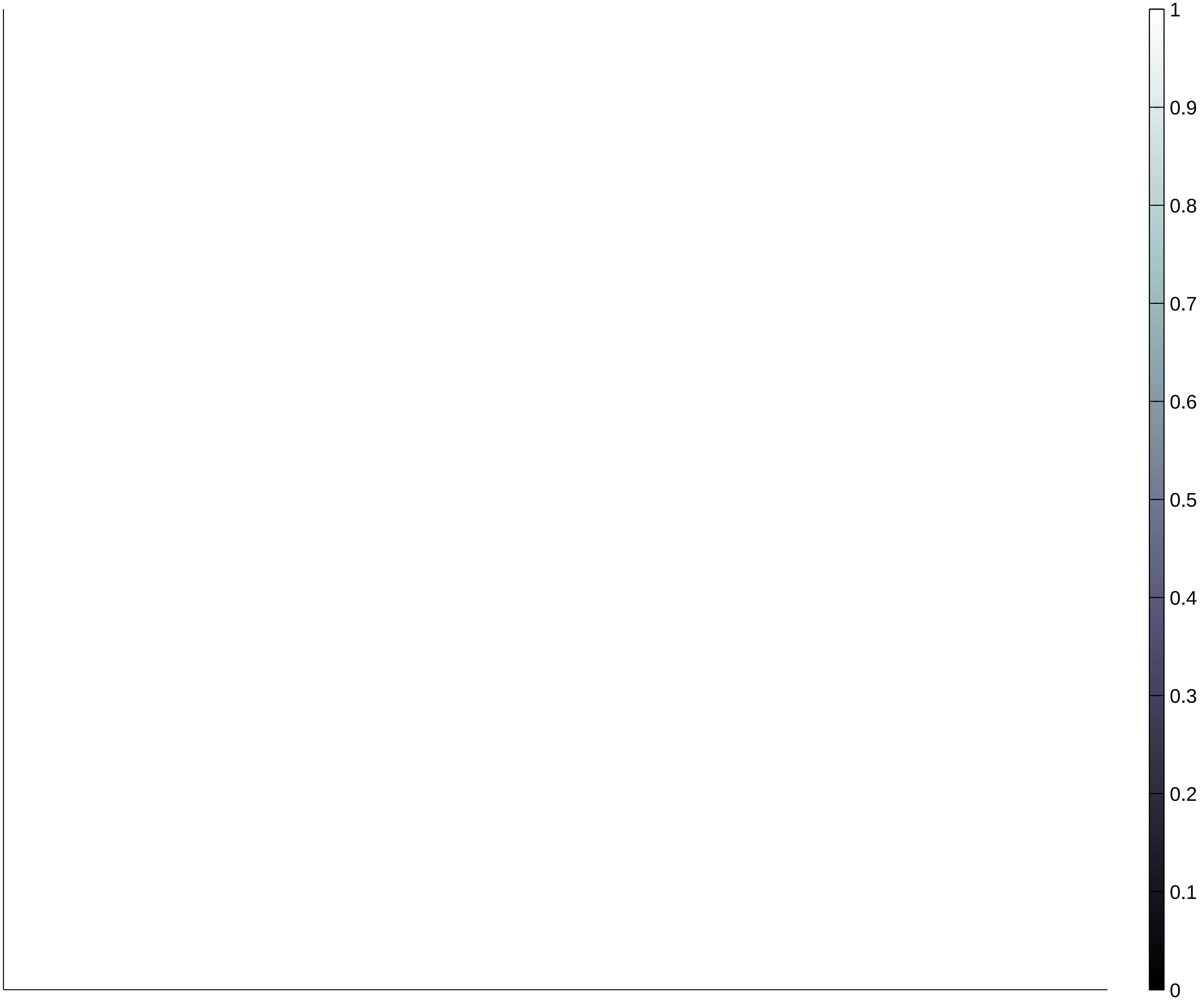

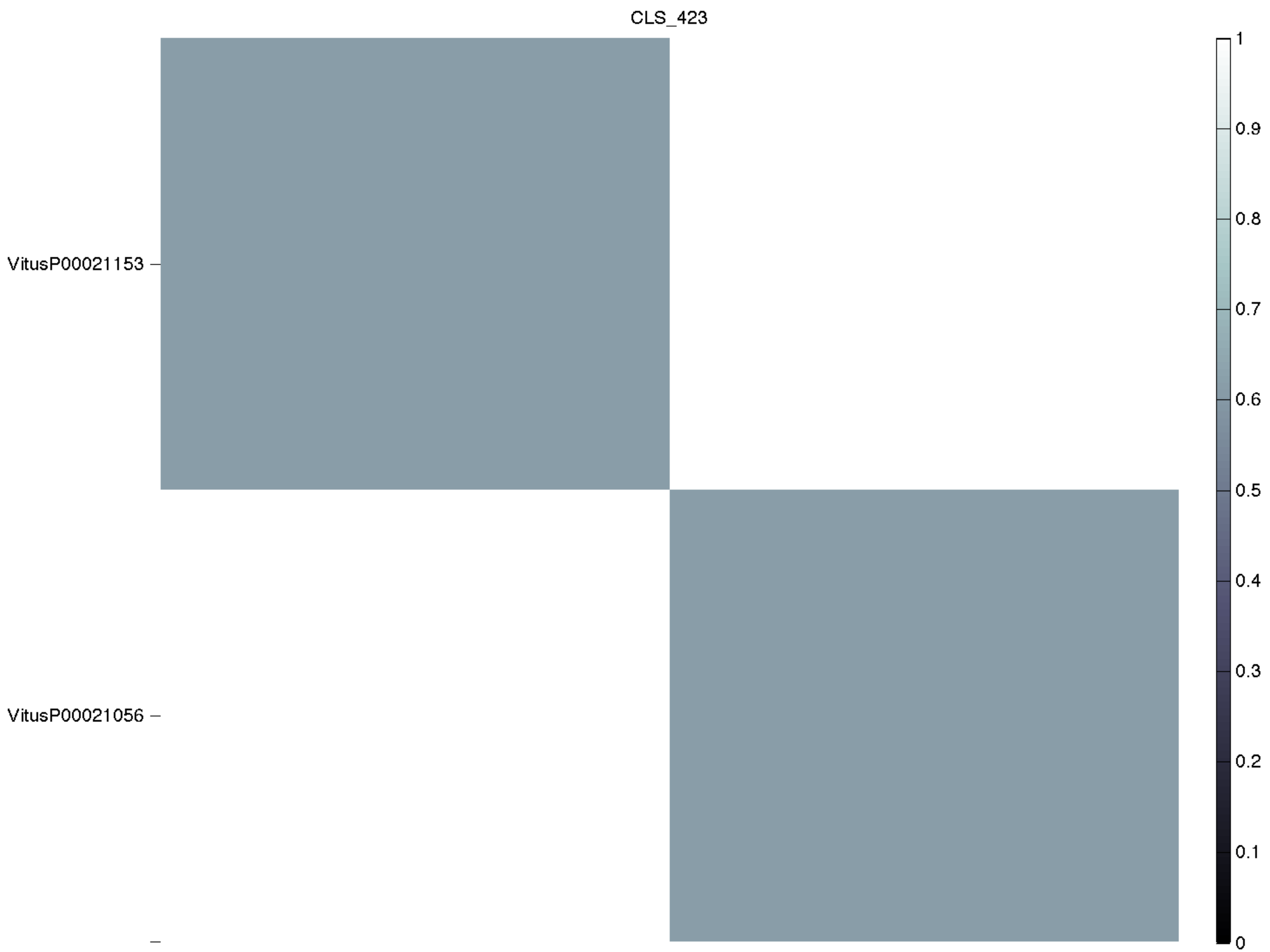
CLS_423
VitusP00021153
VitusP00021056
1
0.9
0.8
0.7
0.6
0.5
0.4
0.3
0.2
0.1
0

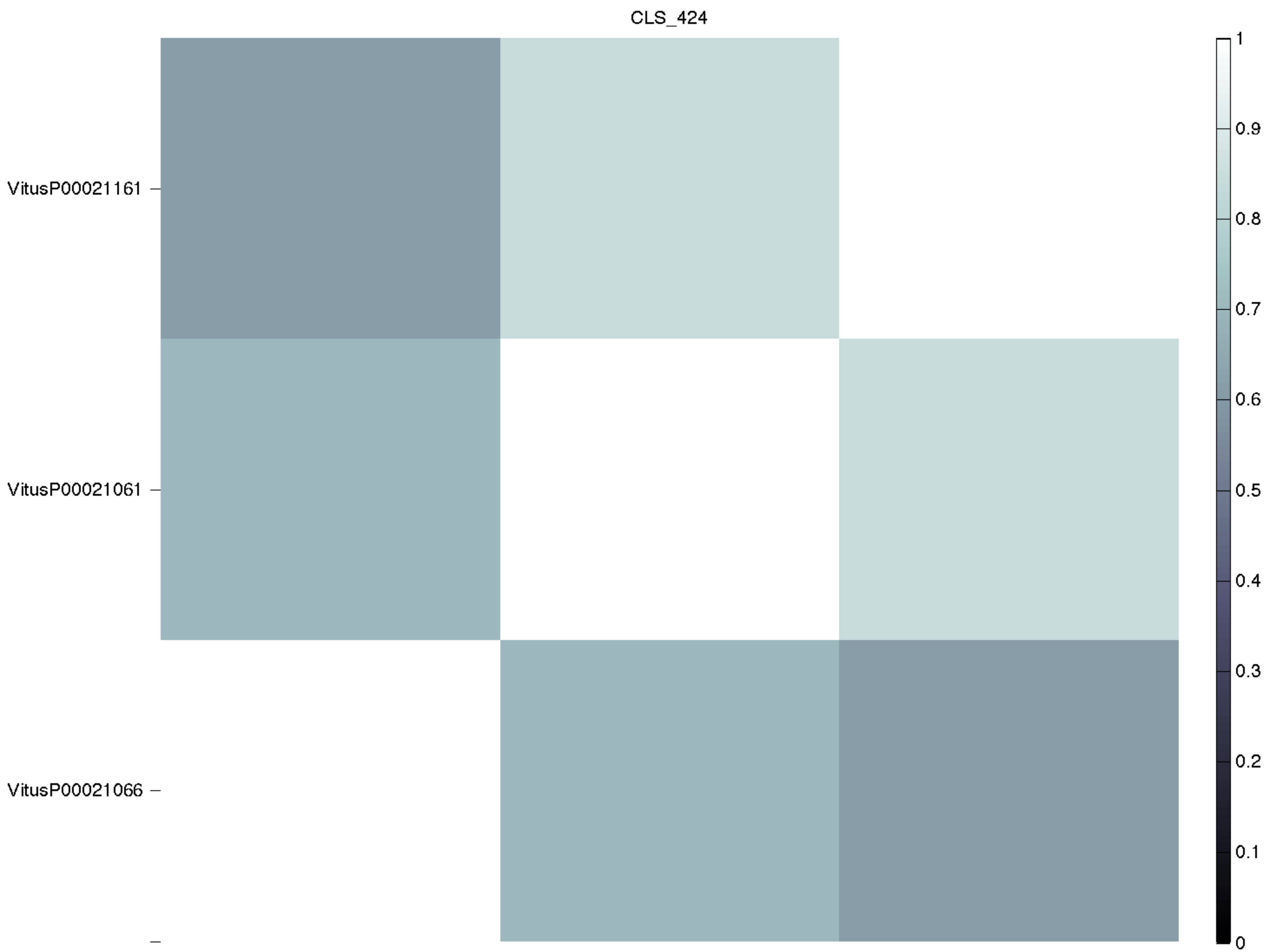
CLS_424
VitusP00021161
VitusP00021061
VitusP00021066
1
0.9
0.8
0.7
0.6
0.5
0.4
0.3
0.2
0.1
0

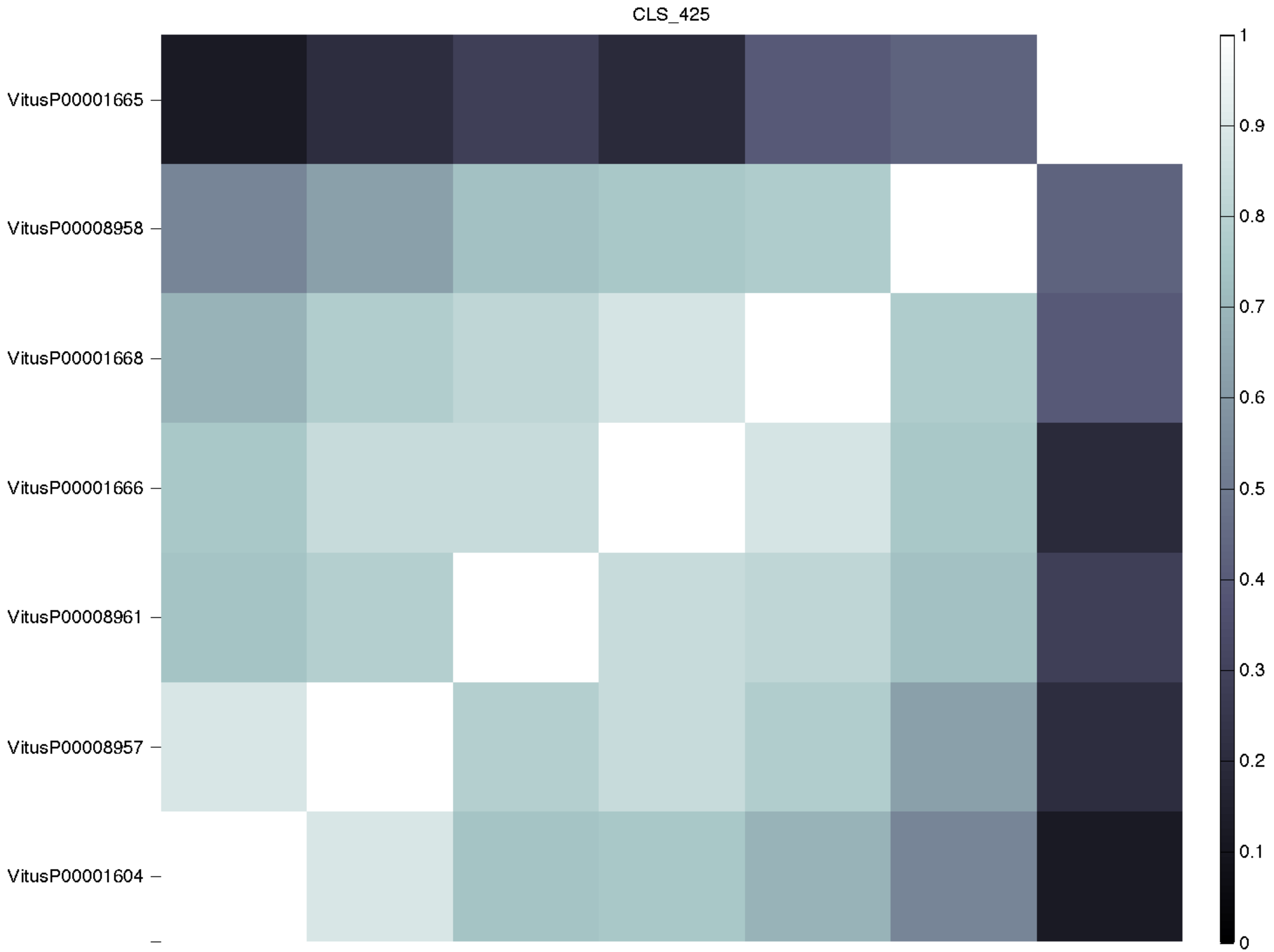
CLS_425
VitusP00001665
VitusP00008958
VitusP00001668
VitusP00001666
VitusP00008961
VitusP00008957
VitusP00001604
1
0.9
0.8
0.7
0.6
0.5
0.4
0.3
0.2
0.1
0

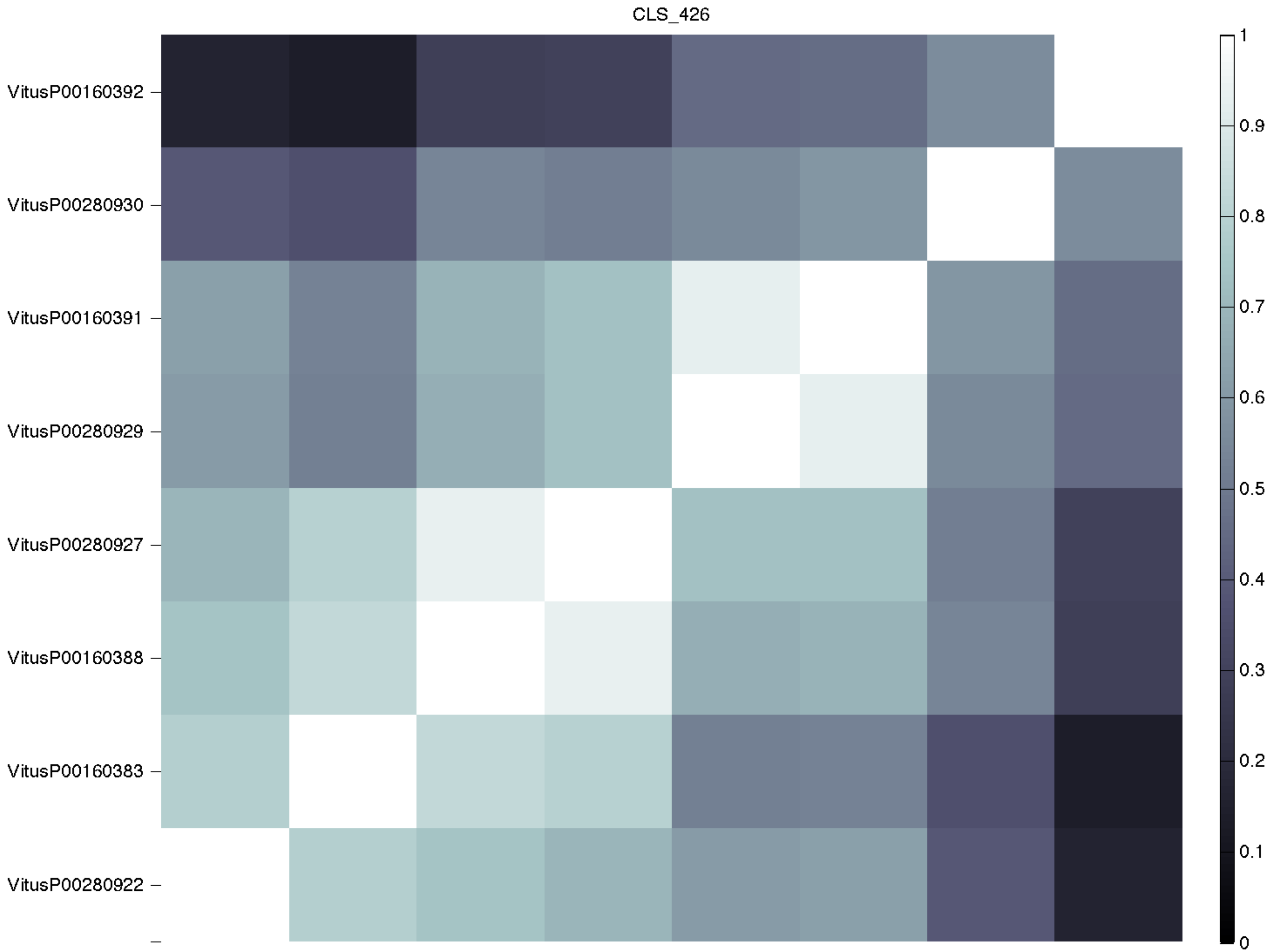
CLS_426
VitusP00160392
VitusP00280930
VitusP00160391
VitusP00280929
VitusP00280927
VitusP00160388
VitusP00160383
VitusP00280922
1
0.9
0.8
0.7
0.6
0.5
0.4
0.3
0.2
0.1
0

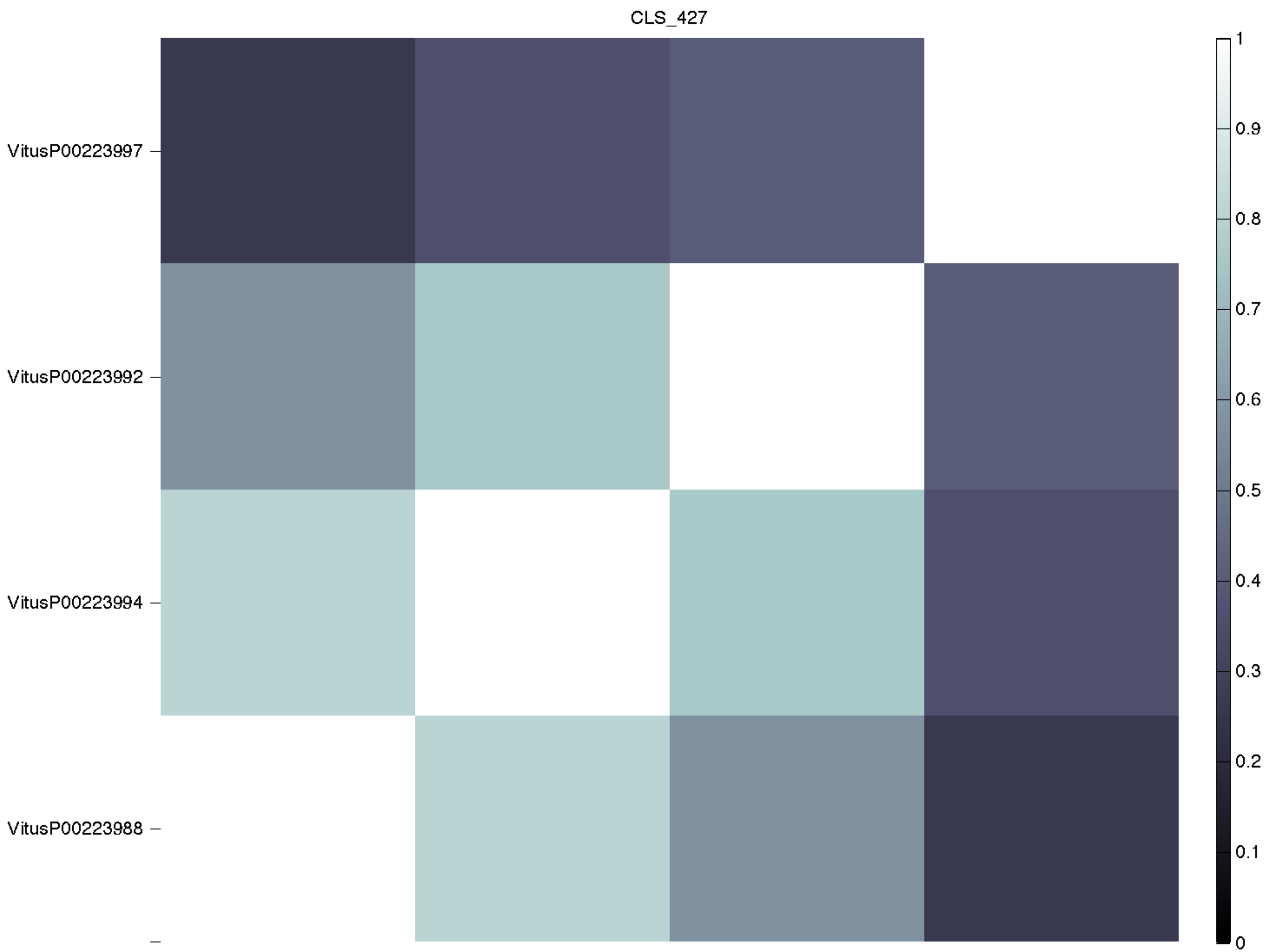
CLS_427
VitusP00223997
VitusP00223992
VitusP00223994
VitusP00223988
1
0.9
0.8
0.7
0.6
0.5
0.4
0.3
0.2
0.1
0

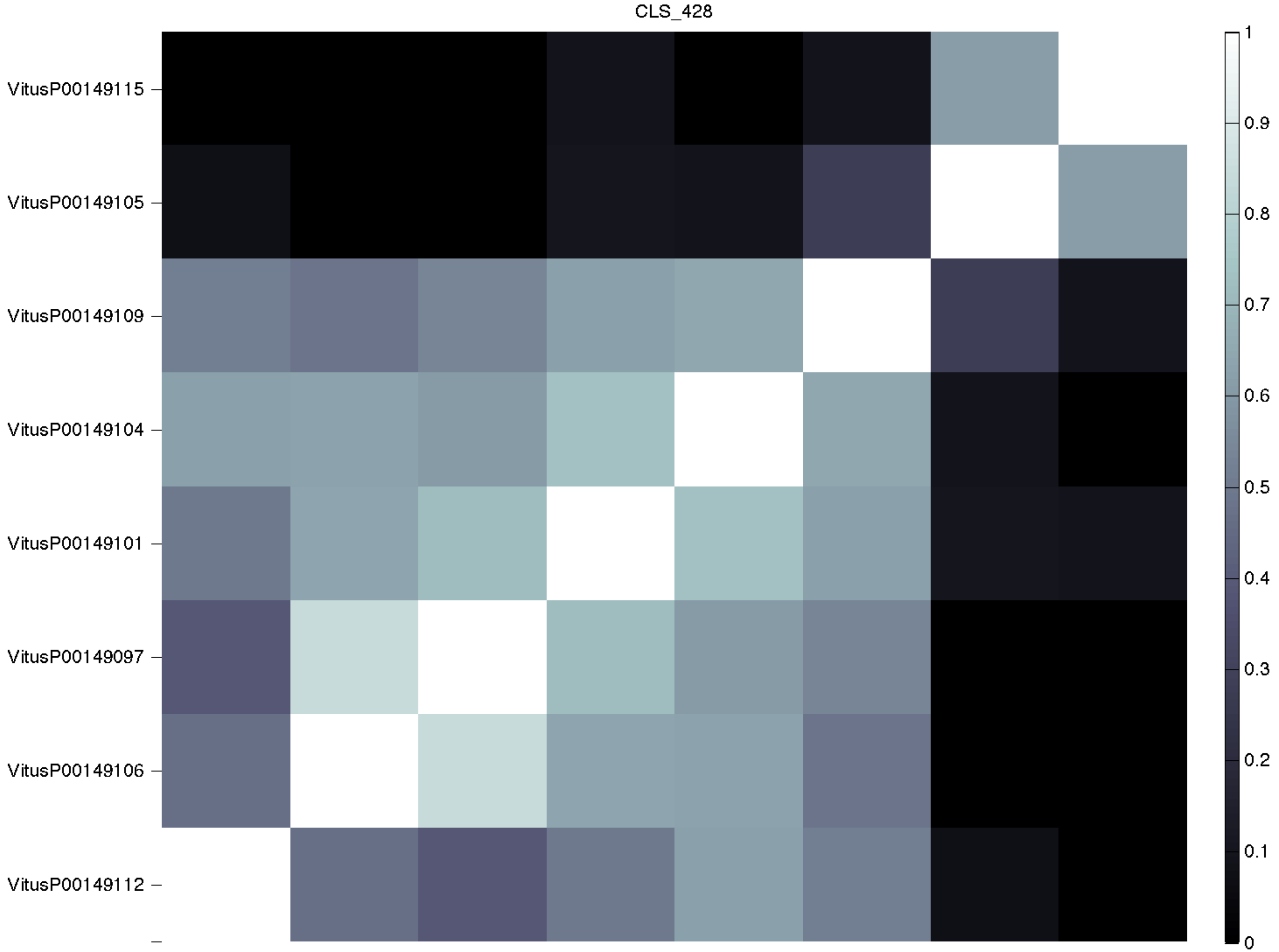

CLS_428
VitusP00149115
VitusP00149105
VitusP00149109
VitusP00149104
VitusP00149101
VitusP00149097
VitusP00149106
VitusP00149112
1
0.9
0.8
0.7
0.6
0.5
0.4
0.3
0.2
0.1
0

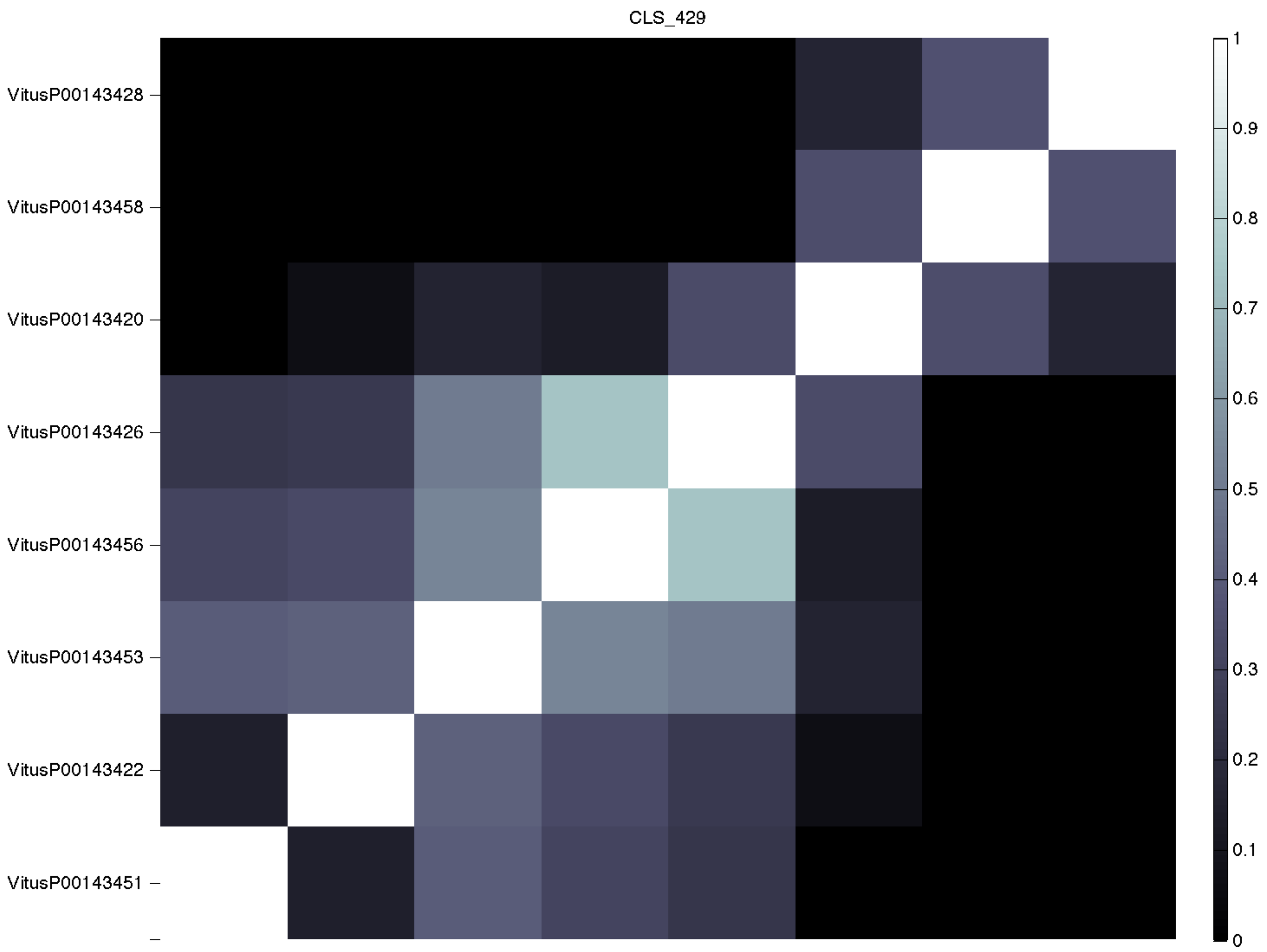
CLS_429
VitusP00143428
VitusP00143458
VitusP00143420
VitusP00143426
VitusP00143456
VitusP00143453
VitusP00143422
VitusP00143451
1
0.9
0.8
0.7
0.6
0.5
0.4
0.3
0.2
0.1
0

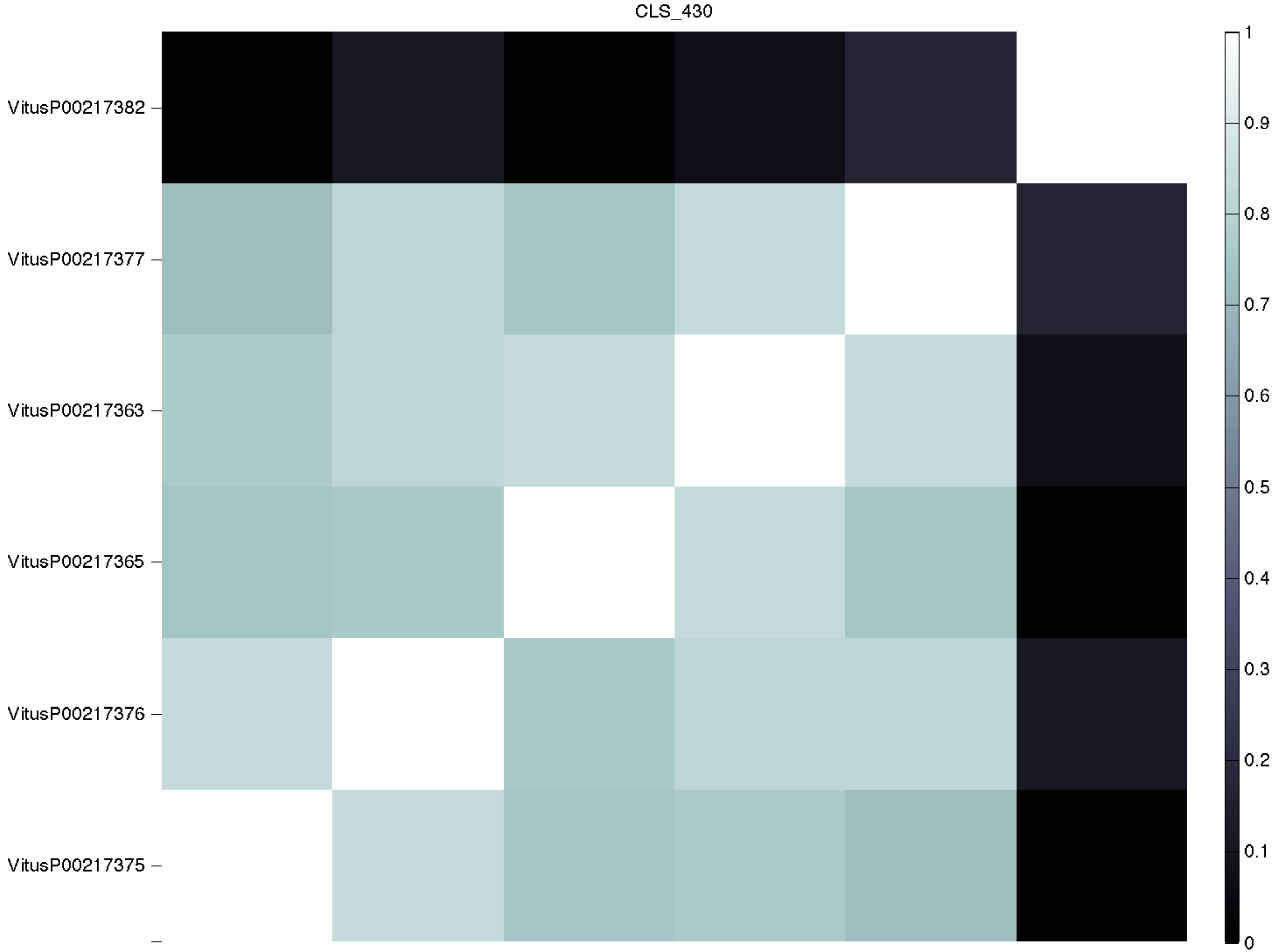
CLS_430
VitusP00217382
VitusP00217377
VitusP00217363
VitusP00217365
VitusP00217376
VitusP00217375
1
0.9
0.8
0.7
0.6
0.5
0.4
0.3
0.2
0.1
0

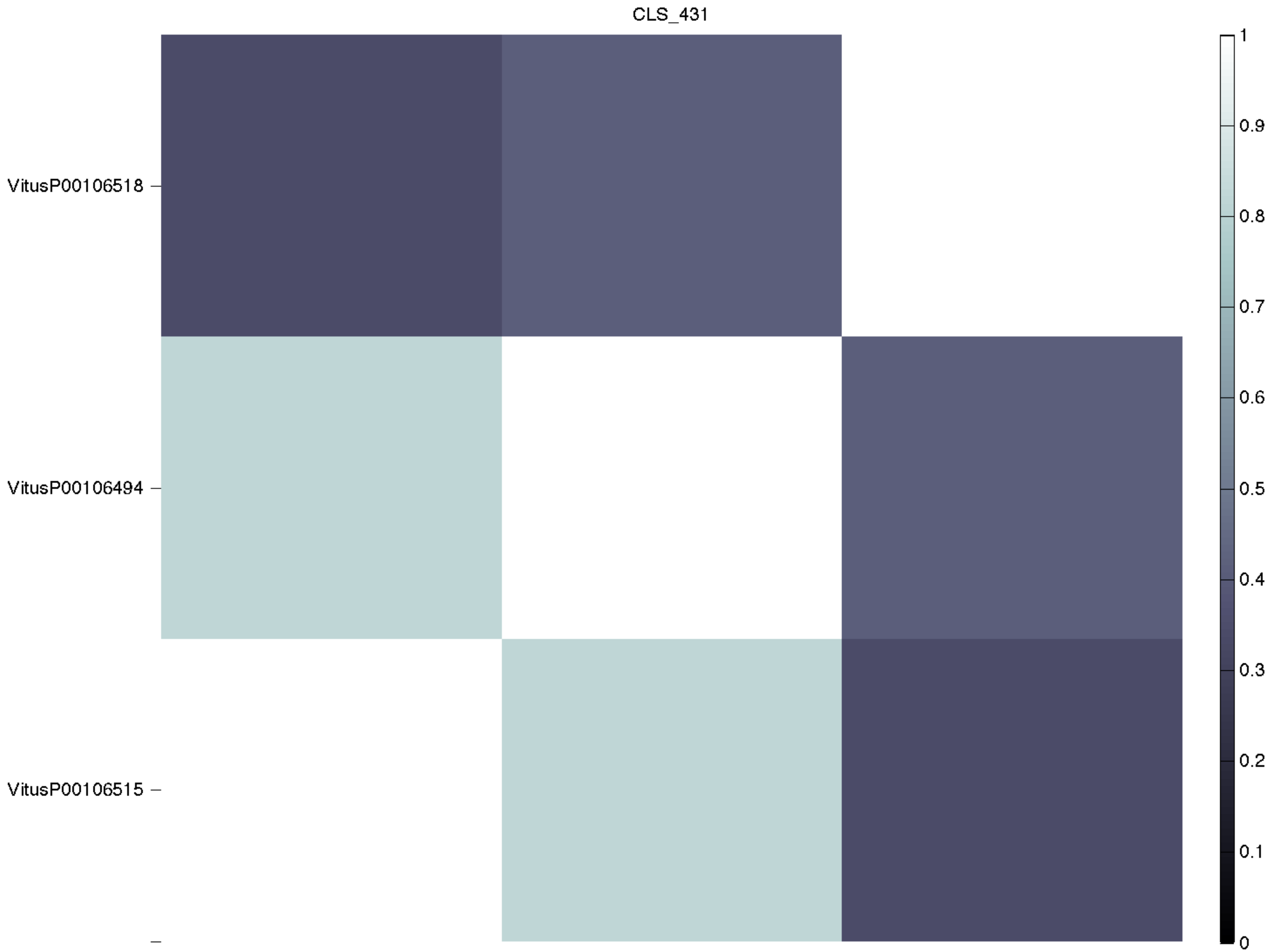

CLS_431
VitusP00106518
VitusP00106494
VitusP00106515
1
0.9
0.8
0.7
0.6
0.5
0.4
0.3
0.2
0.1
0

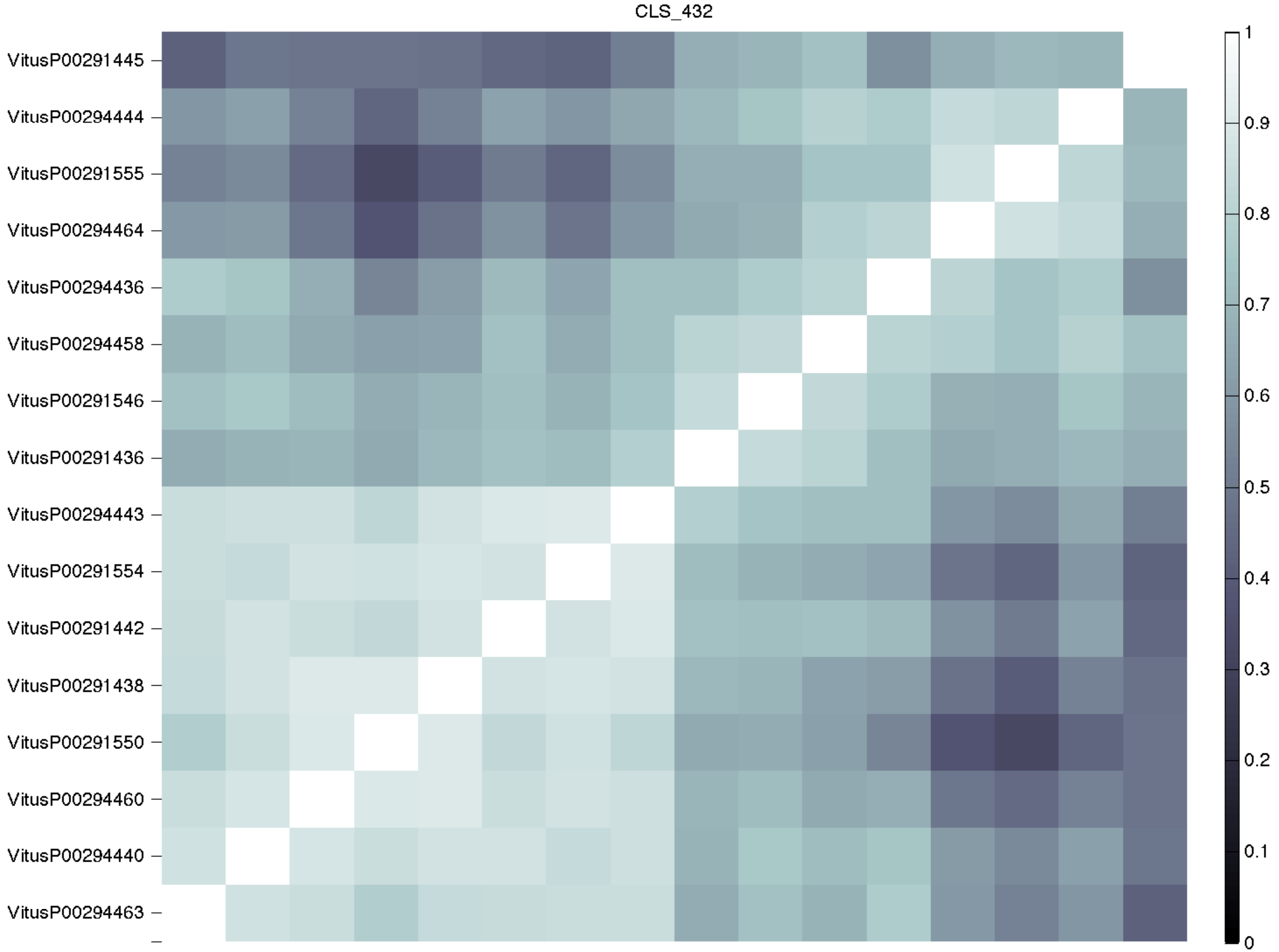
CLS_432
VitusP00291445
VitusP00294444
VitusP00291555
VitusP00294464
VitusP00294436
VitusP00294458
VitusP00291546
VitusP00291436
VitusP00294443
VitusP00291554
VitusP00291442
VitusP00291438
VitusP00291550
VitusP00294460
VitusP00294440
VitusP00294463
1
0.9
0.8
0.7
0.6
0.5
0.4
0.3
0.2
0.1
0

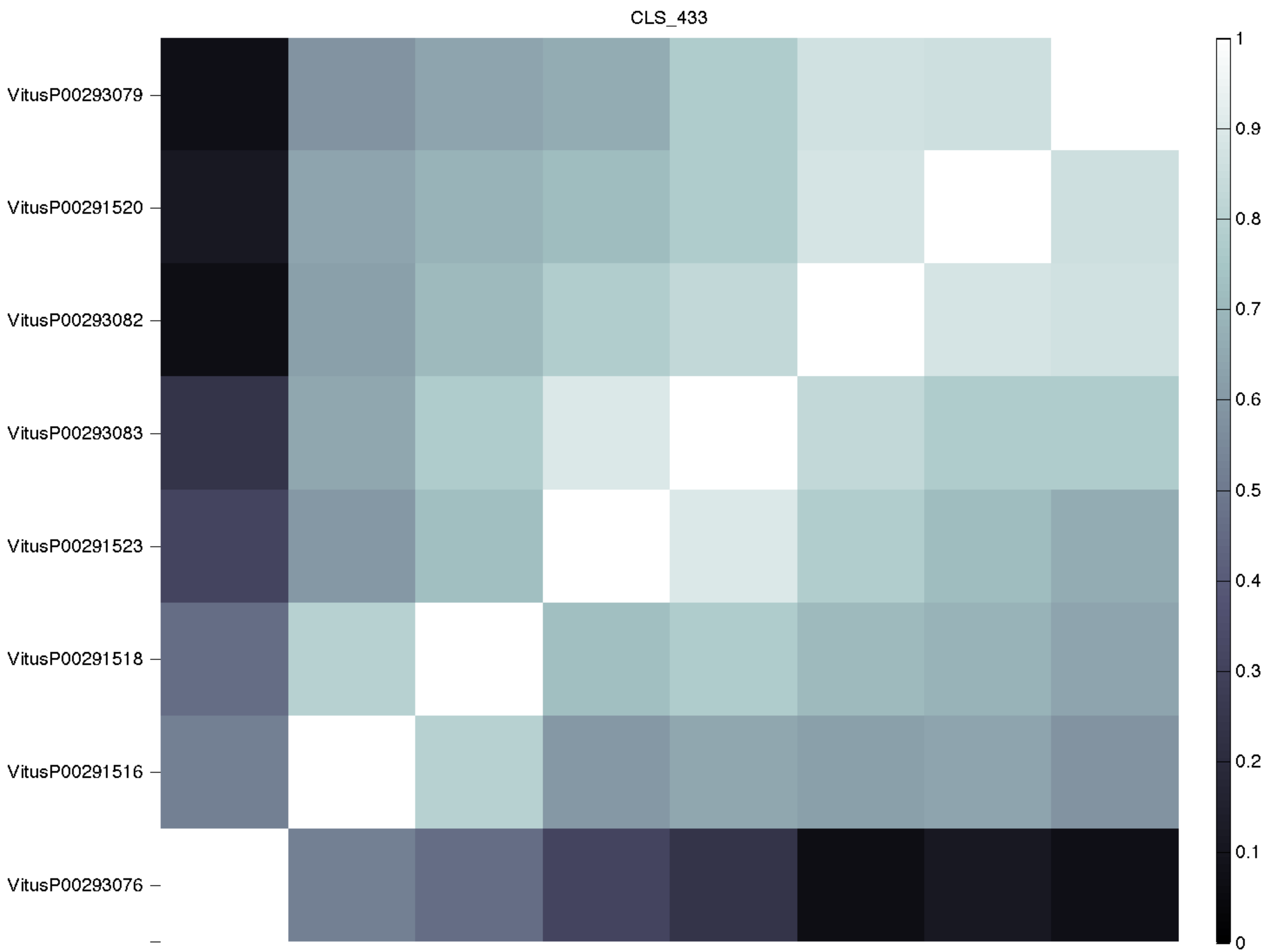

CLS_433
VitusP00293079
VitusP00291520
VitusP00293082
VitusP00293083
VitusP00291523
VitusP00291518
VitusP00291516
VitusP00293076
1
0.9
0.8
0.7
0.6
0.5
0.4
0.3
0.2
0.1
0

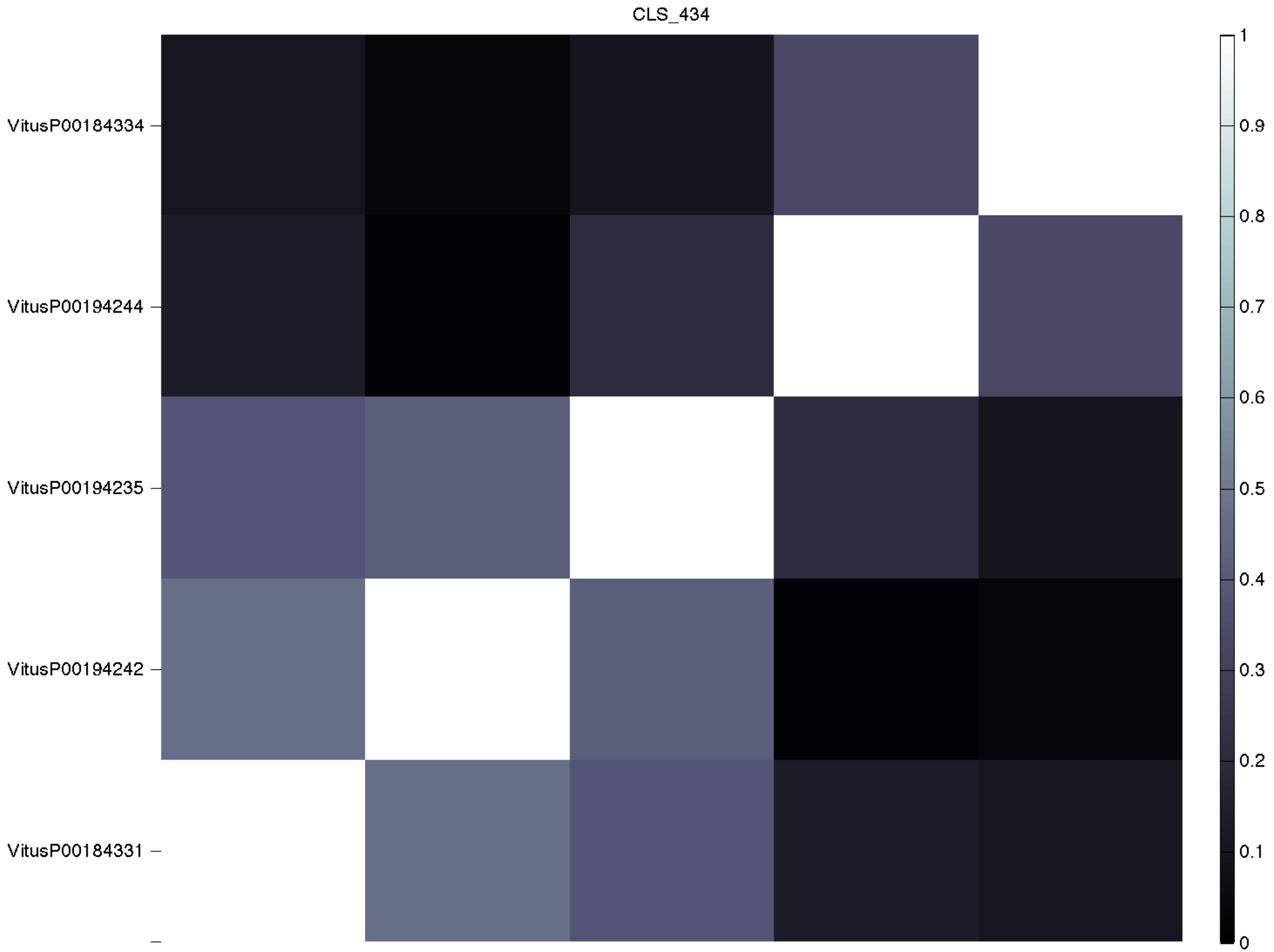
CLS_434
VitusP00184334
VitusP00194244
VitusP00194235
VitusP00194242
VitusP00184331
1
0.9
0.8
0.7
0.6
0.5
0.4
0.3
0.2
0.1
0

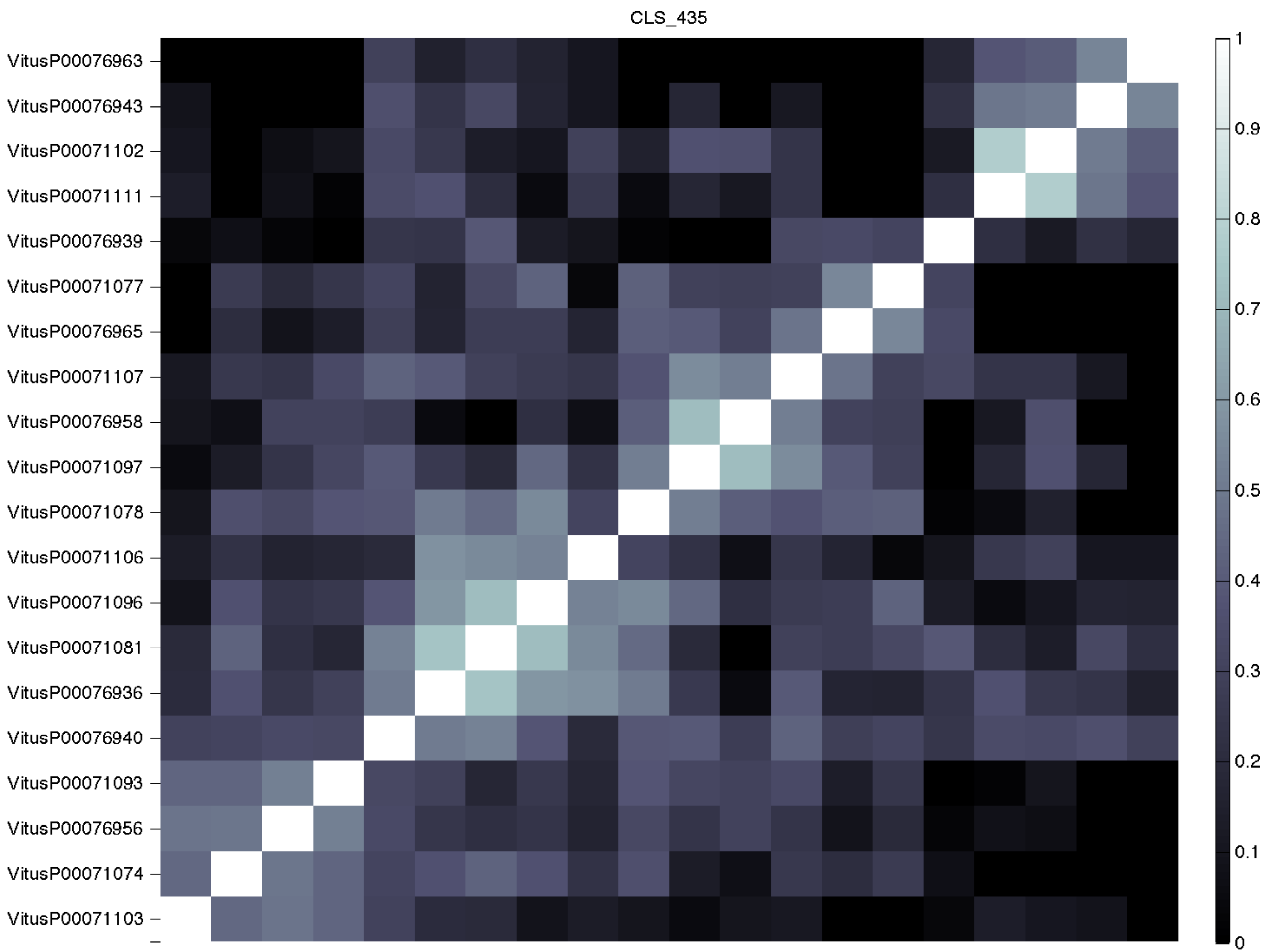

CLS_435
VitusP00076963
VitusP00076943
VitusP00071102
VitusP00071111
VitusP00076939
VitusP00071077
VitusP00076965
VitusP00071107
VitusP00076958
VitusP00071097
VitusP00071078
VitusP00071106
VitusP00071096
VitusP00071081
VitusP00076936
VitusP00076940
VitusP00071093
VitusP00076956
VitusP00071074
VitusP00071103
1
0.9
0.8
0.7
0.6
0.5
0.4
0.3
0.2
0.1
0

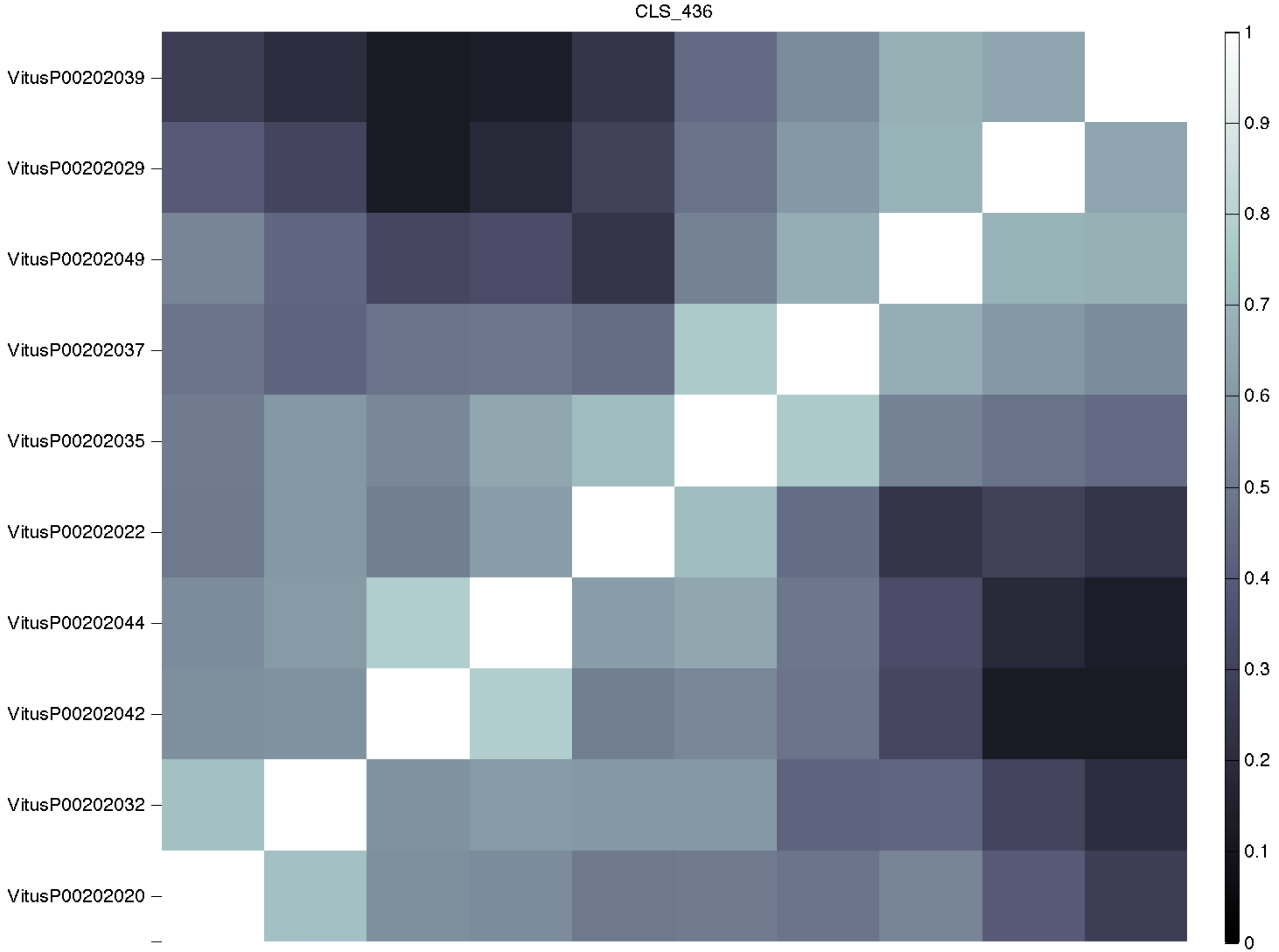

CLS_436
VitusP00202039
VitusP00202029
VitusP00202049
VitusP00202037
VitusP00202035
VitusP00202022
VitusP00202044
VitusP00202042
VitusP00202032
VitusP00202020
1
0.9
0.8
0.7
0.6
0.5
0.4
0.3
0.2
0.1
0

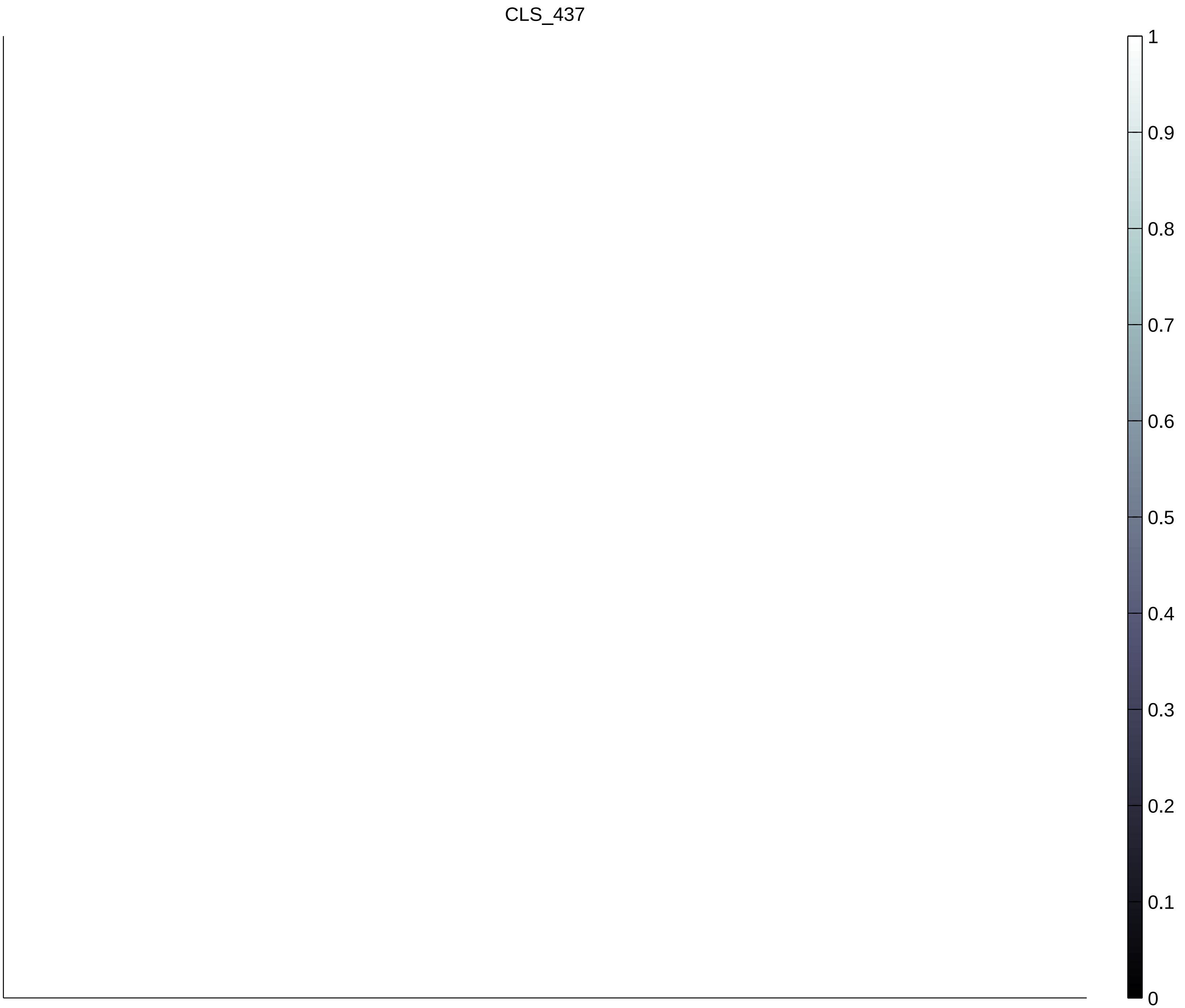
CLS_437
1
0.9
0.8
0.7
0.6
0.5
0.4
0.3
0.2
0.1
0

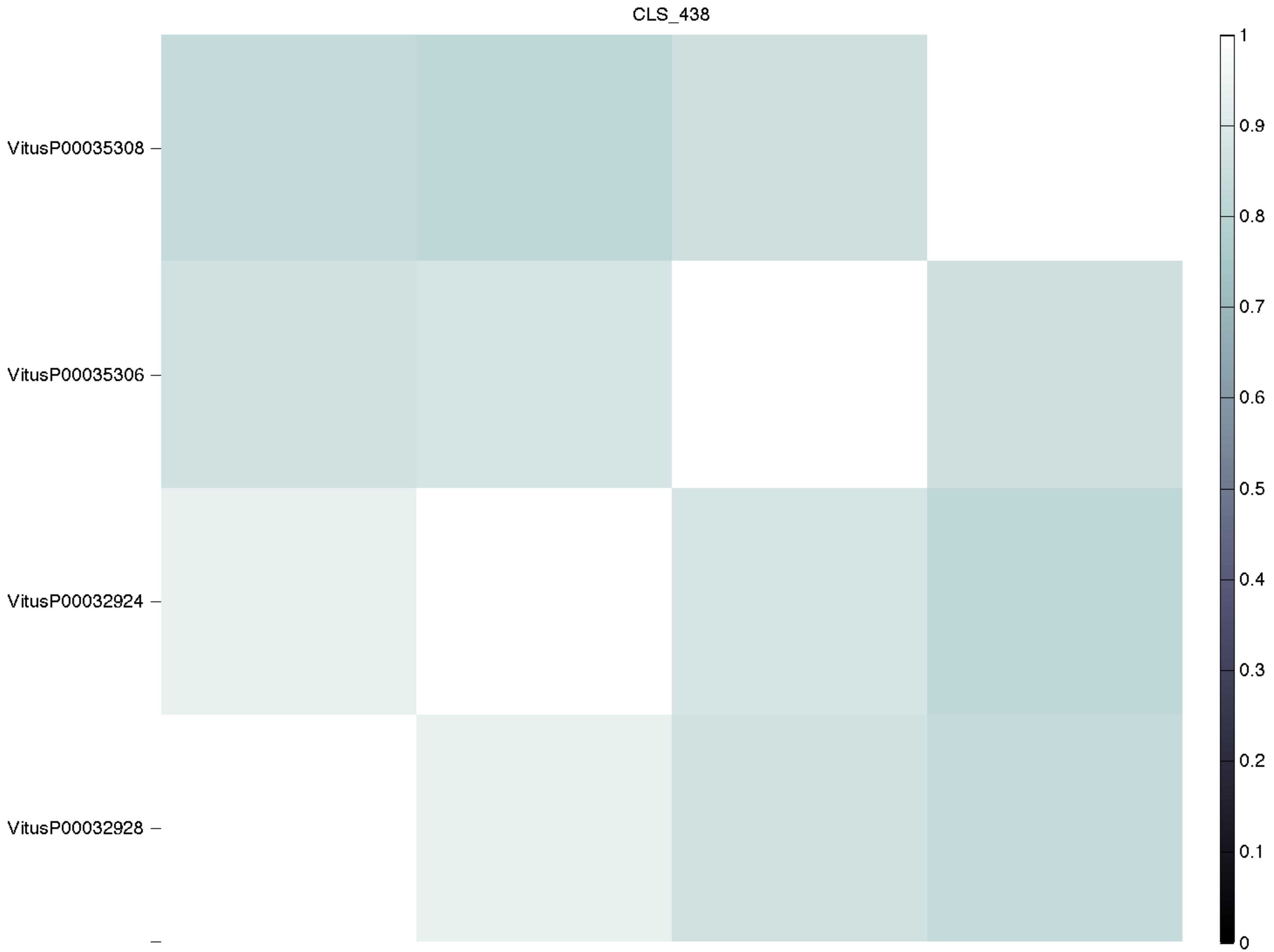
CLS_438
VitusP00035308
VitusP00035306
VitusP00032924
VitusP00032928
1
0.9
0.8
0.7
0.6
0.5
0.4
0.3
0.2
0.1
0

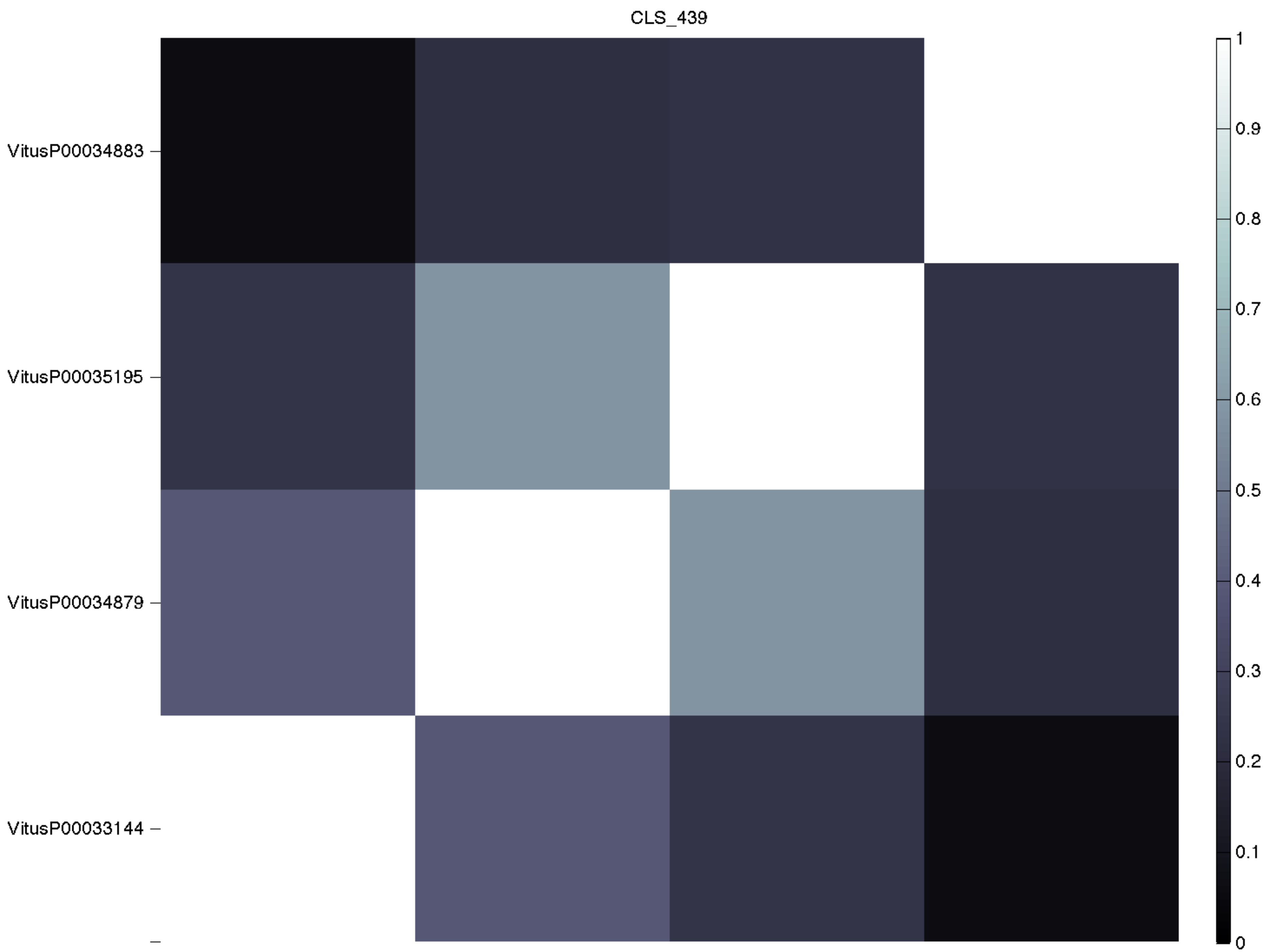
CLS_439
VitusP00034883
VitusP00035195
VitusP00034879
VitusP00033144
1
0.9
0.8
0.7
0.6
0.5
0.4
0.3
0.2
0.1
0

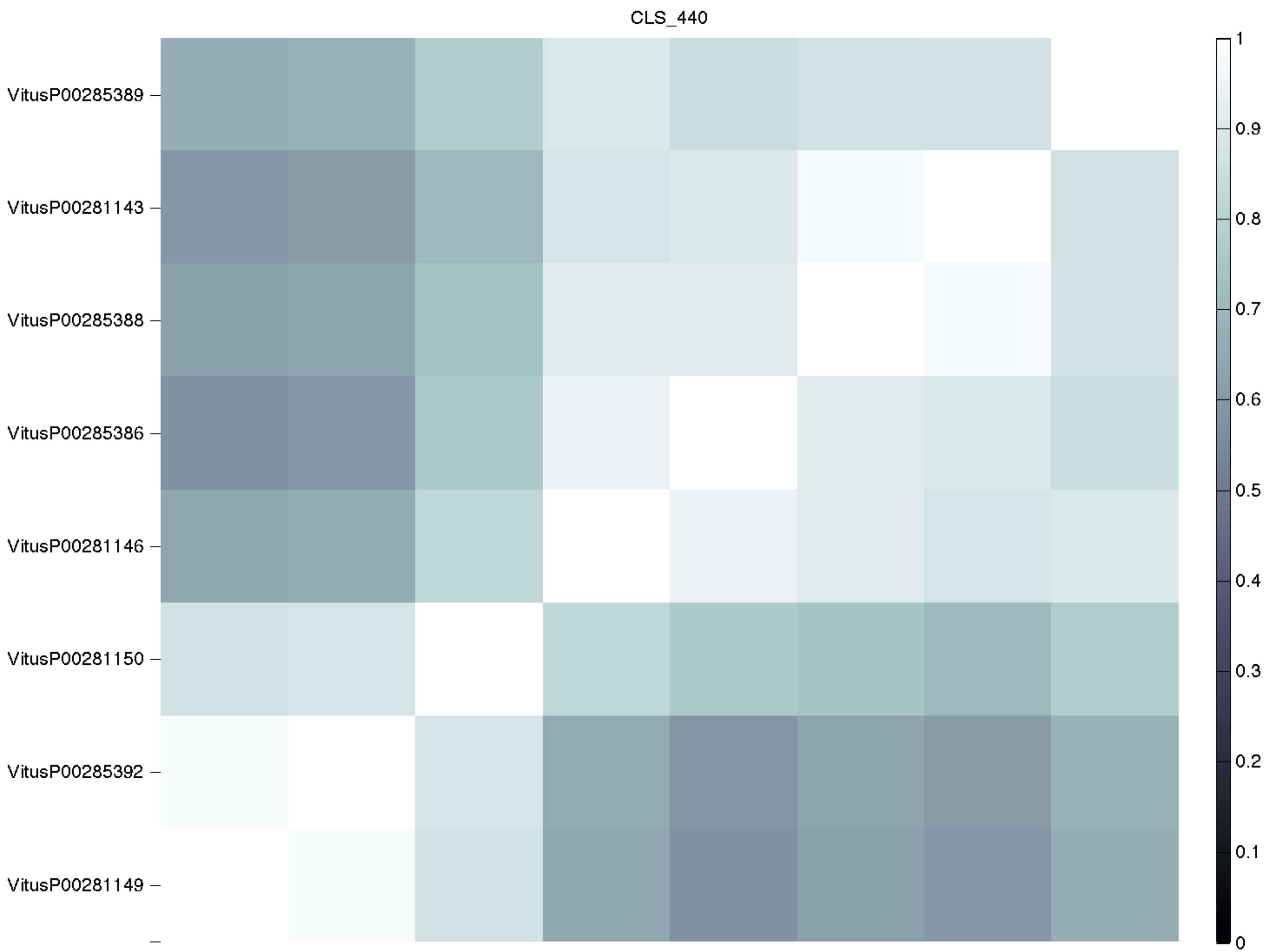

CLS_440
VitusP00285389
VitusP00281143
VitusP00285388
VitusP00285386
VitusP00281146
VitusP00281150
VitusP00285392
VitusP00281149
1
0.9
0.8
0.7
0.6
0.5
0.4
0.3
0.2
0.1
0

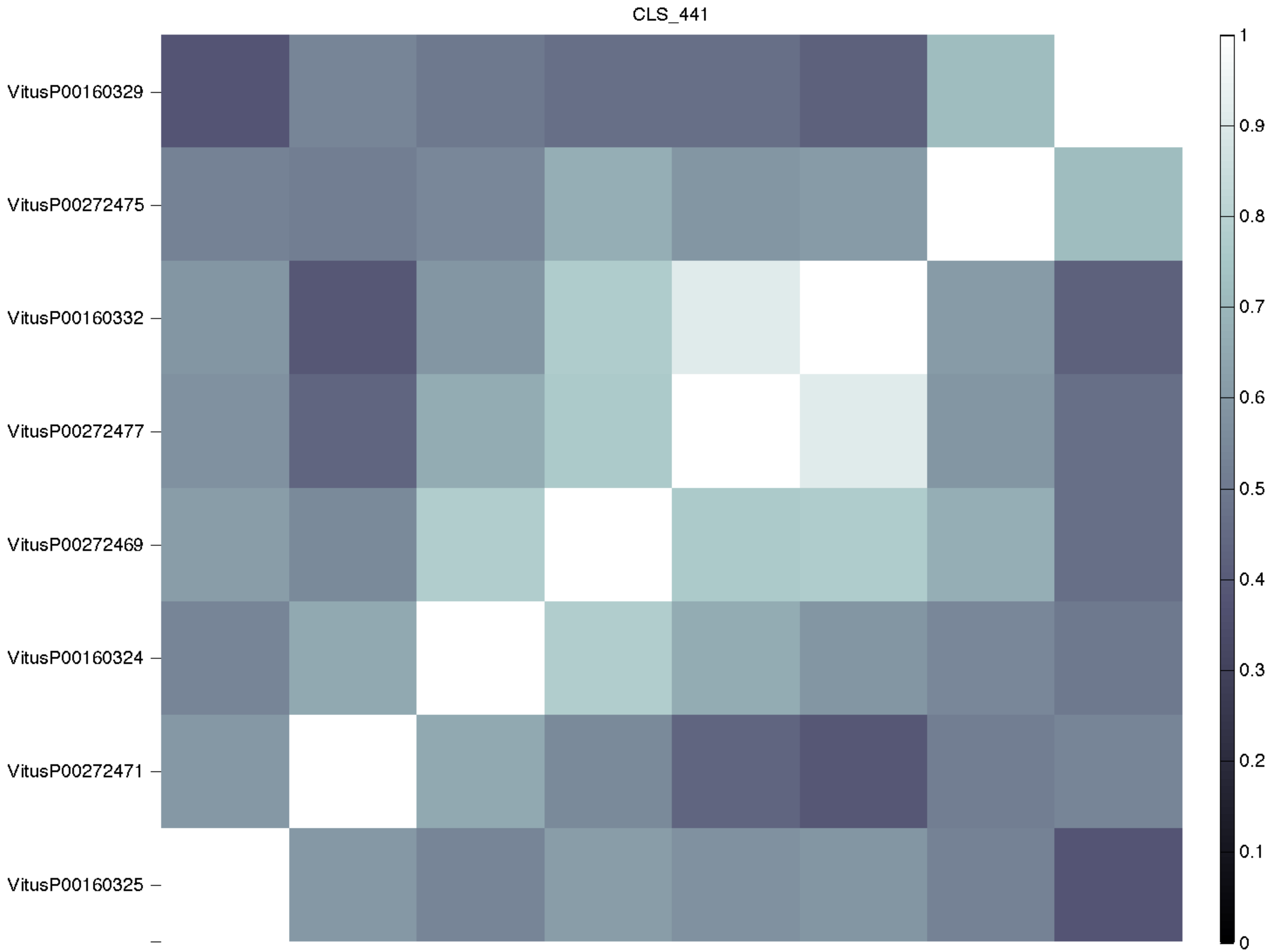
CLS_441
VitusP00160329
VitusP00272475
VitusP00160332
VitusP00272477
VitusP00272469
VitusP00160324
VitusP00272471
VitusP00160325
1
0.9
0.8
0.7
0.6
0.5
0.4
0.3
0.2
0.1
0

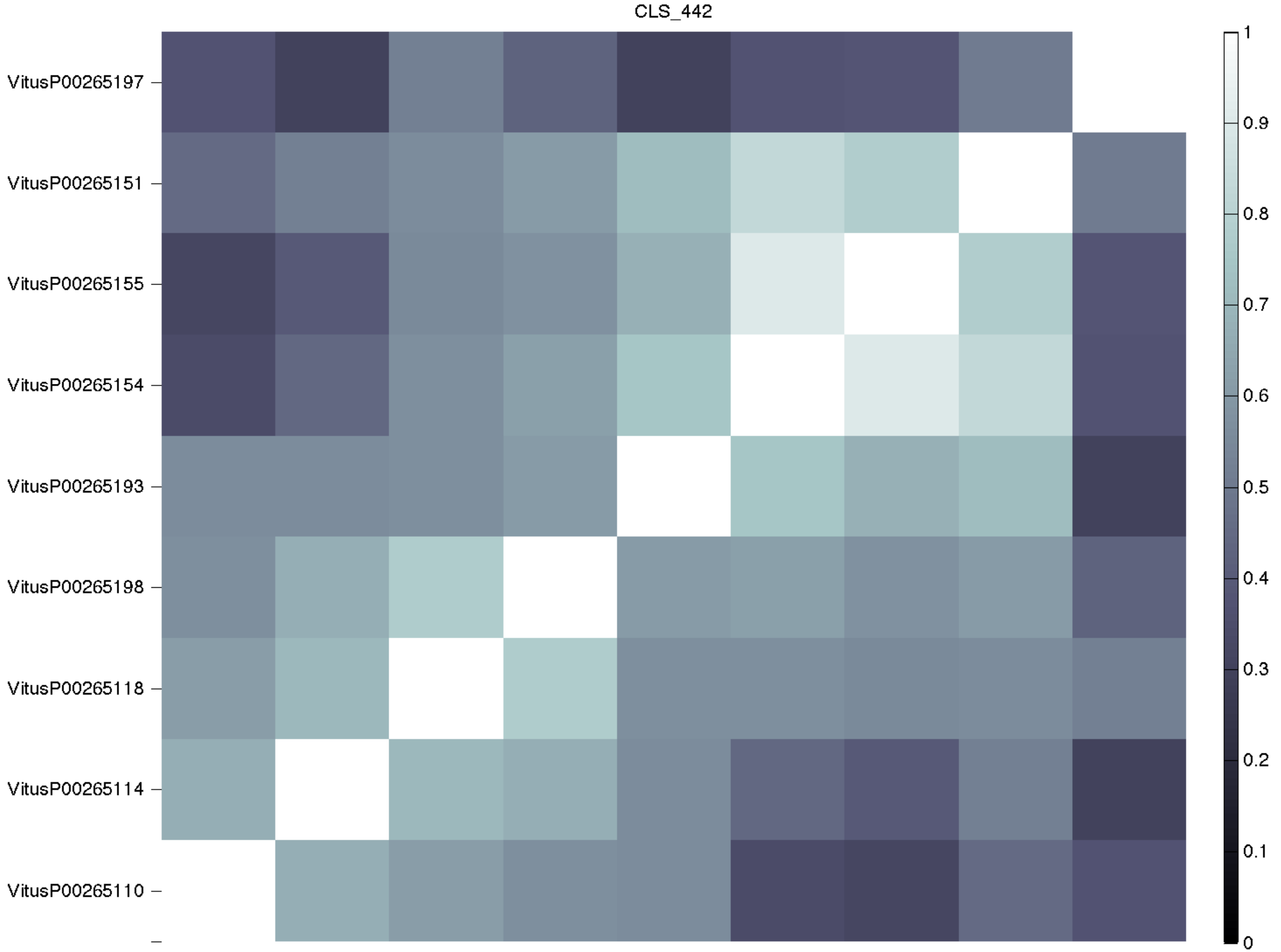
CLS_442
VitusP00265197
VitusP00265151
VitusP00265155
VitusP00265154
VitusP00265193
VitusP00265198
VitusP00265118
VitusP00265114
VitusP00265110
1
0.9
0.8
0.7
0.6
0.5
0.4
0.3
0.2
0.1
0

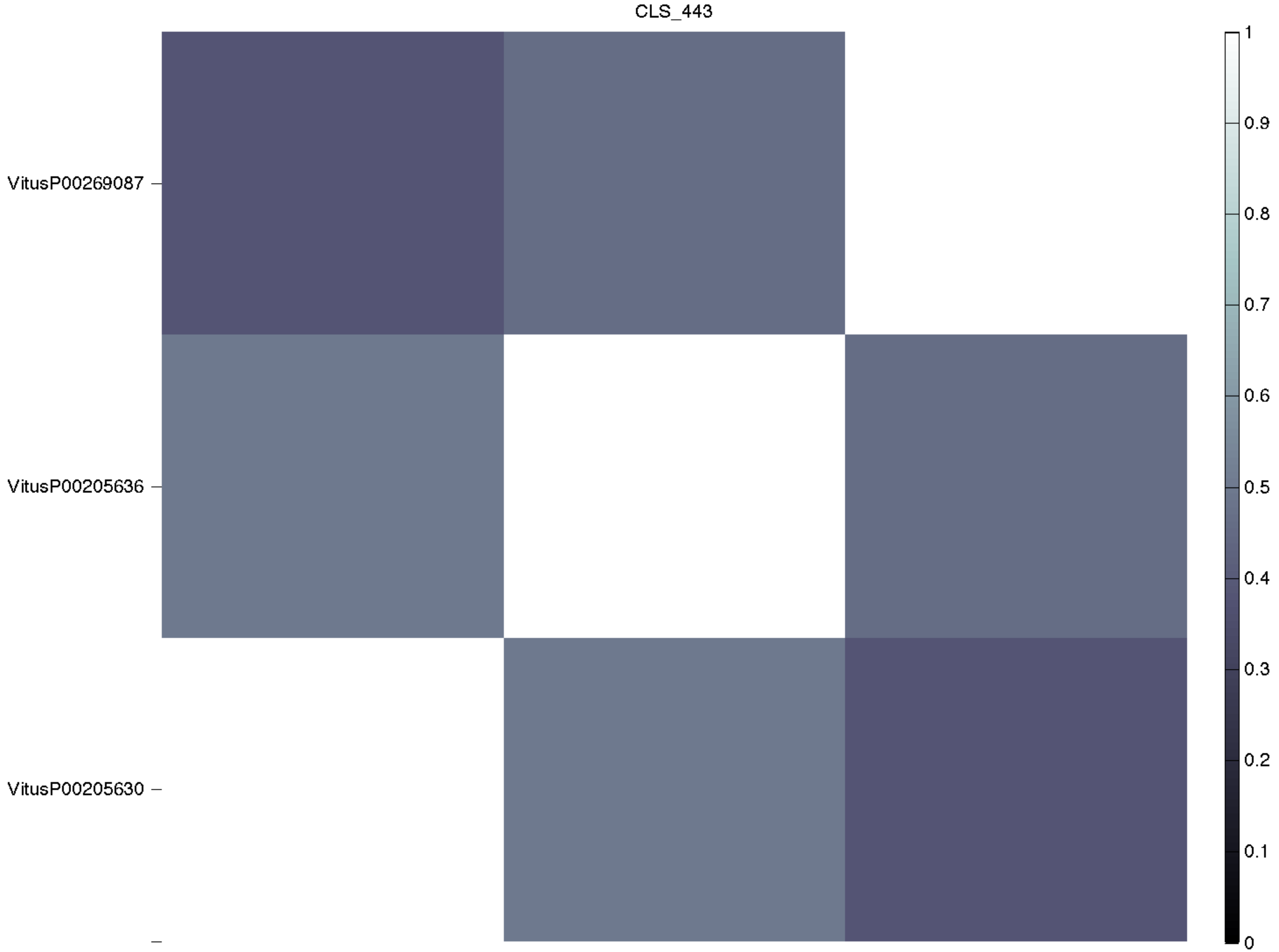
CLS_443
VitusP00269087
VitusP00205636
VitusP00205630
1
0.9
0.8
0.7
0.6
0.5
0.4
0.3
0.2
0.1
0

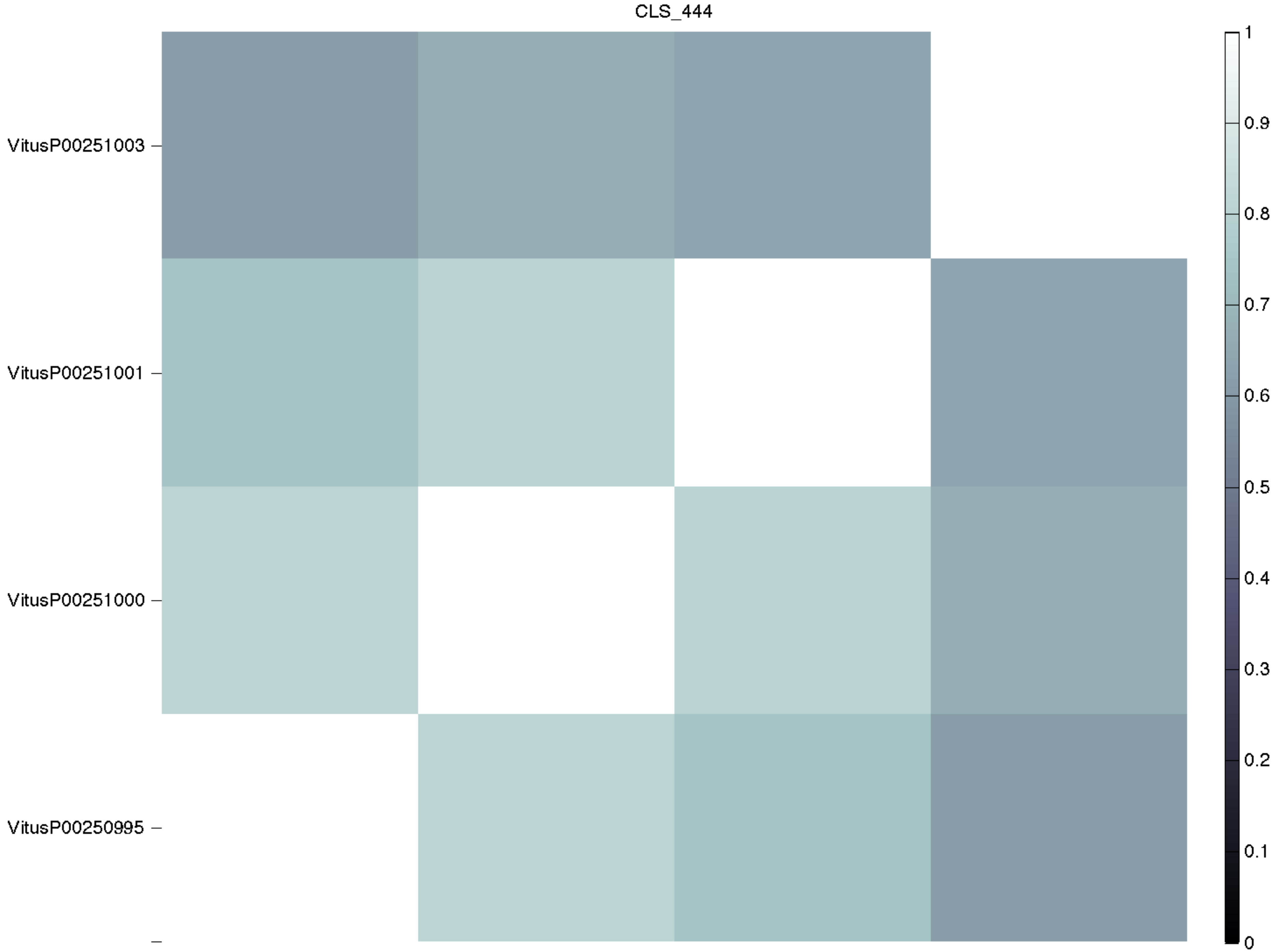
CLS_444
VitusP00251003
VitusP00251001
VitusP00251000
VitusP00250995
1
0.9
0.8
0.7
0.6
0.5
0.4
0.3
0.2
0.1
0

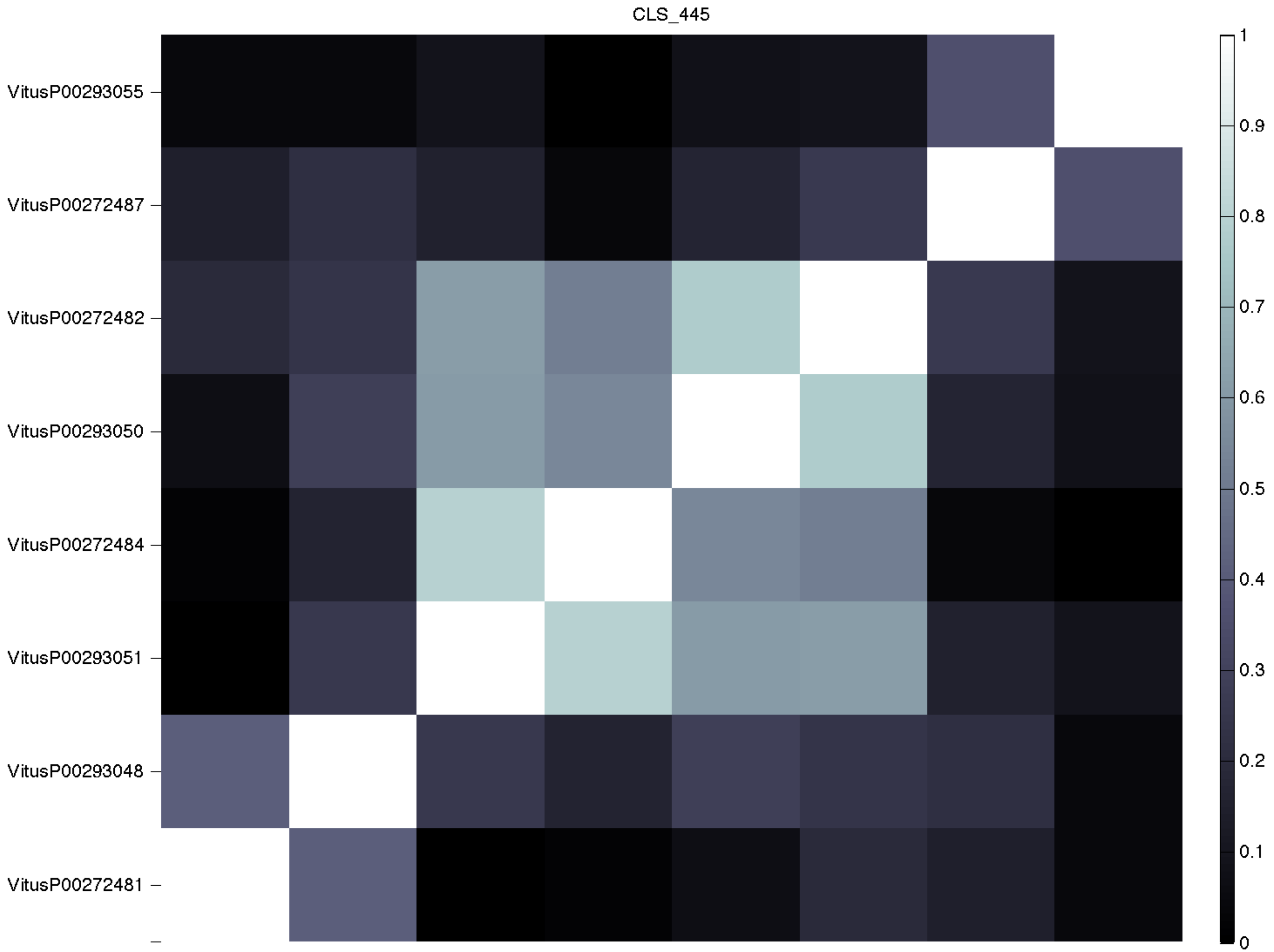

CLS_445
VitusP00293055
VitusP00272487
VitusP00272482
VitusP00293050
VitusP00272484
VitusP00293051
VitusP00293048
VitusP00272481
1
0.9
0.8
0.7
0.6
0.5
0.4
0.3
0.2
0.1
0

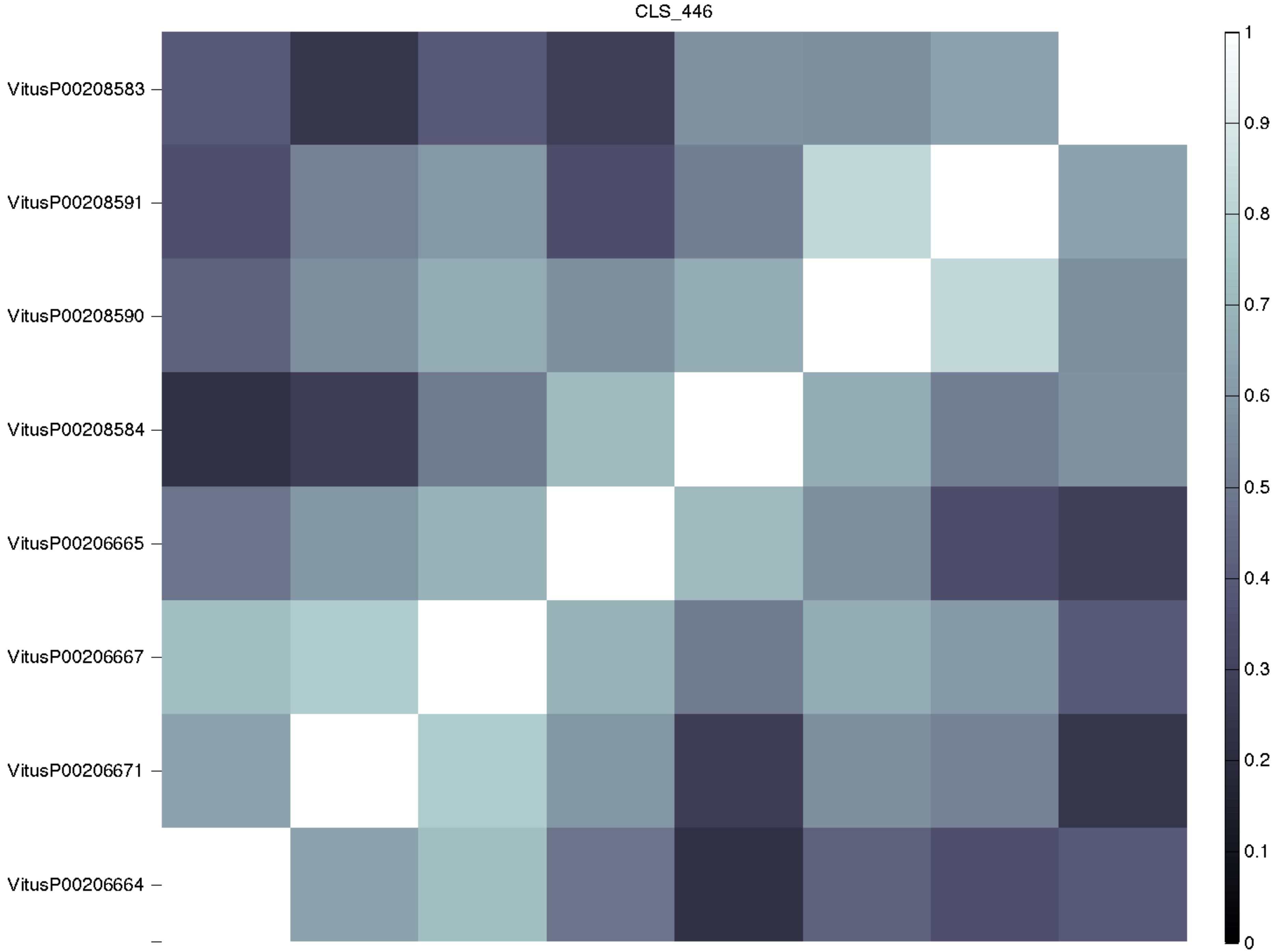

CLS_446
VitusP00208583
VitusP00208591
VitusP00208590
VitusP00208584
VitusP00206665
VitusP00206667
VitusP00206671
VitusP00206664
1
0.9
0.8
0.7
0.6
0.5
0.4
0.3
0.2
0.1
0

CLS_447

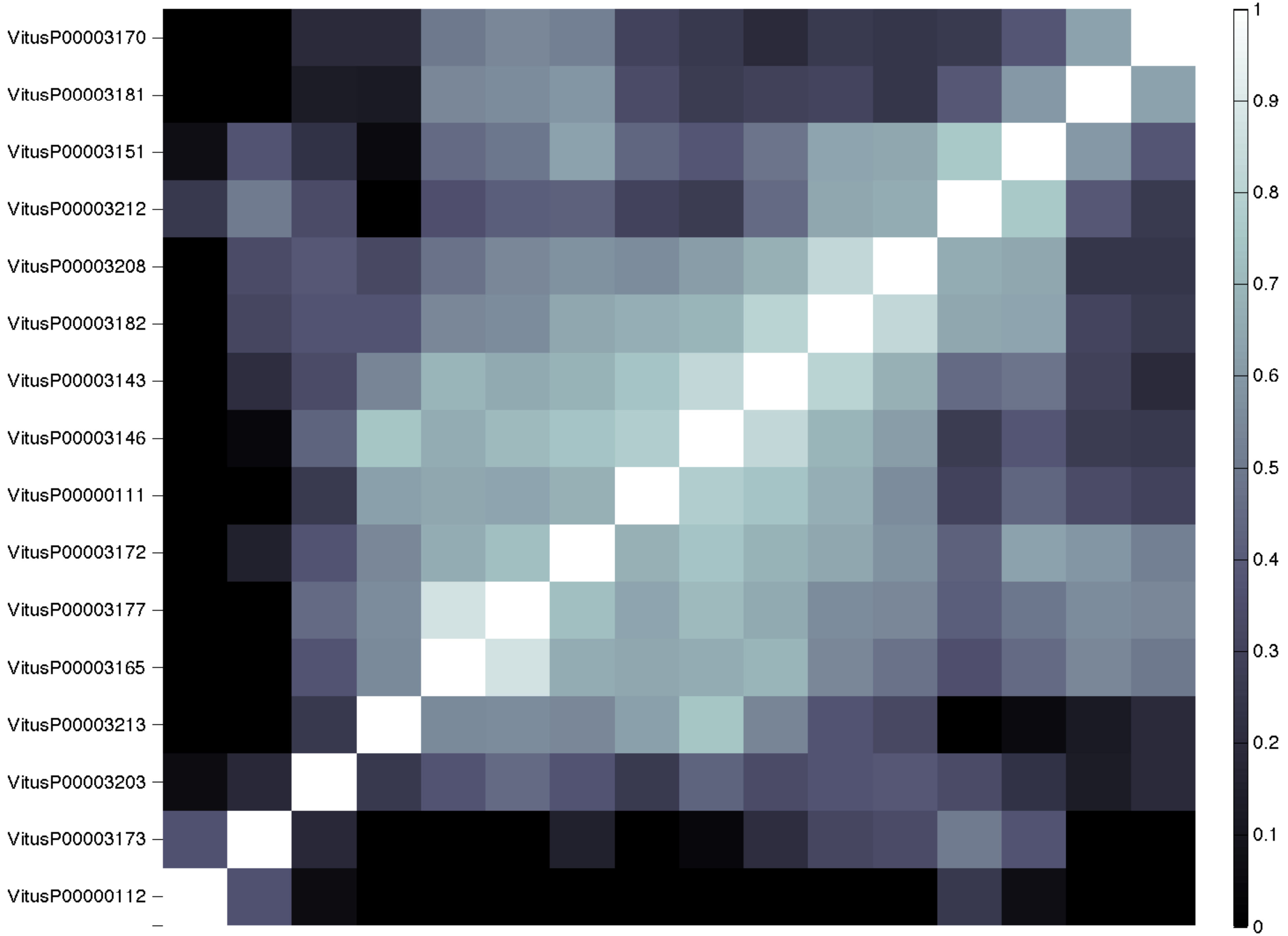

VitusP00003170
VitusP00003181
VitusP00003151
VitusP00003212
VitusP00003208
VitusP00003182
VitusP00003143
VitusP00003146
VitusP00000111
VitusP00003172
VitusP00003177
VitusP00003165
VitusP00003213
VitusP00003203
VitusP00003173
VitusP00000112
1
0.9
0.8
0.7
0.6
0.5
0.4
0.3
0.2
0.1
0

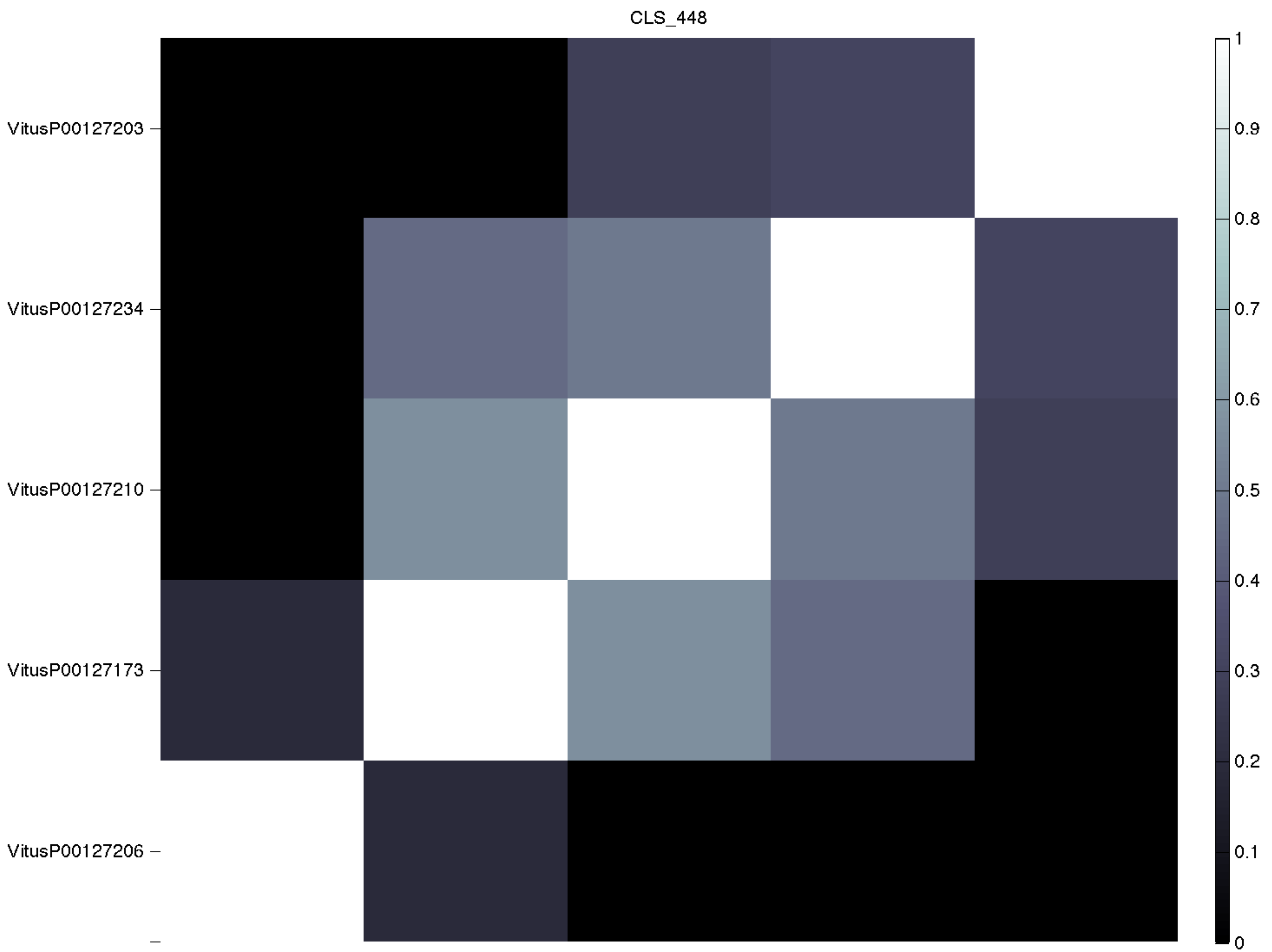
CLS_448
VitusP00127203
VitusP00127234
VitusP00127210
VitusP00127173
VitusP00127206
1
0.9
0.8
0.7
0.6
0.5
0.4
0.3
0.2
0.1
0

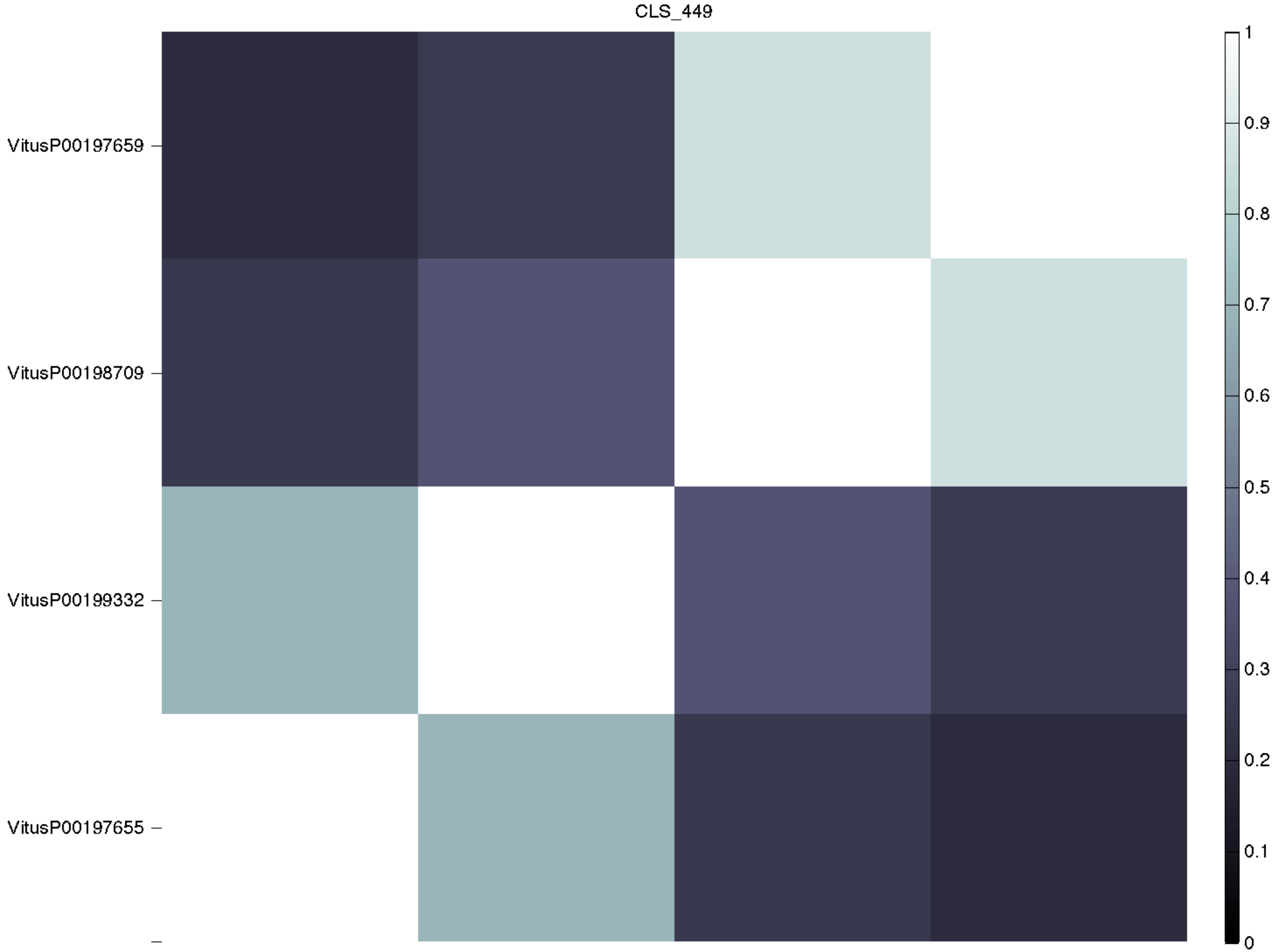

CLS_449
VitusP00197659
VitusP00198709
VitusP00199332
VitusP00197655
1
0.9
0.8
0.7
0.6
0.5
0.4
0.3
0.2
0.1
0

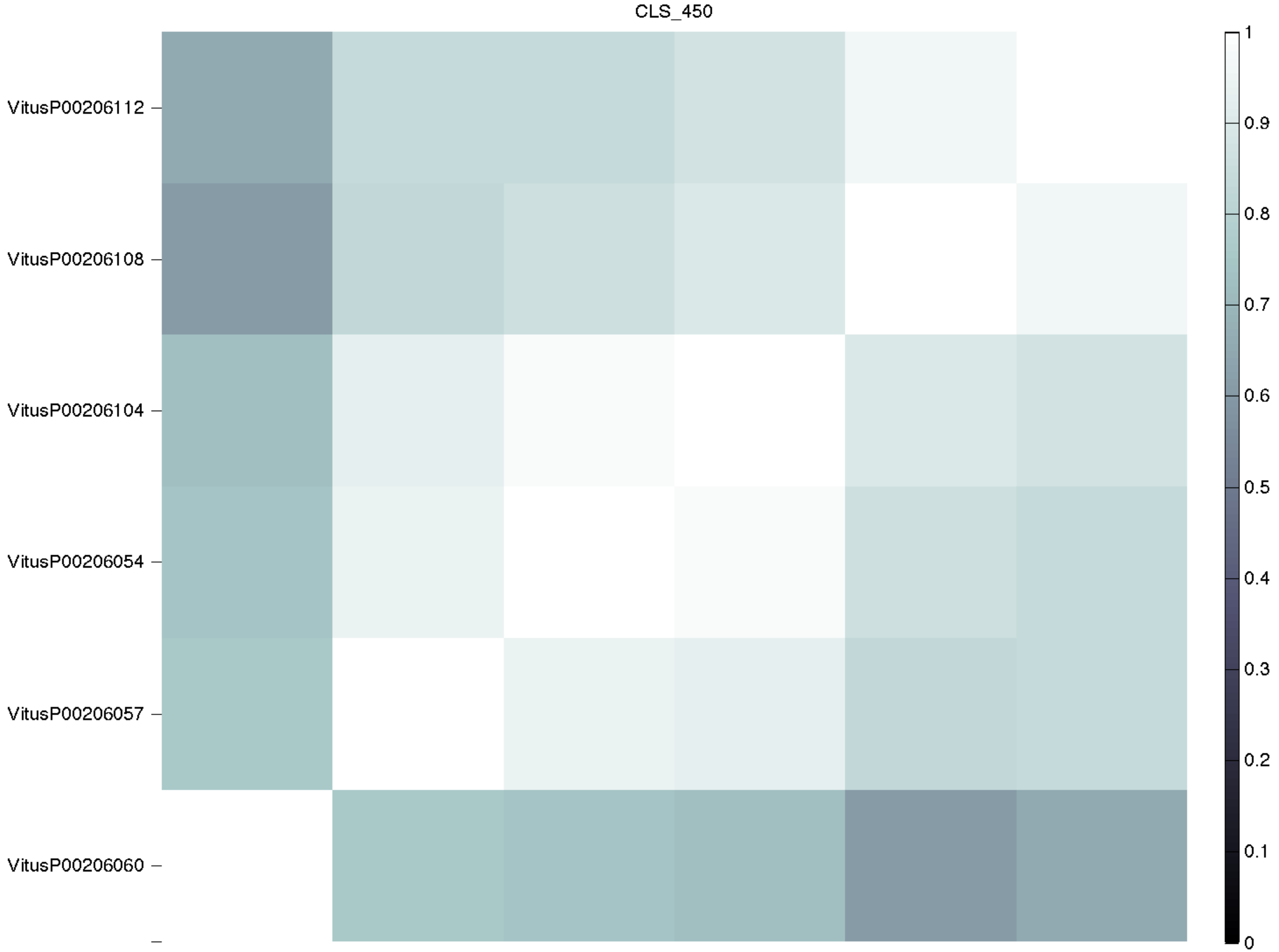
CLS_450
VitusP00206112
VitusP00206108
VitusP00206104
VitusP00206054
VitusP00206057
VitusP00206060
1
0.9
0.8
0.7
0.6
0.5
0.4
0.3
0.2
0.1
0

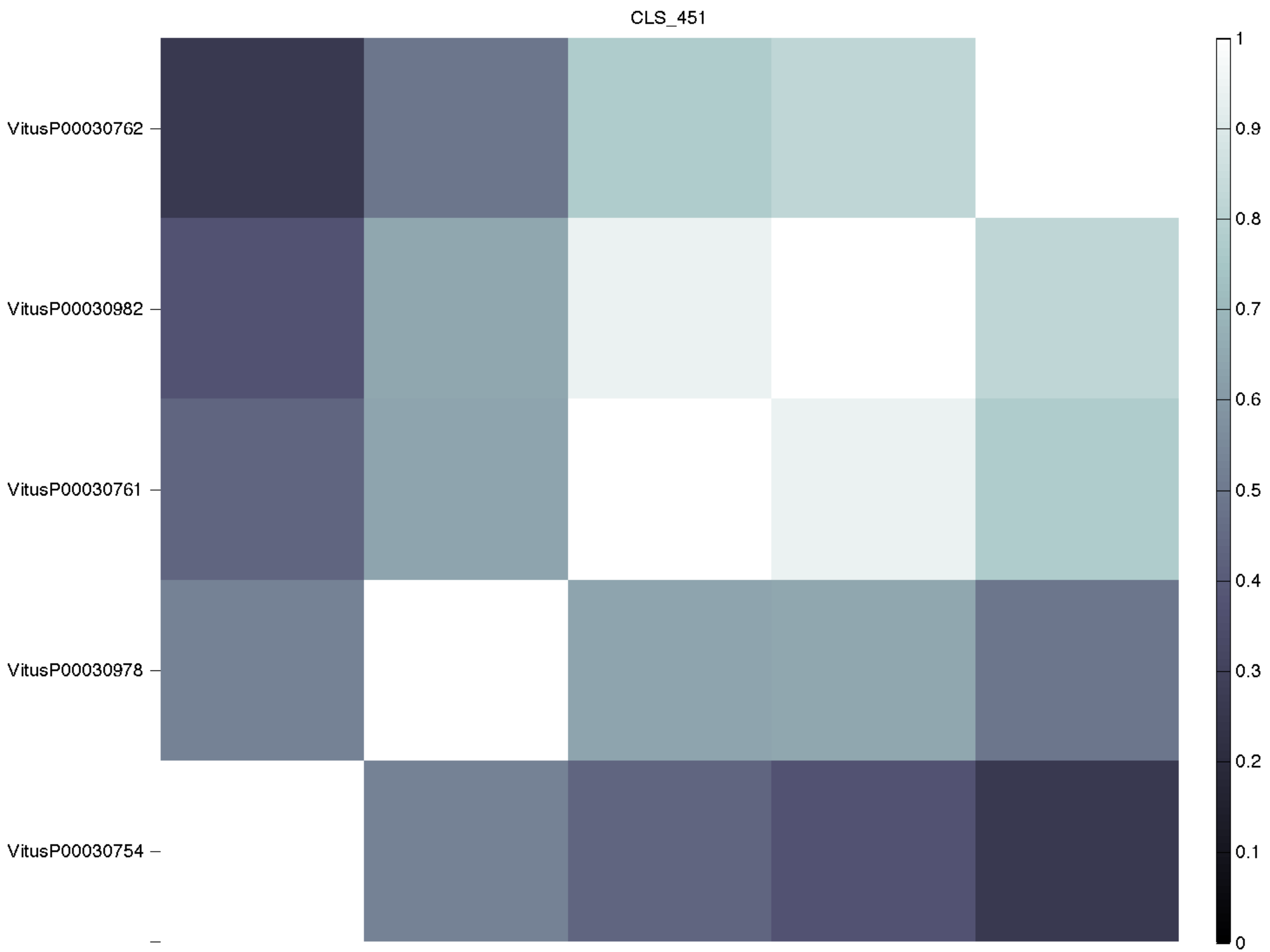

CLS_451
VitusP00030762
VitusP00030982
VitusP00030761
VitusP00030978
VitusP00030754
1
0.9
0.8
0.7
0.6
0.5
0.4
0.3
0.2
0.1
0

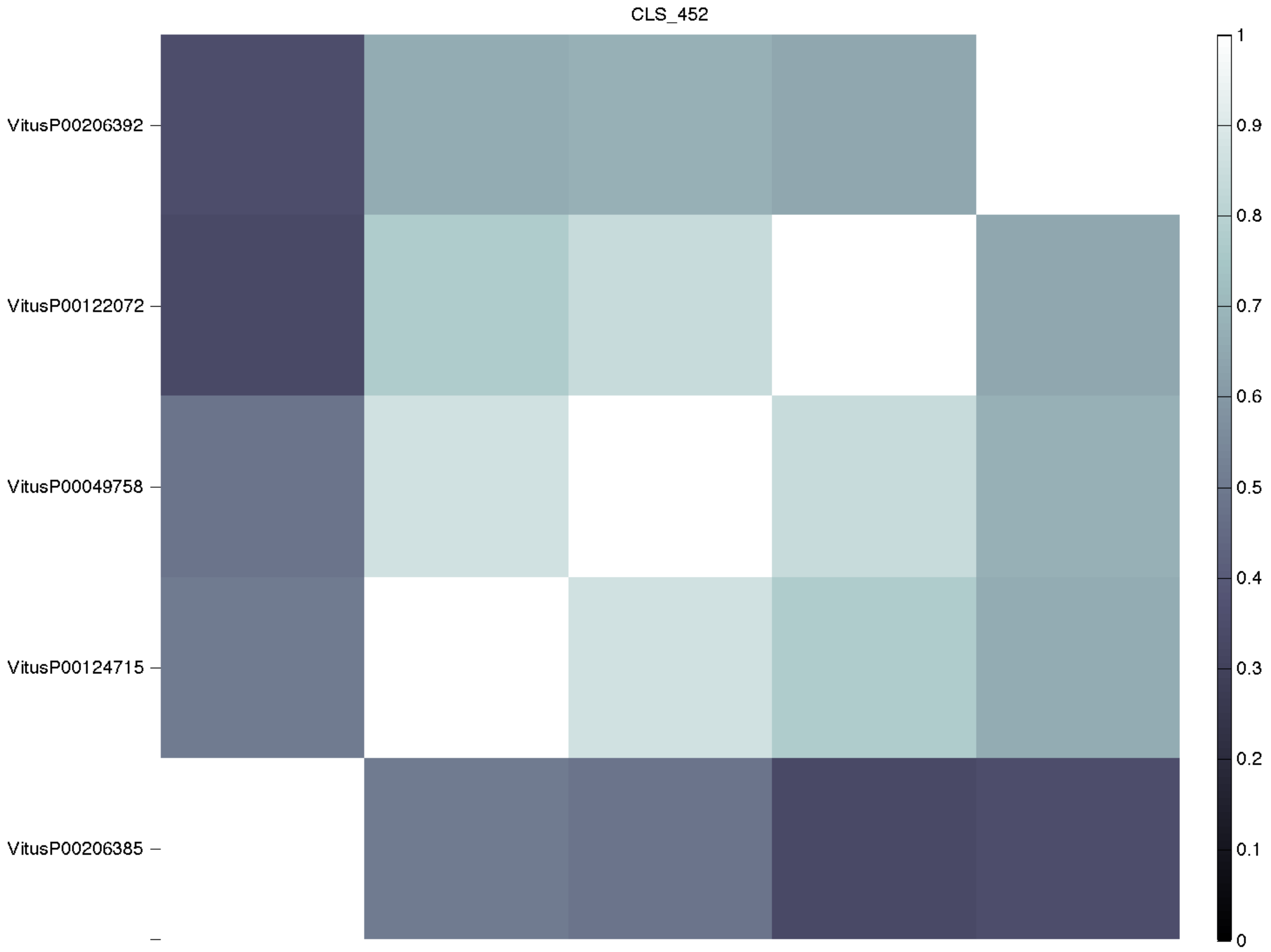
CLS_452
VitusP00206392
VitusP00122072
VitusP00049758
VitusP00124715
VitusP00206385
1
0.9
0.8
0.7
0.6
0.5
0.4
0.3
0.2
0.1
0

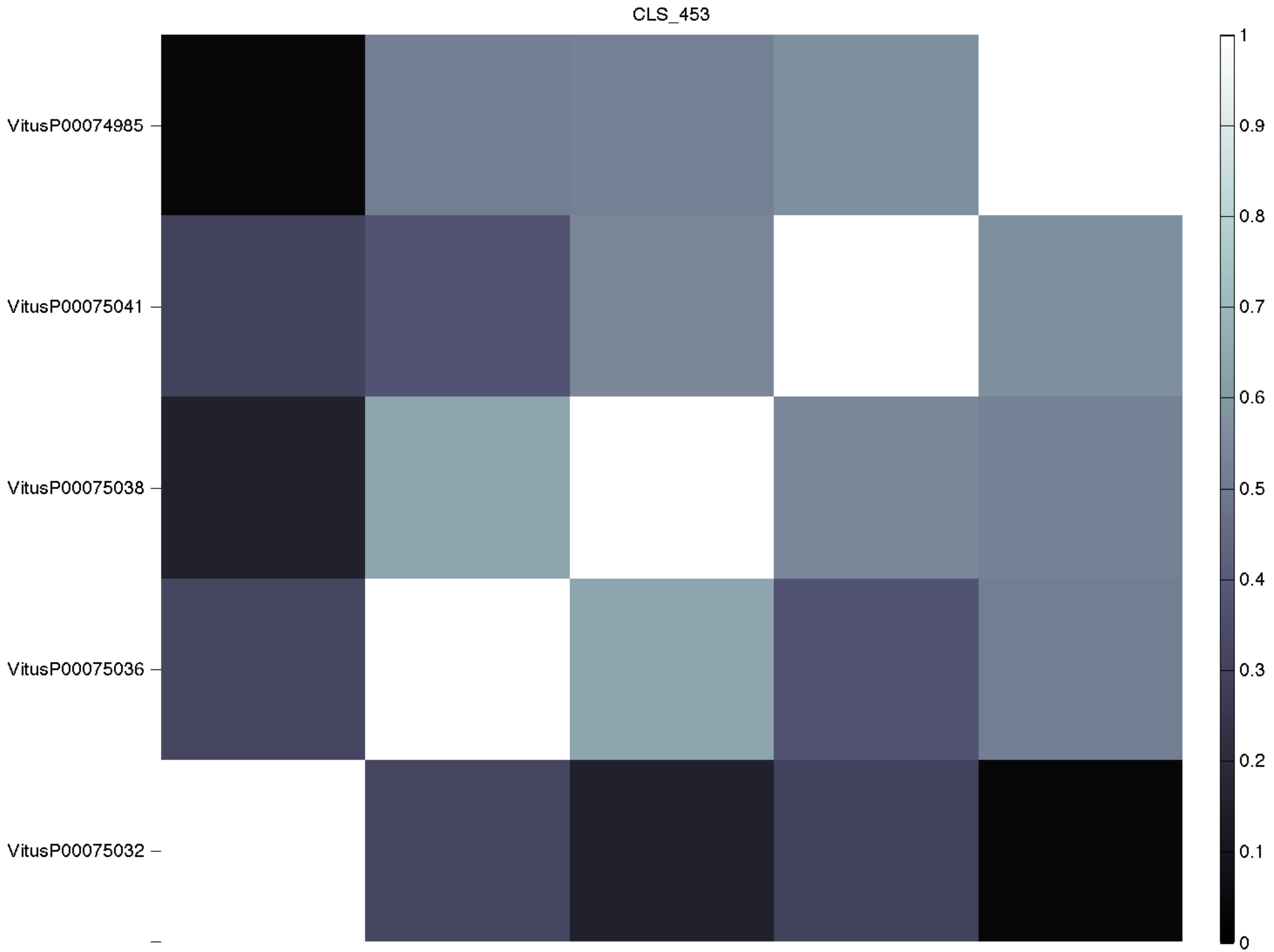
CLS_453
VitusP00074985
VitusP00075041
VitusP00075038
VitusP00075036
VitusP00075032
1
0.9
0.8
0.7
0.6
0.5
0.4
0.3
0.2
0.1
0

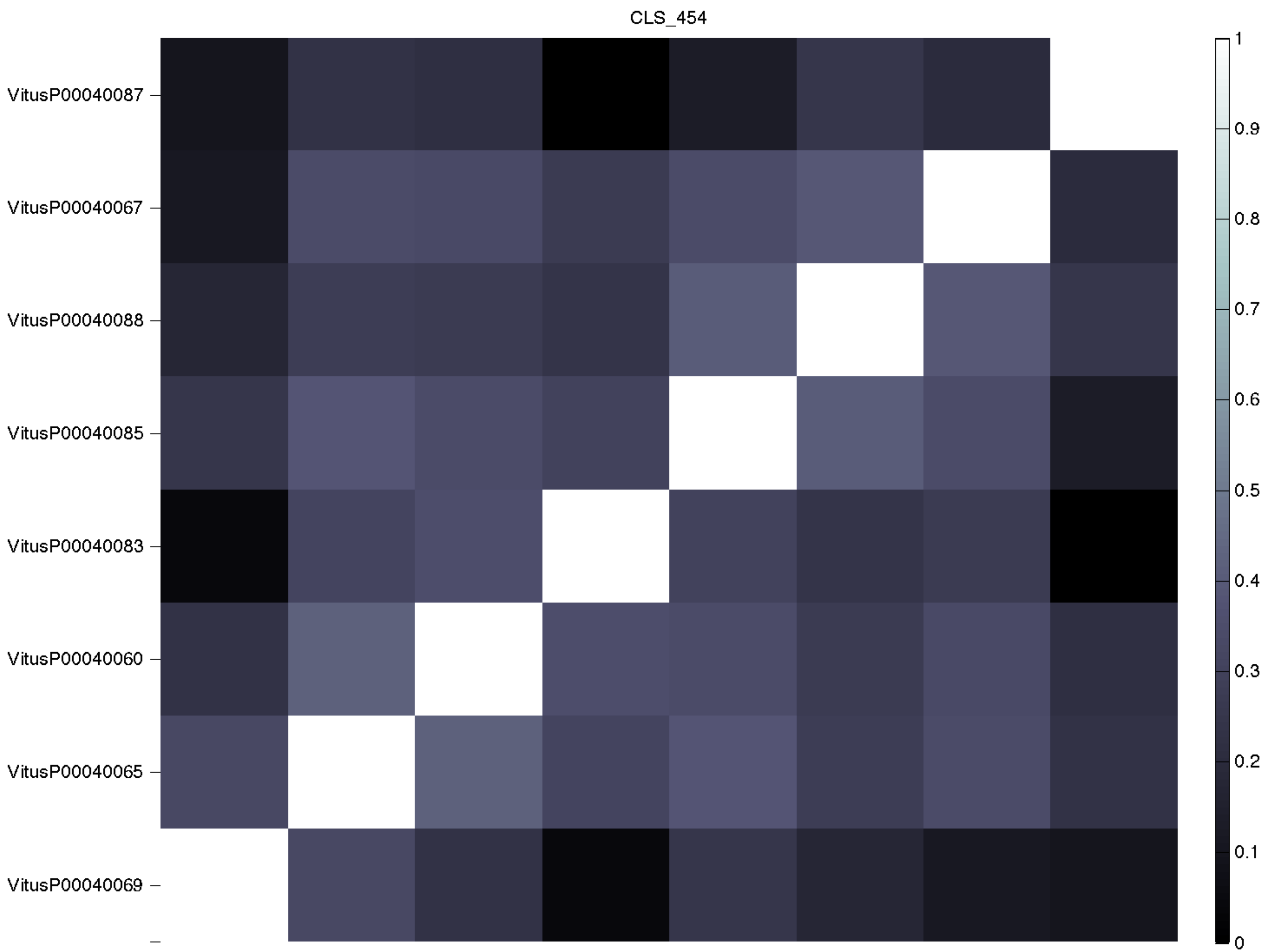

CLS_454
VitusP00040087
VitusP00040067
VitusP00040088
VitusP00040085
VitusP00040083
VitusP00040060
VitusP00040065
VitusP00040069
1
0.9
0.8
0.7
0.6
0.5
0.4
0.3
0.2
0.1
0

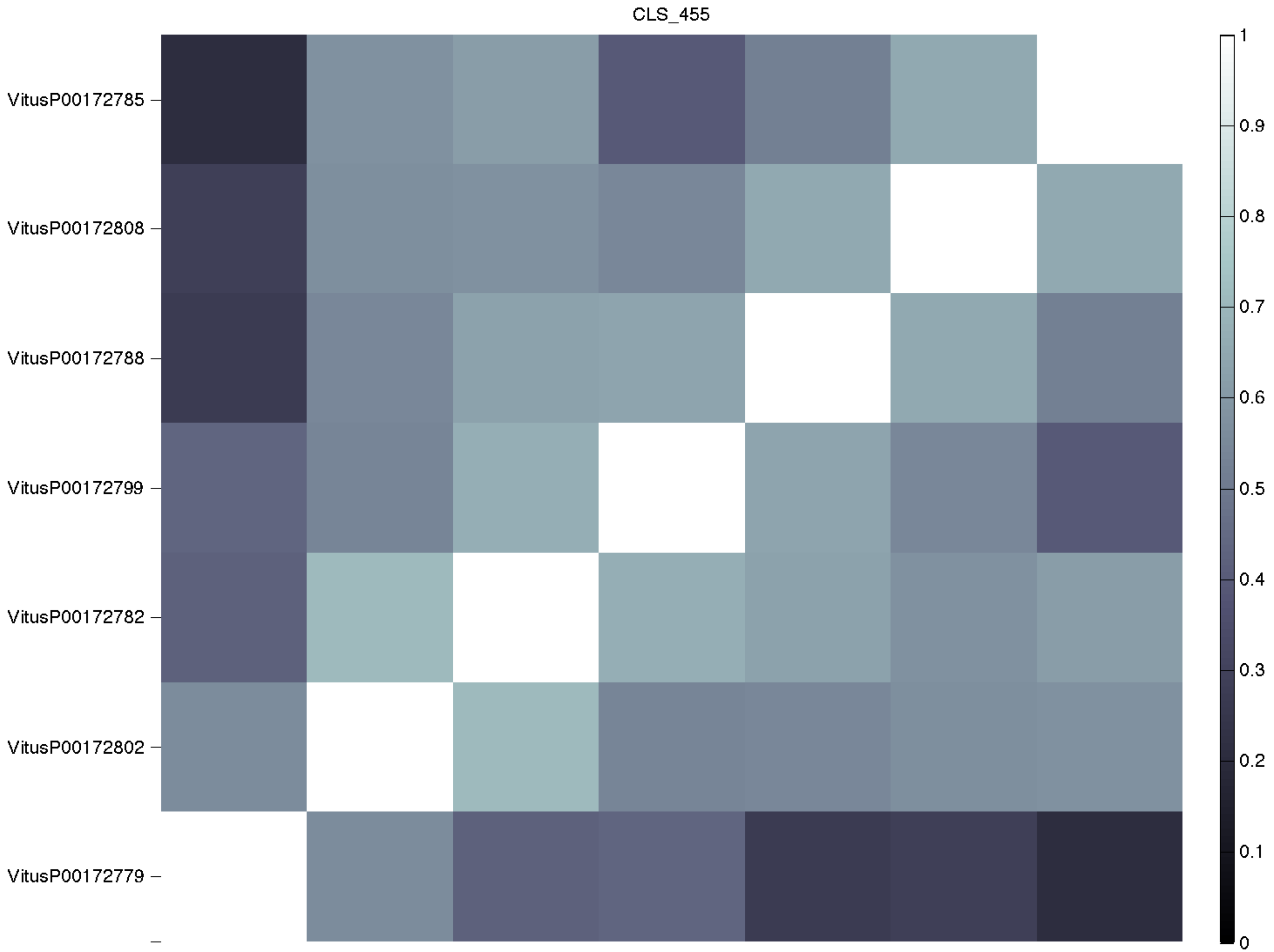
CLS_455
VitusP00172785
VitusP00172808
VitusP00172788
VitusP00172799
VitusP00172782
VitusP00172802
VitusP00172779
1
0.9
0.8
0.7
0.6
0.5
0.4
0.3
0.2
0.1
0

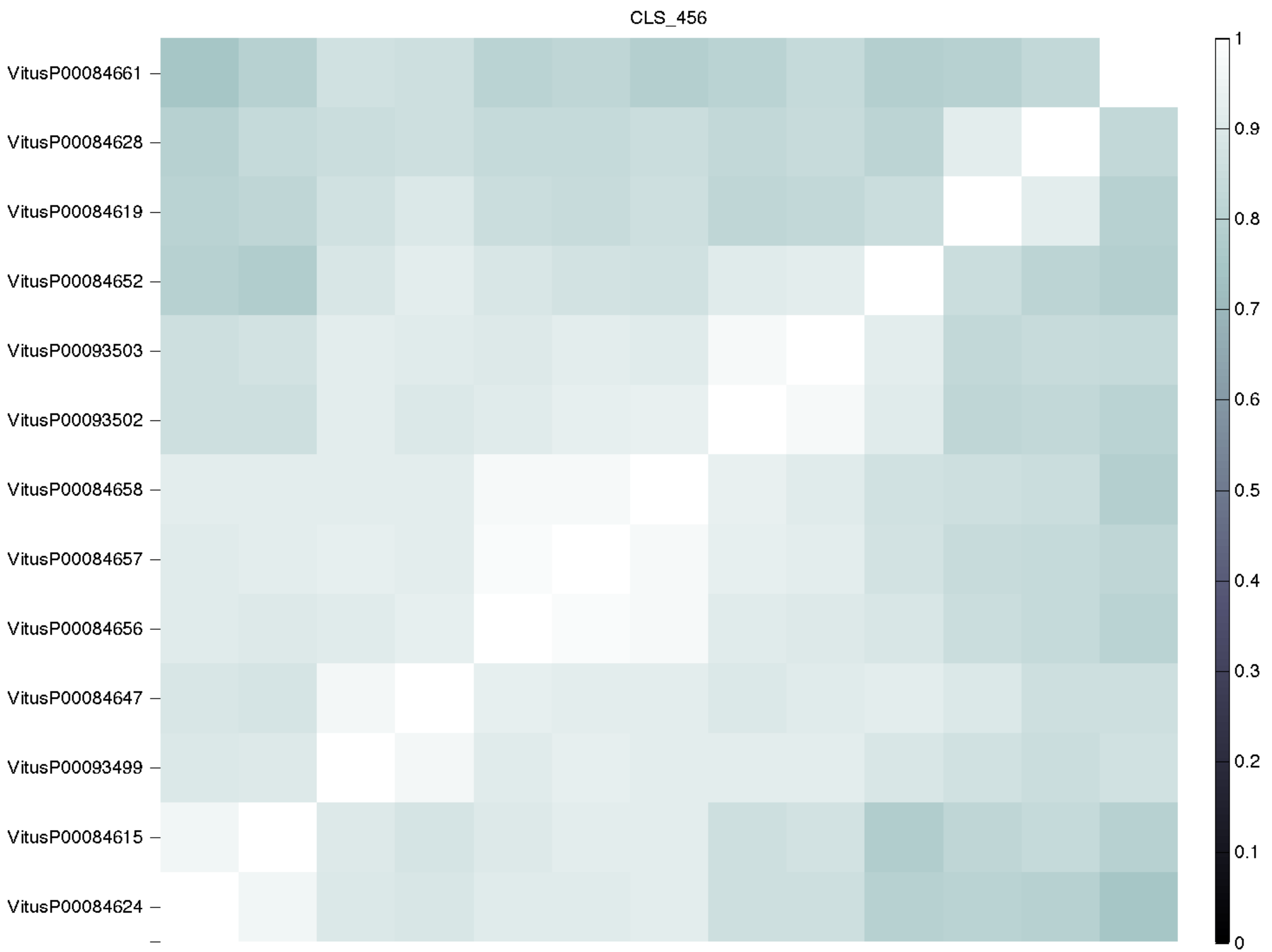
CLS_456
VitusP00084661
VitusP00084628
VitusP00084619
VitusP00084652
VitusP00093503
VitusP00093502
VitusP00084658
VitusP00084657
VitusP00084656
VitusP00084647
VitusP00093499
VitusP00084615
VitusP00084624
1
0.9
0.8
0.7
0.6
0.5
0.4
0.3
0.2
0.1
0

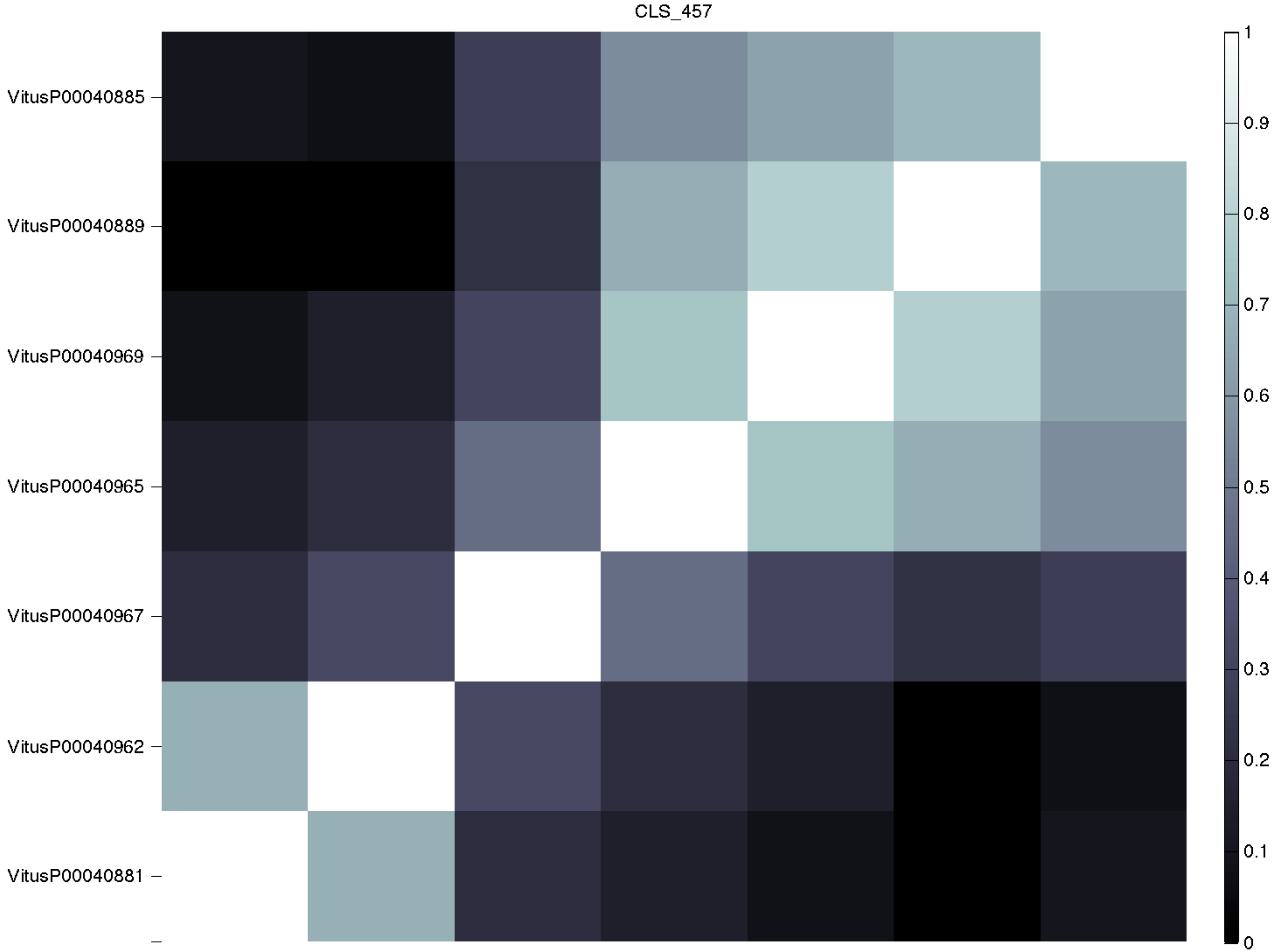
CLS_457
VitusP00040885
VitusP00040889
VitusP00040969
VitusP00040965
VitusP00040967
VitusP00040962
VitusP00040881
1
0.9
0.8
0.7
0.6
0.5
0.4
0.3
0.2
0.1
0

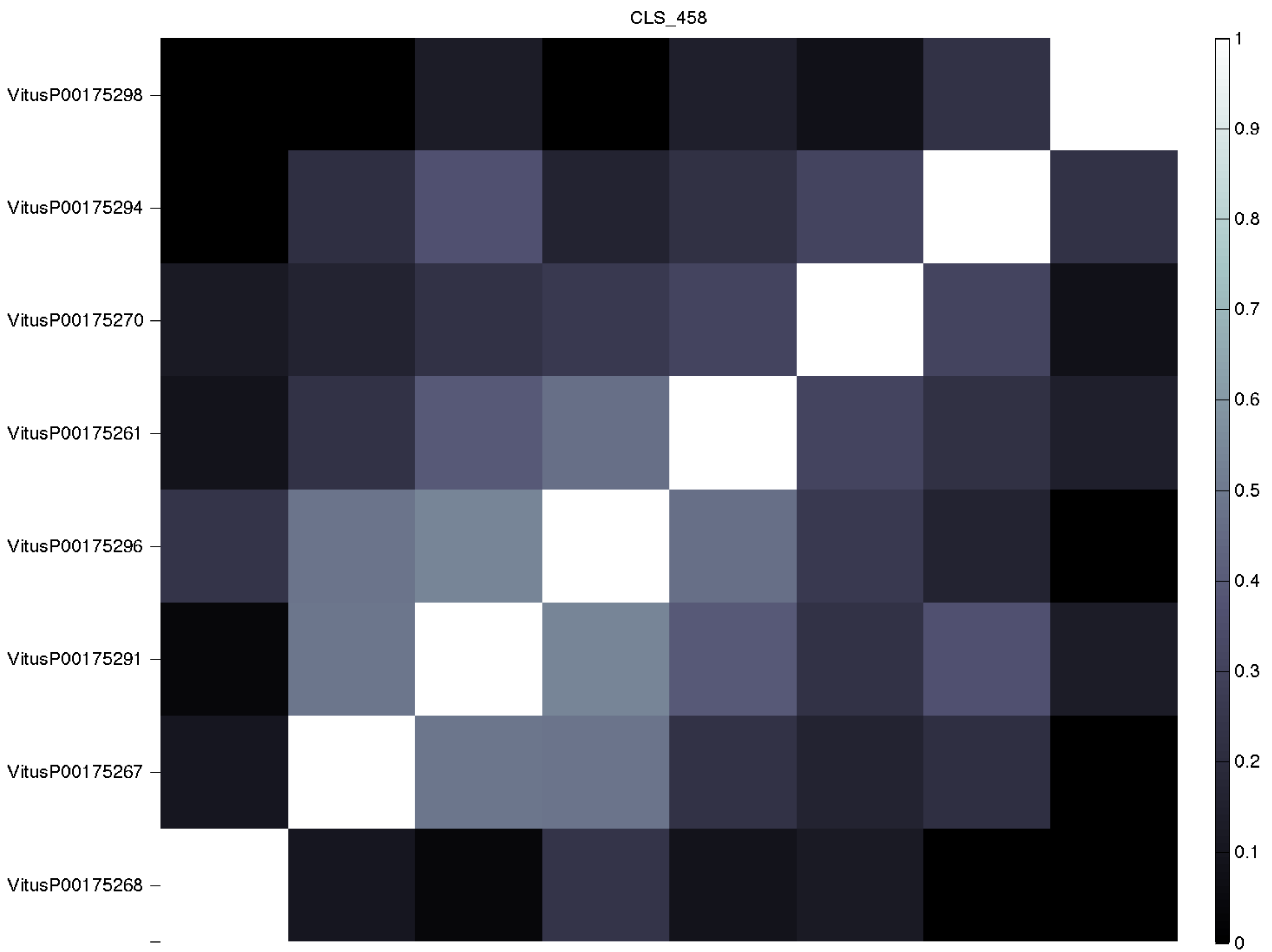

CLS_458
VitusP00175298
VitusP00175294
VitusP00175270
VitusP00175261
VitusP00175296
VitusP00175291
VitusP00175267
VitusP00175268
1
0.9
0.8
0.7
0.6
0.5
0.4
0.3
0.2
0.1
0

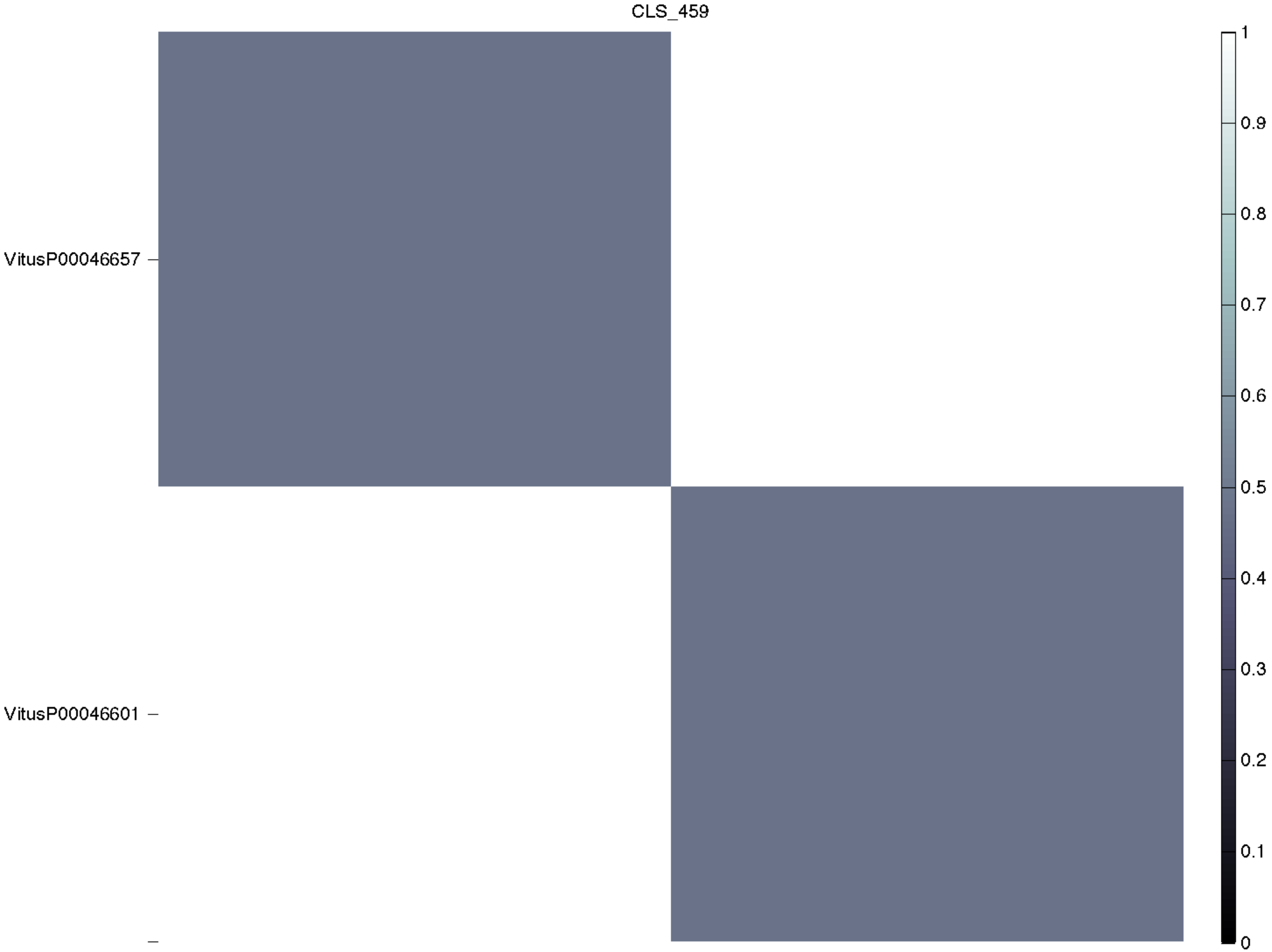
CLS_459
VitusP00046657
VitusP00046601
1
0.9
0.8
0.7
0.6
0.5
0.4
0.3
0.2
0.1
0

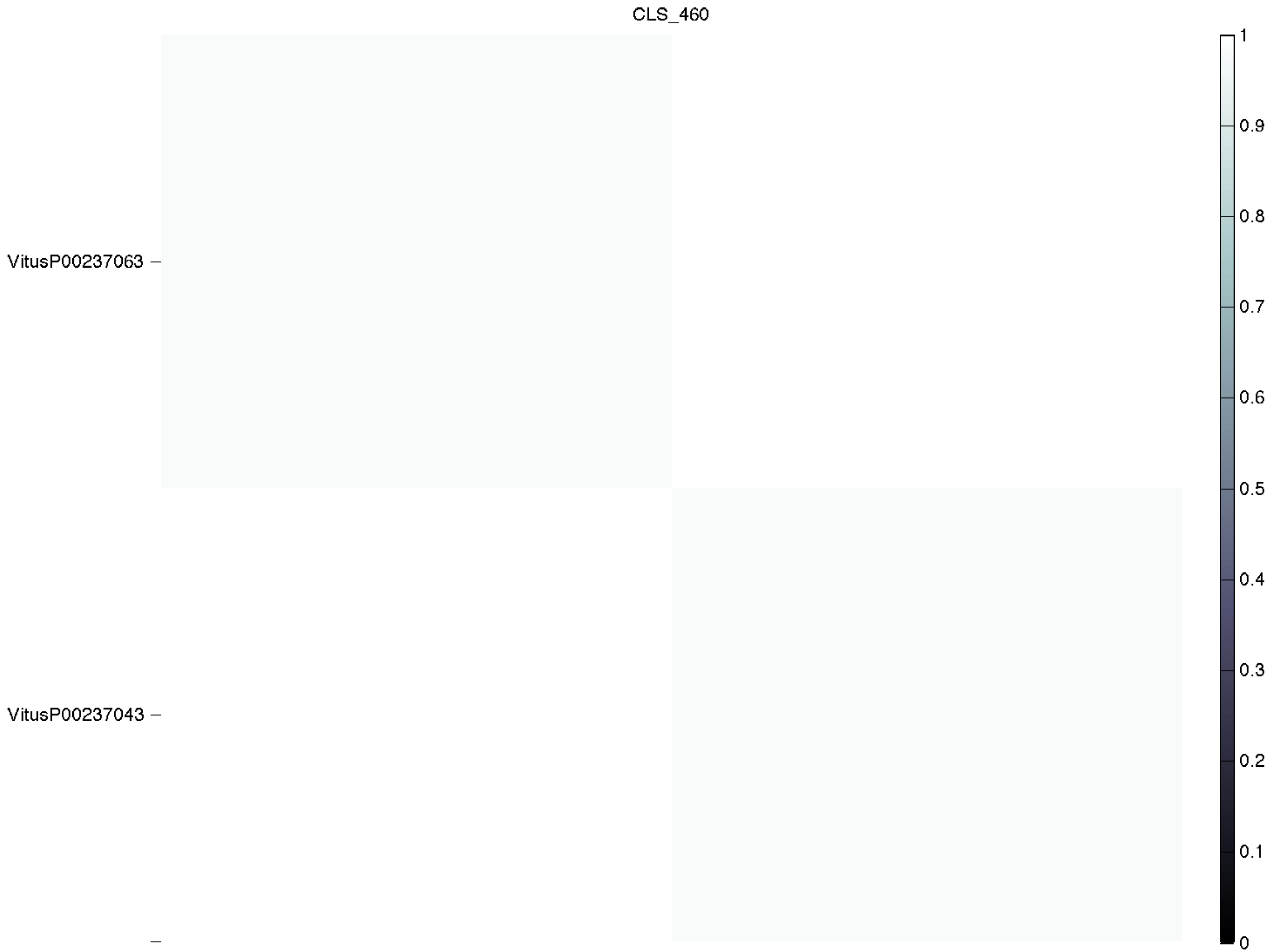
CLS_460
VitusP00237063
VitusP00237043
1
0.9
0.8
0.7
0.6
0.5
0.4
0.3
0.2
0.1
0

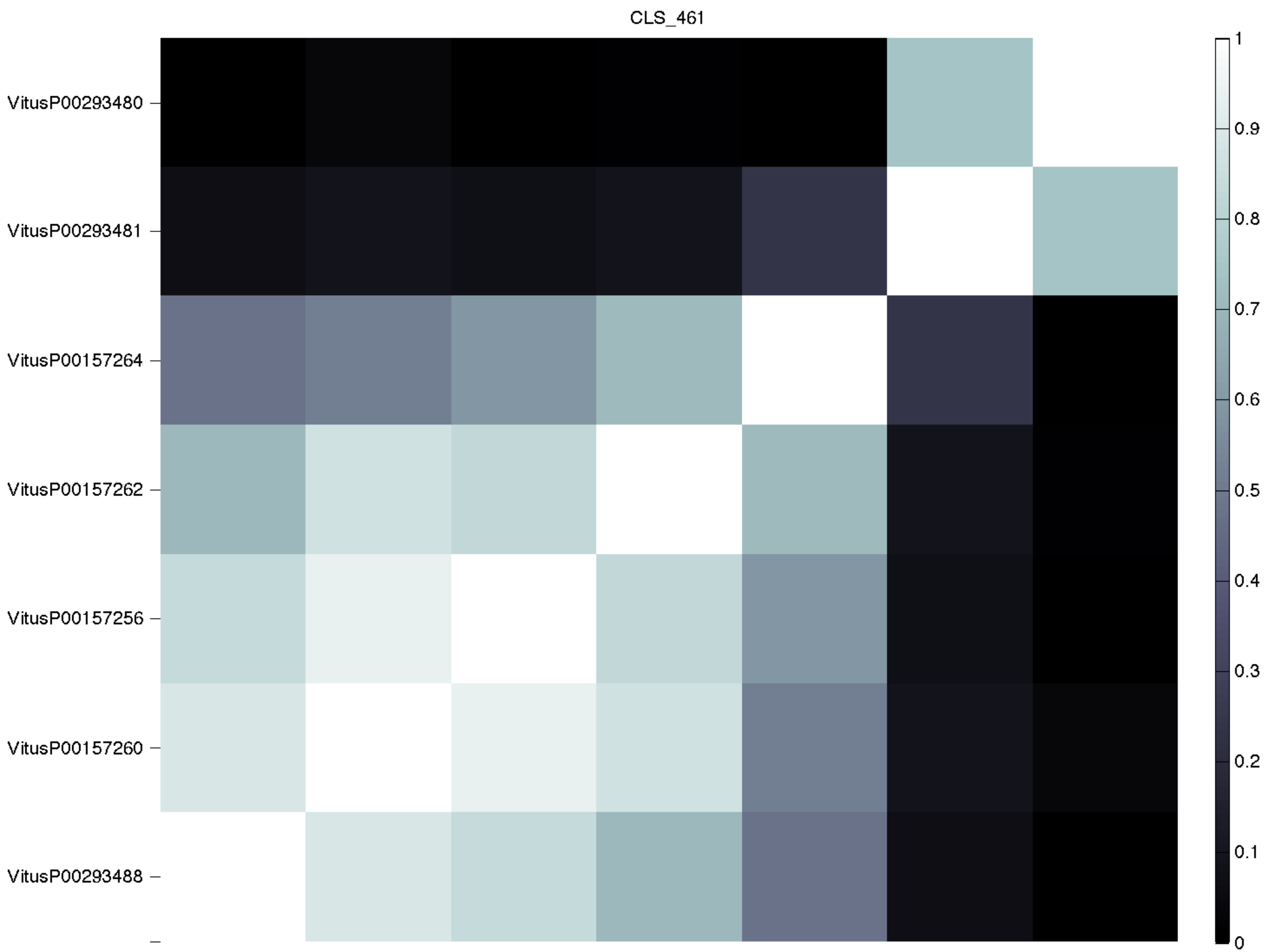
CLS_461
VitusP00293480
VitusP00293481
VitusP00157264
VitusP00157262
VitusP00157256
VitusP00157260
VitusP00293488
1
0.9
0.8
0.7
0.6
0.5
0.4
0.3
0.2
0.1
0

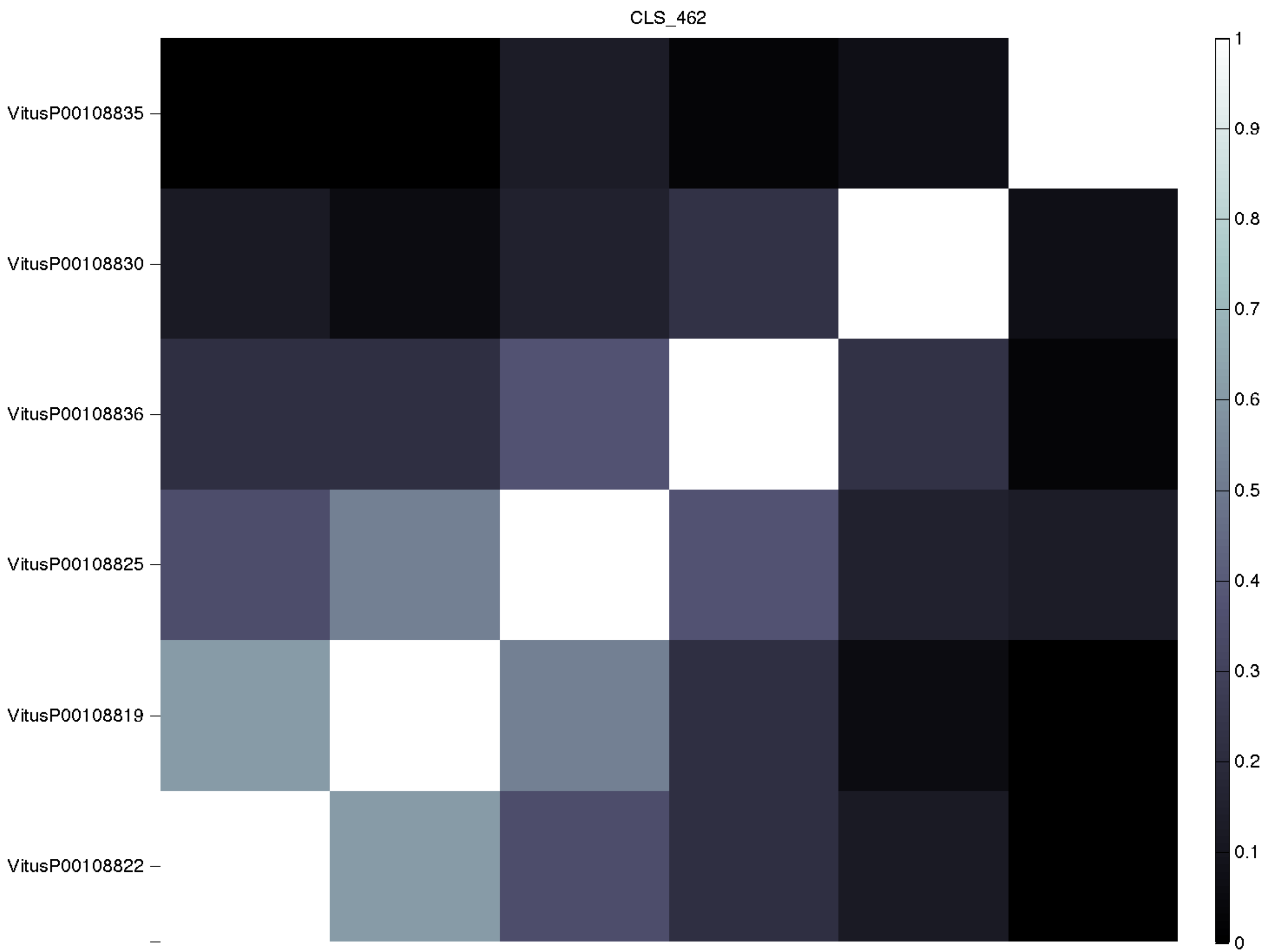
CLS_462
VitusP00108835
VitusP00108830
VitusP00108836
VitusP00108825
VitusP00108819
VitusP00108822
1
0.9
0.8
0.7
0.6
0.5
0.4
0.3
0.2
0.1
0

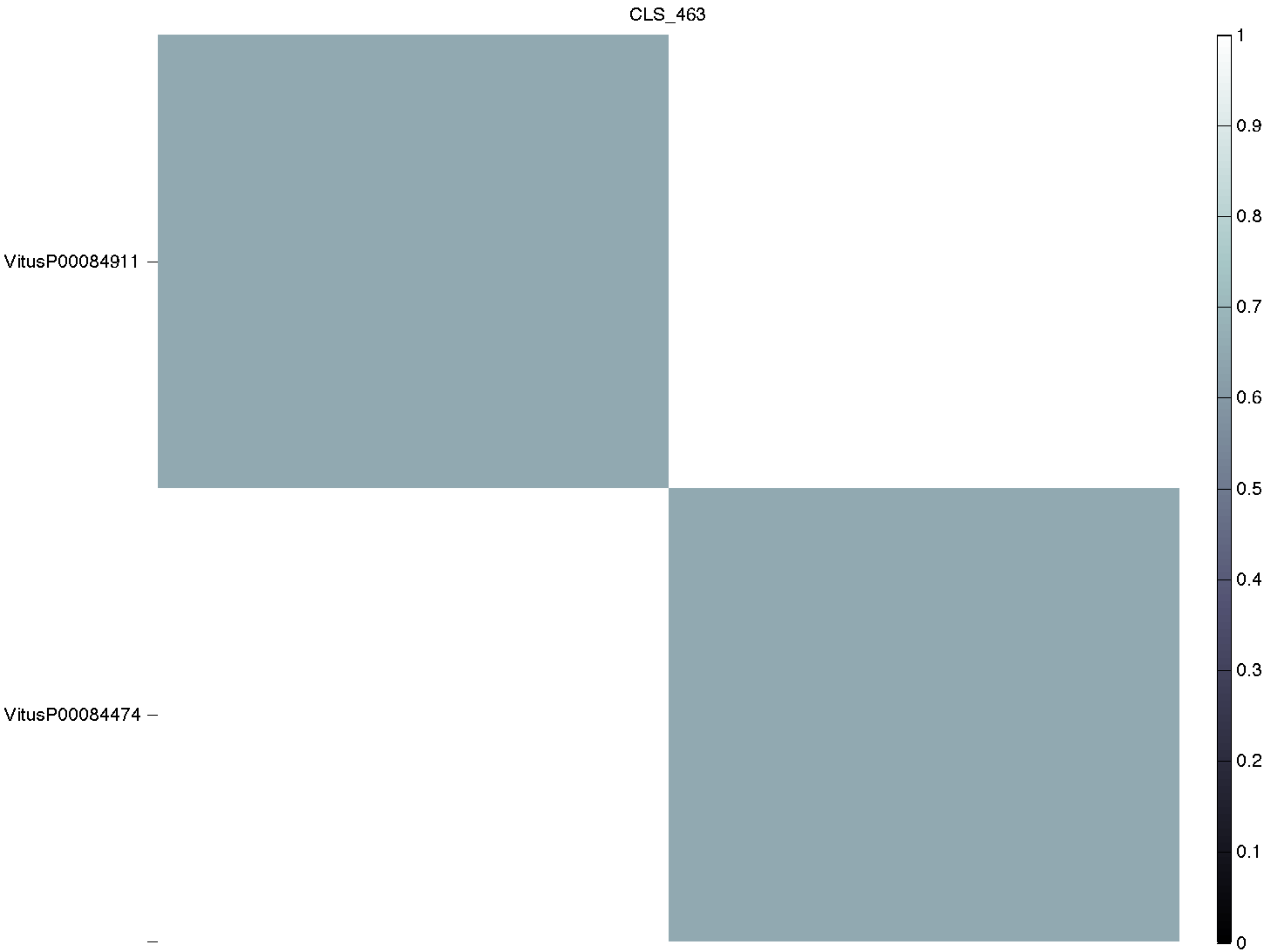

CLS_463
VitusP00084911
VitusP00084474
1
0.9
0.8
0.7
0.6
0.5
0.4
0.3
0.2
0.1
0

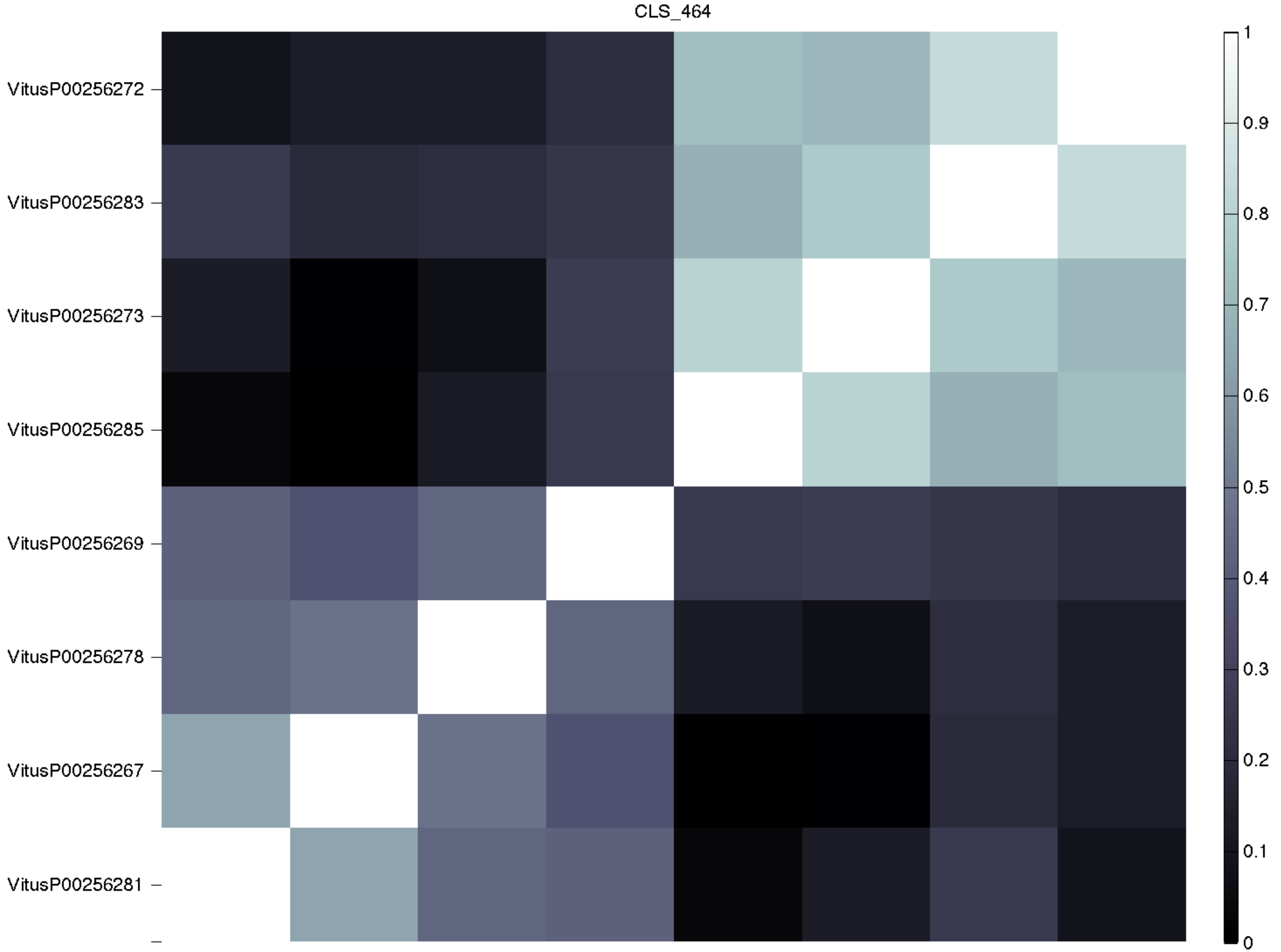
CLS_464
VitusP00256272
VitusP00256283
VitusP00256273
VitusP00256285
VitusP00256269
VitusP00256278
VitusP00256267
VitusP00256281
1
0.9
0.8
0.7
0.6
0.5
0.4
0.3
0.2
0.1
0

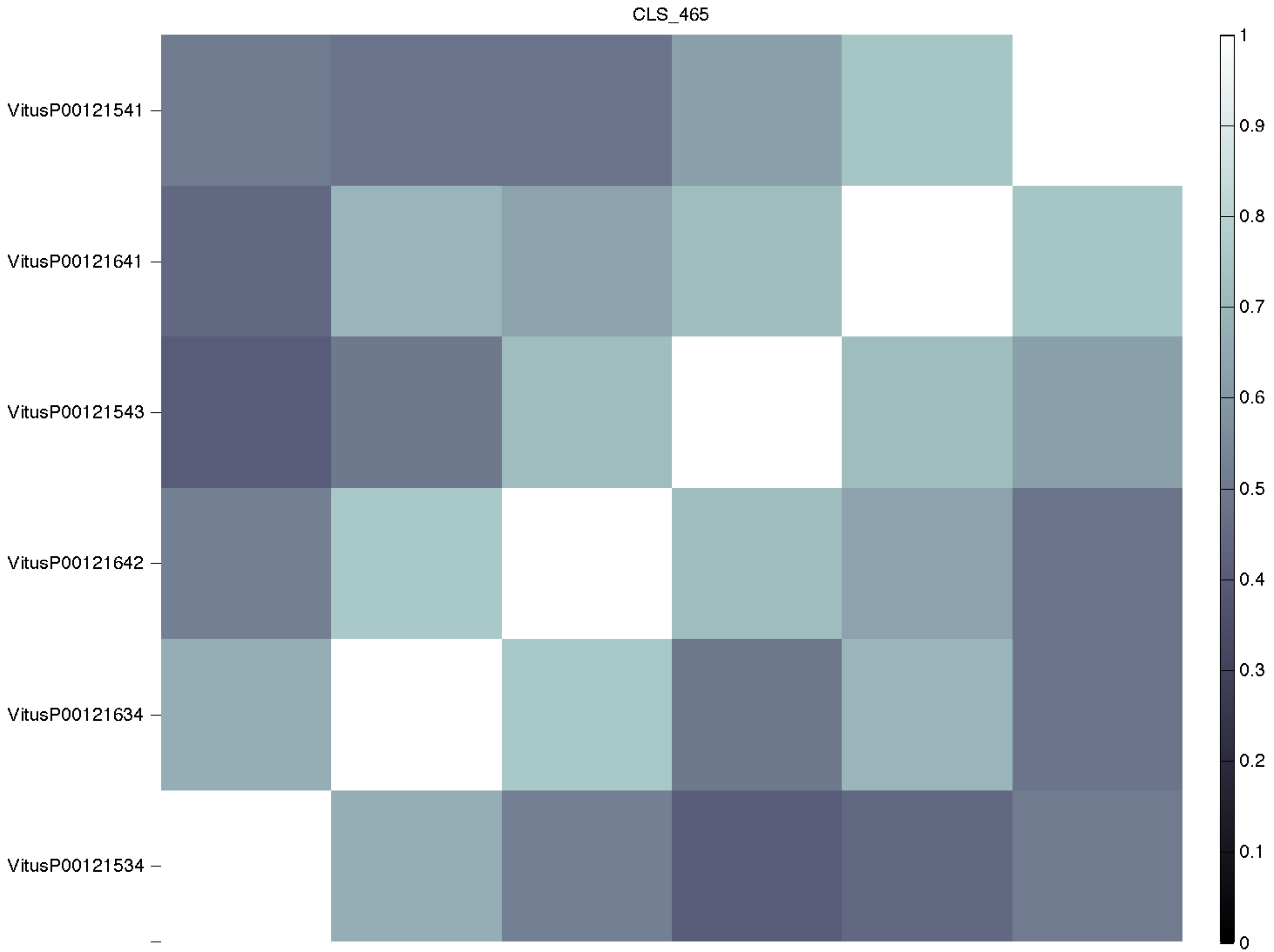

CLS_465
VitusP00121541
VitusP00121641
VitusP00121543
VitusP00121642
VitusP00121634
VitusP00121534
1
0.9
0.8
0.7
0.6
0.5
0.4
0.3
0.2
0.1
0

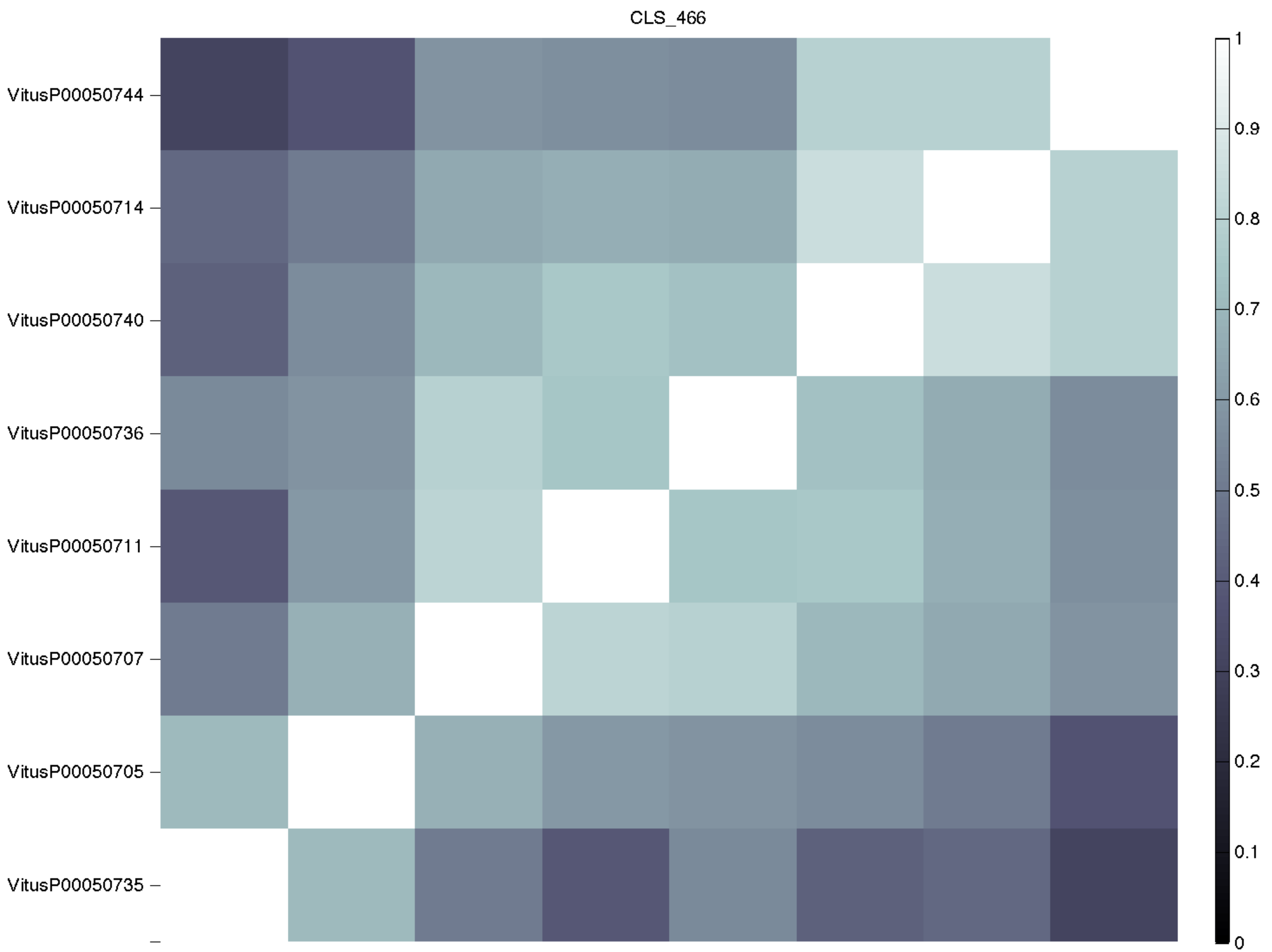

CLS_466
VitusP00050744
VitusP00050714
VitusP00050740
VitusP00050736
VitusP00050711
VitusP00050707
VitusP00050705
VitusP00050735
1
0.9
0.8
0.7
0.6
0.5
0.4
0.3
0.2
0.1
0
